# Low tristetraprolin expression activates phenotypic plasticity and primes transition to lethal prostate cancer in mice

**DOI:** 10.1172/JCI175680

**Published:** 2024-11-19

**Authors:** Katherine L. Morel, Beatriz Germán, Anis A. Hamid, Jagpreet S. Nanda, Simon Linder, Andries M. Bergman, Henk van der Poel, Ingrid Hofland, Elise M. Bekers, Shana Y. Trostel, Deborah L. Burkhart, Scott Wilkinson, Anson T. Ku, Minhyung Kim, Jina Kim, Duanduan Ma, Jasmine T. Plummer, Sungyong You, Xiaofeng A. Su, Wilbert Zwart, Adam G. Sowalsky, Christopher J. Sweeney, Leigh Ellis

**Affiliations:** 1 South Australian Immunogenomics Cancer Institute, University of Adelaide, Adelaide, South Australia, Australia.; 2 Center for Prostate Disease Research, Murtha Cancer Center Research Program, Department of Surgery, Uniformed Services University of the Health Sciences, Bethesda, Maryland, USA.; 3 Walter Reed National Military Medical Center, Bethesda, Maryland, USA.; 4 The Henry M. Jackson Foundation for the Advancement of Military Medicine Inc., Bethesda, Maryland, USA.; 5 Genitourinary Malignancies Branch, Center for Cancer Research, National Cancer Institute, Bethesda, Maryland, USA.; 6 Department of Medical Oncology, Dana-Farber Cancer Institute, Harvard Medical School, Boston, Massachusetts, USA.; 7 Department of Surgery, University of Melbourne, Melbourne, Victoria, Australia.; 8 Division of Hematology and Oncology, Department of Medicine, Cedars-Sinai Medical Center, Los Angeles, California, USA.; 9 Division of Oncogenomics, Oncode Institute;; 10 Division of Medical Oncology;; 11 Division of Urology;; 12 Core Facility Molecular Pathology and Biobanking; and; 13 Division of Pathology; Netherlands Cancer Institute, Amsterdam, Netherlands.; 14 Department of Biomedical Sciences, Cedars-Sinai Medical Center, Los Angeles, California, USA.; 15 David H. Koch Institute for Integrative Cancer Research, Bioinformatics and Computing Facility of Swanson Biotechnology Center, Massachusetts Institute of Technology, Cambridge, Massachusetts, USA.; 16 Department of Developmental Neurobiology, St. Jude Children’s Research Hospital, Memphis, Tennessee, USA.; 17 Division of Urology, Department of Surgery, Cedars-Sinai Medical Center, Los Angeles, California, USA.; 18 Cedars-Sinai Samuel Oschin Comprehensive Cancer Institute, Los Angeles, California, USA.

**Keywords:** Cell biology, Oncology, Mouse models, Prostate cancer

## Abstract

Phenotypic plasticity is a hallmark of cancer and is increasingly realized as a mechanism of resistance to androgen receptor–targeted (AR-targeted) therapy. Now that many prostate cancer (PCa) patients are treated upfront with AR-targeted agents, it is critical to identify actionable mechanisms that drive phenotypic plasticity, to prevent the emergence of resistance. We showed that loss of tristetraprolin (TTP; gene *ZFP36*) increased NF-κB activation, and was associated with higher rates of aggressive disease and early recurrence in primary PCa. We also examined the clinical and biological impact of *ZFP36* loss with co-loss of *PTEN*, a known driver of PCa. Analysis of multiple independent primary PCa cohorts demonstrated that *PTEN* and *ZFP36* co-loss was associated with increased recurrence risk. Engineering prostate-specific *Zfp36* deletion in vivo induced prostatic intraepithelial neoplasia, and, with *Pten* codeletion, resulted in rapid progression to castration-resistant adenocarcinoma. *Zfp36* loss altered the cell state driven by *Pten* loss, as demonstrated by enrichment of epithelial–mesenchymal transition (EMT), inflammation, TNF-α/NF-κB, and IL-6–JAK/STAT3 gene sets. Additionally, our work revealed that *ZFP36* loss also induced enrichment of multiple gene sets involved in mononuclear cell migration, chemotaxis, and proliferation. Use of the NF-κB inhibitor dimethylaminoparthenolide (DMAPT) induced marked therapeutic responses in tumors with *PTEN* and *ZFP36* co-loss and reversed castration resistance.

## Abstract 2

Tristetraprolin loss, or reduced expression, activates NF-κB induced phenotypic plasticity and primes the transition to lethal prostate cancer, which can be mitigated by DMAPT treatment

## Introduction

Multiple factors have been shown to drive prostate cancer (PCa) progression, including inflammation (1, 2) and NF-κB (p65) activation (3, 4). Chronic inflammation is commonly observed in prostate tumors, although the exact stimuli required to initiate and maintain prostatic inflammation are not fully understood. This inflammatory response consists of the recruitment and expansion of leukocytes including myeloid cells, macrophages, and lymphocytes in the prostate (5–7). Immunohistochemical analysis has shown that p65 is active in early PCa, including prostate intraepithelial neoplasia (PIN) and low- and high-grade PCa (8, 9). In vitro and in vivo modeling has linked constitutive NF-κB activation to many of the hallmarks of cancer, including proliferation and evasion of apoptosis, which can be abrogated by NF-κB inhibition (10, 11).

Because of the complex feedback control mechanisms, markers of NF-κB activation that associate with poor PCa outcomes have been elusive (12). With a systems biology approach focused on identifying regulators associated with development of lethal PCa, we previously identified tristetraprolin (TTP) as a key node in controlling NF-κB activation and progression to lethal PCa (13). We noted that patients with lower *ZFP36* were more likely to relapse and die of PCa (14–17) and that silencing *ZFP36* made non-transformed prostate cells (RWPE-1) proliferate more rapidly, while overexpressing *ZFP36* decreased proliferation of the LNCaP PCa cell line (14).

Tristetraprolin is a member of the TPA-inducible sequence 11 (TIS11) family of RNA-binding proteins that directly binds to *cis*-acting adenosine- and uridine-rich elements in the 3′-UTR of target mRNA, leading to recruitment of enzymes for the rapid shortening of the poly(A) tail, which results in transcript deadenylation and degradation (18, 19). This post-transcriptional regulation of mRNA stability allows cells to respond to intracellular and extracellular stimuli. Relevant to our work is that TTP also mediates degradation of TNF-α mRNA (an NF-κB activator) and IL-1β (20). Loss of TTP therefore results in NF-κB hyperactivation with associated inflammatory conditions including arthritis and dermatitis (21–23). Inflammation plays a crucial role during PCa initiation, progression, and metastasis (24). Chronic elevation of proinflammatory genes is known to promote tumor evolution by increased proliferation, angiogenesis, metastasis, survival, and drug resistance of cancer cells (25, 26). Because TTP can negatively regulate many inflammatory and oncogenic cytokines (27), this inflammatory-suppressive feature of TTP may prevent the occurrence and progression of many cancers, including PCa. In addition to mediating expression of inflammatory genes, TTP has also been demonstrated to mediate degradation and control expression of oncogenes and tumor suppressor genes including *c-Myc*, *c-JUN*, and *p53* (19). Moreover, neuronal differentiation exhibited dependence on TTP expression and function (19, 28), implying that TTP may be an important player in determining cell identity and tumor evolution.

TTP has previously been described as a “prospective” cancer tumor suppressor (18, 29, 30). Here, we established the role of TTP as a tumor suppressor in PCa and determined that NF-κB activation contributes to the deleterious effects of TTP loss within tumor cells. Given the finding that TTP loss occurs early in PCa development, we used genetically engineered mouse models (GEMMs) to investigate the impact of TTP loss in combination with PTEN loss on prostate epithelial cell transformation to cancer. Mice with co-loss of TTP and PTEN had a significant increase in PCa progression and a decrease in responsiveness to castration compared with GEMMs with PTEN loss alone. We demonstrate that this aggressive phenotype, driven by TTP loss, induces phenotypic plasticity, and that this altered plasticity occurs independent of RB1 and P53. This is shown by the observation of decreased androgen receptor (AR) expression and function, and derepression of gene signatures enriched for nervous system development and leukocyte cell identity and function. Our data provide new insight toward mechanistic understanding of phenotypic plasticity involving a TTP/p65 signaling axis that can be countered by pharmaceutical targeting of NF-κB. Finally, these data provide strong rationale for clinical assessment of enhancement of the efficacy of hormonal therapy by NF-κB inhibition.

## Results

### Loss of ZFP36/TTP in prostate cancer patients selects for aggressive disease.

**Figure 1:**
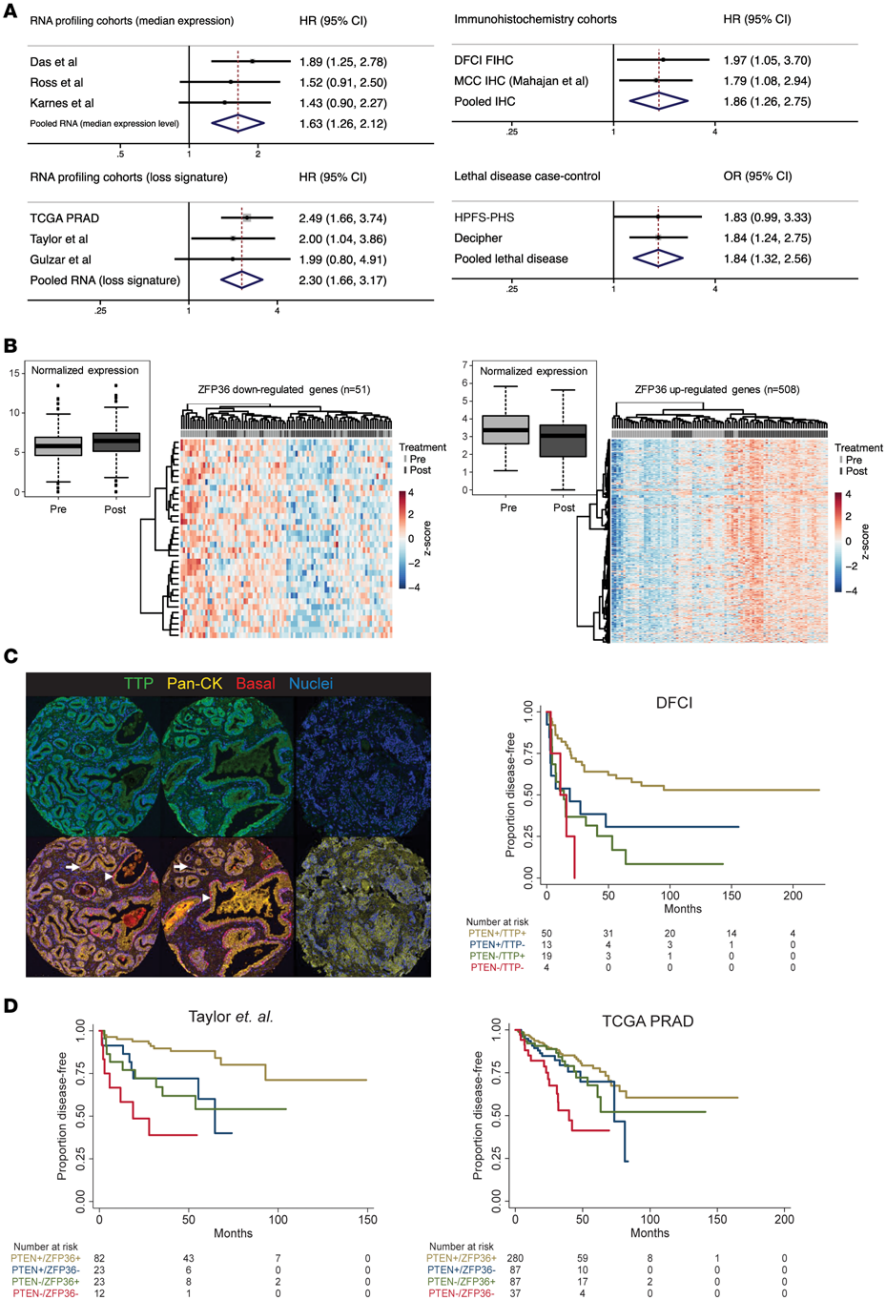
*ZFP36*/TTP and clinical outcomes. (**A**) RNA- and IHC-based forest plots depicting *ZFP36*/TTP expression related to clinical outcomes (biochemical recurrence and disease-free survival) and risk of lethal PCa (case-control cohorts). TCGA PRAD, The Cancer Genome Atlas Prostate Adenocarcinoma data set; DFCI FIHC, Dana-Farber Cancer Institute Fluorescent IHC; HPHS-PHS, Health Professionals Follow-up Study and Physicians’ Health Study. (**B**) Upregulated and downregulated genes were identified by differential expression analysis of TCGA PRAD cases divided by lower-quartile expression of *ZFP36*. (**C**) Representative images of immunofluorescent (IF) staining for pan-cytokeratin (yellow) and basal (red) markers, as well as TTP (green) in human PCa used for expression analysis. Benign glands (arrowheads) costain for pan-cytokeratin and basal cocktails; tumor cells (arrows) demonstrate absent basal expression. Far right images display diffuse prostate tumor with absent TTP expression. (**D**) Kaplan-Meier survival analysis demonstrating that TTP deficiency, measured by protein expression (DFCI, refs. 40, 69) and *ZFP36* mRNA expression (TCGA PRAD, ref. 35; Taylor et al., ref. 34), results in shorter disease-free-survival, and even shorter disease-free survival in combination with PTEN deficiency.

In previously published, independent cohorts of localized hormone-sensitive PCa, meta-analysis identified a consistent association with *ZFP36*/TTP loss and increased risk of biochemical recurrence and development of lethal disease after curative therapy (Figure 1A, left) (14, 15). *ZFP36*/TTP loss was defined by expression below the cohort median. We then developed a *ZFP36* loss signature (Figure 1B), which was applied to whole-transcriptome datasets with associated clinical outcomes, and a consistent prognostic effect was again observed (Figure 1A, bottom left). To confirm that TTP protein expression holds a similar association, a tissue microarray was created from a cohort of men with localized PCa undergoing radical prostatectomy (RP), and fluorescent immunohistochemistry (IHC) for TTP was performed on RP specimens (Figure 1C). Loss of TTP protein expression was significantly associated with shorter disease-free survival, a finding consistent with a previously reported independent cohort (Mahajan et al.) (15). The combined results are reported as pooled meta-analysis (Figure 1A, top right). We further documented in a meta-analysis of published data (14) that localized PCa with *ZFP36* RNA levels in the lower quartile was associated with an almost 2-fold risk of lethal PCa (metastatic disease or death from PCa) (Figure 1A, bottom right).

Given the reports that co-loss of 2 or more tumor suppressor genes can drive more aggressive disease (31–33), we explored the proposition that low expression of TTP increases the aggressiveness of tumors with PTEN loss. We investigated TTP and PTEN as 2 complimentary, but not necessarily interacting, tumor suppressor pathways in PCa. We performed additional IHC for PTEN and observed that men within the lower quartile of expression of loss of one tumor suppressor had shorter disease-free survival (PTEN low: HR 2.86, 95% CI 1.61–5.10, *P* = 0.0002; TTP low: HR 1.97, 95% CI 1.05–3.70, *P* = 0.03) (Figure 1D, left). Notably, men with both low TTP and low PTEN expression had the poorest disease-free survival (HR 2.98, 95% CI 1.05–8.46, *P* = 0.03), which remained significant in multivariable analysis adjusting for prognostic clinicopathological factors of Gleason score, tumor stage, and prostate-specific antigen (PSA) (HR 4.50, 95% CI 1.03–19.61, *P* = 0.045). The finding that lower levels of TTP and PTEN portended the highest risk of relapse was independently supported by gene expression analyses from 2 independent cohorts (34, 35) noting that patients with transcript levels of *ZFP36* or *PTEN* alone in the lower quartile had poorer disease-free survival than men with higher levels of both *ZFP36* and *PTEN*, and that men with concurrent low levels of *ZFP36* and *PTEN* had the highest risk of relapse (Figure 1D). These data validate in multiple independent datasets that low TTP/*ZFP36* expression is related to poor prognosis in PCa, and that the additional loss of PTEN expression corresponds with even more aggressive disease.

### TTP and ZFP36 expression is upregulated in prostate tumors in response to enzalutamide treatment.

**Figure 2:**
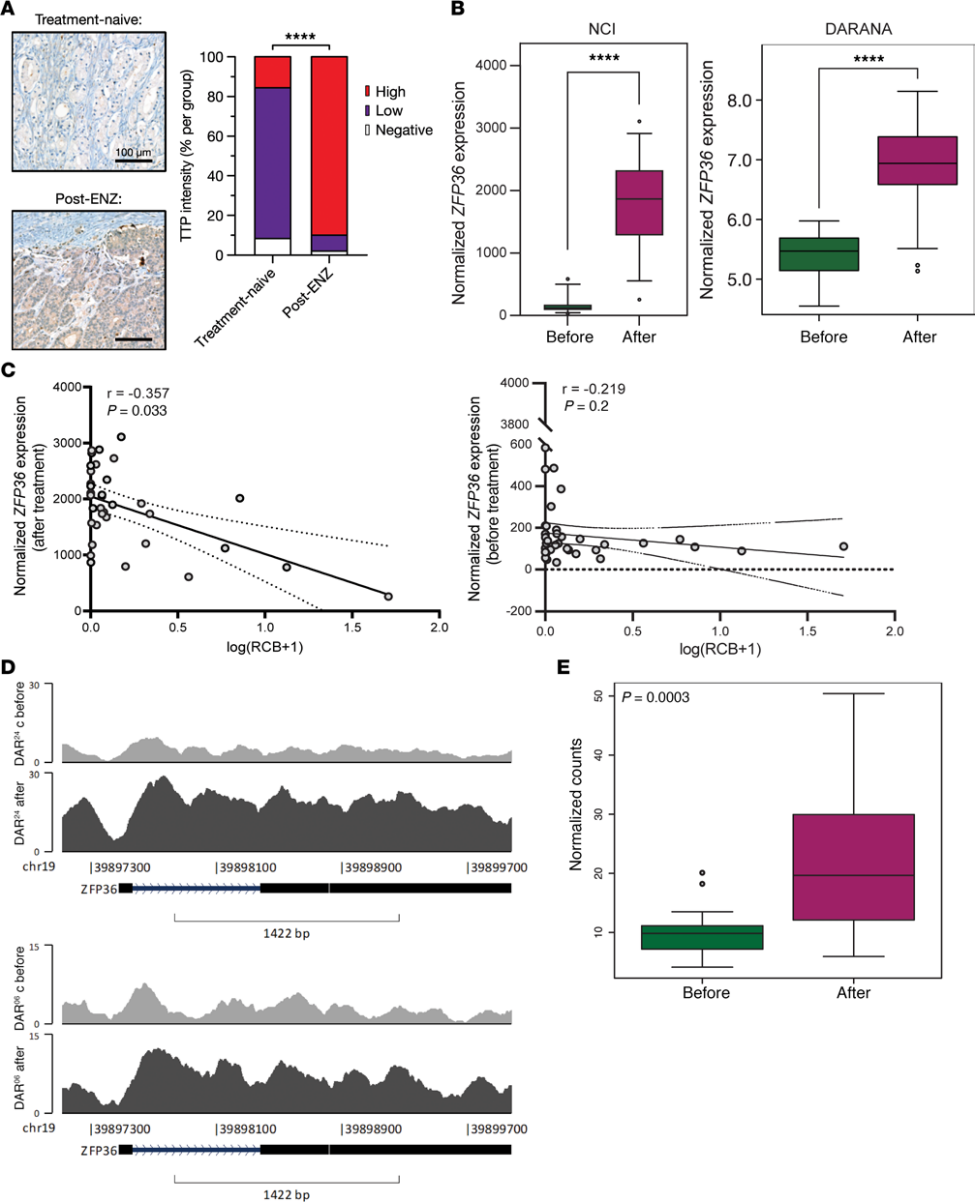
*ZFP36*/TTP expression in response to enzalutamide. (**A**) Representative images and quantification of TTP IHC staining intensity in DARANA patient tissues comparing treatment-naive and post-enzalutamide samples. ****P < 0.0001 (Fisher’s exact test). Scale bars: 100 μm. (**B**) *ZFP36* expression from RNA-Seq before and after enzalutamide in the NCI and DARANA clinical studies. *n* = 36–52, ****P < 0.0001 (2-tailed paired-samples *t* test). (**C**) Correlation of normalized *ZFP36* expression versus volume of post-treatment residual cancer burden (RCB) in pre- and post-enzalutamide samples. *n* = 36 patients (non-parametric Spearman’s correlation). (**D**) Representative H3K27 acetylation tracks at the *ZFP36* locus from 2 DARANA patients, comparing pre- and post-enzalutamide samples. (**E**) Quantification of H3K27 acetylation signal at the *ZFP36* locus before and after enzalutamide treatment (paired-samples *t* test).

To examine the clinical response of *ZFP36*/TTP to treatment with enzalutamide (a nonsteroidal anti-androgen therapy), we examined 2 clinical cohorts where the prostate tumors from the same patient were investigated before and after enzalutamide treatment. PCa samples from the DARANA (Dynamics of Androgen Receptor Genomics and Transcriptomics After Neoadjuvant Androgen Ablation) study (36) displayed significant upregulation of *ZFP36*/TTP at the protein (*P* < 0.0001; Figure 2A) and gene expression (*P* = 0.0001; Figure 2B) levels following enzalutamide treatment. Similar RNA *ZFP36* upregulation was observed in an independent neoadjuvant androgen deprivation therapy (ADT) study, the National Cancer Institute (NCI) dataset of paired pre- and post-treatment PCa samples treated with neoadjuvant ADT plus enzalutamide (*P* < 0.0001; Figure 2B). In the NCI tumor dataset, we examined the correlation between *ZFP36* expression and the volume of post-treatment residual tumor (Figure 2C). *ZFP36* expression before treatment did not correlate with the volume of post-treatment residual tumor (Spearman’s *R* value = –0.2187, *P* = 0.2). However, there was a significant negative correlation of *ZFP36* expression in post-treatment samples (Spearman’s *R* value = –0.3566, *P* = 0.033), indicating that the largest residual tumors after enzalutamide occurred in patients in whom *ZFP36* RNA expression was low before and after treatment, we concluded that patients with low ZFP36 levels are worse responders to therapy. Following enzalutamide treatment, patient samples from the DARANA study showed significant increases in H3K27 acetylation at the *ZFP36* locus after enzalutamide (*P* = 0.0003; Figure 2, D and E), epigenetically confirming enhanced transcriptional activation of this locus following treatment.

### Loss of Zfp36 accelerates disease progression in a mouse model of prostate adenocarcinoma driven by Pten deletion.

To model the clinical finding that low expression of *ZFP36* synergizes with *PTEN* loss to drive aggressive PCa progression, we engineered *Zfp36* deletion in a previously characterized mouse model of prostate adenocarcinoma induced by *Pten* deletion (37). Our model used expression of the PB-Cre4 transgene (38) to ensure prostate epithelial cell–exclusive deletion of *Zfp36*- and *Pten*-floxed alleles (Supplemental Figure 1, A and B; supplemental material available online with this article; https://doi.org/10.1172/JCI175680DS1). PB-Cre4 *Pten^f/f^* (*Pten^f/f^*) mice develop PIN by 6–8 weeks and progress to invasive adenocarcinoma with rare incidence of metastatic disease (37). In parallel, PB-Cre4 *Zfp36^+/f^* (*Zfp36^+/f^*) and PB-Cre4 *Zfp36^f/f^* (*Zfp36^f/f^*) mice developed PIN but did not progress to adenocarcinoma (Supplemental Figure 2A). The effect of *ZFP36* loss alone was also assessed in vitro using a primary human prostate epithelial model immortalized by expression of human telomerase reverse transcriptase (hTERT) with coexpression of AR (957E/hTERT, PrEC-AR) (39). Loss of *ZFP36* expression in this model resulted in overall increased cell proliferation (Supplemental Figure 2B). Notably, this model showed that *ZFP36* loss increased proliferation. With *ZFP36* intact, the cells exhibited the previously reported androgen-mediated anti-growth phenotype (stimulated by R1881, also known as methyltrienolone, a synthetic androgen) (39), which was attenuated by *ZFP36* loss (Supplemental Figure 2B). Further examination of these genotypes using GEMM-derived 2D cell lines demonstrated that only homozygote loss of *Zfp36* enabled cell transformation and growth, equivalent to that of organoids exhibiting *Pten* loss (Supplemental Figure 2C). Together, these data clearly demonstrate that TTP can reprogram AR signaling and induce PIN, a precursor lesion to primary prostate adenocarcinoma.

**Figure 3:**
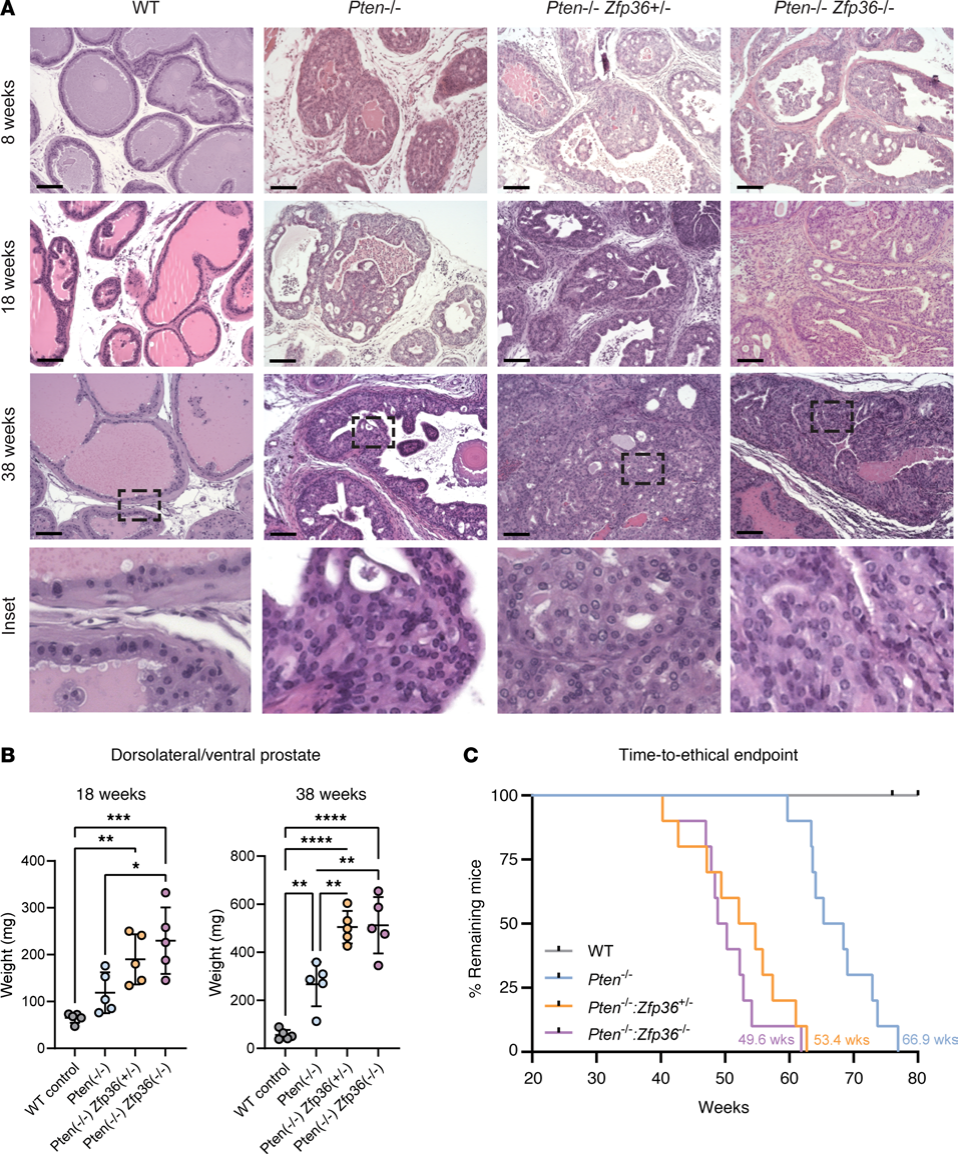
*Zfp36* loss accelerates progression of prostate cancer in *Pten*-null murine tumors. (**A**) H&E staining of murine tumors highlighting morphological progression of wild-type (WT), *Pten^f/f^* *Zfp36^+/+^* (*Pten^–/–^*), *Pten^f/f^* *Zfp36^f/+^* (*Pten^–/–^* *Zfp36^+/–^*), and *Pten^f/f^* *Zfp36^f/+^* (*Pten^–/–^* *Zfp36^–/–^*) dorsolateral prostate tissue at 8, 18, and 38 weeks. Scale bars: 100 μm. (**B**) Comparative weight of dorsolateral and ventral prostate tissue in GEMMs at 18 and 38 weeks. *n* = 5 mice per genotype, **P < 0.005, ***P < 0.0005, ****P < 0.0001 (1-way ANOVA with Tukey’s post hoc). (**C**) Kaplan-Meier graphs from GEMM aging studies show that prostate-specific deletion of *Zfp36* significantly reduces time to ethical endpoint in PCa driven by loss of *Pten* *n* = 10 mice per genotype (Mantel-Cox log-rank test).

Histological analyses of collected prostates from mice harboring prostate-specific loss of *Pten* either alone or in combination with heterozygous or homozygous loss of *Zfp36* clearly indicated that the loss of *Zfp36* significantly increased progression of PCa initiation and progression of primary disease (Figure 3A). Given the cystic anterior prostate phenotype commonly observed *Pten-*null GEMMs, we took weight measurements from dorsolateral and ventral prostate lobes. The combined weights of the dorsolateral and ventral prostate lobes in mice with combined *Pten* and *Zfp36* loss (PB-Cre4 *Pten^f/f^* *Zfp36^+/f^*, 190.0 ± 53.5 mg; PB-Cre4 *Pten^f/f^* *Zfp36^f/f^*, 229.8 ± 71.1 mg) were significantly increased compared with those in *Pten^f/f^* mice (118.8 ± 43.7 mg) at 18 weeks. This increase in combined dorsolateral and ventral prostate lobe weight was also present at 38 weeks of age (Figure 3B). Accordingly, mice with combined *Pten* and *Zfp36* loss exhibited shorter median survival of 53.4 weeks for *Pten^f/f^* *Zfp36^+/f^* and 49.6 weeks for *Pten^f/f^* *Zfp36^f/f^* mice compared with 66.9 weeks for *Pten^f/f^* mice (*P* < 0.0001) (Figure 3C).

**Figure 4:**
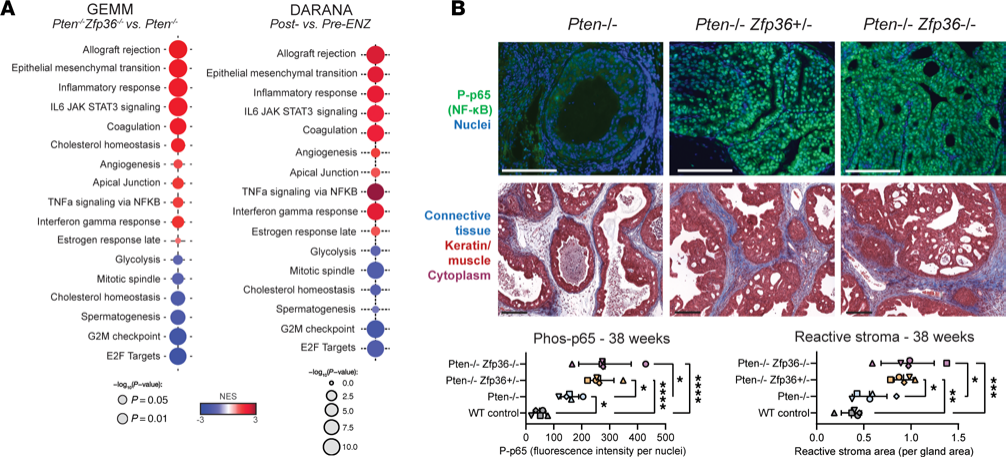
*Zfp36* loss increases an inflammatory prostate cancer phenotype in *Pten*-null murine tumors. (**A**) GSEA from RNA-Seq of endpoint GEMM PCa tumors comparing *Pten^–/–^* and *Pten^–/–^* *Zfp36^–/–^* GEMMs, highlighting positively and negatively enriched Hallmark pathways. (**B**) Phospho-p65 IF and Masson’s trichrome staining PCa in *Pten^–/–^*, *Pten^–/–^* *Zfp36^+/–^*, and *Pten^–/–^* *Zfp36^–/–^* GEMM dorsolateral prostate tissue at 38 weeks, with corresponding quantification. Scale bars: 100 μm. *n* = 5 mice per genotype; each mouse has been assigned a unique symbol for comparison across IHC analyses; *P < 0.05, **P < 0.005, ***P < 0.0005, ****P < 0.0001 (1-way ANOVA with Tukey’s post hoc).

To further characterize changes mediated by *Zfp36* loss, RNA sequencing (RNA-Seq) from mouse tumors was performed. Given the molecular regulation of NF-κB activity by *Zfp36* (14), it was not surprising that gene set enrichment analysis (GSEA) Hallmark analysis indicated marked enrichment for inflammatory-associated gene signatures (Figure 4A and Supplemental Table 3). GSEA Hallmark analysis data comparing *Pten^f/f^* *Zfp36^f/f^* versus *Pten^f/f^* GEMM prostate tumors were strikingly similar to DARANA clinical data comparing pre- versus post-enzalutamide prostate tumors (Figure 4A). Further validation of NF-κB activation (phosphorylated p65) and overall stromal inflammation was performed by IHC on tumors extracted from GEMMs at 38 weeks (Figure 4B). These data confirmed that the observed aggressive nature of prostate tumors harboring *Pten Zfp36* loss was in part due to a significant increase in tumor cell NF-κB activity and a resulting overall inflammatory phenotype.

**Figure 5:**
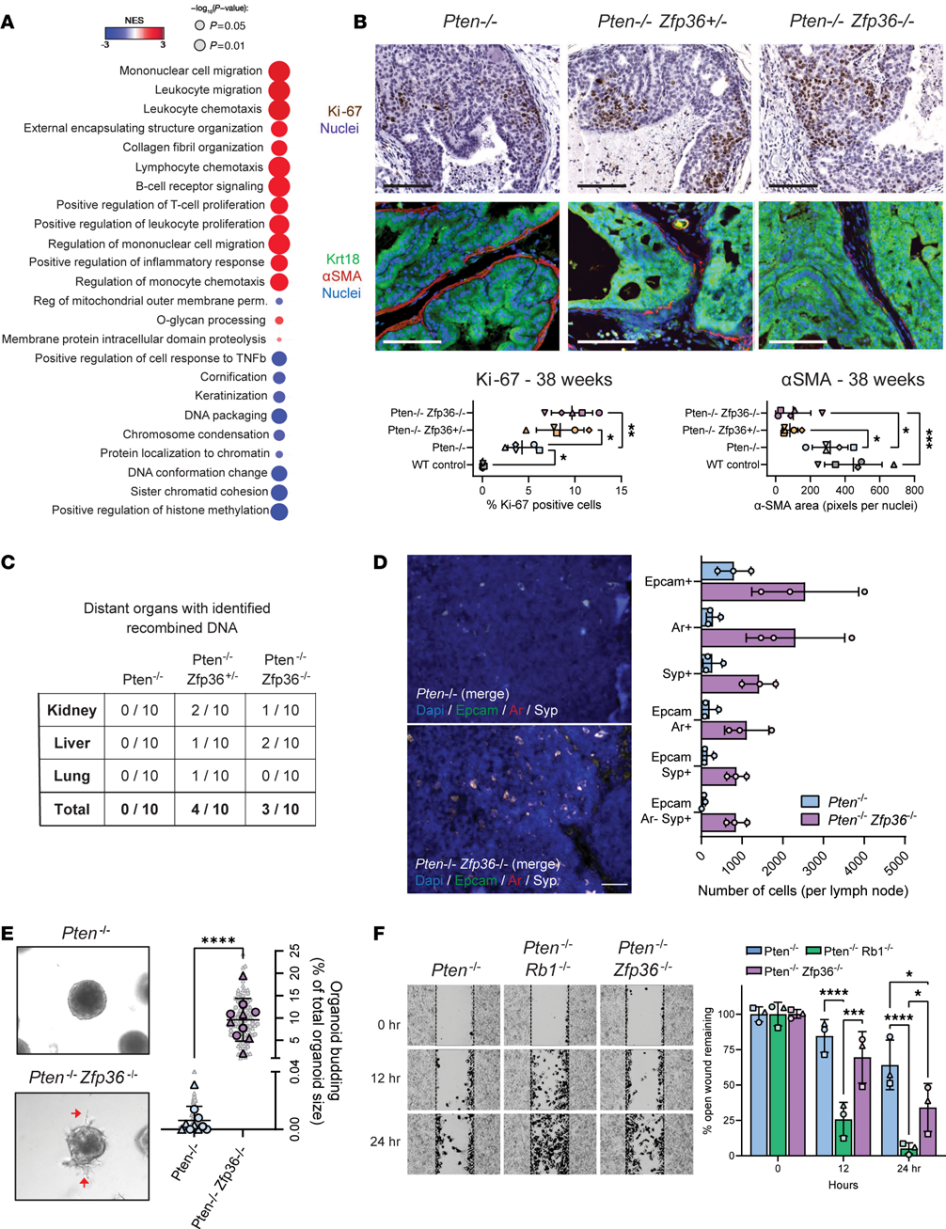
Increased metastatic potential occurs with *Zfp36* loss in *Pten*-null murine tumors. (**A**) GSEA from RNA-Seq of endpoint GEMM PCa tumors comparing *Pten^–/–^* and *Pten^–/–^* *Zfp36^–/–^* GEMMs, highlighting significant positively and negatively enriched GOBP pathways. (**B**) Ki-67 IHC and Krt18 and αSMA IF staining PCa in *Pten^–/–^*, *Pten^–/–^* *Zfp36^+/–^*, and *Pten^–/–^* *Zfp36^–/–^* GEMM dorsolateral prostate tissue at 38 weeks, with corresponding quantification. Increased tumor cell proliferation and basement membrane breakdown are observed with loss of *Zfp36*. *n* = 5 mice per genotype, *P < 0.05, **P < 0.005, ***P < 0.0005 (1-way ANOVA with Tukey’s post hoc). Scale bar: 100 μm. (**C**) Number of mice that displayed PCa cells in distant organs by recombination PCR in *Pten^–/–^*, *Pten^–/–^* *Zfp36^+/–^*, and *Pten^–/–^* *Zfp36^–/–^* GEMMs. (**D**) Representative images and quantification of the full scan in FFPE sections of tumor-adjacent pelvic lymph nodes (LNs) in *Pten^–/–^* and *Pten^–/–^ Zfp36^–/–^* mice. LNs were stained by multiplex IHC for Epcam (green), AR (red), and synaptophysin (Syp; white). Positive cells identify epithelial/tumoral cells metastasizing LNs (*n* = 3 mice per genotype). Scale bar: 50 μm. (**E**) Representative images and quantification of budding in GEMM-derived organoids highlighting increased invasive and metastatic potential of *Pten^–/–^* *Zfp36^–/–^* organoids. *n* = 5 unique organoid lines per genotype with experiment repeated once; experiments 1 and 2 are denoted by different symbols (technical replicates are underlaid in gray); ****P < 0.0001 (2-tailed Student’s *t* test). (**F**) Scratch assay in GEMM-derived 2D cells, comparing *Pten^–/–^* and *Pten^–/–^* *Zfp36^–/–^* wound healing with that of *Pten^–/–^* *Rb1^–/–^*, a previously described metastatic, neuroendocrine PCa murine cell line (32). Representative images of scratch assay have been overlaid with areas identified as wound infiltrate in black. *n* = 1 cell line per genotype with experiment repeated twice; experiments 1, 2, and 3 are denoted by unique symbols; *P < 0.05, ***P < 0.001, ****P < 0.0001 (2-way ANOVA with Tukey’s post hoc).

Additional analysis using Gene Ontology Biological Process (GOBP) from our RNA-Seq from GEMM tumors identified substantial positive enrichment of inflammation and leukocyte gene expression signatures in *Pten^f/f^* *Zfp36^f/f^* mice compared with *Pten^f/f^* mice (Figure 5A and Supplemental Table 4). Owing to this enrichment of immune-related gene signatures and concern that an immune infiltrate could be the cause of these results, we performed additional RNA-Seq on *Pten-*deleted 2D PCa cell lines generated from our GEMMs (32). We further engineered these cells for *Zfp36* loss by use of CRISPR/Cas9. RNA-Seq from these 2D cell lines showed complementary results, indicating that the observed induction of immune-related gene signatures was autonomous to the tumor cell (Supplemental Tables 5 and 6 and Supplemental Figure 3). Leukocyte gene signatures that were enriched in our gene expression data from prostate luminal epithelial cells were related to chemotaxis, proliferation, and migration. We validated these attributes in tumor tissue from mice, indicating that loss of *Zfp36* significantly increased overall cell proliferation, in line with increased proliferation in prostate cell lines (Supplemental Figure 2) and potential for invasion by degradation of the basement membrane, as assessed by α-smooth muscle actin (αSMA) staining (Figure 5B). Aligned with this aggressive phenotype was an increased incidence of distant spread to the kidney, liver, and lung (detected by recombination PCR) and macrometastasis to the pelvic lymph nodes in a subset of mice examined with *Zfp36* loss (Figure 5, C and D). Loss of *Zfp36* increased the number of epithelial cells detected in pelvic lymph nodes (Epcam^+^AR^+^) with expression of synaptophysin (Syp), a characteristic marker of neuroendocrine PCa (Figure 5D and Supplemental Figure 4). No metastasis to the bone was observed with *Zfp36* loss. This in vivo metastatic phenotype was further validated by an increase in invasive potential (organoid budding in soft Matrigel) and cell motility in GEMM-derived ex vivo models (Figure 5, E and F). Wound closure of *Pten^f/f^* *Zfp36^f/f^* cells occurred significantly faster than that of *Pten^f/f^* cells, but was slower in comparison with a previously described metastatic, neuroendocrine PCa model, *Pten^f/f^* *Rb1^f/f^* (32), which hints that loss of *Zfp36* may result in an intermediate phenotype, poised to become aggressively metastatic.

### Zfp36 loss induces tumor cell changes associated with phenotypic plasticity.

**Figure 6:**
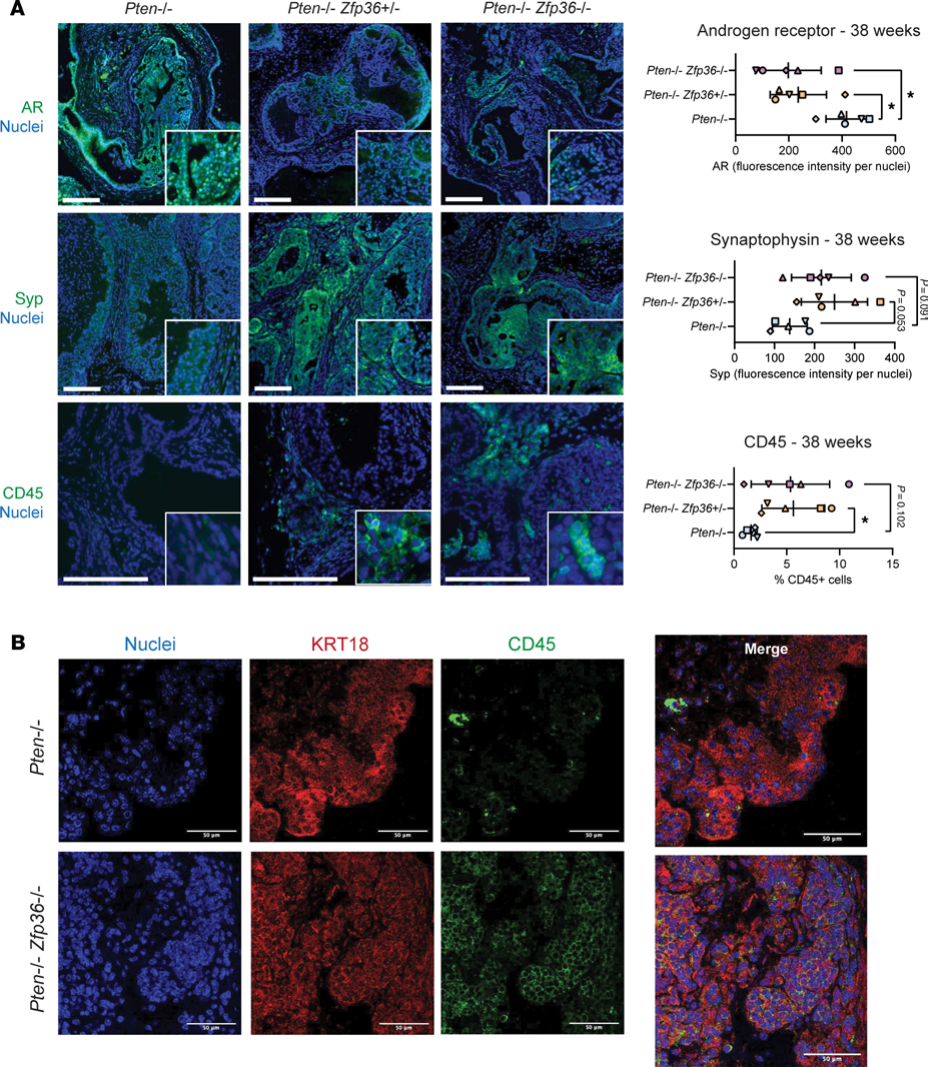
Loss of *Zfp36* induces phenotypic plasticity in *Pten*-null murine prostate tumors. (**A**) AR, synaptophysin (Syp), and CD45 IF staining PCa in *Pten^–/–^*, *Pten^–/–^* *Zfp36^+/–^*, and *Pten^–/–^* *Zfp36^–/–^* GEMM dorsolateral prostate tissue at 38 weeks, with corresponding quantification. Scale bars: 100 μm. *n* = 5 mice per genotype, *P < 0.05 (1-way ANOVA with Tukey’s post hoc). (**B**) Dual Krt8 and CD45 IF staining in *Pten^–/–^* and *Pten^–/–^* *Zfp36^–/–^* GEMM dorsolateral prostate tissue at 38 weeks. Scale bars: 50 μm.

Given the observed rapid disease progression and gene set enrichments following loss of *Zfp36*, we hypothesized that phenotypic plasticity was occurring given similar findings when loss of other tumor suppressors occur with PTEN loss (31, 32). To that end, tumors extracted from *Pten^f/f^*, *Pten^f/f^* *Zfp36^+/f^*, and *Pten^f/f^* *Zfp36^f/f^* GEMMs at 38 weeks were interrogated by immunofluorescent staining to examine AR, synaptophysin, and CD45 antigen (CD45) expression. Tumors from *Pten^f/f^* mice retained AR and had minimal synaptophysin and CD45 expression, while GEMM tumors with combined loss of *Pten* and *Zfp36* displayed an inversed staining pattern that included reduced AR and increased synaptophysin and CD45 in a heterogeneous manner (Figure 6A). To validate that the increase in CD45^+^ cells was occurring in the luminal prostate cell population, as suggested by our gene expression data, we performed costaining of cytokeratin-8 (Krt8; a marker for luminal prostate epithelium) and CD45. Figure 6B provides clear validation that prostate luminal cells from mice with *Pten^f/f^* *Zfp36^f/f^* deletion do coexpress the luminal marker Krt8 with CD45. The pockets of CD45-positive PCa cells were observed to some extent in all *Pten^f/f^* *Zfp36^f/f^* tumors, in a heterogeneous manner (38-week-old tumors and ethical-endpoint tumors). These data indicate that *Zfp36/ZFP36* is a critical mediator of prostate luminal cell identity.

### Loss of Zfp36 increases resistance to hormonal therapy in Pten-null tumors.

**Figure 7:**
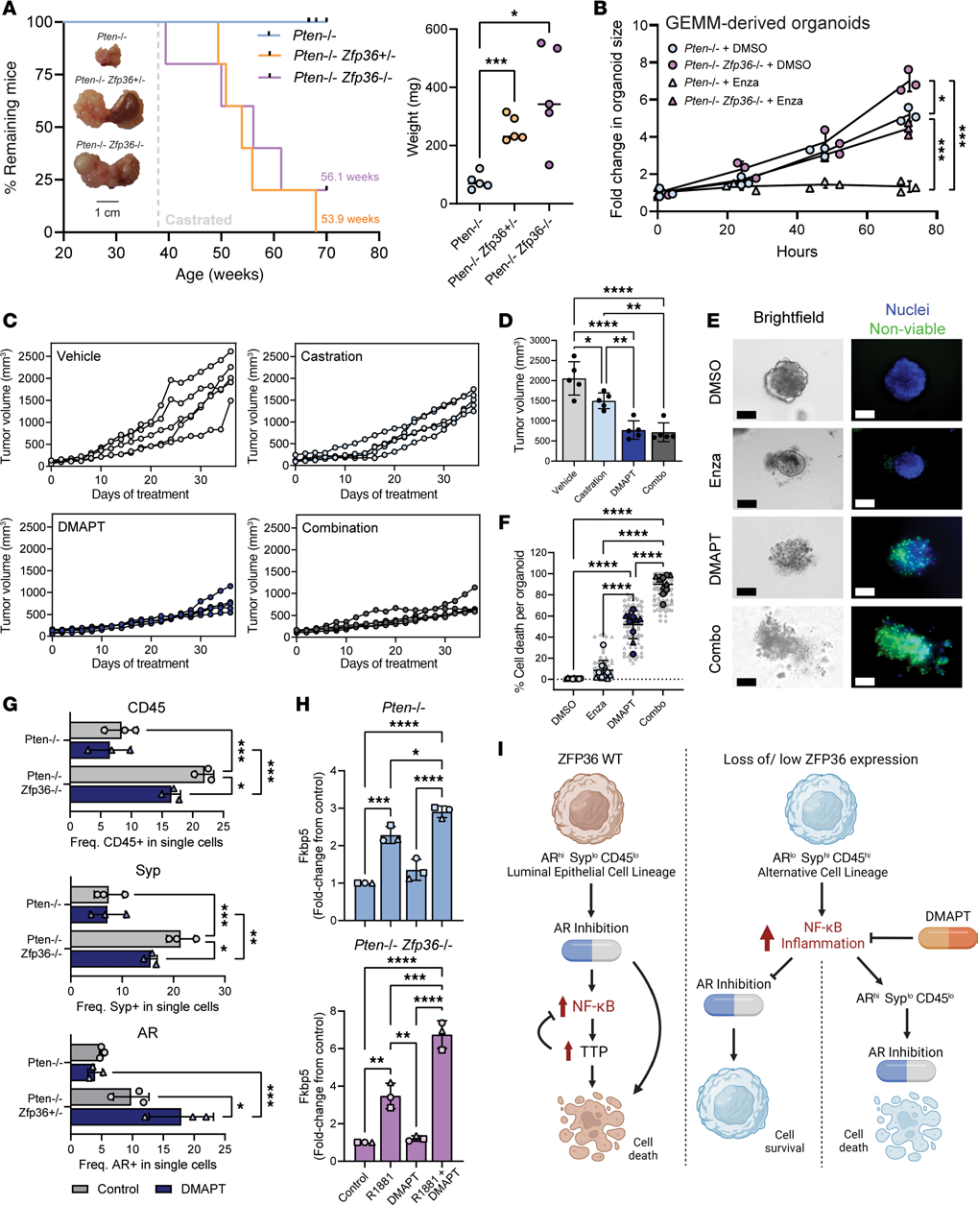
*Zfp36* loss drives castration resistance in *Pten*-null murine tumors, which is counteracted with the NF-κB inhibitor DMAPT. (**A**) Kaplan-Meier graph aged GEMMs (Mantel-Cox log-rank test). Whole prostate weights 12 weeks after castration. *n* = 5 mice per genotype, *P < 0.05, ***P < 0.0005 (1-way ANOVA with Tukey’s post hoc). (**B**) GEMM-derived organoid growth with and without enzalutamide (10 μM). *n* = 1 unique organoid line per genotype, repeated twice; experiments are denoted by unique symbols; *P < 0.05, ***P < 0.0005 (2-tailed multiple *t* test). (**C**) Allograft tumor growth in mice treated with DMAPT (100 mg/kg/d) or vehicle with or without surgical castration. *n* = 5 mice per treatment group. (**D**) Allograft endpoint tumor volumes. *n* = 5 mice per treatment group, *P < 0.05, **P < 0.005, ****P < 0.0001 (1-way ANOVA with Tukey’s post hoc). (**E** and **F**) Representative images (**E**) and quantification (**F**) of cell death (green) in GEMM-derived PCa organoids treated with DMAPT (5 μM), enzalutamide (10 μM), or the combination of both for 72 hours. *n* = 5 unique organoid lines per genotype, repeated once; experiments 1 and 2 are denoted by unique symbols (technical replicates are underlaid in gray); ****P < 0.0001 (1-way ANOVA with Tukey’s post hoc). Scale bars: 100 μm. (**G**) Flow cytometry quantification for CD45, synaptophysin, and AR expression in GEMM-derived PCa organoids treated with DMAPT (5 μM) or DMSO vehicle for 72 hours. *n* = 3 per genotype. *P < 0.05; **P < 0.005; ***P < 0.0005 (2-way ANOVA with Fisher’s least significant difference (LSD) test). (**H**) Fold change of *Fkbp5* in GEMM-derived 2D cell lines treated with DMAPT (5 μM) or DMSO vehicle for 72 hours with or without R1881 (10 nM) stimulation. *n* = 1 organoid line per genotype, repeated twice; experiments 1, 2, and 3 are denoted by unique symbols; *P < 0.05, **P < 0.005, ***P < 0.0005, ****P < 0.0001 (1-way ANOVA with Tukey’s post hoc). (**I**) Schematic overview with *ZFP36* intact, epithelial cells present with a luminal lineage phenotype and sensitivity to AR inhibition. In response to increased NF-κB expression, ZFP36/TTP increases as part of a negative-feedback loop. Loss of *ZFP36* results in an alternative epithelial cell lineage phenotype, with uncontrolled NF-κB activation and reduced response to AR inhibition. DMAPT treatment restores a more luminal epithelial cell type and sensitivity to AR inhibition.

To test the response to androgen deprivation, we surgically castrated 38-week-old, tumor-bearing *Pten^f/f^*, *Pten^f/f^* *Zfp36^+/f^*, and *Pten^f/f^* *Zfp36^f/f^* mice and monitored their survival. Castration, as expected, appeared to be curative in *Pten^f/f^* mice, whereas castration of *Pten^f/f^* *Zfp36^+/f^* and *Pten^f/f^* *Zfp36^f/f^* mice resulted in a median survival of 56.1 and 53.9 weeks, respectively (Figure 7A), indicating little or no effect of androgen deprivation. Notably, there was no observed increased survival benefit following castration in *Pten^f/f^ Zfp36^+/f^* and *Pten^f/f^ Zfp36^f/f^* mice. Further, a cohort of mice had tumors excised for analysis 12 weeks after castration and demonstrated a significant increase in prostate weight (Figure 7A). These initial observations were validated in GEMM-derived ex vivo models, either as tumor transplants in naive wild-type mice or as 3D organoids in culture (Figure 7B and Supplemental Figure 5A). All in vitro, in vivo, and ex vivo models independently demonstrated that loss of *Zfp36* drove rapid acquisition to a castration-resistant phenotype. It had previously been reported that castration resistance could be driven by phenotypic plasticity by loss of the tumor suppressor genes *Pten*, *Rb1*, and/or *Tp53* (31, 32, 40). Using our GEMM-derived ex vivo models, we assessed whether the observed phenotypic plasticity and resistance to castration driven by *Zfp36* loss were associated with *Rb1* and *Tp53* loss of function. Using the Mdm2 antagonist (or “*p53* activator”) nutlin-3 (5 μM) and the CDK4/6 inhibitor palbociclib (2 μM), we showed that the percentage of surviving cells after nutlin or palbociclib following *Zfp36* loss did change, and thus the aggressive phenotype mediated by *Zfp36* loss that we described was independent of the functional loss of *Rb1* or *Tp53* Supplemental Figure 5B). In addition, genotyping and deep sequencing analysis of *Pten^f/f^* and *Pten^f/f^* *Zfp36^f/f^* dorsolateral and ventral prostate (DLVP) tissue showed no significant variants in the exonic region of Rb1 and Trp53 genome between both models (Supplemental Figure 5D and Supplemental Figure 6, A and B).

### TTP loss increases sensitivity of Pten-null tumors to NF-κB inhibition.

With the evidence that the phenotypic effects of Zfp36 loss in *Pten*-null tumors are associated with increased NF-κB activity, we sought to determine whether an oral NF-κB inhibitor, dimethylaminoparthenolide (DMAPT) (11, 41, 42), could (a) demonstrate effect as monotherapy in vivo in tumors with *Zfp36* loss, and (b) resensitize tumors with *Zfp36* loss to castration. Allograft in vivo models were generated by subcutaneous injection of GEMM-derived *Pten^f/f^* *Zfp36^f/f^* cells into C57BL/6N mice. Surgical castration (1,500 mm^3^ mean end point tumor volume) had a minimal effect on tumor growth in comparison with vehicle-treated (2,056 mm^3^) mice (Figure 7, C and D). Treatment with DMAPT (100 mg/kg/d) alone (772.5 mm^3^) or in combination with surgical castration (717.9 mm^3^) reduced tumor growth by 63.4%–65.1% compared with vehicle. Castration and/or DMAPT treatment did not significantly affect mouse weight over the treatment period (Supplemental Figure 7). Similarly, in vitro, GEMM-derived *Pten^f/f^* *Zfp36^f/f^* organoids showed a significant cell death response to DMAPT (5 μM) both alone and in combination with enzalutamide (5 μM) (Figure 7, E and F). To confirm that the effects of DMAPT are dependent on NF-κB activation in *Pten^f/f^ Zfp36^f/f^* cells, we silenced p65/NF-κB. This significantly reduced the antitumor response to DMAPT and concurrent sensitization to enzalutamide (Supplemental Figure 8). In addition to improving control of tumor growth and response to AR inhibition, treatment of *Pten^f/f^* *Zfp36^f/f^* PCa organoids with DMAPT partially reversed the phenotypic effects of *Zfp36* loss. This included reduction of CD45 and synaptophysin expression and increased AR expression and function (Figure 7, G and H). Jointly, these data confirm pharmaceutical inhibition of NF-κB as a viable strategy to treat and reverse therapy resistance in PCa.

## Discussion

Metastatic PCa remains lethal despite marked extension of overall survival with the addition of docetaxel, abiraterone, apalutamide, or enzalutamide to testosterone suppression (43–47). We have previously demonstrated that combinatorial loss of the tumor suppressor genes *PTEN*, *RB1*, and/or *P53* drives phenotypic plasticity, metastatic progression, and resistance to AR signaling inhibition (ARSI) (32, 33). Moreover, single-cell RNA-Seq analysis using these mouse models of phenotypic plasticity (32) suggests that the induction of inflammatory response pathways is associated with multilineage potential of tumor cells (48). In the context of hormone-sensitive metastatic PCa, *PTEN* is a well-described tumor suppressor gene whose loss occurs early in about 40% of patients (40, 49). Based on the premise that concurrent loss of 2 or more tumor suppressor genes including *PTEN*, *P53*, and *RB1* is associated with more aggressive metastatic PCa (31, 32), and *P53* loss and *RB1* loss are often late events first noted in castration-resistant disease, we asked whether an alternate route of phenotypic plasticity could be activated in the context of *PTEN* loss and potentially independent of *P53* and *RB1* loss.

Resistance to ARSI can be mediated by both nuclear NF-κB localization/activation (50, 51) and phenotypic plasticity with neuroendocrine differentiation (52, 53). Both events predict poor prognosis for patients, but their precise contribution to PCa progression is unknown. We recently demonstrated that neoadjuvant ARSI treatment of high-risk PCa patients resulted in significant enrichment of NF-κB signaling and a gene signature associated with neuroendocrine-like disease state (36). Also, we previously identified *ZFP36* as a key regulator of NF-κB signaling (14) and as such hypothesized that *ZFP36* loss may drive PCa progression by creating a pro-proliferative, inflammatory tumor environment, and features of phenotypic plasticity. In recent years, independent research groups have suggested that *ZFP36* functions as a tumor suppressor since its expression is suppressed in various tumor types compared with normal tissues. In both breast and PCa, low *ZFP36* expression is a negative prognostic indicator, and it is associated with rapid tumor progression and poor clinical outcome (16, 54). Here, we confirmed that *ZFP36* is biologically relevant in PCa, resulting in increased tumor progression and resistance to ARSI in *PTEN*-null patients and tumor models. Additionally, loss of *ZFP36* in normal epithelial cells results in cell transformation substantial enough to develop prostatic hyperplasia and PIN, a precursor to prostate adenocarcinoma. This implies that a “second hit,” such as loss of *PTEN*, appears to be required to drive prostate cells to cancer. We have previously reported upregulation of NF-κB/p65 in PCa following castration (11), and analysis of DARANA patient tumors (Figure 4A) confirmed increased activation of inflammatory response genes, including NF-κB, in post-enzalutamide samples. In fact, TNF-α signaling via NF-κB was the top positive enrichment from RNA-Seq of post-enzalutamide tumor samples (Figure 4A). The matching upregulation of *ZFP36*/TTP in these post-enzalutamide tumors (Figure 2) clearly highlights the functional role of TTP to negatively regulate NF-κB–driven inflammation (Figure 7I). Examination of the NCI neoadjuvant ADT tumor dataset demonstrates that *ZFP36* expression is not only elevated upon enzalutamide treatment, but also differs between responders and non-responders (Figure 2C). Despite a global increase in *ZFP36* expression, the lowest levels of *ZFP36* activation after treatment were observed in patients with the greatest residual tumor burden, highlighting the clear effect that functional TTP appears to be having on response to therapy. We suggest that this points to the importance of functional TTP feedback pathways in therapeutic response of PCa. In the absence of functional TTP (in GEMMs and GEMM-derived models) inflammation is uncontrolled and GSEA Hallmark analysis looks remarkably like that of post-enzalutamide tumors (Figure 4A).

Where tumor development was already being driven by loss of the tumor suppressor Pten, additional loss of Zfp36 resulted in a pronounced increase in PCa progression in vivo. Although *Pten^f/f^ Zfp36^+/f^* and *Pten^f/f^ Zfp36^f/f^* mice did not develop lethal macrometastatic disease, local and distant dissemination (micro-metastasis) was observed in a subset of mice harboring Zfp36 loss. We attribute this lethal phenotype, at least partially, to the induction of phenotypic plasticity. While the enrichment of nervous system development gene sets is noted, inflammation and leukocyte cell identity and function gene sets are more significantly enriched, highlighting the ability of PCa cells to activate a process termed lymphocyte mimicry. Cancer cell lymphocyte (immune) mimicry has recently been described as a distinct cancer hallmark (55) and refers to tumor cells taking on immune-like genetic profiles or bulk tumors trafficking in greater numbers of immune cells (56, 57). Our data demonstrate that activation of lymphocyte mimicry by loss of *Zfp36* is tumor cell autonomous, supporting the concept of tumor cell phenotypic plasticity. While uncommon, previous research has noted the presence of solid tumor cells expressing both epithelial and leukocyte markers (58, 59). It had been previously noted that lung cancer cells could activate a lymphocyte mimicry program by adoption of gene activation normally restricted to lymphocytes. These attributes were noted to include anchorage-independent mobilization, chemokine response, and modulation of local inflammatory conditions. It was observed that these lung cancer cells gained this ability via the upregulation of the lymphocyte-restricted transcription factor and chromatin regulator Aiolos (gene *IKZF3*) (60). It was later shown that Aiolos cooperated with STAT3 to drive chemokine receptor expression and promote breast cancer metastasis (61). Further, it has been reported that chromosomally unstable cancer cells can engage in a form of lymphocyte mimicry to avoid cell death. In these cancer cells, chronic activation of cytosolic DNA sensing pathways, including STING, resulted in altered interferon signalling and upregulation of NF-κB activity. The cells substituted a lethal epithelial response to inflammation with that of myeloid-derived cells, resulting in increased metastasis (62).

These results confirm that *ZFP36* is an important component driving prostate cell identity, and that loss of *ZFP36* results in sustained activation of alternate transcriptional programs. We and others have previously demonstrated the role that NF-κB plays in modulating response to ADT (3, 11, 63). The data presented here clearly demonstrate that loss of *ZFP36* increases resistance to anti-androgen therapy, at least in part because of unregulated NF-κB activation. Further, our findings suggest that loss of *ZFP36* occurs in localized disease and is sufficient to induce multiple inflammatory pathways and phenotypic plasticity. This highlights the biological importance of *ZFP36* in PCa and provides the scientific basis to develop a new therapeutic strategy using NF-κB inhibitors to treat patients who present with PCa with low or absent *ZFP36* expression.

## Methods

### Sex as a biological variable.

Our study exclusively examined male mice because the disease modeled is only relevant in males.

### Identification of ZFP36 gene signature.

We defined a *ZFP36* gene signature by identifying differentially expressed genes (DEGs) between 2 groups. To this end, we first selected samples with high (upper 75%) and low (lower 25%) expression of *ZFP36* in The Cancer Genome Atlas Prostate Adenocarcinoma (TCGA PRAD) data (35). We then identified DEGs between *ZFP36*-high and -low groups using an integrated hypothesis testing method as previously reported (64). Briefly: (a) *T*-statistics, rank-sum statistics, and log_2_-median-ratio between the groups were computed for each gene. (b) Empirical distributions of the null hypothesis were estimated by calculation of the 3 statistics for the genes after random permutation of the samples 10,000 times. (c) For each gene, 3 *P* values of the observed statistics were computed using their corresponding empirical distributions of null hypothesis by 2-tailed test. (d) The 3 *P* values were combined into an overall *P* value using Stouffer’s method (65). (e) Lastly, we performed multiple testing correction through Storey’s method (66). DEGs were defined as the genes with a false discovery rate (FDR) less than 0.05 and absolute log_2_-median-ratio greater than 0.58 (1.5-fold). The *z* score method was used to quantify signature activation (51 upregulated genes in *ZFP36*-low group) and applied to localized PCa validation cohorts (TCGA PRAD [ref. 35]; Gulzar et al. [ref. 67]; Taylor et al. [ref. 34]; dichotomized by median *z* score) with available clinical outcomes data (biochemical recurrence or disease-free survival) using the Kaplan-Meier method, and *P* values were calculated by the log-rank test. The HR and upper and lower bound of 95% CI of previously published hormone-sensitive PCa cohorts associating TTP expression (by RNA profiling or IHC) with biochemical recurrence (15) were employed for meta-analysis using the meta function in STATA version 17.0 (StataCorp). Meta-analysis was also performed of 2 previously published cohorts (Health Professionals Follow-up Study and Physicians’ Health Study, ref.14; GenomeDx Decipher, ref. 68) correlating *ZFP36* loss with lethal PCa (PCa death or metastases).

### Fluorescent immunohistochemistry and spectral imaging in human tissue microarrays.

A retrospective cohort of 112 patients with localized PCa who underwent RP (40, 69) was annotated for clinicopathological features, disease outcomes, and follow-up. Archival formalin-fixed paraffin-embedded (FFPE) RP specimens were used to construct 3 tissue microarrays (TMAs). Fluorescent IHC (FIHC) for PTEN was performed using a multiplexed tyramide signal amplification method, with AMACR masking tumor epithelium and a DAPI counterstain. Staining, imaging, quantification, and summarization of PTEN expression were performed as previously described (40), with loss defined as the lower cohort quartile of combined cytoplasmic and nuclear expression. FIHC for TTP was performed for 1 hour using the same TMAs, with additional pan-cytokeratin and basal staining, with DAPI counterstain. The distribution of cytoplasmic TTP expression intensity was quantified at an individual cell level across all cores. Cases with a microarray core exhibiting lower-quartile TTP expression intensity in more than two-thirds of the tumor cells were deemed to have TTP loss. FIHC antibody details are outlined in Supplemental Table 1.

To examine the clinical effect of compound PTEN and TTP loss, the lower quartile of normalized RNA expression of PTEN and TTP in TCGA PRAD (35) and Taylor et al. (34) cohorts defined loss of expression. For these cohorts and the Dana Farber Cancer Institute (DFCI) FIHC cohort, the PSA-based endpoint of disease-free survival was estimated using the Kaplan-Meier method and the Cox proportional hazards model estimated the hazard ratio (HR) and 95% confidence interval (95% CI); *P* values for association tests between gene expression and endpoints were calculated using the log-rank test. In the DFCI FIHC cohort, multivariable analysis was performed adjusting from Gleason score, tumor stage (T stage), and PSA level at diagnosis.

### DARANA clinical data.

Patients with localized PCa were enrolled in the DARANA (Dynamics of Androgen Receptor Genomics and Transcriptomics After Neoadjuvant Androgen Ablation) study (ClinicalTrials.gov NCT03297385) before RP. Patients were treated with neoadjuvant enzalutamide for 3 months before prostatectomy, with tumor biopsies taken before and after treatment. Gene expression (RNA-Seq) and ChIP-Seq tumor data were generated as described in ref. 36. For IHC analysis, the enzalutamide-treated patient cohort (*n* = 50) was matched in a 1:2 ratio to untreated control patients (not receiving enzalutamide before prostatectomy; *n* = 109) based on clinicopathological parameters (initial PSA, Gleason score, TNM stage, age) using the R package MatchIt (v4.1.0) (70). TMAs were prepared containing 3 cores per FFPE tumor sample. Tumor-dense areas in FFPE megablocks were marked by an expert pathologist on an H&E slide. TMAs were stained with antibody for 60 minutes at room temperature. Bound antibody was detected using the OptiView DAB Detection Kit (Ventana Medical Systems), and slides were counterstained with hematoxylin. Antibody details are listed in Supplemental Table 1. TTP staining intensity (negative, low, high) in tumor cells was scored by an expert pathologist.

### National Cancer Institute clinical data.

Patients with intermediate- to high-risk PCa were enrolled in a clinical trial of 6 months of neoadjuvant ADT plus enzalutamide (71, 72) prior to RP. Usable RNA was obtained from paired pre- and post-treatment tissues (including complete pathological responders) from 36 patients, using the biopsy (pretreatment) or whole-mount prostatectomy (post-treatment) block best corresponding to the index lesion visualized by multiparametric MRI. Usable baseline biopsy RNA was available from 37 patients. RNA was extracted from ribbon curls without microdissection using the QIAGEN FFPE RNeasy kit. Paired-end RNA-Seq libraries were generated using the Illumina TruSeq Stranded Total RNA kit with Ribo-Zero/Globin depletion. Raw RNA-Seq reads were deposited to the Database of Genotypes and Phenotypes (dbGaP; phs001938.v3.p1), and processed data were deposited to the Gene Expression Omnibus database (GEO GSE183100).

### Generation of GEMM models.

Prostate-specific *Pten*/*Zfp36*-knockout mice were achieved by crossing of the *loxP*-flanked *Pten* mice (*Pten^f/f^*) (73) with *loxP*-flanked *Zfp36* mice (*Zfp36^f/f^*) (74) and mice expressing Cre recombinase under the control of the rat probasin gene promoter, which is specific for cells of the epithelial cells of the prostate (PB-Cre4) (38). Homozygous *Pten^f/f^* mice and PB-Cre4 mice on a C57BL/6 background were purchased from The Jackson Laboratory (32), and homozygous *Zfp36^f/f^* mice were a gift from Perry Blackshear (National Institute of Environmental Health Sciences, Durham, North Carolina, USA). Heterozygous matings, male PB-Cre4 *Pten^+/f^* *Zfp36^+/f^* crossed with female *Pten^+/f^* *Zfp36^+/f^* mice, were used to generate prostate-specific *Pten*/*Zfp36*-knockout mice and their littermate wild-type (WT) control mice. All experimental mice had *Pten* homozygous deletion, while *Zfp36* status was examined in WT (PB-Cre4 *Pten^f/f^* *Zfp36^+/+^*), heterozygous (PB-Cre4 *Pten^f/f^* *Zfp36^+/f^*), or homozygous (PB-Cre4 *Pten^f/f^* *Zfp36^f/f^*) states (Supplemental Figure 1A). Littermate male mice carrying floxed *Pten* and *Zfp36* alleles but lacking the PB-Cre4 transgene were used as WT controls.

For PCR genotyping, genomic DNA from ear-notch clips was extracted by alkaline lysis. Tissue samples were incubated in 75 μL of 25 mM NaOH/0.2 mM EDTA at 95°C for 30 minutes, cooled, and mixed with 75 μL of 40 mM Tris-HCL (pH 5.5) to neutralize the reaction. All PCR reactions were run using 2 μL of extracted DNA in GoTaq PCR Master Mix (Promega) and the following cycling protocol: 95°C for 2 minutes; 35 cycles of 95°C for 30 seconds, 60°C for 60 seconds, and 72°C for 90 seconds; and 72°C for 5 minutes. PCR products were visualized on a 1.8% agarose gel (in 1× TAE buffer, 20 mM Acetate and 1 mM EDTA [TAE] buffer) containing 1× SYBR Safe DNA dye (Invitrogen).

The PB-Cre transgene was detected using the primer pair PbF1 and Cre4R2 to generate a transgene-positive (331 bp) amplicon. *Pten*-floxed alleles were detected using the primer pair PtenF and PtenR to generate WT (124 bp) or floxed (321 bp) amplicons. *Zfp36*-floxed alleles were detected using the primer pair Zfp36F1 and Zfp36R2 to generate WT (327 bp) or floxed (514 bp) amplicons (Supplemental Figure 1B).

Recombination of the *Pten* allele was examined using the primer pair PtenDeltaF1 and PtenDeltaR2, which amplified deleted *Pten* alleles (397 bp). Recombination of the *Zfp36* allele was examined in multiplex reactions using Zfp36F3, Zfp36R2, and Zfp36R4 primers, which amplified the WT (683 bp), floxed (870 bp), or deleted *Zfp36* alleles (769 bp) (Supplemental Figure 1B). All primers are described in Supplemental Table 2.

### Aging GEMM studies.

To examine the role of TTP in PCa driven by *Pten* loss, male experimental mice were generated and allowed to age to 8 weeks, 18 weeks, or 38 weeks or until an ethical endpoint was reached (prostate tumor > 15 mm diameter assessed by palpation, or decline in animal health), as determined by Dana-Farber Cancer Institute IACUC and veterinarian stipulations. All aging mice were monitored daily for signs of declining health and manually palpated for prostate tumor development once a week until tumors were detected and then daily following palpation of a prostate tumor. For castration studies, mice received surgical or sham castration at 38 weeks of age and allowed to progress to 50 weeks of age or until a humane endpoint was reached. After necropsy, whole genitourinary, whole prostate, and individual prostate lobe weights were measured, and tissues collected. Pelvic lymph nodes and kidney, liver, and lung tissue samples were collected for assessment of metastatic dissemination. Tissue samples were collected for FFPE blocks and, where possible, digested and stored in cryopreservation media (10% DMSO in Advanced DMEM/F12, Gibco, Thermo Fisher Scientific) or snap-frozen.

### GEMM tumor RNA-Seq analysis.

The quality of sequenced reads from the RNA-Seq data was assessed, and low-quality reads were filtered using the FastQC tool (Babraham Bioinformatics). Sequence alignment and quantification were performed using the STAR-RSEM pipeline (75, 76). Reads overlapping exons in the annotation of Genome Reference Consortium Mouse Build 38 (GRCm38) were identified. To reduce systemic bias between samples, the Trimmed Mean Method was applied to gene-level expression counts (77). Genes were filtered out and excluded from downstream analysis if they failed to achieve raw read counts of at least 2 across all the libraries. For the functional enrichment analysis, we used gene set enrichment analysis (GSEA) software (78). Briefly, (a) the Hallmark and Gene Ontology Biological Process gene sets were obtained from MSigDB (79), (b) log_2_-median-ratio was used to order genes in the data in a descending manner, (c) enrichment score (ES) was computed using Kolmogorov-Smirnov running sum statistics for each gene set, and (d) significance of the ES was computed using a distribution of null hypothesis, which was generated by doing 1,000 random permutations.

### Deep sequencing and identification of Trp53 and Rb1 gene variants.

The genomic DNA was extracted by DNeasy Blood & Tissue kit (QIAGEN, catalog 69504). The genomic DNA was processed by Nextera XT DNA Library Preparation Kit (Illumina). Quality control was done using the standard routine pipeline at the Massachusetts Institute of Technology (MIT) BioMicroCenter before sequencing. An S4 lane was used to sequence no more than 5-sample pooled libraries on an Illumina NovaSeq 6000 platform with a 150 + 150 bases paired-end run and 10 + 24 nucleotide indexes. The whole-genome sequencing reads were mapped to Trp53 and Rb1 references downloaded from the UCSC Genome Browser from an mm10 referencing genome using bwa/0.7.17 to select relevant sequences for downstream computing. The mapped sequences were selected using samtools/1.10 –F 4 and were converted to FASTQ files using samtools/1.10 fastq. The mapped FASTQ files ere then aligned to mm10 reference using bwa/0.7.17. The duplicate sequences were marked using Picard 2.18.26 MarkDuplicates (https://broadinstitute.github.io/picard/). The variations were called using samtools/1.10 mpileup and then subset to exonic regions using samtools/1.10 tabix. The variation comparison between different samples was performed using MIT informatics core in-house tools. The BAM files were visualized, and the variants identified in the exonic regions were confirmed by Integrative Genomics Viewer.

### CRISPR-knockout cell lines and RNA-Seq.

For CRISPR/Cas9–mediated knockout cell line generation, guide RNA (gRNA) sequences CATGACCTGTCATCCGACCA,AAGCGGGCGTTGTCGCTACG (targeting murine Zfp36) and GAGCTCGGTCTTGTATCGAG (targeting human ZFP36), were cloned into the lenti-CRISPR/Cas9v2 vector (Addgene, 52961) according to the protocol of the Zhang laboratory (80). The scrambled gRNA sequence CACCGCGTGATGGTCTCGATTGAGT was used as a negative control. Viral infection was performed as described by the RNAi Consortium (Broad Institute) laboratory protocol “Lentivirus production of shRNA or ORF-pLX clones,” and single clones were isolated following puromycin selection (2 μg/mL; Sigma-Aldrich, P8833). Parental PrEC-957E/hTERT-AR cells were a gift from John Isaacs (Johns Hopkins University, Baltimore, Maryland, USA). RNA-Seq data were aligned with STAR to mouse reference genome mm10 (GRCm38), quantified using RSEM, and normalized. GSEA was performed in GenePattern (genepattern.org). Genotypes were compared as described, using 10,000 gene set permutations to generate normalized enrichments scores, with FDR *q* value less than 0.25 considered significant.

### Immunohistochemical and immunofluorescent staining and quantification of GEMM tissues.

For all staining, 4-μm-thick sections were cut from FFPE blocks and dried onto positively charged microscope slides. All antibodies used are listed in Supplemental Table 1.

For IHC, tissue sections were stained using the ImmPRESS HRP Anti-Mouse IgG (Peroxidase) Polymer Detection Kit (Vector Laboratories) as previously described (81). Tissue sections were imaged using an EVOS FL Auto 2 Cell Imaging microscope (Thermo Fisher Scientific). Images were deidentified, and 50 random fields per section were run through QuPath image analysis software (82) to quantify positive DAB-stained cells (as a percentage of total cells).

For immunofluorescence, tissue sections were prepared using standard deparaffinization, rehydration, and sodium citrate antigen retrieval methods. Sections were blocked with 5% goat serum in PBST for 1 hour at room temperature, followed by incubation with primary antibody diluted in 5% goat serum/PBST overnight at 4°C. Where a conjugated primary antibody was used, slides were washed in PBST for 2 minutes 4 times and coverslipped with Vectashield DAPI mounting medium. Where a non-conjugated primary antibody was used, slides were washed in PBST for 2 minutes 4 times and blocked with 5% goat serum/PBST for 15 minutes at room temperature. Sections were incubated with the appropriate fluorescent secondary antibody diluted in 1% goat serum/PBST for 1 hour at room temperature, then washed in PBST for 2 minutes 4 times. Slides were coverslipped with Vectashield DAPI mounting medium (Vector Laboratories). For analysis, 20 representative images from each tissue section were taken using an EVOS FL Auto 2 Cell Imaging microscope. Staining intensity and area were scored using analysis pipelines generated for CellProfiler software (83).

Multiplex IHC based on tyramide signal amplification was performed using an OPAL 7-color Manual IHC kit (Akoya Biosciences, catalog OP-000003KT). Briefly, slides were deparaffinized, rehydrated by graded ethanol, and fixed 30 minutes in 10% neutral-buffered formalin. First and subsequent antigen retrieval were performed using Opal AR6 buffer (Akoya Biosciences) in a microwave at 200 W for 15 minutes. Endogenous peroxidase activity was blocked using 3% hydrogen peroxide, and nonspecific binding sites were blocked using Antibody Diluent/Block buffer (Akoya Biosciences). Antibodies used are listed in Supplemental Table 1. After incubation with Opal Polymer HRP Ms+Rb (Akoya Biosciences), fluorescence signals were visualized using Opal fluorophores (Akoya Biosciences; dilution 1:100). The staining was performed in the following order: AR (first position, Opal 570), Syp (second position, Opal 690), Epcam (third position, FITC-conjugated). All slides were mounted in DAPI Fluoromount-G medium (SouthernBiotech, catalog 0100-20). Full tissue sections were scanned using an EVOS FL Auto 2 Cell Imaging microscope (Thermo Fisher Scientific) and run through QuPath image analysis software (56) to quantify positive stained cells.

For trichrome staining, tissue sections were stained using the Trichrome Stain Kit (Connective Tissue Stain) (Abcam, ab150686) according to the manufacturer’s instructions. Tissue sections were imaged using an EVOS FL Auto 2 Cell Imaging microscope. Images were deidentified, and 50 random fields per section were run through QuPath image analysis software (82) to quantify positive reactive stroma-stained area (as a percentage of total cells analyzed).

### Generation of GEMM-derived 2D and 3D cell lines.

Organoid (3D) models were generated from GEMM prostate tissue using previously described methods (84). To generate 2D cell lines, organoid cultures were allowed to grow as a single layer in 2D in standard organoid culture medium. Over multiple passages, organoid culture medium was replaced in a stepwise process with standard DMEM supplemented with 10% FBS and passaging until phenotypical and growth rate stability was achieved.

### Organoid budding assay.

To prepare for assays, organoid cultures were seeded as single cells in 40 mL Matrigel droplets in 6-well tissue culture plates and cultured for 2 days at 37°C to allow organoid formation. After organoid formation, medium was aspirated, and Matrigel droplets were manually detached from tissue culture plates and incubated with dispase II (Thermo Fisher Scientific, 17105041) at a final concentration of 1–2 mg/mL in Advanced DMEM/F12 (Thermo Fisher Scientific, 12634028) for 30 minutes with gentle shaking at 37°C to remove whole organoids from the Matrigel. Organoids were gently washed twice with PBS and resuspended in 30 mL 33% Matrigel (in Advanced DMEM/F12) at a concentration of 10–20 organoids per well in 96-well tissue culture plates. Starting 24 hours after replating, daily bright-field images of each organoid were taken using an EVOS FL Auto 2 Cell Imaging microscope. Organoid budding was assessed using an image analysis pipeline generated for CellProfiler software (83) to identify the organoid area deviating beyond the primary sphere shape.

### 2D wound healing assay.

GEMM-derived 2D cells (from PB-Cre4 *Pten^f/f^* and PB-Cre4 *Pten^f/f^* *Zfp36^f/f^* mice, as well as a previously described *Pten*/*Rb1*-null murine cell line) (32) were seeded into 24-well tissue culture plates such that they would reach confluence after 48 hours. Once the cultures were confluent, a wound was generated by drawing of a sterile p1000 tip gently across the center of each well. Each well was gently washed with PBS to remove all loose cells and debris. Tissue culture plates were subsequently imaged daily using an EVOS FL Auto 2 Cell microscope, and wound healing was assessed using the ImageJ Wound Healing Assay macro (https://biii.eu/wound-healing-assay-analysis-imagej) (NIH).

### 2D cell cycle assay.

GEMM-derived 2D cells (from PB-Cre4 *Pten^f/f^* and PB-Cre4 *Pten^f/f^ Zfp36^f/f^* mice) were seeded into 6-well tissue culture plates such they would reach confluence after 48 hours. Once the cultures were confluent, cells were harvested and stained with propidium iodide. Cells were then analyzed by flow cytometry using 493 nm excitation.

### Organoid growth assessment.

For assays, organoid cultures were seeded sparsely as single cells in 40 mL Matrigel in 96-well tissue culture plates and cultured for 2 days at 37°C to allow organoid formation. After organoid formation, daily bright-field images of individual organoids were taken using an EVOS FL Auto 2 Cell Imaging microscope. Organoid area was measured from images using CellProfiler software (83) and organoid growth assessed as a daily fold change in organoid area relative to day 0. For each cell line and treatment condition, 10 individual organoids were measured.

### Organoid therapy assay.

Organoids were established as described above for growth assessment assays. Once formed, organoids were treated with vehicle (0.1% DMSO), 10 μM enzalutamide (MedChemExpress), 5 μM dimethylaminoparthenolide (DMAPT), or the combination of enzalutamide and DMAPT for 72 hours. After treatment, cells were incubated with ReadyProbes Cell Viability Imaging Kit (Blue/Green, Invitrogen) per well, as per the manufacturer’s instructions, for 30 minutes at room temperature, and *Z*-stack images of stained cells were taken using an EVOS FL Auto 2 Cell Imaging microscope. The percentage of cell death was calculated by identification of the percentage of non-viable cells per organoid in at least 10 organoids for each treatment condition.

### In vivo therapy experiment.

Experiments were carried out on 10-week-old male C57BL/6 mice (The Jackson Laboratory). Allograft models were generated by subcutaneous injection of 2 × 10^6^ GEMM-derived 2D cells (from PB-Cre4 *Pten^f/f^* and PB-Cre4 *Pten^f/f^* *Zfp36^f/f^* mice) per animal in 100 μL Matrigel (50% in PBS). Tumors were allowed to establish and grow to approximately 100 mm^3^ before being randomly allocated to vehicle (water), surgical castration, DMAPT (100 mg/kg/d by oral gavage), or castration plus DMAPT combination. All non-castrated treatment groups also received a sham castration. Tumor volume and animal weight were measured every 2 days. Tumor volume was measured by caliper and expressed in cubic millimeters [tumor volume = 0.5(*a* × *b*^2^), where *a* and *b* represent the long and short diameter, respectively]. Treatment toxicities were assessed by body weight, decreased food consumption, signs of dehydration, hunching, ruffled fur appearance, inactivity, or non-responsive behavior.

### Quantitative reverse transcription PCR.

The quantitative PCRs were performed in accordance with Minimum Information for Publication of Quantitative Real-Time PCR Experiments guidelines (85). RNA was harvested using a standard TRIzol (Thermo Fisher Scientific, 15596018) protocol according to the manufacturer’s instructions. Complementary DNA was synthesized using the High-Capacity cDNA Reverse Transcription Kit (Thermo Fisher Scientific, 4368813) according to the manufacturer’s instructions. The iTaq Universal SYBR Green Supermix (Bio-Rad, 1725120) was used for PCRs with the cycling conditions recommended in the manufacturer’s instructions. The primers used are detailed in Supplemental Table 2.

### Western blot.

Subconfluent treated cells were washed twice with cold PBS, trypsinized, and then lysed in Pierce RIPA buffer (Thermo Fisher Scientific, 89900) with PhosSTOP inhibitor cocktail (Sigma-Aldrich, PHOSS-RO) at 4°C for 30 minutes. Protein concentrations were measured by a Pierce BCA Protein Assay Kit (Thermo Fisher Scientific, 23225). Proteins were separated by SDS-PAGE (10% Mini-PROTEAN TGX Precast Gel, Bio-Rad, 4561036) and transferred to a PVDF membrane (Bio-Rad, 1620177). The membrane was blocked in 3% BSA in TBST for 1 hour at room temperature and then blotted with primary antibodies overnight at 4°C.

### Statistics.

Data are represented as mean ± SD (unless otherwise indicated). Numbers (*n*) of mice or biological replicates and *P* values are shown in figure legends. For comparison of groups, 1-way ANOVA with post hoc Tukey’s testing (unless otherwise indicated) or 2-tailed, unpaired Student’s *t* tests were used as indicated in figure legends. For comparison of patient responses before and after enzalutamide treatment, 2-tailed paired-sample *t* tests were used for analysis. Tumor growth curves were analyzed using multiple 2-tailed unpaired *t* tests Survival data and time-to-ethical-endpoint GEMM data were analyzed using log-rank (Mantel-Cox) tests. *P* values less than 0.05 were considered as significantly different from the null hypothesis across all experiments as indicated by asterisks in all figures. Statistical calculations were carried out in Prism v9.0 (GraphPad Software) and Stata v15.1 (StataCorp). R, version 4.2.2, was used for bioinformatic analyses.

### Study approval.

All animal breeding and experiments were approved by and performed in accordance with the guidelines of the Dana-Farber Cancer Institute Institutional Animal Care and Use Committee (Animal protocol 19-005).

### Data availability.

For DARANA clinical data, all tissue ChIP-seq and RNA-seq raw data have been deposited in the European Genome-Phenome Archive (EGA) (EGAS00001006017 and EGAS00001006016, respectively). All processed tissue ChIP-seq and RNA-seq data have been deposited in the Gene Expression Omnibus (GEO) database (GSE197781). For NCI clinical data, raw RNA-seq reads have been deposited to dbGaP (phs001938.v3.p1) and processed data has been deposited to GEO (GSE183100). Individual data values for all figures are available in the Supporting Data Values file.

## Author contributions

LE, CJS, KLM, and BG conceptualized this project. LE, KLM, AAH, and BG established the methodology. KLM, AAH and BG conducted most of the experiments, data collection and analysis, with additional investigation and data collection by LE, JN, DB, AB, HvdP, IH, EB, ST, SW, AK, MK, JK, DM, JP, SY, and XA. KLM, AAH, SL, and BG visualized the graphs and figures for the manuscript. LE, CJS, KLM, and AAH wrote the original manuscript, and LE, CJS, KLM, AAH, AGS, WZ, SL,and BG reviewed and edited subsequent manuscript drafts. All authors read and approved the final version.

## Supplementary Material

Supplemental data

Unedited blot and gel images

Supplemental tables 1-7

Supporting data values

## Version 1 11/19/2024

In-Press Preview

## Version 2 01/16/2025

Electronic publication
