# Supplementary material for: Low tristetraprolin expression activates phenotypic plasticity and primes transition to lethal prostate cancer in mice
Source: J Clin Invest. 2024 Nov 19;135(2):e175680. doi: 10.1172/JCI175680 (PMC11735106; doi:10.1172/JCI175680)
Supplement: Supplemental data [file jci-135-175680-s263.pdf]

Figure S1

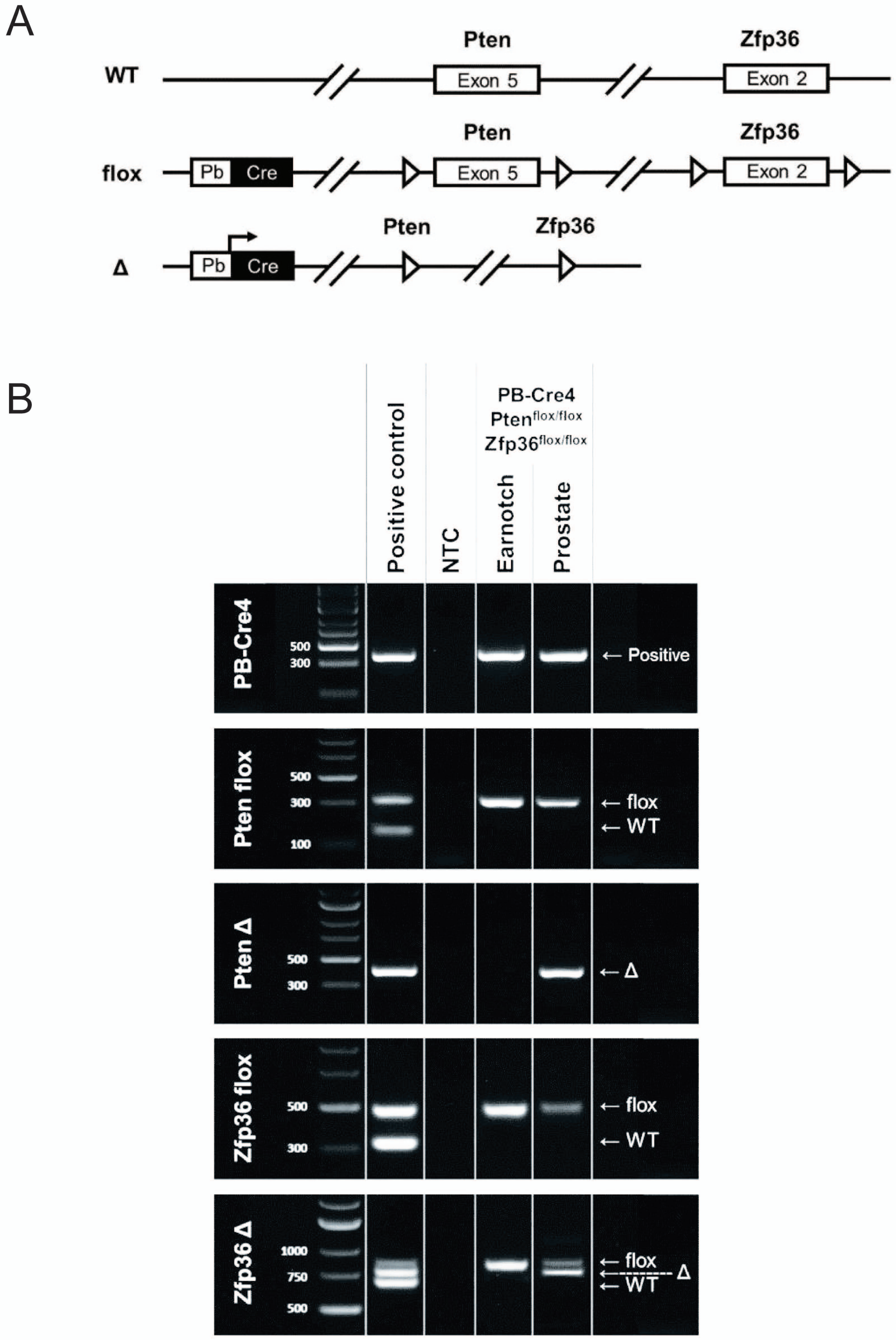

Supplementary Figure 1: Genetics of prostate specific Pten/Ttp deleted GEMM mice.

(A) Schema of generated GEMM alleles including wild-type (WT), floxed (flox) and recombined (Δ) alleles.

(B) Genotyping and recombination of PCR products from ear notch and prostate DNA samples of a PB-Cre4/Pten$^{f/f}$/*Zfp36*$^{f/f}$ mouse, highlighting PB-Cre4, Pten and *Zfp36* status.

Figure S2

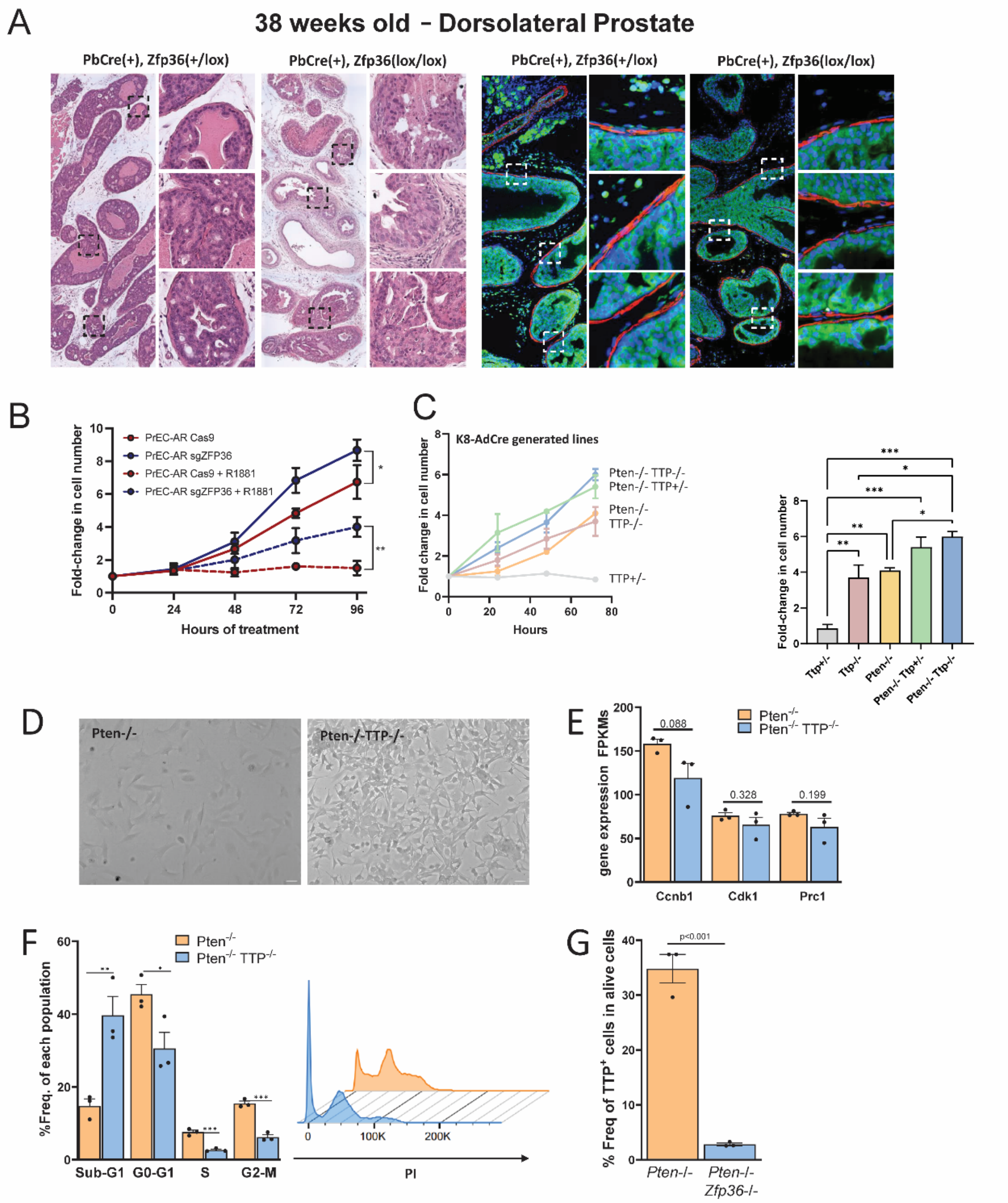

Supplementary Figure 2: Homozygous *Zfp36* loss results in progression to prostatic intraepithelial neoplasia.

(A) hematoxylin and eosin IHC and Krt18 (green) and alpha-SMA (red) IF staining of *Zfp36*+/- and *Zfp36*-/- murine dorsolateral prostate tissue 38 weeks.
(B) Proliferation of PrEC-AR cells with and without *ZFP36*, +/- R1881 stimulation.
(C) Proliferation and matching quantification of GEMM-derived 2D cell lines, n=3, *$p<0.05$, **$p<0.005$, ***$p<0.0005$ (one-way ANOVA with Tukey's post hoc).
(D) Representative image of GEMM-derived 2D cell lines.
(E) FPKMs values of Ccnb1, Cdk1 and Prc1 from bulk RNAseq analysis in GEMM-derived 2D cell lines. n=3 replicates per genotype (student t-test).

(F) Quantification and histogram of the flow cytometry analysis indicating the proportion of cells in the sub-G1, G1, S and G2-M phases of the cell cycle, n=3, *p<0.05, **p<0.005, ***p<0.001 (student t-test).
(G) Quantification by flow cytometry analysis of the TTP protein expression in Pten-/- and Pten-/-TTP-/- 2D cell lines, n=3, *p<0.05, **p<0.005, ***p<0.001 (student t-test).

Figure S3

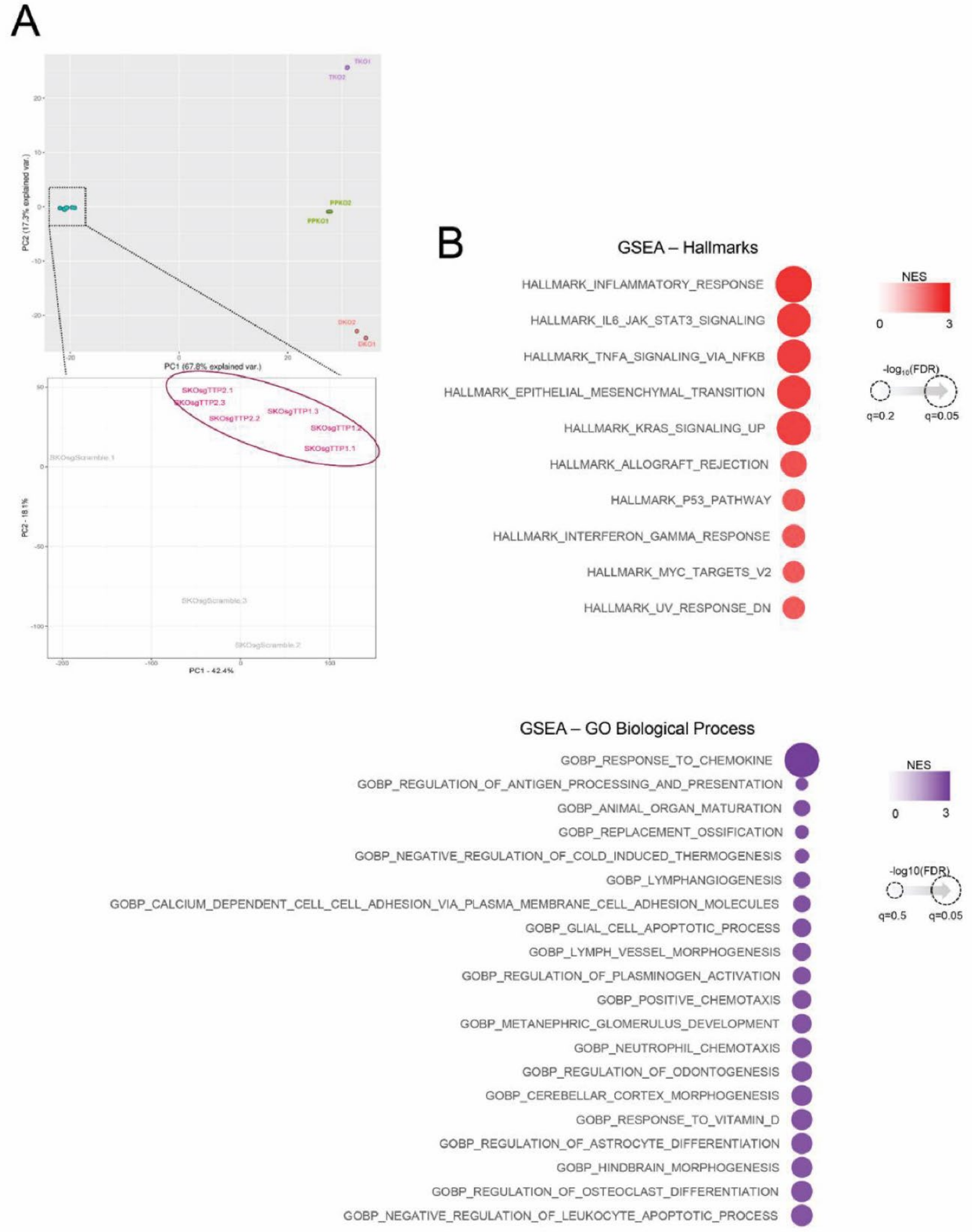

Supplementary Figure 3: Validation of in vivo RNA-seq results are tumor cell autonomous.

(A) Principal component analysis plot of GEMM-derived 2D cell lines demonstrating separation of SKO (PBCre4:*Pten*$^{f/f}$) and SKO-TTP (PBCre4:*Pten*$^{f/f}$:*Zfp36*$^{f/f}$) from PPKO (PBCre4:*Pten*$^{f/f}$:*p53*$^{f/f}$), DKO (PBCre4:*Pten*$^{f/f}$:*Rb1*$^{f/f}$) and TKO

(PBCre4:*Pten*$^{f/f}$:*Rb1*$^{f/f}$:*p53*$^{f/f}$) samples. SKO-TTP samples cluster and separate from SKO control.

(B) Gene set enrichment analysis of SKO-TTP versus SKO demonstrates significant enrichment for immune and inflammatory gene sets in the SKO-TTP group.

Figure S4

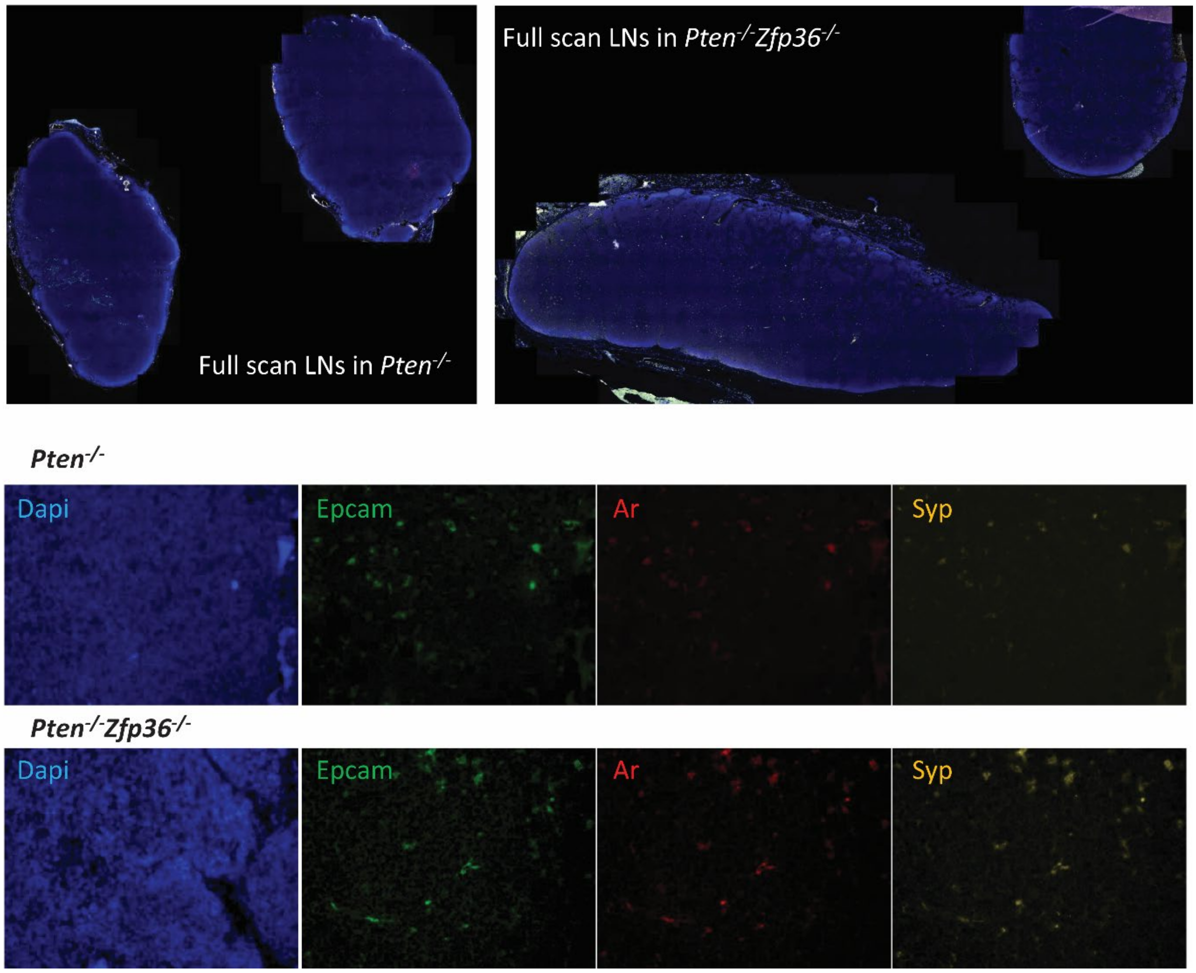

Supplementary Figure 4: Increased metastatic potential occurs with *Zfp36* loss *Pten*-null murine tumors.

A) Full scan images and representative images of the single staining's in the FFPE sections of the tumoral adjacent pelvic lymph nodes (LNs) in Pten$^{-/-}$ and Pten$^{-/-}$Zfp36$^{-/-}$ mice. LNs were stained by multiplex IHC for Epcam (green), Ar (red) and Synaptophysin, Syp (yellow). Positive cells identify epithelial/tumoral cells metastasizing LNs. These data have previously been presented in-part in Figure 5D as merged IF images.

Figure S5

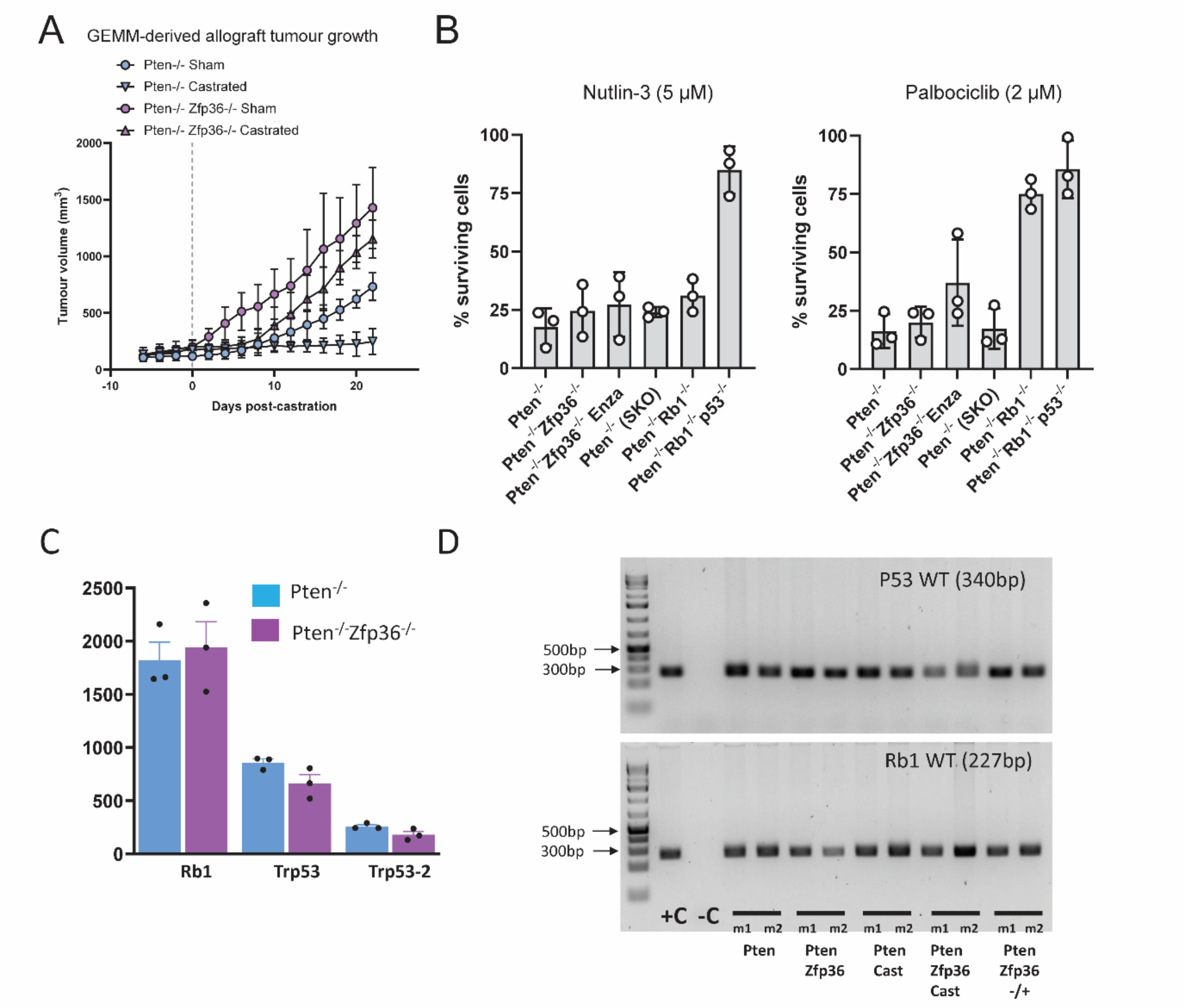

Supplementary Figure 5: Loss of *Zfp36* drives castration resistance independent of *Tp53* and *Rb1* loss of function.

(A) In vivo tumor growth in GEMM-derived allograft models following surgical castration or sham castration at 7 days post-tumor implantation, n=5 mice per group, +/-1SD.

(B) GEMM-derived 2D cell lines treated for 72 hrs with Nutlin-3 (5 µM) and Palbociclib (2 µM). GEMM-derived cell lines involving *Zfp36* loss generated for this manuscript were compared to previously published genetically defined murine models; $Pten^{f/f}$ (SKO), $Pten^{f/f}$:$Rb1^{f/f}$ (DKO), *and* $Pten^{f/f}$:$Rb1^{f/f}$:$p53^{f/f}$ (TKO) (32).

(C) Gene signature score for Rb1 and Trp53 in $Pten^{-/-}$ and $Pten^{-/-}Zfp36^{-/-}$ 2D cell lines. The Rb1 gene signature was generated from the data set from Chen W.S. et al (86), and Trp53 gene signatures were generated from the data set from Aubrey B.J et al (87).

(D) Genotyping PCR for Trp53 (WT band 340bp) and Rb1 (WT band 227bp). +c (positive control), -c (negative control, no DNA in the mix), m1: mouse #1, m2: mouse #2, Cast: castrated.

Figure S6

A

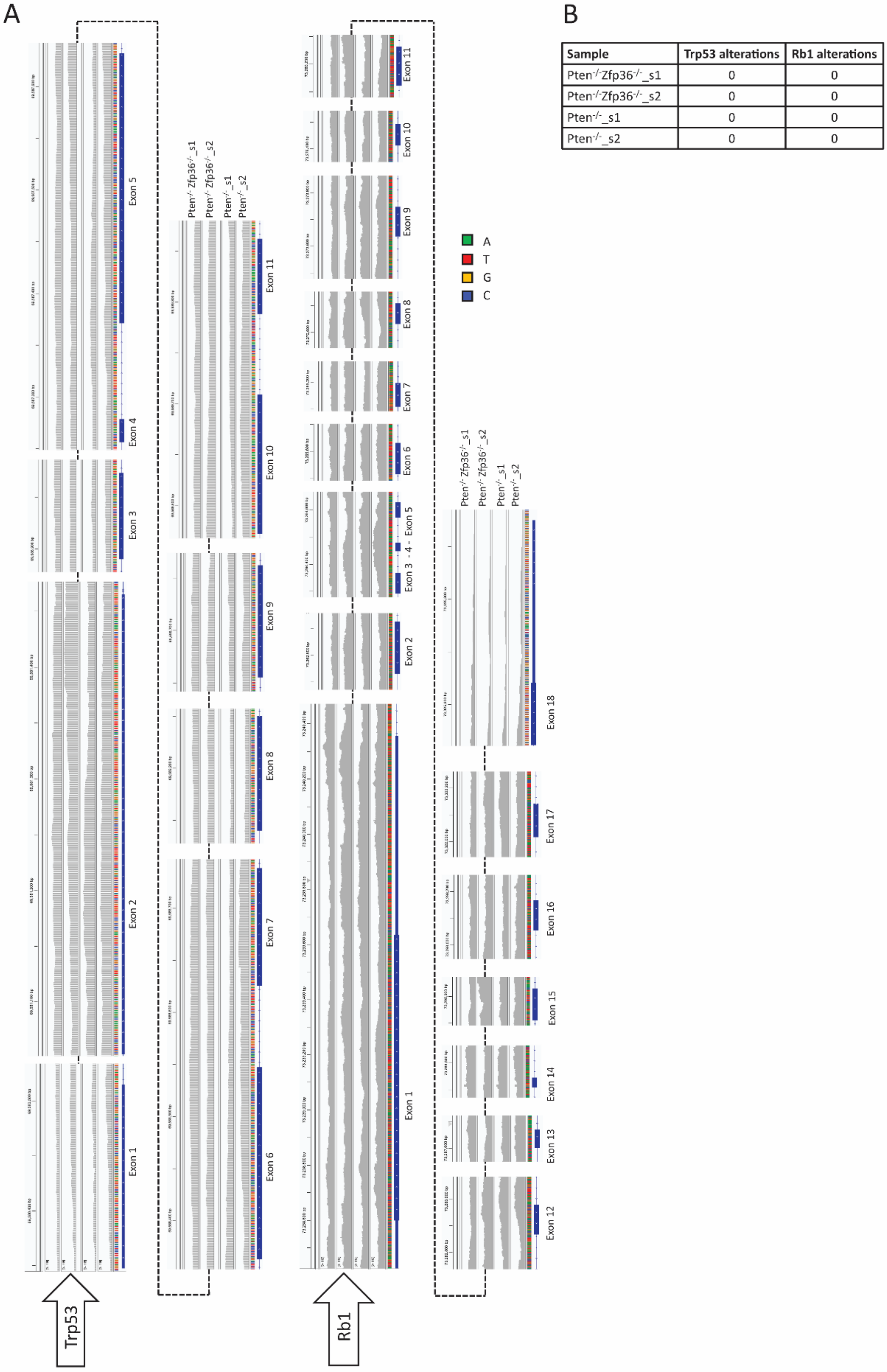

B

Sample	Trp53 alterations	Rb1 alterations
Pten$^{-/-}$Zfp36$^{-/-}$_s1	0	0
Pten$^{-/-}$Zfp36$^{-/-}$_s2	0	0
Pten$^{-/-}$_s1	0	0
Pten$^{-/-}$_s2	0	0

Supplementary Figure 6: Deep sequencing showed that mice with loss of *Zfp36* have present and intact *Tp53* and *Rb1* sequence.

(A) Track of the deep sequencing of Trp53 and Rb1 exonic regions indicating that no genomic variations were detected in the DLVP prostatic tissue of $Pten^{-/-}$ and $Pten^{-/-}$ $Zfp36^{-/-}$ mice.

(B) Table indicating that no variants were detected in the Trp53 and Rb1 exonic regions.

Figure S7

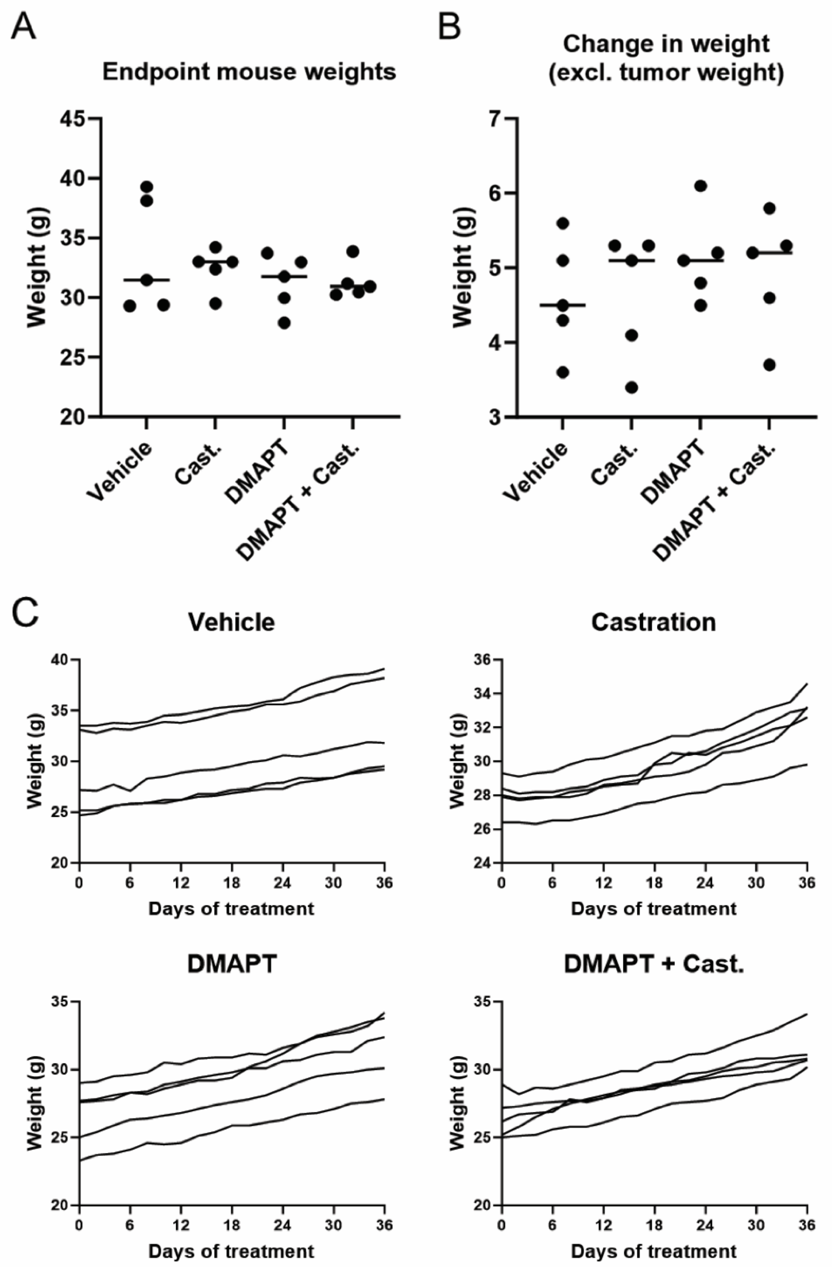

Supplementary Figure 7: Castration and/or DMAPT treatment does not significantly affect in vivo weight.

(A) Endpoint weights in vehicle, castrated, DMAPT and castration plus DMAPT treated *Zfp36/Pten*-null allograft mice.
(B) Change in mouse weight over the treatment period, excluding the final tumor weight.
(C) Individual mouse weights over the study period. No single or combination treatment significantly altered mouse weights over the entire study period.

Figure S8

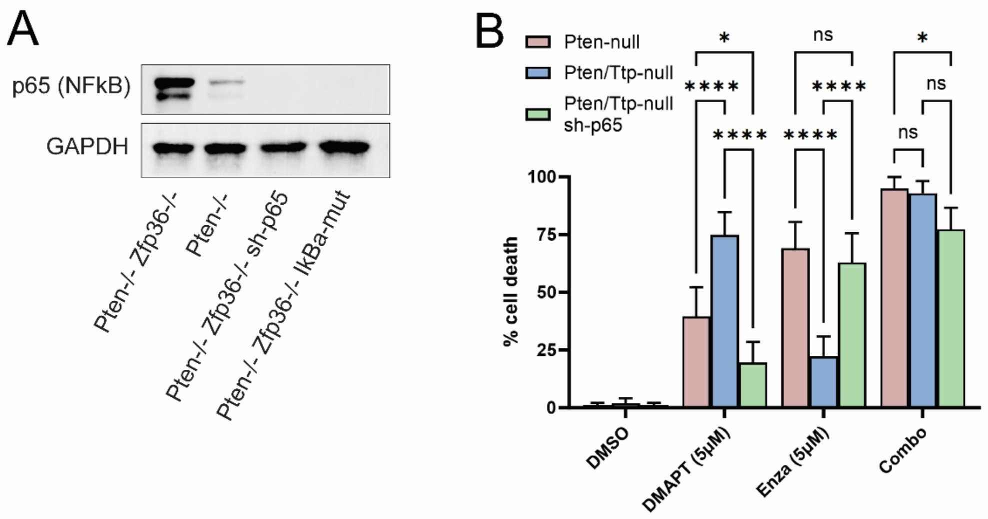

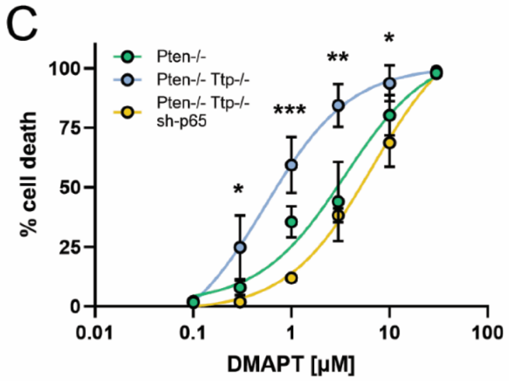

Cell line	LD50
Pten-/-	4.52 μM
Pten-/-Ttp-/-	0.76 μM
Pten-/-Ttp-/- sh-p65	8.66 μM

Supplementary Figure 8: Functional effects of p65 inhibition in TTP-null GEMM-derived cell lines.

(A) Phosphorylated p65 protein expression in parental and modified cell lines.

(B) Measurement of cell death in parental and modified cell lines treated with vehicle, DMAPT (5 μM), enzalutamide (5 μM) or the combination for 72 hours. Inhibition of p65 restores enzalutamide sensitivity and reduces efficacy of DMAPT. n=3, +/- 1SEM, n=3 per genotype, *p<0.05, ****p<0.0001 (two-way ANOVA with Fisher's LSD).

(C) DMAPT dose-response cell death curves for parental and modified cell lines, with corresponding LD50 values (μM). n=3 per genotype.

Supplementary Table 1

Antibody	Catalog Number	Source	Applicat	Concentrat
ZFP36 / Tristetraprolin (mouse)	LS-B1572-50	LSBio	WB	1/1000
			IF	1/100
ZFP36 / Tristetraprolin (human)	LS-B5606	LSBio	IF	1/200
ZFP36 / Tristetraprolin (human/DARANA)	ab119779	Abcam	IHC	1/100
PTEN	9188	Cell Signaling Technology	IF	1/100
Pan-CK	83957	Cell Signaling Technology	IF	1/200
p63	ab124762	Abcam	IF	1/200
Androgen Receptor	5153	Cell Signaling Technology	IF	1/200
Synaptophysin	ab52636	Abcam	IF	1/100
Ki-67	12075	Cell Signaling Technology	IHC	1/200
Cytokeratin 18 / Krt18	ab133263	Abcam	IF	1/100
Phospho-NFκB / p65	3033	Cell Signaling Technology	WB	1/1000
			IF	1/200
CD45	70257	Cell Signaling Technology	IF	1/100
α-Smooth Muscle Actin	36110	Cell Signaling Technology	IF	1/100
Anti-rabbit IgG (Alexa Fluor® 488 Conjugate)	4412	Cell Signaling Technology	IF	1/200
Epcam	ab237384	Abcam	IF	1/200
StarBright Blue 700 Goat Anti-Rabbit IgG	12004161	BioRad	WB	1/2500
StarBright™ Blue 520 Goat Anti-Rabbit IgG	12005869	BioRad	WB	1/2500
hFAB™ Rhodamine Anti-GAPDH Primary Antibody	12004168	BioRad	WB	1/2000

Supplementary Table 2

Primer name	Usage	Sequence
PbF1	GEMM genotyping	AGG CAA ATT TTG GTG TAC GG
Cre4R2	GEMM genotyping	GCA AAC GGA CAG CAG AAG CAT TT
PtenF	GEMM genotyping	CAA GCA CTC TGC GAA CTG AG
PtenR	GEMM genotyping	AAG TTT TTG AAG GCA AGA TGC
Zfp36F1	GEMM genotyping	GAA CCC TCT CTC GAT CGG GGA TAC
Zfp36R2	GEMM genotyping	GGA TGG AGT CCG AGT TTA TGT TCC AA
Zfp36F3	GEMM recombination	CTG GCT GGA AAT GAG AGA GG
Zfp36R4	GEMM recombination	CAC CCC TTA CGC CAG AAC TA
PtenDeltaF1	GEMM recombination	ACT CAA GGC AGG GAT GAG C
PtenDeltaR1	GEMM recombination	GCT TGA TAT CGA ATT CCT GCA GC
mGusb-F	qPCR housekeeping	CCG ACC TCT CGA ACA ACC G
mGusb-R	qPCR housekeeping	GCT TCC CGT TCA TAC CAC ACC
m_ZFP36_135pb_F	qPCR	TGTCGGACCTACTCAGAAAGCG
m_ZFP36_135bp_R	qPCR	CTGGAGGTAGAACTTGTGGCAG
m_p65_NFKB_F	qPCR	TGACCCCTGTCCTCTCACATCCG
m_p65_NFKB_R	qPCR	CAGCTCCCAGAGTTCCGGTT
FKBP5_Ex1_F	qPCR	GCGGCGACAGGTCTTCTA
FKBP5_Ex1_R	qPCR	GCTTTGATAACCTGGCCTTG
m_sgZfp36_1_oligo1	sgRNA design	CACCGCATGACCTGTCATCCGACCA
m_sgZfp36_1_oligo2	sgRNA design	AAACTGGTCGGATGACAGGTCATGC
m_sgZfp36_2_oligo1	sgRNA design	CACCGAAGCGGGCGTTGTCGCTACG
m_sgZfp36_2_oligo2	sgRNA design	AAACCGTAGCGACAACGCCCGCTTC
m_sgZfp36_5_oligo1	sgRNA design	CACCGCTCGGACTCCATCCCGTCTG
m_sgZfp36_5_oligo2	sgRNA design	AAACCAGACGGGATGGAGTCCGAGC

Supplementary Table 3

Geneset - Hallmark sets	NES	P
HALLMARK_ALLOGRAFT_REJECTION	2.453974623	4.22E-05
HALLMARK_EPITHELIAL_MESENCHYMAL_TRANSITION	2.281589779	4.22E-05
HALLMARK_INFLAMMATORY_RESPONSE	2.269457579	4.22E-05
HALLMARK_IL6_JAK_STAT3_SIGNALING	2.131816129	4.22E-05
HALLMARK_COAGULATION	1.880544617	0.000253357
HALLMARK_KRAS_SIGNALING_UP	1.773479209	0.000506714
HALLMARK_MYOGENESIS	1.620077905	0.003251415
HALLMARK_IL2_STAT5_SIGNALING	1.617247305	0.003420319
HALLMARK_COMPLEMENT	1.56677247	0.005953889
HALLMARK_BILE_ACID_METABOLISM	1.55434632	0.007136222
HALLMARK_PEROXISOME	1.492371075	0.013639051
HALLMARK_INTERFERON_GAMMA_RESPONSE	1.489031402	0.014145765
HALLMARK_APICAL_JUNCTION	1.417670962	0.027151423
HALLMARK_TNFA_SIGNALING_VIA_NFKB	1.357685121	0.044675281
HALLMARK_UV_RESPONSE_DN	1.352127205	0.046786589
HALLMARK_PANCREAS_BETA_CELLS	1.319275383	0.061396841
HALLMARK_UV_RESPONSE_UP	1.298951401	0.071573347
HALLMARK_ANGIOGENESIS	1.230402842	0.121020184
HALLMARK_OXIDATIVE_PHOSPHORYLATION	1.148037507	0.211721983
HALLMARK_APOPTOSIS	1.119198189	0.252216874
HALLMARK_ESTROGEN_RESPONSE_EARLY	1.108985421	0.267925006
HALLMARK_ADIPOGENESIS	1.092476985	0.295414239
HALLMARK_FATTY_ACID_METABOLISM	1.020075463	0.418587957
HALLMARK_APICAL_SURFACE	1.017184924	0.42449962
HALLMARK_PI3K_AKT_MTOR_SIGNALING	1.011948994	0.435478422
HALLMARK_ESTROGEN_RESPONSE_LATE	1.011880211	0.435520649
HALLMARK_HYPOXIA	0.962950014	0.536187822
HALLMARK_NOTCH_SIGNALING	0.90481486	0.659614897
HALLMARK_HEDGEHOG_SIGNALING	0.891917338	0.686259606
HALLMARK_XENOBIOTIC_METABOLISM	0.812953144	0.841060721
HALLMARK_TGF_BETA_SIGNALING	0.769358589	0.903893252
HALLMARK_P53_PATHWAY	0.732312922	0.941305633
HALLMARK_ANDROGEN_RESPONSE	0.727861986	0.944937083
HALLMARK_REACTIVE_OXIGEN_SPECIES_PATHWAY	0.647712185	0.984629677
HALLMARK_DNA_REPAIR	-0.60035244	0.99626943
HALLMARK_PROTEIN_SECRETION	-0.78524349	0.918549223
HALLMARK_INTERFERON_ALPHA_RESPONSE	-0.87241455	0.777202073
HALLMARK_WNT_BETA_CATENIN_SIGNALING	-0.87747218	0.76373057
HALLMARK_UNFOLDED_PROTEIN_RESPONSE	-0.98563802	0.484559585
HALLMARK_HEME_METABOLISM	-1.15830907	0.164766839
HALLMARK_MTORC1_SIGNALING	-1.20073995	0.120207254
HALLMARK_KRAS_SIGNALING_DN	-1.22062723	0.100310881
HALLMARK_GLYCOLYSIS	-1.2662965	0.070880829
HALLMARK_MYC_TARGETS_V2	-1.33228357	0.044974093
HALLMARK_MYC_TARGETS_V1	-1.35551477	0.036062176
HALLMARK_MITOTIC_SPINDLE	-1.40229166	0.023834197
HALLMARK_CHOLESTEROL_HOMEOSTASIS	-1.65635869	0.002901554
HALLMARK_SPERMATOGENESIS	-1.73578345	0.001658031
HALLMARK_G2M_CHECKPOINT	-2.00747285	0.000207254
HALLMARK_E2F_TARGETS	-2.13375447	0.000207254

Supplementary Table 4

Geneset - GOBP sets	NES	P
MONONUCLEAR CELL MIGRATION	2.516778133	6.93191E-07
LEUKOCYTE MIGRATION	2.482074403	6.93191E-07
LEUKOCYTE CHEMOTAXIS	2.460612562	6.93191E-07
CELL CHEMOTAXIS	2.441641752	6.93191E-07
LYMPHOCYTE MIGRATION	2.3470405	6.93191E-07
MYELOID LEUKOCYTE MIGRATION	2.34285082	6.93191E-07
ADAPTIVE IMMUNE RESPONSE	2.330743778	6.93191E-07
NEUTROPHIL MIGRATION	2.273608847	6.93191E-07
GRANULOCYTE MIGRATION	2.270937688	6.93191E-07
HUMORAL IMMUNE RESPONSE	2.249789792	6.93191E-07
LYMPHOCYTE CHEMOTAXIS	2.244778414	6.93191E-07
NEUTROPHIL CHEMOTAXIS	2.242069499	6.93191E-07
GRANULOCYTE CHEMOTAXIS	2.223011971	6.93191E-07
REGULATION OF MONONUCLEAR CELL MIGRATION	2.214354257	6.93191E-07
NEGATIVE REGULATION OF MONONUCLEAR CELL MIGRATIO	2.208572707	6.93191E-07
REGULATION OF COMPLEMENT ACTIVATION	2.199919219	6.93191E-07
REGULATION OF LYMPHOCYTE CHEMOTAXIS	2.192785508	6.93191E-07
INTERFERON GAMMA PRODUCTION	2.190035008	6.93191E-07
POSITIVE REGULATION OF CELL ACTIVATION	2.184271994	6.93191E-07
REGULATION OF CYTOSOLIC CALCIUM ION CONCENTRATIO	2.178476513	6.93191E-07
REGULATION OF CHEMOTAXIS	2.170705456	6.93191E-07
B CELL RECEPTOR SIGNALING PATHWAY	2.166274092	6.93191E-07
INFLAMMATORY RESPONSE	2.153077617	2.77276E-06
MONOCYTE CHEMOTAXIS	2.14980774	2.77276E-06
COMPLEMENT ACTIVATION	2.142787408	3.46595E-06
NATURAL KILLER CELL ACTIVATION	2.134583323	5.54553E-06
TAXIS	2.133418356	5.54553E-06
NEGATIVE REGULATION OF LEUKOCYTE MIGRATION	2.13310785	5.54553E-06
POSITIVE REGULATION OF LEUKOCYTE PROLIFERATION	2.132501597	5.54553E-06
INOSITOL PHOSPHATE BIOSYNTHETIC PROCESS	2.130127737	6.23872E-06
REGULATION OF NMDA RECEPTOR ACTIVITY	2.116902801	6.93191E-06
RESPIRATORY BURST	2.11232272	6.93191E-06
POSITIVE REGULATION OF INTERFERON GAMMA PRODUCTI	2.105675585	9.01148E-06
REGULATION OF LEUKOCYTE CHEMOTAXIS	2.103623393	9.01148E-06
REGULATION OF LEUKOCYTE MIGRATION	2.086243371	1.52502E-05
T CELL SELECTION	2.080670422	1.59434E-05
INTERLEUKIN 6 PRODUCTION	2.079408348	1.59434E-05
MACROPHAGE ACTIVATION	2.07314406	2.01025E-05
PHOSPHOLIPASE C ACTIVATING G PROTEIN COUPLED REC	2.06850354	2.14889E-05
CELLULAR DEFENSE RESPONSE	2.065734994	2.28753E-05
CELL KILLING	2.064157353	2.35685E-05
CELLULAR EXTRAVASATION	2.061522459	2.63413E-05
POSITIVE REGULATION OF LIPASE ACTIVITY	2.041910661	3.32732E-05
NEGATIVE REGULATION OF CHEMOTAXIS	2.040807964	3.32732E-05
REGULATION OF PHOSPHOLIPASE C ACTIVITY	2.03949209	3.46595E-05
MAST CELL ACTIVATION	2.033502985	4.08983E-05
DIVALENT INORGANIC CATION HOMEOSTASIS	2.030787682	4.15915E-05
REGULATION OF MONOCYTE CHEMOTAXIS	2.024756328	4.57506E-05
T CELL MIGRATION	2.017576089	5.26825E-05
NATURAL KILLER CELL MEDIATED IMMUNITY	2.015378798	5.54553E-05

MICROGLIAL CELL ACTIVATION	2.014805609	5.61485E-05
POSITIVE REGULATION OF ERK1 AND ERK2 CASCADE	2.013206325	5.89212E-05
NEGATIVE REGULATION OF INTERFERON GAMMA PRODUCT	2.005911594	7.07055E-05
OLEFINIC COMPOUND METABOLIC PROCESS	1.999695015	7.9717E-05
POSITIVE REGULATION OF T CELL PROLIFERATION	1.997755536	8.11033E-05
POSITIVE REGULATION OF LEUKOCYTE CELL CELL ADHESIO	1.996997034	8.11033E-05
CELLULAR RESPONSE TO VASCULAR ENDOTHELIAL GROWT	1.996526667	8.11033E-05
REGULATION OF CELL ACTIVATION	1.990030096	9.01148E-05
POSITIVE REGULATION OF CELL CELL ADHESION	1.985408521	9.98195E-05
INTERLEUKIN 12 PRODUCTION	1.984984004	0.000101899
REGULATION OF PHAGOCYTOSIS	1.978734167	0.000113683
DENDRITIC CELL MIGRATION	1.977165425	0.000117842
MAST CELL ACTIVATION INVOLVED IN IMMUNE RESPONSE	1.967654382	0.000131706
POSITIVE T CELL SELECTION	1.967630036	0.000131706
DENDRITIC CELL CHEMOTAXIS	1.966688046	0.000131706
POSITIVE REGULATION OF INFLAMMATORY RESPONSE	1.966268051	0.000133093
PURINERGIC NUCLEOTIDE RECEPTOR SIGNALING PATHWAY	1.964884943	0.000137252
REGULATION OF PHOSPHOLIPASE ACTIVITY	1.964061008	0.000140718
POSITIVE REGULATION OF PHAGOCYTOSIS	1.959698202	0.000146956
ERK1 AND ERK2 CASCADE	1.957126785	0.000151809
POSITIVE REGULATION OF CHEMOTAXIS	1.95496845	0.000155968
POSITIVE REGULATION OF RECEPTOR SIGNALING PATHWA	1.954942889	0.000155968
POSITIVE REGULATION OF CHEMOKINE PRODUCTION	1.9515884	0.000165673
MULTICELLULAR ORGANISMAL RESPONSE TO STRESS	1.948550123	0.000171911
THYMIC T CELL SELECTION	1.948535957	0.000171911
POLYOL BIOSYNTHETIC PROCESS	1.947751527	0.000173991
PHAGOCYTOSIS	1.944088868	0.000183002
ACTIVATION OF PHOSPHOLIPASE C ACTIVITY	1.943151474	0.000185775
LEUKOCYTE MEDIATED CYTOTOXICITY	1.936057938	0.000211423
NEUROINFLAMMATORY RESPONSE	1.929668283	0.000228753
COLLAGEN METABOLIC PROCESS	1.928560385	0.000231526
RESPONSE TO CHEMOKINE	1.928366928	0.000232912
LEUKOCYTE CELL CELL ADHESION	1.925061413	0.000246083
PRODUCTION OF MOLECULAR MEDIATOR INVOLVED IN INFL	1.923767906	0.000250242
EXTERNAL ENCAPSULATING STRUCTURE ORGANIZATION	1.922619794	0.000253015
LYMPHOCYTE MEDIATED IMMUNITY	1.922556417	0.000253708
KERATAN SULFATE METABOLIC PROCESS	1.917998725	0.000269651
LEUKOTRIENE METABOLIC PROCESS	1.910219045	0.000305004
LYMPHOCYTE ACTIVATION	1.90949293	0.00030847
ALPHA BETA T CELL ACTIVATION	1.907393372	0.000318175
POSITIVE REGULATION OF CYTOKINE PRODUCTION	1.906519676	0.000320254
CALCIUM ION TRANSPORT INTO CYTOSOL	1.902024873	0.000336891
POSITIVE REGULATION OF VASOCONSTRICTION	1.899215417	0.000350061
POSITIVE REGULATION OF INTERLEUKIN 12 PRODUCTION	1.896051882	0.000364618
POSITIVE REGULATION OF LIPID KINASE ACTIVITY	1.895710807	0.000369471
B CELL ACTIVATION	1.895549088	0.000370164
POSITIVE REGULATION OF RESPONSE TO EXTERNAL STIMU	1.895485475	0.000370164
REGULATION OF CELL KILLING	1.895453545	0.000370164
MACROPHAGE DIFFERENTIATION	1.892000313	0.000388187
REGULATION OF COLLAGEN METABOLIC PROCESS	1.888718463	0.00040621
REGULATION OF LEUKOCYTE PROLIFERATION	1.8880951	0.000408289
COLLAGEN FIBRIL ORGANIZATION	1.886379563	0.000416608
REGULATION OF LYMPHOCYTE MIGRATION	1.88615565	0.000417301

MYELOID LEUKOCYTE ACTIVATION	1.886009151	0.000418687
MACROPHAGE MIGRATION	1.882104321	0.000433244
REGULATION OF INFLAMMATORY RESPONSE	1.879695837	0.000445722
TUMOR NECROSIS FACTOR SUPERFAMILY CYTOKINE PROD	1.878352056	0.000451267
KERATAN SULFATE BIOSYNTHETIC PROCESS	1.875473033	0.00047137
POSITIVE REGULATION OF INTERLEUKIN 6 PRODUCTION	1.873170251	0.000487313
COAGULATION	1.872442818	0.000490086
G PROTEIN COUPLED RECEPTOR SIGNALING PATHWAY	1.867047211	0.000532371
POSITIVE REGULATION OF MACROPHAGE ACTIVATION	1.866125758	0.000537223
REGULATION OF NON CANONICAL WNT SIGNALING PATHWA	1.863715729	0.000560791
POSITIVE REGULATION OF CELL KILLING	1.862945127	0.000564951
LEUKOCYTE MEDIATED IMMUNITY	1.86053788	0.00058436
REGULATION OF LYMPHOCYTE ACTIVATION	1.859917175	0.000589212
POSITIVE REGULATION OF CALCIUM ION TRANSPORT	1.859293925	0.000594758
RENAL SYSTEM VASCULATURE DEVELOPMENT	1.858340149	0.000600997
POSITIVE REGULATION OF LEUKOCYTE MIGRATION	1.857931088	0.000605156
APOPTOTIC CELL CLEARANCE	1.854760499	0.000630111
REGULATION OF MYOBLAST DIFFERENTIATION	1.85029163	0.000667543
PITUITARY GLAND DEVELOPMENT	1.846503052	0.00069527
T CELL ACTIVATION	1.845062657	0.000713293
PLATELET DEGRANULATION	1.844333664	0.000721612
ALPHA BETA T CELL PROLIFERATION	1.841301538	0.00074518
REGULATION OF HUMORAL IMMUNE RESPONSE	1.838994739	0.000765283
VASCULAR ENDOTHELIAL GROWTH FACTOR SIGNALING PA	1.838818477	0.000765283
SPECIFICATION OF ANIMAL ORGAN IDENTITY	1.8379184	0.000771521
MYELOID DENDRITIC CELL ACTIVATION	1.837617329	0.000773601
SULFUR COMPOUND CATABOLIC PROCESS	1.837267067	0.00077776
RESPONSE TO WOUNDING	1.836812731	0.000782613
REGULATION OF SYNCYTIUM FORMATION BY PLASMA MEM	1.834145234	0.000814499
PLATELET ACTIVATION	1.832217077	0.000840147
REGULATION OF LEUKOCYTE DEGRANULATION	1.827523781	0.000888671
CYTOSOLIC CALCIUM ION TRANSPORT	1.825583369	0.000910853
MATURE B CELL DIFFERENTIATION INVOLVED IN IMMUNE R	1.825351746	0.000912932
CELL PROLIFERATION INVOLVED IN KIDNEY DEVELOPMENT	1.825003066	0.000915012
WOUND HEALING	1.820842698	0.000967001
POSITIVE REGULATION OF DEFENSE RESPONSE	1.819415278	0.000979479
POSITIVE REGULATION OF IMMUNE RESPONSE	1.811468115	0.001071673
REGULATION OF NATURAL KILLER CELL MEDIATED IMMUNIT	1.811070105	0.001081378
LEUKOCYTE TETHERING OR ROLLING	1.800931622	0.001231107
CELL ACTIVATION INVOLVED IN IMMUNE RESPONSE	1.800597976	0.001235266
NEGATIVE REGULATION OF ENDOTHELIAL CELL PROLIFERA	1.799616032	0.00124913
POSITIVE REGULATION OF REPRODUCTIVE PROCESS	1.798100546	0.001277551
NEGATIVE REGULATION OF CELL ACTIVATION	1.797954103	0.001277551
GLOMERULUS DEVELOPMENT	1.797217595	0.001286562
REGULATION OF B CELL ACTIVATION	1.795416461	0.001319142
NEGATIVE REGULATION OF INTERLEUKIN 6 PRODUCTION	1.794953447	0.001327461
CYTOKINE MEDIATED SIGNALING PATHWAY	1.794588417	0.001335779
REGULATION OF MACROPHAGE MIGRATION	1.793803485	0.001355188
POSITIVE REGULATION OF TUMOR NECROSIS FACTOR SUP	1.793277992	0.001364893
LEUKOCYTE PROLIFERATION	1.791833641	0.001387768
CYCLIC NUCLEOTIDE METABOLIC PROCESS	1.790659043	0.001413416
DEFENSE RESPONSE TO BACTERIUM	1.79029073	0.001419655
REGULATION OF T HELPER 1 TYPE IMMUNE RESPONSE	1.786191862	0.001498679

REGULATION OF MAST CELL ACTIVATION	1.7859004	0.001504917
POSITIVE REGULATION OF MONONUCLEAR CELL MIGRATIO	1.78413259	0.001535418
REGULATION OF LEUKOCYTE MEDIATED IMMUNITY	1.783043917	0.001553441
T CELL PROLIFERATION	1.780135782	0.001602657
POSITIVE REGULATION OF ALPHA BETA T CELL ACTIVATION	1.78006827	0.001604737
LEUKOCYTE DIFFERENTIATION	1.779460026	0.001617908
REGULATION OF BLOOD PRESSURE	1.778617816	0.001633158
REGULATION OF PHOSPHATIDYLINOSITOL 3 KINASE ACTIVI	1.778431225	0.001635237
POSITIVE REGULATION OF LEUKOCYTE CHEMOTAXIS	1.77779414	0.001648408
REGULATION OF WOUND HEALING	1.777199323	0.001660192
FEAR RESPONSE	1.775974749	0.001684454
CYTOKINE PRODUCTION	1.775158937	0.001698318
MEMBRANE RAFT ORGANIZATION	1.774843897	0.001705943
MATURE B CELL DIFFERENTIATION	1.774755346	0.001710102
LONG CHAIN FATTY ACID METABOLIC PROCESS	1.774731019	0.001710102
POSITIVE REGULATION OF CELL ADHESION	1.774321051	0.001721886
ACTIVATION OF IMMUNE RESPONSE	1.773340288	0.001743375
REGULATION OF B CELL PROLIFERATION	1.772209065	0.001769023
CALCIUM ION TRANSPORT	1.772057824	0.001771796
ADAPTIVE IMMUNE RESPONSE BASED ON SOMATIC RECOM	1.771294324	0.00178358
ICOSANOID METABOLIC PROCESS	1.766184052	0.001882013
LYMPHOCYTE ACTIVATION INVOLVED IN IMMUNE RESPONS	1.76562677	0.001897957
REGULATION OF PLATELET ACTIVATION	1.765471264	0.001900036
REGULATION OF CELL CELL ADHESION	1.763300338	0.001954798
POSITIVE REGULATION OF IMMUNE EFFECTOR PROCESS	1.763144667	0.001956878
T HELPER 1 TYPE IMMUNE RESPONSE	1.762636642	0.001971435
PROXIMAL DISTAL PATTERN FORMATION	1.762071449	0.001980446
METAL ION HOMEOSTASIS	1.760216057	0.002029663
RESPONSE TO BACTERIUM	1.75846476	0.002075414
NEURON PROJECTION REGENERATION	1.757517258	0.002095516
POSITIVE REGULATION OF B CELL PROLIFERATION	1.756221098	0.002131562
CHEMOKINE PRODUCTION	1.754399145	0.002181472
HUMORAL IMMUNE RESPONSE MEDIATED BY CIRCULATING	1.75356638	0.002204347
REGULATION OF B CELL DIFFERENTIATION	1.751526214	0.002248018
COLLAGEN CATABOLIC PROCESS	1.750230418	0.002277132
REGULATION OF T CELL ACTIVATION	1.750184162	0.002279212
POSITIVE REGULATION OF MAPK CASCADE	1.749290795	0.002310405
RENAL SYSTEM PROCESS	1.748134604	0.002341599
LEUKOCYTE HOMEOSTASIS	1.746505498	0.002390122
T CELL ACTIVATION INVOLVED IN IMMUNE RESPONSE	1.745647499	0.002410918
RESPONSE TO INTERFERON GAMMA	1.745264627	0.002423395
B CELL PROLIFERATION	1.744878096	0.002436566
CYTOKINE PRODUCTION INVOLVED IN INFLAMMATORY RES	1.743466859	0.002471919
COLLAGEN BIOSYNTHETIC PROCESS	1.742078019	0.002507272
POLYOL METABOLIC PROCESS	1.740051483	0.002556488
T CELL LINEAGE COMMITMENT	1.739216205	0.002573818
REGULATION OF LIPASE ACTIVITY	1.73900431	0.00257867
MYELOID LEUKOCYTE DIFFERENTIATION	1.738636389	0.002583523
SMOOTH MUSCLE CELL PROLIFERATION	1.738163187	0.002595307
REGULATION OF IMMUNE EFFECTOR PROCESS	1.736630606	0.002641057
ANIMAL ORGAN FORMATION	1.736462357	0.00264591
MYELOID LEUKOCYTE MEDIATED IMMUNITY	1.734115727	0.002714536
TRANSMISSION OF NERVE IMPULSE	1.731684673	0.002790787

POSITIVE REGULATION OF B CELL ACTIVATION	1.731563319	0.002794946
T CELL MEDIATED CYTOTOXICITY	1.73149856	0.002798412
MONONUCLEAR CELL DIFFERENTIATION	1.730289359	0.002834458
REGULATION OF LIPID KINASE ACTIVITY	1.730062581	0.002838617
POSITIVE REGULATION OF VASCULATURE DEVELOPMENT	1.729762237	0.002849015
NEGATIVE REGULATION OF SMOOTH MUSCLE CELL PROLIF	1.729421355	0.002858026
REGULATION OF COAGULATION	1.729137398	0.002865651
POSITIVE REGULATION OF PHOSPHATIDYLINOSITOL 3 KINA	1.726953364	0.002928038
ARACHIDONIC ACID METABOLIC PROCESS	1.71939936	0.003187292
UNSATURATED FATTY ACID METABOLIC PROCESS	1.71890137	0.003199769
POSITIVE REGULATION OF PROTEIN KINASE B SIGNALING	1.716459053	0.003287111
LYMPHOCYTE COSTIMULATION	1.715359393	0.003318998
BLOOD VESSEL ENDOTHELIAL CELL PROLIFERATION INVOL	1.715332766	0.003321771
RECEPTOR SIGNALING PATHWAY VIA STAT	1.71379169	0.003381385
REGULATION OF MAPK CASCADE	1.71336216	0.00339317
MUSCLE CONTRACTION	1.710294442	0.003505467
REGULATION OF LEUKOCYTE MEDIATED CYTOTOXICITY	1.708387167	0.003576865
POSITIVE REGULATION OF INTERLEUKIN 8 PRODUCTION	1.708041896	0.003590729
ZYMOGEN ACTIVATION	1.706900075	0.003635786
REGULATION OF MACROPHAGE ACTIVATION	1.706641245	0.003648264
ENDOTHELIAL CELL PROLIFERATION	1.706543444	0.003652423
METAL ION TRANSPORT	1.701071648	0.003871471
PLATELET AGGREGATION	1.700288821	0.003898506
INTERLEUKIN 10 PRODUCTION	1.699738092	0.003919301
INTERLEUKIN 17 PRODUCTION	1.699159845	0.00394495
CELL CELL ADHESION	1.698685151	0.003965745
INTERLEUKIN 4 PRODUCTION	1.695804721	0.004096758
REGULATION OF CELL ADHESION	1.694418951	0.004157759
ACUTE INFLAMMATORY RESPONSE	1.694355713	0.004161918
MUSCLE FILAMENT SLIDING	1.694338228	0.004163305
POSITIVE REGULATION OF LIPID METABOLIC PROCESS	1.692649435	0.004242328
POSITIVE REGULATION OF LOCOMOTION	1.692536717	0.004245794
CELLULAR ION HOMEOSTASIS	1.69244632	0.00424926
LYSOSOME LOCALIZATION	1.690615561	0.004322045
GLYCOSPHINGOLIPID METABOLIC PROCESS	1.68918053	0.00438166
CALCIUM ION TRANSMEMBRANE TRANSPORT	1.687605076	0.004446127
RENAL FILTRATION	1.686421879	0.004501582
INTEGRIN ACTIVATION	1.685508006	0.00454248
MORPHOGENESIS OF AN EPITHELIAL FOLD	1.685095251	0.004552878
GRANULOCYTE DIFFERENTIATION	1.684525831	0.004588231
NEGATIVE REGULATION OF WOUND HEALING	1.684272399	0.004601401
REGULATION OF MEMBRANE DEPOLARIZATION	1.683435385	0.004649925
ICOSANOID BIOSYNTHETIC PROCESS	1.682306573	0.004697755
NEGATIVE REGULATION OF COAGULATION	1.680416345	0.004789949
BIOMINERALIZATION	1.680028232	0.004808665
MITOCHONDRIAL DEPOLARIZATION	1.679898029	0.004819063
REGULATION OF TUBE SIZE	1.679803095	0.004825302
NEGATIVE REGULATION OF REACTIVE OXYGEN SPECIES BI	1.679125148	0.004855802
CRANIAL NERVE MORPHOGENESIS	1.678862411	0.004863428
CALCIUM MEDIATED SIGNALING	1.676606852	0.004977804
NEGATIVE REGULATION OF LIPID METABOLIC PROCESS	1.676019979	0.005003452
BLOOD VESSEL MORPHOGENESIS	1.675407886	0.005030487
NEGATIVE REGULATION OF LOCOMOTION	1.675159098	0.005050589

POSITIVE REGULATION OF GLIOGENESIS	1.674977276	0.005057521
ODONTOGENESIS OF DENTIN CONTAINING TOOTH	1.673180202	0.005156647
T CELL DIFFERENTIATION INVOLVED IN IMMUNE RESPONSE	1.672460102	0.005196852
ADENYLATE CYCLASE ACTIVATING G PROTEIN COUPLED RI	1.668727903	0.005406196
REGULATION OF BEHAVIOR	1.668390837	0.005425605
T CELL DIFFERENTIATION IN THYMUS	1.66724428	0.005476901
AMINOGLYCAN CATABOLIC PROCESS	1.666842709	0.00550047
REGULATION OF REGULATED SECRETORY PATHWAY	1.665747191	0.005554539
NATURAL KILLER CELL DIFFERENTIATION	1.66491422	0.005605835
AMINOGLYCAN METABOLIC PROCESS	1.664085497	0.00566129
DENDRITIC CELL DIFFERENTIATION	1.662689673	0.005748632
MACROPHAGE CHEMOTAXIS	1.662246616	0.005770814
MUCOPOLYSACCHARIDE METABOLIC PROCESS	1.662239468	0.005770814
REGULATION OF LEUKOCYTE APOPTOTIC PROCESS	1.66191086	0.005787451
PROTEIN KINASE B SIGNALING	1.660053299	0.005885884
REGULATION OF MYELOID LEUKOCYTE MEDIATED IMMUNIT	1.658921236	0.005951044
HOMOTYPIC CELL CELL ADHESION	1.658797773	0.005959362
POSITIVE REGULATION OF INTERLEUKIN 2 PRODUCTION	1.658506903	0.005978079
RESPONSE TO PURINE CONTAINING COMPOUND	1.657594169	0.00603492
ION HOMEOSTASIS	1.656781314	0.006077205
POSITIVE REGULATION OF VASCULAR ENDOTHELIAL GROW	1.656713267	0.006079978
TOLERANCE INDUCTION	1.656277393	0.006109092
REGULATION OF NEUROTRANSMITTER RECEPTOR ACTIVITY	1.655234447	0.006174945
LYMPHOCYTE HOMEOSTASIS	1.654157164	0.006231093
NEGATIVE REGULATION OF RESPONSE TO WOUNDING	1.653707765	0.00626298
REGULATION OF RESPONSE TO WOUNDING	1.653409369	0.006281003
REGULATION OF ENDOTHELIAL CELL CHEMOTAXIS	1.653021411	0.006301799
POSITIVE REGULATION OF LYMPHOCYTE MIGRATION	1.651406235	0.006400925
VASCULAR ENDOTHELIAL GROWTH FACTOR PRODUCTION	1.650952985	0.006434198
CATECHOL CONTAINING COMPOUND METABOLIC PROCESS	1.650922671	0.006434891
GLIAL CELL MIGRATION	1.65079856	0.006443903
POSITIVE REGULATION OF MACROPHAGE MIGRATION	1.650604648	0.006455687
NEGATIVE REGULATION OF MYOBLAST DIFFERENTIATION	1.650337895	0.006468858
RESPONSE TO MOLECULE OF BACTERIAL ORIGIN	1.649802257	0.006509756
ALPHA BETA T CELL DIFFERENTIATION	1.648281153	0.006614428
REGULATION OF NATURAL KILLER CELL ACTIVATION	1.647980532	0.006628985
REGULATION OF BODY FLUID LEVELS	1.647421317	0.006662258
BONE MINERALIZATION	1.64533914	0.006790498
REGULATION OF LIPID METABOLIC PROCESS	1.64448128	0.006852192
REGULATION OF ALPHA BETA T CELL ACTIVATION	1.643143039	0.006951319
PATHWAY RESTRICTED SMAD PROTEIN PHOSPHORYLATIOI	1.641929863	0.007044899
REGULATION OF VASCULATURE DEVELOPMENT	1.641235612	0.007099661
B CELL MEDIATED IMMUNITY	1.6388868	0.00725355
POSITIVE REGULATION OF COLLAGEN METABOLIC PROCES	1.637849882	0.007347824
REGULATION OF MAST CELL ACTIVATION INVOLVED IN IMMI	1.636502019	0.007448336
NEGATIVE REGULATION OF LEUKOCYTE APOPTOTIC PROCI	1.634608944	0.007583509
POSITIVE REGULATION OF CHOLESTEROL EFFLUX	1.634132295	0.007616782
MYOBLAST DIFFERENTIATION	1.634027601	0.007624407
T CELL DIFFERENTIATION	1.633345541	0.007667385
B CELL DIFFERENTIATION	1.632551887	0.007721454
DEFENSE RESPONSE TO GRAM POSITIVE BACTERIUM	1.63239738	0.007733931
REGULATION OF MACROPHAGE CHEMOTAXIS	1.631360734	0.007810875
NEGATIVE REGULATION OF EPITHELIAL CELL MIGRATION	1.631067072	0.007836523

SYNCYTIUM FORMATION	1.63034759	0.007895445
IMMUNE RESPONSE REGULATING SIGNALING PATHWAY	1.630303279	0.007900297
NEGATIVE REGULATION OF IMMUNE SYSTEM PROCESS	1.63029031	0.007901683
POSITIVE REGULATION OF PEPTIDYL TYROSINE PHOSPHOR	1.629552292	0.007947434
POSITIVE REGULATION OF NIK NF KAPPAB SIGNALING	1.629473281	0.0079509
CONNECTIVE TISSUE DEVELOPMENT	1.628592285	0.008018139
POSITIVE REGULATION OF ENDOCYTOSIS	1.628057031	0.008056265
NEGATIVE CHEMOTAXIS	1.627912727	0.008066663
REGULATION OF LIPID CATABOLIC PROCESS	1.626481653	0.00817688
RESPONSE TO ATP	1.62623806	0.008190744
POSITIVE REGULATION OF LYMPHOCYTE DIFFERENTIATION	1.625243228	0.008266995
CELL SUBSTRATE ADHESION	1.623995903	0.008379985
REGULATION OF DEFENSE RESPONSE	1.623549153	0.008414645
LEUKOCYTE APOPTOTIC PROCESS	1.623448451	0.008416724
HETEROTYPIC CELL CELL ADHESION	1.623019882	0.008446531
OSSIFICATION	1.622748205	0.008467327
ANGIOGENESIS INVOLVED IN WOUND HEALING	1.622701127	0.0084701
NEGATIVE REGULATION OF CELLULAR RESPONSE TO GROW	1.622159229	0.008504759
REGULATION OF NEUTROPHIL MIGRATION	1.622040076	0.008522782
GLOMERULAR EPITHELIUM DEVELOPMENT	1.621790147	0.008544271
NEGATIVE REGULATION OF NEURON PROJECTION DEVELO	1.618829245	0.008784115
POSITIVE REGULATION OF ACTIN FILAMENT BUNDLE ASSEM	1.618730177	0.00879382
ADENYLATE CYCLASE MODULATING G PROTEIN COUPLED R	1.618020424	0.008853434
REGULATORY T CELL DIFFERENTIATION	1.617912458	0.008864526
REGULATION OF ANIMAL ORGAN FORMATION	1.615783858	0.009057233
REGULATION OF LYMPHOCYTE MEDIATED IMMUNITY	1.615779185	0.009057926
DIENCEPHALON DEVELOPMENT	1.610648242	0.009514045
CELLULAR RESPONSE TO LIPOPROTEIN PARTICLE STIMULU	1.609758205	0.009590296
REGULATION OF VASCULAR ENDOTHELIAL GROWTH FACTO	1.604861943	0.010003438
POSITIVE REGULATION OF RHO PROTEIN SIGNAL TRANSDU	1.603413349	0.010121974
INTEGRIN MEDIATED SIGNALING PATHWAY	1.600534436	0.010365284
ODONTOGENESIS	1.598682812	0.010539968
NEGATIVE REGULATION OF TUMOR NECROSIS FACTOR SUP	1.596799678	0.010725743
LEUKOCYTE ADHESION TO VASCULAR ENDOTHELIAL CELL	1.596520118	0.010746539
INOSITOL PHOSPHATE METABOLIC PROCESS	1.593899581	0.010994008
HEART FORMATION	1.593513419	0.011032134
SKELETAL MUSCLE ORGAN DEVELOPMENT	1.59240192	0.011134033
CELLULAR RESPONSE TO MOLECULE OF BACTERIAL ORIGIN	1.590716483	0.011314262
SPHINGOLIPID METABOLIC PROCESS	1.59050354	0.011340604
CD4 POSITIVE ALPHA BETA T CELL DIFFERENTIATION	1.589610221	0.011441809
PROTEIN KINASE C SIGNALING	1.587944224	0.011627585
REGULATION OF NIK NF KAPPAB SIGNALING	1.586772214	0.011733643
POSITIVE REGULATION OF EMBRYONIC DEVELOPMENT	1.586331943	0.011775234
LOCALIZATION WITHIN MEMBRANE	1.586029751	0.011809201
INTERLEUKIN 8 PRODUCTION	1.585299926	0.011886838
RESPONSE TO AXON INJURY	1.584014654	0.012014385
RESPONSE TO ORGANOPHOSPHORUS	1.581523964	0.012274332
INTERLEUKIN 1 PRODUCTION	1.581408867	0.012286116
UNSATURATED FATTY ACID BIOSYNTHETIC PROCESS	1.578089014	0.01265628
T CELL APOPTOTIC PROCESS	1.577959124	0.012667371
POSITIVE REGULATION OF EPITHELIAL CELL APOPTOTIC PR	1.575215285	0.012979307
GLUTAMATE SECRETION	1.575052512	0.012992477
CELLULAR RESPONSE TO BIOTIC STIMULUS	1.574876569	0.01301258

MEMBRANE LIPID METABOLIC PROCESS	1.574630862	0.013041694
NEGATIVE REGULATION OF LEUKOCYTE MEDIATED IMMUNI	1.574623726	0.013043774
REGULATION OF EPITHELIAL CELL APOPTOTIC PROCESS	1.574448831	0.013065263
ACTIVATION OF ADENYLATE CYCLASE ACTIVITY	1.573685251	0.013136661
NEGATIVE REGULATION OF CELL ADHESION	1.573296199	0.013179639
NEGATIVE REGULATION OF PROTEIN MATURATION	1.570774697	0.013472166
POSITIVE REGULATION OF ALPHA BETA T CELL DIFFERENTI	1.570204279	0.013535246
LIPOSACCHARIDE METABOLIC PROCESS	1.568469411	0.013738351
RECEPTOR MEDIATED ENDOCYTOSIS	1.568156404	0.013774397
SUPEROXIDE METABOLIC PROCESS	1.568132729	0.01377509
GLOMERULAR EPITHELIAL CELL DIFFERENTIATION	1.567484602	0.013845796
TOLL LIKE RECEPTOR SIGNALING PATHWAY	1.56738128	0.01385758
REACTIVE OXYGEN SPECIES METABOLIC PROCESS	1.566283722	0.013996218
SPHINGOLIPID BIOSYNTHETIC PROCESS	1.566025429	0.014023252
REGULATION OF HOMOTYPIC CELL CELL ADHESION	1.565283404	0.014121686
AMINOGLYCAN BIOSYNTHETIC PROCESS	1.564737267	0.014187539
REGULATION OF BMP SIGNALING PATHWAY	1.564220058	0.014256165
REGULATION OF CELL ADHESION MEDIATED BY INTEGRIN	1.561232348	0.014615237
MYOTUBE DIFFERENTIATION	1.561107446	0.014632567
DOPAMINE METABOLIC PROCESS	1.559947642	0.014760808
REGULATION OF CATION CHANNEL ACTIVITY	1.55981078	0.01478091
CELLULAR RESPONSE TO NUTRIENT	1.559154136	0.014873798
REGULATION OF CELL FATE COMMITMENT	1.557996087	0.015017981
NEGATIVE REGULATION OF PROTEIN LOCALIZATION TO ME	1.555609289	0.015322985
CELL RECOGNITION	1.555105295	0.015383986
POSITIVE REGULATION OF WOUND HEALING	1.554269833	0.01549351
REGULATION OF PEPTIDASE ACTIVITY	1.553623544	0.015582932
CARTILAGE DEVELOPMENT	1.553195909	0.015630069
POSITIVE REGULATION OF SYNAPTIC TRANSMISSION	1.553169271	0.015633535
CELLULAR RESPONSE TO VITAMIN	1.552783112	0.015682751
NEGATIVE REGULATION OF PEPTIDASE ACTIVITY	1.551512341	0.015844958
BODY MORPHOGENESIS	1.55093041	0.015923982
POSITIVE REGULATION OF INTERLEUKIN 1 PRODUCTION	1.549934666	0.01606262
CARDIAC ATRIUM MORPHOGENESIS	1.549728417	0.016095893
SPHINGOID METABOLIC PROCESS	1.548156891	0.016329499
CELLULAR RESPONSE TO CAMP	1.547444086	0.016413375
REGULATION OF TUMOR NECROSIS FACTOR MEDIATED SIG	1.546955554	0.016477148
CELL ADHESION MEDIATED BY INTEGRIN	1.546372284	0.01654924
ENDOTHELIAL CELL MIGRATION	1.546259515	0.016561024
B CELL ACTIVATION INVOLVED IN IMMUNE RESPONSE	1.546059148	0.016588752
POSITIVE REGULATION OF TYROSINE PHOSPHORYLATION (	1.545377124	0.016682333
POSITIVE REGULATION OF HEMOPOIESIS	1.544835578	0.016768288
CELLULAR RESPONSE TO PROSTAGLANDIN STIMULUS	1.544802592	0.016773834
HEAD MORPHOGENESIS	1.544722622	0.016784925
REGULATION OF ENDOTHELIAL CELL MIGRATION	1.543153926	0.016992189
POSITIVE REGULATION OF RESPONSE TO WOUNDING	1.543152796	0.016992189
POSITIVE REGULATION OF RECEPTOR MEDIATED ENDOCYT	1.542920576	0.01702061
REGULATION OF MACROPHAGE DIFFERENTIATION	1.542770166	0.017040019
CERAMIDE METABOLIC PROCESS	1.542518805	0.017080224
NEGATIVE REGULATION OF COLD INDUCED THERMOGENES	1.542081827	0.017137066
HOMEOSTASIS OF NUMBER OF CELLS	1.541216643	0.017255602
TEMPERATURE HOMEOSTASIS	1.540583754	0.017337398
POSITIVE REGULATION OF SMOOTH MUSCLE CELL PROLIFE	1.539864852	0.01744415

NEGATIVE REGULATION OF INFLAMMATORY RESPONSE	1.538850533	0.017579322
ANTIMICROBIAL HUMORAL RESPONSE	1.53810995	0.017690232
REPLACEMENT OSSIFICATION	1.537939667	0.01771796
EPITHELIAL CELL PROLIFERATION	1.537576489	0.017769256
NEGATIVE REGULATION OF SMALL MOLECULE METABOLIC	1.535433413	0.018096442
SENSORY SYSTEM DEVELOPMENT	1.535364807	0.018111692
NEGATIVE REGULATION OF ESTABLISHMENT OF PROTEIN L	1.53481748	0.018198341
PEPTIDYL TYROSINE MODIFICATION	1.534305415	0.01827182
REGULATION OF SYNAPTIC TRANSMISSION GLUTAMATERG	1.533830152	0.018353616
ENDOTHELIAL CELL APOPTOTIC PROCESS	1.533267043	0.018427094
REGULATION OF CALCIUM MEDIATED SIGNALING	1.532691359	0.018524834
NEGATIVE REGULATION OF PROTEIN LOCALIZATION TO CE	1.532688223	0.018525527
CD4 POSITIVE ALPHA BETA T CELL ACTIVATION	1.530215079	0.018907476
REGULATION OF LEUKOCYTE DIFFERENTIATION	1.53013963	0.018917874
REGULATION OF ENDOTHELIAL CELL DIFFERENTIATION	1.530002218	0.018946988
CIRCULATORY SYSTEM PROCESS	1.529232377	0.019040568
DIOL METABOLIC PROCESS	1.528867108	0.019089785
C21 STEROID HORMONE METABOLIC PROCESS	1.528147731	0.019202775
MEMBRANE DEPOLARIZATION	1.526907935	0.019384391
SEQUESTERING OF CALCIUM ION	1.526707386	0.019412812
CERAMIDE BIOSYNTHETIC PROCESS	1.526222424	0.019495302
FAT CELL DIFFERENTIATION	1.525935224	0.019545904
LIPOPOLYSACCHARIDE MEDIATED SIGNALING PATHWAY	1.524210238	0.019834272
DETECTION OF EXTERNAL BIOTIC STIMULUS	1.524076506	0.019859227
VASCULAR PROCESS IN CIRCULATORY SYSTEM	1.523330982	0.019989547
POSITIVE REGULATION OF INTERLEUKIN 10 PRODUCTION	1.522250352	0.020176708
REGULATION OF GRANULOCYTE CHEMOTAXIS	1.521825499	0.020244641
POSITIVE REGULATION OF LEUKOCYTE MEDIATED IMMUNIT	1.520823129	0.020415166
REGULATION OF LYMPHOCYTE DIFFERENTIATION	1.520043448	0.02052469
CELL FATE COMMITMENT	1.51999142	0.020528156
MUSCLE SYSTEM PROCESS	1.517691398	0.020910104
REGULATION OF SYSTEM PROCESS	1.516204928	0.021166585
FOREBRAIN GENERATION OF NEURONS	1.515518338	0.021275416
ENDOTHELIAL CELL CHEMOTAXIS	1.51532516	0.021316314
MYELOID CELL DIFFERENTIATION	1.514493332	0.021438316
POSITIVE REGULATION OF PATHWAY RESTRICTED SMAD P	1.514345587	0.021459805
POSITIVE REGULATION OF ENDOTHELIAL CELL PROLIFERAT	1.513087947	0.021679546
ACTIN FILAMENT BASED MOVEMENT	1.511715747	0.021924243
LIPID EXPORT FROM CELL	1.508729319	0.022455227
PERIPHERAL NERVOUS SYSTEM DEVELOPMENT	1.507945536	0.022614661
REGULATION OF T CELL MIGRATION	1.507018848	0.022773401
REGULATION OF DEFENSE RESPONSE TO VIRUS BY VIRUS	1.5069719	0.02277964
MULTI MULTICELLULAR ORGANISM PROCESS	1.506634469	0.022839948
REGENERATION	1.506192954	0.022927983
NEGATIVE REGULATION OF OXIDATIVE STRESS INDUCED IN	1.505099076	0.023125542
SUPEROXIDE ANION GENERATION	1.5045143	0.023235067
RESPONSE TO FUNGUS	1.504209431	0.023296067
INTERFERON GAMMA MEDIATED SIGNALING PATHWAY	1.503683871	0.023402126
CELL JUNCTION DISASSEMBLY	1.501948843	0.023733471
SECOND MESSENGER MEDIATED SIGNALING	1.501328731	0.02386795
NEGATIVE REGULATION OF BMP SIGNALING PATHWAY	1.500114035	0.024100862
EYE MORPHOGENESIS	1.499793839	0.024168795
REGULATION OF NEUTROPHIL CHEMOTAXIS	1.499787096	0.024168795

LYMPH VESSEL DEVELOPMENT	1.498071401	0.024505686
INTERLEUKIN 1 BETA PRODUCTION	1.497919936	0.024534106
INSULIN LIKE GROWTH FACTOR RECEPTOR SIGNALING PAT	1.49773193	0.024568073
T CELL MEDIATED IMMUNITY	1.497021783	0.02471295
NEGATIVE REGULATION OF RESPONSE TO EXTERNAL STIM	1.495363838	0.025030431
SPINAL CORD MOTOR NEURON DIFFERENTIATION	1.493217416	0.025442186
RESPONSE TO PROSTAGLANDIN E	1.492886498	0.025521903
ANTIGEN RECEPTOR MEDIATED SIGNALING PATHWAY	1.492586062	0.025582211
POSITIVE REGULATION OF ANTIGEN RECEPTOR MEDIATED	1.490360343	0.026018228
MYOBLAST FUSION	1.489750117	0.026131218
NEGATIVE REGULATION OF CELL PROJECTION ORGANIZATI	1.489601279	0.026156173
NEUROTRANSMITTER SECRETION	1.489508822	0.026174196
REGULATION OF CELLULAR EXTRAVASATION	1.488756905	0.026334323
REGULATION OF INNATE IMMUNE RESPONSE	1.487774545	0.026518712
VASODILATION	1.486399931	0.026797375
POSITIVE REGULATION OF SMALL GTPASE MEDIATED SIGN/	1.486189875	0.026843819
OVULATION CYCLE PROCESS	1.483869273	0.027297165
NEGATIVE REGULATION OF BLOOD VESSEL ENDOTHELIAL (	1.482463203	0.027603556
POSITIVE REGULATION OF CALCIUM ION TRANSPORT INTO	1.482324584	0.027631977
FOAM CELL DIFFERENTIATION	1.482263555	0.027643761
REGULATION OF VESICLE MEDIATED TRANSPORT	1.481684821	0.027769922
ALCOHOL METABOLIC PROCESS	1.481578368	0.027798342
REGULATION OF SUPEROXIDE METABOLIC PROCESS	1.48056034	0.027997288
POSITIVE REGULATION OF STRESS FIBER ASSEMBLY	1.480158831	0.028085323
MUSCLE CELL PROLIFERATION	1.479957808	0.028126222
REGULATION OF METAL ION TRANSPORT	1.478195063	0.028524113
REGULATION OF MEMBRANE PROTEIN ECTODOMAIN PROTE	1.477808837	0.028599671
NEGATIVE REGULATION OF LYMPHOCYTE ACTIVATION	1.476979725	0.028782674
NEGATIVE REGULATION OF LYMPHOCYTE MEDIATED IMMUN	1.476731261	0.028830504
PHARYNGEAL SYSTEM DEVELOPMENT	1.475079464	0.029179179
TYROSINE PHOSPHORYLATION OF STAT PROTEIN	1.474735763	0.029245725
REGULATION OF CARTILAGE DEVELOPMENT	1.474215539	0.029386443
C21 STEROID HORMONE BIOSYNTHETIC PROCESS	1.474099475	0.029407932
CELLULAR RESPONSE TO ACID CHEMICAL	1.472797548	0.02967689
REGULATION OF EXTRACELLULAR MATRIX ORGANIZATION	1.471658514	0.029922973
MUSCLE STRUCTURE DEVELOPMENT	1.471343837	0.029995064
TISSUE MIGRATION	1.469836488	0.030352058
REGULATION OF MACROPHAGE DERIVED FOAM CELL DIFFE	1.466874368	0.031017521
CELL SURFACE RECEPTOR SIGNALING PATHWAY INVOLVED	1.466297924	0.031149227
TUMOR NECROSIS FACTOR MEDIATED SIGNALING PATHWA	1.465893946	0.031231024
REGULATION OF REACTIVE OXYGEN SPECIES METABOLIC I	1.465137468	0.031420265
STRIATED MUSCLE CONTRACTION	1.464814907	0.03149513
RESPONSE TO TUMOR NECROSIS FACTOR	1.464098755	0.031676052
EPITHELIAL CELL APOPTOTIC PROCESS	1.463606299	0.031789736
REGULATION OF SECRETION	1.462232733	0.032134945
PHOSPHATIDYLINOSITOL PHOSPHORYLATION	1.460814185	0.032499563
POSITIVE REGULATION OF PHOSPHATASE ACTIVITY	1.460618445	0.032543234
SENSORY ORGAN MORPHOGENESIS	1.460410308	0.032600076
MULTICELLULAR ORGANISMAL HOMEOSTASIS	1.459633344	0.032765749
MEMBRANE LIPID BIOSYNTHETIC PROCESS	1.458303053	0.033088776
MESENCHYME MORPHOGENESIS	1.457177809	0.033384768
RESPONSE TO GROWTH FACTOR	1.456823112	0.03347211
RESPONSE TO BMP	1.456822873	0.03347211

REGULATION OF CELL SHAPE	1.456462396	0.033578862
REGULATION OF ACTIN FILAMENT BUNDLE ASSEMBLY	1.454994821	0.033961503
POSITIVE REGULATION OF IMMUNOGLOBULIN PRODUCTION	1.45264298	0.034515362
MUSCLE ORGAN DEVELOPMENT	1.451870682	0.034717081
ACIDIC AMINO ACID TRANSPORT	1.451140437	0.034892458
REGULATION OF PHOSPHATIDYLINOSITOL 3 KINASE SIGNAL	1.45111488	0.034902856
TOLL LIKE RECEPTOR 9 SIGNALING PATHWAY	1.449943281	0.03522727
LONG TERM SYNAPTIC POTENTIATION	1.449768832	0.035270941
NEGATIVE REGULATION OF SECRETION	1.449377401	0.035374226
VASOCONSTRICTION	1.449374522	0.035374919
MULTICELLULAR ORGANISMAL SIGNALING	1.448862495	0.035507319
POSITIVE REGULATION OF JNK CASCADE	1.448554318	0.03558149
RESPONSE TO CAMP	1.445324274	0.036443126
RECEPTOR CLUSTERING	1.445179845	0.036477786
POSITIVE REGULATION OF BMP SIGNALING PATHWAY	1.44495613	0.036540866
CELL DIFFERENTIATION IN SPINAL CORD	1.444572504	0.036638606
CALCIUM ION IMPORT	1.444426117	0.036676039
POSITIVE REGULATION OF STRESS ACTIVATED PROTEIN KI	1.444253995	0.036716937
GLIAL CELL PROLIFERATION	1.443484256	0.036937372
REGULATION OF TRANSMEMBRANE TRANSPORT	1.443294479	0.03699352
REGULATION OF FAT CELL DIFFERENTIATION	1.442488217	0.03722158
SALIVARY GLAND DEVELOPMENT	1.442190181	0.037313774
REGULATION OF EPITHELIAL CELL MIGRATION	1.442045967	0.037362298
NEGATIVE REGULATION OF PEPTIDE SECRETION	1.441730128	0.037445481
CATION TRANSMEMBRANE TRANSPORT	1.440488565	0.037777519
REGULATION OF ADAPTIVE IMMUNE RESPONSE	1.440246097	0.037844759
NERVE DEVELOPMENT	1.439686048	0.038002113
CELL MATRIX ADHESION	1.439612833	0.038021522
POSITIVE REGULATION OF NEURON DIFFERENTIATION	1.438778872	0.038259287
NEGATIVE REGULATION OF CYTOKINE PRODUCTION	1.438291428	0.038404857
DIGESTIVE TRACT MORPHOGENESIS	1.438259061	0.038413868
REGULATION OF ALPHA BETA T CELL DIFFERENTIATION	1.436679163	0.038863056
REGULATION OF OSSIFICATION	1.436396983	0.038947625
MONOCARBOXYLIC ACID BIOSYNTHETIC PROCESS	1.435784653	0.039119537
NEGATIVE REGULATION OF ATP METABOLIC PROCESS	1.435613205	0.03917014
ENDOCYTOSIS	1.435128129	0.039303925
SULFUR COMPOUND METABOLIC PROCESS	1.434954702	0.039356608
REGULATION OF BLOOD CIRCULATION	1.434872088	0.039378097
LIPID OXIDATION	1.434821132	0.039392654
PLATELET DERIVED GROWTH FACTOR RECEPTOR SIGNALI	1.434801288	0.039399586
SMOOTH MUSCLE CONTRACTION	1.43266654	0.040019298
PHOSPHATIDYLINOSITOL METABOLIC PROCESS	1.431010039	0.040494827
REGULATION OF HORMONE BIOSYNTHETIC PROCESS	1.430556964	0.040626534
POSITIVE REGULATION OF PRODUCTION OF MOLECULAR M	1.43040912	0.040671591
REGULATION OF STRIATED MUSCLE CONTRACTION	1.430392526	0.040675057
EMBRYO IMPLANTATION	1.427810856	0.041409146
POSITIVE REGULATION OF PROTEIN TYROSINE KINASE ACT	1.427676058	0.041452124
REGULATION OF NEURON DIFFERENTIATION	1.426724521	0.041730787
LYMPH VESSEL MORPHOGENESIS	1.425521302	0.042069757
MUSCLE TISSUE DEVELOPMENT	1.424348147	0.042414966
POSITIVE REGULATION OF CD4 POSITIVE ALPHA BETA T CE	1.424039029	0.04252865
REGULATION OF CALCIUM ION TRANSPORT INTO CYTOSOL	1.424024587	0.042531422
MYOFIBRIL ASSEMBLY	1.423364253	0.042721357

REGULATION OF T CELL APOPTOTIC PROCESS	1.422671789	0.042932087
POSITIVE REGULATION OF ENDOTHELIAL CELL MIGRATION	1.422664323	0.042934166
BLOOD VESSEL ENDOTHELIAL CELL MIGRATION	1.421614787	0.043255114
REGULATION OF T CELL DIFFERENTIATION	1.421597111	0.043264125
NEURON PROJECTION GUIDANCE	1.420668091	0.043553186
NEGATIVE REGULATION OF CELL DIFFERENTIATION	1.419922813	0.043779859
POSITIVE REGULATION OF RESPONSE TO BIOTIC STIMULUS	1.419570472	0.043910872
LIPID BIOSYNTHETIC PROCESS	1.418998422	0.044108432
NEGATIVE REGULATION OF TRANSPORT	1.41866302	0.044211717
DICARBOXYLIC ACID TRANSPORT	1.418662118	0.04421241
NEGATIVE REGULATION OF AXONOGENESIS	1.418468695	0.044269945
ACTIN MEDIATED CELL CONTRACTION	1.418194919	0.044348969
NEGATIVE REGULATION OF SMOOTH MUSCLE CELL MIGRAT	1.415915674	0.045056024
POSITIVE REGULATION OF CD4 POSITIVE ALPHA BETA T CE	1.414843935	0.045401233
AXIS ELONGATION	1.414541318	0.045504518
RIBONUCLEOTIDE CATABOLIC PROCESS	1.414422075	0.045549576
VASCULAR ENDOTHELIAL GROWTH FACTOR RECEPTOR SIG	1.414195006	0.045623747
REGULATION OF ACTIN CYTOSKELETON REORGANIZATION	1.414088297	0.045659793
GLIOGENESIS	1.411002238	0.04664967
REGULATION OF BIOMINERALIZATION	1.410275949	0.046887434
POSITIVE REGULATION OF FIBROBLAST PROLIFERATION	1.408829196	0.047367815
REGULATION OF MYELOID LEUKOCYTE DIFFERENTIATION	1.408604212	0.047435055
INOSITOL LIPID MEDIATED SIGNALING	1.407987649	0.047635387
IMMUNOGLOBULIN PRODUCTION	1.407690823	0.047731741
REGULATION OF FATTY ACID OXIDATION	1.407319018	0.047876618
INTERLEUKIN 2 PRODUCTION	1.407134455	0.047945243
REGULATION OF OXIDOREDUCTASE ACTIVITY	1.406532669	0.048130325
HYALURONAN METABOLIC PROCESS	1.406147078	0.048261339
SYNAPTIC TRANSMISSION GLUTAMATERGIC	1.405710316	0.048397897
NEGATIVE REGULATION OF EXOCYTOSIS	1.405229046	0.048567036
POSITIVE REGULATION OF GLIAL CELL DIFFERENTIATION	1.404717542	0.048729936
DETECTION OF CHEMICAL STIMULUS	1.404676701	0.048744493
POSITIVE REGULATION OF PRI MIRNA TRANSCRIPTION BY F	1.403534795	0.049127827
JNK CASCADE	1.40337129	0.049183976
REGULATION OF JNK CASCADE	1.402966458	0.049324693
REGULATION OF PEPTIDYL TYROSINE PHOSPHORYLATION	1.401769997	0.049796756
REGULATION OF NEUROTRANSMITTER LEVELS	1.401749417	0.049806461
VENTRAL SPINAL CORD DEVELOPMENT	1.401529298	0.04987786
ORGANIC ACID BIOSYNTHETIC PROCESS	1.401276687	0.049970747
NEUROTRANSMITTER TRANSPORT	1.400544718	0.050224455
NEGATIVE REGULATION OF NERVOUS SYSTEM DEVELOPME	1.399456072	0.050598778
LIPID MODIFICATION	1.398969354	0.050781781
REGULATION OF REPRODUCTIVE PROCESS	1.398737931	0.050871896
TOLL LIKE RECEPTOR 3 SIGNALING PATHWAY	1.39842721	0.050982806
DEFINITIVE HEMOPOIESIS	1.398196993	0.051067375
BEHAVIOR	1.398131035	0.05109233
STRIATED MUSCLE CELL DIFFERENTIATION	1.3966563	0.051617076
REGULATION OF ANATOMICAL STRUCTURE SIZE	1.396462281	0.051693327
NEURON MATURATION	1.396291079	0.051757794
B CELL HOMEOSTASIS	1.396275189	0.051764725
REGULATION OF T CELL MEDIATED IMMUNITY	1.396130742	0.051809783
RESPONSE TO NUTRIENT	1.39603085	0.051845136
POSITIVE REGULATION OF CELL DEATH	1.395898051	0.051894352

REGULATION OF CELLULAR RESPONSE TO GROWTH FACTO	1.393684795	0.052676965
NEGATIVE REGULATION OF LYMPHOCYTE APOPTOTIC PRO	1.393279481	0.052825308
ENDODERMAL CELL DIFFERENTIATION	1.392750578	0.053017321
REGULATION OF GTPASE ACTIVITY	1.391946252	0.053295984
AMINE METABOLIC PROCESS	1.391476747	0.053491464
REGULATION OF MAP KINASE ACTIVITY	1.391260509	0.053576727
REGULATION OF CARDIAC MUSCLE CONTRACTION	1.390961607	0.053689023
NEGATIVE REGULATION OF CATION CHANNEL ACTIVITY	1.390896841	0.053713285
VASCULAR ASSOCIATED SMOOTH MUSCLE CELL DIFFEREN	1.38978434	0.054109097
PEPTIDE CROSS LINKING	1.389385872	0.054258826
CARDIAC ATRIUM DEVELOPMENT	1.389334772	0.054278236
REGULATION OF VASOCONSTRICTION	1.389088291	0.054359339
POSITIVE REGULATION OF STEROL TRANSPORT	1.389072167	0.054364885
REGULATION OF CYSTEINE TYPE ENDOPEPTIDASE ACTIVIT	1.387284993	0.055029655
NEGATIVE REGULATION OF SMOOTHENED SIGNALING PATH	1.387162613	0.055076098
MEMBRANE BIOGENESIS	1.386328855	0.055368625
POSITIVE REGULATION OF T CELL MIGRATION	1.386216464	0.05539358
REGULATION OF LONG TERM SYNAPTIC POTENTIATION	1.386125335	0.055430319
NEGATIVE REGULATION OF EPITHELIAL CELL PROLIFERATI	1.386098843	0.055438637
PRIMARY ALCOHOL METABOLIC PROCESS	1.385986017	0.055478149
RESPONSE TO AMYLOID BETA	1.385582231	0.055635504
REGULATION OF RECEPTOR SIGNALING PATHWAY VIA STA	1.384905501	0.055900303
NEPHRON DEVELOPMENT	1.384584454	0.056013293
MYELOID DENDRITIC CELL DIFFERENTIATION	1.384469487	0.056054884
OSTEOCLAST DIFFERENTIATION	1.384047572	0.056203227
REGULATION OF EXOCYTOSIS	1.383700114	0.05631691
ORGANIC HYDROXY COMPOUND CATABOLIC PROCESS	1.38343632	0.056404252
ACTIN CYTOSKELETON REORGANIZATION	1.383202721	0.056506845
SENSORY ORGAN DEVELOPMENT	1.382747558	0.056704404
POSITIVE CHEMOTAXIS	1.382547284	0.05677095
GENITALIA DEVELOPMENT	1.382456268	0.056811155
SMALL GTPASE MEDIATED SIGNAL TRANSDUCTION	1.381896375	0.057040602
ENDODERM FORMATION	1.3816582	0.057135569
CRANIAL NERVE DEVELOPMENT	1.381011269	0.057392049
CARDIAC MUSCLE CONTRACTION	1.380263966	0.057675564
RESPONSE TO FIBROBLAST GROWTH FACTOR	1.379710238	0.057894613
ACTIVATION OF MAPK ACTIVITY	1.377537776	0.058777738
POSITIVE REGULATION OF LIPID LOCALIZATION	1.37670895	0.059091754
MESENCHYME DEVELOPMENT	1.376092367	0.059370416
ACTIVATION OF INNATE IMMUNE RESPONSE	1.375059189	0.059767615
POSITIVE REGULATION OF LIPID BIOSYNTHETIC PROCESS	1.374993332	0.059794649
REGULATION OF HEMOPOIESIS	1.37492468	0.059820297
ORGANIC HYDROXY COMPOUND METABOLIC PROCESS	1.374787268	0.059874366
POSITIVE REGULATION OF GTPASE ACTIVITY	1.374123095	0.060162734
POSITIVE REGULATION OF REACTIVE OXYGEN SPECIES ME	1.37371642	0.060338804
SKELETAL MUSCLE CONTRACTION	1.373685408	0.060352668
REGULATION OF TOLL LIKE RECEPTOR SIGNALING PATHWA	1.372943972	0.060687479
REGULATION OF RENAL SYSTEM PROCESS	1.372459918	0.0608788
ERYTHROCYTE DEVELOPMENT	1.371565713	0.061277385
POSITIVE REGULATION OF PEPTIDASE ACTIVITY	1.371215059	0.061429887
GLYCOLIPID BIOSYNTHETIC PROCESS	1.371133999	0.06146108
NEURON PROJECTION EXTENSION INVOLVED IN NEURON P	1.369670276	0.062077327
CD8 POSITIVE ALPHA BETA T CELL ACTIVATION	1.368206236	0.062678323

REGULATION OF SYSTEMIC ARTERIAL BLOOD PRESSURE	1.368084374	0.062735165
POSITIVE REGULATION OF SMALL MOLECULE METABOLIC P	1.367937936	0.062801018
ANIMAL ORGAN REGENERATION	1.36742932	0.063017294
AMEBOIDAL TYPE CELL MIGRATION	1.365586755	0.063845657
INORGANIC ION TRANSMEMBRANE TRANSPORT	1.365074775	0.064061239
ADENYLATE CYCLASE INHIBITING G PROTEIN COUPLED RE(	1.364469634	0.064336436
POSITIVE REGULATION OF MAP KINASE ACTIVITY	1.364465311	0.064337129
POSITIVE REGULATION OF ADAPTIVE IMMUNE RESPONSE	1.364334013	0.064378028
NEGATIVE REGULATION OF LIPID BIOSYNTHETIC PROCESS	1.364092274	0.064468836
NEGATIVE REGULATION OF STEROID METABOLIC PROCESS	1.363910592	0.064542314
POSITIVE REGULATION OF EPITHELIAL CELL MIGRATION	1.36356577	0.064694123
REGULATION OF LYMPHOCYTE APOPTOTIC PROCESS	1.360939274	0.065853831
CELL DIFFERENTIATION INVOLVED IN KIDNEY DEVELOPMEN	1.360526097	0.066030595
LOCOMOTORY BEHAVIOR	1.360248325	0.066152596
LIPID STORAGE	1.359742355	0.066373724
FC RECEPTOR MEDIATED STIMULATORY SIGNALING PATHW	1.359722214	0.066384815
TRANSMEMBRANE RECEPTOR PROTEIN SERINE THREONINI	1.358677806	0.066863117
SMOOTH MUSCLE CELL DIFFERENTIATION	1.357727628	0.067285963
NEGATIVE REGULATION OF CYSTEINE TYPE ENDOPEPTIDAS	1.35709243	0.067572944
REGULATION OF CELL SUBSTRATE ADHESION	1.356359175	0.067901517
PATTERN RECOGNITION RECEPTOR SIGNALING PATHWAY	1.356192079	0.067968063
POSITIVE REGULATION OF EPITHELIAL CELL PROLIFERATIO	1.355821048	0.068148986
REGULATION OF CARDIAC CONDUCTION	1.355293003	0.068395762
REGULATION OF LIPID BIOSYNTHETIC PROCESS	1.354885806	0.068575992
NERVOUS SYSTEM PROCESS	1.353735781	0.069085487
OSTEOBLAST PROLIFERATION	1.353415144	0.069230364
OVULATION CYCLE	1.352834756	0.069506254
REGULATION OF NEURON PROJECTION DEVELOPMENT	1.352082777	0.069854929
POSITIVE REGULATION OF CELLULAR CARBOHYDRATE MET	1.350162207	0.070731122
REGULATION OF NEUROTRANSMITTER TRANSPORT	1.349762039	0.070932841
CHONDROCYTE DIFFERENTIATION	1.349434502	0.071102673
NEGATIVE REGULATION OF MYELOID LEUKOCYTE DIFFERE	1.347754641	0.071889444
NEGATIVE REGULATION OF IMMUNE RESPONSE	1.34773622	0.071894297
POSITIVE REGULATION OF SIGNALING RECEPTOR ACTIVITY	1.34637319	0.072543123
MUSCLE ORGAN MORPHOGENESIS	1.345819854	0.072834957
REGULATION OF IMMUNOGLOBULIN PRODUCTION	1.345453638	0.073022118
REGULATION OF LIPID LOCALIZATION	1.344866513	0.073296622
ALCOHOL BIOSYNTHETIC PROCESS	1.34404973	0.073704911
MEMBRANE INVAGINATION	1.343781923	0.073849788
LIPID CATABOLIC PROCESS	1.343497238	0.073992586
REGULATION OF STRESS ACTIVATED PROTEIN KINASE SIGI	1.342919703	0.074298976
INFLAMMATORY RESPONSE TO ANTIGENIC STIMULUS	1.342863891	0.074321851
REGULATION OF RESPONSE TO OXIDATIVE STRESS	1.342736245	0.074380773
AXON DEVELOPMENT	1.342317207	0.074579718
ETHER METABOLIC PROCESS	1.341838553	0.07481471
NEGATIVE REGULATION OF DNA BINDING TRANSCRIPTION I	1.341835978	0.074816096
AXON EXTENSION	1.341690003	0.074891654
SKELETAL SYSTEM DEVELOPMENT	1.341258461	0.075085748
SMAD PROTEIN SIGNAL TRANSDUCTION	1.340771619	0.075347081
REACTIVE OXYGEN SPECIES BIOSYNTHETIC PROCESS	1.340387131	0.075547413
ORGANIC HYDROXY COMPOUND BIOSYNTHETIC PROCESS	1.340374856	0.075556424
ACTIVATED T CELL PROLIFERATION	1.340365062	0.075561277
SPROUTING ANGIOGENESIS	1.340081575	0.075704074

STRESS FIBER ASSEMBLY	1.3386962	0.076410436
ADAPTIVE THERMOGENESIS	1.338280728	0.076612847
REGULATION OF REACTIVE OXYGEN SPECIES BIOSYNTHET	1.338074445	0.07671544
EAR DEVELOPMENT	1.338029546	0.076742474
FATTY ACID METABOLIC PROCESS	1.337313618	0.077093229
POSITIVE REGULATION OF HEART GROWTH	1.337262332	0.077116104
POSITIVE REGULATION OF PROTEIN KINASE ACTIVITY	1.337041231	0.07723256
REGULATION OF RESPONSE TO CYTOKINE STIMULUS	1.336818506	0.077346937
NEGATIVE REGULATION OF CELL CELL ADHESION	1.336269239	0.077622133
FATTY ACID BIOSYNTHETIC PROCESS	1.336236642	0.077635304
BONE DEVELOPMENT	1.336206775	0.077649168
PURINE CONTAINING COMPOUND CATABOLIC PROCESS	1.335996263	0.077764931
ACTIVATION OF PROTEIN KINASE B ACTIVITY	1.335698273	0.077927137
NEGATIVE REGULATION OF INTRACELLULAR TRANSPORT	1.333957639	0.07880749
REGULATION OF STEROID METABOLIC PROCESS	1.333478367	0.079020993
LEARNING	1.333098155	0.0792137
INNER EAR MORPHOGENESIS	1.332696432	0.079425816
CELLULAR RESPONSE TO NITROGEN COMPOUND	1.332038469	0.07976548
REGULATION OF GLIAL CELL PROLIFERATION	1.331727126	0.079918675
LYMPHOCYTE APOPTOTIC PROCESS	1.331209872	0.080180008
POSTSYNAPTIC MEMBRANE ORGANIZATION	1.331188934	0.080189712
POSITIVE REGULATION OF CYTOSKELETON ORGANIZATION	1.330345349	0.08065831
REGULATION OF RECEPTOR MEDIATED ENDOCYTOSIS	1.330285869	0.080688117
REGULATION OF GLIOGENESIS	1.330045215	0.080815664
COENZYME A METABOLIC PROCESS	1.329949101	0.080878051
NEGATIVE REGULATION OF CANONICAL WNT SIGNALING PA	1.329564982	0.081068679
MUSCLE FIBER DEVELOPMENT	1.329067138	0.081322386
CARBOHYDRATE DERIVATIVE CATABOLIC PROCESS	1.327245491	0.082273444
REGULATION OF ACTIN FILAMENT BASED PROCESS	1.326908291	0.08245506
ACTIN FILAMENT ORGANIZATION	1.326521886	0.082644302
MAINTENANCE OF LOCATION IN CELL	1.325984758	0.082915339
ALCOHOL CATABOLIC PROCESS	1.3258665	0.082986738
SIGNAL RELEASE	1.325740333	0.083048432
REACTIVE NITROGEN SPECIES METABOLIC PROCESS	1.325568667	0.083135774
POSITIVE REGULATION OF CYTOSOLIC CALCIUM ION CONC	1.325467244	0.083196082
NEGATIVE REGULATION OF ADAPTIVE IMMUNE RESPONSE	1.325195008	0.083333333
BIOLOGICAL PROCESS INVOLVED IN INTRASPECIES INTERA	1.325032091	0.083412357
REGULATION OF DNA BINDING TRANSCRIPTION FACTOR AC	1.32329202	0.084360642
REGULATION OF BONE MINERALIZATION	1.32197889	0.085124539
POSITIVE REGULATION OF NF KAPPAB TRANSCRIPTION FAC	1.321399326	0.085453804
REGULATION OF ENDOCYTOSIS	1.320635757	0.08587873
CAMERA TYPE EYE MORPHOGENESIS	1.320556134	0.085923095
REGULATION OF TRANSMEMBRANE RECEPTOR PROTEIN SI	1.320300384	0.086061733
NEGATIVE REGULATION OF CELL POPULATION PROLIFERAT	1.3189264	0.086816618
HEART PROCESS	1.317587792	0.087522979
REGULATION OF TRANSPORTER ACTIVITY	1.316843117	0.087945826
NEGATIVE REGULATION OF TRANSMEMBRANE RECEPTOR P	1.316132783	0.08831391
T HELPER 17 TYPE IMMUNE RESPONSE	1.316043293	0.088359661
NEGATIVE REGULATION OF CELLULAR RESPONSE TO INSUI	1.315233557	0.08881786
ACYLGLYCEROL HOMEOSTASIS	1.315134862	0.088868463
PTERIDINE CONTAINING COMPOUND METABOLIC PROCESS	1.314513995	0.089235161
FATTY ACID DERIVATIVE METABOLIC PROCESS	1.313473392	0.089845862
NEGATIVE REGULATION OF PEPTIDE HORMONE SECRETION	1.313296558	0.089961625

SKELETAL MUSCLE CELL DIFFERENTIATION	1.313159036	0.090046887
ARTERY MORPHOGENESIS	1.312855648	0.090207015
NEGATIVE REGULATION OF ION TRANSPORT	1.312612986	0.090329016
INNATE IMMUNE RESPONSE ACTIVATING SIGNAL TRANSDU(	1.312561546	0.09036021
ANTIGEN PROCESSING AND PRESENTATION	1.312346702	0.090485677
RESPONSE TO PROSTAGLANDIN	1.311431394	0.09102498
REGULATION OF VASCULAR ASSOCIATED SMOOTH MUSCLE	1.310992737	0.091272449
SULFUR COMPOUND BIOSYNTHETIC PROCESS	1.310986493	0.091275915
ENDODERM DEVELOPMENT	1.309889698	0.091922662
STEROID METABOLIC PROCESS	1.309704656	0.092040505
PRODUCTION OF MOLECULAR MEDIATOR OF IMMUNE RESP	1.309539052	0.09214171
ACTIN FILAMENT BUNDLE ORGANIZATION	1.30950917	0.092158347
LYTIC VACUOLE ORGANIZATION	1.308895955	0.092494545
REGULATION OF NITRIC OXIDE METABOLIC PROCESS	1.308282173	0.092833515
NEUROMUSCULAR PROCESS CONTROLLING BALANCE	1.307550895	0.093277157
ASTROCYTE DEVELOPMENT	1.307446108	0.093328453
POSITIVE REGULATION OF BONE MINERALIZATION	1.307217526	0.093440057
POTASSIUM ION TRANSPORT	1.306405904	0.093904495
REGULATION OF HEART CONTRACTION	1.306232812	0.094003621
CELLULAR HORMONE METABOLIC PROCESS	1.305775606	0.094296148
MUSCLE CELL DIFFERENTIATION	1.305630773	0.094394581
SYNAPTIC SIGNALING	1.305258016	0.094619175
REGULATION OF ALCOHOL BIOSYNTHETIC PROCESS	1.30426835	0.095231956
RESPONSE TO METAL ION	1.303980834	0.095385844
MULTICELLULAR ORGANISMAL MOVEMENT	1.303870513	0.095441992
RESPONSE TO STEROL	1.303866078	0.095447538
NEGATIVE REGULATION OF IMMUNE EFFECTOR PROCESS	1.303505841	0.095671439
POSITIVE REGULATION OF REGULATED SECRETORY PATHW	1.302562856	0.096258571
PRI MIRNA TRANSCRIPTION BY RNA POLYMERASE II	1.302206996	0.096476233
REGULATION OF PROTEIN TYROSINE KINASE ACTIVITY	1.302192116	0.096483858
NEGATIVE REGULATION OF ANION TRANSPORT	1.302165571	0.096503268
INORGANIC ION IMPORT ACROSS PLASMA MEMBRANE	1.301981479	0.096607246
COGNITION	1.301856972	0.096675872
CELL FATE DETERMINATION	1.300383578	0.097592271
REGULATION OF MEIOTIC CELL CYCLE	1.299384169	0.098216836
POSITIVE REGULATION OF BIOMINERALIZATION	1.298540993	0.098760991
REGULATION OF CATION TRANSMEMBRANE TRANSPORT	1.298017901	0.099085404
RESPONSE TO VITAMIN D	1.297097743	0.099660752
REGULATION OF INSULIN RECEPTOR SIGNALING PATHWAY	1.296196172	0.100219464
POSITIVE REGULATION OF NEURON PROJECTION DEVELOP	1.295844	0.100435047
POSITIVE REGULATION OF CATION TRANSMEMBRANE TRAN	1.294777037	0.101112987
RENAL WATER HOMEOSTASIS	1.293580249	0.101838758
MESENCHYMAL CELL DIFFERENTIATION	1.293481965	0.101905305
NEGATIVE REGULATION OF HORMONE SECRETION	1.293345457	0.101986408
SMOOTH MUSCLE TISSUE DEVELOPMENT	1.293082638	0.102140989
LIPID PHOSPHORYLATION	1.293077826	0.102143069
NEGATIVE REGULATION OF AXON EXTENSION INVOLVED IN	1.293067502	0.102148615
NEGATIVE REGULATION OF MYELOID CELL DIFFERENTIATIO	1.293067408	0.102149308
CELL MORPHOGENESIS INVOLVED IN DIFFERENTIATION	1.292144828	0.102735054
ATP SYNTHESIS COUPLED ELECTRON TRANSPORT	1.291855713	0.102926375
REGULATION OF MYELINATION	1.291090367	0.103434484
STRIATED MUSCLE CELL DEVELOPMENT	1.290858359	0.103589759
POSITIVE REGULATION OF NUCLEAR DIVISION	1.29053497	0.103802568

NITRIC OXIDE SYNTHASE BIOSYNTHETIC PROCESS	1.29045296	0.103858717
NEGATIVE REGULATION OF RELEASE OF CYTOCHROME C F	1.289736734	0.104316916
AMYLOID BETA CLEARANCE	1.288547243	0.105111313
CENTRAL NERVOUS SYSTEM NEURON DIFFERENTIATION	1.287738918	0.105634672
CAMERA TYPE EYE DEVELOPMENT	1.286664695	0.106335488
C4 DICARBOXYLATE TRANSPORT	1.286306274	0.106570479
CELL MORPHOGENESIS INVOLVED IN NEURON DIFFERENTIA	1.285656391	0.107018974
REGULATION OF SIGNALING RECEPTOR ACTIVITY	1.285612573	0.107046008
NEGATIVE REGULATION OF LEUKOCYTE CELL CELL ADHESI	1.285598291	0.107054327
LIVER MORPHOGENESIS	1.285452056	0.107143748
EPITHELIAL TUBE BRANCHING INVOLVED IN LUNG MORPHO	1.284959003	0.107473707
POSITIVE REGULATION OF CYSTEINE TYPE ENDOPEPTIDAS	1.284389357	0.107838326
NEGATIVE REGULATION OF INTRACELLULAR PROTEIN TRAN	1.283581987	0.108381094
REGULATION OF LIPID STORAGE	1.283258546	0.108607768
NEGATIVE REGULATION OF ENDOTHELIAL CELL APOPTOTIC	1.282665076	0.1090015
POSITIVE REGULATION OF P38MAPK CASCADE	1.282614597	0.10903616
EPITHELIAL CELL DIFFERENTIATION INVOLVED IN KIDNEY D	1.282504952	0.109103399
REGULATION OF ACTIN FILAMENT ORGANIZATION	1.282371355	0.109181037
REGULATION OF MEIOTIC NUCLEAR DIVISION	1.281722113	0.10961844
POSITIVE REGULATION OF FAT CELL DIFFERENTIATION	1.281220634	0.109963649
BRANCH ELONGATION OF AN EPITHELIUM	1.28095428	0.110144572
POSITIVE REGULATION OF B CELL MEDIATED IMMUNITY	1.280529809	0.110420462
REGULATION OF PRODUCTION OF MOLECULAR MEDIATOR (	1.280471788	0.110456508
POSITIVE REGULATION OF ORGAN GROWTH	1.28037925	0.110519588
EXTRACELLULAR MATRIX DISASSEMBLY	1.279978831	0.110788546
PHOSPHATIDYLINOSITOL 3 KINASE SIGNALING	1.279442664	0.111130289
POSITIVE REGULATION OF CATION CHANNEL ACTIVITY	1.278538614	0.111737525
REGULATION OF POSITIVE CHEMOTAXIS	1.278137943	0.112016881
AMINE CATABOLIC PROCESS	1.2768841	0.112872971
NEGATIVE REGULATION OF CELL SUBSTRATE ADHESION	1.275595647	0.11373738
NEGATIVE REGULATION OF HEMOPOIESIS	1.275252606	0.113979997
POSITIVE REGULATION OF MORPHOGENESIS OF AN EPITHE	1.27491966	0.114192114
NEGATIVE REGULATION OF CELL SUBSTRATE JUNCTION OF	1.274694463	0.114350161
NEGATIVE REGULATION OF ERK1 AND ERK2 CASCADE	1.27467709	0.114362639
ENDOCHONDRAL BONE MORPHOGENESIS	1.273578837	0.115126535
TISSUE REMODELING	1.272929247	0.115559086
TRANSFORMING GROWTH FACTOR BETA PRODUCTION	1.272861373	0.115614541
CELLULAR RESPONSE TO EXTERNAL STIMULUS	1.272586988	0.115803783
INNERVATION	1.27208705	0.116153844
NON CANONICAL WNT SIGNALING PATHWAY	1.272010135	0.116214845
NEURAL CREST CELL MIGRATION	1.271821626	0.116361108
REGULATION OF MUSCLE CONTRACTION	1.271686676	0.116462314
LAMELLIPODIUM ASSEMBLY	1.271290445	0.116736124
POSITIVE REGULATION OF OSTEOBLAST DIFFERENTIATION	1.271012369	0.116928138
UROGENITAL SYSTEM DEVELOPMENT	1.27021782	0.117502794
NEGATIVE REGULATION OF APOPTOTIC SIGNALING PATHW	1.269420702	0.118074676
NEURON FATE COMMITMENT	1.268125324	0.11896612
CHLORIDE TRANSPORT	1.267838634	0.119171997
REGULATION OF CELLULAR KETONE METABOLIC PROCESS	1.267734451	0.119256567
MONOCARBOXYLIC ACID METABOLIC PROCESS	1.266401404	0.120229807
REGULATION OF SMOOTHENED SIGNALING PATHWAY	1.265465738	0.120902895
NEGATIVE REGULATION OF MAP KINASE ACTIVITY	1.264027197	0.121971795
RECEPTOR INTERNALIZATION	1.263792343	0.122138854

TOLL LIKE RECEPTOR 2 SIGNALING PATHWAY	1.263006729	0.122716283
EXTRACELLULAR MATRIX ASSEMBLY	1.262692551	0.122951274
MONOAMINE TRANSPORT	1.262340602	0.123213994
POSITIVE REGULATION OF PROTEIN EXPORT FROM NUCLEU	1.26229373	0.12324796
OSTEOBLAST DIFFERENTIATION	1.261873311	0.123560589
REGULATION OF SMOOTH MUSCLE CELL DIFFERENTIATION	1.261295581	0.124022254
MAMMARY GLAND MORPHOGENESIS	1.260444122	0.124696729
ANTIBACTERIAL HUMORAL RESPONSE	1.259988015	0.125014904
POSITIVE REGULATION OF LIPID TRANSPORT	1.258345364	0.126271659
MEMBRANE PROTEIN PROTEOLYSIS	1.258090823	0.1264609
NADH DEHYDROGENASE COMPLEX ASSEMBLY	1.257466367	0.126916326
POSITIVE REGULATION OF NERVOUS SYSTEM PROCESS	1.257412629	0.126955145
REGULATION OF MORPHOGENESIS OF AN EPITHELIUM	1.257204076	0.127115965
ASSOCIATIVE LEARNING	1.257095376	0.127212319
MEMORY	1.256580978	0.127603972
MAMMARY GLAND DUCT MORPHOGENESIS	1.255700923	0.128275674
TISSUE HOMEOSTASIS	1.25336559	0.130084209
HOMEOSTASIS OF NUMBER OF CELLS WITHIN A TISSUE	1.253155275	0.130258893
NEPHRON EPITHELIUM DEVELOPMENT	1.253046005	0.130329598
NEGATIVE REGULATION OF FATTY ACID METABOLIC PROCE	1.252952105	0.130405849
POSITIVE REGULATION OF SECRETION	1.25202139	0.131135779
RESPONSE TO CATECHOLAMINE	1.251378165	0.131632104
POSITIVE REGULATION OF EPITHELIAL TO MESENCHYMAL T	1.250118666	0.132631685
POSITIVE REGULATION OF BLOOD VESSEL ENDOTHELIAL C	1.250117918	0.132632379
CELLULAR RESPONSE TO EXTRACELLULAR STIMULUS	1.249693976	0.132952633
RESPONSE TO DRUG	1.249608266	0.133027497
ZINC ION HOMEOSTASIS	1.249007351	0.133485697
REGULATION OF RESPONSE TO BIOTIC STIMULUS	1.248954792	0.133529368
NEGATIVE REGULATION OF INTRINSIC APOPTOTIC SIGNALI	1.248596764	0.133831599
LUNG CELL DIFFERENTIATION	1.248490196	0.133911316
ARTERY DEVELOPMENT	1.248385691	0.133984101
RAS PROTEIN SIGNAL TRANSDUCTION	1.247849721	0.134397243
MYELOID CELL HOMEOSTASIS	1.247754501	0.134472107
NEGATIVE REGULATION OF EPITHELIAL CELL APOPTOTIC P	1.246285744	0.135646373
ANOIKIS	1.245857622	0.135972173
AMYLOID FIBRIL FORMATION	1.245657928	0.136148936
BONE MORPHOGENESIS	1.245215228	0.136458793
POSITIVE REGULATION OF CELL SUBSTRATE ADHESION	1.245180251	0.136484441
REGULATION OF SMALL GTPASE MEDIATED SIGNAL TRANSI	1.244342516	0.137165154
NEURON PROJECTION ARBORIZATION	1.244134266	0.137334986
POSITIVE REGULATION OF OLIGODENDROCYTE DIFFERENT	1.243492073	0.137870129
NEGATIVE REGULATION OF WNT SIGNALING PATHWAY	1.243147072	0.138130076
NEGATIVE REGULATION OF DNA BINDING	1.242899318	0.138336647
KIDNEY EPITHELIUM DEVELOPMENT	1.242608837	0.138562627
REGULATION OF PROTEOLYSIS	1.242589677	0.138586196
REGULATION OF CELLULAR RESPIRATION	1.242179557	0.138916154
MONOCARBOXYLIC ACID TRANSPORT	1.241830532	0.139213533
POSITIVE REGULATION OF ION TRANSPORT	1.241713996	0.139305728
NEGATIVE REGULATION OF VASCULATURE DEVELOPMENT	1.241561856	0.139431195
REGULATION OF TISSUE REMODELING	1.241244855	0.139677971
PHOTORECEPTOR CELL DIFFERENTIATION	1.240742518	0.140067545
NEGATIVE REGULATION OF STRIATED MUSCLE CELL APOPT	1.240691945	0.140111909
REGIONALIZATION	1.239825562	0.140851543

REGULATION OF MYELOID CELL DIFFERENTIATION	1.239824359	0.140851543
NEGATIVE REGULATION OF CELL DEVELOPMENT	1.238384116	0.142081957
ACTIVATION OF PROTEIN KINASE ACTIVITY	1.237517194	0.14281674
REGULATION OF RELEASE OF SEQUESTERED CALCIUM ION	1.237504717	0.142822978
REGULATION OF ANIMAL ORGAN MORPHOGENESIS	1.237493573	0.142833376
RESPIRATORY SYSTEM DEVELOPMENT	1.237355688	0.142950526
NEGATIVE REGULATION OF RESPONSE TO CYTOKINE STIMU	1.237160698	0.143114812
POSITIVE REGULATION OF PROTEOLYSIS	1.237108209	0.143150165
RESPONSE TO MURAMYL DIPEPTIDE	1.237104136	0.14315571
ENDOTHELIUM DEVELOPMENT	1.237032116	0.143213938
MAINTENANCE OF LOCATION	1.236934405	0.143309598
NEGATIVE REGULATION OF ENDOCYTOSIS	1.236844085	0.143378918
ACTIN NUCLEATION	1.236742381	0.143464873
POSITIVE REGULATION OF OSSIFICATION	1.23641731	0.143750468
RESPONSE TO EXTRACELLULAR STIMULUS	1.235970421	0.144115086
REGULATION OF OSTEOBLAST PROLIFERATION	1.234715748	0.145140316
REGULATION OF CD4 POSITIVE ALPHA BETA T CELL ACTIVA	1.234212459	0.14558881
POSITIVE REGULATION OF STRIATED MUSCLE CELL DIFFER	1.233856136	0.145877871
BONE REMODELING	1.233352681	0.146313888
CELL CELL RECOGNITION	1.233301204	0.146352707
O GLYCAN PROCESSING	1.233043159	0.146573141
REGULATION OF FATTY ACID TRANSPORT	1.232907841	0.14669445
REGULATION OF LIPID TRANSPORT	1.232877074	0.146720791
POSITIVE REGULATION OF TRANSFERASE ACTIVITY	1.232688744	0.146878145
POSITIVE REGULATION OF FATTY ACID METABOLIC PROCES	1.232310466	0.147215036
SKELETAL SYSTEM MORPHOGENESIS	1.232109886	0.1473918
APOPTOTIC PROCESS INVOLVED IN DEVELOPMENT	1.231807714	0.147646894
POSITIVE REGULATION OF I KAPPAB KINASE NF KAPPAB SIG	1.231346762	0.148047558
RESPONSE TO HYDROPEROXIDE	1.230823161	0.148519621
DIGESTION	1.230178115	0.14907764
NEGATIVE REGULATION OF CELL MATRIX ADHESION	1.229263252	0.149891446
EMBRYONIC FORELIMB MORPHOGENESIS	1.228267333	0.150752389
POSITIVE REGULATION OF COLD INDUCED THERMOGENESI	1.227686092	0.15126951
POSITIVE REGULATION OF DNA BINDING TRANSCRIPTION F	1.227172543	0.151696515
REGULATION OF ANTIGEN RECEPTOR MEDIATED SIGNALING	1.22711744	0.151750584
POSITIVE REGULATION OF ORGANIC ACID TRANSPORT	1.227022594	0.151842779
NEGATIVE REGULATION OF CELL JUNCTION ASSEMBLY	1.226842785	0.151998054
STRESS ACTIVATED PROTEIN KINASE SIGNALING CASCADE	1.226793065	0.152050736
REGULATION OF CELL DEVELOPMENT	1.226395758	0.152379309
P38MAPK CASCADE	1.22547709	0.153177864
MYELOID CELL APOPTOTIC PROCESS	1.225415582	0.153226388
PREASSEMBLY OF GPI ANCHOR IN ER MEMBRANE	1.22537303	0.153276298
ASTROCYTE DIFFERENTIATION	1.222920615	0.155439053
T CELL HOMEOSTASIS	1.221849496	0.156423384
CELLULAR COMPONENT ASSEMBLY INVOLVED IN MORPHOG	1.221424382	0.156806026
GLAND MORPHOGENESIS	1.221393102	0.15683306
REGULATION OF SYNAPTIC VESICLE EXOCYTOSIS	1.221047308	0.157183815
SENSORY PERCEPTION OF PAIN	1.220939303	0.157276702
POSITIVE REGULATION OF DEPHOSPHORYLATION	1.220096898	0.158038519
CHOLESTEROL EFFLUX	1.219916343	0.158204885
REGULATION OF EXTRINSIC APOPTOTIC SIGNALING PATHW	1.219527351	0.158559106
RELEASE OF SEQUESTERED CALCIUM ION INTO CYTOSOL E	1.21941335	0.158652686
SUBSTRATE DEPENDENT CELL MIGRATION	1.219245081	0.158796177

POSITIVE REGULATION OF CELL DEVELOPMENT	1.218657339	0.159334093
NEGATIVE REGULATION OF MAPK CASCADE	1.217607411	0.160308026
KIDNEY MORPHOGENESIS	1.217551138	0.160366254
SYNAPSE ORGANIZATION	1.216837699	0.160991513
EPITHELIAL TO MESENCHYMAL TRANSITION	1.216738185	0.161080241
REGULATION OF FIBROBLAST GROWTH FACTOR RECEPTOI	1.216625505	0.161185606
FORMATION OF PRIMARY GERM LAYER	1.215705339	0.162031992
CARDIAC EPITHELIAL TO MESENCHYMAL TRANSITION	1.215662593	0.162059027
MITOCHONDRIAL RESPIRATORY CHAIN COMPLEX ASSEMBL	1.215148464	0.162538715
DEVELOPMENTAL GROWTH INVOLVED IN MORPHOGENESIS	1.214617899	0.163019096
NEGATIVE REGULATION OF ALPHA BETA T CELL ACTIVATIO	1.214584942	0.163050983
RESPONSE TO VITAMIN	1.214050574	0.163540376
GLUTATHIONE METABOLIC PROCESS	1.21388493	0.163683866
STEROL HOMEOSTASIS	1.213629903	0.163914006
PHOSPHOLIPID METABOLIC PROCESS	1.211601643	0.165867418
DEVELOPMENTAL GROWTH	1.211486754	0.165977635
NEGATIVE REGULATION OF DEFENSE RESPONSE	1.21093421	0.166510006
PINOCYTOSIS	1.210009977	0.167361244
RESPONSE TO CORTICOSTEROID	1.209786622	0.16756019
MUSCLE CELL DEVELOPMENT	1.209620198	0.16771893
MITOCHONDRIAL ELECTRON TRANSPORT CYTOCHROME C	1.209544232	0.167793795
VASCULAR TRANSPORT	1.208943804	0.168367064
NEGATIVE REGULATION OF PROTEOLYSIS	1.208792039	0.168525112
PHAGOSOME MATURATION	1.208669355	0.168635329
NEGATIVE REGULATION OF MUSCLE CELL APOPTOTIC PRO	1.208521637	0.16877466
EPIDERMIS MORPHOGENESIS	1.208355449	0.168925776
ENSHEATHMENT OF NEURONS	1.208146874	0.169121949
POSITIVE REGULATION OF TRANSMEMBRANE TRANSPORT	1.207783641	0.169454681
REGULATION OF ACUTE INFLAMMATORY RESPONSE	1.206231117	0.170902063
BRANCHING INVOLVED IN SALIVARY GLAND MORPHOGENES	1.206196822	0.170931177
ACTOMYOSIN STRUCTURE ORGANIZATION	1.205856456	0.171265295
EYE PHOTORECEPTOR CELL DIFFERENTIATION	1.205650143	0.171460082
REGULATION OF NITRIC OXIDE SYNTHASE ACTIVITY	1.205443026	0.171651403
EXCRETION	1.204974834	0.172097124
LIPID HOMEOSTASIS	1.203935152	0.173127899
POSITIVE REGULATION OF CARTILAGE DEVELOPMENT	1.202818212	0.174176004
REGULATION OF CD4 POSITIVE ALPHA BETA T CELL DIFFER	1.202673676	0.174332665
ORGANIC HYDROXY COMPOUND TRANSPORT	1.202462715	0.174536463
NEGATIVE REGULATION OF AMYLOID PRECURSOR PROTEIN	1.202251427	0.174752739
REGULATION OF MYELOID CELL APOPTOTIC PROCESS	1.202132824	0.174853251
POSITIVE REGULATION OF GRANULOCYTE CHEMOTAXIS	1.202061437	0.174917025
REGULATION OF FATTY ACID METABOLIC PROCESS	1.201923987	0.175045265
REGULATION OF ACTOMYOSIN STRUCTURE ORGANIZATION	1.201907889	0.175063288
TISSUE MORPHOGENESIS	1.201758033	0.175198461
STRIATED MUSCLE ADAPTATION	1.201526936	0.17542652
CELLULAR RESPONSE TO CADMIUM ION	1.201358206	0.175596352
PROSTANOID BIOSYNTHETIC PROCESS	1.201085265	0.175847287
I KAPPAB KINASE NF KAPPAB SIGNALING	1.200665849	0.176267361
SEGMENTATION	1.199195186	0.177716823
CLATHRIN DEPENDENT ENDOCYTOSIS	1.19916953	0.177737619
CYTOKINE PRODUCTION INVOLVED IN IMMUNE RESPONSE	1.198810779	0.178099465
RESPONSE TO DOPAMINE	1.198550845	0.178344854
CELLULAR BIOGENIC AMINE METABOLIC PROCESS	1.197935333	0.178992988

ORGANIC ACID TRANSPORT	1.197633027	0.179318094
NEGATIVE REGULATION OF TRANSPORTER ACTIVITY	1.197579938	0.179370084
REGULATION OF STEROID BIOSYNTHETIC PROCESS	1.19732526	0.179620325
EAR MORPHOGENESIS	1.196796137	0.180147844
NEGATIVE REGULATION OF REPRODUCTIVE PROCESS	1.196473814	0.180470178
NUCLEOSIDE BISPHOSPHATE METABOLIC PROCESS	1.195835415	0.181115538
TYPE 2 IMMUNE RESPONSE	1.195772549	0.181180005
CELLULAR CARBOHYDRATE METABOLIC PROCESS	1.195039548	0.18190231
REGULATION OF HORMONE LEVELS	1.194375814	0.182560841
LAMELLIPODIUM ORGANIZATION	1.194193056	0.18276048
RESPONSE TO RETINOIC ACID	1.194130933	0.182818708
CARDIAC MUSCLE CELL DIFFERENTIATION	1.193812156	0.183155599
MODULATION BY SYMBIONT OF ENTRY INTO HOST	1.193567537	0.183403762
REGULATION OF EPITHELIAL TO MESENCHYMAL TRANSITIO	1.192462312	0.184499003
ENDOPLASMIC RETICULUM CALCIUM ION HOMEOSTASIS	1.192460877	0.184501083
SMALL MOLECULE BIOSYNTHETIC PROCESS	1.192457	0.184507321
PLASMA MEMBRANE ORGANIZATION	1.191933926	0.185084056
DEFENSE RESPONSE TO GRAM NEGATIVE BACTERIUM	1.191859291	0.185165853
CELLULAR RESPONSE TO VITAMIN D	1.191169921	0.185886078
CELLULAR KETONE METABOLIC PROCESS	1.191164769	0.185891624
NEGATIVE REGULATION OF LEUKOCYTE PROLIFERATION	1.190909686	0.186160582
REGULATION OF MUSCLE ORGAN DEVELOPMENT	1.190762889	0.186300606
REGULATION OF EPITHELIAL CELL DIFFERENTIATION	1.18884732	0.188256098
REGULATION OF SMAD PROTEIN SIGNAL TRANSDUCTION	1.18847071	0.188638739
FACE DEVELOPMENT	1.188407121	0.188713604
BONE RESORPTION	1.187780121	0.189381147
POSITIVE REGULATION OF ERBB SIGNALING PATHWAY	1.187333879	0.189823403
POSITIVE REGULATION OF WNT SIGNALING PATHWAY	1.187081785	0.190095134
METAL ION EXPORT	1.186861782	0.19030517
BLOOD VESSEL REMODELING	1.186716299	0.19048332
POSITIVE REGULATION OF ANIMAL ORGAN MORPHOGENES	1.185820165	0.191421208
REGULATION OF CELLULAR RESPONSE TO INSULIN STIMUL	1.185170684	0.192054784
REGULATION OF HORMONE METABOLIC PROCESS	1.184694342	0.192564973
REGULATION OF MONOOXYGENASE ACTIVITY	1.184371838	0.192908795
ELECTRON TRANSPORT CHAIN	1.182749322	0.194629295
REGULATION OF RHO PROTEIN SIGNAL TRANSDUCTION	1.1823265	0.195098586
REGULATION OF B CELL MEDIATED IMMUNITY	1.182140805	0.19531902
NEGATIVE REGULATION OF OSSIFICATION	1.181819807	0.195669775
REGULATION OF SODIUM ION TRANSPORT	1.181644328	0.195842379
RESPIRATORY ELECTRON TRANSPORT CHAIN	1.181001718	0.19650715
REGULATION OF ANOIKIS	1.180661887	0.196855825
NEURAL CREST CELL DIFFERENTIATION	1.18051131	0.197011793
SMOOTHENED SIGNALING PATHWAY	1.180194853	0.197338979
NEGATIVE REGULATION OF AXON EXTENSION	1.180156972	0.197375718
CELLULAR RESPONSE TO OSMOTIC STRESS	1.179652485	0.197903236
DOPAMINERGIC NEURON DIFFERENTIATION	1.179257495	0.198320537
NEGATIVE REGULATION OF GROWTH	1.178883343	0.198739224
FOREBRAIN DEVELOPMENT	1.178468887	0.199165537
POSITIVE REGULATION OF PEPTIDYL SERINE PHOSPHORYL	1.178345498	0.199301402
ROOF OF MOUTH DEVELOPMENT	1.178231282	0.19940954
METANEPHROS DEVELOPMENT	1.177781236	0.19989408
NEUROMUSCULAR PROCESS	1.177450629	0.200255926
GLYCEROPHOSPHOLIPID METABOLIC PROCESS	1.17734463	0.200364064

POSITIVE REGULATION OF TRANSPORTER ACTIVITY	1.175671765	0.202143485
PEROXISOMAL TRANSPORT	1.175404895	0.202436705
ACTIVATION OF CYSTEINE TYPE ENDOPEPTIDASE ACTIVITY	1.174341751	0.203583936
POSITIVE REGULATION OF RELEASE OF SEQUESTERED CA	1.174253281	0.203683062
RESPIRATORY SYSTEM PROCESS	1.17408214	0.203897951
MEMBRANE PROTEIN ECTODOMAIN PROTEOLYSIS	1.174000967	0.203980441
PEROXISOMAL MEMBRANE TRANSPORT	1.173579426	0.204433788
NEGATIVE REGULATION OF LYMPHOCYTE DIFFERENTIATIO	1.173545895	0.204464981
ENERGY HOMEOSTASIS	1.173096899	0.204928033
CELLULAR RESPONSE TO AMYLOID BETA	1.173040662	0.204983488
CELLULAR RESPONSE TO ORGANIC CYCLIC COMPOUND	1.172766769	0.205301663
MIDBRAIN DEVELOPMENT	1.171941281	0.206231925
NEGATIVE REGULATION OF BINDING	1.171616294	0.206588225
REGULATION OF OXIDATIVE STRESS INDUCED INTRINSIC A	1.171609496	0.206594464
REGULATION OF NEURONAL SYNAPTIC PLASTICITY	1.17143031	0.206786478
REGULATION OF CALCIUM ION TRANSMEMBRANE TRANSPO	1.170857669	0.20741035
REGULATION OF RESPONSE TO REACTIVE OXYGEN SPECIE	1.170553677	0.207754172
REGULATION OF OSTEOCLAST DIFFERENTIATION	1.169771073	0.208599172
REGULATION OF CHOLESTEROL EFFLUX	1.169382447	0.20902895
INCLUSION BODY ASSEMBLY	1.168844533	0.209588356
GLYCEROLIPID METABOLIC PROCESS	1.168470546	0.209993872
FATTY ACID TRANSPORT	1.168148102	0.210357104
IMMUNOGLOBULIN PRODUCTION INVOLVED IN IMMUNOGLO	1.168108456	0.210397309
NEGATIVE REGULATION OF OXIDATIVE STRESS INDUCED C	1.167864999	0.210667654
POSITIVE REGULATION OF VASCULAR ASSOCIATED SMOOT	1.167842784	0.210691222
POSITIVE REGULATION OF NUCLEOCYTOPLASMIC TRANSP	1.167342696	0.211234684
RHYTHMIC BEHAVIOR	1.16670165	0.211994421
PROSTANOID METABOLIC PROCESS	1.166301702	0.212423506
RESPONSE TO CALCIUM ION	1.166186225	0.212541349
RESPONSE TO ACID CHEMICAL	1.165113084	0.213752353
REGULATION OF TRANS SYNAPTIC SIGNALING	1.165008603	0.213873662
REPRODUCTIVE BEHAVIOR	1.164979453	0.213904162
POSITIVE REGULATION OF T HELPER CELL DIFFERENTIATIC	1.164979389	0.213904162
REGULATION OF SYNAPTIC PLASTICITY	1.164870013	0.21401438
REGULATION OF PROTEIN SERINE THREONINE KINASE ACT	1.164825755	0.214058744
HEART MORPHOGENESIS	1.164512521	0.214397021
NEGATIVE REGULATION OF FAT CELL DIFFERENTIATION	1.164489393	0.214426135
ENDOTHELIAL CELL DEVELOPMENT	1.16404245	0.214917607
CENTRAL NERVOUS SYSTEM NEURON DEVELOPMENT	1.163869643	0.215095757
OLIGOSACCHARIDE METABOLIC PROCESS	1.163264892	0.215823608
GLIAL CELL DIFFERENTIATION	1.163070659	0.216055134
NEGATIVE REGULATION OF NF KAPPAB TRANSCRIPTION FA	1.16278583	0.216379547
REGULATION OF G PROTEIN COUPLED RECEPTOR SIGNALI	1.162349281	0.216885576
CELLULAR RESPONSE TO LIPID	1.162189393	0.217061647
KETONE BIOSYNTHETIC PROCESS	1.162174806	0.217080363
EXOCRINE SYSTEM DEVELOPMENT	1.161676625	0.217657098
POSITIVE REGULATION OF EXOCYTOSIS	1.161470182	0.217887237
POSITIVE REGULATION OF PROTEASOMAL UBIQUITIN DEPE	1.160825302	0.218607463
HINDLIMB MORPHOGENESIS	1.160673329	0.218789772
CELLULAR RESPONSE TO CHEMICAL STRESS	1.160397926	0.219112106
AMINE BIOSYNTHETIC PROCESS	1.160384299	0.219128742
POSITIVE REGULATION OF NEUROGENESIS	1.160376326	0.219134981
HEART VALVE DEVELOPMENT	1.159022902	0.220723775

MOTOR NEURON AXON GUIDANCE	1.158909458	0.220854094
REGULATION OF CELL PROJECTION ORGANIZATION	1.158799907	0.220979562
NEGATIVE REGULATION OF PROTEIN SERINE THREONINE K	1.156478098	0.223652506
NEUROTROPHIN SIGNALING PATHWAY	1.15598338	0.224236866
CELLULAR RESPONSE TO DRUG	1.15551845	0.224792112
POSITIVE REGULATION OF LIPID STORAGE	1.153913014	0.226662341
ORGAN GROWTH	1.153773395	0.226819002
FEMALE SEX DIFFERENTIATION	1.15376729	0.226826627
MEMBRANE LIPID CATABOLIC PROCESS	1.153137769	0.227596069
SENSORY PERCEPTION OF LIGHT STIMULUS	1.152840273	0.227944744
PHOSPHOLIPID DEPHOSPHORYLATION	1.152761499	0.22804803
CELLULAR RESPIRATION	1.15274977	0.228062587
NEGATIVE REGULATION OF TOLL LIKE RECEPTOR SIGNALIN	1.152590726	0.228261533
REGULATION OF SUPRAMOLECULAR FIBER ORGANIZATION	1.152519669	0.228343329
PROTEIN LOCALIZATION TO SYNAPSE	1.152519352	0.228343329
NEGATIVE REGULATION OF PHOSPHORYLATION	1.152415961	0.228466717
POSITIVE REGULATION OF DEVELOPMENTAL GROWTH	1.152400349	0.228481967
ADRENERGIC RECEPTOR SIGNALING PATHWAY	1.152377095	0.228506922
EMBRYONIC DIGESTIVE TRACT DEVELOPMENT	1.152291374	0.228603969
STEROID BIOSYNTHETIC PROCESS	1.152128284	0.228816779
MESENCHYMAL CELL PROLIFERATION	1.151834712	0.22914951
REGULATION OF T HELPER CELL DIFFERENTIATION	1.151731249	0.229259728
ESTABLISHMENT OF ENDOTHELIAL BARRIER	1.151592488	0.229421934
NEPHRON MORPHOGENESIS	1.151361834	0.229690199
PROTEIN AUTOPHOSPHORYLATION	1.149927197	0.231360789
NEGATIVE REGULATION OF CALCIUM MEDIATED SIGNALING	1.149552231	0.231804431
ESTABLISHMENT OF TISSUE POLARITY	1.149531911	0.231829386
PURINE NUCLEOSIDE MONOPHOSPHATE BIOSYNTHETIC PR	1.148964261	0.232537134
TERPENOID METABOLIC PROCESS	1.148858822	0.232684091
REGULATION OF MITOCHONDRIAL MEMBRANE POTENTIAL	1.147912062	0.233846572
CELLULAR RESPONSE TO FATTY ACID	1.147881923	0.233893709
REGULATION OF WNT SIGNALING PATHWAY	1.147766491	0.234025415
REGULATION OF APOPTOTIC SIGNALING PATHWAY	1.147332091	0.234582741
CELLULAR RESPONSE TO RETINOIC ACID	1.146613783	0.235465173
POSITIVE REGULATION OF DNA RECOMBINATION	1.145662352	0.236615176
REGULATION OF NERVOUS SYSTEM PROCESS	1.145303713	0.237060898
CELLULAR COMPONENT MORPHOGENESIS	1.144828356	0.237677145
POSITIVE REGULATION OF SIGNAL TRANSDUCTION BY P53	1.144360578	0.238246948
REGULATION OF CELL MORPHOGENESIS	1.144279335	0.238339142
POSITIVE REGULATION OF NEUTROPHIL MIGRATION	1.143272419	0.239553613
MITOCHONDRIAL ELECTRON TRANSPORT NADH TO UBIQUI	1.142753902	0.240192042
CANONICAL WNT SIGNALING PATHWAY	1.142097637	0.241051598
REGULATION OF CHONDROCYTE DIFFERENTIATION	1.140978828	0.242492049
CELLULAR RESPONSE TO INORGANIC SUBSTANCE	1.140193534	0.243499949
PEPTIDYL TYROSINE DEPHOSPHORYLATION	1.139602191	0.244229186
POSITIVE REGULATION OF MUSCLE CELL DIFFERENTIATION	1.139508584	0.244341482
SENSORY PERCEPTION	1.139433673	0.244429518
STEROID HORMONE BIOSYNTHETIC PROCESS	1.139282169	0.244592418
T CELL RECEPTOR SIGNALING PATHWAY	1.138599679	0.245434645
FC EPSILON RECEPTOR SIGNALING PATHWAY	1.138599653	0.245434645
POSITIVE REGULATION OF ATP METABOLIC PROCESS	1.138492277	0.245561499
POSITIVE REGULATION OF CARDIAC MUSCLE CELL PROLIFE	1.138325621	0.24578332
EMBRYONIC ORGAN MORPHOGENESIS	1.137831678	0.246409271

VERY LONG CHAIN FATTY ACID METABOLIC PROCESS	1.137507757	0.246793299
SYNAPTIC VESICLE EXOCYTOSIS	1.137473093	0.246841822
PROSTATE GLAND MORPHOGENESIS	1.13741159	0.24691738
LUNG ALVEOLUS DEVELOPMENT	1.136732288	0.247733266
REGULATION OF SMALL MOLECULE METABOLIC PROCESS	1.136454939	0.248089566
ENTRY INTO HOST	1.136186858	0.248448639
RESPONSE TO HYPEROXIA	1.135626251	0.249186194
RESPONSE TO INORGANIC SUBSTANCE	1.135386626	0.249489811
REGULATION OF OSTEOBLAST DIFFERENTIATION	1.134580236	0.250504643
REGULATION OF RELEASE OF CYTOCHROME C FROM MITO	1.13456332	0.250525439
HORMONE TRANSPORT	1.134563258	0.250526132
LIPID LOCALIZATION	1.133746298	0.251568691
CARDIAC MUSCLE TISSUE MORPHOGENESIS	1.132410592	0.253321771
HORMONE BIOSYNTHETIC PROCESS	1.131402044	0.254615265
NEURON PROJECTION EXTENSION	1.130592148	0.255661983
RESPONSE TO NERVE GROWTH FACTOR	1.130072963	0.256349629
FATTY ACID DERIVATIVE BIOSYNTHETIC PROCESS	1.129244743	0.257440018
DETECTION OF BIOTIC STIMULUS	1.129198998	0.257490621
REGULATION OF CELLULAR CARBOHYDRATE METABOLIC P	1.128922779	0.257854546
NEGATIVE REGULATION OF RECEPTOR SIGNALING PATHW	1.128480523	0.258431281
REGULATION OF PROTEIN AUTOPHOSPHORYLATION	1.128417688	0.258510305
POSITIVE REGULATION OF PROTEIN SERINE THREONINE KI	1.127915922	0.259166757
REGULATION OF CELLULAR COMPONENT SIZE	1.127697545	0.259448192
NEGATIVE REGULATION OF OSTEOCLAST DIFFERENTIATIO	1.127162836	0.260147622
PROSTATE GLAND DEVELOPMENT	1.127133201	0.260185747
REGULATION OF DNA BINDING	1.125883539	0.261836928
PROTEIN PROCESSING	1.125098753	0.262862851
PHOSPHOLIPID TRANSPORT	1.125006089	0.262991091
INTERLEUKIN 7 MEDIATED SIGNALING PATHWAY	1.124981864	0.263021592
NEGATIVE REGULATION OF PROTEIN BINDING	1.123511728	0.26491123
NEGATIVE REGULATION OF B CELL ACTIVATION	1.12234058	0.266465364
ACTIN POLYMERIZATION OR DEPOLYMERIZATION	1.122219527	0.266643514
REGULATION OF PEPTIDE SECRETION	1.121706836	0.267301352
RESPONSE TO OXIDATIVE STRESS	1.120797523	0.268467993
DIOL BIOSYNTHETIC PROCESS	1.120625364	0.268692586
REGULATION OF HEART RATE	1.119747883	0.269841897
ANTIGEN PROCESSING AND PRESENTATION OF PEPTIDE O	1.119194054	0.270593316
CARBOXYLIC ACID TRANSPORT	1.118995812	0.270842172
REGULATION OF ACTIN FILAMENT LENGTH	1.11893966	0.270920502
SEMAPHORIN PLEXIN SIGNALING PATHWAY	1.117858529	0.27239284
NEGATIVE REGULATION OF CATALYTIC ACTIVITY	1.117748035	0.272535637
MULTICELLULAR ORGANISM GROWTH	1.117676707	0.272629911
LENS DEVELOPMENT IN CAMERA TYPE EYE	1.117319615	0.273092269
FOLIC ACID CONTAINING COMPOUND METABOLIC PROCESS	1.116210933	0.274568073
PEPTIDE HORMONE SECRETION	1.115791712	0.275163524
PEPTIDYL THREONINE DEPHOSPHORYLATION	1.115363989	0.275762441
TRABECULA MORPHOGENESIS	1.114982639	0.276294118
CARTILAGE DEVELOPMENT INVOLVED IN ENDOCHONDRAL	1.114831462	0.276482666
MYELOID LEUKOCYTE CYTOKINE PRODUCTION	1.11460862	0.276816091
CELLULAR MODIFIED AMINO ACID BIOSYNTHETIC PROCESS	1.113037128	0.278917153
PHOSPHATIDYLINOSITOL BIOSYNTHETIC PROCESS	1.112859593	0.279127883
ANTIGEN PROCESSING AND PRESENTATION OF PEPTIDE A	1.11253132	0.279584695
REGULATION OF EXTRINSIC APOPTOTIC SIGNALING PATHW	1.112458591	0.27969006

NEGATIVE REGULATION OF NEURON DEATH	1.112227537	0.280006849
PH REDUCTION	1.110811683	0.281922828
SARCOPLASMIC RETICULUM CALCIUM ION TRANSPORT	1.110739963	0.282023341
REGULATION OF PROTEIN EXPORT FROM NUCLEUS	1.11043275	0.282458665
REGULATION OF NEUROGENESIS	1.110019114	0.283019456
LUNG EPITHELIUM DEVELOPMENT	1.10994845	0.283120662
POSITIVE REGULATION OF CANONICAL WNT SIGNALING PA	1.10861069	0.284954152
PLACENTA BLOOD VESSEL DEVELOPMENT	1.107835965	0.286109702
OXIDATIVE PHOSPHORYLATION	1.106791664	0.287568175
REGULATION OF CELL SIZE	1.106145146	0.288490119
BILE ACID METABOLIC PROCESS	1.106076494	0.288586473
RESPONSE TO INTERLEUKIN 1	1.105067072	0.28999573
POSITIVE REGULATION OF CYTOKINE PRODUCTION INVOLV	1.104704618	0.290492055
CELL SURFACE RECEPTOR SIGNALING PATHWAY INVOLVED	1.104113159	0.291352305
CARBOHYDRATE DERIVATIVE BIOSYNTHETIC PROCESS	1.103531872	0.292164724
RENAL TUBULE DEVELOPMENT	1.102880739	0.293066566
REGULATION OF MITOCHONDRIAL GENE EXPRESSION	1.102705027	0.293309876
NEGATIVE REGULATION OF TRANSMEMBRANE TRANSPORT	1.102702367	0.293311955
REGULATION OF MYOTUBE DIFFERENTIATION	1.102318683	0.293849178
REGULATION OF FATTY ACID BIOSYNTHETIC PROCESS	1.101932254	0.294403731
NEUROBLAST PROLIFERATION	1.101319005	0.295259129
NEGATIVE REGULATION OF CELL GROWTH	1.101087518	0.295592553
RESPONSE TO STEROL DEPLETION	1.100882279	0.295893398
EMBRYONIC HEART TUBE MORPHOGENESIS	1.100536695	0.296398041
POSITIVE REGULATION OF PROTEIN BINDING	1.099913744	0.297286019
CARDIAC MUSCLE TISSUE DEVELOPMENT	1.099790116	0.297466249
FIBROBLAST PROLIFERATION	1.099763838	0.297501601
REGULATION OF GROWTH	1.099678488	0.29762291
NEGATIVE REGULATION OF SMALL GTPASE MEDIATED SIGN	1.098938848	0.298696662
VESICLE FUSION TO PLASMA MEMBRANE	1.098891177	0.29875697
CELL CELL SIGNALING BY WNT	1.098754562	0.298942745
NEGATIVE REGULATION OF REACTIVE OXYGEN SPECIES M	1.098685342	0.299051576
EMBRYONIC HEART TUBE DEVELOPMENT	1.098643808	0.299120202
REGULATION OF CARBOHYDRATE METABOLIC PROCESS	1.098445053	0.299403024
NEGATIVE REGULATION OF INTRACELLULAR SIGNAL TRANS	1.098204145	0.29976487
PURINE CONTAINING COMPOUND METABOLIC PROCESS	1.097792616	0.300352002
POSITIVE REGULATION OF RESPONSE TO CYTOKINE STIMU	1.097412495	0.3008816
INSULIN SECRETION	1.097017529	0.301456949
POSITIVE REGULATION OF CARBOHYDRATE METABOLIC PR	1.096934965	0.301581723
DEVELOPMENTAL INDUCTION	1.09666799	0.301949114
POSITIVE REGULATION OF ACTIN FILAMENT POLYMERIZATI	1.096586144	0.302058638
TELENCEPHALON DEVELOPMENT	1.095783075	0.303165664
PATTERN SPECIFICATION PROCESS	1.095282568	0.303888662
VISUAL BEHAVIOR	1.09508572	0.304198519
POSITIVE REGULATION OF T CELL MEDIATED IMMUNITY	1.095002737	0.304312202
SMOOTH MUSCLE CELL MIGRATION	1.094866835	0.304493818
VESICLE CARGO LOADING	1.094599205	0.304904187
CILIUM OR FLAGELLUM DEPENDENT CELL MOTILITY	1.093588335	0.306391082
RESPONSE TO MECHANICAL STIMULUS	1.093250311	0.306880474
TOR SIGNALING	1.093064737	0.307150819
ANATOMICAL STRUCTURE MATURATION	1.092845648	0.307454437
REGULATION OF TYPE 2 IMMUNE RESPONSE	1.092842878	0.307458596
RESPONSE TO OSMOTIC STRESS	1.092306893	0.308196151

POLYSACCHARIDE CATABOLIC PROCESS	1.092072423	0.308540667
ENDOCRINE SYSTEM DEVELOPMENT	1.09178743	0.308935786
POSITIVE REGULATION OF TOLL LIKE RECEPTOR SIGNALIN(	1.091781844	0.308946877
REGULATION OF NUCLEOCYTOPLASMIC TRANSPORT	1.091059282	0.31000122
REGULATION OF INTRINSIC APOPTOTIC SIGNALING PATHW	1.090472296	0.310848993
RESPONSE TO PEPTIDE	1.09036555	0.311016745
REGULATION OF NEURAL PRECURSOR CELL PROLIFERATIC	1.08961258	0.312116146
RELEASE OF CYTOCHROME C FROM MITOCHONDRIA	1.089350423	0.312498094
AGING	1.089232262	0.312683869
ANTERIOR POSTERIOR AXIS SPECIFICATION	1.087357121	0.315454553
REGULATION OF NEURON PROJECTION REGENERATION	1.086838049	0.316223995
PROTEIN O LINKED GLYCOSYLATION	1.08648109	0.31676815
EPITHELIAL TUBE MORPHOGENESIS	1.086412483	0.316869356
POSITIVE REGULATION OF EPITHELIAL CELL DIFFERENTIAT	1.085970392	0.317574331
REGULATION OF INTRINSIC APOPTOTIC SIGNALING PATHW	1.085598908	0.318092838
FC RECEPTOR SIGNALING PATHWAY	1.085431413	0.31834308
NEGATIVE REGULATION OF NEURON APOPTOTIC PROCESS	1.08536243	0.318429728
ACTION POTENTIAL	1.084924338	0.319070237
CELL PART MORPHOGENESIS	1.08461318	0.319530516
MYOTUBE CELL DEVELOPMENT	1.083782324	0.320733202
ORGANOPHOSPHATE BIOSYNTHETIC PROCESS	1.083631719	0.32098691
NOTCH SIGNALING PATHWAY	1.083132261	0.321766056
GLIAL CELL DEVELOPMENT	1.081334172	0.324448705
LONG CHAIN FATTY ACYL COA METABOLIC PROCESS	1.080493927	0.325799041
AORTA MORPHOGENESIS	1.079900603	0.326688405
REGULATION OF VASCULAR PERMEABILITY	1.079576523	0.32719998
REGULATION OF ACTIN NUCLEATION	1.078858111	0.328267494
DETECTION OF ABIOTIC STIMULUS	1.078581031	0.32869242
MATING	1.078304997	0.329099323
MORPHOGENESIS OF AN EPITHELIUM	1.077927666	0.329666353
PEPTIDE SECRETION	1.077787403	0.329870845
ACTIVATION OF GTPASE ACTIVITY	1.076633775	0.331635016
PORE COMPLEX ASSEMBLY	1.075804576	0.332918112
POSITIVE REGULATION OF TRANSMEMBRANE RECEPTOR P	1.075222224	0.333801237
POSITIVE REGULATION OF MONOOXYGENASE ACTIVITY	1.075101664	0.333986319
INORGANIC ANION TRANSPORT	1.075036312	0.33409723
REGULATION OF MEMBRANE POTENTIAL	1.074864423	0.334369654
FOREBRAIN CELL MIGRATION	1.074801179	0.334463928
POSITIVE REGULATION OF TOR SIGNALING	1.074758035	0.334531167
SULFUR AMINO ACID METABOLIC PROCESS	1.074746855	0.33455127
CELLULAR RESPONSE TO TOXIC SUBSTANCE	1.074647928	0.334713476
RESPONSE TO REACTIVE OXYGEN SPECIES	1.074473323	0.334967184
NEGATIVE REGULATION OF SIGNALING RECEPTOR ACTIVIT	1.07404939	0.335621557
REGULATION OF MUSCLE SYSTEM PROCESS	1.07398403	0.33571999
GLUTAMATE RECEPTOR SIGNALING PATHWAY	1.073537491	0.336419419
REGULATION OF RAS PROTEIN SIGNAL TRANSDUCTION	1.072873842	0.337391966
NEGATIVE REGULATION OF ENDOPLASMIC RETICULUM STR	1.072594725	0.337827983
REGULATION OF TRIGLYCERIDE METABOLIC PROCESS	1.072311222	0.338270932
REGULATION OF DEVELOPMENTAL GROWTH	1.072008282	0.338736063
NEGATIVE REGULATION OF SIGNAL TRANSDUCTION BY P53	1.071528969	0.339446584
GPI ANCHOR METABOLIC PROCESS	1.071357884	0.33972802
POSITIVE REGULATION OF CALCIUM ION TRANSMEMBRANE	1.070891893	0.340491916
ERYTHROCYTE HOMEOSTASIS	1.070749305	0.340713044

REGULATION OF PROTEIN LOCALIZATION TO PLASMA MEMI	1.070445336	0.341176095
ENERGY DERIVATION BY OXIDATION OF ORGANIC COMPOU	1.069823428	0.342098039
DENDRITIC SPINE MORPHOGENESIS	1.069483083	0.342617239
CARDIAC CONDUCTION	1.069312199	0.342879959
POSITIVE REGULATION OF EXTRACELLULAR MATRIX ORGAI	1.068936012	0.343454614
REGULATION OF EXTENT OF CELL GROWTH	1.067904703	0.345028157
NEURAL PRECURSOR CELL PROLIFERATION	1.067779799	0.345220171
HORMONE METABOLIC PROCESS	1.06659598	0.347110503
NEGATIVE REGULATION OF SYNAPTIC TRANSMISSION	1.066456228	0.347328165
PROTEIN KINASE A SIGNALING	1.066160938	0.347792603
LIPOPROTEIN METABOLIC PROCESS	1.065682073	0.348532931
ANTIMICROBIAL HUMORAL IMMUNE RESPONSE MEDIATED E	1.06522935	0.349254542
SUBSTRATE ADHESION DEPENDENT CELL SPREADING	1.064329552	0.350604185
FATTY ACID BETA OXIDATION	1.063738115	0.351527516
POSITIVE REGULATION OF PHOSPHOPROTEIN PHOSPHATA	1.063722061	0.35155247
RECEPTOR METABOLIC PROCESS	1.063562993	0.351806871
REGULATION OF BONE REMODELING	1.062794728	0.353032433
FORELIMB MORPHOGENESIS	1.062507634	0.353466371
LIPID TRANSLOCATION	1.061904492	0.354438917
RESPONSE TO TRANSFORMING GROWTH FACTOR BETA	1.061791598	0.354614988
SPINAL CORD DEVELOPMENT	1.061782553	0.354630238
EMBRYONIC MORPHOGENESIS	1.061562384	0.354956731
RESPONSE TO VIRUS	1.061453191	0.355145279
ANIMAL ORGAN MATURATION	1.060721418	0.356270328
LIPOPROTEIN BIOSYNTHETIC PROCESS	1.059494036	0.358212649
POSITIVE REGULATION OF UBIQUITIN DEPENDENT PROTEIN	1.059416655	0.358342969
POSITIVE REGULATION OF GROWTH	1.059250781	0.358618859
PROGRAMMED NECROTIC CELL DEATH	1.058160247	0.360340052
POSITIVE REGULATION OF BLOOD CIRCULATION	1.057695651	0.361088005
REGULATION OF HEART RATE BY CARDIAC CONDUCTION	1.057148238	0.361951027
POSITIVE REGULATION OF MYELOID CELL DIFFERENTIATIOI	1.055701209	0.364272524
PROTEIN TETRAMERIZATION	1.05569649	0.364282229
AMINO ACID TRANSPORT	1.055479491	0.36463437
AUTONOMIC NERVOUS SYSTEM DEVELOPMENT	1.055467092	0.364648927
MYELOID CELL DEVELOPMENT	1.055430683	0.364717552
REGULATION OF PEPTIDYL SERINE PHOSPHORYLATION	1.055161836	0.365155649
RESPONSE TO ESTRADIOL	1.054009962	0.367012014
POSITIVE REGULATION OF NITRIC OXIDE METABOLIC PROC	1.052127773	0.37010226
COCHLEA DEVELOPMENT	1.051874637	0.370525799
REGULATION OF PEPTIDE HORMONE SECRETION	1.051461404	0.371199581
ACTIN FILAMENT BASED TRANSPORT	1.050647659	0.372480598
VITAMIN TRANSPORT	1.050388875	0.372913842
INTERLEUKIN 1 MEDIATED SIGNALING PATHWAY	1.050202179	0.373208448
BILE ACID BIOSYNTHETIC PROCESS	1.050030487	0.373497509
SEMI LUNAR VALVE DEVELOPMENT	1.049852754	0.373791422
REGULATION OF EMBRYONIC DEVELOPMENT	1.049837899	0.373819149
CELLULAR RESPONSE TO HORMONE STIMULUS	1.048433976	0.37615243
CARDIOCYTE DIFFERENTIATION	1.047547805	0.37757763
PHOSPHOLIPID BIOSYNTHETIC PROCESS	1.046424436	0.379409041
PHOTORECEPTOR CELL DEVELOPMENT	1.046301487	0.379596896
REGULATION OF MULTICELLULAR ORGANISM GROWTH	1.046055799	0.379996867
HEAD DEVELOPMENT	1.045606161	0.380699069
MUSCLE CELL APOPTOTIC PROCESS	1.044894041	0.381849073

DICARBOXYLIC ACID METABOLIC PROCESS	1.044046628	0.383216045
ORGANOPHOSPHATE ESTER TRANSPORT	1.043752649	0.383711677
NEGATIVE REGULATION OF KINASE ACTIVITY	1.042755962	0.385310175
NEURON RECOGNITION	1.042075675	0.386432451
NON MOTILE CILIUM ASSEMBLY	1.041376367	0.387566512
NEGATIVE REGULATION OF JNK CASCADE	1.040812251	0.388494001
RESPONSE TO ALCOHOL	1.040667993	0.388724834
ENDOCARDIAL CUSHION FORMATION	1.039184817	0.391189821
POSITIVE REGULATION OF NOTCH SIGNALING PATHWAY	1.038651737	0.392093742
POSITIVE REGULATION OF EXTRINSIC APOPTOTIC SIGNALI	1.038555155	0.392237925
VACUOLE ORGANIZATION	1.038546513	0.392252482
CELL MATURATION	1.038507135	0.392321801
AMYLOID PRECURSOR PROTEIN METABOLIC PROCESS	1.03776706	0.39360975
REGULATION OF PROTEIN IMPORT	1.03712456	0.394702912
HINDBRAIN DEVELOPMENT	1.036601521	0.395586731
DIGESTIVE SYSTEM PROCESS	1.036487374	0.395776665
CELLULAR MODIFIED AMINO ACID METABOLIC PROCESS	1.036364352	0.395979077
DEVELOPMENTAL MATURATION	1.035575386	0.397337038
REGULATION OF OXIDATIVE STRESS INDUCED CELL DEATH	1.035493421	0.397485381
REGULATION OF PLATELET DERIVED GROWTH FACTOR RE	1.034950343	0.398358801
REGULATION OF PROGRAMMED NECROTIC CELL DEATH	1.034919815	0.398408018
NEGATIVE REGULATION OF T CELL PROLIFERATION	1.034874582	0.398487735
ANION HOMEOSTASIS	1.034252062	0.39951435
MALE SEX DIFFERENTIATION	1.033914905	0.400063358
REGULATION OF AXONOGENESIS	1.033440966	0.400903505
POSITIVE REGULATION OF COAGULATION	1.033016856	0.401634821
HEMATOPOIETIC PROGENITOR CELL DIFFERENTIATION	1.032849094	0.401937053
NEURON PROJECTION ORGANIZATION	1.032831235	0.40196478
MECHANORECEPTOR DIFFERENTIATION	1.032253103	0.402975453
RECEPTOR LOCALIZATION TO SYNAPSE	1.032174576	0.40311825
MOVEMENT IN HOST ENVIRONMENT	1.031950174	0.40350713
ENDOCARDIAL CUSHION DEVELOPMENT	1.03181505	0.403732417
REGULATION OF TOR SIGNALING	1.031135711	0.404891432
POSITIVE REGULATION OF TRANSCRIPTION FROM RNA POL	1.031040361	0.405070969
REGULATION OF SUBSTRATE ADHESION DEPENDENT CELL	1.030950198	0.405217232
REGULATION OF CYTOSKELETON ORGANIZATION	1.03088326	0.405337154
REGULATION OF RECEPTOR INTERNALIZATION	1.03036282	0.406206416
GLYCOSYLATION	1.030135043	0.406625796
AEROBIC RESPIRATION	1.028939393	0.40868388
CEREBRAL CORTEX CELL MIGRATION	1.028175915	0.410009261
NEGATIVE REGULATION OF EXTRINSIC APOPTOTIC SIGNAL	1.028067053	0.410203354
METANEPHRIC NEPHRON DEVELOPMENT	1.02798563	0.410348231
BIOLOGICAL PROCESS INVOLVED IN INTERACTION WITH HO	1.026605779	0.41270716
REGULATION OF MEMBRANE REPOLARIZATION	1.026373932	0.413107131
CARDIAC SEPTUM MORPHOGENESIS	1.025668517	0.414313283
REGULATION OF T CELL RECEPTOR SIGNALING PATHWAY	1.02520248	0.415117385
CELL JUNCTION ORGANIZATION	1.025059661	0.415365547
EYE PHOTORECEPTOR CELL DEVELOPMENT	1.024483907	0.416372754
AXIS SPECIFICATION	1.024376135	0.416552983
PEROXISOME ORGANIZATION	1.024311837	0.416646564
ICOSANOID TRANSPORT	1.024161992	0.416925227
CELLULAR RESPONSE TO PEPTIDE	1.023162925	0.418600669
NIK NF KAPPAB SIGNALING	1.02311556	0.418685932

NEGATIVE REGULATION OF HYDROLASE ACTIVITY	1.023081617	0.418753171
HEART DEVELOPMENT	1.022599705	0.419573216
RESPONSE TO TEMPERATURE STIMULUS	1.022587022	0.419594705
CELL FATE SPECIFICATION	1.021478092	0.421530094
POSITIVE REGULATION OF PROTEOLYSIS INVOLVED IN CEL	1.021369569	0.421702699
ANTIGEN PROCESSING AND PRESENTATION OF EXOGENOU	1.021337943	0.421751222
EPITHELIAL CELL DIFFERENTIATION	1.021329037	0.421767859
GASTRULATION	1.020584233	0.423064126
NEGATIVE REGULATION OF NUCLEOCYTOPLASMIC TRANSP	1.020577469	0.423073137
MUSCLE CELL MIGRATION	1.020176668	0.423774646
CELLULAR RESPONSE TO CALCIUM ION	1.019612398	0.424738182
REGULATION OF DNA RECOMBINATION	1.018971754	0.425847287
REGULATION OF GLIAL CELL DIFFERENTIATION	1.018477863	0.426702685
REGULATION OF PLASMA LIPOPROTEIN PARTICLE LEVELS	1.018283231	0.42704928
REGULATION OF CALCIUM ION IMPORT	1.017901141	0.42773138
CENTRAL NERVOUS SYSTEM NEURON AXONOGENESIS	1.017898966	0.42773346
SCHWANN CELL DIFFERENTIATION	1.017075467	0.429148262
CORTICAL CYTOSKELETON ORGANIZATION	1.016853872	0.429560711
RESPONSE TO ISCHEMIA	1.016731726	0.429770748
REGULATION OF STEROL TRANSPORT	1.016716521	0.429799169
MORPHOGENESIS OF A POLARIZED EPITHELIUM	1.016655548	0.429879579
REGULATION OF KERATINOCYTE PROLIFERATION	1.016440967	0.430242118
CELLULAR RESPONSE TO STEROL	1.015557464	0.431790706
RELAXATION OF MUSCLE	1.015513196	0.431853093
REGULATION OF AMYLOID PRECURSOR PROTEIN CATABOL	1.015181399	0.432444385
DENDRITIC SPINE DEVELOPMENT	1.014539647	0.433530615
MESONEPHRIC TUBULE MORPHOGENESIS	1.014429768	0.433733027
ATRIOVENTRICULAR VALVE DEVELOPMENT	1.014219124	0.434141317
DEVELOPMENT OF PRIMARY FEMALE SEXUAL CHARACTERI	1.013912948	0.434659823
MYD88 DEPENDENT TOLL LIKE RECEPTOR SIGNALING PATH	1.013029542	0.436222969
EXTRINSIC APOPTOTIC SIGNALING PATHWAY	1.01292302	0.436406665
FIBROBLAST GROWTH FACTOR RECEPTOR SIGNALING PAT	1.012756831	0.436699884
POSTSYNAPSE ASSEMBLY	1.012393637	0.437293949
REGULATION OF CELL MORPHOGENESIS INVOLVED IN DIFF	1.010996888	0.439830335
REGULATION OF DEFENSE RESPONSE TO VIRUS	1.009577035	0.442345231
NEGATIVE REGULATION OF PROTEIN TYROSINE KINASE AC	1.008680133	0.443945116
NEGATIVE REGULATION OF STRESS ACTIVATED PROTEIN K	1.006993177	0.447014565
REGULATION OF PROTEIN CONTAINING COMPLEX ASSEMBL	1.006985772	0.447020804
RESPONSE TO KETONE	1.006584427	0.447765984
REGULATION OF NERVOUS SYSTEM DEVELOPMENT	1.006237967	0.448356583
STEM CELL DIFFERENTIATION	1.005566016	0.449553724
NEUROTRANSMITTER UPTAKE	1.005051251	0.450469429
POSITIVE REGULATION OF MYELOID LEUKOCYTE DIFFEREN	1.004551414	0.451398305
RESPONSE TO INTERLEUKIN 7	1.004529994	0.45143643
DETOXIFICATION	1.004087248	0.452222509
FEEDING BEHAVIOR	1.0040756	0.452248157
PHOSPHATIDYLETHANOLAMINE METABOLIC PROCESS	1.003854074	0.452640503
STEROL METABOLIC PROCESS	1.003741656	0.452838755
CELLULAR RESPONSE TO ALCOHOL	1.003658226	0.452973235
PROTEASOMAL UBIQUITIN INDEPENDENT PROTEIN CATABO	1.003376618	0.453509764
NEGATIVE REGULATION OF PHOSPHORUS METABOLIC PRO	1.002691178	0.454691655
POSITIVE REGULATION OF SMOOTHENED SIGNALING PATH	1.002622555	0.454817122
RESPONSE TO INCREASED OXYGEN LEVELS	1.002058041	0.455828488

NEGATIVE REGULATION OF ALPHA BETA T CELL DIFFERENT	1.001890781	0.45612656
ORGANELLE MEMBRANE FUSION	1.001381265	0.456987503
POSITIVE REGULATION OF ISOTYPE SWITCHING	1.000767768	0.458109086
HEART GROWTH	1.000270429	0.458993598
REGULATION OF PATTERN RECOGNITION RECEPTOR SIGNA	0.999956688	0.45955023
RHO PROTEIN SIGNAL TRANSDUCTION	0.99905938	0.461197252
RAC PROTEIN SIGNAL TRANSDUCTION	0.997500669	0.463974174
MORPHOGENESIS OF A BRANCHING STRUCTURE	0.997413533	0.46414262
PHOSPHATIDYLSERINE METABOLIC PROCESS	0.997166654	0.464593887
POST TRANSLATIONAL PROTEIN MODIFICATION	0.996987065	0.464914835
NEGATIVE REGULATION OF ACTIN FILAMENT BUNDLE ASSE	0.996439515	0.465898472
ENDOCARDIAL CUSHION MORPHOGENESIS	0.995569076	0.467495584
AMINE TRANSPORT	0.995278613	0.468021716
EMBRYONIC ORGAN DEVELOPMENT	0.994499225	0.469411564
CELL GROWTH	0.99387125	0.47051651
L AMINO ACID TRANSPORT	0.993248512	0.471660275
CARBOHYDRATE METABOLIC PROCESS	0.99199351	0.47395751
PURINE CONTAINING COMPOUND BIOSYNTHETIC PROCESS	0.991715846	0.474473244
PROTEIN LIPID COMPLEX SUBUNIT ORGANIZATION	0.991448256	0.47495016
INSULIN RECEPTOR SIGNALING PATHWAY	0.991387569	0.475047899
EPITHELIAL CELL DEVELOPMENT	0.990438034	0.476753842
NEGATIVE REGULATION OF CELLULAR PROTEIN LOCALIZAT	0.990397037	0.476838412
ISOPRENOID METABOLIC PROCESS	0.990157567	0.477305622
CIRCADIAN REGULATION OF GENE EXPRESSION	0.989438082	0.478644174
T CELL CYTOKINE PRODUCTION	0.989039839	0.479363706
AORTA DEVELOPMENT	0.988928635	0.479579289
REGULATION OF NEUROBLAST PROLIFERATION	0.988577175	0.480196922
REGULATION OF CELLULAR AMINE METABOLIC PROCESS	0.986832843	0.483445908
CARDIAC CELL DEVELOPMENT	0.986561925	0.483946391
ORGANIC ACID TRANSMEMBRANE TRANSPORT	0.986455631	0.484132167
POSITIVE REGULATION OF CELL PROJECTION ORGANIZATI(	0.986392464	0.484238918
ENDOCRINE HORMONE SECRETION	0.985979658	0.485015985
HOMOLOGOUS RECOMBINATION	0.984695319	0.487325697
POSITIVE REGULATION OF SUBSTRATE ADHESION DEPEND	0.983552816	0.489457259
BASEMENT MEMBRANE ORGANIZATION	0.983006652	0.490439511
MESONEPHROS DEVELOPMENT	0.982768395	0.490884539
DEVELOPMENTAL CELL GROWTH	0.981934161	0.492395002
BRANCHING MORPHOGENESIS OF AN EPITHELIAL TUBE	0.981884044	0.492476799
SPECIFICATION OF SYMMETRY	0.98157928	0.493068784
REGULATION OF MULTI ORGANISM PROCESS	0.981336647	0.493533915
EXPORT ACROSS PLASMA MEMBRANE	0.981325791	0.493562336
REGULATION OF CARDIOCYTE DIFFERENTIATION	0.981171783	0.493834067
POSITIVE REGULATION OF ERYTHROCYTE DIFFERENTIATIC	0.981098937	0.49397825
PEPTIDYL ASPARAGINE MODIFICATION	0.979266219	0.497426182
RESPONSE TO INTERLEUKIN 12	0.97860987	0.498662142
FATTY ACID CATABOLIC PROCESS	0.978518674	0.49884653
RESPIRATORY GASEOUS EXCHANGE BY RESPIRATORY SYS	0.977995926	0.499842646
REGULATION OF EXTRINSIC APOPTOTIC SIGNALING PATHW	0.977849836	0.500142797
FATTY ACID TRANSMEMBRANE TRANSPORT	0.977633877	0.500555246
HISTONE H4 K5 ACETYLATION	0.977614961	0.500591985
REGULATION OF ISOTYPE SWITCHING	0.976491605	0.502664626
REGULATION OF HORMONE SECRETION	0.974891317	0.505639801
BONE GROWTH	0.974680205	0.506023136

SECRETORY GRANULE ORGANIZATION	0.974166265	0.506985285
MYD88 INDEPENDENT TOLL LIKE RECEPTOR SIGNALING PA	0.973939924	0.507412291
CELLULAR LIPID CATABOLIC PROCESS	0.97388671	0.507519042
NEGATIVE REGULATION OF RECEPTOR SIGNALING PATHW	0.97363455	0.508007742
CARDIAC CHAMBER MORPHOGENESIS	0.97356741	0.508145687
POSITIVE REGULATION OF NERVOUS SYSTEM DEVELOPME	0.973410301	0.508430588
NEGATIVE REGULATION OF DOUBLE STRAND BREAK REPAI	0.972978326	0.50920211
REGULATION OF HAIR CYCLE	0.972813232	0.509510579
PROTEIN LOCALIZATION TO CELL JUNCTION	0.971785943	0.511459139
NEGATIVE REGULATION OF NIK NF KAPPAB SIGNALING	0.971737151	0.511527765
DEFENSE RESPONSE TO VIRUS	0.970679653	0.513455529
POSITIVE REGULATION OF CELLULAR PROTEIN CATABOLIC	0.970351729	0.51411406
FIBROBLAST MIGRATION	0.970134869	0.514511259
VENTRICULAR CARDIAC MUSCLE CELL ACTION POTENTIAL	0.969921714	0.51490915
ANTIGEN PROCESSING AND PRESENTATION OF PEPTIDE AI	0.9696965	0.515286939
DORSAL VENTRAL NEURAL TUBE PATTERNING	0.969558711	0.515569761
NUCLEOSIDE PHOSPHATE CATABOLIC PROCESS	0.969517492	0.515650171
REGULATION OF MEMBRANE LIPID DISTRIBUTION	0.969483947	0.515722957
NEURON DEATH	0.968999319	0.516660844
KILLING OF CELLS OF OTHER ORGANISM	0.968555247	0.517480196
REGULATION OF RECEPTOR BINDING	0.967914564	0.518672484
POSITIVE REGULATION OF PROTEIN DEPHOSPHORYLATION	0.96784679	0.518804883
CELL REDOX HOMEOSTASIS	0.96774103	0.51901492
MAMMARY GLAND EPITHELIUM DEVELOPMENT	0.966649701	0.521009231
THIOESTER METABOLIC PROCESS	0.966172309	0.521846605
REGULATION OF PROTEIN MATURATION	0.966065016	0.522048324
GLYCEROLIPID BIOSYNTHETIC PROCESS	0.965563029	0.522970961
POSITIVE REGULATION OF PROTEIN CATABOLIC PROCESS	0.965052341	0.523879041
CELLULAR RESPONSE TO MECHANICAL STIMULUS	0.964189459	0.525509426
REGULATION OF PROTEIN LOCALIZATION TO CELL PERIPHE	0.963738723	0.526371756
PROTEIN HOMOTETRAMERIZATION	0.963406164	0.526977604
POSITIVE REGULATION OF CELLULAR COMPONENT BIOGEN	0.963098628	0.527570283
REGULATION OF CALCIUM ION TRANSMEMBRANE TRANSPC	0.962951793	0.527852411
POSTSYNAPSE ORGANIZATION	0.962551231	0.528585807
NEGATIVE REGULATION OF EXTRINSIC APOPTOTIC SIGNAL	0.96241739	0.528841595
NEGATIVE REGULATION OF INTERLEUKIN 1 PRODUCTION	0.962171804	0.529283157
RIBOSE PHOSPHATE BIOSYNTHETIC PROCESS	0.96179376	0.53000269
POSITIVE REGULATION OF CELL MORPHOGENESIS INVOLVI	0.961724825	0.530139941
GLYCOPROTEIN METABOLIC PROCESS	0.961023588	0.531413333
CELLULAR MONOVALENT INORGANIC CATION HOMEOSTASI	0.960837885	0.531752303
CELLULAR RESPONSE TO ALKALOID	0.960126443	0.533122742
POSITIVE REGULATION OF NEURAL PRECURSOR CELL PRO	0.959130657	0.534978414
VACUOLAR ACIDIFICATION	0.958428508	0.536335682
IMPORT ACROSS PLASMA MEMBRANE	0.958005143	0.53715642
NEGATIVE REGULATION OF RHO PROTEIN SIGNAL TRANSDI	0.95688704	0.539299766
REGULATION OF MORPHOGENESIS OF A BRANCHING STRU	0.956845635	0.539374631
NEGATIVE REGULATION OF TRANSFERASE ACTIVITY	0.955838838	0.541354384
NEGATIVE REGULATION OF INTRINSIC APOPTOTIC SIGNALI	0.955599991	0.541802879
REGULATION OF SYSTEMIC ARTERIAL BLOOD PRESSURE M	0.955230258	0.542523104
MULTI ORGANISM LOCALIZATION	0.955091234	0.542805233
MEMBRANE REPOLARIZATION	0.955067516	0.542849597
MITOCHONDRIAL CYTOCHROME C OXIDASE ASSEMBLY	0.954562368	0.543831155
REGULATION OF SPROUTING ANGIOGENESIS	0.954456454	0.544017624

VENTRICULAR SEPTUM MORPHOGENESIS	0.953435992	0.54601332
SECONDARY PALATE DEVELOPMENT	0.952977893	0.546880502
REGULATION OF ERYTHROCYTE DIFFERENTIATION	0.952621088	0.547564682
ACTIVIN RECEPTOR SIGNALING PATHWAY	0.952109658	0.548528217
PHOSPHOLIPID CATABOLIC PROCESS	0.951307122	0.550029669
RESPONSE TO DSRNA	0.950912259	0.550776235
PEPTIDYL GLUTAMIC ACID MODIFICATION	0.949313798	0.553971845
STRIATED MUSCLE CELL APOPTOTIC PROCESS	0.949014153	0.554556205
GENERATION OF PRECURSOR METABOLITES AND ENERGY	0.948947377	0.554675434
APPENDAGE MORPHOGENESIS	0.948638306	0.555229294
NEGATIVE REGULATION OF CATION TRANSMEMBRANE TRA	0.948526966	0.555442796
CELL CELL ADHESION VIA PLASMA MEMBRANE ADHESION M	0.948007118	0.55646248
REGULATION OF ARP2 3 COMPLEX MEDIATED ACTIN NUCLE	0.947683477	0.557088432
ORGANELLE FUSION	0.94696806	0.558416586
NEURAL TUBE PATTERNING	0.946915312	0.558505314
RUFFLE ORGANIZATION	0.946208842	0.559831388
NEGATIVE REGULATION OF G PROTEIN COUPLED RECEPTO	0.945694402	0.560836515
EMBRYONIC APPENDAGE MORPHOGENESIS	0.945255737	0.561646855
NECROPTOTIC PROCESS	0.944984651	0.562141794
NCRNA EXPORT FROM NUCLEUS	0.944738684	0.562634652
AMYLOID PRECURSOR PROTEIN CATABOLIC PROCESS	0.944504589	0.563046408
RHYTHMIC PROCESS	0.944391932	0.563256445
PHENOL CONTAINING COMPOUND METABOLIC PROCESS	0.944300457	0.563425583
CELL PROJECTION ASSEMBLY	0.943912745	0.564195025
CELLULAR ALDEHYDE METABOLIC PROCESS	0.943232381	0.565512781
POSITIVE REGULATION OF AXONOGENESIS	0.942096604	0.567611763
GLYCOPROTEIN BIOSYNTHETIC PROCESS	0.941813002	0.568141361
POSITIVE REGULATION OF ANION TRANSPORT	0.941805652	0.568148986
AMINO SUGAR METABOLIC PROCESS	0.941675395	0.568399228
EXOCYTIC PROCESS	0.941563509	0.568606492
ORGANIC ANION TRANSPORT	0.941407031	0.568901098
TRANSCYTOSIS	0.941295834	0.569139556
NEGATIVE REGULATION OF INTRINSIC APOPTOTIC SIGNALI	0.941242108	0.569234523
INOSITOL PHOSPHATE MEDIATED SIGNALING	0.940907988	0.56985077
RESPONSE TO ESTROGEN	0.940643681	0.570378288
MANNOSYLATION	0.940067299	0.571481848
RESPONSE TO NICOTINE	0.938715218	0.574041802
VITAMIN METABOLIC PROCESS	0.938055885	0.575277762
POSITIVE REGULATION OF DEFENSE RESPONSE TO VIRUS	0.9377097	0.575927975
LYMPHOID PROGENITOR CELL DIFFERENTIATION	0.937243906	0.57677852
GLAND DEVELOPMENT	0.936554314	0.578150345
RAB PROTEIN SIGNAL TRANSDUCTION	0.936477153	0.578277892
ORGANIC ACID CATABOLIC PROCESS	0.936307654	0.578583589
SENSORY PERCEPTION OF CHEMICAL STIMULUS	0.934875868	0.581336943
POSITIVE REGULATION OF MUSCLE CONTRACTION	0.93419077	0.582693518
NEGATIVE REGULATION OF VIRAL PROCESS	0.932909126	0.585173062
LIVER REGENERATION	0.932645946	0.585673546
TRANSFERRIN TRANSPORT	0.932328077	0.586300884
REGULATION OF DNA DAMAGE RESPONSE SIGNAL TRANSD	0.931764281	0.587371864
RESPONSE TO THYROID HORMONE	0.930975949	0.588876088
MEGAKARYOCYTE DIFFERENTIATION	0.930975502	0.588876088
SARCOMERE ORGANIZATION	0.930662526	0.589459062
MESODERM DEVELOPMENT	0.930442025	0.589870124

LONG TERM MEMORY	0.929123618	0.59242176
ESTABLISHMENT OF PROTEIN LOCALIZATION TO ENDOPLAS	0.928992916	0.592668536
RESPONSE TO GROWTH HORMONE	0.928575182	0.593496899
CALCIUM DEPENDENT CELL CELL ADHESION VIA PLASMA M	0.928198391	0.594202567
CARDIAC MUSCLE CELL CONTRACTION	0.927519565	0.59548497
POSITIVE REGULATION OF ORGANELLE ORGANIZATION	0.927343442	0.595807997
REGULATION OF PEPTIDE TRANSPORT	0.926877861	0.596678645
ORGANIC CATION TRANSPORT	0.926132433	0.598093448
REGULATION OF PROTEIN POLYMERIZATION	0.925594732	0.599090256
I KAPPAB PHOSPHORYLATION	0.92523247	0.599784834
POSITIVE REGULATION OF CELL DIVISION	0.922829183	0.604450702
NECROTIC CELL DEATH	0.922733494	0.60463925
VIRAL LIFE CYCLE	0.922353448	0.605351157
RESPONSE TO TOXIC SUBSTANCE	0.921908776	0.606232896
CALCINEURIN MEDIATED SIGNALING	0.920390114	0.609095081
RESPONSE TO STEROID HORMONE	0.919126522	0.611550363
LONG CHAIN FATTY ACID TRANSPORT	0.916516775	0.616550349
CELLULAR CARBOHYDRATE BIOSYNTHETIC PROCESS	0.916066677	0.617383565
FOCAL ADHESION ASSEMBLY	0.915821173	0.61784523
POSITIVE REGULATION OF SMOOTH MUSCLE CELL MIGRATI	0.915564097	0.61831452
LUNG MORPHOGENESIS	0.915466523	0.61849267
POSITIVE REGULATION OF PEPTIDE SECRETION	0.913283945	0.622703112
SODIUM ION TRANSMEMBRANE TRANSPORT	0.913214653	0.622852148
POSITIVE REGULATION OF NITRIC OXIDE SYNTHASE ACTIVI	0.912072629	0.625077291
CELLULAR OXIDANT DETOXIFICATION	0.911769214	0.625676208
APPENDAGE DEVELOPMENT	0.911270271	0.626618947
REGULATION OF CARBOHYDRATE BIOSYNTHETIC PROCESS	0.910799529	0.627493061
RESPONSE TO MANGANESE ION	0.91003365	0.628925887
CELL MIGRATION INVOLVED IN SPROUTING ANGIOGENESIS	0.9080756	0.632642083
SEX DIFFERENTIATION	0.9076303	0.633528675
NUCLEOBASE CONTAINING SMALL MOLECULE METABOLIC P	0.907534069	0.633741484
OLIGODENDROCYTE DIFFERENTIATION	0.906790062	0.635109843
INNER EAR RECEPTOR CELL DEVELOPMENT	0.9066136	0.635443961
FATTY ACYL COA METABOLIC PROCESS	0.905444228	0.637678809
MEMBRANE PROTEIN INTRACELLULAR DOMAIN PROTEOLYS	0.90380478	0.640771827
NEURON MIGRATION	0.903473987	0.641407483
IMPORT INTO NUCLEUS	0.903385106	0.641598803
NEGATIVE REGULATION OF MACROAUTOPHAGY	0.902776755	0.642786933
PHAGOSOME ACIDIFICATION	0.90276479	0.642815353
NEGATIVE REGULATION OF DNA RECOMBINATION	0.902509192	0.64333594
CHONDROITIN SULFATE BIOSYNTHETIC PROCESS	0.902146046	0.644082506
ARP2 3 COMPLEX MEDIATED ACTIN NUCLEATION	0.901744964	0.644836698
PURINE NUCLEOSIDE MONOPHOSPHATE METABOLIC PROC	0.90172827	0.644863733
ACETYL COA BIOSYNTHETIC PROCESS	0.901514837	0.645272022
WATER HOMEOSTASIS	0.901400092	0.645486911
ICOSANOID SECRETION	0.901300315	0.645690709
INTERFERON ALPHA PRODUCTION	0.900680331	0.646849725
ASPARTATE FAMILY AMINO ACID BIOSYNTHETIC PROCESS	0.900486644	0.647210877
SOMATIC RECOMBINATION OF IMMUNOGLOBULIN GENE SE	0.900359588	0.647468744
POSITIVE REGULATION OF FOCAL ADHESION ASSEMBLY	0.899997011	0.648108559
NUCLEOBASE BIOSYNTHETIC PROCESS	0.899967769	0.648161935
NEUROMUSCULAR JUNCTION DEVELOPMENT	0.899890529	0.648306119
CILIUM MOVEMENT	0.899646501	0.648777488

REGULATION OF CELL SUBSTRATE JUNCTION ORGANIZATI(	0.898567227	0.650831413
REGULATION OF CELLULAR RESPONSE TO HEAT	0.897894283	0.652086782
PROTEIN LOCALIZATION TO POSTSYNAPSE	0.897605116	0.652635096
ENDOCRINE PROCESS	0.897405703	0.653003874
PROTEIN LOCALIZATION TO CELL PERIPHERY	0.897224822	0.653339378
OLIGODENDROCYTE DEVELOPMENT	0.896263941	0.655184652
NUCLEOTIDE TRANSPORT	0.89550122	0.656614705
REPRODUCTIVE SYSTEM DEVELOPMENT	0.895080693	0.65740009
RESPONSE TO INSULIN	0.894214806	0.65905751
SPERM MOTILITY	0.893769431	0.659905282
REGULATION OF SMOOTH MUSCLE CONTRACTION	0.893554703	0.660328129
SODIUM ION TRANSPORT	0.89333915	0.66073018
RESPONSE TO HEAT	0.892809709	0.661769273
MEMBRANE FUSION	0.892671618	0.662019515
NUCLEOTIDE BINDING DOMAIN LEUCINE RICH REPEAT CON	0.892658359	0.66204239
NEGATIVE REGULATION OF INNATE IMMUNE RESPONSE	0.892330007	0.662683592
POSITIVE REGULATION OF REACTIVE OXYGEN SPECIES BI(	0.89210921	0.663079404
CELLULAR RESPONSE TO GROWTH HORMONE STIMULUS	0.891858446	0.663541069
CARBOHYDRATE BIOSYNTHETIC PROCESS	0.891852904	0.66355216
CARDIAC MUSCLE CELL MEMBRANE REPOLARIZATION	0.891515732	0.664216237
RESPONSE TO OXYGEN LEVELS	0.888960421	0.669001334
RESPONSE TO XENOBIOTIC STIMULUS	0.888849329	0.669218996
REGULATION OF VACUOLE ORGANIZATION	0.888630306	0.669634217
ENERGY RESERVE METABOLIC PROCESS	0.888570916	0.669754139
POSITIVE REGULATION OF APOPTOTIC SIGNALING PATHWA	0.888220167	0.670401579
LABYRINTHINE LAYER MORPHOGENESIS	0.888190806	0.670452875
REGULATION OF CELL JUNCTION ASSEMBLY	0.887624094	0.671514844
REGULATION OF FIBROBLAST MIGRATION	0.887594832	0.67156822
SMALL MOLECULE CATABOLIC PROCESS	0.887467485	0.671814302
POSITIVE REGULATION OF BINDING	0.887303496	0.672127625
VESICLE ORGANIZATION	0.886082093	0.674435257
REGULATION OF CHOLESTEROL METABOLIC PROCESS	0.884288535	0.677872098
PROTEIN N LINKED GLYCOSYLATION	0.883440663	0.67945188
ATP METABOLIC PROCESS	0.882580441	0.681080186
PLASMA LIPOPROTEIN PARTICLE CLEARANCE	0.882365637	0.681500952
DEVELOPMENT OF PRIMARY SEXUAL CHARACTERISTICS	0.88231107	0.681597306
PROTEIN LOCALIZATION TO PLASMA MEMBRANE	0.880631178	0.684748552
NEGATIVE REGULATION OF RESPONSE TO DNA DAMAGE ST	0.880498454	0.684999487
TRNA TRANSPORT	0.879698572	0.686517575
SYNAPSE MATURATION	0.878847722	0.688133403
POSITIVE REGULATION OF PROTEIN CONTAINING COMPLEX	0.878387117	0.689018608
MODULATION BY SYMBIONT OF HOST PROCESS	0.878292936	0.689184281
REGULATION OF LEUKOCYTE ADHESION TO VASCULAR ENI	0.878073946	0.689566229
MITOCHONDRIAL RNA METABOLIC PROCESS	0.876921574	0.691744235
RESPONSE TO FATTY ACID	0.876183981	0.693160424
RESPONSE TO LEPTIN	0.875503169	0.694394304
POSITIVE REGULATION OF PROTEIN IMPORT	0.875393967	0.694596022
ANTERIOR POSTERIOR PATTERN SPECIFICATION	0.875179234	0.694984209
POSITIVE REGULATION OF NUCLEOTIDE METABOLIC PROCI	0.875005028	0.695302384
MAMMARY GLAND DEVELOPMENT	0.873304322	0.698467494
SCHWANN CELL DEVELOPMENT	0.871649812	0.701489113
POSITIVE REGULATION OF CALCIUM ION TRANSMEMBRANE	0.871567881	0.701636762
IMPORT INTO CELL	0.871182543	0.702345897

RNA POLYMERASE II PREINITIATION COMPLEX ASSEMBLY	0.871177339	0.702359067
PROTEIN MATURATION	0.871043316	0.70260515
REGULATION OF PROTEIN LOCALIZATION TO MEMBRANE	0.869725334	0.705029239
PURINE NUCLEOTIDE TRANSPORT	0.869548794	0.705357811
INNER EAR AUDITORY RECEPTOR CELL DIFFERENTIATION	0.869215611	0.705956035
MONOCYTE DIFFERENTIATION	0.86875295	0.706817671
REGULATION OF PROTEASOMAL UBIQUITIN DEPENDENT PR	0.867826809	0.708479943
AUTOPHAGOSOME ORGANIZATION	0.867820822	0.708491034
NEGATIVE REGULATION OF CALCIUM ION TRANSMEMBRANE	0.867704903	0.708710083
INTERLEUKIN 6 MEDIATED SIGNALING PATHWAY	0.86722508	0.709610538
NEGATIVE REGULATION OF STEM CELL DIFFERENTIATION	0.866303216	0.711283901
SIGNAL TRANSDUCTION IN ABSENCE OF LIGAND	0.86619827	0.711489085
REGULATION OF ACTIN FILAMENT BASED MOVEMENT	0.865645999	0.712499757
TUBE FORMATION	0.864678871	0.714268087
EMBRYONIC PLACENTA MORPHOGENESIS	0.863920586	0.715685663
POST EMBRYONIC DEVELOPMENT	0.863196187	0.717001339
SINGLE FERTILIZATION	0.863020545	0.717307036
NEGATIVE REGULATION OF CD4 POSITIVE ALPHA BETA T CE	0.862988388	0.717370117
PEPTIDYL SERINE MODIFICATION	0.862655123	0.717960715
NADP METABOLIC PROCESS	0.862583171	0.718078558
MESODERM MORPHOGENESIS	0.861327707	0.720351531
CARDIAC MUSCLE CELL PROLIFERATION	0.860672105	0.721543126
POSITIVE REGULATION OF RELEASE OF CYTOCHROME C FR	0.859797555	0.723123601
PEPTIDYL ARGININE MODIFICATION	0.859770885	0.723172818
ESTABLISHMENT OF PIGMENT GRANULE LOCALIZATION	0.85919251	0.724204286
PIGMENT GRANULE LOCALIZATION	0.85919251	0.724204286
DEPHOSPHORYLATION	0.85886246	0.724792805
NEGATIVE REGULATION OF AUTOPHAGY	0.858478367	0.725481837
REGULATION OF CELLULAR AMINO ACID METABOLIC PROCE	0.858417691	0.725594134
NEGATIVE REGULATION OF GTPASE ACTIVITY	0.85822776	0.725952514
NEGATIVE REGULATION OF PROTEIN MODIFICATION PROCE	0.857859811	0.72663392
RUFFLE ASSEMBLY	0.857335368	0.727595376
RESPONSE TO FLUID SHEAR STRESS	0.857111979	0.727993961
POSTSYNAPTIC SIGNAL TRANSDUCTION	0.856509867	0.729059395
MITOCHONDRIAL TRANSCRIPTION	0.85482958	0.732105276
MONOCARBOXYLIC ACID CATABOLIC PROCESS	0.853950323	0.733668422
ORGANOPHOSPHATE CATABOLIC PROCESS	0.85359609	0.7342916
CHONDROCYTE DEVELOPMENT	0.853570218	0.734335965
PROTEIN POLYMERIZATION	0.853307148	0.734794857
REGULATION OF INTRACELLULAR PROTEIN TRANSPORT	0.852210828	0.736705984
CELLULAR AMINO ACID BIOSYNTHETIC PROCESS	0.851545442	0.737837272
REGULATION OF BINDING	0.851092603	0.738637214
PHOSPHATIDIC ACID METABOLIC PROCESS	0.850890509	0.739003912
ADULT BEHAVIOR	0.850608998	0.739506476
DENDRITE MORPHOGENESIS	0.850608046	0.739507169
REGULATION OF CELL MATRIX ADHESION	0.850504219	0.739688092
GUANOSINE CONTAINING COMPOUND METABOLIC PROCES	0.84984478	0.74083463
REGULATION OF PROTEIN LOCALIZATION TO SYNAPSE	0.849313508	0.741794006
TISSUE REGENERATION	0.848880182	0.742550277
PHOSPHATIDYLCHOLINE ACYL CHAIN REMODELING	0.848328965	0.743513813
HEART TRABECULA MORPHOGENESIS	0.848302435	0.743563029
DORSAL VENTRAL PATTERN FORMATION	0.846621882	0.746459181
NUCLEOBASE CONTAINING SMALL MOLECULE BIOSYNTHET	0.846615065	0.746472351

EMBRYONIC SKELETAL SYSTEM DEVELOPMENT	0.846205088	0.747198122
MITOCHONDRIAL GENOME MAINTENANCE	0.845816077	0.747853881
INTRINSIC APOPTOTIC SIGNALING PATHWAY IN RESPONSE	0.845490533	0.7484424
GTP METABOLIC PROCESS	0.845362265	0.748656596
CELLULAR RESPONSE TO HEAT	0.845272624	0.748815337
SMOOTH MUSCLE CELL APOPTOTIC PROCESS	0.845052431	0.749188273
REGULATION OF RECEPTOR LOCALIZATION TO SYNAPSE	0.843290455	0.752193256
NUCLEOSIDE CATABOLIC PROCESS	0.84249411	0.753560922
NEGATIVE REGULATION OF INTRACELLULAR STEROID HOR	0.839816298	0.758154698
CYTOCHROME COMPLEX ASSEMBLY	0.838821827	0.759815583
BRAIN MORPHOGENESIS	0.83825605	0.760800608
POSITIVE REGULATION OF PROTEIN POLYMERIZATION	0.838189586	0.760910132
POSITIVE REGULATION OF PATTERN RECOGNITION RECEPT	0.837755191	0.761624119
POSITIVE REGULATION OF PROTEIN DEACETYLATION	0.837556987	0.761959623
REGULATION OF INTRINSIC APOPTOTIC SIGNALING PATHW	0.837457309	0.762113511
INACTIVATION OF MAPK ACTIVITY	0.837429345	0.76215441
GLYCEROPHOSPHOLIPID CATABOLIC PROCESS	0.837294111	0.762372765
REGULATION OF CELL CYCLE CHECKPOINT	0.836812663	0.763169934
RESPONSE TO HYDROGEN PEROXIDE	0.836241072	0.764175754
MAINTENANCE OF PROTEIN LOCATION IN CELL	0.835718133	0.765048482
TRANSITION METAL ION HOMEOSTASIS	0.834951374	0.766358613
NEGATIVE REGULATION OF SIGNAL TRANSDUCTION IN ABS	0.834006781	0.767947406
REGULATION OF CELL MIGRATION INVOLVED IN SPROUTIN	0.834006371	0.767947406
TRICARBOXYLIC ACID CYCLE	0.833304081	0.769080773
WATER SOLUBLE VITAMIN METABOLIC PROCESS	0.832375387	0.770673726
CEREBRAL CORTEX RADIALLY ORIENTED CELL MIGRATION	0.831748393	0.771711433
ADULT LOCOMOTORY BEHAVIOR	0.831537086	0.77206704
CELLULAR RESPONSE TO REACTIVE OXYGEN SPECIES	0.830872531	0.773152577
REGULATION OF CYTOPLASMIC TRANSPORT	0.830662811	0.773498479
NEGATIVE REGULATION OF DNA METABOLIC PROCESS	0.829789894	0.77493685
HEMATOPOIETIC STEM CELL PROLIFERATION	0.829663468	0.775135103
NEGATIVE REGULATION OF CARBOHYDRATE METABOLIC PI	0.828569722	0.776924229
INTERMEMBRANE LIPID TRANSFER	0.828417244	0.777170311
CELL DEATH IN RESPONSE TO OXIDATIVE STRESS	0.828352368	0.777270131
POSITIVE REGULATION OF INTRINSIC APOPTOTIC SIGNALIN	0.826848588	0.779763539
MAINTENANCE OF BLOOD BRAIN BARRIER	0.825971215	0.781161012
RETROGRADE TRANSPORT ENDOSOME TO GOLGI	0.825920588	0.78124766
VESICLE MEDIATED TRANSPORT IN SYNAPSE	0.824845717	0.783019456
SUBSTANTIA NIGRA DEVELOPMENT	0.824688637	0.783272471
POSITIVE REGULATION OF DNA BINDING	0.824195536	0.78405855
SOMATIC DIVERSIFICATION OF IMMUNOGLOBULINS	0.823055997	0.785901744
RESPONSE TO CARBOHYDRATE	0.823024861	0.785951654
RETINA HOMEOSTASIS	0.822462523	0.786859041
RESPONSE TO CADMIUM ION	0.821862034	0.787837827
NUCLEOSIDE BISPHOSPHATE BIOSYNTHETIC PROCESS	0.821560353	0.788327219
PROTEIN IMPORT	0.821059601	0.789151423
INTRINSIC APOPTOTIC SIGNALING PATHWAY IN RESPONSE	0.820451168	0.790121198
DETECTION OF LIGHT STIMULUS	0.820366741	0.790257063
REGULATION OF MITOCHONDRIAL FISSION	0.819415792	0.79179456
QUINONE METABOLIC PROCESS	0.81905367	0.792369216
REGULATION OF AUTOPHAGOSOME ASSEMBLY	0.81834894	0.793507435
NEGATIVE REGULATION OF DEVELOPMENTAL GROWTH	0.818038558	0.794011385
PROTEIN LOCALIZATION TO CELL SURFACE	0.817930916	0.794181217

WNT SIGNALING PATHWAY CALCIUM MODULATING PATHWA	0.817309281	0.795169014
CARDIAC MUSCLE CELL ACTION POTENTIAL INVOLVED IN C	0.817270845	0.795230708
NEUROTROPHIN TRK RECEPTOR SIGNALING PATHWAY	0.816127008	0.797058652
NEGATIVE REGULATION OF CALCIUM ION TRANSMEMBRANE	0.815865914	0.797451692
ATP SYNTHESIS COUPLED PROTON TRANSPORT	0.814563036	0.799512548
NEGATIVE REGULATION OF PROTEIN CONTAINING COMPLE	0.813910841	0.800519061
GLUTAMINE METABOLIC PROCESS	0.813828648	0.800650768
REGULATION OF CARDIAC MUSCLE CONTRACTION BY CALC	0.813044267	0.801858306
NUCLEOBASE CONTAINING SMALL MOLECULE CATABOLIC P	0.812974472	0.801976842
REGULATION OF JUN KINASE ACTIVITY	0.81253719	0.80263468
PROTEIN LOCALIZATION TO MEMBRANE	0.811986454	0.803519192
NEGATIVE REGULATION OF SUPRAMOLECULAR FIBER ORG	0.81193893	0.80359267
MICROVILLUS ORGANIZATION	0.811578022	0.804140984
REGULATION OF T CELL DIFFERENTIATION IN THYMUS	0.811082094	0.804915278
LENS FIBER CELL DIFFERENTIATION	0.810865963	0.805242464
REGULATION OF KIDNEY DEVELOPMENT	0.810761293	0.805422001
SOMITOGENESIS	0.810383168	0.806009827
RIBONUCLEOSIDE MONOPHOSPHATE METABOLIC PROCESS	0.810327783	0.806102021
PURINE NUCLEOSIDE BIOSYNTHETIC PROCESS	0.809963909	0.806652415
POSITIVE REGULATION OF JUN KINASE ACTIVITY	0.809473514	0.807396209
NEGATIVE REGULATION OF INTERLEUKIN 2 PRODUCTION	0.808831221	0.808389551
SOMITE DEVELOPMENT	0.808157178	0.809431417
REGULATION OF MONOCYTE DIFFERENTIATION	0.807968059	0.809718398
CELLULAR RESPONSE TO GAMMA RADIATION	0.807407854	0.810564784
FAT SOLUBLE VITAMIN METABOLIC PROCESS	0.807378036	0.810606376
POSITIVE REGULATION OF CELL JUNCTION ASSEMBLY	0.807359343	0.810629944
HEME METABOLIC PROCESS	0.806856638	0.811341851
APOPTOTIC SIGNALING PATHWAY	0.806441727	0.811963644
RESPONSE TO GAMMA RADIATION	0.806070897	0.812521662
DIGESTIVE SYSTEM DEVELOPMENT	0.806066036	0.812530674
RESPONSE TO COLD	0.805654467	0.813135136
L ALPHA AMINO ACID TRANSMEMBRANE TRANSPORT	0.804567924	0.814745419
ACTIVATION OF JUN KINASE ACTIVITY	0.804395493	0.814994274
CELLULAR RESPONSE TO STEROL DEPLETION	0.804228869	0.815236891
REGULATION OF ANDROGEN RECEPTOR SIGNALING PATHW	0.803488608	0.816330053
NEGATIVE REGULATION OF CATABOLIC PROCESS	0.801528461	0.819210261
POTASSIUM ION IMPORT ACROSS PLASMA MEMBRANE	0.800706701	0.820428891
PURINE NUCLEOSIDE METABOLIC PROCESS	0.80012812	0.821253095
POSITIVE REGULATION OF OXIDOREDUCTASE ACTIVITY	0.799575797	0.822066208
REGULATION OF DENDRITIC SPINE MORPHOGENESIS	0.799531422	0.822152164
MOLTING CYCLE	0.799333686	0.822421122
MITOCHONDRIAL TRANSLATION	0.798322799	0.823935744
POSITIVE REGULATION OF CELL CYCLE G2 M PHASE TRANS	0.797783628	0.824732914
PROTEIN MANNOSYLATION	0.796960837	0.825946691
HISTONE H4 K16 ACETYLATION	0.796503945	0.826602449
PRESYNAPSE ORGANIZATION	0.796479056	0.826642655
ENDOPLASMIC RETICULUM ORGANIZATION	0.795656585	0.827782954
REGULATION OF POSTSYNAPSE ORGANIZATION	0.795536623	0.827965956
RESPIRATORY CHAIN COMPLEX IV ASSEMBLY	0.79511291	0.828603692
NEGATIVE REGULATION OF VIRAL GENOME REPLICATION	0.793706817	0.830587604
POSITIVE REGULATION OF GLUCOSE METABOLIC PROCESS	0.793602987	0.830742186
NEGATIVE REGULATION OF RESPONSE TO ENDOPLASMIC R	0.793474062	0.830919643
CELLULAR RESPONSE TO VIRUS	0.793092746	0.831476968

NUCLEOSIDE PHOSPHATE BIOSYNTHETIC PROCESS	0.792824904	0.831863075
CELLULAR TRANSITION METAL ION HOMEOSTASIS	0.792400646	0.832471697
CHONDROITIN SULFATE PROTEOGLYCAN METABOLIC PROC	0.792151017	0.832821065
PEPTIDYL CYSTEINE MODIFICATION	0.791263028	0.834085445
NEGATIVE REGULATION OF DEFENSE RESPONSE TO VIRUS	0.789189287	0.836975358
RESPONSE TO ZINC ION	0.789153352	0.837033586
AORTIC VALVE MORPHOGENESIS	0.788618129	0.837756585
MORPHOGENESIS OF AN EPITHELIAL SHEET	0.78809038	0.83847681
POSITIVE REGULATION OF ANION TRANSMEMBRANE TRANS	0.787580273	0.839167228
REGULATION OF CALCIUM ION DEPENDENT EXOCYTOSIS	0.785640604	0.841874139
NCRNA CATABOLIC PROCESS	0.785529378	0.842025948
SOMATIC DIVERSIFICATION OF IMMUNE RECEPTORS	0.784803199	0.84301929
PROTEIN INSERTION INTO ER MEMBRANE	0.784387245	0.843535024
TORC1 SIGNALING	0.784194992	0.843807448
REGULATION OF MITOCHONDRION ORGANIZATION	0.784086063	0.843948859
CLEAVAGE INVOLVED IN RRNA PROCESSING	0.783922448	0.844177612
KERATINOCYTE PROLIFERATION	0.783460004	0.844800098
POSITIVE REGULATION OF PROTEIN LOCALIZATION TO CEL	0.783320495	0.844986566
NEGATIVE REGULATION OF PEPTIDYL SERINE PHOSPHORY	0.782883295	0.845602813
CALCIUM ION REGULATED EXOCYTOSIS	0.782378385	0.846289072
PROTEIN DEPHOSPHORYLATION	0.782197209	0.846490097
REGULATION OF AUTOPHAGY	0.782093449	0.846628042
REGULATION OF ATP METABOLIC PROCESS	0.781845947	0.84694691
REGULATION OF DEFENSE RESPONSE TO VIRUS BY HOST	0.78051997	0.848648
POSITIVE REGULATION OF POTASSIUM ION TRANSPORT	0.780415335	0.848789411
ALPHA AMINO ACID BIOSYNTHETIC PROCESS	0.779863172	0.849482602
VENTRICULAR SEPTUM DEVELOPMENT	0.77983727	0.84951241
POSITIVE REGULATION OF HISTONE DEACETYLATION	0.779589909	0.849849993
REGULATION OF SODIUM ION TRANSMEMBRANE TRANSPOI	0.779349102	0.850178566
PROTEIN LOCALIZATION TO ENDOPLASMIC RETICULUM	0.779189114	0.85040108
REGULATION OF MACROAUTOPHAGY	0.777732221	0.852319833
PROTEIN TARGETING TO VACUOLE	0.77665245	0.853710374
REGULATION OF NUCLEASE ACTIVITY	0.776622705	0.853739488
MITOCHONDRIAL RNA PROCESSING	0.774348742	0.856639105
SOMATIC STEM CELL POPULATION MAINTENANCE	0.773631459	0.857518765
MITOCHONDRIAL ATP SYNTHESIS COUPLED PROTON TRAN	0.772956125	0.858376242
ESTABLISHMENT OF ORGANELLE LOCALIZATION	0.772754472	0.858607074
REGULATION OF GOLGI ORGANIZATION	0.772250482	0.859230946
AMINO ACID TRANSMEMBRANE TRANSPORT	0.771694771	0.859931069
REGULATION OF NECROTIC CELL DEATH	0.771033673	0.860742796
POSITIVE REGULATION OF CELL SUBSTRATE JUNCTION OR	0.770881736	0.860937582
MITOCHONDRION ORGANIZATION	0.770847096	0.860987492
CELLULAR RESPONSE TO DSRNA	0.769268711	0.863012303
RESPONSE TO ANGIOTENSIN	0.769103016	0.863212635
POSITIVE REGULATION OF CELL CYCLE PHASE TRANSITION	0.767379889	0.865322015
PROTEIN LOCALIZATION TO LYSOSOME	0.76614636	0.866800591
NEUTRAL LIPID BIOSYNTHETIC PROCESS	0.765116012	0.868098938
PROTEIN LOCALIZATION TO VACUOLE	0.764702638	0.868602888
REGULATION OF DNA METHYLATION	0.764357281	0.869027814
REGULATION OF NOTCH SIGNALING PATHWAY	0.763604957	0.869931735
RESPONSE TO AUDITORY STIMULUS	0.763452004	0.870123055
SELECTIVE AUTOPHAGY	0.762270937	0.871537165
ENDOMEMBRANE SYSTEM ORGANIZATION	0.759812506	0.87446659

VESICLE DOCKING	0.759641724	0.874687717
REGULATION OF PROTEIN DEACETYLATION	0.759604475	0.874734161
RRNA CATABOLIC PROCESS	0.759478706	0.874890822
PROTEIN TRANSMEMBRANE TRANSPORT	0.758393065	0.876148271
REGULATION OF RUFFLE ASSEMBLY	0.757408061	0.877287183
MITOCHONDRIAL GENE EXPRESSION	0.757099985	0.877640711
HISTONE H4 ACETYLATION	0.755904947	0.87902224
CARDIAC CHAMBER DEVELOPMENT	0.754994633	0.880090448
POSITIVE REGULATION OF EPIDERMAL CELL DIFFERENTIAT	0.753428221	0.881864323
PROTEIN PALMITOYLATION	0.75228546	0.883114146
NEURAL NUCLEUS DEVELOPMENT	0.75220082	0.883213966
POSITIVE REGULATION OF GLUCOSE TRANSMEMBRANE TR	0.75169708	0.883744257
GOLGI TO VACUOLE TRANSPORT	0.75139156	0.884100557
REGULATION OF HISTONE DEACETYLATION	0.750684434	0.884912977
SPERM EGG RECOGNITION	0.750238214	0.885387119
POSITIVE REGULATION OF MITOTIC NUCLEAR DIVISION	0.74740151	0.888409432
THIOESTER BIOSYNTHETIC PROCESS	0.746982464	0.88885862
PLASMA MEMBRANE TUBULATION	0.745489237	0.890458504
PROTEIN LOCALIZATION TO GOLGI APPARATUS	0.74464205	0.89133747
MITOPHAGY	0.743897549	0.892123549
NEGATIVE REGULATION OF PROTEIN POLYMERIZATION	0.743443068	0.892584521
STRIATED MUSCLE CELL PROLIFERATION	0.74275848	0.89330336
REGULATION OF TRANSCRIPTION REGULATORY REGION DI	0.742653398	0.893423975
POSITIVE REGULATION OF FILOPODIUM ASSEMBLY	0.741249775	0.894872051
EMBRYONIC DIGIT MORPHOGENESIS	0.741104472	0.895021087
DOPAMINE TRANSPORT	0.740858198	0.895251919
RIBONUCLEOSIDE METABOLIC PROCESS	0.739692732	0.896442128
POSITIVE REGULATION OF INTERFERON ALPHA PRODUCTIO	0.738849902	0.897321094
ORGANELLE LOCALIZATION	0.736298694	0.899904617
TOLL LIKE RECEPTOR 4 SIGNALING PATHWAY	0.736253295	0.899942049
REGULATION OF MESENCHYMAL CELL PROLIFERATION	0.732343339	0.9037851
RESPONSE TO EPIDERMAL GROWTH FACTOR	0.731746252	0.904390949
PEPTIDYL L CYSTEINE S PALMITOYLATION	0.731286068	0.904858852
RIBONUCLEOSIDE MONOPHOSPHATE BIOSYNTHETIC PROC	0.731149677	0.904973922
REGULATION OF OLIGODENDROCYTE DIFFERENTIATION	0.730722879	0.905382212
REGULATION OF PROTEIN STABILITY	0.730376338	0.905710091
REGULATION OF INTRACELLULAR STEROID HORMONE REC	0.729698378	0.906360304
RESPONSE TO PROGESTERONE	0.728930189	0.907084689
REGULATION OF GENE SILENCING BY RNA	0.727909713	0.908054463
BRANCHING INVOLVED IN MAMMARY GLAND DUCT MORPHO	0.727654102	0.908291534
REGULATION OF EPIDERMIS DEVELOPMENT	0.726312306	0.909552448
REGULATION OF RECEPTOR RECYCLING	0.725384342	0.910393982
NEGATIVE REGULATION OF ACTIN FILAMENT POLYMERIZAT	0.723982728	0.911626475
COPII COATED VESICLE BUDDING	0.723809442	0.911780364
CELL CELL ADHESION MEDIATED BY CADHERIN	0.723622297	0.911950889
NUCLEOSIDE TRIPHOSPHATE METABOLIC PROCESS	0.718822375	0.916254911
REGULATION OF ERBB SIGNALING PATHWAY	0.718481483	0.916531494
CYTOSOLIC TRANSPORT	0.718100974	0.916847589
CRISTAE FORMATION	0.716961565	0.917910944
EXTRINSIC APOPTOTIC SIGNALING PATHWAY VIA DEATH DO	0.716771116	0.91806206
GLYCEROL ETHER METABOLIC PROCESS	0.715597717	0.919062335
POSITIVE REGULATION OF TYPE I INTERFERON PRODUCTIO	0.713432526	0.920897904
ACETYL COA METABOLIC PROCESS	0.71293155	0.921308966

REGULATION OF RESPONSE TO DNA DAMAGE STIMULUS	0.711939209	0.922156046
RESPONSE TO ALKALOID	0.711676209	0.922366776
REGULATION OF STEM CELL DIFFERENTIATION	0.711295053	0.922682871
CENTRAL NERVOUS SYSTEM PROJECTION NEURON AXONO	0.711162178	0.922792395
PROCESS UTILIZING AUTOPHAGIC MECHANISM	0.711016963	0.922920635
NEGATIVE REGULATION OF MITOCHONDRION ORGANIZATIO	0.710249903	0.923536882
MITOCHONDRIAL CALCIUM ION TRANSMEMBRANE TRANSPO	0.709755444	0.923926455
EXECUTION PHASE OF APOPTOSIS	0.709059751	0.924499724
CELL VOLUME HOMEOSTASIS	0.708410329	0.925033481
PEPTIDYL SERINE DEPHOSPHORYLATION	0.70556507	0.927298829
PROTEIN LOCALIZATION TO MICROTUBULE ORGANIZING CE	0.705282414	0.92752065
NEGATIVE REGULATION OF CYTOSKELETON ORGANIZATIO	0.703757168	0.928715711
RNA PHOSPHODIESTER BOND HYDROLYSIS EXONUCLEOLY	0.702581799	0.929605768
CEREBELLAR CORTEX MORPHOGENESIS	0.700167246	0.931414304
ZINC ION TRANSPORT	0.699938081	0.931577203
ANDROGEN METABOLIC PROCESS	0.698966657	0.932337634
POSITIVE REGULATION OF GLUCOSE IMPORT	0.698718323	0.93251925
MEMBRANE DOCKING	0.697471404	0.933455058
REGULATION OF RESPONSE TO ENDOPLASMIC RETICULUM	0.69741335	0.933495956
MODULATION BY SYMBIONT OF HOST CELLULAR PROCESS	0.694592708	0.935581074
REGULATION OF NUCLEOTIDE METABOLIC PROCESS	0.694561737	0.93560187
REGULATION OF POTASSIUM ION TRANSMEMBRANE TRANS	0.693788935	0.936175139
POSITIVE REGULATION OF MESENCHYMAL CELL PROLIFER/	0.693383455	0.936460734
PHOSPHATIDYLINOSITOL DEPHOSPHORYLATION	0.69334644	0.936487768
MATURATION OF 5 8S RRNA FROM TRICISTRONIC RRNA TR/	0.692846526	0.936830204
REGULATION OF CARBOHYDRATE CATABOLIC PROCESS	0.692783813	0.936875955
RNA PHOSPHODIESTER BOND HYDROLYSIS	0.689643422	0.939087927
REGULATION OF RESPONSE TO INTERFERON GAMMA	0.689600385	0.9391212
CELLULAR COMPONENT MAINTENANCE	0.68903363	0.939512853
TRNA WOBBLE BASE MODIFICATION	0.688637105	0.939794982
MACROAUTOPHAGY	0.685772216	0.941819793
CARDIAC MUSCLE CELL ACTION POTENTIAL	0.685511261	0.941991011
RESPONSE TO INTERFERON BETA	0.684411495	0.942761146
REGULATION OF TORC1 SIGNALING	0.683500583	0.943379472
3 UTR MEDIATED MRNA STABILIZATION	0.68286333	0.943801625
3 UTR MEDIATED MRNA DESTABILIZATION	0.679784471	0.945779992
MAINTENANCE OF PROTEIN LOCATION	0.677846451	0.946996542
HINDBRAIN MORPHOGENESIS	0.67706596	0.947481776
CELL SUBSTRATE JUNCTION ORGANIZATION	0.676972875	0.947540004
POSITIVE REGULATION OF EPIDERMIS DEVELOPMENT	0.676269947	0.947955919
NUCLEOSIDE METABOLIC PROCESS	0.675018006	0.94871011
MITOCHONDRION MORPHOGENESIS	0.674095489	0.949244561
PROTEIN LOCALIZATION TO KINETOCHORE	0.673787753	0.949424097
NEGATIVE REGULATION OF CELLULAR SENESCENCE	0.672304809	0.950342575
ESTABLISHMENT OF PROTEIN LOCALIZATION TO ORGANELI	0.67171353	0.950700955
NEGATIVE REGULATION OF EPITHELIAL TO MESENCHYMAL	0.670281868	0.951541795
VESICLE BUDDING FROM MEMBRANE	0.669983066	0.951734502
REGULATION OF ESTABLISHMENT OR MAINTENANCE OF CE	0.667497093	0.95312643
POSITIVE REGULATION OF INTERFERON BETA PRODUCTIOI	0.665014405	0.954507959
PROTEIN SUMOYLATION	0.664259997	0.95492734
LATE ENDOSOME TO VACUOLE TRANSPORT	0.663481153	0.955355039
DNA DOUBLE STRAND BREAK PROCESSING	0.663390597	0.955408414
RESPONSE TO OXYGEN RADICAL	0.657686338	0.958426568

MITOCHONDRIAL FUSION	0.656258519	0.959138475
NEGATIVE REGULATION OF CD4 POSITIVE ALPHA BETA T CE	0.655884836	0.959327023
SNRNA PROCESSING	0.654148075	0.960165784
ESTABLISHMENT OF PROTEIN LOCALIZATION TO VACUOLE	0.652964985	0.960748064
CYTOPLASMIC PATTERN RECOGNITION RECEPTOR SIGNAL	0.650983093	0.961699122
REGULATION OF PHOSPHOLIPID METABOLIC PROCESS	0.650474551	0.961959068
HYDROGEN PEROXIDE METABOLIC PROCESS	0.647026919	0.963579749
ORGANIC CYCLIC COMPOUND CATABOLIC PROCESS	0.646799948	0.963694125
PROTEIN CATABOLIC PROCESS IN THE VACUOLE	0.645131761	0.964441385
GOLGI ORGANIZATION	0.644321761	0.964803924
PROTEIN AUTOUBIQUITINATION	0.642577541	0.965548411
POST GOLGI VESICLE MEDIATED TRANSPORT	0.641666026	0.965987894
RIBONUCLEOSIDE TRIPHOSPHATE METABOLIC PROCESS	0.641154944	0.966197238
CELLULAR PIGMENTATION	0.640953861	0.966264477
REGULATION OF VIRAL TRANSCRIPTION	0.637207122	0.967831782
TYPE I INTERFERON PRODUCTION	0.635932333	0.96837663
POSITIVE REGULATION OF MITOCHONDRIAL TRANSLATION	0.635377435	0.968621326
GLYCOSYL COMPOUND CATABOLIC PROCESS	0.634179256	0.969141913
POSITIVE REGULATION OF SODIUM ION TRANSPORT	0.633707809	0.969337393
RESPONSE TO AMINO ACID STARVATION	0.63286295	0.969668738
ENDOSOMAL TRANSPORT	0.632198041	0.969937003
NEGATIVE REGULATION OF DNA BIOSYNTHETIC PROCESS	0.631750714	0.970131096
PHOTOPERIODISM	0.63041531	0.970653069
NCRNA 3 END PROCESSING	0.627726603	0.971662355
EXTRACELLULAR TRANSPORT	0.627235616	0.971845357
REGULATION OF TRANSCRIPTION BY RNA POLYMERASE III	0.626525095	0.972083122
CELLULAR METABOLIC COMPOUND SALVAGE	0.626005222	0.972258499
PROTEIN K11 LINKED UBIQUITINATION	0.624211099	0.972937826
HORMONE MEDIATED SIGNALING PATHWAY	0.618962108	0.974746361
MITOCHONDRIAL TRANSLATIONAL TERMINATION	0.618116277	0.975038888
NEURON DEATH IN RESPONSE TO OXIDATIVE STRESS	0.61632249	0.975617702
NEGATIVE REGULATION OF TELOMERE MAINTENANCE VIA T	0.615311072	0.975955286
INTRACELLULAR STEROID HORMONE RECEPTOR SIGNALING	0.615140038	0.976023219
REGULATION OF OXIDATIVE STRESS INDUCED NEURON DEA	0.614797812	0.976121652
PROTEIN TARGETING TO LYSOSOME	0.613715223	0.976506373
REGULATION OF INTRACELLULAR ESTROGEN RECEPTOR S	0.61352067	0.976571533
REGULATION OF ERAD PATHWAY	0.613498923	0.976581931
REGULATION OF HEMATOPOIETIC STEM CELL DIFFERENTIA	0.61063096	0.977517046
PROTEIN TARGETING	0.610301757	0.97762657
MITOCHONDRION LOCALIZATION	0.610117138	0.977685491
REGULATION OF MICROTUBULE BASED MOVEMENT	0.606849834	0.978715573
VACUOLAR TRANSPORT	0.604520989	0.979393513
ACTIN FILAMENT DEPOLYMERIZATION	0.602082521	0.980045113
TRNA MODIFICATION	0.597954036	0.981221458
ENTRAINMENT OF CIRCADIAN CLOCK	0.596557187	0.981591622
NEGATIVE REGULATION OF DNA REPAIR	0.596250582	0.98168035
EXTRACELLULAR VESICLE BIOGENESIS	0.594956738	0.982041503
REGULATION OF TOLL LIKE RECEPTOR 4 SIGNALING PATHW	0.594054807	0.982288972
VESICLE LOCALIZATION	0.590307875	0.983215075
INTERFERON BETA PRODUCTION	0.587880905	0.983810526
VESICLE TARGETING	0.587572242	0.983882618
POLARIZED EPITHELIAL CELL DIFFERENTIATION	0.583675779	0.98484338
THYMUS DEVELOPMENT	0.580750883	0.985499832

DNA TEMPLATED TRANSCRIPTION TERMINATION	0.579896216	0.985696005
MITOTIC RECOMBINATION	0.579891561	0.985696698
TRANSLATIONAL TERMINATION	0.578130904	0.986062703
MITOCHONDRIAL FISSION	0.574333073	0.986880669
ENDOCYTIC RECYCLING	0.573332626	0.987083774
HEMATOPOIETIC STEM CELL DIFFERENTIATION	0.571412186	0.987461563
REGULATION OF CENTROSOME CYCLE	0.567461532	0.988188027
ISOPRENOID BIOSYNTHETIC PROCESS	0.564797248	0.98865801
PROTEIN LOCALIZATION TO CILIUM	0.564744572	0.988665635
REGULATION OF LIPOPOLYSACCHARIDE MEDIATED SIGNAL	0.563816663	0.988816751
REGULATION OF INTRINSIC APOPTOTIC SIGNALING PATHW	0.555781122	0.990158768
PROTEIN ADP RIBOSYLATION	0.55461001	0.990359101
NEGATIVE REGULATION OF DNA DAMAGE RESPONSE SIGN	0.554327854	0.990404851
PROTEIN FOLDING	0.548669823	0.991254703
PROTEIN LIPID COMPLEX ASSEMBLY	0.547221263	0.991446717
NEGATIVE REGULATION OF PLASMA MEMBRANE BOUNDED	0.545027772	0.99174063
TRANSCRIPTION PREINITIATION COMPLEX ASSEMBLY	0.544605011	0.991796779
SYNAPTIC VESICLE LOCALIZATION	0.542392371	0.992077521
REGULATION OF CENTRIOLE REPLICATION	0.540330165	0.992350638
REGULATION OF TYPE I INTERFERON MEDIATED SIGNALING	0.536028657	0.992893407
NEGATIVE REGULATION OF ORGANELLE ASSEMBLY	0.534689037	0.993063238
NEGATIVE REGULATION OF RNA CATABOLIC PROCESS	0.530394212	0.993578279
STEROID HORMONE MEDIATED SIGNALING PATHWAY	0.528505314	0.993808419
COTRANSLATIONAL PROTEIN TARGETING TO MEMBRANE	0.527255086	0.993943591
NUCLEAR ENVELOPE REASSEMBLY	0.525614812	0.994106491
PYRIMIDINE RIBONUCLEOTIDE METABOLIC PROCESS	0.51878506	0.994796216
VESICLE TARGETING TO FROM OR WITHIN GOLGI	0.514658881	0.995147664
ENDONUCLEOLYTIC CLEAVAGE INVOLVED IN RRNA PROCES	0.504153441	0.996018311
CENTRIOLE ASSEMBLY	0.50358057	0.996064755
PROTEIN LOCALIZATION TO CHROMOSOME CENTROMERIC	0.495193109	0.996694866
HISTONE H2A ACETYLATION	0.493662991	0.99680231
MATURATION OF 5 8S RRNA	0.493434714	0.99682172
REGULATION OF HEMATOPOIETIC PROGENITOR CELL DIFFE	0.482564458	0.997447671
ESTABLISHMENT OF MITOCHONDRION LOCALIZATION	0.464792868	0.998314853
PROTEIN TRANSMEMBRANE IMPORT INTO INTRACELLULAR	0.464791499	0.998314853
NEGATIVE REGULATION OF CELL AGING	0.461787935	0.998428536
TERMINATION OF RNA POLYMERASE I TRANSCRIPTION	0.461128903	0.99845973
NUCLEAR TRANSCRIBED MRNA CATABOLIC PROCESS NONS	0.460820084	0.998468741
VENTRICULAR SYSTEM DEVELOPMENT	0.458132962	0.998560936
MRNA CLEAVAGE	0.456864059	0.998602527
RIBOSOMAL SMALL SUBUNIT BIOGENESIS	0.408419538	0.999639541
TRANSLATIONAL ELONGATION	-0.385453875	0.999872448
UBIQUINONE METABOLIC PROCESS	-0.385778273	0.999872448
PROTEIN MONOUBIQUITINATION	-0.429534039	0.999407414
INNER MITOCHONDRIAL MEMBRANE ORGANIZATION	-0.442608593	0.999136366
TERMINATION OF RNA POLYMERASE II TRANSCRIPTION	-0.475578211	0.997964482
NUCLEAR TRANSCRIBED MRNA CATABOLIC PROCESS EXON	-0.479768619	0.997741266
GLYCOPROTEIN CATABOLIC PROCESS	-0.491628711	0.996983926
INTRACELLULAR ESTROGEN RECEPTOR SIGNALING PATHW	-0.492352546	0.996914835
PROTEIN TARGETING TO MEMBRANE	-0.49402961	0.996805884
RESPONSE TO TYPE I INTERFERON	-0.497255318	0.996572039
NUCLEAR MEMBRANE ORGANIZATION	-0.504941859	0.995982111
HISTONE MRNA METABOLIC PROCESS	-0.511553468	0.995408127

VIRAL GENE EXPRESSION	-0.518957687	0.994650786
PROTEIN IMPORT INTO MITOCHONDRIAL MATRIX	-0.525070014	0.994020998
BARBED END ACTIN FILAMENT CAPPING	-0.534232226	0.992907575
PROTEIN PEPTIDYL PROLYL ISOMERIZATION	-0.53446964	0.992883659
SCF DEPENDENT PROTEASOMAL UBIQUITIN DEPENDENT PI	-0.544066874	0.991520447
RRNA METHYLATION	-0.550852871	0.990462828
MITOCHONDRIAL TRANSMEMBRANE TRANSPORT	-0.558038324	0.989378636
SPLICEOSOMAL SNRNP ASSEMBLY	-0.563266935	0.988406051
PROTEIN K48 LINKED UBIQUITINATION	-0.564833807	0.988084514
PROTEIN INSERTION INTO MEMBRANE	-0.580888883	0.984720329
REGULATION OF KERATINOCYTE DIFFERENTIATION	-0.583463999	0.984106485
LIPID IMPORT INTO CELL	-0.583501051	0.984095855
VIRAL BUDDING	-0.588432161	0.982748587
RIBONUCLEOSIDE TRIPHOSPHATE BIOSYNTHETIC PROCES	-0.591220587	0.982025792
EXIT FROM MITOSIS	-0.591733686	0.981890268
DEOXYRIBONUCLEOTIDE METABOLIC PROCESS	-0.594014042	0.981212648
EMBRYONIC HEMOPOIESIS	-0.596783644	0.980410133
NEGATIVE REGULATION OF TELOMERE MAINTENANCE VIA T	-0.599042283	0.979806918
PROTEIN LOCALIZATION TO CELL CELL JUNCTION	-0.600719247	0.979349857
SYNAPTIC VESICLE TRANSPORT	-0.603907265	0.97840916
ENDOPLASMIC RETICULUM TO GOLGI VESICLE MEDIATED T	-0.604282057	0.978292238
RNA MODIFICATION	-0.605855009	0.977752799
NEGATIVE REGULATION OF MRNA CATABOLIC PROCESS	-0.611920528	0.975850148
POSITIVE REGULATION OF MUSCLE CELL APOPTOTIC PROC	-0.613496352	0.975278821
CHAPERONE MEDIATED PROTEIN FOLDING	-0.614087804	0.975060919
FATTY ACYL COA BIOSYNTHETIC PROCESS	-0.615323963	0.974635746
DNA CATABOLIC PROCESS ENDONUCLEOLYTIC	-0.615496342	0.974569313
MULTIVESICULAR BODY SORTING PATHWAY	-0.620876512	0.972746381
NEGATIVE REGULATION OF ACTIN FILAMENT DEPOLYMERIZ	-0.622323688	0.972198971
GLYCOSYL COMPOUND BIOSYNTHETIC PROCESS	-0.624917219	0.971178554
REGULATION OF GENE SILENCING	-0.626156281	0.970686947
CORTICAL ACTIN CYTOSKELETON ORGANIZATION	-0.629028626	0.9696373
CHAPERONE COFACTOR DEPENDENT PROTEIN REFOLDING	-0.629343105	0.969501776
RNA DECAPPING	-0.629834845	0.969270588
MATURATION OF SSU RRNA	-0.631800176	0.96860094
RESPONSE TO TESTOSTERONE	-0.632179403	0.968462759
LYSOSOMAL TRANSPORT	-0.633683629	0.967811712
LATE ENDOSOME TO VACUOLE TRANSPORT VIA MULTIVESI	-0.634735515	0.967341364
GOLGI TO PLASMA MEMBRANE TRANSPORT	-0.635597178	0.966987938
DNA CATABOLIC PROCESS	-0.638116189	0.965855914
REGULATION OF TRANSLATIONAL FIDELITY	-0.638723191	0.965582209
PROTEIN HETEROOLIGOMERIZATION	-0.638852297	0.965515775
POSITIVE REGULATION OF ATPASE ACTIVITY	-0.640272891	0.964917875
REGULATION OF ATPASE ACTIVITY	-0.641476474	0.964375779
POSITIVE REGULATION OF LAMELLIPODIUM ASSEMBLY	-0.644677056	0.962823896
BINDING OF SPERM TO ZONA PELLUCIDA	-0.649464123	0.960504043
POSITIVE REGULATION OF VIRAL TRANSCRIPTION	-0.650341182	0.960054954
PROTEIN K63 LINKED UBIQUITINATION	-0.652933466	0.958843209
NEGATIVE REGULATION OF NOTCH SIGNALING PATHWAY	-0.653816404	0.958386148
CELLULAR MODIFIED AMINO ACID CATABOLIC PROCESS	-0.656830046	0.956900698
GLYCOSYL COMPOUND METABOLIC PROCESS	-0.657655687	0.956467553
NUCLEOSIDE TRIPHOSPHATE BIOSYNTHETIC PROCESS	-0.660379011	0.955043222
POSITIVE REGULATION OF PROTEIN MATURATION	-0.660660768	0.954857208

POSITIVE REGULATION OF TELOMERE MAINTENANCE VIA T	-0.661689542	0.954248679
RIBOSOME ASSEMBLY	-0.66443769	0.952744628
TRANSCRIPTION BY RNA POLYMERASE III	-0.665729689	0.952040434
IRON SULFUR CLUSTER ASSEMBLY	-0.67163964	0.948633732
PROTON TRANSMEMBRANE TRANSPORT	-0.672135984	0.948391914
PURINE NUCLEOBASE METABOLIC PROCESS	-0.673145908	0.947767441
POSITIVE REGULATION OF BONE RESORPTION	-0.674962407	0.946656675
DE NOVO PROTEIN FOLDING	-0.676474905	0.945715979
EMBRYONIC CRANIAL SKELETON MORPHOGENESIS	-0.676772368	0.945519336
DEOXYRIBOSE PHOSPHATE CATABOLIC PROCESS	-0.678912399	0.944211928
PROTEIN DESTABILIZATION	-0.680295278	0.943420042
NEGATIVE REGULATION OF NEURON DIFFERENTIATION	-0.681182037	0.942920463
GOLGI VESICLE TRANSPORT	-0.681967544	0.942442143
PYRIMIDINE NUCLEOSIDE METABOLIC PROCESS	-0.68252104	0.94209669
HOMOLOGOUS CHROMOSOME PAIRING AT MEIOSIS	-0.683678787	0.941320748
NUCLEAR TRANSCRIBED MRNA CATABOLIC PROCESS	-0.684840643	0.940550121
NEUTRAL LIPID CATABOLIC PROCESS	-0.684934747	0.940465087
ATP BIOSYNTHETIC PROCESS	-0.686575125	0.939447328
POSITIVE REGULATION OF DNA BIOSYNTHETIC PROCESS	-0.691600523	0.935809437
REGULATION OF SODIUM ION TRANSMEMBRANE TRANSPOI	-0.692381542	0.935264684
BRANCHED CHAIN AMINO ACID METABOLIC PROCESS	-0.692676321	0.935036153
NEGATIVE REGULATION OF TOR SIGNALING	-0.694054358	0.934090142
SERINE FAMILY AMINO ACID METABOLIC PROCESS	-0.694196067	0.933967905
NEGATIVE REGULATION OF TELOMERE MAINTENANCE	-0.694224598	0.933941331
POSITIVE REGULATION OF DENDRITIC SPINE DEVELOPMEN	-0.69498107	0.933388606
APOPTOTIC MITOCHONDRIAL CHANGES	-0.700925984	0.928857851
TELOMERE CAPPING	-0.703521856	0.926785131
REGULATION OF TELOMERE CAPPING	-0.704060534	0.926365272
B CELL APOPTOTIC PROCESS	-0.704192355	0.926269608
PYRIMIDINE NUCLEOSIDE MONOPHOSPHATE METABOLIC PI	-0.705039365	0.925642477
PROTEIN STABILIZATION	-0.706273419	0.924677865
REGULATION OF DENDRITIC SPINE DEVELOPMENT	-0.708601238	0.922732696
RESPONSE TO MITOCHONDRIAL DEPOLARISATION	-0.711120643	0.920713122
VIRION ASSEMBLY	-0.712998767	0.919169211
MACROMOLECULE DEACYLATION	-0.713702354	0.918600542
NUCLEOBASE METABOLIC PROCESS	-0.716777891	0.915937893
NEGATIVE REGULATION OF ANTIGEN RECEPTOR MEDIATED	-0.719509198	0.913620697
GLUCOSAMINE CONTAINING COMPOUND METABOLIC PROC	-0.720226526	0.912985595
CELL COMMUNICATION INVOLVED IN CARDIAC CONDUCTIO	-0.720468423	0.912762379
TRANSCRIPTION ELONGATION FROM RNA POLYMERASE I P	-0.721778221	0.911590494
REGULATION OF TRANSCRIPTION FROM RNA POLYMERASE	-0.72204318	0.911332733
NEURONAL STEM CELL POPULATION MAINTENANCE	-0.724382673	0.909283928
PROTEIN INSERTION INTO MITOCHONDRIAL MEMBRANE INV	-0.724517554	0.909174977
ENDOSOME TRANSPORT VIA MULTIVESICULAR BODY SORT	-0.725687087	0.908125331
PEPTIDE CATABOLIC PROCESS	-0.726105312	0.907753304
REGULATION OF CHROMATIN ASSEMBLY OR DISASSEMBLY	-0.726277455	0.907599178
LOW DENSITY LIPOPROTEIN PARTICLE CLEARANCE	-0.729672018	0.904492755
ANDROGEN RECEPTOR SIGNALING PATHWAY	-0.730643683	0.903536114
RESPONSE TO ISOQUINOLINE ALKALOID	-0.731270515	0.902940872
REGULATION OF CAMP MEDIATED SIGNALING	-0.731411674	0.902808005
TRANSCRIPTION INITIATION FROM RNA POLYMERASE I PRC	-0.731842756	0.90240409
RNA METHYLATION	-0.734102005	0.900225076
POSITIVE REGULATION OF AXON EXTENSION	-0.734577705	0.899765357

RNA DESTABILIZATION	-0.737500271	0.896855577
PIGMENT GRANULE ORGANIZATION	-0.737758364	0.896629703
PROTEIN POLYUBIQUITINATION	-0.738334735	0.896063691
REGULATION OF LAMELLIPODIUM ASSEMBLY	-0.738724152	0.895659776
POSITIVE REGULATION OF MITOCHONDRIAL FISSION	-0.739359169	0.895088449
REGULATION OF MRNA CATABOLIC PROCESS	-0.739460509	0.895011387
RNA 5 END PROCESSING	-0.74016737	0.89432048
POSITIVE REGULATION OF DNA REPAIR	-0.741987271	0.89251615
PYRIDINE CONTAINING COMPOUND BIOSYNTHETIC PROCES	-0.742169546	0.892290277
RNA PHOSPHODIESTER BOND HYDROLYSIS ENDONUCLEOL	-0.743315705	0.891144966
MULTIVESICULAR BODY ORGANIZATION	-0.743464847	0.891006784
REGULATION OF FILOPODIUM ASSEMBLY	-0.743992576	0.890424828
TRNA PROCESSING	-0.746464577	0.887794067
CARDIOLIPIN METABOLIC PROCESS	-0.746929665	0.887321062
REGULATION OF SYNAPTIC TRANSMISSION GABAERGIC	-0.74785399	0.886388337
CRANIAL SKELETAL SYSTEM DEVELOPMENT	-0.747969331	0.886255471
MITOCHONDRIAL TRANSPORT	-0.748032786	0.886183723
MODULATION BY VIRUS OF HOST PROCESS	-0.748959717	0.88518988
VASCULAR ASSOCIATED SMOOTH MUSCLE CELL MIGRATIO	-0.74914495	0.884971978
POSITIVE REGULATION OF PROTEIN LOCALIZATION TO MEM	-0.749781989	0.884331561
ESTABLISHMENT OF PROTEIN LOCALIZATION TO MITOCHON	-0.750600818	0.883515759
REGULATION OF VESICLE FUSION	-0.75148481	0.88254849
CHONDROITIN SULFATE PROTEOGLYCAN BIOSYNTHETIC PI	-0.75234221	0.881605136
LIPID DROPLET ORGANIZATION	-0.754950013	0.878713957
CEREBELLAR CORTEX DEVELOPMENT	-0.757172152	0.876351587
SPLEEN DEVELOPMENT	-0.758346012	0.875187674
REGULATION OF MITOTIC SPINDLE ASSEMBLY	-0.760327724	0.872944884
NEGATIVE REGULATION OF TRANSFORMING GROWTH FACT	-0.760672078	0.872583487
RIBOSOMAL LARGE SUBUNIT ASSEMBLY	-0.760810332	0.872432019
VIRAL BUDDING VIA HOST ESCRT COMPLEX	-0.760874511	0.872389501
NEGATIVE REGULATION OF CYCLIN DEPENDENT PROTEIN K	-0.762006592	0.871079436
ADIPOSE TISSUE DEVELOPMENT	-0.762110425	0.870957198
ENDOPLASMIC RETICULUM TUBULAR NETWORK ORGANIZA	-0.763986885	0.868807415
CELLULAR AMINO ACID CATABOLIC PROCESS	-0.766318336	0.86589232
POSITIVE REGULATION OF RESPONSE TO DNA DAMAGE ST	-0.766369322	0.865836515
PROTEIN QUALITY CONTROL FOR MISFOLDED OR INCOMPL	-0.766517588	0.865687705
POSITIVE REGULATION OF MITOCHONDRION ORGANIZATIO	-0.766864794	0.865262531
NUCLEAR ENVELOPE ORGANIZATION	-0.767075468	0.864980854
TELOMERASE RNA LOCALIZATION	-0.768919984	0.862812469
POSITIVE REGULATION OF G1 S TRANSITION OF MITOTIC CI	-0.770158886	0.861271215
ATTACHMENT OF SPINDLE MICROTUBULES TO KINETOCHO	-0.772679013	0.858215281
ESTABLISHMENT OF PROTEIN LOCALIZATION TO MEMBRAN	-0.773526013	0.85720018
MRNA TRANSPORT	-0.774943093	0.855340046
REGULATION OF DOUBLE STRAND BREAK REPAIR VIA HOM	-0.77745847	0.851941315
PYRIMIDINE CONTAINING COMPOUND CATABOLIC PROCESS	-0.778542472	0.850429292
RNA CATABOLIC PROCESS	-0.778620825	0.850333628
AXO DENDRITIC TRANSPORT	-0.779319233	0.849408876
POSITIVE REGULATION OF MULTICELLULAR ORGANISM GR	-0.780631828	0.847724126
PYRUVATE METABOLIC PROCESS	-0.781491474	0.84654427
POSITIVE REGULATION OF DOUBLE STRAND BREAK REPAIR	-0.782512513	0.845117281
RNA CAPPING	-0.783987531	0.843209316
CILIARY BASAL BODY PLASMA MEMBRANE DOCKING	-0.784417031	0.842683163
REGULATION OF DNA TEMPLATED TRANSCRIPTION IN RESP	-0.784965634	0.841965683

INTESTINAL ABSORPTION	-0.785801777	0.840828344
MAINTENANCE OF PROTEIN LOCALIZATION IN ORGANELLE	-0.786764771	0.839438558
PROTEIN TARGETING TO MITOCHONDRION	-0.786883392	0.839303034
NUCLEOSIDE MONOPHOSPHATE BIOSYNTHETIC PROCESS	-0.787429073	0.838521778
NEGATIVE REGULATION OF MRNA SPLICING VIA SPLICEOSO	-0.787643268	0.838232129
REGULATION OF CELL PROJECTION ASSEMBLY	-0.788084334	0.837698005
MATURATION OF SSU RRNA FROM TRICISTRONIC RRNA TRA	-0.789779843	0.835468501
ERAD PATHWAY	-0.790046898	0.835120391
TRNA METHYLATION	-0.790552989	0.834400253
NEGATIVE REGULATION OF TYPE I INTERFERON PRODUCTI	-0.790624051	0.834307246
EPITHELIAL CELL MORPHOGENESIS	-0.791533348	0.833005153
REGULATION OF DNA BIOSYNTHETIC PROCESS	-0.792363695	0.831902359
UBIQUITIN DEPENDENT ERAD PATHWAY	-0.79305909	0.830927117
POSITIVE REGULATION OF MUSCLE HYPERTROPHY	-0.793139017	0.830823481
POSTTRANSCRIPTIONAL REGULATION OF GENE EXPRESSIO	-0.794476683	0.82900055
SNRNA METABOLIC PROCESS	-0.794918685	0.828389363
POSITIVE REGULATION OF RESPONSE TO ENDOPLASMIC RI	-0.795110675	0.828147546
NEGATIVE REGULATION OF CELL CYCLE G2 M PHASE TRAN	-0.795203578	0.828017336
REGULATION OF CALCINEURIN MEDIATED SIGNALING	-0.795503235	0.827576219
REGULATION OF PHOSPHATASE ACTIVITY	-0.79616959	0.826651467
POSITIVE REGULATION OF CELL GROWTH	-0.796398248	0.826287412
HISTONE MONOUBIQUITINATION	-0.796613762	0.825971189
G0 TO G1 TRANSITION	-0.796933381	0.825527414
ACTIVATION OF MAPKK ACTIVITY	-0.797334128	0.824929514
MITOCHONDRIAL CALCIUM ION HOMEOSTASIS	-0.79749681	0.824716927
POSITIVE REGULATION OF PROTEIN LOCALIZATION TO NUC	-0.798520137	0.823250079
RESPONSE TO ELECTRICAL STIMULUS	-0.799277539	0.822181831
HETEROPHILIC CELL CELL ADHESION VIA PLASMA MEMBRA	-0.800975237	0.819652049
POSITIVE REGULATION OF DENDRITE MORPHOGENESIS	-0.801379218	0.819086037
REGULATION OF PROTEIN SUMOYLATION	-0.802002787	0.818262263
ENDOPLASMIC RETICULUM TO CYTOSOL TRANSPORT	-0.802264989	0.817898208
POSITIVE REGULATION OF ORGANELLE ASSEMBLY	-0.802936417	0.816859191
PROTEIN DEMANNOSYLATION	-0.803394239	0.816189542
REGULATION OF PHOSPHOPROTEIN PHOSPHATASE ACTIVI	-0.803517524	0.816011501
METENCEPHALON DEVELOPMENT	-0.804141109	0.815070805
REGULATION OF DENDRITE MORPHOGENESIS	-0.804304406	0.81482633
AXONEME ASSEMBLY	-0.804614749	0.814379898
ER NUCLEUS SIGNALING PATHWAY	-0.807826958	0.809740192
PROTEIN COMPLEX OLIGOMERIZATION	-0.809769562	0.806883558
REGULATION OF CYCLASE ACTIVITY	-0.811703598	0.803917973
PHOSPHATE ION TRANSPORT	-0.811788602	0.803793079
PROTEIN HYDROXYLATION	-0.811896762	0.803646925
TRIGLYCERIDE BIOSYNTHETIC PROCESS	-0.812404892	0.802876298
REGULATION OF ACTION POTENTIAL	-0.814365863	0.799862882
OVARIAN FOLLICLE DEVELOPMENT	-0.814709768	0.799360645
INTRINSIC APOPTOTIC SIGNALING PATHWAY BY P53 CLASS	-0.815225508	0.798512956
VESICLE MEDIATED TRANSPORT TO THE PLASMA MEMBRAN	-0.815910328	0.797487225
PROTEIN LOCALIZATION TO MITOCHONDRION	-0.817464322	0.795114226
MODIFICATION OF SYNAPTIC STRUCTURE	-0.817633248	0.79484052
REGULATION OF VOLTAGE GATED CALCIUM CHANNEL ACTI	-0.818764961	0.793081365
POSITIVE REGULATION OF PLASMA MEMBRANE BOUNDED C	-0.819293171	0.792316053
EPIBOLY	-0.819407753	0.792156613
CYTOPLASMIC PATTERN RECOGNITION RECEPTOR SIGNAL	-0.819494431	0.792015774

HISTONE DEACETYLATION	-0.819585226	0.79188025
CELLULAR PROTEIN COMPLEX DISASSEMBLY	-0.820180678	0.790982071
INTRA GOLGI VESICLE MEDIATED TRANSPORT	-0.822026556	0.787942081
PEPTIDYL PROLINE MODIFICATION	-0.823667918	0.785260831
HISTONE H3 DEACETYLATION	-0.82416217	0.784476917
NEGATIVE REGULATION OF RNA SPLICING	-0.825148024	0.782967551
OUTFLOW TRACT SEPTUM MORPHOGENESIS	-0.8252067	0.782871887
VESICLE DOCKING INVOLVED IN EXOCYTOSIS	-0.826204731	0.781234969
NUCLEOBASE CONTAINING COMPOUND TRANSPORT	-0.826479897	0.780793852
UBIQUITIN DEPENDENT PROTEIN CATABOLIC PROCESS VIA	-0.82672174	0.780344763
CORONARY VASCULATURE DEVELOPMENT	-0.826860163	0.780129518
MRNA MODIFICATION	-0.827139805	0.779608681
REGULATION OF SPINDLE ASSEMBLY	-0.832237502	0.771299197
POSITIVE REGULATION OF AMYLOID PRECURSOR PROTEIN	-0.83455941	0.767592216
NEGATIVE REGULATION OF RESPONSE TO BIOTIC STIMULU	-0.835366261	0.766186486
POSITIVE REGULATION OF PROTEIN CONTAINING COMPLEX	-0.836533863	0.764201458
NUCLEOTIDE PHOSPHORYLATION	-0.836556013	0.764153626
NUCLEOBASE CONTAINING SMALL MOLECULE INTERCONVE	-0.836883725	0.763598243
LACTATION	-0.837821336	0.762062304
REGULATION OF PROTEIN POLYUBIQUITINATION	-0.838846089	0.760467903
POSITIVE REGULATION OF CELLULAR AMIDE METABOLIC PI	-0.83971868	0.759009027
POSITIVE REGULATION OF DNA METABOLIC PROCESS	-0.840086138	0.758368609
NUCLEOSIDE MONOPHOSPHATE METABOLIC PROCESS	-0.840160151	0.758246372
MRNA EXPORT FROM NUCLEUS	-0.841365931	0.756261343
CELLULAR RESPONSE TO OXYGEN LEVELS	-0.843493925	0.752554362
POSITIVE REGULATION OF VIRAL LIFE CYCLE	-0.844282295	0.751334646
DENDRITE DEVELOPMENT	-0.844475313	0.751044997
GLYCEROLIPID CATABOLIC PROCESS	-0.84450685	0.750989193
APOPTOTIC DNA FRAGMENTATION	-0.844967414	0.750152132
NUCLEOTIDE EXCISION REPAIR DNA DAMAGE RECOGNITIOI	-0.845859057	0.748597592
AMINO ACID IMPORT	-0.84586901	0.748586963
POSITIVE REGULATION OF PROTEIN MODIFICATION BY SMA	-0.848272624	0.744492011
ENDOSOME ORGANIZATION	-0.849841621	0.741832019
MITOTIC DNA REPLICATION	-0.850167249	0.741250063
POSITIVE REGULATION OF TRANSCRIPTION FROM RNA POL	-0.850223309	0.741117196
PHOTOTRANSDUCTION	-0.850373714	0.740878036
CELLULAR CARBOHYDRATE CATABOLIC PROCESS	-0.850522305	0.740527268
EMBRYONIC PLACENTA DEVELOPMENT	-0.852250881	0.737458047
CELLULAR PROTEIN CONTAINING COMPLEX LOCALIZATION	-0.852449817	0.737115251
REGULATION OF GLYCOPROTEIN METABOLIC PROCESS	-0.852730748	0.736642246
POSITIVE REGULATION OF CELL CYCLE G1 S PHASE TRANS	-0.853134734	0.735977912
PROTEIN EXIT FROM ENDOPLASMIC RETICULUM	-0.855011087	0.732534007
NEGATIVE REGULATION OF FIBROBLAST PROLIFERATION	-0.855734063	0.731231914
RESPONSE TO COPPER ION	-0.856741525	0.729345206
MODIFIED AMINO ACID TRANSPORT	-0.856862208	0.729076816
EMBRYONIC SKELETAL SYSTEM MORPHOGENESIS	-0.857477824	0.728024511
NEGATIVE REGULATION OF I KAPPAB KINASE NF KAPPAB SI	-0.859514111	0.72439725
REGULATION OF PROTEIN EXIT FROM ENDOPLASMIC RETIC	-0.861300551	0.721144673
CELLULAR AMINO ACID METABOLIC PROCESS	-0.861657465	0.720482997
ARF PROTEIN SIGNAL TRANSDUCTION	-0.863337871	0.717440349
RRNA MODIFICATION	-0.863360888	0.717403147
VIRAL GENOME REPLICATION	-0.863893136	0.716380073
SNRNA TRANSCRIPTION	-0.86404243	0.716093081

POSITIVE REGULATION OF MITOTIC CELL CYCLE	-0.865151338	0.71407085
SYNAPTIC MEMBRANE ADHESION	-0.865196588	0.713985815
REGULATION OF DNA METABOLIC PROCESS	-0.866699511	0.711285964
NEGATIVE REGULATION OF CELLULAR CATABOLIC PROCES	-0.867146658	0.710472819
REGULATION OF MEGAKARYOCYTE DIFFERENTIATION	-0.868098021	0.708705692
MATERNAL PROCESS INVOLVED IN FEMALE PREGNANCY	-0.870160655	0.704812698
RESPONSE TO MUSCLE STRETCH	-0.870380271	0.704424727
PYRIMIDINE RIBONUCLEOTIDE BIOSYNTHETIC PROCESS	-0.87192493	0.701483058
DNA REPLICATION INITIATION	-0.872268965	0.700837326
VIRAL RELEASE FROM HOST CELL	-0.873741897	0.697906286
REGULATION OF GLUCOSE METABOLIC PROCESS	-0.874080887	0.697241953
REGULATION OF DNA REPAIR	-0.874820793	0.695833566
TRNA METABOLIC PROCESS	-0.876456065	0.69279889
NUCLEAR TRANSCRIBED MRNA CATABOLIC PROCESS DEAD	-0.876809441	0.692123927
POSITIVE REGULATION OF POSTTRANSCRIPTIONAL GENE S	-0.877633034	0.690569387
NUCLEOSIDE DIPHOSPHATE METABOLIC PROCESS	-0.877825948	0.690245192
HISTONE EXCHANGE	-0.879449513	0.687258349
REGULATION OF MEMBRANE PERMEABILITY	-0.881153867	0.684003114
REGULATION OF TELOMERASE ACTIVITY	-0.881273991	0.683763954
POSITIVE REGULATION OF CELL MATRIX ADHESION	-0.881541534	0.683251089
PROTEIN DEGLYCOSYLATION	-0.882175101	0.682071232
GOLGI TO PLASMA MEMBRANE PROTEIN TRANSPORT	-0.882317158	0.68178424
RNA LOCALIZATION	-0.88323442	0.680011799
REGULATION OF PROTEIN DEPHOSPHORYLATION	-0.883361001	0.67979124
RETROGRADE TRANSPORT ENDOSOME TO PLASMA MEMBR	-0.883835368	0.678879774
FERTILIZATION	-0.884359812	0.678021455
PROTEIN LOCALIZATION TO CYTOSKELETON	-0.884452077	0.677835442
INTRACELLULAR RECEPTOR SIGNALING PATHWAY	-0.885624549	0.67570426
CARDIAC SEPTUM DEVELOPMENT	-0.885763046	0.67542524
POSITIVE REGULATION OF CELL MIGRATION INVOLVED IN S	-0.885807175	0.675340205
CELLULAR SENESCENCE	-0.888800149	0.669738545
STRESS GRANULE ASSEMBLY	-0.889647039	0.668061767
NEGATIVE REGULATION OF ORGANELLE ORGANIZATION	-0.890037116	0.667352259
NEGATIVE REGULATION OF ANION TRANSMEMBRANE TRAN	-0.890520371	0.66645408
VESICLE CYTOSKELETAL TRAFFICKING	-0.891369487	0.664846393
ESTABLISHMENT OF RNA LOCALIZATION	-0.891628604	0.664365415
CELLULAR RESPONSE TO GLUCOSE STARVATION	-0.892644136	0.662311296
CARBOHYDRATE CATABOLIC PROCESS	-0.893718346	0.660257177
STEM CELL DIVISION	-0.89611634	0.65543943
SECRETION BY TISSUE	-0.89643732	0.65476181
NAD METABOLIC PROCESS	-0.896603376	0.654450902
REGULATION OF CELL DIVISION	-0.896700745	0.654288804
CELLULAR GLUCAN METABOLIC PROCESS	-0.896925189	0.653802512
MODULATION BY HOST OF VIRAL PROCESS	-0.898686368	0.650366579
PYRIDINE CONTAINING COMPOUND METABOLIC PROCESS	-0.899090633	0.649603924
MAINTENANCE OF PROTEIN LOCATION IN NUCLEUS	-0.89957708	0.648684487
MITOCHONDRIAL MEMBRANE ORGANIZATION	-0.899627181	0.648583508
NAD BIOSYNTHETIC PROCESS	-0.899979589	0.647871343
REGULATION OF SYSTEMIC ARTERIAL BLOOD PRESSURE B	-0.900410437	0.646909388
CARBOHYDRATE DERIVATIVE TRANSPORT	-0.900420548	0.646898758
INOSITOL PHOSPHATE CATABOLIC PROCESS	-0.900444419	0.646842954
POSITIVE REGULATION OF TELOMERE MAINTENANCE	-0.900640186	0.646433725
POSTSYNAPTIC SPECIALIZATION ORGANIZATION	-0.900960804	0.645777363

PRODUCTION OF SMALL RNA INVOLVED IN GENE SILENCINC	-0.901158942	0.645368134
PHOSPHORYLATED CARBOHYDRATE DEPHOSPHORYLATIO	-0.901778199	0.644233452
IRE1 MEDIATED UNFOLDED PROTEIN RESPONSE	-0.902823483	0.642229822
REGULATION OF POTASSIUM ION TRANSPORT	-0.904046554	0.639931228
RNA EXPORT FROM NUCLEUS	-0.904470347	0.639070252
EPHRIN RECEPTOR SIGNALING PATHWAY	-0.904583569	0.63882312
CHROMATIN REMODELING AT CENTROMERE	-0.904733899	0.638560044
REGULATION OF ENDOPLASMIC RETICULUM STRESS INDUC	-0.904868132	0.638281024
REGULATION OF ENDOPLASMIC RETICULUM UNFOLDED PR	-0.905893285	0.636311939
NUCLEIC ACID PHOSPHODIESTER BOND HYDROLYSIS	-0.906082539	0.635966486
CELLULAR RESPONSE TO CARBOHYDRATE STIMULUS	-0.910501219	0.627292947
HIPPOCAMPUS DEVELOPMENT	-0.91097944	0.626338964
CELLULAR RESPONSE TO ABIOTIC STIMULUS	-0.911246947	0.625812812
NEGATIVE REGULATION OF MUSCLE CONTRACTION	-0.91138123	0.625563023
SULFUR COMPOUND TRANSPORT	-0.911460578	0.625430156
HEPARAN SULFATE PROTEOGLYCAN METABOLIC PROCESS	-0.911785671	0.624720648
POSITIVE REGULATION OF SPROUTING ANGIOGENESIS	-0.912250526	0.623795895
CELLULAR RESPONSE TO STARVATION	-0.913030888	0.622299816
DOSAGE COMPENSATION	-0.913341799	0.621635483
RIBOSOME BIOGENESIS	-0.9137284	0.620832968
MONOVALENT INORGANIC CATION HOMEOSTASIS	-0.914056736	0.620195208
CARBOHYDRATE HOMEOSTASIS	-0.916023739	0.616371304
VESICLE MEDIATED TRANSPORT BETWEEN ENDOSOMAL CO	-0.916456384	0.615475782
LIMBIC SYSTEM DEVELOPMENT	-0.917462284	0.613456208
NUCLEAR TRANSPORT	-0.917556986	0.613328656
SYNAPTIC TRANSMISSION GABAERGIC	-0.917788522	0.612879567
POSITIVE REGULATION OF TRANSCRIPTION REGULATORY F	-0.918180955	0.612039849
INNER EAR RECEPTOR CELL STEREOCILIUM ORGANIZATION	-0.918270635	0.611851179
TRANSLATIONAL INITIATION	-0.919351079	0.609658878
CELL FATE COMMITMENT INVOLVED IN FORMATION OF PRIM	-0.922022255	0.604041274
POSITIVE REGULATION OF PROTEIN AUTOPHOSPHORYLATI	-0.924549896	0.5990508
REGULATION OF DEPHOSPHORYLATION	-0.92479445	0.598577795
REGULATION OF VIRAL LIFE CYCLE	-0.925140373	0.597852343
MALE MEIOTIC NUCLEAR DIVISION	-0.925544422	0.596962136
GENETIC IMPRINTING	-0.925599749	0.596839898
PIGMENTATION	-0.92571307	0.59661934
CELLULAR IRON ION HOMEOSTASIS	-0.928201859	0.591519915
NEURAL TUBE DEVELOPMENT	-0.928791889	0.590305514
REGULATION OF AUTOPHAGY OF MITOCHONDRION	-0.92916666	0.589588033
POSITIVE REGULATION OF MICROTUBULE POLYMERIZATION	-0.929200928	0.589510971
RESPONSE TO STARVATION	-0.930100244	0.58775713
HISTONE H2A UBIQUITINATION	-0.930212589	0.58751797
POSITIVE REGULATION OF PROTEIN ACETYLATION	-0.9303087	0.587326642
NEGATIVE REGULATION OF PEPTIDYL TYROSINE PHOSPHO	-0.930345254	0.587260209
BETA CATENIN DESTRUCTION COMPLEX DISASSEMBLY	-0.930381873	0.587172517
MRNA METHYLATION	-0.930831151	0.586303569
TRIGLYCERIDE METABOLIC PROCESS	-0.931616292	0.584701196
NUCLEUS ORGANIZATION	-0.931703467	0.584525812
POSITIVE REGULATION OF CATABOLIC PROCESS	-0.933185847	0.58155757
NEGATIVE REGULATION OF NUCLEAR DIVISION	-0.934785127	0.578243874
NEGATIVE REGULATION OF MITOTIC CELL CYCLE	-0.934874548	0.578052546
NEUTRAL LIPID METABOLIC PROCESS	-0.935074063	0.577645974
ENDOPLASMIC RETICULUM MANNOSE TRIMMING	-0.936847441	0.574148922

REGULATION OF TELOMERE MAINTENANCE VIA TELOMERE	-0.937239972	0.573314519
REGULATION OF CELLULAR AMIDE METABOLIC PROCESS	-0.937554218	0.572716619
NEURON CELLULAR HOMEOSTASIS	-0.938365826	0.571071729
REGULATION OF EARLY ENDOSOME TO LATE ENDOSOME T	-0.939625927	0.56849943
RESPONSE TO RADIATION	-0.939686412	0.568355934
PROTEIN HOMOOLIGOMERIZATION	-0.94010948	0.567404608
NEGATIVE REGULATION OF CELL CYCLE PROCESS	-0.940664103	0.566365591
MALE GAMETE GENERATION	-0.94099598	0.565695943
CARDIAC VENTRICLE DEVELOPMENT	-0.941223176	0.565225594
MEIOSIS I CELL CYCLE PROCESS	-0.942575395	0.562395534
REGULATION OF PROTEASOMAL PROTEIN CATABOLIC PRO	-0.943112339	0.561290083
ESTABLISHMENT OF PROTEIN LOCALIZATION TO PLASMA M	-0.945237648	0.557030376
POSITIVE REGULATION OF TRANSLATIONAL INITIATION	-0.945473211	0.556530797
ALPHA AMINO ACID METABOLIC PROCESS	-0.946181581	0.555196815
OUTFLOW TRACT MORPHOGENESIS	-0.946962842	0.553575842
CELLULAR RESPONSE TO IONIZING RADIATION	-0.947017841	0.553474863
CELLULAR RESPONSE TO INSULIN STIMULUS	-0.947040335	0.553424374
INTERSTRAND CROSS LINK REPAIR	-0.94706874	0.553360598
REGULATION OF PROTEIN DEPOLYMERIZATION	-0.947558354	0.552348153
CHEMICAL SYNAPTIC TRANSMISSION POSTSYNAPTIC	-0.948690221	0.550022986
PROTEASOMAL PROTEIN CATABOLIC PROCESS	-0.949191583	0.54899194
EPIDERMIS DEVELOPMENT	-0.949599775	0.548255859
ANATOMICAL STRUCTURE HOMEOSTASIS	-0.95161061	0.544229998
RESPONSE TO COCAINE	-0.951975803	0.543485944
ALPHA AMINO ACID CATABOLIC PROCESS	-0.953487183	0.540560219
CYTOPLASMIC SEQUESTERING OF PROTEIN	-0.954149226	0.539207636
REGULATION OF CARDIAC MUSCLE CELL PROLIFERATION	-0.955249114	0.536988762
PIGMENT METABOLIC PROCESS	-0.955401885	0.53666191
PHOSPHATIDYLCHOLINE METABOLIC PROCESS	-0.955853437	0.535652123
NUCLEAR EXPORT	-0.95608425	0.535245551
REGULATION OF LYASE ACTIVITY	-0.958147991	0.531211718
REGULATION OF NUCLEAR DIVISION	-0.958320183	0.530866264
INTRINSIC APOPTOTIC SIGNALING PATHWAY	-0.959174381	0.529131025
POSITIVE REGULATION OF TRANSLATION	-0.959238447	0.528984872
NEGATIVE REGULATION OF PROTEIN LOCALIZATION TO NU	-0.959289022	0.528883893
GLUCOSE METABOLIC PROCESS	-0.959512367	0.528442776
REGULATION OF PROTEIN CATABOLIC PROCESS	-0.959969108	0.527456905
POLYOL CATABOLIC PROCESS	-0.960213881	0.526957326
CELLULAR RESPONSE TO PEPTIDE HORMONE STIMULUS	-0.960221249	0.526941382
TRANSFORMING GROWTH FACTOR BETA RECEPTOR SIGNA	-0.960497101	0.52641523
SIGNAL TRANSDUCTION IN RESPONSE TO DNA DAMAGE	-0.960763129	0.525910336
PHOSPHATIDYLGLYCEROL METABOLIC PROCESS	-0.960943233	0.525588799
REGULATION OF GENERATION OF PRECURSOR METABOLIT	-0.961340148	0.524791599
POLYSACCHARIDE METABOLIC PROCESS	-0.961845737	0.523797756
CELLULAR RESPONSE TO ESTRADIOL STIMULUS	-0.962083923	0.523353981
PYRIMIDINE DEOXYRIBONUCLEOTIDE METABOLIC PROCESS	-0.962759506	0.52196951
PYRIMIDINE NUCLEOTIDE METABOLIC PROCESS	-0.963509579	0.520399025
EPITHELIAL TUBE FORMATION	-0.966683544	0.514024081
REGULATION OF INTRACELLULAR TRANSPORT	-0.968222152	0.511063811
SPERMATID DIFFERENTIATION	-0.968229388	0.511058496
TRANSITION METAL ION TRANSPORT	-0.968705363	0.510069968
POSITIVE REGULATION OF TELOMERASE ACTIVITY	-0.968783573	0.509921157
REGULATION OF PH	-0.968894654	0.509684654

ORGANELLE DISASSEMBLY	-0.969787382	0.507890954
NEGATIVE REGULATION OF CELL CYCLE	-0.970922942	0.505605646
MAMMARY GLAND EPITHELIAL CELL PROLIFERATION	-0.971432289	0.50454537
CELL JUNCTION MAINTENANCE	-0.971747105	0.503904952
HEPARAN SULFATE PROTEOGLYCAN BIOSYNTHETIC PROCE	-0.972775378	0.501840204
CIRCADIAN RHYTHM	-0.973020838	0.501367198
RESPONSE TO PH	-0.973789669	0.499895035
POSITIVE REGULATION OF GLYCOLYTIC PROCESS	-0.974173642	0.499150982
VIRAL RNA GENOME REPLICATION	-0.97624483	0.495162323
AUTOPHAGOSOME MATURATION	-0.976393965	0.494912534
NEGATIVE REGULATION OF PROTEIN CONTAINING COMPLE	-0.978319865	0.491250727
NEGATIVE REGULATION OF CALCIUM ION TRANSPORT	-0.978927982	0.490198423
CELLULAR RESPONSE TO HYDROGEN PEROXIDE	-0.979543538	0.489023881
IRON ION HOMEOSTASIS	-0.98164005	0.48483858
REGULATION OF MITOTIC SISTER CHROMATID SEGREGATIO	-0.983400956	0.481575374
MESODERMAL CELL DIFFERENTIATION	-0.98360667	0.481126284
DETECTION OF STIMULUS INVOLVED IN SENSORY PERCEPT	-0.983999564	0.480321112
MULTICELLULAR ORGANISM REPRODUCTION	-0.984131717	0.480028806
REGULATION OF GLUCONEOGENESIS	-0.984616415	0.479045592
PLACENTA DEVELOPMENT	-0.984693398	0.478931327
REGULATION OF CELL CYCLE PHASE TRANSITION	-0.985338926	0.477764757
RESPONSE TO IONIZING RADIATION	-0.986630899	0.475160569
REGULATION OF PROTEIN BINDING	-0.986757001	0.474913437
POSITIVE REGULATION OF INTRACELLULAR TRANSPORT	-0.988232593	0.471966454
REGULATION OF PROTEIN LOCALIZATION TO NUCLEUS	-0.988420818	0.471610371
DRUG METABOLIC PROCESS	-0.988452193	0.471557224
HISTONE H3 K9 METHYLATION	-0.988655917	0.471187855
RETINOL METABOLIC PROCESS	-0.989892523	0.468868002
PROTEIN PHOSPHOPANTETHEINYLATION	-0.990907537	0.466861715
REGULATION OF ESTABLISHMENT OF PROTEIN LOCALIZATI	-0.993067559	0.462641868
SKIN EPIDERMIS DEVELOPMENT	-0.99343473	0.461924388
ERBB2 SIGNALING PATHWAY	-0.993828496	0.461230824
BONE CELL DEVELOPMENT	-0.994111055	0.460675441
CELL DEATH IN RESPONSE TO HYDROGEN PEROXIDE	-0.994635965	0.459710829
RRNA METABOLIC PROCESS	-0.995157906	0.458780762
DNA DEMETHYLATION	-0.995201412	0.458679783
CELL CYCLE G1 S PHASE TRANSITION	-0.996765944	0.455645108
AUTOPHAGY OF MITOCHONDRION	-0.997985657	0.453311968
GLUCOSE IMPORT	-0.999134649	0.451119668
RECEPTOR CATABOLIC PROCESS	-0.999514553	0.450404845
NEGATIVE REGULATION OF CELL CYCLE PHASE TRANSITIO	-0.99982818	0.449767085
REGULATION OF DOUBLE STRAND BREAK REPAIR	-1.001061454	0.447410029
NEGATIVE REGULATION OF MRNA METABOLIC PROCESS	-1.003028076	0.443865146
RNA POLYADENYLATION	-1.00426565	0.441590468
CYCLIC NUCLEOTIDE MEDIATED SIGNALING	-1.004920582	0.440461101
PRIMARY NEURAL TUBE FORMATION	-1.004980336	0.440341521
CHROMOSOME LOCALIZATION	-1.005851156	0.438585023
PROTEIN LOCALIZATION TO NUCLEUS	-1.005912938	0.438499988
LABYRINTHINE LAYER DEVELOPMENT	-1.007024401	0.436435239
PROTEOGLYCAN METABOLIC PROCESS	-1.007297238	0.435970206
DETECTION OF MECHANICAL STIMULUS	-1.007446827	0.435691186
REGULATION OF GLUCAN BIOSYNTHETIC PROCESS	-1.009753227	0.43142085
SEXUAL REPRODUCTION	-1.009765956	0.431394277

CHAPERONE MEDIATED PROTEIN COMPLEX ASSEMBLY	-1.010515058	0.42998589
NEGATIVE REGULATION OF RECEPTOR MEDIATED ENDOCY	-1.010545764	0.429911484
SODIUM ION HOMEOSTASIS	-1.01123222	0.428606733
IRON ION TRANSPORT	-1.011826008	0.427546457
CELL AGING	-1.011864338	0.427487995
METANEPHROS MORPHOGENESIS	-1.01244003	0.426401146
STEROL TRANSPORT	-1.013476823	0.424410803
NCRNA PROCESSING	-1.013591535	0.424192901
POSITIVE REGULATION OF AUTOPHAGY	-1.013618073	0.42413444
ANTEROGRADE AXONAL TRANSPORT	-1.014505927	0.422577242
POSITIVE REGULATION OF MEMBRANE PERMEABILITY	-1.015080446	0.421463819
MONOSACCHARIDE METABOLIC PROCESS	-1.017544974	0.417042015
PHENOL CONTAINING COMPOUND BIOSYNTHETIC PROCESS	-1.017692035	0.416816142
CELLULAR RESPONSE TO STEROID HORMONE STIMULUS	-1.018058148	0.41615978
EPIDERMAL CELL DIFFERENTIATION	-1.019257719	0.413964822
POSITIVE REGULATION OF CYTOKINESIS	-1.019731838	0.413037413
TRANSEPITHELIAL TRANSPORT	-1.020915331	0.411012524
NEGATIVE REGULATION OF PROTEIN DEPHOSPHORYLATIO	-1.020987537	0.410879657
MITOTIC G2 M TRANSITION CHECKPOINT	-1.021283372	0.410353505
XENOBIOTIC TRANSPORT	-1.022533977	0.408240925
DNA RECOMBINATION	-1.023275629	0.407013236
RESPONSE TO LIGHT STIMULUS	-1.02364611	0.406277155
CELLULAR COMPONENT DISASSEMBLY	-1.024774325	0.404223035
NUCLEOTIDE SUGAR BIOSYNTHETIC PROCESS	-1.025163362	0.403553387
REGULATION OF MICROTUBULE POLYMERIZATION	-1.025169287	0.403540101
REGULATION OF BIOLOGICAL PROCESS INVOLVED IN SYMB	-1.025542734	0.402904998
RESPONSE TO PEPTIDE HORMONE	-1.025698199	0.402631292
CELLULAR RESPONSE TO RADIATION	-1.025997554	0.402102483
POSITIVE REGULATION OF GLYCOPROTEIN METABOLIC PRO	-1.026532865	0.401127241
RESPONSE TO MONOSACCHARIDE	-1.027306684	0.399835777
CENTROSOME DUPLICATION	-1.028628923	0.397521239
EXCITATORY SYNAPSE ASSEMBLY	-1.029206707	0.396553969
MATURATION OF LSU RRNA	-1.030212783	0.394701807
AMYLOID BETA METABOLIC PROCESS	-1.031700373	0.392132165
POSITIVE REGULATION OF PEPTIDYL LYSINE ACETYLATION	-1.032617012	0.390620142
INORGANIC ANION TRANSMEMBRANE TRANSPORT	-1.032690398	0.390476646
REGULATION OF CELL CYCLE G1 S PHASE TRANSITION	-1.032958394	0.390072731
REGULATION OF TRANSCRIPTION BY RNA POLYMERASE I	-1.033075494	0.389886718
CENTROMERE COMPLEX ASSEMBLY	-1.033114699	0.389812312
RESPONSE TO ENDOPLASMIC RETICULUM STRESS	-1.033200285	0.38969539
PYRIMIDINE CONTAINING COMPOUND METABOLIC PROCESS	-1.03452879	0.387516376
ANION TRANSMEMBRANE TRANSPORT	-1.034921203	0.386772322
NEUTRAL AMINO ACID TRANSPORT	-1.035086708	0.386519876
CHROMOSOME SEPARATION	-1.035533121	0.385717361
BODY FLUID SECRETION	-1.035617186	0.385581837
ESTABLISHMENT OR MAINTENANCE OF MONOPOLAR CELL I	-1.035864806	0.385177922
BLASTOCYST FORMATION	-1.037294629	0.38272786
MUSCLE ADAPTATION	-1.0384541	0.380835838
CARBOHYDRATE PHOSPHORYLATION	-1.038799725	0.380330944
MORPHOGENESIS OF EMBRYONIC EPITHELIUM	-1.03912023	0.37976759
NEURAL RETINA DEVELOPMENT	-1.03965856	0.378914585
METAPHASE PLATE CONGRESSION	-1.040024974	0.378348573
REGULATION OF TRANSCRIPTION ELONGATION FROM RNA	-1.040657443	0.3773255

AROMATIC AMINO ACID FAMILY METABOLIC PROCESS	-1.040735377	0.377203262
EMBRYONIC EYE MORPHOGENESIS	-1.041445956	0.376018091
SENSORY PERCEPTION OF MECHANICAL STIMULUS	-1.042251842	0.374723969
CYTOPLASMIC TRANSLATION	-1.042689657	0.374006489
HISTONE H3 ACETYLATION	-1.042807841	0.373852364
PROTEIN LOCALIZATION TO CHROMOSOME TELOMERIC RE	-1.043325106	0.372951528
MATERNAL PLACENTA DEVELOPMENT	-1.043419923	0.372797402
PROTEIN CONTAINING COMPLEX LOCALIZATION	-1.04371546	0.372340341
RETINA MORPHOGENESIS IN CAMERA TYPE EYE	-1.043901396	0.372048034
PANCREAS DEVELOPMENT	-1.044806231	0.37054664
CAMP MEDIATED SIGNALING	-1.045586349	0.369223288
RIG I SIGNALING PATHWAY	-1.046126631	0.368293221
REGULATION OF PROTEIN TARGETING TO MITOCHONDRION	-1.047020011	0.366826372
NEGATIVE REGULATION OF CELL CYCLE G1 S PHASE TRANS	-1.047086044	0.366712107
REGULATION OF TRANSLATION IN RESPONSE TO STRESS	-1.047827052	0.365556167
POSITIVE REGULATION OF ESTABLISHMENT OF PROTEIN LO	-1.048238886	0.364939665
RESPONSE TO PLATELET DERIVED GROWTH FACTOR	-1.048336432	0.364758967
REGULATION OF CELLULAR PROTEIN LOCALIZATION	-1.048910908	0.363855473
DNA DAMAGE RESPONSE SIGNAL TRANSDUCTION BY P53 C	-1.049482623	0.362920091
RETROGRADE AXONAL TRANSPORT	-1.049624459	0.362718134
CELLULAR RESPONSE TO KETONE	-1.049813991	0.362404568
REGULATION OF TRANSCRIPTION INVOLVED IN G1 S TRANS	-1.051756066	0.359138705
POSITIVE REGULATION OF INTRACELLULAR PROTEIN TRAN	-1.052387069	0.358150177
CYTOSKELETON DEPENDENT INTRACELLULAR TRANSPORT	-1.053987914	0.355522073
INTERMEDIATE FILAMENT BASED PROCESS	-1.055350181	0.353428094
CILIUM ORGANIZATION	-1.055627256	0.352944459
CELLULAR RESPONSE TO UV	-1.056427442	0.351684883
NEURON APOPTOTIC PROCESS	-1.056914977	0.350807962
REGULATION OF DOUBLE STRAND BREAK REPAIR VIA NONH	-1.058467108	0.348347271
CELL JUNCTION ASSEMBLY	-1.060652674	0.344996373
PYRIMIDINE CONTAINING COMPOUND BIOSYNTHETIC PROC	-1.061131602	0.344207145
NEGATIVE REGULATION OF PROTEIN KINASE B SIGNALING	-1.061314988	0.343896236
GENE SILENCING	-1.061864682	0.343048547
REGULATION OF CIRCADIAN RHYTHM	-1.062013574	0.342820016
HEPATICOBILIARY SYSTEM DEVELOPMENT	-1.062819862	0.341504636
POSITIVE REGULATION OF T CELL CYTOKINE PRODUCTION	-1.063612836	0.340266318
TRANSCRIPTION BY RNA POLYMERASE I	-1.066723244	0.335552207
DETECTION OF STIMULUS	-1.067509055	0.334252771
HOMOPHILIC CELL ADHESION VIA PLASMA MEMBRANE ADH	-1.068857635	0.332257113
REGULATION OF CARDIAC MUSCLE CELL ACTION POTENTIA	-1.069378052	0.331483829
REGULATION OF LAMELLIPODIUM ORGANIZATION	-1.069941677	0.330691943
ERBB SIGNALING PATHWAY	-1.070083186	0.330516559
CARBOHYDRATE TRANSPORT	-1.072947269	0.326272797
REGULATION OF MUSCLE ADAPTATION	-1.075988357	0.321516169
REGULATION OF MICROTUBULE BASED PROCESS	-1.076363432	0.320952814
WOUND HEALING SPREADING OF EPIDERMAL CELLS	-1.076411669	0.320878408
NCRNA TRANSCRIPTION	-1.076433321	0.320827919
TOXIN TRANSPORT	-1.077885751	0.318521353
MICROTUBULE POLYMERIZATION	-1.07795313	0.31841506
HISTONE PHOSPHORYLATION	-1.07942433	0.316323738
REGULATION OF ORGANIC ACID TRANSPORT	-1.080195048	0.315234231
CELL COMMUNICATION BY ELECTRICAL COUPLING	-1.080437667	0.314891435
CELL CYCLE PHASE TRANSITION	-1.080550508	0.314705421

FILOPODIUM ASSEMBLY	-1.081571072	0.31320137
REGULATION OF CELL CYCLE G2 M PHASE TRANSITION	-1.082571379	0.311633543
REGULATION OF GLUCOSE IMPORT	-1.084129384	0.309247257
STEROL BIOSYNTHETIC PROCESS	-1.084835767	0.308224183
REGULATION OF SYNAPSE STRUCTURE OR ACTIVITY	-1.085419713	0.307347263
REGULATION OF CELLULAR PROTEIN CATABOLIC PROCESS	-1.086147693	0.306356077
POSITIVE REGULATION OF MRNA CATABOLIC PROCESS	-1.086216894	0.306286987
MITOTIC SPINDLE ASSEMBLY	-1.086402331	0.306045169
SYNAPSE ASSEMBLY	-1.086952859	0.305247969
REGULATION OF GENE EXPRESSION EPIGENETIC	-1.087322296	0.304650069
RECEPTOR RECYCLING	-1.087933365	0.303680142
DNA REPLICATION INDEPENDENT NUCLEOSOME ORGANIZA	-1.088273978	0.303188535
CYTOKINESIS	-1.088420534	0.302941403
MICROTUBULE BASED TRANSPORT	-1.090594335	0.299731344
BROWN FAT CELL DIFFERENTIATION	-1.091382247	0.298647151
MODULATION OF EXCITATORY POSTSYNAPTIC POTENTIAL	-1.091655492	0.298272467
NEGATIVE REGULATION OF GENE SILENCING	-1.092844323	0.296614291
NEGATIVE REGULATION OF MICROTUBULE POLYMERIZATIO	-1.093003409	0.296348557
HAIR CELL DIFFERENTIATION	-1.093397977	0.295825062
NCRNA METABOLIC PROCESS	-1.095712297	0.292700037
REGULATION OF VIRAL INDUCED CYTOPLASMIC PATTERN R	-1.095961508	0.292346612
INTRINSIC APOPTOTIC SIGNALING PATHWAY IN RESPONSE	-1.095995508	0.292296123
NUCLEOTIDE EXCISION REPAIR DNA DUPLEX UNWINDING	-1.096044577	0.292229689
RETINA DEVELOPMENT IN CAMERA TYPE EYE	-1.096450221	0.291668992
RNA 3 END PROCESSING	-1.096809916	0.291121581
ASPARTATE FAMILY AMINO ACID METABOLIC PROCESS	-1.097489067	0.290210115
POSITIVE REGULATION OF TRANSCRIPTION BY RNA POLYM	-1.09762537	0.290045361
GLUCAN BIOSYNTHETIC PROCESS	-1.098430819	0.288971798
CARBOHYDRATE TRANSMEMBRANE TRANSPORT	-1.100042179	0.286811385
RESPONSE TO TOPOLOGICALLY INCORRECT PROTEIN	-1.100210803	0.286598798
VASCULOGENESIS	-1.100398418	0.286303834
REGULATION OF SIGNAL TRANSDUCTION BY P53 CLASS ME	-1.100652962	0.285982297
NADH METABOLIC PROCESS	-1.102680271	0.283189439
TRANSPORT ALONG MICROTUBULE	-1.103583828	0.281940492
INTEGRATED STRESS RESPONSE SIGNALING	-1.103683227	0.281823569
EPIDERMAL GROWTH FACTOR RECEPTOR SIGNALING PATH	-1.104964992	0.28011756
REGULATION OF MITOCHONDRIAL MEMBRANE PERMEABILIT	-1.105207951	0.279777422
SKIN DEVELOPMENT	-1.106215409	0.278398265
REGULATION OF PROTEIN CONTAINING COMPLEX DISASSE	-1.106277485	0.278318545
PYRIMIDINE NUCLEOSIDE TRIPHOSPHATE METABOLIC PRO	-1.106855241	0.277574492
CELLULAR RESPONSE TO LIGHT STIMULUS	-1.107672717	0.276474355
DENDRITE EXTENSION	-1.107816653	0.276275055
MICROTUBULE POLYMERIZATION OR DEPOLYMERIZATION	-1.10830004	0.275600092
REGULATION OF BICELLULAR TIGHT JUNCTION ASSEMBLY	-1.108610398	0.275212122
CELL CYCLE ARREST	-1.109224997	0.274361775
BRANCHING INVOLVED IN BLOOD VESSEL MORPHOGENESIS	-1.109453849	0.274058839
REGULATION OF UBIQUITIN DEPENDENT PROTEIN CATABOL	-1.11020712	0.273046394
MITOTIC METAPHASE PLATE CONGRESSION	-1.110445428	0.2727222
ONE CARBON METABOLIC PROCESS	-1.110719696	0.272408634
RESPONSE TO GONADOTROPIN	-1.111045553	0.271980803
TRANSCRIPTION ELONGATION FROM RNA POLYMERASE II F	-1.111934024	0.270819548
RESPONSE TO AMPHETAMINE	-1.111989051	0.270753115
HETEROCHROMATIN ORGANIZATION	-1.112527896	0.270022348

REGULATION OF DENDRITE DEVELOPMENT	-1.114122386	0.267928369
ESTABLISHMENT OF PROTEIN LOCALIZATION TO TELOMERE	-1.114277797	0.267710468
NEGATIVE REGULATION OF PROTEIN MODIFICATION BY SM	-1.116066561	0.265401244
REGULATION OF CYTOKINESIS	-1.116390317	0.264949497
MEIOTIC CELL CYCLE PROCESS	-1.116554107	0.264760827
MICROTUBULE BASED MOVEMENT	-1.117745255	0.26323286
CEREBRAL CORTEX DEVELOPMENT	-1.118000996	0.262945867
REGULATION OF VIRAL GENOME REPLICATION	-1.118940987	0.261824472
REGULATION OF MUSCLE CELL DIFFERENTIATION	-1.120502097	0.259868675
NEGATIVE REGULATION OF LIPID LOCALIZATION	-1.121438006	0.258710077
VENTRICULAR CARDIAC MUSCLE TISSUE DEVELOPMENT	-1.122047824	0.257936793
ESTABLISHMENT OF PROTEIN LOCALIZATION TO CHROMOS	-1.122050664	0.257931478
HYPEROSMOTIC RESPONSE	-1.123191637	0.256525748
REGULATION OF MICROTUBULE DEPOLYMERIZATION	-1.123298115	0.256395539
CYTOPLASMIC MICROTUBULE ORGANIZATION	-1.12333342	0.25634505
NEUROTRANSMITTER RECEPTOR TRANSPORT	-1.123440679	0.256198896
GAMETE GENERATION	-1.123697214	0.255906589
MITOTIC CYTOKINETIC PROCESS	-1.124672602	0.2546603
NEGATIVE REGULATION OF PROTEIN CATABOLIC PROCESS	-1.125732578	0.253328975
RECOMBINATIONAL REPAIR	-1.125865951	0.253161563
NEURAL TUBE FORMATION	-1.126278521	0.252643383
RESPONSE TO GLUCAGON	-1.126862361	0.25197905
PIGMENT BIOSYNTHETIC PROCESS	-1.127019179	0.251790379
REGULATION OF MITOCHONDRIAL OUTER MEMBRANE PERM	-1.127081951	0.251713316
NUCLEAR TRANSCRIBED MRNA POLY A TAIL SHORTENING	-1.128091772	0.250509544
CELL CELL JUNCTION ASSEMBLY	-1.128942224	0.249465212
PROTEIN DEPOLYMERIZATION	-1.129452927	0.248907171
DNA MODIFICATION	-1.12948682	0.248872626
REGULATION OF ORGANELLE ASSEMBLY	-1.130120944	0.248096684
NEGATIVE REGULATION OF EPITHELIAL CELL DIFFERENTIAT	-1.130414145	0.247735287
NEGATIVE REGULATION OF UBIQUITIN PROTEIN TRANSFER	-1.13095573	0.24704438
POSITIVE REGULATION OF MACROAUTOPHAGY	-1.132266262	0.245418092
EMBRYO DEVELOPMENT ENDING IN BIRTH OR EGG HATCHI	-1.13382097	0.243438378
PALLIUM DEVELOPMENT	-1.136093076	0.240735869
EMBRYONIC AXIS SPECIFICATION	-1.137674416	0.238780071
REGULATION OF PROTEIN LOCALIZATION TO CELL SURFAC	-1.138305216	0.238065248
NEGATIVE REGULATION OF METAPHASE ANAPHASE TRANS	-1.138365994	0.237974899
REGULATION OF GLYCOGEN METABOLIC PROCESS	-1.138821426	0.237462033
REGULATION OF MICROTUBULE POLYMERIZATION OR DEPO	-1.138828009	0.237451404
SPINDLE LOCALIZATION	-1.139068579	0.237169727
METANEPHRIC NEPHRON MORPHOGENESIS	-1.139812735	0.236306093
DNA DEALKYLATION	-1.142415042	0.233329879
CELLULAR COMPONENT DISASSEMBLY INVOLVED IN EXECU	-1.142894182	0.232729321
SPLICEOSOMAL COMPLEX ASSEMBLY	-1.143405103	0.232195197
RESPONSE TO INTERLEUKIN 6	-1.144418033	0.231017998
REGULATION OF SYNAPSE ASSEMBLY	-1.144498452	0.230924991
POSITIVE REGULATION OF CELLULAR RESPONSE TO INSUL	-1.146312341	0.228825697
EMBRYONIC PATTERN SPECIFICATION	-1.146877707	0.228209196
REGULATION OF MITOTIC CELL CYCLE	-1.147692642	0.227194094
AMINO ACID ACTIVATION	-1.148051225	0.226840669
MISMATCH REPAIR	-1.148940047	0.225918574
MICROTUBULE BUNDLE FORMATION	-1.150158026	0.224494243
DNA TEMPLATED TRANSCRIPTION ELONGATION	-1.151226068	0.223279841

POSITIVE REGULATION OF PEPTIDE HORMONE SECRETION	-1.151378887	0.223077884
REGULATION OF POSTSYNAPTIC MEMBRANE POTENTIAL	-1.151408897	0.22305131
NEGATIVE REGULATION OF CHROMOSOME ORGANIZATION	-1.15277249	0.22149677
NEGATIVE REGULATION OF OSTEOBLAST DIFFERENTIATION	-1.154349201	0.219806706
PROTEIN LOCALIZATION TO CHROMATIN	-1.154761047	0.219349644
PROTEIN LOCALIZATION TO CHROMOSOME	-1.154904463	0.219187547
RIBONUCLEOPROTEIN COMPLEX BIOGENESIS	-1.155173698	0.218871324
DOPAMINE RECEPTOR SIGNALING PATHWAY	-1.155896539	0.21804755
KERATINOCYTE DIFFERENTIATION	-1.156063273	0.217840278
PRESYNAPTIC ENDOCYTOSIS	-1.156563302	0.217340699
REGULATION OF PROTEIN MODIFICATION BY SMALL PROTE	-1.159037095	0.214723225
REGULATION OF SYNAPTIC VESICLE RECYCLING	-1.159304042	0.214460149
ENDOSOME TO LYSOSOME TRANSPORT	-1.159918529	0.213817074
POSITIVE REGULATION OF CELL CYCLE PROCESS	-1.160704931	0.213057077
PYRIMIDINE NUCLEOTIDE BIOSYNTHETIC PROCESS	-1.160834019	0.212921553
ANAPHASE PROMOTING COMPLEX DEPENDENT CATABOLIC	-1.162593837	0.211122538
TETRAPYRROLE METABOLIC PROCESS	-1.162816416	0.210886035
REGULATION OF MITOTIC NUCLEAR DIVISION	-1.163489507	0.210192471
CHROMATIN SILENCING	-1.164076966	0.209589256
REGULATION OF CHOLESTEROL BIOSYNTHETIC PROCESS	-1.16528418	0.208226043
REGULATION OF CHROMOSOME SEPARATION	-1.165291644	0.208218071
CELL CYCLE G2 M PHASE TRANSITION	-1.16563767	0.20788059
POSITIVE REGULATION OF SYNAPSE ASSEMBLY	-1.166631994	0.20687346
MONOSACCHARIDE BIOSYNTHETIC PROCESS	-1.167698232	0.205773324
REGULATION OF GLUCOSE TRANSMEMBRANE TRANSPORT	-1.168049256	0.205443815
POLYSACCHARIDE BIOSYNTHETIC PROCESS	-1.168639591	0.204899061
POSITIVE REGULATION OF VIRAL PROCESS	-1.169443894	0.204011512
REGULATION OF TELOMERE MAINTENANCE	-1.170462988	0.203028298
POSITIVE REGULATION OF MITOCHONDRIAL OUTER MEMBR	-1.171285855	0.202172636
RESPONSE TO MINERALOCORTICOID	-1.17190571	0.201529562
COLUMNAR CUBOIDAL EPITHELIAL CELL DIFFERENTIATION	-1.172563471	0.200907745
MULTICELLULAR ORGANISM AGING	-1.172633131	0.200809424
MODULATION OF PROCESS OF OTHER ORGANISM	-1.174809146	0.198481599
REGULATION OF HEART GROWTH	-1.178513574	0.194835737
NEGATIVE REGULATION OF DEPHOSPHORYLATION	-1.178664892	0.194686926
HOMOLOGOUS CHROMOSOME SEGREGATION	-1.179933247	0.193437979
ESTABLISHMENT OF EPITHELIAL CELL POLARITY	-1.180390948	0.192959659
SECONDARY METABOLIC PROCESS	-1.180631113	0.192747072
REGULATION OF TRANSLATIONAL INITIATION	-1.180989863	0.192422878
PROTEIN CONTAINING COMPLEX DISASSEMBLY	-1.181523156	0.191886096
CARDIAC VENTRICLE MORPHOGENESIS	-1.182047199	0.191354629
PROTEIN ACYLATION	-1.182841569	0.190650436
POSITIVE REGULATION OF LAMELLIPODIUM ORGANIZATION	-1.182968839	0.190541485
NUCLEOLAR LARGE RRNA TRANSCRIPTION BY RNA POLYME	-1.183786395	0.189813375
POSITIVE REGULATION OF CELLULAR PROTEIN LOCALIZATI	-1.184249937	0.189401489
NUCLEUS LOCALIZATION	-1.184859358	0.188758414
IN UTERO EMBRYONIC DEVELOPMENT	-1.185529341	0.188083451
EMBRYONIC CAMERA TYPE EYE MORPHOGENESIS	-1.185640192	0.187958556
REGULATION OF POSTSYNAPTIC MEMBRANE NEUROTRANS	-1.186471437	0.187179957
REGULATION OF BROWN FAT CELL DIFFERENTIATION	-1.186607059	0.187052405
RESPONSE TO LEAD ION	-1.187233442	0.18640933
MICROTUBULE ORGANIZING CENTER ORGANIZATION	-1.187368126	0.186255205
RESPONSE TO MISFOLDED PROTEIN	-1.188027962	0.185585557

REGULATION OF EPIDERMAL GROWTH FACTOR ACTIVATED	-1.189382656	0.184291435
NUCLEOTIDE EXCISION REPAIR PREINCISION COMPLEX ASS	-1.189710197	0.183935352
INTRINSIC APOPTOTIC SIGNALING PATHWAY IN RESPONSE	-1.190591577	0.183148781
REGULATION OF AMINO ACID TRANSPORT	-1.191114133	0.182675776
PROTEOGLYCAN BIOSYNTHETIC PROCESS	-1.191148094	0.182630601
INSULIN SECRETION INVOLVED IN CELLULAR RESPONSE TO	-1.191788985	0.181998156
REGULATION OF ANION TRANSMEMBRANE TRANSPORT	-1.191874005	0.181950324
REGULATION OF CELL AGING	-1.192591006	0.181232844
SPINDLE ASSEMBLY	-1.19267077	0.181150466
NEGATIVE REGULATION OF PRODUCTION OF MOLECULAR M	-1.192953803	0.180908649
RRNA TRANSCRIPTION	-1.195903244	0.178277888
REGULATION OF OXIDATIVE PHOSPHORYLATION	-1.196353356	0.177873973
INTRACELLULAR LIPID TRANSPORT	-1.196701669	0.177515233
METAPHASE ANAPHASE TRANSITION OF CELL CYCLE	-1.197164765	0.177151178
NEGATIVE REGULATION OF GENE EXPRESSION EPIGENETIC	-1.200911509	0.173749791
RESPONSE TO IRON ION	-1.201394343	0.173276785
ORGANELLE TRANSPORT ALONG MICROTUBULE	-1.2033842	0.171488399
RESPONSE TO ETHANOL	-1.2037163	0.171222666
REGULATION OF CYTOPLASMIC TRANSLATION	-1.204003231	0.17099945
CELLULAR GLUCOSE HOMEOSTASIS	-1.205249905	0.16996309
PROTEIN K63 LINKED DEUBIQUITINATION	-1.20682049	0.168599877
MAINTENANCE OF CELL NUMBER	-1.20735465	0.168161417
REGULATION OF CHROMOSOME SEGREGATION	-1.207778583	0.167781418
PHOTORECEPTOR CELL MAINTENANCE	-1.207913148	0.167643237
RESPONSE TO DEXAMETHASONE	-1.208087133	0.167507713
MITOCHONDRIAL OUTER MEMBRANE PERMEABILIZATION	-1.211118742	0.164946043
APICAL JUNCTION ASSEMBLY	-1.211445779	0.164661708
PORPHYRIN CONTAINING COMPOUND METABOLIC PROCES	-1.211771475	0.164361429
PEPTIDYL LYSINE ACETYLATION	-1.21376157	0.162623533
PROTEIN TRANSPORT ALONG MICROTUBULE	-1.216164079	0.160598644
P BODY ASSEMBLY	-1.216291191	0.16050298
REGULATION OF MRNA METABOLIC PROCESS	-1.216779578	0.160067177
PROTEIN MODIFICATION BY SMALL PROTEIN REMOVAL	-1.217377742	0.159607459
RESPONSE TO LEUKEMIA INHIBITORY FACTOR	-1.218192738	0.158879349
CELLULAR RESPONSE TO TOPOLOGICALLY INCORRECT PR	-1.218713454	0.158464805
RESPONSE TO ACTIVITY	-1.220535676	0.156989985
ESTABLISHMENT OR MAINTENANCE OF CELL POLARITY	-1.221379157	0.15634691
INTRACILIARY TRANSPORT	-1.221942044	0.155900478
NUCLEAR MIGRATION	-1.223329556	0.15478174
REGULATION OF STEM CELL POPULATION MAINTENANCE	-1.223639583	0.154516006
PROTEIN LOCALIZATION TO MICROTUBULE	-1.224594658	0.153822442
RESPONSE TO X RAY	-1.225894165	0.152855173
REGULATION OF PROTEIN TARGETING TO MEMBRANE	-1.226181546	0.152621327
CELLULAR PROCESS INVOLVED IN REPRODUCTION IN MULT	-1.226680984	0.152251958
ENDOPLASMIC RETICULUM UNFOLDED PROTEIN RESPONSE	-1.228396146	0.150870144
PHOSPHATIDYLCHOLINE BIOSYNTHETIC PROCESS	-1.22880228	0.150476859
ESTABLISHMENT OR MAINTENANCE OF BIPOLAR CELL POLA	-1.228865743	0.150429027
ESTABLISHMENT OF SPINDLE ORIENTATION	-1.231592308	0.148311131
MUSCLE HYPERTROPHY	-1.232309654	0.147750434
STEM CELL PROLIFERATION	-1.234889027	0.145664427
MEIOTIC CHROMOSOME SEGREGATION	-1.236535984	0.144399535
PROTEIN ACETYLATION	-1.237753983	0.143424294
DNA METHYLATION	-1.238855076	0.142550031

REGULATION OF ORGAN GROWTH	-1.242243272	0.14004948
REGULATION OF CHROMATIN BINDING	-1.245300636	0.13780669
POSITIVE REGULATION OF GENE EXPRESSION EPIGENETIC	-1.246833595	0.136775644
REGULATION OF NUCLEOTIDE BIOSYNTHETIC PROCESS	-1.247165465	0.136493967
REGULATION OF TRANSCRIPTION INITIATION FROM RNA PC	-1.248615214	0.135441662
POSITIVE REGULATION OF ESTABLISHMENT OF PROTEIN LC	-1.248686758	0.135391173
RESPONSE TO AMINE	-1.249641795	0.134697609
CYTOSKELETON DEPENDENT CYTOKINESIS	-1.253047348	0.132242232
REGULATION OF NEURON MIGRATION	-1.253722766	0.131769226
REGULATION OF DNA DEPENDENT DNA REPLICATION	-1.253978539	0.131575241
REGULATION OF CYCLIN DEPENDENT PROTEIN KINASE ACT	-1.254670097	0.131104893
POSITIVE REGULATION OF DNA REPLICATION	-1.256381449	0.129895806
REGULATION OF TRANSLATIONAL INITIATION IN RESPONSE	-1.258193105	0.1286256
ESTABLISHMENT OF CELL POLARITY	-1.258785108	0.128232315
RIBOSOMAL LARGE SUBUNIT BIOGENESIS	-1.259083019	0.128054273
REGULATION OF DNA TEMPLATED TRANSCRIPTION INITIATI	-1.260001134	0.127363367
NEGATIVE REGULATION OF ERBB SIGNALING PATHWAY	-1.263738067	0.124892046
REGULATION OF POLYSACCHARIDE BIOSYNTHETIC PROCE	-1.265406183	0.123725476
POSITIVE REGULATION OF CYCLIN DEPENDENT PROTEIN KI	-1.266535065	0.123021283
MRNA SPLICE SITE SELECTION	-1.267218665	0.122532333
MODULATION OF PROCESS OF OTHER ORGANISM INVOLVE	-1.267676872	0.122205481
EMBRYONIC CAMERA TYPE EYE DEVELOPMENT	-1.26900411	0.121392337
GLUTAMINE FAMILY AMINO ACID METABOLIC PROCESS	-1.269435374	0.121062827
ORGANELLE FISSION	-1.271142014	0.119888286
REGULATION OF PROTON TRANSPORT	-1.271739168	0.119473742
RESPONSE TO INTERLEUKIN 4	-1.271789675	0.119433882
POSITIVE REGULATION OF PROTEIN TARGETING TO MEMBF	-1.272844321	0.118756261
RETROGRADE VESICLE MEDIATED TRANSPORT GOLGI TO E	-1.275851309	0.11689347
POSITIVE REGULATION OF HORMONE SECRETION	-1.277930025	0.11564718
DNA METHYLATION OR DEMETHYLATION	-1.279369256	0.11477026
REGULATION OF RIG I SIGNALING PATHWAY	-1.279891542	0.114454037
MEIOTIC CELL CYCLE	-1.280178621	0.114294597
MIRNA METABOLIC PROCESS	-1.280463954	0.114084668
DEVELOPMENTAL PIGMENTATION	-1.282112136	0.11304565
NEGATIVE REGULATION OF MUSCLE CELL DIFFERENTIATIO	-1.284844433	0.111344957
PEPTIDYL LYSINE MODIFICATION	-1.285057512	0.111209432
MICROTUBULE DEPOLYMERIZATION	-1.285581514	0.110792231
CELL CELL JUNCTION ORGANIZATION	-1.285711321	0.11068328
REGULATION OF CILIUM ASSEMBLY	-1.28595569	0.110545099
TRANSCRIPTION INITIATION FROM RNA POLYMERASE II PR	-1.287243859	0.109793073
SIGNAL TRANSDUCTION INVOLVED IN CELL CYCLE CHECKP	-1.287714875	0.109551256
REGULATION OF SPINDLE ORGANIZATION	-1.288303535	0.109197831
POSITIVE REGULATION OF CELL CYCLE	-1.288483762	0.109080908
REGULATION OF POLYSACCHARIDE METABOLIC PROCESS	-1.288600556	0.109019789
NUCLEOTIDE EXCISION REPAIR	-1.288815368	0.108870978
SKELETAL MUSCLE TISSUE REGENERATION	-1.290504254	0.107855877
POSITIVE REGULATION OF CHROMOSOME ORGANIZATION	-1.291159166	0.107481193
POSITIVE REGULATION OF CHROMATIN ORGANIZATION	-1.292098798	0.106931125
RESPONSE TO ANTIBIOTIC	-1.293246201	0.106160498
NEGATIVE REGULATION OF DNA REPLICATION	-1.293699008	0.10591868
ESTABLISHMENT OF MITOTIC SPINDLE LOCALIZATION	-1.294629857	0.105352668
CELLULAR RESPONSE TO DEXAMETHASONE STIMULUS	-1.297683604	0.103580226
RNA DEPENDENT DNA BIOSYNTHETIC PROCESS	-1.299386016	0.102615614

REGULATION OF STRIATED MUSCLE CELL DIFFERENTIATIO	-1.299736554	0.102416314
NEGATIVE REGULATION OF HISTONE MODIFICATION	-1.300143091	0.10220107
CELL DIFFERENTIATION INVOLVED IN EMBRYONIC PLACENT	-1.303003847	0.10059604
PEPTIDYL THREONINE MODIFICATION	-1.305340073	0.099368352
HISTONE UBIQUITINATION	-1.3058951	0.099139821
DNA TEMPLATED TRANSCRIPTION INITIATION	-1.306618819	0.098693389
REGULATION OF DNA TEMPLATED TRANSCRIPTION ELONG/	-1.307316051	0.098289474
POSITIVE REGULATION OF NEURON DEATH	-1.308519008	0.097579966
POSITIVE REGULATION OF TRANSCRIPTION OF NOTCH REC	-1.309702878	0.096960807
POSITIVE REGULATION OF CELL CYCLE ARREST	-1.314293846	0.094508088
POSITIVE REGULATION OF INSULIN SECRETION	-1.314434586	0.094438997
MITOTIC CELL CYCLE CHECKPOINT	-1.315233519	0.094003194
RESPONSE TO UV	-1.315975803	0.09358865
POSITIVE REGULATION OF INSULIN SECRETION INVOLVED I	-1.316977517	0.093038582
NON RECOMBINATIONAL REPAIR	-1.319288218	0.091893271
MITOTIC G2 DNA DAMAGE CHECKPOINT	-1.319730463	0.091683341
VESICLE TRANSPORT ALONG MICROTUBULE	-1.320122627	0.091460125
POSITIVE REGULATION OF STEM CELL PROLIFERATION	-1.32094599	0.091040267
CHROMOSOME ORGANIZATION INVOLVED IN MEIOTIC CELL	-1.32111542	0.090941945
GLOBAL GENOME NUCLEOTIDE EXCISION REPAIR	-1.321121957	0.090939288
REGULATION OF PROTEIN TARGETING	-1.322074667	0.090397192
FEMALE MEIOTIC NUCLEAR DIVISION	-1.322328781	0.090301528
RETINOIC ACID RECEPTOR SIGNALING PATHWAY	-1.322571707	0.090155374
CELL CYCLE CHECKPOINT	-1.323035204	0.089897613
HISTONE H3 K9 MODIFICATION	-1.324604417	0.08910307
REGULATION OF PROTEIN ACETYLATION	-1.325088766	0.088845309
DEMETHYLATION	-1.326943628	0.087917899
MRNA 3 END PROCESSING	-1.326949141	0.087915242
SYNAPTIC VESICLE RECYCLING	-1.329005101	0.086868252
ADHERENS JUNCTION ORGANIZATION	-1.329049029	0.086839021
POSITIVE REGULATION OF CILIUM ASSEMBLY	-1.332264027	0.085303082
POSITIVE REGULATION OF PROTEIN TARGETING TO MITOC	-1.333027536	0.084899167
MRNA CIS SPLICING VIA SPLICEOSOME	-1.335934837	0.083448263
GLANDULAR EPITHELIAL CELL DIFFERENTIATION	-1.337027806	0.082900852
OOCYTE DIFFERENTIATION	-1.337536846	0.082682951
CELLULAR RESPONSE TO ARSENIC CONTAINING SUBSTAN(	-1.33781852	0.082571343
POSITIVE REGULATION OF DNA TEMPLATED TRANSCRIPTIO	-1.33824986	0.082395959
REGULATION OF DNA REPLICATION	-1.340445718	0.08133834
CHROMATIN ORGANIZATION INVOLVED IN REGULATION OF	-1.341146403	0.081040718
NEGATIVE REGULATION OF CELLULAR AMIDE METABOLIC P	-1.342518932	0.080373727
POSITIVE REGULATION OF TRANSCRIPTION INITIATION FRO	-1.343756325	0.079836946
REGULATION OF MICROTUBULE CYTOSKELETON ORGANIZ/	-1.343760967	0.079834289
MITOTIC CYTOKINESIS	-1.345379109	0.07905569
METHYLATION	-1.356539902	0.074322978
TETRAPYRROLE BIOSYNTHETIC PROCESS	-1.357254906	0.074046615
PEPTIDYL LYSINE TRIMETHYLATION	-1.357765549	0.073849972
RIBONUCLEOPROTEIN COMPLEX SUBUNIT ORGANIZATION	-1.359420931	0.073140464
REGULATION OF PEPTIDYL LYSINE ACETYLATION	-1.359593043	0.073068716
ESTABLISHMENT OF MITOTIC SPINDLE ORIENTATION	-1.363095413	0.071540749
CELLULAR RESPONSE TO CORTICOSTEROID STIMULUS	-1.372445177	0.067756705
INTRACILIARY TRANSPORT INVOLVED IN CILIUM ASSEMBLY	-1.373023056	0.06751223
NEGATIVE REGULATION OF CELLULAR PROTEIN CATABOLI(	-1.374060189	0.067041882
RESPONSE TO ARSENIC CONTAINING SUBSTANCE	-1.376796851	0.066042725

BETA CATENIN TCF COMPLEX ASSEMBLY	-1.379441153	0.064963847
VASCULAR ASSOCIATED SMOOTH MUSCLE CONTRACTION	-1.379717678	0.064889442
TRANSCRIPTION COUPLED NUCLEOTIDE EXCISION REPAIR	-1.381310017	0.064185248
MACROMOLECULE METHYLATION	-1.381584385	0.064078955
TELOMERE MAINTENANCE VIA TELOMERE LENGTHENING	-1.385187127	0.062710428
GLUCOSE CATABOLIC PROCESS	-1.38676851	0.062054066
DOUBLE STRAND BREAK REPAIR	-1.387090234	0.061926514
POSITIVE REGULATION OF PEPTIDYL THREONINE PHOSPHO	-1.387287293	0.061868053
MITOTIC DNA INTEGRITY CHECKPOINT	-1.397473015	0.057975058
FEMALE GAMETE GENERATION	-1.39775522	0.057852821
ENTEROENDOCRINE CELL DIFFERENTIATION	-1.397805166	0.057839534
GERM CELL DEVELOPMENT	-1.401327529	0.056646391
CYTOKINETIC PROCESS	-1.40765407	0.054318567
DNA STRAND ELONGATION INVOLVED IN DNA REPLICATION	-1.407754896	0.054291993
NEGATIVE REGULATION OF MRNA PROCESSING	-1.412273764	0.052798571
POSITIVE REGULATION OF VIRAL GENOME REPLICATION	-1.416101633	0.051626687
COLUMNAR CUBOIDAL EPITHELIAL CELL DEVELOPMENT	-1.418749731	0.05073648
FLUID TRANSPORT	-1.419655468	0.050428229
POSITIVE REGULATION OF RNA SPLICING	-1.419750338	0.050414943
REGULATION OF PEPTIDYL THREONINE PHOSPHORYLATION	-1.420606816	0.050101377
PIGMENT CELL DIFFERENTIATION	-1.422171903	0.049612428
MITOTIC SPINDLE ORGANIZATION	-1.424842047	0.048687676
REGULATION OF MUSCLE HYPERTROPHY	-1.429523614	0.047404183
CELL DIVISION	-1.429578348	0.047385582
RNA SPLICING	-1.431491475	0.046864744
NUCLEOTIDE EXCISION REPAIR PREINCISION COMPLEX STA	-1.431691302	0.046822227
MICROTUBULE CYTOSKELETON ORGANIZATION	-1.432227439	0.046665444
DNA REPAIR	-1.433302285	0.046314676
DNA BIOSYNTHETIC PROCESS	-1.43803153	0.044837198
POSITIVE REGULATION OF MRNA METABOLIC PROCESS	-1.453492018	0.040678471
NEGATIVE REGULATION OF PEPTIDYL LYSINE ACETYLATION	-1.454991996	0.040293157
NEGATIVE REGULATION OF STRIATED MUSCLE CELL DIFFE	-1.455268222	0.040224066
REGULATION OF RNA SPLICING	-1.456988965	0.039822809
SIGNAL TRANSDUCTION BY P53 CLASS MEDIATOR	-1.458456811	0.03941358
GLANDULAR EPITHELIAL CELL DEVELOPMENT	-1.459198126	0.039235538
REGULATION OF HISTONE MODIFICATION	-1.462051082	0.038571205
MICROTUBULE CYTOSKELETON ORGANIZATION INVOLVED	-1.462407986	0.038480855
RNA SPLICING VIA TRANSESTERIFICATION REACTIONS	-1.463940462	0.038095542
MITOTIC G1 S TRANSITION CHECKPOINT	-1.468967914	0.036905056
GLYCOLYTIC PROCESS THROUGH FRUCTOSE 6 PHOSPHAT	-1.474303816	0.035568417
REGULATION OF CELLULAR RESPONSE TO TRANSFORMING	-1.478408327	0.034545343
G2 DNA DAMAGE CHECKPOINT	-1.478648144	0.034492197
REGULATION OF STEM CELL PROLIFERATION	-1.479166977	0.034396533
POSITIVE REGULATION OF DNA TEMPLATED TRANSCRIPTIO	-1.479740114	0.034274295
INTRACELLULAR STEROL TRANSPORT	-1.479942	0.034231778
REGULATION OF CELL CYCLE ARREST	-1.48322277	0.03351164
PYRIMIDINE NUCLEOSIDE TRIPHOSPHATE BIOSYNTHETIC P	-1.483629386	0.033447864
MONOSACCHARIDE CATABOLIC PROCESS	-1.486214983	0.032860594
NEGATIVE REGULATION OF CHROMATIN ORGANIZATION	-1.487982851	0.032446049
NEGATIVE REGULATION OF LIPID TRANSPORT	-1.488626829	0.032334441
OOGENESIS	-1.494281181	0.031125354
NEGATIVE REGULATION OF PROTEASOMAL PROTEIN CATA	-1.497934563	0.030360042
HISTONE DEUBIQUITINATION	-1.499016905	0.030150113

REGULATION OF CHROMOSOME ORGANIZATION	-1.499506171	0.030057106
MRNA PROCESSING	-1.499856615	0.029990673
MICROTUBULE NUCLEATION	-1.501549586	0.029618646
DNA INTEGRITY CHECKPOINT	-1.504045855	0.029135011
NEGATIVE REGULATION OF PROTEOLYSIS INVOLVED IN CEI	-1.513394248	0.027373199
NEGATIVE REGULATION OF PROTEASOMAL UBIQUITIN DEPI	-1.518242364	0.026461733
NUCLEAR CHROMOSOME SEGREGATION	-1.520260776	0.026033902
REGULATION OF SISTER CHROMATID COHESION	-1.521853767	0.025744253
MODULATION BY HOST OF SYMBIONT PROCESS	-1.522658185	0.025598099
COVALENT CHROMATIN MODIFICATION	-1.52728854	0.024763697
REGULATION OF ALTERNATIVE MRNA SPLICING VIA SPLICE(	-1.535352597	0.023411114
MITOTIC SISTER CHROMATID SEGREGATION	-1.537260771	0.023092233
CHROMOSOME CONDENSATION	-1.547150231	0.021484546
POSTREPLICATION REPAIR	-1.547918647	0.021375596
PROTEIN DNA COMPLEX DISASSEMBLY	-1.551155751	0.020915877
MITOTIC NUCLEAR DIVISION	-1.553014765	0.020650143
NEGATIVE REGULATION OF UBIQUITIN DEPENDENT PROTEI	-1.555397183	0.020267487
SPINDLE ORGANIZATION	-1.556399353	0.02010539
BLASTOCYST GROWTH	-1.558094475	0.019836999
ATP DEPENDENT CHROMATIN REMODELING	-1.561018102	0.019398539
PROTEIN DEMETHYLATION	-1.562593986	0.019183295
POSITIVE REGULATION OF MRNA SPLICING VIA SPLICEOSO	-1.563191432	0.019092946
REGULATION OF MRNA SPLICING VIA SPLICEOSOME	-1.563527943	0.019063715
PROTEIN LOCALIZATION TO ENDOSOME	-1.564759788	0.01893882
PHYSIOLOGICAL CARDIAC MUSCLE HYPERTROPHY	-1.569347623	0.018348892
NUCLEOTIDE SUGAR METABOLIC PROCESS	-1.575019683	0.017546377
NUCLEOTIDE EXCISION REPAIR DNA INCISION	-1.577717116	0.017222182
CHROMOSOME SEGREGATION	-1.579534847	0.016956449
ENDOCRINE PANCREAS DEVELOPMENT	-1.581323944	0.016704002
MICROTUBULE ORGANIZING CENTER LOCALIZATION	-1.585161055	0.016215053
CHROMATIN DISASSEMBLY	-1.588219492	0.015869599
BIOLOGICAL PROCESS INVOLVED IN INTERACTION WITH SY	-1.593502054	0.015207923
TELOMERE MAINTENANCE VIA SEMI CONSERVATIVE REPLIC	-1.594831379	0.015067084
CYTOPLASMIC TRANSLATIONAL INITIATION	-1.601596075	0.014325688
ERROR FREE TRANSLESION SYNTHESIS	-1.60367768	0.014078556
KERATINIZATION	-1.608784802	0.013480656
MITOTIC SISTER CHROMATID COHESION	-1.618259726	0.012441638
PROTEIN DNA COMPLEX SUBUNIT ORGANIZATION	-1.622525133	0.012013808
NEGATIVE REGULATION OF PROTEIN ACETYLATION	-1.624846885	0.011793249
CHROMATIN REMODELING	-1.62859228	0.011437166
HIPPO SIGNALING	-1.63062812	0.011227237
REGULATION OF CHROMATIN ORGANIZATION	-1.63529171	0.010807378
CELL CYCLE DNA REPLICATION	-1.6408675	0.010329058
NUCLEOSOME ASSEMBLY	-1.640902407	0.010329058
REGULATION OF UBIQUITIN PROTEIN LIGASE ACTIVITY	-1.646054089	0.009906542
ALTERNATIVE MRNA SPLICING VIA SPLICEOSOME	-1.646698106	0.009845423
ERROR PRONE TRANSLESION SYNTHESIS	-1.647410797	0.00977899
CORNIFICATION	-1.649933614	0.009630179
REGULATION OF MRNA POLYADENYLATION	-1.650459238	0.009587661
BICARBONATE TRANSPORT	-1.65708244	0.009135915
POSITIVE REGULATION OF MRNA PROCESSING	-1.658392981	0.009034936
TRANSLESION SYNTHESIS	-1.659603153	0.008952559
KINETOCHORE ORGANIZATION	-1.665908191	0.008577875

BLASTOCYST DEVELOPMENT	-1.692921507	0.006863894
DNA STRAND ELONGATION	-1.697471974	0.006638021
POSITIVE REGULATION OF UBIQUITIN PROTEIN TRANSFERA	-1.698322787	0.006587531
DNA DAMAGE RESPONSE DETECTION OF DNA DAMAGE	-1.699655922	0.006510469
REGULATION OF HISTONE METHYLATION	-1.702877478	0.006321798
TELOMERE ORGANIZATION	-1.708977243	0.005984316
DNA DEPENDENT DNA REPLICATION MAINTENANCE OF FIDE	-1.711716896	0.005878023
DNA SYNTHESIS INVOLVED IN DNA REPAIR	-1.714847514	0.005710611
NEGATIVE REGULATION OF MUSCLE HYPERTROPHY	-1.715911195	0.005673408
PROTEIN METHYLATION	-1.719966495	0.005463479
NEGATIVE REGULATION OF TRANSLATIONAL INITIATION	-1.724655847	0.005274808
REGULATION OF MRNA 3 END PROCESSING	-1.736009763	0.004785859
DNA DEPENDENT DNA REPLICATION	-1.737786719	0.004679565
SISTER CHROMATID SEGREGATION	-1.739045109	0.00464502
DNA REPLICATION	-1.758728494	0.003964742
REPLICATION FORK PROCESSING	-1.766485524	0.003725582
REGULATION OF UBIQUITIN PROTEIN TRANSFERASE ACTIVI	-1.767195627	0.003701666
N TERMINAL PROTEIN AMINO ACID MODIFICATION	-1.767586214	0.003691037
POSITIVE REGULATION OF NEURON APOPTOTIC PROCESS	-1.7704472	0.003590058
MICROTUBULE ANCHORING	-1.773078695	0.003486422
PEPTIDYL LYSINE METHYLATION	-1.779578877	0.003295094
NUCLEOTIDE EXCISION REPAIR DNA GAP FILLING	-1.792310546	0.002952298
REGULATION OF HISTONE H3 K4 METHYLATION	-1.797867528	0.002822089
DNA LIGATION	-1.808072904	0.002572299
BASE EXCISION REPAIR	-1.812148705	0.00248195
PROTEIN K48 LINKED DEUBIQUITINATION	-1.827693282	0.002131182
DNA GEOMETRIC CHANGE	-1.842308603	0.001830903
REGULATION OF HIPPO SIGNALING	-1.849505347	0.001708666
CHROMATIN ASSEMBLY OR DISASSEMBLY	-1.877692858	0.00127552
REGULATION OF MRNA PROCESSING	-1.879229094	0.001262234
HISTONE METHYLATION	-1.892194416	0.001065591
POSITIVE REGULATION OF CELLULAR RESPONSE TO TRANS	-1.896690207	0.001009787
NUCLEOSOME ORGANIZATION	-1.901705043	0.000935382
NEGATIVE REGULATION OF ORGAN GROWTH	-1.911789687	0.00083706
DNA PACKAGING	-1.942235857	0.000595243
HISTONE H3 K4 METHYLATION	-1.962144483	0.000459719
DNA CONFORMATION CHANGE	-1.969691025	0.000430488
SISTER CHROMATID COHESION	-2.03429909	0.000220559
POSITIVE REGULATION OF HISTONE METHYLATION	-2.122307424	8.23774E-05

Supplementary Table 5

Geneset - Hallmark sets	NES	P	FDR q-val
HALLMARK_INFLAMMATORY_RESPONSE	1.6466669	0	0.01599043
HALLMARK_IL6_JAK_STAT3_SIGNALING	1.5724483	0.002121	0.029705856
HALLMARK_TNFA_SIGNALING_VIA_NFKB	1.5530348	0	0.028556587
HALLMARK_EPITHELIAL_MESENCHYMAL_TRA	1.5236094	0	0.029071704
HALLMARK_KRAS_SIGNALING_UP	1.5146432	0	0.026684672
HALLMARK_ALLOGRAFT_REJECTION	1.3874598	0.007136	0.11609811
HALLMARK_P53_PATHWAY	1.3320295	0.012109	0.19881003
HALLMARK_INTERFERON_GAMMA_RESPONSE	1.3249388	0.023232	0.18708932
HALLMARK_MYC_TARGETS_V2	1.301257	0.081678	0.21919467
HALLMARK_UV_RESPONSE_DN	1.299219	0.039959	0.2016024
HALLMARK_MYOGENESIS	1.2959063	0.028311	0.1908483
HALLMARK_APICAL_JUNCTION	1.2783505	0.039715	0.21282354
HALLMARK_XENOBIOTIC_METABOLISM	1.2542982	0.061929	0.2499985
HALLMARK_TGF_BETA_SIGNALING	1.2445881	0.15115	0.25586566
HALLMARK_MYC_TARGETS_V1	1.2034445	0.077856	0.35505375
HALLMARK_WNT_BETA_CATENIN_SIGNALING	1.196455	0.209513	0.35467395
HALLMARK_UV_RESPONSE_UP	1.1803861	0.147808	0.386701
HALLMARK_COMPLEMENT	1.1780354	0.148828	0.37240186
HALLMARK_COAGULATION	1.130386	0.227651	0.51719075
HALLMARK_KRAS_SIGNALING_DN	1.1284252	0.214141	0.4985319
HALLMARK_PROTEIN_SECRETION	1.1080431	0.292147	0.5496658
HALLMARK_INTERFERON_ALPHA_RESPONSE	1.0973475	0.303609	0.5644147
HALLMARK_ESTROGEN_RESPONSE_LATE	1.0387559	0.398176	0.7737346
HALLMARK_G2M_CHECKPOINT	1.0237713	0.450151	0.7991119
HALLMARK_APOPTOSIS	0.99313873	0.52444	0.8824922
HALLMARK_HYPOXIA	0.99266595	0.52834	0.85003424
HALLMARK_IL2_STAT5_SIGNALING	0.95380706	0.614141	0.9533733
HALLMARK_UNFOLDED_PROTEIN_RESPONSE	0.9502598	0.596398	0.9311605
HALLMARK_MITOTIC_SPINDLE	0.9462565	0.633232	0.91121984
HALLMARK_HEDGEHOG_SIGNALING	0.9093537	0.63357	0.99067384
HALLMARK_E2F_TARGETS	0.9079018	0.720484	0.96241486
HALLMARK_ANDROGEN_RESPONSE	0.88885677	0.70582	0.9804933
HALLMARK_SPERMATOGENESIS	0.8874697	0.712357	0.95385194
HALLMARK_APICAL_SURFACE	0.8733808	0.682074	0.9567295
HALLMARK_REACTIVE_OXYGEN_SPECIES_PA	0.84025663	0.731959	0.9962802
HALLMARK_DNA_REPAIR	0.8122155	0.867211	1
HALLMARK_HEME_METABOLISM	0.74248844	0.945455	1
HALLMARK_PEROXISOME	0.73119754	0.91858	1
HALLMARK_OXIDATIVE_PHOSPHORYLATION	0.7179193	0.965691	1
HALLMARK_FATTY_ACID_METABOLISM	0.7099273	0.973442	1
HALLMARK_ADIPOGENESIS	0.67660904	0.984833	1
HALLMARK_PI3K_AKT_MTOR_SIGNALING	0.67036974	0.956933	0.99595803
HALLMARK_MTORC1_SIGNALING	0.5775876	1	0.99548405
HALLMARK_NOTCH_SIGNALING	-1.0093335	0.369863	0.42432967
HALLMARK_PANCREAS_BETA_CELLS	-1.0166521	0.397516	0.47668064
HALLMARK_ANGIOGENESIS	-1.0528208	0.333333	0.46028396
HALLMARK_GLYCOLYSIS	-1.1185268	0.125	0.38314503
HALLMARK_BILE_ACID_METABOLISM	-1.1580241	0.219512	0.3870173
HALLMARK_ESTROGEN_RESPONSE_EARLY	-1.2344588	0.083333	0.3876459
HALLMARK_CHOLESTEROL_HOMEOSTASIS	-1.4986923	0	0.09770502

Supplementary Table 6

Geneset - GOBP sets	NES	P	FDR q-val
GOBP_RESPONSE_TO_CHEMOKINE	1.91437	0	0.03384
GOBP_REGULATION_OF_ANTIGEN_PROCESSING_AND_PR	1.749203	0.001	0.63293
GOBP_ANIMAL_ORGAN_MATURATION	1.743728	0.002	0.46242
GOBP_REPLACEMENT_OSSIFICATION	1.714176	0	0.57721
GOBP_NEGATIVE_REGULATION_OF_COLD_INDUCED_THE	1.703185	0.002	0.55313
GOBP_LYMPHANGIOGENESIS	1.70314	0.005	0.46094
GOBP_CALCIUM_DEPENDENT_CELL_CELL_ADHESION_VIA	1.697478	0	0.43023
GOBP_GLIAL_CELL_APOPTOTIC_PROCESS	1.697343	0.001	0.3767
GOBP_LYMPH_VESSEL_MORPHOGENESIS	1.687463	0.004	0.39133
GOBP_REGULATION_OF_PLASMINOGEN_ACTIVATION	1.678483	0.004	0.40114
GOBP_POSITIVE_CHEMOTAXIS	1.678236	0	0.36594
GOBP_METANEPHRIC_GLOMERULUS_DEVELOPMENT	1.678089	0.003	0.33586
GOBP_NEUTROPHIL_CHEMOTAXIS	1.675808	0.001	0.32274
GOBP_REGULATION_OF_ODONTOGENESIS	1.674366	0.008	0.30637
GOBP_CEREBELLAR_CORTEX_MORPHOGENESIS	1.67269	0.001	0.29332
GOBP_RESPONSE_TO_VITAMIN_D	1.670869	0.001	0.28363
GOBP_REGULATION_OF_ASTROCYTE_DIFFERENTIATION	1.667895	0.002	0.2797
GOBP_HINDBRAIN_MORPHOGENESIS	1.662367	0	0.28666
GOBP_REGULATION_OF_OSTEOCLAST_DIFFERENTIATION	1.662257	0	0.27225
GOBP_NEGATIVE_REGULATION_OF_LEUKOCYTE_APOPTO	1.661645	0	0.26177
GOBP_CHONDROITIN_SULFATE_PROTEOGLYCAN_METABO	1.659243	0.007	0.25722
GOBP_REGULATION_OF_MYELOID_LEUKOCYTE_DIFFEREI	1.646705	0	0.29855
GOBP_DETECTION_OF_TEMPERATURE_STIMULUS	1.637488	0.01	0.33042
GOBP_WALKING_BEHAVIOR	1.635758	0.006	0.32341
GOBP_SENSORY_PERCEPTION_OF_TASTE	1.634985	0.001	0.31334
GOBP_CENTRAL_NERVOUS_SYSTEM_PROJECTION_NEUR	1.632504	0.006	0.31216
GOBP_BONE_MATURATION	1.632498	0.004	0.3006
GOBP_CELL_AGGREGATION	1.631478	0.008	0.29445
GOBP_NEUTROPHIL_MIGRATION	1.631166	0	0.28567
GOBP_OSTEOCLAST_DIFFERENTIATION	1.630586	0	0.27821
GOBP_REGULATION_OF_LEUKOCYTE_ADHESION_TO_VAS	1.625945	0.005	0.28762
GOBP_MEMBRANE_DEPOLARIZATION	1.622346	0	0.29322
GOBP_STEROID_CATABOLIC_PROCESS	1.615983	0.005	0.31054
GOBP_NEURON_MATURATION	1.615832	0.005	0.30223
GOBP_LEUKOCYTE_TETHERING_OR_ROLLING	1.614844	0.004	0.2976
GOBP_REGULATION_OF_PLATELET_ACTIVATION	1.60998	0	0.31092
GOBP_POSITIVE_REGULATION_OF_VASCULAR_ENDOTHE	1.606835	0.007	0.31604
GOBP_MEMBRANE_DEPOLARIZATION_DURING_ACTION_P	1.589454	0.007	0.39047
GOBP_POSITIVE_REGULATION_OF_TOR_SIGNALING	1.58736	0.009	0.39133
GOBP_EPHRIN_RECEPTOR_SIGNALING_PATHWAY	1.586553	0.002	0.38545
GOBP_LEUKOCYTE_ADHESION_TO_VASCULAR_ENDOTHE	1.581781	0.005	0.40063
GOBP_ESTABLISHMENT_OF_TISSUE_POLARITY	1.579129	0.003	0.4056
GOBP_PROTEIN_ACTIVATION_CASCADE	1.576183	0.008	0.41232
GOBP_POSITIVE_REGULATION_OF_ERK1_AND_ERK2_CAS	1.574575	0	0.41131
GOBP_POSITIVE_REGULATION_OF_CYTOSOLIC_CALCIUM	1.573469	0.008	0.40812
GOBP_INTERLEUKIN_2_PRODUCTION	1.573004	0.005	0.40174
GOBP_MYOBLAST_DIFFERENTIATION	1.572528	0.001	0.39594
GOBP_EPIDERMIS_MORPHOGENESIS	1.57154	0.011	0.393
GOBP_POSITIVE_REGULATION_OF_CELL_MIGRATION_INV	1.571171	0.012	0.38695
GOBP_CEREBRAL_CORTEX_NEURON_DIFFERENTIATION	1.569751	0.014	0.38641

GOBP_NEGATIVE_REGULATION_OF_MYOBLAST_DIFFEREI	1.568802	0.01	0.38315
GOBP_SPINAL_CORD_DEVELOPMENT	1.5685	0.002	0.37744
GOBP_CHONDROITIN_SULFATE_PROTEOGLYCAN_BIOSYN	1.568264	0.007	0.37124
GOBP_NEGATIVE_REGULATION_OF_OSTEOCLAST_DIFFEF	1.567459	0.012	0.36866
GOBP_HOMOPHILIC_CELL_ADHESION_VIA_PLASMA_MEME	1.566597	0	0.36639
GOBP_LYMPH_VESSEL_DEVELOPMENT	1.565614	0.008	0.36448
GOBP_COPULATION	1.565519	0.015	0.35868
GOBP_NEGATIVE_REGULATION_OF_B_CELL_ACTIVATION	1.565221	0.01	0.35379
GOBP_CENTRAL_NERVOUS_SYSTEM_NEURON_AXONOGE	1.563714	0.019	0.35493
GOBP_PIRNA_METABOLIC_PROCESS	1.563302	0.013	0.35093
GOBP_REGULATION_OF_PHOSPHOLIPASE_C_ACTIVITY	1.562543	0.009	0.34851
GOBP_POSITIVE_REGULATION_OF_BONE_MINERALIZATIO	1.562181	0.011	0.34429
GOBP_CELL_CELL_SIGNALING_INVOLVED_IN_CARDIAC_C	1.561925	0.006	0.33972
GOBP_APOPTOTIC_PROCESS_INVOLVED_IN_MORPHOGEI	1.559762	0.011	0.34382
GOBP_PROTEOGLYCAN_METABOLIC_PROCESS	1.559711	0.002	0.33869
GOBP_RETINA_VASCULATURE_DEVELOPMENT_IN_CAMEF	1.558847	0.016	0.33754
GOBP_POSITIVE_REGULATION_OF_LEUKOCYTE_ADHESIC	1.558629	0.015	0.33327
GOBP_MONOCYTE_CHEMOTAXIS	1.55816	0.004	0.3303
GOBP_CYCLIC_NUCLEOTIDE_BIOSYNTHETIC_PROCESS	1.556713	0.018	0.33131
GOBP_CELL_FATE_DETERMINATION	1.555704	0.012	0.33063
GOBP_SENSORY_PERCEPTION_OF_TEMPERATURE_STIMU	1.552351	0.012	0.34037
GOBP_PROXIMAL_DISTAL_PATTERN_FORMATION	1.550207	0.01	0.34391
GOBP_CELLULAR_RESPONSE_TO_INTERLEUKIN_1	1.545602	0.004	0.35845
GOBP_DICARBOXYLIC_ACID_CATABOLIC_PROCESS	1.545335	0.016	0.35479
GOBP_REGULATION_OF_MEMBRANE_DEPOLARIZATION	1.54525	0.01	0.35047
GOBP_RENAL_FILTRATION	1.544362	0.01	0.34976
GOBP_ASTROCYTE_DIFFERENTIATION	1.543338	0.005	0.34931
GOBP_REGULATION_OF_FIBROBLAST_GROWTH_FACTOR	1.542133	0.01	0.34954
GOBP_NEGATIVE_REGULATION_OF_COAGULATION	1.54097	0.006	0.34999
GOBP_POSITIVE_REGULATION_OF_MYELOID_LEUKOCYTE	1.540695	0.003	0.34687
GOBP_ANTIMICROBIAL_HUMORAL_RESPONSE	1.540267	0.01	0.34454
GOBP_POSITIVE_REGULATION_OF_ENDOTHELIAL_CELL_F	1.539928	0.001	0.34193
GOBP_CEREBELLAR_CORTEX_DEVELOPMENT	1.538013	0.009	0.34559
GOBP_NON_CANONICAL_WNT_SIGNALING_PATHWAY	1.537241	0.005	0.34468
GOBP_TONGUE_DEVELOPMENT	1.537123	0.023	0.34094
GOBP_RESPONSE_TO_NUTRIENT	1.536818	0	0.33844
GOBP_RESPONSE_TO_INTERLEUKIN_1	1.536283	0.001	0.33657
GOBP_DETECTION_OF_TEMPERATURE_STIMULUS_INVOL\	1.536264	0.016	0.33281
GOBP_NEUROINFLAMMATORY_RESPONSE	1.536014	0.021	0.33003
GOBP_INTERLEUKIN_10_PRODUCTION	1.531974	0.003	0.34291
GOBP_MORPHOGENESIS_OF_A_POLARIZED_EPITHELIUM	1.531347	0.003	0.34179
GOBP_PHOSPHOLIPASE_C_ACTIVATING_G_PROTEIN_COU	1.530831	0.007	0.34009
GOBP_CENTRAL_NERVOUS_SYSTEM_NEURON_DIFFEREN	1.530191	0.001	0.33905
GOBP_VASCULAR_WOUND_HEALING	1.528835	0.026	0.34088
GOBP_VASCULAR_ENDOTHELIAL_GROWTH_FACTOR_SIGI	1.528709	0.01	0.33775
GOBP_NEURONAL_ACTION_POTENTIAL	1.527672	0.022	0.33853
GOBP_CHONDROITIN_SULFATE_BIOSYNTHETIC_PROCESS	1.527096	0.019	0.33723
GOBP_ANGIOGENESIS_INVOLVED_IN_WOUND_HEALING	1.524231	0.016	0.34527
GOBP_RESPONSE_TO_AMYLOID_BETA	1.524039	0.008	0.34259
GOBP_MODULATION_BY_HOST_OF_SYMBIONT_PROCESS	1.522841	0.009	0.34375
GOBP_REFLEX	1.52268	0.019	0.34103
GOBP_RENAL_SYSTEM_VASCULATURE_DEVELOPMENT	1.52225	0.009	0.33954
GOBP_RETINOIC_ACID_METABOLIC_PROCESS	1.522192	0.012	0.33642

GOBP_LEUKOCYTE_MIGRATION_INVOLVED_IN_INFLAMMA	1.521241	0.021	0.3373
GOBP_CELLULAR_RESPONSE_TO_INTERFERON_GAMMA	1.520662	0.005	0.3363
GOBP_APOPTOTIC_PROCESS_INVOLVED_IN_DEVELOPME	1.520356	0.009	0.33438
GOBP_REGULATION_OF_GLIAL_CELL_DIFFERENTIATION	1.520342	0.006	0.33131
GOBP_CENTRAL_NERVOUS_SYSTEM_NEURON_DEVELOPI	1.519904	0.012	0.32979
GOBP_ANTIMICROBIAL_HUMORAL_IMMUNE_RESPONSE_M	1.517895	0.01	0.33429
GOBP_NEGATIVE_REGULATION_OF_SMOOTH_MUSCLE_C	1.51657	0.027	0.33638
GOBP_REGULATION_OF_HOMOTYPIC_CELL_CELL_ADHES	1.515026	0.021	0.33905
GOBP_NEGATIVE_REGULATION_OF_TUMOR_NECROSIS_F	1.514467	0.008	0.33834
GOBP_REGULATION_OF_MYOBLAST_FUSION	1.514199	0.018	0.33644
GOBP_NEGATIVE_REGULATION_OF_MYELOID_LEUKOCYT	1.513383	0.019	0.33684
GOBP_GRANULOCYTE_CHEMOTAXIS	1.511951	0.004	0.33914
GOBP_RESPONSE_TO_LITHIUM_ION	1.511127	0.02	0.33946
GOBP_ACTIVATION_OF_PHOSPHOLIPASE_C_ACTIVITY	1.506809	0.023	0.35418
GOBP_SENSORY_PERCEPTION_OF_CHEMICAL_STIMULUS	1.506242	0.011	0.35343
GOBP_REGULATION_OF_SKELETAL_MUSCLE_TISSUE_DEV	1.506099	0.029	0.35101
GOBP_POSITIVE_REGULATION_OF_REGULATED_SECRETO	1.504178	0.013	0.35597
GOBP_TERPENOID_METABOLIC_PROCESS	1.503352	0.011	0.35631
GOBP_REGULATION_OF_ESTABLISHMENT_OF_PLANAR_P	1.503111	0.013	0.35436
GOBP_CELL_JUNCTION_MAINTENANCE	1.502684	0.017	0.35298
GOBP_THYMIC_T_CELL_SELECTION	1.501169	0.03	0.3562
GOBP_CHONDROCYTE_DIFFERENTIATION	1.500764	0.002	0.3551
GOBP_REGULATION_OF_MYOBLAST_DIFFERENTIATION	1.500211	0.016	0.35475
GOBP_REGULATION_OF_T_HELPER_1_TYPE_IMMUNE_RES	1.500142	0.028	0.35219
GOBP_CELL_CELL_ADHESION_VIA_PLASMA_MEMBRANE_A	1.499119	0	0.35359
GOBP_METANEPHRIC_NEPHRON_DEVELOPMENT	1.498598	0.017	0.35298
GOBP_NEGATIVE_REGULATION_OF_INTERLEUKIN_2_PRO	1.495684	0.037	0.3619
GOBP_POSITIVE_REGULATION_OF_INTERLEUKIN_10_PRO	1.494932	0.018	0.36216
GOBP_MYELOID_LEUKOCYTE_MIGRATION	1.493737	0.001	0.36416
GOBP_KERATINIZATION	1.493355	0.013	0.36284
GOBP_CHONDROCYTE_PROLIFERATION	1.493189	0.035	0.36089
GOBP_CHEMOSENSORY_BEHAVIOR	1.492995	0.032	0.35896
GOBP_NEGATIVE_REGULATION_OF_BONE_REMODELING	1.492166	0.031	0.35977
GOBP_DORSAL_VENTRAL_PATTERN_FORMATION	1.491912	0.01	0.35825
GOBP_INFLAMMATORY_CELL_APOPTOTIC_PROCESS	1.491866	0.033	0.35581
GOBP_HETEROPHILIC_CELL_CELL_ADHESION_VIA_PLASM	1.491546	0.024	0.35452
GOBP_GRANULOCYTE_MIGRATION	1.488814	0.003	0.36271
GOBP_INTERFERON_ALPHA_PRODUCTION	1.488038	0.025	0.36341
GOBP_POSITIVE_REGULATION_OF_PHAGOCYTOSIS	1.487885	0.008	0.3614
GOBP_CELLULAR_RESPONSE_TO_MOLECULE_OF_BACTE	1.485572	0.002	0.36773
GOBP_REGULATION_OF_OSTEOBLAST_PROLIFERATION	1.483578	0.033	0.37328
GOBP_MEMORY	1.48211	0.003	0.3767
GOBP_PROTEIN_KINASE_A_SIGNALING	1.481237	0.017	0.37779
GOBP_STEM_CELL_DIVISION	1.480506	0.025	0.37808
GOBP_SYNAPTIC_TRANSMISSION_CHOLINERGIC	1.480488	0.032	0.37556
GOBP_REGULATION_OF_COLLATERAL_SPROUTING	1.480081	0.027	0.37489
GOBP_REGULATION_OF_MUSCLE_ORGAN_DEVELOPMENT	1.475485	0.023	0.39111
GOBP_NEGATIVE_REGULATION_OF_MYELOID_CELL_DIFFI	1.475415	0.007	0.38883
GOBP_G_PROTEIN_COUPLED_ACETYLCHOLINE_RECEPTO	1.475307	0.038	0.38677
GOBP_RESPONSE_TO_MOLECULE_OF_BACTERIAL_ORIGI	1.474618	0	0.38715
GOBP_ENDOTHELIAL_CELL_PROLIFERATION	1.473381	0.005	0.38984
GOBP_ANATOMICAL_STRUCTURE_ARRANGEMENT	1.471475	0.038	0.39543
GOBP_NEGATIVE_REGULATION_OF_INTRINSIC_APOPTOT	1.46996	0.022	0.39936

GOBP_REGULATION_OF_CELLULAR_EXTRAVASATION	1.469561	0.021	0.39852
GOBP_NEGATIVE_REGULATION_OF_HEMOPOIESIS	1.468542	0.007	0.40037
GOBP_POSITIVE_REGULATION_OF_PHOSPHOLIPASE_ACT	1.468234	0.016	0.3992
GOBP_POSITIVE_REGULATION_OF_EPIDERMIS_DEVELOPI	1.466583	0.04	0.40378
GOBP_POSITIVE_REGULATION_OF_PATTERN_RECOGNITI(	1.466226	0.021	0.40281
GOBP_NERVE_DEVELOPMENT	1.466179	0.009	0.40052
GOBP_SENSORY_PERCEPTION_OF_PAIN	1.465727	0.007	0.39995
GOBP_BLOOD_VESSEL_ENDOTHELIAL_CELL_PROLIFERAT	1.46495	0.035	0.40077
GOBP_POSITIVE_REGULATION_OF_SYNCYTIUM_FORMATI	1.463742	0.039	0.40355
GOBP_CELLULAR_RESPONSE_TO_THYROID_HORMONE_S	1.462888	0.035	0.40466
GOBP_POSTSYNAPTIC_SPECIALIZATION_ORGANIZATION	1.46113	0.038	0.40997
GOBP_POSITIVE_REGULATION_OF_MIRNA_TRANSCRIPTIC	1.460964	0.032	0.40817
GOBP_DETECTION_OF_TEMPERATURE_STIMULUS_INVOL\	1.460452	0.045	0.40778
GOBP_POSITIVE_REGULATION_OF_EPITHELIAL_CELL_PR(	1.460201	0.002	0.40645
GOBP_POSITIVE_REGULATION_OF_MUSCLE_TISSUE_DEV	1.459858	0.035	0.40551
GOBP_SNRNA_TRANSCRIPTION	1.459475	0.059	0.40483
GOBP_TEMPERATURE_HOMEOSTASIS	1.458604	0.002	0.40636
GOBP_TRACHEA_DEVELOPMENT	1.455329	0.046	0.41799
GOBP_REGULATION_OF_BONE_REMODELING	1.454546	0.028	0.41881
GOBP_CALCIUM_MEDIATED_SIGNALING_USING_INTRACEL	1.454434	0.04	0.41691
GOBP_HUMORAL_IMMUNE_RESPONSE	1.454342	0.005	0.41501
GOBP_INFLAMMATORY_RESPONSE_TO_ANTIGENIC_STIMU	1.453776	0.019	0.41526
GOBP_LYMPHOID_PROGENITOR_CELL_DIFFERENTIATION	1.453481	0.05	0.41421
GOBP_POSITIVE_REGULATION_OF_CHEMOKINE_PRODUC	1.451379	0.027	0.42066
GOBP_POSTSYNAPTIC_MEMBRANE_ORGANIZATION	1.451194	0.036	0.4192
GOBP_LEUKOCYTE_APOPTOTIC_PROCESS	1.450844	0.008	0.41818
GOBP_PH_REDUCTION	1.450655	0.031	0.41685
GOBP_POSITIVE_REGULATION_OF_RECEPTOR_MEDIATEI	1.450447	0.022	0.41554
GOBP_REGULATION_OF_HUMORAL_IMMUNE_RESPONSE	1.448585	0.034	0.4215
GOBP_POSTSYNAPTIC_SIGNAL_TRANSDUCTION	1.448144	0.032	0.42102
GOBP_REGULATION_OF_SYNCYTIUM_FORMATION_BY_PL	1.447849	0.043	0.42017
GOBP_REGULATION_OF_T_CELL_DIFFERENTIATION_IN_TI	1.446927	0.041	0.4218
GOBP_DETECTION_OF_STIMULUS_INVOLVED_IN_SENSOR	1.445905	0.048	0.42374
GOBP_RESPONSE_TO_FOLLICLE_STIMULATING_HORMON	1.4455	0.045	0.42317
GOBP_EMBRYONIC_DIGIT_MORPHOGENESIS	1.444848	0.023	0.42387
GOBP_POSITIVE_REGULATION_BY_HOST_OF_VIRAL_TRAI	1.444378	0.058	0.42375
GOBP_REGULATION_OF_PROTEIN_MATURATION	1.444039	0.013	0.42314
GOBP_GLIAL_CELL_MIGRATION	1.44326	0.028	0.42429
GOBP_REGULATION_OF_LEUKOCYTE_APOPTOTIC_PROCI	1.442432	0.022	0.42542
GOBP_RESPONSE_TO_ARSENIC_CONTAINING_SUBSTANC	1.441583	0.036	0.42697
GOBP_NEGATIVE_REGULATION_OF_PLATELET_ACTIVATI(	1.44153	0.059	0.42503
GOBP_SECRETORY_GRANULE_ORGANIZATION	1.441493	0.047	0.42303
GOBP_POSITIVE_REGULATION_OF_OSTEOCLAST_DIFFER	1.439377	0.048	0.43002
GOBP_POSITIVE_REGULATION_OF_PEPTIDYL_TYROSINE_	1.439254	0.002	0.42833
GOBP_PROSTAGLANDIN_TRANSPORT	1.438354	0.05	0.4301
GOBP_RESPIRATORY_GASEOUS_EXCHANGE_BY_RESPIR/	1.43792	0.019	0.42985
GOBP_NEGATIVE_REGULATION_OF_SMALL_GTPASE_MED	1.437348	0.025	0.43011
GOBP_DETECTION_OF_STIMULUS_INVOLVED_IN_SENSOR	1.436672	0.011	0.43104
GOBP_AXONEME_ASSEMBLY	1.43646	0.014	0.42974
GOBP_REGULATION_OF_PLATELET_AGGREGATION	1.436241	0.049	0.42859
GOBP_REGULATION_OF_BMP_SIGNALING_PATHWAY	1.435947	0.007	0.42777
GOBP_INNERVATION	1.435745	0.035	0.42662
GOBP_RESPONSE_TO_DSRNA	1.435221	0.038	0.42694

GOBP_REGULATION_OF_MYELOID_CELL_DIFFERENTIATIO	1.434659	0.005	0.42726
GOBP_ASSOCIATIVE_LEARNING	1.433786	0.021	0.42907
GOBP_RESPONSE_TO_VITAMIN	1.432503	0.012	0.43251
GOBP_REGULATION_OF_BONE_RESORPTION	1.432078	0.037	0.43244
GOBP_MITOCHONDRIAL_RNA_METABOLIC_PROCESS	1.431559	0.024	0.43256
GOBP_CELLULAR_RESPONSE_TO_PROSTAGLANDIN_STIM	1.430673	0.044	0.43417
GOBP_REGULATION_OF_FEEDING_BEHAVIOR	1.430591	0.055	0.4325
GOBP_REGULATION_OF_TISSUE_REMODELING	1.429969	0.017	0.43303
GOBP_REGULATION_OF_EPIDERMIS_DEVELOPMENT	1.429728	0.029	0.43201
GOBP_NEGATIVE_REGULATION_OF_BLOOD_PRESSURE	1.42942	0.046	0.43131
GOBP_REGULATION_OF_NON_CANONICAL_WNT_SIGNALI	1.429123	0.054	0.43075
GOBP_NEGATIVE_REGULATION_OF_CELL_ACTIVATION	1.428204	0.004	0.43268
GOBP_ANIMAL_ORGAN_REGENERATION	1.428141	0.021	0.43103
GOBP_COLLATERAL_SPROUTING	1.427191	0.044	0.43311
GOBP_GLOMERULUS_DEVELOPMENT	1.427156	0.025	0.43133
GOBP_REGULATION_OF_CARDIOCYTE_DIFFERENTIATION	1.426856	0.043	0.4307
GOBP_RETINOL_METABOLIC_PROCESS	1.425927	0.043	0.43277
GOBP_CEREBRAL_CORTEX_RADIALLY_ORIENTED_CELL_M	1.425311	0.037	0.4335
GOBP_NEGATIVE_REGULATION_OF_PROTEIN_MATURATIO	1.424879	0.053	0.43345
GOBP_CELL_JUNCTION_DISASSEMBLY	1.424757	0.049	0.43207
GOBP_REGULATION_OF_INTRINSIC_APOPTOTIC_SIGNALIN	1.424644	0.041	0.43069
GOBP_INFLAMMASOME_COMPLEX_ASSEMBLY	1.423951	0.041	0.43163
GOBP_REGULATION_OF_CELL_MATURATION	1.423827	0.051	0.43028
GOBP_VASCULAR_ENDOTHELIAL_GROWTH_FACTOR_PRO	1.423696	0.04	0.42902
GOBP_HEMATOPOIETIC_PROGENITOR_CELL_DIFFERENTI	1.422687	0.009	0.4314
GOBP_REGULATION_OF_ACTIN_CYTOSKELETON_REORGA	1.42167	0.034	0.43398
GOBP_REGULATION_OF_ANIMAL_ORGAN_MORPHOGENES	1.421113	0.012	0.43452
GOBP_ADAPTIVE_THERMOGENESIS	1.421034	0.005	0.43295
GOBP_POSITIVE_REGULATION_OF_INTERFERON_BETA_P	1.420727	0.036	0.43245
GOBP_POSITIVE_REGULATION_OF_WOUND_HEALING	1.420582	0.028	0.43122
GOBP_DEFENSE_RESPONSE_TO_GRAM_NEGATIVE_BACT	1.419421	0.042	0.43446
GOBP_INTERFERON_BETA_PRODUCTION	1.418199	0.033	0.43757
GOBP_POSITIVE_REGULATION_OF_INTERFERON_ALPHA_	1.41762	0.054	0.43808
GOBP_NEGATIVE_REGULATION_OF_REPRODUCTIVE_PRO	1.417522	0.033	0.43674
GOBP_CELLULAR_RESPONSE_TO_BIOTIC_STIMULUS	1.417257	0.002	0.43598
GOBP_ISOPRENOID_METABOLIC_PROCESS	1.41702	0.019	0.43522
GOBP_INOSITOL_LIPID_MEDIATED_SIGNALING	1.416374	0.004	0.43617
GOBP_EPITHELIAL_CELL_DIFFERENTIATION_INVOLVED_IN	1.416335	0.038	0.43455
GOBP_REGULATION_OF_INFLAMMATORY_RESPONSE_TO_	1.41609	0.034	0.43386
GOBP_MYELOID_CELL_APOPTOTIC_PROCESS	1.415981	0.039	0.43248
GOBP_CELLULAR_MODIFIED_AMINO_ACID_CATABOLIC_PR	1.414853	0.059	0.43548
GOBP_ACROSOME_REACTION	1.414127	0.061	0.43687
GOBP_REGULATION_OF_SYNAPTIC_VESICLE_EXOCYTOSI	1.413981	0.037	0.43576
GOBP_NEGATIVE_REGULATION_OF_T_CELL_DIFFERENTIA	1.413832	0.043	0.43463
GOBP_REGULATION_OF_POSITIVE_CHEMOTAXIS	1.413804	0.05	0.43302
GOBP_POSITIVE_REGULATION_OF_RESPONSE_TO_CYTO	1.41347	0.034	0.43266
GOBP_CHEMOKINE_PRODUCTION	1.413147	0.021	0.43229
GOBP_NEGATIVE_REGULATION_OF_EPITHELIAL_CELL_DIF	1.412943	0.038	0.43149
GOBP_REGULATION_OF_PHOSPHATIDYLINOSITOL_3_KINA	1.4129	0.023	0.42998
GOBP_CELLULAR_HORMONE_METABOLIC_PROCESS	1.412759	0.017	0.42892
GOBP_NEGATIVE_REGULATION_OF_INFLAMMATORY_RES	1.412268	0.061	0.4294
GOBP_CELLULAR_EXTRAVASATION	1.412231	0.034	0.42792
GOBP_POSITIVE_REGULATION_OF_SYNAPTIC_TRANSMISS	1.410891	0.051	0.43175

GOBP_NEGATIVE_REGULATION_OF_LEUKOCYTE_MEDIATI	1.410463	0.027	0.43177
GOBP_POSITIVE_REGULATION_OF_EPIDERMAL_CELL_DIF	1.409582	0.057	0.43359
GOBP_MATING	1.408801	0.063	0.43516
GOBP_CELL_CHEMOTAXIS	1.408741	0	0.43376
GOBP_CELL_CELL_ADHESION_MEDIATED_BY_INTEGRIN	1.40791	0.058	0.43551
GOBP_GLIOGENESIS	1.407847	0	0.43413
GOBP_POSITIVE_REGULATION_OF_INTERLEUKIN_12_PRO	1.406696	0.05	0.43726
GOBP_CELL_REDOX_HOMEOSTASIS	1.406645	0.045	0.43587
GOBP_REGULATION_OF_COAGULATION	1.406587	0.022	0.43451
GOBP_TRNA_MODIFICATION	1.406405	0.019	0.43372
GOBP_BLASTOCYST_GROWTH	1.406397	0.063	0.43218
GOBP_MAINTENANCE_OF_SYNAPSE_STRUCTURE	1.40592	0.067	0.43257
GOBP_MODULATION_BY_HOST_OF_VIRAL_PROCESS	1.405155	0.064	0.43404
GOBP_NEGATIVE_REGULATION_OF_LYMPHOCYTE_APOPT	1.404701	0.057	0.43443
GOBP_RESPONSE_TO_IONIZING_RADIATION	1.40465	0.005	0.433
GOBP_MATURATION_OF_LSU_RRNA	1.404401	0.066	0.43247
GOBP_OSTEOCLAST_DEVELOPMENT	1.404362	0.044	0.43106
GOBP_MORPHOGENESIS_OF_AN_ENDOTHELIUM	1.40423	0.064	0.43007
GOBP_XENOBIOTIC_METABOLIC_PROCESS	1.403779	0.029	0.43041
GOBP_DETECTION_OF_STIMULUS	1.403367	0.003	0.43049
GOBP_EATING_BEHAVIOR	1.402671	0.058	0.43168
GOBP_PHOTOTRANSDUCTION	1.401856	0.058	0.4337
GOBP_POSITIVE_REGULATION_OF_RESPONSE_TO_WOUN	1.401126	0.036	0.43523
GOBP_NEGATIVE_REGULATION_OF_TYPE_I_INTERFERON	1.401122	0.052	0.43371
GOBP_MEMBRANE_DEPOLARIZATION_DURING_CARDIAC_	1.400008	0.044	0.43668
GOBP_NEGATIVE_REGULATION_OF_RESPONSE_TO_WOU	1.399719	0.023	0.43638
GOBP_SECONDARY_METABOLIC_PROCESS	1.399565	0.049	0.43554
GOBP_POSITIVE_REGULATION_OF_CREB_TRANSCRIPTIO	1.399442	0.067	0.4346
GOBP_CELLULAR_RESPONSE_TO_VASCULAR_ENDOTHEL	1.39757	0.04	0.44075
GOBP_REGULATION_OF_CHONDROCYTE_DIFFERENTIATI	1.397559	0.038	0.43927
GOBP_REGULATION_OF_MICROGLIAL_CELL_ACTIVATION	1.397545	0.087	0.43781
GOBP_ASTROCYTE_DEVELOPMENT	1.397387	0.057	0.43691
GOBP_LEARNING	1.397263	0.014	0.43598
GOBP_HINDBRAIN_DEVELOPMENT	1.397254	0.006	0.43456
GOBP_NEGATIVE_REGULATION_OF_CELL_DEVELOPMENT	1.396832	0.008	0.4348
GOBP_POSITIVE_REGULATION_OF_LIPASE_ACTIVITY	1.396073	0.043	0.43636
GOBP_GLIAL_CELL_DEVELOPMENT	1.395722	0.017	0.43635
GOBP_POSITIVE_REGULATION_OF_VASCULATURE_DEVEL	1.395685	0.012	0.43507
GOBP_PROTEIN_TARGETING_TO_LYSOSOME	1.394322	0.066	0.43915
GOBP_ADULT_LOCOMOTORY_BEHAVIOR	1.394284	0.027	0.43784
GOBP_DETECTION_OF_MECHANICAL_STIMULUS_INVOLVE	1.394016	0.049	0.43748
GOBP_REGULATION_OF_CELL_ADHESION_MEDIATED_BY_	1.393944	0.045	0.43635
GOBP_EMBRYONIC_HINDLIMB_MORPHOGENESIS	1.393683	0.066	0.43609
GOBP_MATURATION_OF_LSU_RRNA_FROM_TRICISTRONI	1.393492	0.075	0.43541
GOBP_POSITIVE_REGULATION_OF_ADAPTIVE_IMMUNE_R	1.39322	0.027	0.43501
GOBP_LEUKOCYTE_MIGRATION	1.393098	0.001	0.43411
GOBP_POSITIVE_REGULATION_OF_LIPID_KINASE_ACTIVIT	1.393002	0.057	0.43307
GOBP_POSITIVE_REGULATION_OF_FATTY_ACID_METABO	1.39289	0.05	0.43208
GOBP_MESENCHYME_MORPHOGENESIS	1.392585	0.031	0.43189
GOBP_REGULATION_OF_CELLULAR_RESPONSE_TO_GRO	1.392477	0.002	0.4309
GOBP_EMBRYONIC_APPENDAGE_MORPHOGENESIS	1.392338	0.016	0.43015
GOBP_POSITIVE_REGULATION_OF_BIOMINERALIZATION	1.392254	0.032	0.42915
GOBP_REGULATION_OF_LIPOPOLYSACCHARIDE_MEDIATE	1.392142	0.065	0.42828

GOBP_REGULATION_OF_REGULATED_SECRETORY_PATH	1.391365	0.01	0.42997
GOBP_POSTSYNAPSE_ASSEMBLY	1.391359	0.073	0.42863
GOBP_MRNA_TRANSCRIPTION	1.390983	0.063	0.4288
GOBP_VENTRAL_SPINAL_CORD_DEVELOPMENT	1.390461	0.05	0.42941
GOBP_MACROPHAGE_DIFFERENTIATION	1.390422	0.046	0.42825
GOBP_NEGATIVE_REGULATION_OF_SMOOTH_MUSCLE_CI	1.390335	0.041	0.42729
GOBP_NEURON_MIGRATION	1.389999	0.012	0.42727
GOBP_NEGATIVE_REGULATION_OF_INTRINSIC_APOPTOTI	1.3896	0.067	0.42749
GOBP_RESPONSE_TO_THYROID_HORMONE	1.389554	0.065	0.42637
GOBP_CELL_DIFFERENTIATION_INVOLVED_IN_METANEPH	1.389049	0.065	0.42718
GOBP_BONE_REMODELING	1.387614	0.032	0.43162
GOBP_GLIAL_CELL_DIFFERENTIATION	1.387405	0.003	0.43108
GOBP_POSITIVE_REGULATION_OF_TOLL_LIKE_RECEPTOF	1.387396	0.064	0.4298
GOBP_TRANSMISSION_OF_NERVE_IMPULSE	1.386694	0.039	0.43143
GOBP_DEFENSE_RESPONSE_TO_BACTERIUM	1.385202	0.008	0.43591
GOBP_METANEPHROS_DEVELOPMENT	1.384659	0.031	0.43667
GOBP_RESPONSE_TO_PH	1.384008	0.064	0.43795
GOBP_LIVER_REGENERATION	1.38281	0.063	0.44135
GOBP_VASCULAR_ENDOTHELIAL_CELL_PROLIFERATION	1.381965	0.083	0.44338
GOBP_SPERM_FLAGELLUM_ASSEMBLY	1.380498	0.049	0.44791
GOBP_OSTEOBLAST_PROLIFERATION	1.38049	0.071	0.4466
GOBP_POSITIVE_REGULATION_OF_PROTEIN_TYROSINE_I	1.380316	0.043	0.44588
GOBP_POSTSYNAPSE_ORGANIZATION	1.37955	0.016	0.44757
GOBP_WATER_TRANSPORT	1.379543	0.068	0.44628
GOBP_ESTROGEN_METABOLIC_PROCESS	1.379293	0.073	0.4459
GOBP_NEGATIVE_REGULATION_OF_CELL_SUBSTRATE_AI	1.379232	0.048	0.44485
GOBP_ODONTOGENESIS	1.37893	0.02	0.44475
GOBP_VACUOLAR_ACIDIFICATION	1.377883	0.078	0.4477
GOBP_RESPONSE_TO_GAMMA_RADIATION	1.377424	0.04	0.44823
GOBP_INNATE_IMMUNE_RESPONSE_ACTIVATING_SIGNAL	1.376319	0.084	0.45123
GOBP_POSITIVE_REGULATION_OF_OSSIFICATION	1.375907	0.058	0.45155
GOBP_BIOLOGICAL_PROCESS_INVOLVED_IN_INTERACTIC	1.375886	0.036	0.45035
GOBP_DENDRITIC_SPINE_MORPHOGENESIS	1.37548	0.035	0.45051
GOBP_TOLERANCE_INDUCTION	1.3754	0.06	0.44956
GOBP_REGULATION_OF_CARTILAGE_DEVELOPMENT	1.375275	0.036	0.44872
GOBP_POSITIVE_REGULATION_OF_LEUKOCYTE_CELL_CE	1.374282	0.01	0.45148
GOBP_CEREBRAL_CORTEX_CELL_MIGRATION	1.373732	0.058	0.4525
GOBP_NEGATIVE_REGULATION_OF_NEURON_DEATH	1.37365	0.001	0.4515
GOBP_NEGATIVE_REGULATION_OF_INNATE_IMMUNE_RES	1.373229	0.047	0.45191
GOBP_TYPE_I_INTERFERON_PRODUCTION	1.372936	0.023	0.45184
GOBP_L_GLUTAMATE_IMPORT_ACROSS_PLASMA_MEMBR	1.37287	0.083	0.45086
GOBP_CEREBELLAR_CORTEX_FORMATION	1.372621	0.064	0.45057
GOBP_GLIAL_CELL_PROLIFERATION	1.371669	0.063	0.4532
GOBP_NEGATIVE_REGULATION_OF_ATP_DEPENDENT_AC	1.37152	0.093	0.45256
GOBP_RETINAL_GANGLION_CELL_AXON_GUIDANCE	1.371053	0.067	0.45308
GOBP_ERK1_AND_ERK2_CASCADE	1.369974	0.003	0.45616
GOBP_AXON_DEVELOPMENT	1.369851	0.001	0.45538
GOBP_POSITIVE_REGULATION_OF_DNA_REPLICATION	1.369704	0.058	0.45472
GOBP_T_CELL_DIFFERENTIATION_IN_THYMUS	1.36951	0.04	0.45427
GOBP_TAXIS	1.369489	0	0.45311
GOBP_POSITIVE_REGULATION_OF_CELL_CELL_ADHESION	1.369047	0.004	0.45379
GOBP_NEGATIVE_REGULATION_OF_ENDOTHELIAL_CELL_	1.36841	0.08	0.45512
GOBP_CELLULAR_RESPONSE_TO_XENOBIOTIC_STIMULUS	1.368166	0.02	0.4549

GOBP_REGULATION_OF_STRIATED_MUSCLE_CELL_DIFFE	1.367522	0.046	0.45614
GOBP_BILE_ACID_AND_BILE_SALT_TRANSPORT	1.367334	0.082	0.45568
GOBP_POSITIVE_REGULATION_OF_INTERLEUKIN_2_PROD	1.367305	0.081	0.45453
GOBP_REGULATION_OF_DOUBLE_STRAND_BREAK_REPAI	1.366598	0.087	0.45608
GOBP_SECONDARY_PALATE_DEVELOPMENT	1.366575	0.078	0.45496
GOBP_RESPONSE_TO_X_RAY	1.36623	0.07	0.45516
GOBP_REGULATION_OF_CELLULAR_PH	1.365958	0.045	0.45506
GOBP_PTERIDINE_CONTAINING_COMPOUND_METABOLIC_	1.365577	0.06	0.45541
GOBP_REGULATION_OF_BLOOD_PRESSURE	1.365446	0.012	0.45474
GOBP_NEGATIVE_REGULATION_OF_VASCULAR_PERMEAE	1.365405	0.077	0.45367
GOBP_FOLIC_ACID_CONTAINING_COMPOUND_METABOLIC	1.364656	0.099	0.45537
GOBP_POSITIVE_REGULATION_OF_MACROPHAGE_MIGRA	1.364005	0.087	0.45666
GOBP_NEGATIVE_REGULATION_OF_MONONUCLEAR_CELI	1.363927	0.079	0.45579
GOBP_MYELOID_LEUKOCYTE_DIFFERENTIATION	1.363435	0.008	0.4565
GOBP_LEUKOCYTE_CHEMOTAXIS	1.362777	0.007	0.45797
GOBP_RESPONSE_TO_BMP	1.362388	0.012	0.45841
GOBP_REGULATION_OF_CELL_SUBSTRATE_JUNCTION_O	1.362113	0.043	0.45823
GOBP_NEGATIVE_REGULATION_OF_NERVOUS_SYSTEM_D	1.362051	0.023	0.4573
GOBP_POSITIVE_REGULATION_OF_RECEPTOR_INTERNAL	1.361609	0.082	0.45789
GOBP_POSITIVE_REGULATION_OF_COAGULATION	1.361582	0.083	0.45683
GOBP_NEGATIVE_REGULATION_OF_MUSCLE_CELL_DIFFE	1.361567	0.058	0.45573
GOBP_NEGATIVE_REGULATION_OF_STEROID_METABOLIC	1.361537	0.083	0.45468
GOBP_URETER_DEVELOPMENT	1.361058	0.093	0.45539
GOBP_SEGMENTATION	1.360287	0.031	0.45719
GOBP_SENSORY_PERCEPTION	1.359932	0	0.45744
GOBP_REGULATION_OF_MAST_CELL_ACTIVATION_INVOLV	1.359139	0.079	0.45948
GOBP_POSITIVE_REGULATION_OF_MYOBLAST_DIFFEREN	1.3588	0.104	0.45968
GOBP_CALCIUM_ION_REGULATED_EXOCYTOSIS	1.35877	0.042	0.4586
GOBP_SENSORY_PERCEPTION_OF_MECHANICAL_STIMUL	1.358672	0.013	0.45785
GOBP_NEURON_PROJECTION_GUIDANCE	1.358663	0.006	0.45671
GOBP_DETECTION_OF_ABIOTIC_STIMULUS	1.357696	0.025	0.45941
GOBP_REGULATION_OF_CELL_CELL_ADHESION	1.357695	0.003	0.45826
GOBP_REGULATION_OF_ENDOCYTIC_RECYCLING	1.357688	0.092	0.45714
GOBP_NEGATIVE_REGULATION_OF_SYSTEMIC_ARTERIAL	1.356563	0.111	0.46052
GOBP_RESPONSE_TO_LEUKEMIA_INHIBITORY_FACTOR	1.355567	0.043	0.46324
GOBP_MITOCHONDRIAL_GENE_EXPRESSION	1.355367	0.028	0.46295
GOBP_RESPONSE_TO_INTERFERON_GAMMA	1.354999	0.025	0.46323
GOBP_REGULATION_OF_BONE_MINERALIZATION	1.354526	0.046	0.46394
GOBP_RESPONSE_TO_XENOBIOTIC_STIMULUS	1.353714	0.001	0.46604
GOBP_REGULATION_OF_MYELOID_CELL_APOPTOTIC_PR(	1.353599	0.083	0.46539
GOBP_REGULATION_OF_B_CELL_PROLIFERATION	1.353424	0.058	0.46493
GOBP_REGULATION_OF_MUSCLE_CELL_DIFFERENTIATIOI	1.353421	0.027	0.4638
GOBP_MATURATION_OF_SSU_RRNA	1.353098	0.054	0.46396
GOBP_REGULATION_OF_EXTRINSIC_APOPTOTIC_SIGNALI	1.352688	0.067	0.46444
GOBP_SPROUTING_ANGIOGENESIS	1.35263	0.02	0.46356
GOBP_NEGATIVE_REGULATION_OF_CARTILAGE_DEVELOI	1.352546	0.072	0.46278
GOBP_METENCEPHALON_DEVELOPMENT	1.35188	0.031	0.46431
GOBP_REGULATION_OF_B_CELL_APOPTOTIC_PROCESS	1.351244	0.088	0.46576
GOBP_TISSUE_REMODELING	1.350956	0.018	0.46576
GOBP_REGULATION_OF_CENTRIOLE_REPLICATION	1.350457	0.088	0.4666
GOBP_EMBRYO_IMPLANTATION	1.350457	0.065	0.46549
GOBP_POSITIVE_REGULATION_OF_NUCLEOTIDE_BIOSYNT	1.349906	0.09	0.46669
GOBP_REGULATION_OF_INTEGRIN_ACTIVATION	1.34978	0.101	0.46612

GOBP_RNA_METHYLATION	1.349518	0.05	0.466
GOBP_POSITIVE_REGULATION_OF_RESPONSE_TO_BIOTI(	1.349429	0.02	0.46527
GOBP_CELL_ADHESION_MEDIATED_BY_INTEGRIN	1.349323	0.038	0.46458
GOBP_POSITIVE_REGULATION_OF_NEURON_PROJECTIOI	1.348886	0.013	0.46521
GOBP_RESPONSE_TO_TUMOR_NECROSIS_FACTOR	1.348787	0.009	0.46455
GOBP_RIBOSOME_BIOGENESIS	1.348554	0.002	0.46441
GOBP_PHOSPHATIDYLINOSITOL_3_KINASE_SIGNALING	1.347338	0.026	0.4681
GOBP_REGULATION_OF_POSTSYNAPSE_ORGANIZATION	1.347338	0.039	0.46701
GOBP_POSITIVE_REGULATION_OF_VASCULAR_ENDOTHE	1.347278	0.094	0.46615
GOBP_DNA_METHYLATION_INVOLVED_IN_GAMETE_GENE	1.346985	0.102	0.46626
GOBP_REGULATION_OF_PROTEIN_KINASE_A_SIGNALING	1.346777	0.083	0.46601
GOBP_L_GLUTAMATE_TRANSMEMBRANE_TRANSPORT	1.346251	0.092	0.46694
GOBP_MESODERMAL_CELL_DIFFERENTIATION	1.345973	0.081	0.46699
GOBP_CELL_SUBSTRATE_JUNCTION_ORGANIZATION	1.345868	0.04	0.46628
GOBP_NEGATIVE_REGULATION_OF_CELL_ADHESION	1.345685	0.006	0.46598
GOBP_REGULATION_OF_TUMOR_NECROSIS_FACTOR_ME	1.343905	0.075	0.47191
GOBP_CELLULAR_RESPONSE_TO_PROSTAGLANDIN_E_ST	1.343628	0.102	0.47189
GOBP_CAMP_METABOLIC_PROCESS	1.343414	0.098	0.47169
GOBP_CELL_MORPHOGENESIS_INVOLVED_IN_NEURON_D	1.343352	0	0.47085
GOBP_OVULATION_CYCLE	1.343151	0.053	0.47065
GOBP_TUMOR_NECROSIS_FACTOR_SUPERFAMILY_CYTO	1.343069	0.018	0.46989
GOBP_REGULATION_OF_MIRNA_TRANSCRIPTION	1.34248	0.068	0.47119
GOBP_NEGATIVE_REGULATION_OF_RESPONSE_TO_CYTO	1.342014	0.042	0.47202
GOBP_LEUKOCYTE_CELL_CELL_ADHESION	1.341669	0.002	0.47221
GOBP_FIBRINOLYSIS	1.341651	0.106	0.47121
GOBP_RESPONSE_TO_FIBROBLAST_GROWTH_FACTOR	1.34157	0.045	0.47044
GOBP_NEGATIVE_REGULATION_OF_CYTOKINE_PRODUCT	1.340667	0.117	0.47296
GOBP_GAMMA_DELTA_T_CELL_ACTIVATION	1.340609	0.116	0.47217
GOBP_NEUROTRANSMITTER_RECEPTOR_TRANSPORT	1.339785	0.101	0.47438
GOBP_NEGATIVE_REGULATION_OF_MEIOTIC_CELL_CYCL	1.339729	0.114	0.47358
GOBP_NEGATIVE_REGULATION_OF_HISTONE_METHYLATI	1.339717	0.109	0.47261
GOBP_NEGATIVE_REGULATION_OF_PROTEIN_SECRETIOI	1.338785	0.072	0.47527
GOBP_MAINTENANCE_OF_BLOOD_BRAIN_BARRIER	1.338698	0.079	0.47456
GOBP_CALCIUM_INDEPENDENT_CELL_CELL_ADHESION_V	1.338582	0.083	0.47394
GOBP_MULTICELLULAR_ORGANISMAL_HOMEOSTASIS	1.338521	0	0.47313
GOBP_AXONAL_TRANSPORT	1.338121	0.056	0.47371
GOBP_NEGATIVE_REGULATION_OF_WOUND_HEALING	1.337748	0.053	0.47412
GOBP_RNA_LOCALIZATION	1.337166	0.021	0.47537
GOBP_NONRIBOSOMAL_PEPTIDE_BIOSYNTHETIC_PROCE	1.337013	0.103	0.47496
GOBP_POSITIVE_REGULATION_OF_GLUCOSE_METABOLI(	1.336858	0.086	0.47454
GOBP_NEGATIVE_REGULATION_OF_FIBROBLAST_GROWT	1.335604	0.11	0.47861
GOBP_EPIBOLY	1.335174	0.102	0.47925
GOBP_SUBSTRATE_ADHESION_DEPENDENT_CELL_SPREA	1.33509	0.035	0.47852
GOBP_NEGATIVE_REGULATION_OF_GLIAL_CELL_DIFFERE	1.334862	0.101	0.47834
GOBP_REGULATION_OF_MONOCYTE_CHEMOTAXIS	1.334856	0.097	0.47733
GOBP_CELL_DIFFERENTIATION_IN_SPINAL_CORD	1.334776	0.094	0.47666
GOBP_DETECTION_OF_CHEMICAL_STIMULUS_INVOLVED_	1.334629	0.106	0.47627
GOBP_EYE_PHOTORECEPTOR_CELL_DEVELOPMENT	1.334076	0.091	0.47741
GOBP_POSITIVE_REGULATION_OF_SMALL_GTPASE_MEDI	1.334056	0.053	0.47648
GOBP_CENTROSOME_DUPLICATION	1.334001	0.056	0.47567
GOBP_MONONUCLEAR_CELL_MIGRATION	1.333847	0.016	0.47525
GOBP_REGULATION_OF_VASCULATURE_DEVELOPMENT	1.333741	0.009	0.47462
GOBP_NEGATIVE_REGULATION_OF_LYMPHOCYTE_MEDIA	1.333088	0.077	0.47632

GOBP_CARDIAC_RIGHT_VENTRICLE_MORPHOGENESIS	1.333028	0.102	0.47552
GOBP_TROPHECTODERMAL_CELL_DIFFERENTIATION	1.332946	0.109	0.47482
GOBP_LYMPHOCYTE_CHEMOTAXIS	1.332815	0.075	0.47428
GOBP_MITOCHONDRIAL_TRANSCRIPTION	1.332537	0.107	0.47434
GOBP_FOCAL_ADHESION_ASSEMBLY	1.332391	0.048	0.47384
GOBP_T_CELL_SELECTION	1.332328	0.073	0.47311
GOBP_REGULATION_OF_LONG_TERM_SYNAPTIC_POTENT	1.331503	0.082	0.47534
GOBP_NEGATIVE_REGULATION_OF_IMMUNE_SYSTEM_PR	1.331395	0.003	0.47476
GOBP_REGULATION_OF_CYSTEINE_TYPE_ENDOPEPTIDA	1.331171	0.1	0.47464
GOBP_INTEGRIN_ACTIVATION	1.330555	0.105	0.4761
GOBP_ENERGY_HOMEOSTASIS	1.330108	0.086	0.47694
GOBP_ASPARTATE_FAMILY_AMINO_ACID_METABOLIC_PR	1.3297	0.092	0.47762
GOBP_REGULATION_OF_LEUKOCYTE_DIFFERENTIATION	1.329513	0.005	0.47734
GOBP_BONE_MINERALIZATION	1.329146	0.032	0.47776
GOBP_AMINO_SUGAR_METABOLIC_PROCESS	1.327571	0.096	0.48297
GOBP_ESTABLISHMENT_OF_RNA_LOCALIZATION	1.326923	0.022	0.48464
GOBP_POSITIVE_REGULATION_OF_GLIAL_CELL_DIFFERE	1.326851	0.096	0.48395
GOBP_POSITIVE_REGULATION_OF_NEURAL_PRECURSOR	1.326613	0.073	0.4839
GOBP_POSITIVE_REGULATION_OF_PROTEIN_AUTOPHOSI	1.32649	0.117	0.48338
GOBP_TOLL_LIKE_RECEPTOR_2_SIGNALING_PATHWAY	1.326212	0.115	0.48356
GOBP_NUCLEOBASE_CONTAINING_COMPOUND_TRANSPO	1.326173	0.013	0.48275
GOBP_VISUAL_BEHAVIOR	1.32497	0.084	0.48654
GOBP_NCRNA_PROCESSING	1.324906	0.001	0.48578
GOBP_NEGATIVE_REGULATION_OF_IMMUNE_RESPONSE	1.324884	0.031	0.48488
GOBP_REGULATION_OF_MITOCHONDRIAL_OUTER_MEMBI	1.324816	0.111	0.48417
GOBP_NEGATIVE_REGULATION_OF_REGULATED_SECRET	1.324546	0.109	0.48436
GOBP_NEGATIVE_REGULATION_OF_RESPONSE_TO_DNA_	1.324414	0.056	0.48395
GOBP_INTERLEUKIN_12_PRODUCTION	1.323487	0.074	0.48673
GOBP_LYMPHOCYTE_MIGRATION	1.323373	0.047	0.48618
GOBP_CELLULAR_RESPONSE_TO_RADIATION	1.323075	0.019	0.48649
GOBP_REGULATION_OF_RESPONSE_TO_WOUNDING	1.321679	0.023	0.49105
GOBP_POSITIVE_REGULATION_OF_TYROSINE_PHOSPHOI	1.320442	0.077	0.49498
GOBP_MOTOR_NEURON_AXON_GUIDANCE	1.320365	0.1	0.49432
GOBP_REGULATION_OF_ENDOTHELIAL_CELL_DIFFERENT	1.320024	0.091	0.49478
GOBP_LAMELLIPODIUM_ASSEMBLY	1.319569	0.071	0.49559
GOBP_POSITIVE_REGULATION_OF_LONG_TERM_SYNAPTI	1.319535	0.126	0.49472
GOBP_REGULATION_OF_AXONOGENESIS	1.319184	0.022	0.49518
GOBP_FEEDING_BEHAVIOR	1.318593	0.07	0.4966
GOBP_REGULATION_OF_DENDRITIC_SPINE_MORPHOGEN	1.31843	0.09	0.49626
GOBP_MITOTIC_DNA_INTEGRITY_CHECKPOINT_SIGNALIN	1.318099	0.061	0.49658
GOBP_REGULATION_OF_EXTRINSIC_APOPTOTIC_SIGNALI	1.31772	0.026	0.49715
GOBP_CYTOPLASMIC_PATTERN_RECOGNITION_RECEPTO	1.317715	0.085	0.4962
GOBP_NEGATIVE_REGULATION_OF_STRIATED_MUSCLE_	1.317522	0.101	0.49603
GOBP_POSITIVE_REGULATION_OF_SMOOTH_MUSCLE_CE	1.317521	0.095	0.49508
GOBP_ESTABLISHMENT_OF_PROTEIN_LOCALIZATION_TO	1.317456	0.084	0.49439
GOBP_BONE_RESORPTION	1.317391	0.076	0.49372
GOBP_NEGATIVE_REGULATION_OF_LYMPHOCYTE_DIFFEI	1.317285	0.08	0.49313
GOBP_NEUROTRANSMITTER_UPTAKE	1.316615	0.098	0.49477
GOBP_POSITIVE_REGULATION_OF_GLUCOSE_TRANSMEM	1.316442	0.099	0.4945
GOBP_REGULATION_OF_NEUROTRANSMITTER_LEVELS	1.316319	0.018	0.49404
GOBP_POSITIVE_REGULATION_OF_CELL_ADHESION_MED	1.316015	0.12	0.49427
GOBP_POSITIVE_REGULATION_OF_HEMOPOIESIS	1.315771	0.019	0.49431
GOBP_PROTEIN_INSERTION_INTO_MEMBRANE	1.315731	0.075	0.49354

GOBP_POSITIVE_REGULATION_OF_LOCOMOTION	1.314987	0	0.49552
GOBP_REGULATION_OF_LYMPHOCYTE_APOPTOTIC_PRO(	1.314743	0.075	0.4956
GOBP_COMPLEMENT_ACTIVATION	1.314541	0.114	0.4955
GOBP_NCRNA_METABOLIC_PROCESS	1.314469	0	0.4949
GOBP_POSITIVE_REGULATION_OF_CHONDROCYTE_DIFFE	1.31442	0.142	0.4942
GOBP_POSITIVE_REGULATION_OF_DOUBLE_STRAND_BRE	1.313578	0.12	0.49662
GOBP_COGNITION	1.312752	0.011	0.49892
GOBP_SOMATIC_STEM_CELL_POPULATION_MAINTENANC	1.312737	0.104	0.49803
GOBP_SNRNA_METABOLIC_PROCESS	1.312654	0.083	0.49744
GOBP_PHOTOTRANSDUCTION_VISIBLE_LIGHT	1.311652	0.127	0.50049
GOBP_POSITIVE_REGULATION_OF_ENDOTHELIAL_CELL_M	1.311267	0.046	0.50111
GOBP_NEGATIVE_REGULATION_OF_CELL_MATRIX_ADHES	1.311189	0.109	0.50048
GOBP_REGULATION_OF_PROTEIN_POLYUBIQUITINATION	1.310992	0.104	0.50039
GOBP_REGULATION_OF_CELLULAR_RESPONSE_TO_TRAN	1.310622	0.038	0.50096
GOBP_REGULATION_OF_TRANSLATIONAL_ELONGATION	1.310106	0.142	0.50197
GOBP_REGULATION_OF_TUBE_SIZE	1.308395	0.038	0.50803
GOBP_RIBONUCLEOPROTEIN_COMPLEX_BIOGENESIS	1.307731	0.001	0.50985
GOBP_MRNA_TRANSPORT	1.307452	0.041	0.51006
GOBP_REGULATION_OF_DNA_REPLICATION	1.307398	0.047	0.50934
GOBP_POSITIVE_REGULATION_OF_CELL_ADHESION	1.307371	0.005	0.5085
GOBP_CELLULAR_RESPONSE_TO_LIGHT_STIMULUS	1.306861	0.043	0.5096
GOBP_REGULATION_OF_WATER_LOSS_VIA_SKIN	1.30622	0.104	0.51118
GOBP_NEUROTRANSMITTER_TRANSPORT	1.306152	0.018	0.51052
GOBP_RESPONSE_TO_RADIATION	1.306059	0.007	0.51001
GOBP_MOTILE_CILIUM_ASSEMBLY	1.30588	0.103	0.50975
GOBP_REGULATION_OF_SMALL_GTPASE_MEDIATED_SIGN	1.305603	0.009	0.50991
GOBP_REGULATION_OF_LIPASE_ACTIVITY	1.305473	0.07	0.50953
GOBP_PROSTANOID_METABOLIC_PROCESS	1.305402	0.113	0.50891
GOBP_ACIDIC_AMINO_ACID_TRANSPORT	1.30531	0.098	0.50833
GOBP_HYALURONAN_METABOLIC_PROCESS	1.305277	0.112	0.50754
GOBP_RESPONSE_TO_BACTERIUM	1.304735	0.001	0.50883
GOBP_CELL_AGING	1.303995	0.048	0.51098
GOBP_NEGATIVE_REGULATION_OF_CELL_SUBSTRATE_JU	1.303935	0.134	0.51037
GOBP_RIBOSOMAL_SMALL_SUBUNIT_BIOGENESIS	1.303914	0.073	0.50952
GOBP_DETECTION_OF_LIGHT_STIMULUS	1.303315	0.086	0.51091
GOBP_POSITIVE_REGULATION_OF_PHOSPHATIDYLINOSIT	1.303124	0.081	0.5108
GOBP_NEGATIVE_REGULATION_OF_ALPHA_BETA_T_CELL	1.303084	0.113	0.51007
GOBP_HINDLIMB_MORPHOGENESIS	1.303024	0.121	0.5094
GOBP_POSITIVE_REGULATION_OF_TORC1_SIGNALING	1.302744	0.146	0.50967
GOBP_DOUBLE_STRAND_BREAK_REPAIR_VIA_NONHOMOL	1.302716	0.089	0.5089
GOBP_REGULATION_OF_PATTERN_RECOGNITION_RECEP	1.302611	0.053	0.50845
GOBP_VASCULAR_PROCESS_IN_CIRCULATORY_SYSTEM	1.302597	0.015	0.50759
GOBP_TELENCEPHALON_GLIAL_CELL_MIGRATION	1.301281	0.131	0.51194
GOBP_SPERM_MOTILITY	1.300779	0.063	0.51297
GOBP_MUCOPOLYSACCHARIDE_METABOLIC_PROCESS	1.300747	0.062	0.51217
GOBP_POSITIVE_REGULATION_OF_LYMPHOCYTE_MIGRAT	1.300413	0.106	0.51267
GOBP_COLLAGEN_METABOLIC_PROCESS	1.299438	0.072	0.51571
GOBP_PROTEIN_LOCALIZATION_TO_SYNAPSE	1.299367	0.087	0.51511
GOBP_NEGATIVE_REGULATION_OF_LEUKOCYTE_CELL_C	1.299307	0.046	0.51445
GOBP_NEURON_PROJECTION_ORGANIZATION	1.299295	0.063	0.51361
GOBP_RAB_PROTEIN_SIGNAL_TRANSDUCTION	1.298478	0.123	0.51601
GOBP_RRNA_METABOLIC_PROCESS	1.298433	0.018	0.5153
GOBP_RESPONSE_TO_ANTIBIOTIC	1.298088	0.107	0.51582

GOBP_REGULATION_OF_SMOOTH_MUSCLE_CONTRACTIC	1.297831	0.093	0.51584
GOBP_POSITIVE_REGULATION_OF_LEUKOCYTE_PROLIFE	1.297791	0.05	0.5151
GOBP_CD4_POSITIVE_OR_CD8_POSITIVE_ALPHA_BETA_T	1.297365	0.132	0.51597
GOBP_HORMONE_METABOLIC_PROCESS	1.29733	0.017	0.51521
GOBP_POSITIVE_REGULATION_OF_CARTILAGE_DEVELOP	1.296415	0.111	0.51787
GOBP_SKIN_EPIDERMIS_DEVELOPMENT	1.296334	0.073	0.51728
GOBP_ZYMOGEN_ACTIVATION	1.296077	0.099	0.51749
GOBP_HUMORAL_IMMUNE_RESPONSE_MEDIATED_BY_CIF	1.295895	0.125	0.5173
GOBP_REGULATION_OF_LEUKOCYTE_MIGRATION	1.295678	0.027	0.51738
GOBP_NEGATIVE_REGULATION_OF_TYPE_I_INTERFERON	1.295606	0.153	0.51676
GOBP_POSITIVE_REGULATION_OF_EPITHELIAL_CELL_DIF	1.295521	0.108	0.51627
GOBP_VESICLE_MEDIATED_TRANSPORT_IN_SYNAPSE	1.29548	0.027	0.51553
GOBP_REGULATION_OF_HORMONE_BIOSYNTHETIC_PROC	1.295202	0.13	0.5158
GOBP_PROTEIN_TRIMERIZATION	1.295046	0.147	0.51552
GOBP_PROTEIN_TARGETING_TO_VACUOLE	1.294949	0.117	0.515
GOBP_PEPTIDYL_TYROSINE_DEPHOSPHORYLATION	1.293911	0.067	0.51819
GOBP_REGULATION_OF_EPITHELIAL_CELL_DIFFERENTIAT	1.292873	0.063	0.52152
GOBP_POSITIVE_REGULATION_OF_T_HELPER_1_TYPE_IM	1.292683	0.151	0.52143
GOBP_NEGATIVE_REGULATION_OF_INTERFERON_GAMMA	1.291851	0.137	0.52386
GOBP_REGULATION_OF_MAST_CELL_ACTIVATION	1.291771	0.093	0.5233
GOBP_NEGATIVE_REGULATION_OF_LEUKOCYTE_MIGRAT	1.291012	0.117	0.52536
GOBP_DEVELOPMENT_OF_PRIMARY_FEMALE_SEXUAL_CH	1.290784	0.068	0.5255
GOBP_SYNAPSE_ASSEMBLY	1.290454	0.032	0.52596
GOBP_REGULATION_OF_ALTERNATIVE_MRNA_SPLICING_	1.290426	0.099	0.52519
GOBP_PHAGOCYTOSIS_RECOGNITION	1.289943	0.153	0.52621
GOBP_REGULATION_OF_EPITHELIAL_TO_MESENCHYMAL_	1.289865	0.076	0.5257
GOBP_NEGATIVE_REGULATION_OF_TRANSFORMING_GRC	1.289513	0.086	0.52625
GOBP_ENDOCHONDRAL_BONE_MORPHOGENESIS	1.288921	0.095	0.52783
GOBP_REGULATION_OF_CELL_DIVISION	1.28886	0.038	0.52722
GOBP_REGULATION_OF_CELLULAR_RESPONSE_TO_HEAT	1.28869	0.14	0.52713
GOBP_REGULATED_EXOCYTOSIS	1.288563	0.027	0.52678
GOBP_WATER_SOLUBLE_VITAMIN_METABOLIC_PROCESS	1.288462	0.1	0.5263
GOBP_ACTIN_CYTOSKELETON_REORGANIZATION	1.288081	0.065	0.52701
GOBP_POSITIVE_REGULATION_OF_PROTEIN_KINASE_B_S	1.287906	0.059	0.52679
GOBP_NEGATIVE_REGULATION_OF_CD4_POSITIVE_ALPHA	1.286982	0.146	0.52968
GOBP_B_CELL_PROLIFERATION	1.286715	0.095	0.52981
GOBP_REGULATION_OF_PROTEIN_LOCALIZATION_TO_SY	1.286652	0.146	0.52919
GOBP_TRANSMEMBRANE_RECEPTOR_PROTEIN_SERINE_	1.286146	0.008	0.53036
GOBP_LIPOPOLYSACCHARIDE_MEDIATED_SIGNALING_PA	1.286102	0.111	0.52968
GOBP_POSITIVE_REGULATION_OF_TYPE_I_INTERFERON_	1.286007	0.09	0.52922
GOBP_PLASMINOGEN_ACTIVATION	1.284842	0.141	0.53308
GOBP_NEGATIVE_REGULATION_OF_MITOTIC_CELL_CYCLI	1.284474	0.034	0.5337
GOBP_POSITIVE_REGULATION_OF_MACROPHAGE_CHEMC	1.284305	0.166	0.53354
GOBP_NEGATIVE_REGULATION_OF_TISSUE_REMODELINC	1.283406	0.155	0.53631
GOBP_REGULATION_OF_PEPTIDYL_TYROSINE_PHOSPHO	1.283194	0.025	0.53627
GOBP_RESPONSE_TO_HEAT	1.282794	0.077	0.53693
GOBP_POSITIVE_REGULATION_OF_T_CELL_PROLIFERATIC	1.282732	0.088	0.53629
GOBP_REGULATION_OF_RELEASE_OF_SEQUESTERED_CA	1.282644	0.094	0.53581
GOBP_NEGATIVE_REGULATION_OF_AXONOGENESIS	1.282501	0.087	0.5355
GOBP_POSITIVE_REGULATION_OF_NOTCH_SIGNALING_PA	1.282319	0.101	0.53538
GOBP_POSITIVE_REGULATION_OF_BMP_SIGNALING_PATH	1.282286	0.125	0.53467
GOBP_CRANIAL_NERVE_DEVELOPMENT	1.28217	0.122	0.5343
GOBP_DOSAGE_COMPENSATION	1.281944	0.156	0.53436

GOBP_NUCLEAR_TRANSCRIBED_MRNA_POLY_A_TAIL_SH(	1.280966	0.137	0.53764
GOBP_REGULATION_OF_PHOSPHOLIPASE_ACTIVITY	1.280931	0.107	0.53695
GOBP_LYMPHOCYTE_APOPTOTIC_PROCESS	1.280348	0.086	0.53846
GOBP_EXTERNAL_ENCAPSULATING_STRUCTURE_ORGAN	1.280292	0.01	0.53787
GOBP_RESPONSE_TO_PROTOZOAN	1.280284	0.158	0.53706
GOBP_CELLULAR_SENESCENCE	1.280059	0.083	0.53711
GOBP_CELLULAR_RESPONSE_TO_GAMMA_RADIATION	1.279751	0.121	0.53754
GOBP_CYTOKINE_PRODUCTION_INVOLVED_IN_INFLAMMA	1.279513	0.117	0.53772
GOBP_POSITIVE_T_CELL_SELECTION	1.279197	0.134	0.53815
GOBP_CELLULAR_RESPONSE_TO_UV	1.279176	0.078	0.53743
GOBP_CELLULAR_COMPONENT_MAINTENANCE	1.27907	0.101	0.53705
GOBP_SKELETAL_SYSTEM_DEVELOPMENT	1.278732	0.002	0.53763
GOBP_NEGATIVE_REGULATION_OF_PEPTIDYL_THREONIN	1.278503	0.155	0.53771
GOBP_REGULATION_OF_RESPONSE_TO_DNA_DAMAGE_S	1.278126	0.032	0.53838
GOBP_RNA_MODIFICATION	1.278025	0.04	0.53796
GOBP_CELLULAR_RESPONSE_TO_HEAT	1.278007	0.101	0.5372
GOBP_REGULATION_OF_HEMOPOIESIS	1.277466	0.013	0.5386
GOBP_IRON_ION_HOMEOSTASIS	1.276937	0.097	0.53996
GOBP_ODONTOGENESIS_OF_DENTIN_CONTAINING_TOOT	1.276908	0.091	0.53924
GOBP_CELL_PROLIFERATION_INVOLVED_IN_KIDNEY_DEV	1.276867	0.139	0.53859
GOBP_DECIDUALIZATION	1.276757	0.146	0.53824
GOBP_RESPONSE_TO_MURAMYL_DIPEPTIDE	1.27641	0.154	0.53876
GOBP_REGULATION_OF_LEUKOCYTE_DEGRANULATION	1.276318	0.136	0.53834
GOBP_PERIPHERAL_NERVOUS_SYSTEM_DEVELOPMENT	1.276164	0.088	0.53815
GOBP_SKELETAL_SYSTEM_MORPHOGENESIS	1.275998	0.031	0.53798
GOBP_NEGATIVE_REGULATION_OF_CD4_POSITIVE_ALPH/	1.275997	0.138	0.53717
GOBP_POSITIVE_REGULATION_OF_B_CELL_PROLIFERATI(	1.275129	0.137	0.53975
GOBP_DENDRITIC_CELL_MIGRATION	1.27488	0.147	0.5399
GOBP_RESPONSE_TO_FUNGUS	1.274629	0.112	0.54002
GOBP_MORPHOGENESIS_OF_AN_EPITHELIUM	1.27339	0.005	0.54404
GOBP_REGULATION_OF_PROTEIN_K63_LINKED_UBIQUITIN	1.27309	0.147	0.54442
GOBP_HEMOSTASIS	1.273019	0.036	0.54388
GOBP_COLLAGEN_CATABOLIC_PROCESS	1.272995	0.155	0.54319
GOBP_DICARBOXYLIC_ACID_METABOLIC_PROCESS	1.272853	0.087	0.54292
GOBP_NEGATIVE_REGULATION_OF_DEVELOPMENTAL_GF	1.272738	0.094	0.54255
GOBP_REGULATION_OF_CELL_SUBSTRATE_ADHESION	1.272575	0.028	0.54237
GOBP_REGULATION_OF_CELL_AGING	1.272391	0.126	0.54234
GOBP_CELL_DIFFERENTIATION_INVOLVED_IN_KIDNEY_DE	1.272109	0.104	0.5427
GOBP_ESTABLISHMENT_OF_PLANAR_POLARITY_OF_EMBI	1.271539	0.167	0.54417
GOBP_POSITIVE_REGULATION_OF_GTPASE_ACTIVITY	1.271435	0.025	0.54378
GOBP_NEURON_DEATH	1.271337	0.012	0.54338
GOBP_REGULATION_OF_NEUROTRANSMITTER_TRANSPO	1.270975	0.092	0.54388
GOBP_NEUROBLAST_PROLIFERATION	1.270785	0.116	0.54386
GOBP_NEGATIVE_REGULATION_OF_INTRINSIC_APOPTOT	1.27004	0.068	0.54608
GOBP_RESPONSE_TO_METAL_ION	1.270007	0.018	0.54542
GOBP_REGULATION_OF_WOUND_HEALING	1.269875	0.072	0.54514
GOBP_POSITIVE_REGULATION_OF_NITRIC_OXIDE_SYNTH	1.26984	0.152	0.54447
GOBP_ENDODERM_FORMATION	1.269467	0.126	0.54519
GOBP_APPENDAGE_DEVELOPMENT	1.26943	0.044	0.54452
GOBP_DENDRITIC_SPINE_DEVELOPMENT	1.269238	0.079	0.54452
GOBP_TYROSINE_PHOSPHORYLATION_OF_STAT_PROTEI	1.268964	0.106	0.54481
GOBP_POSITIVE_REGULATION_OF_BLOOD_VESSEL_END(	1.26869	0.141	0.54509
GOBP_POSITIVE_REGULATION_OF_NUCLEOTIDE_METABC	1.268573	0.131	0.54472

GOBP_REGULATION_OF_PROTEIN_KINASE_C_SIGNALING	1.268325	0.17	0.54493
GOBP_RENAL_VESICLE_DEVELOPMENT	1.268082	0.174	0.54516
GOBP_MATURATION_OF_5_8S_RRNA	1.267864	0.14	0.5453
GOBP_BLASTOCYST_FORMATION	1.267584	0.155	0.5457
GOBP_ORGAN_OR_TISSUE_SPECIFIC_IMMUNE_RESPONS	1.267149	0.183	0.54662
GOBP_ATRIAL_CARDIAC_MUSCLE_CELL_TO_AV_NODE_CE	1.267131	0.175	0.54589
GOBP_REGULATION_OF_PH	1.267098	0.116	0.54526
GOBP_APPENDAGE_MORPHOGENESIS	1.266758	0.067	0.54584
GOBP_CYCLIC_NUCLEOTIDE_METABOLIC_PROCESS	1.266752	0.151	0.54508
GOBP_DETECTION_OF_EXTERNAL_BIOTIC_STIMULUS	1.266727	0.163	0.54439
GOBP_OSTEOBLAST_DEVELOPMENT	1.266356	0.169	0.54513
GOBP_LOCOMOTORY_BEHAVIOR	1.265261	0.054	0.54881
GOBP_MICROTUBULE_BUNDLE_FORMATION	1.264897	0.082	0.54949
GOBP_POSITIVE_REGULATION_OF_TRANSFORMING_GRO	1.264817	0.163	0.54903
GOBP_TRANSPORT_OF_VIRUS	1.264603	0.161	0.5491
GOBP_RELEASE_OF_SEQUESTERED_CALCIUM_ION_INTO_	1.264589	0.15	0.54838
GOBP_RESPONSE_TO_ESTROGEN	1.26408	0.116	0.5497
GOBP_FOREBRAIN_CELL_MIGRATION	1.263872	0.126	0.54973
GOBP_RESPONSE_TO_LIGHT_STIMULUS	1.263652	0.022	0.54983
GOBP_REGULATION_OF_CELL_DEVELOPMENT	1.263319	0.007	0.55044
GOBP_POSITIVE_REGULATION_OF_LEUKOCYTE_MIGRATI	1.263099	0.069	0.55055
GOBP_MICROTUBULE_ORGANIZING_CENTER_ORGANIZAT	1.262931	0.059	0.55042
GOBP_TRABECULA_FORMATION	1.262638	0.167	0.55077
GOBP_NEGATIVE_REGULATION_OF_TRANSMEMBRANE_R	1.262486	0.07	0.5506
GOBP_RESPONSE_TO_PROSTAGLANDIN	1.261592	0.153	0.55346
GOBP_NEGATIVE_REGULATION_OF_T_HELPER_CELL_DIF	1.261254	0.167	0.55407
GOBP_OVULATION_CYCLE_PROCESS	1.261143	0.15	0.55374
GOBP_CELLULAR_RESPONSE_TO_VITAMIN_D	1.2611	0.16	0.55315
GOBP_REGULATION_OF_ADAPTIVE_IMMUNE_RESPONSE	1.260992	0.057	0.55275
GOBP_REGULATION_OF_LAMELLIPODIUM_ASSEMBLY	1.26099	0.133	0.55199
GOBP_DENDRITE_MORPHOGENESIS	1.260935	0.07	0.55145
GOBP_NEGATIVE_REGULATION_OF_EXTRINSIC_APOPTOT	1.260502	0.106	0.5523
GOBP_CORONARY_VASCULATURE_DEVELOPMENT	1.260446	0.141	0.55175
GOBP_LEUKOCYTE_DEGRANULATION	1.259919	0.116	0.55308
GOBP_AXIS_SPECIFICATION	1.259782	0.101	0.55286
GOBP_POSITIVE_REGULATION_OF_IMMUNE_EFFECTOR_F	1.259647	0.048	0.55263
GOBP_CELLULAR_RESPONSE_TO_AMYLOID_BETA	1.259645	0.142	0.55187
GOBP_DENDRITIC_CELL_CHEMOTAXIS	1.259271	0.188	0.55259
GOBP_PHOSPHATE_ION_TRANSPORT	1.258567	0.155	0.55468
GOBP_REGULATION_OF_RAS_PROTEIN_SIGNAL_TRANSDU	1.258187	0.057	0.55552
GOBP_XENOBIOTIC_TRANSPORT	1.257545	0.168	0.55741
GOBP_POSITIVE_REGULATION_OF_STRIATED_MUSCLE_C	1.257466	0.147	0.55697
GOBP_REGULATION_OF_TOLL_LIKE_RECEPTOR_4_SIGNA	1.257185	0.149	0.55732
GOBP_REGULATION_OF_RHO_PROTEIN_SIGNAL_TRANSD	1.256857	0.091	0.55782
GOBP_T_CELL_LINEAGE_COMMITMENT	1.256261	0.176	0.55944
GOBP_NEGATIVE_REGULATION_OF_EPITHELIAL_TO_MES	1.256255	0.161	0.55869
GOBP_MATURATION_OF_5_8S_RRNA_FROM_TRICISTRON	1.255824	0.168	0.55957
GOBP_MAST_CELL_ACTIVATION_INVOLVED_IN_IMMUNE_F	1.255451	0.126	0.56034
GOBP_NEGATIVE_REGULATION_OF_NEURON_APOPTOTIC	1.254937	0.069	0.56152
GOBP_NEGATIVE_REGULATION_OF_CELL_CELL_ADHESIO	1.254837	0.062	0.56118
GOBP_MYOBLAST_FUSION	1.254757	0.166	0.56071
GOBP_REGULATION_OF_BIOMINERALIZATION	1.254216	0.101	0.56218
GOBP_RESPONSE_TO_PROSTAGLANDIN_E	1.254081	0.19	0.56201

GOBP_REGULATION_OF_B_CELL_MEDIATED_IMMUNITY	1.253326	0.14	0.56424
GOBP_MACROPHAGE_MIGRATION	1.253164	0.15	0.56415
GOBP_NEGATIVE_REGULATION_OF_CELL_CYCLE_G2_M_F	1.253072	0.125	0.56376
GOBP_NEGATIVE_REGULATION_OF_MITOTIC_CELL_CYCL	1.252949	0.067	0.56348
GOBP_TRANSFORMING_GROWTH_FACTOR_BETA_RECEP	1.252859	0.054	0.56313
GOBP_MRNA_MODIFICATION	1.252681	0.186	0.56307
GOBP_ICOSANOID_TRANSPORT	1.252626	0.147	0.56253
GOBP_MOLTING_CYCLE	1.252369	0.083	0.56279
GOBP_REGULATION_OF_INTRINSIC_APOPTOTIC_SIGNALI	1.252246	0.18	0.56251
GOBP_POSITIVE_REGULATION_OF_CARBOHYDRATE_MET	1.251836	0.111	0.5635
GOBP_CELLULAR_RESPONSE_TO_IONIZING_RADIATION	1.251452	0.116	0.5643
GOBP_SECONDARY_METABOLITE_BIOSYNTHETIC_PROCE	1.251421	0.175	0.56367
GOBP_MONOCARBOXYLIC_ACID_TRANSPORT	1.251221	0.105	0.56372
GOBP_GENE_SILENCING_BY_RNA	1.251152	0.088	0.56324
GOBP_NEGATIVE_REGULATION_OF_CELL_CYCLE_G1_S_F	1.251139	0.122	0.56253
GOBP_NEGATIVE_REGULATION_OF_DNA_RECOMBINATIO	1.251055	0.153	0.56209
GOBP_NEUROTRANSMITTER_RECEPTOR_TRANSPORT_TO	1.250522	0.169	0.56355
GOBP_PROTEOGLYCAN_BIOSYNTHETIC_PROCESS	1.250503	0.141	0.56289
GOBP_CELL_SUBSTRATE_ADHESION	1.250287	0.012	0.56302
GOBP_NEUROMUSCULAR_PROCESS_CONTROLLING_BAL	1.249748	0.133	0.56435
GOBP_B_CELL_ACTIVATION	1.249522	0.051	0.56448
GOBP_MITOTIC_CELL_CYCLE_CHECKPOINT_SIGNALING	1.249164	0.092	0.56523
GOBP_HISTONE_PHOSPHORYLATION	1.248782	0.144	0.56595
GOBP_SMALL_GTPASE_MEDIATED_SIGNAL_TRANSDUCTI	1.248303	0.003	0.56712
GOBP_NEGATIVE_REGULATION_OF_LYMPHOCYTE_ACTIV	1.248163	0.075	0.56697
GOBP_CHOLESTEROL_EFFLUX	1.248019	0.157	0.56687
GOBP_POSITIVE_REGULATION_OF_COLLAGEN_METABOL	1.247696	0.165	0.56746
GOBP_GLUTATHIONE_METABOLIC_PROCESS	1.247497	0.134	0.56751
GOBP_CARDIAC_VENTRICLE_MORPHOGENESIS	1.247112	0.116	0.56832
GOBP_MUSCLE_CELL_PROLIFERATION	1.246996	0.06	0.56806
GOBP_CELLULAR_PROCESS_INVOLVED_IN_REPRODUCTI	1.246634	0.029	0.5687
GOBP_REGULATION_OF_CATECHOLAMINE_METABOLIC_P	1.246585	0.185	0.56815
GOBP_RESPONSE_TO_LECTIN	1.246535	0.19	0.56761
GOBP_PROTEIN_LOCALIZATION_TO_LYSOSOME	1.246268	0.138	0.56791
GOBP_NITRIC_OXIDE_SYNTHASE_BIOSYNTHETIC_PROCE	1.246095	0.188	0.56788
GOBP_REGULATION_OF_MORPHOGENESIS_OF_AN_EPITH	1.246082	0.13	0.56719
GOBP_REGULATION_OF_GLIOGENESIS	1.246041	0.113	0.56663
GOBP_REGULATION_OF_VASCULAR_PERMEABILITY	1.245917	0.14	0.56647
GOBP_REGULATION_OF_EXTENT_OF_CELL_GROWTH	1.245906	0.105	0.56578
GOBP_MATERNAL_PLACENTA_DEVELOPMENT	1.245741	0.151	0.56572
GOBP_NEURAL_PRECURSOR_CELL_PROLIFERATION	1.24499	0.08	0.56791
GOBP_CLEAVAGE_INVOLVED_IN_RRNA_PROCESSING	1.244879	0.184	0.56765
GOBP_POSITIVE_REGULATION_OF_TRANSCRIPTION_BY_F	1.244808	0.19	0.56722
GOBP_CARTILAGE_DEVELOPMENT	1.244542	0.061	0.56754
GOBP_ACTION_POTENTIAL	1.244404	0.086	0.5674
GOBP_NEGATIVE_REGULATION_OF_RESPONSE_TO_BIOT	1.244185	0.099	0.56757
GOBP_REGULATION_OF_LEUKOCYTE_CHEMOTAXIS	1.24392	0.102	0.56788
GOBP_RESPONSE_TO_RETINOIC_ACID	1.243601	0.107	0.56846
GOBP_DEFENSE_RESPONSE_TO_FUNGUS	1.243579	0.159	0.56781
GOBP_T_HELPER_1_TYPE_IMMUNE_RESPONSE	1.243397	0.137	0.56781
GOBP_DETECTION_OF_VISIBLE_LIGHT	1.243231	0.148	0.56776
GOBP_CELLULAR_RESPONSE_TO_PH	1.243177	0.183	0.56729
GOBP_AUTONOMIC_NERVOUS_SYSTEM_DEVELOPMENT	1.243	0.159	0.56727

GOBP_REGULATION_OF_CHOLESTEROL_EFFLUX	1.242834	0.167	0.56725
GOBP_CELLULAR_RESPONSE_TO_LIPOPROTEIN_PARTICL	1.242234	0.162	0.56899
GOBP_RESPONSE_TO_TRANSFORMING_GROWTH_FACTO	1.24222	0.037	0.56833
GOBP_NEGATIVE_REGULATION_OF_CHONDROCYTE_DIFF	1.242062	0.177	0.56823
GOBP_DNA_INTEGRITY_CHECKPOINT_SIGNALING	1.241782	0.092	0.56865
GOBP_NEGATIVE_REGULATION_OF_GROWTH	1.24146	0.053	0.56926
GOBP_RECEPTOR_LOCALIZATION_TO_SYNAPSE	1.240239	0.13	0.57339
GOBP_REGULATION_OF_HEMATOPOIETIC_PROGENITOR_	1.239311	0.177	0.5764
GOBP_BLOOD_VESSEL_REMODELING	1.238818	0.156	0.57774
GOBP_NEUROTRANSMITTER_REUPTAKE	1.238805	0.166	0.57708
GOBP_POSITIVE_REGULATION_OF_MAPK_CASCADE	1.238443	0.011	0.57777
GOBP_RETROGRADE_AXONAL_TRANSPORT	1.238167	0.167	0.57821
GOBP_DEOXYRIBONUCLEOTIDE_BIOSYNTHETIC_PROCES	1.238137	0.208	0.5776
GOBP_RECEPTOR_CLUSTERING	1.237743	0.146	0.57843
GOBP_REGULATION_OF_NEURON_PROJECTION_DEVELOI	1.237296	0.014	0.57952
GOBP_LEUKOCYTE_PROLIFERATION	1.237295	0.053	0.5788
GOBP_PODOSOME_ASSEMBLY	1.236984	0.199	0.57923
GOBP_APOPTOTIC_CELL_CLEARANCE	1.23697	0.163	0.57857
GOBP_CILIUM_OR_FLAGELLUM_DEPENDENT_CELL_MOTIL	1.236957	0.096	0.57791
GOBP_REGULATION_OF_RECEPTOR_BINDING	1.23689	0.172	0.57747
GOBP_REGULATION_OF_CELL_MORPHOGENESIS_INVOLV	1.236599	0.133	0.57796
GOBP_POSITIVE_REGULATION_OF_EPITHELIAL_TO_MESE	1.236261	0.155	0.57865
GOBP_DENDRITE_DEVELOPMENT	1.235895	0.059	0.57933
GOBP_ANTEROGRADE_AXONAL_TRANSPORT	1.2357	0.153	0.57938
GOBP_POSITIVE_REGULATION_OF_SMALL_MOLECULE_ME	1.235644	0.082	0.5789
GOBP_REGULATION_OF_DOUBLE_STRAND_BREAK_REPAI	1.235445	0.109	0.57899
GOBP_NEGATIVE_REGULATION_OF_DNA_BINDING	1.235333	0.151	0.57869
GOBP_REGULATION_OF_T_CELL_DIFFERENTIATION	1.234776	0.1	0.58014
GOBP_REGULATION_OF_NUCLEAR_TRANSCRIBED_MRNA_	1.234594	0.2	0.58013
GOBP_EXCITATORY_SYNAPSE_ASSEMBLY	1.234469	0.193	0.57996
GOBP_NEUROTRANSMITTER_SECRETION	1.234449	0.075	0.57933
GOBP_REGULATION_OF_SUBSTRATE_ADHESION_DEPEND	1.234117	0.137	0.57992
GOBP_EXTRINSIC_APOPTOTIC_SIGNALING_PATHWAY_VIA	1.233765	0.128	0.58055
GOBP_REGULATION_OF_LYSOSOMAL_LUMEN_PH	1.233707	0.192	0.58008
GOBP_SEQUESTERING_OF_CALCIUM_ION	1.233179	0.115	0.58161
GOBP_COCHLEA_MORPHOGENESIS	1.232846	0.193	0.58219
GOBP_MITOCHONDRION_MORPHOGENESIS	1.23282	0.204	0.58157
GOBP_MITOTIC_G2_DNA_DAMAGE_CHECKPOINT_SIGNALI	1.232516	0.186	0.5821
GOBP_MUSCLE_ORGAN_DEVELOPMENT	1.231847	0.039	0.5841
GOBP_REGULATION_OF_VOLTAGE_GATED_CALCIUM_CHA	1.231758	0.177	0.58375
GOBP_HISTONE_H4_ACETYLATION	1.231688	0.127	0.58332
GOBP_PROTEIN_LOCALIZATION_TO_CELL_SURFACE	1.231615	0.15	0.5829
GOBP_LEUKOCYTE_DIFFERENTIATION	1.231304	0.011	0.58344
GOBP_ADAPTIVE_IMMUNE_RESPONSE	1.231302	0.032	0.58276
GOBP_REGULATION_OF_GENE_SILENCING_BY_RNA	1.230655	0.161	0.58467
GOBP_RECEPTOR_SIGNALING_PATHWAY_VIA_STAT	1.230227	0.103	0.58564
GOBP_G_PROTEIN_COUPLED_RECEPTOR_SIGNALING_PA	1.229828	0.183	0.58656
GOBP_DIGESTIVE_SYSTEM_DEVELOPMENT	1.229264	0.101	0.58825
GOBP_RESPONSE_TO_COPPER_ION	1.228873	0.19	0.58909
GOBP_REGULATION_OF_HISTONE_METHYLATION	1.228678	0.15	0.58917
GOBP_REGULATION_OF_RESPONSE_TO_CYTOKINE_STIM	1.227945	0.097	0.59139
GOBP_REGULATION_OF_NEURAL_PRECURSOR_CELL_PR	1.227941	0.142	0.5907
GOBP_HISTONE_H3_K4_TRIMETHYLATION	1.227664	0.197	0.59115

GOBP_RESPONSE_TO_ISCHEMIA	1.22741	0.178	0.59147
GOBP_MONOCYTE_CHEMOTACTIC_PROTEIN_1_PRODUCT	1.227244	0.21	0.59147
GOBP_REGULATION_OF_NEUROGENESIS	1.227002	0.031	0.59175
GOBP_NOTOCHORD_DEVELOPMENT	1.226728	0.206	0.59214
GOBP_REGULATION_OF_FIBROBLAST_MIGRATION	1.226702	0.173	0.59153
GOBP_NEGATIVE_REGULATION_OF_VIRAL_GENOME_REP	1.226037	0.187	0.59357
GOBP_MANNOSYLATION	1.225989	0.176	0.59304
GOBP_POSITIVE_REGULATION_OF_MUSCLE_CELL_DIFFEI	1.225893	0.169	0.59272
GOBP_NEGATIVE_REGULATION_OF_ADAPTIVE_IMMUNE_I	1.225517	0.165	0.59354
GOBP_REGULATION_OF_TRANSMEMBRANE_RECEPTOR_I	1.225275	0.063	0.59381
GOBP_REGULATION_OF_LEUKOCYTE_MEDIATED_IMMUNI	1.224888	0.087	0.59465
GOBP_REGULATION_OF_CELL_ACTIVATION	1.224801	0.012	0.59431
GOBP_POSITIVE_REGULATION_OF_LEUKOCYTE_MEDIATE	1.224154	0.123	0.59617
GOBP_LENS_DEVELOPMENT_IN_CAMERA_TYPE_EYE	1.223435	0.146	0.59829
GOBP_RIBOSOMAL_LARGE_SUBUNIT_BIOGENESIS	1.223414	0.145	0.59768
GOBP_REGULATION_OF_GTPASE_ACTIVITY	1.223141	0.032	0.59808
GOBP_MITOTIC_G1_S_TRANSITION_CHECKPOINT_SIGNAL	1.223019	0.189	0.59788
GOBP_REGULATION_OF_OXIDATIVE_STRESS_INDUCED_C	1.222696	0.158	0.59848
GOBP_NEGATIVE_REGULATION_OF_SECRETION	1.222417	0.104	0.5989
GOBP_PEPTIDYL_TYROSINE_MODIFICATION	1.222271	0.031	0.59876
GOBP_BLASTOCYST_DEVELOPMENT	1.22199	0.124	0.59915
GOBP_POSITIVE_REGULATION_OF_ACTIN_CYTOSKELETO	1.221923	0.202	0.59871
GOBP_DEVELOPMENTAL_CELL_GROWTH	1.221829	0.067	0.59842
GOBP_DNA_METHYLATION	1.221436	0.149	0.59934
GOBP_NEGATIVE_REGULATION_OF_ENDOTHELIAL_CELL_	1.221398	0.186	0.59879
GOBP_CONNECTIVE_TISSUE_DEVELOPMENT	1.221383	0.069	0.59818
GOBP_RNA_EXPORT_FROM_NUCLEUS	1.221319	0.147	0.59773
GOBP_T_CELL_DIFFERENTIATION	1.221052	0.065	0.59812
GOBP_VENTRICULAR_SYSTEM_DEVELOPMENT	1.22097	0.194	0.59776
GOBP_CELLULAR_RESPONSE_TO_DSRNA	1.22076	0.214	0.59786
GOBP_RESPONSE_TO_OXYGEN_LEVELS	1.220629	0.036	0.5977
GOBP_POSITIVE_REGULATION_OF_DOUBLE_STRAND_BRI	1.220528	0.163	0.59743
GOBP_REGULATION_OF_LYMPHOCYTE_DIFFERENTIATION	1.220508	0.107	0.59682
GOBP_ACUTE_PHASE_RESPONSE	1.220399	0.196	0.59656
GOBP_REGULATION_OF_LYMPHOCYTE_MIGRATION	1.220206	0.161	0.59671
GOBP_B_CELL_DIFFERENTIATION	1.220172	0.133	0.59615
GOBP_SENSORY_PERCEPTION_OF_SMELL	1.22016	0.217	0.59553
GOBP_EPITHELIAL_CELL_PROLIFERATION	1.219793	0.034	0.5962
GOBP_POSITIVE_REGULATION_OF_RESPONSE_TO_DNA_I	1.218613	0.119	0.60029
GOBP_CRANIAL_NERVE_MORPHOGENESIS	1.218395	0.202	0.60049
GOBP_REGULATION_OF_PHAGOCYTOSIS	1.218296	0.134	0.60021
GOBP_MESENCHYME_DEVELOPMENT	1.218221	0.041	0.59982
GOBP_CELL_CYCLE_CHECKPOINT_SIGNALING	1.218206	0.091	0.59922
GOBP_ADAPTIVE_IMMUNE_RESPONSE_BASED_ON_SOMA	1.218203	0.074	0.59855
GOBP_REGULATION_OF_STEM_CELL_DIFFERENTIATION	1.217852	0.172	0.59923
GOBP_RESPONSE_TO_ACETYLCHOLINE	1.217361	0.203	0.6005
GOBP_ARACHIDONIC_ACID_METABOLIC_PROCESS	1.216964	0.195	0.6014
GOBP_NEGATIVE_REGULATION_OF_ALPHA_BETA_T_CELL	1.216913	0.183	0.60094
GOBP_NEGATIVE_REGULATION_OF_APOPTOTIC_SIGNALI	1.216019	0.06	0.60395
GOBP_REGULATION_OF_CELL_MIGRATION_INVOLVED_IN_	1.21583	0.188	0.60404
GOBP_RESPONSE_TO_CORTICOSTEROID	1.2158	0.112	0.6035
GOBP_AXO_DENDRITIC_TRANSPORT	1.215643	0.156	0.60346
GOBP_REGULATION_OF_MACROPHAGE_ACTIVATION	1.215087	0.165	0.60495

GOBP_MUSCLE_CELL_MIGRATION	1.214893	0.174	0.60507
GOBP_DETECTION_OF_MECHANICAL_STIMULUS	1.21473	0.184	0.60504
GOBP_CELL_COMMUNICATION_INVOLVED_IN_CARDIAC_C	1.214691	0.183	0.60451
GOBP_MACROPHAGE_ACTIVATION	1.214637	0.132	0.60405
GOBP_MICROTUBULE_DEPOLYMERIZATION	1.21449	0.192	0.60401
GOBP_REGULATION_OF_CHROMOSOME_SEGREGATION	1.214269	0.166	0.60423
GOBP_REGULATION_OF_EXOCYTOSIS	1.214124	0.079	0.60418
GOBP_HYPOTHALAMUS_DEVELOPMENT	1.213994	0.235	0.604
GOBP_ANATOMICAL_STRUCTURE_HOMEOSTASIS	1.213585	0.062	0.60502
GOBP_REGULATION_OF_MACROPHAGE_MIGRATION	1.213572	0.181	0.60441
GOBP_FEMALE_SEX_DIFFERENTIATION	1.213527	0.12	0.60393
GOBP_NEGATIVE_REGULATION_OF_CELL_CYCLE_PROCE	1.21328	0.06	0.60427
GOBP_SMOOTH_MUSCLE_CELL_PROLIFERATION	1.213196	0.099	0.60394
GOBP_GERM_CELL_DEVELOPMENT	1.213068	0.072	0.60382
GOBP_CELLULAR_RESPONSE_TO_VIRUS	1.212699	0.161	0.60455
GOBP_RESPONSE_TO_CALCIUM_ION	1.212643	0.109	0.60411
GOBP_NEGATIVE_REGULATION_OF_PEPTIDE_SECRETION	1.212526	0.205	0.6039
GOBP_REGULATION_OF_B_CELL_ACTIVATION	1.212169	0.129	0.60471
GOBP_TRNA_METABOLIC_PROCESS	1.212111	0.09	0.60426
GOBP_SENSORY_ORGAN_DEVELOPMENT	1.212021	0.017	0.60398
GOBP_REGULATION_OF_LIPOPROTEIN_METABOLIC_PROC	1.21201	0.222	0.60336
GOBP_OOGENESIS	1.21155	0.148	0.60453
GOBP_WOUND_HEALING	1.211153	0.031	0.60552
GOBP_PEPTIDYL_L_CYSTEINE_S_PALMITOYLATION	1.2108	0.216	0.60631
GOBP_RESPONSE_TO_ACIDIC_PH	1.210717	0.201	0.60599
GOBP_REGULATION_OF_MONONUCLEAR_CELL_MIGRATIO	1.209964	0.146	0.60839
GOBP_PSEUDOURIDINE_SYNTHESIS	1.209076	0.205	0.61129
GOBP_REGULATION_OF_CALCIUM_ION_TRANSPORT_INTO	1.208855	0.15	0.61156
GOBP_RESPONSE_TO_NERVE_GROWTH_FACTOR	1.208435	0.182	0.61254
GOBP_IRON_ION_TRANSPORT	1.208431	0.177	0.6119
GOBP_RESPONSE_TO_WOUNDING	1.208	0.014	0.61291
GOBP_NEGATIVE_REGULATION_OF_LEUKOCYTE_PROLIFE	1.207662	0.168	0.61361
GOBP_POSITIVE_REGULATION_OF_CELL_ACTIVATION	1.207479	0.055	0.61366
GOBP_AXON_ENSHEATHMENT_IN_CENTRAL_NERVOUS_S	1.207413	0.229	0.61326
GOBP_REGULATION_OF_CALCIUM_ION_DEPENDENT_EXO	1.207362	0.204	0.61281
GOBP_MULTI_MULTICELLULAR_ORGANISM_PROCESS	1.2073	0.098	0.6124
GOBP_REGULATION_OF_SIGNAL_TRANSDUCTION_BY_P53	1.207229	0.152	0.61205
GOBP_REGULATION_OF_AMPA_RECEPTOR_ACTIVITY	1.207018	0.211	0.61224
GOBP_POSITIVE_REGULATION_OF_P38MAPK_CASCADE	1.206963	0.209	0.6118
GOBP_RESPONSE_TO_MUSCLE_STRETCH	1.206442	0.23	0.61323
GOBP_CEREBELLAR_PURKINJE_CELL_LAYER_MORPHOGE	1.206433	0.237	0.61261
GOBP_MESODERM_DEVELOPMENT	1.205974	0.144	0.61383
GOBP_NEGATIVE_REGULATION_OF_IMMUNE_EFFECTOR_	1.205525	0.168	0.61492
GOBP_EPIDERMAL_CELL_DIFFERENTIATION	1.204957	0.093	0.61649
GOBP_DICARBOXYLIC_ACID_TRANSPORT	1.204579	0.173	0.61733
GOBP_REGULATION_OF_MEMBRANE_POTENTIAL	1.204481	0.035	0.61705
GOBP_OSSIFICATION	1.204326	0.033	0.61705
GOBP_ENDODERM_DEVELOPMENT	1.204191	0.154	0.61694
GOBP_PROTEIN_LOCALIZATION_TO_VACUOLE	1.204092	0.186	0.61666
GOBP_POSITIVE_REGULATION_OF_LYMPHOCYTE_DIFFER	1.203854	0.15	0.61694
GOBP_B_CELL_ACTIVATION_INVOLVED_IN_IMMUNE_RESP	1.203798	0.175	0.61651
GOBP_RRNA_METHYLATION	1.203642	0.22	0.61653
GOBP_FLUID_TRANSPORT	1.203336	0.208	0.6171

GOBP_POSITIVE_REGULATION_OF_NEUROBLAST_PROLIF	1.203231	0.226	0.61686
GOBP_POSITIVE_REGULATION_OF_SIGNALING_RECEPTO	1.203042	0.203	0.61701
GOBP_DETECTION_OF_BIOTIC_STIMULUS	1.202978	0.209	0.6166
GOBP_NEGATIVE_REGULATION_OF_CELLULAR_RESPONS	1.202856	0.161	0.61643
GOBP_NEGATIVE_REGULATION_OF_MUSCLE_CONTRACTI	1.202344	0.226	0.61787
GOBP_BONE_MORPHOGENESIS	1.20231	0.148	0.61737
GOBP_CHONDROCYTE_DEVELOPMENT	1.202147	0.219	0.61736
GOBP_RESPIRATORY_SYSTEM_PROCESS	1.201957	0.213	0.61751
GOBP_SYNAPTIC_VESICLE_LOCALIZATION	1.201876	0.188	0.61717
GOBP_DENDRITIC_CELL_CYTOKINE_PRODUCTION	1.201474	0.236	0.61811
GOBP_LIVER_MORPHOGENESIS	1.201309	0.211	0.61811
GOBP_PULMONARY_VALVE_DEVELOPMENT	1.201295	0.225	0.61753
GOBP_RESPONSE_TO_UV	1.201245	0.127	0.61708
GOBP_REGULATION_OF_BODY_FLUID_LEVELS	1.200881	0.053	0.61788
GOBP_ADENYLATE_CYCLASE_MODULATING_G_PROTEIN_	1.200709	0.088	0.61789
GOBP_EPIDERMIS_DEVELOPMENT	1.200538	0.07	0.61794
GOBP_PROTEIN_K48_LINKED_UBIQUITINATION	1.200486	0.177	0.6175
GOBP_REGULATION_OF_LEUKOCYTE_PROLIFERATION	1.200438	0.076	0.61707
GOBP_POST_EMBRYONIC_DEVELOPMENT	1.200367	0.167	0.6167
GOBP_NEGATIVE_REGULATION_OF_PEPTIDASE_ACTIVITY	1.200306	0.121	0.61633
GOBP_LYMPHOCYTE_ACTIVATION_INVOLVED_IN_IMMUNE	1.200257	0.115	0.61589
GOBP_KERATINOCYTE_DIFFERENTIATION	1.200125	0.148	0.61575
GOBP_CARDIAC_VENTRICLE_DEVELOPMENT	1.19975	0.123	0.61656
GOBP_CELL_MIGRATION_INVOLVED_IN_HEART_DEVELOP	1.199613	0.233	0.61646
GOBP_SENSORY_PERCEPTION_OF_LIGHT_STIMULUS	1.199602	0.088	0.61586
GOBP_MITOTIC_G2_M_TRANSITION_CHECKPOINT	1.199406	0.185	0.61604
GOBP_T_CELL_ACTIVATION	1.19939	0.043	0.61547
GOBP_REGULATION_OF_DEVELOPMENTAL_GROWTH	1.199376	0.068	0.61489
GOBP_REGULATION_OF_CELL_MATRIX_ADHESION	1.199356	0.143	0.61435
GOBP_REGENERATION	1.199352	0.107	0.61374
GOBP_PATTERN_RECOGNITION_RECEPTOR_SIGNALING_	1.199099	0.115	0.6141
GOBP_PYRIDINE_CONTAINING_COMPOUND_METABOLIC_	1.198983	0.204	0.61394
GOBP_VASCULAR_TRANSPORT	1.198919	0.17	0.61358
GOBP_NEGATIVE_REGULATION_OF_SMOOTH_MUSCLE_C	1.19806	0.234	0.61636
GOBP_NEGATIVE_REGULATION_OF_NUCLEAR_DIVISION	1.197379	0.185	0.61847
GOBP_SYNAPSE_ORGANIZATION	1.196357	0.042	0.62182
GOBP_CELL_FATE_COMMITMENT	1.196234	0.088	0.62166
GOBP_CAMERA_TYPE_EYE_DEVELOPMENT	1.19589	0.062	0.62236
GOBP_MONONUCLEAR_CELL_DIFFERENTIATION	1.195861	0.049	0.62185
GOBP_VITAMIN_METABOLIC_PROCESS	1.195794	0.152	0.6215
GOBP_REGULATION_OF_MACROPHAGE_DIFFERENTIATIO	1.195456	0.237	0.62228
GOBP_MONOCYTE_DIFFERENTIATION	1.195262	0.216	0.62244
GOBP_INTRINSIC_APOPTOTIC_SIGNALING_PATHWAY_IN_	1.195214	0.241	0.62203
GOBP_SLEEP	1.195128	0.224	0.62175
GOBP_MONOUBIQUITINATED_PROTEIN_DEUBIQUITINATIO	1.19486	0.232	0.62218
GOBP_MYOTUBE_DIFFERENTIATION	1.194852	0.154	0.62159
GOBP_REGULATION_OF_MITOTIC_NUCLEAR_DIVISION	1.194551	0.148	0.62214
GOBP_SPERMATID_DIFFERENTIATION	1.194361	0.133	0.62226
GOBP_NEUROTRANSMITTER_RECEPTOR_INTERNALIZATIO	1.194358	0.239	0.62165
GOBP_DOPAMINERGIC_NEURON_DIFFERENTIATION	1.194242	0.225	0.6215
GOBP_PATTERN_SPECIFICATION_PROCESS	1.194093	0.037	0.62151
GOBP_POSITIVE_REGULATION_OF_DNA_BIOSYNTHETIC_	1.194085	0.163	0.62094
GOBP_P38MAPK_CASCADE	1.19353	0.175	0.62247

GOBP_NEGATIVE_REGULATION_OF_T_CELL_PROLIFERAT	1.193334	0.207	0.62266
GOBP_NUCLEOTIDE_BINDING_DOMAIN_LEUCINE_RICH_RE	1.193308	0.227	0.62215
GOBP_LONG_TERM_SYNAPTIC_POTENTIATION	1.193035	0.161	0.62256
GOBP_NADPH_REGENERATION	1.192981	0.23	0.62215
GOBP_PHENOL_CONTAINING_COMPOUND_METABOLIC_PI	1.192932	0.171	0.62173
GOBP_REGULATION_OF_T_CELL_ACTIVATION	1.192832	0.068	0.62154
GOBP_NEGATIVE_REGULATION_OF_RESPONSE_TO_EXTE	1.192736	0.054	0.62127
GOBP_UNSATURATED_FATTY_ACID_METABOLIC_PROCES	1.19248	0.186	0.62169
GOBP_MACROPHAGE_CHEMOTAXIS	1.19247	0.218	0.62112
GOBP_REGULATION_OF_CYTOPLASMIC_TRANSLATION	1.191914	0.231	0.6227
GOBP_T_CELL_ACTIVATION_INVOLVED_IN_IMMUNE_RESP	1.191839	0.181	0.62239
GOBP_WHITE_FAT_CELL_DIFFERENTIATION	1.191755	0.25	0.62211
GOBP_REPLICATION_FORK_PROCESSING	1.19126	0.21	0.62346
GOBP_SEMAPHORIN_PLEXIN_SIGNALING_PATHWAY_INVO	1.191156	0.252	0.62329
GOBP_SMOOTH_MUSCLE_CELL_MIGRATION	1.191133	0.19	0.62277
GOBP_NEGATIVE_REGULATION_OF_SIGNAL_TRANSDUCT	1.190983	0.201	0.62273
GOBP_POSITIVE_REGULATION_OF_LEUKOCYTE_APOPTOT	1.190563	0.228	0.62372
GOBP_POSITIVE_REGULATION_OF_SIGNAL_TRANSDUCTIO	1.190541	0.243	0.62319
GOBP_POSITIVE_REGULATION_OF_LYMPHOCYTE_MEDIAT	1.190519	0.186	0.62266
GOBP_SOMITOGENESIS	1.190467	0.209	0.62228
GOBP_RESPONSE_TO_MECHANICAL_STIMULUS	1.190197	0.103	0.62271
GOBP_B_CELL_APOPTOTIC_PROCESS	1.189636	0.23	0.62424
GOBP_AGING	1.189421	0.075	0.62449
GOBP_SKIN_DEVELOPMENT	1.189237	0.087	0.62455
GOBP_NEGATIVE_REGULATION_OF_EPITHELIAL_CELL_AP	1.18916	0.204	0.62423
GOBP_REGULATION_OF_MYOTUBE_DIFFERENTIATION	1.189138	0.218	0.62371
GOBP_NUCLEAR_TRANSCRIBED_MRNA_CATABOLIC_PRO(	1.189068	0.187	0.62335
GOBP_ALTERNATIVE_MRNA_SPLICING_VIA_SPLICEOSOME	1.188954	0.167	0.62319
GOBP_NUCLEAR_EXPORT	1.188866	0.127	0.62291
GOBP_PROTEIN_KINASE_B_SIGNALING	1.188765	0.111	0.62268
GOBP_DNA_REPLICATION_CHECKPOINT_SIGNALING	1.188224	0.261	0.62426
GOBP_DEVELOPMENTAL_GROWTH_INVOLVED_IN_MORPH	1.188035	0.093	0.62439
GOBP_REGULATION_OF_INNATE_IMMUNE_RESPONSE	1.187943	0.132	0.62414
GOBP_POSITIVE_REGULATION_OF_GLIOGENESIS	1.187901	0.206	0.62372
GOBP_POSITIVE_REGULATION_OF_B_CELL_MEDIATED_IM	1.187665	0.225	0.62405
GOBP_NEURON_APOPTOTIC_PROCESS	1.187653	0.101	0.62349
GOBP_POSITIVE_REGULATION_OF_SMOOTH_MUSCLE_CO	1.187562	0.227	0.62325
GOBP_PULMONARY_VALVE_MORPHOGENESIS	1.187561	0.241	0.62265
GOBP_VASCULAR_ENDOTHELIAL_GROWTH_FACTOR_REC	1.187376	0.203	0.6228
GOBP_POSITIVE_REGULATION_OF_RESPONSE_TO_EXTER	1.18684	0.059	0.62439
GOBP_TRNA_PROCESSING	1.186476	0.13	0.6252
GOBP_NEGATIVE_REGULATION_OF_CELL_CYCLE	1.186456	0.049	0.62469
GOBP_REGULATION_OF_SYNAPSE_STRUCTURE_OR_ACT	1.18632	0.107	0.62462
GOBP_REGULATION_OF_DNA_DAMAGE_RESPONSE_SIGN	1.186062	0.245	0.62504
GOBP_NEGATIVE_REGULATION_OF_VASCULATURE_DEVE	1.186019	0.184	0.62462
GOBP_HOMEOSTASIS_OF_NUMBER_OF_CELLS_WITHIN_A	1.185984	0.234	0.62416
GOBP_VENOUS_BLOOD_VESSEL_DEVELOPMENT	1.185608	0.252	0.62505
GOBP_NEGATIVE_REGULATION_OF_CELL_CYCLE_PHASE_	1.185607	0.093	0.62446
GOBP_AXONAL_FASCICULATION	1.185546	0.26	0.62411
GOBP_ANATOMICAL_STRUCTURE_MATURATION	1.184978	0.12	0.62569
GOBP_SYNCYTIUM_FORMATION	1.184894	0.21	0.62543
GOBP_CELLULAR_RESPONSE_TO_OXYGEN_LEVELS	1.184862	0.129	0.62495
GOBP_EPITHELIAL_TO_MESENCHYMAL_TRANSITION	1.184739	0.128	0.62482

GOBP_NEGATIVE_REGULATION_OF_RHO_PROTEIN_SIGNA	1.184619	0.243	0.62468
GOBP_ACTIVATION_OF_INNATE_IMMUNE_RESPONSE	1.18379	0.232	0.62729
GOBP_POSITIVE_REGULATION_OF_HYDROLASE_ACTIVITY	1.183721	0.03	0.62698
GOBP_REGULATION_OF_T_HELPER_CELL_DIFFERENTIATI	1.183552	0.254	0.62705
GOBP_POSITIVE_REGULATION_OF_EXCITATORY_POSTSY	1.18347	0.246	0.62676
GOBP_CELL_ACTIVATION_INVOLVED_IN_IMMUNE_RESPON	1.18347	0.098	0.62617
GOBP_TRNA_METHYLATION	1.182997	0.22	0.62735
GOBP_REGULATION_OF_NERVOUS_SYSTEM_DEVELOPME	1.182844	0.052	0.62737
GOBP_RRNA_MODIFICATION	1.18255	0.23	0.62796
GOBP_PROTEIN_MANNOSYLATION	1.182318	0.263	0.62827
GOBP_REGULATION_OF_VIRAL_INDUCED_CYTOPLASMIC_	1.182232	0.258	0.62803
GOBP_INTERLEUKIN_4_PRODUCTION	1.181978	0.255	0.62843
GOBP_REGULATION_OF_DOUBLE_STRAND_BREAK_REPAI	1.181593	0.217	0.62941
GOBP_NEGATIVE_REGULATION_OF_DEFENSE_RESPONSE	1.181587	0.128	0.62884
GOBP_MICROGLIAL_CELL_ACTIVATION	1.181442	0.225	0.62882
GOBP_REGIONALIZATION	1.181407	0.074	0.62837
GOBP_RESPONSE_TO_OSMOTIC_STRESS	1.181321	0.186	0.62817
GOBP_HISTONE_METHYLATION	1.181321	0.131	0.62759
GOBP_CEREBRAL_CORTEX_DEVELOPMENT	1.181208	0.172	0.62749
GOBP_POSITIVE_REGULATION_OF_JNK_CASCADE	1.181177	0.172	0.62701
GOBP_DIGESTIVE_TRACT_MORPHOGENESIS	1.18081	0.222	0.62783
GOBP_PRIMARY_ALCOHOL_METABOLIC_PROCESS	1.180457	0.193	0.62859
GOBP_REGULATION_OF_PROTEIN_TYROSINE_KINASE_AC	1.180456	0.182	0.62801
GOBP_CELL_DEATH_IN_RESPONSE_TO_OXIDATIVE_STRE	1.180389	0.212	0.62767
GOBP_PRESYNAPSE_ORGANIZATION	1.180308	0.213	0.62737
GOBP_TRANSITION_METAL_ION_HOMEOSTASIS	1.17992	0.167	0.62831
GOBP_DNA_DAMAGE_RESPONSE_SIGNAL_TRANSDUCTIO	1.179747	0.263	0.6284
GOBP_POSITIVE_REGULATION_OF_NUCLEAR_TRANSCRIB	1.179615	0.264	0.62836
GOBP_TRNA_WOBBLE_BASE_MODIFICATION	1.179502	0.255	0.62822
GOBP_REGULATION_OF_MEMBRANE_PROTEIN_ECTODOM	1.179391	0.256	0.62808
GOBP_EAR_DEVELOPMENT	1.179279	0.129	0.62795
GOBP_AMINOGLYCAN_BIOSYNTHETIC_PROCESS	1.179119	0.202	0.62799
GOBP_PROTEIN_CONTAINING_COMPLEX_LOCALIZATION	1.179109	0.154	0.62745
GOBP_CD4_POSITIVE_ALPHA_BETA_T_CELL_ACTIVATION	1.178984	0.193	0.62737
GOBP_MITOCHONDRIAL_TRANSLATION	1.178692	0.203	0.62796
GOBP_PATHWAY_RESTRICTED_SMAD_PROTEIN_PHOSPH	1.178605	0.198	0.62777
GOBP_REGULATION_OF_RECEPTOR_SIGNALING_PATHWA	1.178518	0.188	0.62755
GOBP_REGULATION_OF_RESPONSE_TO_BIOTIC_STIMULU	1.17837	0.076	0.62755
GOBP_NEURON_FATE_COMMITMENT	1.178147	0.216	0.62781
GOBP_T_HELPER_1_CELL_DIFFERENTIATION	1.178077	0.241	0.62751
GOBP_NEGATIVE_REGULATION_OF_PROTEOLYSIS	1.178068	0.114	0.62699
GOBP_MITOCHONDRIAL_DEPOLARIZATION	1.177554	0.258	0.62843
GOBP_STRIATED_MUSCLE_CELL_PROLIFERATION	1.177508	0.224	0.62802
GOBP_NEURON_FATE_SPECIFICATION	1.177345	0.261	0.62812
GOBP_POSITIVE_REGULATION_OF_CELLULAR_CARBOHYD	1.175804	0.219	0.63344
GOBP_REGULATION_OF_RECEPTOR_INTERNALIZATION	1.175558	0.203	0.63382
GOBP_SKELETAL_MUSCLE_CELL_DIFFERENTIATION	1.175553	0.196	0.63326
GOBP_REGULATION_OF_CELLULAR_AMINE_METABOLIC_F	1.175217	0.261	0.63404
GOBP_AROMATIC_AMINO_ACID_FAMILY_METABOLIC_PRO	1.175158	0.241	0.63372
GOBP_RESPONSE_TO_REACTIVE_OXYGEN_SPECIES	1.174774	0.142	0.63468
GOBP_PROTEIN_LOCALIZATION_TO_CELL_JUNCTION	1.174699	0.173	0.63441
GOBP_CELLULAR_RESPONSE_TO_RETINOIC_ACID	1.174498	0.224	0.63462
GOBP_EXTRINSIC_APOPTOTIC_SIGNALING_PATHWAY	1.174478	0.114	0.63412

GOBP_HIPPOCAMPUS_DEVELOPMENT	1.174377	0.212	0.63395
GOBP_ANTIGEN_PROCESSING_AND_PRESENTATION_OF_	1.174261	0.252	0.63383
GOBP_CYTOKINE_MEDIATED_SIGNALING_PATHWAY	1.174211	0.065	0.63346
GOBP_LONG_CHAIN_FATTY_ACYL_COA_METABOLIC_PRO	1.17417	0.264	0.63305
GOBP_LONG_CHAIN_FATTY_ACYL_COA_BIOSYNTHETIC_F	1.173908	0.258	0.63347
GOBP_CARDIAC_MUSCLE_CELL_ACTION_POTENTIAL_INV(	1.173431	0.234	0.63471
GOBP_REGULATION_OF_IMMUNE_EFFECTOR_PROCESS	1.172909	0.105	0.63622
GOBP_RESPONSE_TO_CADMIUM_ION	1.172862	0.237	0.63585
GOBP_REGULATION_OF_SMAD_PROTEIN_SIGNAL_TRANS	1.172295	0.255	0.63747
GOBP_BIOLOGICAL_PROCESS_INVOLVED_IN_INTRASPECI	1.17199	0.231	0.63802
GOBP_REGULATION_OF_TOLL_LIKE_RECEPTOR_SIGNALII	1.170942	0.224	0.64152
GOBP_POSITIVE_REGULATION_OF_ACUTE_INFLAMMATOF	1.170912	0.269	0.64106
GOBP_ALPHA_BETA_T_CELL_DIFFERENTIATION	1.170833	0.208	0.64081
GOBP_NEGATIVE_REGULATION_OF_REACTIVE_OXYGEN_	1.170434	0.227	0.64185
GOBP_DNA_METHYLATION_OR_DEMETHYLATION	1.170317	0.225	0.64174
GOBP_BIOMINERALIZATION	1.170304	0.17	0.64121
GOBP_RESPONSE_TO_VIRUS	1.170128	0.099	0.64132
GOBP_REGULATION_OF_ENDOTHELIAL_CELL_MIGRATION	1.170024	0.14	0.64116
GOBP_POSITIVE_REGULATION_OF_RELEASE_OF_SEQUES	1.16965	0.247	0.64205
GOBP_CELLULAR_RESPONSE_TO_CADMIUM_ION	1.169617	0.264	0.6416
GOBP_MAINTENANCE_OF_CELL_NUMBER	1.169377	0.178	0.64198
GOBP_OLIGOPEPTIDE_TRANSPORT	1.16913	0.276	0.64236
GOBP_CENTRIOLE_ASSEMBLY	1.168372	0.253	0.64478
GOBP_BONE_DEVELOPMENT	1.167976	0.142	0.64572
GOBP_REGULATION_OF_APOPTOTIC_SIGNALING_PATHW	1.16789	0.087	0.64544
GOBP_ANTIBACTERIAL_HUMORAL_RESPONSE	1.167746	0.246	0.64542
GOBP_MECHANORECEPTOR_DIFFERENTIATION	1.167539	0.22	0.64569
GOBP_NEGATIVE_REGULATION_OF_CELL_GROWTH	1.167108	0.172	0.64675
GOBP_PURINERGIC_NUCLEOTIDE_RECEPTOR_SIGNALING	1.167006	0.258	0.64656
GOBP_SYNAPTIC_VESICLE_EXOCYTOSIS	1.166929	0.195	0.64631
GOBP_REGULATION_OF_REPRODUCTIVE_PROCESS	1.166821	0.149	0.64615
GOBP_ACUTE_INFLAMMATORY_RESPONSE	1.166802	0.2	0.64567
GOBP_CEREBELLAR_PURKINJE_CELL_LAYER_DEVELOPM	1.166776	0.254	0.64521
GOBP_DENDRITIC_CELL_DIFFERENTIATION	1.166569	0.247	0.64549
GOBP_POSITIVE_REGULATION_OF_OXIDATIVE_STRESS_I	1.166082	0.263	0.6468
GOBP_COLLAGEN_FIBRIL_ORGANIZATION	1.166073	0.223	0.64628
GOBP_NEGATIVE_REGULATION_OF_PROTEIN_LOCALIZAT	1.16602	0.167	0.64591
GOBP_DNA_MODIFICATION	1.165903	0.189	0.6458
GOBP_ADENYLATE_CYCLASE_ACTIVATING_G_PROTEIN_C	1.165899	0.178	0.64525
GOBP_VASCULOGENESIS	1.16581	0.203	0.64504
GOBP_AXONEMAL_DYNEIN_COMPLEX_ASSEMBLY	1.165704	0.27	0.64489
GOBP_POSITIVE_REGULATION_OF_CYTOKINE_PRODUCTI	1.165063	0.072	0.6468
GOBP_RESPONSE_TO_PROGESTERONE	1.165043	0.252	0.6463
GOBP_NEURON_PROJECTION_EXTENSION	1.164648	0.164	0.64723
GOBP_REGULATION_OF_OSSIFICATION	1.164507	0.17	0.64721
GOBP_ASPARTATE_FAMILY_AMINO_ACID_BIOSYNTHETIC_	1.164133	0.27	0.64813
GOBP_MESODERM_MORPHOGENESIS	1.16405	0.222	0.64788
GOBP_TOLL_LIKE_RECEPTOR_4_SIGNALING_PATHWAY	1.163884	0.244	0.64798
GOBP_KINETOCHORE_ORGANIZATION	1.16337	0.253	0.64933
GOBP_NEGATIVE_REGULATION_OF_CELLULAR_SENESCE	1.162833	0.281	0.65088
GOBP_MITOCHONDRIAL_RNA_PROCESSING	1.162529	0.278	0.65154
GOBP_REGULATION_OF_CARDIAC_MUSCLE_CELL_ACTIOI	1.162104	0.267	0.65263
GOBP_REGULATION_OF_KIDNEY_DEVELOPMENT	1.162044	0.261	0.65233

GOBP_REGULATION_OF_EXTRACELLULAR_MATRIX_ORGA	1.161849	0.244	0.6525
GOBP_RNA_5_END_PROCESSING	1.16145	0.289	0.65353
GOBP_THYROID_HORMONE_METABOLIC_PROCESS	1.161181	0.262	0.65404
GOBP_CELL_MIGRATION_INVOLVED_IN_SPROUTING_ANG	1.160897	0.235	0.6546
GOBP_REGULATION_OF_PHOSPHATIDYLINOSITOL_3_KINA	1.160675	0.247	0.65489
GOBP_AMINOGLYCAN_METABOLIC_PROCESS	1.160262	0.186	0.65589
GOBP_RAS_PROTEIN_SIGNAL_TRANSDUCTION	1.160228	0.095	0.65546
GOBP_CARDIAC_MUSCLE_CELL_DIFFERENTIATION	1.160169	0.213	0.65512
GOBP_POSITIVE_REGULATION_OF_INNATE_IMMUNE_RES	1.16015	0.209	0.65464
GOBP_POSITIVE_REGULATION_OF_SYNAPTIC_TRANSMISS	1.159678	0.196	0.65584
GOBP_RHO_PROTEIN_SIGNAL_TRANSDUCTION	1.158933	0.19	0.65814
GOBP_REGULATION_OF_MACROPHAGE_CHEMOTAXIS	1.158771	0.274	0.65824
GOBP_SIGNAL_TRANSDUCTION_IN_RESPONSE_TO_DNA_	1.158736	0.166	0.6578
GOBP_SENSORY_SYSTEM_DEVELOPMENT	1.158699	0.084	0.65739
GOBP_MESONEPHROS_DEVELOPMENT	1.158205	0.225	0.65872
GOBP_DNA_DEPENDENT_DNA_REPLICATION_MAINTENAN	1.158091	0.258	0.65862
GOBP_PEROXISOME_PROLIFERATOR_ACTIVATED_RECEP	1.15802	0.281	0.65835
GOBP_CARDIAC_MUSCLE_TISSUE_DEVELOPMENT	1.157994	0.164	0.65791
GOBP_G1_TO_G0_TRANSITION	1.157781	0.288	0.65813
GOBP_NEGATIVE_REGULATION_OF_CELL_KILLING	1.157401	0.309	0.6591
GOBP_NUCLEOTIDE_BINDING_OLIGOMERIZATION_DOMAI	1.15721	0.282	0.65932
GOBP_POSITIVE_REGULATION_OF_CELL_MORPHOGENES	1.157063	0.231	0.65934
GOBP_REGULATION_OF_CELLULAR_RESPONSE_TO_VASC	1.156833	0.275	0.65967
GOBP_FEMALE_GAMETE_GENERATION	1.156672	0.188	0.65975
GOBP_POSITIVE_REGULATION_OF_CELL_DEVELOPMENT	1.156637	0.126	0.65929
GOBP_RNA_INTERFERENCE	1.156472	0.293	0.65943
GOBP_FORMATION_OF_PRIMARY_GERM_LAYER	1.156079	0.222	0.66039
GOBP_POSITIVE_REGULATION_OF_MYELOID_CELL_DIFFE	1.155883	0.232	0.66063
GOBP_NEGATIVE_REGULATION_OF_PROTEIN_LOCALIZAT	1.155799	0.281	0.66039
GOBP_ADULT_BEHAVIOR	1.155775	0.197	0.65994
GOBP_SOMITE_DEVELOPMENT	1.155383	0.23	0.6609
GOBP_POSITIVE_REGULATION_OF_TELOMERASE_RNA_L	1.155198	0.294	0.6611
GOBP_CALCIUM_ION_TRANSPORT_INTO_CYTOSOL	1.154756	0.187	0.66222
GOBP_MUSCLE_ORGAN_MORPHOGENESIS	1.154481	0.222	0.6627
GOBP_POSITIVE_REGULATION_OF_CHEMOTAXIS	1.154245	0.202	0.66307
GOBP_HISTONE_MRNA_METABOLIC_PROCESS	1.153934	0.272	0.66372
GOBP_POSITIVE_REGULATION_OF_MACROPHAGE_ACTIV	1.153892	0.287	0.66332
GOBP_REGULATION_OF_PRODUCTION_OF_SMALL_RNA_I	1.153796	0.28	0.66314
GOBP_REGULATION_OF_PODOSOME_ASSEMBLY	1.153655	0.289	0.66313
GOBP_POSITIVE_REGULATION_OF_EPITHELIAL_CELL_MIG	1.153454	0.179	0.66333
GOBP_HEPARAN_SULFATE_PROTEOGLYCAN_METABOLIC	1.153418	0.278	0.66291
GOBP_POSITIVE_REGULATION_OF_EXOCYTOSIS	1.153372	0.238	0.66254
GOBP_REGULATION_OF_SKELETAL_MUSCLE_CELL_DIFFE	1.153185	0.287	0.66273
GOBP_NEGATIVE_REGULATION_OF_DNA_METABOLIC_PR	1.152752	0.207	0.66387
GOBP_REGULATION_OF_PHOSPHOLIPID_BIOSYNTHETIC_	1.15257	0.294	0.66404
GOBP_NEGATIVE_REGULATION_OF_STEM_CELL_DIFFERE	1.152428	0.29	0.66403
GOBP_SINGLE_FERTILIZATION	1.152147	0.199	0.66458
GOBP_RESPONSE_TO_ALKALOID	1.15184	0.24	0.6652
GOBP_REGULATION_OF_EXTRINSIC_APOPTOTIC_SIGNALI	1.151679	0.262	0.66528
GOBP_DOPAMINE_TRANSPORT	1.151541	0.269	0.66525
GOBP_REGULATION_OF_ALPHA_BETA_T_CELL_DIFFEREN	1.151524	0.249	0.66475
GOBP_FC_RECEPTOR_SIGNALING_PATHWAY	1.151364	0.271	0.66479
GOBP_RESPONSE_TO_INORGANIC_SUBSTANCE	1.151123	0.071	0.6652

GOBP_POSITIVE_REGULATION_OF_MONOCYTE_CHEMOTA	1.150608	0.29	0.66666
GOBP_POSITIVE_REGULATION_OF_CELLULAR_RESPONSE	1.150361	0.277	0.6671
GOBP_MYELOID_LEUKOCYTE_ACTIVATION	1.150269	0.168	0.66688
GOBP_PROSTANOID_BIOSYNTHETIC_PROCESS	1.150181	0.278	0.66666
GOBP_PLATELET_ACTIVATION	1.149729	0.204	0.66781
GOBP_FIBROBLAST_GROWTH_FACTOR_RECEPTOR_SIGN	1.149635	0.237	0.66764
GOBP_POSITIVE_REGULATION_OF_MORPHOGENESIS_OF_	1.149509	0.271	0.66757
GOBP_EXOCYTOSIS	1.149389	0.12	0.66749
GOBP_INTERLEUKIN_17_PRODUCTION	1.14897	0.291	0.66855
GOBP_NEGATIVE_REGULATION_OF_PRODUCTION_OF_MO	1.14896	0.277	0.66804
GOBP_DEFENSE_RESPONSE_TO_GRAM_POSITIVE_BACTE	1.148771	0.244	0.66828
GOBP_RESPONSE_TO_NITRIC_OXIDE	1.14871	0.302	0.66797
GOBP_NEGATIVE_REGULATION_OF_OXIDATIVE_STRESS_	1.148672	0.288	0.66758
GOBP_RESPONSE_TO_ESTRADIOL	1.148566	0.212	0.66743
GOBP_REGULATION_OF_DNA_RECOMBINATION	1.148016	0.234	0.66909
GOBP_POSITIVE_REGULATION_OF_B_CELL_ACTIVATION	1.147524	0.242	0.67046
GOBP_REGULATION_OF_CENTROSOME_CYCLE	1.147064	0.275	0.67168
GOBP_PLATELET_DERIVED_GROWTH_FACTOR_RECEPTO	1.147029	0.252	0.67126
GOBP_REGULATION_OF_CHEMOTAXIS	1.146749	0.179	0.67181
GOBP_MUSCLE_CELL_CELLULAR_HOMEOSTASIS	1.14672	0.28	0.67139
GOBP_MORPHOGENESIS_OF_AN_EPITHELIAL_SHEET	1.146478	0.271	0.67179
GOBP_INSULIN_LIKE_GROWTH_FACTOR_RECEPTOR_SIGN	1.146305	0.276	0.67191
GOBP_REGULATION_OF_PROTEIN_AUTOPHOSPHORYLAT	1.146273	0.266	0.67149
GOBP_MONOAMINE_TRANSPORT	1.145686	0.241	0.67319
GOBP_REGULATION_OF_RESPONSE_TO_EXTRACELLULAR	1.145559	0.273	0.6731
GOBP_PHAGOCYTOSIS	1.145465	0.163	0.67292
GOBP_REGULATION_OF_CD4_POSITIVE_ALPHA_BETA_T_C	1.145284	0.254	0.67311
GOBP_CARDIAC_MUSCLE_CELL_PROLIFERATION	1.145195	0.271	0.67292
GOBP_CARDIAC_CONDUCTION_SYSTEM_DEVELOPMENT	1.145088	0.289	0.67275
GOBP_FIBROBLAST_MIGRATION	1.144922	0.239	0.67285
GOBP_RESPONSE_TO_AMINO_ACID_STARVATION	1.144888	0.274	0.67246
GOBP_REGULATION_OF_SENSORY_PERCEPTION	1.144872	0.289	0.67197
GOBP_REGULATION_OF_T_HELPER_17_TYPE_IMMUNE_RE	1.14474	0.285	0.67194
GOBP_TELENCEPHALON_DEVELOPMENT	1.144607	0.162	0.67188
GOBP_POSITIVE_REGULATION_OF_STEROL_TRANSPORT	1.144594	0.296	0.67141
GOBP_SARCOPLASMIC_RETICULUM_CALCIUM_ION_TRANS	1.144136	0.272	0.67264
GOBP_REGULATION_OF_TRANS_SYNAPTIC_SIGNALING	1.14335	0.1	0.67517
GOBP_NCRNA_TRANSCRIPTION	1.143044	0.271	0.67586
GOBP_VERY_LONG_CHAIN_FATTY_ACID_METABOLIC_PRO	1.142983	0.3	0.67558
GOBP_CELLULAR_RESPONSE_TO_ARSENIC_CONTAINING	1.142949	0.289	0.67517
GOBP_REGULATION_OF_LYMPHOCYTE_MEDIATED_IMMUN	1.142899	0.229	0.67484
GOBP_MATURE_B_CELL_DIFFERENTIATION	1.142644	0.289	0.67527
GOBP_MRNA_EXPORT_FROM_NUCLEUS	1.142357	0.254	0.67584
GOBP_REGULATION_OF_DNA_REPAIR	1.14225	0.209	0.6757
GOBP_RETINA_HOMEOSTASIS	1.142247	0.241	0.67518
GOBP_POSITIVE_REGULATION_OF_CELL_DIVISION	1.142146	0.249	0.67502
GOBP_CELLULAR_RESPONSE_TO_ALCOHOL	1.142015	0.258	0.67499
GOBP_POSITIVE_REGULATION_OF_FATTY_ACID_BIOSYNT	1.141766	0.299	0.6754
GOBP_ENDOPLASMIC_RETICULUM_TUBULAR_NETWORK_	1.141369	0.3	0.67638
GOBP_NEGATIVE_REGULATION_OF_CELL_PROJECTION_O	1.140762	0.189	0.67819
GOBP_EMBRYONIC_AXIS_SPECIFICATION	1.140638	0.275	0.67815
GOBP_CELLULAR_RESPONSE_TO_ABIOTIC_STIMULUS	1.140587	0.134	0.67781
GOBP_RETINA_DEVELOPMENT_IN_CAMERA_TYPE_EYE	1.140468	0.228	0.67771

GOBP_CELLULAR_MONOVALENT_INORGANIC_CATION_HO	1.140136	0.25	0.67849
GOBP_POSITIVE_REGULATION_OF_DENDRITE_MORPHOG	1.139917	0.272	0.67879
GOBP_REGULATION_OF_NEUTROPHIL_MIGRATION	1.13916	0.277	0.68115
GOBP_LYMPHOCYTE_MEDIATED_IMMUNITY	1.139092	0.185	0.68088
GOBP_FOREBRAIN_GENERATION_OF_NEURONS	1.13905	0.281	0.68053
GOBP_CD4_POSITIVE_ALPHA_BETA_T_CELL_DIFFERENTIA	1.139017	0.253	0.68011
GOBP_REGULATION_OF_P38MAPK_CASCADE	1.13886	0.29	0.68019
GOBP_REGULATION_OF_ESTABLISHMENT_OR_MAINTENA	1.138754	0.303	0.68006
GOBP_CELL_DIFFERENTIATION_IN_HINDBRAIN	1.138666	0.302	0.67985
GOBP_REGULATION_OF_VESICLE_MEDIATED_TRANSPORT	1.138454	0.091	0.68011
GOBP_REGULATION_OF_CHROMOSOME_SEPARATION	1.137951	0.257	0.68144
GOBP_CELLULAR_RESPONSE_TO_REACTIVE_NITROGEN_	1.137904	0.296	0.68109
GOBP_REGULATION_OF_NIK_NF_KAPPAB_SIGNALING	1.137879	0.241	0.68067
GOBP_OSTEOBLAST_DIFFERENTIATION	1.137545	0.167	0.68144
GOBP_TETRAHYDROFOLATE_METABOLIC_PROCESS	1.137466	0.311	0.68122
GOBP_NEGATIVE_REGULATION_OF_NEURON_PROJECTIO	1.137168	0.224	0.68184
GOBP_REGULATION_OF_BEHAVIOR	1.136717	0.275	0.68303
GOBP_POSITIVE_REGULATION_OF_MONONUCLEAR_CELL	1.136705	0.28	0.68254
GOBP_POSITIVE_REGULATION_OF_NIK_NF_KAPPAB_SIGN	1.136622	0.282	0.68229
GOBP_PROTEIN_LOCALIZATION_TO_CYTOSKELETON	1.136368	0.26	0.68277
GOBP_ALPHA_BETA_T_CELL_ACTIVATION	1.136256	0.235	0.68269
GOBP_POSITIVE_REGULATION_OF_SYNAPSE_ASSEMBLY	1.135978	0.272	0.68323
GOBP_RESPONSE_TO_TEMPERATURE_STIMULUS	1.135974	0.194	0.68271
GOBP_PROTEIN_K63_LINKED_UBIQUITINATION	1.135928	0.282	0.68235
GOBP_MEMBRANE_RAFT_ORGANIZATION	1.135694	0.301	0.68273
GOBP_CELLULAR_ALDEHYDE_METABOLIC_PROCESS	1.135443	0.275	0.6832
GOBP_EMBRYONIC_SKELETAL_SYSTEM_MORPHOGENESI	1.135186	0.262	0.68366
GOBP_MULTICELLULAR_ORGANISMAL_RESPONSE_TO_ST	1.134973	0.256	0.68392
GOBP_BODY_FLUID_SECRETION	1.134948	0.25	0.68349
GOBP_REGULATION_OF_PEPTIDYL_THREONINE_PHOSPH	1.134865	0.282	0.68327
GOBP_REGULATION_OF_FILOPODIUM_ASSEMBLY	1.13449	0.283	0.68417
GOBP_FOREBRAIN_DEVELOPMENT	1.134471	0.15	0.68371
GOBP_POSITIVE_REGULATION_OF_RECEPTOR_SIGNALIN	1.134466	0.296	0.68321
GOBP_HEPARAN_SULFATE_PROTEOGLYCAN_BIOSYNTHE	1.134053	0.3	0.68426
GOBP_POSITIVE_REGULATION_OF_ENDOCYTOSIS	1.133582	0.248	0.6855
GOBP_PRODUCTION_OF_MOLECULAR_MEDIATOR_INVOLV	1.133332	0.261	0.68592
GOBP_NEGATIVE_REGULATION_OF_OXIDATIVE_STRESS_	1.133067	0.289	0.68644
GOBP_MYELOID_CELL_DIFFERENTIATION	1.133066	0.14	0.68592
GOBP_MICROTUBULE_ANCHORING	1.132999	0.282	0.68567
GOBP_DENDRITE_EXTENSION	1.13292	0.296	0.68548
GOBP_POSITIVE_REGULATION_OF_TRANSLATIONAL_INITI	1.132651	0.299	0.68601
GOBP_SODIUM_ION_TRANSPORT	1.132607	0.201	0.68568
GOBP_SYNAPTIC_TRANSMISSION_GLUTAMATERGIC	1.132205	0.267	0.68665
GOBP_NEUROMUSCULAR_JUNCTION_DEVELOPMENT	1.131977	0.277	0.68704
GOBP_NEGATIVE_REGULATION_OF_INTERLEUKIN_12_PRO	1.131878	0.297	0.68687
GOBP_REGULATION_OF_NEURON_PROJECTION_REGENE	1.131856	0.324	0.68643
GOBP_METHYLATION	1.131827	0.13	0.68604
GOBP_SODIUM_ION_TRANSMEMBRANE_TRANSPORT	1.131541	0.221	0.68664
GOBP_NEURON_DEATH_IN_RESPONSE_TO_OXIDATIVE_ST	1.130987	0.29	0.68818
GOBP_REGULATION_OF_ACTIN_FILAMENT_BASED_MOVEI	1.13095	0.313	0.6878
GOBP_LYTIC_VACUOLE_ORGANIZATION	1.13092	0.27	0.68739
GOBP_TISSUE_MIGRATION	1.130229	0.158	0.68951
GOBP_RESPONSE_TO_INCREASED_OXYGEN_LEVELS	1.130126	0.317	0.68939

GOBP_RESPONSE_TO_DOPAMINE	1.129969	0.272	0.68948
GOBP_CARDIAC_CHAMBER_DEVELOPMENT	1.129701	0.225	0.69
GOBP_REGULATION_OF_KERATINOCYTE_DIFFERENTIATI(	1.129603	0.32	0.68986
GOBP_SPERMATID_NUCLEUS_DIFFERENTIATION	1.129445	0.33	0.68996
GOBP_REGULATION_OF_LYMPHOCYTE_ACTIVATION	1.129199	0.132	0.69038
GOBP_ENDONUCLEOLYTIC_CLEAVAGE_INVOLVED_IN_RRI	1.12885	0.334	0.69114
GOBP_TRANSLATIONAL_INITIATION	1.128738	0.239	0.69106
GOBP_REGULATION_OF_COLLAGEN_METABOLIC_PROCES	1.12782	0.309	0.69409
GOBP_ENDOTHELIAL_CELL_MIGRATION	1.127644	0.195	0.69423
GOBP_DNA_DAMAGE_RESPONSE_SIGNAL_TRANSDUCTIO	1.127618	0.268	0.69381
GOBP_POSITIVE_REGULATION_OF_GLYCOLYTIC_PROCES	1.127286	0.316	0.69452
GOBP_NEGATIVE_REGULATION_OF_VASCULAR_ASSOCIA	1.126418	0.307	0.69728
GOBP_B_CELL_MEDIATED_IMMUNITY	1.126267	0.264	0.69734
GOBP_DNA_REPLICATION	1.126241	0.183	0.69691
GOBP_CILIUM_MOVEMENT	1.126068	0.245	0.69703
GOBP_POSITIVE_REGULATION_OF_VIRAL_GENOME_REPL	1.125639	0.298	0.6981
GOBP_SECRETION_BY_TISSUE	1.125572	0.301	0.69785
GOBP_POSITIVE_REGULATION_OF_TUMOR_NECROSIS_F/	1.125456	0.267	0.69778
GOBP_REGULATION_OF_URINE_VOLUME	1.125439	0.311	0.69732
GOBP_LONG_TERM_SYNAPTIC_DEPRESSION	1.125351	0.317	0.69715
GOBP_REGULATION_OF_NUCLEOCYTOPLASMIC_TRANSP(	1.12532	0.253	0.69675
GOBP_CELLULAR_RESPONSE_TO_INORGANIC_SUBSTAN(	1.125078	0.206	0.69717
GOBP_POSITIVE_REGULATION_OF_STRESS_ACTIVATED_	1.124718	0.255	0.69801
GOBP_POSITIVE_REGULATION_OF_POSTTRANSCRIPTION	1.124682	0.315	0.69766
GOBP_POSITIVE_REGULATION_OF_BEHAVIOR	1.124089	0.317	0.69938
GOBP_NEGATIVE_REGULATION_OF_T_CELL_APOPTOTIC_	1.123924	0.319	0.69949
GOBP_POSITIVE_REGULATION_OF_CELL_SUBSTRATE_AD	1.123847	0.254	0.69929
GOBP_OVARIAN_FOLLICLE_DEVELOPMENT	1.123826	0.317	0.69886
GOBP_REGULATION_OF_RRNA_PROCESSING	1.123678	0.317	0.69891
GOBP_POSITIVE_REGULATION_OF_RHO_PROTEIN_SIGNA	1.123526	0.31	0.69896
GOBP_POSITIVE_REGULATION_OF_INTERLEUKIN_17_PRO	1.123103	0.316	0.70008
GOBP_ENGULFMENT_OF_APOPTOTIC_CELL	1.123057	0.334	0.69974
GOBP_RNA_POLYADENYLATION	1.122959	0.29	0.6996
GOBP_METAPHASE_ANAPHASE_TRANSITION_OF_CELL_C	1.122667	0.268	0.70015
GOBP_LUNG_EPITHELIUM_DEVELOPMENT	1.122663	0.302	0.69965
GOBP_BONE_CELL_DEVELOPMENT	1.122647	0.317	0.69919
GOBP_MAST_CELL_ACTIVATION	1.122624	0.287	0.69877
GOBP_DEVELOPMENTAL_MATURATION	1.122556	0.199	0.6985
GOBP_REGULATION_OF_DENDRITIC_SPINE_DEVELOPMEN	1.12252	0.277	0.69811
GOBP_REGULATION_OF_POSTSYNAPTIC_MEMBRANE_PO	1.122516	0.258	0.69762
GOBP_ESTABLISHMENT_OR_MAINTENANCE_OF_BIPOLAR	1.122017	0.302	0.69901
GOBP_UROGENITAL_SYSTEM_DEVELOPMENT	1.122015	0.164	0.6985
GOBP_POSITIVE_REGULATION_OF_SMOOTH_MUSCLE_CE	1.121993	0.26	0.69808
GOBP_POSITIVE_REGULATION_OF_LEUKOCYTE_CHEMOT	1.121884	0.267	0.69796
GOBP_NEGATIVE_REGULATION_OF_TUMOR_NECROSIS_F	1.121463	0.335	0.69906
GOBP_TOLL_LIKE_RECEPTOR_SIGNALING_PATHWAY	1.121408	0.262	0.69876
GOBP_MUSCLE_CONTRACTION	1.121171	0.18	0.69913
GOBP_MYELOID_DENDRITIC_CELL_ACTIVATION	1.121059	0.326	0.69905
GOBP_REGULATION_OF_SYNAPTIC_TRANSMISSION_GLUT	1.120996	0.279	0.69877
GOBP_REGULATION_OF_HISTONE_H3_K9_METHYLATION	1.120917	0.314	0.69854
GOBP_GLUTAMATE_METABOLIC_PROCESS	1.120484	0.318	0.69966
GOBP_EMBRYONIC_ORGAN_MORPHOGENESIS	1.120423	0.185	0.69938
GOBP_EMBRYONIC_DIGESTIVE_TRACT_DEVELOPMENT	1.120272	0.3	0.69944

GOBP_RESPONSE_TO_PURINE_CONTAINING_COMPOUND	1.120171	0.266	0.69936
GOBP_BENZENE_CONTAINING_COMPOUND_METABOLIC_F	1.119988	0.318	0.69954
GOBP_POSITIVE_REGULATION_OF_GROWTH	1.119316	0.224	0.70162
GOBP_MESENCHYMAL_TO_EPITHELIAL_TRANSITION	1.119186	0.353	0.70158
GOBP_POSITIVE_REGULATION_OF_NEUROGENESIS	1.11896	0.237	0.70202
GOBP_REGULATION_OF_NEURON_MIGRATION	1.118786	0.299	0.70218
GOBP_POSITIVE_REGULATION_OF_NF_KAPPAB_TRANSCF	1.118619	0.253	0.7023
GOBP_REGULATION_OF_PEPTIDASE_ACTIVITY	1.118502	0.159	0.70223
GOBP_SECOND_MESSENGER_MEDIATED_SIGNALING	1.117605	0.198	0.705
GOBP_NUCLEOTIDE_TRANSPORT	1.11735	0.321	0.70546
GOBP_L_AMINO_ACID_TRANSPORT	1.117315	0.289	0.70508
GOBP_CELL_MATRIX_ADHESION	1.117255	0.209	0.70482
GOBP_MYELOID_CELL_ACTIVATION_INVOLVED_IN_IMMUN	1.117248	0.281	0.70433
GOBP_NEGATIVE_REGULATION_OF_MAP_KINASE_ACTIVIT	1.117141	0.295	0.70422
GOBP_EMBRYONIC_SKELETAL_SYSTEM_DEVELOPMENT	1.116925	0.259	0.70451
GOBP_NEURAL_TUBE_FORMATION	1.116816	0.27	0.70442
GOBP_T_CELL_MIGRATION	1.116761	0.288	0.70413
GOBP_CELLULAR_RESPONSE_TO_LIPID	1.116756	0.118	0.70364
GOBP_REGULATION_OF_CYTOKINESIS	1.116747	0.291	0.70317
GOBP_REGULATION_OF_MEIOTIC_CELL_CYCLE	1.116581	0.292	0.70332
GOBP_MATURATION_OF_SSU_RRNA_FROM_TRICISTRONI(	1.116533	0.317	0.70301
GOBP_COLLAGEN_BIOSYNTHETIC_PROCESS	1.116416	0.31	0.70295
GOBP_AMYLOID_BETA_CLEARANCE	1.116305	0.317	0.70287
GOBP_BLOOD_VESSEL_ENDOTHELIAL_CELL_MIGRATION	1.116117	0.277	0.70312
GOBP_NEGATIVE_REGULATION_OF_CANONICAL_WNT_SI(	1.115944	0.264	0.70327
GOBP_RNA_3_END_PROCESSING	1.115534	0.278	0.70431
GOBP_REGULATION_OF_LYMPHOCYTE_CHEMOTAXIS	1.115323	0.328	0.70462
GOBP_POSITIVE_REGULATION_OF_ATP_METABOLIC_PRO	1.115272	0.326	0.70431
GOBP_OLEFINIC_COMPOUND_METABOLIC_PROCESS	1.115223	0.277	0.70399
GOBP_RESPONSE_TO_OXYGEN_RADICAL	1.115098	0.326	0.70397
GOBP_POSITIVE_REGULATION_OF_MEMBRANE_PERMEAE	1.114832	0.308	0.7044
GOBP_WOUND_HEALING_SPREADING_OF_EPIDERMAL_CE	1.11449	0.328	0.70525
GOBP_CYTOPLASMIC_PATTERN_RECOGNITION_RECEPTO	1.114339	0.336	0.70533
GOBP_KERATINOCYTE_MIGRATION	1.114297	0.347	0.70501
GOBP_CELL_RECOGNITION	1.114291	0.265	0.70454
GOBP_REGULATION_OF_INTRINSIC_APOPTOTIC_SIGNALII	1.114283	0.251	0.70407
GOBP_OLIGOSACCHARIDE_LIPID_INTERMEDIATE_BIOSYN	1.113827	0.341	0.70528
GOBP_EMBRYONIC_ORGAN_DEVELOPMENT	1.113607	0.158	0.70561
GOBP_POSITIVE_REGULATION_OF_LIPID_METABOLIC_PR(	1.113571	0.257	0.70525
GOBP_NEGATIVE_REGULATION_OF_DNA_BIOSYNTHETIC_	1.113259	0.31	0.70593
GOBP_REPRODUCTIVE_SYSTEM_DEVELOPMENT	1.113233	0.155	0.70553
GOBP_REGULATION_OF_RIG_I_SIGNALING_PATHWAY	1.113163	0.323	0.70528
GOBP_POSITIVE_REGULATION_OF_PROTEIN_TARGETING	1.112618	0.323	0.70684
GOBP_POSITIVE_REGULATION_OF_IMMUNE_RESPONSE	1.112362	0.17	0.70731
GOBP_AMINOGLYCAN_CATABOLIC_PROCESS	1.112286	0.312	0.70712
GOBP_REGULATION_OF_PROTEIN_TARGETING_TO_MEME	1.112139	0.329	0.70716
GOBP_MRNA_CLEAVAGE	1.111875	0.34	0.7077
GOBP_NEGATIVE_REGULATION_OF_LOCOMOTION	1.11146	0.2	0.70877
GOBP_REGULATION_OF_STRESS_ACTIVATED_PROTEIN_K	1.11143	0.249	0.70838
GOBP_POSITIVE_REGULATION_OF_DNA_REPAIR	1.111389	0.301	0.70804
GOBP_CELLULAR_MODIFIED_AMINO_ACID_METABOLIC_PF	1.111062	0.251	0.70876
GOBP_ORGAN_GROWTH	1.110955	0.263	0.70866
GOBP_CARDIOCYTE_DIFFERENTIATION	1.110294	0.266	0.71069

GOBP_MESENCHYMAL_CELL_DIFFERENTIATION	1.110089	0.216	0.71096
GOBP_MRNA_METHYLATION	1.110066	0.314	0.71056
GOBP_NADP_METABOLIC_PROCESS	1.10995	0.333	0.7105
GOBP_PROTEIN_LOCALIZATION_TO_CILIUM	1.109687	0.305	0.71102
GOBP_REGULATION_OF_NUCLEAR_DIVISION	1.108688	0.268	0.71431
GOBP_MITOTIC_SISTER_CHROMATID_COHESION	1.108679	0.34	0.71385
GOBP_MODULATION_OF_EXCITATORY_POSTSYNAPTIC_P	1.108486	0.336	0.71408
GOBP_TRANSPORT_ALONG_MICROTUBULE	1.108384	0.273	0.71397
GOBP_NEGATIVE_REGULATION_OF_GLIOGENESIS	1.10785	0.344	0.71548
GOBP_CARDIAC_MUSCLE_CELL_ACTION_POTENTIAL	1.107822	0.307	0.71508
GOBP_PYRIDINE_NUCLEOTIDE_BIOSYNTHETIC_PROCESS	1.107817	0.335	0.7146
GOBP_COCHLEA_DEVELOPMENT	1.107778	0.306	0.71424
GOBP_MUSCLE_TISSUE_DEVELOPMENT	1.107763	0.197	0.71381
GOBP_CARDIOBLAST_DIFFERENTIATION	1.10726	0.338	0.71522
GOBP_NATURAL_KILLER_CELL_MEDIATED_IMMUNITY	1.107215	0.31	0.71492
GOBP_PROTEIN_LOCALIZATION_TO_CONDENSED_CHROM	1.106948	0.337	0.71536
GOBP_POSITIVE_REGULATION_OF_NERVOUS_SYSTEM_D	1.106945	0.231	0.71488
GOBP_POSITIVE_REGULATION_OF_SMAD_PROTEIN_SIGN	1.106764	0.342	0.71505
GOBP_REGULATION_OF_T_CELL_PROLIFERATION	1.106005	0.262	0.71737
GOBP_NEGATIVE_REGULATION_OF_SYNAPTIC_TRANSMIS	1.105977	0.316	0.71698
GOBP_RESPONSE_TO_IMMOBILIZATION_STRESS	1.105873	0.357	0.71687
GOBP_EPITHELIAL_TUBE_MORPHOGENESIS	1.105768	0.211	0.71679
GOBP_REGULATION_OF_NEURON_DIFFERENTIATION	1.105706	0.258	0.7165
GOBP_CELLULAR_DEFENSE_RESPONSE	1.104933	0.329	0.71892
GOBP_FC_EPSILON_RECEPTOR_SIGNALING_PATHWAY	1.104147	0.347	0.72145
GOBP_KINETOCHORE_ASSEMBLY	1.104006	0.375	0.72148
GOBP_REGULATION_OF_TRANSLATIONAL_INITIATION	1.103663	0.297	0.72228
GOBP_VACUOLAR_LOCALIZATION	1.103602	0.296	0.72198
GOBP_PROTEIN_AUTOUBIQUITINATION	1.103437	0.324	0.72208
GOBP_MUSCLE_CELL_DIFFERENTIATION	1.103388	0.186	0.72177
GOBP_ASTROCYTE_ACTIVATION	1.103064	0.332	0.72248
GOBP_DEFENSE_RESPONSE_TO_SYMBIONT	1.103055	0.259	0.72202
GOBP_PROTEIN_LOCALIZATION_TO_CHROMOSOME_CENT	1.102981	0.343	0.72182
GOBP_POSITIVE_REGULATION_OF_COLD_INDUCED_THEF	1.102954	0.293	0.72143
GOBP_RESPONSE_TO_DIETARY_EXCESS	1.102827	0.347	0.72142
GOBP_POSITIVE_REGULATION_OF_GLUCOSE_IMPORT	1.102793	0.343	0.72104
GOBP_AXON_EXTENSION	1.102668	0.286	0.72101
GOBP_PROTEIN_LOCALIZATION_TO_MICROTUBULE_ORGA	1.101666	0.339	0.7243
GOBP_RESPONSE_TO_TOXIC_SUBSTANCE	1.101435	0.272	0.72469
GOBP_REGULATION_OF_MITOTIC_CELL_CYCLE	1.101394	0.181	0.72437
GOBP_ONE_CARBON_METABOLIC_PROCESS	1.101319	0.341	0.72416
GOBP_EMBRYONIC_PATTERN_SPECIFICATION	1.100219	0.319	0.72775
GOBP_ESTABLISHMENT_OF_PROTEIN_LOCALIZATION_TO	1.100175	0.258	0.72743
GOBP_CGMP_MEDIATED_SIGNALING	1.100067	0.359	0.72735
GOBP_TUBE_FORMATION	1.099583	0.272	0.7287
GOBP_NEGATIVE_REGULATION_OF_NF_KAPPAB_TRANSC	1.099538	0.306	0.72837
GOBP_LIMBIC_SYSTEM_DEVELOPMENT	1.099527	0.305	0.72792
GOBP_FAT_SOLUBLE_VITAMIN_METABOLIC_PROCESS	1.099042	0.34	0.72922
GOBP_POSITIVE_REGULATION_OF_NEURON_APOPTOTIC_	1.098993	0.341	0.7289
GOBP_PURINE_CONTAINING_COMPOUND_TRANSMEMBRA	1.098632	0.366	0.72974
GOBP_FERTILIZATION	1.098625	0.278	0.72928
GOBP_GAMMA_AMINOBUTYRIC_ACID_SIGNALING_PATHW	1.097921	0.34	0.73143
GOBP_IMPORT_INTO_NUCLEUS	1.097428	0.285	0.73287

GOBP_REGULATION_OF_NEUTROPHIL_CHEMOTAXIS	1.097415	0.353	0.73243
GOBP_LENS_FIBER_CELL_DIFFERENTIATION	1.097173	0.352	0.73286
GOBP_CARDIAC_CHAMBER_MORPHOGENESIS	1.096985	0.309	0.73307
GOBP_POSITIVE_REGULATION_OF_EXTRINSIC_APOPTOTI	1.09688	0.344	0.73296
GOBP_PURINE_NUCLEOTIDE_TRANSPORT	1.096435	0.369	0.73423
GOBP_POSITIVE_REGULATION_OF_DEVELOPMENTAL_GR	1.096405	0.294	0.73385
GOBP_REGULATION_OF_TRANSCRIPTION_BY_RNA_POLY	1.095977	0.347	0.73497
GOBP_NUCLEOTIDE_TRANSMEMBRANE_TRANSPORT	1.095647	0.364	0.73571
GOBP_REGULATION_OF_OLIGODENDROCYTE_DIFFERENT	1.095384	0.341	0.73619
GOBP_REGULATION_OF_RNA_SPLICING	1.095243	0.299	0.73621
GOBP_PLASMA_MEMBRANE_TUBULATION	1.095012	0.361	0.73657
GOBP_RESPONSE_TO_PLATELET_DERIVED_GROWTH_FA	1.094338	0.364	0.73857
GOBP_RESPONSE_TO_ACTIVITY	1.094115	0.331	0.7389
GOBP_REGULATION_OF_PROTEIN_CONTAINING_COMPLE	1.093892	0.23	0.73926
GOBP_EMBRYONIC_FORELIMB_MORPHOGENESIS	1.093753	0.341	0.73929
GOBP_TYPE_2_IMMUNE_RESPONSE	1.093751	0.349	0.73881
GOBP_REGULATION_OF_RESPIRATORY_GASEOUS_EXCH	1.093523	0.344	0.73923
GOBP_PROTEIN_METHYLATION	1.093018	0.28	0.74068
GOBP_MICROTUBULE_NUCLEATION	1.092997	0.35	0.74028
GOBP_PEPTIDYL_PROLINE_MODIFICATION	1.092902	0.343	0.74016
GOBP_MEMBRANE_LIPID_CATABOLIC_PROCESS	1.09288	0.366	0.73976
GOBP_SOMATIC_DIVERSIFICATION_OF_IMMUNOGLOBULIN	1.092466	0.33	0.74079
GOBP_SENSORY_ORGAN_MORPHOGENESIS	1.092431	0.247	0.74042
GOBP_REGULATION_OF_RAC_PROTEIN_SIGNAL_TRANSD	1.092142	0.377	0.74095
GOBP_RESPONSE_TO_ALCOHOL	1.092032	0.271	0.74085
GOBP_NUCLEAR_TRANSPORT	1.091858	0.259	0.74102
GOBP_NEGATIVE_REGULATION_OF_NEURAL_PRECURSOR	1.09166	0.385	0.74123
GOBP_CANONICAL_WNT_SIGNALING_PATHWAY	1.091607	0.238	0.74093
GOBP_IMMUNE_RESPONSE_REGULATING_SIGNALING_PA	1.091575	0.225	0.74056
GOBP_MACROPHAGE_ACTIVATION_INVOLVED_IN_IMMUNE	1.091544	0.366	0.74019
GOBP_GLUCOSAMINE_CONTAINING_COMPOUND_METABO	1.090945	0.352	0.74196
GOBP_REGULATION_OF_CD4_POSITIVE_ALPHA_BETA_T_	1.090166	0.336	0.74438
GOBP_REGULATION_OF_PRESYNAPSE_ORGANIZATION	1.090102	0.363	0.74413
GOBP_C4_DICARBOXYLATE_TRANSPORT	1.089809	0.359	0.74472
GOBP_REGULATION_OF_PRODUCTION_OF_MOLECULAR_	1.089726	0.313	0.74452
GOBP_OLIGODENDROCYTE_DEVELOPMENT	1.089724	0.347	0.74404
GOBP_TOLL_LIKE_RECEPTOR_9_SIGNALING_PATHWAY	1.089618	0.388	0.74392
GOBP_ORGANIC_ANION_TRANSPORT	1.089542	0.247	0.74373
GOBP_WATER_HOMEOSTASIS	1.089412	0.346	0.74375
GOBP_POSITIVE_REGULATION_OF_WNT_SIGNALING_PAT	1.089392	0.319	0.74334
GOBP_HETEROTYPIC_CELL_CELL_ADHESION	1.089349	0.353	0.74301
GOBP_REGULATION_OF_CELL_SIZE	1.089328	0.292	0.7426
GOBP_POSITIVE_REGULATION_OF_T_CELL_MIGRATION	1.08903	0.381	0.74324
GOBP_TOOTH_MINERALIZATION	1.088483	0.362	0.74476
GOBP_REGULATION_OF_RESPONSE_TO_INTERFERON_G	1.088481	0.374	0.74428
GOBP_SMOOTH_MUSCLE_CONTRACTION	1.088341	0.33	0.74429
GOBP_REGULATION_OF_NEUROBLAST_PROLIFERATION	1.088136	0.373	0.74455
GOBP_POSITIVE_REGULATION_OF_CELL_DEATH	1.087946	0.18	0.74472
GOBP_NEGATIVE_REGULATION_OF_LEUKOCYTE_CHEMOT	1.087705	0.375	0.74512
GOBP_TRANSPOSITION	1.087698	0.368	0.74467
GOBP_REGULATION_OF_MICROTUBULE_DEPOLYMERIZAT	1.087498	0.375	0.74487
GOBP_PROTEIN_O_LINKED_GLYCOSYLATION	1.087464	0.335	0.74451
GOBP_REGULATION_OF_ACTIN_FILAMENT_BASED_PROCE	1.087411	0.234	0.74423

GOBP_REGULATION_OF_TOR_SIGNALING	1.087234	0.318	0.74438
GOBP_NEGATIVE_REGULATION_OF_CELLULAR_AMIDE_ME	1.08698	0.29	0.7448
GOBP_NEGATIVE_REGULATION_OF_CYTOKINE_PRODUCT	1.08697	0.282	0.74435
GOBP_STRESS_ACTIVATED_PROTEIN_KINASE_SIGNALING	1.086884	0.283	0.74417
GOBP_IMMUNE_EFFECTOR_PROCESS	1.086763	0.216	0.74412
GOBP_NEGATIVE_REGULATION_OF_PROTEIN_LOCALIZAT	1.086632	0.374	0.74413
GOBP_LOW_DENSITY_LIPOPROTEIN_PARTICLE_CLEARAN	1.086518	0.378	0.74408
GOBP_GLUTAMATE_SECRETION	1.086484	0.388	0.74372
GOBP_STRIATED_MUSCLE_CELL_DIFFERENTIATION	1.086301	0.283	0.7439
GOBP_LIPOPROTEIN_BIOSYNTHETIC_PROCESS	1.086006	0.34	0.74447
GOBP_CELL_CYCLE_G1_S_PHASE_TRANSITION	1.085954	0.289	0.7442
GOBP_OOCYTE_DIFFERENTIATION	1.085516	0.352	0.7453
GOBP_KILLING_OF_CELLS_OF_ANOTHER_ORGANISM	1.085496	0.375	0.74488
GOBP_NEGATIVE_REGULATION_OF_LIPID_STORAGE	1.085482	0.372	0.74445
GOBP_TELOMERASE_RNA_LOCALIZATION	1.085152	0.359	0.74521
GOBP_REGULATION_OF_ALPHA_BETA_T_CELL_ACTIVATIO	1.085055	0.328	0.74506
GOBP_REGULATION_OF_PROTEIN_DEACETYLATION	1.085034	0.356	0.74466
GOBP_NEGATIVE_REGULATION_OF_INFLAMMATORY_RES	1.084792	0.308	0.74507
GOBP_REGULATION_OF_NATURAL_KILLER_CELL_MEDIAT	1.084413	0.36	0.74595
GOBP_FEMALE_MEIOTIC_NUCLEAR_DIVISION	1.084289	0.374	0.74591
GOBP_POSITIVE_REGULATION_OF_T_CELL_CYTOKINE_PF	1.083876	0.372	0.74693
GOBP_VASODILATION	1.083798	0.37	0.74675
GOBP_NUCLEOTIDE_SUGAR_METABOLIC_PROCESS	1.083119	0.371	0.74877
GOBP_CELLULAR_RESPONSE_TO_OXYGEN_RADICAL	1.082893	0.387	0.74912
GOBP_REGULATION_OF_MRNA_PROCESSING	1.082791	0.342	0.74903
GOBP_OLIGODENDROCYTE_DIFFERENTIATION	1.0827	0.341	0.74888
GOBP_IMMUNOGLOBULIN_PRODUCTION	1.082645	0.331	0.74862
GOBP_PLATELET_AGGREGATION	1.082603	0.334	0.74829
GOBP_RECOMBINATIONAL_REPAIR	1.082334	0.305	0.74881
GOBP_POSITIVE_REGULATION_OF_PROTEIN_POLYUBIQU	1.082221	0.374	0.74876
GOBP_REGULATION_OF_HORMONE_LEVELS	1.081766	0.247	0.74987
GOBP_FATTY_ACYL_COA_METABOLIC_PROCESS	1.081639	0.353	0.74984
GOBP_ROOF_OF_MOUTH_DEVELOPMENT	1.08124	0.347	0.75084
GOBP_STEM_CELL_DIFFERENTIATION	1.080251	0.309	0.75393
GOBP_FATTY_ACYL_COA_BIOSYNTHETIC_PROCESS	1.080237	0.378	0.75351
GOBP_VIRAL_GENOME_REPLICATION	1.080003	0.339	0.75389
GOBP_MAGNESIUM_ION_TRANSPORT	1.079932	0.387	0.75368
GOBP_NIK_NF_KAPPAB_SIGNALING	1.079467	0.327	0.75491
GOBP_DOPAMINE_METABOLIC_PROCESS	1.07907	0.366	0.75585
GOBP_POSITIVE_REGULATION_OF_CELL_PROJECTION_O	1.079017	0.272	0.75557
GOBP_REGULATION_OF_DNA_METABOLIC_PROCESS	1.07869	0.266	0.75629
GOBP_POSITIVE_REGULATION_OF_ORGAN_GROWTH	1.078502	0.366	0.7565
GOBP_MICROTUBULE_POLYMERIZATION_OR_DEPOLYMEF	1.077884	0.342	0.75827
GOBP_EXTRACELLULAR_MATRIX_DISASSEMBLY	1.077869	0.358	0.75785
GOBP_POSTSYNAPTIC_SPECIALIZATION_ASSEMBLY	1.077226	0.383	0.75974
GOBP_METANEPHRIC_NEPHRON_MORPHOGENESIS	1.077151	0.376	0.75955
GOBP_VENTRICULAR_SEPTUM_DEVELOPMENT	1.077071	0.353	0.75937
GOBP_CELLULAR_RESPONSE_TO_VITAMIN	1.077024	0.379	0.75905
GOBP_RESPONSE_TO_ELECTRICAL_STIMULUS	1.076956	0.373	0.75883
GOBP_MAMMARY_GLAND_EPITHELIAL_CELL_DIFFERENTIA	1.076845	0.381	0.75875
GOBP_POSITIVE_REGULATION_OF_CIRCADIAN_RHYTHM	1.076713	0.386	0.75872
GOBP_MITOTIC_RECOMBINATION	1.076	0.372	0.76084
GOBP_REGULATION_OF_SUPEROXIDE_ANION_GENERATIO	1.075797	0.367	0.76113

GOBP_REGULATION_OF_EPITHELIAL_CELL_MIGRATION	1.07566	0.312	0.76115
GOBP_ACTIN_MEDIATED_CELL_CONTRACTION	1.075604	0.363	0.76088
GOBP_REGULATION_OF_BRANCHING_INVOLVED_IN_URET	1.075589	0.396	0.76046
GOBP_CELL_CYCLE_G2_M_PHASE_TRANSITION	1.075478	0.315	0.7604
GOBP_CARDIAC_MUSCLE_TISSUE_MORPHOGENESIS	1.074953	0.359	0.76193
GOBP_REGULATION_OF_PROTEASOMAL_UBIQUITIN_DEPE	1.074923	0.339	0.76155
GOBP_PORPHYRIN_CONTAINING_COMPOUND_METABOLIC	1.074525	0.375	0.76252
GOBP_ENDODERMAL_CELL_DIFFERENTIATION	1.074477	0.383	0.76223
GOBP_NITRIC_OXIDE_MEDIATED_SIGNAL_TRANSDUCTION	1.074465	0.375	0.7618
GOBP_DSRNA_PROCESSING	1.07394	0.379	0.76321
GOBP_REGULATION_OF_RECEPTOR_MEDIATED_ENDOCY	1.073748	0.357	0.76341
GOBP_SYNAPTIC_VESICLE_RECYCLING	1.07365	0.374	0.76329
GOBP_MICROTUBULE_BASED_MOVEMENT	1.073634	0.29	0.76288
GOBP_CELLULAR_RESPONSE_TO_TOXIC_SUBSTANCE	1.073561	0.351	0.76267
GOBP_REGULATION_OF_ISOTYPE_SWITCHING	1.073561	0.363	0.7622
GOBP_MYELOID_LEUKOCYTE_CYTOKINE_PRODUCTION	1.072703	0.365	0.76488
GOBP_METANEPHROS_MORPHOGENESIS	1.07262	0.377	0.76469
GOBP_RESPONSE_TO_EXTRACELLULAR_STIMULUS	1.072579	0.261	0.76435
GOBP_POSITIVE_REGULATION_OF_STEM_CELL_PROLIFEI	1.072542	0.393	0.76402
GOBP_REGULATION_OF_MICROTUBULE_BASED_PROCESS	1.072303	0.326	0.76444
GOBP_CELLULAR_RESPONSE_TO_ALKALOID	1.071971	0.37	0.76519
GOBP_SOMATIC_DIVERSIFICATION_OF_IMMUNOGLOBULIN	1.07184	0.357	0.76519
GOBP_MITOTIC_CELL_CYCLE_PHASE_TRANSITION	1.071828	0.27	0.76475
GOBP_MODULATION_BY_HOST_OF_VIRAL_GENOME_REPL	1.07171	0.393	0.76471
GOBP_REGULATION_OF_GRANULOCYTE_CHEMOTAXIS	1.071588	0.4	0.76468
GOBP_ANTERIOR_POSTERIOR_PATTERN_SPECIFICATION	1.071258	0.335	0.76542
GOBP_NEGATIVE_REGULATION_OF_CELL_AGING	1.071207	0.385	0.76514
GOBP_MRNA_3_END_PROCESSING	1.0711	0.366	0.76506
GOBP_PROTEIN_LOCALIZATION_TO_CILIARY_MEMBRANE	1.070924	0.408	0.76524
GOBP_POSITIVE_REGULATION_OF_INTERLEUKIN_8_PROD	1.070353	0.374	0.76679
GOBP_POSITIVE_REGULATION_OF_CELL_CYCLE_G1_S_PH	1.070218	0.364	0.76679
GOBP_PEPTIDYL_SERINE_MODIFICATION	1.070112	0.293	0.76671
GOBP_POSITIVE_REGULATION_OF_ALPHA_BETA_T_CELL_	1.069987	0.372	0.7667
GOBP_CELL_JUNCTION_ASSEMBLY	1.069877	0.283	0.76663
GOBP_ACTIN_FILAMENT_BASED_MOVEMENT	1.069608	0.365	0.76714
GOBP_PROTEIN_IMPORT	1.06948	0.325	0.76715
GOBP_REGULATION_OF_MITOTIC_CELL_CYCLE_PHASE_T	1.069449	0.305	0.76679
GOBP_PYRIDINE_NUCLEOTIDE_METABOLIC_PROCESS	1.069279	0.391	0.76695
GOBP_NEPHRON_DEVELOPMENT	1.069221	0.35	0.76669
GOBP_NEGATIVE_REGULATION_OF_INTRACELLULAR_SIG	1.068805	0.259	0.76774
GOBP_RHYTHMIC_PROCESS	1.068445	0.301	0.76854
GOBP_SYMPATHETIC_NERVOUS_SYSTEM_DEVELOPMENT	1.068427	0.409	0.76813
GOBP_POSITIVE_REGULATION_OF_MACROPHAGE_DIFFEI	1.068422	0.404	0.76767
GOBP_NEGATIVE_REGULATION_OF_TRANSLATIONAL_INIT	1.068344	0.387	0.76747
GOBP_NEGATIVE_REGULATION_OF_OSSIFICATION	1.068299	0.385	0.76718
GOBP_LIPID_IMPORT_INTO_CELL	1.068214	0.398	0.76702
GOBP_POSITIVE_REGULATION_OF_CHOLESTEROL_EFFLU	1.068144	0.397	0.7668
GOBP_NEGATIVE_REGULATION_OF_BMP_SIGNALING_PAT	1.067886	0.373	0.7673
GOBP_REGULATION_OF_SECRETION	1.067835	0.253	0.76702
GOBP_SMAD_PROTEIN_SIGNAL_TRANSDUCTION	1.067747	0.378	0.76688
GOBP_CELL_CELL_JUNCTION_MAINTENANCE	1.067454	0.398	0.76746
GOBP_IN_UTERO_EMBRYONIC_DEVELOPMENT	1.067219	0.291	0.76783
GOBP_MULTICELLULAR_ORGANISMAL_SIGNALING	1.0672	0.351	0.76743

GOBP_REGULATION_OF_IMMUNOGLOBULIN_PRODUCTION	1.067107	0.396	0.7673
GOBP_POST_TRANSLATIONAL_PROTEIN_MODIFICATION	1.066999	0.372	0.76723
GOBP_IRON_SULFUR_CLUSTER_ASSEMBLY	1.066734	0.387	0.76778
GOBP_HEAD_MORPHOGENESIS	1.066557	0.368	0.76795
GOBP_DNA_GEOMETRIC_CHANGE	1.066531	0.353	0.76758
GOBP_POSITIVE_REGULATION_OF_ALPHA_BETA_T_CELL_	1.066493	0.389	0.76725
GOBP_PROSTATE_GLAND_MORPHOGENESIS	1.066116	0.392	0.7681
GOBP_AMEBOIDAL_TYPE_CELL_MIGRATION	1.065823	0.289	0.76869
GOBP_T_CELL_PROLIFERATION	1.065734	0.324	0.76855
GOBP_PIGMENT_METABOLIC_PROCESS	1.065687	0.371	0.76825
GOBP_CYCLIC_NUCLEOTIDE_MEDIATED_SIGNALING	1.065655	0.369	0.76789
GOBP_NEGATIVE_REGULATION_OF_NIK_NF_KAPPAB_SIG	1.065435	0.4	0.76822
GOBP_TRANSLATIONAL_ELONGATION	1.065177	0.379	0.76871
GOBP_HOMOTYPIC_CELL_CELL_ADHESION	1.065089	0.372	0.76856
GOBP_NEGATIVE_REGULATION_OF_EXOCYTOSIS	1.064966	0.395	0.76853
GOBP_SCHWANN_CELL_DEVELOPMENT	1.064862	0.388	0.76846
GOBP_RESPONSE_TO_STEROID_HORMONE	1.064615	0.316	0.76886
GOBP_PROTEIN_LOCALIZATION_TO_NUCLEUS	1.06454	0.332	0.76868
GOBP_REGULATION_OF_T_CELL_MIGRATION	1.064456	0.407	0.76852
GOBP_MUSCLE_SYSTEM_PROCESS	1.064408	0.293	0.76823
GOBP_LYSOSOMAL_PROTEIN_CATABOLIC_PROCESS	1.064161	0.405	0.76867
GOBP_T_HELPER_17_TYPE_IMMUNE_RESPONSE	1.064027	0.389	0.76868
GOBP_THYROID_HORMONE_GENERATION	1.063924	0.418	0.76856
GOBP_AMIDE_TRANSPORT	1.063862	0.351	0.76832
GOBP_REGULATION_OF_NITRIC_OXIDE_METABOLIC_PRO	1.063135	0.392	0.77047
GOBP_REGULATION_OF_CALCINEURIN_MEDIATED_SIGNA	1.062969	0.388	0.77059
GOBP_ANION_TRANSPORT	1.062968	0.278	0.77013
GOBP_TORC1_SIGNALING	1.062727	0.389	0.7705
GOBP_NEGATIVE_REGULATION_OF_HISTONE_MODIFICAT	1.062598	0.379	0.77051
GOBP_REGULATION_OF_HORMONE_METABOLIC_PROCES	1.062503	0.389	0.77038
GOBP_REGULATION_OF_HISTONE_MODIFICATION	1.062282	0.359	0.77071
GOBP_PEPTIDYL_ARGININE_METHYLATION	1.062095	0.414	0.77092
GOBP_CD4_POSITIVE_ALPHA_BETA_T_CELL_CYTOKINE_P	1.062069	0.391	0.77055
GOBP_PRODUCTION_OF_MOLECULAR_MEDIATOR_OF_IMM	1.061805	0.353	0.77108
GOBP_MYELOID_CELL_HOMEOSTASIS	1.061639	0.358	0.7712
GOBP_REGULATION_OF_LIPID_KINASE_ACTIVITY	1.061539	0.407	0.77107
GOBP_REGULATION_OF_MITOCHONDRIAL_MEMBRANE_PE	1.061069	0.393	0.77225
GOBP_CARDIAC_MUSCLE_CELL_CONTRACTION	1.06103	0.399	0.77193
GOBP_TRABECULA_MORPHOGENESIS	1.060852	0.383	0.77213
GOBP_PORE_COMPLEX_ASSEMBLY	1.0608	0.402	0.77187
GOBP_BINDING_OF_SPERM_TO_ZONA_PELLUCIDA	1.060375	0.399	0.7729
GOBP_POSITIVE_REGULATION_OF_CELL_GROWTH	1.060341	0.36	0.77256
GOBP_SIGNAL_TRANSDUCTION_BY_P53_CLASS_MEDIATO	1.060333	0.373	0.77213
GOBP_CELL_GROWTH	1.060304	0.297	0.77177
GOBP_NEURAL_TUBE_DEVELOPMENT	1.060041	0.353	0.77224
GOBP_PROTEIN_LIPID_COMPLEX_ASSEMBLY	1.059462	0.413	0.77384
GOBP_FORELIMB_MORPHOGENESIS	1.059324	0.401	0.77387
GOBP_CARDIAC_SEPTUM_DEVELOPMENT	1.05918	0.369	0.77394
GOBP_POSITIVE_REGULATION_OF_CALCIUM_MEDIATED_S	1.058942	0.398	0.77432
GOBP_CHEMICAL_SYNAPTIC_TRANSMISSION_POSTSYNAP	1.058819	0.387	0.77429
GOBP_CARBOXYLIC_ACID_TRANSPORT	1.058762	0.353	0.77404
GOBP_POSITIVE_REGULATION_OF_SPROUTING_ANGIOGE	1.058737	0.413	0.77366
GOBP_MORPHOGENESIS_OF_EMBRYONIC_EPITHELIUM	1.058548	0.37	0.77385

GOBP_ADENYLATE_CYCLASE_INHIBITING_G_PROTEIN_CC	1.058149	0.38	0.77482
GOBP_TYPE_B_PANCREATIC_CELL_PROLIFERATION	1.057719	0.402	0.77589
GOBP_COPPER_ION_TRANSPORT	1.057715	0.409	0.77545
GOBP_RESPIRATORY_SYSTEM_DEVELOPMENT	1.057508	0.365	0.77571
GOBP_NEGATIVE_REGULATION_OF_BINDING	1.057241	0.366	0.7762
GOBP_REGULATION_OF_CYTOSKELETON_ORGANIZATION	1.057057	0.308	0.77641
GOBP_MEIOTIC_CHROMOSOME_SEPARATION	1.057023	0.419	0.77607
GOBP_VENTRICULAR_CARDIAC_MUSCLE_TISSUE_MORPH	1.05651	0.404	0.77742
GOBP_MYD88_DEPENDENT_TOLL_LIKE_RECEPTOR_SIGNA	1.056374	0.415	0.77742
GOBP_PALLIUM_DEVELOPMENT	1.056331	0.351	0.77711
GOBP_GLAND_MORPHOGENESIS	1.056165	0.382	0.77722
GOBP_HISTONE_H3_K9_METHYLATION	1.055753	0.399	0.77821
GOBP_GASTRULATION	1.055423	0.374	0.77899
GOBP_ICOSANOID_METABOLIC_PROCESS	1.055217	0.379	0.77926
GOBP_POSITIVE_REGULATION_OF_NEURON_DEATH	1.055144	0.387	0.77908
GOBP_REGULATION_OF_VIRAL_GENOME_REPLICATION	1.054836	0.395	0.77972
GOBP_POSITIVE_REGULATION_OF_NUCLEOCYTOPLASMIC	1.05482	0.394	0.77932
GOBP_PEPTIDYL_LYSINE_METHYLATION	1.054788	0.382	0.77899
GOBP_KIDNEY_EPITHELIUM_DEVELOPMENT	1.054689	0.397	0.77889
GOBP_GPI_ANCHOR_METABOLIC_PROCESS	1.054569	0.407	0.77886
GOBP_BICARBONATE_TRANSPORT	1.05398	0.415	0.78048
GOBP_CERAMIDE_CATABOLIC_PROCESS	1.05397	0.42	0.78006
GOBP_JNK_CASCADE	1.053677	0.374	0.78068
GOBP_DOUBLE_STRAND_BREAK_REPAIR	1.053626	0.355	0.78041
GOBP_EPITHELIAL_TUBE_FORMATION	1.053588	0.368	0.78008
GOBP_HOMEOSTASIS_OF_NUMBER_OF_CELLS	1.053501	0.353	0.77995
GOBP_REGULATION_OF_UBIQUITIN_DEPENDENT_PROTEI	1.05302	0.37	0.7812
GOBP_POSITIVE_REGULATION_OF_LAMELLIPODIUM_ASSE	1.053005	0.41	0.78081
GOBP_INTERLEUKIN_1_MEDIATED_SIGNALING_PATHWAY	1.052938	0.412	0.78057
GOBP_CARDIAC_EPITHELIAL_TO_MESENCHYMAL_TRANSI	1.052852	0.422	0.78044
GOBP_EYE_MORPHOGENESIS	1.05258	0.374	0.78088
GOBP_MULTICELLULAR_ORGANISMAL_MOVEMENT	1.052323	0.396	0.78135
GOBP_POSITIVE_REGULATION_OF_DNA_METABOLIC_PRO	1.051727	0.378	0.78299
GOBP_REGULATION_OF_GLUCOSE_TRANSMEMBRANE_TR	1.051641	0.403	0.78284
GOBP_NEGATIVE_REGULATION_OF_EPITHELIAL_CELL_PR	1.051559	0.382	0.78269
GOBP_FATTY_ACID_TRANSPORT	1.051472	0.399	0.78253
GOBP_CYTOKINESIS	1.051466	0.392	0.78211
GOBP_PANCREAS_DEVELOPMENT	1.051453	0.397	0.7817
GOBP_REGULATION_OF_MITOCHONDRIAL_GENE_EXPRES	1.051251	0.415	0.78196
GOBP_INTERLEUKIN_6_PRODUCTION	1.051228	0.4	0.78157
GOBP_PROTEIN_O_LINKED_MANNOSYLATION	1.050931	0.401	0.78217
GOBP_INORGANIC_ANION_TRANSPORT	1.050858	0.38	0.782
GOBP_RECEPTOR_MEDIATED_ENDOCYTOSIS	1.050626	0.379	0.78233
GOBP_REGULATION_OF_INTRACELLULAR_TRANSPORT	1.050451	0.352	0.78248
GOBP_NEGATIVE_REGULATION_OF_DNA_BINDING_TRANS	1.050176	0.387	0.78298
GOBP_MRNA_SPLICE_SITE_SELECTION	1.04988	0.403	0.78358
GOBP_AMINO_ACID_TRANSPORT	1.049697	0.394	0.78376
GOBP_CHOLESTEROL_STORAGE	1.04965	0.428	0.78346
GOBP_POSITIVE_REGULATION_OF_CELLULAR_AMIDE_ME	1.04951	0.396	0.78348
GOBP_NEUROMUSCULAR_PROCESS	1.04937	0.395	0.78352
GOBP_VASOCONSTRICTION	1.049124	0.414	0.78393
GOBP_MITOTIC_NUCLEAR_DIVISION	1.049037	0.358	0.78378
GOBP_ESTABLISHMENT_OF_CELL_POLARITY	1.049033	0.393	0.78335

GOBP_POSTTRANSCRIPTIONAL_REGULATION_OF_GENE_	1.048701	0.312	0.78403
GOBP_NEUROPEPTIDE_SIGNALING_PATHWAY	1.048092	0.421	0.78568
GOBP_RESPONSE_TO_KETONE	1.047847	0.377	0.78608
GOBP_MODIFIED_AMINO_ACID_TRANSPORT	1.047539	0.423	0.7867
GOBP_POSITIVE_REGULATION_OF_TRANSMEMBRANE_RE	1.047415	0.394	0.78668
GOBP_REACTIVE_NITROGEN_SPECIES_METABOLIC_PROC	1.047008	0.411	0.78764
GOBP_MATERNAL_PROCESS_INVOLVED_IN_FEMALE_PRE	1.046914	0.41	0.78753
GOBP_INTRINSIC_APOPTOTIC_SIGNALING_PATHWAY_IN_	1.046889	0.409	0.78716
GOBP_CATECHOLAMINE_SECRETION	1.046783	0.411	0.7871
GOBP_DIACYLGLYCEROL_METABOLIC_PROCESS	1.046558	0.411	0.78739
GOBP_REGULATION_OF_ANATOMICAL_STRUCTURE_SIZE	1.046536	0.331	0.78702
GOBP_CELLULAR_RESPONSE_TO_ATP	1.045908	0.409	0.78876
GOBP_ENDOSOME_TO_LYSOSOME_TRANSPORT	1.045731	0.421	0.78893
GOBP_POSITIVE_REGULATION_OF_BLOOD_PRESSURE	1.045515	0.417	0.78926
GOBP_PROTEIN_LOCALIZATION_TO_CHROMOSOME_TELO	1.045462	0.432	0.78898
GOBP_HEART_MORPHOGENESIS	1.045448	0.367	0.78859
GOBP_LENS_MORPHOGENESIS_IN_CAMERA_TYPE_EYE	1.045277	0.433	0.78873
GOBP_CARBOHYDRATE_TRANSMEMBRANE_TRANSPORT	1.045261	0.407	0.78834
GOBP_NUCLEAR_TRANSCRIBED_MRNA_CATABOLIC_PROC	1.045047	0.41	0.78865
GOBP_CYTOSOLIC_CALCIUM_ION_TRANSPORT	1.044652	0.388	0.78958
GOBP_REGULATION_OF_RYANODINE_SENSITIVE_CALCIUI	1.0446	0.429	0.78931
GOBP_REGULATION_OF_SYNAPTIC_PLASTICITY	1.044121	0.392	0.79047
GOBP_T_CELL_APOPTOTIC_PROCESS	1.043673	0.422	0.79157
GOBP_ORGANELLE_TRANSPORT_ALONG_MICROTUBULE	1.043435	0.406	0.79197
GOBP_POSITIVE_REGULATION_OF_PROTEIN_CONTAINING	1.043315	0.399	0.79193
GOBP_RESPONSE_TO_HYDROGEN_PEROXIDE	1.043222	0.432	0.7918
GOBP_POSITIVE_REGULATION_OF_G1_S_TRANSITION_OF	1.043001	0.42	0.79212
GOBP_INTERLEUKIN_6_MEDIATED_SIGNALING_PATHWAY	1.042829	0.433	0.79228
GOBP_REGULATION_OF_FATTY_ACID_BETA_OXIDATION	1.042829	0.432	0.79184
GOBP_ARTERY_DEVELOPMENT	1.042762	0.413	0.79164
GOBP_VASCULAR_ASSOCIATED_SMOOTH_MUSCLE_CELL_	1.042553	0.409	0.79189
GOBP_POSITIVE_REGULATION_OF_DNA_BINDING_TRANS	1.042512	0.38	0.79159
GOBP_NEGATIVE_REGULATION_OF_MAPK_CASCADE	1.042137	0.407	0.79248
GOBP_VASCULAR_ASSOCIATED_SMOOTH_MUSCLE_CONT	1.041874	0.423	0.79295
GOBP_RESPONSE_TO_OXIDATIVE_STRESS	1.041372	0.364	0.79422
GOBP_REGULATION_OF_JNK_CASCADE	1.041014	0.41	0.79501
GOBP_PEPTIDE_TRANSPORT	1.040934	0.39	0.79483
GOBP_OLFACTORY_LOBE_DEVELOPMENT	1.040612	0.436	0.79551
GOBP_REGULATION_OF_CELL_CYCLE_G2_M_PHASE_TRA	1.040364	0.413	0.79593
GOBP_REGULATION_OF_CARDIAC_MUSCLE_CELL_PROLIF	1.040348	0.412	0.79554
GOBP_SIGNAL_RELEASE	1.040148	0.36	0.79576
GOBP_ACTIVATION_OF_GTPASE_ACTIVITY	1.039895	0.412	0.79617
GOBP_NEGATIVE_REGULATION_OF_CELLULAR_MACROMO	1.03954	0.406	0.79695
GOBP_CELLULAR_RESPONSE_TO_NUTRIENT	1.039511	0.423	0.79659
GOBP_ESTABLISHMENT_OF_PROTEIN_LOCALIZATION_TO_	1.039493	0.435	0.79621
GOBP_CAMERA_TYPE_EYE_MORPHOGENESIS	1.039382	0.416	0.79617
GOBP_POSITIVE_REGULATION_OF_MEIOTIC_CELL_CYCLE	1.038963	0.418	0.79714
GOBP_LEUKOCYTE_MEDIATED_IMMUNITY	1.038797	0.397	0.79729
GOBP_REGULATION_OF_MEMBRANE_INVAGINATION	1.038669	0.43	0.79729
GOBP_PROTEIN_LOCALIZATION_TO_CHROMOSOME	1.038268	0.422	0.7982
GOBP_RESPONSE_TO_IRON_ION	1.037689	0.407	0.79973
GOBP_HIPPO_SIGNALING	1.03744	0.424	0.80015
GOBP_CYTOSKELETON_DEPENDENT_INTRACELLULAR_TF	1.037334	0.418	0.80005

GOBP_CELL_CYCLE_PHASE_TRANSITION	1.037087	0.374	0.80047
GOBP_NEURAL_CREST_CELL_MIGRATION	1.036924	0.412	0.80056
GOBP_POSITIVE_REGULATION_OF_SUBSTRATE_ADHESIO	1.036731	0.425	0.80079
GOBP_POSITIVE_REGULATION_OF_CALCIUM_ION_TRANSI	1.036332	0.435	0.80168
GOBP_CELLULAR_IRON_ION_HOMEOSTASIS	1.036207	0.406	0.80166
GOBP_REGULATION_OF_DNA_BINDING_TRANSCRIPTION_	1.03591	0.375	0.80221
GOBP_T_CELL_DIFFERENTIATION_INVOLVED_IN_IMMUNE_	1.035865	0.449	0.80194
GOBP_POSITIVE_REGULATION_OF_T_HELPER_CELL_DIFF	1.035849	0.433	0.80156
GOBP_REGULATION_OF_MONOCYTE_DIFFERENTIATION	1.035729	0.458	0.8015
GOBP_REGULATION_OF_ACUTE_INFLAMMATORY_RESPON	1.035718	0.43	0.8011
GOBP_POSITIVE_REGULATION_OF_DENDRITIC_SPINE_DE	1.035223	0.431	0.80236
GOBP_REGULATION_OF_MAP_KINASE_ACTIVITY	1.035206	0.409	0.80197
GOBP_POSITIVE_REGULATION_OF_G_PROTEIN_COUPLED	1.03481	0.448	0.80286
GOBP_NEGATIVE_REGULATION_OF_DNA_REPLICATION	1.034551	0.434	0.80328
GOBP_POSITIVE_REGULATION_OF_DOUBLE_STRAND_BRE	1.034464	0.452	0.80315
GOBP_POSITIVE_REGULATION_OF_HISTONE_METHYLATIO	1.03429	0.443	0.80329
GOBP_PLACENTA_DEVELOPMENT	1.034239	0.439	0.80302
GOBP_POSITIVE_REGULATION_OF_AMINE_TRANSPORT	1.034084	0.416	0.8031
GOBP_TOR_SIGNALING	1.0339	0.415	0.80325
GOBP_PROTEIN_LIPID_COMPLEX_SUBUNIT_ORGANIZATIO	1.03388	0.433	0.80288
GOBP_REGULATION_OF_CELL_CYCLE_PHASE_TRANSITIO	1.033796	0.389	0.80273
GOBP_RIG_I_SIGNALING_PATHWAY	1.033496	0.449	0.80334
GOBP_I_KAPPAB_PHOSPHORYLATION	1.033463	0.447	0.80301
GOBP_INTRINSIC_APOPTOTIC_SIGNALING_PATHWAY	1.032997	0.419	0.80416
GOBP_REGULATION_OF_INTRACELLULAR_PROTEIN_TRAN	1.032976	0.399	0.80379
GOBP_PROTEIN_MODIFICATION_BY_SMALL_PROTEIN_REI	1.032775	0.428	0.80402
GOBP_PROTEIN_LOCALIZATION_TO_EXTRACELLULAR_RE	1.032591	0.402	0.8042
GOBP_REGULATION_OF_CARDIAC_MUSCLE_CONTRACTIO	1.032026	0.45	0.80565
GOBP_FOREBRAIN_NEURON_DIFFERENTIATION	1.032023	0.431	0.80523
GOBP_POSITIVE_REGULATION_OF_SECRETION	1.031947	0.404	0.80505
GOBP_PEPTIDYL_THREONINE_MODIFICATION	1.031878	0.431	0.80483
GOBP_ENDOTHELIUM_DEVELOPMENT	1.031756	0.436	0.8048
GOBP_POSITIVE_REGULATION_OF_CYTOKINE_PRODUCTI	1.031382	0.432	0.8056
GOBP_EXOCRINE_SYSTEM_DEVELOPMENT	1.030803	0.448	0.80708
GOBP_POSITIVE_REGULATION_OF_PROTEIN_BINDING	1.030464	0.438	0.80777
GOBP_LIPID_PHOSPHORYLATION	1.030453	0.448	0.80737
GOBP_REGULATION_OF_MYELOID_LEUKOCYTE_MEDIATE	1.030149	0.429	0.80795
GOBP_SYNAPTIC_VESICLE_CYTOSKELETAL_TRANSPORT	1.030144	0.461	0.80752
GOBP_INTRINSIC_APOPTOTIC_SIGNALING_PATHWAY_BY_	1.029739	0.445	0.80845
GOBP_REGULATION_OF_LYASE_ACTIVITY	1.029707	0.446	0.80814
GOBP_NEGATIVE_REGULATION_OF_ESTABLISHMENT_OF_	1.029654	0.433	0.80788
GOBP_VIRAL_RNA_GENOME_REPLICATION	1.029647	0.445	0.80747
GOBP_NEGATIVE_REGULATION_OF_T_CELL_MEDIATED_I	1.029474	0.431	0.80761
GOBP_LIGAND_GATED_ION_CHANNEL_SIGNALING_PATHW	1.029366	0.441	0.80755
GOBP_POSITIVE_REGULATION_OF_DNA_TEMPLATED_TRA	1.028849	0.446	0.8089
GOBP_APOPTOTIC_MITOCHONDRIAL_CHANGES	1.028682	0.447	0.80905
GOBP_RESPONSE_TO_ETHANOL	1.028575	0.448	0.80897
GOBP_CYTOPLASMIC_MICROTUBULE_ORGANIZATION	1.028515	0.44	0.80872
GOBP_ATTACHMENT_OF_SPINDLE_MICROTUBULES_TO_K	1.028053	0.455	0.80985
GOBP_EMBRYONIC_CRANIAL_SKELETON_MORPHOGENES	1.027754	0.451	0.81042
GOBP_POSITIVE_REGULATION_OF_PRODUCTION_OF_MO	1.027715	0.446	0.81012
GOBP_DEVELOPMENT_OF_PRIMARY_SEXUAL_CHARACTE	1.027546	0.434	0.81026
GOBP_NEGATIVE_REGULATION_OF_POSTTRANSCRIPTION	1.02723	0.481	0.81085

GOBP_RENAL_SYSTEM_PROCESS	1.027131	0.423	0.81075
GOBP_GTP_METABOLIC_PROCESS	1.026511	0.456	0.81241
GOBP_REGULATION_OF_ACTIVIN_RECEPTOR_SIGNALING	1.026436	0.465	0.81222
GOBP_ORGANIC_HYDROXY_COMPOUND_TRANSPORT	1.025885	0.438	0.81364
GOBP_INTERFERON_GAMMA_MEDIATED_SIGNALING_PAT	1.02582	0.446	0.8134
GOBP_POSITIVE_REGULATION_OF_INTERLEUKIN_6_PROD	1.02568	0.437	0.81344
GOBP_REGULATION_OF_ORGAN_GROWTH	1.025664	0.444	0.81307
GOBP_CALCIUM_MEDIATED_SIGNALING	1.024736	0.443	0.81578
GOBP_PROTEIN_PEPTIDYL_PROLYL_ISOMERIZATION	1.023825	0.471	0.81841
GOBP_POSITIVE_REGULATION_OF_CELL_CYCLE_PROCES	1.023755	0.458	0.81821
GOBP_POSITIVE_REGULATION_OF_NEUROTRANSMITTER_	1.023552	0.466	0.81849
GOBP_REGULATION_OF_OSTEOBLAST_DIFFERENTIATION	1.022925	0.444	0.82015
GOBP_POSITIVE_REGULATION_OF_CELLULAR_RESPONSE	1.022813	0.445	0.82006
GOBP_HISTONE_H3_K14_ACETYLATION	1.022665	0.46	0.82013
GOBP_REGULATION_OF_WNT_SIGNALING_PATHWAY	1.022563	0.441	0.82005
GOBP_CELLULAR_RESPONSE_TO_CALCIUM_ION	1.02232	0.444	0.82041
GOBP_GRANULOCYTE_ACTIVATION	1.022188	0.451	0.82042
GOBP_LEFT_RIGHT_PATTERN_FORMATION	1.022175	0.458	0.82002
GOBP_REGULATION_OF_MRNA_SPLICING_VIA_SPLICEOS	1.021707	0.457	0.82118
GOBP_HISTONE_H3_K9_MODIFICATION	1.021622	0.47	0.82103
GOBP_NEGATIVE_REGULATION_OF_TOLL_LIKE_RECEPTO	1.02154	0.452	0.82086
GOBP_POSITIVE_REGULATION_OF_PEPTIDASE_ACTIVITY	1.021089	0.459	0.82188
GOBP_NEGATIVE_REGULATION_OF_VIRAL_PROCESS	1.020984	0.473	0.8218
GOBP_REGULATION_OF_DEFENSE_RESPONSE_TO_VIRUS	1.020819	0.443	0.82193
GOBP_REGULATION_OF_CELL_CYCLE_G1_S_PHASE_TRA	1.020816	0.465	0.8215
GOBP_INTESTINAL_EPITHELIAL_CELL_DIFFERENTIATION	1.020741	0.473	0.82131
GOBP_REGULATION_OF_SYSTEMIC_ARTERIAL_BLOOD_P	1.020545	0.452	0.82154
GOBP_MAINTENANCE_OF_LOCATION_IN_CELL	1.020306	0.438	0.82187
GOBP_NEGATIVE_REGULATION_OF_PHOSPHORYLATION	1.020267	0.454	0.82156
GOBP_MULTICELLULAR_ORGANISM_AGING	1.020085	0.455	0.82174
GOBP_REGULATION_OF_HEART_RATE	1.020061	0.452	0.82138
GOBP_MALE_MEIOTIC_NUCLEAR_DIVISION	1.019891	0.449	0.82151
GOBP_POSITIVE_REGULATION_OF_NITRIC_OXIDE_METAB	1.019817	0.471	0.82132
GOBP_REGULATION_OF_POSTSYNAPTIC_MEMBRANE_NE	1.019646	0.452	0.82148
GOBP_NEGATIVE_REGULATION_OF_AXON_EXTENSION	1.019558	0.462	0.82132
GOBP_MESENCHYMAL_CELL_PROLIFERATION	1.019106	0.457	0.82237
GOBP_GLOMERULAR_EPITHELIUM_DEVELOPMENT	1.01876	0.468	0.8231
GOBP_REGULATION_OF_CYTOSOLIC_CALCIUM_ION_CON	1.018628	0.46	0.82308
GOBP_NEGATIVE_REGULATION_OF_OSTEOBLAST_DIFFE	1.018604	0.466	0.82272
GOBP_OLIGOSACCHARIDE_METABOLIC_PROCESS	1.018401	0.458	0.82296
GOBP_RNA_DESTABILIZATION	1.018194	0.468	0.82319
GOBP_PROTEIN_ACETYLATION	1.018113	0.456	0.82301
GOBP_NEGATIVE_REGULATION_OF_FIBROBLAST_PROLIF	1.018107	0.442	0.8226
GOBP_CHROMOSOME_SEPARATION	1.018042	0.464	0.82238
GOBP_REGULATION_OF_MRNA_POLYADENYLATION	1.017934	0.458	0.82231
GOBP_DIENCEPHALON_DEVELOPMENT	1.017889	0.466	0.82202
GOBP_REGULATION_OF_PROTEIN_SECRETION	1.017756	0.476	0.82202
GOBP_BIOLOGICAL_PROCESS_INVOLVED_IN_SYMBIOTIC_	1.017486	0.461	0.82248
GOBP_REGULATION_OF_DNA_BIOSYNTHETIC_PROCESS	1.017359	0.471	0.82245
GOBP_POSITIVE_REGULATION_OF_CELL_CYCLE	1.017225	0.45	0.82247
GOBP_REGULATION_OF_PROTEIN_SERINE_THREONINE_K	1.017093	0.441	0.82243
GOBP_REGULATION_OF_PROTEIN_LOCALIZATION_TO_ME	1.017059	0.457	0.8221
GOBP_REGULATION_OF_CELLULAR_AMIDE_METABOLIC_F	1.016824	0.439	0.8224

GOBP_RNA_SPLICING_VIA_TRANSESTERIFICATION_REAC	1.016519	0.439	0.82301
GOBP_VENTRICULAR_CARDIAC_MUSCLE_TISSUE_DEVEL(	1.016505	0.474	0.82261
GOBP_DNA_DEPENDENT_DNA_REPLICATION	1.016424	0.468	0.82245
GOBP_RIBONUCLEOPROTEIN_COMPLEX_SUBUNIT_ORGAI	1.016188	0.465	0.82284
GOBP_HISTONE_H3_ACETYLATION	1.015533	0.472	0.82454
GOBP_LEUKOCYTE_HOMEOSTASIS	1.015295	0.472	0.82488
GOBP_AMINO_ACID_ACTIVATION	1.015255	0.459	0.82459
GOBP_NEGATIVE_REGULATION_OF_PLASMA_MEMBRANE_	1.015184	0.481	0.82439
GOBP_NEGATIVE_REGULATION_OF_DNA_REPAIR	1.014682	0.459	0.8256
GOBP_NEGATIVE_REGULATION_OF_CELL_DIVISION	1.014572	0.466	0.82553
GOBP_REGULATION_OF_DENDRITE_DEVELOPMENT	1.014544	0.464	0.82519
GOBP_POSITIVE_REGULATION_OF_ANIMAL_ORGAN_MOR	1.014498	0.468	0.82491
GOBP_POSITIVE_REGULATION_OF_TELOMERASE_ACTIVIT	1.014419	0.442	0.82472
GOBP_REGULATION_OF_MITOTIC_SISTER_CHROMATID_S	1.014304	0.454	0.82467
GOBP_PYRIMIDINE_RIBONUCLEOTIDE_BIOSYNTHETIC_PR	1.014256	0.474	0.82439
GOBP_VASCULAR_ASSOCIATED_SMOOTH_MUSCLE_CELL_	1.014136	0.474	0.82433
GOBP_REGULATION_OF_CYCLIN_DEPENDENT_PROTEIN_I	1.013744	0.464	0.82524
GOBP_TUBULIN_DEACETYLATION	1.013641	0.464	0.82514
GOBP_POSITIVE_REGULATION_OF_KINASE_ACTIVITY	1.013562	0.457	0.82496
GOBP_POSITIVE_REGULATION_OF_TRANSLATION	1.013213	0.474	0.82566
GOBP_POSITIVE_REGULATION_OF_CYCLIN_DEPENDENT_	1.013212	0.465	0.82524
GOBP_FOREBRAIN_NEURON_DEVELOPMENT	1.01317	0.453	0.82495
GOBP_CELL_SURFACE_RECEPTOR_SIGNALING_PATHWAY	1.013046	0.449	0.82492
GOBP_CIRCADIAN_RHYTHM	1.013026	0.474	0.82456
GOBP_POSITIVE_REGULATION_OF_LIPID_CATABOLIC_PR(	1.012959	0.472	0.82436
GOBP_PHAGOSOME_MATURATION	1.012899	0.484	0.82412
GOBP_PHOTORECEPTOR_CELL_MAINTENANCE	1.012528	0.468	0.82491
GOBP_NCRNA_3_END_PROCESSING	1.012524	0.47	0.8245
GOBP_NEGATIVE_REGULATION_OF_HYDROLASE_ACTIVIT	1.012128	0.475	0.82535
GOBP_ERYTHROCYTE_HOMEOSTASIS	1.011675	0.482	0.8264
GOBP_NEGATIVE_REGULATION_OF_INTERLEUKIN_6_PRO	1.011665	0.473	0.82601
GOBP_REGULATION_OF_MICROTUBULE_BASED_MOVEME	1.011663	0.479	0.82558
GOBP_CARDIAC_SEPTUM_MORPHOGENESIS	1.011502	0.463	0.82567
GOBP_HISTONE_H3_K9_ACETYLATION	1.011436	0.46	0.82546
GOBP_NEGATIVE_REGULATION_OF_WNT_SIGNALING_PAT	1.011415	0.494	0.82509
GOBP_ATRIOVENTRICULAR_VALVE_DEVELOPMENT	1.010911	0.468	0.82629
GOBP_MEMBRANE_INVAGINATION	1.01086	0.49	0.82601
GOBP_CELLULAR_RESPONSE_TO_FATTY_ACID	1.010811	0.471	0.82576
GOBP_REGULATION_OF_PROTEIN_BINDING	1.01059	0.475	0.82604
GOBP_REGULATION_OF_SYSTEMIC_ARTERIAL_BLOOD_PI	1.009972	0.47	0.82761
GOBP_REGULATION_OF_CELLULAR_COMPONENT_SIZE	1.009902	0.462	0.82743
GOBP_INTERFERON_GAMMA_PRODUCTION	1.009599	0.478	0.82791
GOBP_POSITIVE_REGULATION_OF_IMMUNOGLOBULIN_PR	1.009386	0.482	0.82815
GOBP_REGULATION_OF_MRNA_3_END_PROCESSING	1.009134	0.481	0.82852
GOBP_REGULATION_OF_HEART_CONTRACTION	1.009119	0.492	0.82815
GOBP_POSITIVE_REGULATION_OF_PROTEIN_LOCALIZATI(	1.008821	0.465	0.82869
GOBP_PROTEIN_COMPLEX_OLIGOMERIZATION	1.008731	0.475	0.82857
GOBP_REGULATION_OF_STEROID_BIOSYNTHETIC_PROCE	1.008631	0.473	0.82846
GOBP_POSITIVE_REGULATION_OF_CYTOKINE_PRODUCTI	1.008598	0.474	0.82813
GOBP_ESTABLISHMENT_OR_MAINTENANCE_OF_CELL_PO	1.00856	0.485	0.82783
GOBP_REGULATION_OF_GLUCAN_BIOSYNTHETIC_PROCE	1.008318	0.475	0.82815
GOBP_POSITIVE_REGULATION_OF_CELL_SUBSTRATE_JU	1.008209	0.469	0.82805
GOBP_CELLULAR_RESPONSE_TO_CHEMICAL_STRESS	1.0079	0.492	0.82861

GOBP_CELL_FATE_SPECIFICATION	1.007616	0.466	0.82914
GOBP_NEGATIVE_REGULATION_OF_PEPTIDYL_SERINE_PI	1.007083	0.479	0.8304
GOBP_FIBROBLAST_APOPTOTIC_PROCESS	1.007048	0.485	0.83009
GOBP_TRANSITION_METAL_ION_TRANSPORT	1.006534	0.492	0.83131
GOBP_NEGATIVE_REGULATION_OF_KINASE_ACTIVITY	1.00624	0.493	0.83182
GOBP_CILIUM_ORGANIZATION	1.006192	0.489	0.83154
GOBP_REGULATION_OF_FATTY_ACID_OXIDATION	1.006187	0.47	0.83114
GOBP_REGULATION_OF_NEUROTRANSMITTER_RECEPTO	1.005675	0.488	0.83235
GOBP_CELL_KILLING	1.005615	0.485	0.83212
GOBP_REPRODUCTIVE_BEHAVIOR	1.005541	0.469	0.83195
GOBP_CALCIUM_ION_IMPORT_INTO_CYTOSOL	1.00549	0.494	0.83166
GOBP_HISTONE_DEUBIQUITINATION	1.004747	0.48	0.83358
GOBP_REGULATION_OF_MEMBRANE_PERMEABILITY	1.00439	0.488	0.83426
GOBP_CHROMOSOME_CONDENSATION	1.003645	0.482	0.83617
GOBP_REGULATION_OF_BLOOD_CIRCULATION	1.00362	0.481	0.83582
GOBP_FILOPODIUM_ASSEMBLY	1.003516	0.492	0.83574
GOBP_NEGATIVE_REGULATION_OF_ADENYLATE_CYCLAS	1.003431	0.486	0.83557
GOBP_NEGATIVE_REGULATION_OF_PROTEIN_SERINE_TH	1.002692	0.497	0.83749
GOBP_REGULATION_OF_PEPTIDE_TRANSPORT	1.002065	0.506	0.83904
GOBP_CARDIAC_CELL_DEVELOPMENT	1.002002	0.485	0.83879
GOBP_PROTEIN_TARGETING_TO_MEMBRANE	1.00194	0.48	0.83858
GOBP_RNA_SPLICING	1.001932	0.513	0.83818
GOBP_REGULATION_OF_RESPONSE_TO_OXIDATIVE_STRI	1.001498	0.49	0.83912
GOBP_ORGANIC_ACID_TRANSPORT	1.001473	0.507	0.83877
GOBP_REGULATION_OF_MITOCHONDRIAL_MEMBRANE_PI	1.001446	0.471	0.83844
GOBP_CYTOSOLIC_TRANSPORT	1.001261	0.489	0.8386
GOBP_VIRAL_LIFE_CYCLE	1.001232	0.512	0.83826
GOBP_MICROTUBULE_BASED_TRANSPORT	1.001079	0.475	0.83832
GOBP_NEGATIVE_REGULATION_OF_FAT_CELL_DIFFEREN	1.00069	0.493	0.83912
GOBP_REGULATION_OF_CYTOPLASMIC_TRANSPORT	1.000627	0.487	0.8389
GOBP_SCHWANN_CELL_DIFFERENTIATION	1.000614	0.503	0.83851
GOBP_REGULATION_OF_VASCULAR_ASSOCIATED_SMOO	1.000513	0.483	0.83841
GOBP_KERATINOCYTE_PROLIFERATION	1.000135	0.499	0.83923
GOBP_POLYSACCHARIDE_BIOSYNTHETIC_PROCESS	1.000059	0.493	0.83906
GOBP_REGULATION_OF_CELL_MORPHOGENESIS	1.000005	0.508	0.83881
GOBP_PHOTORECEPTOR_CELL_DEVELOPMENT	0.99992	0.493	0.83867
GOBP_REGULATION_OF_CELLULAR_RESPONSE_TO_INSU	0.999788	0.497	0.83864
GOBP_POSITIVE_REGULATION_OF_TRANSFERASE_ACTIV	0.999724	0.495	0.83841
GOBP_NEGATIVE_REGULATION_OF_PROTEASOMAL_PRO	0.999658	0.5	0.8382
GOBP_ORGANIC_HYDROXY_COMPOUND_CATABOLIC_PR	0.999549	0.489	0.83809
GOBP_NEGATIVE_REGULATION_OF_METAPHASE_ANAPHA	0.999432	0.487	0.83802
GOBP_REGULATION_OF_MRNA_METABOLIC_PROCESS	0.999367	0.513	0.83781
GOBP_POSITIVE_REGULATION_OF_PROTEIN_LOCALIZATI	0.999257	0.514	0.83772
GOBP_NEGATIVE_REGULATION_OF_STRESS_ACTIVATED_	0.999084	0.496	0.83785
GOBP_IMPORT_INTO_CELL	0.999044	0.505	0.83754
GOBP_INNER_EAR_RECEPTOR_CELL_STEREOCILIUM_OR	0.998984	0.487	0.83732
GOBP_STRIATED_MUSCLE_CELL_DEVELOPMENT	0.998747	0.503	0.83764
GOBP_SPINDLE_LOCALIZATION	0.998628	0.483	0.83757
GOBP_POSITIVE_REGULATION_OF_CELLULAR_COMPONE	0.998029	0.503	0.839
GOBP_PROTEIN_NEDDYLATION	0.997732	0.504	0.83949
GOBP_LAMELLIPODIUM_ORGANIZATION	0.997694	0.497	0.83918
GOBP_CELLULAR_RESPONSE_TO_GONADOTROPIN_STIMI	0.997338	0.493	0.83984
GOBP_L_ALPHA_AMINO_ACID_TRANSMEMBRANE_TRANSI	0.996992	0.514	0.8405

GOBP_REGULATION_OF_T_HELPER_17_CELL_DIFFERENT	0.996845	0.493	0.84056
GOBP_REGULATION_OF_PLATELET_DERIVED_GROWTH_F	0.996348	0.499	0.84169
GOBP_REGULATION_OF_GLIAL_CELL_PROLIFERATION	0.99624	0.49	0.84162
GOBP_RIBOSOME_ASSEMBLY	0.996233	0.485	0.84122
GOBP_POSITIVE_REGULATION_OF_NEURON_DIFFERENTI/	0.996157	0.501	0.84104
GOBP_ENDOTHELIAL_CELL_APOPTOTIC_PROCESS	0.995654	0.496	0.8422
GOBP_DIOL_METABOLIC_PROCESS	0.995636	0.492	0.84184
GOBP_CHROMOSOME_SEGREGATION	0.995357	0.513	0.84227
GOBP_POSITIVE_REGULATION_OF_NUCLEAR_DIVISION	0.995317	0.508	0.84197
GOBP_MAMMARY_GLAND_ALVEOLUS_DEVELOPMENT	0.994878	0.499	0.84291
GOBP_SOMATIC_DIVERSIFICATION_OF_IMMUNE_RECEPT(	0.994113	0.501	0.84485
GOBP_PROTEIN_ACYLATION	0.994045	0.518	0.84465
GOBP_SMOOTH_MUSCLE_TISSUE_DEVELOPMENT	0.9939	0.491	0.84467
GOBP_PROTEASOME_MEDIATED_UBIQUITIN_DEPENDENT	0.993834	0.527	0.84445
GOBP_RESPONSE_TO_MAGNESIUM_ION	0.993781	0.498	0.8442
GOBP_CELL_FATE_COMMITMENT_INVOLVED_IN_FORMATI	0.993722	0.503	0.84397
GOBP_TRANSFORMING_GROWTH_FACTOR_BETA_PRODU	0.993565	0.51	0.84402
GOBP_SYNAPTIC_TRANSMISSION_GABAERGIC	0.993344	0.494	0.84426
GOBP_NEGATIVE_REGULATION_OF_PROTEIN_MODIFICAT	0.993338	0.528	0.84386
GOBP_POSITIVE_REGULATION_OF_GLUCONEOGENESIS	0.992374	0.499	0.84635
GOBP_RESPONSE_TO_AXON_INJURY	0.992111	0.514	0.84673
GOBP_POSITIVE_REGULATION_OF_DNA_RECOMBINATION	0.991548	0.515	0.84803
GOBP_NEGATIVE_REGULATION_OF_ACTIN_FILAMENT_BU	0.991515	0.507	0.84772
GOBP_RESPONSE_TO_TUMOR_CELL	0.991493	0.518	0.84737
GOBP_SEX_DIFFERENTIATION	0.991431	0.531	0.84714
GOBP_T_HELPER_17_CELL_DIFFERENTIATION	0.990732	0.494	0.84888
GOBP_REGULATION_OF_SIGNALING_RECEPTOR_ACTIVIT	0.99072	0.525	0.8485
GOBP_NEGATIVE_REGULATION_OF_TRANSFERASE_ACTI\	0.990544	0.525	0.84864
GOBP_PURINE_CONTAINING_COMPOUND_BIOSYNTHETIC	0.990461	0.523	0.84847
GOBP_DOPAMINE_RECEPTOR_SIGNALING_PATHWAY	0.990346	0.516	0.8484
GOBP_N_TERMINAL_PROTEIN_AMINO_ACID_ACETYLATIOI	0.990325	0.506	0.84804
GOBP_REGULATION_OF_SYSTEMIC_ARTERIAL_BLOOD_PI	0.990112	0.519	0.84829
GOBP_CYTOSKELETON_DEPENDENT_CYTOKINESIS	0.989668	0.498	0.84923
GOBP_MITOCHONDRIAL_DNA_REPLICATION	0.989573	0.517	0.84911
GOBP_REGULATION_OF_ION_TRANSMEMBRANE_TRANSP	0.989319	0.543	0.84949
GOBP_PYRIDINE_CONTAINING_COMPOUND_BIOSYNTHETI	0.989315	0.498	0.84908
GOBP_SPLICEOSOMAL_COMPLEX_ASSEMBLY	0.989161	0.503	0.84912
GOBP_NEGATIVE_REGULATION_OF_ORGAN_GROWTH	0.988528	0.499	0.85064
GOBP_INTERLEUKIN_8_PRODUCTION	0.98852	0.526	0.85024
GOBP_POSITIVE_REGULATION_OF_LIPID_TRANSPORT	0.988446	0.524	0.85006
GOBP_MICROTUBULE_ORGANIZING_CENTER_LOCALIZATI	0.98844	0.499	0.84967
GOBP_POSITIVE_REGULATION_OF_BLOOD_CIRCULATION	0.98834	0.513	0.84958
GOBP_NEGATIVE_REGULATION_OF_HORMONE_SECRETI(	0.988148	0.531	0.84974
GOBP_POSITIVE_REGULATION_OF_TRANSCRIPTION_FRO	0.988055	0.504	0.84961
GOBP_ESTABLISHMENT_OF_PROTEIN_LOCALIZATION_TO	0.987602	0.545	0.8506
GOBP_NEGATIVE_REGULATION_OF_ANION_TRANSPORT	0.987339	0.506	0.85099
GOBP_NEUROMUSCULAR_PROCESS_CONTROLLING_POS	0.987233	0.5	0.8509
GOBP_PHOSPHATIDYLGLYCEROL_METABOLIC_PROCESS	0.987225	0.521	0.85051
GOBP_FAT_CELL_DIFFERENTIATION	0.987135	0.538	0.85037
GOBP_REGULATION_OF_ICOSANOID_SECRETION	0.987072	0.499	0.85015
GOBP_REGULATION_OF_PROTEIN_IMPORT	0.986828	0.533	0.85044
GOBP_CELLULAR_RESPONSE_TO_KETONE	0.986569	0.51	0.85079
GOBP_REGULATION_OF_CELL_PROJECTION_ASSEMBLY	0.986423	0.519	0.85081

GOBP_POSITIVE_REGULATION_OF_CANONICAL_WNT_SIG	0.98636	0.521	0.85062
GOBP_MAINTENANCE_OF_LOCATION	0.986342	0.554	0.85026
GOBP_EMBRYONIC_PLACENTA_MORPHOGENESIS	0.985683	0.498	0.85185
GOBP_CELLULAR_OXIDANT_DETOXIFICATION	0.985386	0.517	0.85229
GOBP_PROTEIN_FOLDING	0.985099	0.555	0.85275
GOBP_REGULATION_OF_EPITHELIAL_CELL_APOPTOTIC_F	0.984996	0.527	0.85265
GOBP_REGULATION_OF_NOTCH_SIGNALING_PATHWAY	0.984984	0.509	0.85227
GOBP_MEIOTIC_CHROMOSOME_SEGREGATION	0.984882	0.516	0.85217
GOBP_RESOLUTION_OF_MEIOTIC_RECOMBINATION_INTE	0.984706	0.523	0.85228
GOBP_DNA_TEMPLATED_TRANSCRIPTION_INITIATION	0.984465	0.519	0.85258
GOBP_NEPHRON_TUBULE_FORMATION	0.984087	0.512	0.85329
GOBP_DNA_DEALKYLATION	0.983726	0.532	0.85397
GOBP_DIVALENT_INORGANIC_CATION_HOMEOSTASIS	0.98371	0.564	0.85361
GOBP_HISTONE_H3_K9_TRIMETHYLATION	0.983688	0.509	0.85326
GOBP_REGULATION_OF_KETONE_BIOSYNTHETIC_PROCE	0.983672	0.504	0.8529
GOBP_STRESS_FIBER_ASSEMBLY	0.982899	0.537	0.85482
GOBP_NEGATIVE_REGULATION_OF_PROTEASOMAL_UBIQ	0.982879	0.501	0.85447
GOBP_IMMUNOGLOBULIN_PRODUCTION_INVOLVED_IN_IM	0.982685	0.518	0.85465
GOBP_CELL_CELL_SIGNALING_BY_WNT	0.98257	0.56	0.8546
GOBP_POSITIVE_REGULATION_OF_MYELOID_LEUKOCYTE	0.982311	0.515	0.85494
GOBP_PROTEIN_CATABOLIC_PROCESS_IN_THE_VACUOLE	0.982125	0.504	0.85507
GOBP_RESPONSE_TO_HYPEROXIA	0.981823	0.505	0.85556
GOBP_POSITIVE_REGULATION_OF_REPRODUCTIVE_PRO(	0.981188	0.543	0.85702
GOBP_NEGATIVE_REGULATION_OF_LYASE_ACTIVITY	0.981125	0.516	0.85679
GOBP_REGULATION_OF_DENDRITE_MORPHOGENESIS	0.981124	0.515	0.85638
GOBP_PROTEIN_KINASE_C_ACTIVATING_G_PROTEIN_COU	0.981048	0.52	0.85618
GOBP_CARBOHYDRATE_DERIVATIVE_TRANSPORT	0.980849	0.526	0.85639
GOBP_RESPONSE_TO_HYDROPEROXIDE	0.980702	0.511	0.85639
GOBP_PHOSPHATE_ION_TRANSMEMBRANE_TRANSPORT	0.980548	0.518	0.85645
GOBP_REGULATION_OF_HIPPO_SIGNALING	0.980003	0.518	0.85767
GOBP_REGULATION_OF_CANONICAL_WNT_SIGNALING_PA	0.979568	0.549	0.85855
GOBP_REGULATION_OF_INFLAMMATORY_RESPONSE	0.97954	0.575	0.85822
GOBP_NEGATIVE_REGULATION_OF_DEFENSE_RESPONSE	0.979535	0.517	0.85782
GOBP_PROSTATE_GLAND_DEVELOPMENT	0.979501	0.523	0.85751
GOBP_CENTROMERE_COMPLEX_ASSEMBLY	0.979498	0.531	0.85711
GOBP_CELLULAR_TRANSITION_METAL_ION_HOMEOSTASI	0.979492	0.539	0.85672
GOBP_CHEMOKINE_C_X_C_MOTIF_LIGAND_2_PRODUCTIC	0.979465	0.519	0.85639
GOBP_REGULATION_OF_POLYSACCHARIDE_METABOLIC_	0.979311	0.525	0.85642
GOBP_POSITIVE_REGULATION_OF_LEUKOCYTE_DEGRAN	0.979214	0.501	0.85628
GOBP_NEGATIVE_REGULATION_OF_LIPID_BIOSYNTHETIC	0.979046	0.514	0.85637
GOBP_POSITIVE_REGULATION_OF_AXONOGENESIS	0.978909	0.514	0.85635
GOBP_MYELOID_DENDRITIC_CELL_DIFFERENTIATION	0.978287	0.519	0.85777
GOBP_REGULATION_OF_ENDOTHELIAL_CELL_CHEMOTAX	0.978016	0.506	0.85818
GOBP_REGULATION_OF_MITOCHONDRIAL_MEMBRANE_PC	0.978012	0.526	0.85778
GOBP_REGULATION_OF_LIPID_STORAGE	0.9777	0.515	0.85828
GOBP_CELLULAR_RESPONSE_TO_ORGANIC_CYCLIC_COM	0.977589	0.577	0.85821
GOBP_REGULATION_OF_HEART_RATE_BY_CARDIAC_CON	0.977219	0.511	0.85887
GOBP_LACTATION	0.976968	0.519	0.85924
GOBP_LIPID_TRANSLOCATION	0.976908	0.537	0.85902
GOBP_SPINDLE_ORGANIZATION	0.976903	0.557	0.85863
GOBP_DEVELOPMENTAL_PIGMENTATION	0.976845	0.533	0.85839
GOBP_NCRNA_CATABOLIC_PROCESS	0.976669	0.532	0.85851
GOBP_OUTFLOW_TRACT_SEPTUM_MORPHOGENESIS	0.976554	0.527	0.85846

GOBP_ADHERENS_JUNCTION_ORGANIZATION	0.976542	0.536	0.8581
GOBP_REGULATION_OF_MORPHOGENESIS_OF_A_BRANC	0.976401	0.517	0.8581
GOBP_LYSOSOMAL_TRANSPORT	0.976239	0.549	0.85816
GOBP_NEGATIVE_REGULATION_OF_RELEASE_OF_CYTOC	0.976139	0.523	0.85804
GOBP_ENDOCRINE_PANCREAS_DEVELOPMENT	0.976032	0.519	0.85794
GOBP_POSITIVE_REGULATION_OF_ISOTYPE_SWITCHING	0.975997	0.524	0.85765
GOBP_INSULIN_SECRETION	0.975946	0.554	0.85739
GOBP_APICAL_PROTEIN_LOCALIZATION	0.97591	0.543	0.85708
GOBP_LIPID_CATABOLIC_PROCESS	0.975751	0.587	0.85716
GOBP_MORPHOGENESIS_OF_A_BRANCHING_STRUCTURE	0.975361	0.569	0.85788
GOBP_MRNA_PROCESSING	0.975258	0.591	0.85776
GOBP_ACUTE_INFLAMMATORY_RESPONSE_TO_ANTIGENI	0.975175	0.537	0.8576
GOBP_ACTIVATION_OF_IMMUNE_RESPONSE	0.97512	0.563	0.85734
GOBP_DETERMINATION_OF_ADULT_LIFESPAN	0.974836	0.524	0.85782
GOBP_REGULATION_OF_TRANSMEMBRANE_TRANSPORT	0.974698	0.614	0.85781
GOBP_CELLULAR_CARBOHYDRATE_BIOSYNTHETIC_PROC	0.974659	0.527	0.85753
GOBP_POSITIVE_REGULATION_OF_MULTICELLULAR_ORG	0.974646	0.543	0.85717
GOBP_NEGATIVE_CHEMOTAXIS	0.974635	0.525	0.85679
GOBP_NEGATIVE_REGULATION_OF_PROTEIN_BINDING	0.974549	0.544	0.85664
GOBP_NEURON_RECOGNITION	0.973883	0.533	0.85817
GOBP_PROTEIN_KINASE_C_SIGNALING	0.973251	0.529	0.85959
GOBP_POSITIVE_REGULATION_OF_MAST_CELL_ACTIVATI	0.97312	0.537	0.85957
GOBP_REGULATION_OF_CALCIUM_ION_TRANSPORT	0.972956	0.601	0.85966
GOBP_NEGATIVE_REGULATION_OF_SMOOTHENED_SIGNA	0.972927	0.529	0.85934
GOBP_POSITIVE_REGULATION_OF_PROTEIN_KINASE_ACT	0.972901	0.584	0.85902
GOBP_POSITIVE_REGULATION_OF_UBIQUITIN_DEPENDEN	0.972815	0.551	0.85887
GOBP_POSITIVE_REGULATION_OF_PATHWAY_RESTRICTE	0.972601	0.518	0.85911
GOBP_PEPTIDYL_GLUTAMIC_ACID_MODIFICATION	0.972482	0.524	0.85907
GOBP_PYRIMIDINE_NUCLEOTIDE_METABOLIC_PROCESS	0.971798	0.536	0.86066
GOBP_MITOCHONDRIAL_OUTER_MEMBRANE_PERMEABILI	0.971391	0.54	0.86145
GOBP_INORGANIC_ANION_TRANSMEMBRANE_TRANSPOR	0.971302	0.544	0.8613
GOBP_POSITIVE_REGULATION_OF_PROTEOLYSIS	0.971257	0.608	0.86103
GOBP_ORGANISM_EMERGENCE_FROM_PROTECTIVE_STR	0.970961	0.537	0.86147
GOBP_PHARYNGEAL_SYSTEM_DEVELOPMENT	0.970662	0.522	0.86192
GOBP_STRIATED_MUSCLE_CONTRACTION	0.970257	0.581	0.86266
GOBP_FACE_DEVELOPMENT	0.970042	0.516	0.86288
GOBP_RECEPTOR_INTERNALIZATION	0.969694	0.554	0.86347
GOBP_TOXIN_TRANSPORT	0.969615	0.543	0.86331
GOBP_POSITIVE_REGULATION_OF_PROTEIN_SECRETION	0.969614	0.551	0.86291
GOBP_REGULATION_OF_ANOIKIS	0.96949	0.511	0.86287
GOBP_UTERUS_DEVELOPMENT	0.969475	0.552	0.8625
GOBP_TRANSCRIPTION_BY_RNA_POLYMERASE_III	0.968831	0.538	0.86396
GOBP_NEGATIVE_REGULATION_OF_CHEMOKINE_PRODUC	0.968713	0.505	0.8639
GOBP_LIPOPROTEIN_LOCALIZATION	0.968628	0.534	0.86375
GOBP_RESPONSE_TO_PAIN	0.968299	0.521	0.86431
GOBP_PROTEIN_CONTAINING_COMPLEX_REMODELING	0.968083	0.535	0.86455
GOBP_MUSCLE_CELL_DEVELOPMENT	0.967848	0.57	0.8648
GOBP_PHOSPHATIDYLINOSITOL_METABOLIC_PROCESS	0.967555	0.575	0.86525
GOBP_CELLULAR_RESPONSE_TO_GLUCOSE_STARVATION	0.967538	0.546	0.86489
GOBP_PIGMENT_CELL_DIFFERENTIATION	0.967428	0.546	0.86479
GOBP_DNA_RECOMBINATION	0.967255	0.574	0.86488
GOBP_REGULATION_OF_CALCIUM_ION_TRANSMEMBRANE	0.967029	0.572	0.86514
GOBP_REGULATION_OF_FATTY_ACID_TRANSPORT	0.96701	0.528	0.86478

GOBP_ADAPTATION_OF_SIGNALING_PATHWAY	0.966993	0.548	0.86443
GOBP_SISTER_CHROMATID_SEGREGATION	0.966387	0.583	0.86574
GOBP_NUCLEOSIDE_TRIPHOSPHATE_BIOSYNTHETIC_PRO	0.966047	0.56	0.86635
GOBP_MYELOID_CELL_DEVELOPMENT	0.965721	0.544	0.86686
GOBP_NEGATIVE_REGULATION_OF_CELLULAR_RESPONS	0.965558	0.54	0.86691
GOBP_GLUCAN_BIOSYNTHETIC_PROCESS	0.964992	0.556	0.86809
GOBP_INNER_EAR_MORPHOGENESIS	0.964887	0.554	0.86801
GOBP_TRANSCRIPTION_BY_RNA_POLYMERASE_I	0.964725	0.548	0.86809
GOBP_POSITIVE_REGULATION_OF_PROTEIN_EXPORT_FR	0.964197	0.553	0.86917
GOBP_VIRAL_PROCESS	0.963786	0.62	0.86995
GOBP_ENDOCARDIAL_CUSHION_DEVELOPMENT	0.963747	0.547	0.86966
GOBP_POSITIVE_REGULATION_OF_DEFENSE_RESPONSE	0.963442	0.6	0.87016
GOBP_INTEGRIN_MEDIATED_SIGNALING_PATHWAY	0.963248	0.565	0.8703
GOBP_REGULATION_OF_T_CELL_APOPTOTIC_PROCESS	0.963164	0.554	0.87014
GOBP_NEGATIVE_REGULATION_OF_PHAGOCYTOSIS	0.96305	0.525	0.87008
GOBP_CELLULAR_RESPONSE_TO_EXTRACELLULAR_STIM	0.963033	0.606	0.86973
GOBP_POSITIVE_REGULATION_OF_DNA_TEMPLATED_TRA	0.962512	0.554	0.87079
GOBP_POSITIVE_REGULATION_OF_T_CELL_MEDIATED_IM	0.962446	0.547	0.87056
GOBP_ANION_TRANSMEMBRANE_TRANSPORT	0.962143	0.598	0.87099
GOBP_CARDIOLIPIN_METABOLIC_PROCESS	0.962081	0.554	0.87078
GOBP_I_KAPPAB_KINASE_NF_KAPPAB_SIGNALING	0.961959	0.612	0.87072
GOBP_NEGATIVE_REGULATION_OF_EXTRINSIC_APOPTOT	0.961869	0.541	0.87057
GOBP_LONG_CHAIN_FATTY_ACID_TRANSPORT	0.961679	0.549	0.87069
GOBP_DOPAMINE_SECRETION	0.961405	0.564	0.87106
GOBP_RAC_PROTEIN_SIGNAL_TRANSDUCTION	0.961277	0.551	0.87103
GOBP_MEMBRANE_BIOGENESIS	0.96087	0.569	0.8718
GOBP_REGULATION_OF_ORGANELLE_ASSEMBLY	0.960818	0.621	0.87154
GOBP_FEAR_RESPONSE	0.96063	0.546	0.87167
GOBP_SEMAPHORIN_PLEXIN_SIGNALING_PATHWAY	0.960588	0.548	0.87139
GOBP_REGULATION_OF_DEFENSE_RESPONSE	0.960099	0.655	0.87232
GOBP_HORMONE_BIOSYNTHETIC_PROCESS	0.959612	0.544	0.87322
GOBP_NUCLEAR_CHROMOSOME_SEGREGATION	0.959583	0.606	0.87292
GOBP_RESPONSE_TO_AUDITORY_STIMULUS	0.959515	0.54	0.87271
GOBP_POSITIVE_REGULATION_OF_AMINO_ACID_TRANSP	0.959312	0.543	0.8729
GOBP_REGULATION_OF_AUTOPHAGY	0.959192	0.617	0.87284
GOBP_RESPONSE_TO_ATP	0.959047	0.545	0.87284
GOBP_VENTRICULAR_SEPTUM_MORPHOGENESIS	0.959003	0.566	0.87257
GOBP_NEGATIVE_REGULATION_OF_MACROAUTOPHAGY	0.958793	0.544	0.87273
GOBP_PROTEIN_DEPHOSPHORYLATION	0.958518	0.616	0.87315
GOBP_REGULATION_OF_PROTEASOMAL_PROTEIN_CATAB	0.958453	0.6	0.87292
GOBP_ORGANIC_HYDROXY_COMPOUND_METABOLIC_PRO	0.958364	0.656	0.87278
GOBP_ESTABLISHMENT_OF_MITOTIC_SPINDLE_LOCALIZA	0.958172	0.546	0.87292
GOBP_AMINE_TRANSPORT	0.9578	0.563	0.87356
GOBP_MITOTIC_SISTER_CHROMATID_SEGREGATION	0.957783	0.596	0.87321
GOBP_NEGATIVE_REGULATION_OF_PROTEIN_MODIFICAT	0.957307	0.595	0.87413
GOBP_ANTIGEN_PROCESSING_AND_PRESENTATION_OF_	0.957139	0.55	0.8742
GOBP_REGULATION_OF_CYCLASE_ACTIVITY	0.957084	0.572	0.87396
GOBP_NEGATIVE_REGULATION_OF_CELL_JUNCTION_ASS	0.956915	0.54	0.87405
GOBP_REGULATION_OF_TRANSPOSITION	0.956707	0.526	0.87427
GOBP_MICROVILLUS_ASSEMBLY	0.956349	0.54	0.87489
GOBP_T_CELL_MEDIATED_IMMUNITY	0.956199	0.586	0.87492
GOBP_MITOCHONDRIAL_OUTER_MEMBRANE_PERMEABILI	0.956162	0.556	0.87463
GOBP_PEPTIDE_CATABOLIC_PROCESS	0.955173	0.548	0.87691

GOBP_POSITIVE_REGULATION_OF_NEUTROPHIL_MIGRAT	0.955065	0.573	0.87681
GOBP_INTERSTRAND_CROSS_LINK_REPAIR	0.954792	0.547	0.87714
GOBP_REGULATION_OF_CELL_KILLING	0.954769	0.558	0.8768
GOBP_PHENOL_CONTAINING_COMPOUND_BIOSYNTHETIC	0.95452	0.558	0.87708
GOBP_CELLULAR_PIGMENTATION	0.954382	0.557	0.87707
GOBP_REGULATION_OF_SYSTEM_PROCESS	0.954146	0.672	0.87732
GOBP_CARDIAC_ATRIUM_DEVELOPMENT	0.953816	0.556	0.87783
GOBP_DNA_DOUBLE_STRAND_BREAK_PROCESSING	0.953634	0.549	0.87795
GOBP_CALCIUM_ION_TRANSMEMBRANE_TRANSPORT	0.953062	0.641	0.87918
GOBP_PROTEIN_POLYUBIQUITINATION	0.953051	0.628	0.87882
GOBP_MESENCHYMAL_CELL_DEVELOPMENT	0.952769	0.572	0.87916
GOBP_TUMOR_NECROSIS_FACTOR_MEDIATED_SIGNALIN	0.952276	0.585	0.88012
GOBP_FC_RECEPTOR_MEDIATED_STIMULATORY_SIGNAL	0.952227	0.552	0.87987
GOBP_POSITIVE_REGULATION_OF_LIPID_LOCALIZATION	0.95179	0.581	0.88068
GOBP_ORGANIC_ACID_TRANSMEMBRANE_TRANSPORT	0.951659	0.592	0.88064
GOBP_CARDIAC_NEURAL_CREST_CELL_DIFFERENTIATIO	0.951448	0.55	0.88081
GOBP_REGULATION_OF_ACTOMYOSIN_STRUCTURE_ORG	0.951311	0.585	0.88079
GOBP_NEGATIVE_REGULATION_OF_NOTCH_SIGNALING_P	0.950604	0.56	0.88234
GOBP_SPHINGOID_METABOLIC_PROCESS	0.950257	0.578	0.88286
GOBP_REGULATION_OF_DNA_TEMPLATED_TRANSCRIPTI	0.94995	0.579	0.88327
GOBP_REGULATION_OF_STEROID_METABOLIC_PROCESS	0.949703	0.585	0.88353
GOBP_SYNAPTIC_MEMBRANE_ADHESION	0.949446	0.567	0.88381
GOBP_SYNAPTIC_TRANSMISSION_DOPAMINERGIC	0.949397	0.568	0.88355
GOBP_BODY_MORPHOGENESIS	0.949375	0.567	0.88323
GOBP_ESTABLISHMENT_OR_MAINTENANCE_OF_MONOPO	0.949158	0.553	0.88344
GOBP_NEGATIVE_REGULATION_OF_SIGNAL_TRANSDUCT	0.948921	0.547	0.88369
GOBP_POSITIVE_REGULATION_OF_STRESS_FIBER_ASSE	0.948789	0.565	0.88365
GOBP_REGULATION_OF_DNA_METHYLATION	0.948684	0.551	0.88355
GOBP_SKELETAL_MUSCLE_ORGAN_DEVELOPMENT	0.9483	0.606	0.8842
GOBP_PROTEIN_SUMOYLATION	0.948069	0.571	0.88445
GOBP_REGULATION_OF_ESTABLISHMENT_OF_PROTEIN_L	0.948004	0.684	0.88425
GOBP_LIPID_OXIDATION	0.947979	0.598	0.88394
GOBP_PROTEIN_MONOUBIQUITINATION	0.947931	0.577	0.88366
GOBP_CYTOKINE_PRODUCTION_INVOLVED_IN_IMMUNE_F	0.947845	0.585	0.88351
GOBP_REGULATION_OF_ARP2_3_COMPLEX_MEDIATED_A	0.947745	0.55	0.88339
GOBP_NEGATIVE_REGULATION_OF_MITOCHONDRION_OR	0.947376	0.579	0.88402
GOBP_BONE_GROWTH	0.946568	0.577	0.88584
GOBP_INNER_EAR_AUDITORY_RECEPTOR_CELL_DIFFERE	0.946539	0.586	0.88552
GOBP_NEGATIVE_REGULATION_OF_OXIDATIVE_STRESS_	0.946017	0.575	0.88656
GOBP_SNRNA_PROCESSING	0.94597	0.554	0.88629
GOBP_SEROTONIN_RECEPTOR_SIGNALING_PATHWAY	0.945921	0.537	0.88603
GOBP_NOREPINEPHRINE_TRANSPORT	0.945843	0.579	0.88586
GOBP_CARDIAC_MUSCLE_CONTRACTION	0.945753	0.604	0.88574
GOBP_HEART_TRABECULA_MORPHOGENESIS	0.945512	0.568	0.88598
GOBP_POSITIVE_REGULATION_OF_MITOTIC_NUCLEAR_DI	0.945508	0.561	0.8856
GOBP_PEPTIDYL_LYSINE_TRIMETHYLATION	0.945368	0.582	0.88558
GOBP_ACTIVIN_RECEPTOR_SIGNALING_PATHWAY	0.945293	0.565	0.88538
GOBP_CAMP_MEDIATED_SIGNALING	0.944046	0.575	0.88831
GOBP_DNA_REPLICATION_INITIATION	0.943933	0.598	0.88822
GOBP_NEGATIVE_REGULATION_OF_PHOSPHORUS_META	0.943873	0.689	0.88799
GOBP_3_UTR_MEDIATED_MRNA_DESTABILIZATION	0.943816	0.571	0.88776
GOBP_POSITIVE_REGULATION_OF_ACTIVATED_T_CELL_P	0.943755	0.538	0.88753
GOBP_POSITIVE_REGULATION_OF_VASCULAR_ASSOCIAT	0.942738	0.567	0.88986

GOBP_POSITIVE_REGULATION_OF_PROTEASOMAL_UBIQU	0.942324	0.606	0.89057
GOBP_STEROID_METABOLIC_PROCESS	0.942102	0.656	0.89075
GOBP_REGULATION_OF_CHROMOSOME_ORGANIZATION	0.941753	0.674	0.89129
GOBP_AMINO_ACID_TRANSMEMBRANE_TRANSPORT	0.941555	0.584	0.89142
GOBP_AMINO_ACID_IMPORT_ACROSS_PLASMA_MEMBRAN	0.941502	0.572	0.89116
GOBP_NEGATIVE_REGULATION_OF_CYSTEINE_TYPE_END	0.941485	0.586	0.89082
GOBP_REGULATION_OF_EPIDERMAL_GROWTH_FACTOR_	0.94106	0.569	0.89157
GOBP_MEIOTIC_CELL_CYCLE	0.94089	0.659	0.89163
GOBP_REGULATION_OF_VASCULAR_ENDOTHELIAL_GROW	0.94087	0.569	0.89129
GOBP_SERINE_FAMILY_AMINO_ACID_METABOLIC_PROCE	0.940789	0.577	0.8911
GOBP_POSITIVE_REGULATION_OF_NEUROTRANSMITTER_	0.940618	0.578	0.89115
GOBP_GRANULOCYTE_DIFFERENTIATION	0.940508	0.588	0.89106
GOBP_REGULATION_OF_NITRIC_OXIDE_SYNTHASE_ACTIV	0.940477	0.559	0.89075
GOBP_OOCYTE_MATURATION	0.940473	0.563	0.89037
GOBP_PYRIMIDINE_CONTAINING_COMPOUND_METABOLIC	0.939757	0.596	0.89183
GOBP_REGULATION_OF_CYSTEINE_TYPE_ENDOPEPTIDAS	0.939404	0.655	0.89237
GOBP_POSITIVE_REGULATION_OF_PROTEIN_ACETYLATIO	0.939062	0.575	0.89287
GOBP_REGULATION_OF_MACROAUTOPHAGY	0.938477	0.645	0.894
GOBP_REGULATION_OF_CIRCADIAN_SLEEP_WAKE_CYCL	0.938183	0.573	0.89437
GOBP_NEGATIVE_REGULATION_OF_PROTEOLYSIS_INVOL	0.938143	0.611	0.89407
GOBP_CALCINEURIN_MEDIATED_SIGNALING	0.938133	0.584	0.89371
GOBP_RESPONSE_TO_PEPTIDE	0.937964	0.73	0.89377
GOBP_PLASMA_LIPOPROTEIN_PARTICLE_CLEARANCE	0.937525	0.595	0.89449
GOBP_NEURON_PROJECTION_REGENERATION	0.937423	0.582	0.89437
GOBP_INTRACELLULAR_RECEPTOR_SIGNALING_PATHWA	0.936771	0.685	0.89568
GOBP_POSITIVE_REGULATION_OF_ERBB_SIGNALING_PAT	0.936528	0.591	0.89593
GOBP_HISTONE_H3_K4_METHYLATION	0.93651	0.61	0.89559
GOBP_POSITIVE_REGULATION_OF_CALCIUM_ION_TRANS	0.936412	0.606	0.89545
GOBP_ENSHEATHMENT_OF_NEURONS	0.936341	0.644	0.89526
GOBP_CELL_CELL_RECOGNITION	0.936286	0.611	0.89501
GOBP_GLYCOPROTEIN_METABOLIC_PROCESS	0.935981	0.696	0.89542
GOBP_SULFUR_COMPOUND_BIOSYNTHETIC_PROCESS	0.935947	0.629	0.89512
GOBP_CELLULAR_RESPONSE_TO_REACTIVE_OXYGEN_SP	0.93569	0.634	0.89538
GOBP_POSITIVE_REGULATION_OF_I_KAPPAB_KINASE_NF	0.935062	0.641	0.89661
GOBP_INOSITOL_PHOSPHATE_MEDIATED_SIGNALING	0.934972	0.601	0.89646
GOBP_POSITIVE_REGULATION_OF_FIBROBLAST_PROLIFE	0.934591	0.578	0.89704
GOBP_POSITIVE_REGULATION_OF_PLASMA_MEMBRANE_	0.93456	0.637	0.89673
GOBP_ESTABLISHMENT_OF_MITOCHONDRION_LOCALIZAT	0.934437	0.584	0.89666
GOBP_MESONEPHRIC_TUBULE_MORPHOGENESIS	0.934435	0.612	0.89628
GOBP_IMMUNE_RESPONSE_REGULATING_CELL_SURFACE	0.934418	0.643	0.89594
GOBP_EMBRYONIC_PLACENTA_DEVELOPMENT	0.934384	0.628	0.89564
GOBP_PEPTIDE_CROSS_LINKING	0.934381	0.595	0.89525
GOBP_REGULATION_OF_OXIDATIVE_STRESS_INDUCED_IN	0.934092	0.6	0.89562
GOBP_TETRAPYRROLE_METABOLIC_PROCESS	0.933869	0.592	0.89582
GOBP_CHLORIDE_TRANSMEMBRANE_TRANSPORT	0.933799	0.614	0.89561
GOBP_PROTEIN_EXPORT_FROM_NUCLEUS	0.933712	0.618	0.89543
GOBP_ENDOCRINE_SYSTEM_DEVELOPMENT	0.933166	0.631	0.89649
GOBP_AXONAL_TRANSPORT_OF_MITOCHONDRION	0.932739	0.574	0.89719
GOBP_RETINA_MORPHOGENESIS_IN_CAMERA_TYPE_EYE	0.932427	0.602	0.89763
GOBP_POSITIVE_REGULATION_OF_HISTONE_H3_K4_METH	0.932341	0.578	0.89745
GOBP_RESPONSE_TO_CAMP	0.932152	0.611	0.89753
GOBP_PERICARDIUM_DEVELOPMENT	0.932007	0.577	0.89751
GOBP_HEART_VALVE_DEVELOPMENT	0.93194	0.623	0.8973

GOBP_PEPTIDYL_THREONINE_DEPHOSPHORYLATION	0.931861	0.574	0.89709
GOBP_NEGATIVE_REGULATION_OF_TRANSPORT	0.931786	0.706	0.8969
GOBP_REGULATION_OF_PROTEIN_LOCALIZATION_TO_NU	0.931739	0.629	0.89662
GOBP_CELLULAR_RESPONSE_TO_OSMOTIC_STRESS	0.931701	0.593	0.89634
GOBP_EXTRACELLULAR_VESICLE_BIOGENESIS	0.931472	0.584	0.89654
GOBP_LINOLEIC_ACID_METABOLIC_PROCESS	0.930826	0.595	0.89778
GOBP_SULFUR_COMPOUND_METABOLIC_PROCESS	0.930445	0.702	0.89835
GOBP_REGULATION_OF_INSULIN_RECEPTOR_SIGNALING	0.929879	0.608	0.89939
GOBP_TYPE_B_PANCREATIC_CELL_DEVELOPMENT	0.929874	0.592	0.89901
GOBP_REGULATION_OF_BINDING	0.929652	0.695	0.89922
GOBP_NEGATIVE_REGULATION_OF_JNK_CASCADE	0.929504	0.59	0.89922
GOBP_POSITIVE_REGULATION_OF_PROTEOLYSIS_INVOLV	0.929399	0.664	0.89911
GOBP_POSITIVE_REGULATION_OF_ESTABLISHMENT_OF_	0.929361	0.591	0.89884
GOBP_HEART_PROCESS	0.929307	0.685	0.89859
GOBP_ENTRY_INTO_HOST	0.92919	0.652	0.8985
GOBP_POSITIVE_REGULATION_OF_EXTRACELLULAR_MAT	0.929158	0.587	0.8982
GOBP_IMMUNE_RESPONSE_TO_TUMOR_CELL	0.928964	0.574	0.89831
GOBP_REGULATION_OF_LAMELLIPODIUM_ORGANIZATION	0.928814	0.613	0.8983
GOBP_POSITIVE_REGULATION_OF_CELL_KILLING	0.928546	0.59	0.89857
GOBP_CATECHOL_CONTAINING_COMPOUND_METABOLIC	0.928501	0.606	0.89831
GOBP_REGULATION_OF_ADENYLATE_CYCLASE_ACTIVITY	0.928463	0.576	0.89803
GOBP_POSITIVE_REGULATION_OF_CELL_JUNCTION_ASSE	0.928181	0.643	0.89837
GOBP_RIBONUCLEOSIDE_TRIPHOSPHATE_BIOSYNTHETIC	0.928121	0.62	0.89815
GOBP_REGULATION_OF_SUPRAMOLECULAR_FIBER_ORG	0.928119	0.725	0.89777
GOBP_RELEASE_OF_CYTOCHROME_C_FROM_MITOCHON	0.927987	0.607	0.89772
GOBP_LIPID_LOCALIZATION	0.92774	0.732	0.89795
GOBP_DEPHOSPHORYLATION	0.927651	0.73	0.89779
GOBP_PHOSPHATIDYLETHANOLAMINE_METABOLIC_PROC	0.927609	0.582	0.89751
GOBP_POSITIVE_REGULATION_OF_MITOCHONDRION_OR	0.927194	0.627	0.89817
GOBP_RESPONSE_TO_ZINC_ION	0.926888	0.589	0.89855
GOBP_REGULATION_OF_LIPID_LOCALIZATION	0.926562	0.644	0.89898
GOBP_REGULATION_OF_KERATINOCYTE_PROLIFERATION	0.926533	0.613	0.89868
GOBP_PROTEIN_AUTOPHOSPHORYLATION	0.926493	0.681	0.8984
GOBP_REGULATION_OF_LIPID_BIOSYNTHETIC_PROCESS	0.926282	0.656	0.89853
GOBP_NEGATIVE_REGULATION_OF_LIPID_LOCALIZATION	0.926002	0.599	0.89883
GOBP_FOREBRAIN_REGIONALIZATION	0.92596	0.579	0.89855
GOBP_REGULATION_OF_STEM_CELL_POPULATION_MAINT	0.92577	0.604	0.89864
GOBP_DNA_REPAIR	0.925564	0.767	0.89877
GOBP_POSITIVE_REGULATION_OF_APOPTOTIC_SIGNALIN	0.925534	0.646	0.89847
GOBP_HEMATOPOIETIC_STEM_CELL_DIFFERENTIATION	0.925273	0.6	0.89873
GOBP_REGULATION_OF_GLYCOGEN_METABOLIC_PROCE	0.925043	0.615	0.89892
GOBP_REGULATION_OF_MICROTUBULE_CYTOSKELETON_	0.92483	0.68	0.89908
GOBP_PHOSPHATIDYLCHOLINE_METABOLIC_PROCESS	0.924585	0.611	0.89932
GOBP_REGULATION_OF_DNA_TEMPLATED_TRANSCRIPTIO	0.924416	0.609	0.89934
GOBP_POSITIVE_REGULATION_OF_RUFFLE_ASSEMBLY	0.924194	0.596	0.89952
GOBP_GOLGI_TO_ENDOSOME_TRANSPORT	0.923964	0.604	0.8997
GOBP_PROTEIN_PROCESSING	0.923849	0.704	0.89961
GOBP_AMINE_METABOLIC_PROCESS	0.923141	0.644	0.90097
GOBP_AMYLOID_FIBRIL_FORMATION	0.922972	0.594	0.90101
GOBP_NEGATIVE_REGULATION_OF_INTRACELLULAR_TRA	0.922729	0.6	0.90124
GOBP_RESPONSE_TO_COCAINE	0.922548	0.62	0.90131
GOBP_NEGATIVE_REGULATION_OF_GENE_EXPRESSION_	0.922355	0.64	0.9014
GOBP_NEGATIVE_REGULATION_OF_LIPID_METABOLIC_PR	0.92229	0.634	0.90117

GOBP_POSITIVE_REGULATION_OF_PROTEIN_LOCALIZATI(	0.922213	0.761	0.90099
GOBP_NEGATIVE_REGULATION_OF_CHEMOTAXIS	0.922036	0.616	0.90101
GOBP_NEGATIVE_REGULATION_OF_NEURON_DIFFERENT	0.921672	0.608	0.90154
GOBP_CALCIUM_ION_TRANSPORT	0.921438	0.746	0.90175
GOBP_HEPATICOBILIARY_SYSTEM_DEVELOPMENT	0.921244	0.684	0.90185
GOBP_MULTICELLULAR_ORGANISM_GROWTH	0.921143	0.68	0.9017
GOBP_PROTEASOMAL_PROTEIN_CATABOLIC_PROCESS	0.921121	0.777	0.90138
GOBP_REGULATION_OF_CELL_JUNCTION_ASSEMBLY	0.921097	0.685	0.90107
GOBP_PROTEIN_TARGETING	0.920638	0.739	0.90179
GOBP_OLEFINIC_COMPOUND_BIOSYNTHETIC_PROCESS	0.920576	0.578	0.90156
GOBP_PROTEIN_LOCALIZATION_TO_GOLGI_APPARATUS	0.92046	0.605	0.90147
GOBP_PITUITARY_GLAND_DEVELOPMENT	0.920319	0.626	0.90144
GOBP_P_BODY_ASSEMBLY	0.920295	0.612	0.90112
GOBP_REGULATION_OF_ERBB_SIGNALING_PATHWAY	0.919993	0.629	0.9015
GOBP_REGULATION_OF_SYNAPSE_ASSEMBLY	0.919856	0.654	0.90146
GOBP_REGULATION_OF_DENDRITE_EXTENSION	0.919687	0.624	0.90149
GOBP_REGULATION_OF_STEROL_TRANSPORT	0.919642	0.603	0.90123
GOBP_REGULATION_OF_PROTEIN_ACETYLATION	0.919463	0.647	0.90129
GOBP_EMBRYONIC_HEART_TUBE_DEVELOPMENT	0.919268	0.645	0.9014
GOBP_SKELETAL_MUSCLE_CONTRACTION	0.919015	0.621	0.90165
GOBP_REGULATION_OF_ERYTHROCYTE_DIFFERENTIATIC	0.918983	0.635	0.90136
GOBP_REGULATION_OF_OXIDOREDUCTASE_ACTIVITY	0.918952	0.671	0.90105
GOBP_POSITIVE_REGULATION_OF_INTERLEUKIN_4_PROD	0.918885	0.613	0.90085
GOBP_PROTEIN_TRANSMEMBRANE_IMPORT_INTO_INTRA	0.918442	0.61	0.90153
GOBP_POSITIVE_REGULATION_OF_LIPID_BIOSYNTHETIC_	0.918427	0.62	0.90119
GOBP_ENDOTHELIAL_CELL_CHEMOTAXIS	0.918391	0.592	0.9009
GOBP_CELLULAR_RESPONSE_TO_EXTERNAL_STIMULUS	0.917992	0.736	0.90146
GOBP_PYRIMIDINE_NUCLEOSIDE_TRIPHOSPHATE_BIOSYN	0.9178	0.603	0.90157
GOBP_POSITIVE_REGULATION_OF_CD4_POSITIVE_ALPHA	0.917724	0.6	0.90139
GOBP_INTERLEUKIN_1_PRODUCTION	0.917534	0.654	0.90146
GOBP_REGULATION_OF_PROTEIN_EXPORT_FROM_NUCLE	0.917441	0.612	0.9013
GOBP_MICROTUBULE_CYTOSKELETON_ORGANIZATION_II	0.917248	0.667	0.90139
GOBP_VIRAL_TRANSCRIPTION	0.917204	0.612	0.90113
GOBP_REGULATION_OF_T_CELL_MEDIATED_IMMUNITY	0.917094	0.641	0.90103
GOBP_RNA_CATABOLIC_PROCESS	0.917041	0.747	0.90078
GOBP_REGULATION_OF_MRNA_CATABOLIC_PROCESS	0.916864	0.669	0.90083
GOBP_NEURAL_CREST_CELL_DIFFERENTIATION	0.916313	0.642	0.90175
GOBP_SUPEROXIDE_METABOLIC_PROCESS	0.915994	0.638	0.90214
GOBP_DORSAL_VENTRAL_NEURAL_TUBE_PATTERNING	0.915366	0.607	0.90325
GOBP_FATTY_ACID_DERIVATIVE_BIOSYNTHETIC_PROCES	0.915353	0.623	0.90291
GOBP_PHOSPHATIDYLINOSITOL_PHOSPHATE_BIOSYNTHE	0.915145	0.642	0.90302
GOBP_ORGANIC_ACID_CATABOLIC_PROCESS	0.915116	0.716	0.90271
GOBP_PROTEIN_HOMOOLIGOMERIZATION	0.914978	0.685	0.90268
GOBP_PROTEIN_LOCALIZATION_TO_MICROTUBULE	0.914921	0.611	0.90245
GOBP_EAR_MORPHOGENESIS	0.914719	0.651	0.90257
GOBP_MITOCHONDRION_DISTRIBUTION	0.914538	0.619	0.90265
GOBP_REGULATION_OF_PEPTIDYL_SERINE_PHOSPHORY	0.914468	0.688	0.90245
GOBP_ENDOCARDIAL_CUSHION_FORMATION	0.914373	0.621	0.90231
GOBP_SPECIFICATION_OF_SYMMETRY	0.913956	0.68	0.90289
GOBP_DIOL_BIOSYNTHETIC_PROCESS	0.913938	0.599	0.90256
GOBP_HEART_GROWTH	0.913527	0.649	0.90319
GOBP_MITOCHONDRION_LOCALIZATION	0.913071	0.643	0.90391
GOBP_LONG_CHAIN_FATTY_ACID_METABOLIC_PROCESS	0.913045	0.651	0.90361

GOBP_SULFUR_COMPOUND_TRANSPORT	0.91298	0.646	0.90338
GOBP_ESTABLISHMENT_OF_EPITHELIAL_CELL_APICAL_BA	0.912925	0.611	0.90313
GOBP_SPLICEOSOMAL_SNRNP_ASSEMBLY	0.912832	0.613	0.90298
GOBP_PEPTIDYL_LYSINE_MODIFICATION	0.912567	0.774	0.90322
GOBP_REGULATION_OF_CELL_SHAPE	0.91252	0.682	0.90297
GOBP_POSITIVE_REGULATION_OF_TELOMERE_MAINTENA	0.911324	0.631	0.90542
GOBP_NEGATIVE_REGULATION_OF_CHROMOSOME_ORGA	0.911046	0.658	0.90571
GOBP_PEPTIDYL_LYSINE_ACETYLATION	0.910697	0.707	0.90614
GOBP_PYRIMIDINE_RIBONUCLEOTIDE_METABOLIC_PROC	0.910688	0.618	0.9058
GOBP_RNA_STABILIZATION	0.910608	0.635	0.90561
GOBP_DETOXIFICATION	0.910562	0.672	0.90534
GOBP_ORGANIC_CYCLIC_COMPOUND_CATABOLIC_PROC	0.91048	0.798	0.90518
GOBP_POSITIVE_REGULATION_OF_CHROMOSOME_ORGA	0.910444	0.655	0.9049
GOBP_GLYCEROPHOSPHOLIPID_CATABOLIC_PROCESS	0.91036	0.615	0.90472
GOBP_ISOPRENOID_BIOSYNTHETIC_PROCESS	0.910358	0.617	0.90435
GOBP_CELLULAR_RESPONSE_TO_HYDROGEN_PEROXIDE	0.909999	0.657	0.90485
GOBP_POSITIVE_REGULATION_OF_LYASE_ACTIVITY	0.909996	0.629	0.90449
GOBP_NEGATIVE_REGULATION_OF_PROTEIN_CATABOLIC	0.90984	0.68	0.90451
GOBP_AMINO_ACID_IMPORT	0.9098	0.645	0.90424
GOBP_ORGANELLE_FISSION	0.909711	0.798	0.90406
GOBP_BRAIN_MORPHOGENESIS	0.909642	0.627	0.90386
GOBP_PROTEIN_K63_LINKED_DEUBIQUITINATION	0.909617	0.631	0.90355
GOBP_NUCLEOSIDE_PHOSPHATE_BIOSYNTHETIC_PROCE	0.909493	0.73	0.90346
GOBP_VESICLE_MEDIATED_TRANSPORT_BETWEEN_ENDO	0.909335	0.641	0.90345
GOBP_REGULATION_OF_DNA_BINDING	0.90904	0.681	0.90378
GOBP_PROTON_TRANSMEMBRANE_TRANSPORT	0.908663	0.673	0.90429
GOBP_LUNG_CELL_DIFFERENTIATION	0.908447	0.622	0.90442
GOBP_POLYSACCHARIDE_METABOLIC_PROCESS	0.908405	0.661	0.90415
GOBP_CELL_PROLIFERATION_IN_FOREBRAIN	0.908254	0.62	0.90415
GOBP_POSITIVE_REGULATION_OF_DENDRITIC_SPINE_MO	0.908218	0.609	0.90386
GOBP_REGULATION_OF_CILIUM_MOVEMENT	0.907945	0.611	0.9041
GOBP_RHYTHMIC_BEHAVIOR	0.907908	0.633	0.90383
GOBP_POSITIVE_REGULATION_OF_INTRACELLULAR_TRAN	0.907893	0.72	0.90349
GOBP_DENDRITE_SELF_AVOIDANCE	0.907709	0.623	0.90357
GOBP_ENDOSOMAL_TRANSPORT	0.907698	0.75	0.90323
GOBP_CELLULAR_AMINO_ACID_METABOLIC_PROCESS	0.907665	0.754	0.90293
GOBP_POSITIVE_REGULATION_OF_CYCLASE_ACTIVITY	0.907539	0.632	0.90285
GOBP_ACTIN_FILAMENT_POLYMERIZATION	0.907455	0.697	0.90266
GOBP_NEGATIVE_REGULATION_OF_CYTOKINE_PRODUCT	0.907441	0.623	0.90232
GOBP_REGULATION_OF_METAL_ION_TRANSPORT	0.907367	0.774	0.90213
GOBP_LIPID_DROPLET_ORGANIZATION	0.906962	0.633	0.9027
GOBP_DENDRITIC_SPINE_MAINTENANCE	0.906243	0.588	0.90401
GOBP_POSITIVE_REGULATION_OF_MRNA_METABOLIC_PR	0.905947	0.698	0.9043
GOBP_MALE_GAMETE_GENERATION	0.905811	0.802	0.90425
GOBP_V_D_J_RECOMBINATION	0.905688	0.619	0.90415
GOBP_DNA_BIOSYNTHETIC_PROCESS	0.905615	0.719	0.90395
GOBP_POSITIVE_REGULATION_OF_OSTEOBLAST_DIFFER	0.905151	0.668	0.90461
GOBP_REGULATION_OF_DNA_DAMAGE_CHECKPOINT	0.904966	0.619	0.90468
GOBP_POSITIVE_REGULATION_OF_CARDIAC_MUSCLE_CE	0.904541	0.627	0.90529
GOBP_HISTONE_H4_K16_ACETYLATION	0.904221	0.628	0.90569
GOBP_THIOESTER_METABOLIC_PROCESS	0.904161	0.674	0.90545
GOBP_OLIGOSACCHARIDE_BIOSYNTHETIC_PROCESS	0.904122	0.621	0.90517
GOBP_NEUROMUSCULAR_SYNAPTIC_TRANSMISSION	0.904049	0.648	0.90498

GOBP_POSITIVE_REGULATION_OF_INTRACELLULAR_PRO	0.903553	0.708	0.90575
GOBP_NUCLEAR_BODY_ORGANIZATION	0.903421	0.607	0.90569
GOBP_ACID_SECRETION	0.90317	0.627	0.90585
GOBP_REGULATION_OF_NERVOUS_SYSTEM_PROCESS	0.903078	0.705	0.9057
GOBP_MONOCARBOXYLIC_ACID_CATABOLIC_PROCESS	0.902948	0.709	0.90565
GOBP_PROTEIN_MATURATION	0.902918	0.778	0.90535
GOBP_NEURON_PROJECTION_EXTENSION_INVOLVED_IN_	0.902883	0.634	0.90507
GOBP_LIPID_STORAGE	0.902659	0.665	0.90522
GOBP_POSITIVE_REGULATION_OF_VIRAL_PROCESS	0.902624	0.649	0.90494
GOBP_REGULATION_OF_CATION_CHANNEL_ACTIVITY	0.902391	0.72	0.90511
GOBP_REGULATION_OF_ACTIN_FILAMENT_BUNDLE_ASSE	0.902143	0.68	0.9053
GOBP_UNSATURATED_FATTY_ACID_BIOSYNTHETIC_PRO(	0.901937	0.639	0.90539
GOBP_PROTEIN_LOCALIZATION_TO_POSTSYNAPSE	0.901709	0.65	0.90552
GOBP_RESPONSE_TO_FATTY_ACID	0.901536	0.654	0.90556
GOBP_TRANSEPITHELIAL_TRANSPORT	0.901311	0.632	0.90569
GOBP_POSITIVE_REGULATION_OF_MUSCLE_CONTRACTI(	0.901101	0.644	0.9058
GOBP_MONOCARBOXYLIC_ACID_METABOLIC_PROCESS	0.901049	0.839	0.90554
GOBP_CELLULAR_GLUCOSE_HOMEOSTASIS	0.900588	0.708	0.90623
GOBP_SEMI_LUNAR_VALVE_DEVELOPMENT	0.900581	0.637	0.90589
GOBP_REACTIVE_OXYGEN_SPECIES_METABOLIC_PROCE	0.900238	0.732	0.90627
GOBP_PROTEIN_TETRAMERIZATION	0.900083	0.672	0.90628
GOBP_NON_MEMBRANE_BOUNDED_ORGANELLE_ASSEME	0.899554	0.793	0.90709
GOBP_POSITIVE_REGULATION_OF_TRANSCRIPTION_ELO	0.899506	0.633	0.90684
GOBP_SPERM_EGG_RECOGNITION	0.899151	0.623	0.90727
GOBP_MRNA_CATABOLIC_PROCESS	0.898709	0.768	0.90789
GOBP_REGULATION_OF_CILIUM_ASSEMBLY	0.898581	0.673	0.90782
GOBP_PYRIMIDINE_NUCLEOSIDE_TRIPHOSPHATE_METAB	0.898452	0.616	0.90775
GOBP_POSITIVE_REGULATION_OF_MICROTUBULE_POLYM	0.898123	0.65	0.90812
GOBP_T_CELL_CHEMOTAXIS	0.898118	0.611	0.90777
GOBP_ORGANELLE_LOCALIZATION	0.898114	0.843	0.90741
GOBP_AORTA_DEVELOPMENT	0.897954	0.653	0.90743
GOBP_POSITIVE_REGULATION_OF_SODIUM_ION_TRANSP	0.897924	0.619	0.90712
GOBP_REGULATION_OF_MULTICELLULAR_ORGANISM_GR	0.89776	0.663	0.90711
GOBP_NEGATIVE_REGULATION_OF_PROTEIN_LOCALIZAT	0.897033	0.656	0.90837
GOBP_REGULATION_OF_MUSCLE_CONTRACTION	0.89662	0.719	0.9089
GOBP_RIBOSOMAL_LARGE_SUBUNIT_ASSEMBLY	0.896016	0.647	0.90982
GOBP_REGULATION_OF_PROTEIN_SUMOYLATION	0.895947	0.65	0.90962
GOBP_LIPOPROTEIN_METABOLIC_PROCESS	0.895812	0.724	0.90958
GOBP_ANTIGEN_PROCESSING_AND_PRESENTATION	0.895682	0.654	0.90951
GOBP_POSITIVE_REGULATION_OF_ORGANELLE_ORGANIZ	0.895613	0.832	0.90929
GOBP_REGULATION_OF_PLASMA_LIPOPROTEIN_PARTICL	0.895399	0.664	0.90941
GOBP_EPITHELIAL_CELL_APOPTOTIC_PROCESS	0.894643	0.687	0.91066
GOBP_POTASSIUM_ION_TRANSPORT	0.894465	0.769	0.91068
GOBP_REGULATION_OF_HAIR_FOLLICLE_DEVELOPMENT	0.894369	0.636	0.91053
GOBP_REGULATION_OF_B_CELL_DIFFERENTIATION	0.893985	0.652	0.91102
GOBP_REGULATION_OF_SPROUTING_ANGIOGENESIS	0.89384	0.651	0.91098
GOBP_POSITIVE_REGULATION_OF_PROTEIN_SERINE_THI	0.893752	0.771	0.91082
GOBP_REGULATION_OF_PROTEIN_MODIFICATION_BY_SM	0.893697	0.788	0.91057
GOBP_POSITIVE_REGULATION_OF_CYSTEINE_TYPE_END(	0.893133	0.734	0.91145
GOBP_HEMOGLOBIN_METABOLIC_PROCESS	0.893095	0.653	0.91117
GOBP_SMALL_MOLECULE_CATABOLIC_PROCESS	0.89306	0.808	0.91089
GOBP_NEURON_CELLULAR_HOMEOSTASIS	0.893052	0.655	0.91055
GOBP_POSITIVE_REGULATION_OF_CHROMOSOME_SEPAI	0.892199	0.65	0.91202

GOBP_OUTFLOW_TRACT_MORPHOGENESIS	0.892085	0.707	0.91192
GOBP_NEGATIVE_REGULATION_OF_UBIQUITIN_DEPENDE	0.891294	0.66	0.91328
GOBP_REGULATION_OF_SYNAPTIC_TRANSMISSION_GABA	0.891175	0.64	0.91318
GOBP_RENAL_WATER_HOMEOSTASIS	0.890482	0.634	0.91434
GOBP_POSITIVE_REGULATION_OF_MONOOXYGENASE_AC	0.889799	0.645	0.91544
GOBP_PIGMENTATION	0.889772	0.709	0.91514
GOBP_PHOSPHATIDYLINOSITOL_BIOSYNTHETIC_PROCES	0.889495	0.717	0.91537
GOBP_LEUKOCYTE_MEDIATED_CYTOTOXICITY	0.88942	0.685	0.91517
GOBP_O_GLYCAN_PROCESSING	0.889024	0.664	0.91565
GOBP_RNA_SURVEILLANCE	0.888554	0.636	0.91631
GOBP_PROTEIN_HOMOTETRAMERIZATION	0.888532	0.685	0.916
GOBP_REGULATION_OF_MEMBRANE_LIPID_DISTRIBUTION	0.888374	0.663	0.91598
GOBP_HAIR_CELL_DIFFERENTIATION	0.888119	0.66	0.91611
GOBP_GLYCEROPHOSPHOLIPID_METABOLIC_PROCESS	0.888006	0.801	0.916
GOBP_REGULATION_OF_ACTIN_FILAMENT_ORGANIZATIO	0.887797	0.79	0.91609
GOBP_CD8_POSITIVE_ALPHA_BETA_T_CELL_ACTIVATION	0.88741	0.642	0.91654
GOBP_REGULATION_OF_CAMP_MEDIATED_SIGNALING	0.887382	0.66	0.91624
GOBP_REGULATION_OF_CALCIUM_ION_TRANSMEMBRANE	0.886431	0.682	0.91783
GOBP_PHOTOPERIODISM	0.886417	0.647	0.91751
GOBP_POLARIZED_EPITHELIAL_CELL_DIFFERENTIATION	0.886282	0.65	0.91743
GOBP_POSITIVE_REGULATION_OF_CELL_CYCLE_PHASE_	0.886201	0.712	0.91725
GOBP_ESTABLISHMENT_OF_SPINDLE_ORIENTATION	0.88544	0.668	0.91851
GOBP_POSITIVE_REGULATION_OF_CD4_POSITIVE_ALPHA	0.885166	0.659	0.91875
GOBP_POSITIVE_REGULATION_OF_ION_TRANSPORT	0.885037	0.782	0.91866
GOBP_POSITIVE_REGULATION_OF_ALPHA_BETA_T_CELL_	0.884764	0.649	0.91884
GOBP_PARASYMPATHETIC_NERVOUS_SYSTEM_DEVELOP	0.884392	0.65	0.91926
GOBP_ENDOCARDIAL_CUSHION_MORPHOGENESIS	0.884139	0.667	0.91943
GOBP_RECEPTOR_METABOLIC_PROCESS	0.884063	0.744	0.91924
GOBP_VESICLE_CARGO_LOADING	0.884025	0.646	0.91897
GOBP_LONG_TERM_MEMORY	0.883962	0.647	0.91874
GOBP_REGULATION_OF_HAIR_CYCLE	0.883511	0.651	0.91931
GOBP_ATRIAL_SEPTUM_DEVELOPMENT	0.883292	0.661	0.91941
GOBP_PINOCYTOSIS	0.882979	0.665	0.91971
GOBP_T_CELL_CYTOKINE_PRODUCTION	0.881704	0.671	0.92196
GOBP_HOMOLOGOUS_CHROMOSOME_PAIRING_AT_MEIO	0.881631	0.671	0.92176
GOBP_PROTEIN_HYDROXYLATION	0.881585	0.68	0.9215
GOBP_REGULATION_OF_MITOCHONDRION_ORGANIZATIO	0.88139	0.744	0.92156
GOBP_FATTY_ACID_BETA_OXIDATION	0.880938	0.701	0.92212
GOBP_POSITIVE_REGULATION_OF_HEART_GROWTH	0.880573	0.658	0.92251
GOBP_POSITIVE_REGULATION_OF_ANION_TRANSPORT	0.879853	0.691	0.92363
GOBP_REGULATION_OF_CELL_CYCLE_CHECKPOINT	0.879496	0.669	0.92404
GOBP_LYMPHOCYTE_COSTIMULATION	0.879494	0.673	0.92369
GOBP_SEROTONIN_TRANSPORT	0.878986	0.641	0.9244
GOBP_ACTIVATION_OF_ADENYLATE_CYCLASE_ACTIVITY	0.878905	0.666	0.92421
GOBP_POSITIVE_REGULATION_OF_CALCIUM_ION_TRANSI	0.878833	0.739	0.92399
GOBP_MONOVALENT_INORGANIC_CATION_HOMEOSTASIS	0.87868	0.74	0.92397
GOBP_RRNA_CATABOLIC_PROCESS	0.878619	0.66	0.92375
GOBP_INTEGRATED_STRESS_RESPONSE_SIGNALING	0.878417	0.653	0.9238
GOBP_STEM_CELL_PROLIFERATION	0.878409	0.698	0.92345
GOBP_REGULATION_OF_CALCIUM_MEDIATED_SIGNALING	0.878163	0.694	0.92359
GOBP_GLANDULAR_EPITHELIAL_CELL_DEVELOPMENT	0.878074	0.647	0.92341
GOBP_REGULATION_OF_MONOOXYGENASE_ACTIVITY	0.878047	0.671	0.92311
GOBP_REGULATION_OF_CATION_TRANSMEMBRANE_TRA	0.877714	0.838	0.92342

GOBP_GLUTAMATE_RECEPTOR_SIGNALING_PATHWAY	0.877456	0.688	0.92359
GOBP_VACUOLAR_TRANSPORT	0.877437	0.754	0.92328
GOBP_REGULATION_OF_INSULIN_SECRETION	0.877251	0.737	0.92331
GOBP_CELL_VOLUME_HOMEOSTASIS	0.876883	0.671	0.9237
GOBP_RESPONSE_TO_GONADOTROPIN	0.876505	0.672	0.92411
GOBP_FOAM_CELL_DIFFERENTIATION	0.875939	0.67	0.92492
GOBP_ENTEROENDOCRINE_CELL_DIFFERENTIATION	0.875805	0.666	0.92483
GOBP_REGULATION_OF_LIPID_TRANSPORT	0.875555	0.731	0.92501
GOBP_CARTILAGE_DEVELOPMENT_INVOLVED_IN_ENDOC	0.875197	0.691	0.92533
GOBP_B_CELL_HOMEOSTASIS	0.875062	0.664	0.92526
GOBP_PHOSPHATIDYLCHOLINE_BIOSYNTHETIC_PROCESS	0.87455	0.671	0.92592
GOBP_RESPONSE_TO_MANGANESE_ION	0.87437	0.655	0.92594
GOBP_POSITIVE_REGULATION_OF_ESTABLISHMENT_OF_	0.874154	0.83	0.926
GOBP_CELL_MATURATION	0.873963	0.752	0.92602
GOBP_BIOLOGICAL_PROCESS_INVOLVED_IN_INTERACTIC	0.873583	0.778	0.9264
GOBP_POSITIVE_REGULATION_OF_MYELOID_LEUKOCYTE	0.873469	0.658	0.92627
GOBP_CELLULAR_AMINO_ACID_BIOSYNTHETIC_PROCESS	0.873158	0.699	0.92656
GOBP_MYOTUBE_CELL_DEVELOPMENT	0.873139	0.672	0.92625
GOBP_CATECHOLAMINE_UPTAKE	0.873095	0.641	0.92599
GOBP_HEART_VALVE_MORPHOGENESIS	0.873046	0.698	0.92574
GOBP_HISTONE_MODIFICATION	0.87289	0.874	0.92569
GOBP_NEURONAL_STEM_CELL_POPULATION_MAINTENAN	0.872239	0.657	0.92662
GOBP_GLAND_DEVELOPMENT	0.872087	0.872	0.92656
GOBP_RESPONSE_TO_LEAD_ION	0.871496	0.668	0.92739
GOBP_CELLULAR_RESPONSE_TO_PEPTIDE	0.871419	0.852	0.92718
GOBP_POSITIVE_REGULATION_OF_RELEASE_OF_CYTOCH	0.871165	0.679	0.92735
GOBP_NEGATIVE_REGULATION_OF_STRIATED_MUSCLE_(	0.871109	0.69	0.92711
GOBP_POSITIVE_REGULATION_OF_TRANSCRIPTION_BY_F	0.871097	0.683	0.92678
GOBP_REGULATION_OF_LIPID_METABOLIC_PROCESS	0.870953	0.846	0.9267
GOBP_BLASTODERM_SEGMENTATION	0.87034	0.656	0.92755
GOBP_TRANSCRIPTION_INITIATION_FROM_RNA_POLYMEF	0.8703	0.735	0.92727
GOBP_DEMETHYLATION	0.869914	0.697	0.92769
GOBP_NEUROTROPHIN_SIGNALING_PATHWAY	0.869912	0.701	0.92734
GOBP_REGULATION_OF_RECEPTOR_LOCALIZATION_TO_S	0.869639	0.656	0.92751
GOBP_PIGMENT_GRANULE_LOCALIZATION	0.869584	0.667	0.92726
GOBP_REGULATION_OF_HEART_GROWTH	0.869093	0.7	0.92791
GOBP_PROTEIN_INSERTION_INTO_ER_MEMBRANE	0.868786	0.692	0.92814
GOBP_KIDNEY_MESENCHYME_DEVELOPMENT	0.867988	0.681	0.92937
GOBP_LIPID_HOMEOSTASIS	0.867934	0.767	0.92913
GOBP_REGULATION_OF_PROTEIN_LOCALIZATION_TO_CE	0.867901	0.7	0.92885
GOBP_CIRCADIAN_REGULATION_OF_GENE_EXPRESSION	0.86785	0.708	0.9286
GOBP_ANTERIOR_POSTERIOR_AXIS_SPECIFICATION	0.867642	0.706	0.92867
GOBP_POST_GOLGI_VESICLE_MEDIATED_TRANSPORT	0.867618	0.743	0.92837
GOBP_GROWTH_PLATE_CARTILAGE_DEVELOPMENT	0.86684	0.658	0.92954
GOBP_ARTERY_MORPHOGENESIS	0.866648	0.744	0.92956
GOBP_MAINTENANCE_OF_PROTEIN_LOCATION_IN_NUCLE	0.866413	0.67	0.92967
GOBP_REGULATION_OF_TRANSPORTER_ACTIVITY	0.86629	0.824	0.92955
GOBP_NUCLEUS_ORGANIZATION	0.865806	0.764	0.93012
GOBP_POSITIVE_REGULATION_OF_ACTIN_FILAMENT_BUN	0.865763	0.708	0.92984
GOBP_ERBB_SIGNALING_PATHWAY	0.865746	0.753	0.92952
GOBP_POSITIVE_REGULATION_OF_TRANSCRIPTION_FRO	0.865631	0.675	0.92937
GOBP_INSULIN_RECEPTOR_SIGNALING_PATHWAY	0.8654	0.762	0.92945
GOBP_REGULATION_OF_ENDOPLASMIC_RETICULUM_STR	0.864734	0.693	0.9304

GOBP_PIGMENT_GRANULE_ORGANIZATION	0.86461	0.694	0.93029
GOBP_MITOTIC_CHROMOSOME_CONDENSATION	0.86436	0.686	0.93043
GOBP_SIGNAL_TRANSDUCTION_IN_ABSENCE_OF_LIGAND	0.864341	0.729	0.93011
GOBP_TISSUE_REGENERATION	0.864117	0.723	0.93019
GOBP_NEGATIVE_REGULATION_OF_CATABOLIC_PROCES	0.863694	0.864	0.93064
GOBP_MEMBRANE_LIPID_METABOLIC_PROCESS	0.863646	0.797	0.93037
GOBP_PEPTIDYL_LYSINE_DIMETHYLATION	0.86349	0.662	0.93032
GOBP_REGULATION_OF_CARBOHYDRATE_BIOSYNTHETIC	0.863425	0.726	0.9301
GOBP_FATTY_ACID_DERIVATIVE_METABOLIC_PROCESS	0.863181	0.738	0.9302
GOBP_FIBROBLAST_PROLIFERATION	0.863178	0.732	0.92985
GOBP_MITOTIC_SPINDLE_ORGANIZATION	0.863028	0.767	0.92979
GOBP_NEUROTRANSMITTER_METABOLIC_PROCESS	0.863021	0.666	0.92945
GOBP_REGULATION_OF_FATTY_ACID_BIOSYNTHETIC_PR	0.86284	0.68	0.92944
GOBP_ESTABLISHMENT_OF_ORGANELLE_LOCALIZATION	0.86269	0.88	0.92939
GOBP_POSITIVE_REGULATION_OF_MITOTIC_CELL_CYCLE	0.86251	0.756	0.92937
GOBP_CYTOPLASMIC_TRANSLATIONAL_INITIATION	0.862458	0.676	0.92912
GOBP_PROTEIN_IMPORT_INTO_MITOCHONDRIAL_MATRIX	0.862371	0.68	0.92894
GOBP_EXCRETION	0.862369	0.732	0.92859
GOBP_PROTEIN_DESTABILIZATION	0.862356	0.702	0.92827
GOBP_STEROL_TRANSPORT	0.862272	0.756	0.92807
GOBP_VESICLE_LOCALIZATION	0.861309	0.815	0.92954
GOBP_REGULATION_OF_ATP_BIOSYNTHETIC_PROCESS	0.861071	0.671	0.92964
GOBP_POSITIVE_REGULATION_OF_MITOCHONDRIAL_TRA	0.860797	0.668	0.92982
GOBP_NEPHRON_EPITHELIUM_DEVELOPMENT	0.860026	0.769	0.93092
GOBP_POSITIVE_REGULATION_OF_PROTEIN_LOCALIZATI	0.859826	0.741	0.93099
GOBP_HORMONE_TRANSPORT	0.859104	0.847	0.93202
GOBP_NEGATIVE_REGULATION_OF_RNA_CATABOLIC_PR	0.858945	0.723	0.93197
GOBP_REGULATION_OF_PROTEIN_LOCALIZATION_TO_PL	0.858689	0.745	0.93209
GOBP_DIGESTION	0.858582	0.762	0.93194
GOBP_ACTIVATION_OF_CYSTEINE_TYPE_ENDOPEPTIDAS	0.858526	0.737	0.9317
GOBP_BRANCHING_MORPHOGENESIS_OF_AN_EPITHELIA	0.85848	0.785	0.93144
GOBP_PROTEIN_POLYMERIZATION	0.85793	0.836	0.93212
GOBP_RENAL_SYSTEM_PROCESS_INVOLVED_IN_REGULA	0.857902	0.661	0.93182
GOBP_SALIVARY_GLAND_DEVELOPMENT	0.857795	0.728	0.93167
GOBP_POSITIVE_REGULATION_OF_T_CELL_MEDIATED_CY	0.85774	0.691	0.93143
GOBP_REGULATION_OF_VASCULOGENESIS	0.857685	0.657	0.93119
GOBP_PROTEIN_DEMANNOSYLATION	0.85725	0.687	0.93168
GOBP_REGULATION_OF_RELEASE_OF_CYTOCHROME_C_	0.857237	0.726	0.93135
GOBP_GLYCEROLIPID_METABOLIC_PROCESS	0.856879	0.893	0.93164
GOBP_POSITIVE_REGULATION_OF_ORGANELLE_ASSEMB	0.856743	0.743	0.93156
GOBP_ESTABLISHMENT_OF_EPITHELIAL_CELL_POLARITY	0.856513	0.685	0.93165
GOBP_ORGANOPHOSPHATE_ESTER_TRANSPORT	0.856107	0.782	0.93205
GOBP_GLUCOSE_IMPORT	0.855935	0.742	0.93202
GOBP_NEGATIVE_REGULATION_OF_ORGANELLE_ASSEMB	0.855759	0.716	0.93199
GOBP_NUCLEIC_ACID_PHOSPHODIESTER_BOND_HYDROL	0.855588	0.839	0.93194
GOBP_INTERMEDIATE_FILAMENT_BASED_PROCESS	0.85543	0.711	0.93189
GOBP_ACTIN_FILAMENT_ORGANIZATION	0.855355	0.896	0.93169
GOBP_REGULATION_OF_ANION_TRANSPORT	0.855255	0.739	0.93153
GOBP_SPHINGOMYELIN_METABOLIC_PROCESS	0.855081	0.678	0.93151
GOBP_NEGATIVE_REGULATION_OF_ORGANELLE_ORGANI	0.854909	0.879	0.93149
GOBP_CELLULAR_RESPONSE_TO_CARBOHYDRATE_STIM	0.854612	0.775	0.93169
GOBP_REGULATION_OF_NUCLEASE_ACTIVITY	0.854456	0.691	0.93164
GOBP_MALE_SEX_DIFFERENTIATION	0.85418	0.787	0.93182

GOBP_CRANIAL_SKELETAL_SYSTEM_DEVELOPMENT	0.853628	0.722	0.93247
GOBP_NEGATIVE_REGULATION_OF_PROTEIN_CONTAININ	0.85361	0.775	0.93216
GOBP_REGULATION_OF_TRANSLATION_IN_RESPONSE_T(	0.852951	0.706	0.93302
GOBP_LUNG_MORPHOGENESIS	0.852868	0.73	0.93283
GOBP_INTESTINAL_LIPID_ABSORPTION	0.852867	0.691	0.93249
GOBP_MICROTUBULE_POLYMERIZATION	0.852843	0.756	0.93219
GOBP_SPHINGOLIPID_METABOLIC_PROCESS	0.852654	0.786	0.93217
GOBP_REGULATION_OF_VASOCONSTRICTION	0.852421	0.74	0.93226
GOBP_LATE_ENDOSOME_TO_LYSOSOME_TRANSPORT	0.852086	0.697	0.93253
GOBP_SMOOTHENED_SIGNALING_PATHWAY	0.852036	0.799	0.93227
GOBP_RIBOSE_PHOSPHATE_BIOSYNTHETIC_PROCESS	0.851983	0.814	0.93202
GOBP_MYELOID_LEUKOCYTE_MEDIATED_IMMUNITY	0.851715	0.733	0.9322
GOBP_POSITIVE_REGULATION_OF_NATURAL_KILLER_CEL	0.851412	0.695	0.9324
GOBP_SYNAPTIC_VESICLE_TRANSPORT	0.851303	0.719	0.93226
GOBP_POSITIVE_REGULATION_OF_EMBRYONIC_DEVELOF	0.851253	0.687	0.93201
GOBP_N_TERMINAL_PROTEIN_AMINO_ACID_MODIFICATIO	0.851198	0.674	0.93177
GOBP_STORE_OPERATED_CALCIUM_ENTRY	0.851104	0.684	0.9316
GOBP_CELL_CYCLE_DNA_REPLICATION	0.850958	0.711	0.93152
GOBP_CELL_DIFFERENTIATION_INVOLVED_IN_EMBRYONI	0.849815	0.689	0.93328
GOBP_ESTABLISHMENT_OF_PROTEIN_LOCALIZATION_TO_	0.84962	0.695	0.9333
GOBP_HEMATOPOIETIC_STEM_CELL_PROLIFERATION	0.849586	0.693	0.93302
GOBP_PRESYNAPTIC_ENDOCYTOSIS	0.84923	0.749	0.93331
GOBP_REGULATION_OF_DNA_DEPENDENT_DNA_REPLICA	0.848872	0.735	0.93359
GOBP_ANTIGEN_PROCESSING_AND_PRESENTATION_OF_	0.848792	0.702	0.93339
GOBP_HOMOLOGOUS_CHROMOSOME_SEGREGATION	0.848569	0.726	0.93345
GOBP_CHAPERONE_MEDIATED_AUTOPHAGY	0.848282	0.703	0.93361
GOBP_NEPHRON_MORPHOGENESIS	0.84809	0.765	0.93362
GOBP_CELL_CELL_ADHESION_MEDIATED_BY_CADHERIN	0.848061	0.706	0.93333
GOBP_REGULATION_OF_RESPONSE_TO_TUMOR_CELL	0.847903	0.689	0.93327
GOBP_POSITIVE_REGULATION_OF_CARDIOCYTE_DIFFERE	0.847808	0.67	0.9331
GOBP_REGULATION_OF_PROTEIN_CATABOLIC_PROCESS	0.8472	0.894	0.93384
GOBP_FATTY_ACID_METABOLIC_PROCESS	0.847186	0.882	0.93352
GOBP_GLYCOSAMINOGLYCAN_CATABOLIC_PROCESS	0.846413	0.687	0.93455
GOBP_DETECTION_OF_LIGHT_STIMULUS_INVOLVED_IN_S	0.846105	0.704	0.93475
GOBP_ACTOMYOSIN_STRUCTURE_ORGANIZATION	0.845829	0.827	0.93492
GOBP_GLYCEROLIPID_BIOSYNTHETIC_PROCESS	0.845434	0.851	0.93529
GOBP_REGULATION_OF_ENDOCYTOSIS	0.845245	0.84	0.93528
GOBP_LIPID_EXPORT_FROM_CELL	0.845095	0.711	0.93518
GOBP_KETONE_BIOSYNTHETIC_PROCESS	0.844795	0.745	0.93538
GOBP_MITOTIC_CYTOKINESIS	0.844479	0.762	0.9356
GOBP_REGULATION_OF_CELLULAR_PROTEIN_CATABOLIC	0.844397	0.864	0.93541
GOBP_CARBOHYDRATE_TRANSPORT	0.844063	0.794	0.93564
GOBP_INTERLEUKIN_5_PRODUCTION	0.843621	0.701	0.9361
GOBP_KIDNEY_MORPHOGENESIS	0.843321	0.785	0.93627
GOBP_PURINE_NUCLEOSIDE_MONOPHOSPHATE_BIOSYN	0.843074	0.699	0.93638
GOBP_VESICLE_TRANSPORT_ALONG_MICROTUBULE	0.842538	0.736	0.93699
GOBP_POSITIVE_REGULATION_OF_INTERLEUKIN_1_PROD	0.842449	0.749	0.9368
GOBP_DE_NOVO_PROTEIN_FOLDING	0.842412	0.711	0.93653
GOBP_CALCIUM_ION_IMPORT	0.842237	0.771	0.93649
GOBP_REGULATION_OF_SODIUM_ION_TRANSPORT	0.84156	0.75	0.93733
GOBP_NEURAL_RETINA_DEVELOPMENT	0.841423	0.758	0.93723
GOBP_POSITIVE_REGULATION_OF_PEPTIDYL_SERINE_PH	0.841422	0.767	0.93689
GOBP_NEGATIVE_REGULATION_OF_CARDIAC_MUSCLE_T	0.84128	0.705	0.93678

GOBP_NEGATIVE_REGULATION_OF_ERK1_AND_ERK2_CA	0.84108	0.765	0.93679
GOBP_ADIPOSE_TISSUE_DEVELOPMENT	0.841061	0.727	0.93648
GOBP_REGULATION_OF_CELL_FATE_COMMITMENT	0.839983	0.7	0.938
GOBP_REGULATION_OF_MICROTUBULE_POLYMERIZATIO	0.839657	0.791	0.93821
GOBP_NEGATIVE_REGULATION_OF_LIPID_TRANSPORT	0.83941	0.711	0.93828
GOBP_NEGATIVE_REGULATION_OF_RECEPTOR_SIGNALI	0.839318	0.72	0.9381
GOBP_REGULATION_OF_MYELINATION	0.839064	0.726	0.93819
GOBP_NEGATIVE_REGULATION_OF_ERBB_SIGNALING_PA	0.838966	0.715	0.93801
GOBP_POSITIVE_REGULATION_OF_FOCAL_ADHESION_AS	0.838938	0.718	0.93772
GOBP_REGULATION_OF_CIRCADIAN_RHYTHM	0.838753	0.794	0.9377
GOBP_POLY_A_PLUS_MRNA_EXPORT_FROM_NUCLEUS	0.838492	0.707	0.93781
GOBP_REGULATION_OF_ACTION_POTENTIAL	0.838273	0.749	0.93786
GOBP_REGULATION_OF_ALCOHOL_BIOSYNTHETIC_PROC	0.837951	0.73	0.93809
GOBP_RESPONSE_TO_SALT_STRESS	0.837851	0.703	0.93791
GOBP_MACROAUTOPHAGY	0.837752	0.885	0.93774
GOBP_MITOCHONDRIAL_GENOME_MAINTENANCE	0.837366	0.727	0.93807
GOBP_GLYCOPROTEIN_BIOSYNTHETIC_PROCESS	0.837283	0.891	0.93787
GOBP_ENDOPLASMIC_RETICULUM_TO_CYTOSOL_TRANS	0.83728	0.727	0.93754
GOBP_NEGATIVE_REGULATION_OF_GTPASE_ACTIVITY	0.836051	0.742	0.9393
GOBP_PROTEIN_DEGLYCOSYLATION	0.83595	0.715	0.93913
GOBP_MITOTIC_INTRA_S_DNA_DAMAGE_CHECKPOINT_SI	0.835768	0.695	0.93912
GOBP_RENAL_TUBULE_DEVELOPMENT	0.835551	0.773	0.93915
GOBP_NUCLEOSIDE_TRIPHOSPHATE_METABOLIC_PROCE	0.835363	0.765	0.93913
GOBP_NEGATIVE_REGULATION_OF_CALCIUM_ION_TRANS	0.834923	0.737	0.93955
GOBP_CELLULAR_RESPONSE_TO_ACID_CHEMICAL	0.834478	0.771	0.93993
GOBP_POSITIVE_REGULATION_OF_METAPHASE_ANAPHA	0.833803	0.709	0.94076
GOBP_POSITIVE_REGULATION_OF_INTERFERON_GAMMA	0.833521	0.766	0.94091
GOBP_ACTIN_FILAMENT_BUNDLE_ORGANIZATION	0.833117	0.836	0.94124
GOBP_LONG_CHAIN_FATTY_ACID_BIOSYNTHETIC_PROCE	0.833107	0.718	0.94092
GOBP_REGULATION_OF_GLYCOPROTEIN_METABOLIC_PR	0.833099	0.748	0.94059
GOBP_NEGATIVE_REGULATION_OF_CILIUM_ASSEMBLY	0.832576	0.712	0.94114
GOBP_REGULATION_OF_NMDA_RECEPTOR_ACTIVITY	0.832153	0.723	0.94153
GOBP_CARBOHYDRATE_BIOSYNTHETIC_PROCESS	0.831687	0.848	0.94198
GOBP_REGULATION_OF_LEUKOCYTE_MEDIATED_CYTOT	0.831571	0.749	0.94185
GOBP_ACTIVATED_T_CELL_PROLIFERATION	0.831456	0.733	0.94172
GOBP_ACTIVATION_OF_PROTEIN_KINASE_ACTIVITY	0.831273	0.82	0.94168
GOBP_CELLULAR_NITROGEN_COMPOUND_CATABOLIC_P	0.83038	0.937	0.94286
GOBP_POSITIVE_REGULATION_OF_INFLAMMATORY_RES	0.830045	0.801	0.94308
GOBP_RNA_PHOSPHODIESTER_BOND_HYDROLYSIS_EXO	0.830011	0.757	0.94279
GOBP_REGULATION_OF_POTASSIUM_ION_TRANSMEMBR	0.829799	0.765	0.9428
GOBP_PERK_MEDIATED_UNFOLDED_PROTEIN_RESPONS	0.829726	0.711	0.94258
GOBP_HISTONE_H2A_UBIQUITINATION	0.829455	0.743	0.94271
GOBP_POSITIVE_REGULATION_OF_ORGANIC_ACID_TRAN	0.829021	0.732	0.94307
GOBP_DNA_CONFORMATION_CHANGE	0.828816	0.891	0.94307
GOBP_RESPONSE_TO_HEPATOCYTE_GROWTH_FACTOR	0.828561	0.721	0.94317
GOBP_CERAMIDE_METABOLIC_PROCESS	0.828425	0.806	0.94304
GOBP_POSITIVE_REGULATION_OF_TRANSMEMBRANE_TR	0.827556	0.879	0.94407
GOBP_TELOMERE_ORGANIZATION	0.827372	0.82	0.94402
GOBP_QUINONE_METABOLIC_PROCESS	0.827215	0.736	0.94395
GOBP_MEIOTIC_CELL_CYCLE_PROCESS	0.826773	0.847	0.94433
GOBP_ACTIN_POLYMERIZATION_OR_DEPOLYMERIZATION	0.826373	0.843	0.94464
GOBP_VESICLE_ORGANIZATION	0.826117	0.892	0.94471
GOBP_INSULIN_SECRETION_INVOLVED_IN_CELLULAR_RE	0.825887	0.771	0.94478

GOBP_POSITIVE_REGULATION_OF_ACTIN_NUCLEATION	0.825792	0.725	0.9446
GOBP_MODIFICATION_OF_SYNAPTIC_STRUCTURE	0.825009	0.74	0.94555
GOBP_NUCLEOTIDE_EXCISION_REPAIR	0.824996	0.754	0.94523
GOBP_GLOMERULAR_EPITHELIAL_CELL_DIFFERENTIATIO	0.824301	0.72	0.94603
GOBP_NEGATIVE_REGULATION_OF_MIRNA_TRANSCRIPTI	0.823998	0.724	0.94618
GOBP_CELLULAR_RESPONSE_TO_HORMONE_STIMULUS	0.823692	0.955	0.94634
GOBP_REGULATION_OF_HISTONE_H3_K4_METHYLATION	0.823438	0.752	0.94643
GOBP_REGULATION_OF_TELOMERASE_ACTIVITY	0.823266	0.762	0.94637
GOBP_REGULATION_OF_HISTONE_DEACETYLATION	0.82285	0.738	0.9467
GOBP_GLUTAMINE_FAMILY_AMINO_ACID_METABOLIC_PR	0.822523	0.788	0.94692
GOBP_REGULATION_OF_SUPEROXIDE_METABOLIC_PROC	0.822463	0.732	0.94668
GOBP_POSITIVE_REGULATION_OF_PROTEIN_IMPORT	0.822457	0.772	0.94636
GOBP_MITOCHONDRIAL_MEMBRANE_ORGANIZATION	0.822429	0.808	0.94606
GOBP_FATTY_ACID_TRANSMEMBRANE_TRANSPORT	0.821182	0.745	0.94775
GOBP_AORTIC_VALVE_MORPHOGENESIS	0.821163	0.754	0.94744
GOBP_REGULATION_OF_MEIOTIC_NUCLEAR_DIVISION	0.820892	0.751	0.94752
GOBP_REGULATION_OF_CARDIAC_MUSCLE_CONTRACTIC	0.820399	0.729	0.94799
GOBP_SPHINGOLIPID_BIOSYNTHETIC_PROCESS	0.819902	0.815	0.94843
GOBP_POSITIVE_REGULATION_OF_CELLULAR_PROTEIN_	0.819823	0.856	0.94822
GOBP_REGULATION_OF_PROTEIN_LOCALIZATION_TO_CE	0.81908	0.857	0.94906
GOBP_HISTONE_H2A_MONOUBIQUITINATION	0.818757	0.731	0.94926
GOBP_POSITIVE_REGULATION_OF_MAP_KINASE_ACTIVIT	0.818706	0.826	0.949
GOBP_NEGATIVE_REGULATION_OF_ENDOPLASMIC_RETIC	0.81861	0.74	0.94881
GOBP_INTRINSIC_APOPTOTIC_SIGNALING_PATHWAY_IN_	0.818409	0.782	0.94879
GOBP_NEGATIVE_REGULATION_OF_OXIDOREDUCTASE_A	0.818142	0.726	0.9489
GOBP_PIGMENT_BIOSYNTHETIC_PROCESS	0.817322	0.779	0.94984
GOBP_POSITIVE_REGULATION_OF_TELOMERE_MAINTENA	0.817167	0.768	0.94976
GOBP_HYPEROSMOTIC_RESPONSE	0.817115	0.763	0.94951
GOBP_GLYCEROL_ETHER_METABOLIC_PROCESS	0.816888	0.745	0.94954
GOBP_REGULATION_OF_TRANSCRIPTION_INVOLVED_IN_	0.816778	0.742	0.94936
GOBP_SPINAL_CORD_MOTOR_NEURON_DIFFERENTIATIO	0.816262	0.748	0.94986
GOBP_PROTEIN_LOCALIZATION_TO_ENDOSOME	0.816188	0.739	0.94964
GOBP_TELOMERE_MAINTENANCE	0.816037	0.851	0.94954
GOBP_RESPONSE_TO_SALT	0.815881	0.734	0.94945
GOBP_POSITIVE_REGULATION_OF_CYTOSKELETON_ORG	0.815653	0.873	0.94949
GOBP_MEMBRANE_PROTEIN_ECTODOMAIN_PROTEOLYSI	0.815188	0.755	0.94985
GOBP_BASE_EXCISION_REPAIR	0.814733	0.773	0.95022
GOBP_POSITIVE_REGULATION_OF_HISTONE_MODIFICATI	0.814465	0.813	0.9503
GOBP_CELLULAR_RESPONSE_TO_MECHANICAL_STIMULU	0.814155	0.795	0.95045
GOBP_LABYRINTHINE_LAYER_DEVELOPMENT	0.81365	0.764	0.95087
GOBP_NEGATIVE_REGULATION_OF_ORGANIC_ACID_TRAN	0.813599	0.739	0.95061
GOBP_ALDITOL_METABOLIC_PROCESS	0.81319	0.732	0.95092
GOBP_REGULATION_OF_MUSCLE_SYSTEM_PROCESS	0.812833	0.879	0.95115
GOBP_REGULATION_OF_HORMONE_SECRETION	0.812084	0.868	0.95193
GOBP_TOLL_LIKE_RECEPTOR_3_SIGNALING_PATHWAY	0.812072	0.756	0.95161
GOBP_RESPONSE_TO_CATECHOLAMINE	0.811793	0.807	0.95172
GOBP_EPITHELIAL_CELL_DEVELOPMENT	0.811241	0.884	0.95225
GOBP_PHOSPHATIDYLINOSITOL_3_PHOSPHATE_BIOSYNT	0.810719	0.77	0.95271
GOBP_FC_GAMMA_RECEPTOR_SIGNALING_PATHWAY	0.810651	0.754	0.95248
GOBP_RETROGRADE_TRANSPORT_ENDOSOME_TO_GOL	0.810521	0.822	0.95234
GOBP_SISTER_CHROMATID_COHESION	0.81047	0.78	0.95207
GOBP_RNA_MEDIATED_GENE_SILENCING_BY_INHIBITION_	0.810318	0.737	0.95196
GOBP_PEPTIDYL_ASPARAGINE_MODIFICATION	0.810051	0.767	0.95203

GOBP_ENERGY_RESERVE_METABOLIC_PROCESS	0.809807	0.809	0.95208
GOBP_POSITIVE_REGULATION_OF_REACTIVE_OXYGEN_S	0.809777	0.789	0.95179
GOBP_RESPONSE_TO_INTERFERON_ALPHA	0.80971	0.748	0.95156
GOBP_REGULATION_OF_VIRAL_PROCESS	0.809522	0.844	0.95149
GOBP_REGULATION_OF_DEFENSE_RESPONSE_TO_VIRUS	0.809508	0.768	0.95118
GOBP_NEGATIVE_REGULATION_OF_INTRACELLULAR_PRO	0.809394	0.775	0.95102
GOBP_CELLULAR_AMINO_ACID_CATABOLIC_PROCESS	0.808359	0.814	0.95221
GOBP_IMPORT_ACROSS_PLASMA_MEMBRANE	0.80794	0.856	0.95252
GOBP_SUBPALLIUM_DEVELOPMENT	0.807641	0.753	0.95263
GOBP_VACUOLE_ORGANIZATION	0.806794	0.889	0.95359
GOBP_GLYCEROPHOSPHOLIPID_BIOSYNTHETIC_PROCES	0.806537	0.901	0.95364
GOBP_REGULATION_OF_EMBRYONIC_DEVELOPMENT	0.806535	0.807	0.95332
GOBP_TIGHT_JUNCTION_ORGANIZATION	0.806085	0.814	0.95365
GOBP_CHROMOSOME_ORGANIZATION_INVOLVED_IN_MEI	0.806072	0.793	0.95334
GOBP_EMBRYONIC_HEART_TUBE_MORPHOGENESIS	0.805612	0.789	0.95368
GOBP_PHOSPHOLIPID_METABOLIC_PROCESS	0.805198	0.945	0.95395
GOBP_PYRIMIDINE_CONTAINING_COMPOUND_BIOSYNTHE	0.805114	0.774	0.95373
GOBP_ANTIGEN_PROCESSING_AND_PRESENTATION_OF_	0.804982	0.786	0.95361
GOBP_PROTEIN_K48_LINKED_DEUBIQUITINATION	0.804537	0.755	0.95396
GOBP_RESPONSE_TO_ENDOPLASMIC_RETICULUM_STRES	0.804409	0.918	0.9538
GOBP_MEMBRANE_LIPID_BIOSYNTHETIC_PROCESS	0.803131	0.855	0.9554
GOBP_REGULATION_OF_GLUCOSE_IMPORT	0.802808	0.804	0.95555
GOBP_NEGATIVE_REGULATION_OF_AUTOPHAGY	0.802729	0.822	0.95532
GOBP_NEGATIVE_REGULATION_OF_RESPONSE_TO_ENDO	0.802492	0.797	0.95535
GOBP_ECTODERM_DEVELOPMENT	0.801218	0.748	0.9569
GOBP_REGULATION_OF_MEMBRANE_REPOLARIZATION	0.801131	0.767	0.95669
GOBP_RIBONUCLEOSIDE_TRIPHOSPHATE_METABOLIC_PR	0.801114	0.802	0.95638
GOBP_HISTONE_MONOUBIQUITINATION	0.800917	0.788	0.95633
GOBP_PURINE_CONTAINING_COMPOUND_METABOLIC_PR	0.800815	0.949	0.95614
GOBP_POSITIVE_REGULATION_OF_MYELINATION	0.800239	0.758	0.95666
GOBP_NEGATIVE_REGULATION_OF_CELLULAR_CATABOL	0.800232	0.933	0.95634
GOBP_EXTRACELLULAR_MATRIX_ASSEMBLY	0.798976	0.802	0.95781
GOBP_ACTIVATION_OF_PROTEIN_KINASE_B_ACTIVITY	0.798893	0.771	0.9576
GOBP_ICOSANOID_BIOSYNTHETIC_PROCESS	0.798599	0.764	0.95769
GOBP_REGULATION_OF_PEPTIDYL_LYSINE_ACETYLATION	0.798526	0.803	0.95747
GOBP_REGULATION_OF_FAT_CELL_DIFFERENTIATION	0.798488	0.849	0.95719
GOBP_LIPOPROTEIN_CATABOLIC_PROCESS	0.798282	0.755	0.95715
GOBP_AMMONIUM_ION_METABOLIC_PROCESS	0.797685	0.764	0.95767
GOBP_POSITIVE_REGULATION_OF_BINDING	0.79745	0.881	0.95769
GOBP_POSITIVE_REGULATION_OF_PROTEIN_MATURATIO	0.796854	0.762	0.95819
GOBP_GOLGI_ORGANIZATION	0.796787	0.88	0.95797
GOBP_CLATHRIN_DEPENDENT_ENDOCYTOSIS	0.796701	0.798	0.95777
GOBP_ACETYL_COA_METABOLIC_PROCESS	0.796262	0.763	0.95807
GOBP_CELL_SURFACE_RECEPTOR_SIGNALING_PATHWAY	0.796243	0.774	0.95777
GOBP_REGULATION_OF_PROTEIN_POLYMERIZATION	0.796134	0.888	0.9576
GOBP_HETEROCHROMATIN_ORGANIZATION	0.79521	0.835	0.95863
GOBP_SULFUR_AMINO_ACID_METABOLIC_PROCESS	0.794698	0.788	0.95901
GOBP_RESPIRATORY_BURST	0.793528	0.794	0.96032
GOBP_NUCLEOSIDE_BISPHOSPHATE_METABOLIC_PROCE	0.792843	0.872	0.96093
GOBP_POSITIVE_REGULATION_OF_CATION_CHANNEL_AC	0.79173	0.808	0.96217
GOBP_NEGATIVE_REGULATION_OF_SUPRAMOLECULAR_F	0.79029	0.889	0.96383
GOBP_RNA_POLYMERASE_II_PREINITIATION_COMPLEX_A	0.790064	0.774	0.96382
GOBP_ORGANOPHOSPHATE_BIOSYNTHETIC_PROCESS	0.789533	0.981	0.96423

GOBP_RESPONSE_TO_FLUID_SHEAR_STRESS	0.789047	0.785	0.9646
GOBP_POSITIVE_REGULATION_OF_MITOTIC_CELL_CYCLE	0.789008	0.85	0.96432
GOBP_DNA_TEMPLATED_TRANSCRIPTION_TERMINATION	0.788697	0.779	0.96441
GOBP_GLYCOPROTEIN_CATABOLIC_PROCESS	0.788183	0.765	0.96478
GOBP_RESPONSE_TO_AMINE	0.787923	0.784	0.96481
GOBP_EPITHELIAL_TO_MESENCHYMAL_TRANSITION_INVO	0.787803	0.778	0.96466
GOBP_HOMOLOGOUS_RECOMBINATION	0.787356	0.823	0.96492
GOBP_HEMATOPOIETIC_STEM_CELL_HOMEOSTASIS	0.787336	0.787	0.96462
GOBP_HYDROGEN_PEROXIDE_METABOLIC_PROCESS	0.787116	0.798	0.9646
GOBP_POSITIVE_REGULATION_OF_INTRINSIC_APOPTOTI	0.786233	0.815	0.96549
GOBP_REGULATION_OF_CELLULAR_KETONE_METABOLIC	0.785719	0.868	0.96586
GOBP_REGULATION_OF_APPETITE	0.785576	0.78	0.96573
GOBP_TRANSCRIPTION_ELONGATION_FROM_RNA_POLYM	0.785094	0.824	0.96606
GOBP_ETHER_METABOLIC_PROCESS	0.78484	0.79	0.96608
GOBP_NEGATIVE_REGULATION_OF_NECROTIC_CELL_DEA	0.784342	0.763	0.96641
GOBP_SKELETAL_MUSCLE_TISSUE_REGENERATION	0.784206	0.813	0.96626
GOBP_REGULATION_OF_REACTIVE_OXYGEN_SPECIES_M	0.783799	0.878	0.96648
GOBP_PHOTORECEPTOR_CELL_DIFFERENTIATION	0.783114	0.827	0.96705
GOBP_NEGATIVE_REGULATION_OF_NUCLEOCYTOPLASMI	0.783071	0.797	0.96679
GOBP_ALCOHOL_CATABOLIC_PROCESS	0.782849	0.823	0.96675
GOBP_NEGATIVE_REGULATION_OF_RESPONSE_TO_OXID	0.78266	0.779	0.96668
GOBP_DNA_TEMPLATED_TRANSCRIPTION_ELONGATION	0.782152	0.853	0.96705
GOBP_NEUTRAL_AMINO_ACID_TRANSPORT	0.780713	0.82	0.96868
GOBP_POSITIVE_REGULATION_OF_AMYLOID_PRECURSOR	0.780674	0.799	0.96841
GOBP_REGULATION_OF_SMALL_MOLECULE_METABOLIC_	0.778324	0.944	0.97118
GOBP_POSITIVE_REGULATION_OF_CYTOKINESIS	0.778281	0.791	0.97091
GOBP_REGULATION_OF_CHROMATIN_BINDING	0.77738	0.793	0.97176
GOBP_FATTY_ACID_CATABOLIC_PROCESS	0.777136	0.864	0.97173
GOBP_SPINDLE_ASSEMBLY	0.777067	0.875	0.9715
GOBP_NUCLEOBASE_CONTAINING_SMALL_MOLECULE_ME	0.776985	0.979	0.97127
GOBP_POSITIVE_REGULATION_OF_OXIDOREDUCTASE_AC	0.776796	0.845	0.97119
GOBP_EMBRYONIC_EYE_MORPHOGENESIS	0.776775	0.812	0.97088
GOBP_POSITIVE_REGULATION_OF_PEPTIDYL_LYSINE_AC	0.776447	0.814	0.97099
GOBP_REGULATION_OF_AMYLOID_BETA_CLEARANCE	0.776444	0.781	0.97066
GOBP_PHOSPHOLIPID_BIOSYNTHETIC_PROCESS	0.775841	0.948	0.97112
GOBP_REGULATION_OF_ACTIN_FILAMENT_LENGTH	0.775459	0.904	0.97127
GOBP_REGULATION_OF_ERAD_PATHWAY	0.775043	0.795	0.97148
GOBP_ALPHA_AMINO_ACID_CATABOLIC_PROCESS	0.775027	0.868	0.97117
GOBP_CELLULAR_RESPONSE_TO_LOW_DENSITY_LIPOPR	0.77475	0.796	0.97117
GOBP_CHLORIDE_TRANSPORT	0.774322	0.881	0.97139
GOBP_MEMBRANE_PROTEIN_PROTEOLYSIS	0.773765	0.848	0.97179
GOBP_RESPONSE_TO_NICOTINE	0.773179	0.826	0.97223
GOBP_TRICARBOXYLIC_ACID_CYCLE	0.772503	0.806	0.97275
GOBP_ALCOHOL_METABOLIC_PROCESS	0.772292	0.958	0.97269
GOBP_ALPHA_AMINO_ACID_METABOLIC_PROCESS	0.771858	0.928	0.97293
GOBP_PLASMA_MEMBRANE_PHOSPHOLIPID_SCRAMBLING	0.771677	0.82	0.97284
GOBP_POSTREPLICATION_REPAIR	0.771637	0.802	0.97256
GOBP_REGULATION_OF_AMYLOID_PRECURSOR_PROTEIN	0.771176	0.823	0.97281
GOBP_REGULATION_OF_STEM_CELL_PROLIFERATION	0.770605	0.839	0.97322
GOBP_NUCLEAR_TRANSCRIBED_MRNA_CATABOLIC_PROC	0.770072	0.801	0.97355
GOBP_MAMMARY_GLAND_EPITHELIAL_CELL_PROLIFERAT	0.769534	0.793	0.97394
GOBP_CHAPERONE_COFACTOR_DEPENDENT_PROTEIN_F	0.768681	0.805	0.9747
GOBP_ESTABLISHMENT_OF_PROTEIN_LOCALIZATION_TO	0.768312	0.792	0.97484

GOBP_STARTLE_RESPONSE	0.768304	0.792	0.97452
GOBP_N_ACETYLGLUCOSAMINE_METABOLIC_PROCESS	0.767726	0.779	0.97492
GOBP_POSITIVE_REGULATION_OF_CATABOLIC_PROCESS	0.767577	0.98	0.97479
GOBP_ESTABLISHMENT_OF_PIGMENT_GRANULE_LOCALIZ	0.767097	0.789	0.97507
GOBP_ADRENAL_GLAND_DEVELOPMENT	0.766537	0.833	0.97543
GOBP_CELLULAR_MODIFIED_AMINO_ACID_BIOSYNTHETIC	0.76636	0.828	0.97531
GOBP_PYRIMIDINE_CONTAINING_COMPOUND_CATABOLIC	0.766358	0.813	0.97498
GOBP_PHOSPHOLIPID_TRANSPORT	0.766336	0.876	0.97468
GOBP_RESPONSE_TO_TESTOSTERONE	0.766209	0.813	0.9745
GOBP_POSITIVE_REGULATION_OF_UBIQUITIN_PROTEIN_T	0.765513	0.803	0.97506
GOBP_REGULATION_OF_MACROPHAGE_DERIVED_FOAM_	0.765055	0.825	0.97528
GOBP_POSITIVE_REGULATION_OF_PROTEIN_CATABOLIC_	0.764991	0.947	0.97504
GOBP_REGULATION_OF_MITOTIC_CELL_CYCLE_SPINDLE_	0.764837	0.796	0.97489
GOBP_INTRACILIARY_TRANSPORT	0.764783	0.819	0.97464
GOBP_RESPONSE_TO_ACID_CHEMICAL	0.764381	0.894	0.97479
GOBP_REGULATION_OF_TRANSCRIPTION_BY_RNA_POLYI	0.763437	0.791	0.97559
GOBP_POSITIVE_REGULATION_OF_PROTEIN_DEACETYLA	0.763081	0.8	0.97571
GOBP_NEUTRAL_LIPID_BIOSYNTHETIC_PROCESS	0.763066	0.812	0.9754
GOBP_CHAPERONE_MEDIATED_PROTEIN_COMPLEX_ASS	0.762896	0.812	0.97527
GOBP_CELLULAR_COMPONENT_ASSEMBLY_INVOLVED_IN	0.76267	0.883	0.97522
GOBP_POSITIVE_REGULATION_OF_FILOPODIUM_ASSEMB	0.76242	0.813	0.97519
GOBP_SODIUM_ION_IMPORT_ACROSS_PLASMA_MEMBRA	0.761631	0.803	0.97581
GOBP_REGULATION_OF_PROTEIN_CONTAINING_COMPLE	0.760425	0.906	0.97697
GOBP_POSITIVE_REGULATION_OF_PROTEIN_MODIFICATI	0.760323	0.908	0.97678
GOBP_CERAMIDE_BIOSYNTHETIC_PROCESS	0.760188	0.862	0.97661
GOBP_POSITIVE_REGULATION_OF_PEPTIDE_SECRETION	0.759708	0.871	0.97687
GOBP_RNA_PHOSPHODIESTER_BOND_HYDROLYSIS	0.759105	0.919	0.97726
GOBP_INTRINSIC_APOPTOTIC_SIGNALING_PATHWAY_IN_	0.756645	0.835	0.97983
GOBP_REGULATION_OF_RESPONSE_TO_ENDOPLASMIC_	0.755889	0.885	0.98038
GOBP_NEGATIVE_REGULATION_OF_CYTOSKELETON_ORG	0.755278	0.926	0.98075
GOBP_IRON_ION_TRANSMEMBRANE_TRANSPORT	0.75464	0.808	0.98117
GOBP_CHAPERONE_MEDIATED_PROTEIN_FOLDING	0.754405	0.867	0.98111
GOBP_NON_MOTILE_CILIUM_ASSEMBLY	0.754111	0.865	0.98113
GOBP_VIRAL_TRANSLATION	0.754072	0.81	0.98085
GOBP_PROTEIN_LOCALIZATION_TO_MITOCHONDRION	0.753042	0.916	0.98172
GOBP_ENDOPLASMIC_RETICULUM_ORGANIZATION	0.752624	0.888	0.98188
GOBP_TELOMERE_MAINTENANCE_VIA_TELOMERE_LENGT	0.752598	0.88	0.98158
GOBP_POSITIVE_REGULATION_OF_ERYTHROCYTE_DIFFE	0.752532	0.821	0.98134
GOBP_VESICLE_CYTOSKELETAL_TRAFFICKING	0.751441	0.883	0.9823
GOBP_RESPONSE_TO_INTERLEUKIN_6	0.751431	0.817	0.98198
GOBP_PROTEIN_PALMITOYLATION	0.750758	0.84	0.98245
GOBP_T_CELL_MEDIATED_CYTOTOXICITY	0.750614	0.829	0.98228
GOBP_RESPONSE_TO_MUSCLE_ACTIVITY	0.749192	0.808	0.98356
GOBP_TRANSLATIONAL_TERMINATION	0.748628	0.795	0.98386
GOBP_POSITIVE_REGULATION_OF_SUPRAMOLECULAR_FI	0.748	0.942	0.98427
GOBP_NEGATIVE_REGULATION_OF_INTRACELLULAR_STE	0.747252	0.836	0.98483
GOBP_NEGATIVE_REGULATION_OF_INTERLEUKIN_1_BETA	0.747009	0.819	0.98478
GOBP_POSITIVE_REGULATION_OF_AUTOPHAGY	0.746553	0.915	0.98497
GOBP_GOLGI_VESICLE_TRANSPORT	0.745231	0.971	0.98611
GOBP_ESTABLISHMENT_OF_PROTEIN_LOCALIZATION_TO	0.744522	0.885	0.98657
GOBP_POSITIVE_REGULATION_OF_CELL_MATRIX_ADHES	0.744487	0.864	0.98629
GOBP_PLASMA_MEMBRANE_FUSION	0.744377	0.821	0.98608
GOBP_ERAD_PATHWAY	0.744355	0.898	0.98577

GOBP_HISTONE_EXCHANGE	0.744205	0.815	0.98561
GOBP_CHROMOSOME_LOCALIZATION	0.744143	0.899	0.98535
GOBP_CARBOHYDRATE_DERIVATIVE_CATABOLIC_PROCE	0.742962	0.926	0.98635
GOBP_NUCLEOSOME_MOBILIZATION	0.742839	0.834	0.98616
GOBP_POSITIVE_REGULATION_OF_MACROAUTOPHAGY	0.742615	0.891	0.98607
GOBP_REGULATION_OF_UBIQUITIN_PROTEIN_TRANSFER	0.742255	0.873	0.98613
GOBP_NEGATIVE_REGULATION_OF_TELOMERE_MAINTEN	0.74219	0.847	0.98587
GOBP_TELOMERE_MAINTENANCE_VIA_RECOMBINATION	0.741978	0.792	0.98577
GOBP_NEGATIVE_REGULATION_OF_EPITHELIAL_CELL_MI	0.741819	0.881	0.98561
GOBP_MAINTENANCE_OF_CELL_POLARITY	0.741455	0.821	0.98569
GOBP_PHAGOLYSOSOME_ASSEMBLY	0.741347	0.833	0.98548
GOBP_REGULATION_OF_UBIQUITIN_PROTEIN_LIGASE_AC	0.741125	0.839	0.98541
GOBP_SELECTIVE_AUTOPHAGY	0.74102	0.885	0.98521
GOBP_NEGATIVE_REGULATION_OF_MRNA_CATABOLIC_P	0.740912	0.887	0.98499
GOBP_COLUMNAR_CUBOIDAL_EPITHELIAL_CELL_DEVELO	0.740873	0.85	0.98471
GOBP_ESTABLISHMENT_OF_PROTEIN_LOCALIZATION_TO	0.74048	0.845	0.98481
GOBP_REGULATION_OF_LONG_TERM_NEURONAL_SYNAP	0.740291	0.831	0.98468
GOBP_REGULATION_OF_PROGRAMMED_NECROTIC_CELL	0.740081	0.833	0.98459
GOBP_PHOSPHOLIPID_CATABOLIC_PROCESS	0.739899	0.878	0.98445
GOBP_UBIQUITIN_DEPENDENT_ERAD_PATHWAY	0.739688	0.912	0.98437
GOBP_REGULATION_OF_SPINDLE_ORGANIZATION	0.739633	0.863	0.9841
GOBP_RRNA_TRANSCRIPTION	0.739092	0.847	0.98438
GOBP_POSITIVE_REGULATION_OF_NERVOUS_SYSTEM_P	0.73884	0.869	0.98433
GOBP_RUFFLE_ASSEMBLY	0.738748	0.864	0.98411
GOBP_ANDROGEN_METABOLIC_PROCESS	0.738614	0.821	0.98393
GOBP_POSITIVE_REGULATION_OF_DNA_BINDING	0.73839	0.885	0.98384
GOBP_MITOCHONDRIAL_TRANSPORT	0.738289	0.939	0.98362
GOBP_RNA_DEPENDENT_DNA_BIOSYNTHETIC_PROCESS	0.738267	0.894	0.98332
GOBP_ALPHA_BETA_T_CELL_PROLIFERATION	0.738096	0.845	0.98318
GOBP_SKELETAL_MUSCLE_ADAPTATION	0.736944	0.84	0.98414
GOBP_REGULATION_OF_EXOSOMAL_SECRETION	0.736717	0.828	0.98406
GOBP_CELLULAR_GLUCAN_METABOLIC_PROCESS	0.73655	0.888	0.98391
GOBP_AMELOGENESIS	0.736085	0.827	0.98409
GOBP_POSITIVE_REGULATION_OF_OLIGODENDROCYTE_	0.735859	0.833	0.984
GOBP_REGULATION_OF_ORGANIC_ACID_TRANSPORT	0.735749	0.876	0.9838
GOBP_REGULATION_OF_ANDROGEN_RECEPTOR_SIGNAL	0.735733	0.836	0.9835
GOBP_POSITIVE_REGULATION_OF_PHOSPHATASE_ACTIV	0.735151	0.858	0.98377
GOBP_CELLULAR_RESPONSE_TO_STARVATION	0.735117	0.936	0.98349
GOBP_POSITIVE_REGULATION_OF_CILIUM_ASSEMBLY	0.734337	0.841	0.98399
GOBP_RESPONSE_TO_STARVATION	0.733983	0.947	0.98404
GOBP_CYTOPLASMIC_SEQUESTERING_OF_PROTEIN	0.733842	0.838	0.98387
GOBP_REGULATION_OF_TRANSCRIPTION_ELONGATION_	0.733765	0.864	0.98363
GOBP_CELLULAR_RESPONSE_TO_STEROL	0.732979	0.832	0.98415
GOBP_CARBOHYDRATE_HOMEOSTASIS	0.731605	0.96	0.98525
GOBP_LIPID_MODIFICATION	0.731262	0.955	0.9853
GOBP_2_OXOGLUTARATE_METABOLIC_PROCESS	0.731248	0.842	0.98499
GOBP_NADH_METABOLIC_PROCESS	0.730058	0.858	0.9859
GOBP_PHOSPHOLIPID_DEPHOSPHORYLATION	0.729661	0.899	0.98599
GOBP_REGULATION_OF_INTRACELLULAR_STEROID_HOR	0.729192	0.9	0.98613
GOBP_T_CELL_HOMEOSTASIS	0.728916	0.86	0.98609
GOBP_MONOSACCHARIDE_METABOLIC_PROCESS	0.728905	0.964	0.98578
GOBP_PROTEIN_LOCALIZATION_TO_CELL_PERIPHERY	0.728777	0.987	0.98559
GOBP_PARAXIAL_MESODERM_DEVELOPMENT	0.72721	0.837	0.98684

GOBP_NUCLEOTIDE_PHOSPHORYLATION	0.726906	0.926	0.98682
GOBP_RIBOSE_PHOSPHATE_METABOLIC_PROCESS	0.726851	0.982	0.98656
GOBP_CELLULAR_LIPID_CATABOLIC_PROCESS	0.726775	0.965	0.98631
GOBP_POLYOL_METABOLIC_PROCESS	0.726725	0.914	0.98604
GOBP_ATP_BIOSYNTHETIC_PROCESS	0.726019	0.872	0.98642
GOBP_MITOTIC_METAPHASE_PLATE_CONGRESSION	0.72578	0.89	0.98635
GOBP_RUFFLE_ORGANIZATION	0.725192	0.893	0.98665
GOBP_REGULATION_OF_PROTEIN_DEPOLYMERIZATION	0.723512	0.917	0.988
GOBP_NUCLEOTIDE_SALVAGE	0.723503	0.825	0.98769
GOBP_GOLGI_TO_PLASMA_MEMBRANE_TRANSPORT	0.723031	0.895	0.98782
GOBP_NEGATIVE_REGULATION_OF_LIPID_CATABOLIC_PR	0.722856	0.846	0.98768
GOBP_POSITIVE_REGULATION_OF_SMOOTHENED_SIGNA	0.722612	0.876	0.98761
GOBP_RESPONSE_TO_CARBOHYDRATE	0.722407	0.956	0.98749
GOBP_POLYOL_CATABOLIC_PROCESS	0.722372	0.839	0.98721
GOBP_REGULATION_OF_NUCLEOTIDE_METABOLIC_PROC	0.722032	0.926	0.98722
GOBP_CYTOPLASMIC_TRANSLATION	0.721741	0.956	0.98719
GOBP_PROTEIN_DEPOLYMERIZATION	0.721105	0.931	0.9875
GOBP_POSITIVE_REGULATION_OF_LIPID_STORAGE	0.721078	0.841	0.9872
GOBP_CARDIAC_MYOFIBRIL_ASSEMBLY	0.720923	0.837	0.98704
GOBP_MEGAKARYOCYTE_DIFFERENTIATION	0.720866	0.878	0.98678
GOBP_EYE_PHOTORECEPTOR_CELL_DIFFERENTIATION	0.720328	0.884	0.98698
GOBP_CYTOKINETIC_PROCESS	0.720166	0.865	0.98681
GOBP_APICAL_JUNCTION_ASSEMBLY	0.719814	0.897	0.98683
GOBP_NEGATIVE_REGULATION_OF_I_KAPPAB_KINASE_NI	0.719775	0.898	0.98655
GOBP_CELLULAR_RESPONSE_TO_FLUID_SHEAR_STRESS	0.718964	0.861	0.98701
GOBP_HORMONE_MEDIATED_SIGNALING_PATHWAY	0.718377	0.972	0.98725
GOBP_CALCIUM_ION_REGULATED_EXOCYTOSIS_OF_NEU	0.717905	0.846	0.98739
GOBP_TRANSCRIPTION_PREINITIATION_COMPLEX_ASSEM	0.717805	0.885	0.98717
GOBP_REGULATION_OF_POSTSYNAPTIC_NEUROTRANSM	0.717685	0.851	0.98697
GOBP_NEGATIVE_REGULATION_OF_PROTEIN_TYROSINE_	0.717092	0.877	0.98721
GOBP_PROTON_TRANSPORTING_TWO_SECTOR_ATPASE_	0.71652	0.838	0.98746
GOBP_CARDIAC_ATRIUM_MORPHOGENESIS	0.715783	0.859	0.98783
GOBP_ORGANELLE_FUSION	0.715164	0.946	0.98809
GOBP_INCLUSION_BODY_ASSEMBLY	0.714305	0.866	0.98861
GOBP_PEPTIDYL_CYSTEINE_MODIFICATION	0.713574	0.889	0.989
GOBP_CORONARY_VASCULATURE_MORPHOGENESIS	0.713184	0.858	0.98906
GOBP_POSITIVE_REGULATION_OF_PROTEIN_POLYMERIZ	0.71248	0.917	0.98941
GOBP_CELLULAR_RESPONSE_TO_STEROID_HORMONE_S	0.712006	0.97	0.9895
GOBP_BRANCHING_INVOLVED_IN_SALIVARY_GLAND_MOF	0.711864	0.865	0.98931
GOBP_ZINC_ION_TRANSPORT	0.711727	0.859	0.98913
GOBP_REGULATION_OF_CALCIUM_ION_IMPORT	0.711569	0.879	0.98896
GOBP_REGULATION_OF_TELOMERE_MAINTENANCE	0.711409	0.916	0.9888
GOBP_NEGATIVE_REGULATION_OF_CALCIUM_ION_TRANS	0.710611	0.887	0.9892
GOBP_NEGATIVE_REGULATION_OF_CELLULAR_PROTEIN_	0.710413	0.914	0.98907
GOBP_REGULATION_OF_ESTABLISHMENT_OF_PROTEIN_L	0.709895	0.911	0.98923
GOBP_ENDOPLASMIC_RETICULUM_TO_GOLGI_VESICLE_M	0.709682	0.958	0.98909
GOBP_INNER_MITOCHONDRIAL_MEMBRANE_ORGANIZATI	0.70934	0.891	0.98908
GOBP_NEGATIVE_REGULATION_OF_TELOMERE_MAINTEN	0.709305	0.853	0.9888
GOBP_ANTIGEN_RECEPTOR_MEDIATED_SIGNALING_PATH	0.707599	0.96	0.99005
GOBP_NEGATIVE_REGULATION_OF_SIGNALING_RECEPTO	0.707259	0.891	0.99004
GOBP_MITOCHONDRIAL_TRANSMEMBRANE_TRANSPORT	0.70713	0.924	0.98983
GOBP_POSITIVE_REGULATION_OF_PROTEIN_LOCALIZATI	0.706641	0.906	0.98996
GOBP_MULTIVESICULAR_BODY_SORTING_PATHWAY	0.705978	0.888	0.99022

GOBP_REGULATION_OF_TYPE_I_INTERFERON_MEDIATED	0.705557	0.9	0.99027
GOBP_DNA_PACKAGING	0.705484	0.954	0.99003
GOBP_REGULATION_OF_GENE_EXPRESSION_EPIGENETIC	0.705362	0.947	0.98982
GOBP_REGULATION_OF_SISTER_CHROMATID_COHESION	0.705297	0.866	0.98956
GOBP_REGULATION_OF_PROTEIN_STABILITY	0.705266	0.985	0.98928
GOBP_COLUMNAR_CUBOIDAL_EPITHELIAL_CELL_DIFFERE	0.705093	0.919	0.98912
GOBP_INTRACELLULAR_ESTROGEN_RECEPTOR_SIGNALI	0.704868	0.894	0.989
GOBP_VESICLE_DOCKING	0.70466	0.901	0.98887
GOBP_CELLULAR_RESPONSE_TO_INSULIN_STIMULUS	0.704594	0.966	0.98861
GOBP_REGULATION_OF_SPINDLE_ASSEMBLY	0.704279	0.878	0.98858
GOBP_POLYSACCHARIDE_CATABOLIC_PROCESS	0.703851	0.851	0.98862
GOBP_CHROMATIN_ORGANIZATION	0.703579	0.998	0.98855
GOBP_SUBSTRATE_DEPENDENT_CELL_MIGRATION	0.703242	0.875	0.98853
GOBP_CELLULAR_RESPONSE_TO_PEPTIDE_HORMONE_S	0.702889	0.982	0.98854
GOBP_NEGATIVE_REGULATION_OF_ENDOCYTOSIS	0.702829	0.914	0.98827
GOBP_RESPONSE_TO_EPIDERMAL_GROWTH_FACTOR	0.702692	0.915	0.98807
GOBP_NEGATIVE_REGULATION_OF_AMYLOID_PRECURSO	0.701683	0.867	0.98861
GOBP_REGULATION_OF_MICROTUBULE_POLYMERIZATIO	0.701437	0.912	0.98851
GOBP_SCF_DEPENDENT_PROTEASOMAL_UBIQUITIN_DEP	0.701363	0.904	0.98827
GOBP_STEROID_HORMONE_MEDIATED_SIGNALING_PATH	0.700742	0.958	0.98851
GOBP_REGULATION_OF_GLUCOSE_METABOLIC_PROCESS	0.700641	0.948	0.98829
GOBP_PROTEIN_STABILIZATION	0.699636	0.98	0.98883
GOBP_VIRAL_GENE_EXPRESSION	0.699425	0.946	0.9887
GOBP_LYMPHOCYTE_HOMEOSTASIS	0.699045	0.913	0.98872
GOBP_NAD_METABOLIC_PROCESS	0.698589	0.879	0.9888
GOBP_NEGATIVE_REGULATION_OF_PROTEIN_KINASE_B_	0.69779	0.905	0.98917
GOBP_NEURAL_TUBE_PATTERNING	0.695966	0.887	0.99042
GOBP_METAPHASE_PLATE_CONGRESSION	0.695951	0.93	0.99012
GOBP_PROTEIN_TRANSMEMBRANE_TRANSPORT	0.695721	0.933	0.99001
GOBP_REGULATION_OF_TELOMERE_MAINTENANCE_VIA_	0.695209	0.93	0.99012
GOBP_MEMBRANE_FUSION	0.695147	0.964	0.98986
GOBP_RESPONSE_TO_PEPTIDE_HORMONE	0.694849	0.994	0.98979
GOBP_REGULATION_OF_ENDOTHELIAL_CELL_DEVELOPM	0.694745	0.859	0.98957
GOBP_NEGATIVE_REGULATION_OF_PROTEIN_CONTAININ	0.694299	0.933	0.98962
GOBP_ENDOMEMBRANE_SYSTEM_ORGANIZATION	0.69303	0.999	0.99036
GOBP_VASCULAR_ASSOCIATED_SMOOTH_MUSCLE_CELL	0.692832	0.889	0.9902
GOBP_RNA_PHOSPHODIESTER_BOND_HYDROLYSIS_END	0.692271	0.935	0.99034
GOBP_NUCLEAR_TRANSCRIBED_MRNA_CATABOLIC_PROC	0.690828	0.917	0.99121
GOBP_ERYTHROCYTE_DEVELOPMENT	0.690007	0.899	0.9916
GOBP_AMP_METABOLIC_PROCESS	0.688945	0.891	0.99215
GOBP_NEGATIVE_REGULATION_OF_MRNA_METABOLIC_P	0.688804	0.954	0.99195
GOBP_NATURAL_KILLER_CELL_ACTIVATION	0.688609	0.938	0.99179
GOBP_CELLULAR_CARBOHYDRATE_CATABOLIC_PROCESS	0.688509	0.896	0.99155
GOBP_POSITIVE_REGULATION_OF_FATTY_ACID_TRANSPO	0.688206	0.868	0.99147
GOBP_INTRACELLULAR_PROTEIN_TRANSMEMBRANE_TRA	0.687548	0.927	0.99168
GOBP_REGULATION_OF_DNA_TEMPLATED_TRANSCRIPTIO	0.68715	0.93	0.99169
GOBP_POSITIVE_REGULATION_OF_DEFENSE_RESPONSE_	0.687063	0.893	0.99145
GOBP_METHIONINE_METABOLIC_PROCESS	0.686381	0.877	0.99167
GOBP_REGULATION_OF_TELOMERE_CAPPING	0.68627	0.902	0.99145
GOBP_REGULATION_OF_LIPID_CATABOLIC_PROCESS	0.68551	0.922	0.99172
GOBP_POSITIVE_REGULATION_OF_INTERLEUKIN_1_BETA	0.685503	0.922	0.99141
GOBP_NEGATIVE_REGULATION_OF_INTERLEUKIN_1_PRO	0.685493	0.899	0.9911
GOBP_LATE_ENDOSOME_TO_VACUOLE_TRANSPORT_VIA	0.685405	0.876	0.99087

GOBP_GASTRULATION_WITH_MOUTH_FORMING_SECOND	0.685365	0.885	0.99059
GOBP_TRANSLESION_SYNTHESIS	0.68529	0.882	0.99034
GOBP_MITOCHONDRIAL_FUSION	0.684717	0.892	0.99048
GOBP_MIRNA_METABOLIC_PROCESS	0.684488	0.881	0.99034
GOBP_ENTRAINMENT_OF_CIRCADIAN_CLOCK	0.684098	0.907	0.99033
GOBP_PROTEIN_DNA_COMPLEX_SUBUNIT_ORGANIZATIOI	0.683975	0.97	0.99011
GOBP_ENDOCYTIC_RECYCLING	0.683894	0.932	0.98987
GOBP_POSITIVE_REGULATION_OF_PEPTIDYL_THREONINE	0.683732	0.894	0.98968
GOBP_NEGATIVE_REGULATION_OF_RECEPTOR_SIGNALIN	0.682822	0.913	0.99007
GOBP_REGULATION_OF_MEGAKARYOCYTE_DIFFERENTIA	0.682772	0.884	0.9898
GOBP_REGULATION_OF_CILIUM_DEPENDENT_CELL_MOTI	0.682597	0.892	0.98962
GOBP_HISTONE_H2A_ACETYLATION	0.682389	0.895	0.98947
GOBP_CELL_CELL_JUNCTION_ORGANIZATION	0.682386	0.985	0.98917
GOBP_REGULATION_OF_CARBOHYDRATE_METABOLIC_PI	0.680833	0.976	0.99004
GOBP_PROTEIN_K11_LINKED_UBIQUITINATION	0.679646	0.905	0.99062
GOBP_RESPONSE_TO_COLD	0.6792	0.941	0.99064
GOBP_S_ADENOSYLMETHIONINE_METABOLIC_PROCESS	0.677677	0.891	0.99146
GOBP_REGULATION_OF_GLUCONEOGENESIS	0.67743	0.94	0.99135
GOBP_DNA_SYNTHESIS_INVOLVED_IN_DNA_REPAIR	0.676288	0.918	0.99185
GOBP_PROTEIN_LOCALIZATION_TO_PLASMA_MEMBRANE	0.675908	0.994	0.9918
GOBP_MEIOSIS_I_CELL_CYCLE_PROCESS	0.675799	0.97	0.99157
GOBP_VESICLE_TARGETING_ROUGH_ER_TO_CIS_GOLGI	0.675589	0.881	0.99141
GOBP_REGULATION_OF_EXTRACELLULAR_MATRIX_ASSE	0.675379	0.896	0.99126
GOBP_SUPPRESSION_OF_VIRAL_RELEASE_BY_HOST	0.674026	0.875	0.99195
GOBP_POSITIVE_REGULATION_OF_TYPE_I_INTERFERON_	0.673741	0.913	0.99184
GOBP_POSITIVE_REGULATION_OF_FAT_CELL_DIFFERENT	0.671868	0.95	0.99283
GOBP_PROTEIN_LOCALIZATION_TO_CHROMATIN	0.671449	0.92	0.99281
GOBP_ADENYLATE_CYCLASE_ACTIVATING_ADRENERGIC_	0.671184	0.894	0.99269
GOBP_POSITIVE_REGULATION_OF_DEPHOSPHORYLATION	0.6697	0.95	0.99343
GOBP_MACROMOLECULE_DEACYLATION	0.668887	0.982	0.99368
GOBP_POSITIVE_REGULATION_OF_CALCIUM_ION_IMPORT	0.666932	0.898	0.99472
GOBP_NEGATIVE_REGULATION_OF_MICROTUBULE_POLY	0.66625	0.925	0.99484
GOBP_REGULATION_OF_JUN_KINASE_ACTIVITY	0.663506	0.945	0.99636
GOBP_INTERMEMBRANE_LIPID_TRANSFER	0.663161	0.934	0.9963
GOBP_MONOSACCHARIDE_BIOSYNTHETIC_PROCESS	0.662814	0.959	0.99622
GOBP_GLUCOSE_METABOLIC_PROCESS	0.662707	0.991	0.99598
GOBP_RIBONUCLEOSIDE_METABOLIC_PROCESS	0.662479	0.911	0.99583
GOBP_POSITIVE_REGULATION_OF_RESPONSE_TO_ENDO	0.662273	0.926	0.99566
GOBP_CELLULAR_RESPONSE_TO_ESTRADIOL_STIMULUS	0.662232	0.926	0.99538
GOBP_MITOCHONDRION_ORGANIZATION	0.6607	0.998	0.99608
GOBP_REGULATION_OF_T_CELL_MEDIATED_CYTOTOXICI	0.660407	0.906	0.99596
GOBP_LYMPH_NODE_DEVELOPMENT	0.660392	0.879	0.99567
GOBP_CIRCADIAN_SLEEP_WAKE_CYCLE	0.659861	0.897	0.9957
GOBP_PROTEIN_DNA_COMPLEX_ASSEMBLY	0.659137	0.975	0.99587
GOBP_CELLULAR_COPPER_ION_HOMEOSTASIS	0.658644	0.911	0.99587
GOBP_TELOMERE_CAPPING	0.658213	0.928	0.99585
GOBP_REGULATION_OF_PROTEIN_TARGETING	0.657277	0.963	0.99615
GOBP_STRESS_GRANULE_ASSEMBLY	0.656341	0.92	0.99642
GOBP_RESPONSE_TO_TOPOLOGICALLY_INCORRECT_PR(	0.654777	0.98	0.9971
GOBP_SULFUR_AMINO_ACID_BIOSYNTHETIC_PROCESS	0.652364	0.911	0.99833
GOBP_POSITIVE_REGULATION_OF_NITRIC_OXIDE_SYNTH	0.652255	0.918	0.99808
GOBP_POSITIVE_REGULATION_OF_HISTONE_ACETYLATIC	0.651636	0.924	0.99815
GOBP_PHOSPHATIDYLINOSITOL_DEPHOSPHORYLATION	0.651498	0.937	0.99792

GOBP_HISTONE_UBIQUITINATION	0.651364	0.942	0.99768
GOBP_PURINE_RIBONUCLEOSIDE_METABOLIC_PROCESS	0.650971	0.899	0.9976
GOBP_CELLULAR_COMPONENT_DISASSEMBLY	0.650704	0.999	0.99746
GOBP_EMBRYONIC_CAMERA_TYPE_EYE_MORPHOGENES	0.64819	0.929	0.99864
GOBP_FATTY_ACID_BIOSYNTHETIC_PROCESS	0.647721	0.981	0.9986
GOBP_NEURAL_CREST_FORMATION	0.647691	0.911	0.99832
GOBP_REGULATION_OF_PROTEIN_EXIT_FROM_ENDOPLA	0.647451	0.903	0.99813
GOBP_CELLULAR_RESPONSE_TO_ESTROGEN_STIMULUS	0.646891	0.913	0.99815
GOBP_VESICLE_MEDIATED_TRANSPORT_TO_THE_PLASM	0.646692	0.988	0.99795
GOBP_REGULATION_OF_HETEROTYPIC_CELL_CELL_ADHE	0.646375	0.903	0.99782
GOBP_NUCLEOLUS_ORGANIZATION	0.646322	0.918	0.99754
GOBP_GMP_METABOLIC_PROCESS	0.646097	0.927	0.99736
GOBP_EXECUTION_PHASE_OF_APOPTOSIS	0.644686	0.97	0.99784
GOBP_EMBRYONIC_HEMOPOIESIS	0.643971	0.921	0.99796
GOBP_POSITIVE_REGULATION_OF_MUSCLE_HYPERTROP	0.642637	0.921	0.99843
GOBP_NEGATIVE_REGULATION_OF_TELOMERE_MAINTEN	0.642326	0.925	0.99828
GOBP_DNA_LIGATION	0.641753	0.906	0.9983
GOBP_INNER_EAR_RECEPTOR_CELL_DEVELOPMENT	0.64117	0.944	0.99832
GOBP_EXIT_FROM_MITOSIS	0.64052	0.937	0.99836
GOBP_PROTEIN_LOCALIZATION_TO_ENDOPLASMIC_RETI	0.640476	0.964	0.99807
GOBP_VITAMIN_TRANSPORT	0.640352	0.947	0.99783
GOBP_SERTOLI_CELL_DIFFERENTIATION	0.64005	0.918	0.99768
GOBP_COPII_COATED_VESICLE_BUDDING	0.640012	0.94	0.9974
GOBP_REGULATION_OF_VACUOLE_ORGANIZATION	0.638967	0.965	0.99764
GOBP_VESICLE_TARGETING	0.638463	0.958	0.99762
GOBP_HISTONE_LYSINE_DEMETHYLATION	0.636887	0.93	0.99815
GOBP_POSITIVE_REGULATION_OF_TELOMERE_CAPPING	0.635708	0.903	0.99847
GOBP_GLYCOSYLATION	0.635486	0.997	0.99828
GOBP_ORGANELLE_MEMBRANE_FUSION	0.634262	0.976	0.99862
GOBP_REGULATION_OF_SMOOTHENED_SIGNALING_PATH	0.63416	0.971	0.99837
GOBP_REGULATION_OF_INSULIN_SECRETION_INVOLVED_	0.633454	0.956	0.99843
GOBP_MAINTENANCE_OF_PROTEIN_LOCATION	0.632711	0.976	0.99852
GOBP_REGULATION_OF_INTRACELLULAR_ESTROGEN_RE	0.632482	0.945	0.99833
GOBP_INTERLEUKIN_13_PRODUCTION	0.628827	0.926	0.99988
GOBP_REGULATION_OF_SYNAPTIC_VESICLE_RECYCLING	0.627177	0.93	1
GOBP_MEMBRANE_DOCKING	0.626242	0.983	1
GOBP_RESPONSE_TO_STEROL	0.625063	0.943	1
GOBP_POSITIVE_REGULATION_OF_HISTONE_DEACETYLA	0.624457	0.943	1
GOBP_RIBONUCLEOTIDE_CATABOLIC_PROCESS	0.624453	0.957	1
GOBP_ARP2_3_COMPLEX_MEDIATED_ACTIN_NUCLEATION	0.624374	0.958	1
GOBP_NADH_DEHYDROGENASE_COMPLEX_ASSEMBLY	0.623828	0.96	1
GOBP_SYNAPSE_MATURATION	0.622319	0.933	1
GOBP_PURINE_NUCLEOTIDE_CATABOLIC_PROCESS	0.622131	0.959	1
GOBP_NUCLEOSIDE_BISPHOSPHATE_BIOSYNTHETIC_PRO	0.622114	0.977	1
GOBP_VESICLE_FUSION_TO_PLASMA_MEMBRANE	0.62207	0.935	0.99979
GOBP_DNA_STRAND_ELONGATION	0.620761	0.951	1
GOBP_ENDOPLASMIC_RETICULUM_UNFOLDED_PROTEIN_	0.620529	0.974	0.99988
GOBP_SYNAPTIC_VESICLE_MEMBRANE_ORGANIZATION	0.620423	0.947	0.99963
GOBP_POSITIVE_REGULATION_OF_MRNA_PROCESSING	0.619991	0.944	0.99952
GOBP_POSITIVE_REGULATION_OF_RNA_SPLICING	0.619536	0.96	0.99943
GOBP_PROGRAMMED_NECROTIC_CELL_DEATH	0.619133	0.959	0.99931
GOBP_PROTEIN_CONTAINING_COMPLEX_DISASSEMBLY	0.617122	0.997	0.9999
GOBP_HISTAMINE_TRANSPORT	0.616711	0.94	0.99977

GOBP_VESICLE_BUDDING_FROM_MEMBRANE	0.616496	0.972	0.99957
GOBP_NEGATIVE_REGULATION_OF_CYCLIN_DEPENDENT_	0.615124	0.969	0.99987
GOBP_NECROTIC_CELL_DEATH	0.61512	0.977	0.99957
GOBP_MITOCHONDRIAL_FISSION	0.614914	0.967	0.99935
GOBP_REGULATION_OF_EXIT_FROM_MITOSIS	0.614453	0.927	0.99925
GOBP_CELLULAR_CARBOHYDRATE_METABOLIC_PROCES	0.612894	1	0.99961
GOBP_PLACENTA_BLOOD_VESSEL_DEVELOPMENT	0.612808	0.947	0.99934
GOBP_MRNA_CIS_SPLICING_VIA_SPLICEOSOME	0.61123	0.949	0.99972
GOBP_DNA_REPLICATION_INDEPENDENT_CHROMATIN_O	0.611079	0.946	0.99947
GOBP_TETRAPYRROLE_BIOSYNTHETIC_PROCESS	0.610183	0.962	0.99953
GOBP_REGULATION_OF_NECROTIC_CELL_DEATH	0.609315	0.958	0.99959
GOBP_PROTEIN_DNA_COMPLEX_DISASSEMBLY	0.609036	0.934	0.99941
GOBP_ACTIN_FILAMENT_BASED_TRANSPORT	0.608804	0.953	0.9992
GOBP_CLATHRIN_COAT_ASSEMBLY	0.607342	0.946	0.9995
GOBP_T_CELL_RECEPTOR_SIGNALING_PATHWAY	0.605913	0.987	0.99979
GOBP_PHASIC_SMOOTH_MUSCLE_CONTRACTION	0.604803	0.944	0.99992
GOBP_HYDROGEN_PEROXIDE_CATABOLIC_PROCESS	0.603893	0.947	0.99998
GOBP_REGULATION_OF_RUFFLE_ASSEMBLY	0.603257	0.962	0.99994
GOBP_GOLGI_TO_VACUOLE_TRANSPORT	0.603076	0.956	0.9997
GOBP_BILE_ACID_METABOLIC_PROCESS	0.597881	0.965	1
GOBP_RESPONSE_TO_CHOLESTEROL	0.596231	0.948	1
GOBP_ESTABLISHMENT_OF_ENDOTHELIAL_BARRIER	0.594145	0.984	1
GOBP_DNA_CATABOLIC_PROCESS	0.593774	0.958	1
GOBP_PURINE_NUCLEOSIDE_MONOPHOSPHATE_METABO	0.592697	0.974	1
GOBP_DNA_UNWINDING_INVOLVED_IN_DNA_REPLICATIO	0.591658	0.96	1
GOBP_MUSCLE_HYPERTROPHY	0.591263	0.983	1
GOBP_PROTEIN_TARGETING_TO_MITOCHONDRION	0.591251	0.995	1
GOBP_IMP_METABOLIC_PROCESS	0.589748	0.954	1
GOBP_REGULATION_OF_BICELLULAR_TIGHT_JUNCTION_	0.588415	0.958	1
GOBP_REGULATION_OF_INCLUSION_BODY_ASSEMBLY	0.58725	0.951	1
GOBP_NEGATIVE_REGULATION_OF_PEPTIDYL_TYROSINE	0.587212	0.978	1
GOBP_ANOIKIS	0.586475	0.971	1
GOBP_RETROGRADE_VESICLE_MEDIATED_TRANSPORT_	0.585346	0.987	1
GOBP_STRIATED_MUSCLE_CELL_APOPTOTIC_PROCESS	0.585235	0.985	1
GOBP_ENDOSOME_ORGANIZATION	0.585037	0.99	1
GOBP_CELLULAR_RESPONSE_TO_UNFOLDED_PROTEIN	0.584956	0.994	1
GOBP_DEFINITIVE_HEMOPOIESIS	0.583897	0.962	1
GOBP_NEGATIVE_REGULATION_OF_TOR_SIGNALING	0.582753	0.972	1
GOBP_CELLULAR_RESPONSE_TO_GROWTH_HORMONE_S	0.582718	0.958	1
GOBP_MITOCHONDRIAL_CALCIUM_ION_TRANSMEMBRANE	0.582078	0.959	1
GOBP_DNA_METHYLATION_DEPENDENT_HETEROCHROM	0.581992	0.96	1
GOBP_PROTEIN_DEMETHYLATION	0.5816	0.962	1
GOBP_CELLULAR_RESPONSE_TO_TOPOLOGICALLY_INCO	0.580458	0.994	1
GOBP_NEGATIVE_REGULATION_OF_MUSCLE_CELL_APOP	0.579478	0.972	1
GOBP_REGULATION_OF_TRIGLYCERIDE_METABOLIC_PRO	0.578929	0.98	0.99989
GOBP_PROTEIN_HETEROOLIGOMERIZATION	0.578781	0.976	0.99963
GOBP_3_UTR_MEDIATED_MRNA_STABILIZATION	0.578703	0.957	0.99935
GOBP_EXOCYTIC_PROCESS	0.578258	0.987	0.99918
GOBP_PYRIMIDINE_NUCLEOSIDE_MONOPHOSPHATE_BIOS	0.575961	0.956	0.99953
GOBP_AUTOPHAGOSOME_ORGANIZATION	0.575562	0.991	0.99934
GOBP_PROTEIN_EXIT_FROM_ENDOPLASMIC_RETICULUM	0.574985	0.982	0.99921
GOBP_PURINE_CONTAINING_COMPOUND_CATABOLIC_PR	0.574075	0.983	0.99916
GOBP_UBIQUINONE_METABOLIC_PROCESS	0.574021	0.958	0.99887

GOBP_ORGANIC_CATION_TRANSPORT	0.573714	0.975	0.99866
GOBP_GLYCOSYL_COMPOUND_METABOLIC_PROCESS	0.570739	0.99	0.99913
GOBP_ENERGY_DERIVATION_BY_OXIDATION_OF_ORGAN	0.569698	1	0.99911
GOBP_REGULATION_OF_CARBOHYDRATE_CATABOLIC_PF	0.568014	0.993	0.99924
GOBP_SEX_DETERMINATION	0.567387	0.963	0.99911
GOBP_AMYLOID_BETA_FORMATION	0.566737	0.988	0.99897
GOBP_CELLULAR_METABOLIC_COMPOUND_SALVAGE	0.565902	0.975	0.99888
GOBP_VESICLE_TARGETING_TO_FROM_OR_WITHIN_GOL	0.564252	0.961	0.99899
GOBP_UBIQUITIN_DEPENDENT_PROTEIN_CATABOLIC_PR	0.563817	0.982	0.99881
GOBP_CHROMATIN_REMODELING	0.563723	0.999	0.99854
GOBP_LIPOSACCHARIDE_METABOLIC_PROCESS	0.560386	0.997	0.99902
GOBP_GOLGI_TO_PLASMA_MEMBRANE_PROTEIN_TRANS	0.557439	0.991	0.99938
GOBP_ANDROGEN_RECEPTOR_SIGNALING_PATHWAY	0.557242	0.984	0.99913
GOBP_NEGATIVE_REGULATION_OF_CALCIUM_ION_TRANS	0.555087	0.966	0.99931
GOBP_NEGATIVE_REGULATION_OF_TRANSCRIPTION_RE	0.554376	0.966	0.99917
GOBP_REGULATION_OF_B_CELL_RECEPTOR_SIGNALING_	0.552882	0.967	0.9992
GOBP_NUCLEAR_MEMBRANE_REASSEMBLY	0.552563	0.972	0.99897
GOBP_RESPONSE_TO_FOOD	0.55029	0.983	0.99912
GOBP_CHROMATIN_ASSEMBLY_OR_DISASSEMBLY	0.547278	1	0.99944
GOBP_REGULATION_OF_GOLGI_ORGANIZATION	0.546924	0.973	0.99921
GOBP_DNA_CATABOLIC_PROCESS_ENDONUCLEOLYTIC	0.546033	0.984	0.99907
GOBP_GLYCOLIPID_BIOSYNTHETIC_PROCESS	0.542328	0.995	0.99945
GOBP_REGULATION_OF_GLYCOLYTIC_PROCESS	0.54119	0.992	0.99936
GOBP_SPERM_CAPACITATION	0.541117	0.971	0.99908
GOBP_MUSCLE_CELL_APOPTOTIC_PROCESS	0.540672	0.994	0.99886
GOBP_RIBONUCLEOSIDE_MONOPHOSPHATE_BIOSYNTHE	0.539639	0.983	0.99876
GOBP_COTRANSLATIONAL_PROTEIN_TARGETING_TO_ME	0.539574	0.972	0.99847
GOBP_DNA_STRAND_ELONGATION_INVOLVED_IN_DNA_R	0.535147	0.97	0.99892
GOBP_MISMATCH_REPAIR	0.532882	0.984	0.99899
GOBP_COPPER_ION_HOMEOSTASIS	0.530357	0.991	0.9991
GOBP_B_CELL_RECEPTOR_SIGNALING_PATHWAY	0.530329	0.999	0.9988
GOBP_MAINTENANCE_OF_PROTEIN_LOCATION_IN_CELL	0.529322	1	0.99865
GOBP_SYNAPTONEMAL_COMPLEX_ORGANIZATION	0.526595	0.982	0.99876
GOBP_ENDOSOME_TRANSPORT_VIA_MULTIVESICULAR_B	0.52609	0.987	0.99853
GOBP_VESICLE_DOCKING_INVOLVED_IN_EXOCYTOSIS	0.525711	0.99	0.99829
GOBP_VIRION_ASSEMBLY	0.521483	0.989	0.99858
GOBP_NUCLEOSOME_ORGANIZATION	0.512602	1	0.99938
GOBP_INOSITOL_PHOSPHATE_CATABOLIC_PROCESS	0.511474	0.982	0.99921
GOBP_CELLULAR_RESPIRATION	0.510679	1	0.999
GOBP_HISTONE_H4_K5_ACETYLATION	0.508922	0.989	0.9989
GOBP_POSITIVE_REGULATION_OF_CELL_CYCLE_G2_M_P	0.505171	0.987	0.99902
GOBP_POSITIVE_REGULATION_OF_AMYLOID_BETA_FORM	0.499725	0.984	0.99924
GOBP_POSITIVE_REGULATION_OF_PHOSPHOPROTEIN_P	0.497396	0.99	0.99916
GOBP_REGULATION_OF_EARLY_ENDOSOME_TO_LATE_E	0.495619	0.986	0.99902
GOBP_MITOTIC_SPINDLE_ASSEMBLY	0.487749	0.999	0.99939
GOBP_NUCLEAR_ENVELOPE_ORGANIZATION	0.452041	1	1
GOBP_NUCLEAR_MEMBRANE_ORGANIZATION	0.451716	0.999	1
GOBP_FACULTATIVE_HETEROCHROMATIN_ASSEMBLY	0.439009	0.998	1
GOBP_HISTONE_H3_DEACETYLATION	0.432781	0.999	1
GOBP_CYTOCHROME_COMPLEX_ASSEMBLY	0.429997	0.998	1
GOBP_MITOCHONDRIAL_RESPIRATORY_CHAIN_COMPLEX	0.42947	0.999	1
GOBP_VIRAL_RELEASE_FROM_HOST_CELL	0.429239	0.999	1
GOBP_RIBONUCLEOSIDE_MONOPHOSPHATE_METABOLIC	0.428865	1	0.99976

GOBP_RESPIRATORY_ELECTRON_TRANSPORT_CHAIN	0.388223	1	1
GOBP_NEGATIVE_REGULATION_OF_RNA_SPLICING	0.361953	1	0.99992
GOBP_NEGATIVE_REGULATION_OF_MRNA_PROCESSING	-0.37931	1	0.99561
GOBP_POSITIVE_REGULATION_OF_VACUOLE_ORGANIZAT	-0.46614	1	0.99681
GOBP_RESPONSE_TO_MISFOLDED_PROTEIN	-0.47131	1	0.99882
GOBP_NEGATIVE_REGULATION_OF_MRNA_SPLICING_VIA_	-0.50545	1	0.99924
GOBP_COENZYME_A_METABOLIC_PROCESS	-0.51254	0.995	1
GOBP_ATP_SYNTHESIS_COUPLED_ELECTRON_TRANSPOI	-0.51822	1	1
GOBP_MITOTIC_CYTOKINETIC_PROCESS	-0.52994	1	1
GOBP_RESPONSE_TO_MITOCHONDRIAL_DEPOLARISATIO	-0.53024	0.995	1
GOBP_NEGATIVE_REGULATION_OF_UBIQUITIN_PROTEIN_	-0.53163	0.995	1
GOBP_REGULATION_OF_DNA_STRAND_ELONGATION	-0.54189	0.986	1
GOBP_LATE_ENDOSOME_TO_VACUOLE_TRANSPORT	-0.55439	0.979	1
GOBP_REGULATION_OF_TRANSCRIPTION_REGULATORY_	-0.56096	1	1
GOBP_PHOSPHATIDIC_ACID_METABOLIC_PROCESS	-0.56436	1	1
GOBP_RESPIRATORY_CHAIN_COMPLEX_IV_ASSEMBLY	-0.56948	0.982	1
GOBP_REGULATION_OF_AUTOPHAGOSOME_MATURATION	-0.57304	0.974	1
GOBP_AUTOPHAGOSOME_MATURATION	-0.57659	1	1
GOBP_NEGATIVE_REGULATION_OF_CELL_MIGRATION_IN\	-0.58343	0.95	1
GOBP_REGULATION_OF_MITOTIC_SPINDLE_ASSEMBLY	-0.58494	0.979	1
GOBP_MITOPHAGY	-0.59063	1	1
GOBP_ANAPHASE_PROMOTING_COMPLEX_DEPENDENT_(	-0.59153	0.972	1
GOBP_VIRAL_BUDDING	-0.5931	0.988	1
GOBP_MIDBODY_ABSCISSION	-0.59965	0.955	1
GOBP_REGULATION_OF_CHROMATIN_ORGANIZATION	-0.60735	0.984	1
GOBP_NEGATIVE_REGULATION_OF_TORC1_SIGNALING	-0.60966	0.985	1
GOBP_NUCLEOLAR_LARGE_RRNA_TRANSCRIPTION_BY_R	-0.61309	0.931	1
GOBP_LOCOMOTORY_EXPLORATION_BEHAVIOR	-0.61469	0.957	1
GOBP_POSITIVE_REGULATION_OF_TRANSCRIPTION_REG	-0.61717	0.98	1
GOBP_HISTONE_DEACETYLATION	-0.62895	1	1
GOBP_MULTIVESICULAR_BODY_ORGANIZATION	-0.63316	0.965	1
GOBP_PROTEIN_N_LINKED_GLYCOSYLATION	-0.64483	0.989	1
GOBP_REGULATION_OF_CHROMATIN_ASSEMBLY	-0.64807	0.943	1
GOBP_N_GLYCAN_PROCESSING	-0.6644	0.939	1
GOBP_AMYLOID_PRECURSOR_PROTEIN_CATABOLIC_PRC	-0.66497	0.978	1
GOBP_MITOCHONDRIAL_CYTOCHROME_C_OXIDASE_ASSI	-0.6653	0.946	1
GOBP_PROTEIN_QUALITY_CONTROL_FOR_MISFOLDED_O	-0.67432	0.936	1
GOBP_OXIDATIVE_PHOSPHORYLATION	-0.6789	1	1
GOBP_POSITIVE_REGULATION_OF_REACTIVE_OXYGEN_S	-0.68064	0.931	1
GOBP_RNA_DECAPPING	-0.68082	0.932	1
GOBP_RESPONSE_TO_ANGIOTENSIN	-0.68441	0.94	1
GOBP_REGULATION_OF_CELL_COMMUNICATION_BY_ELE(	-0.68893	0.893	1
GOBP_NUCLEOSIDE_MONOPHOSPHATE_BIOSYNTHETIC_F	-0.69092	0.956	1
GOBP_ENDOPLASMIC_RETICULUM_CALCIUM_ION_HOMEC	-0.69165	0.939	1
GOBP_REGULATION_OF_ENDOPLASMIC_RETICULUM_UNF	-0.70443	0.932	1
GOBP_PURINE_DEOXYRIBONUCLEOTIDE_METABOLIC_PR(	-0.70795	0.873	1
GOBP_PURINE_NUCLEOSIDE_METABOLIC_PROCESS	-0.70819	0.878	1
GOBP_NEUROTROPHIN_TRK_RECEPTOR_SIGNALING_PAT	-0.71307	0.944	1
GOBP_AMINO_ACID_BETAINE_METABOLIC_PROCESS	-0.71413	0.857	1
GOBP_POSITIVE_REGULATION_OF_PROTEIN_DEPHOSPH(	-0.71645	0.947	1
GOBP_NUCLEOTIDE_SUGAR_BIOSYNTHETIC_PROCESS	-0.71715	0.839	1
GOBP_NEGATIVE_REGULATION_OF_CATION_CHANNEL_A(	-0.71721	0.943	1
GOBP_RIBOSOMAL_SMALL_SUBUNIT_ASSEMBLY	-0.71818	0.863	1

GOBP_NUCLEOSIDE_MONOPHOSPHATE_METABOLIC_PRO	-0.72021	0.978	1
GOBP_AMYLOID_PRECURSOR_PROTEIN_METABOLIC_PRO	-0.72311	0.974	1
GOBP_REGULATION_OF_MITOCHONDRIAL_FISSION	-0.72343	0.932	1
GOBP_PLASMA_MEMBRANE_REPAIR	-0.72456	0.898	1
GOBP_ASYMMETRIC_CELL_DIVISION	-0.72764	0.87	1
GOBP_INTRA_GOLGI_VESICLE_MEDIATED_TRANSPORT	-0.72822	0.949	1
GOBP_REGULATION_OF_CLATHRIN_DEPENDENT_ENDOCY	-0.72856	0.854	1
GOBP_SYNAPTIC_VESICLE_PRIMING	-0.72866	0.864	1
GOBP_PROTEIN_REFOLDING	-0.72957	0.889	1
GOBP_SMOOTH_MUSCLE_CELL_APOPTOTIC_PROCESS	-0.73036	0.889	1
GOBP_PEPTIDYL_SERINE_DEPHOSPHORYLATION	-0.73063	0.88	1
GOBP_ELECTRON_TRANSPORT_CHAIN	-0.7334	1	1
GOBP_ACTIVATION_OF_NF_KAPPAB_INDUCING_KINASE_A	-0.73369	0.87	1
GOBP_GLYCOSYL_COMPOUND_CATABOLIC_PROCESS	-0.73433	0.863	1
GOBP_PLATELET_MORPHOGENESIS	-0.7346	0.861	1
GOBP_POSITIVE_REGULATION_OF_STEM_CELL_DIFFEREN	-0.7433	0.824	1
GOBP_MAINTENANCE_OF_PROTEIN_LOCALIZATION_IN_OF	-0.75112	0.911	1
GOBP_ORGANELLE_DISASSEMBLY	-0.75244	1	1
GOBP_REGULATION_OF_PROTON_TRANSPORT	-0.75362	0.845	1
GOBP_PYRIMIDINE_NUCLEOSIDE_MONOPHOSPHATE_MET	-0.75994	0.838	1
GOBP_REGULATION_OF_VIRAL_TRANSCRIPTION	-0.76004	0.836	1
GOBP_GANGLIOSIDE_METABOLIC_PROCESS	-0.76216	0.848	1
GOBP_NEGATIVE_REGULATION_OF_AMINE_TRANSPORT	-0.76293	0.797	1
GOBP_REGULATION_OF_VESICLE_FUSION	-0.76738	0.803	1
GOBP_ACTIVATION_OF_CYSTEINE_TYPE_ENDOPEPTIDAS	-0.76752	0.805	1
GOBP_PROTEIN_LOCALIZATION_TO_PHAGOPHORE_ASSE	-0.76778	0.763	1
GOBP_NUCLEOSIDE_METABOLIC_PROCESS	-0.76923	0.868	1
GOBP_RESPONSE_TO_MINERALOCORTICOID	-0.76923	0.839	1
GOBP_COPII_COATED_VESICLE_CARGO_LOADING	-0.77143	0.775	1
GOBP_NEGATIVE_REGULATION_OF_INTERLEUKIN_8_PRO	-0.77144	0.802	1
GOBP_GLYCOSPHINGOLIPID_METABOLIC_PROCESS	-0.77201	0.898	1
GOBP_NEUTROPHIL_ACTIVATION_INVOLVED_IN_IMMUNE_	-0.77695	0.801	1
GOBP_REGULATION_OF_ENDOCRINE_PROCESS	-0.77731	0.823	1
GOBP_POSITIVE_REGULATION_OF_MUSCLE_CELL_APOPT	-0.77921	0.817	1
GOBP_POSITIVE_REGULATION_OF_GRANULOCYTE_CHEM	-0.7859	0.774	1
GOBP_NUCLEOSOME_ASSEMBLY	-0.78603	0.871	1
GOBP_POSITIVE_REGULATION_OF_JUN_KINASE_ACTIVITY	-0.78803	0.86	1
GOBP_AEROBIC_RESPIRATION	-0.78885	0.95	1
GOBP_ER_NUCLEUS_SIGNALING_PATHWAY	-0.7937	0.856	1
GOBP_RESPONSE_TO_GROWTH_HORMONE	-0.79987	0.838	1
GOBP_REGULATION_OF_ANTIGEN_RECEPTOR_MEDIATED	-0.81112	0.882	0.99012
GOBP_NEGATIVE_REGULATION_OF_RECEPTOR_MEDIATE	-0.81127	0.764	0.99258
GOBP_EXTRACELLULAR_TRANSPORT	-0.81301	0.825	0.99197
GOBP_DEOXYRIBOSE_PHOSPHATE_METABOLIC_PROCES	-0.81446	0.83	0.9919
GOBP_SULFATE_TRANSPORT	-0.81573	0.743	0.99206
GOBP_POLYAMINE_BIOSYNTHETIC_PROCESS	-0.81644	0.774	0.99349
GOBP_RESPONSE_TO_INTERFERON_BETA	-0.81976	0.723	0.98962
GOBP_ARF_PROTEIN_SIGNAL_TRANSDUCTION	-0.82231	0.729	0.98717
GOBP_LABYRINTHINE_LAYER_BLOOD_VESSEL_DEVELOPM	-0.82628	0.684	0.98177
GOBP_REGULATORY_T_CELL_DIFFERENTIATION	-0.82656	0.747	0.98394
GOBP_REGULATION_OF_CHROMATIN_ASSEMBLY_OR_DIS	-0.83005	0.765	0.97954
GOBP_PROTEIN_ADP_RIBOSYLATION	-0.83233	0.824	0.97749
GOBP_CELLULAR_RESPONSE_TO_DEXAMETHASONE_STI	-0.83503	0.747	0.97449

GOBP_POSITIVE_REGULATION_OF_AXON_EXTENSION	-0.83539	0.769	0.97639
GOBP_PYRIMIDINE_DEOXYRIBONUCLEOTIDE_METABOLIC	-0.83634	0.7	0.97706
GOBP_NUCLEOBASE_BIOSYNTHETIC_PROCESS	-0.83999	0.641	0.97201
GOBP_NEGATIVE_REGULATION_OF_PROTEIN_ACETYLATI	-0.84037	0.698	0.97383
GOBP_AUTOPHAGY_OF_MITOCHONDRION	-0.84416	0.768	0.96807
GOBP_INOSITOL_PHOSPHATE_METABOLIC_PROCESS	-0.8458	0.761	0.9671
GOBP_CHONDROCYTE_DIFFERENTIATION_INVOLVED_IN_	-0.84602	0.637	0.96941
GOBP_REGULATION_OF_PHOSPHOLIPID_METABOLIC_PRO	-0.84896	0.735	0.96543
GOBP_CELLULAR_RESPONSE_TO_COPPER_ION	-0.84931	0.685	0.96739
GOBP_ACTIN_NUCLEATION	-0.84974	0.798	0.96927
GOBP_NEGATIVE_REGULATION_OF_BLOOD_CIRCULATION	-0.85009	0.717	0.97125
GOBP_REGULATION_OF_NEURONAL_SYNAPTIC_PLASTICI	-0.85574	0.763	0.96088
GOBP_EMBRYONIC_BRAIN_DEVELOPMENT	-0.85692	0.689	0.96083
GOBP_VITAMIN_BIOSYNTHETIC_PROCESS	-0.86018	0.678	0.95587
GOBP_PHYSIOLOGICAL_CARDIAC_MUSCLE_HYPERTROPH	-0.86099	0.665	0.95676
GOBP_REGULATION_OF_TRIGLYCERIDE_BIOSYNTHETIC_	-0.86254	0.672	0.95592
GOBP_ARACHIDONIC_ACID_SECRETION	-0.86408	0.724	0.95487
GOBP_POSITIVE_REGULATION_OF_EPITHELIAL_CELL_APO	-0.86652	0.68	0.95163
GOBP_BRANCHED_CHAIN_AMINO_ACID_METABOLIC_PRO	-0.86728	0.665	0.95266
GOBP_REGULATION_OF_PROTEIN_DEPHOSPHORYLATION	-0.8688	0.867	0.95183
GOBP_POSITIVE_REGULATION_OF_INSULIN_SECRETION_	-0.86894	0.645	0.95433
GOBP_ENDOTHELIAL_CELL_DEVELOPMENT	-0.87597	0.744	0.93979
GOBP_RESPONSE_TO_TYPE_I_INTERFERON	-0.87636	0.729	0.9415
GOBP_NEGATIVE_REGULATION_OF_DNA_TEMPLATED_TR	-0.87843	0.626	0.93909
GOBP_BILE_ACID_BIOSYNTHETIC_PROCESS	-0.88084	0.695	0.93562
GOBP_MUSCLE_ADAPTATION	-0.88128	0.78	0.93748
GOBP_EPITHELIAL_STRUCTURE_MAINTENANCE	-0.88167	0.636	0.93935
GOBP_REGULATION_OF_LIPOPROTEIN_PARTICLE_CLEAR	-0.88237	0.668	0.9404
GOBP_NEGATIVE_REGULATION_OF_PEPTIDYL_LYSINE_AC	-0.88848	0.618	0.92749
GOBP_BRANCHING_INVOLVED_IN_BLOOD_VESSEL_MORP	-0.88962	0.655	0.92718
GOBP_GLUCOSE_CATABOLIC_PROCESS	-0.89526	0.593	0.91445
GOBP_APOPTOTIC_DNA_FRAGMENTATION	-0.89595	0.606	0.91538
GOBP_NEGATIVE_REGULATION_OF_T_CELL_RECEPTOR_	-0.89668	0.601	0.91627
GOBP_POLYAMINE_METABOLIC_PROCESS	-0.89867	0.589	0.91373
GOBP_REGULATION_OF_RENAL_SYSTEM_PROCESS	-0.89926	0.577	0.91508
GOBP_NEGATIVE_REGULATION_OF_VIRAL_TRANSCRIPTIO	-0.90062	0.598	0.91414
GOBP_VIRAL_PROTEIN_PROCESSING	-0.90097	0.653	0.91608
GOBP_REGULATION_OF_SMOOTH_MUSCLE_CELL_DIFFER	-0.90313	0.571	0.91288
GOBP_REGULATION_OF_DEPHOSPHORYLATION	-0.90344	0.811	0.91494
GOBP_REGULATION_OF_NATURAL_KILLER_CELL_ACTIVAT	-0.90583	0.591	0.91121
GOBP_AUTOPHAGY_OF_NUCLEUS	-0.90623	0.629	0.91303
GOBP_REGULATION_OF_INSULIN_LIKE_GROWTH_FACTOR	-0.90773	0.566	0.91158
GOBP_RENAL_SODIUM_EXCRETION	-0.90774	0.579	0.91447
GOBP_GASTRIC_ACID_SECRETION	-0.9091	0.617	0.91345
GOBP_GLUTAMINE_FAMILY_AMINO_ACID_CATABOLIC_PRO	-0.91032	0.614	0.91266
GOBP_REGULATION_OF_CELLULAR_CARBOHYDRATE_ME	-0.91096	0.842	0.91389
GOBP_MACROPHAGE_CYTOKINE_PRODUCTION	-0.91161	0.559	0.91489
GOBP_POST_EMBRYONIC_ANIMAL_ORGAN_DEVELOPMEN	-0.91234	0.56	0.91584
GOBP_TRIGLYCERIDE_CATABOLIC_PROCESS	-0.91396	0.613	0.91419
GOBP_CELLULAR_RESPONSE_TO_CAMP	-0.91698	0.64	0.90822
GOBP_PROTEIN_LOCALIZATION_TO_CELL_CELL_JUNCTIO	-0.91863	0.59	0.90647
GOBP_BASEMENT_MEMBRANE_ORGANIZATION	-0.91919	0.61	0.90771
GOBP_NEURON_PROJECTION_ARBORIZATION	-0.923	0.622	0.8994

GOBP_POSITIVE_REGULATION_OF_MRNA_SPLICING_VIA_	-0.92491	0.58	0.89648
GOBP_VITAMIN_TRANSMEMBRANE_TRANSPORT	-0.92555	0.528	0.89766
GOBP_PYRIMIDINE_NUCLEOBASE_METABOLIC_PROCESS	-0.92787	0.51	0.89366
GOBP_MONOSACCHARIDE_CATABOLIC_PROCESS	-0.92811	0.638	0.89596
GOBP_MONOCARBOXYLIC_ACID_BIOSYNTHETIC_PROCES	-0.92913	0.85	0.89596
GOBP_NUCLEOSIDE_DIPHOSPHATE_METABOLIC_PROCES	-0.93487	0.609	0.88145
GOBP_REACTIVE_OXYGEN_SPECIES_BIOSYNTHETIC_PRC	-0.93828	0.527	0.87393
GOBP_ATP_METABOLIC_PROCESS	-0.93916	0.75	0.87419
GOBP_THIOESTER_BIOSYNTHETIC_PROCESS	-0.94067	0.584	0.87255
GOBP_RESPONSE_TO_DEXAMETHASONE	-0.94257	0.521	0.86982
GOBP_NEGATIVE_REGULATION_OF_NUCLEOTIDE_METAB	-0.94259	0.549	0.87277
GOBP_REGULATION_OF_T_CELL_RECEPTOR_SIGNALING_	-0.94338	0.559	0.87337
GOBP_RESPONSE_TO_AMPHETAMINE	-0.94515	0.567	0.87107
GOBP_REGULATION_OF_CHOLESTEROL_BIOSYNTHETIC_I	-0.9456	0.51	0.87268
GOBP_MICROVILLUS_ORGANIZATION	-0.94594	0.593	0.8746
GOBP_PLASMA_MEMBRANE_ORGANIZATION	-0.94672	0.741	0.87512
GOBP_REGULATION_OF_PHOSPHOPROTEIN_PHOSPHATA	-0.94738	0.616	0.87596
GOBP_PROTEIN_TARGETING_TO_PEROXISOME	-0.94765	0.567	0.87819
GOBP_POSITIVE_REGULATION_OF_MITOCHONDRIAL_FISS	-0.94782	0.538	0.88075
GOBP_RETINOIC_ACID_RECEPTOR_SIGNALING_PATHWAY	-0.94877	0.56	0.88088
GOBP_POSITIVE_REGULATION_OF_HORMONE_SECRETIO	-0.95446	0.612	0.86558
GOBP_CARBOHYDRATE_CATABOLIC_PROCESS	-0.95568	0.667	0.86484
GOBP_NUCLEOSIDE_CATABOLIC_PROCESS	-0.95817	0.495	0.86022
GOBP_SEQUESTERING_OF_TRIGLYCERIDE	-0.96065	0.569	0.85542
GOBP_RESPONSE_TO_INSULIN	-0.96142	0.5	0.85597
GOBP_SPLEEN_DEVELOPMENT	-0.96256	0.503	0.85534
GOBP_NUCLEOBASE_CONTAINING_SMALL_MOLECULE_CA	-0.96265	0.526	0.85812
GOBP_INTRACELLULAR_LIPID_TRANSPORT	-0.96283	0.521	0.86074
GOBP_NEGATIVE_REGULATION_OF_SMALL_MOLECULE_M	-0.96313	0.456	0.86287
GOBP_NEGATIVE_REGULATION_OF_ANTIGEN_RECEPTOR	-0.96395	0.476	0.86333
GOBP_CAMERA_TYPE_EYE_PHOTORECEPTOR_CELL_DIFF	-0.96421	0.538	0.86558
GOBP_REGULATION_OF_CAMP_DEPENDENT_PROTEIN_KI	-0.96698	0.508	0.85967
GOBP_NEGATIVE_REGULATION_OF_CALCIUM_MEDIATED_	-0.96703	0.481	0.86271
GOBP_REGULATION_OF_PHOSPHATASE_ACTIVITY	-0.9679	0.487	0.86303
GOBP_RIBONUCLEOSIDE_DIPHOSPHATE_METABOLIC_PR(	-0.96838	0.512	0.86452
GOBP_ZINC_ION_HOMEOSTASIS	-0.97224	0.548	0.85525
GOBP_SULFATION	-0.97663	0.468	0.84403
GOBP_ADRENERGIC_RECEPTOR_SIGNALING_PATHWAY	-0.97936	0.48	0.83808
GOBP_HEART_FIELD_SPECIFICATION	-0.98218	0.475	0.83178
GOBP_RENAL_TUBULAR_SECRETION	-0.98314	0.483	0.83174
GOBP_REGULATION_OF_AMINO_ACID_TRANSPORT	-0.98424	0.453	0.83129
GOBP_OVULATION	-0.98516	0.485	0.83162
GOBP_GLYCEROLIPID_CATABOLIC_PROCESS	-0.98563	0.456	0.83345
GOBP_CARBOHYDRATE_PHOSPHORYLATION	-0.98599	0.448	0.83546
GOBP_REGULATION_OF_EXCRETION	-0.98607	0.441	0.83848
GOBP_EMBRYONIC_CAMERA_TYPE_EYE_DEVELOPMENT	-0.98615	0.478	0.8415
GOBP_NEGATIVE_REGULATION_OF_GLUCONEOGENESIS	-0.98632	0.471	0.84421
GOBP_RESPONSE_TO_MONOSACCHARIDE	-0.98686	0.591	0.84589
GOBP_ARGININE_METABOLIC_PROCESS	-0.98797	0.442	0.8455
GOBP_REGULATION_OF_GENERATION_OF_PRECURSOR_	-0.98882	0.488	0.84581
GOBP_ORGANOPHOSPHATE_CATABOLIC_PROCESS	-0.99123	0.567	0.84064
GOBP_POSITIVE_REGULATION_OF_GLYCOPROTEIN_META	-0.99589	0.476	0.82832
GOBP_MITOCHONDRIAL_ELECTRON_TRANSPORT_NADH_	-1.00022	0.449	0.81673

GOBP_NEGATIVE_REGULATION_OF_ACTIN_FILAMENT_PO	-1.00208	0.436	0.81386
GOBP_STEROID_HORMONE_BIOSYNTHETIC_PROCESS	-1.00266	0.41	0.81516
GOBP_RESPONSE_TO_LEPTIN	-1.00302	0.457	0.81716
GOBP_DEOXYRIBONUCLEOSIDE_MONOPHOSPHATE_MET/	-1.00347	0.435	0.81903
GOBP_POSITIVE_REGULATION_OF_ANTIGEN_RECEPTOR_	-1.00392	0.43	0.82103
GOBP_CHROMATIN_DISASSEMBLY	-1.00668	0.411	0.81503
GOBP_GLANDULAR_EPITHELIAL_CELL_DIFFERENTIATION	-1.0122	0.422	0.79981
GOBP_CELLULAR_RESPONSE_TO_CORTICOSTEROID_STI	-1.01494	0.462	0.79391
GOBP_NEGATIVE_REGULATION_OF_ACTIN_FILAMENT_DE	-1.01549	0.397	0.79522
GOBP_CELL_DEATH_IN_RESPONSE_TO_HYDROGEN_PER(	-1.01865	0.407	0.78814
GOBP_BRANCHED_CHAIN_AMINO_ACID_CATABOLIC_PRO(	-1.01869	0.417	0.79134
GOBP_ORGANIC_ACID_BIOSYNTHETIC_PROCESS	-1.01895	0	0.79388
GOBP_REGULATION_OF_MUSCLE_HYPERTROPHY	-1.02123	0.353	0.78977
GOBP_SERINE_FAMILY_AMINO_ACID_BIOSYNTHETIC_PRO	-1.02146	0.406	0.79239
GOBP_REGULATION_OF_ATP_METABOLIC_PROCESS	-1.02261	0.479	0.79215
GOBP_REGULATION_OF_MUSCLE_ADAPTATION	-1.02267	0.413	0.79535
GOBP_STRIATUM_DEVELOPMENT	-1.03257	0.409	0.76493
GOBP_MALE_MEIOSIS_I	-1.03323	0.422	0.76599
GOBP_INTRACELLULAR_STEROL_TRANSPORT	-1.03426	0.388	0.76569
GOBP_BROWN_FAT_CELL_DIFFERENTIATION	-1.03484	0.391	0.76705
GOBP_NOTCH_SIGNALING_PATHWAY	-1.03777	0.357	0.76042
GOBP_SARCOMERE_ORGANIZATION	-1.04103	0.366	0.75285
GOBP_CELL_CELL_JUNCTION_ASSEMBLY	-1.04271	0.241	0.75038
GOBP_RESPONSE_TO_GLUCAGON	-1.04439	0.411	0.74841
GOBP_POSITIVE_REGULATION_OF_STEROID_BIOSYNTHET	-1.04439	0.363	0.75172
GOBP_ACTIN_MYOSIN_FILAMENT_SLIDING	-1.04644	0.39	0.74844
GOBP_TRIGLYCERIDE_BIOSYNTHETIC_PROCESS	-1.04993	0.352	0.74031
GOBP_SODIUM_ION_HOMEOSTASIS	-1.0506	0.374	0.74143
GOBP_POSITIVE_REGULATION_OF_POTASSIUM_ION_TRA	-1.05157	0.378	0.74162
GOBP_ORGANIC_HYDROXY_COMPOUND_BIOSYNTHETIC_	-1.05223	0.231	0.74285
GOBP_NEGATIVE_REGULATION_OF_RECEPTOR_INTERNA	-1.0552	0.353	0.73607
GOBP_POSITIVE_REGULATION_OF_VIRAL_LIFE_CYCLE	-1.05672	0.373	0.73455
GOBP_POSITIVE_REGULATION_OF_ACTIN_FILAMENT_POL	-1.05886	0.362	0.73073
GOBP_RELAXATION_OF_MUSCLE	-1.06018	0.327	0.72959
GOBP_RESPONSE_TO_ISOQUINOLINE_ALKALOID	-1.0645	0.356	0.71886
GOBP_AXIS_ELONGATION	-1.06459	0.337	0.72194
GOBP_REGULATION_OF_THE_FORCE_OF_HEART_CONTR	-1.06465	0.346	0.72513
GOBP_PEROXISOME_ORGANIZATION	-1.06602	0.333	0.72418
GOBP_REGULATION_OF_CHOLESTEROL_METABOLIC_PR(	-1.0689	0.338	0.71811
GOBP_NEGATIVE_REGULATION_OF_BLOOD_VESSEL_END	-1.06904	0.354	0.72117
GOBP_HEPATOCYTE_APOPTOTIC_PROCESS	-1.06999	0.377	0.72146
GOBP_SUPEROXIDE_ANION_GENERATION	-1.07157	0.365	0.71998
GOBP_IRE1_MEDIATED_UNFOLDED_PROTEIN_RESPONSE	-1.07221	0.318	0.72147
GOBP_POSITIVE_REGULATION_OF_LAMELLIPODIUM_ORG	-1.07254	0.295	0.72387
GOBP_RESPONSE_TO_INTERLEUKIN_4	-1.07356	0.327	0.72395
GOBP_NEUROEPITHELIAL_CELL_DIFFERENTIATION	-1.0738	0.354	0.72663
GOBP_POLYOL_BIOSYNTHETIC_PROCESS	-1.07407	0.33	0.72925
GOBP_NUCLEOSIDE_MONOPHOSPHATE_CATABOLIC_PRO	-1.07514	0.36	0.72957
GOBP_C21_STEROID_HORMONE_METABOLIC_PROCESS	-1.07632	0.326	0.72914
GOBP_CELL_COMMUNICATION_BY_ELECTRICAL_COUPLIN	-1.0776	0.327	0.7284
GOBP_ANTIGEN_PROCESSING_AND_PRESENTATION_OF_	-1.07777	0.335	0.7314
GOBP_REGULATION_OF_RESPONSE_TO_REACTIVE_OXY(	-1.07843	0.313	0.7327
GOBP_CELLULAR_RESPONSE_TO_LEPTIN_STIMULUS	-1.07885	0.363	0.73496

GOBP_METANEPHRIC_EPITHELIUM_DEVELOPMENT	-1.07955	0.335	0.73629
GOBP_LIPID_DIGESTION	-1.08044	0.347	0.73686
GOBP_ADP_METABOLIC_PROCESS	-1.0805	0.25	0.7405
GOBP_KERATAN_SULFATE_METABOLIC_PROCESS	-1.08218	0.344	0.73847
GOBP_MIDDLE_EAR_MORPHOGENESIS	-1.0827	0.33	0.74055
GOBP_NEGATIVE_REGULATION_OF_ION_TRANSMEMBRAN	-1.08856	0.295	0.72442
GOBP_PEPTIDYL_TYROSINE_AUTOPHOSPHORYLATION	-1.08908	0.325	0.72644
GOBP_MUSCLE_HYPERTROPHY_IN_RESPONSE_TO_STRE	-1.08926	0.323	0.72966
GOBP_REGULATION_OF_MESENCHYMAL_CELL_PROLIFER	-1.08974	0.321	0.73193
GOBP_REGULATION_OF_REACTIVE_OXYGEN_SPECIES_BI	-1.09093	0.286	0.73189
GOBP_GLUCOCORTICOID_METABOLIC_PROCESS	-1.09097	0.295	0.73567
GOBP_REGULATION_OF_ATP_DEPENDENT_ACTIVITY	-1.0919	0.25	0.73649
GOBP_CELLULAR_SODIUM_ION_HOMEOSTASIS	-1.09259	0.313	0.73823
GOBP_MELANOCYTE_DIFFERENTIATION	-1.09411	0.304	0.73708
GOBP_REGULATION_OF_NEURON_PROJECTION_ARBORIZ	-1.09558	0.335	0.73617
GOBP_NEGATIVE_REGULATION_OF_ION_TRANSPORT	-1.09806	0.214	0.73176
GOBP_NEUTRAL_LIPID_METABOLIC_PROCESS	-1.09876	0.205	0.73358
GOBP_INOSITOL_PHOSPHATE_BIOSYNTHETIC_PROCESS	-1.10056	0.275	0.73143
GOBP_REGULATION_OF_CARDIAC_MUSCLE_CELL_MEMBR	-1.10067	0.297	0.73507
GOBP_AUDITORY_RECEPTOR_CELL_DEVELOPMENT	-1.1013	0.242	0.737
GOBP_SULFUR_COMPOUND_CATABOLIC_PROCESS	-1.10276	0.298	0.73572
GOBP_GLYCOSPHINGOLIPID_BIOSYNTHETIC_PROCESS	-1.10593	0.268	0.72893
GOBP_PEROXISOMAL_TRANSPORT	-1.10619	0.288	0.73219
GOBP_RECEPTOR_CATABOLIC_PROCESS	-1.11788	0.258	0.69688
GOBP_CELLULAR_KETONE_METABOLIC_PROCESS	-1.11884	0.235	0.69792
GOBP_EPITHELIAL_TUBE_BRANCHING_INVOLVED_IN_LUN	-1.12088	0.267	0.69485
GOBP_NATURAL_KILLER_CELL_DIFFERENTIATION	-1.12092	0.266	0.69882
GOBP_CELL_PROLIFERATION_INVOLVED_IN_HEART_MOR	-1.12141	0.28	0.70131
GOBP_POSITIVE_REGULATION_OF_VASCULAR_PERMEABI	-1.12269	0.299	0.70099
GOBP_INTERMEDIATE_FILAMENT_ORGANIZATION	-1.12338	0.238	0.7027
GOBP_DIGESTIVE_SYSTEM_PROCESS	-1.12505	0.159	0.70125
GOBP_HAIR_FOLLICLE_MATURATION	-1.12807	0.284	0.69533
GOBP_ENDOCRINE_PROCESS	-1.12907	0.159	0.69645
GOBP_NEGATIVE_REGULATION_OF_CARBOHYDRATE_ME	-1.13146	0.216	0.69276
GOBP_NEGATIVE_REGULATION_OF_GLUCOSE_TRANSMEI	-1.132	0.287	0.69511
GOBP_GENITALIA_DEVELOPMENT	-1.13295	0.264	0.69622
GOBP_MAMMARY_GLAND_DEVELOPMENT	-1.13384	0.237	0.69782
GOBP_CELL_COMMUNICATION_BY_ELECTRICAL_COUPLIN	-1.13402	0.268	0.70162
GOBP_REGULATION_OF_FATTY_ACID_METABOLIC_PROCE	-1.13744	0.213	0.69441
GOBP_INDOLE_CONTAINING_COMPOUND_METABOLIC_PR	-1.13946	0.27	0.69189
GOBP_TRIGLYCERIDE_METABOLIC_PROCESS	-1.14	0.176	0.69436
GOBP_FEMALE_GENITALIA_DEVELOPMENT	-1.14021	0.251	0.69819
GOBP_ENDOCRINE_HORMONE_SECRETION	-1.14189	0.164	0.6972
GOBP_SIGNALING_RECEPTOR_LIGAND_PRECURSOR_PRO	-1.14231	0.225	0.70037
GOBP_BRANCHING_INVOLVED_IN_MAMMARY_GLAND_DU	-1.14291	0.262	0.70266
GOBP_STEROID_HORMONE_SECRETION	-1.14742	0.264	0.69234
GOBP_NEGATIVE_REGULATION_OF_FATTY_ACID_METAB	-1.14892	0.233	0.69218
GOBP_NUCLEOSIDE_PHOSPHATE_CATABOLIC_PROCESS	-1.15073	0.141	0.69074
GOBP_VENTRICULAR_CARDIAC_MUSCLE_CELL_MEMBRAN	-1.15266	0.21	0.68879
GOBP_POSITIVE_REGULATION_OF_ALCOHOL_BIOSYNTHE	-1.15442	0.238	0.68757
GOBP_DEVELOPMENTAL_INDUCTION	-1.15566	0.266	0.68791
GOBP_MYELIN_ASSEMBLY	-1.15668	0.234	0.68928
GOBP_REGULATION_OF_NUCLEOTIDE_BIOSYNTHETIC_PR	-1.15894	0.218	0.68638

GOBP_NEGATIVE_REGULATION_OF_ATP_METABOLIC_PRO	-1.15978	0.206	0.6884
GOBP_ACYLGLYCEROL_HOMEOSTASIS	-1.16415	0.189	0.67875
GOBP_AMYLOID_BETA_METABOLIC_PROCESS	-1.16451	0.191	0.6824
GOBP_EXPLORATION_BEHAVIOR	-1.16903	0.26	0.67264
GOBP_VENTRICULAR_CARDIAC_MUSCLE_CELL_ACTION_F	-1.17424	0.21	0.66007
GOBP_REGULATION_OF_ACTIN_NUCLEATION	-1.17565	0.196	0.66026
GOBP_NUCLEAR_MIGRATION	-1.17617	0.221	0.66355
GOBP_ACTIN_FILAMENT_DEPOLYMERIZATION	-1.17768	0.148	0.66361
GOBP_POSITIVE_REGULATION_OF_BROWN_FAT_CELL_DI	-1.1784	0.234	0.66613
GOBP_METANEPHRIC_TUBULE_DEVELOPMENT	-1.17859	0.269	0.67057
GOBP_POSITIVE_REGULATION_OF_STEROID_METABOLIC	-1.18085	0.218	0.66806
GOBP_REGULATION_OF_G_PROTEIN_COUPLED_RECEPTO	-1.18255	0.067	0.66753
GOBP_SMOOTH_MUSCLE_CELL_DIFFERENTIATION	-1.18829	0.153	0.65383
GOBP_STEROID_BIOSYNTHETIC_PROCESS	-1.18928	0	0.65576
GOBP_MEMBRANE_REPOLARIZATION	-1.19212	0.173	0.65166
GOBP_POSITIVE_REGULATION_OF_INSULIN_SECRETION	-1.19298	0.167	0.65401
GOBP_PYRUVATE_METABOLIC_PROCESS	-1.19556	0.104	0.65126
GOBP_STEROL_HOMEOSTASIS	-1.19593	0.157	0.65533
GOBP_NEGATIVE_REGULATION_OF_ION_TRANSMEMBRAN	-1.19741	0.043	0.65564
GOBP_POSITIVE_REGULATION_OF_TRIGLYCERIDE_META	-1.19906	0.194	0.65556
GOBP_LUNG_ALVEOLUS_DEVELOPMENT	-1.19962	0.168	0.65906
GOBP_REGULATION_OF_ANIMAL_ORGAN_FORMATION	-1.20222	0.199	0.65583
GOBP_PROTEIN_AUTOPROCESSING	-1.20223	0.207	0.66126
GOBP_POSITIVE_REGULATION_OF_HEART_RATE	-1.2075	0.186	0.64952
GOBP_LEUKOTRIENE_METABOLIC_PROCESS	-1.21206	0.195	0.64086
GOBP_REGULATION_OF_AUTOPHAGY_OF_MITOCHONDRI	-1.21702	0.174	0.6306
GOBP_NUCLEUS_LOCALIZATION	-1.21841	0.18	0.63147
GOBP_REGULATION_OF_HYDROGEN_PEROXIDE_METABO	-1.22763	0.184	0.60827
GOBP_POSITIVE_REGULATION_OF_TRANSPORTER_ACTIV	-1.22902	0.048	0.60951
GOBP_REGULATION_OF_GASTRULATION	-1.22994	0.17	0.6121
GOBP_MAMMARY_GLAND_DUCT_MORPHOGENESIS	-1.23026	0.145	0.61661
GOBP_ENAMEL_MINERALIZATION	-1.2356	0.194	0.60524
GOBP_STRIATED_MUSCLE_ADAPTATION	-1.23599	0.138	0.60945
GOBP_ORGAN_INDUCTION	-1.2369	0.162	0.61214
GOBP_MIDBRAIN_DEVELOPMENT	-1.23744	0.115	0.61617
GOBP_CELLULAR_COMPONENT_DISASSEMBLY_INVOLVED	-1.23848	0.164	0.61834
GOBP_REGULATION_OF_CARDIAC_MUSCLE_CONTRACTIO	-1.24294	0.096	0.61012
GOBP_NEGATIVE_REGULATION_OF_PROTEIN_POLYMERIZ	-1.24493	0.038	0.6099
GOBP_AORTA_MORPHOGENESIS	-1.2464	0.151	0.61077
GOBP_LEUKOTRIENE_BIOSYNTHETIC_PROCESS	-1.24799	0.173	0.61154
GOBP_NEGATIVE_REGULATION_OF_TRANSPORTER_ACTI	-1.24916	0.107	0.6138
GOBP_STEROL_METABOLIC_PROCESS	-1.25041	0.037	0.6157
GOBP_NEGATIVE_REGULATION_OF_BIOMINERALIZATION	-1.25327	0.168	0.61221
GOBP_REGULATION_OF_BROWN_FAT_CELL_DIFFERENTIA	-1.25643	0.157	0.60714
GOBP_NEGATIVE_REGULATION_OF_MUSCLE_HYPERTROF	-1.25727	0.175	0.61048
GOBP_PURINE_NUCLEOBASE_METABOLIC_PROCESS	-1.25805	0.167	0.61432
GOBP_HEART_FORMATION	-1.25973	0.148	0.61517
GOBP_NEGATIVE_REGULATION_OF_DEPHOSPHORYLATIO	-1.26658	0.114	0.59916
GOBP_RETINA_LAYER_FORMATION	-1.27296	0.101	0.58638
GOBP_MAMMARY_GLAND_EPITHELIUM_DEVELOPMENT	-1.27482	0.071	0.58714
GOBP_REGULATION_OF_CELL_CELL_ADHESION_MEDIATE	-1.27526	0.134	0.59195
GOBP_NEGATIVE_REGULATION_OF_AXON_EXTENSION_IN	-1.27995	0.143	0.58446
GOBP_POSITIVE_REGULATION_OF_ION_TRANSMEMBRAN	-1.28063	0	0.58877

GOBP_NUCLEOBASE_METABOLIC_PROCESS	-1.28391	0.118	0.58573
GOBP_NEUTRAL_LIPID_CATABOLIC_PROCESS	-1.28516	0.113	0.58826
GOBP_CARDIAC_CONDUCTION	-1.28589	0	0.59281
GOBP_NEURAL_NUCLEUS_DEVELOPMENT	-1.28619	0.073	0.59869
GOBP_REGULATION_OF_ANION_TRANSMEMBRANE_TRAN	-1.28968	0.119	0.59398
GOBP_CORTICAL_CYTOSKELETON_ORGANIZATION	-1.28983	0.083	0.60059
GOBP_RESPONSE_TO_STEROL_DEPLETION	-1.29116	0.163	0.60368
GOBP_MORPHOGENESIS_OF_AN_EPITHELIAL_FOLD	-1.29192	0.15	0.60847
GOBP_PYRIMIDINE_NUCLEOTIDE_CATABOLIC_PROCESS	-1.29596	0.138	0.60285
GOBP_BRANCH_ELONGATION_OF_AN_EPITHELIUM	-1.29644	0.158	0.60887
GOBP_LABYRINTHINE_LAYER_MORPHOGENESIS	-1.29807	0.128	0.61093
GOBP_MAMMARY_GLAND_MORPHOGENESIS	-1.29833	0.101	0.61801
GOBP_NEGATIVE_REGULATION_OF_PROTEIN_DEPHOSPH	-1.30198	0.116	0.61407
GOBP_CARDIAC_MUSCLE_CELL_MEMBRANE_REPOLARIZA	-1.30447	0.143	0.61397
GOBP_INORGANIC_ION_IMPORT_ACROSS_PLASMA_MEMB	-1.30484	0.043	0.62094
GOBP_POSITIVE_REGULATION_OF_CALCIUM_ION_TRANS	-1.30872	0.074	0.61666
GOBP_VITAMIN_D_METABOLIC_PROCESS	-1.30963	0.128	0.62192
GOBP_SPECIFICATION_OF_ANIMAL_ORGAN_IDENTITY	-1.30978	0.123	0.63002
GOBP_ALCOHOL_BIOSYNTHETIC_PROCESS	-1.31045	0	0.63661
GOBP_NEGATIVE_REGULATION_OF_TRANSMEMBRANE_T	-1.31133	0	0.64271
GOBP_REGULATION_OF_BIOLOGICAL_PROCESS_INVOLVE	-1.31673	0.083	0.63261
GOBP_MALE_GENITALIA_DEVELOPMENT	-1.3179	0.105	0.63765
GOBP_LAMELLIPODIUM_MORPHOGENESIS	-1.32217	0.133	0.63235
GOBP_POSITIVE_REGULATION_OF_POTASSIUM_ION_TRA	-1.32348	0.083	0.63677
GOBP_REGULATION_OF_CARDIAC_CONDUCTION	-1.32349	0.094	0.64654
GOBP_REGULATION_OF_STRIATED_MUSCLE_CONTRACTI	-1.32441	0.026	0.65343
GOBP_NEUTROPHIL_MEDIATED_IMMUNITY	-1.32573	0.049	0.65932
GOBP_NEGATIVE_REGULATION_OF_PHOSPHATASE_ACTI	-1.32787	0.091	0.66193
GOBP_NEGATIVE_REGULATION_OF_G_PROTEIN_COUPLE	-1.3369	0.04	0.64048
GOBP_NEGATIVE_REGULATION_OF_PHOSPHOPROTEIN_F	-1.3388	0.089	0.64422
GOBP_REGULATION_OF_CELLULAR_RESPIRATION	-1.3396	0.058	0.65212
GOBP_EPITHELIAL_CELL_MORPHOGENESIS	-1.34223	0.087	0.65314
GOBP_REGULATION_OF_SODIUM_ION_TRANSMEMBRANE	-1.34516	0.058	0.65427
GOBP_NEGATIVE_REGULATION_OF_VIRAL_LIFE_CYCLE	-1.34519	0.122	0.66581
GOBP_CELLULAR_RESPONSE_TO_STEROL_DEPLETION	-1.34727	0.122	0.67037
GOBP_THYMUS_DEVELOPMENT	-1.34794	0.108	0.68009
GOBP_AMINE_BIOSYNTHETIC_PROCESS	-1.35121	0.091	0.68109
GOBP_TRANSCYTOSIS	-1.35224	0.112	0.69045
GOBP_ANIMAL_ORGAN_FORMATION	-1.35889	0.02	0.67865
GOBP_ANION_HOMEOSTASIS	-1.36371	0.068	0.674
GOBP_REGULATION_OF_DIGESTIVE_SYSTEM_PROCESS	-1.36476	0.043	0.68386
GOBP_GLUTAMINE_METABOLIC_PROCESS	-1.38077	0.096	0.63781
GOBP_NEGATIVE_REGULATION_OF_VIRAL_ENTRY_INTO_	-1.38368	0.09	0.64014
GOBP_THYROID_GLAND_DEVELOPMENT	-1.38643	0.085	0.6453
GOBP_GAP_JUNCTION_ASSEMBLY	-1.38854	0.082	0.65117
GOBP_NEGATIVE_REGULATION_OF_SODIUM_ION_TRANS	-1.39882	0.071	0.62918
GOBP_SIALYLATION	-1.40356	0.087	0.6274
GOBP_SECONDARY_ALCOHOL_METABOLIC_PROCESS	-1.40456	0	0.639
GOBP_REGULATION_OF_RESPIRATORY_BURST	-1.40979	0.08	0.63545
GOBP_CORTICAL_ACTIN_CYTOSKELETON_ORGANIZATION	-1.41297	0.052	0.64054
GOBP_GLUCOSE_6_PHOSPHATE_METABOLIC_PROCESS	-1.41537	0.085	0.64816
GOBP_REGULATION_OF_SYSTEMIC_ARTERIAL_BLOOD_PI	-1.41768	0.067	0.65592
GOBP_POSITIVE_REGULATION_OF_ATP_DEPENDENT_ACT	-1.42017	0.036	0.66418

GOBP_PEPTIDYL_ARGININE_MODIFICATION	-1.4203	0.071	0.68239
GOBP_ACETYL_COA_BIOSYNTHETIC_PROCESS	-1.42562	0.088	0.6828
GOBP_POSITIVE_REGULATION_OF_MESENCHYMAL_CELL_	-1.42587	0.061	0.70163
GOBP_CATECHOL_CONTAINING_COMPOUND_BIOSYNTHE	-1.42667	0.084	0.71972
GOBP_MITOCHONDRIAL_CALCIUM_ION_HOMEOSTASIS	-1.42685	0.03	0.74138
GOBP_NEGATIVE_REGULATION_OF_POTASSIUM_ION_TRA	-1.43399	0.071	0.73426
GOBP_NEGATIVE_REGULATION_OF_POTASSIUM_ION_TRA	-1.44375	0.067	0.71863
GOBP_RENAL_ABSORPTION	-1.44504	0.058	0.73814
GOBP_POSITIVE_REGULATION_OF_VASOCONSTRICTION	-1.44947	0.059	0.74553
GOBP_POSITIVE_REGULATION_OF_SODIUM_ION_TRANSM	-1.4685	0.064	0.6883
GOBP_POTASSIUM_ION_HOMEOSTASIS	-1.47168	0.07	0.69973
GOBP_DEOXYRIBOSE_PHOSPHATE_CATABOLIC_PROCESS	-1.47351	0.04	0.71992
GOBP_AMINE_CATABOLIC_PROCESS	-1.47606	0.042	0.73827
GOBP_REGULATION_OF_OXIDATIVE_PHOSPHORYLATION	-1.48077	0.026	0.74876
GOBP_REGULATION_OF_RECEPTOR_RECYCLING	-1.48315	0.046	0.77079
GOBP_MYELIN_MAINTENANCE	-1.51127	0.052	0.67967
GOBP_RECEPTOR_RECYCLING	-1.5128	0.041	0.70738
GOBP_REGULATION_OF_POTASSIUM_ION_TRANSPORT	-1.5184	0	0.71977
GOBP_SUBSTANTIA_NIGRA_DEVELOPMENT	-1.53758	0.045	0.6726
GOBP_INTESTINAL_ABSORPTION	-1.53854	0.034	0.70701
GOBP_POTASSIUM_ION_IMPORT_ACROSS_PLASMA_MEMB	-1.56331	0.015	0.6404
GOBP_POSITIVE_REGULATION_OF_PROTEIN_CONTAINING	-1.57725	0.006	0.62036
GOBP_PHOSPHATIDYLSERINE_METABOLIC_PROCESS	-1.60284	0.017	0.56213
GOBP_RELAXATION_OF_CARDIAC_MUSCLE	-1.64064	0.038	0.47218
GOBP_STEROL_BIOSYNTHETIC_PROCESS	-1.65353	0	0.46848
GOBP_BARBED_END_ACTIN_FILAMENT_CAPPING	-1.67171	0.005	0.44875
GOBP_REGULATION_OF_VIRAL_ENTRY_INTO_HOST_CELL	-1.68562	0	0.44704
GOBP_SIGNAL_TRANSDUCTION_INVOLVED_IN_REGULATIO	-1.69593	0.024	0.45947
GOBP_MEMBRANE_REPOLARIZATION_DURING_ACTION_P	-1.81406	0.005	0.20332
GOBP_ACTIN_FILAMENT_SEVERING	-1.81586	0.009	0.22897
GOBP_EXPORT_ACROSS_PLASMA_MEMBRANE	-1.84368	0	0.21308
GOBP_REGULATION_OF_AEROBIC_RESPIRATION	-1.95415	0	0.08297
GOBP_METAL_ION_EXPORT	-2.03601	0	0.04578
GOBP_MEMBRANE_REPOLARIZATION_DURING_CARDIAC_	-2.09348	0	0.03085
GOBP_GENERATION_OF_PRECURSOR_METABOLITES_ANI	---	---	1
GOBP_SMALL_MOLECULE_BIOSYNTHETIC_PROCESS	---	---	1

Supplementary Table 7

Pb_Pten_zfp_intact vs Pb_Pten_intact					
No	Mouse EntrezID	Symbol	Human EntrezID	Fold Change	FDR
1	14679	Gnai3	2773	0.151382477	0.99988
2	12544	Cdc45	8318	-0.102803065	0.54863
3	67608	Narf	26502	0.252045385	0.99988
4	12390	Cav2	858	-0.563491232	0.40102
5	23849	Klf6	1316	-1.242164684	0.22907
6	29871	Scmh1	22955	-0.128568647	0.94548
7	12858	Cox5a	9377	0.436944661	0.03492
8	21385	Tbx2	6909	-0.942073085	0.4382
9	18053	Ngfr	4804	1.92995562	0.0059
10	216795	Wnt9a	7483	1.901672783	0.00646
11	14158	Fer	2241	0.703050756	0.49436
12	74204	Xpo6	23214	-0.522118825	0.41499
13	209446	Tfe3	7030	-0.736481574	0.29838
14	12006	Axin2	8313	0.167352239	0.77241
15	231841	Brat1	221927	-0.19264261	0.99988
16	14673	Gna12	2768	-0.703329815	0.3155
17	18400	Slc22a18	5002	-0.141647412	0.96766
18	72058	Igsf5		-0.236458328	0.86124
19	72614	Pih1d2	120379	-0.634648785	0.51006
20	235339	Dlat	1737	-0.301369224	0.58979
21	12444	Ccnd2	894	-2.369004278	0.04188
22	277463	Gpr107	57720	-0.215391478	0.87783
23	338370	Nalcn	259232	-1.338353289	0.20943
24	72014	Btbd17	388419	-1.035940048	0.02595
25	20558	Slfn4		-0.400793807	0.68253
26	27027	Tspan32	10077	0.720710017	0.22067
27	66355	Gmpr	2766	-2.059190872	0.07664
28	23947	Mid2	11043	0.515405094	0.93433
29	217069	Trim25	7706	-0.277312676	0.57839
30	56077	Dgke	8526	-1.017210245	0.17862
31	74617	Scpep1	59342	2.04044308	0.0045
32	17428	Mnt	4335	0.04046781	0.99053
33	16414	Itgb2		2.160415444	0.00533
34	69692	Hddc2	51020	0.182632321	0.99988
35	21987	Tpd52l1	7164	-1.063381091	0.20497
36	12550	Cdh1	999	-0.727799449	0.31925
37	12716	Ckmt1		0.789939122	0.02595
38	17312	Clec10a		1.986891734	0.00544
39	11684	Alox12	239	1.426414668	0.00388
40		NA		0.409342308	0.36204
41	12846	Comt	1312	1.030047576	0.00574
42	66368	Rtca	8634	-0.343575848	0.56269
43	13171	Dbt	1629	-0.513133247	0.47329
44	23994	Dazap2	9802	0.122519944	0.99988
45	68995	Mcts1		0.458620184	0.48237
46	19700	Rem1	28954	1.340280495	0.09448
47	216131	Trappc10		0.180235217	0.99988

48	216527	Ccm2	83605	-0.404530827	0.74793
49	21379	Tbrg4	9238	-0.615990227	0.42744
50	50528	Tmprss2	7113	0.529530152	0.03116
51		Mx1		-1.404087915	0.26781
52	14089	Fap	2191	0.976190651	0.0482
53	66108	Ndufa9	4704	-0.291250401	0.7853
54	16818	Lck	3932	1.385074286	0.00577
55	30785	Cttnbp2	83992	-2.609551607	0.0461
56	14423	Galnt1	2589	-0.138368487	0.99767
57	67027	Mkrn2	23609	-0.451366992	0.82309
58	19016	Pparg	5468		0.991
59	110157	Raf1	5894	0.173630657	0.99988
60	54204	Septin1	1731	1.944006124	0.05266
61	18591	Pdgfb	5155	1.091562243	0.23077
62	11482	Acvrl1	94	0.642771239	0.03339
63	56149	Tamalin	160622	0.132811425	0.99988
64	11479	Acvr1b	91	0.242594077	0.99988
65	216810	Tom1l2	146691	-0.280852551	0.86915
66	59290	Gpa33	10223	1.485067111	0.00576
67	70465	Wdr77	79084	0.343264343	0.65767
68	11950	Atp5pb	515	0.584489153	0.02604
69	20682	Sox9	6662	-1.138665193	0.18752
70	11991	Hnrnpd	3184	-0.075357015	0.94351
71	100040563	Dynlt1c		0.383519177	0.50868
72	57316	C1d	10438	-0.133658177	0.99988
73	14667	Gm2a	2760	0.533412322	0.06987
74	79264	Krit1		0.212287367	0.64614
75	12727	Clcn4	1183	0.050159775	0.99988
76	15277	Hk2	3099	-1.293858821	0.09148
77	360013	Myo18a	399687	-0.365977922	0.52899
78	107766	Haao	23498	0.186605107	0.73305
79	23833	Cd52	1043	2.481696453	0.00406
80	16950	Loxl3	84695	0.1582052	0.82705
81	18519	Kat2b	8850	-0.586054081	0.53754
82	19344	Rab5b		-0.085315449	0.99988
83	234847	Spg7	6687	-0.250059005	0.81794
84	57429	Sult5a1		0.681249042	0.88428
85	270106	Rpl13	6137	-0.020616381	0.99988
86	234852	Chmp1a	5119	-0.157522736	0.94808
87	68275	Rpa1	6117	-0.037853118	0.99988
88	20317	Serpinf1	5176	0.819929538	0.19123
89	259279	Tubgcp3	10426	0.05521879	0.92702
90	67065	Polr3d	661	-0.318781085	0.89847
91	13205	Ddx3x	1654	-0.742727671	0.30443
92	16161	Il12rb1	3594	1.494140979	0.00837
93	114479	Slc5a5	6528	-0.856052404	0.54938
94	140493	Kcnn3	3782	0.410147101	0.58886
95	237898	Usp32		0.52462165	0.04448
96	232223	Txnrd3	114112	-1.208103778	0.21388
97	14103	Fasl	356		0.40878

98	269401	Zfp512b	57473	-1.533340091	0.17064
99	13002	Dnajc5	80331	-0.81793864	0.27327
100	66314	Tpd52l2	7165	0.31295213	0.40444
101	14265	Fmr1	2332	-0.443044342	0.76504
102	14025	Bcl11a	53335	-0.898485664	0.53317
103	59038	Pxmp4	11264	0.334069654	0.53643
104	53310	Dlg3	1741	-0.05602411	0.86332
105	13972	Gnb1l	54584	0.515544173	0.76002
106	17385	Mmp11	4320	-0.845262038	0.30728
107	20587	Smarcb1	6598	0.155395561	0.89785
108	29816	Hip1r	9026	-0.453984801	0.38023
109	100609	Nsun5	55695	-0.362072893	0.51344
110	72960	Top1mt	116447	-0.560234597	0.34305
111	15395	Hoxa10	3206	0.043656706	0.99026
112	17387	Mmp14	4323	-0.389798884	0.64985
113	69089	Oxa1l	5018	0.115761198	0.991
114	217026	Heatr6	63897	-0.25555351	0.79951
115	20302	Ccl3		-0.423384284	0.95106
116	14038	Wfdc18		-3.42664537	0.02152
117	67781	Ilf2	3608	-0.432497238	0.72306
118	66511	Chtop	26097	0.147728252	0.59021
119	20615	Snapin	23557	0.079904435	0.99988
120	20198	S100a4	6275	1.403181726	0.00646
121	20200	S100a6	6277	0.923651723	0.03535
122	15896	Icam2	3384	0.185035012	0.86569
123	23939	Mapk7	5598	-0.999943683	0.45402
124	13855	Epn2	22905	-0.504219704	0.38367
125	27078	B9d1	27077	-0.388130069	0.99716
126	99683	Sec24b	10427	0.217510632	0.99988
127	212706	N4bp3	23138	0.522227273	0.02178
128	66089	Rmnd5b	64777	0.322463767	0.7106
129	52530	Nhp2	55651	-1.18731212	0.14435
130	72325	Vps9d1	9605	0.500769647	0.47218
131	57247	Zfp276	92822	-0.070579348	0.99988
132	68294	Mfsd10	10227	-0.576352678	0.55251
133	269593	Luzp1	7798	-0.647079608	0.31877
134	67811	Poldip2	26073	-0.716756411	0.29753
135	55978	Ift20	90410	0.407315199	0.71645
136	12833	Col6a1	1291	1.677811642	0.09252
137	59093	Pcbp3	54039	0.189320387	0.83966
138	16859	Lgals9		-0.428801207	0.55251
139	11836	Araf	369	-0.086711409	0.99988
140	18636	Cfp	5199	2.400998322	0.00438
141	21857	Timp1	7076	0.353030146	0.35378
142	22294	Uxt	8409	0.278708268	0.57909
143	94218	Cnnm3	26505	0.719944176	0.02101
144	214895	Lman2l	81562	0.118356547	0.99988
145	54387	Mcm3ap	8888	-1.33095018	0.21847
146	18541	Pcnt	5116	0.489131522	0.01881
147	17119	Mxd1	4084	-0.427290258	0.90772

148	23885	Gmcl1		0.67606107	0.00892
149	66618	Snrnp27		-0.257201378	0.61803
150	114643	Oas1c		-0.668059045	0.49915
151	320634	Ocrl	4952	0.245305161	0.8833
152	12313	Calm1	801	-0.527707839	0.40418
153	28169	Agpat3	56894	-0.450293411	0.3295
154	20359	Sema6b	10501	-0.296273534	0.79357
155	224897	Dpp9	91039	-0.715033618	0.60337
156	54409	Ramp2	10266	1.00750532	0.01374
157	54135	Lsr	51599	-0.651041834	0.35903
158	52857	Gramd1a	57655	-0.087623172	0.99988
159	15451	Hpn	3249	0.300681486	0.74448
160	12709	Ckb	1152	0.806209526	0.47103
161	20683	Sp1	6667	-0.243731173	0.62665
162	16421	Itgb7	3695	0.73423414	0.06511
163	60315	Myg1	60314	0.885119957	0.33055
164	19411	Rarg	5916	-1.482883362	0.20549
165	56612	Pfdn5	5204	-0.108795355	0.94097
166	13642	Efnb2	1948	-0.157824744	0.75527
167	67223	Rrp15	51018	-0.381687954	0.65068
168	11858	Rnd2	8153	0.98304048	0.27467
169	27364	Srr	63826	1.604113205	0.0535
170	68828	Sync	81493	1.746515196	0.19839
171	384061	Fndc5	252995	-1.665723621	0.09709
172	11433	Acp5	54	0.93301151	0.20287
173	71538	Fbxo9	26268	-0.608184894	0.39291
174	73288	Vps50	55610	-0.041527775	0.99988
175	15115	Hars	3035	-0.595077506	0.48921
176	66492	Zmat2	153527	-0.033697779	0.99988
177	68612	Ube2c	11065	-0.130682131	0.99988
178	229512	Smg5	23381	-1.273258498	0.17928
179	12462	Cct3	7203	-0.054878717	0.88106
180	56700	Glmp	112770	0.043864	0.99988
181	17261	Mef2d	4209	-0.608109343	0.55298
182	56463	Snd1	27044	-1.071832559	0.23545
183	12822	Col18a1	80781	0.447280713	0.58476
184	20509	Slc19a1	6573	0.743627602	0.38023
185	16211	Kpnb1	3837	-0.180831368	0.69294
186	19155	Npepps	9520	-0.861492434	0.24087
187	107732	Mrpl10	124995	-0.115352188	0.83547
188	13121	Cyp51	1595	-0.34958039	0.72669
189	66855	Tcf25	22980	-1.000858191	0.22111
190	67951	Tubb6	84617	0.183054161	0.49268
191	23854	Def8	54849	-0.387828074	0.51639
192	17285	Meox1	4222	-0.659514355	0.30098
193	18511	Pax9	5083	1.334341235	0.14124
194	16372	Irx2	153572	-0.094378232	0.86708
195	12842	Col1a1	1277	0.671873765	0.33219
196	16400	Itga3	3675	-0.784112718	0.32719
197	101142	Itfg2	55846	-0.545344771	0.37617

198	22158	Tulp3	7289	-0.993939507	0.22128
199	14885	Gtf2h4	2968	0.07810681	0.97566
200	22154	Tubb5	203068	-0.099397939	0.79179
201	192657	Ell2	22936	0.15164501	0.99988
202	16480	Jup	3728	-0.141635711	0.99988
203	14230	Fkbp10	60681	-0.35399177	0.58253
204	433864	Nom1	64434	-0.626149221	0.31652
205	67458	Ergic1	57222	-0.437681797	0.78562
206	83813	Tnk1	8711	-1.473527178	0.21879
207	21401	Tcea3	6920	-0.321365301	0.81166
208	15982	Ifrd1		0.092461367	0.99988
209	232533	Stk38l	23012	-0.737450009	0.3509
210	78783	Brpf1	7862	-0.486778167	0.54409
211	11677	Akr1b3	231	0.048771479	0.99988
212	14871	Gstt1		-1.358100934	0.15423
213	103140	Gstt3		-0.67829121	0.35981
214	13202	Ddt		-0.591461761	0.32978
215	73608	Marveld3	91862	-0.335532031	0.62323
216	66942	Ddx18	8886	-0.373262009	0.40308
217	24109	Ubl3	5412	0.263742364	0.48725
218	107022	Gramd3	65983	0.802667189	0.01459
219	66143	Eef1e1		0.092127805	0.99988
220	11651	Akt1	207	0.195860746	0.85705
221	16170	Il16	3603	1.901784889	0.0059
222	27060	Tcirg1	10312	-0.712322826	0.43347
223	27419	Naglu	4669	0.256972027	0.99779
224	71743	Coasy	80347	-0.006148213	0.99988
225	319757	Smo	6608	0.273117661	0.47732
226	232670	Tspan33	340348	0.200106846	0.99988
227	66877	Crnkl1	51340	-0.527284098	0.88823
228	74030	Rin2	54453	-0.624857661	0.30663
229	66917	Chordc1	26973	-0.065818667	0.94323
230	28088	Rtcb	51493	-0.408656457	0.5132
231	103136	Pwp1	11137	-0.189433944	0.76849
232	69754	Fbxo7	25793	0.136426407	0.99988
233	12336	Capns1	826	-0.522252621	0.34972
234	14029	Evx2	344191	1.420986795	0.01186
235	15433	Hoxd13	3239	0.68882563	0.45675
236	15432	Hoxd12	3238	-0.309676469	0.64948
237	14275	Folr1	2348	-0.070830252	0.88911
238	20480	Clpb	81570	0.366355467	0.99988
239	235072	Septin7	989	0.01947795	0.99988
240	72881	Zdhhc4	55146	-0.137601054	0.99988
241	19353	Rac1	5879	0.245220565	0.5089
242	227720	Nup214	8021	0.351762816	0.54049
243	108897	Aif1l	83543	0.694459751	0.02148
244	268977	Ltbp1	4052	-1.211578538	0.19479
245	216558	Ugp2	7360	0.103606436	0.85956
246	212528	Trmt1	55621	-0.459071498	0.44778
247	66830	Nacc1	112939	1.07146347	0.16127

248	18032	Nfix	4784	-1.010765136	0.2496
249	20514	Slc1a5	6510	-0.556314174	0.64699
250	22201	Uba1	7317	-0.71243211	0.4295
251	22619	Siae	54414	0.314560457	0.34557
252	69524	Esam	90952	0.557882869	0.44778
253	20686	Spa17	53340	0.719143095	0.01554
254	108160	Fam50a	9130	-0.79870152	0.21288
255	13726	Emd	2010	0.339192867	0.05625
256	70207	Taco1	51204	-0.817962486	0.4267
257	53623	Gria3		0.439762189	0.38173
258	244668	Sipa1l2	57568	-1.043113084	0.30695
259	108011	Ap4e1	23431	-0.350597946	0.59106
260	109778	Blvra	644	0.394541666	0.66181
261	245469	Pdzd4	57595	0.853921216	0.31737
262	15929	Idh3g		0.225480176	0.99988
263	20832	Ssr4	6748	0.317430058	0.53603
264	27061	Bcap31	10134	-0.292399914	0.72295
265	72722	Fam98a	25940	-0.477410052	0.34612
266	16997	Ltbp2	4053	0.641420125	0.56346
267	214162	Kmt2a		-0.714600511	0.22364
268	76568	Ift46	56912	-0.014178114	0.99988
269	67091	Trappc6a	79090	-0.783835567	0.30831
270	20926	Supt6	6830	-0.297742498	0.57995
271	54141	Spag5	10615	-0.222961074	0.9575
272	22248	Unc119	9094	1.78224227	0.00574
273	19376	Rab34	83871	0.282068227	0.98645
274	20316	Sdf2	6388	0.484280919	0.09987
275	12447	Ccne1	898	-0.197939805	0.78281
276	170770	Bbc3	27113	-0.778030246	0.40878
277	19182	Psmc3	5702	0.231523041	0.46249
278	11432	Acp2	53	-0.221782948	0.60462
279	68427	Slc39a13	91252	0.786770063	0.0447
280	14007	Celf2	10659	-0.493822089	0.82835
281	22259	Nr1h3	10062	0.035922278	0.8717
282	20375	Spi1	6688	0.796777419	0.20508
283	67465	Sf3a1	10291	-0.451330115	0.3801
284	20852	Stat6	6778	0.072822208	0.97102
285	16541	Napsa	9476	1.022032556	0.01353
286	101568	Vrk3	51231	0.503509725	0.20222
287	71997	Smg9	56006	-0.462469643	0.33451
288	55982	Paxip1	22976	-1.705654995	0.11394
289	68477	Rmnd5a	64795	-0.520322952	0.32998
290	17454	Mov10	4343	0.017827119	0.99988
291	11853	Rhoc	389	0.786213138	0.04666
292	21678	Tead3	7005	-0.228890797	0.83045
293	19015	Ppard	5467	-0.273028713	0.99988
294	23853	Def6	50619	0.363413308	0.99988
295	18616	Peg3	5178	1.299595109	0.01102
296	76483	Lmf1	64788	-0.64321437	0.35378
297	67563	Ciao3	64428	-0.426155607	0.40878

298	57875	Angptl4	51129	0.352924092	0.991
299	27214	Dbf4	10926	0.232808281	0.60464
300	13163	Daxx	1616	-0.893636095	0.22887
301	54219	Cd320	51293	-0.591328438	0.39006
302	75751	Ipo4	79711	-0.48017831	0.42002
303	74140	Tm9sf1		0.361485084	0.94963
304	56739	Rec8	9985	-0.128438788	0.99988
305	16391	Irf9	10379	0.056645342	0.60279
306	105446	Gmpr2	51292	1.405207765	0.16164
307	67881	Mdp1		0.297543341	0.05646
308	52585	Dhrs1	115817	0.402746159	0.90862
309	234371	Tmem161a	54929	0.298357844	0.17976
310	76813	Armc6	93436	1.181732151	0.06285
311	72368	Borcs8	729991	-0.084122094	0.90897
312	73095	Slc25a42	284439	-1.006022072	0.01133
313	66616	Snx9	51429	0.551372596	0.23425
314	71810	Ranbp3	8498	-0.66393901	0.42699
315	69875	Ndufa11		0.061837482	0.99988
316	13864	Nr2f6	2063	-1.636398686	0.12711
317	67023	Use1	55850	0.356072204	0.12771
318	77090	Ocel1	79629	0.242890829	0.991
319	109880	Braf	673	-0.702277971	0.33755
320	68198	Ndufb2	4708	0.479586924	0.33655
321	20585	Hltf	6596	0.274221444	0.78869
322	68879	Prpf6	24148	0.415336932	0.11327
323	56470	Rgs19	10287	0.311428831	0.05882
324	106861	Abhd3	171586	0.364711218	0.04968
325	20641	Snrpd1	6632	0.398750366	0.32134
326	77832	Tchp		-0.061364034	0.99988
327	21844	Tiam1	7074	1.036900913	0.01098
328	22084	Tsc2	7249	0.430998813	0.04241
329	65962	Slc9a3r2	9351	0.122931973	0.90774
330	67959	Puf60	22827	-0.079372959	0.99988
331	99412	Golga2		-0.407364909	0.9462
332	22245	Uck1	83549	0.297593511	0.69507
333	20259	Scin	85477	0.99233801	0.28659
334	22781	Ikzf4		0.464531137	0.40477
335	103742	Mien1	84299	-0.145768265	0.99988
336	26362	Axl	558	0.307998509	0.75897
337	52132	Ccdc97	90324	0.728003847	0.04606
338	54194	Akap8l	26993	-1.322886339	0.21409
339	68079	Pdcd2l		0.585573961	0.02862
340	98053	Gtf2f1	2962	-0.562923028	0.42759
341	53895	Clpp	8192	-0.362634604	0.53394
342	66400	Alkbh7	84266	0.558170608	0.02595
343	70785	Dennd1c	79958	0.544070866	0.08965
344	69792	Med6	10001	-0.098376647	0.99988
345	18760	Prkd1	5587	1.13555934	0.22329
346	16822	Lcp2	3937	1.481781872	0.00736
347	110750	Cse1l	1434	0.692204443	0.00888

348	67877	Naa20	51126	0.344801999	0.75363
349	23992	Prkra	8575	0.143239869	0.94082
350	14231	Fkbp7	51661	1.389967352	0.16233
351	83435	Plekha3	65977	0.798858404	0.01372
352	56418	Ykt6	10652	-0.414901473	0.57316
353	22385	Baz1b	9031	0.245957091	0.7614
354	224824	Pex6	5190	-0.55566916	0.33296
355	27398	Mrpl2	51069	-0.68546451	0.34022
356	17256	Mea1	4201	-0.209866992	0.88887
357	14711	Gnmt	27232	-1.480806965	0.13965
358	68137	Kdelr1	10945	0.651165207	0.02391
359	70209	Tmem143	55260	0.981892606	0.26528
360	110175	Ggct		-0.041311222	0.96268
361	16450	Jag2	3714	0.714283499	0.6316
362	399566	Btbd6	90135	-0.072900624	0.99988
363	66174	Nudt14	256281	-0.267791895	0.82965
364	105298	Epdr1	54749	1.38509389	0.12554
365	14248	Flii	2314	0.163421583	0.99988
366	21975	Top3a	7156	0.389315781	0.59996
367	235040	Atg4d	84971	-1.491704605	0.1583
368	60507	Qtrt1	81890	-0.275634218	0.67215
369	57435	Plin4	729359	0.14203786	0.32147
370	15193	Hdgfl2	84717	-0.402754279	0.55816
371	27221	Chaf1a	10036	-0.542955951	0.63615
372	11544	Adprh	141	0.332027704	0.33692
373	67846	Tmem39a	55254	0.015583105	0.99988
374	76916	Timmdc1	51300	0.663915508	0.22364
375	85031	Pla1a	51365	-0.218368626	0.99988
376	17216	Mcm2	4171	-0.982508656	0.28159
377	24100	Tpra1	131601	0.181351635	0.99988
378	17936	Nab1	4664	-0.482139274	0.35585
379	26364	Adgre5	976	1.066689397	0.00972
380	16172	Il17ra	23765	0.312795873	0.40725
381	16777	Lamb1	3912	0.620629241	0.89195
382	84036	Kcnn1	3780	-1.173598421	0.02564
383	70807	Arrdc2	27106	0.940471631	0.40725
384	12491	Cd36	948	-1.72160736	0.09341
385	26400	Map2k7		-0.749737416	0.33176
386	21856	Timm44	10469	-0.253848985	0.64349
387	11772	Ap2a2	161	0.26628213	0.9958
388	59047	Pnkp	11284	0.315482279	0.37451
389	75613	Med25	81857	0.761494515	0.00985
390	56457	Clptm1	1209	-0.828212715	0.28896
391	19698	Relb	5971	0.30957882	0.70264
392	53333	Tomm40	10452	-0.806202959	0.31855
393	11816	Apoe	348	1.544067615	0.00652
394	11813	Apoc2		0.554724878	0.44887
395	73389	Hbp1	26959	-0.185813907	0.99988
396	19088	Prkar2b	5577	-0.59339538	0.41934
397	12576	Cdkn1b	1027	-0.412557175	0.62844

398	16600	Klf4	9314	-0.740475054	0.65408
399	11767	Ap1m1	8907	-0.163399497	0.83693
400	17274	Rab8a	4218	0.686860914	0.06049
401	15331	Hmgn2		0.268612833	0.42215
402	67922	Fam32a	26017	0.210804132	0.97346
403	13710	Elf3	1999	-0.803147934	0.37475
404	76205	Stard3nl	83930	0.839587995	0.06463
405	20869	Stk11	6794	-0.078120879	0.99988
406	66043	Atp5d	513	0.022591268	0.99988
407	19060	Ppp5c	5536	-0.040102142	0.99988
408	69131	Cdk12	51755	-1.052453296	0.23138
409	16890	Lipe	3991	-0.117425183	0.99988
410	18475	Pafah1b2	5049	0.288222123	0.46561
411	54610	Tbc1d8	11138	-0.820054824	0.36011
412	52846	Cnot11	55571	0.594158383	0.15724
413	20527	Slc2a3		0.183083833	0.96934
414	60611	Foxj2	55810	0.145971075	0.82529
415	109552	Sri	6717	0.057408718	0.93342
416	13356	Dgcr2		-0.242951442	0.59175
417	54131	Irf3	3661	0.699684361	0.20884
418	75736	Bcl2l12	83596	0.074788942	0.991
419	224893	Zfp959		-0.71537767	0.35364
420	68047	Mpnd	84954	0.359746243	0.97299
421	20405	Sh3gl1	6455	-0.645300561	0.37246
422	50498	Ebi3	10148	2.17008676	0.00388
423	72886	Yju2	55702	-0.20687826	0.80013
424	19386	Ranbp2	5903	-0.306673108	0.59106
425	14773	Grk5	2869	1.31095641	0.00972
426	13544	Dvl3	1857	0.06197888	0.6489
427	27406	Abcf3	55324	0.013170474	0.99988
428	224045	Eif2b5	8893	-1.078077211	0.17797
429	19158	Cyth2	9266	-0.120678361	0.97905
430	224997	Dlgap1		-0.018323337	0.99988
431	15162	Hck	3055	2.691888192	0.00406
432	66163	Mrpl4	51073	-0.820312429	0.36329
433	50868	Keap1	9817	-0.893017322	0.22364
434	11768	Ap1m2	10053	0.133458164	0.55816
435	20340	Glg1	2734	-0.720508578	0.3432
436	208198	Btbd2	55643	-1.127809289	0.36977
437	103236	Csnk1g2	1455	-1.196281921	0.15193
438	216169	Abhd17a	81926	-0.311848921	0.70199
439	208228	Mob3a	126308	0.251532728	0.80895
440	12297	Cacnb3	784	-1.223703957	0.22535
441	66120	Fkbp11	51303	0.529623416	0.24751
442	74351	Ddx23	9416	-2.39923099	0.05427
443	18807	Pld3	23646	0.442628128	0.63858
444	14809	Grik5	2901	0.446200063	0.41179
445	14470	Rabac1	10567	-0.221814376	0.89993
446	27049	Etv3	2117	-0.189924536	0.99988
447	19089	Prkcsh	5589	0.141579693	0.94663

448	14132	Fcgrt		0.634738198	0.51113
449	66394	Nosip	51070	0.16865247	0.87239
450	68845	Pih1d1	55011	-0.209346831	0.79373
451	27207	Rps11	6205	0.039433888	0.99988
452	20924	Supt5	6829	-1.71638579	0.10328
453	54624	Paf1	54623	0.034350949	0.99988
454	66525	Timm50	92609	-0.080499098	0.89195
455	67224	Med29	55588	-0.276303761	0.8323
456	12121	Bicd1	636	-0.052514107	0.97042
457	59287	Ncstn	23385	0.054680295	0.99988
458	19298	Pex19	5824	0.273730572	0.74429
459	21743	Inmt		0.918868551	0.83403
460	72054	Cyp4f18	4051	1.148317012	0.33858
461	23917	Impdh1	3614	0.528443802	0.31947
462	72349	Dusp3		-0.486799203	0.56842
463	19125	Prodh		-0.108744641	0.99988
464	27886	Ess2	8220	-0.607935548	0.49354
465	13358	Slc25a1	6576	-0.449539731	0.59113
466	13353	Dgcr6		-0.397914122	0.77334
467	12305	Ddr1	780	0.454883955	0.94368
468	15937	Ier3	8870	0.282858185	0.68231
469	74764	Klc4	89953	0.549595258	0.49723
470	13870	Ercc1	2067	-0.11086002	0.99988
471	57344	As3mt	57412	0.250515302	0.82965
472	26558	Homer3		0.19467739	0.64888
473	382056	Crtc1	23373	0.512391323	0.3732
474	71673	Rnf215	200312	-0.016403921	0.99988
475	67815	Sec14l2	23541	-0.721346295	0.38572
476	74268	Aven	57099	0.811135358	0.0123
477	12870	Cp	1356	-2.391816636	0.05227
478	74114	Crot	54677	-0.385465111	0.62133
479	20111	Rps6ka1	6195	-0.107283378	0.89863
480	320632	Snrnp200	23020	0.446806393	0.07326
481	26371	Ciao1	9391	0.384160512	0.43835
482	72999	Insig2	51141	-0.201653636	0.89993
483	16650	Kpna6	23633	-0.13747079	0.90295
484	17155	Man1a	4121	0.210380769	0.63401
485	76889	Coq8b		0.311500612	0.98645
486	78656	Brd8	10902	0.290783669	0.55429
487	19348	Kif20a	10112	-0.192799802	0.93165
488	66590	Farsa	2193	-0.586624699	0.4425
489	270076	Gcdh	2639	-0.62436097	0.47861
490	17776	Mast2	23139	1.043268196	0.251
491	13423	Dnase2a	1777	0.030883881	0.99988
492	12317	Calr	811	-0.291142052	0.53701
493	71846	Syce2	256126	1.098153943	0.01017
494	54446	Nfat5	10725	-0.354378264	0.57946
495	67619	Nob1	28987	0.603896876	0.03935
496	76787	Ppfia3	8541	-0.240402161	0.99639
497	14936	Gys1	2997	-1.302846007	0.17584

498	20174	Ruvbl2	10856	0.41673246	0.60948
499	12028	Bax	581	-0.309158015	0.97706
500	16197	Il7r	3575	0.041097284	0.83725
501	21780	Tfam	7019	-0.118658803	0.80362
502	224694	Zfp81		0.392310243	0.99988
503	67468	Mmd	23531	1.029113845	0.39013
504	217082	Hlf	3131	1.858806242	0.15968
505	70186	Fam162a	26355	-0.27059257	0.57665
506	26961	Rpl8	6132	-0.127337002	0.73955
507	66970	Ssbp2	23635	-0.101655256	0.87534
508	67030	Fancl	55120	0.334632685	0.10147
509	14866	Gstm5	2947	0.738451221	0.02058
510	68312	Gstm7	2946	-1.071799053	0.24606
511	20848	Stat3	6774	-0.722928752	0.35204
512	20850	Stat5a	6776	0.538456436	0.03115
513	19285	Cavin1	284119	0.493091145	0.89517
514	26403	Map3k11	4296	-0.173150822	0.99988
515	11652	Akt2	208	0.452453483	0.24121
516	83945	Dnaja3	9093	0.068258117	0.99988
517	15369	Hmox2	3163	-0.532691465	0.54027
518	66626	Cdip1	29965	-0.724579056	0.32184
519	65964	Map3k20	51776	-0.423210019	0.45126
520	66070	Cwc15	51503	0.207260685	0.46998
521	53867	Col5a3	50509	1.234099212	0.01051
522	13433	Dnmt1	1786	-0.763450801	0.35015
523	235036	Ppan		1.449916639	0.00664
524	26360	Angptl2	23452	2.105396942	0.01009
525	12290	Cacna1e	777	0.397115892	0.93801
526	108943	Trmt10a	93587	0.006158867	0.99988
527	14009	Etv1	2115	0.805777228	0.74744
528	16581	Kifc2	90990	-0.450579558	0.64162
529	19156	Psap	5660	0.718496405	0.01514
530	16151	Ikbkg	8517	1.052686036	0.00795
531	70560	Wars2	10352	0.194246683	0.47275
532	12034	Phb2	11331	-0.511176373	0.40369
533	15170	Ptpn6	5777	0.600706152	0.5248
534	13807	Eno2	2026	-1.123189392	0.29276
535	14791	Emg1	10436	0.194522106	0.82705
536	14792	Lpcat3	10162	0.171962436	0.21141
537	66144	Atp6v1f	9296	-0.204755997	0.88823
538	12728	Clcn5	1184	0.147590913	0.80217
539	12725	Clcn3	1182	-0.772376674	0.33662
540	70683	Utp20	27340	0.198808137	0.98154
541	212448	330159F19Ri	9729	-0.016143938	0.99988
542	26554	Cul3	8452	0.461775242	0.24007
543	16156	Il11	3589	1.361237725	0.00692
544	16795	Large1	9215	-0.10442545	0.96578
545	52513	Ddx56	54606	-0.124873218	0.99988
546	103694	Tmed4	222068	-0.167060437	0.64148
547	12122	Bid	637	-0.108946791	0.99988

548	64143	Ralb	5899	-0.93364638	0.14438
549	19047	Ppp1cc	5501	0.157173348	0.15728
550	67838	Dnajb11		-0.197703511	0.99656
551	70573	Tbccd1	55171	-0.198196904	0.95765
552	20256	Clec11a	6320	2.107457977	0.00388
553	243834	Zfp324		0.348642644	0.70776
554	14389	Gab2	9846	0.705960607	0.33468
555	23790	Coro1c	23603	0.297549359	0.71384
556	52440	Tax1bp1	8887	-1.225709002	0.14086
557	29811	Ndrg2	57447	0.080367997	0.99988
558	52535	Mettl17	64745		0.40878
559	268739	Arhgef40	55701	0.270762658	0.60364
560	50767	Pnpla6	10908	-0.434990939	0.50414
561	94178	Mcoln1	57192	0.445024831	0.5826
562	102098	Arhgef18	23370	-0.083026582	0.93165
563	109333	Pkn2	5586	-1.127562515	0.18088
564	12489	Cd33			0.40878
565	110826	Etfb		-0.132522362	0.99816
566	72310	Nkg7	4818	0.184574582	0.75663
567	20911	Stxbp2	6813	-0.331773002	0.68231
568	20392	Sgce	8910	0.731466097	0.91896
569	69993	Chn2		1.744915721	0.00407
570	80707	Wwox	51741	0.291185996	0.66436
571	20492	Slbp	7884	0.394337606	0.43031
572	11826	Aqp1		0.785964011	0.22875
573	56380	Arid3b	10620	-0.261771347	0.73522
574	12798	Cnn2	1265	0.990389097	0.00926
575	66420	Polr2e	5434	0.070963712	0.9861
576	17925	Myo9b	4650	-0.37203339	0.78261
577	79221	Hdac9	9734	-2.025639028	0.06039
578	17085	Ly9	4063	0.801836762	0.01181
579	18106	Cd244a	51744	0.973296534	0.24324
580	13733	Adgre1	2015	2.601237222	0.00388
581	67900	Mtfp1	51537	-0.055156444	0.99988
582	19335	Rab23	51715	-0.61915752	0.54312
583	53869	Rab11a	8766	0.410875619	0.68357
584	217715	Eif2b2	8892	-0.332334489	0.60462
585	78920	Dlst	1743	0.110343578	0.97706
586	29869	Ulk2	9706	1.25982195	0.0062
587	56221	Ccl24	6369	0.783816016	0.1958
588	71520	Grap		1.331230864	0.1497
589	68942	Chmp2b	25978	-0.586073333	0.46392
590	26433	Plod3	8985	0.61949327	0.01821
591	11769	Ap1s1	1174	-0.563356955	0.48195
592	26415	Mapk13	5603	-1.08598802	0.20334
593	20815	Srpk1	6732	-0.38454652	0.45649
594	18008	Nes	10763	0.065678367	0.99988
595	229503	Rrnad1	51093	0.167745268	0.8023
596	15191	Hdgf	3068	-0.668687171	0.23085
597	50492	Thop1	7064	-1.249273907	0.11536

598	57267	Apba3	9546	0.334024396	0.77068
599	17179	Matk	4145	-0.596434093	0.49863
600	59004	Pias4	51588	-0.716854336	0.35685
601	26395	Map2k1	5604	0.438028615	0.09527
602	52551	Sgta	6449	0.224669664	0.79198
603	70544	Tmem242	729515	0.507745598	0.02435
604	74198	Dtx2	113878	0.374368814	0.04663
605	15507	Hspb1	3315	-0.297752703	0.99988
606	54153	Rasa4		1.2504935	0.01591
607	53379	Hnrnpa2b1	3181	-0.016550372	0.99988
608	108017	Fxyd4	53828	-0.025018972	0.98477
609	67736	Ccdc130	81576	1.429913419	0.16494
610	67873	Mri1	84245	-0.479968568	0.78869
611	18749	Prkacb	5567	-0.398618845	0.91977
612	27029	Sgsh	6448	-0.694515131	0.44838
613	13014	Cstb	1476	0.315844337	0.41601
614	23921	Sh2b2	10603	0.12989913	0.99988
615	19305	Pex5	5830	-0.189639179	0.99988
616	104771	Jkamp		0.679016215	0.03205
617	12505	Cd44	960	-0.385938207	0.60189
618	20511	Slc1a2	6506	-1.321449389	0.01917
619	27103	Eif2ak4	440275	-0.164990199	0.75363
620	22388	Wdr1	9948	0.049006924	0.99988
621	117591	Slc2a9	56606	0.236694089	0.43562
622	22402	Ccn4	8840	1.534825458	0.00722
623	17988	Ndrg1	10397	-0.135338346	0.99988
624	17159	Man2b1		0.612888567	0.28159
625	12224	Klf5	688	-0.324796798	0.78632
626	67836	Wdr83		-0.109230037	0.75903
627	21672	Prdx2	7001	-0.280251766	0.6522
628	20020	Polr2a	5430	0.536339456	0.02031
629	80886	Senp3		-2.055124701	0.08542
630	231999	Plekha8	84725	-0.409410941	0.73305
631	66442	Spc25	57405	-1.653569816	0.20731
632	72388	Ripk4	54101	-0.290118817	0.50058
633	22230	Ufd1	7353	-0.199231479	0.85956
634	170740	Zfp287	57336	0.547104286	0.65381
635	19116	Prlr		0.890709198	0.27188
636	56384	Letm1	3954	-0.076660386	0.991
637	56085	Ubqln1	29979	-0.1732764	0.86369
638	94332	Cadm3	57863	0.370756579	0.66485
639	56551	Txn2	25828	0.075439348	0.90772
640	20512	Slc1a3	6507	0.072598075	0.8354
641	58799	Crbn	51185	0.715590837	0.05478
642	17688	Msh6	2956	0.375781843	0.62766
643	225055	Fbxo11	80204	0.245827351	0.95306
644	27368	Tbl2	26608	-1.605761972	0.21336
645	66138	Bud23	114049	-0.760014374	0.2856
646	18073	Nid1	4811	0.057158331	0.99988
647	17218	Mcm5	4174	-0.684573856	0.37763

648	15368	Hmox1	3162	0.726942786	0.02147
649	26936	Mprip	23164	-0.783554689	0.33176
650	71722	Cic	23152	-0.548980714	0.36647
651	18476	Pafah1b3	5050	-0.601935998	0.35486
652	18747	Prkaca	5566	0.051268564	0.99988
653	66929	Asf1b	55723	-0.722479326	0.22012
654	68278	Ddx39a	10212	-0.284402468	0.48431
655	81489	Dnajb1	3337	-0.016633266	0.99988
656	227334	Usp40	55230	-0.241881988	0.99988
657	13046	Celf1	10658	-0.584957497	0.3405
658	68349	Ndufs3	4722	0.658442059	0.03963
659	18984	Por	5447	0.12868379	0.84786
660	16001	Igf1r	3480	-0.419783349	0.65635
661	16337	Insr	3643	0.003583604	0.99988
662	13087	Cyp2a5			0.991
663	21849	Trim28	10155	-0.101662498	0.99988
664	22192	Ube2m	9040	-0.988762862	0.33704
665	11515	Adcy9	115	-0.09205268	0.98415
666	17260	Mef2c	4208	1.223959187	0.11192
667	22083	Ctr9	9646	-1.161038173	0.16841
668	13690	Eif4g2	1982	-0.155012935	0.89876
669	17540	Irag1	10335	1.353984706	0.00926
670	233410	Zfp592	9640	0.190656524	0.75992
671	19185	Psmd4	5710	-0.696107354	0.42547
672	72183	Snx6	58533	0.386080689	0.35532
673	17768	Mthfd2	10797	-0.93023101	0.27467
674	16590	Kit	3815	-0.637248019	0.50521
675	641376	Tomm40l	84134	0.436265163	0.67222
676	103135	Pan2		0.191476391	0.91487
677	12974	Cs		-0.135579378	0.75567
678	11717	Ampd3	272	-0.855070719	0.60118
679	68183	Bcas2	10286	-0.178590966	0.52811
680	13018	Ctcf	10664	-0.263814184	0.50116
681	56513	Pard6a	50855	1.333811225	0.15302
682	19385	Ranbp1	5902	0.285018251	0.94681
683	12503	Cd247	919		0.991
684	53970	Rfx5	5993	0.812040134	0.67877
685	19172	Psmb4	5692	0.475033377	0.28324
686	17394	Mmp8	4317	0.264554233	0.98765
687	22785	Slc30a4	7782	-0.138681933	0.99988
688	59010	Sqor		-0.408007854	0.74422
689	18457	Bloc1s6	26258	0.421689943	0.04193
690	75624	Metap1	23173	-0.759756986	0.30317
691	78308	Gpr108	56927	-0.037291029	0.99988
692	14465	Gata6	2627	0.023796842	0.99988
693	66409	Rsl1d1	26156	-0.401433437	0.92014
694	11789	Apc	324	0.046825657	0.99988
695	13476	Reep5	7905	0.367674575	0.04508
696	66366	Ergic3	51614	0.143456638	0.99988
697	56046	Uqcc1	55245	0.4354961	0.04018

698	17978	Ncoa2	10499	0.653812458	0.23715
699	22026	Nr2c2	7182	-0.410231742	0.63685
700	22025	Nr2c1	7181	-1.740495631	0.11022
701	77626	Smpd4	55627	-0.726893309	0.33662
702	71382	Pex1	5189	0.325614592	0.35294
703	66989	Kctd20	222658	0.020786121	0.99053
704	16407	Itgae	3682	1.110017292	0.15062
705	83429	Ctns	1497	-0.155527595	0.8401
706	74637	Shpk		-0.086502124	0.96229
707	13845	Ephb3	2049	1.511967468	0.22712
708	22156	Tuft1	7286	-0.489508249	0.45333
709	19672	Rcn1		0.140398267	0.94475
710	68015	Trap1	10131	0.062696822	0.99988
711	73261	700037C18R	646174	-0.776121928	0.36752
712	68423	Ankrd13d	338692	0.173657109	0.99988
713	108989	Tpr	7175	0.216151421	0.63927
714	226499	Odr4	54953	-0.293144406	0.73182
715	96875	Prg4	10216	0.340587851	0.37924
716	71723	Dhx34	9704	1.021827782	0.45499
717	70394	Kptn	11133	-0.401445383	0.99988
718	108124	Napa	8775	0.113946372	0.98675
719	24068	Sra1	10011	-0.234734967	0.72073
720	11951	Atp5g1		-0.350430868	0.73818
721	27681	Snf8	11267	-0.301672883	0.43236
722	66411	Tbcb	1155	-0.015954202	0.99988
723	19062	Inpp5k	51763	-0.386329313	0.99988
724	12929	Crkl	1399	-1.051406426	0.32582
725	67425	Eps8l1	54869	-2.234346978	0.05165
726	216705	Clint1	9685	-1.952132492	0.05186
727	68916	Cdkal1	54901	-0.770087031	0.30415
728	56213	Htra1	5654	0.542126998	0.62454
729	22642	Zbtb17	7709	-1.892994733	0.11172
730	74202	Fblim1	54751	1.677503641	0.0075
731	68473	Mob1b	92597	-0.436550286	0.50535
732	110935	Atp6v1b1	525	-0.040766892	0.95917
733	11966	Atp6v1b2	526	0.839560793	0.01439
734	13859	Eps15l1	58513	0.098616571	0.90372
735	21745	Tep1	7011	0.734478367	0.20884
736	219022	Ttc5	91875	-0.217623285	0.99988
737	66246	Osgep	55644	0.662321004	0.02164
738	227290	Aamp	14	-0.455297993	0.59944
739	69660	Tmbim1	64114	0.191970271	0.40287
740	76709	Arpc2	10109	-0.50167628	0.42673
741	75410	Kmt2b	9757	-1.22071018	0.18935
742	58206	Zbtb32	27033	-0.673101066	0.57912
743	69804	Tmem147	10430	0.786738338	0.18486
744	76846	Rps9	6203	-0.130968845	0.77624
745	69714	Tfpt	29844	0.269068049	0.95504
746	71733	Susd2	56241	-0.411795511	0.71896
747	23887	Ggt5	2687	0.159723576	0.99988

748	14598	Ggt1		0.029660482	0.99988
749	68337	Crip2	1397	-0.869593619	0.27609
750	12925	Crip1		1.527101314	0.00837
751	12398	Cbfa2t3	863	1.363505818	0.0074
752	14114	Fbln1	2192	0.633183945	0.58023
753	53328	Pgrmc1	10857	0.258930588	0.13953
754	26912	Gcat		-0.515011889	0.41296
755	21687	Tek	7010	0.853399998	0.45958
756	54325	Elovl1	64834	-1.000897558	0.24892
757	80509	Med8	112950	0.248852725	0.07394
758		Hyi	81888	0.069500775	0.80354
759	107995	Cdc20	991	-0.813655255	0.30098
760	240913	Adamts4	9507	0.157870327	0.80895
761	71740	Nectin4	81607	-0.16811801	0.79387
762	18637	Pfdn2	5202	-0.136098454	0.84336
763	81018	Rnf114	55905	-0.128178076	0.9519
764	77644	330007P06R	63932	-0.994768687	0.21926
765	18011	Neurl1a	9148	0.221651567	0.99988
766	20810	Srm	6723	-0.652879703	0.33437
767	56275	Rbm14	10432	0.652090127	0.81086
768	11474	Actn3	89	2.83798337	0.03824
769	70605	Zdhhc24	254359	0.428127315	0.0912
770	52028	Bbs1		0.207047798	0.96072
771	78797	Ndor1		-0.867643824	0.33293
772	56876	Nsmf	26012	-1.510962148	0.0776
773	228026	Pdk1	5163	-0.684289628	0.24003
774	192156	Mvd	4597	-1.187079156	0.25415
775	13057	Cyba	1535	0.847253808	0.01197
776	69179	Stimate		-1.09698885	0.27564
777	54650	Sfmbt1	51460	-0.767663979	0.32582
778	20536	Slc4a3	6508	-0.902239011	0.41652
779	67177	Cdt1	81620	-0.098521984	0.96363
780	12395	Runx1t1	862	0.597109795	0.89785
781	11821	Aprt	353	-0.230225942	0.69975
782	14884	Gtf2h1	2965	-0.267837247	0.80268
783	15216	Hfe	3077	1.568979002	0.00621
784	57742	Abhd1	84696	0.179626839	0.24843
785	330064	Slc5a6	8884	0.21261474	0.99988
786	69852	Tcf23	150921	0.798743213	0.2123
787	69232	Qrich1	54870	-0.328379469	0.09426
788	71472	Usp19	10869	0.023974071	0.99916
789	18968	Pola1	5422	-0.09233211	0.90528
790	12540	Cdc42	998	0.567358088	0.21278
791	18771	Pknox1	5316	-1.649350298	0.10463
792	57441	Gmnn	51053	-0.265633723	0.99988
793	66834	Acot13	55856	0.614213794	0.30147
794	12567	Cdk4	1019	0.116095698	0.99988
795	14421	B4galnt1	2583	0.064088184	0.99988
796	17299	Mettl1	4234	-0.149775604	0.97856
797	16573	Kif5b	3799	-0.745708211	0.20884

798	78935	Saal1	113174	-0.775481257	0.34612
799	94179	Krt23	25984	-0.268805932	0.99988
800	12799	Cnp	1267	0.134824324	0.84344
801	74407	Odad4	83538	0.688276254	0.57917
802	72043	Sulf2	55959	1.100632813	0.01105
803	20656	Sod2	6648	0.646174355	0.0147
804	70527	Stambp	10617	0.27922114	0.07274
805	14055	Ezh1	2145	0.157074906	0.81665
806	15114	Hap1	9001	-1.504070713	0.22629
807	66180	P3h4	10609	-0.229833474	0.81414
808	12387	Ctnnb1	1499	-1.007548315	0.27029
809	68969	Eif1b	10289	0.783859193	0.01279
810	12667	Chrd	8646	0.674349609	0.45367
811	21762	Psmd2	5708	-1.032437405	0.11022
812	22321	Vars	7407	-0.951398799	0.24865
813	70129	Slc44a4	80736	0.525841411	0.10438
814	193742	Abhd16a	7920	0.306493859	0.15273
815	18010	Neu1	4758	0.055386084	0.99988
816	51793	Ddah2	23564	0.310830122	0.01706
817	114584	Clic1	1192	-0.079533008	0.97082
818	27756	Lsm2	57819	-0.205810987	0.84346
819	98660	Atp1a2	477	0.444719359	0.95477
820	243372	Zfp775		0.921493132	0.22364
821	18120	Mrpl49	740	0.05042166	0.99988
822	329727	Dennd2c	163259	-0.141221499	0.91673
823	17169	Mark3	4140	-0.523030293	0.45862
824	67210	Gatad1	57798	0.482824684	0.02647
825	17113	M6pr	4074	-0.160742456	0.88714
826	241296	Lrrc8a		0.481258587	0.2523
827	51792	Ppp2r1a	5518	-0.536974	0.43106
828	72775	Fance	2178	0.280327364	0.8367
829	28113	Tinf2	26277	0.327379832	0.88748
830	224907	Dus3l		0.281752786	0.53157
831	70359	Gtpbp3	84705	0.40486866	0.48543
832	21812	Tgfbr1	7046	0.219852332	0.86524
833	26556	Homer1	9456	-0.13633319	0.9946
834	12389	Cav1	857	1.785727516	0.00647
835	59046	Arpp19	10776	-0.612328523	0.34082
836	12048	Bcl2l1	598	-0.431581932	0.55204
837	16549	Khsrp	8570	-0.155114677	0.99716
838	13371	Dio2	1734	0.298239709	0.56261
839	234388	Ccdc124	115098	-0.417348475	0.74289
840	12464	Cct4	10575	0.537055285	0.03205
841	66050	610009B22Rik		-0.16511311	0.99988
842	78070	Cpt1c	126129	-0.416547356	0.99053
843	13345	Twist2	117581	-1.05734356	0.18426
844	72611	Zfp655	79027	0.039988103	0.93969
845	11848	Rhoa	387	0.066584813	0.90192
846	328365	Zmiz1	57178	0.171918925	0.77302
847	232339	Ankrd26		-1.212990038	0.23208

848	69748	Aldh16a1	126133	0.238377042	0.19726
849	77134	Hnrnpa0	10949	0.444629753	0.03618
850	65116	Prrg2		-0.668363613	0.37529
851	59013	Hnrnph1	3187	-0.103737219	0.99988
852	76411	Ift43	112752	0.151668795	0.83925
853	15903	Id3	3399	1.417916486	0.01952
854	93760	Arid1a	8289	-0.686050663	0.29776
855	12931	Crlf1	9244	2.00473332	0.13092
856	13033	Ctsd		-0.391932809	0.80284
857	56040	Rplp1		-0.264058472	0.76163
858	64296	Abhd8	79575	0.801654156	0.00959
859	67286	Ift22	64792	-0.785363533	0.45457
860	14365	Fzd3	7976	0.584123837	0.52366
861	68041	Mid1ip1	58526	-0.89814909	0.24307
862	232910	Ap2s1	1175	-0.349273735	0.79274
863	116701	Fgfrl1	53834	0.342729159	0.92577
864	14200	Fhl2	2274	0.848155073	0.01441
865	69683	Emc10	284361	-0.05854621	0.99988
866	68628	Fbxw9		-1.082793065	0.2287
867	55935	Fnbp4	23360	-0.637867178	0.35764
868	67260	Cers4	79603	0.994594558	0.14103
869	74616	Scrn3	79634	0.099748507	0.89543
870	56698	Phax	51808	0.208877687	0.82416
871	21885	Tle1	7088	-0.034117622	0.99988
872	320100	Relt	84957		0.40878
873	20639	Snrpb2	6629	0.307126068	0.56363
874	22190	Ubc	7316	-0.301848611	0.91547
875	68988	Prpf31	26121	-0.220505696	0.99988
876	55942	Sertad1	29950	0.192663933	0.98119
877	52502	Carhsp1	23589	-0.562481912	0.34818
878	13713	Elk3	2004	0.365890819	0.53504
879	80517	Herpud2	64224	0.940769868	0.72508
880	108841	Rdh13	112724	-0.55595319	0.57622
881	67771	Arpc5	10092	0.006860578	0.99988
882	18987	Pou2f2	5452	0.564811025	0.991
883	56615	Mgst1	4257	0.515776844	0.07801
884	18028	Nfib	4781	-0.961692613	0.20706
885	53868	Rab25	57111	-0.939591777	0.4272
886	94232	Ubqln4	56893	-0.232586369	0.76264
887	20084	Rps18		0.383087278	0.31884
888	110954	Rpl10		0.101695639	0.76087
889	267019	Rps15a	6210	0.293073931	0.69565
890	52683	Ncaph2	29781	-0.444396062	0.42043
891	15257	Hipk1	204851	0.599433329	0.18514
892	64297	Gprc5b	51704	-0.695794683	0.80792
893	17156	Man1a2	10905	-0.285281266	0.67074
894	66204	Acyp1		0.566530649	0.72329
895	93671	Cd163	9332	2.960173248	0.00388
896	15184	Hdac5	10014	0.182957484	0.9575
897	56044	Rala	5898	-0.249072479	0.52678

898	22335	Vdac3	7419	-0.02965828	0.99988
899	21427	Vps72	6944	-0.554281995	0.82807
900	14390	Gabpa	2551	0.047167711	0.68253
901	12162	Bmp7	655	0.062328809	0.991
902	56455	Dynll1		0.375932997	0.47454
903	67466	Pdcl	5082	-0.02435968	0.97395
904	223693	Tmem184b	25829	0.149576114	0.81777
905	18016	Nf2	4771	-0.760399372	0.4352
906	67178	Zmat5	55954	0.586178034	0.58979
907	14030	Ewsr1	2130	-0.030311179	0.99988
908	11764	Ap1b1	162	0.341488872	0.40001
909	70377	Derl3	91319		0.991
910	94044	Bcl2l13	23786	-0.528694402	0.3757
911	93838	Dqx1	165545	0.047405645	0.94878
912	20307	Ccl8		3.660125406	0.00356
913	69605	Lnpk	80856	-0.120874344	0.99988
914	71660	Rarres2	5919	1.469222108	0.00908
915	108705	Pttg1ip	754	0.520489117	0.21413
916	28240	Trpm2	7226	0.52126294	0.34186
917	22213	Ube2g2	7327	0.110885269	0.99988
918	17295	Met	4233	0.19410247	0.71878
919	240638	Slc16a12	387700	-1.243248374	0.20272
920	20965	Syn2	6854		0.40878
921	13712	Elk1	2002	0.610457281	0.28058
922	215690	Nav1	89796	0.462415242	0.03517
923	238799	Tnpo1	3842	0.060043262	0.99988
924	67897	Rnmt	8731	-0.315619567	0.58964
925	16535	Kcnq1	3784	0.900233853	0.01098
926	20813	Srp14	6727	0.330707984	0.29219
927	107951	Cdk9	1025	-0.334304828	0.66697
928	30933	Tor2a	27433	-0.410090263	0.45517
929	14287	Fpgs	2356	-0.483111624	0.72791
930	239719	Mrtfb	57496	-0.445464344	0.4703
931	12419	Cbx5		-0.220859307	0.64162
932	80287	Apobec3	164668	-0.129552751	0.73642
933	192166	Sardh	1757	0.207556959	0.99988
934	22325	Vav2	7410	1.128068785	0.01089
935	14373	G0s2	50486	1.010749573	0.3352
936	66233	Dmap1	55929	0.069892671	0.99988
937	215999	Mcu	90550	-0.395055223	0.55424
938	110279	Bcr	613	-0.380241095	0.58979
939	18301	Fxyd5		1.297946947	0.00722
940	21422	Tfcp2	7024	0.255540735	0.16953
941	19009	Pou6f1	5463	-0.059111371	0.98595
942	22221	Ubp1	7342	0.10060682	0.99988
943	74137	Nuak2	81788	-0.220682343	0.99988
944	56542	Cilk1	22858	0.067266945	0.99988
945	67680	Sdhb	6390	0.603608861	0.01042
946	67826	Snap47	116841	-0.623556396	0.47385
947	70750	Kdsr	2531	-0.186468234	0.80808

948	20479	Vps4b	9525	-0.24680584	0.59578
949	75617	Rps25	6230	0.270703371	0.41537
950	66826	Taz	6901	0.121628581	0.99988
951	11671	Aldh3a2	224	-0.276489202	0.75363
952	56395	Tmem115	11070	0.044598202	0.99988
953	15587	Hyal2	8692	0.187435035	0.99058
954	15586	Hyal1	3373	-0.91610473	0.1946
955	80385	Tusc2	11334	0.835616118	0.22923
956	56032	Nprl2	10641	0.056313263	0.99988
957	56289	Rassf1	11186	-0.042440198	0.97042
958	71889	Epn3	55040	-1.01739839	0.2696
959	17254	Slc3a2	6520	-0.376189147	0.52374
960	53319	Nxf1	10482	-0.549321903	0.4844
961	56389	Stx5a	6811	-0.361386235	0.7835
962	57916	Tnfrsf13b	23495	0.278119855	0.89543
963	19130	Prox1	5629	1.774324641	0.00594
964	72503	610507B11Ri	9703	0.414930157	0.30144
965	98404	AI597479	79074	-0.263591396	0.58337
966	67893	Tmem86a	144110	0.953430555	0.62665
967	70110	Ifi35	3430	-0.199988225	0.89833
968	66599	Rdm1	201299	1.116812207	0.10704
969	18002	Nedd8	4738	0.46811204	0.40021
970	53334	Gosr1	9527	-0.048497287	0.97541
971	68836	Mrpl52	122704	0.04340596	0.9542
972	226976	Kansl3	55683	0.665972379	0.03226
973	14051	Eya4	2070	-0.152613543	0.9806
974	13593	Ebf3	253738	-0.263982534	0.99356
975	14084	Faf1	11124	-0.628123733	0.52678
976	67493	Mettl16	79066	0.151025702	0.99988
977	75761	Apol7a		-0.603115032	0.30315
978	66268	Pigyl	84992	0.642321873	0.3222
979	67039	Rbm25	58517	-1.12094884	0.22722
980	19165	Psen2		0.328751065	0.33125
981	235674	Acaa1b		1.317709463	0.01364
982	18799	Plcd1	5333	0.628522491	0.05663
983	76267	Fads1	3992	2.297218966	0.00544
984	56873	Lmbr1	64327	-1.125159107	0.18016
985	27267	Cars	833	-0.851429723	0.19329
986	22113	Phlda2	7262	-0.293586613	0.98124
987	22413	Wnt2	7472	0.95452811	0.00994
988	105785	Kdelr3	11015	-0.968895379	0.22163
989	56369	Apip	51074	1.064777331	0.14324
990	27402	Pdhx	8050	0.775956247	0.09341
991	234729	Vac14	55697	-0.694208307	0.28043
992	68279	Mcoln2	255231	0.399627775	0.991
993	20537	Slc5a1	6523	-0.504833928	0.42355
994	67605	Akt1s1	84335	-0.220120793	0.69277
995	21376	Tbrg1	84897	-0.116462771	0.87452
996	11565	Adssl1	122622	0.770928807	0.32983
997	72308	Brf1	2972	0.215000168	0.69432

998	18263	Odc1	4953	0.45535996	0.03679
999	66628	Thg1l	54974	0.746293506	0.79574
1000	11492	Adam19		-0.082203804	0.9534
1001	230721	Pabpc4	8761	-0.879337572	0.2496
1002	63872	Zfp296	162979	0.050973413	0.99988
1003	70616	Sugp1	57794	0.41443819	0.78574
1004	71755	Dhdh		0.776465786	0.01936
1005	70300	Fuz	80199	-0.744957245	0.69159
1006	18648	Pgam1		-0.138459239	0.74847
1007	14020	Evi5	7813	-0.485112231	0.30315
1008	213027	Evi5l	115704	0.232098188	0.85685
1009	102209	Snapc2	6618	-1.098063179	0.29236
1010	216963	Git1	28964	0.236055484	0.82065
1011	56356	Gltp	51228	-0.414516125	0.69601
1012	12175	Bnip2	663	0.472498671	0.03506
1013	12455	Ccnt1	904	-0.96254851	0.27456
1014	224024	Scarf2	91179	-0.132177199	0.99988
1015	52592	Brms1l	84312	-0.540757595	0.82658
1016	94112	Med15	51586	-1.406000823	0.16168
1017	67422	Dhdds	79947	0.08963719	0.99988
1018	230806	Crybg2	55057	0.187283377	0.99988
1019	67586	Ubxn11	91544	0.930029854	0.01483
1020	74094	Tjap1	93643	0.729095327	0.19738
1021	13661	Ehf	26298	-1.386131034	0.13341
1022	66480	Rpl15	6138	0.090659564	0.99988
1023	66074	Tmem167	153339	0.049205131	0.97297
1024	117167	Steap4	79689	0.661640563	0.56527
1025	66308	Mplkip		-0.638371258	0.63266
1026	16551	Kif11	3832	0.846295404	0.2775
1027	68240	Rpa3	6119	-1.155140053	0.23496
1028	74568	Mlkl	197259	-0.426192342	0.62053
1029	320938	Tnpo3	23534	-0.569089658	0.39272
1030	320244	Ttll5		-0.411610489	0.68954
1031	69930	Zfp715		0.360338792	0.33906
1032	20103	Rps5	6193	0.113577532	0.85722
1033	330814	Adgrl1	22859	-1.597558406	0.16289
1034	75723	Amotl1	154810	-0.01102898	0.99988
1035	104156	Etv5	2119	1.114674103	0.01649
1036	70575	Gfod2	81577	-0.491613312	0.85339
1037	102124	Enkd1	84080	-0.715955908	0.49697
1038	11972	Atp6v0d1	9114	0.260284805	0.99197
1039	19280	Ptprs	5802	0.201362604	0.81989
1040	98396	Slc41a1	254428	-0.088056361	0.78079
1041	74562	Fer1l4		0.478094326	0.95167
1042	270210	Zfp651	92999	0.530923085	0.38013
1043	58202	Nelfb	25920	0.007027243	0.99988
1044	72392	Tmem175	84286	0.36629909	0.33231
1045	27883	Tango2	128989	-0.197984106	0.991
1046	19378	Aldh1a2	8854	0.025066601	0.99053
1047	226646	Ndufs2	4720	0.351114352	0.04608

1048	381629	Atraid	51374	-0.791805535	0.41784
1049	69719	Cad	790	-0.692533737	0.35526
1050	70163	Lypd8		3.628007587	0.00388
1051	79566	Sh3bp5l	80851	-0.300719807	0.81206
1052	67979	Atad1	84896	-0.128255967	0.99988
1053	19211	Pten	5728	0.346078087	0.04673
1054	18611	Pea15a	8682	0.343240038	0.65158
1055	53600	Timm23		0.649262107	0.03743
1056	69769	Tnfaip8l2	79626	0.788908335	0.20337
1057	70047	Trnt1	51095	-0.670517827	0.33174
1058	70274	Ly6g6e		0.960123389	0.03656
1059	110147	Ehmt2	10919	-0.12707222	0.99039
1060	66126	Elof1	84337	0.03256916	0.99988
1061	216154	Med16	10025	-0.824284324	0.24605
1062	20442	St3gal1	6482	1.555280565	0.0074
1063	216157	Tmem259	91304	-0.11657548	0.95694
1064	77733	Rnf170		-0.013817237	0.96811
1065	76686	Clip3	25999	-1.364688437	0.19626
1066	69189	Mcemp1	199675	1.263260829	0.00651
1067	27045	Nit1	4817	0.37244536	0.0453
1068	114604	Prdm15	63977	0.293174245	0.75395
1069	70238	Rnf168	165918	0.524930479	0.30442
1070	66061	Dynlt2b		0.928385495	0.01269
1071	56398	Chp1	11261	0.327539578	0.04083
1072	63873	Trpv4	59341	0.976397022	0.18296
1073	100503085	Klhl3	26249	-0.534510116	0.62871
1074	67510	Tvp23b		0.990058728	0.00934
1075	56354	Dnajc7	7266	-0.552832197	0.4295
1076	12301	Cacybp	27101	-0.021465698	0.99988
1077	76779	Cluap1		0.16949144	0.95986
1078	69747	Zswim7	125150	1.371974916	0.32375
1079	17991	Ndufa2	4695	0.180580458	0.97042
1080	66449	Pam16		0.156738051	0.89246
1081	83396	Glis2	84662	1.228137224	0.28119
1082	12864	Cox6c	1345	0.774602698	0.01366
1083	83675	Bicc1	80114	0.243381135	0.991
1084	268470	Ube2z	65264	-0.141252526	0.68128
1085	72477	Tmem87b	84910	-1.050717767	0.22582
1086	17222	Anapc1	64682	-0.694096942	0.5449
1087	17289	Mertk	10461	1.090491952	0.00577
1088	22088	Tsg101	7251	-0.521650911	0.44695
1089	246694	Hps5	11234	0.866885862	0.01497
1090	26407	Map3k4	4216	0.470767248	0.43236
1091	68718	Rnf166	115992	0.759936575	0.01728
1092	105522	Ankrd28	23243	-0.238571675	0.60111
1093	66384	Srp19	6728	-0.084474657	0.98392
1094	268752	Wdfy2	115825	0.11977608	0.991
1095	78287	Rbsn	64145	0.745805103	0.02079
1096	64658	Mrps25	64432	-0.002531539	0.99988
1097	27369	Dguok	1716	0.652393577	0.80184

1098	100072	Camta1	23261	1.040860614	0.12199
1099	12977	Csf1	1435	0.703622388	0.32717
1100	229707	Strip1	85369	-0.223870784	0.61797
1101	67863	Slc25a11	8402	0.399525781	0.0355
1102	66531	Cmc2		-0.618674331	0.55816
1103	231600	Chfr	55743	-0.844359752	0.40118
1104	13522	Adam28	10863	0.847263423	0.19565
1105	21766	Tex261	113419	-0.388640915	0.67674
1106	67544	Fam120b	84498	-0.293564398	0.7614
1107	21374	Tbp	6908	-0.347102364	0.68231
1108	19170	Psmb1	5689	0.152173489	0.52103
1109	18567	Pdcd2	5134	-0.085560443	0.99988
1110	13388	Dll1	28514	0.33806187	0.49641
1111	78688	Nol3	8996	1.332968442	0.01322
1112	234686	Fhod1	29109	-0.19211002	0.89867
1113	277973	Slc9a5	6553	-0.291775757	0.64119
1114	234683	Elmo3	79767	-0.121376358	0.92082
1115	20855	Stc1	6781		0.40878
1116	17686	Msh3	4437	-0.307507485	0.76504
1117	66320	Tmem208	29100	-0.304824524	0.5948
1118	20932	Surf4	6836	0.21037378	0.47385
1119	20931	Surf2	6835	-0.684818428	0.56514
1120	27362	Dnajb9	4189	1.247029535	0.00647
1121	234344	Naf1		0.837666288	0.18935
1122	22612	Yes1	7525	0.337961571	0.69581
1123	19046	Ppp1cb	5500	-0.728188288	0.26685
1124	70231	Gorasp2	26003	-0.577824964	0.34451
1125	66637	Tsen15	116461	0.617292828	0.02454
1126	76740	Efr3a	23167	-0.237170487	0.66285
1127	59005	Trappc2l	51693	-0.048290001	0.99988
1128	257633	Acsf3	197322	0.186092052	0.99988
1129	13680	Ddx19a	55308	0.368531294	0.79179
1130	50917	Galns	2588	0.158290671	0.48431
1131	14461	Gata2	2624	1.478269542	0.0244
1132	12496	Entpd2	954	0.895040856	0.22917
1133	227624	Rabl6	55684	-0.569346827	0.59996
1134	59022	Edf1	8721	0.35396537	0.72244
1135	18146	Npdc1	56654	-0.565906913	0.2856
1136	30839	Fbxw5	54461	0.189722188	0.48558
1137	50799	Slc25a13	10165	1.000725106	0.14324
1138	22196	Ube2i	7329	-1.343788863	0.06441
1139	68327	Tsr3	115939	1.068762217	0.00985
1140	74154	Unkl	64718	1.059117721	0.31607
1141	233328	Lrrk1	79705	-0.899865393	0.28119
1142	56847	Aldh1a3	220	0.253781453	0.99988
1143	109711	Actn1	87	0.140243177	0.99988
1144	64383	Sirt2		0.401531072	0.02821
1145	15388	Hnrnpl		-0.468487906	0.48698
1146	70769	Nolc1	9221	-0.160587813	0.63356
1147	213819	Casd1	64921	0.679967758	0.01855

1148	215748	Cnksr3		0.398275017	0.04341
1149	53332	Mtmr1		-0.456591684	0.59186
1150	15354	Hmgb3		-0.483041734	0.52953
1151	17756	Map2	4133	-0.28223955	0.991
1152	74519	Cyp2j9		0.620149324	0.0513
1153	11303	Abca1	19	0.133065685	0.9281
1154	66536	Nipsnap3b		0.383880177	0.25338
1155	66192	Lage3	8270	-0.395760576	0.62557
1156	27643	Ubl4a	8266	-0.649213867	0.46289
1157	14567	Gdi1	2664	-0.407184469	0.41716
1158	70097	Sash1	23328	-0.370140693	0.59382
1159	17873	Gadd45b	4616	1.262637957	0.27352
1160	30925	Slamf6	114836	-0.197514153	0.95058
1161	27218	Slamf1	6504	-0.038766584	0.991
1162	66220	Zdhhc12	84885	0.273999593	0.92885
1163	13804	Endog	2021	0.032381895	0.99988
1164	13058	Cybb	1536	1.461937584	0.02026
1165	57437	Golga7	51125	0.783637567	0.02965
1166	22439	Xk	7504	0.871189158	0.2848
1167	76477	Pcolce2	26577	0.530955603	0.90372
1168	12506	Cd48	962	0.678984193	0.04304
1169	270166	Clpx	10845	-0.15467634	0.69761
1170	67976	Trabd	80305	-0.739032059	0.24307
1171	69440	Dennd6b	414918	0.57124822	0.85513
1172	12522	Cd83	9308	1.678613612	0.00544
1173	14939	Gzmb			0.991
1174	12915	Atf6b	1388	0.489771628	0.32442
1175	18132	Notch4		0.017176676	0.99988
1176	54397	Ppt2		-0.09937171	0.99988
1177	260297	Prrt1	80863	0.658270535	0.31739
1178	54197	Rnf5	6048	-0.29459885	0.65533
1179	381356	Cacfd1	11094	-0.658170808	0.33674
1180	15273	Hivep2	3097	0.764953383	0.04341
1181	11863	Arnt	405	0.317171941	0.05245
1182	16398	Itga2	3673	0.406532781	0.02535
1183	17434	Mocs2	4338	0.787624636	0.00994
1184	66176	Nat9	26151	1.040322701	0.25731
1185	16956	Lpl		0.536993917	0.97042
1186	11974	Atp6v0e	8992	-0.018285252	0.99988
1187	57908	Zfp318	24149	-1.142370539	0.15601
1188	14462	Gata3	2625	-0.179589649	0.88959
1189	16776	Lama5	3911	-0.066517498	0.99988
1190	70358	Steap1		-1.675118187	0.11975
1191	74051	Steap2	261729	-1.148065979	0.17757
1192	15481	Hspa8	3312	0.276586473	0.06845
1193	321007	Serac1	84947	-0.807064699	0.34398
1194	72621	Pdzd11	51248	-0.611777916	0.45745
1195	19166	Psma2	5683	0.541100544	0.02623
1196	75398	Mrpl32	64983	-0.024689969	0.99988
1197	84505	Setdb1	9869	0.018252588	0.99397

1198	71790	Anxa9	8416		0.991
1199	229589	Prune1	58497	0.52518532	0.60754
1200	76893	Cers2	29956	0.10462663	0.15297
1201	12343	Capza2	830	0.321460881	0.31651
1202	67220	Plekho1	51177	-0.881278646	0.23011
1203	22365	Vps45	11311	-0.4685784	0.52951
1204	70767	Prpf3	9129	-0.294165038	0.62066
1205	66471	Anp32e	81611	0.210333608	0.99667
1206	226548	Aph1a	51107	0.176841473	0.17215
1207	68652	Tab2	23118	-0.196987535	0.69159
1208	67418	Ppil4	85313	-0.526342666	0.59323
1209	12793	Cnih1	10175	-0.290866469	0.60764
1210	13860	Eps8	2059	-0.017817438	0.99988
1211	20933	Med22	6837	-0.798071302	0.30926
1212	20930	Surf1	6834	0.697255241	0.45222
1213	66999	Med28	80306	0.186368907	0.08807
1214	110391	Qdpr	5860	0.294364719	0.49384
1215	18412	Sqstm1	8878	0.465047853	0.26317
1216	18024	Nfe2l2	4780	-0.299565362	0.7427
1217	20183	Rxrg	6258	-0.976461312	0.26012
1218	20181	Rxra	6256	0.193716554	0.89847
1219	229595	Adamtsl4	54507	-1.015930608	0.03732
1220	80891	Fcrls	83417	2.323834314	0.00438
1221	11801	Cd5l	922	1.592723884	0.09488
1222	67763	Prpsap1	5635	-0.252127317	0.97752
1223	54392	Ncapg	64151	-0.459294086	0.45862
1224	209707	Lcorl	254251	2.702502218	0.01942
1225	16993	Lta4h	4048	-0.707997428	0.36308
1226	56431	Dstn	11034	-0.311626893	0.7695
1227	26914	Macroh2a1	9555	-0.593657	0.31112
1228	114674	Gtf2ird2		-0.242896794	0.99988
1229	69168	Bola1	51027	0.322291778	0.72035
1230	80909	Castor2	729438	0.416074205	0.82477
1231	14129	Fcgr1		1.9919279	0.00621
1232	17969	Ncf1	653361	1.009683753	0.01657
1233	11566	Adss	159	-0.057383438	0.80163
1234	12289	Cacna1d	776	0.469252258	0.70348
1235	218865	Chdh	55349	-0.100020726	0.87239
1236	56249	Actr8	93973	0.016902979	0.99988
1237	76612	Lrrc27	80313	-0.705758181	0.23751
1238	14272	Fnta	2339	0.62961826	0.01881
1239	72198	Mtrex	23517	0.030532179	0.94092
1240	16803	Lbp	3929	0.591987946	0.02656
1241	12614	Celsr1	9620	-0.76478398	0.29633
1242	14247	Fli1	2313	0.582641382	0.02443
1243	243362	Stard13	90627	0.551612997	0.02595
1244	215193	Utp25	27042	1.117234874	0.01788
1245	15483	Hsd11b1	3290	1.266344643	0.1372
1246	14991	H2-M3		-0.149600212	0.99691
1247	74365	Lonrf3	79836	-0.052261656	0.88529

1248	67126	Atp5e	514	0.415913954	0.29098
1249	57314	Nelfcd	51497	-0.441053049	0.55816
1250	64138	Ctsz	1522	0.484520719	0.45952
1251	66390	Prelid3b	51012	-0.755493354	0.41015
1252	214791	Sertad4	56256	0.085074967	0.69294
1253	22209	Ube2a	7319	-0.606102434	0.67244
1254	11740	Slc25a5	292	0.182954235	0.73275
1255	66496	Ppdpf	79144	0.36392065	0.38014
1256	102866	Pls3	5358	0.329358484	0.05734
1257	77015	Mpped2	744	-1.228763843	0.18518
1258	67050	Nkap	79576	-0.307400866	0.47867
1259	54405	Ndufa1	4694	0.950476987	0.0109
1260	22431	Wt1	7490	2.364457212	0.00407
1261	13557	E2f3	1871	0.213246856	0.67337
1262	12946	Cr1l	1378	0.262783659	0.11368
1263	67533	Ppfibp1	8496	-0.097972447	0.99988
1264	17221	Cd46	4179	0.434722191	0.98076
1265	12490	Cd34	947	0.496712101	0.63843
1266	67759	Plgrkt	55848	0.517263314	0.02867
1267	60533	Cd274	29126	-0.48762715	0.81166
1268	66596	Gtf3a	2971	-0.599035734	0.42153
1269	76366	Mtif3	219402	-0.164858407	0.99988
1270	140887	Lnx2	222484	1.202275042	0.01115
1271	226419	Dyrk3	8444	-0.476743837	0.63993
1272	17164	Mapkapk2	9261	0.200465008	0.0791
1273	16784	Lamp2		0.244483629	0.0358
1274	54138	Atxn10	25814	-0.120744445	0.6594
1275	239554	Foxred2	80020	0.357178976	0.29639
1276	55944	Eif3d	8664	-0.07458841	0.9519
1277	15081	H3f3b	440093	-0.314860431	0.44257
1278	18141	Nup50		-0.36563719	0.48303
1279	67042	Ift27	11020	-0.022320173	0.99816
1280	23970	Pacsin2	11252	0.757464595	0.01194
1281	12763	Cmah		0.871516511	0.01143
1282	223723	Ttll12	23170	-1.02711569	0.27649
1283	12124	Bik	638	-0.870610434	0.55704
1284	268741	Tox4	9878	0.330418272	0.38074
1285	68735	Mrps18c	51023	0.375180355	0.36523
1286	240725	Sulf1	23213	0.346200551	0.74228
1287	67996	Srsf6	6431	0.276797083	0.55816
1288	18803	Plcg1	5335	-0.386005109	0.62094
1289	70382	Kctd2	23510	0.294069503	0.12819
1290	71753	Tmprss6	164656	0.221503054	0.83184
1291	69259	Kctd5	54442	-0.930401143	0.24003
1292	68145	Etaa1	54465	0.141904637	0.60193
1293	20568	Slpi	6590	2.781070334	0.00566
1294	20971	Sdc4	6385	-0.964531259	0.26511
1295	16164	Il13ra1	3597	-0.079142772	0.99988
1296	17966	Nbr1	4077	0.332695631	0.35792
1297	19157	Cyth1	9267	-1.886581564	0.08253

1298	74194	Rnd3	390	1.346706041	0.14942
1299	68106	Nt5c3b	115024	0.362234966	0.45469
1300	52469	Coa3	28958	-0.090759636	0.99988
1301	57911	Gsdma		-1.977820599	0.09286
1302	23989	Med24	9862	-1.569473058	0.09941
1303	22123	Psmd3	5709	-0.155910377	0.74228
1304	50912	Exosc10	5394	0.267026726	0.90531
1305	67201	Glod4	51031	-0.339339767	0.61403
1306	68299	Vps53	55275	0.039842393	0.99988
1307	216965	Taok1	57551	-0.633620253	0.25118
1308	76233	Dnttip1	116092	0.086857768	0.99988
1309	21925	Tnnc2	7125	2.741013886	0.04413
1310	170789	Acot8	10005	1.061153761	0.6526
1311	52685	Cd300lg	146894		0.991
1312	18099	Nlk	51701	0.188486551	0.90414
1313	22032	Traf4	9618	-0.80489176	0.30315
1314	11676	Aldoc	230	0.116785424	0.9548
1315	217154	Stac2	342667	-0.503875018	0.15099
1316	19921	Rpl19	6143	-0.023800804	0.99988
1317	140859	Nek8	284086	-1.45200231	0.11694
1318	72324	Plxdc1	57125	0.833189315	0.01707
1319	75869	Arl5b	221079	0.499064113	0.74317
1320	22680	Zfp207	7756	0.17135881	0.95504
1321	69077	Psmd11	5717	-0.216457796	0.80932
1322	56745	C1qtnf1	114897	0.897793408	0.25496
1323	21858	Timp2	7077	0.000443922	0.99989
1324	76014	Zc3h18	124245	-1.456022497	0.16748
1325	21974	Top2b	7155	0.477480102	0.03775
1326	16010	Igfbp4	3487	1.688368713	0.10938
1327	23834	Cdc6	990	0.428545887	0.93625
1328	52615	Suz12	23512	-1.097403711	0.22235
1329	237877	Atad5	79915	-0.440724415	0.25253
1330	54394	Crlf3	51379	0.596073536	0.00724
1331	217169	Tns4	84951	1.746969157	0.00646
1332	21927	Tnfaip1	7126	-0.971816879	0.1497
1333	109934	Abr	29	0.265932194	0.97147
1334	268451	Rab11fip4	84440	-1.162625329	0.1339
1335	21939	Cd40	958	2.513212264	0.00388
1336	228875	Slc35c2	51006	-1.53355846	0.19632
1337	228876	Zfp334	55713	1.555121844	0.00773
1338	140579	Elmo2	63916	0.616495183	0.18512
1339	78889	Wsb1	26118	-0.099681637	0.99988
1340	76080	Ttpal	79183	-0.465234323	0.68773
1341	59040	Rhot1	55288	0.175982562	0.99988
1342	11486	Ada	100	0.411947647	0.76092
1343	26943	Serinc3	10955	0.390323424	0.03311
1344	74451	Pgs1	9489	-0.714670049	0.48351
1345	11799	Birc5	332	-0.073921293	0.99988
1346	71562	Afmid	125061	-1.104195733	0.33125
1347	78928	Pigt	51604	0.924039621	0.01143

1348	67701	Wfdc2	10406	-0.113455846	0.98957
1349	52840	Dbndd2	55861	0.727760821	0.01618
1350	17395	Mmp9	4318	2.82119259	0.00356
1351	80860	Ghdc	84514	-0.378721025	0.78002
1352	18830	Pltp	5360	1.458267117	0.06782
1353	20499	Slc12a7	10723	0.121347273	0.8865
1354	19025	Ctsa	5476	-0.634321876	0.62152
1355	71971	Zswim1	90204	-1.124039406	0.25336
1356	20498	Slc12a4	6560	0.321973701	0.95199
1357	17913	Myo1c	4641	-0.007581753	0.99988
1358	12928	Crk	1398	-0.728707333	0.33427
1359	12867	Cox7c	1350	0.434004605	0.02615
1360	18738	Pitpna	5306	-0.061370223	0.95917
1361	21428	Mlx	6945	1.521368477	0.02372
1362	67998	Retreg3	162427	-0.408559783	0.5314
1363	59091	Jph2	57158	1.046386311	0.00975
1364	80861	Dhx58	79132	-1.313436092	0.28449
1365	271457	Rab5a	5868	0.202161709	0.991
1366	71966	Nkiras2	28511	0.225038175	0.87985
1367	26931	Ppp2r5c	5527	0.121134349	0.24705
1368	245866	Ift52	51098	-0.764330836	0.33886
1369	17865	Mybl2	4605	-1.594737057	0.15308
1370	14049	Eya2	2139	0.669596474	0.23497
1371	56336	B4galt5	9334	1.747621583	0.00533
1372	19223	Ptgis	5740	0.578091786	0.6358
1373	320405	Cadps2	93664	0.050324395	0.99847
1374	228889	Ddx27	55661	-0.787540606	0.23085
1375	19159	Cyth3	9265	-0.270991907	0.96811
1376	72318	Cyth4	27128	2.625082536	0.00388
1377	170758	Rac3	5881	-0.171945791	0.94358
1378	74778	Rrp7a	27341	-0.489988506	0.41755
1379	109754	Cyb5r3	1727	0.225819581	0.41147
1380	70422	Ints2	57508	-0.165929659	0.99988
1381	76199	Med13l	23389	-0.670733449	0.3525
1382	68024	H2bc4		-0.136646638	0.99988
1383	17135	Mafk	7975	-0.730550475	0.43816
1384	19014	Med1	5469	-0.396440164	0.65589
1385	13867	Erbb3		-0.221984519	0.62816
1386	59045	Stard3	10948	0.374749391	0.65112
1387	22780	Ikzf3	22806	0.12514237	0.93548
1388	17305	Mfng	4242	1.624184862	0.00733
1389	75909	Vmp1	81671	-0.865914464	0.26686
1390	56207	Uchl5	51377	0.597137594	0.41015
1391	69367	Glrx2		-0.680013422	0.33374
1392	20822	Ro60	6738	-0.116230497	0.89785
1393	58231	Stk4	6789	0.017975769	0.99988
1394	192201	Wfdc15b		1.22160652	0.0214
1395	18858	Pmp22	5376	1.370649281	0.10704
1396	14566	Gdf9	2661	1.038777553	0.13649
1397	67966	Zcchc10	54819	0.19888271	0.8717

1398	19175	Psmb6	5694	-0.181657733	0.9548
1399	216873	Spag7	9552	-0.649157714	0.20508
1400	67145	Tomm34	10953	-0.159772405	0.92668
1401	54401	Ywhab	7529	0.270148303	0.35576
1402	16706	Ksr1	8844	0.382733267	0.66285
1403	14778	Gpx3	2878	0.47390971	0.89638
1404	11749	Anxa6	309	-0.757699222	0.19297
1405	52712	Zkscan6	7566	-0.50036442	0.41147
1406	16647	Kpna2		0.275830352	0.85932
1407	66313	Smurf2	64750	-0.418745201	0.46145
1408	320162	Cep95	90799	-1.051009782	0.19212
1409	22344	Vezf1	7716	-0.458806597	0.55831
1410	70393	Cuedc1	404093	-0.359694313	0.54865
1411	110809	Srsf1	6426	0.027351596	0.99988
1412	66610	Abi3	51225	0.23891941	0.66558
1413	71774	Shroom1	134549	-1.163155596	0.23975
1414	16568	Kif3a	11127	-1.387846698	0.1497
1415	20362	Septin8	23176	0.114070896	0.99988
1416	170749	Mtmr4	9110	-0.104218133	0.92694
1417	217038	Mrm1	79922	-0.789195278	0.29072
1418	76719	Kansl1	284058	-0.428816804	0.67413
1419	66771	Gid4	79018	-0.440475065	0.92691
1420	17912	Myo1b	4430	-0.102555018	0.93165
1421	67487	Dhx40	79665	0.429276654	0.03528
1422	77864	Ypel2	388403	0.446222971	0.07241
1423	11640	Akap1	8165	0.055034322	0.99988
1424	68979	Nol11	25926	-0.274834916	0.97042
1425	116891	Derl2	51009	-0.508106403	0.60274
1426	12261	C1qbp	708	0.049564198	0.99988
1427	69723	Rpain	84268	1.214719	0.38014
1428	114644	Slc13a3	64849	1.485067111	0.0041
1429	16499	Kcnab3	9196	-0.947555867	0.38217
1430	216848	Chd3	1107	-1.243361534	0.16168
1431	216850	Kdm6b	23135	-1.653741278	0.05216
1432	66884	Appbp2	10513	0.223551476	0.97377
1433	11541	Adora2b	136	1.993284524	0.00741
1434	20185	Ncor1	9611	-0.789059892	0.21751
1435	22368	Trpv2	51393	2.178417539	0.00678
1436	73139	Cenpv	201161	0.206735988	0.99988
1437	22658	Pcgf2	7703	-0.439394829	0.60745
1438	67480	Cwc25	54883	-0.382639752	0.78646
1439	108083	Pip4k2b	8396	0.447511651	0.2416
1440	68729	Trim37	4591	0.592441309	0.02459
1441	67181	Ctdnep1	23399	-0.468964497	0.59089
1442	54351	Elp5	23587	1.319386477	0.06983
1443	20528	Slc2a4	6517	0.314647153	0.15905
1444	56486	Gabarap	11337	-0.039937419	0.9838
1445	53624	Cldn7		-1.698392416	0.13824
1446	70419	810408A11Rik		-0.742525852	0.01881
1447	78246	Phf23	79142	-0.175707319	0.80953

1448	11370	Acadvl	37	0.82463511	0.01649
1449	27041	G3bp1	10146	-0.116244368	0.99988
1450	11927	Atox1	475	0.66024718	0.01732
1451	20692	Sparc	6678	-0.432447291	0.69573
1452	237781	Mief2	125170	-0.45114133	0.92014
1453	21386	Tbx3	6926	-0.517258468	0.61857
1454	17393	Mmp7	4316	1.08131285	0.14257
1455	56405	Dusp14	11072	-0.123323124	0.89968
1456	217031	Tada2a	6871	-0.581650473	0.52485
1457	22778	Ikzf1	10320	1.052130967	0.01701
1458	103711	Pnpo	55163	-0.458979642	0.41663
1459	16834	Cog1	9382	0.532523447	0.32814
1460	12412	Cbx1	10951	0.31121959	0.75527
1461	80280	Cdk5rap3	80279	-0.29574993	0.54076
1462	56358	Copz2	51226	-0.347821145	0.77139
1463	68066	Slc25a39	51629	-1.073602117	0.27081
1464	78912	Sp2	6668	-1.148849739	0.3266
1465	56321	Aatf	26574	-1.134572439	0.19857
1466	13424	Dync1h1	1778	-0.329718858	0.74381
1467	103737	Pex12	5193	0.122659191	0.89849
1468	83431	Ndel1	81565	-0.254044167	0.84786
1469	71998	Slc25a35	399512	-0.636214254	0.32523
1470	75580	Zbtb4	57659	-0.43076768	0.43471
1471	24070	Mpdu1	9526	0.11562425	0.99988
1472	23879	Fxr2	9513	0.514882718	0.37705
1473	228033	Atp5g3	518	0.681515629	0.01855
1474	12514	Cd68		1.104909651	0.00984
1475	14081	Acsl1	2180	0.002048881	0.99989
1476	217265	Abca5	23461	0.057911256	0.92702
1477	319822	Smyd4	114826	-0.612311578	0.57314
1478	16985	Lsp1	4046	0.656080009	0.48143
1479	319740	Zfyve27	118813	-0.661264303	0.18424
1480	69534	Avpi1	60370	-0.136505916	0.96415
1481	17880	Myh11	4629	1.740548879	0.01151
1482	19364	Rad51d	5892	0.217317492	0.48487
1483	217012	Unc45b	146862	0.932249217	0.0192
1484	211347	Pank3	79646	0.116754615	0.99988
1485	104458	Rars	5917	-0.132967558	0.83403
1486	211652	Wwc1	23286	-1.127031762	0.18424
1487	68572	Mrpl58	3396	0.126497577	0.99988
1488	14149	Fdxr		-1.181223088	0.23004
1489	67036	Mrpl45	84311	-0.514648948	0.40369
1490	16362	Irf1	3659	0.526458503	0.25338
1491	20520	Slc22a5		0.357248585	0.76849
1492	18452	P4ha2	8974	0.191069038	0.35568
1493	109689	Arrb1	408	0.648038455	0.19646
1494	66102	Cxcl16	58191	-0.030125341	0.99988
1495	75273	Pelp1	27043	-0.635333192	0.56261
1496	66172	Med11	400569	-0.636022255	0.76578
1497	20305	Ccl6		2.014945264	0.00406

1498	20303	Ccl4		0.176604532	0.82658
1499	24083	Natd1	256302	-0.199399521	0.83184
1500	26397	Map2k3	5606	0.039997571	0.99988
1501	22629	Ywhah	7533	0.157266354	0.99988
1502	53890	Sart3	9733	-0.681392891	0.29167
1503	242705	E2f2	1870	0.068699515	0.99988
1504	244141	Nars2	79731	0.342730646	0.60532
1505	58246	Slc35b4	84912	0.826233405	0.19329
1506	67789	Dalrd3	55152	-0.862230813	0.25715
1507	66437	Fis1	51024	-0.17397755	0.98675
1508	18822	Plod1	5351	0.610542997	0.02378
1509	19340	Rab3d	9545	-0.446636807	0.4251
1510	69572	Mfsd3	113655	-0.497893236	0.53504
1511	68267	Slc25a22	79751	0.021050481	0.99988
1512	54411	Atp6ap1	537	0.355828049	0.73818
1513	69537	Dnase1l1	1774	0.664000868	0.37342
1514	11670	Aldh3a1	218	0.614020297	0.48422
1515	20308	Ccl9		2.886950869	0.00356
1516	69938	Scrn1	9805	0.485972644	0.67453
1517	79555	BC005537	81688	-0.236375065	0.76163
1518	71780	Isyna1	51477	1.008006401	0.09426
1519	70791	Hars2	23438	0.630501167	0.27705
1520	69094	Tmem160	54958	0.322447667	0.60705
1521	19345	Rab5c	5878	0.458610036	0.59585
1522	17448	Mdh2	4191	-0.250497712	0.68757
1523	14950	H13	81502	0.179026489	0.99988
1524	74315	Rnf145	153830	-0.141333543	0.99988
1525	11973	Atp6v1e1	529	0.189734026	0.05715
1526	238323	Rps6kl1	83694	0.086786257	0.99988
1527	232807	Ppp1r12c	54776	0.353913097	0.4295
1528	11622	Ahr		0.324263697	0.71984
1529	270058	Map1s	55201	0.334374629	0.99988
1530	13479	Dpep1	1800	-0.219720291	0.99988
1531	68366	Tmem129	92305	0.316152792	0.61446
1532	67842	Nop9	161424	-0.021544692	0.99988
1533	11975	Atp6v0a1	535	-0.447785727	0.84786
1534	19183	Psmc3ip	29893	-0.009994094	0.99988
1535	14786	Grb7	2886	0.869947893	0.26685
1536	71893	Noxo1	124056	-0.980177675	0.15428
1537	11754	Aoc3	8639	0.874955193	0.45717
1538	78266	Zfp687	57592	-0.246555084	0.99572
1539	71584	Gdpd2	54857	0.109261029	0.95936
1540	101966	D8Ertd738e	28974	0.100638096	0.99988
1541	103655	Sec14l4	284904	-1.527993718	0.10226
1542	12315	Calm3	808	0.030831391	0.93084
1543	26572	Cops3	8533	0.409614772	0.39507
1544	14232	Fkbp8	23770	-0.309540516	0.56261
1545	53817	Ddx39b	7919	0.078161859	0.92973
1546	67903	Gipc1	10755	-0.110600862	0.96307
1547	68385	Tlcd1	116238	0.094771303	0.89014

1548	70310	Plscr3	57048	-0.626740262	0.29324
1549	52666	Arhgef25		-0.139926457	0.99988
1550	67439	Xab2	56949	0.266297439	0.74969
1551	12539	Cdc37	11140	0.012411509	0.99988
1552	19341	Rab4a	5867	-0.180316173	0.84891
1553	106628	Trip10	9322	0.533096749	0.63959
1554	26893	Cops6	10980	-0.507702139	0.37201
1555	22187	Ubb	7314	0.187066351	0.42857
1556	11781	Ap4m1	9179	0.101339753	0.57989
1557	27357	Gyg	2992	0.943733331	0.57293
1558	52377	Rcn3		0.754353905	0.22008
1559	102857	Slc6a8		-0.528546771	0.61603
1560	13496	Arid3a	1820	-0.067608787	0.99988
1561	66530	Ubxn6	80700	-0.353659853	0.71054
1562	28106	Mydgf	56005	0.281559061	0.04197
1563	13056	Cyb561	1534	-0.020060851	0.99988
1564	20358	Sema6a	57556	0.207655981	0.91879
1565	72654	Ccdc12	151903	0.976934223	0.20337
1566	66117	Fmc1	154791	1.060467223	0.00544
1567	23797	Akt3	10000	1.654654314	0.01881
1568	67707	Mrpl24	79590	-0.131672672	0.89847
1569	74412	Gle1	2733	1.041263861	0.00932
1570	67217	L3hypdh	112849	-0.207581585	0.99988
1571	17101	Lyst	1130	1.445382841	0.00843
1572	270066	Slc35e1	79939	-0.185922928	0.77567
1573	353499	Tmc4	147798	-0.427082171	0.69277
1574	233066	Syne4	163183	-0.714555246	0.40178
1575	69920	Polr2i	5438	-0.310092026	0.59323
1576	16661	Krt10	3858	0.665486673	0.48431
1577	66084	Rmnd1	55005	-0.448276869	0.43998
1578	13982	Esr1	2099	1.280629561	0.00773
1579	67141	Fbxo5	26271	-1.25590778	0.15062
1580	108853	Mtrf1l	54516	-0.494384781	0.78646
1581	15182	Hdac2	3066	-0.095088224	0.96958
1582	14302	Frk	2444	0.357230139	0.05312
1583	66521	Rwdd1	51389	-0.164857894	0.93165
1584	78808	Stxbp5	134957	0.843225692	0.45319
1585	66847	Hint3	135114	0.491095913	0.31706
1586	73681	Trmt11	60487	0.701059463	0.30954
1587	23924	Katna1	11104	0.240121471	0.7025
1588	18537	Pcmt1	5110	0.425690603	0.08624
1589	237253	Lrp11	84918	0.892016716	0.04812
1590	67851	Mtres1	51250	0.172370361	0.24565
1591	140740	Sec63	11231	-0.215502856	0.57946
1592	54198	Snx3	8724	0.135661294	0.50153
1593	66253	Aig1	51390	0.003925146	0.99988
1594	66757	Adat2	134637	-0.58018273	0.77644
1595	56535	Pex3	8504	-0.636460692	0.37157
1596	66848	Fuca2	2519	0.294772874	0.75725
1597	103268	Cep57l1	285753	1.038214257	0.02031

1598	353258	Ltv1		-0.267100343	0.60128
1599	22634	Plagl1	5325	0.570734011	0.16854
1600	53599	Cd164	8763	-0.400799065	0.32951
1601	22288	Utrn	7402	0.064457371	0.99988
1602	20598	Smpd2	6610	0.08873756	0.98889
1603	171580	Mical1	64780	0.887581131	0.18016
1604	268294	Zbtb24	9841	0.389004579	0.39449
1605	67844	Rab32		1.014451681	0.0123
1606	67371	Gtf3c6	112495	-0.440424858	0.60705
1607	72472	Slc16a10	117247	-0.043437216	0.99646
1608	19714	Rev3l	5980	-1.232027358	0.1705
1609	103213	Traf3ip2	10758	-1.039703951	0.18112
1610	14360	Fyn	2534	-0.269041125	0.99988
1611	16775	Lama4	3910	0.010897282	0.99988
1612	19072	Prep	5550	0.575758378	0.26245
1613	21929	Tnfaip3	7128	0.556401259	0.49402
1614	64058	Perp	64065	-0.337939746	0.59302
1615	215821	Arfgef3	57221	-0.881814177	0.47207
1616	56016	Hebp2	23593	-0.781983276	0.29572
1617	19707	Reps1	85021	-0.154027199	0.99988
1618	66403	Asf1a	25842	0.201471638	0.09286
1619	94221	Gopc		0.094724494	0.87898
1620	76563	Qrsl1	55278	-0.065387137	0.99988
1621	170728	Rtn4ip1	84816	-0.163796993	0.86115
1622	11630	Crybg1	202	-0.546829802	0.39734
1623	66201	Vta1	51534	0.269937685	0.97856
1624	57319	Smpdl3a	10924	0.716417847	0.0409
1625	28193	Reep3	221035	-0.63864609	0.41147
1626	18768	Pkib	5570	-0.416249371	0.44852
1627	56442	Serinc1	57515	0.024544062	0.99988
1628	15500	Hsf2	3298	0.622351844	0.35902
1629	72780	Rspo3	84870	0.261704116	0.74269
1630	19272	Ptprk	5796	-0.327860943	0.52124
1631	66686	Dcbld1	285761	1.07617661	0.00934
1632	52713	Ccdc59	29080	-0.30273678	0.75823
1633	16773	Lama2	3908	0.796229892	0.2496
1634	17931	Ppp1r12a	4659	-0.172654571	0.99988
1635	18451	P4ha1	5033	0.451962234	0.26902
1636	103080	Septin10	151011	-1.021476971	0.22012
1637	110829	Lims1		0.369236598	0.42547
1638	52696	Zwint	11130	0.472758078	0.29273
1639	216080	Ube2d1	7321	-0.457218295	0.86369
1640	13179	Dcn	1634	0.864942813	0.45316
1641	69563	Mrln	100507027	0.401534824	0.38109
1642	13516	Epyc	1833	1.122921964	0.01074
1643	12534	Cdk1		-0.83777864	0.30831
1644	67972	Atp2b1	490	-0.134987184	0.71055
1645	69288	Rhobtb1		0.767414236	0.21178
1646	71371	Arid5b	84159	-0.130792124	0.99988
1647	67019	Actr6		-0.608869322	0.53653

1648	75089	Uhrf1bp1l		0.230231038	0.7025
1649	382406	Poc1b	282809	-0.599034974	0.35248
1650	67603	Dusp6	1848	0.843834127	0.08248
1651	21917	Tmpo		0.088391272	0.90569
1652	17311	Kitl	4254	0.343943748	0.32689
1653	19164	Psen1	5663	-0.208880385	0.991
1654	20393	Sgk1	6446	-0.529702107	0.56568
1655	216274	Cep290	80184	-0.054980657	0.95106
1656	67454	Ikbip		0.288214729	0.93597
1657	56422	Hbs1l	10767	-0.43241118	0.46005
1658	13822	Epb41l2	2037	0.998944488	0.17863
1659	11783	Apaf1		1.448046808	0.00797
1660	17863	Myb		0.043947917	0.97856
1661	70208	Med23	9439	0.996019831	0.00837
1662	52906	Ahi1		-0.465881357	0.78259
1663	11846	Arg1	383	-2.401804722	0.04262
1664	17997	Nedd1		0.5703119	0.78872
1665	209558	Enpp3	5169	-1.441448662	0.17216
1666	17761	Map7		-0.828356888	0.4432
1667	14219	Ccn2	1490	1.184139744	0.15974
1668	53331	Stx7	8417	0.613606555	0.02623
1669	59012	Moxd1	26002	0.848591867	0.05634
1670	18634	Pex7	5191	0.389790641	0.3837
1671	15979	Ifngr1	3459	0.54241686	0.59996
1672	26464	Vnn3	55350	-0.162870535	0.99988
1673	237459	Cdk17	5128	1.075191237	0.19688
1674	69878	Snrpf		0.020126403	0.99988
1675	57764	Ntn4	59277	-1.063088351	0.26302
1676	13998	Fgd6	55785	0.139145964	0.99988
1677	66414	Ndufa12	55967	0.151854331	0.80565
1678	319880	Tmcc3	57458	-1.15764539	0.20093
1679	77048	Cep83	51134	-0.36359848	0.78063
1680	216233	Socs2	8835	-0.085252465	0.99779
1681	71207	Nudt4		0.212963622	0.6103
1682	77976	Nuak1	9891	-1.013489548	0.48351
1683	216198	Tcp11l2	255394	0.754462183	0.26256
1684	12952	Cry1	1407	0.348911574	0.85106
1685	74007	Btbd11	121551	-0.098899639	0.90175
1686	21859	Timp3	7078	1.24496997	0.15771
1687	22027	Hsp90b1	7184	-0.379831074	0.64021
1688	16000	Igf1	3479	0.545494193	0.93113
1689	67282	Washc3	51019	0.903858412	0.01273
1690	71712	Dram1	55332	1.729444829	0.00933
1691	216225	Slc5a8	160728	-3.706753071	0.02391
1692	93759	Sirt1	23411	0.629258121	0.87476
1693	67345	Herc4	26091	0.273502439	0.57314
1694	432467	Hnrnph3	3189	1.168402905	0.01089
1695	70432	Rufy2	55680	0.250967122	0.99988
1696	67500	Ccar1	55749	0.353283466	0.22553
1697	56200	Ddx21	9188	-0.592242104	0.36223

1698	94213	Ddx50	79009	0.21004888	0.90259
1699	19073	Srgn	5552	1.333165068	0.00837
1700	30930	Vps26a	9559	0.071313185	0.60118
1701	338359	Supv3l1	6832	-0.604485036	0.38042
1702	69894	Fam241b		-0.053449843	0.99988
1703	71361	Aifm2	84883	-0.303514113	0.99988
1704	404634	Macroh2a2	55506	-0.364240055	0.4291
1705	71767	Tysnd1	219743	0.208424609	0.99988
1706	20224	Sar1a	56681	-0.278330732	0.55816
1707	67895	Ppa1	5464	0.134720843	0.53565
1708	13688	Eif4ebp2	1979	-0.527417256	0.36857
1709	27355	Pald1	27143	1.002512019	0.02435
1710	20397	Sgpl1	8879	0.414773692	0.03353
1711	13180	Pcbd1	5092	0.026875329	0.99988
1712	107449	Unc5b	219699	0.1724227	0.99988
1713	71279	Slc29a3	55315	-0.711020487	0.42676
1714	74048	Vsir	64115	1.877713397	0.00544
1715	20503	Slc16a7	9194	1.65062901	0.00664
1716	320398	Lrig3	121227	-0.247271435	0.77629
1717	52717	Anapc16	119504	-0.160519109	0.65159
1718	74747	Ddit4	54541	0.268878197	0.31523
1719	56709	Dnajb12	54788	-1.410174037	0.13004
1720	216001	Micu1	10367	-0.43669387	0.51447
1721	71902	Cand1	55832	0.024960269	0.99988
1722	56480	Tbk1	29110	0.268910565	0.1293
1723	66249	Pno1	56902	1.135246452	0.09815
1724	56193	Plek	5341	1.591556278	0.00812
1725	117600	Srgap1	57522	-2.13944947	0.11938
1726	13649	Egfr	1956	-0.497341379	0.86113
1727	54140	Avpr1a	552	1.225835701	0.2049
1728	14479	Usp15	9958	0.177737411	0.85944
1729	245944	Vps54	51542	-0.421913709	0.48543
1730	66687	Tbc1d15	64786	0.186664023	0.84112
1731	216344	Rab21	23011	0.170994318	0.82274
1732	66374	310011J03Ri	148223	-0.355577698	0.67928
1733	67245	Peli1	57162	-0.206776081	0.70446
1734	66816	Thap2		-0.468780292	0.48887
1735	14160	Lgr5	8549	-0.571968396	0.8458
1736	55963	Slc1a4	6509	-1.177610076	0.22364
1737	94176	Dock2	1794	2.662975686	0.00388
1738	19324	Rab1a	5861	0.493148731	0.02287
1739	14431	Gamt	2593		0.40878
1740	19279	Ptprr	5801		0.991
1741	66713	Actr2	10097	-0.128992007	0.99988
1742	75406	Ndufs7	374291	-0.220617724	0.99988
1743	19263	Ptprb	5787	-0.121409779	0.99988
1744	68114	Pwwp3a	84939	-0.431672675	0.52331
1745	216643	Gabrp	2568	2.111903065	0.06335
1746	17268	Meis1	4211	0.148421661	0.99988
1747	66594	Uqcr11		-0.193269177	0.57917

1748	72068	Cnot2	4848	-0.076683887	0.97348
1749	21423	Tcf3	6929	-0.048848042	0.93113
1750	327826	Frs2	10818	0.233942053	0.99988
1751	64050	Yeats4	8089	0.370365997	0.43386
1752	12808	Cobl	23242	-0.605657806	0.49412
1753	14783	Grb10	2887	0.705637506	0.4679
1754	67332	Snrpd3		0.285787741	0.05773
1755	260315	Nav3	89795	-0.8624572	0.47061
1756	70574	Cpm	1368	-1.202807457	0.224
1757	17246	Mdm2	4193	-0.091909409	0.99988
1758	52679	E2f7	144455	-0.161726899	0.78118
1759	13008	Csrp2	1466	1.493544039	0.01873
1760	237542	Osbpl8	114882	0.22687304	0.19646
1761	17347	Mknk2	2872	0.485123899	0.27456
1762	104248	Cabin1	23523	-0.411843364	0.46079
1763	11776	Ap3d1	8943	-0.200341749	0.99988
1764	21664	Phlda1	22822	-0.872723074	0.37464
1765	17245	Mdm1	56890	-1.065282752	0.04137
1766	24117	Wif1	11197	3.878632601	0.00356
1767	30055	Timm13	26517	-1.109013671	0.19207
1768	230895	Vps13d	55187	0.425303122	0.35792
1769	66225	Llph	84298	-0.280693004	0.50511
1770	68212	Tmbim4		0.577112615	0.0294
1771	73914	Irak3	11213	1.089033269	0.04821
1772	117599	Helb	92797	0.43163273	0.44448
1773	15468	Prmt2	3275	0.808256836	0.01717
1774	64451	Dip2a	23181	-0.540757595	0.62454
1775	15353	Hmg20b	10362	-1.05336223	0.24843
1776	56371	Fzr1	51343	0.097332138	0.99988
1777	103425	Ncln	56926	-0.11915938	0.99988
1778	12834	Col6a2	1292	1.249782136	0.204
1779	67933	Hcfc2	29915	-0.137062273	0.79357
1780	18045	Nfyb	4801	-0.693452229	0.33123
1781	50493	Txnrd1	7296	0.423416347	0.0293
1782	67905	Ppm1m	132160	0.376789357	0.65044
1783	28109	D10Wsu102e	121053	-0.67729417	0.39858
1784	77305	Wdr82	80335	0.146083379	0.69089
1785	80294	Pofut2	23275	0.64123836	0.41537
1786	215335	Slc36a1	206358	-0.317574264	0.72829
1787	110532	Adarb1	104	0.265995421	0.99988
1788	216190	Appl2	55198	-0.456204647	0.53734
1789	246049	Slc36a2	153201	0.405146683	0.56882
1790	20610	Sumo3	6612	0.257533627	0.45245
1791	15254	Hint1	3094	0.512129864	0.06172
1792	75530	Lyrm7	90624	0.754149105	0.01116
1793	103583	Fbxw11	23291	-0.482058066	0.43504
1794	216578	Papolg	64895	-0.28355787	0.72098
1795	19696	Rel	5966	-0.105210953	0.99988
1796	18641	Pfkl	5211	-1.27784383	0.21178
1797	16199	Il9r	3581	1.504962668	0.07959

1798	74467	Pus10	150962	-0.503929621	0.41413
1799	13650	Rhbdf1	64285	0.235437886	0.99053
1800	72129	Pex13	5194	-0.042392433	0.94575
1801	67884	Cfap410	755	-0.239668831	0.75567
1802	268395	Mpg	4350	-0.157505429	0.8193
1803	268390	Ahsa2		0.512776098	0.02944
1804	17168	Nprl3	8131	0.113105143	0.99988
1805	103573	Xpo1	7514	0.256509281	0.4499
1806	67579	Cpeb4	80315	-0.201121425	0.64148
1807	20856	Stc2	8614	-0.381675883	0.48476
1808	65257	Asb3	51130	1.84208819	0.09987
1809	216150	Cdc34		-0.716021338	0.23077
1810	110012	Tpgs1	91978	0.001002839	0.99989
1811	68044	Chac2	494143	0.779923083	0.26896
1812	66753	Erlec1	27248	-0.100106261	0.89847
1813	216148	Shc2		-0.249508525	0.98977
1814	20742	Sptbn1	6711	0.077262981	0.99988
1815	216560	Wdpcp	51057	-0.650910076	0.38978
1816	17449	Mdh1	4190	0.683602572	0.01394
1817	73106	Prss57	400668	1.25036672	0.01314
1818	83554	Fstl3	10272	1.283567869	0.00613
1819	12450	Ccng1	900	0.091421749	0.99988
1820	52653	Nudcd2	134492	-0.570453506	0.38657
1821	216151	Polrmt	5442	-0.888079446	0.30672
1822	15366	Hmmr	3161	-0.064885184	0.95929
1823	76884	Cyfip2	26999	0.60194767	0.06845
1824	17308	Mgat1	4245	0.077549009	0.91657
1825	19052	Ppp2ca	5515	-0.542235632	0.45862
1826	14257	Flt4	2324	1.071285443	0.00837
1827	15384	Hnrnpab	3182	-0.553781143	0.36418
1828	72947	Phykpl	85007	0.429913617	0.4372
1829	15525	Hspa4	3308	0.64447433	0.07528
1830	104625	Cnot6	57472	-0.2015571	0.68869
1831	14584	Gfpt2	9945	1.038214257	0.17406
1832	21408	Zfp354a		-0.858060198	0.17719
1833	26420	Mapk9	5601	0.334600548	0.6635
1834	12330	Canx	821	-0.39283284	0.3364
1835	14694	Rack1	10399	0.22397321	0.71486
1836	216724	Rufy1	80230	-0.518886534	0.61627
1837	59044	Rnf130	55819	0.171412538	0.69294
1838	17001	Ltc4s		3.180250878	0.00356
1839	19360	Rad50		-0.062843216	0.99988
1840	12750	Clk4	57396	-0.55397287	0.33906
1841	66397	Sar1b	51128	0.004432735	0.99988
1842	76901	Jade2	23338	0.635947372	0.48409
1843	30794	Pdlim4	8572	0.3688814	0.68262
1844	22210	Ube2b	7320	0.123844599	0.8024
1845	52626	Cdkn2aipnl	91368	0.067892459	0.99988
1846	84035	Kremen1	83999	0.969162999	0.24222
1847	380684	Nefh	4744	0.073040429	0.95348

1848	66213	Med7	9443	1.024061184	0.20048
1849	171285	Havcr2	84868		0.40878
1850	57783	Tnip1	10318	0.543271641	0.37246
1851	22333	Vdac1	7416	-0.25254703	0.83807
1852	16204	Fabp6	2172	0.911807117	0.17356
1853	22271	Upp1	7378	2.317119451	0.00388
1854	193116	Slu7	10569	-0.597088355	0.34305
1855	75452	Ascc2	84164	-0.736749257	0.30663
1856	15574	Hus1	3364	0.594125495	0.37969
1857	30939	Pttg1		0.822668104	0.01281
1858	545938	Zfp607a		1.234006101	0.26897
1859	319939	Tns3	64759	0.369394535	0.38376
1860	12227	Btg2	7832	-0.416511949	0.57901
1861	71962	Castor1	652968	-0.240310179	0.99988
1862	16009	Igfbp3	3486	1.06503761	0.19344
1863	64934	Pes1	23481	-0.193886149	0.95534
1864	21452	Tcn2	6948	-0.05220014	0.99988
1865	246177	Myo1g	64005	0.788194695	0.23819
1866	29856	Smtn	6525	-0.448543109	0.93113
1867	11844	Arf5	381	0.096921136	0.97582
1868	67862	310033P09R	79169	-0.347218768	0.68076
1869	14923	Guk1	2987	0.219641986	0.60462
1870	193670	Rnf185	91445	-0.35161432	0.62133
1871	16886	Limk2	3985	-0.49785147	0.42125
1872	56218	Patz1	23598	-0.203047692	0.84916
1873	74203	Eif4enif1	56478	0.176394365	0.99988
1874	94091	Trim11	81559	-0.458258748	0.42576
1875	18293	Ogdh	4967	-0.337278209	0.56106
1876	13494	Drg1	4733	0.436371231	0.6198
1877	68585	Rtn4	57142	-0.081652919	0.89975
1878	76784	Mtif2	4528	-0.047575422	0.99988
1879	78294	Rps27a		0.552085944	0.22718
1880	216618	Cfap36	112942	0.221512804	0.18424
1881	104570	Ppp4r3b	57223	-0.258527157	0.73522
1882	71701	Pnpt1	87178	-0.166434009	0.94878
1883	216616	Efemp1	2202	0.917511503	0.79697
1884	18972	Pold2	5425	-1.50721316	0.17465
1885	268417	Zkscan17	84838	-0.14057969	0.86942
1886	11568	Aebp1	165	1.20490393	0.3051
1887	54125	Polm	27434	-1.062937646	0.27937
1888	13169	Dbnl	28988	0.067397878	0.90569
1889	64660	Mrps24	64951	0.053326222	0.99988
1890	104479	Ccdc117	150275	0.002417487	0.99989
1891	68097	Dynll2	140735	-0.414351467	0.73182
1892	22433	Xbp1	7494	0.207288748	0.43622
1893	20922	Supt4a	6827	0.349899051	0.68231
1894	18952	Septin4		1.094851208	0.04733
1895	66140	Ska2	348235	0.201819808	0.9787
1896	74133	Smg8	55181	-0.071088624	0.98477
1897	108660	Rnf187		0.330607817	0.66181

1898	56427	Tubd1	51174	0.547104286	0.01946
1899	216767	Mrpl22	29093	1.009007293	0.02443
1900	69125	Cnot8	9337	-0.307753197	0.75288
1901	72508	Rps6kb1		-0.674138896	0.34794
1902	50724	Sap30l	79685	0.170797193	0.99988
1903	171212	Galnt10	55568	-0.982820247	0.24531
1904	76892	Rnft1	51136	-0.220137843	0.8493
1905	216760	Mfap3	4238	-0.122157054	0.89195
1906	67726	Fam114a2	10827	-0.11040428	0.99988
1907	53892	Ppm1d	8493	0.025135798	0.99988
1908	448850	Znhit3	9326	0.491894658	0.72244
1909	66196	Myo19	80179	0.693315718	0.1752
1910	212627	Prpsap2	5636	0.254730326	0.92099
1911	217039	Ggnbp2	79893	-0.468925703	0.50862
1912	107476	Acaca	31	-0.80937219	0.30766
1913	20425	Shmt1	6470	-0.51803304	0.48743
1914	16897	Llgl1	3996	0.638853242	0.23718
1915	13495	Drg2	1819	0.594678923	0.99315
1916	20787	Srebf1	6720	0.142513456	0.83176
1917	71943	Tom1l1	10040	-0.339136028	0.45442
1918	20913	Stxbp4	252983	0.797436859	0.36073
1919	66912	Bzw2	28969	-2.282688641	0.04111
1920	68626	Elac2	60528	0.130839685	0.99988
1921	18559	Pctp	58488	0.064932279	0.99988
1922	28071	Polr1f	221830	-0.59710986	0.39703
1923	380753	Atxn7l1	222255	-0.909879745	0.28119
1924	19027	Sypl	6856	0.966190603	0.01314
1925	71853	Pdia6	10130	0.277166084	0.3547
1926	59027	Nampt	10135	0.401867454	0.06685
1927	30955	Pik3cg	5294	0.782005313	0.24531
1928	71169	Nbas	51594	0.185775111	0.86369
1929	66109	Tspan13	27075	-1.127753823	0.21865
1930	19878	Rock2	9475	-0.615160646	0.45676
1931	23795	Agr2	10551	-0.507195315	0.4296
1932	17775	Laptm4a	9741	0.92607536	0.00972
1933	76820	Cyria	81553	0.255579024	0.61626
1934	217463	Snx13	23161	0.373473685	0.75763
1935	20969	Sdc1	6382	-0.022602669	0.99988
1936	14245	Lpin1	23175	0.166386501	0.99988
1937	80913	Pum2	23369	0.220593647	0.97675
1938	217410	Trib2	28951	1.840668102	0.00652
1939	58240	Hs1bp3	64342	0.297695541	0.83579
1940	105005	Lratd1	151354	-0.648679284	0.45844
1941	67241	Smc6	79677	-0.70189569	0.30663
1942	13929	Amz2	51321	-0.306086666	0.98653
1943	14674	Gna13	10672	-0.292061315	0.99988
1944	19084	Prkar1a	5573	0.668613008	0.01605
1945	208659	Fam20a	54757	1.058689013	0.29464
1946	27404	Abca8b	10351	0.71612555	0.25487
1947	105014	Rdh14	57665	0.511058197	0.52811

1948	26399	Map2k6	5608		0.40878
1949	208439	Klhl29	114818	-0.053449843	0.99988
1950	217449	Trappc12	51112	0.260976262	0.40843
1951	104923	Adi1	55256	-0.345887031	0.58914
1952	19819	Rnaseh1		0.260586217	0.92997
1953	217379	Ubxn2a	165324	0.556401259	0.02776
1954	22169	Cmpk2	129607	-0.706854208	0.34415
1955	20403	Itsn2	50618	-0.459309005	0.41105
1956	58185	Rsad2	91543	-1.406324029	0.10918
1957	108089	Rnf144a	9781	-0.22618408	0.99988
1958	15902	Id2	3398	0.039019243	0.98645
1959	67216	Mboat2	129642	-0.732664449	0.48253
1960	17977	Ncoa1	8648	-0.534802542	0.43674
1961	20135	Rrm2	6241	-0.374819775	0.82504
1962	12033	Bcap29	55973	0.107485551	0.99988
1963	23985	Slc26a4	5172	-3.290288963	0.01168
1964	52504	Cenpo	79172	0.311407463	0.99988
1965	194655	Klf11	8462	1.751367416	0.00566
1966	195733	Grhl1	29841	-0.176733993	0.87985
1967	217378	Dnajc27	51277	0.88488321	0.18281
1968	104836	Cbll1	79872	-0.071197541	0.99988
1969	13435	Dnmt3a	1788	-0.28760875	0.62539
1970	13382	Dld	1738	0.558882406	0.01881
1971	16570	Kif3c	3797	-0.806067611	0.87834
1972	24057	Sh3yl1	26751	0.312330921	0.83807
1973	19325	Rab10	10890	-0.220792113	0.76421
1974	69675	Pxdn	7837	0.215501088	0.87288
1975	20292	Ccl11	6356	-0.22505754	0.99656
1976	78394	Ddx52	11056	-0.514244462	0.45203
1977	21410	Hnf1b	6928	-0.756865911	0.57989
1978	70439	Taf15	8148	-0.106436316	0.89785
1979	11421	Ace	1636	-1.688987799	0.15099
1980	217232	Cdc27	996	0.028800028	0.9519
1981	16416	Itgb3		0.3342379	0.66583
1982	52686	Mettl2		-1.099301095	0.20996
1983	217011	Nle1	54475	0.320005673	0.77982
1984	24086	Tlk2	11011	0.345731045	0.04816
1985	67338	Rffl	117584	0.875528583	0.11536
1986	16882	Lig3	3980	-0.555101357	0.4362
1987	26406	Map3k3	4215	-0.073061462	0.99988
1988	72047	Ddx42	11325	-0.980852763	0.25652
1989	56095	Ftsj3	117246	-0.42277738	0.47039
1990	19184	Psmc5	5705	0.06642737	0.84632
1991	78943	Ern1	2081	0.498217757	0.37879
1992	18015	Nf1	4763	-0.995930385	0.29737
1993	18613	Pecam1	5175	1.302650821	0.01989
1994	50776	Polg2	11232	0.204020964	0.57303
1995	13207	Ddx5	1655	-0.423097638	0.41569
1996	66997	Psmd12	5718	0.290252188	0.53504
1997	78455	Helz	9931	-0.758574272	0.28853

1998	76380	Cep112	201134	1.728438507	0.13094
1999	58222	Rab37	326624	0.1749023	0.79078
2000	26941	Slc9a3r1	9368	-0.848297409	0.33214
2001	50773	Nt5c	30833	-0.294067912	0.75419
2002	15374	Jpt1	51155	-0.550967379	0.43354
2003	170930	Sumo2	6613	0.229490214	0.41633
2004	445007	Nup85	79902	0.511137909	0.07801
2005	260302	Gga3	23163	0.260040653	0.79983
2006	74148	Cluh	23277	-0.20228757	0.97675
2007	69674	Mif4gd	57409	0.700486378	0.27338
2008	67283	Slc25a19	60386	1.404870228	0.08867
2009	18472	Pafah1b1	5048	-0.14834072	0.95306
2010	71947	Tmem94	9772	-0.517568424	0.87985
2011	170472	Recql5	9400	0.423400537	0.48182
2012	57230	Sap30bp	29115	-0.313998014	0.80152
2013	192897	Itgb4	3691	-2.594194145	0.07827
2014	14635	Galk1	2584	-0.224360823	0.62766
2015	217331	Unk	85451	0.969132061	0.30282
2016	217333	Trim47	91107	1.803461841	0.01051
2017	11484	Aspa	443	0.127750347	0.99988
2018	60441	Mrpl38	64978	-0.133327827	0.87037
2019	217335	Fbf1	85302	0.145616557	0.99988
2020	11430	Acox1	51	-0.016845864	0.99589
2021	69535	Ten1		-0.05309661	0.99988
2022	217337	Srp68	6730	0.02567309	0.99988
2023	217325	Llgl2	3993	0.301890771	0.40563
2024	66874	Ncbp3	55421	0.431729554	0.41147
2025	55984	Camkk1	84254	0.461048932	0.46884
2026	53313	Atp2a3	489	-0.868436119	0.35482
2027	11736	Ankfy1	51479	-0.084673822	0.99988
2028	53413	Exoc7	23265	-0.935401837	0.32303
2029	67128	Ube2g1	7326	0.070128216	0.99988
2030	67279	Med31	51003	0.542601499	0.30722
2031	217342	Ube2o	63893	-0.883728853	0.3364
2032	52700	Txndc17	84817	0.634260927	0.23969
2033	217344	Rhbdf2	79651	-0.205468644	0.90322
2034	114886	Cygb	114757	0.426862675	0.63286
2035	66293	Snhg16		-0.842446402	0.45013
2036	67622	Mxra7		0.920650771	0.01242
2037	54189	Rabep1	9135	-0.297485081	0.50868
2038	69900	Mfsd11	79157	0.891275532	0.27172
2039	16562	Kif1c	10749	0.29175632	0.63383
2040	74136	Sec14l1	6397	-0.033930427	0.99988
2041	50932	Mink1	50488	0.754637851	0.3405
2042	18806	Pld2	5338	-1.04933658	0.2533
2043	52466	Slc46a1	113235	0.715163889	0.22843
2044	104457	610010K14Ri	124944	0.84629845	0.01557
2045	70451	Dhrs13	147015	-1.347904404	0.17456
2046	66601	Tmigd1	388364	0.466209788	0.3667
2047	104184	Blmh	642	-0.076864375	0.8994

2048	12874	Cpd	1362	0.076853922	0.99988
2049	56322	Timm22	29928	0.18361033	0.99988
2050	18230	Nxn	64359	-0.020360994	0.99988
2051	76566	Rflnb	359845	-0.350071108	0.42226
2052	380714	Rph3al	9501	0.121347273	0.99988
2053	13447	Doc2b	8447	-1.381474258	0.1865
2054	22627	Ywhae	7531	-0.40352813	0.44051
2055	192159	Prpf8	10594	0.242975846	0.39746
2056	18103	Nme2	4831	0.484218895	0.0279
2057	70834	Spag9	9043	-0.339806238	0.92239
2058	67684	Luc7l3	51747	0.066917204	0.95641
2059	71452	Ankrd40	91369	0.021147231	0.99988
2060	76408	Abcc3	8714	-0.330802754	0.77629
2061	217119	Xylt2	64132	0.240865634	0.37037
2062	98238	Lrrc59	55379	0.208341128	0.43752
2063	110172	Slc35b1	10237	-0.381087182	0.50521
2064	74479	Snx11	29916	-0.10299029	0.87025
2065	217140	Scrn2	90507	-0.533401512	0.50631
2066	72194	Fbxl20	84961	-0.414363542	0.55573
2067	13385	Dlg4	1742	1.294919267	0.01827
2068	13543	Dvl2	1856	0.103690906	0.99988
2069	217166	Nr1d1	9572	-0.061823964	0.99988
2070	18626	Per1	5187	0.950181535	0.01542
2071	22318	Vamp2	6844	0.079914968	0.99988
2072	66910	Tmem107	84314	0.46331817	0.59884
2073	20877	Aurkb	9212	-0.021985144	0.99988
2074	68964	Ctc1	80169	-0.987130541	0.24554
2075	237823	Pfas	5198	-0.266787148	0.72294
2076	77579	Myh10	4628	0.740913199	0.45207
2077	320207	Pik3r5	23533	0.590540546	0.39574
2078	18208	Ntn1	9423	1.011191744	0.36971
2079	55943	Stx8	9482	-0.106871709	0.99988
2080	216835	Usp43	124739	-0.351211799	0.84891
2081	66358	Adprm	56985	0.547080307	0.42122
2082	16669	Krt19	3880	-0.620826903	0.54338
2083	21973	Top2a	7153	-1.598015912	0.11022
2084	104112	Acly	47	0.955953149	0.01186
2085	14534	Kat2a	2648	0.651081971	0.68609
2086	20851	Stat5b	6777	1.483728355	0.00862
2087	76547	Tmem101	84336	-0.92179429	0.30659
2088	268490	Lsm12	124801	-0.185132581	0.85289
2089	21429	Ubtf	7343	-0.402387787	0.59613
2090	52715	Ccdc43	124808	-1.183275971	0.22712
2091	75689	Higd1b	51751	1.217857811	0.00906
2092	20624	Eftud2	9343	0.521282519	0.08608
2093	73293	Ccdc103	388389	-0.124503112	0.93222
2094	68087	Dcakd	79877	0.747370021	0.01965
2095	18107	Nmt1	4836	-0.286369093	0.79237
2096	72469	Plcd3	113026	-0.151362262	0.80638
2097	53859	Map3k14	9020	-0.059887744	0.99988

2098	56494	Gosr2		-0.185167947	0.68231
2099	66689	Klhl28	54813	-0.749188232	0.41755
2100	30795	Fkbp3	2287	0.864926051	0.02499
2101	76983	Scfd1	23256	-0.03623045	0.99988
2102	94186	Strn3	29966	0.003542966	0.99988
2103	11782	Ap4s1	11154	0.573981306	0.49774
2104	328092	Dtd2	112487	-0.537615911	0.58036
2105	108800	Ston2	85439	-0.742934562	0.35568
2106	83602	Gtf2a1	2957	0.001217343	0.99989
2107	20338	Sel1l	6400	0.143583507	0.77941
2108	109065	Dnaaf2	55172	0.079244624	0.99988
2109	18974	Pole2		-0.181770239	0.7595
2110	69554	Klhdc2	23588	-0.027176732	0.99988
2111	66244	Nemf	9147	-1.216007607	0.14614
2112	20334	Sec23a	10484	0.941201072	0.01054
2113	217666	L2hgdh	79944	1.07873853	0.00926
2114	78232	Trappc6b	122553	0.276736746	0.78707
2115	18949	Pnn	5411	-0.221404083	0.98645
2116	338320	Mia2		0.093292463	0.91648
2117	14420	Galc	2581	0.506612421	0.02514
2118	104871	Spata7	55812	-0.680555758	0.78781
2119	24000	Ptpn21	11099	-0.154319751	0.96992
2120	75553	Zc3h14	79882	-0.260283696	0.81388
2121	76260	Ttc8	123016	1.090358029	0.0123
2122	245841	Polr2h	5437	-0.041179217	0.98105
2123	59032	Ppp2r3c	55012	-0.314561874	0.99988
2124	66132	Prorp		0.193759948	0.89264
2125	26443	Psma6	5687	0.67410914	0.02595
2126	18035	Nfkbia	4792	0.012578774	0.99053
2127	56784	Ralgapa1	253959	-0.34348077	0.56609
2128	217588	Mbip	51562	-0.128437093	0.99988
2129	14874	Gstz1	2954	-0.63228809	0.46166
2130	20773	Sptlc2	9517	0.085959586	0.96733
2131	217737	Ahsa1	10598	-0.697656091	0.28598
2132	104799	Vipas39	63894	0.237399452	0.98407
2133	66354	Snw1	22938	-1.93375182	0.07306
2134	380773	Slirp	81892	0.344021686	0.0348
2135	72113	Adck1	57143	-0.171781899	0.86113
2136	664883	Nova1	4857	-0.081243718	0.99988
2137	108156	Mthfd1	4522	-0.089694223	0.99988
2138	26932	Ppp2r5e	5529	0.212624983	0.61583
2139	81535	Sgpp1	81537	-0.498140991	0.37019
2140	238276	Akap5	9495	1.131587808	0.10123
2141	20741	Sptb	6710	-0.445746525	0.6316
2142	104886	Rab15	376267	-0.154368815	0.99988
2143	53618	Fut8	2530	0.046380648	0.99988
2144	73991	Atl1	51062	-0.049371271	0.96948
2145	64010	Sav1	60485	0.710908882	0.11767
2146	18080	Nin	51199	-1.019146305	0.23451
2147	110095	Pygl	5836	1.315984955	0.12406

2148	12062	Bdkrb2	624	0.351626214	0.31233
2149	72736	Tmx1	81542	1.15968021	0.00903
2150	56444	Actr10	55860	0.712312614	0.09532
2151	30056	Timm9	26520	0.730630686	0.37018
2152	104001	Rtn1	6252	2.327961282	0.00388
2153	20716	Serpina3n		1.356563316	0.00544
2154	66375	Dhrs7	51635	0.0570237	0.99988
2155	19042	Ppm1a		-0.198324129	0.49281
2156	94040	Clmn	79789	-1.254961229	0.18298
2157	73046	Glrx5	51218	-0.315552268	0.45625
2158	17420	Mnat1	4331	-0.062210666	0.99988
2159	18755	Prkch		0.68566376	0.01855
2160	15251	Hif1a	3091	0.268923938	0.05271
2161	18789	Papola	10914	0.249297823	0.66836
2162	56217	Mpp5	64398	0.050255582	0.99988
2163	75627	Snapc1		0.785194134	0.52811
2164	73834	Atp6v1d	51382	-0.125266067	0.99988
2165	22367	Vrk1	7443	0.729892433	0.20947
2166	13665	Eif2s1	1965	0.178773964	0.48715
2167	27260	Plek2	26499	0.65998534	0.57243
2168	110417	Pigh	5283	0.254524879	0.44448
2169	53612	Vti1b	10490	-0.51779466	0.40517
2170	12192	Zfp36l1	677	-0.02959578	0.90295
2171	108760	Galnt16	57452	1.310833835	0.00544
2172	13877	Erh	2079	-0.401786575	0.58716
2173	217684	Susd6	9766	0.529191203	0.55816
2174	20384	Srsf5	6430	-0.258659409	0.65658
2175	64075	Smoc1	64093	1.337502681	0.20468
2176	105940408	Gm20498	100529257	0.687526187	0.40787
2177	54604	Pcnx	22990	-0.283537448	0.6903
2178	217893	Pacs2	23241	0.275377378	0.35347
2179	116870	Mta1	9112	0.159129521	0.82667
2180	207615	Wdr37	22884	-1.095132118	0.20323
2181	69237	Gtpbp4	23560	0.239162675	0.0783
2182	66505	Zmynd11	10771	-0.812536885	0.31855
2183	52635	Esyt2	57488	-0.061380925	0.99716
2184	217946	Cdca7l	55536	-0.79407213	0.26089
2185	104884	Tdp1	55775	0.482357275	0.40986
2186	19179	Psmc1	5700	0.005355872	0.99988
2187	217827	Nrde2	55051	-0.566376853	0.40878
2188	73086	Rps6ka5	9252	-0.834171328	0.45745
2189	68339	Ccdc88c	440193	-0.662261386	0.37659
2190	217830	Dglucy	80017	-0.721094023	0.47329
2191	23876	Fbln5	10516	1.060183179	0.34692
2192	74413	Tc2n	123036	-0.692009478	0.33823
2193	109181	Trip11	9321	-0.877356867	0.24716
2194	110616	Atxn3	92552	-0.49040386	0.35657
2195	19141	Lgmn	5641	-0.031269049	0.99988
2196	27277	Golga5	9950	-0.36163638	0.72018
2197	69617	Pitrm1	10531	0.702408567	0.54815

2198	56421	Pfkp	5214	0.384773485	0.92668
2199	65256	Asb2	51676	3.057465097	0.00388
2200	622402	Akr1c12		0.095357917	0.93088
2201	27384	Akr1c13		-0.879487449	0.28616
2202	56349	Net1	10276	0.397103732	0.2601
2203	243931	Tshz3	57616	0.904448525	0.58979
2204	14569	Gdi2	2665	-0.004781394	0.99988
2205	73828	Dcaf4		1.091695822	0.09498
2206	18222	Numb	8650	-1.062212653	0.18016
2207	171210	Acot2		-0.040818146	0.89195
2208	217707	Coq6	51004	-0.374895479	0.68927
2209	12499	Entpd5	957	-0.47627513	0.80895
2210	104776	Aldh6a1	4329	-0.413229272	0.51608
2211	19300	Abcd4	5826	0.000906231	0.99988
2212	74316	Isca2	122961	-0.104533909	0.99294
2213	67963	Npc2	10577	0.123272867	0.78548
2214	73736	Fcf1		-0.56026485	0.58979
2215	56531	Ylpm1	56252	-0.638396189	0.38014
2216	217716	Mlh3	27030	-0.135251124	0.99988
2217	68581	Tmed10	10972	-0.168169459	0.78469
2218	14281	Fos	2353	1.184607	0.02724
2219	58520	Erg28	11161	-0.372632085	0.73291
2220	21809	Tgfb3	7043	0.652067906	0.3353
2221	70373	Gpatch2l	55668	-0.89810013	0.68273
2222	68737	Angel1	23357	0.709707884	0.40271
2223	12454	Ccnk	8812	0.331125384	0.08198
2224	214305	Hhipl1	84439	0.840806308	0.78079
2225	14026	Evl	51466	0.690010293	0.46294
2226	70059	Degs2	123099	1.447504824	0.00797
2227	22632	Yy1	7528	-0.2103368	0.74202
2228	214663	Slc25a29	123096	-1.763824868	0.09131
2229	22375	Wars	7453	-0.642869678	0.35494
2230	17263	Meg3		-0.118869757	0.99988
2231	15519	Hsp90aa1		-0.857888676	0.18211
2232	72805	Zfp839	55778	0.432872441	0.98653
2233	14137	Fdft1	2222	-0.260981864	0.48073
2234	104859	Tecpr2	9895	-0.647548699	0.72035
2235	67236	Cinp	51550	-0.343272311	0.62302
2236	22031	Traf3	7187	0.635922601	0.51226
2237	93835	Amn	81693	-0.557260133	0.6841
2238	217866	Cdc42bpb	9578	-2.162112379	0.08319
2239	74190	Exoc3l4	91828	-0.552220097	0.73572
2240	21928	Tnfaip2	7127	0.331364329	0.39872
2241	217869	Eif5	1983	-0.410758612	0.44925
2242	21981	Ppp1r13b	23368	0.043199419	0.99988
2243	68520	Zfyve21	79038	0.55795403	0.54834
2244	16593	Klc1		1.179489628	0.00972
2245	70257	Atp5mpl	9556	0.583968753	0.03617
2246	668303	Kif26a	26153	0.218495654	0.95504
2247	56696	Gpr132		1.339940395	0.02314

2248	14593	Ggps1	9453	-0.14121843	0.991
2249	83924	Gpr137b	7107	0.650640823	0.22364
2250	238505	Mtr	4548	1.388334368	0.00894
2251	14634	Gli3	2737	0.529841415	0.02203
2252	20379	Sfrp4	6424	1.360707297	0.00833
2253	27052	Aoah	313	1.264748219	0.00952
2254	19720	Trim27	5987	-0.442602544	0.46791
2255	72739	Zkscan3		-0.164049286	0.80923
2256	68732	Carmil1	55604	-0.89454435	0.45637
2257	380836	Mrs2	57380	-0.537375934	0.41537
2258	66482	Exoc2	55770	0.708928312	0.26511
2259	21418	Tfap2a	7020	-1.644282877	0.17817
2260	14538	Gcnt2	2651	-0.214714028	0.83434
2261	66154	Tmem14c	51522	0.173483098	0.99988
2262	18003	Nedd9	4739	1.307321668	0.00798
2263	110521	Hivep1	3096	0.19398665	0.99988
2264	13614	Edn1	1906	-2.026984628	0.05097
2265	76137	Mcur1	63933	-0.467403662	0.57803
2266	218210	Nup153	9972	0.959592272	0.0128
2267	16553	Kif13a	63971	0.514819723	0.05676
2268	22017	Tpmt	7172	-1.195884499	0.26469
2269	110052	Dek	7913	-0.488420863	0.51701
2270	15904	Id4	3400	0.172935934	0.99988
2271	66329	Susd3	203328	1.55809854	0.00591
2272	75678	Ippk	64768	0.253929679	0.99988
2273	66695	Aspn	54829	1.573411252	0.18174
2274	18295	Ogn	4969	0.927005195	0.75016
2275	66336	Cenpp	401541	-0.232701965	0.72724
2276	70930	Nol8	55035	0.285971104	0.63226
2277	20729	Spin1	10927	0.316970482	0.41147
2278	78903	Wrnip1	56897	-0.357310935	0.91657
2279	19766	Ripk1	8737	0.058035422	0.99988
2280	66895	Pxdc1	221749	1.10132214	0.3364
2281	19134	Prpf4b	8899	-1.258060767	0.15428
2282	69123	Eci3		-3.205947474	0.01649
2283	23986	Eci2		0.414146617	0.28343
2284	208366	Rpp40	10799	-0.360463484	0.99988
2285	69955	Fars2	10667	0.608001566	0.50713
2286	17084	Ly86	9450	0.432151788	0.07193
2287	107513	Ssr1	6745	-0.585174577	0.6089
2288	71340	Riok1	83732	-0.28236509	0.57865
2289	67797	Snrnp48	154007	-0.409362902	0.49396
2290	108652	Slc35b3	51000	-0.024922345	0.99988
2291	20354	Sema4d	10507	0.933365103	0.85142
2292	23882	Gadd45g	10912	-0.386952644	0.75157
2293	14120	Fbp2	8789	1.181947317	0.2905
2294	20963	Syk	6850	1.659017435	0.00797
2295	72061	Aopep	84909	-0.440521694	0.42355
2296	11992	Auh	549	0.382771004	0.27447
2297	14088	Fancc	2176	-0.748799337	0.3273

2298	26564	Ror2	4920	0.378648977	0.26928
2299	19206	Ptch1	5727	-0.195917893	0.99988
2300	268656	Sptlc1	10558	-0.279187849	0.58242
2301	17702	Msx2	4488	0.140663403	0.99988
2302	76251	Ercc6l2		0.445048026	0.75677
2303	14057	Sfxn1	94081	-0.431953179	0.65419
2304	56541	Habp4	22927	0.369398645	0.38286
2305	13039	Ctsl		0.117640245	0.99988
2306	26919	Zfp346	23567	1.053312809	0.00972
2307	66129	Prxl2c	195827	-0.032409568	0.99988
2308	105278	Cdk20	23552	-0.550504366	0.59203
2309	66890	Lman2	10960	0.649024843	0.02278
2310	17121	Mxd3	83463	-1.046682284	0.21319
2311	66494	Prelid1		0.132732824	0.99988
2312	18193	Nsd1	64324	-0.295518456	0.62732
2313	67399	Pdlim7	9260	0.900050486	0.03103
2314	72935	Ddx41	51428	-0.163667658	0.99988
2315	212483	Fam193b	54540	-1.098130703	0.35105
2316	72562	Pcbd2	84105	0.672107897	0.19519
2317	69672	Txndc15	79770	0.269477455	0.80176
2318	212880	Ddx46	9879	-0.739933057	0.2905
2319	12328	Caml	819	-1.749839605	0.12618
2320	218271	B4galt7	11285	-0.182659256	0.93998
2321	57266	Cxcl14	9547	1.923930576	0.00564
2322	328258	Slc25a48	153328	-0.72770512	0.35433
2323	212281	Zfp729a		1.888732208	0.01018
2324	170936	Zfp369		0.314119844	0.10059
2325	19210	Ptdss1	9791	0.081372335	0.77455
2326	66410	Mterf3	51001	0.821729933	0.10048
2327	67530	Uqcrb	7381	1.10283544	0.0079
2328	69577	Fastkd3	79072	0.864262104	0.20008
2329	12626	Cetn3	1070	0.262633222	0.0681
2330	17129	Smad5	4090	-0.521311351	0.43977
2331	15387	Hnrnpk	3190	0.191933911	0.82477
2332	66671	Ccnh	902	0.547986503	0.22567
2333	218397	Rasa1	5921	-0.243374224	0.65122
2334	70153	210016F16Ri	84267	0.250232516	0.72715
2335	56278	Gkap1	80318	-0.081740778	0.93077
2336	114304	Slc28a3	64078	-1.02439221	0.24661
2337	78689	Naa35	60560	-0.148024441	0.99977
2338	105348	Golm1	51280	0.448225821	0.54849
2339	67269	Agtpbp1	23287	1.6751834	0.00664
2340	69635	Dapk1	1612	0.135430583	0.99988
2341	72293	Nkd2	85409	0.505652302	0.05215
2342	69716	Trip13	9319	0.285102929	0.97042
2343	18570	Pdcd6	10016	0.157398074	0.78646
2344	66945	Sdha		0.026782861	0.99988
2345	67433	Ccdc127	133957	0.022031234	0.99988
2346	80898	Erap1	51752	0.460284452	0.26955
2347	12380	Cast	831	-0.623405887	0.30673

2348	73296	Rhobtb3	22836	0.490159719	0.02883
2349	93692	Glrx	2745	0.125476152	0.75527
2350	77041	Arsk	153642	-1.109290338	0.2865
2351	78925	Srd5a1	6715	0.50550149	0.0189
2352	28114	Nsun2	54888	-0.285611075	0.78781
2353	78771	Mctp1	79772	0.801836762	0.0096
2354	105377	Slf1	84250	0.390660659	0.99656
2355	28077	Med10		0.499387893	0.24145
2356	407785	Ndufs6	4726	-0.240708885	0.64119
2357	94066	Mrpl36	64979	-0.140674344	0.99816
2358	210992	Lpcat1	79888	0.809412473	0.01867
2359	218335	Clptm1l	81037	0.516057001	0.10431
2360	21752	Tert	7015	-0.109916258	0.94097
2361	13003	Vcan	1462	0.249966543	0.99988
2362	108138	Xrcc4	7518	-0.802664557	0.59884
2363	66795	Atg10	83734	1.115042992	0.2076
2364	69085	Zcchc9	84240	-0.793476271	0.28041
2365	17079	Cd180	4064	-0.680491014	0.40418
2366	69048	Slc30a5	64924	-0.421654553	0.93776
2367	19356	Rad17	5884	0.37834783	0.07394
2368	218518	Marveld2	153562	-0.970456709	0.23135
2369	18260	Ocln	100506658	0.013929508	0.99988
2370	23894	Gtf2h2		0.29021638	0.5815
2371	17940	Naip1		-0.113234381	0.79595
2372	20365	Serf1	8293	0.093285188	0.74391
2373	20595	Smn1		-0.190782038	0.69368
2374	78038	Mccc2	64087	0.092481709	0.9292
2375	68927	Ptcd2	79810	-0.377972059	0.82658
2376	218490	Btf3	689	-0.66844548	0.38023
2377	68558	Ankra2	57763	0.55802914	0.05731
2378	110596	Arhgef28	64283	-1.205791105	0.2058
2379	15212	Hexb	3074	0.442368822	0.10291
2380	320806	Gfm2	84340	-0.424082469	0.56044
2381	27015	Polk	51426	-0.399784614	0.67959
2382	68018	Cert1	10087	0.444143287	0.03336
2383	15357	Hmgcr	3156	0.704020427	0.03984
2384	67463	Poc5	134359	-0.453260194	0.45946
2385	544963	Iqgap2	10788	-0.931433945	0.37201
2386	14063	F2rl1	2150	-0.545588647	0.43471
2387	66549	Aggf1		0.174828853	0.99988
2388	218461	Pde8b		-0.083015109	0.99988
2389	11774	Ap3b1	8546	-0.148929383	0.61803
2390	107767	Scamp1	9522	0.027063811	0.99988
2391	57748	Jmy	133746	0.014743171	0.99988
2392	66254	Dimt1	27292	-0.382512718	0.99988
2393	16563	Kif2a	3796	-0.620667854	0.37332
2394	71991	Ercc8	1161	0.651902958	0.8309
2395	74559	Elovl7	79993	-0.311381455	0.69671
2396	238871	Pde4d	5144	-0.828815078	0.55034
2397	20620	Plk2	10769	-0.184261725	0.733

2398	218442	Serinc5	256987	0.250653764	0.9979
2399	382793	Mtx3	345778	-0.930436768	0.28284
2400	218441	Zfyve16		0.021870228	0.98919
2401	13361	Dhfr		-1.123189392	0.38014
2402	19418	Rasgrf2	5924	0.058623994	0.91589
2403	59079	Erbin	55914	-0.639165524	0.3987
2404	75805	Nln	57486	-0.459844181	0.81937
2405	66975	Trappc13	80006	0.349027625	0.7381
2406	81003	Trim23	373	0.072075972	0.99988
2407	238831	Ppwd1	23398	-0.795991185	0.27467
2408	60411	Cenpk	64105	0.050145214	0.99988
2409	67285	Cwc27	10283	0.505832282	0.09185
2410	67288	Srek1ip1	285672	-0.334377803	0.49077
2411	52882	Rgs7bp	401190	-0.941150852	0.22871
2412	71816	Rnf180	285671	1.252644672	0.06285
2413	52552	Parp8	79668	-0.956393112	0.1946
2414	13723	Emb	133418	1.073029302	0.00933
2415	59054	Mrps30	10884	-0.040146425	0.8973
2416	14165	Fgf10	2255	1.347682922	0.00817
2417	218756	Slc4a7	9497	-0.851941776	0.24939
2418	66413	Psmd6	9861	0.171115863	0.41125
2419	246103	Atxn7	6314	-0.29665466	0.82473
2420		NA		0.353556643	0.65424
2421	74430	Cfap20dc	200844	1.153223182	0.01089
2422	68263	Pdhb	5162	0.630228322	0.22905
2423	18300	Oit1	131177	-2.161308284	0.06859
2424	93732	Acox2	8309	-0.282369612	0.7594
2425	71393	Kctd6	200845	0.369097356	0.72636
2426	26401	Map3k1	4214	-0.390746739	0.67674
2427	16195	Il6st	3572	0.696131751	0.51657
2428	69590	Gpx8	493869	0.371396991	0.37311
2429	17993	Ndufs4	4724	0.127437795	0.19247
2430	14313	Fst	10468	0.36588078	0.8309
2431	54169	Kat6b	23522	-0.373347409	0.66888
2432	67630	Samd8	142891	-0.009556399	0.99646
2433	22334	Vdac2	7417	0.06955968	0.99988
2434	69721	Nkiras1	28512	-0.173386477	0.99988
2435	69156	Comtd1	118881	0.001159324	0.99988
2436	22194	Ube2e1	7324	0.034336245	0.99988
2437	353187	Nr1d2	9975	1.106658121	0.00933
2438	21834	Thrb	7068	-0.167034567	0.90922
2439	71228	Dlg5	9231	-0.175796405	0.83528
2440	59007	Ngly1	55768	-0.141451703	0.92384
2441	71147	Oxsm	54995	-0.233569487	0.97675
2442	70564	Prxl2a	84293	-2.107620791	0.17196
2443	14661	Glud1		0.63066843	0.01701
2444	20390	Sftpd	6441	-1.955955755	0.11592
2445	12166	Bmpr1a	657	-0.487993305	0.70173
2446	170677	Cdhr1	92211	-0.354382622	0.96811
2447	68045	Rtraf	51637	0.609609801	0.01344

2448	67725	Nudt13	25961	0.575178821	0.02594
2449	70601	Ecd	11319	-0.729484882	0.29276
2450	108671	Dnajc9	23234	0.417678383	0.49408
2451	11750	Anxa7	310	0.156047648	0.95468
2452	74843	Mss51	118490	-1.14593508	0.1583
2453	19056	Ppp3cb	5532	0.14694321	0.14112
2454	268721	Zswim8	23053	-0.440619526	0.51485
2455	12325	Camk2g	818	0.827471092	0.01622
2456	22330	Vcl	7414	0.814900763	0.48431
2457	55946	Ap3m1	26985	0.10312463	0.92422
2458	70561	Txndc16	57544	0.025472342	0.99988
2459	50527	Ero1a	30001	-0.91601143	0.32935
2460	67089	Psmc6	5706	0.151692866	0.48351
2461	12159	Bmp4	652	0.858513425	0.03007
2462	74480	Samd4	23034	0.581022368	0.66885
2463	218975	Mapk1ip1l		0.062143803	0.99397
2464	16709	Ktn1	3895	-1.109862247	0.18257
2465	93834	Peli2	57161	2.903521012	0.00406
2466	11744	Anxa11	311	-0.501085634	0.44887
2467		NA		0.964593006	0.01357
2468	105675	Ppif	10105	-0.272548394	0.60168
2469	83997	Slmap	7871	-0.23929215	0.71204
2470	58809	Rnase4	6038	0.95264299	0.23751
2471	11843	Arf4	378	-0.18862531	0.79983
2472	78416	Rnase6	6039	0.845197006	0.0177
2473	56794	Hacl1		0.059562506	0.99988
2474	14744	Gpr65	8477	1.287481054	0.16247
2475	74427	Eaf1	85403	0.800953992	0.0128
2476	67011	Mettl6	131965	0.057364005	0.99988
2477	24056	Sh3bp5	9467	-0.791491021	0.42002
2478	12339	Capn7	23473	0.668731966	0.02588
2479	71704	Arhgef3	50650	-0.002056312	0.99988
2480	26363	Btd	686	1.032831099	0.01298
2481	104416	Bap1	8314	-0.982010413	0.27471
2482	71838	Phf7	51533	-0.995842376	0.26269
2483	78754	Galnt15	117248	-0.290786589	0.99988
2484	105638	Dph3	285381	0.350711998	0.04954
2485	218885	Oxnad1	92106	0.28113334	0.37299
2486	64652	Nisch	11188	0.12251504	0.90772
2487	26430	Parg	8505	-0.006328474	0.99638
2488	76485	Glt8d1	55830	1.053557356	0.03027
2489	69019	Spcs1	28972	0.43771283	0.4826
2490	23955	Nek4	6787	-0.22712972	0.69786
2491	68177	Ebpl	84650	-0.788091477	0.31728
2492	16648	Kpna3	3839	-0.972516921	0.28274
2493	66674	Spryd7	57213	-0.078116081	0.99988
2494	67153	Rnaseh2b	79621	0.329863401	0.48823
2495	26419	Mapk8	5599	-0.557986181	0.40182
2496	66645	Pspc1	55269	0.365653247	0.6966
2497	13030	Ctsb	1508	0.215413605	0.17535

2498	14560	Gdf10	2662	2.584492564	0.05945
2499	76007	Zmym2	7750	-0.316512699	0.52678
2500	68631	Cryl1	51084	-0.357030566	0.55831
2501	18753	Prkcd	5580	-0.182763308	0.72098
2502	11752	Anxa8		1.846630376	0.05912
2503	68043	Eef1akmt1	221143	0.643210885	0.02504
2504	57258	Xpo4	64328	-0.495288397	0.54205
2505	21881	Tkt	7086	-0.734295476	0.32717
2506	72400	Pinx1	54984	0.784242751	0.24705
2507	50523	Lats2		0.458387815	0.10001
2508	75901	Dcp1a	55802	-0.941490852	0.3837
2509	20220	Sap18		0.277974647	0.34451
2510	67840	Mrpl57	78988	0.281963718	0.71868
2511	75965	Zdhhc20	253832	-0.53960401	0.22712
2512	219150	Hmbox1	79618	-0.730891833	0.53005
2513	68514	Micu2	221154	0.319261212	0.05882
2514	14180	Fgf9	2254	2.290147846	0.0053
2515	210925	Ints9	55756	-0.566847171	0.37959
2516	54616	Extl3	2137	0.067099176	0.99988
2517	69008	Cab39l	81617	0.101721505	0.77708
2518	71891	Cdadc1	81602	0.143951929	0.99988
2519	50769	Atp8a2	51761	-0.416312283	0.96645
2520	219135	Mtmr6	9107	0.971600749	0.01456
2521	219140	Spata13		0.132895144	0.96441
2522	70478	Mipep	4285	-0.21485031	0.99988
2523	22418	Wnt5a	7474	1.450878653	0.13297
2524	13885	Esd	2098	0.269090894	0.75829
2525	18826	Lcp1	3936	0.776864153	0.01627
2526	67302	Zc3h13	23091	-0.20023511	0.70026
2527	67554	Slc25a30	253512	-0.247749138	0.61605
2528	67467	Gpalpp1		0.25334864	0.67946
2529	27275	Nufip1	26747	-1.80720813	0.12279
2530	21807	Tsc22d1	8848	-0.085999665	0.92393
2531	66148	Dnajc15	29103	-0.177210021	0.97933
2532	108670	Epsti1	94240	-0.180316173	0.99988
2533	219181	Akap11	11215	0.180607795	0.10328
2534	66214	Rgcc	28984	-0.307994393	0.98457
2535	219249	Tdrd3	81550	-0.093195468	0.99988
2536	66897	Naa16	79612	-0.58161036	0.53065
2537	56419	Diaph3	81624	-0.093909843	0.94328
2538	22380	Wbp4	11193	-0.179749104	0.65869
2539	67955	Sugt1	10910	0.462277798	0.03364
2540	380924	Olfm4	10562	2.279501792	0.21239
2541	74195	Elp3	55140	0.384841795	0.68102
2542	71145	Scara5	286133	0.153997897	0.99988
2543	52033	Pbk	55872	-0.877810104	0.27194
2544	67179	Ccdc25	55246	-0.518542564	0.54409
2545	12759	Clu	1191	-0.704797611	0.4267
2546	13850	Ephx2	2053	2.557414768	0.00438
2547	66854	Trim35	23087	0.808102894	0.01327

2548	12934	Dpysl2	1808	1.013328128	0.2058
2549	12177	Bnip3l	665	-0.905559545	0.26511
2550	71978	Ppp2r2a	5520	-0.384397218	0.4602
2551	13592	Ebf2	64641	-1.13573622	0.3273
2552	58860	Adamdec1	27299	-0.558144324	0.86129
2553	52023	Pibf1	10464	1.639939486	0.00719
2554	100862375	Entpd4b		-0.584803645	0.48422
2555	77744	Bora	79866	-1.352508614	0.37133
2556	21933	Tnfrsf10b		-0.232336371	0.93745
2557	246710	Rhobtb2	23221	0.747508163	0.07705
2558	57784	Bin3	55909	-1.595873868	0.15099
2559	213019	Pdlim2		0.775769109	0.01741
2560	213053	Slc39a14	23516	0.123711606	0.99988
2561	239170	Fam160b2	64760	-0.338078563	0.84064
2562	12153	Bmp1	649	0.503958773	0.1164
2563	65246	Xpo7	23039	-0.124553717	0.89847
2564	13449	Dok2	9046	2.030377039	0.00504
2565	14586	Gfra2	2675	1.954540078	0.00634
2566	19645	Rb1	5925	0.150048285	0.78259
2567	105670	Rcbtb2	1102	0.665748222	0.02395
2568	16432	Itm2b	9445	0.409231303	0.03528
2569	67381	Med4	29079	0.579036214	0.02833
2570	20916	Sucla2	8803	0.184576574	0.67812
2571	50933	Uchl3		0.035497732	0.99988
2572	24064	Spry2	10253	-0.944168007	0.30349
2573	74213	Rbm26	64062	-0.494855171	0.55659
2574	72486	Obi1	79596	1.099250202	0.1072
2575	50789	Fbxl3	26224	0.293389727	0.99988
2576	211286	Cln5		0.690179605	0.89572
2577	13190	Dct	1638	-0.749969331	0.64458
2578	76355	Tgds	23483	-1.104467586	0.01876
2579	58245	Gpr180	160897	0.520075825	0.56492
2580	58187	Cldn10	9071	-1.133210802	0.19344
2581	100037258	Dnajc3	5611	-0.089849333	0.89847
2582	105559	Mbnl2	10150	-0.977353375	0.24426
2583	71175	Nipbl	25836	-0.578424303	0.31475
2584	170762	Nup155	9631	0.548578091	0.25652
2585	18414	Osmr	9180	0.434893763	0.34726
2586	23880	Fyb	2533	0.355611068	0.98124
2587	13132	Dab2	1601	1.403156734	0.00583
2588	67515	Ttc33	23548	0.73926635	0.03541
2589	76338	Rab2b	84932	-0.490052645	0.53504
2590	56335	Mettl3	56339	-0.24134302	0.97809
2591	13135	Dad1	1603	0.416808204	0.58707
2592	65107	Lrp10	26020	-0.53637704	0.54528
2593	219072	Haus4		0.103439528	0.81258
2594	16475	Ajuba	84962	0.371156122	0.99988
2595	74359	931414P19R	60686	0.589401644	0.0123
2596	50934	Slc7a8	23428	1.225840733	0.01444
2597	12274	C6	729	0.025271585	0.99988

2598	106052	Fbxo4	26272	-0.278684966	0.58767
2599	56215	Acin1	22985	-0.931485237	0.1583
2600	67041	Oxct1	5019	-1.273248515	0.06935
2601	14000	Drosha	29102	0.395911296	0.62302
2602	19173	Psmb5	5693	0.455356642	0.40378
2603	12050	Pabpn1	8106	-0.96823344	0.36857
2604	77877	030458C11R	55322	0.20181745	0.91901
2605	68070	Pdzd2	23037	-0.869907472	0.30096
2606	59049	Slc22a17	51310	-1.119395816	0.36073
2607	66629	Golph3	64083	0.088979477	0.90074
2608	22763	Zfr	51663	-0.05072521	0.99988
2609	13644	Efs	10278	-0.031916767	0.99988
2610	68966	Ngdn	25983	0.261077142	0.04515
2611	20024	Sub1	10923	0.315163998	0.05873
2612	28200	Dhrs4		0.59457485	0.41499
2613	28199	Dcaf11	80344	0.645721116	0.40947
2614	19186	Psme1	5720	-0.178241123	0.79397
2615	85308	Emc9	51016	0.464586173	0.09687
2616	21816	Tgm1	7051		0.991
2617	104110	Adcy4	196883	1.551170869	0.10435
2618	56532	Ripk3	11035	0.504267976	0.03291
2619	654795	Sdr39u1	56948	-0.007790208	0.99988
2620	432731	Zscan26	7741	-0.24286493	0.991
2621	20356	Sema5a	9037	0.911296866	0.41621
2622	12465	Cct5	22948	-0.466411046	0.4134
2623	69574	Cmbl	134147	0.937417702	0.06062
2624	252967	Ropn1l	83853	0.802921612	0.24174
2625	67434	Ankrd33b	651746	-0.050133829	0.991
2626	18163	Ctnnd2	1501	0.190083513	0.91311
2627	110960	Tars	6897	0.066185342	0.85956
2628	17117	Amacr	23600	0.046425445	0.92651
2629	75646	Rai14	26064	-0.2737354	0.5796
2630	67832	Brix1	55299	0.520526999	0.32303
2631	19355	Rad1	5810	0.14951533	0.9063
2632	68646	Nadk2	133686	0.848089691	0.0155
2633	67154	Mtdh	92140	-0.419276216	0.41669
2634	114128	Laptm4b	55353	0.404447537	0.42759
2635	15529	Sdc2	6383	-0.244830642	0.99988
2636	223435	Trio	7204	-0.678022809	0.50292
2637	11732	Ank	56172	-1.425891479	0.15428
2638	66270	Retreg1	54463	-0.001945569	0.99988
2639	17909	Myo10	4651	-1.545526344	0.13588
2640	30945	Rnf19a	25897	-0.113169831	0.96864
2641	18458	Pabpc1		-0.575858261	0.58942
2642	22631	Ywhaz	7534	-0.534814732	0.29106
2643	252973	Grhl2	79977	-0.402815834	0.58233
2644	382985	Rrm2b	50484	0.183446975	0.99988
2645	66335	Atp6v1c1	528	0.052816432	0.68321
2646	14368	Fzd6	8323	-0.248008886	0.78261
2647	69906	Slc25a32		0.096198995	0.99988

2648	223499	Dcaf13	25879	-0.729477082	0.34642
2649	75766	Dcstamp	81501	0.214308833	0.99988
2650	239393	Lrp12	29967	0.680337307	0.05359
2651	22762	Zfpm2	23414	0.121812886	0.99988
2652	170719	Oxr1	55074	-0.305918277	0.68103
2653	11600	Angpt1	284	0.467140899	0.90708
2654	68135	Eif3h	8667	-0.557396244	0.34636
2655	78581	Utp23	84294	0.326122285	0.38618
2656	19357	Rad21	5885	-0.116950867	0.99988
2657	20419	Shcbp1	79801	-1.219238663	0.25527
2658	15473	Rida	10247	0.614475879	0.40066
2659	17181	Matn2	4147	2.158119449	0.0057
2660	67724	Pop1	10940	-0.434539516	0.59106
2661	56274	Stk3	6788	-1.026950814	0.25638
2662	13992	Khdrbs3	10656	0.54183727	0.40271
2663	16341	Eif3e	3646	-0.517854065	0.40878
2664	66736	Emc2	9694	-0.127015872	0.99988
2665	223527	Eny2	56943	0.221371669	0.74969
2666	55960	Ebag9	9166	0.398918353	0.64055
2667	319613	Sybu	55638	0.780677092	0.01732
2668	17869	Myc	4609	-0.105725034	0.99988
2669	223593	Washc5	9897	0.204738856	0.06822
2670	20775	Sqle	6713	-1.699016309	0.10666
2671	211401	Mtss1	9788	0.436827879	0.33986
2672	66218	Ndufb9	4715	-0.143121222	0.89867
2673	67731	Fbxo32	114907	-1.002536313	0.304
2674	76773	Wdyhv1	55093	-0.498718763	0.70199
2675	70472	Atad2		-0.855020054	0.18602
2676	22770	Zhx1	11244	0.314530462	0.62525
2677	210544	Tbc1d31	93594	-1.694978953	0.0155
2678	67819	Derl1	79139	0.280730026	0.64387
2679	105837	Mtbp	27085	-0.040558345	0.82596
2680	68537	Mrpl13	28998	0.731957863	0.17477
2681	12818	Col14a1	7373	0.669467895	0.8338
2682	20491	Sla	6503	2.181582452	0.00388
2683	13196	Asap1	50807	-0.082250944	0.89659
2684	223601	Cyrib	51571	1.076449919	0.00969
2685	22422	Wnt7b	7477	-0.370522771	0.72801
2686	29870	Gtse1	51512	-1.583088998	0.17439
2687	72026	Trmu	55687	-1.016800536	0.32204
2688	223770	Brd1	23774	-0.536889415	0.27917
2689	21685	Tef	7008	0.559631554	0.26606
2690	20286	Zc3h7b	23264	0.053757615	0.99988
2691	19387	Rangap1	5905	-0.624394119	0.52716
2692	214669	L3mbtl2	83746	-0.409745938	0.56901
2693	56438	Rbx1	9978	0.162669254	0.1077
2694	321003	Xpnpep3	63929	0.586863768	0.61668
2695	70356	St13	6767	-0.849921028	0.23202
2696	20524	Slc25a17	10478	-0.167863803	0.99988
2697	11564	Adsl	158	0.594780256	0.10434

2698	213956	Fam83f	113828	-0.470483261	0.69628
2699	239555	Mief1	54471	-1.052365844	0.22364
2700	66513	Tab1	10454	0.033393708	0.99988
2701	20972	Syngr1	9145	0.883441276	0.17992
2702	97998	Deptor	64798	0.270619198	0.72412
2703	54152	Dnal4		0.591615165	0.03563
2704	73340	Nptxr	23467	-1.756837349	0.15044
2705	72107	Dscc1	79075	0.503958925	0.37225
2706	18606	Enpp2	5168	1.133772153	0.18486
2707	74158	Josd1	9929	0.046502089	0.99988
2708	223696	Tomm22	56993	-0.083335341	0.92311
2709	73739	Cby1	25776	-0.059884527	0.94892
2710	27373	Csnk1e		-1.545541951	0.13347
2711	73225	Fam118a	55007	0.6620889	0.45863
2712	20401	Sh3bp1		0.219158164	0.66081
2713	68653	Samm50	25813	0.313220234	0.80601
2714	170736	Parvb	29780	1.637054749	0.0075
2715	64099	Parvg	64098		0.991
2716	72709	C1qtnf6	114904	0.25703971	0.99988
2717	319953	Ttll1	25809	-0.450639658	0.69316
2718	17886	Myh9	4627	-0.620082543	0.33333
2719	67130	Ndufa6	4700	0.425191373	0.0244
2720	19230	Twf1	5756	-0.394375248	0.38499
2721	69029	Smdt1	91689	0.095026198	0.99988
2722	17939	Naga	4668	-0.763739781	0.30663
2723	24050	Septin3	55964	1.959732835	0.00406
2724	67760	Slc38a2	54407	-0.318321397	0.64985
2725	20788	Srebf2	6721	-0.479156813	0.42122
2726	71919	Rpap3	79657	-0.602210655	0.30594
2727	19011	Endou	8909	0.383172683	0.43009
2728	223864	Rapgef3	10411	2.682037673	0.00388
2729	14375	Xrcc6	2547	0.004090774	0.99989
2730	28075	Desi1	27351	-0.156065062	0.8923
2731	29858	Pmm1	5372	-0.432994272	0.95917
2732	56233	Hdac7	51564	-0.20044782	0.91487
2733	78929	Polr3h	171568	0.138569366	0.99988
2734	11429	Aco2	50	-0.03577111	0.99988
2735	22337	Vdr	7421	-0.322279021	0.99988
2736	105855	Nckap1l		2.825096217	0.00388
2737	21935	Tnfrsf17	608	1.225840733	0.01101
2738	106200	Txndc11	51061	-0.3925591	0.52176
2739	56722	Litaf	9516	-0.44813984	0.53643
2740	26425	Nubp1	4682	0.481121163	0.29838
2741	12265	Ciita	4261	1.322355116	0.00817
2742	13731	Emp2	2013	-0.127775831	0.8864
2743	69053	810013L24Ri	29035	0.89965493	0.01482
2744	12053	Bcl6	604	0.492072765	0.88929
2745	12737	Cldn1	9076	0.01845744	0.93776
2746	16180	Il1rap	3556	-0.214442405	0.99988
2747	72615	Anks3	124401	0.396387452	0.66292

2748	66911	Nudt16l1	84309	0.502986374	0.30954
2749	17237	Mgrn1	23295	0.541324783	0.11448
2750	12914	Crebbp	1387	-0.520117551	0.31663
2751	71591	Zfp251	90987	-0.398810463	0.4271
2752	15205	Hes1	3280	-0.168412555	0.60394
2753	74120	Zfp263	10127	-0.759913484	0.31233
2754	224088	Atp13a3	79572	-0.360720258	0.548
2755	54483	Mefv	4210	0.554610939	0.46908
2756	74022	Glyr1	84656	-0.244287002	0.68189
2757	224090	Tmem44	93109	-0.479677781	0.6316
2758	224092	Lsg1	55341	-0.144867681	0.99988
2759	66049	Rogdi	79641	-0.646211277	0.53572
2760	70511	Eef2kmt		-0.001001698	0.99988
2761	50505	Ercc4	2072	0.014470266	0.99988
2762	76282	Gpt	2875	0.413693538	0.65789
2763	11815	Apod	347	2.64337552	0.00664
2764	268822	Adck5	203054	0.551959232	0.44695
2765	66445	Cyc1	1537	-0.036721788	0.98154
2766	106025	Sharpin	81858	-0.429255323	0.57884
2767	68877	Maf1	84232	0.146169288	0.99053
2768	59053	Hgh1	51236	-1.160587699	0.22364
2769	13350	Dgat1	8694	0.20127788	0.99988
2770	15499	Hsf1	3297	-0.241488941	0.99988
2771	12181	Bop1	23246	0.337718304	0.99988
2772	223658	Mroh1	727957	-0.202328843	0.6147
2773	30840	Fbxl6	26233	0.381879994	0.40517
2774	52710	Slc52a2		0.904682416	0.33039
2775	14731	Gpaa1	8733	-0.790853757	0.32473
2776	75475	Oplah	26873	0.880834977	0.0894
2777	66168	Grina	2907	-0.147867645	0.69261
2778	18810	Plec	5339	-0.635310862	0.37342
2779	105782	Scrib	23513	-0.221848262	0.6037
2780	22122	Gfus	7264	-0.96181831	0.26955
2781	69146	Gsdmd	79792	0.865117441	0.74574
2782	68453	Gpihbp1	338328	-0.05059533	0.99656
2783	100041546	Ly6c2		1.628193938	0.00837
2784	17069	Ly6e	4061	-1.087772218	0.25011
2785	23936	Lynx1	66004	0.086083512	0.95986
2786	72373	Psca	8000	1.752203711	0.15546
2787	271377	Zbtb11	27107	0.682890906	0.04757
2788	74201	Cep97	79598	0.577622417	0.02788
2789	14083	Ptk2	5747	0.296161691	0.99053
2790	29857	Mapk12	6300	-1.161034905	0.34762
2791	105847	Lmf2	91289	-0.3009344	0.59226
2792	12651	Chkb		0.14215956	0.86848
2793	11883	Arsa	410	-0.064980066	0.99988
2794	68708	Rabl2		0.127721347	0.99988
2795	16564	Kif21a	55605	-0.967875992	0.37599
2796	67057	Yaf2	10138	-0.203498943	0.98124
2797	67197	Zcrb1	85437	-0.489582632	0.27194

2798	11658	Alcam	214	-0.100692597	0.95917
2799	208650	Cblb	868	0.17549332	0.86115
2800		Dubr		0.885366391	0.23011
2801	70508	Bbx	56987	-1.007743809	0.2744
2802	58998	Nectin3	25945	-1.292880678	0.23706
2803	84544	Cd96	10225		0.991
2804	17470	Cd200	4345	2.046184295	0.08501
2805	67841	Atg3	64422	0.499996756	0.17322
2806	74102	Slc35a5	55032	0.224043025	0.70822
2807	67896	Ccdc80	151887	0.414439166	0.5185
2808	66067	Gtpbp8	29083	0.437932035	0.28293
2809	72083	Mzt2		0.76249354	0.01704
2810	19090	Prkdc	5591	0.691448955	0.02229
2811	17217	Mcm4	4173	0.94037462	0.00846
2812	70620	Ube2v2	7336	0.581701049	0.01366
2813	20583	Snai2	6591	-0.684812043	0.58636
2814	66086	Cep20	123811	0.1859544	0.40878
2815	67203	Nde1	54820	0.182463991	0.8023
2816	93734	Mpv17l		-0.294623488	0.75527
2817	94184	Pdxdc1	23042	-0.056829104	0.94351
2818	18203	Ntan1	123803	0.112583501	0.75419
2819	106298	Rrn3	54700	-0.690115136	0.50535
2820	67118	Bfar	51283	0.536063488	0.02315
2821	74108	Parn	5073	1.192601291	0.06971
2822	117606	Boc	91653	0.876922518	0.23067
2823	320007	Sidt1	54847	-0.718036379	0.43426
2824	72117	Naa50	80218	0.857893013	0.22923
2825	212153	Ccdc191	57577	-0.408624847	0.38269
2826	15260	Hira	7290	0.724692935	0.69608
2827	106248	Qtrt2	79691	-0.347821145	0.81534
2828	18100	Mrpl40	64976	0.130027628	0.49302
2829	74185	Gbe1	2632	-0.100330925	0.79766
2830	56490	Zbtb20	26137	-0.902056641	0.17131
2831	252870	Usp7	7874	-0.589088807	0.37299
2832	54128	Pmm2	5373	-1.016447522	0.25415
2833	94223	Dgcr8		-0.799695461	0.31713
2834	15547	Trmt2a	27037	-0.115396903	0.78869
2835	56297	Arl6	84100	0.343187773	0.62665
2836	224273	Crybg3	131544	0.438065763	0.85932
2837	67014	Riox2	84864	0.006580513	0.99988
2838	12892	Cpox		0.707205283	0.02224
2839	224250	Cldnd1	56650	0.329660316	0.99988
2840	54613	St3gal6	10402	1.31783065	0.00843
2841	66497	Cmss1	84319	0.4049174	0.9188
2842	67581	Tbc1d23	55773	-0.301889153	0.61797
2843	224023	Klhl22	84861	0.355665625	0.72801
2844	52633	Nit2	56954	0.245289425	0.86008
2845	28185	Tomm70a	9868	0.472728299	0.61372
2846	56277	Tmem45a		0.316895167	0.98071
2847	224022	Slc7a4	6545	-0.780622715	0.29227

2848	21787	Tfg	10342	0.126608962	0.66466
2849	18440	P2rx6	9127	-0.110407839	0.99988
2850	69009	Thap7	80764	-0.975469944	0.26342
2851	66863	Lztr1	8216	0.057765172	0.80953
2852	72168	Aifm3	150209	-0.858529485	0.45457
2853	67474	Snap29	9342	0.979561475	0.01234
2854	64136	Sdf2l1	23753	0.359081433	0.68938
2855	13383	Dlg1	1739	-0.085998411	0.93453
2856	66053	Ppil2	23759	-0.235137328	0.78236
2857	320213	Senp5	205564	-1.066781594	0.20079
2858	106369	Ypel1	29799	-0.27756255	0.91088
2859	68092	Ncbp2	22916	-0.013321813	0.99988
2860	21976	Top3b	8940	-0.731905685	0.47831
2861	224105	Pak2	5062	0.102148924	0.85752
2862	68980	Wdr53	348793	-0.17792644	0.99223
2863	224014	Fgd4	121512	0.933511208	0.00922
2864	74006	Dnm1l	10059	-0.889157872	0.24824
2865	207683	Igsf11	152404	0.508423226	0.3722
2866	51789	Tnk2	10188	-0.295648667	0.65248
2867	70120	Yars2	51067	0.354225747	0.14324
2868	56375	B4galt4	8702	0.678228746	0.23686
2869	22042	Tfrc	7037	-1.167288158	0.28745
2870	12549	Arhgap31	57514	0.621767217	0.28235
2871	69823	Fyttd1	84248	-0.832538454	0.25176
2872	70144	Lrch3	84859	0.160428491	0.99988
2873	239833	Lmln	89782	0.039065213	0.89111
2874	64082	Popdc2	64091	0.609685912	0.42228
2875	106326	Osbpl11	114885	0.149019965	0.85413
2876	69150	Snx4	8723	0.474037938	0.31925
2877	22661	Zfp148	7707	-0.452497068	0.43674
2878	56637	Gsk3b	2932	-1.213939721	0.15099
2879	22247	Umps	7372	0.508896058	0.25359
2880	14314	Fstl1	11167	0.771481862	0.02882
2881	16419	Itgb5	3693	-0.634402169	0.60204
2882	68194	Ndufb4	4710	0.680041847	0.01837
2883	27416	Abcc5	10057	1.022746082	0.00933
2884	67657	Rabl3	285282	-0.330644752	0.91311
2885	74197	Gtf2e1	2960	0.955501363	0.01098
2886	15163	Hcls1	3059	1.853973463	0.00561
2887	239839	Ccdc14	64770	0.248300288	0.74801
2888	107589	Mylk	4638	3.827170214	0.00356
2889	320299	Iqcb1	9657	0.488128171	0.87223
2890	106389	Eaf2	55840	1.596089463	0.21585
2891	224129	Adcy5	111	0.546876059	0.75395
2892	11773	Ap2m1	1173	0.853717068	0.21581
2893	12724	Clcn2	1181	1.723895917	0.00926
2894	72599	Pdia5	10954	0.952621625	0.01649
2895	224132	Slc49a4	84925	0.224120091	0.35849
2896	66667	Hspbap1	79663	-0.410462919	0.76768
2897	75826	Senp2		-0.429459373	0.54315

2898	66664	Tmem41a		-1.129088773	0.22128
2899	20462	Tra2b	6434	-0.216158287	0.68432
2900	246048	Chodl	140578	-1.134175809	0.23077
2901	12228	Btg3	10950	0.429681384	0.33768
2902	67102	D16Ertd472e	54149	-0.082888673	0.99988
2903	13052	Cxadr		-0.578674814	0.46908
2904	30940	Usp25	29761	-0.550154299	0.68497
2905	59083	Fetub	26998	-0.809518297	0.31851
2906	67742	Samsn1	64092	0.26661648	0.17434
2907	106344	Rfc4	5984	-1.368620997	0.15686
2908	13682	Eif4a2	1974	0.442291653	0.06476
2909	20440	St6gal1	6480	1.356661514	0.10655
2910	17174	Masp1	5648	0.437081645	0.92014
2911	27393	Mrpl39	54148	0.215782372	0.99053
2912	11957	Atp5j	522	0.381640557	0.28629
2913	11820	App	351	0.610829008	0.0205
2914	11504	Adamts1	9510	0.49384833	0.60882
2915	23794	Adamts5	11096	0.350240263	0.96072
2916	23872	Ets2	2114	-0.372306091	0.74793
2917	13548	Dyrk1a	1859	0.125546302	0.99988
2918	13185	Vps26c	10311	0.196188574	0.76675
2919	57738	Slc15a2	6565	-0.038650887	0.99988
2920	106347	Ildr1	286676	-0.095536784	0.81985
2921	12524	Cd86	942	1.886773114	0.00972
2922	16646	Kpna1	3836	-0.333067641	0.56261
2923	80285	Parp9	83666	-0.427678009	0.51657
2924	68146	Arl13b	200894	-0.713348441	0.37928
2925	19128	Pros1	5627	1.377981574	0.0287
2926	56088	Psmg1	8624	0.039935552	0.99988
2927	93871	Brwd1	54014	-1.043032766	0.24007
2928	52793	Fam3b	54097	1.176893366	0.09868
2929	56176	Pigp	51227	0.615884013	0.18247
2930	170765	Ripply3	53820	1.344374017	0.00817
2931	110749	Chaf1b	8208	-1.32014088	0.20915
2932	70028	Dop1b	9980	0.70879212	0.0209
2933	109857	Cbr3	874	0.985710867	0.19632
2934	224440	Setd4	54093	0.817215024	0.29735
2935	209195	Clic6	54102	0.797995238	0.19626
2936	54720	Rcan1	1827	0.183813905	0.99988
2937	12394	Runx1	861	-0.316674832	0.59996
2938	28080	Atp5o		0.43066584	0.60729
2939	16443	Itsn1	6453	0.551204209	0.29554
2940	60364	Donson		-0.227670953	0.77825
2941	20658	Son	6651	-0.307547627	0.57088
2942	14450	Gart	2618	-0.07421601	0.96075
2943	77975	Tmem50b	757	-0.001819173	0.99988
2944	15980	Ifngr2	3460	0.311500794	0.48074
2945	15975	Ifnar1	3454	-0.024884815	0.99988
2946	16155	Il10rb		0.430197917	0.30766
2947	15976	Ifnar2	3455	-0.138917159	0.9383

2948	68001	Cfap298		-0.779921967	0.28598
2949	104015	Synj1	8867	0.411589005	0.1198
2950	67367	Paxbp1	94104	-1.182617764	0.17289
2951	66578	Mis18a	54069	0.960316958	0.01117
2952	20655	Sod1	6647	0.375096892	0.0436
2953	224432	Scaf4	57466	-0.987251829	0.25731
2954	69612	Kansl2	54934	0.010881117	0.99988
2955	11512	Adcy6	112	1.169311886	0.14962
2956	13800	Enah	55740	-1.08600434	0.19222
2957	22410	Wnt10b	7480	0.04186276	0.76462
2958	74775	Lmbr1l	55716	-0.641017327	0.35657
2959	22143	Tuba1b	10376	0.973259467	0.01009
2960	54614	Prpf40b	25766	-0.552711537	0.40581
2961	22379	Fmnl3	91010	-0.035035465	0.99988
2962	110213	Tmbim6	7009	-0.103369478	0.86374
2963	26934	Racgap1	29127	-0.053384212	0.99988
2964	83797	Smarcd1	6602	-0.289531983	0.68613
2965	14555	Gpd1	2819	-0.1134115	0.97945
2966	66379	Cox14	84987	0.237489062	0.15529
2967	71949	Cers5	91012	-0.402819076	0.63927
2968	65970	Lima1	51474	-0.678836101	0.22535
2969	207214	Larp4	113251	-0.726059278	0.28584
2970	239667	Dip2b	57609	-0.568099484	0.57676
2971	11908	Atf1	466	-0.100770344	0.99988
2972	18174	Slc11a2	4891	-0.233082032	0.81665
2973	109901	Cela1	1990	-0.10054939	0.99988
2974	59033	Slc4a8	9498	0.902307014	0.17976
2975	15370	Nr4a1	3164	1.765556639	0.00621
2976	93707	Pcdhgc4	56098	-0.000933043	0.99988
2977	110310	Krt7	3855	-0.19908254	0.87783
2978	16668	Krt18	3875	-0.647553271	0.32623
2979	246277	Csad	51380	-0.583705803	0.4026
2980	16012	Igfbp6	3489	1.293784553	0.02897
2981	66151	Prr13		0.505614328	0.23676
2982	26404	Map3k12		-0.233191769	0.74046
2983	21357	Tarbp2		-0.568251381	0.40386
2984	54615	Npff		0.70830803	0.71914
2985	67488	Calcoco1		0.706784185	0.63034
2986	246102	Rttn	25914	-0.874049616	0.33823
2987	12575	Cdkn1a	1026	-0.023020648	0.99988
2988	52014	Nus1	116150	0.746478995	0.01538
2989	72140	Cep89	84902	0.266861269	0.66832
2990	70380	Mospd1	56180	1.049747003	0.01333
2991	68050	Akirin1	79647	0.434490899	0.03382
2992	55985	Cxcl13	10563	1.620240862	0.22887
2993	57080	Gtf2ird1	9569	-0.828857631	0.24531
2994	74485	Lrrc71	149499	0.293391465	0.64026
2995	12457	Noct	25819	0.195980896	0.72306
2996	17250	Abcc1	4363	0.078161859	0.99988
2997	68202	Ndufa5	4698	0.436704534	0.04096

2998	14199	Fhl1		1.617925807	0.08172
2999	76467	Msrb2	22921	0.090064524	0.89195
3000	19718	Rfc2	5982	0.336136555	0.40589
3001	68184	Denr	8562	-0.295910876	0.61803
3002	27374	Prmt5	10419	-0.230673612	0.91672
3003	69083	Sult1c2	6819	3.556621752	0.00356
3004	14938	Gzma	3001	2.135079716	0.00544
3005	27426	Nagpa	51172	0.346187738	0.63869
3006	71446	Get1		0.29574362	0.7316
3007	117198	Ivns1abp	10625	0.537662855	0.4602
3008	69671	Tmem52	339456	0.543805256	0.57408
3009	67053	Rpp14	109703458	0.2985138	0.58169
3010	105727	Slc38a1	81539	-0.316417998	0.72329
3011	56310	Gps2	2874	-0.411722723	0.60288
3012	12215	Bsg	682	0.149052577	0.991
3013	67776	Vwa5a		0.751719724	0.01733
3014	14789	P3h3	10536	0.953114615	0.15816
3015	16169	Il15ra	3601	0.763971093	0.32867
3016	230779	Serinc2	347735	-0.607756035	0.40182
3017	20300	Ccl25		-0.815306314	0.40066
3018	16529	Kcnk5	8645	0.730784877	0.25181
3019	235587	Parp3	10039	0.148252368	0.73147
3020	109652	Acy1		-0.148842706	0.98477
3021	77432	530002B09Rik		-4.125814683	0.02278
3022	76737	Creld2	79174	0.282852157	0.74448
3023	23999	Twf2	11344	0.53820037	0.01876
3024	675812	Zfp605	100289635	-0.231541996	0.92315
3025	140499	Ube2j2	118424	-0.209606693	0.75914
3026	69104	Marchf5	54708	-0.328926307	0.67216
3027	69185	Dtwd1	56986	0.250226915	0.99988
3028	67866	Wfdc1	58189		0.40878
3029	17858	Mx2		0.151490961	0.99988
3030	70235	Poc1a	25886	-0.599688413	0.62211
3031	22051	Trip6	7205	0.159676458	0.9719
3032	56620	Clec4n	93978	3.505534322	0.00356
3033	213990	Agap3	116988	0.230120704	0.80924
3034	66058	Tmem176a	55365	-0.905649905	0.29203
3035	228993	Slc17a9	63910	-0.035838924	0.94687
3036	73181	Nfatc4	4776	0.857160194	0.53388
3037	320951	Pisd		-0.142823649	0.99988
3038	21991	Tpi1	7167	-0.835087555	0.26018
3039	19328	Rab12	201475	-0.739812916	0.41058
3040	59092	Pcbp4	57060	-0.634617231	0.33125
3041	14793	Cdca3	83461	-1.103364855	0.26054
3042	67389	C1qtnf12	388581	0.783343613	0.06595
3043	17151	Ccndbp1	23582	0.230653387	0.73063
3044	66627	Ogfod2	79676	-0.299394305	0.59187
3045	64656	Mrps23	51649	-0.132217028	0.9979
3046	69159	Rhebl1	121268	0.880612107	0.05099
3047	78887	Sfi1	9814	-0.517180766	0.49171

3048	72084	Pigx	54965	0.823832749	0.03487
3049	24001	Tiam2	26230	1.155526751	0.01197
3050	20975	Synj2	8871	-0.779834232	0.24053
3051	100037282	Rsph3b		-1.165549904	0.10059
3052	20112	Rps6ka2	6196	-0.313053159	0.31233
3053	50873	Prkn	5071	0.632542759	0.24492
3054	68262	Agpat4	56895	0.861779711	0.04992
3055	20517	Slc22a1	6580	-1.550011536	0.3727
3056	16004	Igf2r	3482	0.12849145	0.99988
3057	110460	Acat2		-0.724481346	0.3392
3058	240028	Lnpep	4012	0.359810432	0.16959
3059	12648	Chd1	1105	-0.233437295	0.99988
3060	55951	Mpc1	51660	0.147819798	0.99988
3061	23984	Pde10a	10846	1.061284929	0.39846
3062	72057	Phf10	55274	0.767466408	0.02462
3063	21826	Thbs2	7058	0.105598627	0.99988
3064	64074	Smoc2	64094	1.245250349	0.01017
3065	22709	Zfp51		0.250515302	0.991
3066	353502	Hcfc1r1	54985	-0.116723592	0.99988
3067	27279	Tnfrsf12a		-1.38996497	0.16121
3068	76498	Paqr4	124222	-0.058550036	0.99988
3069	76917	Flywch2	114984	1.415402455	0.0089
3070	74011	Slc25a27	9481	-0.988908682	0.27473
3071	27226	Pla2g7	7941	0.700201593	0.0287
3072	94185	Tnfrsf21	27242	-0.436846248	0.75663
3073	78249	Adgrf4	221393	-0.91884574	0.27873
3074	83815	Cenpq	55166	0.383772539	0.2434
3075	17850	Mmut	4594	0.010901413	0.99988
3076	72238	Tbc1d5	9779	-0.001175117	0.99988
3077	20230	Satb1	6304	0.417715051	0.6932
3078	71702	Cdc5l		-0.296596539	0.46701
3079	224805	Aars2	57505	-0.473463961	0.54694
3080	68463	Mrpl14	64928	0.299406294	0.55288
3081	72415	Sgo1	151648	-0.73384737	0.50521
3082	63959	Slc29a1	2030	-0.350191096	0.93548
3083	15516	Hsp90ab1	3326	-0.771819258	0.2905
3084	18037	Nfkbie	4794	0.08572122	0.99988
3085	21645	Tcte1	202500		0.991
3086	22339	Vegfa	7422	-0.518793992	0.56261
3087	56055	Gtpbp2	54676	-0.135850877	0.73975
3088	80905	Polh	5429	0.846146729	0.01545
3089	224796	Clic5	53405	0.9085992	0.15771
3090	83965	Enpp5	59084	-0.311767197	0.69783
3091	224794	Enpp4	22875	0.710498762	0.42735
3092	56050	Cyp39a1	51302	1.784605743	0.00504
3093	50758	Fbxl17	64839	-0.467717953	0.74269
3094	75564	Rsph9	221421	0.045402028	0.99691
3095	68565	Mrps18a	55168	-0.499721348	0.53259
3096	224823	Rrp36	88745	-0.390958665	0.69388
3097	71461	Ptk7	5754	0.62744323	0.48651

3098	72029	Cnpy3	10695	0.149424673	0.4315
3099	224826	Ubr2	23304	-0.457642034	0.38269
3100	63856	Taf8	129685	-0.007615091	0.99988
3101	14913	Guca1a	2978	-1.00991517	0.21195
3102	53414	Bysl	705	-0.820964716	0.3364
3103	21425	Tfeb	7942	-0.339419744	0.99988
3104	74123	Foxp4	116113	-1.801612128	0.0652
3105	83433	Trem2	54209	-0.10841476	0.991
3106	18044	Nfya	4800	0.027535984	0.99988
3107	57261	Brd4	23476	-0.729906552	0.32523
3108	106504	Stk38	11329	-0.278359709	0.82101
3109	68816	Ppil1	51645	0.588680793	0.33038
3110	74116	Pi16	221476	0.46608186	0.34373
3111	56462	Mtch1	23787	-0.060276163	0.99988
3112	26382	Fgd2	221472	1.75849124	0.01378
3113	18712	Pim1	5292	0.93982025	0.00837
3114	68597	Ccdc167	154467	0.184298509	0.99988
3115	74157	Cmtr1	23070	-1.151279785	0.22927
3116	109801	Glo1	2739	-0.151469932	0.89844
3117	11307	Abcg1	9619	-0.239546962	0.88959
3118	22092	Rsph1	89765	-0.489166846	0.95511
3119	224674	Slc37a1	54020	0.244243804	0.74269
3120	57773	Wdr4	10785	-0.103284488	0.94627
3121	78330	Ndufv3	4731	-0.210809601	0.99988
3122	12411	Cbs		-1.652766018	0.16844
3123	17691	Sik1		1.086185569	0.17917
3124	268970	Arhgap28	79822	-0.157296446	0.99988
3125	13823	Epb41l3	23136	-0.281827994	0.9565
3126	56399	Akap8	10270	-0.443585956	0.35873
3127	67268	Myl12a	10627	-0.085987499	0.92858
3128	17929	Myom1	8736	1.090202213	0.0123
3129	22404	Wiz	58525	-0.268817224	0.60462
3130	64898	Lpin2	9663	-1.726764057	0.1333
3131	246707	Emilin2	84034	1.187055301	0.03684
3132	74355	Smchd1	23347	0.08721308	0.86725
3133	170716	Cyp4f13		0.002430311	0.99989
3134	67052	Ndc80	10403	-0.298366201	0.64319
3135	78785	Clip4	79745	-0.284579756	0.81989
3136	77889	Lbh	81606	0.200284893	0.62525
3137	71685	Galnt14	79623	-0.242582714	0.99988
3138	57440	Ehd3	30845	0.381020347	0.84859
3139	22436	Xdh	7498	0.057610845	0.98477
3140	66310	Dpy30	84661	-0.341075572	0.60462
3141	50850	Spast	6683	0.360520231	0.96168
3142	210148	Slc30a6	55676	0.38264689	0.63624
3143	75292	Prkd3	23683	0.535397547	0.24007
3144	67864	Yipf4	84272	1.047854631	0.00734
3145	12211	Birc6	57448	0.107755875	0.99988
3146	50766	Crim1	51232	0.024861381	0.99988
3147	74199	Vit	5212	0.74791155	0.84777

3148	268980	Strn	6801	-0.009031114	0.9979
3149	74196	Ttc27	55622	-0.286292921	0.60288
3150	19106	Eif2ak2	5610	0.850925114	0.01578
3151	12607	Cebpz	10153	-0.710477171	0.24307
3152	73694	Ndufaf7	55471	-0.106094044	0.98653
3153	224938	Pja2	9867	0.175237711	0.65156
3154	70536	Qpct	25797	-2.324669021	0.09142
3155	17158	Man2a1	4124	0.435111705	0.03115
3156	13078	Cyp1b1	1545	0.92031617	0.77348
3157	30960	Vapa	9218	0.284597026	0.58233
3158	72692	Hnrnpll	92906	0.093931623	0.99988
3159	19765	Ralbp1	10928	-0.486740901	0.6037
3160	225027	Srsf7	6432	0.630449449	0.02191
3161	65960	Twsg1	57045	0.559315126	0.02862
3162	72900	Ndufv2	4729	0.078020464	0.87991
3163	68767	Washc1		0.444589795	0.02957
3164	28006	Washc2		-0.224818847	0.68595
3165	72016	Tedc2	80178	0.378580951	0.90285
3166	72416	Lrpprc	10128	0.882905448	0.58308
3167	11984	Atp6v0c	527	-0.142933636	0.86369
3168	18607	Pdpk1	5170	-1.16812146	0.12775
3169	213760	Prepl	9581	-0.300060031	0.64493
3170	20234	Sbp		-5.581597085	0.01715
3171	27410	Abca3	21	-0.956628052	0.26961
3172	13177	Eci1	1632	0.746725687	0.02062
3173	78586	Srbd1	55133	1.320407577	0.01051
3174	66705	Dnase1l2	1775	0.099923622	0.89785
3175	13560	E4f1	1877	-1.018395143	0.26606
3176	13819	Epas1	2034	0.658284822	0.02476
3177	56716	Mlst8	64223	0.559908539	0.26928
3178	104215	Rhoq	23433	0.128511354	0.54196
3179	18701	Pigf	5281	-1.425724876	0.03115
3180	56724	Cript	9419	-0.225011301	0.65963
3181	193813	Mcfd2	90411	0.705023308	0.30811
3182	17685	Msh2	4436	0.389423363	0.04556
3183	14651	Hagh	3029	0.622244152	0.18855
3184	79043	Spsb3	90864	0.2371675	0.99847
3185	30957	Mapk8ip3	23162	-0.835450637	0.56492
3186	12266	C3	718	1.296326021	0.00814
3187	52009	Jpt2	90861	-0.151459576	0.96923
3188	407831	Tmem204	79652	0.973973691	0.03252
3189	106633	Ift140	9742	0.854058226	0.02352
3190	71718	Telo2	9894	-0.610504492	0.52451
3191	72836	Pot1b		0.330813092	0.80638
3192	56520	Nme4	4833	-0.621992415	0.4602
3193	60455	Pgap6	58986	0.949148032	0.19789
3194	68611	Mrpl28	10573	-0.210520281	0.85155
3195	12005	Axin1	8312	0.425683746	0.44461
3196	106581	Fam234a	83986	0.293333371	0.99816
3197	66978	Luc7l	55692	-1.186681108	0.19351

3198	19252	Dusp1	1843	1.319691204	0.11183
3199	224630	Bnip1	662	-0.359322096	0.64889
3200	67675	Cuta	51596	-0.411395891	0.86915
3201	66905	Plin3	10226	-0.933366551	0.19626
3202	193796	Kdm4b	23030	-0.358898493	0.75914
3203	67267	Uqcc2	84300	-0.054575831	0.99988
3204	64144	Mllt1	4298	-0.554281897	0.47897
3205	56409	Nudt3	11165	-0.035291868	0.99988
3206	30051	Spdef	25803	-0.867479035	0.3224
3207	20630	Snrpc	6631	0.745966151	0.03351
3208	68776	Taf11		-0.233670258	0.88714
3209	224650	Anks1	23294	0.470189559	0.4679
3210	224656	Zfp523	7629	-1.138291352	0.30446
3211	14229	Fkbp5	2289	0.133345281	0.74755
3212	67993	Nudt12	83594	0.402343302	0.62665
3213	71745	Cul2	8453	-0.403075611	0.5804
3214	68010	Bambi	25805	0.057911256	0.99988
3215	67440	Mtpap	55149	0.083044838	0.78729
3216	26410	Map3k8	1326	-0.234104113	0.99988
3217	225115	Svil	6840	0.200276776	0.90774
3218	21417	Zeb1	6935	0.253915691	0.99988
3219	13831	Epc1	80314	0.522190047	0.01545
3220	20662	Sos1	6654	-0.210630405	0.95961
3221	225028	Map4k3	8491	0.973975988	0.12182
3222	106522	Pkdcc	91461	0.82548974	0.03115
3223	20463	Cox7a2l	9167	0.287325229	0.59437
3224	240174	Thada	63892	-1.227936651	0.3155
3225	213575	Dync2li1	51626	0.348560963	0.68262
3226	67453	Slc25a46	91137	0.419327651	0.86711
3227	269003	Sap130	79595	-0.072582095	0.99988
3228	108013	Celf4	56853	-1.046786221	0.26012
3229	66648	Tpgs2	25941	1.417071712	0.00447
3230	106957	Slc39a6	25800	-0.448858069	0.4244
3231	58523	Elp2	55250	-0.162638066	0.58461
3232	68046	700062C07R	83608	-0.537038589	0.43816
3233	69256	Zfp397	84307	-0.179978823	0.88231
3234	212307	Mapre2	10982	-0.219011942	0.66764
3235	225131	Wac	51322	-0.327895912	0.48743
3236	67974	Ccny	219771	-0.327968376	0.85685
3237	225160	Thoc1	9984	-0.62259412	0.56635
3238	19877	Rock1	6093	-0.150694205	0.82257
3239	77805	Esco1	114799	-0.321931989	0.69388
3240	225164	Mib1	57534	-0.390428966	0.66999
3241	208292	Zfp871		0.008296344	0.99988
3242	224697	Adamts10	81794	0.775979508	0.02476
3243	17916	Myo1f	4542	2.922259689	0.00407
3244	16580	Kifc5b		-0.426813009	0.94092
3245	13527	Dtna	1837	-1.444477024	0.15728
3246	12558	Cdh2	1000	-0.053952861	0.99988
3247	21356	Tapbp	6892	-0.73585165	0.42761

3248	14976	Pfdn6	10471	0.07004397	0.99988
3249	57315	Wdr46	9277	-0.910348768	0.09868
3250	56515	Rnf138		-0.226858044	0.60454
3251	224705	Vps52	6293	-0.291092962	0.62421
3252	19763	Ring1	6015	-0.485063811	0.89032
3253	14977	Slc39a7	7922	-0.830369002	0.29276
3254	13506	Dsc2		-0.576863524	0.40525
3255	14312	Brd2	6046	-0.317914437	0.57219
3256	16913	Psmb8	5696	0.123967495	0.99988
3257	21355	Tap2		0.078470379	0.89774
3258	67199	Pfdn1	5201	-0.320432583	0.80116
3259	72512	Sting1	340061	-0.509261498	0.38641
3260	76594	Dnajc18	202052	-0.587058616	0.52331
3261	71242	Spata24		-1.456122738	0.19425
3262	69816	Mzb1	51237	1.674207545	0.00888
3263	81500	Sil1	64374	-0.684159727	0.72035
3264	15526	Hspa9	3313	-0.299624975	0.59649
3265	225363	Etf1	2107	-0.617521229	0.38631
3266	27632	Nelfe	7936	0.2323013	0.46469
3267	12263	C2	717	1.119952452	0.03461
3268	13824	Epb41l4a	64097	-0.223119193	0.91036
3269	170459	Stard4	134429	-2.076912456	0.12595
3270	53603	Tslp	85480	0.662676564	0.37464
3271	30948	Bin1	274	-0.681141631	0.34681
3272	13872	Ercc3	2071	-0.327095631	0.96358
3273	26405	Map3k2	10746	0.116231648	0.99988
3274	73473	Iws1	55677	-0.472934728	0.41843
3275	13001	Csnk2b		-0.798301825	0.29783
3276	224727	Bag6	7917	-0.702381066	0.35773
3277	53761	Prrc2a	7916	-1.681468044	0.14438
3278	225341	Lims2	55679	0.940474366	0.76836
3279	11629	Aif1	199	1.694548389	0.00952
3280	16994	Ltb	4050	0.758474211	0.02372
3281	74320	Wdr33	55339	0.03456471	0.99988
3282	66237	Atp6v1g2	534	0.268280368	0.72098
3283	66878	Riok3	8780	-0.255210137	0.75839
3284	76482	Rmc1	29919	0.39769257	0.53617
3285	18145	Npc1	4864	-0.073054632	0.991
3286	94064	Mrpl27	51264	0.446010638	0.65731
3287	225207	Zfp521	25925	1.211504951	0.12036
3288	16774	Lama3	3909	0.294462	0.99672
3289	69192	Dhx16	8449	-0.381715981	0.87409
3290	16210	Impact	55364	0.344821684	0.58979
3291	72747	Ttc39c	125488	-0.783784212	0.41105
3292	65113	Ndfip1	80762	0.364144861	0.3152
3293	73242	Atat1	79969	0.782460591	0.49727
3294	24066	Spry4	81848	0.907559916	0.31855
3295	14670	Gnl1	2794	0.267021296	0.75251
3296	71132	Cabyr	26256	-0.7144441	0.48479
3297	14815	Nr3c1	2908	0.05175949	0.93478

3298	66973	Mrps18b	28973	-0.694957632	0.32067
3299	66839	Dele1	9812	-0.522364889	0.76504
3300	67676	Rpp21	79897	-0.255452324	0.80792
3301	106952	Arap3	64411	1.215399338	0.0692
3302	15183	Hdac3	8841	-0.138965167	0.88372
3303	13367	Diaph1	1729	-0.173290935	0.81989
3304	22670	Trim26	7726	0.021881552	0.99988
3305	54393	Gabbr1	2550	-1.642060706	0.14308
3306	70640	Dcp2	167227	-0.589591215	0.39772
3307	24010	Ik	3550	0.43588969	0.03295
3308	225467	Pggt1b	5229	-0.466698351	0.30954
3309	105853	Mal2	114569	-0.756144907	0.27473
3310	11777	Ap3s1	1176	-0.115047509	0.83265
3311	108857	Ankhd1		-0.901042532	0.20621
3312	15200	Hbegf	1839	-0.100867107	0.99988
3313	67180	Yipf5	81555	-0.357576831	0.50187
3314	225432	Rbm27		-0.858910616	0.313
3315	107045	Lars	51520	-0.204446345	0.58461
3316	56070	Tcerg1	10915	-0.469109617	0.43154
3317	22240	Dpysl3	1809	0.338124257	0.9704
3318	20730	Spink1	6690	-3.426880309	0.10999
3319	68857	Dtwd2	285605	0.423173684	0.53627
3320	15488	Hsd17b4	3295	0.113008916	0.99988
3321	80718	Rab27b	5874	-0.372776886	0.58933
3322	17191	Mbd2	8932	-0.224927943	0.8433
3323	17128	Smad4	4089	-1.908742218	0.03618
3324	66286	Sec11c	90701	0.660076247	0.32983
3325	58801	Pmaip1	5366	1.406886088	0.00832
3326	14680	Gnal	2774	-0.650533662	0.50735
3327	114663	Impa2	3613	-0.315390992	0.6985
3328	69597	Afg3l2	10939	-0.261328403	0.98675
3329	67222	Srfbp1	153443	-0.161881515	0.71131
3330	16948	Lox	4015	0.165253258	0.45453
3331	68166	Spire1	56907	-0.0075699	0.99988
3332	67847	Sncaip	9627	-0.561517186	0.83875
3333	69226	Snx24	28966	-1.439785287	0.19812
3334	107047	Psmg2	56984	0.554208079	0.02064
3335	19038	Ppic	5480	0.439203542	0.91586
3336	19255	Ptpn2	5771	0.05432577	0.99988
3337	70799	Cep192	55125	-0.458254346	0.59205
3338	52662	Ldlrad4	753	0.844142261	0.20213
3339	240427	Setbp1	26040	-0.234008776	0.70077
3340	107029	Me2	4200	0.900136553	0.19384
3341	74322	Cxxc1	30827	0.080545028	0.99988
3342	17190	Mbd1	4152	-0.769825623	0.42534
3343	17126	Smad2	4087	0.32280107	0.05383
3344	20689	Sall3	27164	-0.80647312	0.28834
3345	50771	Atp9b	374868	-0.548129734	0.39013
3346	68731	Rbfa	79863	-0.43384479	0.41179
3347	27366	Gm16286		-0.742597682	0.37341

3348	93687	Csnk1a1	1452	-0.62696874	0.26955
3349	240334	Pcyox1l	78991		0.991
3350	17714	Grpel2	134266	-0.334621951	0.73046
3351	108123	Napg	8774	-0.43431087	0.55766
3352	53382	Txnl1	9352	0.217828445	0.99016
3353	70223	Nars	4677	-0.630675614	0.36204
3354	14151	Fech	2235	-0.013280708	0.99988
3355	83814	Nedd4l	23327	0.01113034	0.99988
3356	16906	Lmnb1	4001	-0.725495963	0.30506
3357	77422	330018D20R	401207	-0.217032868	0.99816
3358	73137	Prrc1	133619	0.034524896	0.99988
3359	20496	Slc12a2	6558	0.334362572	0.66885
3360	14119	Fbn2	2201	-0.551935006	0.35509
3361	66307	Isoc1	51015	0.434920007	0.30673
3362	67665	Dctn4	51164	-0.392995424	0.44829
3363	66810	Rbm22	55696	0.042166076	0.99988
3364	20044	Rps14	6208	-0.321066387	0.70567
3365	16149	Cd74	972	0.46730909	0.1077
3366	21453	Tcof1	6949	-0.478933901	0.34951
3367	67988	Tmx3	54495	0.231353278	0.38023
3368	18596	Pdgfrb	5159	0.840356953	0.18841
3369	12978	Csf1r	1436	0.728145653	0.0155
3370	14682	Gnaq		0.017735163	0.98477
3371	107272	Psat1	29968	0.456539038	0.40867
3372	21888	Tle4	7091	-0.64150385	0.38042
3373	66054	Cndp2	55748	0.31811858	0.95106
3374	67105	Timm21	29090	0.16622332	0.78093
3375	109672	Cyb5a	1528	0.439748606	0.33086
3376	66514	Asrgl1	80150	-1.424597074	0.13297
3377	16952	Anxa1	301	-0.429998994	0.86801
3378	16319	Incenp	3619	-0.729448389	0.44852
3379	14319	Fth1	2495	0.619000012	0.19344
3380	74760	Rab3il1	5866	1.209840876	0.1198
3381	60527	Fads3	3995	0.202649059	0.87025
3382	56473	Fads2	9415	0.88006098	0.01482
3383	72982	Tmem138	51524	-0.403882034	0.93851
3384	68642	Tmem216	51259	1.621499608	0.0066
3385	66072	Sdhaf2	54949	0.582374884	0.0461
3386	12511	Cd6	923	0.91126081	0.23085
3387	109225	Ms4a7	58475	1.601272706	0.0159
3388	12482	Ms4a1	931	2.211596397	0.06838
3389	64380	Ms4a4c		0.948205974	0.22496
3390	69774	Ms4a6b		1.581584136	0.00773
3391	66607	Ms4a4d		1.539852632	0.00674
3392	68774	Ms4a6d		2.136006746	0.00527
3393	94063	Mrpl16	54948	0.585397328	0.35194
3394	76303	Osbp	5007	-0.610895891	0.41122
3395	107373	Fam111a	63901	0.198581057	0.78102
3396	109910	Zfp91		-0.954636333	0.18905
3397	107321	Lpxn	9404	0.320732788	0.75164

3398	14675	Gna14	9630	-1.017347938	0.4432
3399	54391	Rfk	55312	-0.024838711	0.98675
3400	18552	Pcsk5	5125	-0.414295388	0.57219
3401	20409	Ostf1	26578	0.511840622	0.02757
3402	67383	Carnmt1	138199	0.187998515	0.99838
3403	108673	Ccdc86	79080	-1.384234936	0.14274
3404	28000	Prpf19	27339	-0.085570369	0.99988
3405	65221	Slc15a3	51296	-1.385532069	0.21995
3406	13194	Ddb1	1642	-0.152071428	0.81989
3407	14156	Fen1	2237	-0.569690702	0.33355
3408	54525	Syt7	9066	-0.719680485	0.55831
3409	26358	Aldh1a7		-0.85049328	0.40308
3410	22682	Zfand5	7763	0.220840649	0.3364
3411	83921	Cemip2	23670	0.139126246	0.99988
3412	20168	Rtn3	10313	-0.476503599	0.37597
3413	109168	Atl3	25923	-0.488633419	0.50723
3414	70999	Naa40	79829	-0.41545474	0.41147
3415	381236	Lipo3		1.422072411	0.00952
3416	107260	Otub1	55611	-0.711106563	0.48558
3417	240505	Cdc42bpg	55561	0.218022745	0.46836
3418	13660	Ehd1	10938	0.101800252	0.24196
3419	329015	Atg2a	23130	0.349607773	0.90772
3420	52024	Ankrd22	118932	-0.955681416	0.43637
3421	225849	Ppp2r5b	5526	0.736976597	0.63927
3422	14102	Fas	355	0.469686395	0.31915
3423	67072	Cdc37l1	55664	-0.572275315	0.30811
3424	16889	Lipa	3988	0.352240037	0.12279
3425	56248	Ak3	50808	-0.180033852	0.99947
3426	59028	Rcl1	10171	0.242163615	0.95106
3427	69024	Snx15	29907	-0.036304659	0.99988
3428	16452	Jak2	3717	0.52599542	0.01882
3429	66406	Sac3d1	29901	0.9589658	0.13768
3430	67849	Cdca5	113130	-0.701637957	0.53208
3431	81909	Zfpl1	7542	0.630470697	0.51198
3432	240641	Kif20b	9585	-2.326401358	0.02418
3433	68505	Vps51	738	0.359312875	0.05293
3434	73166	Tm7sf2	7108	-0.229635912	0.8575
3435	54364	Rpp30	10556	0.593010283	0.04621
3436	107765	Ankrd1	27063	-0.166758106	0.90174
3437	76073	Pcgf5	84333	-0.257995487	0.80933
3438	74126	Syvn1	84447	0.004412051	0.99988
3439	77125	Il33	90865	0.969579281	0.01098
3440	74493	Tnks2	80351	0.345772958	0.77929
3441	21873	Tjp2		-0.82989495	0.30728
3442	67457	Frmd8	83786	0.245549	0.70121
3443	109113	Uhrf2	115426	0.398361473	0.06352
3444	107375	Slc25a45	283130	1.63943614	0.11933
3445	19367	Rad9a	5883	0.476473188	0.72148
3446	19708	Dpf2	5977	0.08653986	0.83579
3447	104174	Gldc	2731	-0.608955225	0.71686

3448	353242	Mrpl21	219927	-0.172856975	0.92082
3449	58988	Rps6kb2	6199	0.53568716	0.37402
3450	20589	Ighmbp2	3508	-0.57621949	0.79951
3451	18969	Pola2		0.813883665	0.29504
3452	23789	Coro1b	57175	-0.310255231	0.67708
3453	69860	Eif1ad	84285	0.201140904	0.87446
3454	12660	Chka	1119	-1.288807574	0.13252
3455	23825	Banf1		-0.865300677	0.3406
3456	66990	Tmem134	80194	-0.137202055	0.78034
3457	11632	Aip	9049	0.524976803	0.07072
3458	18739	Pitpnm1	9600	-0.128386517	0.96441
3459	319322	Sf3b2	10992	0.018487381	0.99988
3460	69745	Pold4		0.551860376	0.24307
3461	107975	Pacs1	55690	-0.549546808	0.37859
3462	52004	Cdk2ap2	10263	0.408591555	0.65774
3463	110355	Grk2	156	0.418010716	0.28443
3464	71670	Acy3	91703	-0.243962824	0.88819
3465	18719	Pip5k1b	8395	-1.714793739	0.15963
3466		Gm49405		-0.771195146	0.32623
3467	76308	Rab1b	81876	-1.14006628	0.2445
3468	68090	Yif1a	10897	-0.55038733	0.37529
3469	226043	Cbwd1		-0.985912803	0.20476
3470	225870	Rin1	9610	-0.489896036	0.57798
3471	67689	Aldh3b1	221	1.869211608	0.00407
3472	54447	Asah2		1.106902064	0.01039
3473	19671	Rce1	9986	-0.166633979	0.99988
3474	13340	Slc29a2	3177	0.101605347	0.89833
3475	18563	Pcx	5091	-1.380616485	0.16844
3476	17330	Minpp1	9562	-0.828324651	0.35347
3477	319924	Apba1	320	0.535476091	0.55018
3478	23972	Papss2	9060	-0.775286133	0.32442
3479	12894	Cpt1a	1374	0.013975115	0.99988
3480	240518	Peli3	246330	-0.154172099	0.89867
3481	66419	Mrpl11	65003	0.112651421	0.99988
3482	71711	Mus81	80198	0.722309058	0.0189
3483	52036	Ppp6r3	55291	0.239164238	0.11415
3484	58859	Efemp2	30008	0.185883635	0.99988
3485	13041	Ctsw	1521	-0.586259112	0.40986
3486	58249	Fibp	9158	-0.65956587	0.44818
3487	66556	Drap1	10589	-0.237952231	0.76433
3488	67155	Smarca2	6595	-0.939295602	0.20706
3489	22359	Vldlr	7436	0.040597671	0.99988
3490	68209	Rnaseh2c	84153	0.085637758	0.99988
3491	19697	Rela	5970	0.247685738	0.69859
3492	114601	Ehbp1l1	254102	0.699241144	0.21861
3493	17826	Fam89b	23625	-0.015557647	0.99988
3494	16998	Ltbp3	4054	0.00155177	0.99988
3495	78891	Scyl1	57410	-0.943748595	0.17731
3496	12333	Capn1	823	0.070664153	0.99578
3497	226026	Smc5	23137	-0.133245946	0.89517

3498	56327	Arl2	402	-0.191924991	0.83709
3499	17283	Men1	4221	0.224799751	0.97133
3500	22668	Sf1	7536	-0.171536745	0.72319
3501	54683	Prdx5	25824	-0.053532152	0.93113
3502	26379	Esrra	2101	0.004770793	0.99988
3503	107173	Gpr137	56834	-0.465181454	0.53658
3504	12015	Bad	572	0.101251211	0.99988
3505	18797	Plcb3	5331	-0.346828048	0.58814
3506	22340	Vegfb	7423	-1.034813519	0.22685
3507	57431	Dnajc4	3338	0.564162131	0.91348
3508	108101	Fermt3	83706	0.66484901	0.02459
3509	20867	Stip1	10963	-0.295161939	0.61562
3510	104383	Rcor2	283248	-0.565392702	0.45341
3511	13728	Mark2	2011	-0.906431871	0.24826
3512	68229	Spindoc	144097	0.335463834	0.73076
3513	56072	Lgals12	85329	1.255749501	0.11415
3514	13006	Smc3	9126	-1.570559718	0.1567
3515	18569	Pdcd4	27250	-0.379462219	0.54781
3516	56392	Shoc2	8036	-0.731105173	0.33674
3517	14732	Gpam	57678	-0.635704136	0.25181
3518	433256	Acsl5	51703	-0.342798207	0.72965
3519	66980	Zdhhc6	64429	-0.174716541	0.95702
3520	53611	Vti1a	143187	-0.014552407	0.99988
3521	21416	Tcf7l2	6934	-0.248948014	0.50308
3522	15242	Hhex		0.451175159	0.72791
3523	74107	Cep55	55165	-0.671871862	0.54067
3524	13669	Eif3a	8661	-0.847062913	0.24003
3525	67894	Dennd10	404636	-0.711435219	0.33743
3526	11757	Prdx3	10935	-0.835368338	0.51121
3527	74055	Plce1	51196	-0.003230852	0.99988
3528	57753	Noc3l	64318	-0.257824497	0.99821
3529	15201	Hells	3070	-1.474131723	0.16284
3530	20411	Sorbs1	10580	1.102063279	0.00769
3531	56454	Aldh18a1	5832	-0.243935543	0.65408
3532	67590	Tctn3	26123	1.746561011	0.00777
3533	240665	Ccnj	54619	-0.704994734	0.42576
3534	107358	Tm9sf3	56889	0.338868975	0.30875
3535	83490	Pik3ap1	118788	-0.386992363	0.49408
3536	212391	Lcor	84458	-0.905718988	0.23922
3537	76479	Smndc1	10285	-0.542048017	0.38966
3538	17859	Mxi1	4601	-0.254072509	0.80673
3539	27360	Add3	120	0.201814742	0.88233
3540	170750	Xpnpep1	7511	-0.312792754	0.68524
3541	93679	Trim8	81603	-0.009228962	0.99988
3542	56350	Arl3	403	1.081055868	0.00934
3543	94279	Sfxn2	118980	0.480091149	0.89847
3544	17161	Maoa	4128	0.581214329	0.49634
3545	72018	Fundc1	139341	0.459143624	0.02776
3546	76952	Nt5c2	22978	0.319489497	0.77672
3547	20288	Msr1	4481	1.117635133	0.01357

3548	18572	Pdcd11	22984	-1.000765082	0.25175
3549	226182	Taf5	6877	-1.270830152	0.19922
3550	71041	Pcgf6	84108	-1.070953326	0.27564
3551	71398	Tasl	80231	1.210940607	0.15816
3552	14933	Gk		0.55170351	0.52566
3553	20874	Slk	9748	0.192281761	0.99988
3554	12821	Col17a1	1308	-1.774424767	0.11645
3555	67788	Sfr1	119392	0.277425639	0.04892
3556	14873	Gsto1	9446	0.028043122	0.99988
3557	226243	Habp2	3026	1.853109816	0.00439
3558	12369	Casp7	840	1.287852515	0.16807
3559	55947	Dclre1a	9937	0.48218777	0.77068
3560	66866	Nhlrc2	374354	-0.514038149	0.40878
3561	226251	Ablim1	3983	-0.016550416	0.99988
3562	72133	Trub1	142940	0.286970967	0.99816
3563	14585	Gfra1	2674	0.670812175	0.48698
3564	73442	Hspa12a	259217	0.840464176	0.01698
3565	214084	Slc18a2	6571	0.599941966	0.59323
3566	67290	110040N11R	123207	0.809847904	0.20743
3567	83962	Btbd1	53339	0.388970454	0.08583
3568	29877	Hdgfl3	50810	0.447884712	0.90216
3569	12173	Bnc1	646	1.497399585	0.00652
3570	59058	Bhlhe22	27319	0.643832625	0.23048
3571	18453	P4hb	5034	-0.264797768	0.54935
3572	192662	Arhgdia	396	-0.561025675	0.35326
3573	101861	Ints4	92105	0.124589589	0.8717
3574	21681	Alyref		0.372520345	0.84891
3575	66156	Anapc11	51529	0.71990821	0.00994
3576	68671	Pcyt2	5833	-1.7517707	0.12609
3577	209011	Sirt7	51547	-0.029217164	0.99988
3578	54473	Tollip	54472	0.451600139	0.29572
3579	209027	Pycr1	5831	-1.555869068	0.1497
3580	68938	Aspscr1	79058	-1.277067583	0.12922
3581	20892	Cenpx		0.084966516	0.99988
3582	217366	Lrrc45	201255	0.258554373	0.79144
3583	101513	Mob2	81532	-0.117288347	0.99988
3584	12409	Cbr2		0.56401674	0.05926
3585	94275	Maged1	9500	0.053016535	0.99988
3586	14104	Fasn	2194	-1.381348658	0.14041
3587	71085	Arhgap19		1.221950323	0.2375
3588	68730	Dus1l	64118	-0.00958692	0.99988
3589	209318	Gps1	2873	-0.144472635	0.89477
3590	74168	Zdhhc16	84287	-1.349124183	0.18633
3591	19719	Rfng	5986	-0.412979084	0.51528
3592	72199	Mms19	64210	-0.456509015	0.44263
3593	80879	Slc16a3	9123	1.294919267	0.18309
3594	104318	Csnk1d	1453	0.562705493	0.02635
3595	209588	Sectm1a		-1.23935297	0.02265
3596	66179	Ogfod3	79701	0.48170334	0.59534
3597	217371	Rab40b	10966	0.18987539	0.90775

3598	226122	Ubtd1	80019	-0.590649112	0.56158
3599	66840	Wdr45b	56270	-0.086978855	0.99988
3600	67432	Hoga1	112817	-1.333846281	0.20472
3601	84095	Pi4k2a	55361	0.088715202	0.99988
3602	52013	R3hcc1l	27291	0.105178734	0.83156
3603	192236	Hps1	3257	-0.727782608	0.4432
3604	14718	Got1	2805	1.134423275	0.09604
3605	93685	Entpd7	57089	0.885075764	0.0119
3606	66388	Cutc	51076	0.435746948	0.991
3607	71972	Dnmbp	23268	-0.259875878	0.79242
3608	226144	Erlin1	10613	-0.227996203	0.7316
3609	12675	Chuk	1147	-0.159836751	0.991
3610	72502	Cwf19l1	55280	-0.632287533	0.54744
3611	20250	Scd2		-0.613800308	0.35097
3612	67264	Ndufb8		-0.181110013	0.99988
3613	94067	Mrpl43	84545	0.058128766	0.99988
3614	226153	Twnk	56652	0.105444837	0.87783
3615	94280	Sfxn3	81855	0.128726643	0.73182
3616	107250	Kazald1	81621	0.79685754	0.04794
3617	12234	Btrc	8945	-0.257812184	0.8023
3618	56626	Poll	27343	-0.21308423	0.80808
3619	76055	Oga	10724	-0.508753037	0.42122
3620	16825	Ldb1	8861	-0.317014849	0.99988
3621	107338	Gbf1	8729	-1.284639351	0.19561
3622	18034	Nfkb2	4791	-0.911249004	0.58127
3623	54130	Actr1a	10121	0.181547654	0.86113
3624	24069	Sufu	51684	-0.841981239	0.54815
3625	15211	Hexa		0.385112231	0.02886
3626	23806	Arih1	25820	0.695156897	0.01889
3627	102774	Bbs4	585	-0.49149977	0.68757
3628	72141	Adpgk	83440	-0.960477239	0.22134
3629	67287	Parp6	56965	0.209383057	0.85226
3630	29806	Limd1	8994	0.448454639	0.03729
3631	83493	Sacm1l	22908	0.43618223	0.41683
3632	17281	Fyco1	79443	-1.289392943	0.14917
3633	22599	Slc6a20b		0.932410369	0.02461
3634	93730	Lztfl1	54585	0.697578431	0.02238
3635	21372	Tbl1x		-1.611716866	0.1583
3636	80892	Zfhx4	79776	0.534144609	0.75513
3637	66611	Ribc1	158787	0.357033791	0.64985
3638	15108	Hsd17b10	3028	-0.333894894	0.84891
3639	59026	Huwe1	10075	-0.764240435	0.2639
3640	69499	Tsr2	90121	-0.827633871	0.27863
3641	237107	Gnl3l	54552	0.183593806	0.99988
3642	80884	Maged2	10916	0.086996595	0.90383
3643	77622	Apex2	27301	-0.500775684	0.70623
3644	11656	Alas2	212	1.179229062	0.03715
3645	66082	Abhd6	57406	0.729740514	0.01627
3646	286940	Flnb	2317	0.293260425	0.05632
3647	218832	Polr3a	11128	0.459166793	0.08332

3648	20229	Sat1	6303	0.497680737	0.24104
3649	56360	Acot9		-0.090643377	0.87136
3650	53381	Prdx4	10549	0.328858923	0.08001
3651	20088	Rps24	6229	-0.243291476	0.77282
3652	19271	Ptprj	5795	-0.91460646	0.33674
3653	53325	Banp	54971	0.490162941	0.19512
3654	320910	Itgb8	3696	-0.125500564	0.99988
3655	20688	Sp4	6671	0.28529863	0.9519
3656	11982	Atp10a	57194	1.021594678	0.01209
3657	22215	Ube3a	7337	-0.337395165	0.46499
3658	18599	Padi1	29943	-0.979437939	0.40595
3659	18602	Padi4	23569	-0.270249335	0.99988
3660	20591	Kdm5c	8242	-0.705813352	0.54076
3661	66711	Sbds	51119	0.189677213	0.6899
3662	56715	Rabgef1		-0.128902468	0.92561
3663	16404	Itga7		0.694357714	0.01444
3664	12512	Cd63		0.247015382	0.06987
3665	14561	Gdf11		-0.69643176	0.20605
3666	66844	Ormdl2		0.254767535	0.94955
3667	74330	Dnajc14		-0.033650631	0.99988
3668	58223	Mmp19		1.047132661	0.19048
3669	13139	Dgka		-0.073627086	0.9861
3670	12566	Cdk2		-0.081375357	0.88844
3671	20431	Pmel		0.180270673	0.99988
3672	27370	Rps26		-0.36574943	0.59826
3673	18813	Pa2g4		-0.954425393	0.25638
3674	23943	Esyt1		0.354515141	0.64824
3675	68094	Smarcc2		-0.812702483	0.24121
3676	208092	Chmp6	79643	-0.16141415	0.97675
3677	108100	Baiap2	10458	-1.109876957	0.22364
3678	67588	Rnf41		0.84505688	0.19517
3679	69917	Nabp2		-1.363471801	0.19935
3680	78777	Tepsin	146705	-0.212593584	0.75897
3681	56530	Cnpy2		0.620026841	0.42397
3682	71885	Faap100	80233	0.401367859	0.2058
3683	11947	Atp5b	506	0.298782452	0.37598
3684	19075	Prim1		0.189926503	0.99988
3685	17937	Nab2	4665	0.731903284	0.01992
3686	108037	Shmt2	6472	0.439028852	0.61794
3687	71750	R3hdm2	22864	-0.035173765	0.99988
3688	14632	Gli1	2735	-0.451772361	0.0678
3689	13198	Ddit3	1649	0.24263398	0.991
3690	110962	Mbd6	114785	-0.492422776	0.44852
3691	69654	Dctn2	10540	-0.500592051	0.40568
3692	72354	Ttc4	7268	0.947141541	0.03176
3693	117150	Pip4k2c	79837	0.130766289	0.88226
3694	76987	Hdhd2	84064	0.030053009	0.99988
3695	17344	Pias2	9063	0.04937152	0.99988
3696	11946	Atp5a1	498	0.252710145	0.05332
3697	68876	Atp23	91419	0.384870588	0.27522

3698	170822	Usp33	23032	-1.979556173	0.12745
3699	12729	Clns1a	1207	-0.141792944	0.99988
3700	218693	Paip1	10605	-0.203034265	0.99988
3701	18115	Nnt		0.197481679	0.45367
3702	244233	Scart1		0.087655553	0.99988
3703	212503	Paox		0.671072277	0.28449
3704	93747	Echs1	1892	-0.807522229	0.27562
3705	69064	Fuom	282969	-0.497318492	0.93623
3706	69752	Zfp511	118472	0.808521524	0.23338
3707	11501	Adam8	101	-0.5280815	0.75682
3708	74237	Tubgcp2	10844	-0.174996138	0.80163
3709	52389	Adgra1	84435	0.395217597	0.0454
3710	212111	Inpp5a	3632	-0.027876913	0.99988
3711	13106	Cyp2e1	1571	-1.342385222	0.59912
3712	76974	Urah		0.191757444	0.99988
3713	54399	Bet1l		-0.565342922	0.28547
3714	101489	Ric8a	60626	-0.621694523	0.49074
3715	64384	Sirt3	23410	-0.210157497	0.83655
3716	23997	Psmd13	5719	-0.413350347	0.63381
3717	68713	Ifitm1		0.846357773	0.28834
3718	66141	Ifitm3		-0.104494234	0.94169
3719	24058	Sigirr	59307	0.554737626	0.07298
3720	27388	Ptdss2	81490	-0.178375021	0.99988
3721	54123	Irf7	3665	-0.023114149	0.99988
3722	15461	Hras	3265	-0.591268071	0.38474
3723	21351	Taldo1	6888	-0.656743723	0.37887
3724	98845	Eps8l2	64787	-0.23655509	0.75663
3725	71448	Tmem80	283232	0.797958128	0.15353
3726	67186	Rplp2		0.13739761	0.88929
3727	66853	Pnpla2	57104	-0.331890404	0.56527
3728	12476	Cd151	977	-0.153038366	0.99988
3729	64540	Tspan4	7106	0.356321953	0.59505
3730	68038	Chid1	66005	0.643870505	0.01886
3731	73067	Tmem192	201931	-0.102536488	0.82255
3732	68117	Apool	139322	-0.640769863	0.36217
3733	12662	Chm	1121	-0.191118831	0.71211
3734	12909	Crcp	27297	0.417613384	0.32582
3735	109900	Asl		-0.801585395	0.2699
3736	110006	Gusb	2990	-0.078701143	0.98561
3737	67902	Sumf2	25870	0.475393069	0.991
3738	68059	Tm9sf2	9375	0.641284671	0.01985
3739	69634	Clybl	171425	-1.489436395	0.12128
3740	223254	Farp1	10160	-0.109658676	0.99988
3741	105445	Dock9	23348	0.135452683	0.99988
3742	217351	Tnrc6c	57690	0.14534448	0.85752
3743	217353	Tmc6	11322	-0.275377509	0.6489
3744	21877	Tk1	7083	-0.434900232	0.60595
3745	76025	Cant1	124583	0.456684907	0.47867
3746	52897	Rbfox3	146713	-0.435613054	0.59593
3747	12416	Cbx2	84733	-0.698307706	0.33077

3748	30951	Cbx8	57332	-0.157855896	0.99988
3749	14387	Gaa	2548	0.127907323	0.85956
3750	192170	Eif4a3		-0.014731375	0.99988
3751	74370	Rptor	57521	-0.293453399	0.64106
3752	18584	Pde8a	5151	1.311572387	0.22876
3753	12877	Cpeb1		-0.03948547	0.99988
3754	66282	Tma16		0.203730798	0.72329
3755	93837	Dach2	117154	-0.999344804	0.32194
3756	80902	Zfp202	7753	-1.078710249	0.22111
3757	54160	Copg2	26958	-1.804011164	0.15062
3758	27205	Podxl	5420	0.191913006	0.47104
3759	27418	Mkln1	4289	0.20654302	0.52159
3760	12013	Bach1	571	-0.194946787	0.88226
3761	12469	Cct8	10694	-0.024814976	0.99988
3762	74112	Usp16	10600	-0.502976441	0.41395
3763	70998	Phf6	84295	0.230948721	0.63076
3764	15452	Hprt	3251	-0.231102226	0.96229
3765	66658	Ccdc51	79714	0.750869181	0.02064
3766	235610	Atrip	84126	0.291562086	0.9542
3767	66940	Shisa5	51246	0.385457239	0.63451
3768	270198	Pfkfb4	5210	1.936496993	0.00621
3769	12836	Col7a1		-1.244923931	0.40878
3770	22273	Uqcrc1	7384	-0.153004721	0.99988
3771	236915	Arhgef9	23229	0.016689961	0.99988
3772	245684	Cnksr2	22866	1.006999329	0.13841
3773	192216	Tmem47	83604	0.423173684	0.47885
3774	11689	Alox5	240	1.203049942	0.09562
3775	71779	Marchf8	220972	-0.025782788	0.96966
3776	71968	Wdr73	84942	-0.71063345	0.27917
3777	68039	Nmb	4828	-0.370695691	0.70784
3778	56529	Sec11a	23478	0.324890962	0.086
3779	14755	Pigq	9091	-0.330968511	0.92858
3780	224624	Rab40c	57799	0.225244481	0.92973
3781	68347	Mettl26	84326	-0.334305655	0.75283
3782	68241	Mcrip2	84331	0.138458617	0.99988
3783	214952	Rhot2		0.769195096	0.38999
3784	72106	Jmjd8	339123	-0.087748323	0.98645
3785	268933	Wdr24	84219	0.025867821	0.99988
3786	110639	Prps2	5634	-0.288327879	0.73316
3787	20970	Sdc3	9672	1.594549093	0.00577
3788	97212	Hadha	3030	-0.493697863	0.40836
3789	22171	Tyms	7298	-1.284230015	0.17173
3790	18415	Hspa4l	22824	-0.198482331	0.9979
3791	20873	Plk4	10733	0.217823921	0.68421
3792	72175	Mfsd8	256471	-0.206338451	0.74567
3793	214048	Larp1b	55132	0.15799432	0.99988
3794	269424	Jade1	79960	-0.231527829	0.57493
3795	73852	D3Ertd751e	132321	0.401201819	0.04314
3796	83691	Crispld1	83690	-0.230683199	0.98331
3797	17087	Ly96	23643	0.698918319	0.37529

3798	209378	Itih5	80760	1.006783624	0.40878
3799	11949	Atp5c1	509	-0.41089183	0.57454
3800	209361	Taf3	83860	-0.65956169	0.33713
3801	4930412O13Rik			-0.084096093	0.99988
3802	21922	Clec3b	7123	1.416170066	0.58605
3803	66446	Exosc7	23016	-0.011530326	0.99988
3804	69035	Zdhhc3	51304	-0.22173787	0.96516
3805	331046	Tgm4	7047	-0.026067266	0.99988
3806	108116	Slco3a1	28232	0.098103645	0.99821
3807	72157	Pgm1	5236	0.184076531	0.99988
3808	27376	Slc25a10		-0.541073091	0.49296
3809	15239	Hgs	9146	0.042502821	0.98506
3810	67115	Rpl14		0.039220458	0.99988
3811	192678	Rassf3	283349	-0.340833536	0.54647
3812	12768	Ccr1		2.01102176	0.00544
3813	16412	Itgb1	3688	-0.198306219	0.84669
3814	18186	Nrp1	8829	-1.027431083	0.23085
3815	93742	Pard3	56288	-0.565064047	0.40418
3816	26557	Homer2	9455	-1.018333655	0.22111
3817	209692	Dhtkd1	55526	0.922796768	0.11594
3818	57743	Sec61a2	55176	-1.329840874	0.24371
3819	53893	Nudt5	11164	-0.058514069	0.89195
3820	101095	Zfp282	8427	-0.203604545	0.91027
3821	12304	Pdia4	9601	-0.468469768	0.91426
3822	66383	Iscu		0.421674565	0.95893
3823	80752	Fam20c	56975	0.914885971	0.37246
3824	18590	Pdgfa	5154	-0.541849621	0.40878
3825	67604	Get4	51608	-0.081043742	0.87562
3826	11798	Xiap	331	-0.578068039	0.49634
3827	20843	Stag2	10735	0.006045348	0.99988
3828	67044	Higd2a		0.064716701	0.99988
3829	28126	Nop16	51491	0.761025506	0.00837
3830	56795	Arl10	285598	-1.14562251	0.28329
3831	97820	833439L19Ri	57179	0.00928828	0.99988
3832	73666	Thoc3	84321	-0.475126832	0.62567
3833	76577	Faf2	23197	-0.395917305	0.41672
3834	74257	Tspan17	26262	0.100857874	0.99988
3835	107448	Unc5a	90249	0.798743213	0.21413
3836	20184	Uimc1	51720	-0.166894049	0.82473
3837	17131	Smad7	4092	-0.138959547	0.86293
3838	17919	Myo5b		-0.063954297	0.95106
3839	12364	Casp12	100506742	0.493589626	0.238
3840	12362	Casp1	834	0.241624409	0.71973
3841	69149	Kbtbd3	143879	0.804080198	0.01132
3842	67618	Aasdhppt	60496	0.152382087	0.99988
3843	244672	Cwf19l2	143884	0.296980397	0.55663
3844	67667	Alkbh8	91801	0.361805488	0.47417
3845	20671	Sox17	64321	-0.092009812	0.99988
3846	18777	Lypla1	10434	0.000345653	0.99989
3847	12421	Rb1cc1	9821	0.039550115	0.99988

3848	71096	Sntg1	54212	1.460071168	0.00832
3849	76187	Adhfe1		0.556387624	0.6516
3850	170755	Sgk3		0.90646534	0.01196
3851	26754	Cops5	10987	-0.019419441	0.99988
3852	29819	Stau2	27067	0.264187349	0.39037
3853	98711	Rdh10	157506	1.038968938	0.00775
3854	21749	Terf1	7013	0.543284397	0.01649
3855	74229	Paqr8	85315	-0.92650282	0.35332
3856	14048	Eya1	2138	-0.527798396	0.41537
3857	75712	Tmem14a	28978	0.269043521	0.97856
3858	14859	Gsta3	221357	1.259016113	0.00544
3859	72265	Tram1	23471	0.001346684	0.99988
3860	212442	Lactb2	51110	0.132157197	0.65122
3861	66799	Ube2w		0.648850363	0.04022
3862	70397	Tmem70	54968	0.993560895	0.00812
3863	18711	Pikfyve	200576	0.446264001	0.97242
3864	15926	Idh1	3417	0.584241743	0.22876
3865	67099	Mettl21a	151194	-0.16377048	0.99988
3866	12912	Creb1	1385	0.133531531	0.991
3867	93691	Klf7	8609	0.252828762	0.66686
3868	75619	Fastkd2	22868	0.263419733	0.53699
3869	23792	Adam23	8745	0.621642897	0.08576
3870	55949	Eef1b2	1933	0.15192795	0.99303
3871	227197	Ndufs1	4719	0.667834821	0.03164
3872	18187	Nrp2	8828	1.046732279	0.01491
3873	68115	Maip1	79568	-0.571348096	0.27482
3874	74013	Rftn2	130132	0.579476449	0.60755
3875	19070	Mob4		0.143972417	0.81477
3876	15510	Hspd1	3329	-0.503532273	0.32303
3877	67876	Coq10b	80219	0.588691298	0.52678
3878	81898	Sf3b1	23451	-0.177424492	0.76704
3879	227059	Slc39a10	57181	0.495963652	0.45457
3880	53945	Slc40a1	30061	0.578714683	0.01535
3881	73674	Wdr75	84128	0.770008699	0.019
3882	22779	Ikzf2		-1.773167284	0.05886
3883	14768	Lancl1		0.759309836	0.78385
3884	11363	Acadl	33	-0.257793301	0.96953
3885	68691	Kansl1l	151050	0.038538222	0.99988
3886	66646	Rpe		-0.175476734	0.83509
3887	54167	Icos	29851		0.40878
3888	77300	Raph1	65059	-0.372685424	0.43721
3889	241066	Carf	79800	-0.012380942	0.99988
3890	57750	Wdr12	55759	-0.465908444	0.65312
3891	55989	Nop58	51602	-0.134873081	0.85639
3892	22218	Sumo1		-0.321360973	0.49074
3893	74018	Als2	57679	0.29155156	0.17899
3894	227154	Stradb	55437	-0.320438162	0.87985
3895	70827	Trak2	66008	-0.176073419	0.90626
3896	12370	Casp8	841	0.050756869	0.99988
3897	12633	Cflar	8837	0.140767982	0.99988

3898	66495	Ndufb3	4709	1.089665154	0.00952
3899	12747	Clk1	1195	-0.805051043	0.29188
3900	70225	Ppil3	53938	-0.205403104	0.94734
3901	65102	Nif3l1	60491	0.469716548	0.33309
3902	18393	Orc2	4999	0.468225351	0.53394
3903	12832	Col5a2	1290	1.142774461	0.00902
3904	12825	Col3a1	1281	0.635619721	0.22972
3905	72050	Poglut2	79070	0.635549936	0.54027
3906	22592	Ercc5		-0.563070131	0.38199
3907	75623	Tex30	93081	0.777099054	0.24865
3908	78896	Ecrg4	84417	0.186494667	0.99988
3909	226999	Slc9a2	6549	-2.013528215	0.06119
3910	16174	Il18rap	8807	0.617182288	0.98217
3911	17082	Il1rl1	9173	0.547158052	0.62004
3912	16182	Il18r1	8809	1.65778932	0.00576
3913	16177	Il1r1	3554	0.124023548	0.99988
3914	16178	Il1r2	7850	0.492997045	0.70642
3915	26921	Map4k4	9448	-0.575515201	0.59187
3916	18143	Npas2	4862	-0.548456702	0.85705
3917	68833	Pdcl3	79031	0.2431133	0.56421
3918	56210	Rev1	51455	-0.949039904	0.77971
3919	226982	Eif5b	9669	-0.650493654	0.2827
3920	107734	Mrpl30		-0.418786987	0.40878
3921	69028	Mitd1	129531	-0.118971083	0.94214
3922	72097	Cracdl	343990	0.424728784	0.82658
3923	98267	Stk17b	9262	0.524563318	0.02776
3924	70396	Asnsd1		0.815027519	0.01372
3925	227102	Ormdl1		-0.841963004	0.34641
3926	227099	Pms1	5378	0.046100891	0.79627
3927	16329	Inpp1	3628	-1.586590266	0.17817
3928	14660	Gls	2744	0.305265874	0.99197
3929	20846	Stat1	6772	0.118181981	0.94943
3930	109019	Nabp1	64859	0.396323513	0.0277
3931	56363	Tmeff2	23671	-0.831417214	0.57999
3932	269181	Mgat4a	11320	-0.148779375	0.991
3933	67387	Unc50	25972	1.102212324	0.00918
3934	76178	Coa5	493753	0.19608786	0.44852
3935	269180	Inpp4a	3631	0.718130934	0.80565
3936	56030	Tmem131	23505	0.243005709	0.43515
3937	22637	Zap70	7535	2.349850926	0.00439
3938	20353	Sema4c	54910	0.724980956	0.0147
3939	226971	Plekhb2	55041	0.048198283	0.99988
3940	19253	Ptpn18	26469	0.327442714	0.04886
3941	27993	Imp4	92856	0.498536917	0.02851
3942	13518	Dst	667	-0.721796561	0.49408
3943	19076	Prim2	5558	-0.065876566	0.99988
3944	77264	Zfp142	7701	0.183351047	0.59585
3945	76867	Rhbdd1	84236	-0.427549443	0.43956
3946	75734	Mff	56947	0.422549548	0.27177
3947	68187	Fam135a	57579	-0.543540753	0.40878

3948	68002	Sdhaf4	135154	0.523337021	0.37029
3949	98366	Smap1	60682	0.425994384	0.04242
3950	280645	B3gat2	135152	-0.82895242	0.53658
3951	70155	Ogfrl1	79627	-0.256680457	0.66875
3952	15463	Agfg1	3267	0.110661496	0.99988
3953	77629	Sphkap	80309	0.370756579	0.69275
3954	20297	Ccl20	6364	-1.802625381	0.11843
3955	104086	Cyp27a1	1593	0.9428633	0.22364
3956	57751	Rnf25	64320	-0.171257759	0.7398
3957	66821	Bcs1l	617	0.511331389	0.57084
3958	18802	Plcd4	84812	0.849394376	0.23135
3959	58184	Cnot9	9125	0.298111301	0.97186
3960	227292	Ctdsp1	58190	-0.508877317	0.44925
3961	18173	Slc11a1	6556	1.816050228	0.00952
3962	56695	Pnkd	25953	-0.020838341	0.99988
3963	12765	Cxcr2	3579	0.911405112	0.24826
3964	68606	Ppm1f	9647	1.069817636	0.01172
3965	16011	Igfbp5	3488	-0.058082907	0.99988
3966	22596	Xrcc5	7520	0.160993979	0.45457
3967	108147	Atic	471	0.851788041	0.15929
3968	14268	Fn1	2335	0.009265772	0.99988
3969	68818	Zfand2b	130617	0.494200822	0.03316
3970	74104	Abcb6	10058	0.126056206	0.99988
3971	52231	Ankzf1	55139	-0.006959375	0.99988
3972	74577	Glb1l	79411	0.264487724	0.41466
3973	20872	Stk16	8576	-0.198139539	0.74969
3974	22145	Tuba4a	7277	-0.390355395	0.50153
3975	56812	Dnajb2	3300	-0.601347505	0.56605
3976	22626	Slc23a3	151295		0.991
3977	11790	Speg	10290	0.368539513	0.95309
3978	13346	Des	1674	1.956307411	0.00664
3979	13437	Dnpep	23549	0.351752409	0.03177
3980	98733	Obsl1	23363	-0.053952861	0.99988
3981	71728	Stk11ip	114790	0.37101431	0.45745
3982	14897	Trip12	9320	-0.244584695	0.67674
3983	20684	Sp100	11262	0.282614233	0.33692
3984	64294	Itm2c	81618	-0.268446178	0.97675
3985	70247	Psmd1	5707	-0.878947238	0.18422
3986	17975	Ncl	4691	-0.502521303	0.28043
3987	13838	Epha4	2043	-0.385973955	0.62133
3988	19231	Ptma		-0.321700781	0.75897
3989	18582	Pde6d	5147	1.203972588	0.38014
3990	26895	Cops7b	64708	-0.511503986	0.44859
3991	23874	Farsb	10056	-0.079422067	0.99988
3992	69163	Mrpl44	65080	-0.001605559	0.99988
3993	20720	Serpine2	5270	0.904129242	0.29273
3994	26987	Eif4e2	9470	-0.011375206	0.99988
3995	98363	Efhd1	80303	0.618402027	0.9519
3996	53972	Ngef	25791	0.791134552	0.28588
3997	67273	Ndufa10	4705	-0.649347897	0.33861

3998	108657	Rnpepl1	57140	0.056374371	0.99988
3999	23830	Capn10	11132	1.241111377	0.00858
4000	64095	Gpr35	2859		0.991
4001	69821	Mterf4	130916	0.051466354	0.99988
4002	269224	Pask	23178	-0.141373372	0.99988
4003	66385	Ppp1r7		-0.168618555	0.87991
4004	18000	Septin2		-0.739319994	0.42622
4005	59041	Stk25	10494	-0.415597408	0.61871
4006	51800	Bok	666	0.172485379	0.99988
4007	67026	Thap4	51078	-0.205324689	0.97968
4008	66615	Atg4b	23192	-0.331549182	0.59463
4009	21915	Dtymk	1841	0.076950053	0.98452
4010	66262	Ing5	84289	0.075730261	0.88928
4011	18566	Pdcd1	5133	1.419099845	0.00908
4012	16331	Inpp5d	3635	1.437839782	0.00406
4013	77040	Atg16l1	55054	-0.423618004	0.58979
4014	171531	Mlph	79083	-1.228558499	0.24006
4015	19329	Rab17	64284	-0.288697593	0.75231
4016	16978	Lrrfip1	9208	-0.73809448	0.31728
4017	50880	Scly	51540	-0.397137675	0.43721
4018	67444	Ilkap	80895	-0.451773068	0.47417
4019	65247	Asb1	51665	1.344374017	0.00721
4020	208727	Hdac4	9759	0.187740175	0.66875
4021	20725	Serpinb8	5271	0.689688637	0.0214
4022	26889	Cln8	2055	0.094272135	0.99988
4023	227446	Relch	57614	-0.571768579	0.54027
4024	21934	Tnfrsf11a	8792	0.872022666	0.03343
4025	66957	Serpinb11	89778	0.016224128	0.99988
4026	252876	Gin1	54826	0.182390143	0.91311
4027	18484	Pam	5066	0.38727129	0.29737
4028	70829	Ccdc93	54520	-0.127586282	0.99988
4029	74117	Actr3	10096	0.316171506	0.36244
4030	74150	Slc35f5	80255	0.292640673	0.35903
4031	71111	Gpr39	2863	0.593111336	0.56215
4032	72949	Ccnt2	905	0.167313238	0.85142
4033	67812	Ubxn4	23190	-0.703956623	0.20757
4034	17219	Mcm6	4175	0.38534883	0.32982
4035	226414	Dars	1615	0.064611683	0.90772
4036	64214	Rgs18	64407	1.098948553	0.01073
4037	50778	Rgs1	5996	1.266013236	0.01196
4038	19735	Rgs2	5997	-0.172407144	0.99988
4039	214498	Cdc73	79577	0.239152826	0.91239
4040	12628	Cfh		0.537101606	0.64998
4041	22099	Tsn	7247	-0.27166864	0.59205
4042	67949	Nifk	84365	0.058430202	0.99988
4043	81879	Tfcp2l1	29842	0.121421726	0.8332
4044	226352	Epb41l5	57669	-0.343329831	0.73852
4045	19258	Ptpn4	5775	-1.070695101	0.32332
4046	13167	Dbi	1622	0.557064982	0.01357
4047	73103	110009E18Rik		1.700434605	0.00533

4048	68428	Steap3	55240	0.449372619	0.77644
4049	59125	Nek7	140609	-0.719839593	0.2987
4050	19264	Ptprc	5788	1.572864085	0.01571
4051	13136	Cd55		0.672698316	0.34531
4052	67997	Ddx59	83479	0.092127805	0.95306
4053	18640	Pfkfb2	5208	-0.536860677	0.86113
4054	66241	Tmem9	252839	-0.444829589	0.65767
4055	18772	Pkp1	5317	-1.582464017	0.14688
4056	21956	Tnnt2	7139	-0.838769653	0.68231
4057	18703	Pigr	5284	-0.862674391	0.24506
4058	13007	Csrp1	1465	0.181661286	0.99988
4059	14270	Srgap2		0.983300649	0.06236
4060	68724	Arl8a	127829	0.884121567	0.01263
4061	16865	Eif2d	1939	-1.507672817	0.1115
4062	67196	Ube2t	29089	-0.135251124	0.99988
4063	54354	Rassf5	83593	0.649655427	0.46181
4064	226422	Rab29	8934	-0.173956043	0.99988
4065	98415	Nucks1	64710	-1.290174111	0.14414
4066	212980	Slc45a3	85414	-0.975506339	0.14962
4067	13714	Elk4	2005	0.171823776	0.99988
4068	18557	Cdk18	5129	0.684745403	0.40525
4069	213464	Rbbp5	5929	-0.086541088	0.99988
4070	269116	Nfasc	23114	1.035850967	0.20481
4071	16980	Lrrn2	10446	0.594950724	0.87534
4072	240752	Pik3c2b	5287	-0.399091837	0.47437
4073	240756	Klhl12	59349	-0.41359955	0.42182
4074	72017	Cyb5r1	51706	-0.215937208	0.74254
4075	72674	Adipor1	51094	-0.097356947	0.82085
4076	68507	Ppfia4	8497	0.750063784	0.02191
4077	381290	Atp2b4	493	1.280041746	0.01052
4078	208263	Tor1aip1	26092	0.0810534	0.97936
4079	19775	Xpr1	9213	-0.731956477	0.39172
4080	58244	Stx6	10228	-0.72805595	0.3364
4081	15064	Mr1	3140	-0.49101103	0.63555
4082	14645	Glul	2752	-0.219102237	0.69291
4083	19734	Rgs16	6004	1.234075575	0.0109
4084	226519	Lamc1	3915	-0.141419671	0.98197
4085	16782	Lamc2	3918	-1.386698932	0.13052
4086	17970	Ncf2	4688	2.009544315	0.00504
4087	19731	Rgl1	23179	0.72783042	0.03141
4088	63913	Niban1	116496	-0.331088095	0.68758
4089	19821	Rnf2	6045	0.269679073	0.99988
4090	67426	Coq8a	56997	0.848907454	0.01456
4091	226751	Cdc42bpa	8476	-0.593702236	0.38001
4092	226747	Ahctf1	25909	0.828902862	0.22804
4093	15278	Tfb2m	64216	-0.210397229	0.64827
4094	269152	Kif26b	55083	0.758227491	0.72801
4095	68226	Efcab2	84288	0.676773414	0.20213
4096	11545	Parp1	142	0.465660211	0.55466
4097	170760	Acbd3	64746	-0.348895668	0.8023

4098	66359	Cox20	116228	0.565233845	0.03556
4099	78825	Desi2	51029	0.046495825	0.95199
4100	76816	Sdccag8	10806	0.24645516	0.10438
4101	12334	Capn2	824	0.148077952	0.89987
4102	209456	Trp53bp2	7159	-0.925050719	0.43009
4103	27058	Srp9	6726	0.193335123	0.56386
4104	67459	Nvl	4931	-1.108917764	0.22535
4105	208795	Tmem63a	9725	-0.963375561	0.39146
4106	69051	Pycr2		0.226371688	0.40867
4107	14194	Fh1	2271	0.309377889	0.07197
4108	26388	Ifi202b		-0.00291605	0.99988
4109	381308	Ifi211		1.291525501	0.36964
4110	68440	Dusp23	54935	-1.640181996	0.16019
4111	71870	Cfap45	25790	-0.336918788	0.73777
4112	21346	Tagln2	8407	-0.436639805	0.54978
4113	98365	Slamf9	89886	1.546641182	0.01459
4114	12847	Copa	1314	-0.038789093	0.991
4115	98193	Dcaf8	50717	-0.446038181	0.40878
4116	93840	Vangl2	57216	-1.238665378	0.16441
4117	80914	Uck2	7371	-1.03001025	0.24307
4118	18986	Pou2f1	5451	-0.505188532	0.47429
4119	68481	Mpzl1	9019	-1.648574194	0.11022
4120	70456	Mpc2	25874	0.27508975	0.04605
4121	74106	Dcaf6	55827	-1.392406043	0.20351
4122	16963	Xcl1		0.251762408	0.13917
4123	56429	Dpt	1805	1.883514641	0.07247
4124	171567	Nme7	29922	-0.045343739	0.94285
4125	11931	Atp1b1	481	0.185924565	0.84891
4126	66352	Blzf1	8548	-1.251949792	0.17797
4127	74895	Ccdc181	57821	-0.069945529	0.99988
4128	14067	F5	2153	0.755030502	0.29038
4129	20344	Selp	6403	-0.35065025	0.78093
4130	20343	Sell	6402	1.756615438	0.00542
4131	240880	Scyl3	57147	0.231437912	0.99988
4132	16579	Kifap3	22920	0.073036065	0.99988
4133	18933	Prrx1	5396	0.815816905	0.24402
4134	89867	Sec16b	89866	0.379449277	0.991
4135	78255	Ralgps2	55103	-0.882708648	0.4382
4136	11352	Abl2	27	-0.129778893	0.80354
4137	20652	Soat1	6646	-0.384156544	0.46908
4138	226830	Smyd2	56950	-0.602876472	0.595
4139	19250	Ptpn14	5784	-0.017142541	0.99988
4140	108000	Cenpf	1063	-0.699900988	0.37529
4141	226823	Kctd3	51133	-0.433626126	0.57057
4142	26381	Esrrg	2104	-0.089878082	0.95552
4143	107508	Eprs	2058	-0.698659082	0.32222
4144	12902	Cr2	1380	-1.421789802	0.04723
4145	23827	Bpnt1	10380	1.440740476	0.19569
4146	381314	Iars2	55699	0.254063279	0.85388
4147	226778	Mark1	4139	-0.447646893	0.44813

4148	226856	Lpgat1	9926	-0.201939156	0.99988
4149	226849	Ppp2r5a	5525	0.848205365	0.01322
4150	11910	Atf3	467	-0.547811833	0.64511
4151	381319	Batf3	55509	1.017440384	0.01298
4152	68972	Tatdn3	128387	0.630980964	0.60462
4153	52477	Angel2	90806	0.533026954	0.28465
4154	22033	Traf5	7188	0.917441348	0.1165
4155	54139	Irf6	3664	-0.178281064	0.84786
4156	16780	Lamb3	3914	-0.264659042	0.66174
4157	18845	Plxna2	5362	0.142127251	0.94107
4158	22278	Usf1	7391	-0.74480148	0.51148
4159	18108	Nmt2	9397	-0.97824855	0.24222
4160	227525	Dclre1c	64421	0.31411053	0.56261
4161	75472	Cfap126	257177	-0.054521127	0.90879
4162	66540	Fam107b	83641	0.309750412	0.37475
4163	14130	Fcgr2b		2.588371167	0.00388
4164	209630	Frmd4a	55691	1.251814979	0.00914
4165	80915	Dusp12	11266	0.208039446	0.99988
4166	109079	Sephs1	22929	0.171319016	0.99024
4167	226641	Atf6	22926	-0.403942632	0.73177
4168	16922	Phyh	5264	0.157369676	0.92332
4169	16589	Uhmk1	127933	-0.041557196	0.99988
4170	70024	Mcm10	55388	-0.754739554	0.21676
4171	107652	Uap1	6675	0.313321283	0.99382
4172	71648	Optn	10133	-1.74831601	0.08581
4173	18214	Ddr2	4921	0.819962421	0.73757
4174	15490	Hsd17b7	51478	-1.085806399	0.22917
4175	74186	Ccdc3	83643	0.673074509	0.40828
4176	19737	Rgs5	8490	1.052829999	0.16681
4177	71233	Enkur	219670	0.935039404	0.03097
4178	66977	Nuf2	83540	-0.444488744	0.60705
4179	56752	Aldh9a1		0.010942646	0.99988
4180	66447	Mgst3	4259	0.096425363	0.99988
4181	14262	Fmo3	2328	0.467183221	0.7326
4182	226564	Fmo4	2329	-0.913420396	0.13938
4183	71449	Eef1aknmt	51603	-0.121832363	0.95012
4184	53330	Vamp4	8674	0.279359347	0.99905
4185	17926	Myoc	4653	2.152004557	0.07424
4186	67292	Pigc	5279	0.693513203	0.0216
4187	11758	Prdx6		-0.949485814	0.2905
4188	226541	Klhl20	27252	0.383172683	0.04501
4189	74455	Nsun6	221078	-0.10074285	0.84786
4190	70454	Cenpl	91687	0.32192031	0.55286
4191	226539	Dars2	55157	0.852190346	0.501
4192	17533	Mrc1	4360	2.556342012	0.00388
4193	11905	Serpinc1	462	0.668493394	0.29398
4194	20844	Stam	8027	-0.361131514	0.76772
4195	29809	Rabgap1l	9910	0.395488869	0.79436
4196	13434	Trdmt1	1787	1.376540375	0.00858
4197	20163	Rsu1	6251	-0.688808433	0.35792

4198	22352	Vim	7431	1.264578903	0.13241
4199	19212	Pter	9317	-0.829092701	0.35764
4200	18718	Pip4k2a	5305	1.520345979	0.00919
4201	12151	Bmi1	648	0.251389197	0.38572
4202	13418	Dnajc1	64215	-0.359017294	0.4432
4203	17354	Mllt10	8028	-1.311052939	0.325
4204	67448	Plxdc2	84898	0.857765288	0.40936
4205	59126	Nek6	10783	0.324512188	0.50862
4206	19177	Psmb7	5695	0.307650658	0.68674
4207	67857	Ppp6c	5537	-0.262388487	0.66812
4208	76899	Golga1	2800	-0.144904117	0.86053
4209	74192	Arpc5l	81873	0.399361235	0.15883
4210	26428	Orc4	5000	0.054338106	0.99988
4211	16574	Kif5c	3800	-0.173885227	0.94223
4212	109129	Mmadhc	27249	0.013213327	0.9462
4213	66960	Mindy3	80013	-0.06552271	0.99988
4214	241226	Itga8	8516	1.412965619	0.00785
4215	16184	Il2ra	3559	1.00459928	0.01657
4216	76857	Spopl	339745	-0.988894685	0.34967
4217	170768	Pfkfb3	5209	0.069313934	0.99242
4218	27377	Yme1l1	10730	-0.002324584	0.99988
4219	67121	Mastl	84930	-1.203746424	0.14161
4220	74159	Acbd5	91452	-0.101367622	0.991
4221	329165	Abi2	10152	-0.399896851	0.58979
4222	56075	Pdss1	23590	-0.975824024	0.52678
4223	263803	Pkn3	29941	0.360423477	0.93433
4224	54519	Apbb1ip	54518	0.488587437	0.08283
4225	71834	Zbtb43	23099	-0.104343829	0.99988
4226	18286	Odf2	4957	-0.465180509	0.5421
4227	227738	Lrsam1	90678	-0.157602174	0.82101
4228	227737	Niban2	64855	-0.321213868	0.65346
4229	20910	Stxbp1	6812	-0.288562636	0.991
4230	227683	Coq4	51117	-0.140345321	0.99988
4231	68975	Med27	9442	-0.137818342	0.99988
4232	22130	Ttf1	7270	0.568256825	0.07868
4233	227674	Ddx31	64794	0.943054685	0.01399
4234	13481	Dpm2	8818	-0.501561938	0.62525
4235	50935	St6galnac6		-0.869064974	0.2905
4236	64930	Tsc1	7248	-0.157588824	0.97741
4237	13805	Eng	2022	0.22067294	0.64319
4238	70239	Gtf3c5	9328	-1.622430417	0.00926
4239	11636	Ak1	203	0.982972828	0.32302
4240	227731	Slc25a25	114789	0.905253136	0.01123
4241	96979	Ptges2	80142	-0.627752357	0.40494
4242	19730	Ralgds		0.818594159	0.20446
4243	16819	Lcn2	3934	-1.586854199	0.20125
4244	18227	Nr4a2	4929	0.822435046	0.01467
4245	14571	Gpd2	2820	-0.581021252	0.45989
4246	69327	700007K13R	138162	-0.482697836	0.80587
4247	227929	Cytip	9595	-0.036895585	0.99988

4248	56177	Olfm1	10439	1.689879233	0.00388
4249	11477	Acvr1	90	0.685669809	0.24121
4250	12831	Col5a1	1289	0.855635313	0.01548
4251	76654	Upp2	151531	0.258898196	0.6635
4252	11350	Abl1	25	0.629862565	0.57622
4253	320267	Fubp3	8939	-0.878098031	0.24007
4254	30934	Tor1b	27348	-0.067879408	0.82705
4255	30931	Tor1a	1861	0.372711715	0.5804
4256	227707	BC005624	51759	-0.138623433	0.83994
4257	12908	Crat	1384	-0.238543639	0.66643
4258	74270	Usp20	10868	-1.039277749	0.30438
4259	57170	Dolpp1	57171	-0.032279714	0.99988
4260	66617	Ntmt1	28989	-0.826930725	0.325
4261	108958	Miga2	84895	0.963895114	0.14788
4262	227700	Sh3glb2	56904	-0.065221058	0.99988
4263	14828	Hspa5	3309	0.450215354	0.03988
4264	66691	Gapvd1	26130	-0.493938818	0.43426
4265	66998	Psmd5	5711	-0.121819242	0.86124
4266	77996	Cutal		-1.401956807	0.19868
4267	24136	Zeb2	9839	0.727066846	0.01837
4268	22029	Traf1	7185	1.458493944	0.00734
4269	68365	Rab14	51552	-0.542139549	0.34122
4270	227753	Gsn	2934	0.667547701	0.96083
4271	13830	Stom	2040	-1.230476996	0.19145
4272	69601	Dab2ip	153090	-0.543707163	0.39601
4273	67871	Mrrf	92399	0.784697962	0.3704
4274	67889	Rbm18	92400	0.621747103	0.02527
4275	227960	Gca	25801	-0.083591101	0.99988
4276	68375	Ndufa8	4702	0.224866408	0.45952
4277	71586	Ifih1	64135	-0.238681229	0.99988
4278	59029	Psmd14	10213	0.455937637	0.02564
4279	20744	Strbp	55342	-0.302710133	0.99779
4280	140858	Wdr5	11091	0.004563516	0.99988
4281	67382	Brd3	8019	-0.852803463	0.19287
4282	353156	Egfl7	51162	-0.66857367	0.46014
4283	67512	Agpat2	10555	-0.536225006	0.50224
4284	227648	Sec16a	9919	-0.515091902	0.49105
4285	64436	Inpp5e	56623	-1.173972083	0.29338
4286	66865	Pmpca	23203	-0.591702852	0.53754
4287	68112	Entr1	10807	-0.87457454	0.24307
4288	67839	Gpsm1	26086	0.127171611	0.99988
4289	67991	Nacc2	138151	-0.17848268	0.99988
4290	227634	Camsap1	157922	-0.918239119	0.14438
4291	14133	Fcna		1.988076031	0.00613
4292	51875	Tmem141	85014	0.187540765	0.65242
4293	22030	Traf2	7186	0.271524513	0.8023
4294	11305	Abca2	20	0.0564774	0.99988
4295	64685	Nmi	9111	0.806927894	0.3987
4296	227620	Uap1l1	91373	-0.209492257	0.66697
4297	83768	Dpp7	29952	0.356008164	0.41575

4298	65103	Arl6ip6	151188	0.157640212	0.99988
4299	227618	Lrrc26	389816	-0.556193882	0.45862
4300	99152	Anapc2	29882	-0.077296979	0.99988
4301	68475	Ssna1	8636	0.062985598	0.99988
4302	56878	Rbms1	5937	-0.05675404	0.99988
4303	16420	Itgb6	3694	0.81954164	0.52375
4304	215705	Arrdc1	92714	0.278491119	0.98032
4305	67187	Zmynd19	116225	-1.244020316	0.14414
4306	57438	Marchf7	64844	-0.164946511	0.75164
4307	215632	Psd4	23550	0.205703352	0.91872
4308	17076	Ly75		0.13357523	0.99988
4309	16181	Il1rn	3557	2.234316941	0.00407
4310	140483	Hnmt	3176	0.567996476	0.04242
4311	407823	Baz2b	29994	-0.844279204	0.27386
4312	72137	Wdsub1	151525	0.614115488	0.84336
4313	76747	Dapl1	92196	-0.951860094	0.43752
4314	227937	Pkp4	8502	-0.572212542	0.52716
4315	14425	Galnt3	2591	-1.108008275	0.24732
4316	69482	Nup35	129401	0.777260374	0.35468
4317	68082	Dusp19	142679	1.294068581	0.00652
4318	50884	Nckap1	10787	-0.237535516	0.61679
4319	20378	Frzb	2487	1.444121524	0.00564
4320	66861	Dnajc10	54431	-1.077180258	0.21459
4321	70599	Itprid2	6744	-0.227304411	0.80358
4322	16401	Itga4	3676	0.498645079	0.32127
4323	78830	Slc25a12	8604	0.363638222	0.03716
4324	22193	Ube2e3	10477	-0.075483776	0.99988
4325	13427	Dync1i2	1781	0.201642975	0.99988
4326	80744	Cwc22		-0.681006259	0.43562
4327	107435	Hat1	8520	0.326948915	0.05245
4328	53416	Stk39	27347	-0.056728439	0.93092
4329	241447	Cers6	253782	0.457140786	0.57989
4330	20833	Ssrp1	6749	-0.740513958	0.20334
4331	58207	Slc43a3	29015	0.805719852	0.17405
4332	72401	Slc43a1	8501	-0.730585901	0.8147
4333	30059	Timm10	26519	0.742032134	0.24687
4334	56791	Ube2l6	9246	-1.149481328	0.24625
4335	98985	Clp1	10978	-0.142125188	0.8496
4336	381379	Med19	219541	-0.956291797	0.27473
4337	21788	Tfpi	7035	0.678831007	0.02196
4338	320720	Fastkd1	79675	0.744835877	0.11866
4339	16410	Itgav	3685	-0.400855061	0.47737
4340	73373	Phospho2	493911	0.441326683	0.61679
4341	69082	Zc3h15	55854	-0.234667376	0.99988
4342	53375	Mtx2	10651	-0.106644339	0.99988
4343	15437	Hoxd8	3234	0.49485738	0.24117
4344	11909	Atf2	1386	0.219242237	0.8458
4345	11435	Chrna1	1134	0.67787655	0.0222
4346	67059	Ola1	29789	-0.343175823	0.51114
4347	20687	Sp3	6670	-1.090988109	0.34641

4348	16403	Itga6	3655	0.013589102	0.99988
4349	212772	Arl14ep	120534	0.123232451	0.99988
4350	107723	Slc12a6		-0.441006012	0.70822
4351	68032	Emc4	51234	0.159181468	0.99988
4352	72425	Katnbl1	79768	0.501747923	0.54855
4353	66181	Nop10	55505	-0.024561837	0.99988
4354	68201	Ccdc34	91057	-0.11332949	0.99068
4355	22343	Lin7c	55327	-0.500773102	0.47417
4356	76501	Commd9	29099	-0.802200922	0.40234
4357	22034	Traf6	7189	-0.351628603	0.72076
4358	68170	Iftap	119710	0.822210387	0.01649
4359	99349	Dnajc24	120526	-0.102295239	0.99988
4360	77766	Elp4	26610	0.410461448	0.55816
4361	98221	Eif3m	10480	-0.247623701	0.991
4362	228410	Cstf3	1479	-0.175630565	0.99634
4363	15259	Hipk3	10114	0.632828837	0.08857
4364	57443	Fbxo3	26273	0.180236998	0.73794
4365	53872	Caprin1	4076	-0.431642323	0.41035
4366	98956	Nat10	55226	-0.659564488	0.60471
4367	13711	Elf5	2001	0.283391531	0.98477
4368	12359	Cat	847	0.577379618	0.16501
4369	210622	Pamr1	25891	0.399099216	0.08818
4370	80985	Trim44	54765	-0.494941712	0.3812
4371	11800	Api5	8539	-0.461708216	0.55004
4372	74569	Ttc17	55761	-0.213312188	0.69765
4373	56348	Hsd17b12	51144	0.072838402	0.99988
4374	14043	Ext2	2132	0.5278314	0.68965
4375	67092	Gatm	2628	0.607205252	0.02722
4376	214968	Sema6d	80031	-0.25077323	0.6847
4377	17876	Myef2		-0.589222327	0.59429
4378	110074	Dut	1854	-0.431839542	0.6985
4379	14118	Fbn1	2200	0.28363793	0.86106
4380	12848	Cops2	9318	-0.121023741	0.93113
4381	69976	Galk2	2585	0.257586323	0.7448
4382	14178	Fgf7	2252	-0.19094335	0.99788
4383	17536	Meis2	4212	-0.294574607	0.58979
4384	12521	Cd82	3732	0.530456322	0.74859
4385	241556	Tspan18	90139	-0.176296753	0.97371
4386	269346	Slc28a2		2.142328537	0.00416
4387	76969	Chst1	8534	-0.184912691	0.99988
4388	18633	Pex16	9409	-0.179488056	0.99988
4389	19099	Mapk8ip1	9479	-1.358311984	0.22449
4390	213696	Duoxa1	90527	-0.885362485	0.77419
4391	20322	Sord	6652	0.182715447	0.38367
4392	26427	Creb3l1	90993	-0.91959781	0.3704
4393	78655	Eif3j1		-0.217540609	0.59955
4394	228564	Frmd5	84978	0.110859668	0.95309
4395	17242	Mdk	4192	-0.624403178	0.99988
4396	241627	Wdr76	79968	-0.299367827	0.77629
4397	241547	Harbi1	283254	-1.432620925	0.19425

4398	51897	Atg13	9776	-0.209745168	0.57803
4399	67693	Hypk	25764	-0.347719137	0.4539
4400	269344	Ell3	80237	-0.152656204	0.99988
4401	228359	Arhgap1	392	-0.152988086	0.85302
4402	14827	Pdia3	2923	-0.01387135	0.99988
4403	228357	Lrp4	4038	0.710679615	0.45288
4404	17754	Map1a	4130	-0.943065782	0.32448
4405	80708	Pacsin3	29763	-0.150866725	0.86402
4406	75894	Adal	161823	1.11436839	0.25652
4407	51885	Tubgcp4	27229	0.307352126	0.13983
4408	22222	Ubr1	197131	0.791433894	0.00975
4409	20614	Snap25	6616		0.40878
4410	59030	Mkks	8195	1.105171626	0.01131
4411	16449	Jag1	182	-0.45521557	0.7376
4412	74243	Slx4ip	128710	0.363906435	0.5292
4413	56428	Mtch2	23788	-0.137291775	0.88887
4414	68968	Cdan1	146059	0.351773124	0.0243
4415	66296	Haus2	55142	-1.255662096	0.28792
4416	66606	Lrrc57		-0.850335928	0.35482
4417	20619	Snap23	8773	0.810736091	0.02176
4418	20402	Zfp106	64397	-0.30712342	0.60827
4419	269338	Vps39	23339	-0.496955105	0.4782
4420	98878	Ehd4	30844	0.035172715	0.99988
4421	17005	Ltk	4058	0.154143758	0.991
4422	22174	Tyro3	7301	0.292153968	0.99988
4423	140629	Ubox5	22888	0.08572122	0.99988
4424	19262	Ptpra	5786	0.085900632	0.82884
4425	76246	Rtf1	23168	-0.043635315	0.95893
4426	69702	Ndufaf1	51103	-0.106379083	0.98116
4427	108907	Nusap1		-0.954922675	0.29836
4428	228602	930402H24R	25943	-0.044684751	0.99988
4429	11990	Atrn	8455	0.390887454	0.28283
4430	69065	Chac1	79094	0.463099348	0.55704
4431	20732	Spint1	6692	0.255186042	0.91606
4432	110751	Adam33	80332	1.637054749	0.00566
4433	20612	Siglec1	6614	1.314424353	0.00664
4434	19361	Rad51	5888	0.396726012	0.59694
4435	76464	Knl1	57082	0.72710904	0.35314
4436	67326	700037H04R	54976	0.437480778	0.46959
4437	70997	Spef1	25876	-0.141973903	0.85293
4438	12531	Cdc25b	994	1.817462248	0.00777
4439	51944	Knstrn	90417	-0.649756327	0.6236
4440	56357	Ivd	3712	-0.334371823	0.71694
4441	228608	Smox	54498	-0.502982112	0.44277
4442	215653	Rassf2	9770	2.039311729	0.01146
4443	54338	Slc23a2	9962	0.592221144	0.04045
4444	70612	Tmem230	29058	0.490158211	0.49171
4445	18538	Pcna	5111	0.220179494	0.73689
4446	74182	Gpcpd1	56261	-0.222128654	0.6455
4447	19419	Rasgrp1	10125		0.40878

4448	68215	Fam98b	283742	-0.281583965	0.97675
4449	114715	Spred1	161742	0.208964873	0.74549
4450	66634	Mcm8		-0.488458155	0.90691
4451	241639	Fermt1	55612	-1.291312116	0.1705
4452	66586	Crls1	54675	-0.129498263	0.93453
4453	12156	Bmp2	650	1.164635814	0.01089
4454	15186	Hdc	3067	1.508158349	0.00566
4455	14391	Gabpb1	2553	-1.21922012	0.18514
4456	84092	Usp8	9101	-0.892953251	0.48755
4457	75083	Usp50	373509	-0.336116042	0.77533
4458	58800	Trpm7	54822	-0.275323796	0.76671
4459	66552	Sppl2a	84888	-0.709603574	0.3058
4460	99138	Stard7	56910	1.738972077	0.05129
4461	13537	Dusp2	1844	1.967339437	0.00447
4462	68126	Fahd2a	51011	-0.249409792	0.83128
4463	77721	Mrps5	64969	-0.353179144	0.75823
4464	17153	Mal	4118	0.588326563	0.30825
4465	192212	Prom2	150696	-0.187981198	0.95058
4466	228576	Mall	7851	1.496596095	0.00837
4467	53885	Nphp1	4867	-0.18981503	0.99988
4468	12125	Bcl2l11	10018	-0.134351058	0.99988
4469	69487	Ndufaf5	79133	1.03861864	0.03589
4470	70370	Fbln7	129804	0.688948789	0.86113
4471	57432	Zc3h8	84524	-0.389238114	0.46079
4472	69737	Ttl	150465	0.444470293	0.27645
4473	20017	Polr1b	84172	-0.989012162	0.24507
4474	20515	Slc20a1	6574	0.395068978	0.44912
4475	16176	Il1b	3553	1.597241285	0.0079
4476	20638	Snrpb	6628	0.068114814	0.92721
4477	67134	Nop56	10528	-1.072067874	0.11624
4478	170718	Idh3b	3420	0.146270067	0.65178
4479	56264	Cpxm1	56265	-0.126749881	0.74793
4480	80743	Vps16	64601	-0.481709883	0.43562
4481	64899	Lpin3	64900	0.097220293	0.99988
4482	12075	Bfsp1	631	-0.292159459	0.72791
4483	81910	Rrbp1	6238	-0.131633522	0.89785
4484	69178	Snx5	27131	0.872413036	0.02372
4485	74528	Mgme1	92667	-0.106343759	0.96967
4486	228714	Kat14	57325	-0.18641252	0.95106
4487	70408	Polr3f	10621	0.652321216	0.05979
4488	26450	Rbbp9	10741	0.962435162	0.01186
4489	27054	Sec23b	10483	-0.342192091	0.53817
4490	66044	Dtd1	92675	0.077975559	0.88511
4491	24128	Xrn2	22803	-0.171660854	0.74807
4492	17064	Cd93	22918	1.156953049	0.01194
4493	17957	Napb	63908	-0.47653948	0.94097
4494	74533	Gzf1	64412	-0.236516446	0.69275
4495	13010	Cst3		0.651919125	0.17387
4496	68738	Acss1	84532	-0.372940961	0.49268
4497	69270	Gins1	9837	-0.080602209	0.9314

4498	386649	Nsfl1c	55968	-0.472362456	0.43843
4499	228765	Sdcbp2	27111	0.181394601	0.99988
4500	73847	Fam110a	83541	-0.237946516	0.82705
4501	11602	Angpt4	51378	0.986275773	0.86725
4502	69698	Slc52a3	113278	0.50530997	0.5858
4503	67231	Tbc1d20	128637	-0.260041163	0.99988
4504	24105	Rbck1	10616	-0.849667086	0.21634
4505	72119	Tpx2	22974	-1.03169129	0.36223
4506	68559	Pdrg1	81572	0.016873375	0.99988
4507	228788	Ccm2l	140706	0.157552017	0.99988
4508	16569	Kif3b	9371	0.370505909	0.57255
4509	13436	Dnmt3b		0.151540465	0.66364
4510	13589	Mapre1	22919	-0.3565918	0.49412
4511	66971	Cdk5rap1	51654	1.212472581	0.02353
4512	20648	Snta1	6640	1.036954997	0.02462
4513	13555	E2f1	1869	-0.122360959	0.78946
4514	67017	Fam210b	116151	0.415670956	0.46192
4515	20878	Aurka	6790	-0.218518078	0.99988
4516	67337	Cstf1	1477	0.036072531	0.99988
4517	18767	Pkia	5569	0.895914922	0.23715
4518	20257	Stmn2	11075	1.958377013	0.00591
4519	66404	Rtf2	51507	-0.646197011	0.32908
4520	21985	Tpd52	7163	-0.344676456	0.60943
4521	94212	Pag1	55824	1.348413691	0.00718
4522	66679	Rae1	8480	0.266587409	0.72513
4523	56190	Rbm38	55544	1.134607081	0.00985
4524	58203	Zbp1	81030	0.909130152	0.44354
4525	19334	Rab22a	57403	0.411806785	0.25652
4526	228960	Stx16		0.328002821	0.36141
4527	14683	Gnas	2778	-0.257676663	0.97103
4528	55980	Impa1	3612	0.054001396	0.65837
4529	16592	Fabp5	2171	-1.058158686	0.33498
4530	74718	Snx16	64089	-1.711728006	0.11463
4531	66371	Chmp4c	92421	-0.600468352	0.55298
4532	19246	Ptpn1	5770	-0.921552403	0.43132
4533	18019	Nfatc2	4773	1.299730761	0.14451
4534	11981	Atp9a	10079	-0.6149802	0.53344
4535	71710	Lrrcc1	85444	0.512605246	0.03093
4536	22722	Zfp64	55734	0.098406388	0.99988
4537	13559	E2f5	1875	-0.880325415	0.28245
4538	71934	Car13	377677	0.771972092	0.2541
4539	12350	Car3	761	-0.637248019	0.21806
4540	12349	Car2	760	-0.76599387	0.42699
4541	26444	Psma7	5688	0.0097932	0.99988
4542	73247	Mrgbp	55257	-1.06172321	0.24006
4543	76425	Gid8	54994	0.58714909	0.02548
4544	228998	Arfgap1	55738	-0.272599954	0.75567
4545	229003	Helz2	85441	-0.348226029	0.8004
4546	229007	Zgpat		0.357459506	0.67224
4547	72147	Zbtb46	140685	0.330761846	0.46469

4548	245867	Pcmtd2	55251	0.226499465	0.38293
4549	19383	Raly	22913	0.726942786	0.17263
4550	269378	Ahcy		0.085080754	0.77827
4551	16396	Itch	83737	-0.80740649	0.75672
4552	74252	Armc1	55156	-0.218235669	0.70199
4553	67472	Mtfr1	9650	0.491516234	0.03393
4554	66734	Map1lc3a	84557	-0.256992863	0.82411
4555	60525	Acss2	55902	1.179076175	0.02019
4556	14854	Gss	2937	0.351687762	0.66111
4557	19124	Procr	10544	0.071284863	0.91207
4558	17391	Mmp24	10893	-0.2093847	0.99988
4559	16418	Eif6	3692	-0.441597813	0.5966
4560	12807	Hps3	84343	0.062801881	0.98415
4561	18041	Nfs1	9054	0.50474479	0.02181
4562	170791	Rbm39	9584	-0.536298312	0.39006
4563	13821	Epb41l1	2036	-0.785599628	0.35792
4564	68295	Aar2	25980	-0.162559816	0.72895
4565	81004	Tbl1xr1	79718	-0.429934564	0.60837
4566	29812	Ndrg3	57446	-0.5553679	0.6316
4567	66934	Dsn1	79980	-0.280007741	0.84859
4568	77799	Sla2	84174	0.514536014	0.74197
4569	67388	Rab5if	55969	-0.095823376	0.99988
4570	56045	Samhd1	25939	0.357531157	0.10581
4571	19650	Rbl1	5933	-0.372776024	0.79325
4572	20014	Rpn2	6185	-0.12436596	0.84237
4573	20779	Src	6714	-0.620194255	0.52129
4574	66642	Ctnnbl1	56259	0.098059496	0.83528
4575	75425	Tti1	9675	0.088866135	0.9519
4576	70470	Rprd1b	58490	-0.66859752	0.40207
4577	228850	Ralgapb	57148	-0.170183313	0.72791
4578	71878	Fam83d	81610	1.14824396	0.0123
4579	71715	Dhx35	60625	-1.151532411	0.37566
4580	22403	Ccn5	8839	1.028179473	0.57255
4581	20482	Skil	6498	0.565699137	0.21674
4582	22401	Zmat3	64393	-0.585045886	0.40219
4583	18706	Pik3ca	5290	-0.137203222	0.93704
4584	67778	Zfp639	51193	-0.130451326	0.92332
4585	67414	Mfn1	55669	-0.633831625	0.41675
4586	14696	Gnb4	59345	-0.309072552	0.99988
4587	56456	Actl6a	86	0.518568636	0.69975
4588	66046	Ndufb5		0.449567497	0.03487
4589	58869	Pex5l	51555		0.40878
4590	67120	Ttc14	151613	-0.637362208	0.35657
4591	17979	Ncoa3	8202	0.135182141	0.78226
4592	67713	Dnajc19	131118	0.076104941	0.87985
4593	14359	Fxr1	8087	-0.476117919	0.65197
4594	14013	Mecom	2122	0.41044672	0.0362
4595	665113	Tnik	23043	-0.639681327	0.4834
4596	18805	Pld1	5337	0.79914716	0.81534
4597	320024	Nceh1	57552	0.550992473	0.31606

4598	13605	Ect2	1894	0.112357963	0.99988
4599	69276	Sec62	7095	0.12689717	0.78259
4600	114893	Dcun1d1		-0.272133364	0.59613
4601	72039	Mccc1	56922	0.788744949	0.39825
4602	229211	Acad9	28976	-0.112919389	0.99988
4603	11747	Anxa5	308	-0.492114559	0.48422
4604	50911	Exosc9	5393	1.120753733	0.00849
4605	12428	Ccna2	890	0.28161542	0.82473
4606	16183	Il2	3558	0.495956492	0.42002
4607	57815	Spata5	166378	1.750175401	0.04344
4608	26570	Slc7a11	23657	0.477497371	0.63927
4609	19338	Rab33b	83452	0.526325145	0.41621
4610	67542	Cog6	57511	-0.005949937	0.99988
4611	67890	Ufm1	51569	-0.080244693	0.80153
4612	50706	Postn	10631	0.53710895	0.49634
4613	56790	Supt20	55578	-1.100378351	0.20468
4614	69639	Exosc8	11340	0.76405845	0.14638
4615	56758	Mbnl1	4154	0.244424364	0.8603
4616	72162	Dhx36	170506	-0.988257358	0.20621
4617	28030	Gfm1	85476	-0.001266958	0.99988
4618	66868	Mfsd1	64747	0.722649328	0.01701
4619	30953	Schip1		2.504642116	0.02422
4620	68259	Ift80	57560	0.6539785	0.40066
4621	16649	Kpna4	3840	0.224144526	0.94838
4622	242083	Ppm1l	151742	0.232744128	0.75897
4623	97112	Nmd3	51068	0.535824576	0.45946
4624	13175	Dclk1	9201	-0.284316356	0.77629
4625	26422	Nbea	26960	-0.250946602	0.75672
4626	17112	Tm4sf1	4071	0.832453613	0.26606
4627	97064	Wwtr1	25937	-0.665461619	0.43192
4628	67738	Ppid	5481	0.305696945	0.75897
4629	18645	Pfn2	5217	-0.541277863	0.44726
4630	72033	Tsc22d2	9819	0.001962362	0.99988
4631	28146	Serp1	27230	0.170255971	0.11051
4632	66841	Etfdh	2110	-0.038350805	0.99988
4633	229317	Eif2a	83939	0.384568261	0.56553
4634	17380	Mme	4311	-1.325837312	0.20861
4635	11416	Slc33a1	9197	0.011027858	0.99988
4636	229363	Gmps	8833	0.462623496	0.27454
4637	16497	Kcnab1	7881	1.204301338	0.39825
4638	67437	Ssr3	6747	0.069266569	0.99988
4639	56706	Ccnl1	57018	-0.731769563	0.21958
4640	19288	Ptx3	5806	0.632542759	0.3364
4641	20713	Serpini1	5274		0.40878
4642	56426	Pdcd10	11235	-0.130473691	0.75697
4643	19260	Ptpn22	26191	0.687078734	0.19299
4644	140917	Dclre1b	64858	-1.36314294	0.1952
4645	99543	Olfml3	56944	1.116094908	0.0155
4646	18176	Nras	4893	-0.282410564	0.61121
4647	66641	Sike1	80143	0.439608604	0.12745

4648	70747	Tspan2	10100	0.665953458	0.13824
4649	229658	Vangl1	81839	-0.145169454	0.83434
4650	19221	Ptgfrn	5738	0.169795148	0.71459
4651	14547	Gdap2	54834	-0.145385925	0.99988
4652	15360	Hmgcs2	3158	0.659310653	0.61942
4653	18129	Notch2	4853	1.161867237	0.00873
4654	20333	Sec22b	9554	0.265111721	0.62774
4655	66921	Prpf38b	55119	-0.534857729	0.35611
4656	20912	Stxbp3	6814	0.7044186	0.01391
4657	76123	Gpsm2	29899	0.335627732	0.93704
4658	229725	Clcc1	23155	0.496007965	0.02101
4659	109674	Ampd2	271	0.581878068	0.02658
4660	229709	Ahcyl1	10768	-0.267625879	0.64863
4661	229699	Slc16a4	9122	-0.944589974	0.22449
4662	67171	Dram2	128338	-0.069641854	0.91487
4663	72121	Dennd2d	79961	-0.204673006	0.91901
4664	53975	Ddx20	11218	0.392520942	0.40797
4665	20195	S100a11	6282	-0.339278187	0.93116
4666	18160	Npr1	4881	-0.096691596	0.99988
4667	26568	Slc27a3	11000	0.671418045	0.48048
4668	229543	Ints3	65123	0.196142174	0.99988
4669	68328	Rab13		0.507200672	0.02932
4670	74343	Crtc2	200186	-0.920456909	0.43717
4671	23922	Jtb		0.042189509	0.99988
4672	78284	Creb3l4	148327	-1.010365438	0.27533
4673	59069	Tpm3		-0.252961299	0.59919
4674	99650	933434E20Rik		0.255582383	0.38098
4675	23897	Hax1	10456	0.191733136	0.93704
4676	16194	Il6ra	3570	0.046130916	0.99988
4677	11444	Chrnb2	1141	-0.25405616	0.7594
4678	56417	Adar	103	0.5853658	0.57687
4679	68603	Pmvk	10654	0.183414398	0.99988
4680	19729	Slc50a1	55974	-0.160056945	0.97196
4681	13636	Efna1	1942	-0.381740587	0.99016
4682	68659	Gask1b	51313	1.032506167	0.20205
4683	70652	Tmem144	55314	-0.746202507	0.31834
4684	229782	Slc35a3	23443	0.050747153	0.99397
4685	72776	Sass6	163786	-0.35276188	0.52678
4686	109245	Lrrc39	127495	-0.101506834	0.99988
4687	22329	Vcam1	7412	1.673310176	0.00717
4688	58193	Extl2	2135	0.203233211	0.77068
4689	12814	Col11a1	1301	0.343495902	0.74185
4690	28036	Larp7		-0.622189626	0.48182
4691	19142	Prss12	8492	0.802507151	0.38421
4692	67225	Rnpc3	55599	-1.572418726	0.08163
4693	71519	Cyp2u1	113612	0.015509389	0.99989
4694	15107	Hadh	3033	0.608912281	0.14274
4695	80890	Trim2	23321	-1.067945437	0.26201
4696	66815	Mcub	55013	1.522388231	0.00765
4697	24088	Tlr2	7097	0.195754392	0.65408

4698	20319	Sfrp2	6423	0.92431066	0.57314
4699	12368	Casp6	839	0.348481043	0.44022
4700	53317	Plrg1	5356	0.513917925	0.02781
4701	66350	Pla2g12a	81579	-0.607291836	0.33821
4702	79235	Lrat	9227	-1.304376652	0.28445
4703	54195	Gucy1b1	2983	-1.467393436	0.19457
4704	76561	Snx7	51375	-0.46109877	0.58905
4705	68147	Gar1	54433	-0.763083047	0.38009
4706	56720	Tdo2	6999	0.212059512	0.84256
4707	74776	Ppa2	27068	0.249982418	0.8398
4708	229445	Ctso	1519	0.482952302	0.07886
4709	71793	Ints12	57117	-0.304871737	0.99988
4710	13645	Egf	1950	-1.91927547	0.07596
4711	67553	Gstcd	79807	0.071419423	0.99988
4712	54635	Pdgfc	56034	0.920650771	0.11226
4713	14658	Glrb	2743	-0.167573976	0.67674
4714	71481	Alpk1	80216	1.466055235	0.00566
4715	13722	Aimp1	9255	-0.181303235	0.70348
4716	271981	Tbck	93627	0.7472288	0.01802
4717	56811	Dkk2	27123	2.55276951	0.00356
4718	23971	Papss1	9061	-0.579939274	0.44207
4719	51886	Fubp1	8880	-0.970105351	0.27649
4720	67035	Dnajb4	11080	0.1023684	0.99988
4721	19220	Ptgfr	5737	-0.115677353	0.99988
4722	99899	Ifi44	10561	-0.740287712	0.60462
4723	13639	Efna4	1945	-1.238486652	0.27894
4724	11490	Adam15	8751	0.135445776	0.99988
4725	22724	Zbtb7b	51043	-0.331188089	0.991
4726	54124	Cks1b		0.402761473	0.03311
4727	21827	Thbs3	7059	1.281931029	0.13875
4728	14466	Gba	2629	0.13108821	0.99988
4729	24045	Scamp3	10067	-0.428825466	0.57262
4730	192195	Ash1l	55870	-0.001141476	0.99988
4731	19769	Rit1	6016	-0.271998555	0.76549
4732	16800	Arhgef2	9181	0.35717805	0.31839
4733	74200	Khdc4	22889	0.338130681	0.85104
4734	83409	Lamtor2	28956	-0.12848894	0.90973
4735	16905	Lmna	4000	-0.016401293	0.99988
4736	20351	Sema4a	64218	-0.273490589	0.51984
4737	67037	Pmf1		0.27877317	0.65242
4738	404710	Iqgap3	128239	0.212445459	0.99988
4739	66614	Gpatch4	54865	-0.455920394	0.58017
4740	246703	Naxe	128240	-0.158147661	0.99816
4741	27371	Sh2d2a	9047	0.557059085	0.22364
4742	73182	Pear1	375033	0.084403847	0.99988
4743	12479	Cd1d1			0.40878
4744	80877	Lrba	987	-1.000081607	0.25491
4745	20091	Rps3a1	6189	-0.153862656	0.84891
4746	27059	Sh3d19	152503	0.442757734	0.35311
4747	229487	Gatb	5188	0.36134689	0.97975

4748	50754	Fbxw7	55294	-0.765765072	0.32683
4749	14263	Fmo5	2330	1.712365998	0.00578
4750	68058	Chd1l	9557	0.026008386	0.99988
4751	66659	Acp6	51205	1.030908029	0.19683
4752	67549	Gpr89		-1.231624767	0.24625
4753	67845	Rnf115	27246	0.042244815	0.99988
4754	74414	Polr3c	10623	-0.094356776	0.99988
4755	229615	Pias3	10401	0.473978884	0.95278
4756	18632	Pex11b	8799	-0.53449051	0.36977
4757	69870	Polr3gl		0.202554943	0.57681
4758	75137	Rprd2	23248	0.238354189	0.99988
4759	71807	Tars2	80222	0.458250352	0.82012
4760	13038	Ctsk	1513	0.632055028	0.85956
4761	210529	Mettl14	57721	-0.319132745	0.67602
4762	171388	Bnipl	149428	-0.330047232	0.99528
4763	29815	Bcar3	8412	1.437851369	0.00591
4764	14630	Gclm	2730	0.099113766	0.82835
4765	18720	Pip5k1a	8394	-0.181571695	0.91475
4766	19299	Abcd3	5825	-0.118149469	0.73082
4767	14066	F3	2152	-0.629958932	0.89195
4768	99887	Tlcd4	100527978	-0.376070566	0.66885
4769	66568	Rwdd3	25950	1.103338075	0.01132
4770	56195	Ptbp2	58155	-0.658533835	0.51148
4771	76742	Snx27	81609	-0.430397581	0.48572
4772	11532	Adh5	128	0.721366003	0.19517
4773	78523	Mrpl9	65005	-0.269441528	0.78607
4774	53814	Oaz3	51686	0.137063839	0.99988
4775	75778	Them4	117145	0.485729079	0.93704
4776	229877	Rap1gds1	5910	0.39669996	0.52843
4777	19885	Rorc	6097	0.739021843	0.03816
4778	56224	Tspan5	10098	-0.058545246	0.99988
4779	13684	Eif4e	1977	0.653340946	0.01342
4780	17777	Mttp	4547	-1.091007866	0.12452
4781	26377	Dapp1	27071	1.626588778	0.0041
4782	19055	Ppp3ca	5530	-0.004101109	0.99988
4783	18033	Nfkb1	4790	-1.013154732	0.2064
4784	110173	Manba	4126	-1.028469199	0.02422
4785	67006	Cisd2	493856	0.028031611	0.97948
4786	69772	Bdh2	56898	1.572661405	0.03205
4787	68151	Wls	79971	0.117090983	0.89693
4788	53861	Zranb2	9406	0.141816352	0.90026
4789	99633	Adgrl2	23266	-1.154893377	0.224
4790	70285	Rpf1	80135	0.390857241	0.04473
4791	70951	Spata1	100505741	-1.559349825	0.20706
4792	74245	Ctbs	1486	0.797053895	0.03382
4793	12042	Bcl10	8915	0.401538869	0.0436
4794	69219	Ddah1	23576	-0.206842542	0.99988
4795	16007	Ccn1	3491	1.868510895	0.00624
4796	12972	Cryz	1429	-0.228234489	0.69444
4797	65973	Asph	444	-0.204306784	0.76455

4798	60599	Trp53inp1	94241	-0.221831796	0.92618
4799	12448	Ccne2	9134	-0.4045946	0.50458
4800	14579	Gem	2669	0.219978805	0.84891
4801	12557	Cdh17	1015	-1.829539302	0.1237
4802	68099	Cibar1	137392	-0.244773791	0.89045
4803	72519	Pip4p2	55529	1.220617991	0.02635
4804	67460	Decr1	1666	0.324009089	0.64211
4805	27354	Nbn	4683	0.067078822	0.99988
4806	70568	Cpne3	8895	0.44258186	0.28042
4807	66302	Rmdn1	51115	0.37879862	0.61794
4808	72098	Tmem68	137695	-0.247271435	0.63778
4809	116940	Tgs1	96764	-0.483504917	0.39396
4810	67427	Rps20	6224	0.356148952	0.39387
4811	242341	Atp6v0d2	245972	0.37572262	0.04889
4812	68053	Ubxn2b	137886	-0.035637995	0.93453
4813	18201	Nsmaf	8439	0.507811841	0.2533
4814	230027	Coq3	51805	-0.187925886	0.70304
4815	66625	Pnisr	25957	-0.703525726	0.34451
4816	53378	Sdcbp	6386	0.614072743	0.01057
4817	77032	Tstd3		1.477223183	0.00577
4818	51813	Ccnc	892	1.218280388	0.01054
4819	23844	Clca1	1179	0.538445248	0.51226
4820	52184	Odf2l	57489	-0.787995588	0.24307
4821	68493	Ndufaf4	29078	-0.07930644	0.99988
4822	80797	Clca3a2		-1.51974284	0.15099
4823	16911	Lmo4	8543	0.062608784	0.99988
4824	55932	Gbp3		1.113282412	0.1497
4825	14469	Gbp2		0.893710915	0.03509
4826	229906	Gtf2b	2959	0.321289004	0.67983
4827	56376	Pdlim5	10611	0.621328005	0.02459
4828	24018	Rngtt	8732	0.55971358	0.51914
4829	56228	Ube2j1	51465	0.069872837	0.95947
4830	52187	Rragd	58528	-1.013496383	0.216
4831	26885	Casp8ap2	9994	-0.444447202	0.65371
4832	26409	Map3k7	6885	0.375436266	0.09084
4833	433693	Akirin2	55122	0.69598004	0.01157
4834	109093	Rars2	57038	-0.073183798	0.99988
4835	24060	Slc35a1	10559	0.165576889	0.89195
4836	66291	Smim8	57150	0.197254701	0.99988
4837	73205	C9orf72	203228	0.823126628	0.22496
4838	109331	Rnf20	56254	-0.081048314	0.99988
4839	14211	Smc2	10592	0.087774625	0.99988
4840	64424	Polr1e	64425	-0.290527295	0.89111
4841	66362	Exosc3	51010	0.694631766	0.40947
4842	74152	Stra6l		-0.787527282	0.66988
4843	21916	Tmod1	7111	0.406928423	0.64792
4844	22590	Xpa	7507	0.24130856	0.99988
4845	433702	Ncbp1	4686	0.573771257	0.07666
4846	67628	Anp32b	10541	-0.316029258	0.57305
4847	94181	Nans	54187	0.254505102	0.79344

4848	107684	Coro2a	7464	-0.174621442	0.9526
4849	12819	Col15a1	1306	1.124820999	0.22951
4850	76299	Erp44	23071	0.718609899	0.01793
4851	16348	Invs	27130	0.341924771	0.09457
4852	269536	Tex10	54881	-0.11354782	0.7787
4853	230157	Tmeff1	8577	-0.260817214	0.67216
4854	56710	Brinp1	1620	1.477188403	0.09669
4855	72701	Zfp618	114991	-1.004658786	0.25969
4856	21923	Tnc	3371	0.508925263	0.04398
4857	22166	Txn1	7295	0.580477125	0.01791
4858	64817	Svep1	79987	0.859267476	0.34636
4859	67103	Ptgr1	22949	0.12093548	0.75419
4860	22234	Ugcg	7357	0.437629973	0.62843
4861	230257	Ptbp3	9991	0.199730476	0.82473
4862	72479	Hsdl2	84263	-0.458087338	0.65529
4863	209131	Snx30	401548	-0.671556159	0.45356
4864	22696	Zfp37	7539	1.255809515	0.00933
4865	71354	Wdr31	114987	0.375177371	0.77825
4866	192120	Bspry	54836	-1.083179272	0.25466
4867	17025	Alad	210	-1.696638363	0.13577
4868	59001	Pole3	54107	-0.177798015	0.87891
4869	76804	Kdm4c	23081	1.068067473	0.09307
4870	66928	Dmac1	90871	0.530532221	0.031
4871	19266	Ptprd	5789	0.581672786	0.01649
4872	17475	Mpdz	8777	0.727727628	0.39195
4873	68268	Zdhhc21	340481	-0.579434206	0.35213
4874	11428	Aco1	48	0.241275385	0.20205
4875	100504309	Smim27		0.023104224	0.99988
4876	74255	Smu1	55234	0.279064987	0.38293
4877	15502	Dnaja1	3301	1.006174763	0.01009
4878	66408	Aptx	54840	0.310252839	0.991
4879	100434	Slc44a1	23446	0.445105773	0.03226
4880	14595	B4galt1	2683	0.645992808	0.02026
4881	246179	Fktn	2218	-0.176645863	0.74755
4882	12017	Bag1	573	-0.097899292	0.93917
4883	76959	Chmp5	51510	0.911114402	0.00837
4884	52076	Tmem38b	55151	1.790719174	0.00662
4885	74164	Nfx1	4799	-0.278691113	0.90134
4886	19359	Rad23b	5887	-0.001837436	0.99988
4887	230233	Elp1	8518	-0.436520258	0.58605
4888	68926	Ubap2	55833	-0.653715981	0.34138
4889	54357	Epb41l4b	54566	0.384769142	0.59996
4890	11828	Aqp3	360	0.682855771	0.01741
4891	68970	Dcaf12	25853	0.000405768	0.99988
4892	67123	Ubap1	51271	-0.091576626	0.99988
4893	66401	Nudt2	318	-0.55474085	0.64026
4894	69638	Enho	375704	2.367396336	0.0072
4895	53598	Dctn3	11258	0.006694015	0.99988
4896	269523	Vcp	7415	-0.189107432	0.67674
4897	60534	Fancg	2189	-0.23808937	0.80808

4898	56703	Pigo	84720	0.93764103	0.01207
4899	66592	Stoml2	30968	0.295155979	0.91487
4900	22249	Unc13b	10497	-0.381681224	0.41395
4901	21754	Tesk1	7016	0.068524487	0.99988
4902	12517	Cd72	971	0.339275128	0.73305
4903	54390	Sit1	27240	0.020085921	0.91487
4904	622404	Ccdc107	203260	-0.044451173	0.99988
4905	230099	Car9	768	-0.166316312	0.99988
4906	22004	Tpm2		3.356750632	0.00388
4907	21894	Tln1	7094	-0.130492641	0.99988
4908	12913	Creb3	10488	-0.10473421	0.99988
4909	230101	Gba2	57704	0.189060315	0.99988
4910	242406	Rgp1	9827	-0.304167964	0.87037
4911	68917	Hint2	84681	-0.171327432	0.8308
4912	53614	Reck	8434	0.759729264	0.01791
4913	12757	Clta	1211	0.106457736	0.52149
4914	50798	Gne	10020	-1.168434766	0.24003
4915	384009	Glipr2	152007	0.462112111	0.3353
4916	77634	Snapc3	6619	-0.57988703	0.40233
4917	101739	Psip1	11168	-0.816723985	0.51687
4918	11520	Plin2	123	0.745189807	0.02032
4919	20104	Rps6	6194	0.473783391	0.031
4920	70122	Mllt3	4300	-0.999295072	0.27202
4921	66775	Hacd4	401494	0.923398074	0.0146
4922	329908	Usp24	23358	0.831590678	0.41058
4923	67916	Plpp3		-0.128115539	0.99988
4924	108079	Prkaa2	5563	-1.076708226	0.22128
4925	242585	Slc35d1	23169	-0.081853392	0.99988
4926	71148	Mier1	57708	-0.332588525	0.59613
4927	18578	Pde4b	5142	0.812676412	0.01273
4928	11639	Ak4	205	-1.89501895	0.19397
4929	16451	Jak1	3716	-0.018630924	0.99988
4930	320508	Cachd1	57685	0.461176037	0.53754
4931	20441	St3gal3	6487	0.438786654	0.72736
4932	67728	Dph2	1802	-0.651321108	0.37278
4933	53418	B4galt2	8704	0.843327236	0.01701
4934	230612	Slc5a9	200010	0.14507091	0.99988
4935	67621	Bend5	79656	-0.259834075	0.76197
4936	67733	Itgb3bp	23421	1.661234218	0.00664
4937	242557	Atg4c	84938	-0.457046092	0.92239
4938	12580	Cdkn2c	1031	-0.560169243	0.48543
4939	13858	Eps15	2060	1.462258391	0.00738
4940	230603	Ttc39a	22996	-0.626853738	0.39072
4941	67299	Dock7	85440	0.230929587	0.2905
4942	29864	Rnf11	26994	-0.021065504	0.99988
4943	100273	Osbpl9	114883	0.746610487	0.01098
4944	230484	Usp1	7398	-0.520528736	0.37225
4945	94043	Tm2d1	83941	1.195964945	0.18424
4946	18027	Nfia	4774	-0.600266731	0.48487
4947	66073	Txndc12	51060	0.843555097	0.47207

4948	70533	Btf3l4	91408	0.357660265	0.26954
4949	77963	Hook1	51361	-0.474685907	0.53084
4950	75578	Fggy	55277	0.469840729	0.43682
4951	67694	Ift74	80173	-0.092501019	0.94092
4952	18786	Plaa	9373	0.038750243	0.99988
4953	67770	Caap1	79886	1.07458519	0.00795
4954	80912	Pum1	9698	-0.297077277	0.5912
4955	16792	Laptm5	7805	1.315939849	0.00653
4956	319965	Cc2d1b	200014	-1.188454347	0.14942
4957	14726	Pdpn	10630	1.342125919	0.00773
4958	77632	Pramel13		0.614628971	0.6316
4959	67305	Gpx7	2882	1.300293742	0.14414
4960	21938	Tnfrsf1b	7133	-0.224083035	0.86942
4961	242608	Podn	127435	0.891190946	0.22629
4962	52430	Echdc2	55268	0.419734455	0.60287
4963	20280	Scp2	6342	0.119448966	0.99988
4964	12896	Cpt2	1376	0.105013274	0.99988
4965	74098	Czib	54987	0.313810859	0.26928
4966	17149	Magoh	4116	-0.229043385	0.90147
4967	72787	Ndc1	55706	0.182249205	0.06044
4968	77809	Lrrc42	115353	0.476712836	0.37088
4969	56374	Tmem59	9528	0.160165207	0.45568
4970	66526	Tceanc2	127428	1.519743915	0.01774
4971	230582	Cyb5rl		-0.285135199	0.8493
4972	56280	Mrpl37	51253	0.640180359	0.41716
4973	73172	Exo5	64789	0.105411129	0.88285
4974	69181	Dyrk2	8445	0.227434329	0.07918
4975	51797	Ctps	1503	0.101603362	0.99988
4976	16656	Hivep3	59269	0.131768657	0.27055
4977	106564	Ppcs	79717	0.930679335	0.24007
4978	22608	Ybx1	4904	-0.284045043	0.59169
4979	21420	Tfap2c	7022	-0.51941616	0.9399
4980	56401	P3h1	64175	0.404142987	0.29426
4981	69216	Svbp	374969	-0.128245599	0.99988
4982	20525	Slc2a1	6513	-1.016686171	0.27008
4983	54170	Rragc	64121	-0.166871788	0.99016
4984	56309	Mycbp		0.163262643	0.79638
4985	595136	Ndufs5	4725	0.156127183	0.91655
4986	11426	Macf1	23499	0.030782811	0.99988
4987	56031	Ppie	10450	-0.607987737	0.4026
4988	66966	Trit1	54802	-0.266930702	0.92136
4989	16918	Mycl	4610	0.442323941	0.23202
4990	12331	Cap1	10487	-1.221928539	0.5159
4991	19063	Ppt1	5538	0.437683309	0.34442
4992	13844	Ephb2	2048	1.40711506	0.00624
4993	27224	Eloa	6924	0.223770823	0.9864
4994	66193	Pithd1	57095	0.042814659	0.99656
4995	26394	Lypla2	11313	-0.22479388	0.99988
4996	74246	Gale	2582	-1.140493864	0.31453
4997	15356	Hmgcl	3155	0.717072918	0.03896

4998	71665	Fuca1	2517	-0.236381687	0.99988
4999	52830	Pnrc2	55629	0.396798044	0.4134
5000	14105	Srsf10	10772	-0.271822858	0.62665
5001	66743	Rnf220	55182	-0.257851398	0.52811
5002	73804	Kif2c	11004	0.348499072	0.43519
5003	12795	Plk3	1263	0.713362032	0.21716
5004	108067	Eif2b3	8891	-0.701889708	0.3364
5005	22275	Urod	7389	0.943631702	0.24507
5006	68276	Toe1	114034	-0.98867084	0.28437
5007	68394	Ccdc163		0.009154066	0.99989
5008	67096	Mmachc	25974	0.513990498	0.05625
5009	18477	Prdx1	5052	0.484107582	0.02527
5010	58810	Akr1a1	10327	0.03180873	0.99988
5011	50927	Nasp	4678	-0.040080331	0.99988
5012	16351	Ipp	3652	0.557278564	0.06272
5013	18710	Pik3r3		-0.55354028	0.40494
5014	66805	Tspan1	10103	-0.019862361	0.99988
5015	68273	Pomgnt1	55624	-0.917647582	0.33055
5016	68075	Lurap1	541468	-0.584456271	0.58339
5017	19366	Rad54l	8438	-1.182762558	0.32449
5018	72181	Nsun4		-0.146898238	0.98861
5019	17346	Mknk1	8569	1.053312809	0.00802
5020	100465	Mob3c	148932	0.262031587	0.24166
5021	230649	Atpaf1	64756	0.642049666	0.63322
5022	13120	Cyp4b1	1580	1.479954633	0.5916
5023	67182	Pdzk1ip1	10158	-0.538167816	0.40734
5024	66588	Cmpk1	51727	0.607132993	0.04621
5025	69072	Ebna1bp2	10969	0.511757261	0.28745
5026	212647	Aldh4a1	8659	-0.316537807	0.52155
5027	69902	Mrto4	51154	-0.02440571	0.99988
5028	110198	Akr7a5		-0.340841847	0.67652
5029	212555	Slc66a1	54896	0.390751048	0.73975
5030	12345	Capzb	832	-0.189089515	0.99988
5031	72269	Cda	978	0.140241143	0.99988
5032	68943	Pink1	65018	0.246288014	0.7031
5033	13200	Ddost	1650	0.390313669	0.60126
5034	15441	Hp1bp3	50809	0.092401423	0.87044
5035	230861	Eif4g3	8672	-0.103120086	0.91157
5036	15530	Hspg2	3339	-0.346877963	0.96991
5037	19248	Ptpn12	5782	-0.351654577	0.67032
5038	619605	Zcchc17	51538	-0.241571355	0.86487
5039	94242	Tinagl1	64129	-0.242097449	0.66895
5040	67898	Pef1	553115	0.767440671	0.30935
5041	20348	Sema3c	10512	-0.559948552	0.55409
5042	66260	Tmem54	113452	0.814725843	0.26828
5043	19244	Ptp4a2	8073	-0.753374635	0.27327
5044	242669	Azin2	113451	0.240340721	0.90408
5045	20218	Khdrbs1	10657	-0.336169124	0.72801
5046	11637	Ak2	204	-0.473714275	0.41496
5047	75234	Rnf19b	127544	0.234726179	0.98157

5048	66264	Ccdc28b	79140	0.393078883	0.05453
5049	54383	Phc2	1912	-0.600032235	0.47275
5050	76799	Tmem234	56063	-0.566694108	0.40037
5051	54709	Eif3i	8668	-0.011807527	0.99988
5052	230761	Zfp362	149076	-0.184261725	0.8596
5053	433759	Hdac1	3065	0.04202335	0.99988
5054	74552	Nipal3	57185	0.899637885	0.23077
5055	73680	Zbtb8a	653121	-1.237974089	0.20334
5056	51796	Srrm1	10250	-0.750180792	0.46908
5057	107271	Yars	8565	-0.06608925	0.99988
5058	71514	Sfpq	6421	0.219308306	0.11834
5059	68592	Syf2	25949	0.113531842	0.8365
5060	71817	Tmem50a	23585	-0.518253854	0.35351
5061	66146	Maco1	55219	-0.798637814	0.28465
5062	100317	AU040320	79932	-0.499578418	0.58036
5063	16765	Stmn1	3925	-0.584074187	0.40517
5064	26562	Ncdn	23154	-0.417710696	0.60111
5065	26445	Psmb2	5690	0.065758605	0.96363
5066	68040	Zfp593	51042	0.376076332	0.36977
5067	194231	Cnksr1	10256	-1.044277731	0.22875
5068	214150	Ago3	192669	0.829342664	0.43337
5069	73723	Sh3bgrl3	83442	-0.006757537	0.99988
5070	27096	Trappc3	27095	0.111947765	0.94569
5071	100210	Gpn2	54707	-0.705730324	0.32127
5072	245877	Map7d1	55700	0.277199584	0.65122
5073	242691	Gpatch3	63906	0.080522017	0.60397
5074	18221	Nudc	10726	0.067099176	0.9542
5075	20544	Slc9a1	6548	-0.272692581	0.80448
5076	52174	Tmem222	84065	-0.538590931	0.38784
5077	12986	Csf3r	1441	1.433322465	0.00664
5078	269589	Sytl1	84958	-0.2308269	0.97675
5079	66407	Mrps15	64960	0.64950173	0.69261
5080	53608	Map3k6	9064	-0.192517488	0.9198
5081	70088	Meaf6	64769	0.743528683	0.01427
5082	15234	Hgf	3082	0.199047243	0.96072
5083	242687	Wasf2	10163	0.125755337	0.99988
5084	230737	Gnl2	29889	-0.490292661	0.41064
5085	192199	Rspo1	284654	1.940257248	0.00646
5086	52276	Cdca8	55143	-0.597747434	0.42115
5087	14191	Fgr	2268	1.915275899	0.00566
5088	230789	Fam76a	199870	-0.365064505	0.82186
5089	100226	Stx12	23673	0.047105215	0.99988
5090	100336	Ppp1r8	5511	0.210858376	0.99656
5091	20346	Sema3a	10371	0.055499064	0.76462
5092	19891	Rpa2	6118	-1.465924306	0.13804
5093	100340	Smpdl3b	27293	-0.33242748	0.99988
5094	14050	Eya3	2140	0.377728713	0.68572
5095	230734	Yrdc	79693	0.23662655	0.93937
5096	17764	Mtf1	4520	-0.012927682	0.99988
5097	230784	Sesn2	83667	1.546900553	0.0833

5098	16330	Inpp5b	3633	-0.164861068	0.89608
5099	100088	Rcc1	1104	-0.52240043	0.46022
5100	71787	Trnau1ap	54952	0.180076263	0.99988
5101	66464	Taf12	6883	0.377438205	0.31884
5102	56809	Gmeb1	10691	0.518896812	0.505
5103	75062	Sf3a3	10946	0.226808522	0.74709
5104	269587	Epb41	2035	-1.040538449	0.18609
5105	67205	Utp11	51118	-0.332345108	0.78946
5106	19273	Ptpru	10076	0.779658248	0.36549
5107	26922	Mecr	51102	0.439582313	0.33239
5108	57317	Srsf4	6429	-0.889696187	0.04242
5109	12371	Casp9	842	1.102557887	0.00844
5110	69582	Plekhm2	23207	0.00483073	0.99989
5111	213649	Arhgef19	128272	0.154355407	0.96072
5112	213499	Fbxo42	54455	-0.099206597	0.99988
5113	66147	Necap2	55707	-0.43227745	0.8147
5114	18647	Cdk14	5218	0.077168771	0.91311
5115	18600	Padi2	11240	0.107995963	0.991
5116	19181	Psmc2	5701	0.106112254	0.8433
5117	57434	Xrcc2	7516	0.923652471	0.22187
5118	19934	Rpl22	6146	-0.002669751	0.99988
5119	70025	Acot7	11332	1.414680111	0.00773
5120	108099	Prkag2	51422	-0.38640519	0.6324
5121	19744	Rheb	6009	0.424142787	0.10631
5122	74035	Nol9	79707	0.678094578	0.02169
5123	66993	Smarcd3	6604	0.685614728	0.32184
5124	100090	Zbtb48	3104	-0.220120793	0.8881
5125	27407	Abcf2		0.29867774	0.57909
5126	53312	Nub1	51667	-0.042439325	0.99988
5127	22319	Vamp3	9341	0.276851062	0.04194
5128	18628	Per3	8863	1.536987357	0.00664
5129	64295	Tmub1	83590	0.300107774	0.85621
5130	66587	Fastk	10922	-0.069539299	0.99169
5131	63958	Ube4b	10277	0.269008379	0.95965
5132	110208	Pgd	5226	-0.344097698	0.56417
5133	20535	Slc4a2	6522	0.28533494	0.31228
5134	57320	Park7	11315	-0.027949995	0.99988
5135	21942	Tnfrsf9	3604	1.426487289	0.0224
5136	74155	Errfi1	54206	-0.285280694	0.97599
5137	12568	Cdk5	1020	-0.733554761	0.49271
5138	18669	Abcb1b		-0.072900624	0.99814
5139	74610	Abcb8	11194	0.263768479	0.99988
5140	13347	Dffa	1676	0.293064435	0.70173
5141	56273	Pex14	5195	-0.037328788	0.93113
5142	69743	Casz1		-0.942542034	0.19858
5143	18127	Nos3	4846	-0.563394378	0.36223
5144	100198	H6pd	9563	1.756214508	0.01497
5145	70556	Slc25a33	84275	-1.039187941	0.23756
5146	67087	Ctnnbip1	56998	0.388413427	0.60705
5147	654812	Angptl7	10218	2.694893794	0.02893

5148	69151	Lzic	84328	0.221073716	0.84633
5149	56717	Mtor	2475	0.726682283	0.016
5150	66454	Nmnat1	64802	0.724557847	0.24897
5151	84652	Fam126a	84668	-0.431679036	0.72577
5152	66169	Tomm7	54543	0.669676514	0.02014
5153	72772	Rint1	60561	0.728975322	0.0304
5154	230903	Fbxo44	93611	-0.377229513	0.9343
5155	71890	Mad2l2	10459	-0.168381706	0.99988
5156	69188	Kmt2e	55904	-0.830124016	0.27121
5157	11610	Agtrap	57085	0.48763732	0.24914
5158	17769	Mthfr	4524	1.163567407	0.20884
5159	26429	Orc5	5001	-0.401096534	0.98484
5160	22791	Dnajc2	27000	-0.282640337	0.68662
5161	26372	Clcn6	1185	-0.945036291	0.28284
5162	73078	Pmpcb	9512	0.332128644	0.04693
5163	170731	Mfn2	9927	-0.032776194	0.99988
5164	28010	Miip	60672	0.512412683	0.49564
5165	13368	Dffb	1677	-0.574950751	0.37588
5166	72946	Lrrc47	57470	0.20382009	0.991
5167	59002	Wrap73	49856	0.314018133	0.63752
5168	67808	Tprgl	127262	0.283202896	0.61661
5169	230972	Arhgef16	27237	-0.617455089	0.85956
5170	140500	Acap3	116983	0.285234544	0.78781
5171	71957	Ints11	54973	-1.189852108	0.15529
5172	108888	Atad3a	55210	-0.806530427	0.4834
5173	68991	Ssu72	29101	0.33846607	0.05185
5174	668173	Pex10	5192	0.151126476	0.89815
5175	67830	Rer1	11079	0.37306579	0.04097
5176	20481	Ski	6497	-0.113870731	0.99053
5177	18762	Prkcz	5590	-0.885699615	0.28547
5178	269615	Plch2	9651	-0.270686764	0.73217
5179	269614	Pank4	55229	0.780677092	0.01701
5180	66469	Prxl2b	127281	1.010063065	0.01085
5181	76580	Mib2	142678	0.992814001	0.15686
5182	26561	Mmp23	8510	1.124366925	0.01234
5183	12537	Cdk11b		-1.046966325	0.15758
5184	192185	Nadk	65220	0.021015539	0.98653
5185	14688	Gnb1	2782	-0.07441889	0.96358
5186	66448	Mrpl20	55052	0.421150745	0.02454
5187	56036	Ccnl2	81669	-0.872746998	0.26071
5188	74761	Mxra8	54587	0.649720258	0.08603
5189	13542	Dvl1	1855	0.231342897	0.85523
5190	79554	Cptp	80772	-0.648750198	0.37775
5191	22163	Tnfrsf4	7293	1.432606801	0.00621
5192	20318	Sdf4	51150	0.108895674	0.58979
5193	12182	Bst1	683	1.314280608	0.00903
5194	12494	Cd38	952	0.538523702	0.23048
5195	19126	Prom1	8842	0.936838281	0.03879
5196	66768	730480H06R	133015	0.770471106	0.23178
5197	70693	Adgra3	166647	0.587726759	0.95128

5198	81840	Sorcs2	57537	1.146798824	0.01162
5199	70292	Afap1	60312	-1.137682754	0.22629
5200	78558	Htra3	94031	0.513571322	0.71701
5201	78890	Trmt44	152992	-0.252570365	0.68497
5202	80911	Acox3	8310	-0.565407907	0.49431
5203	71729	Rgs12	6002	-1.043419319	0.22098
5204	54426	Hgfac	3083	1.312613482	0.05029
5205	16976	Lrpap1	4043	0.042496821	0.99988
5206	15194	Htt	3064	-0.407366721	0.40878
5207	11518	Add1	118	0.340659269	0.4567
5208	54216	Pcdh7	5099	0.521323745	0.54208
5209	19822	Rnf4	6047	0.323842995	0.90326
5210	24116	Nelfa	7469	0.514584745	0.0303
5211	59056	Evc	2121	-0.379023383	0.93198
5212	71116	Stx18	53407	0.533253712	0.03031
5213	18196	Nsg1	27065	1.74934598	0.01973
5214	75079	Zbtb49	166793	-0.233284192	0.96268
5215	100972	Rab28	9364	0.393410844	0.30315
5216	56874	Rnf32	140545	-0.910361755	0.25934
5217	23950	Dnajb6	10049	0.13954263	0.92973
5218	665270	Plb1	151056	-0.478490817	0.86505
5219	14284	Fosl2	2355	0.880970044	0.1198
5220	20534	Slc4a1ap	22950	-0.861313432	0.3982
5221	13667	Eif2b4	8890	-0.297004376	0.65044
5222	266781	Snx17	9784	-0.747059229	0.31377
5223	14208	Ppm1g	5496	0.120915166	0.99988
5224	192292	Nrbp1	29959	-0.656073217	0.3341
5225	69815	Krtcap3	200634	0.335484178	0.35993
5226	68095	Ociad1	54940	0.001947238	0.99989
5227	433904	Ociad2	132299	1.560051159	0.00726
5228	24051	Sgcb	6443	1.541281949	0.00721
5229	68567	Cgref1	10669	-0.390711683	0.52171
5230	16548	Khk	3795	-0.691023028	0.3352
5231	100952	Emilin1	11117	0.656519884	0.7835
5232	231093	Agbl5	60509	-0.077998916	0.99988
5233	19017	Ppargc1a	10891	-0.153146382	0.99988
5234	13204	Dhx15	1665	-0.867413403	0.21575
5235	66681	Pgm2	55276	-0.729275273	0.67629
5236	211006	Sepsecs	51091	0.710678379	0.23493
5237	57915	Tbc1d1	23216	0.931420594	0.031
5238	74919	Slc35f6	54978	-0.100977558	0.99988
5239	52206	Anapc4	29945	0.435048317	0.04242
5240	12615	Cenpa	1058	-0.582205446	0.74842
5241	16599	Klf3	51274	-0.071188577	0.98116
5242	78796	Zcchc4	29063	-0.211032907	0.75897
5243	68303	Fam114a1	92689	0.355225694	0.04992
5244	67073	Pi4k2b	55300	-0.465835564	0.74088
5245	20531	Slc34a2	10568	0.837569191	0.01089
5246	231238	Sel1l3	23231	0.270736821	0.82926
5247	320661	D5Ertd579e	9778	-0.769741092	0.23856

5248	19687	Rfc1	5981	-0.362024005	0.65328
5249	100855	Tbc1d14	57533	0.728689306	0.02032
5250	231151	Tada2b	93624	-1.313610671	0.20582
5251	17713	Grpel1	80273	-0.112985438	0.99988
5252	79464	Lias	11019	-0.38995585	0.51351
5253	22235	Ugdh	7358	-0.439464136	0.68016
5254	71521	Pds5a	23244	-0.723823981	0.26183
5255	53323	Ube2k	3093	0.035598388	0.99988
5256	74734	Rhoh	399	0.363194919	0.36647
5257	11787	Apbb2	323	-0.595683155	0.32867
5258	231279	Guf1	60558	0.254348686	0.88928
5259	67980	Gnpda2	132789	-1.417725901	0.19107
5260	14400	Gabrb1	2560	2.389553972	0.00817
5261	27784	Commd8	54951	0.324634399	0.57458
5262	21682	Tec	7006	-0.686408757	0.40878
5263	109108	Slc30a9	10463	-0.835848782	0.28283
5264	22223	Uchl1	7345	0.549448246	0.27937
5265	66899	Fip1l1		0.294610237	0.44852
5266	74277	Chic2	26511	0.459225326	0.02026
5267	18595	Pdgfra	5156	0.710209636	0.85525
5268	57357	Srd5a3		0.065636178	0.98653
5269	21982	Tmem165	55858	0.348398276	0.73022
5270	12753	Clock	9575	-0.577806447	0.38023
5271	231327	Ppat	5471	1.061846765	0.01687
5272	67054	Paics	10606	0.080938993	0.91147
5273	19712	Rest	5978	-1.226887032	0.21978
5274	231329	Polr2b	5431	0.191276537	0.89899
5275	12617	Cenpc1	1060	-0.697210239	0.65393
5276	56792	Stap1	26228	0.220385575	0.67178
5277	100727	Ugt2b34		0.184073464	0.78102
5278	433931	Pigg	54872	-0.070579348	0.99988
5279	13486	Dr1	1810	1.465191851	0.01054
5280	17765	Mtf2	22823	-0.935497287	0.46208
5281	67266	Dipk1a	388650	0.052586954	0.99988
5282	20860	Sult1e1		1.090615517	0.73015
5283	14581	Gfi1	2672	0.068001652	0.45503
5284	170823	Glmn	11146	-1.159524944	0.21432
5285	114642	Brdt	676	0.398043461	0.86126
5286	12545	Cdc7	8317	1.00459928	0.01803
5287	21814	Tgfbr3	7049	0.580701289	0.91809
5288	54367	Zfp326	284695	-0.596541343	0.3801
5289	52822	Rufy3	22902	-0.305330061	0.75897
5290	236573	Gbp9		0.949704607	0.8541
5291	27405	Abcg3	9429	0.55408731	0.24834
5292	20750	Spp1	6696	-0.868645085	0.33528
5293	13602	Sparcl1	8404	0.690183472	0.45039
5294	74167	Nudt9	53343	-0.305378538	0.97856
5295	114664	Hsd17b11	51170	0.712030364	0.01748
5296	246293	Klhl8	57563	0.597024425	0.44695
5297	17355	Aff1	4299	0.058591057	0.98653

5298	231510	Gpat3	84803	0.780677092	0.02224
5299	71883	Coq2	27235	-0.172918372	0.99988
5300	75750	Slc10a6	345274	-0.318112588	0.99988
5301	231507	Plac8	51316	1.204524811	0.08511
5302	67870	Enoph1	58478	-0.314251396	0.99988
5303	50926	Hnrnpdl	9987	-0.321637618	0.73186
5304	74596	Cds1	1040	-0.0586648	0.99988
5305	19092	Prkg2	5593	-0.582343275	0.71241
5306	71914	Antxr2	118429	0.487043391	0.69463
5307	22022	Tpst2	8459	0.622360223	0.78102
5308	54723	Tfip11	24144	0.160002187	0.99988
5309	70118	Srrd	402055	0.328104491	0.97977
5310	12962	Crybb3	1417	-0.88815674	0.6493
5311	57816	Tesc	54997	0.642518657	0.05631
5312		Gm9754		-0.465860974	0.58965
5313	72151	Rfc5	5985	0.357851457	0.86915
5314	59043	Wsb2	55884	0.945000087	0.45862
5315	13178	Dck	1633	0.65945519	0.18164
5316	73246	Rassf6	166824	-0.973132717	0.14167
5317	20311	Cxcl5	6372	-0.943380921	0.34069
5318	57349	Ppbp	5473	0.395385742	0.61803
5319	56744	Pf4		1.361414936	0.00926
5320	20309	Cxcl15		-0.900318088	0.24307
5321	665563	Mthfd2l	441024	-0.08163943	0.99988
5322	11839	Areg	374	-0.00291605	0.99988
5323	330122	Cxcl3		-0.632620112	0.81665
5324	14825	Cxcl1		-1.055991641	0.30833
5325	27428	Shroom3	57619	-0.532700355	0.5772
5326	12452	Ccng2	901	0.109409844	0.99988
5327		NA		0.260684355	0.78756
5328	209357	Gtf2h3	2967	0.251021883	0.99988
5329	209354	Eif2b1	1967	-0.737014397	0.39038
5330	67848	Ddx55	57696	-1.020872887	0.26063
5331	56334	Tmed2	10959	-0.434053306	0.50197
5332	75695	Rilpl1	353116	0.536659161	0.38009
5333	13445	Cdk2ap1		-0.404358775	0.55816
5334	68098	Rchy1	25898	-0.059727062	0.99988
5335	80291	Rilpl2	196383	0.299713185	0.33273
5336	76167	Snrnp35	11066	-1.17774798	0.27649
5337	53886	Cdkl2	8999	-0.322089263	0.99024
5338	65105	Arl6ip4	51329	-0.724416891	0.12463
5339	23881	G3bp2	9908	-0.265850559	0.60159
5340	56041	Uso1	8615	0.443521593	0.19107
5341	67111	Naaa	27163	1.949726145	0.00407
5342	208628	Kntc1	9735	1.791095321	0.00438
5343	231452	Sdad1	55153	-1.015132233	0.20134
5344	100561	Slc15a4	121260	0.631217028	0.36594
5345	17329	Cxcl9	4283	1.775920968	0.02078
5346	208606	Rsrc2	65117	-0.028415532	0.99169
5347	12492	Scarb2	950	0.894067485	0.00901

5348	70650	Zcchc8	55596	-0.314762558	0.86725
5349	13852	Stx2	2054	0.386914657	0.11154
5350	19384	Ran		0.274090852	0.05619
5351	231727	B3gnt4	79369	1.230870931	0.06417
5352	14467	Nipsnap2	2631	0.170326274	0.81916
5353	66593	Diablo	56616	0.147587957	0.10447
5354	77573	Vps33a		-0.079118597	0.71694
5355	77045	Bcl7a	605	-0.203404585	0.65139
5356	231769	Sfswap	6433	-0.285459579	0.98477
5357	67151	Psmd9		-0.717596901	0.50185
5358	100678	Psph	5723	0.291239724	0.98575
5359	12466	Cct6a	908	0.114675114	0.97285
5360	17165	Mapkapk5		0.500946158	0.32941
5361	11669	Aldh2	217	-0.337549041	0.60824
5362	72399	Brap	8315	-0.014793946	0.96075
5363	319604	Fam168a	23201	0.44425034	0.48143
5364	56433	Vps29	51699	0.56316532	0.01882
5365	68080	Gpn3	51184		0.991
5366	56378	Arpc3	10094	1.224967322	0.00664
5367	56317	Anapc7	51434	-0.855931991	0.28284
5368	11938	Atp2a2	488	0.619343421	0.01578
5369	18439	P2rx7	5027	1.409899619	0.02279
5370	12589	Ift81	28981	0.071041852	0.99639
5371	18438	P2rx4	5025	0.637783513	0.02215
5372	207565	Camkk2	10645	-0.070121928	0.99988
5373	59008	Anapc5	51433	-0.154884545	0.81099
5374	80751	Rnf34	80196	0.222865419	0.07119
5375	30841	Kdm2b	84678	0.459654103	0.53516
5376	20602	Ncor2	9612	-0.801463378	0.26659
5377	78894	Aacs	65985	-0.504904251	0.52015
5378	11745	Anxa3	306	-0.644299481	0.191
5379	94061	Mrpl1	65008	-0.371465961	0.68231
5380	243197	Mfsd7a	84179	0.504176759	0.63731
5381	19301	Pxmp2	5827	0.730632534	0.29762
5382	72542	Pgam5	192111	0.073588637	0.99988
5383	71782	Ankle2	23141	-0.040412652	0.99988
5384	269682	Golga3	2802	-0.474750299	0.65013
5385	231602	P2rx2	22953	1.086293354	0.72874
5386	69663	Ddx51	317781	0.883599203	0.01531
5387	75560	Ep400	57634	-0.373810062	0.5185
5388	56361	Pus1	80324	0.305063106	0.90211
5389	22241	Ulk1	8408	1.844763143	0.00645
5390	19079	Prkab1	5564	-0.282169479	0.52149
5391	12704	Cit	11113	-0.670798641	0.58763
5392	77407	Rab35	11021	0.690362869	0.01602
5393	50883	Chek2	11200	-0.104547199	0.94563
5394	75387	Sirt4	23409		0.40878
5395	19303	Pxn	5829	-0.284555088	0.96403
5396	64213	St7		-0.627251357	0.59996
5397	69076	Triap1	51499	-0.204147404	0.83839

5398	384281	Gatc		-0.651507856	0.59594
5399	108014	Srsf9	8683	0.261710701	0.87597
5400	11409	Acads	35	-0.585139634	0.51559
5401	68510	Ints1	26173	-0.812924287	0.26781
5402	74585	Sppl3	121665	-0.900641418	0.22435
5403	66506	Psmg3	84262	-0.317769538	0.7398
5404	21753	Tes		-0.121756298	0.75132
5405	68017	Mrm2	29960	0.406069108	0.9848
5406	72357	210016L21Ri	64897	-0.722119233	0.23024
5407	231834	Snx8	29886	0.209801496	0.99267
5408	23962	Oasl2		-0.400462356	0.46995
5409	114142	Foxp2	93986	1.10917617	0.19626
5410	101118	Tmem168	64418	0.377670949	0.38173
5411	16848	Lfng	3955		0.40878
5412	71900	Tmem106b	54664	0.325354215	0.05453
5413	77697	Mmab	326625	0.064929353	0.99768
5414	231858	Radil	55698	0.077292811	0.89003
5415	117146	Ube3b	89910	-0.104827079	0.83725
5416	74781	Wipi2	26100	-0.570409445	0.325
5417	11461	Actb	60	-0.824388302	0.13888
5418	14086	Fscn1	6624	0.990361378	0.24661
5419	231866	Zfp12	7559	0.065743028	0.99988
5420	22256	Ung	7374	-0.100710278	0.97675
5421	100756	Usp30	84749	-0.072569563	0.97599
5422	74111	Rbm19	9904	1.082906417	0.26012
5423	71772	Plbd2	196463	-0.128739201	0.82658
5424	71990	Ddx54	79039	-0.247176842	0.48182
5425	19415	Rasal1	8437	-0.795342943	0.2028
5426	14357	Dtx1	1840	0.32252303	0.9927
5427	23961	Oas1b		-0.585525571	0.3211
5428	231872	Aimp2	7965	0.006267341	0.99988
5429	15467	Eif2ak1	27102	-1.142164261	0.19034
5430	19988	Rpl6	6128	0.278437651	0.07878
5431	67397	Erp29	10961	0.459923794	0.02192
5432	231874	Ccz1		-0.680069624	0.35773
5433	56443	Arpc1a		0.325463772	0.07132
5434	11867	Arpc1b	10095	0.186337583	0.98506
5435	231887	Pdap1	11333	-0.612045964	0.47631
5436	71799	Ptcd1		-0.251370465	0.99779
5437	54188	Cpsf4	10898	-0.157406504	0.68689
5438	67235	Zkscan14	84124	0.714762203	0.3987
5439	75725	Phf14	9678	-0.252752798	0.64698
5440	17992	Ndufa4	4697	0.119074878	0.84471
5441	74132	Rnf6	6049	-0.449478711	0.45387
5442	264064	Cdk8	1024	0.01616652	0.99988
5443	245880	Wasf3	10810	1.038936248	0.0111
5444	170772	Glcci1	113263	-0.147027639	0.8833
5445	22217	Usp12	219333	0.480339597	0.0399
5446	20018	Polr1d	51082	0.058196643	0.99988
5447	72587	Pan3	255967	-0.18763159	0.72923

5448	14254	Flt1	2321	0.661890841	0.41529
5449	66537	Pomp	51371	-0.407433676	0.24865
5450	71706	Slc46a3	283537	-0.17833161	0.99988
5451	77521	Mtus2	23281	-1.174671949	0.53617
5452	381695	N4bp2l2	10443	0.114653016	0.99988
5453	15505	Hsph1	10808	1.044896836	0.0189
5454	70717	Medag	84935	0.977109852	0.12043
5455	12843	Col1a2	1278	0.397039644	0.85388
5456	269831	Tspan12	23554	-0.026067266	0.99988
5457	71777	Ing3	54556	-1.034196051	0.15297
5458	27999	Fam3c	10447	-0.332300798	0.75157
5459	319974	Auts2	26053	-0.931535847	0.33886
5460	16885	Limk1	3984	0.489668878	0.02897
5461	13717	Eln	2006	-0.091247449	0.95986
5462	101185	Pot1a		-0.162985668	0.75292
5463	12054	Bcl7b	9275	0.385564513	0.46868
5464	73178	Wasl	8976	-0.245199131	0.52926
5465	26965	Cul1	8454	-0.214990386	0.99988
5466	14056	Ezh2	2146	-1.377939873	0.14023
5467	68272	Rbm28	55131	-0.577953047	0.36281
5468	71735	Lrwd1	222229	0.263914768	0.66793
5469	13047	Cux1	1523	0.251648398	0.43752
5470	74375	Gcc1	79571	-0.205620267	0.97494
5471	13846	Ephb4	2050	-0.586273852	0.31591
5472	14693	Gnb2	2783	-0.369653031	0.45895
5473	57330	Gigyf1	64599	-0.958843392	0.2181
5474	74097	Pop7	10248	0.508362095	0.84774
5475	18542	Pcolce	5118	1.678641541	0.00533
5476		Gm20605		-0.06881955	0.95936
5477	231801	Agfg2	3268	-1.675916096	0.14414
5478	78829	Tsc22d4	81628	-0.866328215	0.27788
5479	231803	Mepce	56257	0.297923148	0.1333
5480	74570	Zkscan1	7586	-0.471341833	0.45694
5481	17220	Mcm7	4176	-1.246715739	0.12771
5482	29807	Tpk1	27010	0.677991944	0.69432
5483	27053	Asns	440	-1.504333422	0.1403
5484	269823	Pon3	5446	0.236657913	0.66322
5485	109624	Cald1	800	0.558231032	0.53734
5486	14187	Akr1b8		-0.215989844	0.991
5487	20336	Exoc4	60412	-0.477313515	0.43994
5488	12321	Calu	813	-0.24918713	0.991
5489	27056	Irf5	3663	1.240426493	0.01281
5490	74340	Ahcyl2	23382	-0.645156023	0.3702
5491	76788	Klhdc10	23008	-1.310045187	0.22712
5492	58875	Hibadh	11112	-0.432523853	0.40878
5493	353172	Gars	2617	0.17410089	0.85226
5494	11517	Adcyap1r1	117	0.194360437	0.85199
5495	107569	Nt5c3	51251	0.274458896	0.05573
5496	27055	Fkbp9	11328	0.289737412	0.71384
5497	72649	Tmem209	84928	0.257930319	0.89847

5498	78937	Avl9	23080	0.336941842	0.66078
5499	83922	Cep41	95681	0.617619658	0.28547
5500	67138	Herc6	55008	-0.506242775	0.60337
5501	26357	Abcg2		-0.261622814	0.99988
5502	73998	Herc3		-0.144167541	0.77635
5503	65963	Tmem176b	28959	-0.60223295	0.66381
5504	75593	Malsu1	115416	0.737267662	0.02443
5505	93695	Gpnmb	10457	0.655921763	0.22364
5506	101214	Tra2a	29896	-0.912846783	0.30443
5507	54722	Gsdme	1687	-0.264643251	0.99988
5508	71720	Osbpl3	26031	-0.991167738	0.22165
5509	192196	Luc7l2	51631	-0.401265281	0.4782
5510	78781	Zc3hav1	56829	-0.208617928	0.8717
5511	18025	Nfe2l3	9603	-0.526371574	0.65381
5512	21848	Trim24	8805	-0.831067737	0.3829
5513	12417	Cbx3		-0.004223122	0.99988
5514	19242	Ptn	5764	0.380526723	0.7594
5515	14489	Mtpn	136319	0.484889194	0.01827
5516	232748	Tcaf2		-0.227660094	0.99988
5517	22793	Zyx	7791	0.441664165	0.47847
5518	12366	Casp2	835	0.354848841	0.04425
5519	76263	Gstk1	373156	0.007824499	0.99988
5520	13848	Ephb6	2051	-0.432736205	0.95528
5521	56150	Mad2l1	4085	-1.197047047	0.10438
5522	381760	Ssbp1	6742	0.503372631	0.05685
5523	70779	Prdm5	11107	-0.485412275	0.48431
5524	23845	Clec5a	23601	1.405689037	0.05481
5525	69923	Agk	55750	-1.157437999	0.45675
5526	14548	Mrps33	51650	0.084794116	0.82477
5527	54486	Hpgds	27306	1.685748245	0.00388
5528	13990	Smarcad1	56916	0.308729403	0.94358
5529	54484	Mkrn1	23608	0.376944926	0.52678
5530	19331	Rab19	401409	-0.060912126	0.99988
5531	72144	Slc37a3		0.465503205	0.91547
5532	21391	Tbxas1	6916	2.378742268	0.00356
5533	14583	Gfpt1	2673	-0.12717123	0.77575
5534	56748	Nfu1	27247	0.561032419	0.01988
5535	11746	Anxa4	307	-0.402845703	0.61112
5536	66881	Pcyox1	51449	-0.200825481	0.93735
5537	21802	Tgfa	7039	-0.613565292	0.17508
5538	72102	Dusp11	8446	-0.050294842	0.99988
5539	12468	Cct7	10574	-0.16041728	0.73063
5540	18139	Zfp638	27332	0.184702824	0.80733
5541	19695	Reg3g		2.358673867	0.00416
5542	101358	Fbxl14	144699	-0.264619033	0.75823
5543	243548	Prickle2	166336	-0.672397421	0.86113
5544	16206	Lrig1	26018	1.745401291	0.00734
5545	243574	Kbtbd8	84541	0.913732249	0.36204
5546	70020	Ino80b		-0.360471468	0.67178
5547	22377	Wbp1	23559	-0.645706768	0.32134

5548	57377	Mogs	7841	0.151738117	0.99988
5549	68499	Mrpl53	116540	-0.130953182	0.91872
5550	66979	Pole4	56655	0.029891203	0.99988
5551	56284	Mrpl19	9801	-0.597081806	0.55415
5552	232201	Arhgap25	9938	1.503326521	0.00837
5553	72103	Aplf	200558	-0.634501483	0.56989
5554	69834	Rab43	339122	1.312792036	0.00892
5555	57905	Isy1		-0.507551954	0.61114
5556	12785	Cnbp	7555	-0.052033833	0.99988
5557	54161	Copg1	22820	-0.359918799	0.57369
5558	232286	Tmf1	7110	-0.406349032	0.57681
5559	232210	Hmces	56941	-0.53334195	0.45158
5560	22200	Uba3	9039	0.122549969	0.99988
5561	103963	Rpn1	6184	0.295844771	0.6841
5562	232288	Frmd4b	23150	0.367579562	0.74269
5563	108655	Foxp1		-0.644577222	0.37078
5564		Gm20696		-2.6883289	0.04139
5565	232313	Gxylt2	727936	-0.004710356	0.99988
5566	12661	Chl1	10752	1.113830864	0.12745
5567	56505	Ruvbl1	8607	0.705156443	0.04757
5568	53421	Sec61a1	29927	0.556393255	0.44852
5569	80283	Abtb1	80325	0.297448151	0.05795
5570	18844	Plxna1	5361	-0.024200388	0.99988
5571	66098	Chchd6	84303	0.232619094	0.44726
5572	66277	Klf15	28999	-1.491087801	0.02722
5573	107747	Aldh1l1	10840	0.106915839	0.99988
5574	71699	Slc41a3	54946	0.521427676	0.52678
5575	54563	Nup210	23225	1.341230846	0.26511
5576	22591	Xpc	7508	-0.253107194	0.76163
5577	74122	Tmem43	79188	0.220876937	0.99988
5578	21366	Slc6a6	6533	0.410788314	0.01409
5579	243547	Grip2	80852	-0.197704765	0.76456
5580	58911	Sumf1	285362	0.73853549	0.07831
5581	16438	Itpr1	3708	0.749952537	0.38196
5582	20893	Bhlhe40	8553	-0.91247279	0.28282
5583	192193	Edem1	9695	0.702206138	0.01788
5584	67166	Arl8b	55207	-0.263243458	0.64168
5585	24110	Usp18		-0.674761906	0.45745
5586	14412	Slc6a13	6540	-0.476288065	0.89638
5587	19713	Ret	5979	0.798638164	0.01298
5588	232345	A2m	2	0.354978647	0.96558
5589	50530	Mfap5	8076	1.473314356	0.10323
5590	14562	Gdf3	9573	0.672897328	0.10039
5591	30853	Mlf2	8079	0.49757034	0.41621
5592	69202	Ptms		-1.265937024	0.22364
5593	67784	Plxnd1	23129	0.174414732	0.98106
5594	330401	Tmcc1	23023	0.197792386	0.52322
5595	26894	Cops7a	50813	-0.496814661	0.68632
5596	70727	Rasgef1a	221002	0.685680293	0.01452
5597	213895	Bms1	9790	0.575147433	0.08457

5598	56619	Clec4e	26253	0.497217949	0.89642
5599	17474	Clec4d	338339	1.622302021	0.24507
5600	72720	Zfp248	57209	0.551797309	0.53572
5601	69810	Clec4b1		1.922675984	0.00407
5602	26888	Clec4a2		3.663293851	0.00356
5603	27007	Klrk1	22914	2.185457394	0.0064
5604	232409	Clec2e		-1.974722688	0.18424
5605	93694	Clec2d		-0.27918638	0.87037
5606	57436	Gabarapl1	23710	0.664735403	0.01803
5607	16643	Klrd1	3824	0.948466981	0.37299
5608	19365	Rad52	5893	0.582519168	0.10082
5609	16641	Klrc1		0.023019492	0.93548
5610	68465	Adipor2	79602	-0.62406569	0.34177
5611	22419	Wnt5b	81029	0.308191721	0.99988
5612	111173	Erc1	23085	-0.136143883	0.99988
5613	67200	Ccdc77	84318	1.034015141	0.02148
5614	214899	Kdm5a	5927	-0.546407943	0.48743
5615	16633	Klra2		1.592659371	0.01002
5616	66441	Magohb	55110	0.641237365	0.41858
5617	56449	Ybx3	8531	-0.830377408	0.3846
5618	14011	Etv6	2120	-0.210778619	0.76092
5619	66813	Bcl2l14	79370	-0.266666153	0.93704
5620	16974	Lrp6	4040	-0.989142855	0.26928
5621	70686	Dusp16	80824	-0.040064304	0.99988
5622	67755	Ddx47	51202	-0.583336821	0.38618
5623	74525	Fam234b	57613	-0.057967269	0.99988
5624	13730	Emp1	2012	1.253473305	0.0108
5625	54343	Atf7ip	55729	0.164669729	0.84336
5626	66857	Plbd1	79887	2.095860759	0.00393
5627	60321	Wbp11		-0.187079848	0.83579
5628	17313	Mgp	4256	1.906904215	0.00621
5629	11857	Arhgdib	397	1.075940788	0.13628
5630	232441	Rerg	85004	-0.44705294	0.72784
5631	19277	Ptpro	5800	2.831629858	0.00388
5632	20901	Strap	11171	0.097686812	0.99988
5633	232449	Dera	51071	-0.932386113	0.28443
5634	109135	Plekha5	54477	0.083880103	0.99988
5635	11569	Aebp2	121536	0.06712886	0.99988
5636	19691	Recql	5965	-0.205359267	0.74992
5637	66964	Golt1b	51026	0.060327709	0.99988
5638	16832	Ldhb		0.846532145	0.28976
5639	16523	Kcnj8	3764	0.128314291	0.99988
5640	20928	Abcc9	10060	0.580779366	0.5749
5641	58186	Rad18	56852	-0.469468044	0.41147
5642	16651	Sspn	8082	0.771626855	0.0435
5643	79362	Bhlhe41	79365	0.03770199	0.98675
5644	259302	Srgap3	9901	0.710859987	0.84633
5645	71323	Rassf8	11228	0.464717259	0.95309
5646	16970	Lrmp	4033	1.026634229	0.22629
5647	14911	Thumpd3	25917	0.32857623	0.99988

5648	16653	Kras	3845	-0.757729464	0.29038
5649	12035	Bcat1	586	-0.720604293	0.58253
5650	97287	Mtmr14	64419	-0.541816134	0.40234
5651	18294	Ogg1	4968	0.369690439	0.18756
5652	52163	Camk1	8536	0.606236439	0.01671
5653	75320	Etnk1	55500	0.984688736	0.01452
5654	101100	Ttll3		-2.122972408	0.03115
5655	74741	C2cd5	9847	0.398501856	0.73635
5656	171095	Il17rc		-0.777334331	0.40878
5657	12764	Cmas	55907	-0.765651208	0.33752
5658	171508	Creld1	78987	-0.353934053	0.67222
5659	66087	Emc3	55831	-0.259597065	0.94507
5660	16439	Itpr2	3709	-0.663800103	0.32908
5661	108098	Med21	9412	-0.546204463	0.37111
5662	110379	Sec13	6396	0.506896248	0.26783
5663	67015	Ccdc91	55297	0.025149883	0.99988
5664	330450	Far2	55711	-0.690895046	0.30475
5665	67456	Ergic2	51290	-0.638847622	0.40798
5666	387314	Tmtc1	83857	1.629747752	0.08542
5667	232560	Caprin2	65981	-0.788211627	0.40878
5668	320560	Dennd5b	160518	1.308311626	0.00934
5669	74244	Atg7	10533	-0.668678121	0.40308
5670	232334	Vgll4	9686	-1.75129554	0.13783
5671	68971	Tamm41	132001	0.716354651	0.03336
5672	110595	Timp4	7079	0.0421731	0.99988
5673	67088	Cand2	23066	-1.474556303	0.178
5674	17193	Mbd4	8930	-0.494475729	0.46054
5675	81896	Ift122	55764	1.044012789	0.10435
5676	17059	Klrb1c			0.40878
5677	67602	Necap1	25977	1.038619652	0.80173
5678	28019	Ing4	51147	-0.027823629	0.99988
5679	66493	Mrpl51	51258	-0.225718299	0.64146
5680	22317	Vamp1	6843		0.40878
5681	17000	Ltbr	4055	-0.621463802	0.34135
5682	20276	Scnn1a	6337	0.415468023	0.71755
5683	21937	Tnfrsf1a	7132	0.680971104	0.02051
5684	12527	Cd9	928	0.124873952	0.92694
5685	19362	Rad51ap1	10635	1.159670221	0.07392
5686	28040	D6Wsu163e	57102	0.234957642	0.99024
5687	68498	Tspan11	441631	1.254861069	0.12498
5688	109246	Tspan9	10867	-0.413640024	0.49408
5689	21679	Tead4	7004	-1.441008748	0.40577
5690	14228	Fkbp4	2288	-0.361620074	0.7059
5691	93675	Clec2i			0.991
5692	97387	Strn4	29888	-1.270571653	0.28553
5693	109889	Mzf1	7593	1.231013239	0.13413
5694	67370	Zfp606	80095	-0.006051589	0.99988
5695	232944	Mark4	57787	0.78203987	0.06272
5696	13871	Ercc2	2068	-0.564501838	0.37567
5697	20167	Rtn2	6253	1.256953771	0.0096

5698	22323	Vasp	7408	-0.324601007	0.66812
5699	67369	Qpctl	54814	-1.352745924	0.19953
5700	13400	Dmpk		0.682862933	0.40345
5701	13401	Dmwd	1762	0.250187557	0.7797
5702	21946	Pglyrp1	8993	0.326993539	0.3327
5703	56330	Pdcd5	9141	0.297079182	0.02518
5704	19777	Uri1	8725	-1.097462346	0.21881
5705	66161	Pop4	10775	-0.035452123	0.94955
5706	18726	Lilra6			0.40878
5707	57776	Ttyh1		0.039168108	0.99988
5708	664968	Tmem238	388564	-0.266694675	0.63993
5709	19943	Rpl28	6158	-0.237565903	0.667
5710	22185	U2af2	11338	-0.338601438	0.96958
5711	212569	Zfp273		0.851063362	0.36005
5712	20430	Cyfip1	23191	0.274973243	0.87476
5713	15204	Herc2	8924	-0.306092275	0.89518
5714	93790	Nipa2	81614	-0.806970365	0.37299
5715	234353	Psd3		-0.048173588	0.92099
5716	243958	Siglecg		2.155985061	0.00576
5717	210105	Zfp719		-0.460583587	0.89659
5718	243983	Zdhhc13	54503	0.796992479	0.75914
5719	83382	Siglece			0.991
5720	101831	Faap24	91442	-0.225962779	0.61866
5721	52428	Rhpn2	85415	-0.422336006	0.45316
5722	71974	Prmt3	10196	0.797777904	0.02564
5723	117589	Asb7	140460	0.125132007	0.74736
5724	545975	Cers3	204219	-0.371050361	0.77929
5725	68981	Snrpa1	6627	-0.113706911	0.8717
5726	18553	Pcsk6	5046	-1.442642456	0.22364
5727	272396	Tarsl2	123283	-1.145294226	0.19222
5728	21872	Tjp1	7082	-0.517664241	0.72018
5729	67973	Mphosph10	10199	-0.974946556	0.2006
5730	233315	Mtmr10	54893	-0.06536985	0.88959
5731	70461	Crtc3	64784	-0.324800874	0.54314
5732	12144	Blm	641	-0.034316519	0.99988
5733	18550	Furin	5045	0.126875418	0.95936
5734	68695	Hddc3	374659	0.560658232	0.42604
5735	101869	Unc45a	55898	-1.302185671	0.19679
5736	233405	Vps33b		0.0750195	0.99988
5737	29875	Iqgap1	8826	-0.582933211	0.31643
5738	23991	Cib1	10519	-0.836133656	0.30673
5739	20352	Sema4b	10509	0.403950345	0.97941
5740	269951	Idh2	3418	1.033830205	0.10385
5741	18631	Pex11a	8800	-0.85065505	0.46763
5742	11819	Nr2f2	7026	-0.819904238	0.26659
5743	233335	Synm	23336	1.278578704	0.00988
5744	67009	Ttc23	64927	-0.076689268	0.99988
5745	67867	Lrrc28	123355	0.273361079	0.99988
5746	17258	Mef2a	4205	0.380579654	0.90018
5747	72433	Rab38	23682	-1.358259584	0.2083

5748	13032	Ctsc	1075	2.189892573	0.00407
5749	50490	Nox4	50507	2.143230869	0.00388
5750	22177	Tyrobp	7305	1.619849857	0.01363
5751	74206	Sipa1l3	23094	-0.03957877	0.99988
5752	72275	200002D01Rik		-0.233672935	0.59187
5753	77254	Yif1b	90522	-0.496856131	0.59437
5754	233046	Rasgrp4	115727	-0.215740323	0.81476
5755	73833	Fam98c	147965	0.650297327	0.31536
5756	57296	Psmd8	5714	-0.12871023	0.76197
5757	18036	Nfkbib		0.14975972	0.84592
5758	50760	Fbxo17		1.385161149	0.13983
5759	70584	Pak4		-0.601900554	0.37705
5760	23996	Psmc4	5704	-0.104609072	0.88959
5761	71163	Zfp626		0.141485983	0.99988
5762	17304	Mfge8	4240	-0.642695856	0.2856
5763	68048	Aen	64782	-0.685266994	0.28615
5764	76375	Det1		0.631414485	0.51939
5765	67994	Mrps11	64963	-0.185490565	0.7594
5766	67308	Mrpl46	26589	0.031392972	0.99988
5767	66365	Ccdc90b	60492	0.680014797	0.02313
5768	68472	Tmem126b	55863	0.31143266	0.98562
5769	66271	Tmem126a	84233	0.446858836	0.11447
5770	83671	Sytl2	54843	-0.542176978	0.7324
5771	13626	Eed	8726	0.429548617	0.07583
5772	109264	Me3	10873	-1.512266302	0.06845
5773	65098	Zfand6	54469	-0.503717308	0.27013
5774	14085	Fah	2184	-1.624135699	0.15672
5775	75985	Rab30	27314	0.927848326	0.38499
5776	68197	Ndufc2		0.499130104	0.11645
5777	75430	Anapc15		-0.703329815	0.39043
5778	74424	Tmc5	79838	-0.438253755	0.56529
5779	12850	Coq7	10229	-0.251137487	0.99988
5780	54208	Arl6ip1	23204	-0.42700495	0.49893
5781	233789	Smg1	23049	0.507441332	0.29273
5782	53322	Nucb2	4925	-0.306300077	0.95747
5783	18704	Pik3c2a	5286	-1.347287548	0.2021
5784	70572	Ipo5	3843	-0.247768453	0.61578
5785	56372	110004F10Ri	10944	-0.7816473	0.49814
5786	116903	Calcb	797	1.207979604	0.27144
5787	18576	Pde3b	5140	0.202055936	0.07845
5788	17907	Mylpf	29895	2.669840506	0.03716
5789	110033	Kif22	3835	-1.24850846	0.48966
5790	17188	Maz	4150	0.353588218	0.40878
5791	78388	Mvp	9961	-0.026048847	0.99988
5792	52858	Cdipt	10423	-0.395976527	0.45158
5793	56018	Stard10	10809	-1.042448137	0.24117
5794	233875	Ino80e	283899	0.34727814	0.63574
5795	207278	Fchsd2	9873	0.091779799	0.92677
5796	11674	Aldoa		-0.11656382	0.74095
5797	56420	Ppp4c	5531	-1.530423611	0.16548

5798	27276	Plekhb1	58473	0.897646194	0.04309
5799	68616	Gdpd3	79153	-2.349045857	0.18424
5800	19346	Rab6a	5870	0.165468669	0.52203
5801	52443	Mrpl48	51642	0.151111359	0.97042
5802	12721	Coro1a	11151	1.685952208	0.00502
5803	20887	Sult1a1		1.464525481	0.0224
5804	75565	Sgf29	112869	0.650573068	0.23051
5805	56312	Nupr1	26471	-0.267282423	0.73182
5806	72590	Ppme1	51400	-0.3301215	0.51983
5807	12752	Cln3	1201	-0.322771427	0.61603
5808	18020	Nfatc2ip	84901	0.554573599	0.22364
5809	67164	Lipt2	387787	0.308065007	0.8923
5810	67967	Pold3		-0.01021134	0.99988
5811	70314	Rabep2	79874	0.486030331	0.83928
5812	70974	Pgm2l1	283209	-0.463239685	0.41316
5813	20399	Sh2b1	25970	0.804007514	0.01882
5814	101488	Slco2b1	11309	1.404213351	0.01733
5815	56347	Eif3c		-0.620652527	0.28283
5816	71960	Myh14	79784	0.078518401	0.87342
5817	73658	Spns1	83985	0.055819498	0.96528
5818	16797	Lat	27040	0.420649311	0.47454
5819	27050	Rps3		0.156284529	0.22582
5820	60504	Il21r	50615	1.730384898	0.10048
5821	67800	Dgat2	84649	0.885366391	0.01208
5822	16190	Il4ra	3566	1.306908246	0.01817
5823	67711	Nsmce1	197370	0.375720743	0.38767
5824	26440	Psma1		0.53659782	0.09797
5825	72981	Thap12		0.464239116	0.64319
5826	70349	Copb1	1315	0.471918721	0.36091
5827	67420	Far1	84188	0.274450683	0.5912
5828	66190	Acer3	55331	-0.583288767	0.36281
5829	17921	Myo7a	4647	1.44372562	0.01541
5830	30949	Lcmt1	51451	0.800300457	0.18609
5831	70497	Arhgap17	55114	0.175275776	0.80153
5832	68897	Disp1	84976	0.044409052	0.99988
5833	57342	Parva	55742	-0.55683641	0.40878
5834	50781	Dkk3	27122	1.365021467	0.01441
5835	18479	Pak1	5058	0.258300236	0.99988
5836	19647	Rbbp6	5930	0.104086258	0.95323
5837	233913	Rusf1	64755	0.297005412	0.92311
5838	21804	Tgfb1i1	7041	0.398746944	0.92584
5839	12862	Cox6a2	1339	1.422719975	0.09744
5840	16409	Itgam		3.06763816	0.00356
5841	114332	Lyve1	10894	1.911026592	0.00531
5842	67150	Rnf141	50862	0.798407873	0.37111
5843	16411	Itgax	3687	1.348860116	0.01105
5844	66824	Pycard	29108	1.028457787	0.0292
5845	233908	Fus	2521	-0.309416753	0.58979
5846	21677	Tead2	8463	1.67887045	0.00589
5847	12493	Cd37	951	0.061516661	0.98901

5848	76560	Prss8	5652	-0.798198278	0.43713
5849	67773	Kat8	84148	2.071289692	0.00407
5850	12041	Bckdk	10295	-0.460605721	0.52856
5851	20909	Stx4a	6810	0.431859698	0.15758
5852	233902	Fbxl19	54620	-1.286064614	0.20699
5853	12055	Bcl7c	9274	-0.604938359	0.4026
5854	68961	Phkg2	5261	-0.878139034	0.28709
5855	233900	Rnf40	9810	-0.392702391	0.7675
5856	233895	Prr14	78994	0.537092982	0.02776
5857	78921	130019O22Rik		0.495606654	0.94452
5858	18220	Nucb1	4924	-0.81864339	0.24307
5859	16408	Itgal	3683	1.063875105	0.01664
5860	211548	Nomo1		-0.615953465	0.38093
5861	27414	Sergef	26297	0.473404424	0.07145
5862	66508	Lamtor1	55004	0.038963119	0.99988
5863	67865	Rgs10	6001	2.065676966	0.00447
5864	21843	Tial1	7073	0.391643766	0.02648
5865	29810	Bag3	9531	1.187729791	0.00734
5866	14183	Fgfr2	2263	0.307576874	0.46452
5867	11907	Ate1	11101	0.432265684	0.43562
5868	57752	Tacc2	10579	-0.851986948	0.53652
5869	66885	Acadsb	36	-0.734033644	0.40878
5870	55987	Cpxm2	119587	1.587723892	0.00554
5871	18817	Plk1	5347	-0.826893035	0.68213
5872	59288	Dctn5	84516	0.268034169	0.96769
5873	70316	Ndufab1	4706	0.49568478	0.01632
5874	28018	Ubfd1	56061	-0.253994567	0.63304
5875	67417	Ears2	124454	-0.189546976	0.81587
5876	74105	Gga2	23062	-1.637967957	0.12309
5877	20277	Scnn1b	6338	-2.252280745	0.01832
5878	59052	Mettl9	51108	0.319647488	0.68433
5879	12585	Cdr2	1039	0.064939739	0.98575
5880	27397	Mrpl17	63875	0.356721042	0.67738
5881	26939	Polr3e	55718	0.711638332	0.33435
5882	76932	Arfip2	23647	-0.719898832	0.32442
5883	67003	Uqcrc2	7385	0.28287467	0.10975
5884	67983	Pdzd9	255762	0.096133289	0.98224
5885	101867	Rrp8	23378	-1.001180128	0.32375
5886	16202	Ilk	3611	1.65086149	0.05685
5887	12751	Tpp1	1200	0.469383002	0.02021
5888	12971	Crym	1428	-1.194972927	0.25024
5889	233806	Tmem159	57146	-0.084110395	0.9188
5890	20128	Trim30a		-0.281314071	0.65156
5891	73919	Lyrm1	57149	-0.033480555	0.99988
5892	434234	Rexo5	81691	0.627587199	0.62421
5893	71151	Eri2	112479	-0.241227498	0.81665
5894	77590	Chst15	51363	-0.38503435	0.61315
5895	18242	Oat	4942	0.410070181	0.64349
5896	233802	Thumpd1	55623	-0.036297162	0.99988
5897	67133	Gp2	2813	0.476957105	0.98575

5898	77938	Fam53b	9679	0.001438448	0.99995
5899	72096	Eef1akmt2		-0.455747046	0.59996
5900	109359	Abraxas2	23172	-0.499814157	0.51255
5901	20821	Trim21	6737	-0.184996277	0.99988
5902	360216	Zranb1	54764	-0.802911705	0.32146
5903	13017	Ctbp2	1488	-0.467419716	0.59613
5904	20133	Rrm1	6240	0.163115355	0.99988
5905	22276	Uros	7390	-0.633375463	0.54726
5906	66356	Knop1	400506	-0.739543509	0.29273
5907	71517	Vps35l	57020	0.348195337	0.04224
5908	66165	Bccip	56647	-0.017219097	0.99988
5909	101437	Dhx32	55760	-0.543987182	0.45952
5910	20866	Stim1	6786	0.042133129	0.99988
5911	233575	Pgap2	27315	-2.161926226	0.04189
5912	17345	Mki67	4288	-0.947278656	0.30693
5913	70495	Atp6ap2	10159	0.826165871	0.00933
5914	22284	Usp9x		-0.305785589	0.68632
5915	12361	Cask	8573	-0.093082741	0.99988
5916	20947	Swap70	23075	-0.266306882	0.89195
5917	22390	Wee1	7465	-0.100573276	0.96072
5918	56786	Tmem9b	56674	-0.072926755	0.99988
5919	57373	Akip1	56672	0.700187532	0.8496
5920	76954	Denn2b	6764	-0.302097396	0.97042
5921	66085	Eif3f	8665	-0.343015062	0.40878
5922	104130	Ndufb11		0.08068491	0.97778
5923	236732	Rbm10	8241	0.308594991	0.10633
5924	18555	Cdk16	5127	-0.863143021	0.29464
5925	236733	Usp11	8237	2.176214551	0.04809
5926	30926	Glrx3	10539	0.149717434	0.991
5927	72284	LTO1	220064	-1.224724287	0.18988
5928	101772	Ano1	55107	-0.427962037	0.59393
5929	14082	Fadd	8772	-0.457774634	0.65328
5930	13043	Cttn	2017	-0.12668673	0.9998
5931	56774	Slc6a14		-0.175271498	0.99988
5932	78914	Nadsyn1	55191	-0.342903095	0.91039
5933	75974	Dock11	139818	1.196952809	0.00837
5934	72584	Cul4b		-0.498576207	0.65863
5935	21953	Tnni2	7136	0.527926818	0.3044
5936	93761	Smarca1	6594	-1.800872707	0.08524
5937	74131	Sash3	54440	0.843033542	0.21808
5938	56501	Elf4	2000	1.72665116	0.00469
5939	20523	Slc25a14	9016	0.197349533	0.78259
5940	209003	Rbmx2	51634	0.295752172	0.7418
5941	209224	Enox2	10495	0.185545172	0.8833
5942	70415	Stk26	51765	-0.189639179	0.99988
5943	14735	Gpc4	2239	-0.736958826	0.32237
5944	331004	Slc9a9	285195	0.618712177	0.49109
5945	73341	Arhgef6	9459	1.242123424	0.00734
5946	19655	Rbmx	494115	-0.672400902	0.30147
5947	14168	Fgf13	2258	0.740327158	0.49077

5948	54638	Ccdc22	28952	-0.477102441	0.86883
5949	54630	Prickle3	4007	1.005343564	0.08802
5950	18824	Plp2		0.452062281	0.32981
5951	209416	Gpkow	27238	-0.360463484	0.76459
5952	54637	Praf2	11230	2.026828514	0.01116
5953	54648	Ccdc120	90060	0.079212593	0.99988
5954	54645	Gripap1	56850	-0.712562181	0.3812
5955	54644	Otud5	55593	0.203484768	0.99148
5956	18715	Pim2	11040	-0.77941486	0.30566
5957	22232	Slc35a2	7355	0.116909352	0.99463
5958	54633	Pqbp1	10084	0.798414042	0.0155
5959	21855	Timm17b	10245	-0.067465283	0.99988
5960	15185	Hdac6	10013	0.657628037	0.47885
5961	22376	Was	7454	1.216731006	0.38355
5962	73447	Wdr13	64743	0.227456001	0.57998
5963	19652	Rbm3	5935	0.021482606	0.99988
5964	13595	Ebp		0.275976516	0.41716
5965	53627	Porcn	64840	-0.214442405	0.991
5966	54632	Ftsj1	24140	0.609535426	0.84628
5967	19893	Rpgr	6103	-0.563394378	0.61121
5968	67117	Dynlt3	6990	-0.178722815	0.71762
5969	14266	Aff2	2334	0.1749023	0.79078
5970	22327	Vbp1	7411	0.363724594	0.12036
5971	67391	Fundc2	65991	0.20778742	0.99988
5972	210766	Brcc3		0.26771703	0.86839
5973	67790	Rab39b	116442	2.323728961	0.00652
5974	17698	Msn	4478	-0.133325186	0.95731
5975	15203	Heph	9843	0.997171256	0.15758
5976	94190	Ophn1	4983	-0.95663849	0.16998
5977	236920	Stard8	9754	0.740563288	0.78632
5978	13641	Efnb1	1947	-0.12677413	0.99988
5979	18518	Igbp1		0.377704819	0.04684
5980	67683	Pbdc1	51260	0.247612484	0.83213
5981	107528	Magee1	57692	0.29474705	0.99988
5982	22589	Atrx	546	-1.085551217	0.11604
5983	66142	Cox7b	1349	0.799508306	0.01085
5984	67075	Magt1	84061	-0.195859917	0.85705
5985	16431	Itm2a	9452	1.25615349	0.24307
5986	67028	610002M06Rik		0.479538509	0.27917
5987	50887	Hmgn5	79366	-0.60004996	0.37299
5988	56726	Sh3bgrl	6451	0.355164683	0.43354
5989	64103	Tnmd	64102	-0.157854758	0.99988
5990	68792	Srpx2	27286	0.716923618	0.02017
5991	27359	Sytl4	94121	1.214883975	0.34896
5992	108062	Cstf2	1478	-0.084360459	0.94078
5993	237038	Nox1	27035	-0.66588408	0.62185
5994	102920	Cenpi	2491	0.17449598	0.99988
5995	11605	Gla	2717	0.503500853	0.99988
5996	94216	Col4a6	1288	0.110469973	0.99988
5997	12830	Col4a5	1287	0.561192295	0.04036

5998	50790	Acsl4	2182	0.420329303	0.12932
5999	83453	Chrdl1	91851	0.416467774	0.7324
6000	18481	Pak3	5063	-2.475578765	0.0475
6001	320302	Glt28d2		-0.906698127	0.34782
6002	210297	Lrch2	57631	1.06810955	0.03565
6003	382253	Cdkl5	6792	-1.354526967	0.20884
6004	110094	Phka2	5256	-0.374145022	0.68253
6005	11989	Slc7a3	84889	1.084048427	0.18014
6006	237175	Adgrg2	10149	0.611057231	0.92367
6007	18597	Pdha1	5160	0.307945721	0.04088
6008	16186	Il2rg		2.43724572	0.00388
6009	110651	Rps6ka3	6197	0.813323503	0.01186
6010	56364	Zmym3	9203	0.174290937	0.991
6011	53610	Nono	4841	0.737216842	0.27781
6012	270627	Taf1		0.156663643	0.45503
6013	20102	Rps4x	6191	0.194592589	0.86848
6014	70887	Dmrtc1a		0.4036414	0.70027
6015	12212	Chic1	53344	0.154948855	0.73275
6016	192176	Flna	2316	0.08865923	0.87991
6017	11306	Abcb7	22	0.197570178	0.86636
6018	17772	Mtm1		-1.219823289	0.13178
6019	14758	Gpm6b	2824	0.236002078	0.87315
6020	26370	Cetn2	1069	-0.442216853	0.37959
6021	18194	Nsdhl	50814	-0.186362118	0.99988
6022	22673	Zfp185	7739	-0.802482733	0.49126
6023	15159	Hccs	3052	-1.019387891	0.26896
6024	245688	Rbbp7	5931	0.072225685	0.95348
6025	11856	Arhgap6	395	0.154327018	0.99988
6026	67043	Syap1	94056	0.725937047	0.25473
6027	17692	Msl3	10943	-0.146177173	0.64826
6028	55936	Ctps2	56474	0.238817029	0.95765
6029	27081	Zfp275		0.41278218	0.64242
6030	108012	Ap1s2	8905	0.681144706	0.04631
6031	22184	Zrsr2		-0.363446926	0.58565
6032	73738	Haus7	55559	0.038186242	0.99988
6033	56078	Car5b	11238	0.995230155	0.02798
6034	12111	Bgn	633	0.765044333	0.16986
6035	11666	Abcd1	215	1.069918063	0.02563
6036	69656	Pir	8544	-2.01582769	0.13399
6037	14205	Vegfd	2277	0.728448655	0.25284
6038	18700	Piga	5277	-0.584536111	0.41981
6039	15161	Hcfc1	3054	0.151366545	0.99988
6040	19703	Renbp	5973	0.21501383	0.71878
6041	56292	Naa10	8260	-0.605852456	0.29816
6042	12000	Avpr2	554	0.149655919	0.99988
6043	16728	L1cam	3897	0.586949449	0.36204
6044	16179	Irak1	3654	-0.221147491	0.75914
6045	17257	Mecp2	4204	0.525182642	0.01962
6046	18846	Plxna3	55558	-0.269184689	0.913
6047	66294	Fam3a	60343	0.509605805	0.79541

6048	14381	G6pdx		0.301836655	0.96072
6049	17524	Mpp1	4354	0.292504488	0.74391
6050	245474	Dkc1	1736	-0.401483748	0.57781
6051	56397	Morf4l2	9643	0.461262187	0.24007
6052	18823	Plp1	5354	0.441335028	0.63051
6053	66995	Zcchc18		-0.807365323	0.33994
6054	53380	Psmd10	5716	1.06591687	0.15529
6055	78789	Vsig1	340547	1.901938742	0.00577
6056	14605	Tsc22d3	1831	1.302228283	0.12932
6057	19139	Prps1		-0.107565243	0.77086
6058	237073	Rbm41	55285	-0.792504381	0.74943
6059	75746	Morc4	79710	-0.368086286	0.91487
6060	66889	Rnf128	79589	0.51524448	0.03346
6061	50770	Atp11a	23250	0.381859854	0.60226
6062	17207	Mcf2l	23263	0.333791724	0.89833
6063	14068	F7	2155	-0.296561671	0.83285
6064	14058	F10	2159	0.454299676	0.3754
6065	66901	Proz	8858	-0.303078137	0.38023
6066	99375	Cul4a	8451	-0.036491622	0.99988
6067	16783	Lamp1	3916	-0.053195197	0.98772
6068	14456	Gas6	2621	1.299630256	0.01357
6069	19414	Rasa3	22821	1.008680392	0.34574
6070	66423	Coprs	55352	0.130194993	0.90723
6071	17930	Myom2	9172	0.013478218	0.85472
6072	11601	Angpt2	285	0.308469384	0.93354
6073	52123	Agpat5	55326	-0.352991049	0.51695
6074	70160	Vps36	51028	0.07162029	0.70755
6075	18408	Slc25a15	10166	-0.189829281	0.99988
6076	244373	Erlin2	11160	-0.031419231	0.99988
6077	114863	Plpbp	11212	-0.152928687	0.89638
6078	78560	Adgra2	25960	1.958218187	0.00566
6079	66653	Brf2	55290	0.160824793	0.90651
6080	75767	Rab11fip1	80223	-0.664069894	0.50862
6081	13685	Eif4ebp1	1978	-1.277165542	0.13225
6082	170786	Cd209a		-0.004695948	0.99989
6083	24099	Tnfsf13b	10673	1.467554696	0.00843
6084	12826	Col4a1	1282	0.261598591	0.99988
6085	12827	Col4a2	1284	0.518676414	0.31884
6086	19332	Rab20	55647	0.329575566	0.78781
6087	69225	Naxd	55739	-0.364641877	0.48371
6088	320139	Ptpn7	5778	1.352741473	0.00972
6089	102334	Ankrd10	55608	-1.007152697	0.22712
6090	54126	Arhgef7	8874	-1.115977472	0.26961
6091	68192	Leprotl1	23484	-0.028341514	0.99988
6092	22428	Dctn6	10671	0.432850342	0.01441
6093	234267	Gpm6a	2823	2.187934475	0.14124
6094	76294	Asb5	140458	-0.831774577	0.24661
6095	22341	Vegfc	7424	0.632542759	0.02842
6096	11593	Aga	175	0.272019249	0.06935
6097	50768	Dlc1	10395	0.915308086	0.7324

6098	67276	Eri1	90459	0.606413385	0.54528
6099	21951	Tnks	8658	0.203696989	0.99988
6100	67887	Saraf	51669	0.2137151	0.0721
6101	57312	Mrps31	10240	0.058958097	0.99988
6102	102032	Smim19	114926	0.264407687	0.38237
6103	18970	Polb	5423	0.12831647	0.99988
6104	16150	Ikbkb	3551	0.194948723	0.98494
6105	18791	Plat	5327	1.054694147	0.21958
6106	64933	Ap3m2	10947	-0.279764111	0.65276
6107	244349	Kat6a	7994	-0.218756933	0.99988
6108	102247	Gpat4	137964	0.326215062	0.63016
6109	109145	Gins4	84296	0.36155607	0.28967
6110	20377	Sfrp1	6422	0.699221418	0.28119
6111	11502	Adam9		0.789486892	0.2103
6112	69742	Tm2d2		0.593026084	0.02495
6113	83436	Plekha2		-0.150997287	0.99988
6114	20563	Slit2	9353	0.532322304	0.2691
6115	23965	Tenm3	55714	-0.626925575	0.82473
6116	320685	Dctd	1635	0.248379635	0.991
6117	52357	Wwc2	80014	-0.676100959	0.29838
6118	14182	Fgfr1	2260	0.71127569	0.21326
6119	192174	Rwdd4a	201965	-0.002544434	0.99988
6120	71910	Plpp5	84513	-1.051343077	0.20374
6121	20845	Star	6770	-0.470361762	0.40878
6122	23808	Ash2l	9070	-0.363770187	0.45862
6123	234138	Tti2	80185	0.052682469	0.99988
6124	67920	Mak16	84549	-0.251503562	0.5523
6125	22427	Wrn	7486	-2.216356304	0.06955
6126	14782	Gsr	2936	-0.530725303	0.45517
6127	68153	Gtf2e2	2961	-0.243835824	0.75937
6128	19663	Rbpms	11030	-0.301381108	0.74478
6129	14300	Frg1		-0.586296741	0.39574
6130	11886	Asah1	427	1.014457372	0.01664
6131	18536	Pcm1	5108	-0.566916558	0.58964
6132	234199	Fgl1	2267	0.09973759	0.81382
6133	68797	Pdgfrl	5157	1.671643668	0.056
6134	11988	Slc7a2	6542	0.2981093	0.99988
6135	52348	Vps37a	137492	-0.287276557	0.60729
6136	18983	Cnot7	29883	-0.597787254	0.46745
6137	66234	Msmo1	6307	-0.529335508	0.39006
6138	77113	Klhl2	11275	0.055030432	0.99988
6139	108150	Galnt7	51809	-1.341549596	0.20118
6140	60406	Sap30	8819	-0.19211002	0.99988
6141	13617	Ednra	1909	1.541798324	0.00544
6142	234463	Tmem184c	55751	-0.91781836	0.51701
6143	110784	Nr3c2	4306	1.01538149	0.01889
6144	20467	Sin3b	23309	-0.100858231	0.99988
6145	234214	Sorbs2	8470	-0.939943429	0.17361
6146	16363	Irf2	3660	-0.220933172	0.69589
6147	12367	Casp3	836	0.083471851	0.99988

6148	71876	Cenpu	79682	-1.494068336	0.05676
6149	66756	Cfap97	57587	-0.424485234	0.53533
6150	11739	Slc25a4	291	0.572094898	0.39112
6151	192169	Ufsp2	55325	0.474016439	0.03692
6152	53318	Pdlim3	27295	2.360125761	0.00621
6153	67620	Lrp2bp	55805	-0.107793362	0.99988
6154	142980	Tlr3	7098	-0.017247612	0.99988
6155	234309	Cbr4	84869	0.264040828	0.64193
6156	59009	Sh3rf1	57630	0.639843539	0.51011
6157	18004	Nek1	4750	-0.849856722	0.2699
6158	71306	Mfap3l	9848	-1.930309513	0.0707
6159	80750	N4bp1	9683	-1.194199284	0.18095
6160	234549	Heatr3	55027	-0.523206123	0.37422
6161	11513	Adcy7	113	0.915308154	0.05185
6162	26992	Brd7	29117	-0.368235763	0.44852
6163	93960	Nkd1	85407	1.247822366	0.05795
6164	71607	Snx20	124460	0.657736448	0.22496
6165	19651	Rbl2	5934	-0.996906368	0.27974
6166	14339	Aktip	64400	0.120870421	0.92393
6167	13666	Eif2ak3	9451	-0.776210626	0.44695
6168	78833	Gins3	64785	0.990136489	0.53204
6169	66083	Setd6	79918	-0.337007595	0.51211
6170	14719	Got2	2806	-1.008935214	0.23715
6171	12552	Cdh11	1009	0.78851484	0.54595
6172	17125	Smad1	4086	-0.859038456	0.29276
6173	78651	Lsm6	11157	-0.072539109	0.99988
6174	76775	Slc10a7	84068	-0.396049199	0.588
6175	212999	Tnpo2		-0.562209751	0.3984
6176	65114	Vps35	55737	0.194031707	0.7936
6177	56452	Orc6	23594	-0.245293425	0.90772
6178	108682	Gpt2	84706	0.451153414	0.59955
6179	56445	Dnaja2	10294	-0.437860762	0.40896
6180	71927	Itfg1	81533	-0.282541439	0.91629
6181	19724	Rfx1	5989	-0.330481051	0.92291
6182	106529	Tecr	9524	-0.415402439	0.64985
6183	71310	Tbc1d9	23158	-0.571043435	0.62774
6184	30932	Zfp330	27309	-0.175365524	0.7454
6185	16168	Il15	3600	3.50462E-05	0.99988
6186	14388	Gab1	2549	-0.157581161	0.73803
6187	93762	Smarca5	8467	-0.563250954	0.45637
6188	15439	Hp		0.346128537	0.65242
6189	234723	Txnl4b	54957	0.106549262	0.97069
6190	75871	Zfp821	55565	-0.537070039	0.51548
6191	71955	Ist1	9798	0.030960203	0.99988
6192	56749	Dhodh	1723	-0.421344998	0.75897
6193	11765	Ap1g1	164	-0.375128556	0.82613
6194	244650	Phlpp2	23035	0.260391644	0.66436
6195	54352	Irx5	10265	0.032942809	0.99988
6196	17390	Mmp2	4313	0.274567826	0.67654
6197	20444	St3gal2	6483	0.836856287	0.0155

6198	76527	Il34	146433	1.433104477	0.00764
6199	23802	Amfr	267	-0.424836528	0.49915
6200	102339	Cog4	25839	0.167405927	0.99988
6201	68219	Nudt21	11051	0.025415741	0.99988
6202	67378	Bbs2		-0.161846449	0.99988
6203	75796	Cdyl2	124359	-0.266438729	0.99988
6204	17750	Mt2		0.341885948	0.14032
6205	17748	Mt1		0.121522925	0.87828
6206	67528	Nudt7	283927	-0.32717747	0.77629
6207	64209	Herpud1	9709	0.792444399	0.21409
6208	102122	Psme3ip1	80011	0.542545963	0.01862
6209	67801	Pllp	51090	1.379827478	0.00837
6210	107566	Arl2bp	23568	-0.266300568	0.65346
6211	20312	Cx3cl1	6376	-0.266241177	0.65383
6212	20295	Ccl17	6361		0.40878
6213	109006	Ciapin1	57019	-0.706767592	0.45818
6214	67914	Coq9	57017	0.301657196	0.99988
6215	20021	Polr2c	5432	-0.101863475	0.80347
6216	14766	Adgrg1	9289	0.522317878	0.59254
6217	74187	Katnb1	10300	0.722398809	0.47207
6218	16582	Kifc3	3801	-0.254607955	0.80116
6219	333329	Cngb1	1258	0.157068044	0.99988
6220	17388	Mmp15	4324	-0.398227892	0.45457
6221	74166	Tmem38a	79041	0.585145386	0.97371
6222	101985	Usb1	79650	0.137704487	0.48698
6223	14894	Cfap20	29105	0.114569587	0.99988
6224	326618	Tpm4	7171	0.423080865	0.52335
6225	72297	B3gnt3	10331	-0.124944773	0.73729
6226	16453	Jak3	3718	-0.562387816	0.5476
6227	66171	Pgls	25796	-1.256584826	0.09426
6228	26457	Slc27a1	376497	-0.241193355	0.79728
6229	67443	Map1lc3b		0.476132715	0.03146
6230	73711	Mvb12a	93343	-1.007303112	0.26012
6231	234814	Mthfsd	64779	-0.456383909	0.50785
6232	12857	Cox4i1	1327	-0.042836727	0.99988
6233	18117	Emc8	10328	0.19930347	0.99397
6234	68251	Babam1		-0.383527914	0.46908
6235	272551	Gins2	51659	-0.13564629	0.99988
6236	382034	Gse1	23199	-1.448008727	0.20621
6237	102193	Zdhhc7	55625	0.918305325	0.47048
6238	234797	430548M08R	9764	1.756586069	0.00721
6239	78892	Crispld2	83716	-0.051161813	0.99988
6240	22224	Usp10	9100	-0.245410204	0.80616
6241	72042	Cotl1	23406	1.439324062	0.00734
6242	234796	Klhl36	79786	-0.929984193	0.29038
6243	68270	Dnaaf1	123872	0.166334967	0.20222
6244	546071	Mast3	23031	-1.190687846	0.23751
6245	18709	Pik3r2		-0.50179011	0.64336
6246	56453	Mbtps1	8720	0.191200789	0.44637
6247	65972	Ifi30	10437	0.193364808	0.80287

6248	68196	Hsbp1	3281	0.529244324	0.01557
6249	19339	Rab3a	5864	-0.631880951	0.3235
6250	12554	Cdh13	1012	0.088460066	0.99988
6251	68533	Mphosph6	10200	0.157256997	0.57255
6252	50783	Lsm4	25804	-0.220658003	0.67269
6253	66566	Ntpcr	84284	0.807275486	0.2306
6254	74549	Mau2	23383	-1.712557941	0.11879
6255	170759	Atp13a1	57130	-0.579478474	0.32995
6256	70885	Ints10		0.755459149	0.48431
6257	13191	Dctn1		-0.223896113	0.57719
6258	12562	Cdh5	1003	0.294272161	0.65158
6259	65115	Bean1	146227		0.40878
6260	68119	Cmtm3	123920	1.048054491	0.02566
6261	72361	Ces2g		1.601706228	0.00583
6262	234664	Nae1	8883	-0.256736012	0.66793
6263	68523	Ciao2b	51647	-0.277174374	0.99988
6264	56437	Rrad	6236	-0.165484784	0.89659
6265	12400	Cbfb	865	0.106227708	0.73777
6266	71609	Tradd	8717	-0.329560125	0.85807
6267	234678	230025D16R	80262	0.424651108	0.89659
6268	15484	Hsd11b2	3291	1.956912236	0.08986
6269	19171	Psmb10	5699	-0.321155601	0.77667
6270	66369	Dus2	54920	-0.360143333	0.68231
6271	18021	Nfatc3	4775	-0.103304417	0.99988
6272	192654	Pla2g15	23659	1.046515756	0.00905
6273	330836	Slc7a6	9057	-0.360011621	0.98415
6274	58994	Smpd3	55512	-0.527976435	0.42129
6275	22751	Zfp90	146198	0.98233537	0.00959
6276	15118	Has3	3038	-0.072673082	0.99988
6277	116733	Vps4a		0.727986506	0.01732
6278	97484	Cog8		-0.317028117	0.57051
6279	66164	Nip7	51388	0.192322548	0.99988
6280	77116	Mtmr2	8898	-0.111476398	0.94258
6281	21750	Terf2	7014	-0.433838794	0.37265
6282	74360	Cep57	9702	-1.092493575	0.251
6283	66427	Cyb5b	80777	0.27405591	0.5804
6284	270118	Maml2	84441	-0.360339605	0.62525
6285	17535	Mre11a	4361	-0.618169325	0.59616
6286	66894	Wwp2	11060	0.627967621	0.22061
6287	56503	Ankrd49	54851	-0.815877488	0.26377
6288	14608	Gpr83	10888	-2.506598838	0.08195
6289	64931	Izumo1r	390243	0.672897328	0.08624
6290	55991	Panx1	24145	-0.292886111	0.742
6291	234959	Med17		-0.636665656	0.40947
6292	69137	Vstm5	387804	-2.18008335	0.02693
6293	70984	931406C07R	28970	0.537418755	0.35027
6294	75316	Taf1d	79101	-0.733271272	0.28502
6295	85305	Kars	3735	-0.236173034	0.64582
6296	30947	Adat1	23536	1.006996243	0.25718
6297	93739	Gabarapl2	11345	0.802751886	0.03066

6298	234740	Tmem231	79583	0.183343566	0.72301
6299	66817	Tmem170		0.758598176	0.47329
6300	23837	Cfdp1	10428	-0.498357989	0.57458
6301	12927	Bcar1	9564	-0.393201452	0.47218
6302	52815	Ldhd	197257	1.233119966	0.12595
6303	319481	Wdr59	79726	0.472050091	0.29762
6304	234734	Aars	16	-0.39527148	0.45457
6305	73230	Bmper	168667	0.797588364	0.06392
6306	114896	Afg3l1		-0.414803123	0.98665
6307	66948	Acad8	27034	0.03075466	0.99988
6308	11459	Acta1	58	1.559071893	0.0176
6309	56199	Abcb10	23456	-0.00426068	0.99988
6310	382038	Urb2	9816	-1.181022671	0.26928
6311	76332	Cog2	22796	-0.370043924	0.82473
6312	68865	Arv1	64801	1.015128272	0.16205
6313	69551	310022B05R	84886	-0.852094316	0.24294
6314	66523	810004N23R	128061	-0.887027595	0.22688
6315	14712	Gnpat	8443	0.275620787	0.74701
6316	244666	Sprtn	83932	0.036056697	0.99988
6317	112405	Egln1		-0.137070756	0.8601
6318	69091	Vps26b	112936	-0.338080871	0.55251
6319	83964	Jam3	83700	0.880673877	0.29572
6320	102607	Snx19	399979	-0.300914357	0.67773
6321	30806	Adamts8	11095	0.060529561	0.89195
6322	19143	St14	6768	-0.829933796	0.23142
6323	11804	Aplp2	334	-0.823469042	0.29838
6324	11796	Birc3	330	-0.222270301	0.79237
6325	76863	Dcun1d5	84259	0.381233337	0.44227
6326	71785	Pdgfd	80310	0.178677884	0.8603
6327	75747	Sesn3	143686	-0.207387636	0.89785
6328	53376	Usp2	9099	0.355150681	0.43255
6329	21838	Thy1	7070	1.553789874	0.00764
6330	58235	Nectin1	5818	-0.892501583	0.3235
6331	72169	Trim29	23650	-0.614636456	0.56115
6332	102644	Oaf	220323	0.845223985	0.15202
6333	235293	Sc5d	6309	-0.371100148	0.59775
6334	72828	Ubash3b	84959	0.479445045	0.95272
6335	71566	Clmp	79827	0.463132071	0.48844
6336	104444	Rexo2	25996	-0.378409824	0.79662
6337	78252	Nxpe2	120406	-0.443777879	0.86915
6338	75717	Cul5	8065	-0.346980421	0.43628
6339	12023	Barx2	8538	0.979675896	0.19713
6340	23871	Ets1	2113	-0.188028725	0.92721
6341	20443	St3gal4	6484	0.200513917	0.89195
6342	69305	Dcps	28960	0.219026032	0.71686
6343	117149	Tirap	114609	-0.34705511	0.93349
6344	67398	Srpr	6734	0.000565689	0.99989
6345	71989	Rpusd4	84881	0.181159764	0.45341
6346	76192	Abhd12	26090	0.1669414	0.78102
6347	110446	Acat1	38	0.555269058	0.04757

6348	19684	Rdx	5962	-0.683778727	0.3364
6349	14148	Fdx1	2230	0.451890643	0.05619
6350	18985	Pou2af1	5450	2.055204453	0.18021
6351	73699	Ppp2r1b	5519	0.009404038	0.99988
6352	102580	Alg9		0.073343886	0.99988
6353	12955	Cryab	1410	1.20332612	0.09426
6354	66952	310030G06R	91894	-0.137205996	0.92365
6355	330938	Dixdc1	85458	-0.387709209	0.51687
6356	19286	Pts	5805	0.15064437	0.84997
6357	76509	Plet1	349633	-0.049553937	0.99988
6358	54725	Cadm1	23705	-0.409508218	0.69565
6359	215051	Bud13	84811	-0.931814768	0.62385
6360	22687	Zpr1	8882	-0.340513033	0.48715
6361	21345	Tagln	6876	0.017959345	0.99988
6362	23821	Bace1	23621	-1.015958782	0.27068
6363	16154	Il10ra	3587	0.583976756	0.23468
6364	214523	Tmprss4	56649	-0.15507568	0.96811
6365	14012	Mpzl2	10205	-1.726650547	0.05738
6366	12500	Cd3d	915	0.872837409	0.27456
6367	213827	Arcn1	372	-0.398001195	0.72791
6368	13209	Ddx6	1656	-0.179288047	0.60772
6369	56872	Pate4	399968	2.922648263	0.00407
6370	67049	Pus3	83480	0.117425209	0.84891
6371	270151	Nlrx1	79671	0.348292089	0.84707
6372	60409	Trappc4	51399	0.500033099	0.03261
6373	14385	Slc37a4	2542	-1.18742762	0.16967
6374	12282	Hyou1	10525	0.545858465	0.02932
6375	16430	Stt3a	3703	-0.064732737	0.99988
6376	235180	Fez1	9638	0.323279492	0.6627
6377	102423	Hinfp	25988	0.291185996	0.57622
6378	71764	C2cd2l	9854	0.463847241	0.60755
6379	66279	Tmem218	219854	-0.199756228	0.90814
6380	56857	Slc37a2	219855	-0.330644752	0.74001
6381	13478	Dpagt1	1798	-0.116675808	0.99988
6382	74144	Robo4	54538	0.826989855	0.04929
6383	15288	Hmbs	3145	0.221261219	0.77629
6384	71732	Vps11	55823	-0.142977994	0.99988
6385	84004	Mcam	4162	-0.636779538	0.0847
6386	23988	Pin1		-0.203393502	0.991
6387	54721	Tyk2	7297	0.642798586	0.253
6388	18577	Pde4a	5141	0.846390366	0.02661
6389	16201	Ilf3	3609	-0.576369219	0.48143
6390	17083	Tmed1	11018	0.367585171	0.38859
6391	74766	Yipf2	78992	-0.299855514	0.56492
6392	70082	Lysmd2	256586	1.219147606	0.00909
6393	59035	Carm1	10498	-0.362538778	0.58965
6394	50876	Tmod2	29767	0.050384688	0.99988
6395	20586	Smarca4	6597	-0.996841167	0.26685
6396	14697	Gnb5	10681	-0.894688113	0.49842
6397	16835	Ldlr	3949	-0.705526438	0.82597

6398	235041	Kank2	25959	0.786617558	0.01275
6399	319899	Dock6	57572	0.756633236	0.76689
6400	28015	Polr2m	81488	0.31438645	0.10704
6401	11891	Rab27a	5873	-0.359751828	0.57946
6402	15450	Lipc	3990	-0.962921658	0.20832
6403	66660	Sltm	79811	-0.808102709	0.32302
6404	225215	Rsl24d1	51187	0.166786262	0.99988
6405	17999	Nedd4	4734	-0.032332022	0.99988
6406	93836	Rnf111	54778	-0.354140694	0.97277
6407	12442	Ccnb2	9133	-0.909222013	0.29513
6408	71602	Myo1e	4643	0.792588393	0.01881
6409	17427	Mns1	55329	0.6451226	0.33823
6410	21406	Tcf12	6938	-0.620061536	0.3193
6411	12306	Anxa2	302	0.181222561	0.09015
6412	68178	Cgnl1	84952	-0.580609755	0.42545
6413	93697	Ice2	79664	0.196470405	0.99988
6414	19883	Rora	6095	0.175972497	0.84546
6415	55934	Rp9	6100	-0.118633861	0.87828
6416	319480	Itga11	22801	0.685796328	0.89815
6417	14155	Fem1b	10116	-0.129025749	0.84526
6418	76524	Cln6	54982	1.560818672	0.00766
6419	75600	Calml4	91860	-0.371679617	0.57884
6420	11737	Anp32a		0.019555193	0.99947
6421	93683	Glce	26035	0.835096657	0.01742
6422	83946	Phip	55023	-0.208228641	0.62732
6423	71819	Kif23	9493	-1.0634188	0.22329
6424	212531	Sh3bgrl2	83699	-0.633393737	0.3667
6425	12040	Bckdhb	594	-0.40408448	0.88646
6426	26951	Zw10	9183	-0.756865911	0.49651
6427	212943	Tent5a	55603	-0.585572295	0.58003
6428	235323	Usp28	57646	-0.153531258	0.90973
6429	18113	Nnmt	4837	1.798689562	0.00407
6430	67834	Idh3a	3419	-0.024406396	0.99988
6431	21887	Tle3	7090	-0.132241279	0.78002
6432	102462	Imp3	55272	0.202598414	0.14788
6433	207596	Thsd4	79875	0.942655722	0.01741
6434	56294	Ptpn9	5780	0.077307745	0.77644
6435	64602	Ireb2	3658	-1.511748516	0.22496
6436	18746	Pkm	5315	-1.161740647	0.15447
6437	73744	Man2c1	4123	-0.546510267	0.41716
6438	72774	Neil1	79661	-0.216407988	0.76163
6439	66199	Commd4	54939	0.010527862	0.99988
6440	74211	700017B05R	56905	-0.274258979	0.74763
6441	26441	Psma4	5685	-0.164048326	0.69294
6442	78323	Fam219b	57184	0.567702038	0.01803
6443	110119	Mpi	4351	-1.159493241	0.39625
6444	109161	Ube2q2	92912	-1.047747513	0.25658
6445	71742	Ulk3	25989	2.513544955	0.00447
6446	71999	Fbxo22	26263	-0.041218584	0.9958
6447	83961	Nrg4	145957	0.234886734	0.96358

6448	12988	Csk	1445	0.329714908	0.05573
6449	110842	Etfa	2108	0.259126185	0.17116
6450	102414	Clk3	1198	-1.939039347	0.05221
6451	26611	Rcn2	5955	0.501502133	0.03382
6452	19200	Pstpip1	9051	0.960607022	0.01194
6453	13070	Cyp11a1	1583	0.628289322	0.22449
6454	56434	Tspan3	10099	1.732203541	0.0059
6455	69981	Tmem30a	55754	0.453141535	0.40074
6456	66867	Hmg20a	10363	0.021099278	0.9979
6457	12866	Cox7a2	1347	0.52691418	0.02798
6458	12816	Col12a1	1303	0.645399034	0.48303
6459	69106	Stoml1	9399	0.170319932	0.99988
6460	16949	Loxl1	4016	-0.237656092	0.97042
6461	20320	Nptn	27020	0.531617677	0.22496
6462	18007	Neo1	4756	0.127509394	0.89518
6463	68291	Mto1	25821	0.067375957	0.94915
6464	214763	Cgas	115004	0.808595839	0.01806
6465	14860	Gsta4		0.336303746	0.09936
6466	68801	Elovl5	60481	1.361493162	0.00857
6467	14629	Gclc	2729	-1.021347548	0.224
6468	214345	Lrrc1	55227	0.531902205	0.54223
6469	66111	Tmed3	23423	0.084775091	0.87096
6470	13036	Ctsh	1512	1.341048335	0.0089
6471	22003	Tpm1	7168	0.297820877	0.71272
6472	22038	Plscr1		0.139672748	0.99988
6473	80907	Lactb	114294	-0.68445443	0.43236
6474	18828	Plscr2		-0.26302086	0.99988
6475	76459	Car12	771	0.050014521	0.991
6476	26432	Plod2	5352	-1.226383031	0.2103
6477	208117	Aph1b		0.114281231	0.99988
6478	235441	Usp3	9960	0.006597025	0.99988
6479	235527	Plscr4	57088	1.233760804	0.14084
6480	13143	Dapk2	23604	0.55408731	0.24834
6481	68250	Ciao2a	84191	0.205896981	0.73063
6482	56440	Snx1	6642	0.515066572	0.24007
6483	19035	Ppib	5479	0.03583046	0.99988
6484	214897	Csnk1g1	53944	-0.642503697	0.37696
6485	56404	Trip4	9325	-0.184104956	0.70966
6486	71973	Rbpms2	348093	-0.558388605	0.97785
6487	27965	Spg21	51324	0.884250999	0.03285
6488	214424	Parp16	54956	0.643737991	0.28603
6489	74388	Dpp8	54878	-0.108277298	0.99988
6490	213550	Dis3l	115752	-0.642397865	0.34415
6491	66131	Tipin	54962	0.03753548	0.99988
6492	330959	Snapc5	10302	0.616654059	0.07846
6493	67891	Rpl4		0.241364032	0.6229
6494	68014	Zwilch	55055	0.67787655	0.03459
6495	17127	Smad3	4088	0.270990167	0.13566
6496	69478	300009A05R	145853	-0.159932421	0.99988
6497	56469	Pias1	8554	1.045812946	0.01186

6498	67958	U2surp	23350	-0.529128582	0.43943
6499	245000	Atr	545	-0.576689309	0.45319
6500	24127	Xrn1	54464	-0.118065266	0.85384
6501	211586	Tfdp2	7029	-1.027232293	0.3275
6502	11933	Atp1b3	483	0.406239885	0.0903
6503	114713	Rasa2	5922	-0.914856561	0.29711
6504	69519	Rwdd2a	112611	-0.493266318	0.0515
6505	17436	Me1	4199	0.426973802	0.02197
6506	76365	Tbx18	9096	0.096867752	0.98154
6507	23959	Nt5e	4907	0.742811992	0.26008
6508	244962	Snx14	57231	0.613000347	0.31728
6509	56403	Syncrip		-0.272875939	0.77703
6510	71640	Zfp949		0.377107744	0.32504
6511	56693	Crtap	10491	0.727930628	0.45813
6512	67213	Cmtm6	54918	0.068730739	0.99988
6513	235661	Dync1li1	51143	0.222428223	0.8865
6514	102545	Cmtm7	112616	-1.285643213	0.27962
6515	68292	Stt3b	201595	-0.181747203	0.62998
6516	21813	Tgfbr2	7048	0.152664038	0.73182
6517	192287	Slc25a36	55186	-0.312105672	0.61866
6518	74080	Nmnat3	349565	-0.741026334	0.37195
6519	50797	Copb2	9276	-0.17907539	0.78259
6520	64655	Mrps22	56945	0.732945255	0.22187
6521	74769	Pik3cb	5291	0.209408992	0.73352
6522	23873	Faim		-0.377014673	0.92288
6523	74125	Armc8	25852	-0.158694015	0.89195
6524	83703	Dbr1	51163	-0.100595386	0.99988
6525	17532	Mras	22808	0.257919142	0.72028
6526	17973	Nck1	4690	-1.095672648	0.33231
6527	12530	Cdc25a	993	-0.493888942	0.42975
6528	54369	Nme6	10201	-0.718971386	0.32237
6529	17758	Map4	4134	0.437738416	0.43426
6530	72831	Dhx30	22907	-1.052328153	0.24826
6531	20588	Smarcc1	6599	-1.775585882	0.09868
6532	29873	Cspg5	10675	-0.142439619	0.99988
6533	235623	Scap	22937	-0.230770937	0.55187
6534	19225	Ptgs2	5743	0.684488209	0.8717
6535	67169	Nradd		-0.081015552	0.99988
6536	19228	Pth1r	5745	0.002338636	0.99989
6537	17002	Ltf	4057	-0.947712401	0.36647
6538	71268	Lrrfip2	9209	-0.18534521	0.991
6539	17350	Mlh1	4292	1.039564567	0.33445
6540	211770	Trib1	10221	0.352376054	0.07565
6541	18571	Pdcd6ip		0.070683702	0.99988
6542	72179	Fbxl2	25827	-0.30049287	0.89847
6543	17874	Myd88	4615	0.302127313	0.06049
6544	74498	Gorasp1	64689	-0.017731989	0.99988
6545	215418	Csrnp1	64651	1.368897083	0.0991
6546	16785	Rpsa		-0.152315169	0.991
6547	208638	Slc25a38	54977	0.690623185	0.83403

6548	18087	Nktr	4820	-1.522610988	0.14058
6549	26901	Ss18l2	51188	0.417084358	0.66145
6550	66904	Pccb	5096	0.320669744	0.27799
6551	22354	Vipr1	7433	-0.281811696	0.73606
6552	56332	Amotl2	51421	0.610674226	0.47071
6553	28135	Cep63	80254	0.116439705	0.75992
6554	67095	Trak1	22906	-0.372872703	0.62967
6555	67469	Abhd5	51099	-0.067468852	0.99988
6556	20187	Ryk	6259	-0.308764939	0.6316
6557	24059	Slco2a1	6578	1.149007797	0.0199
6558	270192	Rab6b	51560	1.154196187	0.1788
6559	66202	110059G10R	57456	0.367466571	0.69275
6560	20818	Srprb	58477	-0.163119231	0.99716
6561	22041	Trf	7018	1.405237952	0.0054
6562	235559	Topbp1	11073	-1.127282387	0.31651
6563	66663	Uba5	79876	0.335443693	0.66769
6564	74025	Nphp3		-0.578657519	0.35368
6565	235567	Dnajc13	23317	0.512456862	0.46004
6566	56318	Acpp	55	-0.508719595	0.75897
6567	14678	Gnai2	2771	-0.564830733	0.40378
6568	94062	Mrpl3	11222	1.113576753	0.15572
6569	75686	Nudt16		-0.234285912	0.99988
6570	66595	Aste1	28990	0.636624146	0.76849
6571	235574	Atp2c1	27032	-0.990781574	0.28128
6572	75669	Pik3r4	30849	1.499457596	0.00855
6573	68553	Col6a4			0.991
6574	74840	Manf	7873	-0.023950828	0.99988
6575	102626	Mapkapk3	7867	0.775947562	0.17674
6576	12700	Cish	1154	1.343856875	0.10308
6577	69536	Hemk1	51409	-0.83956619	0.38074
6578	83486	Rbm5	10181	-0.154726502	0.90772
6579	19654	Rbm6	10180	-0.495476813	0.51011
6580	72825	Mon1a	84315	-1.090580107	0.18824
6581	19882	Mst1r	4486	0.315568581	0.99089
6582	22036	Traip	10293	-0.394977037	0.87991
6583	235606	Apeh	327	-0.037406623	0.99448
6584	320844	Amigo3	386724	-0.458372617	0.57622
6585	27399	Ip6k1	9807	0.002591007	0.99989
6586	74153	Uba7	7318	-0.054590857	0.99988
6587	80987	Nckipsd	51517	-0.34894335	0.64119
6588	76500	Ip6k2	51447	-0.529830354	0.7106
6589	19087	Prkar2a	5576	-1.191730858	0.26928
6590	57279	Slc25a20	788	0.150797068	0.74772
6591	97541	Qars	5859	0.126099149	0.83848
6592	66257	Nicn1	84276	-1.191554167	0.09252
6593	434437	Amt		0.285158143	0.13092
6594	78267	Klhdc8b	200942	0.587445681	0.72329
6595	22258	Usp4	7375	-0.31841878	0.49443
6596	103850	Nt5m	56953	-0.651321108	0.42115
6597	218543	Srek1		-1.027827521	0.26229

6598	78798	Eml4	27436	-1.039192507	0.20337
6599	330267	Thsd7a	221981	0.693978246	0.56771
6600	216805	Flcn		0.030921115	0.99988
6601	233871	Atxn2l	11273	-0.768566345	0.2856
6602	269941	Chsy1	22856	1.09372245	0.00652
6603	14760	Gpr19	2842	-0.014520836	0.99988
6604	14201	Fhl3	2275	-1.036379967	0.38367
6605	269132	Colgalt2	23127	-0.059139812	0.99779
6606	232430	Crebl2	1389	0.440606514	0.38302
6607	68521	Fam189b	10712	0.704263484	0.13729
6608	246727	Oas3	4940	-0.70178748	0.51114
6609	69399	700025G04R	81563	0.401780032	0.6316
6610	330260	Pon2	5445	0.714613117	0.0167
6611	67939	Prorsd1		0.299815228	0.68189
6612	12509	Cd59a		0.90370782	0.01571
6613	240354	Malt1	10892	-0.967089928	0.43481
6614	246728	Oas2	4939	-0.719526649	0.25496
6615	216799	Nlrp3	114548	-1.279471671	0.16167
6616	16909	Lmo2	4005	0.183548893	0.4523
6617	97827	Exd2	55218	-0.48492319	0.9104
6618	67246	Resf1	55196	-0.121802926	0.99988
6619	71709	Syde1	85360	1.587554742	0.00544
6620	228775	Trib3	57761	-1.737407906	0.14009
6621	67729	Mansc1	54682	0.239193929	0.99988
6622	14276	Folr2	2350	1.379306015	0.00775
6623	218613	Mier3	166968	-0.716053806	0.35509
6624	235406	Snx33	257364	-0.593882747	0.5312
6625	16332	Inppl1	3636	-0.358314738	0.96531
6626	108686	Ccdc88a	55704	-0.440892285	0.43801
6627	252972	Tpcn1	53373	0.433884129	0.55948
6628	233865	Katnip	23247	0.312910315	0.97377
6629	56198	Heyl	26508		0.991
6630	73274	Gpbp1		0.6656697	0.01372
6631	210710	Gab3	139716	1.098551212	0.01117
6632	170756	Slc8b1	80024	0.032936136	0.99988
6633	12068	Bet1		0.611939796	0.29735
6634	216136	Ilvbl	10994	0.602833983	0.24824
6635	66066	Gng11	2791	1.064019722	0.36021
6636	12669	Chrm1	1128	-0.364805909	0.64691
6637	244049	Mctp2	55784	-0.720270298	0.48346
6638	233863	Gtf3c1	2975	-1.332304132	0.21676
6639	216846	Cntrob	116840	0.25222275	0.991
6640	11655	Alas1	211	1.193312667	0.01764
6641	216134	Pdxk	8566	-0.061227278	0.99988
6642	76650	Srxn1	140809	0.175567676	0.13965
6643	321022	Cdv3	55573	-0.143981421	0.99988
6644	214601	Slc10a3	8273	0.970665863	0.78226
6645	69710	Arap1	116985	0.449455038	0.0636
6646	56741	Igdcc4	57722	-1.740495631	0.16085
6647	243725	Ppp1r9a	55607	-0.509591684	0.40862

6648	110816	Pwp2		0.439448741	0.48182
6649	22063	Trpc1	7220		0.40878
6650	76792	Spring1	79794	0.924775482	0.37009
6651	72446	Prr5l	79899	1.639367804	0.0758
6652	67263	Zswim6	57688	-0.909483915	0.27378
6653	239273	Abcc4	10257	-0.539527012	0.40066
6654	18763	Pkd1	5310	0.182358061	0.78869
6655	18442	P2ry2	5029	0.908409422	0.32134
6656	231672	Fbxw8	26259	-0.141204008	0.31271
6657	228769	Psmf1	9491	-0.35298699	0.5645
6658	69780	Smap2	64744	1.023799153	0.16739
6659	266690	Cyb5r4	51167	0.524581705	0.02585
6660	207212	Arhgef17	9828	-0.211370821	0.8723
6661	74205	Acsl3	2181	0.708822516	0.16844
6662	57785	Rangrf	29098	-0.201933628	0.98861
6663	18046	Nfyc	4802	-0.782778542	0.31799
6664	231670	Fbxo21	23014	0.08046081	0.99988
6665	243659	Styk1	55359	0.670402207	0.21861
6666	20501	Slc16a1	6566	0.74850995	0.19586
6667	67526	Atg12	9140	0.560767947	0.23493
6668	433323	Sgpp2	130367	2.525535961	0.00388
6669	121021	Cspg4	1464	1.214663205	0.1958
6670	269473	Lrig2	9860	0.490684322	0.33906
6671	223272	Itgbl1	9358	2.152023398	0.00447
6672	110920	Hspa13	6782	0.806332814	0.13995
6673	71805	Nup93	9688	0.398156021	0.3273
6674	19395	Rasgrp2	10235	1.225840733	0.00621
6675	67489	Ap4b1		0.969070617	0.01444
6676	23980	Pebp1		0.095172856	0.85735
6677	73916	Ift57	55081	0.276865541	0.38977
6678	14225	Fkbp1a		0.394936891	0.44156
6679	108707	Fam207a	85395	-0.588114726	0.36237
6680	74241	Chpf	79586	0.672338455	0.18932
6681	230700	Foxj3	22887	0.717548878	0.11858
6682	105689	Mycbp2	23077	-0.079232281	0.84997
6683	270086	Ogfod1	55239	1.417364606	0.0079
6684	94093	Trim33	51592	0.114567168	0.89659
6685	18018	Nfatc1	4772	-0.832057615	0.43953
6686	69833	Polr2f	5435	0.167683989	0.99988
6687	69080	Gmppa	29926	0.297800916	0.94068
6688	12583	Cdo1	1036	-0.005678123	0.99988
6689	224171	Cip2a	57650	-1.032480857	0.28041
6690	106877	Afap1l1	134265	-1.3646088	0.08664
6691	72691	Calhm2	51063	1.451513136	0.00738
6692	27008	Micall1	85377	-0.090171771	0.99039
6693	223691	Eif3l	51386	-0.38068432	0.48487
6694	244879	Npat	4863	-0.957465388	0.3222
6695	223690	Ankrd54	129138	0.177442134	0.99988
6696	110078	Pygb	5834	-0.539174006	0.48253
6697	380928	Lmo7	4008	-0.469543925	0.54978

6698	18642	Pfkm	5213	0.039049068	0.97811
6699	14457	Gas7	8522	0.758507177	0.45077
6700	12497	Entpd6	955	-0.583134044	0.33374
6701	223870	Senp1	29843	-1.455273183	0.09756
6702	243653	Clec1a	51267	1.137525529	0.18211
6703	210789	Tbc1d4	9882	0.034504347	0.8603
6704	110253	Triobp	11078	-0.318973665	0.60438
6705	71881	Apmap	57136	0.658482715	0.45957
6706	97961	Nol12		-0.37818003	0.75419
6707	218294	Cdc14b		0.154153615	0.83062
6708	16987	Lss	4047	-0.205957742	0.86729
6709	66432	Slc7a6os	84138	-0.809518297	0.31643
6710	67664	Rnf125	54941	0.183690909	0.99988
6711	218734	830406C13Rik		0.066273392	0.94838
6712	70484	Slc35d2		-0.600674349	0.46964
6713	245860	Atg9a		0.318202786	0.85705
6714	216119	Ybey	54059	-0.214307147	0.86124
6715	106039	Gga1	26088	-0.257963937	0.55018
6716	242126	Slc22a15	55356	-0.170861866	0.82658
6717	208177	Phldb2	90102	0.068414704	0.99988
6718	213012	Abhd10	55347	-0.128824202	0.83176
6719	69171	Cnppd1	27013	-0.105401692	0.79357
6720	11928	Atp1a1	476	-0.507831048	0.46166
6721	72662	Dis3	22894	-0.632216215	0.36393
6722	105844	Card10	29775	-1.553595283	0.17361
6723	23945	Mgll	11343	0.174713302	0.99988
6724	66676	Tmed7		0.09099109	0.93069
6725	76789	Mzt1	440145	-0.02689544	0.99988
6726	212998	BC016579	79669	-0.698307706	0.50224
6727	21846	Tie1	7075	-0.560716103	0.85619
6728	270084	Lpcat2	54947	-0.456713417	0.66292
6729	20203	S100b	6285	0.551438346	0.08304
6730	209683	Ttc28	23331	-0.892753455	0.51487
6731	433470	AA467197	84419	0.755687501	0.17289
6732	65967	Eefsec	60678	0.659990877	0.31233
6733	19354	Rac2	5880	1.307588806	0.00533
6734	74044	Ttf2	8458	0.258870237	0.65784
6735	72193	Scaf11	9169	-0.461810822	0.47572
6736	229644	Trim45	80263	0.257758196	0.7902
6737	77044	Arid2	196528	-0.592171556	0.34794
6738	230676	Szt2	23334	0.517410261	0.59242
6739	67534	Ttll4	9654	-0.358913453	0.4571
6740	67843	Slc35a4	113829	0.550170594	0.9708
6741	19274	Ptprm	5797	1.141676419	0.10438
6742	244585	Rpgrip1l	23322	1.104643325	0.01033
6743	269470	Wdr3	10885	0.113672459	0.99988
6744	72844	Kctd17	79734	0.002103195	0.99989
6745	100608	Noc4l	79050	-0.258946188	0.7398
6746	19268	Ptprf	5792	0.124636213	0.99988
6747	210126	Lpp	4026	-0.616906278	0.37428

6748	17319	Mif	4282	-1.014392526	0.16441
6749	99586	Dpyd	1806	-0.07039536	0.99988
6750	50788	Fbxl8		0.630087975	0.02566
6751	14872	Gstt2		-0.568313619	0.38017
6752	240263	Fem1c	56929	-0.501074405	0.40066
6753	67655	Ctdp1	9150	0.391310657	0.75823
6754	230674	Kdm4a	9682	0.191984502	0.79301
6755	81877	Tnxb	7148	0.26799522	0.74317
6756	13430	Dnm2	1785	-0.35491275	0.48143
6757	54371	Chst2	9435	-0.258837131	0.99988
6758	26398	Map2k4	6416	-0.117696443	0.78664
6759	67775	Rtp4	64108	0.146203936	0.99988
6760	208748	Prrg3		0.879846586	0.01364
6761	319651	Usp37	57695	-0.064209289	0.99988
6762	230673	Ipo13	9670	0.241719569	0.95917
6763	110606	Fntb		0.582173468	0.66176
6764	114301	Palmd	54873	-1.72862915	0.15428
6765	114143	Atp6v0b	533	-0.108039996	0.99988
6766	75964	Trappc8		-0.444883372	0.77068
6767	20321	Frrs1	391059	-0.565076064	0.46988
6768	76499	Clasp2	23122	0.661452887	0.49408
6769	77559	Agl	178	0.126618906	0.99988
6770	214254	Nudt15	55270	0.457301989	0.64453
6771	329506	Ctdspl2	51496	-0.317271293	0.80465
6772	68778	Gucd1	83606	-1.156088235	0.2699
6773	78832	Cacul1	143384	-0.045479166	0.99988
6774	69538	Antxr1	84168	0.625307071	0.64563
6775	140546	Eri3	79033	0.760508093	0.01726
6776	103149	Upb1	51733	1.640753672	0.00785
6777	73724	Mcee	84693	1.173322819	0.00777
6778	57321	Terf2ip	54386	0.097261098	0.99988
6779	107999	Gtpbp6	8225	0.340206933	0.98415
6780	67416	Armcx2	9823	2.948403003	0.00388
6781	229780	Trmt13	54482	-3.165739536	0.03768
6782	74392	Specc1l		-0.373631639	0.7185
6783	67168	Lpar6	10161	0.172955907	0.86113
6784	72536	Tagap		1.977043355	0.00613
6785	235130	Adamts15	170689	0.580790722	0.63083
6786	268564	Zbtb1	22890	0.437481167	0.84526
6787	78248	Armcx1	51309	0.135618546	0.2326
6788	57914	Crlf2	64109	1.081420718	0.01673
6789	70086	Cysltr2		-0.083613541	0.97936
6790	66119	Tomm6	100188893	-0.014729842	0.99988
6791	226252	Fam160b1	57700	-0.53200553	0.37597
6792	319448	Fndc3a	22862	0.432217865	0.67596
6793	226527	Cryzl2		0.761252513	0.01514
6794	217980	Larp4b	23185	-0.425563048	0.34305
6795	229776	Cdc14a	8556	0.041430833	0.99988
6796	327655	Ppip5k1	9677	-0.018831771	0.94838
6797	104718	Ttc7b	145567	-0.707269159	0.34687

6798	12363	Casp4		-0.307137087	0.62388
6799	15932	Idua	3425	0.835801011	0.33861
6800	54324	Arhgef5	7984	-0.652500591	0.47126
6801	235459	Gtf2a2	2958	0.117730288	0.72018
6802	72713	Angptl1	9068	0.852534015	0.1752
6803	170737	Znrf1	84937	-0.617086559	0.48921
6804	69740	Dph5	51611	-0.458122045	0.72513
6805	215015	Fam20b	9917	-0.160573562	0.99988
6806	93686	Rbfox2	23543	0.518422971	0.06348
6807	17920	Myo6	4646	0.531139267	0.62665
6808	338521	Fa2h	79152	1.338680927	0.00988
6809	319765	Igf2bp2	10644	0.102550178	0.99988
6810	17984	Ndn	4692	0.790873499	0.41692
6811	72549	Reep4	80346	1.004077979	0.15601
6812	208943	Myo5c	55930	-2.668287343	0.03974
6813	213469	Lgi3	203190	0.350969913	0.42633
6814	234736	Rfwd3	55159	0.070917119	0.99988
6815	268932	Caskin1	57524	1.374470486	0.08604
6816	75735	Pank1	53354	-0.484843773	0.65086
6817	71751	Map3k13	9175	0.307226002	0.98475
6818	69587	Pcgf3	10336	0.06226868	0.99988
6819	319518	Pdpr	55066	-0.931970902	0.38254
6820	225326	Pik3c3	5289	1.814302376	0.00388
6821	57874	Hacd3	51495	0.365519819	0.53494
6822	225289	AW554918	57536	-0.000941433	0.99988
6823	209018	Vps8	23355	0.387285194	0.52787
6824	234733	Ddx19b	11269	-0.883699521	0.19935
6825	223669	Zfp7	7553	0.774104005	0.82658
6826	74081	Cep350	9857	0.478485719	0.28209
6827	104009	Qsox1	5768	0.845909993	0.02595
6828	22228	Ucp2	7351	0.509429359	0.38798
6829	72482	Acbd6	84320	-0.394967177	0.47061
6830	234730	Fcsk	197258	0.54677817	0.42115
6831	668880	Stard9		0.493601381	0.53643
6832	232187	Smyd5	10322	-0.29955406	0.73374
6833	71375	Foxn3	1112	0.47735819	0.01409
6834	11551	Adra2a	150	2.189406535	0.00407
6835	94282	Sfxn5	94097	0.670043137	0.02191
6836	57257	Vav3	10451	1.101409832	0.19157
6837	223664	Lrrc14	9684	-0.590147159	0.6369
6838	101943	Sf3b3	23450	-0.586928902	0.37246
6839	20751	Spr	6697	0.550821717	0.55165
6840	56299	Fkbpl	63943	-1.813930531	0.08001
6841	102060	Gadd45gip1	90480	-0.361886569	0.48698
6842	76915	Mnd1	84057	0.336611443	0.98154
6843	66704	Rbm4b	83759	0.118094836	0.991
6844	244654	Mtss2	92154	-0.16225198	0.99988
6845	229473	Tmem131l	23240	-0.921260027	0.38618
6846	18190	Nrxn2	9379		0.40878
6847	75914	Exoc6b	23233	0.049609848	0.98983

6848	231571	Rpap2	79871	-0.378245812	0.57652
6849	279572	Tlr13		0.101432772	0.71055
6850	142688	Asb13	79754	-0.188808912	0.99988
6851	233276	Tubgcp5	114791	-1.023659237	0.2586
6852	11977	Atp7a	538	-1.173092299	0.22907
6853	108664	Atp6v1h	51606	-0.831081659	0.31233
6854	105203	Tasor2	54906	-1.136534707	0.15115
6855	384214	Ephx4	253152	-0.667450606	0.44695
6856	211499	Tmem87a	25963	0.063871256	0.63259
6857	208624	Alg3	10195	1.270736378	0.50321
6858	21399	Tcea1	6917	0.140421179	0.99988
6859	73062	Ppp1r16a	84988	-1.379987434	0.18221
6860	27395	Mrpl15	29088	0.204837701	0.99723
6861	217364	Engase	64772	-0.070571612	0.99988
6862	234854	Cdk10	8558	0.186415004	0.99988
6863	16601	Klf9	687	-0.681561067	0.55004
6864	170826	Ppargc1b	133522	-0.138470293	0.99988
6865	19039	Lgals3bp	3959	0.113861853	0.81021
6866	241944	Zfp267		-1.011157449	0.14435
6867	218699	Pxk	54899	0.816610972	0.01511
6868	213582	Map9	79884	-0.240873354	0.99988
6869	26390	Mapkbp1	23005	-1.694089275	0.22875
6870	101565	Ccp110	9738	-0.295482804	0.69432
6871	108672	Zdhhc15	158866	0.780098738	0.29441
6872	72344	Usp36	57602	-0.306255816	0.53208
6873	60596	Gucy1a1	2982	1.03551801	0.44383
6874	68953	Chmp2a	27243	0.224784636	0.81059
6875	56209	Gde1	51573	0.313232981	0.69841
6876	381038	Parl		0.027215638	0.99039
6877	52202	Rbm34	23029	0.100722572	0.99816
6878	22346	Vhl		0.673274336	0.0236
6879	66916	Ndufb7	4713	-0.151133709	0.99814
6880	101314	Brk1	55845	0.251868078	0.08018
6881	29808	Mga	23269	-1.141876185	0.16438
6882	74464	Zswim5	57643	0.099052533	0.94663
6883	28105	Trim36	55521	-0.145467608	0.99988
6884	12316	Aspm	259266	-1.237696378	0.11448
6885	19058	Ppp3r1	5534	0.403186477	0.43386
6886	228140	Tnks1bp1	85456	-1.882903716	0.11842
6887	240185	Jcad	57608	0.82891766	0.02893
6888	269870	Zfp446	55663	0.169095053	0.99988
6889	226470	Zbtb41	360023	0.436103529	0.28209
6890	20502	Slc16a2	6567	0.082955255	0.99988
6891	69263	Rfc3	5983	0.456895086	0.42576
6892	319615	Zfp944		-0.39145545	0.50723
6893	12812	Coil	8161	0.506681027	0.31934
6894	230661	Tesk2	10420	-0.136223138	0.93801
6895	218343	Ttc37	9652	0.209069449	0.991
6896	16525	Kcnk1	3775	-1.190389438	0.2219
6897	66943	Slc66a2	80148	0.01530594	0.99074

6898	244891	Scaper	49855	-0.655264175	0.47329
6899	100710	Pds5b	23047	0.611780037	0.23306
6900	94230	Cpsf1	29894	-0.249879449	0.82473
6901	12461	Cct2	10576	-0.252924533	0.64774
6902	225825	Cd226	10666	1.186157877	0.00773
6903	68925	Rpap1	26015	-0.080328055	0.95476
6904	622665	Ccdc17	149483	0.349583728	0.03204
6905	232237	Fgd5	152273	0.165333162	0.67816
6906	212996	Galnt17	64409	0.015724571	0.99988
6907	17095	Lyl1	4066	-0.141215593	0.93955
6908	77110	Gpbp1l1	60313	-0.132876114	0.78869
6909	18679	Phka1	5255	0.372932607	0.71346
6910	241525	Ypel4	219539		0.991
6911	224143	Poglut1	56983	-0.07738562	0.99988
6912	228136	Zdhhc5	25921	-0.086696178	0.8918
6913	232236	Ccdc174	51244	-0.156898284	0.90048
6914	110611	Hdlbp	3069	0.005328084	0.99988
6915	12388	Ctnnd1		-0.210808759	0.75157
6916	74347	Meak7	57707	0.282840868	0.99058
6917	12460	Ccs	9973	-0.197840848	0.93222
6918	73124	Golim4	27333	-0.210007487	0.76585
6919	212919	Kctd7	154881	-0.298937692	0.588
6920	382620	Tmed8	283578	0.160148069	0.53272
6921	69047	Atp2c2	9914	-1.299659973	0.16387
6922	22324	Vav1	7409	1.596846552	0.01503
6923	22021	Tpst1	8460	1.325544433	0.32222
6924	20382	Srsf2	6427	-0.521211953	0.56772
6925	217734	Pomt2	29954	0.637344591	0.41035
6926	216350	Tspan8	7103	1.150501023	0.00493
6927	70661	Sik3	23387	-0.00369445	0.99988
6928	211446	Exoc3	11336	-0.261338099	0.8303
6929	68142	Ino80	54617	-1.219389744	0.33457
6930	207777	Tspoap1	9256	0.374493771	0.95337
6931	217732	Cipc	85457	0.177721909	0.98477
6932	320184	Lrrc58	116064	-0.64792602	0.52881
6933	71874	Mab21l4	79919	-0.081375357	0.95917
6934	108155	Ogt	8473	-0.588343417	0.59867
6935	20289	Scx	642658	-0.227120171	0.64593
6936	216345	Zfc3h1	196441	-1.652216183	0.16481
6937	140703	Emid1	129080	0.835660156	0.0123
6938	12445	Ccnd3	896	0.350152532	0.60322
6939	238330	Irf2bpl	64207	0.211961698	0.991
6940	14073	Faah	2166	-0.3583135	0.84786
6941	279766	Rhbdd3	25807	-1.008826272	0.21808
6942	207742	Rnf43		-0.331159982	0.991
6943	18195	Nsf	4905	-0.785860266	0.36308
6944	72552	Hsdl1	83693	-0.660182262	0.40878
6945	105513	Chmp7	91782	-0.321365301	0.81366
6946	67678	Lsm3	27258	-1.187036176	0.21288
6947	71843	R3hcc1	203069	-1.964547276	0.10434

6948	78926	Gas2l1	10634	0.510050817	0.08171
6949	72170	Chchd4		0.078424887	0.89572
6950	94352	Loxl2	4017	-0.082928973	0.98901
6951	75668	Rasl10a	10633	0.47261762	0.86509
6952	230648	Efcab14	9813	-0.176713817	0.8393
6953	66258	Mrps17		-0.594197925	0.46959
6954	241158	Ankmy1	51281	0.023162158	0.99988
6955	228545	Vps18	57617	0.315475135	0.33474
6956	11920	Atm	472	-0.730427974	0.44829
6957	228543	Rhov	171177	1.189290578	0.65149
6958	15223	Foxj1	2302	0.105293553	0.84468
6959	78787	Usp54	159195	-0.338874056	0.71204
6960	224139	Golgb1	2804	-0.776704703	0.325
6961	232232	Hdac11	79885	0.440169924	0.34451
6962	353047	Plekhm1	9842	0.638847759	0.20805
6963	67712	Slc25a37	51312	-0.353749002	0.75663
6964	215351	Senp6	26054	-1.059204901	0.23957
6965	55979	Agpat1	10554	-1.034598686	0.29658
6966	544817	Arhgap27	201176	-0.041490843	0.99988
6967	217721	Flvcr2	55640	0.348499072	0.45745
6968	109075	Exosc4	54512	-0.298605545	0.52745
6969	69882	Ints14	81556	0.266224271	0.37037
6970	224454	Zdhhc14	79683	0.030408718	0.99988
6971	53314	Batf	10538	1.546900553	0.00674
6972	72895	Setd5	55209	-1.834548539	0.07102
6973	81703	Jdp2	122953	-0.220620211	0.70368
6974	107829	Thoc5	8563	-0.059912212	0.86412
6975	235086	Igsf9b	22997	0.892408701	0.01344
6976	69408	Dnajc17	55192	-0.783030897	0.36842
6977	14027	Evpl	2125	-0.303011551	0.73063
6978	18082	Nipsnap1	8508	-0.308078777	0.80354
6979	217718	Nek9	91754	0.568319051	0.22296
6980	74019	Traf3ip1	26146	-0.380210163	0.54694
6981	327987	Med13	9969	-0.772539695	0.27799
6982	66306	Fam53c	51307	-0.547903775	0.93374
6983	245902	Ccdc15	80071	-1.343143282	0.26777
6984	74032	Sdr42e1	93517	-0.41770755	0.54293
6985	16571	Kif4	24137	0.945652458	0.35509
6986	232227	Iqsec1	9922	0.1767194	0.85226
6987	66949	Trim59	286827	0.551537853	0.47636
6988	13521	Slc26a2	1836	0.258705479	0.77929
6989	66583	Exosc1	51013	-0.32678049	0.70201
6990	208213	Tmem132c	92293		0.991
6991	105440	Kctd9	54793	0.098858283	0.92365
6992	237911	Brip1	83990	0.749933889	0.3193
6993	234779	Plcg2	5336	0.304839281	0.40878
6994	223773	Zbed4	9889	0.239908043	0.99988
6995	22378	Wbp2	23558	0.347428559	0.3364
6996	12402	Cbl	867	-0.334398499	0.63657
6997	67921	Ube2f		-0.068368833	0.94328

6998	66467	Gtf2h5	404672	-0.969961031	0.17909
6999	70099	Smc4	10051	-0.361598065	0.47971
7000	51801	Ramp1	10267	0.407344081	0.46165
7001	74302	Mtmr3	8897	-0.288001802	0.50521
7002	234577	Cpne2	221184	1.510162569	0.00646
7003	209294	Csta1		-2.374914207	0.06809
7004	225913	Tkfc	26007	0.219158164	0.82841
7005	68842	Tulp4	56995	-0.708895245	0.3837
7006	69544	Wdr5b	54554	0.400070857	0.66016
7007	224833	AI661453	647024	0.315633872	0.74174
7008	74440	Cmip	80790	-0.127571288	0.77772
7009	16878	Lif	3976	-0.312963817	0.991
7010	67946	Spata6	54558	0.414066901	0.04027
7011	18744	Pja1	64219	-0.312954794	0.77977
7012	103724	Tbc1d10a	83874	1.207492591	0.00836
7013	240055	Neurl1b	54492	-0.910348768	0.18862
7014	547253	Parp14	54625	0.320465264	0.68773
7015	68133	Gcsh		0.223171632	0.92099
7016	69020	Zfp707		0.500055967	0.05293
7017	80292	Zxdc	79364	-0.110783085	0.80354
7018	108679	Cops8	10920	-0.018189527	0.99988
7019	238257	Tmem30b	161291	-0.722300981	0.27937
7020	76074	Gbp8		-0.357036948	0.70012
7021	76357	Trmt5	57570	0.314682719	0.76197
7022	225912	Cyb561a3	220002	1.211375878	0.04794
7023	70428	Polr3b	55703	0.071794113	0.89518
7024	245527	Eda2r	60401	0.295462449	0.52779
7025	15957	Ifit1		-0.291207552	0.67789
7026	20474	Six4	51804	-0.927950981	0.33674
7027	18764	Pkd2	5311	0.885647363	0.41413
7028	219151	Scara3	51435	0.239492251	0.99145
7029	140721	Caskin2	57513	0.219161851	0.4679
7030	317717	Sec22a	26984	0.156567338	0.99988
7031	54004	Diaph2	1730	-0.198675188	0.98095
7032	67804	Snx2	6643	0.156001169	0.80049
7033	72565	Uaca	55075	-0.513614035	0.52159
7034	68304	Poglut3	143888	0.666615757	0.82085
7035	67708	Pcnx4	64430	-0.073627086	0.92099
7036	66591	Mad2l1bp	9587	0.882017542	0.01105
7037	70823	Hmgxb4	10042	-0.207537182	0.888
7038	14615	Gjc1	10052	-1.200634235	0.36052
7039	218333	Ice1	23379	-0.29734899	0.62844
7040	50759	Fbxo16	157574	0.16778441	0.87239
7041	232854	Zfp418		0.488171538	0.30673
7042	74522	Morc2a		-0.109068826	0.99988
7043	66880	Rsrc1	51319	-0.866462304	0.30147
7044	230597	Zfyve9	9372	-0.149608804	0.90831
7045	319277	Washc4	23325	-0.1472182	0.73063
7046	72278	Ccpg1	9236	0.29847329	0.99039
7047	71679	Atp5h	10476	0.381383902	0.03343

7048	170835	Inpp5j	27124	0.396478399	0.70446
7049	19249	Ptpn13	5783	-0.654784195	0.43255
7050	208846	Daam1	23002	-0.665006951	0.4432
7051	210106	Tent4a	11044	0.738744268	0.37246
7052	217310	Hid1	283987	0.631199571	0.36647
7053	71523	430429K09Rik		0.409212099	0.56727
7054	338365	Slc41a2	84102	0.617674431	0.06013
7055	17918	Myo5a	4644	1.530752411	0.00819
7056	76448	Ppp1r18	170954	1.340256095	0.0066
7057	76967	700049A03R	9786	-0.92665669	0.24606
7058	67074	Mon2	23041	0.20597025	0.09286
7059	69693	Pof1b	79983	-1.411140698	0.2103
7060	230594	Tut4	23318	-0.905272291	0.22624
7061	58250	Chst11	50515	0.444075892	0.502
7062	319468	Ppm1h	57460	0.00731583	0.99988
7063	216505	Pik3ip1	113791	0.453768594	0.03382
7064	245857	Ssh3	54961	-1.036268152	0.27678
7065	210009	Mtrr	4552	0.590578907	0.03918
7066	216395	Rxylt1	10329	-0.235252108	0.80707
7067	237943	Gpatch8	23131	-1.865035187	0.07119
7068	17068	Ly6d	8581	1.801194027	0.00741
7069	414872	Zyg11b	79699	-0.009513335	0.99988
7070	74729	Setmar		1.725535294	0.00798
7071	99929	Tiparp	25976	-0.442681793	0.65627
7072	217305	Cd300ld		1.640222662	0.00652
7073	106585	Ankrd12	23253	-0.477165054	0.51562
7074	240255	Ythdc2	64848	-0.492865927	0.6504
7075	12286	Cacna1a	773	0.935039404	0.01447
7076	68539	Tmem109	79073	0.374326656	0.51237
7077	140780	Bmp2k	55589	0.35512338	0.55908
7078	16399	Itga2b	3674	0.637643327	0.61403
7079	73192	Xpot	11260	0.25526663	0.36646
7080	18515	Pbx2	5089	0.002369985	0.99988
7081	21665	Tdg	6996	0.029914014	0.99988
7082	56320	Dbn1	1627	0.591343224	0.52331
7083	19826	Rnps1	10921	-0.374441939	0.60288
7084	20350	Sema3f	6405	0.277786401	0.53317
7085	217219	Fam171a2	284069	0.227438229	0.99988
7086	75612	Gns	2799	0.068534283	0.93621
7087	14824	Grn	2896	0.144455935	0.7614
7088	73825	Ppp1r21	129285	0.262573093	0.99988
7089	117160	Ttyh2	94015	0.000424524	0.99988
7090	52837	Tmx4	56255	0.177541996	0.89847
7091	231464	Cnot6l	246175	-0.023042155	0.99656
7092	64657	Mrps10	55173	-0.439281611	0.72479
7093	380921	Dgkh	160851	-1.592246879	0.15006
7094	329416	Nostrin	115677	1.092193276	0.00919
7095	56174	Nagk	55577	-0.171646972	0.99988
7096	328329	Mast4	375449	-1.046945009	0.25214
7097	72053	Tmub2	79089	-0.091286905	0.83434

7098	114606	Tle6	79816	0.309155404	0.91737
7099	399510	Map4k5	11183	0.118670831	0.99988
7100	240672	Dusp5	1847	-1.105784278	0.12926
7101	21886	Tle2	7089	-1.014330899	0.51939
7102	14672	Gna11	2767	-0.025909813	0.99988
7103	106512	Gpsm3	63940	0.985047978	0.04309
7104	19336	Rab24	53917	-0.20581235	0.76344
7105	14676	Gna15	2769	0.258180033	0.76721
7106	68401	G6pc3	92579	-1.001486907	0.24427
7107	108811	Ccdc122	160857	-0.876855903	0.28159
7108	382018	Unc13a	23025	-1.106127879	0.23451
7109	72180	Zfp661	7549	-0.851560559	0.27144
7110	20663	Sos2	6655	0.247919725	0.76067
7111	234407	Colgalt1	79709	-0.09850875	0.937
7112	20272	Scn7a	6332	0.816050293	0.79697
7113	74053	Grip1	23426	-0.987687423	0.21958
7114	269061	Cpsf7	79869	-0.432941868	0.86671
7115	78593	Nrip3	56675	0.021442851	0.99988
7116	269113	Nup54	53371	-0.767122389	0.39896
7117	194388	Tet3	200424	-0.615794272	0.46914
7118	67596	Tespa1		1.042043864	0.19222
7119	67557	Larp6	55323	-0.271405042	0.68141
7120	109979	Art3	419	0.750869181	0.48226
7121	84094	Plvap	83483	-0.634467063	0.45862
7122	73668	Ttc21b	79809	0.133978314	0.80932
7123	69470	Tmem127	55654	0.147716987	0.99397
7124	329910	Acot11	26027	0.464775844	0.62421
7125	73822	Mfsd12	126321	-0.049087255	0.99988
7126	15945	Cxcl10	3627	-0.055926247	0.99816
7127	235493	Fam214a	56204	-0.094774681	0.94224
7128	245886	Ankrd27	84079	0.063059824	0.88714
7129	67938	Myl12b	103910	-0.304134119	0.62094
7130	110959	Nudt19	390916	-0.405128026	0.55816
7131	94065	Mrpl34	64981	0.030459551	0.99988
7132	70312	Cactin	58509	-0.05903263	0.99988
7133	20090	Rps29	6235	0.640243752	0.02032
7134	338337	Cog3	83548	0.117578053	0.99988
7135	70598	Filip1	27145	-0.317464367	0.50116
7136	18717	Pip5k1c	23396	1.619404715	0.07394
7137	319876	Cobll1	22837	-0.015467827	0.99988
7138	215387	Ncaph	23397	-0.236064354	0.99039
7139	214597	Sidt2	51092	-0.789787226	0.38173
7140	72135	Pygo1	26108	-0.786400613	0.63555
7141	234395	Ushbp1	83878	0.980569639	0.10445
7142	27375	Tjp3	27134	-0.250994311	0.93875
7143	230576	Ttc22	55001	-0.097236951	0.94194
7144	74754	Dhcr24	1718	-0.068556062	0.99145
7145	105239	Rnf44	22838	0.620029069	0.24705
7146	20166	Rtkn	6242	-1.418511535	0.26777
7147	217207	Dhx8	1659	-0.966232908	0.25715

7148	66047	Mrpl54	116541	-0.436656014	0.53504
7149	80981	Arl4d	379	-0.406647117	0.61408
7150	217030	Synrg	11276	-1.393094855	0.18211
7151	217203	Tmem106a	113277	1.08656779	0.00734
7152	103406	Zfr2	23217	0.448375365	0.73975
7153	233824	Cog7	91949	0.267126709	0.6023
7154	12606	Cebpa	1050	1.525724254	0.01236
7155	320615	Dop1a	23033	-0.296921229	0.61911
7156	13144	Dapk3	1613	-0.309385674	0.82274
7157	231440	Parm1	25849	-0.805064085	0.32222
7158	15466	Hrh2	3274	0.617642633	0.22688
7159	26949	Vat1	10493	-0.232442241	0.8864
7160	13629	Eef2	1938	-0.18544043	0.72244
7161	14236	Foxn2	3344	1.334452127	0.16878
7162	13482	Dpp4	1803	-0.200853324	0.67404
7163	80719	Igsf6	10261	0.516375326	0.10068
7164	217201	Rundc1	146923	-0.062646987	0.98375
7165	16969	Zbtb7a	51341	0.28101779	0.9383
7166	217578	Baz1a	11177	-0.323503406	0.56753
7167	78658	Ncapd3	23310	-0.820264418	0.57906
7168	26396	Map2k2		-0.46722818	0.55224
7169	20304	Ccl5		0.969481464	0.1495
7170	547176	Zc3h12b	340554	0.671157113	0.05664
7171	215194	Kri1	65095	-0.914081892	0.20724
7172	69010	Anapc13	25847	0.685847028	0.02372
7173	107094	Rrp12	23223	0.010393382	0.99988
7174	106794	Dhx57	90957	-0.601299701	0.40234
7175	245522	Zc4h2	55906	1.723511584	0.00533
7176	13631	Eef2k	29904	0.584856331	0.19626
7177	67013	Oma1	115209	0.530025315	0.31228
7178	210376	Mtmr9	66036	0.744976644	0.3943
7179	56208	Becn1	8678	0.746352019	0.15536
7180	70354	Secisbp2l	9728	-0.562527177	0.40066
7181	232146	Eva1a	84141	0.754427915	0.47454
7182	112407	Egln3	112399	-2.277713464	0.05099
7183	73379	Dcbld2	131566	0.092255154	0.99988
7184	69847	Wnk4	65266	1.246750914	0.01033
7185	330361	Gcfc2	6936	0.502667193	0.52323
7186	242584	Dnai4	79819	-0.07309819	0.9115
7187	11855	Arhgap5	394	-0.645461091	0.2905
7188	75420	Secisbp2	79048	0.168033825	0.1062
7189	76826	Nubpl	80224	-0.137131328	0.991
7190	26905	Eif2s3x		-0.503582401	0.36977
7191	244548	Elmod2	255520	0.538965847	0.45895
7192	71770	Ap2b1	163	-0.593415398	0.53533
7193	17342	Mitf	4286	0.019749776	0.99988
7194	18130	Ints6	26512	-1.114389694	0.18862
7195	244871	Zc3h12c	85463	0.763008622	0.70012
7196	57442	Kcne3	10008	0.744823561	0.01738
7197	66860	Tanc1	85461	-0.133077047	0.92584

7198	66206	110059E24R	138241	-0.109969107	0.99988
7199	217198	Plekhh3	79990	-1.651077574	0.13844
7200	213993	Ccdc186	55088	-0.729365739	0.38642
7201	320487	Heatr5a	25938	0.381724624	0.04439
7202	629059	Fam124a	220108	0.480252671	0.45158
7203	24108	Ubd	10537	0.543768264	0.87891
7204	103733	Tubg1	7283	-0.549317457	0.46007
7205	102436	Lars2	23395	-0.26355955	0.65135
7206	13854	Epn1	29924	-0.6692662	0.35684
7207	73218	Sppl2b	56928	0.298479944	0.94097
7208	276950	Slfn8		-0.182997421	0.78946
7209	230514	Leprot	54741	0.705244203	0.01889
7210	66094	Lsm7	51690	-0.429086476	0.44308
7211	66624	Spcs2	9789	-0.17747357	0.77772
7212	236900	Pdk3	5165	0.227464148	0.99988
7213	70681	Abraxas1	84142	-0.321626884	0.57084
7214	66597	Trim13	10206	0.771640674	0.40797
7215	320271	Scai	286205	-0.079283832	0.99988
7216	50877	Neu3	10825	-0.036304659	0.99988
7217	18245	Oaz1	4946	0.401622819	0.05478
7218	101351	Eogt	285203	0.146066249	0.48488
7219	207304	Hectd1	25831	0.473276229	0.32222
7220	214290	Tut7		-1.041790058	0.11871
7221	320712	Abi3bp	25890	0.803745839	0.31925
7222	18769	Pkig	11142	0.662189912	0.22647
7223	15442	Hpse	10855	0.106915839	0.99988
7224	242570	Raver2	55225	-1.221238262	0.15969
7225	78670	Plekhj1	55111	-1.03322909	0.3837
7226	269855	Ssc5d	284297	-0.330323098	0.87705
7227	11554	Adrb1	153	1.475929743	0.00653
7228	320528	Vps13c	54832	-0.493848361	0.57375
7229	217558	G2e3	55632	0.163894282	0.99988
7230	26891	Cops4	51138	0.177964117	0.85689
7231	26563	Ror1	4919	0.801654156	0.34681
7232		NA		0.500497824	0.06042
7233	432486	Gnptab	79158	0.862708632	0.01306
7234	233552	Gdpd5	81544	-0.257995487	0.75196
7235	69162	Sec31a	22872	0.278821306	0.05067
7236	70611	Fbxo33	254170	0.093866244	0.99988
7237	226154	Lzts2	84445	-0.516262528	0.64563
7238	69736	Nup37	79023	0.14880923	0.74567
7239	20293	Ccl12		0.996065349	0.25731
7240	78610	Uvrag	7405	0.199360405	0.99988
7241	80859	Nfkbiz	64332	0.710323951	0.22364
7242	55983	Pdzrn3	23024	-0.508237356	0.73763
7243	74386	Rmi1	80010	0.882355123	0.00959
7244	72056	810055G02R	53838	0.96493407	0.421
7245	20306	Ccl7	6354	1.788465842	0.00504
7246	70757	Hacd2	201562	-0.456713417	0.70138
7247	72171	Shq1	55164	0.330620843	0.64262

7248	18554	Pcsk7	9159	-0.698070873	0.46022
7249	20296	Ccl2	6347	2.180025726	0.00613
7250	227801	Dennd1a	57706	-0.539471374	0.65711
7251	118445	Klf16	83855	-1.226185347	0.32449
7252	66680	Oser1	51526	0.400550167	0.62454
7253	233545	Emsy	56946	-0.325445434	0.39201
7254	103743	Tmem98	26022	0.298272992	0.75567
7255	227800	Rabgap1	23637	0.511114319	0.02661
7256	76478	Haus8	93323	0.852618788	0.49281
7257	338367	Myo1d	4642	0.017834568	0.99988
7258	77862	Thyn1	29087	-0.297547682	0.72791
7259	15375	Foxa1	3169	-0.000517594	0.99988
7260	60530	Fignl1	63979	-0.80695193	0.77878
7261	77630	Prdm8	56978	-0.824197178	0.43664
7262	21954	Tnni3	7137	0.559066532	0.01364
7263	71330	Rcbtb1	55213	0.69549533	0.05186
7264	319625	Galm	130589	0.582724618	0.04337
7265	66724	Tab3	257397	-0.154437343	0.99546
7266	17192	Mbd3	53615	-0.744712929	0.34574
7267	21810	Tgfbi	7045	1.014546353	0.00812
7268	272027	Tstd2	158427	-1.228030265	0.0991
7269	109332	Cdcp1	64866	-0.54588073	0.40878
7270	70335	Reep6		-1.077057812	0.22887
7271	231430	Cox18	285521	-0.420045547	0.67112
7272	171286	Slc12a8	84561	0.12422676	0.98452
7273	213311	Fbxl21		-1.051869905	0.21028
7274	100121	Tdrd7	23424	0.779496143	0.24548
7275	214505	Gnptg	84572	0.623243888	0.11625
7276	72843	Prdm4	11108	-0.901967102	0.33906
7277	20918	Eif1		0.101685861	0.52521
7278	232798	Leng8	114823	-0.377404031	0.87562
7279	12337	Capn5	726	0.15015735	0.5187
7280	16667	Krt17	3872	-0.577272342	0.91942
7281	234384	Mpv17l2	84769	-0.454964358	0.68632
7282	72535	Aldh1b1	219	2.033118465	0.00561
7283	219228	Pcdh17	27253	-0.518238209	0.06712
7284	77087	Ankrd11	29123	-1.39598244	0.12682
7285	242418	Dcaf10	79269	-0.212211173	0.9979
7286	216987	Utp6	55813	0.652189368	0.03814
7287	69707	Iqcg	84223	1.320119666	0.14446
7288	66078	Tsen34	79042	0.282776214	0.65504
7289	69770	Fam174c	55009	1.366503439	0.15428
7290	77582	Mboat7	79143	0.310826522	0.6177
7291	328110	Prpf39	55015	-0.392454886	0.52149
7292	69934	Trmt10b	158234	0.346759886	0.66609
7293	328108	Togaram1	23116	0.830941941	0.01386
7294	237422	Ric8b	55188	0.843570731	0.02064
7295	59090	Midn	90007	-0.906513604	0.27147
7296	233532	Rsf1	51773	-0.456444794	0.43504
7297	100502698	Rubcn	9711	0.348324811	0.35657

7298	232791	Cnot3	4849	-1.096635505	0.15302
7299	76238	Grhpr	9380	-0.551028427	0.40878
7300	224116	Muc20	200958	-0.234175396	0.99988
7301	100503659	Cbarp	255057	-0.53248439	0.41035
7302	66273	Aamdc	28971	0.23581045	0.99988
7303	319885	Zcchc7	84186	-0.608041668	0.3364
7304	269252	Gtf3c4	9329	0.26487365	0.40512
7305	212168	Zswim4	65249	-0.319837383	0.84997
7306	216161	Sbno2	22904	-0.354315029	0.74146
7307	66091	Ndufa3	4696	0.441359247	0.12716
7308	17279	Melk	9833	-0.856595353	0.49209
7309	21835	Thrsp	7069	0.72678721	0.10932
7310	100038882	Isg15	9636	-0.533571876	0.93801
7311	73469	Rnf38	152006	-0.143411319	0.98653
7312	70719	Arhgap45	23526	0.770273108	0.04448
7313	106407	Slc51a	200931	0.12905572	0.26929
7314	381903	Alg8	79053	-0.603445196	0.67402
7315	27261	Dok3	79930	0.799011042	0.33249
7316	244144	Usp35	57558	-1.721291909	0.13768
7317	27403	Abca7	10347	-0.177208023	0.66181
7318	19108	Prkx	5613	-0.070537892	0.90774
7319	114741	Supt16	11198	-1.129665314	0.24121
7320	269060	Dagla	747	0.65840453	0.27733
7321	216156	Wdr18	57418	0.489425916	0.45316
7322	223776	Selenoo	83642	-1.32475681	0.15062
7323	72745	Tmem161b	153396	-1.064291732	0.2856
7324	268882	Fbxo45	200933	0.139569929	0.75583
7325	69190	Dym	54808	-0.744915875	0.29894
7326	234663	Dync1li2	1783	0.30896343	0.2673
7327	118451	Mrps2	51116	-0.048426612	0.97042
7328	171486	Cd99l2		-1.525041953	0.13285
7329	14594	Ggta1		0.866332254	0.84198
7330	109284	R3hdm4	91300	-0.398204546	0.71828
7331	11475	Acta2	59	2.499689035	0.00591
7332	66994	Cep19	84984	-0.231171957	0.92721
7333	320150	Zdhhc17	23390	-0.218518078	0.79392
7334	22160	Twist1	7291	-0.988906717	0.28283
7335	226245	Plekhs1	79949	0.52599343	0.63927
7336	57813	Tk2	7084	-0.647359162	0.32302
7337	223775	Pim3	415116	0.252534085	0.06277
7338	241289	Ppp1r26	9858	0.097901924	0.99988
7339	67486	Polr3g		-1.22581991	0.22688
7340	216152	Plppr3	79948	-0.084441323	0.94737
7341	80289	Lysmd3	116068	0.40426464	0.83773
7342	320209	Ddx11	1663	-0.569784318	0.61911
7343	15931	Ids		0.753848061	0.25638
7344	231386	Ythdc1	91746	-1.096201768	0.18862
7345	78906	Misp	126353	-0.346641986	0.84089
7346	18483	Palm	5064	-0.082970679	0.991
7347	73229	Zfp983		0.986358465	0.24197

7348	114774	Pawr	5074	-1.645179379	0.06049
7349	320799	Zhx3	23051	0.169808121	0.99988
7350	235386	Hykk	123688	-0.251798332	0.99988
7351	12868	Cox8a	1351	0.001824225	0.99988
7352	70294	Rnf126	55658	-0.018441548	0.99988
7353	223753	Cerk	64781	0.640005353	0.0681
7354	231380	Uba6	55236	0.169345905	0.46414
7355	223752	Gramd4	23151	0.569648893	0.93069
7356	19347	Dennd5a	23258	0.393911872	0.66181
7357	102657	Cd276	80381	0.652420616	0.83649
7358	319845	Bbs9	27241	0.43344203	0.44425
7359	15015	H2-Q4		-0.43011507	0.62211
7360	238123	Cog5	10466	-0.12223313	0.91027
7361	208076	Pknox2	63876	-1.590709519	0.1604
7362	214579	Aldh5a1	7915	-0.726283796	0.32082
7363	108837	Ibtk	25998	-0.020091836	0.97146
7364	239570	Ttc38	55020	-0.342724622	0.52703
7365	380660	Acss3	79611	0.195798685	0.99988
7366	30050	Fbxw2	26190	0.383555438	0.25789
7367	219024	Pip4p1	90809	0.11255034	0.84743
7368	238130	Dock4	9732	-0.89829187	0.21646
7369	56196	Tdp2	51567	-0.273120157	0.88714
7370	236790	Ints6l	203522	-0.223518459	0.92332
7371	100213	Rusc2	9853	-0.057453564	0.99988
7372	216742	Fnip1	96459	0.171600092	0.99988
7373	230088	Fam214b	80256	-0.189433944	0.98116
7374	193385	Ripor2	9750	-0.023000884	0.99988
7375	216292	Mettl25	84190	0.066735315	0.991
7376	278279	Tmtc2		-0.469274569	0.46959
7377	78755	Fam122b	159090	0.495667365	0.92973
7378	11546	Parp2	10038	0.06561919	0.89818
7379	224807	Tmem63b	55362	-3.51919132	0.02355
7380	223739	031439G07R	23313	0.573206437	0.19796
7381	56323	Dnajb5	25822	-0.441951746	0.87223
7382	71409	Fmnl2	114793	-0.358331697	0.73163
7383	234373	Sugp2	10147	0.465936581	0.52065
7384	104831	Ptpn23	25930	-0.277077776	0.58874
7385	71726	Smug1		0.24734375	0.89195
7386	227659	Slc2a6	11182	-0.562993747	0.64162
7387	14430	Galt	2592	0.420972543	0.31839
7388	18391	Sigmar1	10280	0.094217455	0.61072
7389	226409	Zranb3	84083	-0.663120101	0.66836
7390	75991	Slain2	57606	0.612213601	0.02032
7391	75423	Arl5a	26225	0.112946675	0.99988
7392	226151	Slf2	55719	-0.627255157	0.38343
7393	215008	Vezt	55591	-0.549414162	0.36204
7394	140792	Colec12	81035	0.661579851	0.41617
7395	226407	Rab3gap1	22930	0.019327027	0.98934
7396	171170	Mbnl3	55796	-0.616667832	0.19222
7397	56307	Metap2	10988	-0.555048083	0.36964

7398	69961	Rpp25l	138716	0.023609252	0.99988
7399	19727	Rfxank	8625	-0.553010724	0.54842
7400	104943	Fam110c	642273	1.472403217	0.02459
7401	113868	Acaa1a		0.238890102	0.40878
7402	17286	Meox2	4223	0.600844445	0.43658
7403	107895	Mgat5	4249	-0.466088791	0.64361
7404	106042	Prickle1	144165	0.578008242	0.46868
7405	20935	Surf6	6838	-0.805373953	0.28532
7406	223828	Pphln1		0.446200063	0.02777
7407	66042	Sostdc1	25928	1.944248136	0.00646
7408	74603	Cd200r3		2.815075451	0.00388
7409	234366	Gatad2a	54815	-0.40642721	0.47454
7410	50708	H1f2	3006	-0.590569898	0.4679
7411	56279	Dipk1b	138311	0.562442858	0.49286
7412	217473	Ankmy2	57037	-0.370562887	0.92343
7413	225998	Rorb	6096	-1.961292054	0.04341
7414	223827	Gxylt1	283464	-0.923741434	0.30415
7415	67184	Ndufa13	51079	0.707436816	0.07133
7416	51869	Rif1	55183	-0.570226669	0.40182
7417	98402	Sh3bp4	23677	-0.96071995	0.25791
7418	212547	Nepro	25871	0.371941792	0.66633
7419	76416	Polr1has		-0.865079548	0.26063
7420	66468	Ska1	220134	-0.890153233	0.3068
7421	106931	Kctd1	284252	0.108707066	0.99988
7422	67615	Ube2r2	54926	0.041184004	0.99988
7423	67419	Armh4	145407	1.241525638	0.00883
7424	78816	Gmip	51291	-0.498909616	0.89849
7425	71684	Rbm43	375287	0.680580339	0.89195
7426	29817	Igfbp7	3490	1.776854075	0.00577
7427	67452	Pnpla8	50640	0.364000594	0.60462
7428	234699	Edc4	23644	-0.120413109	0.80267
7429	66725	Lrrk2	120892		0.40878
7430	213673	530068E07R	56951	0.281338505	0.67674
7431	227644	Snapc4	6621	-0.061061848	0.98957
7432	70646	Naa30	122830	-0.031306515	0.99988
7433	56412	Noa1	84273	-0.163266307	0.88821
7434	74385	Ap5m1	55745	1.476320227	0.08919
7435	207798	Gramd1c	54762	0.809847904	0.6037
7436	407819	BC031181	497661	-0.141919419	0.8614
7437	332175	Zdhhc23	254887	-0.581965976	0.46873
7438	21402	Skp1	6500	-0.264576145	0.49865
7439	66136	Polr1h	30834	0.370681617	0.53504
7440	66661	Srp72	6731	-1.138029434	0.24732
7441	227638	Qsox2	169714	-0.556034337	0.40878
7442	77480	Kidins220	57498	-0.541680559	0.46714
7443	242050	Igsf10	285313	0.769282784	0.8433
7444	218989	Tmem260	54916	0.099529741	0.99988
7445	98766	Ubac1	10422	-0.032925925	0.99988
7446	70839	P2ry12	64805	2.572755652	0.00388
7447	234356	Csgalnact1		0.185473414	0.991

7448	74191	P2ry13	53829	-0.46649162	0.10474
7449	381110	Rmdn2	151393	-0.707868767	0.37904
7450	66870	Serbp1	26135	0.015605983	0.99988
7451	69038	Tmem258	746	0.369370323	0.68773
7452	30946	Abt1	29777	0.175327925	0.88887
7453	140795	P2ry14	9934	1.817240369	0.00406
7454	13197	Gadd45a	1647	-0.50233858	0.44474
7455	77371	Sec24a	10802	-0.003518195	0.99988
7456	244757	Glb1l2	89944	0.996244309	0.14334
7457	76497	Ppp1r11	6992	-0.271274768	0.61453
7458	14701	Gng12	55970	-0.28253788	0.55446
7459	381644	Cep135	9662	-0.017142958	0.99988
7460	545260	Arsi	340075	1.666689795	0.00653
7461	14751	Gpi1	2821	-0.552324016	0.40234
7462	72726	Tbcc	6903	1.087156607	0.00846
7463	20439	Siah2	6478	0.284005462	0.75452
7464	69940	Exoc1	55763	0.261730863	0.66436
7465	12314	Calm2	805	-0.48935975	0.62803
7466	59016	Thap11	57215	0.773274733	0.00934
7467	17022	Lum	4060	1.44129137	0.00837
7468	319594	Hif1an	55662	-0.316221276	0.49564
7469	71302	Arhgap26	23092	1.13361526	0.01143
7470	101543	Wtip	126374	-0.022384794	0.96528
7471	13709	Elf1	1997	-0.676033128	0.29783
7472	214058	Megf11	84465	0.63707918	0.41828
7473	72925	Marchf1	55016	2.313637877	0.00388
7474	224617	Tbc1d24		-0.754265642	0.44695
7475	12226	Btg1	694	-0.751783495	0.28553
7476	216238	Eea1	8411	-0.701797367	0.32981
7477	225358	Fam13b	51306	-1.058984054	0.25338
7478	245386	Tmem255a		0.538703623	0.05185
7479	24017	Rnf13	11342	0.215151566	0.75231
7480	75454	Phpt1	29085	-0.693891565	0.45461
7481	52245	Commd2	51122	0.51300013	0.32151
7482	108723	Card11	84433	0.928452371	0.27378
7483	19024	Ppfibp2	8495	0.118699362	0.25495
7484	77980	Sbf1	6305	-0.593516094	0.43205
7485	260409	Cdc42ep3	10602	0.488815042	0.95449
7486	234595	Slc38a7	55238	0.108377873	0.99053
7487	69942	Rnf113a1		-0.155977944	0.864
7488	216725	Adamts2	9509	0.309514267	0.63927
7489	234594	Cnot1	23019	-0.740855943	0.28283
7490	381062	Ermard		0.495344024	0.08769
7491	231147	Sh3tc1	54436	0.023436661	0.82032
7492	74239	Iqce		-0.377182379	0.91641
7493	243914	Lgi4	163175	-0.445510687	0.72989
7494	71474	Ppp6r2	9701	-1.319290498	0.27081
7495	234593	Ndrg4	65009	1.526634275	0.13229
7496	78339	Ttyh3	80727	0.420357029	0.42576
7497	210982	Bicral	23506	0.056113148	0.99894

7498	56188	Fxyd1	5348	0.618799013	0.92178
7499	68134	Upf3b	65109	-1.092575422	0.23057
7500	229285	Spg20	23111	0.845889584	0.01919
7501	14164	Fgf1	2246	-0.301499309	0.63198
7502	71435	Arhgap21	57584	-0.40103292	0.91975
7503	14960	H2-Aa		2.544249944	0.00388
7504	242939	Cpz	8532	-0.034783652	0.99382
7505	140570	Plxnb2	23654	-0.714861018	0.30608
7506	67484	Eepd1	80820	0.70297009	0.16487
7507	380752	Eipr1	7260	-0.062866786	0.87071
7508	170767	Rfxap	5994	-0.538182036	0.41475
7509	208618	Etl4	56243	-0.134129057	0.85992
7510	103534	Mgat4b	11282	0.864521404	0.34692
7511	74772	Atp13a2	23400	1.193349805	0.01002
7512	66248	Alg5	29880	-0.227348173	0.84859
7513	26373	Clcn7	1186	-0.152111069	0.75288
7514	17766	Nudt1	4521	-0.003108526	0.991
7515	227933	Ccdc148	130940	3.549472905	0.00356
7516	76795	Tbc1d9b	23061	-0.217967874	0.98982
7517	227619	Man1b1	11253	-0.171486685	0.80138
7518	105841	Dennd3	22898	-0.499341122	0.81382
7519	77574	Tcaf1	9747	-0.328305816	0.78084
7520	237500	Tmtc3	160418	-1.044609105	0.71217
7521	223921	Aaas	8086	-0.060378025	0.99988
7522	212139	Cc2d1a	54862	-0.704145453	0.51011
7523	231832	Tmem184a		0.38917581	0.40878
7524	75416	Nop14	8602	-0.408607117	0.45158
7525	239528	Ago2	27161	-0.912035065	0.26929
7526	12283	Cab39	51719	-0.615745567	0.39707
7527	74256	Cyld	1540	0.599188213	0.03563
7528	231830	Micall2	79778	-0.351202794	0.63869
7529	22758	Zscan12	9753	-0.057772299	0.99988
7530	68035	Rbm42	79171	-0.08639618	0.99988
7531	108737	Oxsr1	9943	-1.035984823	0.33661
7532	70892	Ttll7	79739	0.050813916	0.99988
7533	67116	Cuedc2	79004	-0.247656899	0.64985
7534	110323	Cox6b1	1340	0.662278175	0.01704
7535	227613	Tubb4b	10383	0.177190567	0.80163
7536	30045	Dnajc12	56521	-0.45808187	0.89398
7537	227325	Dner	92737	2.494141695	0.00544
7538	209737	Kif15	56992	-0.454533545	0.65236
7539	72404	Wdr44	54521	1.380544906	0.16593
7540	26378	Decr2	26063	-0.431020642	0.75164
7541	68743	Anln	54443	-0.289288116	0.89659
7542	214627	Tent4b	64282	0.223498434	0.7324
7543	67941	Rps27l	51065	0.318004801	0.14885
7544	67455	Klhl13	90293	0.640197123	0.87025
7545	109241	Mbd5	55777	0.210965473	0.99988
7546	241275	Noxa1	10811	-0.585594175	0.48431
7547	382030	Cnep1r1	255919	-0.808415927	0.81378

7548	72090	Entpd8	377841	-2.788221485	0.0616
7549	77053	Sun1	23353	0.739550131	0.01504
7550	194952	Jmjd4	65094	0.425590155	0.70675
7551	245847	Amdhd2		0.108977681	0.99988
7552	106021	Topors	10210	-0.564516365	0.4679
7553	99167	Ssx2ip	117178	-0.615025718	0.43916
7554	101883	Igflr1	79713	0.90406206	0.16581
7555	241274	Pnpla7	375775	0.977159038	0.40787
7556	66340	Psenen		0.108988313	0.83409
7557	20437	Siah1a		0.404241281	0.96779
7558	75660	Lin37	55957	-0.011028591	0.99988
7559	107733	Mrpl41	64975	0.383532489	0.59875
7560	243912	Hspb6	126393	0.384088952	0.991
7561	22417	Wnt4	54361	0.309859819	0.6985
7562	19208	Ptcra	171558	0.497415514	0.91936
7563	67212	Mrpl55	128308	0.886212039	0.23179
7564	233651	Dchs1	8642	0.274542594	0.72018
7565	214804	Syde2	84144	-0.06791323	0.991
7566	17130	Smad6	4091	0.794732401	0.22638
7567	66421	410004B18Ri	148423	0.27374246	0.69501
7568	327762	Dna2	1763	-0.055490232	0.93453
7569	102093	Phkb	5257	-0.024634026	0.99988
7570	52538	Acaa2	10449	-0.500062508	0.59996
7571	233071	Arhgap33	115703	1.146444544	0.45745
7572	622434	Arhgef26	26084	-0.973455833	0.37664
7573	12259	C1qa	712	1.819149837	0.00439
7574	227835	Gtdc1	79712	-0.554818798	0.48791
7575	77683	Ehmt1	79813	-0.579161647	0.3801
7576	74012	Rap2b	5912	-0.359646592	0.80332
7577	12262	C1qc	714	2.136174399	0.00447
7578	72154	Zfp157		-0.718643175	0.65774
7579	74513	Neto2	81831	-0.573250027	0.93665
7580	14370	Fzd8	8325	1.047035767	0.33673
7581	12260	C1qb	713	3.088679754	0.00388
7582	54445	Unc93b1	81622	1.53636938	0.00613
7583	225049	Ttc7	57217	0.254576814	0.3425
7584	243910	Nfkbid	84807	-1.270211765	0.01228
7585	26926	Aifm1	9131	-0.347583202	0.91809
7586	66714	921524J17Ri	388272	-0.561132048	0.6464
7587	99982	Kdm1a	23028	-0.715841361	0.34681
7588	114615	Elac1	55520	-1.662039374	0.11193
7589	235442	Rab8b	51762	0.651658689	0.19626
7590	213068	Tmem71	137835	0.050238775	0.98477
7591	231807	Map11	55262	0.708686091	0.02754
7592	72320	Kifbp	26128	0.454784282	0.02793
7593	320376	Bcorl1	63035	0.194626457	0.99024
7594	223918	Spryd3	84926	-0.489429083	0.63032
7595	66455	Cnpy4	245812	1.123578181	0.01134
7596	66343	Tmem177	80775	0.991314995	0.0362
7597	68999	Anapc10	10393	0.721402287	0.34612

7598	21343	Taf6	6878	0.152832036	0.99988
7599	208884	Zdhhc9	51114	0.228345278	0.07241
7600	18854	Pml	5371	-0.557191698	0.57303
7601	55992	Trim3	10612	-0.172193951	0.86848
7602	73945	Otud4	54726	-0.089582297	0.99988
7603	56488	Nxt1	29107	0.628924259	0.40878
7604	230837	Asap3	55616	-0.297901706	0.38017
7605	22698	Zfp39	148268	0.577872594	0.77563
7606	209039	Tns2	23371	0.931490242	0.01182
7607	170745	Xpnpep2	7512	0.568212689	0.95766
7608	56314	Zfp113	10794	-0.071665221	0.99988
7609	15275	Hk1	3098	-0.228551577	0.77977
7610	268996	Ss18	6760	0.5972937	0.25325
7611	22697	Zscan21	7589	0.436702951	0.35657
7612	109136	Mmaa	166785	-0.076490641	0.99988
7613	70423	Tspan15	23555	-0.129553502	0.8433
7614	16324	Inhbb	3625	-0.156757715	0.99039
7615	20597	Smpd1	6609	0.268164175	0.43497
7616	67869	Paip2	51247	0.233244842	0.40308
7617	109042	Cavin3	112464	1.481708883	0.0079
7618	54673	Sh3glb1	51100	-0.140872374	0.78093
7619	19656	Rbmxl1		0.406051019	0.02279
7620	20249	Scd1		-1.146377921	0.2066
7621	93684	Selenof	9403	0.32844266	0.99988
7622	75841	Rnf139	11236	-1.328217106	0.14009
7623	68260	Trmt12	55039	-0.881128257	0.20014
7624	73836	Slc35b2	347734	-0.158440688	0.75164
7625	76905	Lrg1	116844	-1.73108972	0.24468
7626	215445	Rab11fip3	9727	-0.938174052	0.30673
7627	212123	Dcaf15	90379	-0.400945479	0.31763
7628	56468	Socs5	9655	-0.052660815	0.90866
7629	241694	Ralgapa2	57186	-0.494543503	0.3702
7630	73251	Setd7	80854	-0.175607527	0.63555
7631	210998	Fam91a1	157769	-0.05825316	0.99988
7632	73728	Psd	5662	0.238499877	0.04341
7633	214531	Tmprss13	84000	-0.060916507	0.94557
7634	102182	Prmt9	90826	0.231264781	0.91737
7635	16764	Aff3	3899	-0.783278933	0.45453
7636	78354	Lypd8l		0.197825875	0.99988
7637	78514	Arhgap10	79658	-0.483004169	0.53005
7638	104721	Ddx1	1653	-0.154094976	0.8569
7639	216011	Lrrc20	55222	-0.205287283	0.71565
7640	66377	Ndufc1	4717	0.457243552	0.49462
7641	67749	Mgarp	84709	0.284978354	0.99988
7642	68458	Ppp1r14a	94274	0.958327476	0.32724
7643	243780	Dennd11	57189	-0.606759381	0.22927
7644	69257	Elf2	1998	0.043557982	0.99034
7645	74127	Krt80	144501	0.097063509	0.99988
7646	56368	Cyb561d2	11068	-0.289134136	0.71697
7647	69310	Pacrg	135138	0.724471944	0.01753

7648	76938	Rbm17	84991	-0.057156167	0.99053
7649	68118	Atg101	60673	-0.823602819	0.55251
7650	26968	Islr	3671	-0.570749038	0.54938
7651	231128	Fam193a	8603	-0.893326454	0.23747
7652	24063	Spry1	10252	-0.377971113	0.991
7653	73754	Thap1	55145	1.02259463	0.01296
7654	623661	Lipt1	51601	-1.180430502	0.13804
7655	68929	Mospd3	64598	-0.407656702	0.9565
7656	14173	Fgf2	2247	1.468077759	0.00783
7657	320191	Hook3	84376	-0.689033274	0.4129
7658	17122	Mxd4	10608	-0.643885846	0.32151
7659	17184	Matr3		-0.648762073	0.40867
7660	29876	Clic4	25932	0.289718138	0.34207
7661	103836	Zfp692	55657	-0.480024656	0.57695
7662	74653	Pomk	84197	1.094060536	0.00972
7663	240396	Mex3c	51320	0.283568541	0.74534
7664	16425	Itih2	3698	-2.081042604	0.07834
7665	66939	Aagab	79719	0.047381601	0.99988
7666	52120	Hgsnat	138050	-0.682010994	0.42617
7667	16588	Kin	22944	-1.53738729	0.18792
7668	27981	Rsrp1	57035	0.373791804	0.43562
7669	229227	932438A13R	84162	-0.755354697	0.26317
7670	216766	Gemin5	25929	0.279340319	0.98387
7671	69071	Tmem97	27346	-1.495131219	0.19207
7672	107586	Ovol2	58495	0.359621689	0.37035
7673	207839	Galnt6	11226	-1.005605717	0.26933
7674	20842	Stag1	10274	-0.443023541	0.43953
7675	272589	Tbcel		-0.676504791	0.32405
7676	100017	Ldlrap1	26119	-1.080364391	0.29412
7677	67207	Lsm1	27257	-0.179049645	0.89968
7678	234875	Ttc13	79573	-0.212500278	0.61583
7679	230815	Man1c1	57134	-0.022161972	0.99356
7680	21335	Tacc3	10460	-0.311933245	0.54734
7681	382207	Jade3	9767	0.636223934	0.23823
7682	67384	Bag4	9530	0.564155324	0.49281
7683	215243	Traf3ip3	80342	0.775415932	0.23011
7684	21354	Tap1	6890	-0.021767564	0.99988
7685	71492	Bbs7	55212	-0.218529428	0.86412
7686	50817	Capn15	6650	-1.088842936	0.24307
7687	73158	Larp1	23367	-1.188810184	0.22535
7688	26411	Map4k1	11184	-1.219238663	0.21537
7689	74504	Fam53a	152877	-0.051104126	0.99779
7690	236727	Slc9a7	84679	0.659010447	0.88372
7691	319944	Taf2	6873	-1.297827552	0.24003
7692	83704	Slc12a9	56996	0.135800955	0.96307
7693	60322	Chst7	56548	0.271990031	0.35319
7694	71904	Paqr7	164091	0.165482297	0.84693
7695	68323	Nudt22	84304	-0.344724703	0.67665
7696	226977	Actr1b	10120	0.354703342	0.53603
7697	68614	Letmd1	25875	1.693192764	0.00504

7698	71101	Uvssa	57654	-0.792416556	0.4432
7699	66055	Sf3b6	51639	0.551552116	0.33739
7700	18133	Ccn3	4856	1.949807436	0.0436
7701	270035	Letm2	137994	-0.035134931	0.99988
7702	83701	Srrt	51593	-1.110468032	0.23454
7703	100163	Pafah2	5051	0.264706864	0.99988
7704	22289	Kdm6a	7403	-0.757574197	0.27828
7705	18605	Enpp1	5167	0.137172426	0.91406
7706	13016	Ctbp1	1487	-0.332722601	0.48802
7707	226861	Hhat	55733	0.015070824	0.99988
7708	66926	Trmt6	51605	-0.200593957	0.84191
7709	100689	Spon2	10417	0.303186125	0.99988
7710	116838	Rims2	9699	-1.080287721	0.3943
7711	214742	Rcor3	55758	0.044935929	0.99988
7712	76295	Atp11b	23200	-0.473463961	0.40878
7713	15894	Icam1	3383	0.51164163	0.02387
7714	330723	Htra4		1.379290487	0.00952
7715	94220	Cnnm4	26504	0.941526039	0.25723
7716	67016	Tbc1d2b	23102	0.293480881	0.62837
7717	18787	Serpine1	5054	1.458493944	0.00817
7718	74334	Ranbp10	57610	-0.78350729	0.37342
7719	240283	Dmxl1	1657	-0.417685299	0.91614
7720	24115	Best1	7439	0.722742027	0.02688
7721	71946	Endod1	23052	-0.838804625	0.24984
7722	277854	Depdc5		-0.469107542	0.75611
7723	22782	Slc30a1	7779	0.15116368	0.84786
7724	22361	Vnn1	8876	0.056203756	0.99988
7725	70012	Cep85	64793	-0.050766746	0.99988
7726	214855	Arid5a	10865	-0.277160757	0.76344
7727	76306	Slc18b1	116843	0.354518158	0.10785
7728	54375	Azin1	51582	0.164186063	0.78398
7729	77065	Ints7	25896	-0.002611517	0.99988
7730	21847	Klf10	7071	-0.046239495	0.99988
7731	104732	Tedc1	283643	0.471388534	0.91487
7732	320011	Uggt1	56886	-0.151407269	0.99988
7733	76843	Dtl	51514	-0.052317135	0.99988
7734	331401	Thoc2	57187	-0.36756233	0.46908
7735	75302	Asxl2	55252	0.325940739	0.99988
7736	70790	Ubr5	51366	-0.595886675	0.3876
7737	353169	Slc2a12	154091	-0.732233934	0.41444
7738	56506	Cib2	10518	0.765022957	0.02267
7739	66208	Nenf	29937	-0.197366635	0.99988
7740	214469	Fam168b	130074	0.234185836	0.45862
7741	226970	Arhgef4		-0.752540946	0.35536
7742	74450	Pank2	80025	0.243483528	0.15929
7743	233977	Ppfia1	8500	-0.219206794	0.90295
7744	228607	Mavs	57506	0.149330926	0.97993
7745	75284	Bcdin3d	144233	-0.194768279	0.88398
7746	100504663	Atg14	22863	0.120231592	0.991
7747	74600	Mrpl47	57129	-0.282871321	0.99448

7748	192786	Rapgef6		0.393086829	0.76504
7749	78938	Fbxo34	55030	0.016585484	0.98477
7750	210274	Shank2	22941	-0.596932999	0.76849
7751	218977	Dlgap5	9787	-0.65926946	0.18934
7752	15000	H2-DMb2		-0.887536711	0.60462
7753	101497	Plekhg2	64857	0.571540705	0.0421
7754	503610	Zdhhc18	84243	-0.668482668	0.53504
7755	20055	Rps16	6217	0.25943443	0.71893
7756	51812	Mcrs1	10445	0.062540838	0.95917
7757	22057	Tob1	10140	-0.274803385	0.95476
7758	14528	Gch1	2643	1.252259476	0.0059
7759	217887	Clba1	122616	-0.250473379	0.99847
7760	69073	Kdf1	126695	-0.966970781	0.39112
7761	18102	Nme1	4830	-0.258037248	0.69581
7762	79196	Osbpl5	114879	2.33215112	0.00504
7763	72567	Bclaf1		-1.050320718	0.13875
7764	79201	Tnfrsf23		-1.330335873	0.0722
7765	26942	Spag1	6674	-0.325963637	0.97982
7766	230796	Wdtc1	23038	-0.087856836	0.99905
7767	72391	Cdkn3	1033	0.994239991	0.68773
7768	382639	Zbtb42	100128927	-0.365470729	0.68169
7769	22718	Zfp60		-0.723789788	0.66463
7770	18759	Prkci	5584	-0.252223727	0.56182
7771	666173	Vps13b	157680	-0.429837497	0.63927
7772	14998	H2-DMa	3108	2.697630241	0.00388
7773	241915	Phc3	80012	-0.629614986	0.3005
7774	20516	Slc20a2	6575	-1.077381424	0.30443
7775	71862	Gpr160	26996	0.968487159	0.01018
7776	12577	Cdkn1c	1028	-1.343940104	0.28745
7777	68832	Ldah	60526	-0.680066883	0.50319
7778	319758	Rfx7	64864	-0.877088823	0.28261
7779	70435	Inf2	64423	0.141472114	0.76675
7780	70882	Armc3	219681	-0.135251124	0.88821
7781	11980	Atp8a1	10396	-0.238066654	0.64336
7782	230793	Ahdc1	27245	0.313769679	0.73633
7783	114874	Ddhd1	80821	-0.479787133	0.54854
7784	12520	Cd81	975	-0.173966078	0.87028
7785	58909	Fam13a	10144	-0.82519364	0.38671
7786	52637	Cisd1	55847	0.389351227	0.98796
7787	218952	Fermt2	10979	-0.365131462	0.71825
7788	67878	Tmem33	55161	0.058213707	0.99988
7789	54342	Gnpnat1	64841	-0.266536318	0.93349
7790	80986	Ckap2	26586	-1.145156251	0.20621
7791	80732	Mynn	55892	-0.064980066	0.99988
7792	230787	Themis2	9473	1.649330882	0.00634
7793	77569	Limch1	22998	-0.617826393	0.49271
7794	330721	Nek5	341676	-0.345393469	0.49951
7795	99045	Mrps26	64949	-0.935546724	0.26955
7796	13627	Eef1a1		0.026529592	0.99988
7797	70911	Phyhipl	84457	0.088534307	0.9383

7798	216971	Fam222b	55731	-0.982808869	0.26256
7799	381560	Xkr8	55113	0.271064963	0.51347
7800	228852	Ppp1r16b	26051	0.72091402	0.19965
7801	19217	Ptger2	5732	0.891320796	0.19626
7802	109275	Actr5	79913	-0.42128005	0.45675
7803	66859	Slc16a9	220963	-0.721153146	0.42633
7804	19935	Mrpl23		0.31797659	0.66448
7805	319513	Pced1a	64773	-0.369114206	0.62623
7806	68020	Coa8	84334	0.631773678	0.47454
7807	232023	Vopp1	81552	0.312840863	0.32442
7808	268448	Phf12	57649	-0.792089906	0.40178
7809	333789	N4bp2	55728	0.102745615	0.991
7810	19896	Rpl10a	4736	-0.10973985	0.99988
7811	72826	Fam76b	143684	-0.009975849	0.99988
7812	12385	Ctnna1	1495	-0.927833727	0.28146
7813	109082	Fbxw17		-1.64812903	0.06255
7814	269423	Abhd18	80167	-0.549005869	0.46166
7815	21817	Tgm2	7052	1.37877849	0.0084
7816	68552	Smim14	201895	0.490891489	0.14103
7817	52588	Tspan14	81619	1.059371014	0.01344
7818	243382	Ppm1k	152926	0.877254795	0.2181
7819	382137	Fdxacb1	91893	0.188549448	0.92046
7820	225994	Nmrk1	54981	-0.450321341	0.90175
7821	102639543	Ifi206		0.537672185	0.32446
7822	105148	Iars	3376	0.316156289	0.30663
7823	12876	Cpe	1363	-0.581385917	0.37442
7824	216049	Zfp365	22891	-0.465492668	0.66758
7825	68564	Nufip2	57532	-0.151410053	0.99988
7826	383619	Aim2	9447	-0.38697714	0.94739
7827	13654	Egr2	1959	2.110108217	0.00439
7828	108829	Jmjd1c	221037	-0.091380551	0.99988
7829	67333	Stk35	140901	-0.483934031	0.45503
7830	18218	Dusp8	1850	-0.879902974	0.57917
7831	213081	Wdr19	57728	-0.806322772	0.34794
7832	73173	Pcdh18	54510	0.501566082	0.56158
7833	51788	H2az1	3015	0.763562008	0.01701
7834	217864	Rcor1	23186	-0.22488408	0.84481
7835	19261	Sirpa		1.396009169	0.01204
7836	74251	Ankrd9	122416	-1.001576567	0.24307
7837	76809	Bri3bp	140707	-0.91777681	0.25825
7838	268445	Ankrd13b	124930	-0.841001532	0.40233
7839	243025	Tmem156	80008	0.477070544	0.80565
7840	17995	Ndufv1	4723	-0.345896846	0.71878
7841	234311	Ddx60	55601	-0.898812089	0.52576
7842	237860	Ssh2	85464	0.127341319	0.52314
7843	76895	Bicd2	23299	-0.415217458	0.48488
7844	57376	Smarce1	6605	-0.500077756	0.39175
7845	20778	Scarb1	949	-0.039922753	0.99988
7846	66170	Chchd5	84269	-0.039718047	0.99988
7847	234515	Inpp4b	8821	0.225544269	0.8023

7848	12775	Ccr7	1236	0.846224816	0.45284
7849	30938	Fgd3	89846	0.920582787	0.14124
7850	102566	Ano10	55129	-0.706108885	0.34636
7851	69641	Wdr20		0.126637266	0.93252
7852	237859	Nsrp1	84081	-0.852065532	0.22364
7853	68480	Card19	84270	-0.309634862	0.88959
7854	106205	Zc3h7a	29066	-0.299749705	0.6283
7855	18081	Ninj1	4814	-1.121654164	0.28025
7856	68721	110032A03R	64776	0.138633147	0.18719
7857	20621	Snn	8303	-0.318666263	0.95747
7858	232016	Itprid1	223075	-0.011028591	0.99988
7859	215707	Ccdc92	80212	0.281581465	0.88764
7860	75607	Wnk2	65268	-0.628523957	0.48479
7861	223970	Rmi2	116028	-0.496401265	0.80287
7862	19401	Rara	5914		0.40878
7863	64340	Dhx38	9785	-0.393657256	0.92721
7864	93842	Igsf9	57549	-1.031043704	0.57262
7865	101187	Parp11	57097	0.815851391	0.0112
7866	212285	Arap2	116984	-0.494992282	0.40947
7867	497652	Acd	65057	1.107347553	0.77922
7868	57354	Cramp1l	57585	-0.343847193	0.60732
7869	72612	Hpf1	54969	0.330797835	0.64863
7870	230379	Acer2	340485	0.564115987	0.36062
7871	76138	Ccdc138	165055	0.179892098	0.99988
7872	68524	Wipf2	147179	0.008930909	0.99988
7873	218236	Fam120a	23196	0.292966614	0.9497
7874	21871	Atp6v0a2	23545	-0.176020369	0.99988
7875	329877	Dennd4c	55667	0.176604532	0.76827
7876	18676	Phf2	5253	0.237387766	0.41147
7877	319801	Tigar	57103	-0.580527691	0.63169
7878	140559	Igsf8	93185	0.475251726	0.53997
7879	12703	Socs1	8651	0.835660156	0.13783
7880	70297	Gcc2	9648	0.15653913	0.89193
7881	67390	Mrm3	55178	0.466531946	0.02705
7882	230376	Haus6	54801	-0.131650974	0.8659
7883	58239	Dexi	28955	-0.183843742	0.73022
7884	231051	Kmt2c	58508	-0.708891717	0.33629
7885	107607	Nod1	10392	0.857322897	0.01535
7886	106878	Smim3	85027	-0.373998521	0.62665
7887	68235	Mturn	222166	0.930769106	0.79842
7888	218215	Rnf144b	255488	1.279596629	0.01116
7889	70925	Cdkn2aip	55602	0.211843587	0.15099
7890	338349	Cntln	54875	0.128807569	0.99988
7891	231050	Galnt11	63917	0.65390267	0.45319
7892	231997	Fkbp14	55033	1.01538149	0.01357
7893	381259	Tmem237	65062	1.105915483	0.01089
7894	218214	Kdm1b	221656	0.479822709	0.25691
7895	74143	Opa1	4976	0.046966211	0.99988
7896	70873	Cnbd2	140894	1.35541986	0.05794
7897	243272	Sbno1	55206	-0.259073786	0.62554

7898	320714	Trappc11	60684	0.091493071	0.91862
7899	244810	AW551984		1.17417988	0.26201
7900	228829	Phf20	51230	-0.471382911	0.59979
7901	57810	Cdon	50937	0.052185318	0.96072
7902	108654	Fam210a	125228	0.612662978	0.04448
7903	544696	Tbc1d32	221322	-0.361983516	0.71207
7904	269702	Mphosph9	10198	0.713362032	0.37705
7905	67501	Ccdc50	152137	0.461595274	0.0338
7906	666794	Rbm24	221662	-0.215263408	0.92702
7907	77106	Tmem181a	57583	-0.515966214	0.44798
7908	71069	Stox2	56977	-1.543316345	0.07331
7909	20623	Snrk	54861	-0.546430561	0.5063
7910	18131	Notch3	4854	-0.100914435	0.98522
7911	12523	Cd84	8832	1.608965552	0.11787
7912	66612	Ormdl3	94103	0.176456603	0.99988
7913	12142	Prdm1	639	-0.099950599	0.82705
7914	233744	Spon1	10418	0.410015267	0.36462
7915	11793	Atg5	9474	-0.921433028	0.27661
7916	83679	Pde4dip	9659	0.777574564	0.16844
7917	69863	Ttc39b	158219	-0.508071565	0.82477
7918	213056	Fam126b	285172	-0.308861661	0.98154
7919	218203	Mylip	29116	-0.871961757	0.30333
7920	215113	Slc43a2	124935	-0.377308801	0.82094
7921	75345	Slamf7	57823	0.2186563	0.78408
7922	100910	Chpf2	54480	-1.187036753	0.23451
7923	68815	Btbd10	84280	0.368887843	0.02602
7924	380713	Scarf1	8578	1.203049942	0.00832
7925	15398	Hoxa13	3209	-0.527000557	0.59317
7926	108097	Prkab2	5565	-0.233009757	0.79983
7927	50753	Fbxo8	26269	0.679060156	0.01741
7928	320655	Pgap3	93210	1.218628975	0.16742
7929	15396	Hoxa11	3207	0.150464122	0.99988
7930	66631	Mfsd14b	84641	0.337242814	0.49619
7931	213233	Tapbpl	55080	0.044023375	0.99988
7932	382010	Cep44	80817	-0.248654964	0.8717
7933	380712	Tlcd2	727910	0.487474396	0.07115
7934	408022	Primpol	201973	-0.801982404	0.41843
7935	15405	Hoxa9	3205	0.906913359	0.33808
7936	16456	F11r	50848	0.373404452	0.48201
7937	15404	Hoxa7	3204	0.874983643	0.01123
7938	71365	Pdss2	57107	0.37075968	0.72412
7939	16328	Cep250	11190	1.16387014	0.08857
7940	320878	Mical2	9645	0.263768479	0.75527
7941	109205	Sobp	55084	0.209572415	0.99779
7942	74841	Usp38	84640	0.680183097	0.02158
7943	15402	Hoxa5	3202	-0.419379587	0.92082
7944	77578	Bcl9	607	-0.160710163	0.73334
7945	68667	Trpm4	54795	-0.437453742	0.68215
7946	20361	Sema7a	8482	-1.260611873	0.00959
7947	73102	Slc22a23	63027	0.025005151	0.98772

7948	246257	Ovca2	124641	-0.866837625	0.42261
7949	320678	Iffo1	25900	-0.637248019	0.29784
7950	14109	Fau	2197	-0.382207031	0.4912
7951	110109	Nop2	4839	0.434444399	0.65156
7952	14628	Ostm1	28962	0.382989259	0.28274
7953	68021	Bphl	670	0.535757096	0.02418
7954	103677	Smg6	23293	-0.672391199	0.44925
7955	102141	Snx25	83891	0.182677414	0.99988
7956	233733	Galnt18	374378	-0.529584274	0.44775
7957	225348	Wdr36	134430	0.407663957	0.35582
7958	71982	Snx10	29887	0.176828785	0.90813
7959	67198	Spats2l	26010	-0.318789546	0.99988
7960	108687	Edem2	55741	-0.489317394	0.72727
7961	73467	700066M21R	205327	-0.012380942	0.99988
7962	56407	Trpc4ap	26133	-0.032471128	0.99988
7963	140742	Sesn1	27244	0.451539114	0.53981
7964	104662	Tsr1	55720	-0.20676201	0.60423
7965	208104	Mlxip	22877	0.020006891	0.99988
7966	353170	Txlng	55787	-0.074592399	0.99988
7967	269800	Zfp384	171017	-0.381974398	0.58656
7968	227120	Plcl1	5334	0.75465424	0.55018
7969	97761	Sgsm2	9905	-1.020751738	0.30954
7970	217151	Arl5c	390790	2.185028774	0.04688
7971	213121	Ankrd35	148741	-0.206141521	0.991
7972	66822	Fbxo25	26260	0.546445442	0.08349
7973	16796	Lasp1	3927	0.188914551	0.99016
7974	230393	Focad	54914	0.561820254	0.56643
7975	56406	Ncoa6	23054	0.282693792	0.37195
7976	319934	Sbf2	81846	-1.380002079	0.22198
7977	218138	Gmds	2762	0.191614531	0.75682
7978	60365	Rbm8a		0.023781425	0.99988
7979	68728	Trp53inp2	58476	0.367264699	0.37263
7980	22137	Ttk	7272	-1.212204788	0.4291
7981	228812	Pigu	128869	0.4894157	0.01926
7982	208043	Setd1b	23067	0.001511646	0.99988
7983	20130	Rras	6237	-0.125082583	0.85735
7984	56524	Mpp6	51678	0.374227491	0.72545
7985	56338	Txnip	10628	-0.014276858	0.99988
7986	67031	Upf3a	65110	-0.220060683	0.85685
7987	65112	Pmepa1	56937	-0.66631549	0.35865
7988	233208	Scaf1	58506	-1.081076521	0.21692
7989	56295	Higd1a	25994	0.114810273	0.95556
7990	15220	Foxq1	94234	-0.12806201	0.9243
7991	69957	Cdc16	8881	0.12896023	0.99988
7992	103199	Fig4	9896	0.186494667	0.99988
7993	13653	Egr1	1958	1.273178757	0.13249
7994	98752	Fcrla	84824	0.638807918	0.30965
7995	72748	Hdhd3	81932	-0.757941224	0.76504
7996	26447	Poli	11201	0.281381269	0.64648
7997	22225	Usp5	8078	-0.0362161	0.99988

7998	246198	Mllt6	4302	-0.120391408	0.75164
7999	71713	Cdc40	51362	-0.389183229	0.57293
8000	14794	Spsb2	84727	-0.512275707	0.90372
8001	209773	Dennd2a	27147	-0.05595856	0.99988
8002	70178	Abhd17c	58489	-0.623388124	0.49486
8003	66694	Uqcrfs1	7386	0.257282362	0.06086
8004	320078	Olfml2b	25903	0.875681076	0.89847
8005	75608	Chmp4b	128866	0.458268741	0.06247
8006	70729	Nos1ap	9722	-1.081074348	0.14619
8007	78334	Cdk19	23097	-0.497826193	0.42656
8008	21781	Tfdp1	7027	0.704930228	0.22812
8009	192157	Socs7	30837	0.315023159	0.34451
8010	66491	Polr2l	5441	0.145730929	0.87037
8011		Cdk19os		-0.540214202	0.81577
8012	229603	Otud7b	56957	-0.431877485	0.45862
8013	234076	Tmco3	55002	-0.427525207	0.49296
8014	75210	Prr3	80742	-0.836421609	0.40743
8015	84113	Ptov1	53635	0.074923114	0.91027
8016	67943	Mesd	23184	0.817413064	0.01344
8017	102323	Dcun1d2	55208	-0.149610498	0.82487
8018	243771	Parp12	64761	-0.45357473	0.44925
8019	23886	Gdf15	9518		0.991
8020	67239	Rpf2	84154	-0.441510704	0.3486
8021	66790	Grtp1	79774	0.135745232	0.98036
8022	73174	Tbkbp1	9755	0.665179951	0.33451
8023	16468	Jarid2	3720	-0.286568014	0.73305
8024	233204	Tbc1d17	79735	-0.234876567	0.75288
8025	50908	C1s1		1.210592606	0.21382
8026	319262	Fchsd1	89848	-1.069222697	0.25847
8027	67211	Armc10	83787	0.929039825	0.00972
8028	232371	C1rl	51279	0.276344878	0.99988
8029	19736	Rgs4	5999	-2.869391937	0.11592
8030	12396	Cbfa2t2	9139	-0.623146503	0.51657
8031	71240	Osbpl7	114881	0.489508242	0.68773
8032	235469	Zfp280d		-1.4053631	0.19857
8033	320538	Ubn2	254048	-0.340122463	0.42656
8034	107503	Atf5	22809	0.785493289	0.23819
8035	233424	Tmc3	342125	1.170947169	0.28659
8036	234069	Pcid2	55795	-0.561782993	0.76175
8037	229600	BC028528	79630	1.853109816	0.00447
8038	66209	Inip	58493	0.231741454	0.05676
8039	66515	Cul7	9820	-0.213903276	0.75914
8040	56705	Ranbp9	10048	-0.16262531	0.72791
8041	64339	Fndc4	64838	1.48683097	0.00652
8042	101592	Efl1	79631	0.051399164	0.99988
8043	67661	Ift172	26160	-0.16533731	0.93623
8044	231724	Rad9b	144715	0.291513637	0.44695
8045	96935	Susd4	55061	-0.452143158	0.7324
8046	634731	Susd1	64420	-0.136444058	0.99988
8047	320717	Pptc7	160760	0.515849726	0.1203

8048	83397	Akap12	9590	0.388832874	0.65255
8049	654470	Tctn1	79600	1.237841836	0.00984
8050	100038725	Cep85l	387119	-0.942001524	0.36705
8051	75687	Ripor1	79567	-0.28312183	0.93305
8052	229011	Samd10	140700	-0.837611288	0.29038
8053	14700	Gng10	2790	0.836855111	0.26821
8054	210293	Dock10	55619	1.41516759	0.00502
8055	101471	Phrf1	57661	-0.875074791	0.31619
8056	17210	Mcl1	4170	0.565011802	0.08542
8057	18023	Nfe2l1	4779	-0.805441647	0.42676
8058	66985	Rassf7	8045	1.479100305	0.0079
8059	56205	Ensa	2029	0.028801719	0.99988
8060	69790	Med30	90390	0.674349609	0.48488
8061	107769	Tm6sf1	53346	3.669901692	0.00388
8062	67005	Polr3k	51728	-0.273976314	0.84471
8063	13244	Degs1		0.347772615	0.34201
8064	70552	Lrrc56	115399		0.40878
8065	13040	Ctss	1520	2.056483686	0.00575
8066	18971	Pold1	5424	-0.918353866	0.36415
8067	67148	Ramac		0.212620764	0.08216
8068	208647	Creb3l2	64764	-0.071696342	0.99988
8069	107702	Rnh1	6050	0.015157486	0.99988
8070	226089	Ric1	57589	0.872385208	0.23085
8071	235439	Herc1	8925	-1.224332481	0.22364
8072	14745	Lpar1	1902	0.706025284	0.40946
8073	76688	Arfrp1	10139	1.171220498	0.00952
8074	83925	Trps1	7227	-0.018778626	0.99988
8075	68083	Pak1ip1	55003	0.091042752	0.88714
8076	269400	Rtel1		-0.072738622	0.99988
8077	57423	Atp5j2	9551	-0.260628689	0.65328
8078	15412	Hoxb4	3214	0.205526917	0.43255
8079	66124	Josd2	126119	-0.289246284	0.99806
8080	227743	Mapkap1	79109	-0.137490276	0.84891
8081	102162	Taf5l	27097	-0.599647273	0.29102
8082	15413	Hoxb5	3215	-1.494580505	0.15758
8083	319901	Dsel	92126	-0.166683632	0.99988
8084	229004	Gmeb2	26205	0.322463767	0.63685
8085	54214	Golga4	2803	-0.648414267	0.37201
8086	75007	Mindy1	55793	-0.901163134	0.29838
8087	27425	Atp5l		1.265790566	0.00903
8088	18516	Pbx3	5090	0.518986175	0.40918
8089	15415	Hoxb7	3217	0.586725673	0.25691
8090	231889	Bud31	8896	0.366857317	0.31663
8091	677884	Pakap		1.437785112	0.00533
8092	218121	Mboat1	154141	-0.436810446	0.86157
8093	226757	Wdr26	80232	-0.672766142	0.24879
8094	67429	Nudcd1	84955	0.501095804	0.94943
8095	72543	Mvb12b	89853	-0.312198759	0.49717
8096	70699	Nup205	23165	0.711249427	0.34011
8097	224742	Abcf1	23	-0.349123991	0.66769

8098	545622	Ptpn3	5774	-0.320620584	0.78118
8099	213054	Gabpb2	126626	-0.904914027	0.2556
8100	277250	Kdm3b	51780	-1.178478599	0.20083
8101	77987	Ascc3	10973	0.435614179	0.06136
8102	22351	Vill	50853	-0.548240313	0.52136
8103	13849	Ephx1	2052	0.061653681	0.93851
8104	20360	Sema6c	10500	0.885425246	0.18294
8105	75788	Smurf1	57154	-0.226021175	0.55424
8106	106766	Stap2	55620	-0.957465851	0.42635
8107	53621	Cnot4	4850	-0.590237933	0.38023
8108	13590	Lefty1		0.840417283	0.0155
8109	67695	Ost4	100128731	0.07210915	0.67211
8110	208768	Sde2	163859	-0.300642569	0.5749
8111	380711	Rap1gap2	23108	-0.015087542	0.99988
8112	14710	Gngt2	2793	0.582641382	0.07288
8113	67674	Trmt112	51504	0.384248639	0.4571
8114	54366	Ctnnal1	8727	-1.432203318	0.16878
8115	209462	Hace1	57531	-0.29587234	0.9542
8116	230234	Abitram	54942	-0.894900843	0.43126
8117	68796	Tmem214	54867	-0.981028578	0.2812
8118	241308	Ralgps1	9649	1.411769442	0.00721
8119	76223	Agbl3	340351	-1.060028872	0.40517
8120	68915	Vars2	57176	-0.748897525	0.36329
8121	14537	Gcnt1	2650	1.047933344	0.1082
8122	16558	Kif16b		-0.804641811	0.32867
8123	18673	Phb	5245	-0.097911314	0.93014
8124	228994	Ythdf1	54915	-0.211435048	0.69759
8125	320404	Itpkb	3707	1.481520467	0.00819
8126	66898	Baiap2l1	55971	-0.221213685	0.85981
8127	99326	Garnl3	84253	-0.910789108	0.35347
8128	107650	Pi4kb	5298	0.145860104	0.81587
8129	227449	Zcchc2	54877	0.122952935	0.90035
8130	12183	Bpgm	669	0.062104226	0.89975
8131	11906	Zfhx3	463	-0.680561006	0.30415
8132	68031	Rnf146	81847	-0.156162854	0.98506
8133	223473	Nipal2	79815	-1.241990511	0.17064
8134	79044	Mrps34	65993	0.098271077	0.99988
8135	319278	Shfl	55337	-0.858178335	0.37475
8136	140481	Man2a2	4122	-0.833312657	0.28092
8137	233189	Ctu1		0.74927121	0.01357
8138	215512	Fam117a	81558	0.94050391	0.0401
8139	384783	Irs2	8660	0.348849786	0.81388
8140	319601	Zfp653		0.083969218	0.75663
8141	269261	Rpl12		0.223981032	0.77419
8142	229584	Pogz	23126	0.127594906	0.99656
8143	381175	Ccdc68	80323	-0.148514779	0.99988
8144	217127	Kat7	11143	0.513458288	0.27471
8145	224860	Plcl2	23228	0.428049488	0.10588
8146	23856	Dido1	11083	-0.322364848	0.54694
8147	269955	Rccd1	91433	-1.528424914	0.22728

8148	109232	Sccpdh	51097	0.090460738	0.84763
8149	233406	Prc1	9055	-1.001170413	0.25868
8150	226744	Cnst	163882	0.699666948	0.49116
8151	109115	Supt3	8464	-0.059656612	0.99672
8152	353190	Edc3	80153	-0.069590031	0.91942
8153	108115	Slco4a1	28231	0.14755145	0.99988
8154	22195	Ube2l3		0.040165643	0.99988
8155	18604	Pdk2	5164	0.594979251	0.49889
8156	231876	Lmtk2	22853	-0.109007507	0.99988
8157	19352	Rabggtb	5876	0.265050576	0.38074
8158	217124	Ppp1r9b	84687	-0.097841244	0.99988
8159	17828	Bloc1s5		-0.018010377	0.99988
8160	252966	Cables2	81928	-1.546745302	0.14724
8161	105245	Txndc5	81567	0.652114519	0.02181
8162	100763	Ube3c	9690	0.824927352	0.31763
8163	66481	Rps21	6227	0.571400225	0.25681
8164	12161	Bmp6	654	0.09768519	0.99988
8165	21898	Tlr4	7099	0.859849439	0.01687
8166	54381	Cpq	10404	0.584341944	0.02777
8167	30057	Timm8b	26521	0.092331936	0.55926
8168	212508	Mtg1	92170	-0.364468728	0.59735
8169	73910	Arhgap18	93663	0.79663603	0.01881
8170	75812	Tasp1	55617	0.682855771	0.02454
8171	56436	Adrm1		0.262984025	0.79983
8172	70420	Arpin		-0.037458771	0.99988
8173	98910	Usp6nl	9712	-0.75236226	0.45483
8174	329777	Pigk	10026	0.540854418	0.09252
8175	235169	Foxred1	55572	0.73176264	0.36225
8176	228983	Osbpl2	9885	-0.240673588	0.8659
8177	268465	Eme1	146956	-0.825690039	0.28119
8178	67856	Echdc3	79746	-0.057521088	0.98477
8179	68073	Atpsckmt	134145	-1.284639351	0.18486
8180	17463	Psmd7	5713	-1.233331743	0.21231
8181	108946	Zzz3	26009	-0.475084829	0.49408
8182	52856	Mtg2	26164	0.545504844	0.75006
8183	218820	Zfp503	84858	-0.387637879	0.55974
8184	269397	Ss18l1	26039	-0.070702079	0.96811
8185	68750	Rreb1	6239	-1.124128086	0.21861
8186	237339	L3mbtl3	84456	0.062731911	0.90047
8187	228677	Sptlc3	55304	1.629164084	0.00447
8188	237926	Rsad1	55316	-2.564805961	0.08988
8189	223455	Marchf6	10299	0.283816202	0.40878
8190	68810	Nexn	91624	0.062255058	0.99988
8191	66290	Atp6v1g1	9550	0.531040401	0.02776
8192	241846	Lsm14b	149986	0.156086469	0.99988
8193	74145	F13a1	2162	2.192359523	0.00544
8194	104601	Mycbpap	84073	0.350969913	0.66181
8195	68404	Nrn1	51299	0.451285676	0.95306
8196	104099	Itga9	3680	2.275430009	0.00447
8197	215798	Adgrg6	57211	-0.624751461	0.47978

8198	228980	Taf4	6874	-0.097977615	0.82186
8199	353211	Prune2	158471	1.638471888	0.08603
8200	98828	Cdc123	8872	0.571560367	0.01441
8201	232679	Zc3hc1	51530	-0.119768979	0.93748
8202	54120	Gipc2	54810	0.466568258	0.55705
8203	227541	Camk1d	57118	-0.135083492	0.88823
8204	20227	Sart1	9092	-0.450727796	0.40109
8205	12393	Runx2	860	-0.72455233	0.57231
8206	116873	Stim2	57620	-0.013039647	0.99988
8207	98952	Fam102a	399665	0.539760288	0.0189
8208	22214	Ube2h	7328	0.109216775	0.88887
8209	67899	Cmc1	152100	0.394708557	0.29301
8210	432442	Akap7	9465	-0.44800869	0.55591
8211	170757	Adgrl4	64123	-0.434011579	0.98415
8212	223453	Dap	1611	-0.403674792	0.33808
8213	18975	Polg	5428	0.17287991	0.99988
8214	67249	Tbc1d19	55296	0.531784447	0.28284
8215	105351	AW209491	79020	0.064169461	0.99426
8216	26426	Nubp2	10101	-0.91260353	0.22236
8217	19664	Rbpj	3516	0.331482729	0.34186
8218	268973	Nlrc4	58484	-0.06946336	0.99988
8219	73737	110008P14R	79095	-2.035911108	0.09182
8220	18405	Orm1		0.709626511	0.6932
8221	11534	Adk	132	-0.232184954	0.6899
8222	70796	Zdhhc1	29800	-0.856052404	0.25175
8223	209815	Tbc1d25	4943	-1.488787496	0.1333
8224	54608	Abhd2	11057	0.205880104	0.15302
8225	68379	Ciz1	25792	-1.653676568	0.14885
8226	231871	Daglb	221955	1.208600297	0.54544
8227	210029	Metrnl	284207	0.46889407	0.30304
8228	67769	Gpatch2	55105	-1.877358587	0.11767
8229	16173	Il18	3606	1.029034703	0.01196
8230	75956	Srrm2	23524	-0.14404721	0.84693
8231	94246	Arid4b	51742	-0.671787202	0.33619
8232	52040	Ppp1r10	5514	-0.0346056	0.86395
8233	68028	Rpl22l1	200916	0.251199316	0.51011
8234	108903	Tbcd	6904	-0.197171275	0.85238
8235	20937	Suv39h1	6839	-0.157206699	0.99988
8236	74732	Stx11	8676	2.352407845	0.00447
8237	70430	Tbce		0.350881575	0.94358
8238	69608	Sec24d	9871	0.003440366	0.99988
8239	57444	Isg20	3669	-0.337669764	0.65461
8240	319530	Zfp750	79755	0.123012138	0.98653
8241	21808	Tgfb2	7042	-0.057574994	0.91514
8242	97884	B3galnt2	148789	0.257700082	0.99152
8243	231868	130309D02R	79034	0.295752172	0.75453
8244	226791	Lyplal1	127018	-0.71929365	0.35314
8245	246316	Lgi2	55203	1.472443688	0.13285
8246	238024	Fn3krp	79672	0.46730909	0.54207
8247	99011	Pomt1	10585	-0.473293607	0.55004

8248	227723	Prrc2b	84726	-0.304977046	0.63993
8249	228961	Npepl1	79716	0.022587015	0.99988
8250	83408	Gimap3		0.441220841	0.32237
8251	230316	Megf9	1955	-0.467342611	0.98331
8252	68837	Foxk2	3607	0.41961811	0.36882
8253	27215	Azi2	64343	-0.150419115	0.99988
8254	72007	Fndc3b	64778	0.096151432	0.99988
8255	217370	Cybc1	79415	0.206192618	0.99988
8256	214444	Cdk5rap2	55755	-0.453529115	0.44051
8257	22035	Tnfsf10	8743	-0.058127013	0.96083
8258	238023	Hexdc	284004	-0.509935932	0.87562
8259	17423	Ndst2		0.39951654	0.30667
8260	76438	Rftn1	23180	1.182906072	0.0321
8261	98732	Rab3gap2	25782	0.261325644	0.99988
8262	16008	Igfbp2	3485	2.543192035	0.00616
8263	68867	Rnf122	79845	-0.365417881	0.77935
8264	239706	Mettl22	79091	1.285208521	0.08541
8265	76252	Atp6v0e2	155066	-0.042466727	0.99988
8266	226777	130074G19R	79762	1.061871883	0.72081
8267	54380	Smarcal1	50485	0.074932608	0.89373
8268	227715	Exosc2	23404	0.202553055	0.98653
8269	73068	Fut11	170384	0.563974182	0.01592
8270	233489	Picalm	8301	0.739887017	0.01296
8271	58210	Sectm1b		0.342906821	0.58308
8272	218811	Sec24c	9632	-0.190792999	0.99988
8273	15284	Hlx	3142	-0.817117724	0.52462
8274	54636	Wdr45	11152	0.594846955	0.05722
8275	63953	Dusp10	11221	0.364711218	0.04241
8276	76453	Prss23	11098	0.46562268	0.613
8277	70673	Prdm16	63976	1.388834722	0.00926
8278	320473	Heatr5b	54497	0.181434231	0.99988
8279	66797	Cntnap2	26047	-0.91301243	0.15758
8280	72759	Tmem135	65084	-0.280770325	0.501
8281	54384	Mtmr7	9108	-0.303078137	0.38023
8282	67229	Prpf18		-0.600566121	0.49951
8283	67880	Dcxr	51181	-0.84391286	0.31859
8284	382083	Snx22	79856	0.387010587	0.67691
8285	338467	Morc3	23515	1.216341383	0.01105
8286	19041	Ppl	5493	0.521480337	0.06285
8287	268783	Mtmr12	54545	-0.653758922	0.32237
8288	102791	Tcta	6988	-1.161796082	0.22111
8289	12813	Col10a1			0.40878
8290	77031	Slc9a8	23315	-0.525711623	0.3982
8291	70546	Zdhhc2	51201	0.900604744	0.01143
8292	170644	Ubn1	29855	-0.300058424	0.49033
8293	22393	Wfs1	7466	0.683229698	0.02147
8294	20204	Prrx2	51450	1.507739392	0.13938
8295	231861	Tnrc18	84629	0.27469367	0.91977
8296	78506	Micu3	286097	1.266013236	0.08969
8297	319638	Nt5dc1	221294	0.015070824	0.99382

8298	18188	Nrtn	4902	-1.815313912	0.02285
8299	72323	Asb6	140459	-0.310785765	0.62844
8300	72480	Tspyl4	23270	-1.03659447	0.38999
8301	227526	Cdnf	441549	0.363906435	0.93265
8302	212898	Dse		0.794906524	0.02776
8303	98999	Znfx1	57169	-0.555706614	0.37524
8304	234865	Nup133	55746	0.144193304	0.94082
8305	224648	Uhrf1bp1	54887	-0.985982362	0.25705
8306	110854	Ptpa	5524	-0.935505665	0.24504
8307	13123	Cyp7b1	9420	-0.175268104	0.99988
8308	20371	Foxp3	50943	0.354708711	0.33996
8309	230967	Cep104	9731	-0.389353286	0.68609
8310	54670	Atp8b1	5205	1.161001033	0.00933
8311		NA		0.781400343	0.01299
8312	72580	Zup1	221302	-0.35456545	0.68721
8313	20853	Stau1	6780	-0.667048127	0.30464
8314	17967	Ncam1	4684	0.281448633	0.82658
8315	230959	Ajap1	55966	-0.127188849	0.99988
8316	54646	Ppp1r3f	89801	0.242427635	0.66729
8317	76710	210406O10Rik		-1.160001205	0.22927
8318	207740	Ubald1	124402	0.14956243	0.89285
8319	232035	Ccser1	401145	-1.108218043	0.03696
8320	270163	Myo9a	4649	-0.0797985	0.99988
8321	105428	Fam149b	317662	1.237071094	0.19857
8322	53901	Rcan2	10231	0.195793911	0.99988
8323	207181	Rbms3	27303	-0.457605507	0.5344
8324	67063	Pgap4	84302	0.911893372	0.00662
8325	56424	Stub1	10273	-1.605654503	0.16936
8326	68591	Mocos	55034	0.144469735	0.99988
8327	319582	Trmt9b	57604	-1.001462711	0.26887
8328	277360	Prex1	57580	2.14000718	0.00491
8329	231855	Ap5z1	9907	0.227840886	0.99914
8330	51810	Hnrnpu	3192	-0.056699103	0.99988
8331	244421	Lonrf1	91694	-0.435943516	0.50414
8332	78885	Coro7		0.718799738	0.46988
8333	56282	Mrpl12	6182	-0.144746235	0.89195
8334	246154	Vasn	114990	0.35257517	0.97242
8335	70266	Kyat1		-0.129367211	0.92983
8336	208922	Cpeb3	22849	-0.320218558	0.73063
8337	20182	Rxrb	6257	-0.34671412	0.67778
8338	227695	Spout1	51490	0.233816086	0.98477
8339	57295	Icmt	23463	-1.087250497	0.25527
8340	66431	Oxld1	339229	0.645132466	0.60462
8341	228880	Zmynd8	23613	-0.380643495	0.59
8342	75568	Capsl	133690	0.122669971	0.99988
8343	70296	Tbc1d13	54662	-0.400559063	0.60623
8344	121022	Mrps6	64968	0.505733881	0.26985
8345	66988	Lap3	51056	-0.375393316	0.55283
8346	330222	Sdk1	221935	-0.168542906	0.97554
8347	227693	Zer1	10444	-0.566909523	0.66145

8348	66665	Msantd3	91283	-0.002611517	0.99989
8349	211329	Ncoa7	135112	0.109938369	0.73377
8350	74481	Batf2	116071	0.135688623	0.99988
8351	99526	Usp53	54532	0.343751288	0.98145
8352	217365	Nploc4	55666	-0.311379874	0.55034
8353	320506	Lmbrd2	92255	-0.15596975	0.82705
8354	269608	Plekhg5	57449	0.431526526	0.35036
8355	71820	Dync2i2	89891	-0.282103544	0.92248
8356	381406	Trp53rka		-0.566368839	0.65044
8357	214459	Fnbp1l	54874	-1.055033666	0.25359
8358	66801	Prkrip1	79706	-0.251820052	0.65635
8359	52864	Slx4	84464	-0.22180879	0.95668
8360	56737	Alg2	85365	-0.280007741	0.62525
8361	268515	Bahcc1	57597	-0.533651462	0.47555
8362	53415	Htatip2	10553	-0.469449763	0.55059
8363	269717	Orai2	80228	0.224622854	0.18749
8364	242960	Fbxl5	26234	0.169088521	0.99988
8365	72041	Alkbh4	54784	-2.179920629	0.11756
8366	99480	Dnttip2	30836	-0.350989662	0.61695
8367	69876	Thap3	90326	-0.046331259	0.92393
8368	246738	Dnajc28	54943	1.09149849	0.02822
8369	231214	Cc2d2a	57545	0.068929107	0.99988
8370	230935	Dnajc11	55735	-0.900828284	0.2368
8371	383295	Ypel5	51646	-0.081119341	0.93453
8372	20022	Polr2j		0.109134675	0.99988
8373	230145	Galnt12	79695	0.245877713	0.50868
8374	12009	Cep131	22994	0.566892409	0.6985
8375	231207	Cpeb2	132864	-0.666810752	0.84064
8376	98256	Kmo	8564	0.899252152	0.16236
8377	99151	Cercam	51148	-0.029205914	0.99988
8378	71063	Zfp597	146434	-0.43634752	0.72874
8379	66361	Zfand1	79752	0.051919046	0.90372
8380	73692	Cplane1	65250	-0.494246353	0.57303
8381	228869	Ncoa5	57727	0.319311885	0.99988
8382	103284	Zc3h10		-0.106858492	0.99988
8383	381605	Tbc1d2	55357	-0.009443982	0.99988
8384	227682	Trub2	26995	0.617373565	0.22535
8385	545085	Wdr70	55100	-0.930399257	0.27518
8386	214137	Arhgap29	9411	0.147936803	0.75053
8387	215819	Nhsl1	57224	-1.734609378	0.23338
8388	100502841	Epg5	57724	0.223166045	0.67959
8389	627049	Zfp800	168850	-1.276339186	0.21804
8390	244329	Mcph1	79648	-0.250043328	0.59867
8391	107746	Rapgef1	2889	0.076243438	0.99988
8392	228866	Pcif1	63935	-0.68398336	0.36223
8393	338371	Endov	284131	1.239592852	0.15428
8394	68703	Rere	473	0.265890291	0.39196
8395	213603	Slc44a3	126969	-0.801308876	0.3155
8396	380629	Heca	51696	0.321618759	0.3364
8397	74511	Lrrc17	10234	1.001821681	0.43009

8398	215210	Tmem120a	83862	-0.349773892	0.76721
8399	66789	Alg14	199857	0.048799169	0.99988
8400	14190	Fgl2	10875	1.850144642	0.01296
8401	71617	Armh3	79591	-0.031062512	0.99053
8402	70967	Eva1c	59271	-0.921748665	0.25731
8403	268512	Slc26a11	284129	0.855952574	0.19626
8404	17684	Cited2	10370	-1.127109277	0.26783
8405	74646	Spsb1	80176	-1.654332077	0.12922
8406	210582	Coq10a		-1.651077965	0.05739
8407	215160	Rhbdd2	57414	-0.298740061	0.85293
8408	207932	Urb1	9875	-0.725186271	0.36686
8409	212167	Gsap	54103	-0.874073517	0.45745
8410	18707	Pik3cd	5293	1.706777724	0.01235
8411	19219	Ptger4	5734	1.443864808	0.02185
8412	18798	Plcb4	5332	-0.237623927	0.65683
8413	13138	Dag1	1605	-0.052183546	0.99988
8414	65945	Clstn1	22883	-0.849159368	0.28972
8415	77037	Mrap	56246	-0.574347494	0.57695
8416	320204	Etfbkmt	254013	0.563176597	0.03744
8417	215114	Hip1	3092	-0.122874325	0.99988
8418	69581	Rhou	58480	-0.754029187	0.32237
8419	30046	Zfp292	23036	-0.565925466	0.36529
8420	242860	Rsbn1l	222194	-0.386193991	0.53754
8421	207592	Tbc1d16	125058	0.918148294	0.30175
8422	237256	Zc3h12d	340152	-0.330384742	0.78781
8423	207521	Dtx4	23220	-0.039287218	0.99988
8424	269336	Ccdc32	90416	0.311899062	0.44818
8425	56306	Sinhcaf	58516	-0.411435507	0.92973
8426	68770	Phtf2	57157	1.024160946	0.09364
8427	433667	Ankrd13c	81573	-0.294298964	0.91157
8428	12418	Cbx4	8535	0.002787317	0.99989
8429	214764	Edrf1	26098	0.582717619	0.01955
8430	21853	Timeless		-0.400549391	0.64701
8431	15950	Ifi203		0.023331313	0.99988
8432	50791	Magi2	9863	1.137378568	0.01743
8433	215751	Ginm1	116254	-0.382310583	0.67575
8434	228536	Bahd1	22893	0.456178815	0.1062
8435	14687	Gnaz	2781	-0.916727342	0.24145
8436	19218	Ptger3	5733	1.970065326	0.20621
8437	226139	Cox15		0.11589297	0.99988
8438	16798	Lats1	9113	-1.023381591	0.26511
8439	74998	Rab11fip2	22841	0.350593463	0.99988
8440	213541	Ythdf2	51441	-0.528826033	0.16964
8441	20210	Saa3		-1.951673701	0.15546
8442	15568	Elavl1	1994	0.045388013	0.99397
8443	320727	Ipo8	10526	-0.140773693	0.89195
8444	20847	Stat2		-0.64396832	0.28054
8445	69912	Nup43	348995	-0.361507922	0.88372
8446	320840	Negr1	257194	1.352195682	0.0096
8447	21821	Ift88	8100	-1.815188957	0.10438

8448	56516	Rbms2		-1.063113633	0.21774
8449	50793	Orc3	23595	-0.180745695	0.66436
8450	68342	Ndufb10	4716	0.447271436	0.03617
8451	116848	Baz2a		-1.117713797	0.25305
8452	18796	Plcb2	5330	-0.174061019	0.84177
8453	12236	Bub1b	701	-0.561213506	0.53643
8454	171543	Bmf	90427	0.530652789	0.45946
8455	224613	Flywch1	84256	-1.037006679	0.24705
8456	232539	Klhl42	57542	-0.30228484	0.65789
8457	74411	Plpp6	403313	-0.237861868	0.7872
8458	235283	Gramd1b	57476	-0.021515092	0.99988
8459	232536	Mrps35	60488	-0.026972694	0.99988
8460	12293	Cacna2d1	781	0.809265672	0.10202
8461	66532	Rep15	387849	-0.700638362	0.55633
8462	219105	Zmym5	9205	-0.735645854	0.26996
8463	98376	Gorab	92344	-0.546460373	0.48966
8464	108767	Pnrc1	10957	-1.034799202	0.31883
8465	77252	9430038I01Ri	387723	0.215324726	0.67248
8466	71746	Rgl3	57139	0.340397629	0.89659
8467	109731	Maob		1.227514348	0.16572
8468	23908	Hs2st1		0.381697527	0.03319
8469	21825	Thbs1	7057	0.191013614	0.88185
8470	76281	Tax1bp3	30851	-0.724742537	0.312
8471	67143	Ikzf5	64376	0.109183573	0.88819
8472	55990	Fmo2	2327	0.198043474	0.66436
8473	69113	Alkbh3	221120	0.184706177	0.86335
8474	68277	310057M21R	80007	-0.52780795	0.40219
8475	14261	Fmo1	2326	0.872976916	0.52331
8476	140577	Ankrd6	22881	0.114403633	0.88738
8477	272322	Arntl2	56938	-0.929984193	0.28284
8478	24044	Scamp2	10066	-0.335883924	0.91586
8479	72243	Nemp1	23306	-0.420122889	0.67622
8480	68026	Pclaf	9768	-0.47920004	0.49109
8481	170753	Zfp704	619279	-0.069492591	0.90828
8482	13732	Emp3	2014	1.207862605	0.02285
8483	229905	Kyat3	56267	0.224874937	0.09709
8484	104346	Gas8	2622	-0.840971167	0.33836
8485	226562	Prrc2c	23215	-0.980524953	0.18819
8486	23890	Gpr34		0.508925263	0.12649
8487	67623	Tm7sf3	51768	0.350402742	0.05259
8488	66682	Trappc5	126003	0.183477084	0.78063
8489	67529	Fgfr1op2	26127	-0.078209427	0.99988
8490	108995	Tbc1d10c	374403	0.421890392	0.30728
8491	16971	Lrp1	4035	-0.009534177	0.99988
8492	71177	Ints13	55726	-0.15683969	0.96441
8493	229900	Gbp7		-0.188262999	0.96528
8494	108151	Sema3d	223117	0.194230003	0.99988
8495	234825	Klhdc4	54758	-0.650705579	0.2905
8496	103967	Dnm3	26052	-0.988782426	0.01704
8497	101476	Plekha1	59338	1.389050965	0.00832

8498	66230	Mrps28	28957	0.39697185	0.55566
8499	12014	Bach2	60468	0.713841629	0.20605
8500	329470	Accs	84680	-0.304032667	0.19737
8501	12571	Cdk6	1021	-0.409387684	0.63226
8502	407790	Ndufa4l2	56901	0.140663403	0.99988
8503	399568	Cdin1	84529	0.210185963	0.9383
8504	237754	Btnl9	153579	-0.028156001	0.99988
8505	230073	Ddx58		0.287838633	0.59203
8506	226551	Suco	51430	-0.39055161	0.66292
8507	269623	Rbm48	84060	0.520968872	0.32005
8508	240084	Cchcr1	54535	-1.267853029	0.26777
8509	228491	Zfp770	54989	-1.252681237	0.19145
8510	229731	Slc25a24	29957	-0.571587228	0.39872
8511	321006	Dcaf1	9730	-1.299174251	0.19142
8512	78309	Cul9	23113	0.58098726	0.29601
8513	16196	Il7	3574	1.095643224	0.09039
8514	67872	Nsmce4a	54780	0.511759926	0.11051
8515	329739	Fam102b	284611	0.411961297	0.74842
8516	216445	Arhgap9	64333	-1.576994651	0.11359
8517	94089	Trim7	81786	0.248067869	0.99988
8518	70797	Ankib1	54467	-0.287005321	0.99034
8519	216443	Mars1	4141	0.105328942	0.89195
8520	108077	Skiv2l	6499	-0.290855608	0.7675
8521	67490	Ufl1	23376	0.509825944	0.52758
8522	71458	Bcor	54880	0.624175661	0.02595
8523	211007	Trim41	90933	-0.31426627	0.63441
8524	67636	Etfrf1	144363	0.178806405	0.67959
8525	19302	Pex2	5828	-0.56167955	0.48558
8526	11834	Aqr	9716	-0.386785938	0.68613
8527	19045	Ppp1ca	5499	-0.412651437	0.45818
8528	99512	Wdr47	22911	-1.104630075	0.38207
8529	269881	Map3k10	4294	0.052981942	0.94627
8530	68904	Abhd13	84945	0.203201455	0.19586
8531	100986	Akap9	10142	-1.023908328	0.24222
8532	269514	Fbxl4	26235	0.46424157	0.02833
8533	229722	Elapor1	57535	-1.363544746	0.23224
8534	246696	Slc25a28	81894	0.331223105	0.39703
8535	381305	Rc3h1	149041	-0.422362221	0.43192
8536	69217	Plekha4	57664	1.929110824	0.11994
8537	545725	Mterf1a		-1.986873886	0.15922
8538	71795	Pitpnc1	26207	0.285211083	0.84432
8539	245007	Zbtb38	253461	-0.180369094	0.91065
8540	17872	Ppp1r15a	23645	-0.208373016	0.99905
8541	216441	Slc26a10		-0.557425083	0.38972
8542	225283	Rprd1a	55197	0.459561062	0.04098
8543	216892	Spns2	124976	-0.938213361	0.2192
8544	208449	Sgms1	259230	-1.053381628	0.4267
8545	77593	Usp45	85015	-1.279568266	0.18424
8546	234023	Arglu1	55082	-1.483831244	0.17223
8547	216440	Os9	10956	0.173035648	0.99988

8548	18432	Mybbp1a	10514	0.168927347	0.84103
8549	207704	Gtpbp10	85865	0.88488321	0.05532
8550	233016	Blvrb	645	0.685581979	0.00954
8551	56187	Rabggta	5875	-1.012953077	0.14961
8552	207686	Cfap69	79846	0.410461448	0.64026
8553	207165	Bptf	2186	-0.579048181	0.45517
8554	112403	Dxo	1797	0.395426886	0.45207
8555	327959	Xaf1	54739	0.254585035	0.88929
8556	108075	Ltbp4	8425	1.856841233	0.07496
8557	216438	Marchf9	92979	-0.378723966	0.6896
8558	228361	Ambra1	55626	0.282447198	0.58339
8559	52118	Pvr	5817	0.309421873	0.56772
8560	242362	Manea	79694	-0.017471327	0.99988
8561	66399	Tsfm	10102	-0.137761976	0.92082
8562	214812	Zfp609	23060	-2.543066405	0.04845
8563	80794	Cblc	23624	-0.572775265	0.49296
8564	380732	Milr1	284021	2.130239654	0.00519
8565	68758	Abhd11	83451	-0.237384616	0.67674
8566	69352	Necab1	64168	-0.584386391	0.63937
8567	11496	Adam22	53616	0.364894627	0.72624
8568	21763	Tex2	55852	-0.083438231	0.90774
8569	75786	Ckap5	9793	-1.372052422	0.19812
8570	72201	Otud6b	51633	-0.908742824	0.2523
8571	12267	C3ar1	719	1.869003337	0.00577
8572	79565	Mettl27	155368	-0.738014649	0.33996
8573	104082	Wdr7	23335	-0.896811546	0.28465
8574	14863	Gstm2		-0.162056135	0.89298
8575	235044	Plppr2	64748	-0.520138407	0.4406
8576	11812	Apoc1	341	-0.14076093	0.99988
8577	107182	Btaf1	9044	0.261803934	0.94097
8578	242819	Rundc3b	154661		0.40878
8579	18671	Abcb1a		1.40020145	0.1366
8580	237222	Ofd1	8481	-0.682449845	0.40385
8581	228356	110051M20R	79096	-0.088275899	0.99988
8582	15985	Cd79b	974	2.398056666	0.00516
8583	66011	Ranbp17	64901	-0.604378857	0.81794
8584	71592	Pogk	57645	-0.450489344	0.55466
8585	67139	Mis12	79003	-0.354214219	0.41423
8586	56175	Bace2	25825	0.745760311	0.0634
8587	71529	Kazn	23254	-0.051961191	0.99988
8588	100039795	Ildr2	387597	0.559703112	0.55816
8589	11810	Apobec1	339	-0.476841048	0.99988
8590	214359	Tmem51	55092	-0.121894997	0.99988
8591	74551	Pck2	5106	0.377003959	0.44193
8592	216877	Dhx33	56919	0.561335178	0.76175
8593	237221	Gemin8	54960	0.094830576	0.99074
8594	213783	Plekhg1	57480	-0.993131582	0.1997
8595	227399	Ppip5k2	23262	-0.0478039	0.98391
8596	108653	Rimklb	57494	-1.740886664	0.13094
8597	218850	Tasor	23272	-0.039534486	0.93937

8598	18247	Oaz2	4947	0.118454948	0.919
8599	381101	Dnph1	10591	-1.065464803	0.40517
8600	27984	Efhd2	79180	0.583879778	0.02103
8601	81000	Rad54l2	23132	0.585887678	0.57665
8602	19069	Nup88	4927	-0.288128332	0.65683
8603	13619	Phc1	1911	0.532304759	0.94082
8604	270685	Mthfd1l	25902	-0.503177385	0.42626
8605	15312	Hmgn1	3150	0.666880309	0.12932
8606	228355	Madd	8567	0.113185172	0.91811
8607	213773	Tbl3	10607	-0.443154985	0.55994
8608	107581	Col16a1	1307	0.162368635	0.98982
8609	214063	Dnajc16	23341	0.720333146	0.29785
8610	67803	Limd2	80774	0.533119801	0.44818
8611	11766	Ap1g2	8906	-0.163435007	0.93475
8612	74134	Cyp2s1	29785	-0.385343849	0.92471
8613	20452	St8sia4	7903	1.01184243	0.16413
8614	268396	Sh3pxd2b	285590	-0.120074299	0.99988
8615	216874	Camta2	23125	0.174693717	0.99988
8616	433375	Creg1	8804	0.203028606	0.0783
8617	171463	Il17rd	54756	0.285795456	0.63624
8618	66185	Virma	25962	0.049617239	0.96072
8619	239102	Zfhx2	85446	0.066629345	0.98477
8620	56807	Scamp5	192683	0.797571468	0.05037
8621	226594	Rcsd1	92241	0.86225515	0.17434
8622	16490	Kcna2	3737	0.369690439	0.16168
8623	232989	Hnrnpul1	11100	-0.232866269	0.99988
8624	207920	Esrp1	54845	-0.652475517	0.36204
8625	22384	Eif4h	7458	0.418652359	0.27121
8626	333654	Ppp1r13l	10848	-0.561039803	0.25705
8627	72656	Ints8	55656	0.797923129	0.01399
8628	70510	Rnf167	26001	-0.443822853	0.68253
8629	12508	Cd53	963	1.197456985	0.0074
8630	56743	Lat2	7462	1.835141845	0.0066
8631	72993	Appl1	26060	-0.661383869	0.30504
8632	56381	Spen	23013	-0.786931062	0.44912
8633	78372	Snrnp25	79622	0.581706987	0.26837
8634	106821	Oard1	221443	0.371614733	0.65178
8635	99712	Cept1	10390	0.9636112	0.01371
8636	26374	Cop1	64326	0.349178954	0.45844
8637	22129	Ttc3	7267	-0.252544549	0.76318
8638	67445	C1qtnf4	114900	0.458805873	0.906
8639	230767	Iqcc	55721	0.273721333	0.99303
8640	12309	S100g	795	0.382374009	0.98477
8641	72205	Eml2	24139	-0.82041237	0.47417
8642	21767	Tex264	51368	1.069218826	0.50224
8643	211922	Dennd6a	201627	-0.00561766	0.99988
8644	110948	Hlcs	3141	0.665835137	0.7594
8645	58248	700123O20R	55017	-0.526484505	0.36746
8646	107686	Snrpd2	6633	-0.118599374	0.57121
8647	106757	Catsperd	257062	-0.74634095	0.3235

8648	240888	Gpr161	23432	0.063975134	0.99988
8649	20475	Six5	147912	0.915227418	0.35847
8650	213491	Szrd1	26099	-0.147732668	0.89847
8651	226591	Tiprl	261726	0.511463119	0.32201
8652	108735	Sft2d2	375035	-0.35282889	0.54834
8653	103554	Psme4	23198	-0.37023984	0.42825
8654	213556	Plekhh2	130271	-0.893752333	0.28564
8655	194590	Reps2	9185	0.653869652	0.27661
8656	13875	Erf	2077	0.919578818	0.33679
8657	100383	Bsdc1	55108	-1.065588217	0.38798
8658	230872	Crocc	9696	-0.163093406	0.99988
8659	227195	Ino80d	54891	0.294624037	0.86157
8660	74486	Osbpl10	114884	-0.5295112	0.87223
8661	212198	Wdr25	79446	0.257620156	0.88911
8662	235043	Tmem205	374882	-0.27547062	0.99024
8663	11692	Gfer	2671	-0.07967964	0.85235
8664		Gm21988		-1.072288733	0.15006
8665	30838	Fbxw4	6468	0.246778633	0.3547
8666	116914	Slc19a2	10560	-0.154045014	0.86073
8667	74648	S100pbp	64766	-0.718751364	0.3364
8668	209012	Ulk4	54986	0.445365121	0.32988
8669	216867	Slc16a11	162515	0.565412315	0.54208
8670	16801	Arhgef1	9138	0.461888875	0.25324
8671	214133	Tet2	54790	-1.284003645	0.15768
8672	108911	Rcc2	55920	-0.251154866	0.81665
8673	216864	Mgl2		2.775619629	0.00406
8674	20085	Rps19	6223	0.241685195	0.48431
8675	63955	Cables1	91768	0.516206161	0.97774
8676	11890	Asgr2	433	1.637054749	0.0077
8677	72754	Arhgef10l	55160	1.376452233	0.01496
8678	77669	Arhgef38	54848	-0.497719344	0.60279
8679	243864	Mill2		0.42650213	0.03739
8680	58194	Sh3kbp1	30011	0.000646489	0.99988
8681	105501	Abhd4	63874	0.209262469	0.48558
8682	114249	Npnt	255743	1.775134323	0.00549
8683	70031	Cmtm8	152189	-0.815618709	0.27456
8684	78283	Map7d2	256714	-0.215821351	0.75947
8685	212632	Iffo2		0.044598202	0.9462
8686	66092	Ghitm	27069	0.609699	0.01855
8687	226162	Gm17018	25911	-0.340296508	0.57219
8688	210146	Irgq	126298	-0.75161878	0.41466
8689	72750	Fam117b	150864	-0.068004711	0.97675
8690	72515	Wdr43	23160	-1.029933827	0.27121
8691	107568	Wwp1	11059	-0.390476906	0.70367
8692	14369	Fzd7	8324	-1.31071497	0.34612
8693	53858	Rwdd2b	10069	0.059562506	0.99988
8694	66357	Ostc	58505	0.889314165	0.02005
8695	52808	Tspyl2	64061	-2.355798048	0.04402
8696	140580	Elmo1	9844	1.910688266	0.00837
8697	245666	Iqsec2	23096	-0.029919504	0.99988

8698	18585	Pde9a	5152	-0.149997493	0.92721
8699	17965	Nbl1	4681	0.127766725	0.99988
8700	78100	Msantd4	84437	-0.068632277	0.99988
8701	77605	H2az2	94239	0.43810899	0.09967
8702	213753	Zfp598	90850	-0.120779565	0.92609
8703	100637	N4bp2l1	90634	0.489906282	0.33219
8704	24061	Smc1a	8243	-1.132006326	0.19517
8705	224405	Cyyr1	116159	-1.908688401	0.09142
8706	192656	Ripk2	8767	-0.792953404	0.25555
8707	77056	Tmco4	255104	-0.189413807	0.88439
8708	227058	Dnah7b		-0.851321495	0.35568
8709	12190	Brca2	675	-1.61200383	0.22722
8710	209212	Osgin2	734	0.372645608	0.5098
8711	73162	Otud3	23252	-1.175840362	0.31004
8712	52915	Zmiz2	83637	-0.183635006	0.99988
8713	74142	Lonp1	9361	-0.811388256	0.3364
8714	101540	Prkd2	25865	0.860260785	0.27378
8715	11443	Chrnb1	1140	0.70840205	0.0431
8716	18784	Pla2g5	5322	1.134607081	0.00933
8717	18782	Pla2g2d	26279	0.894293016	0.42676
8718	68544	Trir		-0.699069355	0.31306
8719	208146	Yeats2	55689	-0.471308174	0.43981
8720	74438	Clvs1	157807	0.155495481	0.53205
8721	228482	Arhgap11a		-1.092634428	0.19556
8722	170439	Elovl6	79071	0.164944593	0.99988
8723	75415	Arhgap12	94134	0.102552492	0.89195
8724	320595	Phf8	23133	0.582238776	0.01855
8725	79560	Ublcp1	134510	-0.055777148	0.99988
8726	320790	Chd7	55636	-1.225934346	0.16967
8727	218035	Vps41	27072	-0.391037478	0.53653
8728	225182	Rbbp8	5932	0.511168566	0.05216
8729	68350	Mul1	79594	0.211614389	0.98901
8730	329002	Zfp236	7776	0.492110715	0.69311
8731	12319	Car8	767	0.313301595	0.74046
8732	72296	Rusc1	23623	1.017440384	0.01285
8733	231915	Uspl1	10208	-0.328389132	0.57893
8734	235380	Dmxl2	23312	-0.082072539	0.93792
8735	252838	Tox	9760	0.812038332	0.41716
8736	66827	Ttc1	7265	-0.340541109	0.50723
8737	69562	Cdk13	8621	-0.688756825	0.34797
8738	231912	Katnal1	84056	0.323435955	0.17093
8739	12638	Cftr	1080	-1.184090054	0.22765
8740	98488	Gtf3c3	9330	0.357552728	0.34681
8741	20650	Sntb2	6645	-0.957397704	0.3352
8742	11987	Slc7a1	6541	-0.463983603	0.64609
8743	386612	Thoc6	79228	0.636078705	0.01784
8744	16323	Inhba	3624	1.061544842	0.01054
8745	74737	Pcf11	51585	-0.559407862	0.61562
8746	76559	Atg2b	55102	-0.299042387	0.49619
8747	73845	Ankrd42	338699	0.442809408	0.93894

8748	216853	Wrap53	55135	-0.515750642	0.55573
8749	110351	Rap1gap	5909	0.798853444	0.23468
8750	382245	Tmem29		-0.000557863	0.99989
8751	19732	Rgl2	5863	-0.100296522	0.84237
8752	66256	Ssr2	6746	-0.146703591	0.62649
8753	52874	Pum3	9933	-0.15725571	0.88929
8754	102371	Myzap		-0.009341639	0.99988
8755	71653	Shtn1	57698	-0.677835161	0.40346
8756	243846	Ccdc9	26093	0.391425637	0.97932
8757	16543	Mdfic	29969	0.994084613	0.00773
8758	69962	Mettl18	92342	0.463887043	0.51006
8759	218914	Wapl	23063	-0.456373187	0.39375
8760	192119	Dicer1	23405	-0.419330153	0.48431
8761	18708	Pik3r1	5295	-1.077933363	0.24007
8762	17537	Meis3	56917	-0.888015122	0.24042
8763	227095	Hibch	26275	0.613287702	0.02589
8764	268697	Ccnb1	891	-1.08234608	0.32299
8765	21771	Utp4	84916	1.091695822	0.01298
8766	98682	Mfsd6	54842	-0.9444397	0.25527
8767	235533	Gk5	256356	0.069686296	0.99988
8768	330914	Arhgap32	9743	-0.878056443	0.43674
8769	105450	Mmrn2	79812	0.186310385	0.81659
8770	19933	Rpl21		0.007837098	0.99988
8771	230908	Tardbp	23435	0.287148423	0.75283
8772	75698	Shld2	54537	0.179711738	0.99988
8773	319618	Dcp1b	196513	0.425880148	0.04193
8774	20715	Serpina3g		0.643041315	0.33414
8775	667742	Piezo2	63895	-2.813749413	0.03739
8776	226442	Zfp281	23528	-0.439972773	0.47808
8777	20908	Stx3	6809	-0.873535995	0.26317
8778	27966	Rrp9	9136	-0.492844716	0.42272
8779	15900	Irf8	3394	0.887692765	0.42673
8780	84585	Rnf123	63891	-0.124047618	0.99988
8781	236511	Ago1	26523	0.279021672	0.93097
8782	15002	H2-Ob	3112	1.624880417	0.11149
8783	20678	Sox5	6660	0.244996862	0.75897
8784	80888	Hspb8	26353	-1.575245433	0.12619
8785	14264	Fmod	2331	2.407520928	0.00447
8786	68077	Nop53	29997	-0.383045226	0.44955
8787	67886	Camsap2	23271	0.342477423	0.80869
8788	20364	Selenow	6415	-0.008690742	0.9848
8789	116847	Prelp	5549	1.655924344	0.01186
8790	70551	Tmtc4	84899	1.173558563	0.01033
8791	56699	Cdc42ep4	23580	0.884202211	0.01565
8792	67313	Inava	55765	0.340768679	0.97856
8793	17196	Mbp	4155	0.240841636	0.65326
8794	215446	Entpd3	956	0.72332395	0.02714
8795	75665	Bicdl1	92558	0.44211103	0.61679
8796	276852	D11Wsu47e	55028	1.636433955	0.22364
8797	223267	Ggact	87769	0.046332704	0.99988

8798	28081	Fam104a	84923	0.100351194	0.99988
8799	218506	Mrps27	23107	0.207316129	0.74567
8800	207474	Kctd12b		0.240079919	0.86157
8801	231659	Gcn1	10985	-1.108830412	0.1583
8802	16565	Kif21b	23046	-0.983255201	0.34138
8803	27225	Ddx24	57062	-0.81698441	0.31004
8804	110821	Pcca	5095	-0.533889255	0.65013
8805	69806	Slc39a11	201266	-0.333888016	0.77827
8806	170442	Bbox1	8424	-0.163555732	0.99988
8807	232491	Pyroxd1	79912	-0.127303551	0.99988
8808	246229	Bivm		0.014394562	0.99988
8809	218503	Fcho2	115548	-0.407351847	0.6516
8810	27494	Amot	154796	-0.470574381	0.67911
8811	12861	Cox6a1	1337	0.235746444	0.85685
8812	237091	Lhfpl1	340596	0.406485182	0.96063
8813	238386	Btbd7	55727	-0.094288665	0.96014
8814	69069	Tmem273	170371	0.257890144	0.20884
8815	66622	Ubr7	55148	0.619381547	0.02459
8816	100859931	Gm20604		0.419831363	0.99988
8817	67574	Alg13	79868	0.617921585	0.38023
8818	224020	Pi4ka	5297	0.29982421	0.65122
8819	235431	Coro2b	10391	-0.005758376	0.99988
8820	226041	Pgm5	5239	2.906997569	0.00388
8821	52064	Coq5	84274	-0.743633604	0.33361
8822	12257	Tspo	706	1.005394935	0.01215
8823	235135	Tmem45b	120224	1.516940179	0.00664
8824	50849	Rnf10	9921	-0.695723769	0.57293
8825	54611	Pde3a	5139	-1.018624573	0.29751
8826	105372	Utp15	84135	-1.112540058	0.24426
8827	240753	Plekha6	22874	-0.114557347	0.96929
8828	68526	Gpr155	151556	-1.022853167	0.25533
8829	22019	Tpp2	7174	-0.35952722	0.46805
8830	68889	Ubac2	337867	0.201121148	0.99988
8831	52432	Ppp2r2d	55844	-0.843751167	0.27665
8832	13803	Enc1	8507	-0.603269468	0.45745
8833	69101	Ydjc	150223	-0.2067349	0.98477
8834	69546	Mapk1ip1		0.311251604	0.77941
8835	66935	Cir1	9541	-0.163017578	0.62747
8836	170829	Tram2	9697	0.997111028	0.01378
8837	51786	Cpsf2	53981	-0.182613049	0.6635
8838	16763	Lad1	3898	-1.032911813	0.18906
8839	217262	Abca9	10350	0.101453231	0.93704
8840	27280	Phlda3	23612	-0.771047132	0.32027
8841	71877	Efhc1	114327	1.059159981	0.08172
8842	73826	Poldip3	84271	-0.442068862	0.59203
8843	320557	Fam169a	26049	0.28579286	0.64563
8844	231655	Oasl1	8638	0.17154345	0.96896
8845	217258	Abca8a		1.39598234	0.02285
8846	83672	Sytl3	94120	0.488214688	0.80294
8847	19267	Ptpre	5791	1.584199629	0.00888

8848	50996	Pdcd7	10081	0.01638061	0.99988
8849	225745	Haus1	115106	-0.335094247	0.58823
8850	67281	Rpl37	6167	0.083227925	0.87239
8851	229474	Fhdc1	85462	1.215399338	0.0912
8852	11854	Rhod	29984	-0.234612258	0.9787
8853	68734	Ppp4r3a	55671	0.00229142	0.99989
8854	239319	Card6	84674	1.296186904	0.12452
8855	21411	Tcf20		-0.621597072	0.30663
8856	17215	Mcm3	4172	-0.498041003	0.41362
8857	68420	Ankrd13a	88455	-0.448773654	0.41147
8858	226432	Ipo9	55705	-1.169690841	0.1399
8859	66416	Ndufa7		0.027443037	0.99988
8860	238455	Macc1	346389	-0.051159807	0.99988
8861	77552	Shisa4	149345	0.741515505	0.01105
8862	26431	Git2	9815	-0.292366956	0.57665
8863	70361	Lman1	3998	-0.07098191	0.87164
8864	52639	Wipi1	55062	0.177066756	0.61695
8865	72634	Tdrkh	11022	0.193951859	0.24223
8866	225339	Ammecr1l	83607	-1.356261706	0.18841
8867	104681	Slc16a6	9120	-0.879052061	0.41179
8868	66559	Metap1d	254042	-0.473283379	0.94978
8869	215615	Rnpep	6051	0.072024174	0.99988
8870	106064	AW549877	285636	-0.092205352	0.99988
8871	11603	Agrn	375790	-0.416727309	0.39125
8872	17855	Mvk	4598	-0.546137619	0.57219
8873	272538	Tango6	79613	-0.622920058	0.36006
8874	21936	Tnfrsf18	8784	0.563062283	0.20334
8875	67451	Pkp2	5318	-0.05315031	0.99988
8876	276846	Pigs	94005	0.191952527	0.99988
8877	20194	S100a10	6281	0.711466532	0.03316
8878	407821	Znrf3	84133	-0.260966406	0.66729
8879	75763	Dcaf17	80067	-0.658975946	0.38293
8880	224008	Spidr	23514	0.230624887	0.69983
8881	228019	Mettl8	79828	0.961313382	0.50713
8882	213498	Arhgef11	9826	-0.447860082	0.49656
8883	217944	Rapgef5	9771	-0.306545747	0.68954
8884	72114	Zbed3	84327	-0.078424975	0.957
8885	228012	Tlk1	9874	-0.654797412	0.37624
8886	100705	Acacb	32	1.373414354	0.00544
8887	218460	Wdr41	55255	0.946478742	0.01085
8888	76044	Ncapg2	54892	-1.192570624	0.27447
8889	108645	Mat2b	27430	0.955460254	0.02594
8890	78908	Igsf3	3321	-0.835451088	0.27327
8891	78752	Csgalnact2	55454	0.471280054	0.41669
8892	21371	Tbca	6902	0.42152217	0.0435
8893	213452	Dstyk	25778	0.013085149	0.99988
8894	217935	Dync2i1	55112	-1.248286743	0.17777
8895	207425	Wdr11	55717	0.471538005	0.23545
8896	56220	Zfp386		-0.881761492	0.26685
8897	329421	Myo3b	140469		0.991

8898	68875	Tmcc2	9911	1.727336869	0.00544
8899	76491	Abhd14b	84836	-0.59016424	0.34186
8900	98758	Hnrnpf	3185	-0.099370911	0.99988
8901	11881	Arsb	411	-0.783459167	0.41378
8902	22685	Zfp239	8187	0.476888238	0.05117
8903	80880	Kank3	256949	0.222392597	0.88874
8904	66435	Uggt2	55757	-0.973762532	0.45085
8905	101490	Inpp5f	22876	-0.138958306	0.93365
8906	69668	Ccdc115	84317	0.326055105	0.678
8907	213417	Klhdc8a	55220	-0.627752357	0.40234
8908	246228	Vwa1	64856	0.637452684	0.52043
8909	231637	Ssh1	54434	0.608169981	0.72061
8910	213391	Rassf4	83937	0.363510395	0.37598
8911	228005	Ppig	9360	-0.816214563	0.2287
8912	235184	Msantd2	79684	1.346089933	0.19672
8913	70383	Cox10	1352	-0.416552964	0.73063
8914	277396	Klhl23	151230	0.917341803	0.55705
8915	66573	Dzip1	22873	-0.496876037	0.95081
8916		Gm9774		-0.659917191	0.36752
8917	100715	Tent2	167153	-0.35189817	0.92702
8918	233912	Armc5	79798	-0.255300997	0.85104
8919	320705	Bend6	221336	0.179422917	0.99988
8920	235134	Nfrkb	4798	-1.165464981	0.22946
8921	14747	Cmklr1	1240	0.154890677	0.77368
8922	98403	Zfp451	26036	-0.042397327	0.96966
8923	66433	Chchd7	79145	-0.103528044	0.99988
8924	320541	Slc35e2	728661	-1.289291715	0.21296
8925	381085	Tbc1d22b	55633	-0.059921755	0.99988
8926	75605	Kdm5b	10765	-0.75864582	0.17458
8927	71675	610010F05Ri	84542	-0.412294015	0.40266
8928	68644	Abhd14a	25864	0.228368125	0.13172
8929	107035	Fbxo38	81545	0.17162377	0.99988
8930	213539	Bag2	9532	-0.044338314	0.90134
8931	56068	Ammecr1	9949	-0.218159316	0.68689
8932	17096	Lyn	4067	1.328394698	0.00766
8933	98710	Rabif	5877	0.197102345	0.63286
8934	209760	Tmc7	79905	1.1351932	0.18338
8935	320129	Grk3	157	-0.238145153	0.72791
8936	214425	Cilp	8483	2.084295821	0.00504
8937	16392	Isl1	3670	1.552253153	0.13576
8938	58217	Trem1	54210	-0.605729889	0.92729
8939	237082	Nxt2	55916	0.066735315	0.99988
8940	228071	Sestd1	91404	0.951347053	0.01098
8941	109700	Itga1	3672	0.405179895	0.63858
8942	192187	Stab1	23166	1.166591157	0.01064
8943	101502	Hsd3b7	80270	0.376728845	0.57301
8944	223701	Mrtfa	57591	0.048577565	0.99988
8945	434402	Gm5617	54494	0.518349484	0.991
8946	72795	Ttc19	54902	0.007977614	0.99988
8947	216565	Ehbp1	23301	0.42176079	0.38367

8948	105835	Sgsm3	27352	-0.743361835	0.32309
8949	57439	Tmem183a	92703	-0.824979882	0.24738
8950	66166	S100a14	57402	-0.04073101	0.99988
8951	233904	Setd1a		-0.330863781	0.71131
8952	20196	S100a13	6284	0.845370296	0.14939
8953	66923	Pbrm1	55193	-0.348463698	0.47454
8954	192232	Hps4	89781	0.211225745	0.99988
8955	432572	Specc1	92521	-1.654478005	0.10386
8956	13019	Ctf1	1489	0.785676849	0.09527
8957	218639	Arl15	54622	0.55645089	0.92311
8958	56489	Ikbke	9641	0.265572651	0.15536
8959	17444	Grap2	9402		0.40878
8960	30877	Gnl3	26354	-0.083730855	0.89195
8961	14622	Gjb5	2709	0.508925263	0.50058
8962	99031	Osbpl6	114880	0.170131252	0.93889
8963	216551	Lgalsl		-0.328766026	0.6706
8964	170625	Snx18	112574	-0.495868791	0.46399
8965	14620	Gjb3	2707	-1.4981069	0.1388
8966	241490	Rbm45	129831	-0.119413729	0.87223
8967	69640	Fam83g	644815	-1.182636818	0.33679
8968	80284	Smim12	113444	0.253096521	0.96714
8969	14945	Gzmk	3003	1.578118051	0.00972
8970	381802	Tsen2	80746	-2.23945675	0.12734
8971	229542	Gatad2b	57459	-0.659051874	0.46233
8972	67010	Rbm7		0.048761889	0.90774
8973	229541	Dennd4b	9909	-0.033627601	0.99988
8974	11911	Atf4	468	-0.464660102	0.59826
8975	100177	Zmym6	9204	-0.391507036	0.41669
8976	228061	Agps	8540	0.926395885	0.00908
8977	14123	Fbrs	64319	0.656984084	0.02031
8978	218629	Dhx29	54505	-0.354840576	0.85226
8979	17309	Mgat3	4248	0.345785789	0.96771
8980	76293	Mfap4	4239	0.595944512	0.0262
8981	328977	Zfp532	55205	0.245971025	0.96083
8982	235461	Mindy2	54629	0.773487414	0.04129
8983	67785	Zmym4	9202	-0.368249988	0.72923
8984	252875	Mios	54468	-0.380881313	0.46908
8985	19699	Reln	5649	1.90231706	0.00407
8986	94192	C1galt1	56913	0.176301276	0.98477
8987	66422	Dctpp1	79077	0.528725868	0.09527
8988	52708	Zfp410	57862	-0.165358001	0.99632
8989	66175	Mustn1	389125	1.380669306	0.00832
8990	235497	Leo1	123169	0.161480294	0.99988
8991	269582	Clspn	63967	0.65878511	0.29572
8992	68449	Tbc1d10b	26000	0.402020086	0.04622
8993	382066	Prdm10	56980	0.358482117	0.906
8994	102871	Radx	55086	2.207979072	0.0929
8995	15431	Hoxd11	3237	0.701324799	0.02959
8996	76850	Ago4	192670	-0.8401098	0.35517
8997	70233	Cd2bp2	10421	-0.111631887	0.99988

8998	71238	Sdhaf3	57001	0.043947917	0.99988
8999	216825	Usp22	23326	-0.086257658	0.99988
9000	238317	Mideas	91748	-0.70122704	0.39397
9001	23857	Dmtf1	9988	-0.434176639	0.90862
9002	212439	AA986860	79098	0.356389317	0.05266
9003	74383	Ubap2l	9898	-0.513192393	0.51336
9004	105000	Dnal1	83544	-0.742254096	0.5101
9005	223697	Sun2	25777	-0.465938401	0.43545
9006	14904	Gtpbp1	9567	-0.518649277	0.41179
9007	20422	Sem1		0.347733288	0.10988
9008	228790	Asxl1	171023	-0.108100968	0.86915
9009	20466	Sin3a	25942	-0.55797859	0.40369
9010	100206	Adprs	54936	-0.188412382	0.73081
9011	75729	Fam227a	646851	0.258898196	0.06854
9012	216820	Dhrs7b	25979	0.052047302	0.99988
9013	70427	Mier2	54531	-1.246557567	0.10386
9014	70093	Ube2q1	55585	0.206472915	0.14257
9015	74034	632404H12Rik		-1.290030089	0.19717
9016	76582	Ipo11	51194	0.29209334	0.15903
9017	16923	Sh2b3	10019	0.546039402	0.26769
9018	245622	Fam199x	139231	-0.106678612	0.93369
9019	338523	Kdm7a	80853	-0.14200988	0.66875
9020	20239	Atxn2	6311	-0.922562098	0.22363
9021	233876	Hirip3	8479	-0.102222649	0.99988
9022	65255	Asb4	51666	-1.218351975	0.33679
9023	74178	Stk40	83931	0.336378846	0.55409
9024	229534	Pbxip1	57326	-0.146927885	0.93504
9025	230751	Oscp1	127700	-0.537170596	0.51608
9026	17133	Maff	23764	0.388521347	0.94955
9027	224902	Safb2	9667	-0.902067125	0.25871
9028	20416	Shc1	6464	-0.179127518	0.99988
9029	217695	Zfyve1	53349	0.162756147	0.77825
9030	53357	Pla2g6	8398	-0.541352956	0.32067
9031	14917	Gucy2c	2984	1.264748219	0.01409
9032	319945	Flad1	80308	0.707127633	0.99988
9033	16440	Itpr3	3710	-0.783449627	0.40878
9034	338350	Acad12		-0.644452349	0.37299
9035	268420	Alkbh5	54890	-0.249758383	0.80138
9036	66412	Arrdc4	91947	-1.308462536	0.18424
9037	67936	Wdr55	54853	-0.16132507	0.99988
9038	66541	Immp1l	196294	0.16058022	0.64988
9039	66090	Ypel3	83719	-0.067743748	0.99988
9040	230738	Zc3h12a	80149	0.009359054	0.99988
9041	381126	Garem1	64762	-0.680708694	0.38262
9042	80795	Selenok	58515	0.749979682	0.02287
9043	57339	Jph1	56704	0.070579713	0.96415
9044	50772	Mapk6	5597	-1.265925122	0.29232
9045	108689	Stn1	79991	0.413795627	0.56137
9046	13211	Dhx9	1660	-0.423647191	0.45342
9047	217692	Sipa1l1	26037	-0.666770899	0.31475

9048	69456	Commd10	51397	0.686022217	0.22038
9049	246782	Atpaf2	91647	0.52637991	0.6855
9050	22381	Tceal9	51186	-0.04349346	0.99988
9051	231713	Naa25	80018	-0.426237914	0.47029
9052	338372	Map3k9	4293	-0.342513785	0.62168
9053	231712	Trafd1	10906	-0.280401089	0.83517
9054	107071	Wdr74	54663	0.263103139	0.99988
9055	69480	Ttc9	23508	0.576719403	0.01119
9056	68563	Dpm3	54344	0.513905866	0.08382
9057	101148	Bmt2	154743	-0.431605968	0.51344
9058	218544	Sgtb	54557	-0.637248019	0.42129
9059	269700	Hectd4	283450	-0.031884048	0.99988
9060	15901	Id1	3397	1.851600764	0.00634
9061	66059	Krtcap2	200185	-0.12341473	0.99988
9062	12069	Bex2		0.784697962	0.01701
9063	171504	Apobr	55911	0.425684699	0.60204
9064	15199	Hebp1	50865	0.738568622	0.53065
9065	226517	Smg7	9887	-0.005860323	0.99988
9066	17829	Muc1	4582	1.702407461	0.0459
9067	208194	Exog	9941	0.77546518	0.01962
9068	235315	Rnf214	257160	0.235408436	0.37471
9069	66333	Aqp11	282679	0.597363728	0.22629
9070	329152	Hecw2	57520	-0.020908094	0.99988
9071	14776	Gpx2	2877	-1.851949888	0.10445
9072	77827	Krba1	84626	-1.10130492	0.19522
9073	15227	Foxf1	2294	-0.659514355	0.5799
9074	66405	Mcts2		0.117672091	0.93548
9075	20613	Snai1	6615	1.046815156	0.0358
9076	233065	Alkbh6	84964	-0.1220216	0.96072
9077	27528	Nrep	9315	0.052677676	0.98977
9078	20708	Serpinb6b		0.759413786	0.38999
9079	192200	Wfdc12	128488	2.207390847	0.00797
9080	78751	Zc3h6	376940	-0.137966656	0.99988
9081	76367	Trp53rkb		-0.525777949	0.40947
9082	328309	Gm9776		0.409366412	0.98602
9083	21968	Tom1	10043	0.503315179	0.59441
9084	108909	Aida	64853	0.763948801	0.01657
9085	54601	Foxo4	4303	-1.188020059	0.224
9086	268780	Egflam	133584	1.881519396	0.00589
9087	104175	Sbk1	388228	-0.212127139	0.82705
9088	67774	Borcs5	118426	-0.546222097	0.23085
9089	212114	Nhlrc3	387921	-1.226932878	0.22629
9090	242505	Rasef	158158	-0.208482133	0.97993
9091	14702	Gng2	54331	2.13552222	0.04444
9092	239743	Klhl6	89857	1.369960401	0.00621
9093	227094	Nemp2	100131211	-0.326192491	0.87313
9094	19222	Ptgir	5739	-0.721829092	0.35611
9095	66967	Edem3	80267	0.629242787	0.52442
9096	244667	Disc1	27185	0.250663844	0.97905
9097	101023	Zfp513	130557	-1.051276548	0.1946

9098	211986	Tmem18	129787	0.822069849	0.53092
9099	212514	Spice1		0.516481487	0.23077
9100	244745	Dpy19l1	23333	0.264630291	0.97377
9101	104027	Synpo	11346	0.370125997	0.99988
9102	57890	Il17re	132014	-0.718072963	0.5159
9103	330788	Zfp866		0.234712789	0.99988
9104	22146	Tuba1c	84790	0.092690679	0.9519
9105	15248	Hic1	3090	1.351236786	0.23493
9106	232157	Mob1a	55233	0.650499502	0.06411
9107	66690	Tmem186	25880	0.782406058	0.18174
9108	235542	Ppp2r3a	5523	0.030996782	0.99988
9109	219144	Arl11	115761	1.228646328	0.15062
9110	66459	Pyurf	100996939	-0.054920553	0.93215
9111	218341	Rfesd	317671	0.251348088	0.67176
9112	230709	Zmpste24	10269	0.530063142	0.57361
9113	326622	Upf2	26019	0.257069739	0.90907
9114	100201	Tmem64	169200	-0.353280265	0.69537
9115	230801	Pigv	55650	0.658779274	0.25963
9116	54122	Uevld	55293	-0.431407756	0.42644
9117	236312	Ifi209		0.355506949	0.41755
9118	384309	Trim56	81844	-0.501772171	0.45862
9119	433365	Teddm1b		-0.679548755	0.18248
9120	216821	Tmem11	8834	-0.161656483	0.8156
9121	26879	B3galnt1	8706	1.277978089	0.15758
9122	114585	D17H6S53E	57827	0.219095877	0.77984
9123	381668	Fbrsl1	57666	-0.328080589	0.78301
9124	230726	Rhbdl2	54933	-2.726651794	0.03056
9125	78749	Filip1l	11259	-0.474183687	0.36237
9126	15438	Hoxd9	3235	1.171429767	0.16197
9127	67298	Gprasp1	9737	0.422749903	0.58253
9128	72190	510009E07R	285382	0.023857927	0.99988
9129	238252	Gpr135	64582	-1.413897813	0.25473
9130	170707	Usp48	84196	0.109057037	0.99988
9131	71198	Otud1	220213	0.462305588	0.08145
9132	69573	Hilpda	29923	0.407647933	0.03748
9133	100042807	Eif3j2		0.12942539	0.99988
9134	76943	Psapl1	768239	0.260330934	0.39822
9135	243813	Leng9	94059	-0.543388055	0.65242
9136	103551	Epop	100170841	-0.942216371	0.09252
9137	67078	Pgp	283871	-0.695701752	0.37566
9138	240869	Zbtb37	84614	0.15048282	0.89134
9139	217700	Acot6	641372	0.094940517	0.95765
9140	66873	Tril	9865	0.575106174	0.97856
9141	317757	Gimap5		1.698918632	0.00544
9142	100900	Hscb	150274	0.272428437	0.95747
9143	269254	Setx	23064	-0.551660059	0.36633
9144	67306	Zc2hc1a	51101	0.45301626	0.33858
9145	448987	Fbxl7	23194	0.145623429	0.99988
9146	74762	Mdga1	266727	-1.425032782	0.21641
9147	235534	Pxylp1	92370	-1.137959584	0.23561

9148	193043	Zfp3	124961	0.835096657	0.01394
9149	17392	Mmp3	4314	0.941780269	0.14078
9150	194309	Vps37d	155382	1.551278543	0.05573
9151	67080	700019D03R	84281	-0.4728849	0.78756
9152	407800	Ecm2	1842	1.585827149	0.11717
9153	242408	Fam221b	392307	0.045158375	0.99988
9154	66838	610009L18Rik		0.197293788	0.69651
9155	434325	Tmem221		0.477497371	0.26928
9156	244579	Tox3	27324	-1.039004146	0.25473
9157	233115	Dpy19l3	147991	0.271072407	0.04621
9158	238076	Kcns3	3790	-0.334385425	0.61803
9159	69134	Fam25c		-1.215710386	0.34762
9160	14154	Fem1a		0.319995985	0.62812
9161	68918	190005I06Rik		0.347478049	0.88911
9162	211948	Pde12	201626	0.187794902	0.88959
9163	19989	Rpl7	6129	0.259982954	0.67778
9164	245026	Col6a6	131873	-0.75362133	0.46166
9165	19247	Ptpn11	5781	0.031169504	0.99988
9166	320148	430306N03Rik		0.455723949	0.62762
9167	69430	700048O20Rik			0.991
9168	212733	Bicdl2	146439	-0.643964128	0.52857
9169		D830025C05Rik		-0.754828525	0.61871
9170	66548	Adamtsl5	339366	0.77247302	0.0128
9171	75099	Lysmd4	145748	-0.689225689	0.32265
9172	73149	Clec4a3		2.474383989	0.00405
9173	24075	Taf10	6881	-0.394227703	0.69765
9174	68310	Zmym1	79830	1.317064172	0.00994
9175	211255	Kbtbd7	84078	-0.335043246	0.81665
9176	234967	Slc36a4	120103	-0.224328624	0.83725
9177	14739	S1pr2	9294	0.793810417	0.00819
9178	27223	Trp53bp1	7158	-0.93480064	0.24171
9179	382073	Ccdc84	338657	-1.060530779	0.22191
9180	230822	Ncmap	400746	0.336513044	0.82705
9181	236904	Klhl15	80311	1.277329898	0.13274
9182	231932	Gimap7	168537	1.744915721	0.00848
9183	72145	Wdfy3	23001	-0.2080508	0.69639
9184	72560	Naalad2	10003	1.70282714	0.00662
9185	54199	Ccrl2	9034	0.015070824	0.99988
9186	230753	Thrap3	9967	-0.641754294	0.26008
9187	269999	Orai3	93129	0.283506563	0.05217
9188	13797	Emx2	2018	-1.037674997	0.39375
9189	214552	Cep164	22897	-0.667681319	0.35682
9190	19290	Pura	5813	0.508925084	0.02475
9191		2900052L18Rik		-1.868706169	0.05938
9192	217664	Mgat2	4247	0.695431905	0.00653
9193	243277	Adgrd1	283383	1.354992245	0.02461
9194	28028	Mrpl50	54534	0.028782505	0.89993
9195		NA		2.002394596	0.0111
9196	240660	Slc35g1	159371	0.747576975	0.01895
9197	272359	Irf2bp1	26145	0.41854353	0.6316

9198	545428	Ccdc141	285025	-0.764384203	0.70675
9199	235633	Als2cl	259173	0.821603072	0.4229
9200	16663	Krt13		0.41945838	0.94202
9201	14260	Fmn1	342184	-0.617570358	0.48409
9202	93885	Pcdhb14		-0.520587275	0.6664
9203	216543	Cep68	23177	-1.671962492	0.19344
9204	22183	Zrsr1	8233	-0.341614183	0.58933
9205	20193	S100a1	6271	0.590045633	0.47203
9206	229675	Rsbn1	54665	-0.086886737	0.99988
9207	215257	Il1f9	56300	0.442317522	0.59106
9208	231717	Pheta1	144717	-0.828561575	0.40183
9209	329509	810024B03Rik		-0.100305021	0.99988
9210	11845	Arf6	382	-0.352027047	0.58523
9211	69155	810030O07R	159013	0.666251018	0.01803
9212	77286	Nkrf	55922	0.780677092	0.01732
9213	382252	Bclaf3	256643	-1.362898023	0.13083
9214	76522	Lsm8	51691	0.944151071	0.10082
9215	101202	Hepacam2	253012	1.379505158	0.00832
9216	414084	Tnip3	79931	-0.851076929	0.47555
9217	229672	Bcl2l15	440603	-1.842665829	0.0843
9218	56458	Foxo1	2308	-0.009892791	0.99988
9219	278507	Wfikkn2	124857	-1.137895629	0.45367
9220	80290	Gpr146	115330	0.847436814	0.62844
9221	11830	Aqp5	362	-0.079054062	0.98154
9222	231413	Grsf1	2926	0.279851933	0.05165
9223	78244	Dnajc21	134218	-0.356556541	0.62816
9224	244853	Nxpe4	54827	-0.940868264	0.69368
9225	105193	Nhlrc1	378884	-0.326193708	0.94092
9226	213208	Il20rb	53833	0.333467646	0.99988
9227	239647	Pced1b	91523	0.088638332	0.96921
9228	64291	Osbpl1a	114876	-1.22995711	0.15272
9229	100102	Pcsk9	255738	-1.277608159	0.05245
9230	13024	Ctla2a		0.250766802	0.66292
9231		Gm4895		-1.858126064	0.11197
9232	224912	Crb3	92359	-0.444726537	0.54309
9233	68795	Ubr3	130507	1.088292695	0.00985
9234	216964	Trp53i13	90313	-1.034807231	0.1639
9235	12778	Ackr3	57007	1.503053417	0.00932
9236	231642	Alkbh2	121642		0.991
9237	98432	Phlpp1	23239	-0.056239532	0.99988
9238	277010	Marveld1	83742	-0.369327047	0.69127
9239	67062	Slc25a53	401612	0.867540087	0.01788
9240	319317	Snhg11		-0.939966824	0.45542
9241	210808	Lacc1		-0.941150852	0.38012
9242	319478	Cxxc4	80319	0.046414381	0.99988
9243	69309	Slc16a13	201232	-0.275497925	0.81517
9244	13511	Dsg2		-0.786103005	0.27782
9245	246747	Adig	149685	-0.26738321	0.31767
9246	104725	Sptssa	171546	-0.219147072	0.76344
9247	67768	N6amt1	29104	-1.513823988	0.1181

9248	68813	Dock5	80005	-2.100476632	0.11096
9249	668501	Zfp507	22847	-0.248138289	0.8193
9250	217835	Rin3	79890	0.512831239	0.67481
9251	219134	Shisa2	387914	-0.620428249	0.37044
9252	74349	Fam160a2	84067	-0.931203451	0.29364
9253	74645	Tent5c	54855	-0.00869164	0.99988
9254	66443	Tnfaip8l1	126282	0.671072277	0.33224
9255	232685	Lncpint		-1.111615819	0.24531
9256	69090	Ascc1	51008	-0.126637593	0.99988
9257	21769	Zfand3	60685	-0.154730459	0.99988
9258	77034	510039O18R	90231	-0.530340503	0.65044
9259	224598	Zfp758		0.579641824	0.42633
9260	69556	Bod1		0.031076883	0.99988
9261	16898	Rps2	6187	-0.247940676	0.99988
9262	59036	Dact1	51339	1.658870381	0.00721
9263	268759	930012K11R	541565	0.883125859	0.2233
9264	11431	Acp1	52	-0.786319631	0.28594
9265	170743	Tlr7	51284	1.289302966	0.10683
9266	66818	Smim7	79086	-0.235787357	0.89076
9267	320080	Zbtb39	9880	0.162708629	0.99988
9268	239759	Liph	200879	-1.339057999	0.12452
9269	72931	Swi5	375757	0.46289642	0.0366
9270	68846	Rnf208	727800	0.082134692	0.99988
9271	380686	Cnrip1	25927	-0.35271696	0.68951
9272	207785	Csrnp2	81566	-0.17255719	0.56911
9273	58220	Pard6b	84612	-0.746973849	0.33768
9274	207259	Zbtb7c	201501	1.302737023	0.24003
9275	14362	Fzd1	8321	1.22584179	0.0104
9276	234725	Zfp612	7571	-0.267528007	0.99988
9277	76486	Ly6k	54742	0.722914973	0.9492
9278		2010007H06Rik		-0.533826556	0.60105
9279	230917	Tmem201	199953	-0.154172099	0.99988
9280	233826	Palb2	79728	0.288761986	0.65255
9281	69731	Gemin7	79760	-0.78453461	0.35657
9282	625098	Slc38a6	145389	0.165415352	0.99988
9283	66787	Gskip	51527	-0.736843751	0.4291
9284	240216	230025N22Rik		-0.088351953	0.99988
9285	66222	Serpinb1a		-0.301611367	0.61072
9286	13214	Defb1		0.061516661	0.99988
9287	52575	Trmt10c	54931	-0.268924644	0.99034
9288	52392	Macir	90355	-0.187815668	0.99988
9289	268297	Scml4	256380		0.991
9290	212427	Hjurp	55355	-0.056281172	0.99988
9291	22695	Zfp36	7538	0.5576979	0.28856
9292	328035	Fads6	283985	-0.042541476	0.99838
9293	235626	Setd2	29072	0.302496234	0.39846
9294	69046	Isca1		0.461974916	0.02116
9295	327951	Cyb5d1	124637	-0.460752338	0.89195
9296	30944	Zfp354c	30832	-0.110949793	0.85744
9297	140497	Cd300c2		1.847397173	0.00469

9298	230126	Shb		-0.074321673	0.86113
9299	21897	Tlr1	7096	2.584841132	0.00504
9300	72290	Lsm11	134353	-0.493367805	0.52258
9301	73363	700056E22Rik		-0.053812189	0.99988
9302	224640	Lemd2	221496	-0.354401479	0.52634
9303	99696	Ankrd50	57182	-0.952226333	0.2234
9304	72667	Zfp444	55311	-0.053457919	0.991
9305	68185	Coa4	51287	0.952776488	0.79292
9306	22272	Uqcrq	27089	-0.050653645	0.99988
9307	269033	930503L19Ri	162681	-1.221560189	0.41179
9308	237504	Rassf9	9182	-0.921215243	0.27188
9309	243529	H1f10	8971	-0.461292475	0.7422
9310	238673	Zfp367	195828	0.808025889	0.01553
9311	100048534	Cfap43	80217	1.278139566	0.0262
9312	70802	Pwwp2a	114825	-0.489072459	0.51997
9313	622320	Kctd21	283219	-0.01651229	0.99988
9314	110332	Pp2d1	151649	-1.199270719	0.2028
9315	242864	Napepld	222236	-0.098565025	0.99988
9316	546144	Wdr72	256764	-0.652437184	0.46071
9317	67158	Sft2d3	84826	-1.143873289	0.24234
9318	22117	Tst	7263	0.805427176	0.01372
9319	73747	Shld1	149840	-0.482697836	0.75567
9320	14367	Fzd5	7855	-1.027408396	0.20285
9321	103768	Tubg2	27175	0.348499072	0.34967
9322	207175	Cetn4		-0.524685322	0.65504
9323	18754	Prkce	5581	-0.184959065	0.72306
9324	269878	Megf8	1954	-1.195410734	0.17465
9325	225875	Lrfn4	78999	-0.468607937	0.40843
9326	93878	Pcdhb7		0.297855766	0.78261
9327	72350	Zc2hc1c	79696	0.190970476	0.99988
9328	230259	130308A19R	158405	-0.634037129	0.31233
9329	108086	Rnf216	54476	-0.489571052	0.39013
9330	13609	S1pr1	1901	1.016701941	0.17405
9331	14924	Magi1	9223	-0.742161857	0.61492
9332	225888	Kmt5b	51111	0.130771152	0.99988
9333	67582	Slc25a26	115286	0.115456026	0.99988
9334	13405	Dmd	1756	0.381632716	0.991
9335	211936	Ccdc73	493860	0.671029183	0.52857
9336	67509	Saysd1	55776	-0.273427376	0.68954
9337	69017	Prrt2	112476	-0.935394579	0.35509
9338	76808	Rpl18a	6142	-0.417053165	0.55477
9339	73710	Tubb2b	347733	-0.509178479	0.57589
9340	70325	Pigw	284098	0.643041315	0.03012
9341	78653	Bola3	388962	0.331137455	0.60489
9342	101602	AI467606	283897	2.078265864	0.00407
9343	71923	Borcs6	54785	0.358939896	0.29464
9344	110380	Shroom2	357	-0.28731325	0.64892
9345	12696	Cirbp	1153	-0.699431278	0.55415
9346	381510	Dpy19l4	286148	-0.071110153	0.98415
9347	70675	Vcpip1	80124	0.005351242	0.99989

9348	213484	Nudt18	79873	-0.056260967	0.98645
9349	68618	Eola1		0.498333101	0.33176
9350	69234	Zfp688	146542	0.684354393	0.02349
9351	232976	Zfp574	64763	-0.729892652	0.63514
9352	378462	Morn2	729967	0.135744798	0.99988
9353	195522	Zfp691	51058	-0.677282094	0.34186
9354	26886	Cenph	64946	0.049629004	0.99988
9355	385668	Lca5l	150082	-0.458372617	0.72035
9356	245404	Dcaf12l1		-0.938721645	0.40947
9357	237847	Rtn4rl1	146760	1.868770026	0.01357
9358	231070	Insig1	3638	1.133283214	0.00985
9359	50907	Preb	10113	-0.18625984	0.73217
9360	218454	Lhfpl2	10184	1.843651873	0.00646
9361	78088	Sowahb	345079	0.608127536	0.64191
9362	68636	Fahd1	81889	-0.877102066	0.33679
9363	227545	Proser2	254427	0.005868262	0.99988
9364	229841	Cenpe	1062	-0.663767726	0.47203
9365	94187	Zfp423	23090	-0.347076676	0.8023
9366	243300	Nyap1	222950	0.806080249	0.1946
9367	244237	Tnfrsf26		0.186494667	0.99988
9368	192652	Wdr81	124997	0.377798707	0.93033
9369	67020	Tmem88	92162	-0.273531803	0.93453
9370	12767	Cxcr4	7852	0.526965707	0.07834
9371	17075	Epcam	4072	-0.694680119	0.34681
9372	217826	Kcnk13	56659	1.785739952	0.00873
9373	79263	Trim39	56658	-0.53755388	0.70304
9374	56043	Akr1e1	83592	-0.011032842	0.99988
9375	668661	410002F23Rik		-0.56297801	0.45895
9376	68861	Dipk2a	205428	1.258075324	0.29693
9377	56258	Hnrnph2	3188	0.521686654	0.26783
9378	212090	Tmem60	85025	-0.197949851	0.99988
9379	68033	Cox19	90639	0.856153606	0.01197
9380		2810002D19Rik		0.167352239	0.98901
9381	101197	Zfp956		-0.254823798	0.61803
9382	269954	Ttll13		0.47001311	0.87288
9383	100683	Trrap	8295	-0.714251544	0.22875
9384	66460	Sys1		0.376027942	0.98145
9385	434377	Zfp560	7675	-0.550666985	0.51113
9386	71986	Ddx28	55794	0.127011215	0.25901
9387	16664	Krt14	3861	0.410164456	0.79436
9388	18619	Penk	5179	1.766564185	0.09744
9389	229681	St7l	54879	-0.303004404	0.73536
9390	12091	Glb1	2720	0.836687251	0.01279
9391	233887	Zfp553	197407	-1.732908194	0.12554
9392	66580	Esf1	51575	-0.703776928	0.26897
9393	225608	Sh3tc2	79628	-0.396513198	0.59242
9394	102103	Mtus1	57509	0.215543369	0.85104
9395	320683	Zfp629	23361	-1.059043509	0.23526
9396	98496	Pid1	55022	1.976880617	0.0533
9397	233765	Plekha7	144100	-1.129460097	0.26781

9398	104252	Cdc42ep2	10435	0.263969895	0.79595
9399	106073	Mfsd5	84975	0.670715401	0.28119
9400	276829	Smtnl2	342527	1.168443756	0.01648
9401	114716	Spred2	200734	-0.124154131	0.99988
9402	373864	Col27a1	85301	0.020085921	0.82705
9403	217430	Pqlc3	130814	-0.072587133	0.99988
9404	72338	Wdr89	112840	0.057825219	0.99988
9405	78004	Prr15	222171	-0.521629599	0.45207
9406	212377	Mms22l	253714	0.761252513	0.17434
9407	56844	Tssc4	10078	-0.303449093	0.8274
9408	233893	Zfp764		0.538346384	0.2699
9409	70350	Basp1	10409	-0.534479133	0.39859
9410	78521	230219D22R	134553	0.375594016	0.59593
9411	100503884	Ccdc149	91050	0.496730394	0.28159
9412	434204	Whamm	123720	0.038551512	0.99988
9413		Gm9800		0.05195133	0.99988
9414	12193	Zfp36l2	678	1.578134439	0.00504
9415	19265	Ptprcap	5790	0.479502161	0.03056
9416	20723	Serpinb9		0.727803917	0.02001
9417	214239	Ccdc9b	388115	0.175826965	0.99988
9418	108755	Lyrm2	57226	0.836630476	0.01207
9419	12905	Cradd	8738	0.633567629	0.33214
9420	74558	Gvin1		1.453211064	0.00525
9421	239250	Slitrk6	84189	-0.963651419	0.23715
9422	11549	Adra1a	148	0.979025426	0.02136
9423		Gm9803		0.183941467	0.08391
9424	232164	Paip2b	400961	-0.557730454	0.50485
9425	230279	Tmem268	203197	0.604441496	0.25318
9426	66864	Clec14a	161198	-0.655781519	0.44051
9427	24030	Mrps12		-0.268274122	0.70264
9428	20324	Cavin2	8436	0.941677129	0.5007
9429	232341	Wnk1	65125	-0.574778352	0.35044
9430	240817	Teddm2		-0.354382622	0.97395
9431	26356	Ing1	3621	-0.376284901	0.46469
9432	230125	Slc25a51	92014	-0.058320789	0.97706
9433	207781	C2cd2	25966	-0.114610762	0.98761
9434	320534	Tmem104	54868	0.136059585	0.991
9435	208643	Eif4g1	1981	-0.592894152	0.48844
9436	17749	Polr2k	5440	1.108790786	0.00812
9437	66983	Zfp830	91603	0.205185488	0.88704
9438	140484	Pofut1	23509	0.577114829	0.15546
9439	170460	Stard5	80765	0.705433529	0.19956
9440	215900	Calhm6	441168	0.763564476	0.0125
9441	55988	Snx12	29934	0.088579354	0.76163
9442	432940	Otulin	90268	-0.235774595	0.94346
9443	282619	Sbsn	374897	0.06705846	0.99988
9444	386655	Eid2	163126	0.372085508	0.27733
9445	108954	Ppp1r15b	84919	0.558400512	0.02231
9446	16005	Igfals	3483	1.892907724	0.06595
9447	231549	Lrrc8d	55144	0.747809597	0.02728

9448	232414	Clec9a	283420		0.991
9449	233812	Mosmo	730094	0.774779722	0.13917
9450	574418	Serinc4	619189	1.488740255	0.13752
9451	319675	Cep295	85459	-0.887545272	0.29836
9452	240613	930021J03Ri	158358	-1.227371523	0.13768
9453	225929	Patl1	219988	-0.080834702	0.91815
9454	171167	Fut10	84750	-0.419931955	0.52811
9455	268567	Tmem229b	161145	0.239982725	0.92814
9456	12671	Chrm3	1131	-1.431034036	0.25473
9457	108154	Adamts6	11174	0.348499072	0.40182
9458	108961	E2f8	79733	-0.79886064	0.48431
9459	74352	Zfp84	163115	-1.260107632	0.20446
9460	235505	Cd109	135228	0.677708359	0.53116
9461	93891	Pcdhb20	56122	0.04186276	0.84891
9462	106583	Scaf8	22828	0.80070256	0.01557
9463	18793	Plaur	5329	0.28035159	0.99988
9464	19018	Scand1	51282	-0.769518017	0.25175
9465	271564	Vps13a	23230	0.587673061	0.46192
9466	231805	Pilra		-0.101764394	0.97297
9467	54651	Usp27x	389856	0.2666942	0.06854
9468	22720	Zfp62	643836	0.598408246	0.86645
9469	329828	Myorg	57462	1.068688976	0.07536
9470	217517	Stxbp6	29091	0.576762533	0.91426
9471	320924	Ccbe1	147372	-0.476260356	0.91977
9472	226090	Ermp1	79956	0.650481036	0.26528
9473	66972	Slc25a23	79085	-1.081838088	0.11843
9474	19981	Rpl37a	6168	0.177215987	0.7106
9475	218100	Zfp322a	79692	-0.950032974	0.2353
9476	14619	Gjb2	2706	-1.607739986	0.0833
9477	26451	Rpl27a		0.194170103	0.99988
9478	16469	Jrk	8629	-0.415007493	0.99988
9479	93888	Pcdhb17	57717	0.959085432	0.01123
9480	19659	Rbp1	5947	0.302216293	0.98186
9481	226418	Yod1	55432	-0.680944402	0.29838
9482	52150	Kcnk6	9424	1.838403527	0.00416
9483	192976	Lrrc75a	388341	-0.178772503	0.99988
9484	12070	Bex3	27018	-0.527223648	0.35656
9485	15382	Hnrnpa1		-0.083824387	0.89427
9486	234728	Cmtr2	55783	0.408400285	0.14688
9487	66259	Camk2n1	55450	0.255323934	0.15672
9488	69183	C1qtnf2	114898	1.238755759	0.09527
9489	12856	Cox17		0.758793962	0.00926
9490	229593	Golph3l	55204	-0.244688336	0.75682
9491	11835	Ar	367	0.096831844	0.69294
9492	224093	Fam43a	131583	0.029310947	0.99988
9493	278240	Spin2c			0.991
9494	79233	Zfp319	57567	-1.221509397	0.29072
9495	106840	Unc119b	84747	-0.016969664	0.99988
9496	68281	930430F08Ri	91298	0.829347429	0.1879
9497	380840	Lyrm4	57128	0.564925285	0.48431

9498	233210	Prr12	57479	-0.366276302	0.72791
9499	72267	Lrrc8e	80131	0.812146308	0.47631
9500	71911	Bdh1	622	-0.437465106	0.96413
9501	382117	Tcaim	285343	0.502662934	0.03346
9502	210004	B3gntl1	146712	-0.023094264	0.99988
9503	241327	Olfml2a	169611	-0.40383702	0.7797
9504	14621	Gjb4	127534	0.935039404	0.02215
9505	54201	Zfp316	100131017	-0.390740822	0.55673
9506	67393	Cxxc5	51523	-0.266231278	0.991
9507	76824	Mtfr1l	56181	-0.171183365	0.84609
9508	320351	Tmem251	26175	-0.078099673	0.99988
9509	232196	C87436	54980	-0.838754201	0.38967
9510	211550	Tifa	92610	-0.542761646	0.61803
9511	214987	Chtf8	54921	-0.450444691	0.40878
9512	100342	Tent5b	115572	-0.078540662	0.99988
9513	13000	Csnk2a2	1459	-0.125456486	0.99988
9514	15361	Hmga1	3159	0.145551183	0.99988
9515	69550	Bst2	684	-0.331569217	0.59996
9516	57912	Cdc42se1	56882	-0.158849927	0.69183
9517	66060	Cystm1	84418	-0.547535044	0.40878
9518	216858	Kctd11	147040	0.657674466	0.27188
9519	232431	Gprc5a	9052	-1.065494605	0.11152
9520	320234	Ccdc66	285331	-0.285621689	0.59867
9521	50529	Mrps7	51081	-0.161044437	0.991
9522	105732	Fam83h	286077	-0.883659872	0.29356
9523	80837	Rhoj	57381	0.188542815	0.99988
9524	70846	Ttc6	319089	-2.399172371	0.07841
9525	77781	Epm2aip1	9852	0.526224628	0.68121
9526	71952	Riox1	79697	0.000617329	0.99988
9527	67109	Zfp787	126208	-0.750891345	0.32204
9528	64945	Cldn12	9069	0.11328081	0.56649
9529	17476	Mpeg1		1.837457736	0.00888
9530	78412	Cyren	78996	-0.089563417	0.91648
9531	192734	Lrrc75b	388886	-0.673101066	0.43505
9532	231287	Atp10d	57205	0.565099659	0.6985
9533	320415	Gchfr	2644	-0.75246624	0.74422
9534	73284	Ddit4l	115265	-0.136439565	0.8918
9535	106947	Slc39a3	29985	-0.268691745	0.90576
9536	76415	Fam187b	148109	-0.868512054	0.26123
9537	71678	Brox	148362	0.225409045	0.77629
9538	216197	Ckap4	10970	-1.035744018	0.14129
9539	76608	Hectd3	79654	-0.136958858	0.99988
9540	242736	Pramel12		0.323274174	0.45517
9541	14113	Fbl	2091	-0.638808953	0.62437
9542	270669	Mbtps2	51360	0.161567608	0.87037
9543	20238	Atxn1	6310	-0.24442063	0.68169
9544	15944	Irgm1		-0.813656317	0.40986
9545	68744	Zfp740	283337	0.358127704	0.38663
9546	16995	Ltb4r1		1.587977924	0.00544
9547	68550	Tefm	79736	0.086535478	0.99988

9548	68632	Myct1	80177	0.141018197	0.99988
9549	57869	Adck2		0.965005558	0.52957
9550	18105	Nqo2	4835	-0.091978959	0.95917
9551	114565	Zbtb21	49854	0.504368294	0.3261
9552	271844	Pla2g4f	255189	-1.336323619	0.03842
9553	110796	Tshz1	10194	0.120841827	0.99988
9554	231225	Tapt1	202018	0.196941807	0.83565
9555	212679	Mars2	92935	0.141472116	0.99988
9556	68725	110032F04Ri	401097	-0.693265296	0.23202
9557	22701	Zfp41		1.160309753	0.01677
9558	73490	Mipol1	145282	-0.232941958	0.93597
9559	263876	Spata2	9825	0.625764451	0.19397
9560	235682	Zfp445	353274	0.152485841	0.51687
9561	233280	Nipa1	123606	1.606550263	0.01364
9562	217138	Prr15l	79170	-0.431799793	0.43702
9563	225995	)030056L22R	55071	1.616143936	0.00418
9564	209497	Tmem164	84187	0.29396756	0.78277
9565	67446	Dusp28	285193	-0.011860445	0.99988
9566	83485	Ngrn	51335	0.266962144	0.0681
9567	272381	Lrrc4b	94030	-0.690505116	0.57114
9568	73827	Tmem198b		1.135457992	0.17695
9569	268749	Rnf31		0.160972339	0.99988
9570	231946	Fam221a	340277	-0.104652572	0.96453
9571	106759	Ticam1	148022	-0.572725299	0.45677
9572	67300	Cltc	1213	1.337384915	0.00653
9573	12484	Cd24a	100133941	-0.442293223	0.44207
9574	72020	Zfp654	55279	0.017503604	0.99988
9575	242620	Dmrta2	63950	0.000899198	0.99988
9576	219094	Khnyn	23351	0.84965075	0.06089
9577	214854	Neurl3	93082	0.462319971	0.06411
9578	217125	Samd14	201191	-0.320755587	0.74422
9579	59021	Rab2a	5862	0.089358333	0.92973
9580	110350	Dync2h1	79659	-0.415974987	0.65774
9581	75219	Dusp18	150290	-0.669464839	0.43622
9582	229096	Ythdf3	253943	0.400287796	0.69812
9583	20005	Rpl9		0.209437892	0.99988
9584	434438	Iho1	339834	-0.47113299	0.45862
9585	330050	Fam185a	222234	0.441669034	0.95556
9586	75625	Mageh1	28986	1.474583928	0.0079
9587	407786	Taf9b	51616	-0.177495831	0.99988
9588	319179	H2bc6	8339	-0.315733847	0.8023
9589	277939	C2cd3	26005	0.960123389	0.03844
9590	19224	Ptgs1	5742	1.510121718	0.00544
9591	66048	Emc6	83460	-0.251876272	0.55356
9592	140482	Zfp358	140467	0.651183806	0.02199
9593	55948	Sfn	2810	-0.062432074	0.87354
9594	216860	Neurl4	84461	-0.235851594	0.94112
9595	57814	Kcne4	23704	0.788848588	0.0617
9596	236285	Lancl3	347404	0.92287354	0.01376
9597	226016	Abhd17b	51104	-0.623289164	0.3372

9598	240960	Dnah14	127602	-0.019979113	0.99988
9599	233890	Zfp768	79724	0.330367375	0.80305
9600	108902	B4gat1		-0.32077159	0.96415
9601	234776	Atmin	23300	-0.27783737	0.71204
9602	21815	Tgif1	7050	-0.649594541	0.26685
9603	69274	Ctdspl	10217	-0.319556438	0.95012
9604	235132	Zbtb44	29068	0.613852066	0.02504
9605	399558	Flrt2	23768	0.877720816	0.01491
9606	238377	Gpr68	8111	-1.305909935	0.04794
9607	66932	Rexo1	57455	0.062318481	0.99988
9608	208164	Fam180a	389558	-0.143218448	0.99779
9609	107242	AI837181	83638	-0.295161939	0.59106
9610	268880	Xxylt1	152002	1.156440294	0.00583
9611	11861	Arl4a	10124	-0.102316207	0.99988
9612	268566	Gphn	10243	-0.841413058	0.25243
9613	67068	Dynlrb1	83658	-1.037943918	0.40878
9614	212163	030462N17R	147339	-1.37976526	0.18144
9615	22693	Zfp30	22835	0.557862364	0.38712
9616	320311	Rnf152	220441	0.748269745	0.01627
9617	12740	Cldn4		-1.381490623	0.1126
9618	22110	Tspyl1	7259	0.196739291	0.71645
9619	12945	Dmbt1	1755	-1.321985792	0.63198
9620	217653	Mis18bp1	55320	0.249572771	0.991
9621	67948	Fbxo28	23219	-0.024442332	0.99988
9622	74325	Cltb	1212	0.148473449	0.99988
9623	233724	Tmem41b	440026	0.534740493	0.09138
9624	17035	Lxn	56925	1.58602942	0.00649
9625	209584	Tyw3	127253	0.527823573	0.89985
9626	381680	Nxpe5		1.729309064	0.00576
9627	56525	Zfp235		-0.0673621	0.98746
9628	97159	430005L14R	339448	0.186548275	0.09242
9629	227622	Paxx	286257	-0.629069933	0.53693
9630	103161	Apof		-1.822249773	0.14124
9631	72650	810006K23R	91574	-1.806797983	0.04036
9632	71865	Fbxo30	84085	0.907640502	0.13204
9633	70333	Cd3eap	10849	-0.58324897	0.44852
9634	73390	Msl3l2		0.495667365	0.96994
9635	20116	Rps8		-0.097275694	0.9712
9636	77929	Yipf6	286451	-0.197368651	0.88911
9637	101994	Champ1	283489	-0.351039969	0.6077
9638	66849	Ppp1r2		0.058365581	0.99988
9639	71707	Ubiad1	29914	0.346449425	0.11994
9640	66162	Bola2		0.242797043	0.98562
9641	215384	Fcgbp		2.758225901	0.00356
9642	226178	Wbp1l	54838	0.108985228	0.25705
9643	209086	Samd9l	219285	0.755084172	0.02162
9644	330812	Rnf150	57484	1.077513537	0.01649
9645	209032	Zc3hav1l	92092	0.617642633	0.05861
9646	237211	Fancb	2187	0.821729933	0.18609
9647	102747	Lrrc49	54839	1.430131581	0.11793

9648	73683	Atg16l2	89849	0.555979862	0.07918
9649	230936	Phf13	148479	-0.242422673	0.96967
9650	66643	Lix1	167410	0.12422676	0.99988
9651	268706	Slc38a9	153129	-0.147239583	0.85955
9652	208777	Sned1	25992	-0.891442393	0.38074
9653	14618	Gjb1	2705	-0.152484973	0.91811
9654	246746	Cd300lf		0.402157913	0.94808
9655	56697	Akap10	11216	0.13295239	0.72791
9656	78317	Ccdc88b	283234	0.09973759	0.84891
9657	94092	Trim16	10626	-0.624718975	0.42043
9658	68911	Pygo2	90780	0.502109945	0.68927
9659	71963	Cdca4	55038	0.21694986	0.99988
9660	55950	Bri3	25798	0.333777185	0.74763
9661	68159	Stx19	415117	-0.277552392	0.76003
9662	66887	Lonp2	83752	0.643208293	0.02461
9663	231931	Gimap6	474344	1.531042323	0.00837
9664	269604	Gpr157	80045	-1.610987092	0.03668
9665	59025	Usp14	9097	-0.277522655	0.61115
9666	100532	Rell1	768211	-0.798669161	0.28603
9667	213988	Tnrc6b	23112	-0.527436675	0.44933
9668	228911	Tshz2	128553	0.694722017	0.07297
9669	320816	Ankrd16	54522	-0.130574169	0.99988
9670	93887	Pcdhb16	56123	0.132326466	0.74229
9671	76510	Trappc9	83696	-0.277886529	0.57301
9672	17357	Marcksl1	65108	-0.411553609	0.58979
9673	52331	Stbd1	8987	1.785988761	0.01198
9674	74337	Palm3	342979	-0.287635047	0.9526
9675	225280	Ino80c	125476	0.021651204	0.99988
9676	546336	Prrg1	5638	0.565632239	0.30282
9677	227331	Gigyf2	26058	-1.741367295	0.13729
9678	30058	Timm8a1	1678	-0.102555018	0.89651
9679	68799	Rgmb	285704	0.682497521	0.81485
9680	229504	Isg20l2	81875	-0.228890797	0.88411
9681	76976	Arxes2		2.031809559	0.00768
9682	241576	Ldlrad3	143458	-0.917740902	0.29398
9683	11840	Arf1	375	0.175006024	0.77929
9684	23966	Tenm4	26011	-0.236815589	0.74277
9685	11615	Gm4737		1.250485167	0.0461
9686	93689	Lmod1	25802	1.70623446	0.00837
9687	99730	Taf13	6884	0.721258082	0.0222
9688		4632415L05Rik		-1.129201091	0.30907
9689	229700	Rbm15	64783	0.463887043	0.34256
9690	238247	Arid4a	5926	-0.354862753	0.42133
9691	12495	Entpd1	953	0.746475852	0.66478
9692	12835	Col6a3	1293	0.769892401	0.42635
9693	381022	Kmt2d		0.009464099	0.99988
9694	20345	Selplg	6404	2.055151854	0.00544
9695	210711	Mcmbp	79892	0.28745332	0.31554
9696	78541	Asb8	140461	0.184306477	0.99988
9697	209645	Bend7	222389	0.688276254	0.15379

9698	213573	Cracr2b	283229	0.298772519	0.99988
9699	64450	Gpr85	54329	0.807633748	0.02527
9700	105827	Amigo2	347902	0.174739176	0.77001
9701	100034361	Mfap1b		-0.482925639	0.51701
9702	269529	Fbxo10	26267	0.343771204	0.33554
9703	67702	Rnf149	284996	-1.446579126	0.12819
9704	77128	Crebrf	153222	0.052729707	0.95664
9705	58208	Bcl11b	64919	0.60377538	0.34226
9706	208440	Dip2c	22982	0.884369782	0.23011
9707	381626	Rbm33	155435	-0.140724102	0.75181
9708	20973	Syngr2	9144	-0.352468673	0.62133
9709	408068	Zfp738		0.44314872	0.04608
9710	319710	Frmd6	122786	0.095395866	0.99988
9711	68839	Ankrd46	157567	0.344964972	0.0722
9712	70466	Ckap2l	150468	-0.695873101	0.42635
9713	215723	Mfsd6l	162387	-2.126227626	0.03816
9714	108927	Lhfp	10186	0.708426397	0.2697
9715	69893	Coa7	65260	-0.368587714	0.66379
9716	27047	Omd	4958	-0.774393455	0.02348
9717	382051	Pdp2	57546	-0.133326777	0.99528
9718	14181	Fgfbp1	9982	-1.687129915	0.01931
9719	14062	F2r	2149	0.541839652	0.56404
9720	67296	Socs4	122809	-0.009910757	0.98477
9721	23967	Osr1	130497	-0.538637935	0.93937
9722	241520	Fam171b	165215	1.186818813	0.0146
9723	14633	Gli2	2736	-0.350949726	0.9519
9724		B330016D10Rik		0.67787655	0.03459
9725	240476	Zfp407	55628	0.268305667	0.98921
9726	69773	Timm29	90580	-0.658025758	0.46294
9727	231042	Nupl2	11097	0.353055638	0.80304
9728	70101	Cyp4f16		0.496176064	0.58242
9729	17701	Msx1	4487	1.575654017	0.0098
9730	109050	Inka2	55924	0.433884129	0.5642
9731	320869	Spata33		-0.407335806	0.91774
9732	232934	Mypop	339344	1.912908676	0.00493
9733	215627	Zbtb8b		0.867310171	0.44
9734	228859	Fitm2	128486	0.011386651	0.99988
9735	213393	Depp1	11067	-0.027578589	0.99988
9736	268903	Nrip1	8204	-1.147364203	0.18132
9737	68736	Tyw5	129450	-0.059076499	0.99988
9738	216829	Mmgt2		-0.367868748	0.66292
9739	80901	Cxcr6	10663	2.207979072	0.00388
9740	270152	Jaml		0.973829883	0.00959
9741	102693	Phldb1	23187	-0.256500599	0.99988
9742	57259	Tob2	10766	-0.091441208	0.93023
9743	208967	Thnsl1	79896	1.533400536	0.00554
9744	320145	Sp8	221833	0.26603314	0.42635
9745	226040	Tmem252	169693	1.730722186	0.01051
9746	109154	Mlec	9761	-0.114220858	0.99988
9747	329659	130311K13R	285315	-0.027766974	0.99988

9748	105246056	Gm9828		-0.630143366	0.2028
9749	226101	Myof	26509	0.548116635	0.04245
9750	330695	Ctxn1	404217	-1.05357747	0.10723
9751	241624	Exd1	161829	-0.492672509	0.84401
9752	380664	Lemd3	23592	-0.128437093	0.99988
9753	72440	Rhno1	83695	0.192688138	0.9519
9754	237400	Mex3d	399664	0.350969913	0.12711
9755	76551	Ccdc6	8030	0.541577673	0.05759
9756	52829	Lurap1l	286343	0.261483353	0.98387
9757	97031	Tprn	286262	1.210526383	0.03132
9758	209478	Tbc1d12	23232	-0.433489346	0.72025
9759	70772	Ggnbp1		0.504103619	0.94878
9760	223722	Mcat	27349	-0.323884487	0.52745
9761	56484	Foxo3	2309	0.222912127	0.70567
9762	19944	Rpl29		0.075449628	0.88714
9763	15410	Hoxb3	3213	0.567485033	0.38014
9764	68777	Tmem53	79639	0.016984683	0.99988
9765	19227	Pthlh	5744		0.40878
9766	233571	P2ry6	5031	1.477461913	0.00583
9767	233805	Dcun1d3	123879	0.119177526	0.99988
9768	243538	Cfap100	348807		0.991
9769	72023	Cyb561d1	284613	0.060833383	0.99224
9770	225523	Cep120	153241	0.220544673	0.89195
9771	103710	Slc35e4	339665	-0.162238202	0.83265
9772	107305	Vps37c	55048	0.247276497	0.99779
9773	328133	Slc39a9	55334	-0.011078947	0.99988
9774	104910	Slc25a47	283600	-0.66514664	0.43009
9775	226652	Arhgap30	257106	1.509164877	0.00576
9776	213109	Phf3	23469	-0.117828543	0.65428
9777	192231	Hexim1	10614	0.738240072	0.00972
9778	209225	Zfp710		-0.27525751	0.858
9779	51902	Rnf24	11237	0.854272836	0.01409
9780	13640	Efna5	1946	-1.387379983	0.23101
9781	243853	Fkrp	79147	-0.110076251	0.99988
9782	71131	Zfp689	115509	-0.22529173	0.89364
9783	76041	Ccdc125	202243	-0.977500461	0.21167
9784	101206	Tada3	10474	0.283422273	0.79144
9785	109294	Prex2	80243	-0.655873385	0.32201
9786	244238	Mrgpre	116534	-1.186433323	0.16839
9787	59048	C1galt1c1		-0.190486238	0.99779
9788	243312	Elfn1	392617	0.088659466	0.99988
9789	69814	Prss32		2.796399793	0.00388
9790	269799	Clec4a1	50856	1.935377767	0.00388
9791	74238	Mterf2	80298	1.528720873	0.00675
9792	71703	Armcx3	51566	-0.608652262	0.37878
9793	78618	Acap2	23527	0.007195287	0.99989
9794	107771	Bmyc		-0.39472484	0.49368
9795	225791	Zadh2	284273	0.126954246	0.99988
9796	20768	Sephs2	22928	0.195980896	0.99988
9797	12772	Ccr2	729230	1.838745759	0.00675

9798	320808	Dcaf5	8816	-0.475944853	0.40878
9799	11607	Agtr1a		-0.936690527	0.55816
9800	242297	Fam110b	90362	0.101807195	0.99988
9801	12273	C5ar1	728	2.119018359	0.00388
9802	72672	Zfp518a	9849	-0.036721491	0.99988
9803	331474	Rtl5	340526	1.09433233	0.01105
9804	381511	Pdp1	54704	-0.294884735	0.99988
9805	68140	Tigd2	166815	-0.626116892	0.34465
9806		Gm7324		-1.083703233	0.11618
9807	330216	Mblac1	255374	1.125266272	0.00975
9808	216792	Iba57	200205	-0.127411953	0.99988
9809	280411	Lix1l	128077	0.520183706	0.5255
9810	69890	Zfp219	51222	-0.690206834	0.37705
9811	245828	Trappc1	58485	0.004343758	0.99988
9812	99890	Prmt6	55170	0.683171853	0.03987
9813	72454	Ccdc71	64925	-1.416754785	0.31188
9814	14345	Fut4	2526	-0.157406504	0.87985
9815	20660	Sorl1	6653	-0.675436045	0.23939
9816	22678	Zfp2	80108	0.475256182	0.84786
9817	237782	Smcr8	140775	-0.879183652	0.48488
9818	67956	Kmt5a	387893	-0.197456807	0.80354
9819	227298	Retreg2	79137	-0.177618744	0.99988
9820	71833	Dcaf7	10238	0.611006863	0.29859
9821	16691	Krt8	3856	-0.69272592	0.26075
9822	276919	Gemin4	50628	0.469678974	0.28284
9823	72075	Ogfr	11054	-0.406773978	0.53734
9824	58182	Prokr1		-1.254384186	0.3193
9825	338363	Tmem241	85019	0.349834958	0.99988
9826	26466	Zfp260	339324	-0.026033127	0.9188
9827	103172	Chchd10	400916	-0.204723097	0.88372
9828	22268	Upk1b	7348	1.874471175	0.0066
9829	77951	Cyp20a1	57404	0.607621143	0.22624
9830	93736	Aff4	27125	0.173926878	0.52442
9831	66965	Ctu2	348180	-0.156079937	0.70675
9832	329795	Tmem67	91147	-0.023910046	0.99988
9833	69109	Ccnq	92002	0.984274917	0.01234
9834	102502	Pls1	5357	-0.414603743	0.48743
9835	209200	Dtx3l	151636	-0.957167832	0.37561
9836	212127	Proser1	80209	-0.948932871	0.44448
9837	101685	Spty2d1	144108	0.334872353	0.05186
9838	66475	Rps23	6228	0.681643206	0.02298
9839	104445	Cdc42ep1	11135	-0.501238947	0.47463
9840	50524	Sall2	6297	0.05742776	0.97513
9841	237052	Tceal1	9338	-0.498238309	0.80271
9842	56430	Clip1	6249	-0.304719458	0.61004
9843	20019	Polr1a	25885	0.159492089	0.99988
9844	20776	Tmie	259236	0.325388338	0.6985
9845	381201	Ap5b1	91056	0.689752815	0.49268
9846	22761	Zfpm1	161882	-1.129093275	0.2443
9847	244152	Tsku	25987	0.945126563	0.01119

9848	52570	Ccdc69	26112	0.77569787	0.01882
9849	232879	Zbtb45	84878	-0.363430601	0.7068
9850	15408	Hoxb13	10481	-1.220820053	0.23425
9851	52397	Zfp644	84146	-0.46118344	0.42942
9852	227326	Gpr55	9290		0.40878
9853	235504	Slc17a5	26503	-0.060480016	0.93354
9854	212937	Tifab	497189	1.006195834	0.19626
9855	66367	310022A10Ri	126526	-0.625139416	0.67269
9856	230119	Zbtb5	9925	0.759041164	0.07417
9857	544971	Bdp1	55814	-0.679802596	0.22688
9858	216549	Aftph	54812	0.077977892	0.96267
9859	22666	Zbtb14	7541	-0.113213582	0.99988
9860	72046	Urgcp	55665	0.649222745	0.01459
9861	109305	Orai1	84876	1.06012594	0.01184
9862	338368	Pheta2	150368	-0.0531501	0.991
9863	244202	Nlrp10	338322	-0.908399091	0.3837
9864	319583	Lig4	3981	-0.652387919	0.58041
9865	17381	Mmp12	4321	0.427543683	0.72294
9866	244219	Zfp668	79759	-0.577201062	0.44351
9867	22040	Trex1	11277	-0.412605778	0.56254
9868	233905	Zfp646	9726	0.75354455	0.73522
9869	76117	Arhgap15	55843	1.487331086	0.00797
9870	66483	Rpl36al		-0.083557068	0.83209
9871	319475	Zfp672	79894	0.74397245	0.21432
9872	224904	Micos13	125988	0.154813174	0.99988
9873	19241	Tmsb4x	9087	1.07482231	0.0079
9874	14366	Fzd4	8322	-0.337639481	0.60349
9875	70369	Bag5	9529	1.002592389	0.0066
9876	58172	Sertad2	9792	0.59564979	0.01992
9877	100503043	Armcx4	100131755	0.176979139	0.99988
9878	58996	Arhgap23	57636	-1.744168379	0.09371
9879	73873	Fam161a	84140	-0.052513614	0.99988
9880	193736	Zbtb12	221527	-0.399523846	0.66901
9881	211389	Suox		0.550792613	0.06656
9882	320982	Arl4c	10123	1.406124962	0.00438
9883	268857	Nlrc3	197358	-0.819787359	0.40461
9884	103511	Calhm5	254228	0.712992027	0.47867
9885	109263	Rlf	6018	-0.724673343	0.38093
9886	69953	810025M15Rik		0.362702332	0.05567
9887	19416	Rasd1	51655	0.807981742	0.01172
9888	103765	Tmem17	200728	0.732868504	0.22688
9889	68939	Rasl11b	65997	-0.67218444	0.57906
9890	228368	Slc35c1	55343	-0.532366711	0.41271
9891	78134	Lpar4	2846	0.120049773	0.99988
9892	15270	H2ax	3014	-1.152760232	0.19711
9893	192198	Lrrc4	64101	0.705137774	0.34241
9894	70804	Pgrmc2	10424	-0.472168607	0.43979
9895	227522	Rpp38	10557	1.068688976	0.08749
9896	67291	Ccdc137	339230	-1.685230975	0.07971
9897	66242	Mrps16	51021	-0.014675305	0.99988

9898	71801	Plekhf2	79666	0.290994597	0.05869
9899	319804	Glt1d1	144423	-0.110925119	0.85752
9900	228966	Ppp1r3d	5509	-0.121670002	0.99656
9901	75731	Idnk	414328	-0.298975502	0.58127
9902	56305	Pitpnb	23760	0.813771235	0.3036
9903	231842	Amz1	155185	0.970044264	0.01488
9904	72065	Rap2c	57826	0.906859991	0.16219
9905	66958	Tmx2		-0.092203779	0.90546
9906	72148	Tdrp	157695	0.185711964	0.7901
9907	242109	Zfp697	90874	0.214471389	0.88714
9908	19716	Bex1		-0.019145892	0.99988
9909	229323	Gpr171	29909	1.913046022	0.00566
9910	67610	Rspry1	89970	-0.226827621	0.53734
9911	67912	600012H06R	387263	-0.016261005	0.99988
9912	319660	Agmo	392636	0.309797157	0.55816
9913	229517	Slc25a44	9673	-0.454578947	0.3925
9914	54609	Ubqln2	29978	-0.058144401	0.99988
9915	116748	Lsm10	84967	0.550143479	0.07326
9916	208691	Eif5a2	56648	0.059730697	0.95306
9917	107515	Lgr4	55366	0.589140869	0.28532
9918	230752	Eva1b	55194	0.458697346	0.85388
9919	76793	Snip1	79753	0.385559644	0.97377
9920	239114	Il17d	53342	-0.691463334	0.44272
9921	67556	Pigm	93183	1.27352572	0.00837
9922	14612	Gja4	2701	-2.014221791	0.0832
9923	58180	Hic2	23119	0.849695147	0.05715
9924	243655	Klre1		-0.407656702	0.66845
9925	217995	Heatr1	55127	0.358980772	0.50619
9926	68525	Evc2	132884	-1.169279634	0.23676
9927	338369	Tmem220	388335	0.704233996	0.2533
9928	244418	Prag1	157285	0.369097758	0.69993
9929	57265	Fzd2	2535	0.856167671	0.47329
9930	17300	Foxc1	2296	-0.025247427	0.99741
9931		Gm9843		-1.397883323	0.0823
9932	78757	Rictor	253260	-0.485251364	0.55816
9933	106338	Nsun3	63899	0.82197064	0.02646
9934	118449	Synpo2	171024	1.200167381	0.02009
9935	76947	Ndufaf6	137682	-0.661599317	0.67946
9936	72345	Amer1	139285	-0.554250517	0.44695
9937	16854	Lgals3	3958	1.058322835	0.01143
9938	214639	930486L24Rik		-0.04223009	0.99988
9939	20753	Sprr1a		-2.788685123	0.03974
9940	15430	Hoxd10	3236	1.452435719	0.00768
9941	12642	Ch25h	9023	1.460071168	0.01064
9942	101113	Snx21	90203	-0.092394537	0.99988
9943	56526	Septin6	23157	-0.675007543	0.39079
9944	97130	C77080	57648	-0.319598426	0.92691
9945	106795	Tcf19	6941	-2.241852287	0.08304
9946	243867	Fbxo46	23403	-1.21721578	0.34186
9947	332131	Krt78	196374	1.930225838	0.05186

9948	109229	Fam118b	79607	-0.470789395	0.43545
9949	74165	Fbxl22	283807	1.101455506	0.13762
9950	54420	Cldn8	9073	0.343229373	0.15001
9951	269233	Fam171a1	221061	0.680695543	0.03039
9952	11548	Adra1b	147	0.632542759	0.029
9953	70617	Fam241a	132720	0.254306304	0.96368
9954	66096	Lamtor4	389541	0.581542052	0.23011
9955	240832	Tor1aip2	163590	-0.064541462	0.91006
9956	103806	Maml1	9794	0.944768354	0.34158
9957	100043757	Zfp831	128611		0.40878
9958	22719	Zfp61	7769	-0.222384608	0.58618
9959	433771	Micos10		0.090159623	0.85634
9960	99334	Zscan29	146050	0.100566197	0.99988
9961	100043813	Rps27rt	6232	-0.115298635	0.84839
9962	333433	Gpd1l	23171	0.247000962	0.40878
9963	319370	Ubald2	283991	-0.294077716	0.71895
9964	231503	Tmem150c	441027	-0.578656129	0.5476
9965	320736	Vstm4	196740	0.530491014	0.91406
9966	53951	Gpatch11	253635	0.068754342	0.99988
9967	105787	Prkaa1	5562	0.53143519	0.30594
9968	69662	2310061I04Ri	221545	0.79856419	0.19646
9969	14325	Ftl1		-0.141925746	0.99988
9970	320633	Zbtb26	57684	0.913196342	0.35174
9971	102595	Plekho2		0.72726903	0.01806
9972	71544	Arhgap42	143872	0.228334759	0.97147
9973	22320	Vamp8	8673	-0.641595426	0.28032
9974	64292	Ptges	9536	0.089439232	0.991
9975	213171	Prss27	83886	0.81778449	0.66845
9976	170706	Tmem37	140738	1.840375473	0.00621
9977	57895	Ccdc126	90693	1.546047978	0.12336
9978	117592	B3galt6	126792	-1.156218882	0.33655
9979	230249	Ecpas	23392	-0.793698146	0.31538
9980	78408	Fam131a	131408	0.23916238	0.98861
9981	319922	Vwc2	375567		0.40878
9982	78834	Zfp623	9831	1.839891851	0.00566
9983	230678	Tmem125	128218	-1.133502277	0.21537
9984	11958	Atp5k	521	0.093536944	0.99988
9985	230809	Pdik1l	149420	-1.028547644	0.18662
9986	69694	Tatdn1	83940	0.437052531	0.10704
9987	237988	Cdr2l	30850	0.181434231	0.97346
9988	71929	Tmem123	114908	0.51535248	0.02521
9989	654824	Ankrd37	353322	-0.565156204	0.40612
9990	78826	P2ry10	27334	1.69429742	0.00544
9991	74393	Map10	54627	1.039606626	0.21382
9992	74442	Sgms2	166929	-0.158287534	0.88929
9993	229715	Amigo1	57463	-0.066653318	0.99053
9994	14609	Gja1		0.23614059	0.82473
9995	27356	Insl6	11172	0.280429221	0.99988
9996	18750	Prkca	5578	-1.1303875	0.15847
9997	74777	Selenon	57190	-0.095476399	0.99988

9998	229488	Fam160a1	729830	-0.24895762	0.57884
9999	213350	Gatd1	347862	-0.35610421	0.59203
10000	15476	Hs3st1	9957	0.468989005	0.62984
10001	218311	Zfp455		1.047712897	0.00933
10002	244198	Olfml1	283298	0.477480866	0.99988
10003	70355	Gprc5c	55890	-0.43960049	0.87991
10004	239796	Mb21d2	151963	0.472496269	0.52634
10005	242122	Vtcn1	79679	-0.153004349	0.82926
10006	72852	Mblac2	153364	0.514034644	0.79121
10007	75538	Fam71e1	112703		0.40878
10008	258336	Olfr77		0.032351066	0.99267
10009	317758	Gimap9		1.487296799	0.22364
10010	17961	Nat2		0.931595908	0.01207
10011	11538	Adnp	23394	3.999544204	0.01153
10012	12238	Commd3	23412	0.236270224	0.32201
10013	12705	Cited1	4435	-5.551852184	0.01281
10014	319670	Eml5	161436	-0.502772601	0.50077
10015	101122	Rpusd3	285367	-0.164379829	0.92705
10016	18795	Plcb1	23236	1.554516606	0.00819
10017	66056	Zfp524		0.120942914	0.99988
10018	67698	Fam174a	345757	0.930500615	0.03276
10019	321019	Gpr183	1880	1.716539858	0.00388
10020	236930	Ercc6l	54821	-1.11942985	0.13817
10021	66882	Bzw1	9689	-0.152624688	0.85142
10022	245695	Tceanc	170082	-0.315443312	0.84859
10023	195040	Tmem199	147007	-0.661184261	0.39848
10024	19823	Rnf7		0.692312804	0.01931
10025	209334	Gen1	348654	0.013478218	0.8833
10026	320183	Msrb3	253827	0.696023506	0.26686
10027	66962	Swsap1	126074	-1.015158949	0.30547
10028	93880	Pcdhb9		-0.092009812	0.9115
10029	320563	Islr2	57611	-0.548703599	0.81206
10030		Gm6563		-0.766539277	0.4933
10031	67767	Jagn1	84522	0.865787802	0.18424
10032	71643	Zgrf1		0.381990866	0.60368
10033	319263	Pcmtd1	115294	-0.040803788	0.97242
10034	76800	Usp42	84132	-0.068890139	0.94301
10035	233079	Ffar2	2867	0.796204557	0.56329
10036	24074	Taf7	6879	-1.127132483	0.31867
10037	67885	Mtln	205251	0.600006559	0.02148
10038	279653	Pcdh19	57526	0.738518753	0.82658
10039	59015	Nup160	23279	0.038905231	0.99988
10040	12288	Cacna1c	775	-0.717605906	0.47207
10041	328232	Gfod1	54438	-0.553618486	0.37471
10042	243219	900026A02R	85379	-0.654014839	0.35611
10043	22710	Zfp52		-0.477454123	0.96441
10044	52055	Rab11fip5	26056	-0.036493349	0.99988
10045	241075	Plekhm3	389072	-0.340807287	0.49462
10046	66701	Spryd4		0.980569639	0.01357
10047	22704	Zfp46	80818	0.579802766	0.02527

10048	17846	Commd1		0.74579398	0.15654
10049	52589	Ncald	83988	-0.317554999	0.95272
10050	20471	Six1	6495	-0.686055604	0.34451
10051	75599	Pcdh1	5097	-0.679069579	0.48492
10052	71436	Flrt3	23767	0.029310947	0.99988
10053	81630	Zbtb22	9278	0.226718751	0.83384
10054	22628	Ywhag	7532	-0.34593622	0.44695
10055	56753	Tacstd2	4070	0.498941033	0.031
10056	54711	Plagl2	5326	-1.025546425	0.3364
10057	216516	Ccdc157	550631	-1.531058318	0.19299
10058	329977	Fhad1	114827	-0.780539095	0.25731
10059	12475	Cd14	929	0.259568799	0.67178
10060	241950	Bbs12	166379	-0.282164961	0.99988
10061	233490	Crebzf	58487	0.413259112	0.031
10062	20737	Spn	6693	1.236038237	0.00888
10063	59057	Zfp24	7572	-0.327573035	0.48321
10064	12408	Cbr1		0.314398785	0.61205
10065	270110	Irf2bp2	359948	0.362948663	0.93597
10066	21899	Tlr6	10333	-0.120165777	0.99723
10067	70240	Ufsp1	402682	-0.525796114	0.42122
10068	233274	Siglech		0.059547649	0.99988
10069	545030	Wdfy4	57705	2.307549077	0.00544
10070	17134	Mafg	4097	-0.0678854	0.99988
10071	230098	Arhgef39	84904	-0.054213386	0.99988
10072	66538	Rps19bp1	91582	-0.599306985	0.57377
10073	331392	Gm5124		0.18091545	0.99397
10074	68490	Zfp579	163033	0.321020119	0.99988
10075	66684	Tceal8	90843	-0.140827817	0.90216
10076	194401	Mical3	57553	-0.817684597	0.55816
10077	76108	Rap2a	5911	-0.215770482	0.96811
10078	50709	H1f4	3008	1.088314952	0.00797
10079	53625	B3gnt2	10678	-1.135481048	0.1068
10080	237387	Lrrc3	81543	0.783668218	0.29276
10081	67892	Coa6	388753	-0.472534507	0.72244
10082	100737	Dcun1d4	23142	-0.31780101	0.89596
10083	69807	Trim32	22954	0.629468952	0.22972
10084	23983	Pcbp1	5093	0.200862117	0.83484
10085	71599	Senp8	123228	-0.81343251	0.73081
10086	238037	Wdcp	80304	1.275665187	0.00858
10087	233529	Kctd14	65987	0.576976089	0.37265
10088	75422	Mettl5	29081	-0.568517541	0.89642
10089	320435	Rinl		1.492420363	0.00591
10090	22138	Ttn	7273	3.196295415	0.03088
10091	66107	Wfdc21			0.991
10092	22594	Xrcc1	7515	-0.025558301	0.991
10093	328580	Tubgcp6	85378	-0.405995967	0.51037
10094	216856	Nlgn2	57555	0.04884372	0.99916
10095	333182	Cox6b2	125965	1.052848749	0.01281
10096	20667	Sox12	6666	-0.369364882	0.68415
10097	11842	Arf3	377	-0.878569227	0.24275

10098	17294	Mest	4232	1.959381151	0.0044
10099	223754	Tbc1d22a	25771	0.17201614	0.96505
10100	271005	Klhdc1	122773	0.471626888	0.97377
10101	72572	Spats2	65244	-0.39730797	0.68726
10102	381694	B3glct	145173	0.070240089	0.99988
10103	239081	Tlr11			0.991
10104	213389	Prdm9	11105	1.346183643	0.08113
10105	234086	Erich1	157697	-0.397919979	0.57761
10106	68936	Smim11		0.341654198	0.40563
10107	240754	Lax1	54900	0.4717692	0.56261
10108		Gm9864		0.653888657	0.44695
10109	380608	Tagap1		0.092002472	0.98506
10110	109054	Pfdn4	5203	1.180942376	0.24222
10111	50794	Klf13	51621	-0.013642209	0.99988
10112	228913	Zfp217	7764	0.275110992	0.2064
10113	72823	Pard3b	117583	-0.609679088	0.56553
10114	76088	Dock8	81704	0.600749124	0.49296
10115	51791	Rgs14	10636		0.40878
10116	26384	Gnpda1	10007	1.65386768	0.00591
10117	68617	Mtcl1	23255	-1.594188323	0.09853
10118	242484	630039A03R	401546	-0.727363703	0.85801
10119	320046	730043M19Rik		1.12799242	0.17082
10120	11997	Akr1b7		-0.371633886	0.991
10121	329934	Foxo6	100132074	0.071475998	0.99988
10122	107976	Babam2	9577	-0.113541752	0.92311
10123	320484	Rasal3	64926	-0.280480362	0.99988
10124	232314	Ppp4r2		-0.447043042	0.40369
10125	67097	Rps10		0.291266446	0.40986
10126	50784	Plpp2	8612	-0.283056237	0.65346
10127	11480	Acvr2a	92	-0.037398623	0.99988
10128	104759	Pld4	122618	1.953027809	0.00621
10129	403187	Opa3	80207	-1.289861071	0.14962
10130	210762	Ppp1r36	145376	1.102383423	0.10039
10131	433424	Zeb2os		1.592135681	0.00653
10132	52521	Zfp622	90441	0.272487191	0.43426
10133	14289	Fpr2		-0.095194553	0.92584
10134	102633868	330438D12Rik		0.649647351	0.03731
10135	108143	Taf9		-0.540421605	0.66322
10136	243819	Ppp6r1	22870	-0.606266653	0.26422
10137	72729	Cdc42se2	56990	-0.682569914	0.2916
10138	78913	Ltn1	26046	0.146344474	0.99988
10139	74694	Tbc1d30		0.411976207	0.90216
10140	15129	Hbb-bs		3.336049021	0.00356
10141	30791	Slc39a1	27173	0.029109719	0.99988
10142	329154	Ankrd44	91526	0.390437857	0.04213
10143	13051	Cx3cr1	1524	1.101264393	0.1984
10144	76614	Immt	10989	-0.231822134	0.81309
10145		B630019K06Rik		0.693681086	0.37299
10146	380967	Tmem106c	79022	-0.554198201	0.80571
10147		Hoxd3os1		0.541142461	0.96733

10148	13384	Mpp3	4356	-1.66706031	0.18935
10149	224109	Nrros	375387	1.878014933	0.00544
10150	328370	Rft1		-0.789718966	0.40843
10151	22350	Ezr	7430	-1.032774592	0.21116
10152	227656	Rexo4	57109	-0.619572186	0.4432
10153	320226	Ccdc171	203238	-0.003425095	0.99988
10154	99681	Tchh	7062	-0.870804679	0.18669
10155	66520	610001J05Ri	401397	0.266800461	0.28465
10156	68944	Tmco1	54499	-0.366482611	0.61759
10157	12167	Bmpr1b	658	-1.754121444	0.10429
10158	234413	Zfp961		-0.137761976	0.86487
10159	11964	Atp6v1a	523	-0.072064783	0.99988
10160	620078	130026I21Rik		0.077266241	0.9519
10161	13837	Epha3	2042		0.991
10162	78286	Nav2	89797	-0.929425563	0.42914
10163	227699	Nup188	23511	0.173546898	0.91487
10164	18514	Pbx1	5087	-0.335584496	0.45637
10165	99470	Magi3	260425	-1.433284444	0.21432
10166	209239	Gan	8139	-0.834585349	0.28864
10167	66871	Cpne8	144402	-0.914418302	0.27799
10168		D930048N14Rik		-0.817962486	0.51243
10169	14957	H1f3	3007	-0.217551917	0.84628
10170	170833	Hook2	29911	-0.530744963	0.5101
10171	23859	Dlg2	1740	0.986302068	0.53617
10172	72661	Serp2	387923	0.702863065	0.40308
10173	11491	Adam17	6868	0.080317603	0.99988
10174	67441	Isoc2b		0.639156078	0.5476
10175	263406	Plekhg3	26030	1.716847726	0.36054
10176	211914	Asap2	8853	-0.206929605	0.991
10177	22644	Rnf103	7844	-0.073615848	0.99988
10178	76100	830454E08Rik		0.078470379	0.99988
10179		Gm9887		0.013478218	0.85047
10180	57745	Zfp112		-0.569371534	0.26858
10181	215693	Zmat1	84460	-0.427693385	0.41675
10182	215449	Rap1b	5908	0.975070957	0.01344
10183	16476	Jun	3725	0.53105443	0.43341
10184	226421	Rab7b	338382	0.725408105	0.04439
10185	70549	Tln2	83660	0.298914104	0.991
10186	233833	Tnrc6a	27327	-0.775741315	0.28659
10187	80748	BC004004	221477	-0.086910263	0.84392
10188	269023	Zfp608	57507	-1.35930758	0.35873
10189	17755	Map1b	4131	-0.748790422	0.41716
10190	16642	Klrc2		0.754637851	0.02563
10191	56451	Suclg1	8802	0.345061569	0.42628
10192	66875	Swt1	54823	-0.034030467	0.99988
10193	244183	Trim30b		-0.570379438	0.85956
10194	58887	Repin1	29803	0.013197873	0.99988
10195	224619	Traf7	84231	-1.433711451	0.12651
10196	319997	630001G21Rik		-0.061402924	0.95917
10197	232784	Zfp212	7988	0.054366185	0.96453

10198	246730	Oas1a		-0.230192199	0.97675
10199	72254	700030K09R	84167	0.117731225	0.62491
10200	103468	Nup107	57122	0.73762816	0.43377
10201	320817	Atad2b	54454	-0.3020802	0.62164
10202	58861	Cysltr1	10800	0.773570772	0.0262
10203		Gm9892		-0.164727614	0.74485
10204	56459	Sae1	10055	-0.093609435	0.89334
10205	16477	Junb	3726	0.687492458	0.03958
10206	52250	Reep1	65055	0.607075372	0.3273
10207	18751	Prkcb	5579	1.912544667	0.00544
10208	108159	Ubxn8	7993	0.001666003	0.99995
10209	16779	Lamb2	3913	0.875572102	0.00773
10210	13110	Cyp2j6		0.811825703	0.00934
10211	74026	Msl1	339287	0.373739327	0.53594
10212	66315	Senp7	57337	-0.32435289	0.43932
10213	19091	Prkg1	5592	0.487385363	0.4813
10214	442801	Arhgef15	22899	-0.542250781	0.09802
10215	69724	Rnaseh2a	10535	0.678071173	0.31306
10216	269037	Ctif	9811	0.50084497	0.64349
10217	76454	Fbxo31	79791	-0.575154965	0.40271
10218	226075	Glis3	169792	0.00706926	0.99988
10219	217340	Rnf157	114804	0.51775362	0.08525
10220	14451	Gas1	2619	0.271222765	0.99672
10221	66223	Mrpl35	51318	0.547249622	0.20205
10222	13107	Cyp2f2	1572	1.323010418	0.0089
10223	50995	Uba2	10054	0.371355514	0.48476
10224	15465	Hrh1	3269		0.40878
10225	231991	Creb5	9586	-0.688588788	0.58979
10226	57896	Krcc1	51315	-0.8752719	0.14435
10227	68318	Aph1c		1.923938511	0.05715
10228	12526	Cd8b1		0.548682493	0.56514
10229	67374	Jam2	58494	1.345236542	0.01111
10230	232413	Clec12a	160364	0.790647298	0.02459
10231	108899	700081O15R	65998	0.378514885	0.40878
10232	72635	Lins1	55180	-0.917289658	0.14124
10233	71667	Tmem248	55069	-0.323141376	0.68447
10234	353346	Gpr141	353345	-0.407656702	0.49999
10235	22601	Yap1	10413	-0.012061614	0.99988
10236	12702	Socs3	9021	0.831812963	0.22496
10237	66700	Chmp3	51652	-0.641390137	0.35174
10238	213211	Rnf26		-0.404968982	0.98457
10239	72195	Supt7l	9913	0.342786794	0.99988
10240	19094	Mapk11	5600	-1.604344675	0.17648
10241	19281	Ptprt	11122	-1.358311984	0.2103
10242	14159	Fes	2242	1.24105079	0.11954
10243	12051	Bcl3	602	0.712495788	0.29624
10244	56772	Mllt11	10962	0.20555587	0.99988
10245	56291	Styx		-0.098109933	0.93776
10246	23863	Dand5	199699	-0.900313836	0.55136
10247	76273	Ndfip2	54602	0.274037575	0.02886

10248	11668	Aldh1a1		-0.017510558	0.99988
10249	98685	Trmt1l	81627	-0.145622947	0.91487
10250	77591	Ddx10	1662	-0.994788211	0.28119
10251	19342	Rab4b		-0.018912479	0.99988
10252	107939	Pom121		0.216770177	0.99988
10253	66212	Sec61b		0.366879106	0.04403
10254	74748	Slamf8	56833	2.466072744	0.00388
10255	28295	Gatd3a		0.28449737	0.73729
10256	14455	Gas5		0.711203499	0.06275
10257	208718	Dis3l2	129563	-0.175969202	0.99988
10258	74670	Zfp943		-0.705250221	0.36495
10259	240067	Zfp952		0.463209747	0.43843
10260	236539	Phgdh	26227	-1.665838019	0.06822
10261	52609	Cbx7	23492	0.58052523	0.48253
10262	26416	Mapk14	1432	0.183956277	0.99988
10263	66231	Thoc7	80145	-0.22625781	0.97042
10264	56316	Ggcx	2677	-0.02943898	0.99672
10265	104263	Kdm3a	55818	-0.451921779	0.55591
10266	21413	Tcf4	6925	-0.288714318	0.94346
10267	30941	Usp21	27005	0.474805687	0.59089
10268	230598	Nrd1	4898	-0.45289863	0.41147
10269	16858	Lgals7		0.207155186	0.99988
10270	83410	Cstf2t	23283	0.8107281	0.0435
10271	73212	3110082I17Ri	84310	-1.098773052	0.2206
10272	207818	Smagp	57228	-0.308090981	0.78731
10273	15936	Ier2	9592	0.058407333	0.91426
10274	73830	Eif3k		-0.301967328	0.91943
10275	77097	Tanc2	26115	-0.145214447	0.89815
10276	100494	Zfand2a	90637	-0.322990703	0.51202
10277	224691	Zfp472		0.024404636	0.99988
10278	19895	Rpia	22934	0.435358779	0.58041
10279	14218	Sh3pxd2a	9644	0.060089163	0.94092
10280	102442	Dennd4a	10260	1.389599267	0.03799
10281	110695	Aldh7a1	501	-0.659304195	0.33679
10282	235611	Plxnb1	5364	-0.748713178	0.30588
10283	270802	BC048403	144577	0.9720706	0.6077
10284	319446	Dpep2		1.457062911	0.00652
10285	74103	Nebl	10529	0.304353791	0.99988
10286	235584	Dusp7	1849	1.101453071	0.01295
10287	329384	Ptrh1	138428	1.384965783	0.05293
10288	67772	Chd8	57680	-0.812404031	0.2703
10289	66075	Chchd3	54927	0.53364806	0.21388
10290	217779	Lysmd1	388695	-0.070137905	0.92365
10291	224111	Ubxn7	26043	1.079691425	0.01399
10292	107371	Exoc6	54536	-0.170572114	0.99988
10293	101612	Grwd1	83743	-0.627975278	0.64985
10294	108058	Camk2d		-0.171491021	0.99988
10295	15042	H2-T24		-0.354907358	0.96665
10296	209586	Nudcd3	23386	-0.563779296	0.45862
10297	109658	Txlna	200081	-0.927696284	0.28584

10298	75540	Fpgt	8790	0.006565371	0.99988
10299	100043597	Srcap		-1.262083698	0.19812
10300	72281	Sh2d4a		0.2369749	0.33619
10301	67547	Slc39a8	64116	-0.580262557	0.40517
10302	51798	Ech1		0.553126105	0.10438
10303	232087	Mat2a	4144	-1.653067331	0.12406
10304	67311	Nanp	140838	0.148810783	0.88959
10305	54151	Cyhr1	50626	-0.496368794	0.63555
10306	71994	Cnn3	1266	0.190472684	0.99988
10307	240442	Adnp2	22850	-0.458372617	0.8014
10308	16855	Lgals4		2.375674998	0.00591
10309	242202	Pde5a	8654	0.066653355	0.99988
10310	12525	Cd8a	925	0.294730673	0.9861
10311	69136	Tusc1	286319	0.67787655	0.01733
10312	15531	Ndst1	3340	-0.723087499	0.3364
10313	68346	Sirt5	23408	-0.364360339	0.59944
10314	103466	Nt5dc3		0.925178778	0.0368
10315	319955	Ercc6	2074	-0.715955908	0.44207
10316	56460	Pkp3	11187	-0.64053229	0.35513
10317	60440	Iigp1		0.841417395	0.19993
10318	217109	Utp18	51096	0.120245632	0.99988
10319	60594	Capn12		-0.14978962	0.75513
10320	67704	810037I17Ri	401152	0.729818054	0.01452
10321	319653	Slc25a40	55972	0.236893425	0.99988
10322	27401	Skp2	6502	1.46505039	0.00634
10323	16665	Krt15	3866	-0.747932871	0.58823
10324	73813	Fam83e	54854	0.893987027	0.01643
10325	68588	Cthrc1	115908	-0.771004302	0.60571
10326	76022	Gon4l		-1.032052657	0.18424
10327	107221	Ffar4	338557	-0.170441775	0.75196
10328	226695	Ifi205		0.874909844	0.21459
10329	69786	Tprkb	51002	0.717284177	0.21183
10330	70567	Fra10ac1	118924	-0.468863761	0.67524
10331	14184	Fgfr3	2261	0.363435759	0.82312
10332	16880	Lifr	3977	0.946645774	0.18247
10333	66251	Arfgap3	26286	0.27159302	0.33055
10334	215476	Prr14l	253143	-0.186107906	0.99988
10335	66266	Eapp	55837	0.086861145	0.77759
10336		D130007C19Rik		-0.887551391	0.33739
10337	54451	Cpsf3	51692	0.294919884	0.75164
10338	66292	Mrps21	54460	0.232063776	0.30766
10339	72504	Taf4b	6875	-1.100843689	0.22585
10340	16534	Kcnn4	3783	-1.203142844	0.25037
10341	11852	Rhob	388	-0.349941109	0.96052
10342	269997	Zfp747		-0.037738941	0.991
10343	17248	Mdm4	4194	0.122273355	0.84336
10344	75458	Cklf	51192	2.383111849	0.00463
10345	327978	Slfn5	162394	0.069327179	0.99988
10346	68598	Dnajc8	22826	-0.324633836	0.73639
10347	76687	Spcs3		0.129543806	0.93354

10348	66500	Slc30a7	148867	-0.43365232	0.40345
10349	2900041M22Rik			0.266662534	0.45989
10350	27062	Cadps		-0.550818942	0.65652
10351	68161	930005H10Rik		-1.146144666	0.16844
10352	11983	Atpif1	93974	0.344059043	0.15193
10353	330189	Tmem120b	144404	0.113412886	0.9979
10354	107526	Gimap4	55303	1.54622358	0.00565
10355	14797	Tle5	166	-0.256722216	0.75363
10356	56491	Vapb	9217	0.686616861	0.29276
10357	225010	Lclat1	253558	0.855080658	0.41923
10358	232078	Thnsl2	55258	0.825008399	0.19101
10359	96957	Tmem62	80021	0.124023548	0.96083
10360	67379	Dedd2	162989	-1.277267936	0.20739
10361	328417	Parp4	143	-0.620752795	0.29364
10362	70448	Atad3aos		0.75878882	0.73842
10363	338364	Trim65	201292	-0.406647117	0.90281
10364	237775	Zfp867		0.025821515	0.99988
10365	24055	Sh3bp2	6452	0.26767264	0.99039
10366	11489	Adam12	8038	0.007846281	0.99988
10367	18779	Pla2r1	22925	0.186584295	0.99916
10368	106143	Cggbp1	8545	-0.038654057	0.99988
10369	225876	Kdm2a	22992	-0.653663391	0.37037
10370	17314	Mgmt	4255	-1.167166674	0.19679
10371	70152	Mettl7a1		0.513880034	0.49077
10372	20541	Slc8a1	6546	0.874328021	0.01701
10373	70945	Mmrn1	22915		0.40878
10374	66869	Zfp869		0.238922301	0.9519
10375	244234	Scart2			0.40878
10376	231633	Tmem119	338773	1.791601669	0.00624
10377	72244	600014C10R	83636	0.307258851	0.991
10378	59308	Emcn	51705	0.449143194	0.78301
10379	11487	Adam10	102	0.071048099	0.991
10380	252903	Ap1s3	130340	0.520841979	0.38367
10381	232878	Zscan22	342945	0.652277561	0.46072
10382	244216	Zfp771	51333	-0.308958009	0.75953
10383	97165	Hmgb2	3148	-0.6540054	0.47126
10384	100604	Lrrc8c	84230	1.034375673	0.01704
10385	106639	Vmac	400673	0.240963356	0.99988
10386	218194	Phactr1	221692	0.284368299	0.99988
10387	110265	Msra	4482	-0.179112912	0.94808
10388	319535	Zfp182	7569	-1.448937984	0.1062
10389	319636	Fsd1l	83856	-0.059010973	0.99988
10390	56086	Set	646817	-0.524211519	0.33978
10391	51960	Kctd18	130535	-0.373932883	0.57658
10392	270201	Klhl18	23276	-0.690865054	0.43702
10393	60595	Actn4		-1.134098231	0.24785
10394	69727	Usp46	64854	0.029322151	0.9927
10395	234135	Nsd3	54904	-0.430558067	0.87859
10396	72341	Elp6	54859	-0.649231859	0.33739
10397	226255	Atrnl1	26033	0.07206053	0.99546

10398	106014	Tafa5	25817	-0.157077996	0.99988
10399	72309	Tmem158	25907	0.892651448	0.04991
10400	104401	Pcnx3	399909	0.195723244	0.71914
10401	109620	Dsp	1832	-1.256626869	0.22364
10402	22165	Txk	7294	0.191310573	0.99988
10403	384763	Zfp667	63934	0.676424052	0.33039
10404	68055	Dmac2l	27109	0.429614977	0.23425
10405	71778	Klhl5	51088	0.445915843	0.02793
10406	544922	Zkscan4		0.994299096	0.00945
10407	58802	Kcnmb4	27345	1.515584388	0.12619
10408	215708	Miga1	374986	-0.053457919	0.99988
10409	71583	130008F23Ri	135398	-0.966686043	0.25254
10410	239546	Zfp647	58500	0.006021546	0.99988
10411	347722	Agap1	116987	0.033053159	0.99988
10412	12805	Cntn1	1272	0.407559755	0.52465
10413	328572	Ep300	2033	0.145079591	0.90074
10414	66398	Commd5	28991	0.65337861	0.0155
10415	54132	Pdlim1	9124	0.182231244	0.9519
10416	18029	Nfic	4782	-0.398251468	0.4432
10417	67040	Ddx17	10521	0.021975175	0.99988
10418	69726	Smyd3	64754	-0.528128844	0.40525
10419	69787	Anxa13	312	0.494497403	0.20927
10420	11865	Arntl	406	0.509862955	0.02524
10421	68755	Cgrrf1	10668	1.268380772	0.22289
10422	192136	Sugct	79783	0.022025483	0.99988
10423	16598	Klf2	10365	1.683390317	0.00583
10424	50909	C1ra		1.252531895	0.02776
10425	108900	Fam72a		0.320840235	0.28126
10426	170742	Sertad3	29946	0.453682038	0.80616
10427	240063	Zfp811		1.744915721	0.00537
10428	81702	Ankrd17	26057	-0.134114061	0.79237
10429	17301	Foxd2	2306	-0.169447823	0.96921
10430	74287	Kcmf1	56888	-0.074403353	0.86796
10431	22643	Zfp101		-0.559534433	0.19606
10432	18212	Ntrk2	4915	1.53952856	0.01315
10433	242474	Tmem245	23731	0.020716936	0.99988
10434	11529	Adh7	131	-0.146629031	0.90546
10435	67568	Mrfap1		-0.045688238	0.99988
10436	22755	Zfp93		0.129237939	0.87013
10437	207352	Sec23ip	11196	-0.223266309	0.991
10438	21676	Tead1	7003	-0.119609193	0.8744
10439	21961	Tns1	7145	0.618402027	0.8659
10440		Gm9967		0.90434329	0.01737
10441	66069	Snupn	10073	-0.71291536	0.62381
10442	20538	Slc6a2	6530	0.341678846	0.60274
10443	56324	Stam2	10254	0.115605318	0.81099
10444	14348	Fut9	10690	-1.501733015	0.17054
10445	50762	Fbxo6	26270	0.111869254	0.99988
10446	17760	Map6	4135	-0.162866866	0.91475
10447	319767	Atp10b	23120	-0.263655692	0.75288

10448	211712	Pcdh9	5101	1.472958484	0.30506
10449	17132	Maf	4094	0.578894463	0.35792
10450	69207	Srsf11	9295	-0.798350983	0.30282
10451	16423	Cd47	961	-0.028260558	0.98957
10452	320706	Soga1	140710	-0.067433216	0.96578
10453	226169	Pprc1	23082	-0.450947941	0.51006
10454	432508	Cpsf6	11052	-0.086256998	0.99512
10455	71918	Zcchc24	219654	0.009168928	0.99988
10456	52855	Lair1		2.027844228	0.00447
10457	75620	Kxd1		0.519205904	0.10438
10458	328274	Zfp459		0.540994917	0.89825
10459	72432	Spink5	11005	-4.660161685	0.01363
10460	66953	Cdca7	83879	0.125831365	0.91039
10461	665700	Hmcn2	256158	1.588776445	0.1165
10462	68992	Zfp580	51157	-0.21634537	0.80565
10463	13134	Dach1	1602	-1.398062218	0.26998
10464	207952	Klhl25	64410	-0.071973058	0.98214
10465	14734	Gpc3	2719	2.225161398	0.01753
10466	76781	Mettl4	64863	0.458169774	0.48543
10467	195018	Zzef1	23140	-0.71540758	0.22169
10468	74901	Kbtbd11	9920	1.421840399	0.01557
10469	59042	Cope	11316	0.058175216	0.99988
10470	224019	Tmem191c		-0.654260283	0.32184
10471	234378	Klhl26	55295	-0.785600465	0.54694
10472	105439	Slain1	122060	0.951227162	0.2147
10473	66922	Rras2	22800	0.846401157	0.01627
10474	14600	Ghr	2690	0.868964765	0.59781
10475	74548	Gsdmc4		1.351918692	0.00681
10476	67242	Gemin6	79833	0.294294793	0.05099
10477	66656	Eef1d	1936	0.378252963	0.28584
10478	26874	Abcd2	225	1.342592207	0.00646
10479	21415	Tcf7l1	83439	-1.050503355	0.28293
10480	57778	Fmnl1	752	0.643296105	0.23202
10481	436022	Dnaaf3		0.506793961	0.16233
10482	116871	Mta3	57504	0.341773038	0.85685
10483	270328	Gsdmc3		1.564718155	0.00641
10484	22640	Zfp1	162239	-0.015538401	0.99988
10485	67673	Elob	6923	-0.153701935	0.64242
10486	66510	Rnf181		-0.28637338	0.67204
10487	71564	Izumo4	113177	0.442736345	0.4499
10488	18627	Per2	8864	0.714137128	0.19474
10489	104806	Fancm	57697	0.795046068	0.21231
10490	100043911	Ppp4r1l-ps		-0.768823378	0.49269
10491	232086	Tmem150a	129303	1.770112433	0.00388
10492	246196	Zfp277	11179	0.143730454	0.70966
10493	231326	Aasdh	132949	0.401026046	0.64336
10494	623781	Gm14137	643338	-0.744092007	0.45364
10495	26383	Fto	79068	-0.155487449	0.75897
10496	73024	Emc7	56851	-0.146446278	0.89195
10497	208820	Triqk	286144	0.444154621	0.2443

10498	71908	Cldn23	137075	0.614724033	0.60388
10499	14344	Fut2		-1.061728677	0.24053
10500	16367	Irs1	3667	-1.257951417	0.22712
10501	22757	Zkscan5	23660	-0.942872755	0.43255
10502	257632	Nod2	64127	0.88640235	0.42355
10503	236193	Zfp709		0.28035159	0.99988
10504	12722	Clca3a1		0.851224785	0.03101
10505	338366	Mia3	375056	-0.025022586	0.99382
10506	20201	S100a8	6279	1.393140284	0.07326
10507	20215	Sag	6295	-0.632739332	0.62525
10508	223433	Otulinl	54491	1.651300154	0.00677
10509	20202	S100a9		1.795979152	0.1705
10510	27979	Eif3b	8662	-0.204810568	0.80164
10511	20454	St3gal5	8869	-0.061184785	0.90701
10512	15039	H2-T22		-0.016051096	0.99053
10513	225020	Fez2	9637	0.746970346	0.01772
10514	56386	B4galt6		-0.795298369	0.3235
10515	225471	Ticam2		1.456440878	0.02754
10516	109785	Pgm3	5238	0.10798954	0.99988
10517	94094	Trim34a		0.546980716	0.02435
10518	229694	AI504432		1.901672783	0.19656
10519	103142	Rdh9		0.226076849	0.99988
10520	54607	Socs6	9306	-0.058510772	0.99988
10521	78893	Cnot10	25904	-0.530958749	0.37464
10522	329941	Col8a2	1296		0.40878
10523	225861	Snx32	254122	-0.31493815	0.59
10524	12631	Cfl1	1072	-0.108357595	0.82397
10525	66522	Pgpep1	54858	-0.568968363	0.35982
10526	18150	Npm3	10360	0.353688735	0.0991
10527	226412	R3hdm1	23518	-0.033751586	0.99988
10528	93737	Pard6g	84552	-1.30107557	0.21923
10529	12611	Cebpg	1054	-0.092226953	0.95893
10530	18783	Pla2g4a	5321	0.492434714	0.95337
10531	71941	Cars2	79587	0.556506846	0.31651
10532	27057	Ncoa4	8031	0.51172081	0.03072
10533	321000	Lrif1	55791	0.015545733	0.99988
10534	68121	Cep70	80321	-0.17396487	0.99988
10535	329260	Dennd1b	163486	-0.043906249	0.99988
10536	109314	Prr9	574414		0.991
10537	60361	Ms4a4b		2.569509976	0.00538
10538	331063	Gsdmc2		1.626098819	0.00566
10539	28035	Usp39	10713	-0.37661328	0.45767
10540	100929	Tyw1		-0.445776359	0.4291
10541	69068	Tcim		-0.302166587	0.99039
10542	17847	Usp34	9736	-0.277781399	0.69432
10543	242894	Actr3b		0.977045279	0.27974
10544	233168	AI987944		0.388420376	0.45946
10545	16881	Lig1	3978	-1.378543516	0.1716
10546	231821	Adap1	11033	0.370597615	0.98522
10547	20564	Slit3	6586	1.175267922	0.21616

10548	22134	Tgoln1	10618	-0.481096804	0.41843
10549	105242657	Hoxaas2		1.084351807	0.11226
10550	26448	Mok	5891	-1.425097783	0.28986
10551	109929	Zbtb25	7597	0.420649311	0.48448
10552	70445	Cd248	57124	0.768989064	0.82246
10553	224792	Adgrf5	221395	-0.02766113	0.98675
10554	17425	Foxk1	221937	-0.622817755	0.47288
10555	320782	Tmem154	201799	-0.622841801	0.62665
10556	12608	Cebpb	1051	1.122567104	0.36857
10557	106572	Rab31	11031	0.055685703	0.9542
10558	19204	Ptafr	5724	2.175017691	0.00547
10559	27392	Pign	23556	-0.43739616	0.51485
10560	19820	Rlim	51132	-0.09084624	0.74503
10561	17528	Mpz	4359	0.761562994	0.02032
10562	544752	Tug1		-0.397630828	0.46818
10563	210104	Zfp658		0.134456719	0.91366
10564	69539	Trnp1	388610	0.04590226	0.99988
10565	74665	Drc3	83450	-0.962921658	0.20832
10566	320365	Fry	10129	-0.247974801	0.56527
10567	109151	Chd9	80205	-0.667725534	0.3155
10568	18938	Ppp1r14b		-0.923732686	0.27917
10569	14227	Fkbp2	114841035	-0.509330313	0.49074
10570	13512	Dsg3		-0.91133068	0.31763
10571	67442	Retsat	54884	0.736508307	0.1705
10572	77619	Prelid2	153768	0.229073628	0.99988
10573	20592	Kdm5d	8284	0.543569088	0.64271
10574	224647	Ilrun	64771	-0.496907611	0.42297
10575	232089	Elmod3	84173	0.356669027	0.37904
10576	15939	Ier5	51278	0.541257094	0.52176
10577	235627	Nbeal2	23218	-0.889415139	0.17965
10578	109202	.930024E05Rik		0.313409396	0.76459
10579	12332	Capg	822	-0.080681655	0.95108
10580	18030	Nfil3	4783	-0.621350527	0.55283
10581	211660	Cspp1	79848	-0.914942101	0.22624
10582	52690	Setd3	84193	-0.00517155	0.99988
10583	53424	Tsnax	7257	-0.195892749	0.99988
10584	264134	Ttc26	79989	0.414710712	0.46561
10585	18521	Pcbp2		-0.248150158	0.89645
10586	70676	Gulp1	51454	0.266708336	0.61605
10587	73690	Glipr1	11010		0.40878
10588	93757	Immp2l	83943	0.01886275	0.99988
10589	20469	Sipa1	6494	1.459133909	0.00678
10590	382090	Cep162	22832	-0.059421061	0.89195
10591	67131	Acbd4	79777	-0.848657504	0.19457
10592	99311	Commd7	149951	0.019487843	0.99988
10593	381801	Tatdn2		-0.49081487	0.45888
10594	107817	Jmjd6	23210	-0.086138816	0.99988
10595	104158	Ces1d		1.832955597	0.00892
10596	15925	Ide	3416	-1.194866684	0.16588
10597	67475	Ero1b	56605	0.40191713	0.72669

10598	17178	Fxyd3		-0.062160196	0.99988
10599	112415	Zfp607b		-1.532403995	0.16244
10600	13591	Ebf1	1879	-0.217385936	0.99988
10601	210135	Zfp180	7733	0.149164781	0.70409
10602	66116	Nat8f1		0.375810075	0.99988
10603	26920	Cntrl	11064	-0.475468843	0.59089
10604	18148	Npm1	4869	0.214798348	0.66769
10605	27366	Txnl4a	10907	-0.372170516	0.52103
10606	77945	Rpgrip1	57096	0.033856287	0.99988
10607	71389	Chd6	84181	-0.345630707	0.63683
10608	211488	Ado	84890	0.13232974	0.99988
10609	68487	Tmem140	55281	0.468200075	0.5024
10610	319236	Trim12c		0.191682179	0.7835
10611	66632	Dph6	89978	0.044903265	0.99988
10612	239099	Homez	57594	-0.111327459	0.83925
10613	22072	Prss2		0.428428333	0.92288
10614	233899	Ccdc189	90835	-0.82723063	0.31678
10615	606496	Gsk3a	2931	-1.968940424	0.06559
10616	70591	730455P16R	64149	-0.83551848	0.25907
10617	20269	Scn3a	6328		0.991
10618	382062	AB124611	255809	2.654059609	0.00406
10619	68682	Slc44a2	57153	0.099265673	0.99988
10620	276905	Armc7	79637	1.217950068	0.01388
10621	66349	Dmac2	55101	0.946201291	0.01315
10622	269774	Aak1	22848	-1.09257865	0.12595
10623	76894	Mettl15	196074	0.822426905	0.16614
10624	19646	Rbbp4	5928	0.729173394	0.02147
10625	407788	BC051142	10665	-0.364353597	0.89003
10626	68011	Snrpg	6637	0.716633018	0.01236
10627	20446	St6galnac2	10610	0.140961131	0.99988
10628	231532	Arhgap24	83478	0.34097148	0.75897
10629	67671	Rpl38	6169	0.368001833	0.37705
10630	12043	Bcl2	596	-0.023825135	0.99988
10631	545389	Cep170	9859	0.884349484	0.15459
10632	56632	Sphk2	56848	-0.398401874	0.72244
10633	223672	Apol9a		-0.776856563	0.2223
10634		Gm17494		0.344126282	0.95663
10635	67883	Uxs1	80146	-0.179561376	0.62665
10636	11797	Birc2	329	0.113335907	0.99988
10637	230584	Yipf1	54432	-0.023313698	0.9787
10638	67681	Mrpl18	29074	0.206674485	0.88821
10639	268670	Zfp759		0.446200063	0.06605
10640	107823	Nsd2	7468	-0.605556412	0.48558
10641	24132	Zfp53		-0.703281746	0.60287
10642	214917	Antkmt		-1.054786166	0.2443
10643	76130	Las1l	81887	-0.856788473	0.28128
10644	75739	Mpp7	143098	-0.171848443	0.89518
10645	50496	E2f6	1876	1.479321169	0.00766
10646	66488	Fam136a	84908	-1.33650404	0.17976
10647	73689	Bloc1s2	282991	-0.674213358	0.52348

10648	20747	Spop		-0.522446576	0.3422
10649	230857	Ece1	1889	0.331279229	0.32894
10650	94245	Dtnbp1	84062	0.072606203	0.99988
10651	78697	Pus7	54517	-0.345934433	0.68231
10652	244713	Zfp317	57693	0.243185725	0.99988
10653	56048	Lgals8	3964	0.385835425	0.4271
10654	13664	Eif1a		0.098448108	0.99988
10655	67106	Zbtb8os	339487	0.450346261	0.29735
10656		Cox7c-ps1		-0.121154611	0.89285
10657	70317	Arl16	339231	1.087115542	0.01175
10658	209387	Trim30d		-0.068133457	0.95854
10659	30937	Lmcd1	29995	0.295060539	0.99988
10660	14677	Gnai1	2770	-1.369191589	0.24779
10661	110593	Prdm2	7799	0.02665622	0.98004
10662	105246	Brd9	65980	-0.496483846	0.40418
10663	14433	Gapdh		-0.76201915	0.29276
10664	232946	Bloc1s3	388552	-0.22205908	0.62525
10665	320795	Pkn1	5585	-0.410832696	0.56643
10666	69228	Zfp746	155061	-0.358270833	0.99988
10667	269016	Sh3rf2	153769	-0.365516977	0.68933
10668	20740	Sptan1	6709	0.116249019	0.8193
10669		Gm6169		0.237052793	0.38074
10670	225187	Ankrd29	147463	1.09806064	0.18112
10671	192986	Cyb5d2	124936	-0.004382714	0.99988
10672	234374	Ddx49	54555	-0.331479111	0.62033
10673	104349	Zfp119a		0.457810524	0.21178
10674	19951	Rpl32	6161	0.543805304	0.1952
10675	218314	Zfp595		1.21839067	0.0094
10676	76539	Fam204a	63877	-0.091959387	0.95546
10677	54217	Rpl36	25873	-0.092853153	0.89195
10678		Cbx3-ps6		-1.106095834	0.33451
10679	67230	Zfp329	79673	-0.406023395	0.40369
10680	22646	Zfp105	7584	0.193805769	0.45895
10681	12323	Camk2b	816	0.208301457	0.99988
10682	70450	Unc13d	201294	1.118272355	0.30164
10683	217837	Itpk1	3705	-1.257010768	0.23705
10684	20347	Sema3b	7869	0.220623168	0.78261
10685	235047	Zfp809		0.638367682	0.09342
10686	100019	Mdn1	23195	-0.632280229	0.38458
10687	52398	Septin11	55752	-0.007353687	0.99988
10688	72333	Palld	23022	-0.0607506	0.93776
10689	68519	Eml1	2009	1.406589481	0.00447
10690	66052	Sdhc	6391	0.662202139	0.02378
10691	100416706	Zfp729b		0.522359369	0.325
10692	74039	Nfam1	150372	0.575522016	0.46166
10693		Tpm3-rs7		-0.276436991	0.99988
10694	14862	Gstm1		-0.109035899	0.99988
10695	78923	Chsy3	337876	-0.133774563	0.99988
10696	432555	Gm5431		-0.858272009	0.07088
10697	170748	Smco4	56935	-0.089872267	0.99988

10698	244721	Zfp846		0.49631349	0.91985
10699	232906	Arhgap35	2909	0.081296935	0.8756
10700	22282	Usf2	7392	0.893538467	0.09286
10701	66609	Cryzl1	9946	1.012742828	0.01102
10702	21912	Tspan7		0.212849058	0.48662
10703	319554	Idi1	3422	-0.140664987	0.99988
10704	64659	Mrps14	63931	0.596562284	0.0214
10705	105988	Espl1	9700	0.359637084	0.79823
10706	24135	Zfp68		-1.119355654	0.26012
10707	71567	Mcm9	254394	0.909235318	0.22582
10708	19704	Upf1	5976	-0.249261106	0.71917
10709	218793	Ube2e2	7325	0.174458078	0.98575
10710	192285	Phf21a	51317	-0.193574803	0.80479
10711	330662	Dock1	1793	-0.800879585	0.61072
10712	22746	Zfp85		0.799520317	0.60826
10713	66487	Smim4	440957	0.338933651	0.4544
10714	16687	Krt6a		-1.850816818	0.20884
10715	24015	Abce1	6059	-0.348347654	0.71611
10716	18685	Phtf1	10745	-0.242089396	0.75903
10717	72462	Rrp1b	23076	0.038624415	0.99988
10718	233058	Zfp420	170960	0.677411645	0.02046
10719	98258	Txndc9	10190	0.655857098	0.01057
10720	20310	Cxcl2		-1.34071981	0.30854
10721	18181	Nrf1	4899	-0.101480111	0.95793
10722		Rpl10-ps3		0.364570619	0.04221
10723	23938	Map2k5	5607	-0.192292736	0.93342
10724	387524	Znrf2	223082	0.028111	0.98653
10725	13360	Dhcr7	1717	-0.047385965	0.99988
10726	666926	Gm8369		1.234075575	0.00883
10727	101240	Wdr91	29062	0.276768883	0.99053
10728		Scp2-ps2		0.437651306	0.02372
10729	68152	Fam133b	257415	-0.336047633	0.6893
10730	268449	Rpl23a	6147	0.318455627	0.67777
10731	100503670	Rpl5	6125	0.234091221	0.68043
10732	67511	Tmed9	54732	0.62873442	0.21751
10733	23888	Gpc6	10082	-0.022706795	0.99988
10734	68607	Serhl	253190	-0.142806818	0.94734
10735	50875	Tmod3	29766	0.470322695	0.28943
10736	50755	Fbh1	84893	0.000830477	0.99988
10737	19946	Rpl30	6156	0.237733823	0.72736
10738	14544	Gda	9615	0.385598861	0.52203
10739	65020	Zfp110		0.258048962	0.75796
10740	75705	Eif4b	1975	-0.140628069	0.64648
10741	320679	Samd12	401474	0.424342495	0.28598
10742	22151	Tubb2a	7280	0.881080884	0.00975
10743	72972	Ccser2	54462	-0.323390538	0.83449
10744	76890	Memo1	51072	-0.135637466	0.96054
10745	68364	610030E20R	388969	0.939580945	0.00613
10746	112406	Egln2	112398	-0.099462497	0.99988
10747	14127	Fcer1g	2207	1.742262252	0.00493

10748	72568	Lin9	286826	-0.239817799	0.991
10749	233987	Zfp958		0.058160198	0.99988
10750	18413	Osm	5008	0.817782531	0.11574
10751	21833	Thra	7067	-0.397823758	0.96054
10752	108937	Rnf169	254225	0.193056377	0.99988
10753	110911	Cds2	8760	0.553156732	0.4548
10754	18022	Nfe2			0.991
10755	53605	Nap1l1	4673	0.16650166	0.99988
10756	18733	Pirb		2.068991262	0.00547
10757	66462	Rex1bd	55049	-0.240777823	0.68937
10758	11308	Abi1	10006	0.471136356	0.01865
10759	329003	Zfp516	9658	-0.699513693	0.36168
10760	432769	Zfp708		0.446200063	0.4244
10761	54006	Deaf1	10522	-0.826704882	0.30673
10762	77018	Col25a1	84570	-0.347011518	0.82426
10763	380855	Rsl1		1.100847674	0.01052
10764	81799	C1qtnf3			0.40878
10765	241877	Slc10a5	347051	-0.842408921	0.38601
10766		Gm10053		-0.573841861	0.40494
10767	12630	Cfi		1.154822321	0.10409
10768	214932	Hdhd5	27440		0.991
10769	219189	Vwa8	23078	-0.945086841	0.3352
10770	229279	Hnrnpa3	220988	0.05027634	0.91027
10771	27387	Sh2d3c	10044	1.769399488	0.10205
10772		9630013D21Rik		1.270678054	0.12843
10773	258728	Olfr482		-0.485536075	0.82705
10774	433182	Eno1b		-0.701345511	0.35266
10775	19363	Rad51b	5890	3.016687955	0.00493
10776	19899	Rpl18	6141	-0.220474169	0.95589
10777	246256	Fcgr4		2.176874233	0.00544
10778	213002	Ifitm6		1.20513862	0.14661
10779	17955	Nap1l4	4676	0.643331091	0.30728
10780	240041	Zfp945		0.006996448	0.99988
10781	213006	Mfsd4a	148808	-1.143519007	0.24891
10782	18573	Pde1a	5136	0.328359863	0.99741
10783	54353	Skap2	8935	0.473124051	0.27774
10784	69606	Mtfmt	123263	0.211786294	0.39352
10785	76936	Hnrnpm	4670	-0.347575907	0.63574
10786	74996	Usp47	55031	-0.193906679	0.93113
10787	330474	Zc3h4	23211	0.204327417	0.48697
10788	78304	Naa38	84316	0.475151752	0.26685
10789	12593	Cdyl	9425	0.379284832	0.98419
10790	67025	Rpl11	6135	-0.00387257	0.99988
10791	26569	Slc27a4	10999	-0.250428772	0.76432
10792	72749	Tonsl	4796	-0.280608358	0.70348
10793	74318	Hopx	84525	-0.430188251	0.60943
10794	12982	Csf2ra	1438	0.835660156	0.10683
10795	108052	Slc14a1	6563		0.40878
10796	414077	Wdr83os		-0.354897128	0.82835
10797	14297	Fxn	2395	-0.778112283	0.30315

10798	66707	Nkapl	222698	0.05891419	0.99988
10799	21770	Ppp2r5d	5528	0.064902879	0.99988
10800	11936	Fxyd2			0.40878
10801	242747	Zfp933		1.434059953	0.0177
10802	11468	Actg2	72	1.44372562	0.02148
10803	17187	Max	4149	0.282205086	0.08681
10804	192197	Bcas3	54828	0.305597035	0.96923
10805	231086	Hadhb	3032	-0.344823503	0.4834
10806	19229	Ptk2b	2185	0.06651452	0.99988
10807	103537	Mbtd1	54799	-0.698893889	0.32908
10808	235028	Zfp426	79088	-0.67951592	0.33555
10809	232984	B3gnt8	374907	1.370128555	0.00934
10810	67157	610301B20Ri	157657	0.918098293	0.01274
10811	210973	Kbtbd2	25948	1.129769295	0.00777
10812	195727	Nhs	4810	0.348850957	0.99988
10813	69632	Arhgef12	23365	-0.73858175	0.24121
10814	14131	Fcgr3		1.720833658	0.00577
10815	70103	Znhit1	10467	-0.900950593	0.43622
10816	66152	Uqcr10	29796	-0.068842707	0.99988
10817	21400	Tcea2	6919	0.742009166	0.02098
10818	215814	Ccdc28a	25901	-0.815375989	0.27535
10819	227612	Tor4a	54863	1.197684261	0.00583
10820	68501	Nsmce2	286053	0.874936458	0.01018
10821	54598	Calcrl	10203	0.628336676	0.52758
10822	27204	Syn3	8224	0.231718703	0.81688
10823	16682	Krt4	3851	0.740872966	0.20884
10824	21340	Taf1b	9014	0.259410299	0.84786
10825	232337	Zfp637	7580	-0.260945023	0.80354
10826	338351	Akap17b		-1.395876504	0.18346
10827	53902	Rcan3	11123	0.361535254	0.41843
10828	14251	Flot1	10211	-0.36196787	0.57435
10829	225887	Ndufs8	4728	0.137573533	0.99988
10830	170738	Kcnh7	90134	0.171555959	0.92894
10831	110196	Fdps	2224	-0.313774698	0.54595
10832	75764	Slx1b		0.064026788	0.99988
10833		Rps26-ps1		-0.261768509	0.62677
10834	106582	Nrm	11270	-0.688394579	0.53317
10835	13681	Eif4a1	1973	-0.656969493	0.37475
10836	50780	Rgs3	5998	-0.261891786	0.9861
10837	56298	Atl2	64225	0.019992231	0.99988
10838	270156	Nkapd1	55216	-0.403091981	0.57989
10839	13170	Dbp	1628	1.598313758	0.01222
10840	67161	Sclt1	132320	-0.786270671	0.40118
10841	408067	Zfp874b		5.086075593	0.00169
10842	232811	Kmt5c	84787	-0.097980271	0.99988
10843	231130	Tnip2	79155	-0.097204307	0.82658
10844	67255	Zfp422	7570	0.868242171	0.01311
10845	266632	Irak4	51135	-0.673463484	0.38173
10846	140630	Ube4a	9354	1.047673342	0.03189
10847	19245	Ptp4a3	11156	-1.025839144	0.01076

10848	234358	Zfp930		0.817617194	0.1583
10849	13507	Dsc3		0.13295239	0.34158
10850	94346	Tmem40	55287	-0.262816741	0.72306
10851	17836	Mug1		-2.644122284	0.05383
10852		Rpl21-ps6		-0.710772282	0.7594
10853	67609	930453N24R	285237	-0.50042197	0.44789
10854	14784	Grb2	2885	0.789161999	0.01391
10855	230996	430015G10R	54991	-0.18467136	0.92733
10856	15512	Hspa2	3306	0.866300365	0.02527
10857	72723	Zfp74	148266	0.796722945	0.04556
10858	381921	Taok2	9344	-0.318339579	0.66836
10859	229499	Fcrl1	115350		0.40878
10860	217218	Atxn7l3	56970	-0.307057458	0.85103
10861	212862	Chpt1	56994	-0.249594925	0.67404
10862	16554	Kif13b	23303	0.022292691	0.99988
10863	232440	H2aj		-1.100611049	0.25336
10864	244218	Ctf2		1.420986795	0.00817
10865	27367	Rpl3	6122	-0.274232686	0.68501
10866	330817	Dhps		-0.797301136	0.33086
10867	11690	Alox5ap	241	2.472335116	0.00406
10868	19167	Psma3	5684	0.659282828	0.0293
10869	19889	Rp2	6102	0.134831747	0.88097
10870	214572	Prmt7	54496	0.254018892	0.97785
10871	66603	Gemin2	8487	0.570270859	0.34305
10872	22070	Tpt1	7178	0.207989097	0.99988
10873	241633	Atp8b4	79895	0.032465334	0.89477
10874		Gm10076		0.316583274	0.33427
10875	20719	Serpinb6a		-0.081989191	0.99988
10876	213811	BC002059		-0.108968698	0.99988
10877	117109	Pop5	51367	0.169343341	0.18112
10878	27801	Zdhhc8	29801	-0.655781519	0.39375
10879	215436	Slc35e3	55508	0.274542594	0.99762
10880	232983	Cxcl17	284340	-0.814776016	0.56261
10881	242466	Zfp462	58499	-1.372770103	0.16906
10882	216869	Arrb2	409	1.403472808	0.00888
10883	74580	Pyroxd2	84795	-1.074313665	0.26415
10884	319996	Golm2	113201	1.48914806	0.06764
10885	56009	Alyref2		1.182977203	0.10447
10886	101631	Pwwp2b	170394	-1.540263136	0.12651
10887	14886	Gtf2i	2969	0.555137418	0.49077
10888	11771	Ap2a1	160	-1.021865301	0.21678
10889	66101	Ppih		0.1682048	0.49408
10890	72128	610008E11Rik		-0.12309168	0.9979
10891	407812	Zfp941	162655	-1.245936417	0.55251
10892	245174	Zfp937		-0.04449485	0.99988
10893	15381	Hnrnpc		0.169518756	0.17263
10894	12039	Bckdha		-1.407946009	0.13225
10895		Rpl36a-ps1		0.113133323	0.79266
10896		C030014I23Rik		1.147807756	0.11881
10897	243833	Zfp128	7554		0.991

10898	20649	Sntb1	6641	-0.218704955	0.96481
10899	667823	Trim5		0.084082323	0.99988
10900	320558	Sycp2	10388	-1.51624404	0.00808
10901	56736	Rnf14	9604	0.201249273	0.78706
10902	385643	Kng2		1.08156153	0.0188
10903	54672	Adgrg3	222487	0.498517728	0.84308
10904	60532	Wtap	9589	-0.187931019	0.99074
10905	108960	Irak2	3656	0.831128169	0.01384
10906	320825	Samd5	389432	-1.278036808	0.38074
10907	23832	Xcr1	2829	-0.369123892	0.80869
10908	77519	Zfp266	10781	0.211241389	0.99024
10909	76261	610040J01Ri	55286	-0.810250032	0.42644
10910	30935	Tor3a	64222	0.34289994	0.39287
10911	68742	Tmem219	124446	0.478247515	0.0332
10912	29820	Tnfrsf19	55504	-0.200289871	0.99988
10913	15018	H2-Q7		0.237181777	0.99988
10914	226610	Fam78b	149297	1.007133209	0.22496
10915	17150	Mfap2	4237	0.435003078	0.95839
10916	14969	H2-Eb1		2.215548943	0.00388
10917	80876	Ifitm2		0.172972246	0.22288
10918	13808	Eno3	2027	1.287852515	0.02298
10919	22260	Nr1h2	7376	-0.067682401	0.99988
10920	69547	Nkpd1	284353	-0.886947404	0.53701
10921	57808	Rpl35a	6165	0.040856463	0.99988
10922	319158	H4c9		0.04239839	0.99988
10923	223989	Marf1	9665	-1.158366012	0.15486
10924	54667	Atp8b2	57198	0.446851237	0.83565
10925	225845	Plaat3	11145	0.925995637	0.0155
10926	319155	H4c3		0.411356173	0.99988
10927	69527	Mrps9	64965	0.281323996	0.57622
10928	236794	Slc9a6	10479	0.837865144	0.26175
10929	66205	Cd302	9936	-0.087684638	0.97371
10930	117197	Bloc1s4	55330	0.38439016	0.03762
10931	211945	Plekhh1	57475	-0.122967992	0.92691
10932	69718	Ipmk	253430	-0.162059044	0.99988
10933	59050	Nsa2	10412	-0.272949491	0.52557
10934	15078	H3f3a		0.150056918	0.48422
10935	211484	Tsga10	80705	-1.15431991	0.09142
10936	63986	Gmfg	9535	1.058010894	0.00775
10937	380614	Intu	27152	0.186605107	0.73305
10938	12010	B2m	567	0.095306948	0.99988
10939	14870	Gstp1		-0.222364842	0.99053
10940	230848	Zbtb40	9923	0.658745942	0.16402
10941	104303	Arl1	400	0.022792457	0.99988
10942	75572	Acyp2	98	0.136688323	0.99988
10943	94109	Csmd1	64478	-1.201596088	0.22364
10944	103266	Tmem263	90488	-0.566946109	0.44919
10945	19941	Rpl26	6154	0.256033205	0.43622
10946	328162	Trmt61a	115708	-0.295813934	0.75419
10947	54403	Slc4a4	8671	-1.421600292	0.20884

10948	73712	Dmkn	93099	2.120205919	0.04631
10949	16371	Irx1	79192	1.719013087	0.04135
10950	69386	H4c8		-0.545055077	0.27799
10951	214575	Tdrd5	163589	-0.649231859	0.26695
10952	56447	Copz1		-0.002184879	0.99988
10953	210719	Mkx	283078	1.516754941	0.00504
10954	59014	Rrs1	23212	0.41166071	0.70059
10955	53609	Clasrp	11129	0.535216253	0.69783
10956	18114	Rrp1	8568	-0.415593832	0.46868
10957	258783	Olfr920		-1.217146866	0.36275
10958	68977	Haghl	84264	-0.50764963	0.90192
10959	12560	Cdh3	1001	0.519906747	0.8717
10960	20841	Zfp143	7702	0.104221765	0.99988
10961	17896	Myl4	4635	0.359731996	0.11767
10962	57262	Retnla		3.075989304	0.00406
10963	100041953	Sap18b		0.345394622	0.97358
10964	192173	Mcrip1	348262	-0.426491723	0.38207
10965	66114	Dnajc30	84277	-0.300123298	0.99838
10966	72461	Prcp	5547	1.291688863	0.08607
10967	19043	Ppm1b		-0.240644482	0.99988
10968	17060	Blnk	29760	-0.691760093	0.32388
10969	56194	Prpf40a	55660	-0.854691635	0.24145
10970	433586	Maml3	55534	-2.487654987	0.03541
10971	329679	Fnip2	57600	-0.630583873	0.46813
10972	54402	Stk19	8859	-0.638778132	0.37241
10973	14972	H2-K1		-0.510494909	0.4026
10974	105504	Exoc5	10640	-0.281120674	0.62844
10975	236792	Mmgt1		-0.391813755	0.29072
10976	27998	Exosc5	56915	0.515922376	0.02726
10977	330177	Taok3	51347	-1.161454104	0.2103
10978	72055	Slc38a10	124565	-0.687979608	0.35718
10979	72108	Ddhd2	23259	-0.472929031	0.72874
10980	17938	Naca	4666	0.050350083	0.97706
10981	20315	Cxcl12	6387	0.78832928	0.54386
10982	68479	Phf5a	84844	0.693151978	0.23011
10983	408062	Zfp873		0.456895246	0.99672
10984	23877	Fiz1	84922	-1.125712313	0.251
10985	11481	Acvr2b	93	-0.268359636	0.99988
10986	223917	Krt79	338785	0.532260845	0.78305
10987	142682	Zcchc14	23174	-0.856906489	0.36549
10988	329540	Nol4l	140688	-0.08182097	0.99988
10989	381812	Cracr2a	84766	-0.107553572	0.93548
10990	15258	Hipk2	28996	-0.741508287	0.31215
10991	67727	Stx17	55014	-0.645096736	0.44141
10992	217431	Nol10	79954	0.490613377	0.08607
10993	66278	Smim20	389203	-0.709969726	0.30673
10994	66128	Mrps36	92259	0.464723224	0.02646
10995	20115	Rps7	6201	0.429283277	0.0678
10996	53607	Snrpa		-0.865340733	0.27974
10997	319156	H4c4	8294	-0.088549895	0.99988

10998	12859	Cox5b		0.124333018	0.85285
10999	110308	Krt5		0.311126721	0.7856
11000	75216	Cep128	145508	-0.706701788	0.8323
11001	109323	C1qtnf7	114905	-0.411528749	0.65197
11002	215474	Sec22c	9117	-0.607043184	0.45895
11003	381067	Zfp229		-0.174409695	0.72073
11004	66317	Wdr61	80349	0.223241524	0.16438
11005	382045	Adgrg5	221188	-0.369123892	0.38663
11006	208266	Dot1l	84444	-0.375071532	0.57538
11007	240087	Mdc1	9656	0.250128449	0.7652
11008	108121	U2af1		-0.187567354	0.78646
11009	192191	Med9	55090	0.239007956	0.40182
11010	12488	Cd2ap	23607	-0.537853324	0.49205
11011	66569	Gdpd1	284161	-0.970929982	0.2081
11012	228836	Dlgap4	22839	0.479793826	0.18629
11013	19049	Ppp1r1b	84152	0.628848624	0.29579
11014	14042	Ext1	2131	-0.212780146	0.81748
11015	56448	Cyp2d22		-0.728219419	0.42215
11016	545156	Kalrn	8997	0.039935552	0.97215
11017	665775	Bod1l	259282	-0.784442048	0.32126
11018	67861	Akr1b10		-0.330539282	0.52584
11019	73419	Armt1	79624	0.436125195	0.28119
11020	21333	Tac1	6863	-0.099793886	0.99988
11021	76763	Mospd2	158747	0.382365445	0.87025
11022	11537	Cfd	1675	-0.024159216	0.99988
11023	20068	Rps17		0.319308873	0.05738
11024	20917	Suclg2	8801	-0.592251094	0.43717
11025	12695	Patj	10207	-0.196545604	0.86915
11026	20698	Sphk1	8877	0.100441623	0.99988
11027	208908	Ccdc62	84660	-1.610917294	0.0634
11028	72475	Ssbp3	23648	-0.803097764	0.29735
11029	269585	Zscan20	7579	-0.27698833	0.90134
11030	57782	Rbak	57786	-0.682850409	0.33655
11031	18674	Slc25a3		0.44265102	0.07591
11032	70351	Ppp4r1	9989	0.085095606	0.95337
11033	13897	Ces1e		0.907710526	0.18633
11034	94254	Rcc1l	81554	-0.673528086	0.49931
11035	14252	Flot2	2319	-0.259302987	0.77759
11036	20042	Rps12		0.461206191	0.03739
11037	68436	Rpl34	6164	0.396344225	0.2416
11038	63985	Gmfb	2764	-0.011862345	0.99988
11039	212974	Pgghg	80162	-0.377171943	0.519
11040	22689	Zfp27		1.917222877	0.00544
11041	67732	Iah1	285148	0.374854102	0.36204
11042	18655	Pgk1	5230	-0.378950409	0.36879
11043	16907	Lmnb2	84823	0.416200694	0.49727
11044	19317	Qk	9444	0.697663221	0.49622
11045	239849	Cd200r4		1.925234343	0.00447
11046	328451	Gm10110		-0.331514296	0.47207
11047	228662	Btbd3	22903	0.782986792	0.2181

11048	212986	Scfd2	152579	0.719113624	0.37991
11049	19377	Rai1	10743	0.266708336	0.99988
11050	232853	Zfp954		0.357033791	0.44596
11051	80281	Cttnbp2nl	55917	1.432909165	0.00777
11052	242700	Ifnlr1	163702	-0.371568573	0.52673
11053	98417	Cnih4	29097	0.307849378	0.46908
11054	50786	Hs6st2	90161	-1.211463905	0.24843
11055	72658	700097O09Rik		-0.174733247	0.86071
11056	224671	Btbd9	114781	0.149540501	0.99344
11057	14852	Gspt1	2935	-0.430729489	0.40374
11058	106869	Tnfaip8	25816	0.378048452	0.32967
11059	76089	Rapgef2	9693	-0.277504062	0.99988
11060	231580	Gak	2580	-0.600566124	0.2743
11061	66197	Cks2	1164	-0.939276961	0.26685
11062	21761	Morf4l1		0.689395239	0.0207
11063	19294	Nectin2	5819	-0.556212589	0.37529
11064	13866	Erbb2	2064	0.331440506	0.5645
11065	319195	Rpl17		-0.138798381	0.99988
11066	16413	Itgb1bp1	9270	1.351992437	0.14124
11067	74868	Tmem65	157378	0.494307503	0.91291
11068	66439	Borcs7		-0.061672632	0.95974
11069	22152	Tubb3	10381	0.024009512	0.90576
11070	434624	Ftl1-ps1		-0.053940103	0.99988
11071	68036	Zfp706	51123	0.033552722	0.99988
11072	11841	Arf2		0.170082696	0.50521
11073		Rpl9-ps6		0.247889272	0.99988
11074	224530	Acat3		2.860962853	0.00388
11075	667370	Ifit3b		0.162265342	0.99988
11076	381318	Nsl1	25936	0.713362032	0.0709
11077	11770	Fabp4	2167	-0.426538857	0.97675
11078	272347	Zfp398	57541	0.695819399	0.02313
11079	225651	Mppe1	65258	0.316599438	0.74793
11080	60510	Syt9	143425	0.704037533	0.31619
11081	384059	Tlr12			0.991
11082	12879	Cys1	192668	-0.240359493	0.86848
11083	21854	Timm17a	10440	-0.189761401	0.94963
11084	78795	Armc9	80210	-0.351211799	0.96083
11085	20817	Srpk2	6733	-0.064643138	0.93478
11086	16516	Kcnj15	3772	0.912768889	0.56135
11087	67101	310039H08R	441150	0.970809658	0.04753
11088	320713	Mysm1	114803	-1.161760596	0.17477
11089	76051	Ganc		0.186349424	0.96415
11090	27176	Rpl7a		0.219377356	0.52442
11091	67942	Atp5g2		0.419876565	0.02441
11092	68554	Cebpzos	100505876	0.760131169	0.40962
11093	19044	Ppox	5498	-0.848283563	0.40878
11094	106672	AI413582	221491	-0.92017932	0.30663
11095	269639	Zfp512	84450	1.077104306	0.09041
11096	13663	Ei24	9538	0.173420003	0.99988
11097	67669	Hikeshi	51501	0.869105015	0.01322

11098	67392	833420G17R	375444	-0.495715004	0.61072
11099	11465	Actg1	71	-0.384092634	0.62133
11100	22690	Zfp28	140612	0.011948279	0.99988
11101	215789	Phactr2	9749	0.402153399	0.53617
11102	23918	Impdh2	3615	0.350947136	0.26422
11103	75785	Klhl24	54800	0.734263436	0.01786
11104	170787	Hdac10	83933	-0.450108932	0.76318
11105	11364	Acadm	34	-0.003637846	0.99988
11106	12632	Cfl2	1073	0.147852239	0.99988
11107	237411	Zfp938		-0.113699708	0.93247
11108	66902	Mtap	4507	-0.064630327	0.98477
11109	20849	Stat4	6775	0.620601593	0.6966
11110	100043133	130023H24Rik		0.34537237	0.41798
11111	320940	Atp11c	286410	0.282605192	0.9787
11112	16542	Kdr	3791	0.393893686	0.99988
11113	380768	Ccdc177	56936	-0.992387162	0.16208
11114	66155	Ufc1	51506	0.210910014	0.14883
11115	214642	Cped1	79974	0.462737154	0.62747
11116	67270	Mrpl42	28977	0.202227258	0.48143
11117	15893	Ica1	3382	0.826484889	0.3178
11118	66489	Rpl35		0.613735014	0.20782
11119	56047	Msln	10232	-0.596656702	0.47936
11120	12453	Ccni	10983	-0.845872261	0.27833
11121		2010204K13Rik		-0.244083775	0.97856
11122	69161	Manbal	63905	-0.376141923	0.69019
11123	338354	Zfp780b		-0.766885556	0.66381
11124	69260	Ing2	3622	-0.471483812	0.49209
11125	67144	Lrrc40	55631	-0.551329598	0.38367
11126	26417	Mapk3	5595	0.319509584	0.45457
11127	16561	Kif1b	23095	-0.375012701	0.45745
11128	22688	Zfp26		0.296776265	0.95519
11129	70405	Calml3	810	-0.01651229	0.99988
11130	16531	Kcnma1	3778	-0.722904474	0.57262
11131	18223	Numbl	9253	0.004914663	0.99988
11132	66184	Rps4l		1.477773013	0.00933
11133	72938	Hspb11	51668	0.852585723	0.01672
11134	214580	Pstk	118672	-0.162078027	0.99988
11135	70078	Nol7	51406	0.290042434	0.65789
11136	16828	Ldha	3939	-0.442677008	0.51243
11137	66461	Ptpmt1	114971	0.472917575	0.36204
11138	117171	110038F14Ri	65265	0.446726437	0.02714
11139	56367	Scoc	60592	0.261726875	0.99034
11140	671535	Parp10	84875	-0.669940391	0.41511
11141	74838	Naa15	80155	-0.468718833	0.58233
11142	30963	Hacd1	9200	0.359295112	0.90247
11143	22694	Zfp35		-0.04978778	0.99988
11144	668139	Gvin-ps7		1.725535294	0.00812
11145	320709	Tmem117	84216	-1.519816391	0.14614
11146	19942	Rpl27	6155	0.204416541	0.69812
11147	76179	Usp31	57478	-0.167428682	0.7681

11148	544717	190007I07Rik		1.323436413	0.00914
11149	52705	Krr1	11103	0.122790557	0.8004
11150	72027	Slc39a4	55630	1.118032165	0.02836
11151	26413	Mapk1	5594	-0.047887732	0.99988
11152	207958	Alg11	440138	0.007136514	0.99988
11153	80288	Bcl9l	283149	-1.413788999	0.10431
11154	73130	Tmed5	50999	0.022762572	0.99988
11155	223255	Stk24	8428	-0.991419658	0.15193
11156	232174	Cyp26b1	56603	-0.868649827	0.92577
11157	320916	Wscd2	9671	0.43504261	0.9519
11158	232987	B9d2	80776	-1.078567199	0.28502
11159	67824	Nmral1	57407	-0.075505042	0.99988
11160	319565	Syne2	23224	-1.120905646	0.25017
11161	330286	)630045J12R	57670	0.632373175	0.573
11162	20054	Rps15	6209	0.040172741	0.99988
11163	76633	Lrmda	83938	0.747389878	0.07409
11164	20826	Snu13	4809	0.481821764	0.02788
11165	382118	Zkscan7	55888	-0.642528648	0.40563
11166	20637	Snrnp70	6625	-0.077816277	0.99988
11167	13806	Eno1		-0.824354512	0.25491
11168	269966	Nup98	4928	-0.46528772	0.89045
11169	11761	Aox1	316	-1.660045998	0.08649
11170	231986	Jazf1	221895	-2.31706705	0.02722
11171	71765	Klhdc3	116138	-0.664616374	0.40085
11172	234582	Ccdc102a	92922	1.81115694	0.01372
11173		Gm10134		2.111263349	0.00565
11174	C230062I16Rik			1.090983723	0.00798
11175	76071	Jakmip1	152789	1.168877139	0.12634
11176	56517	Slc22a21		-1.360558293	0.10664
11177	30928	Zbtb18	10472	0.481836774	0.38014
11178	382236	Brwd3	254065	0.264491901	0.87025
11179	93876	Pcdhb5		-0.765070102	0.1361
11180	13063	Cycs		0.504487114	0.06983
11181	94281	Sfxn4	119559	0.511528642	0.52781
11182		Sp3os		0.94819562	0.61884
11183	18383	Tnfrsf11b	4982	0.364711218	0.48195
11184		Gm10136		-0.05309263	0.97675
11185	268291	Rnf217	154214	0.21044318	0.92858
11186	72554	Utp14a		-0.297228712	0.99788
11187	66121	Chchd1	118487	0.412808359	0.68016
11188	230596	Prpf38a	84950	-0.362155202	0.64648
11189	11778	Ap3s2	10239	0.064694144	0.99988
11190	67471	Gpatch1	55094	-0.224001123	0.96083
11191	236266	Alms1	7840	0.763059865	0.39478
11192	58804	Cdc42ep5	148170	-0.31655426	0.97856
11193	66812	Ppcdc	60490	-0.831159684	0.38995
11194	76072	Rnf183	138065	-0.191888417	0.86611
11195	14775	Gpx1	2876	0.599756046	0.05198
11196	107932	Chd4		-0.988205508	0.18448
11197	94249	Slc24a3	57419	2.829186132	0.00388

11198	66576	Uqcrh		-0.404624301	0.3796
11199	69956	Ptcd3	55037	0.488294197	0.49402
11200	192167	Nlgn1	22871	-2.247267851	0.16316
11201	66229	Rpl7l1	285855	0.033955084	0.99988
11202	12916	Crem	1390	0.320894679	0.05689
11203	93681	Zkscan8	7745	0.760240769	0.20863
11204	75221	Dpp3	10072	-0.114174106	0.99988
11205	18624	Pepd	5184	0.537913347	0.04592
11206	268936	Brpf3	27154	0.025180652	0.99988
11207	100041585	Amd2		-0.460053056	0.67178
11208	319192	H2ac19	8337	-1.632316723	0.17196
11209	14536	Nr6a1	2649	-0.980255509	0.27083
11210	108096	Slco1a5		-0.005210538	0.99988
11211	259277	Klk8	11202	0.343187773	0.62665
11212	74254	Gpn1	11321	0.153766368	0.80138
11213	224829	Trerf1	55809	-0.032864434	0.99988
11214	224170	Dzip3	9666	-0.221215876	0.7642
11215	320495	Ipcef1	26034	0.398134866	0.55816
11216	17827	Mtx1	4580	-0.191135183	0.95917
11217	14115	Fbln2	2199	-0.002395978	0.99988
11218	94219	Cnnm2	54805	0.155511113	0.74701
11219	23900	Hcst	10870	2.186400532	0.00564
11220	56738	Mocs1	4337	-0.727736957	0.79342
11221	26896	Med14	9282	0.301851767	0.10156
11222	219103	Cenpj	55835	0.850018675	0.05224
11223	68675	Fam172a	83989	-0.046014424	0.99988
11224	23807	Arih2	10425	0.018320899	0.99988
11225	21955	Tnnt1	7138	-0.829092701	0.28465
11226	216363	Rab3ip	117177	0.742844599	0.17336
11227	105722	Ano6	196527	0.364245407	0.26685
11228	52668	Ifi27		0.058733204	0.98214
11229	15267	H2ac18		0.400259676	0.68231
11230	12654	Chil1	1116		0.40878
11231	403178	Plcxd1	55344	-1.029759787	0.22364
11232	66071	Ethe1	23474	0.827979334	0.68504
11233	243374	Gimap8	155038	-0.541923514	0.62454
11234	232969	Zfp428	126299	-1.278522649	0.20222
11235	74096	Hvcn1	84329	0.286615168	0.99988
11236	72355	Cdpf1	150383	-0.192491664	0.92474
11237	21353	Tank	10010	0.121825449	0.99988
11238	71724	Aox3		-0.634467063	0.69275
11239	76707	Clasp1	23332	-1.236519323	0.20996
11240	69358	Lrrc51	220074	1.0188578	0.01357
11241	30954	Siva1	10572	0.60688444	0.27456
11242		mt-Rnr1		-0.251816264	0.86124
11243		mt-Rnr2		-0.530750854	0.519
11244	17716	mt-Nd1	4535	-0.543513394	0.6543
11245	17717	mt-Nd2	4536	-0.192420035	0.99988
11246	17708	mt-Co1	4512	-0.121097977	0.95504
11247	17709	mt-Co2	4513	0.206950622	0.991

11248	17706	mt-Atp8	4509	1.053548106	0.26955
11249	17705	mt-Atp6	4508	-0.113223623	0.99988
11250	17710	mt-Co3	4514	-0.243118684	0.84637
11251	17718	mt-Nd3	4537	0.047957524	0.99988
11252	17719	mt-Nd4	4538	-0.033842711	0.99988
11253	17721	mt-Nd5	4540	0.02023931	0.99988
11254	17722	mt-Nd6	4541	0.21840911	0.99988
11255	17711	mt-Cytb	4519	-0.147541963	0.99988
11256		NA		1.122832818	0.15838
11257		NA		-0.417871343	0.57125
11258		NA		3.025062638	0.00356
11259		NA		-1.282321919	0.44696
11260	17720	mt-Nd4l	4539	0.382370436	0.99988
11261	320165	Tacc1		0.231471343	0.56672
11262	223978	Cpped1	55313	-0.974204175	0.30475
11263	66077	Aurkaip1	54998	0.490713653	0.12182
11264	20148	Dhrs3	9249	-0.04640371	0.99988
11265	69116	Ubr4	23352	0.26573918	0.68574
11266	74326	Hnrnpr	10236	-0.29837828	0.57906
11267	100169	Phactr4	65979	-0.075734074	0.97395
11268	100038746	Gm1976		-1.663517871	0.15869
11269	77739	Adamtsl1	92949	0.719718087	0.37616
11270	70052	Prpf4	9128	-1.055941906	0.21926
11271	66440	Cdc26	246184	-0.657644132	0.45291
11272	20529	Slc31a1	1317	-0.209614545	0.77099
11273	338355	Fkbp15	23307	0.380294907	0.26685
11274	20530	Slc31a2	1318	0.345749261	0.68946
11275	E230001N04Rik			-0.626925575	0.53643
11276	2510046G10Rik			-0.300535887	0.5645
11277	233726	Ipo7	10527	0.357844661	0.0504
11278	66079	Tmem42	131616	0.238103444	0.43239
11279	215494	Pomgnt2	84892	0.361124518	0.96769
11280	76681	Trim12a		0.761666872	0.19222
11281	101706	Numa1	4926	-0.464088111	0.41775
11282		Gm12918		-0.661148283	0.43843
11283	242291	Bpnt2	54928	-0.243552056	0.64349
11284	83669	Wdr6	11180	-0.692118913	0.45347
11285	16625	Serpina3c		0.949508362	0.82473
11286	238393	Serpina3f		-0.627387772	0.57209
11287	75547	Akap13	11214	-0.44753457	0.38896
11288		Gm10263		0.066735315	0.99988
11289	77853	Msl2	55167	0.751290321	0.22111
11290	217682	Plekhd1	400224	-0.321499582	0.99988
11291	211978	Zfyve26	23503	-0.767840333	0.30475
11292	17252	Rdh11		-1.473426414	0.2103
11293	107885	Mthfs		0.693299808	0.05223
11294	94353	Hmgn3	9324	0.684429634	0.23715
11295	15289	Hmgb1	3146	-0.213839044	0.61015
11296	67070	Lsm14a	26065	0.393935411	0.65717
11297	233103	Garre1	9710	-0.266973989	0.64921

11298	226844	Flvcr1	28982	0.97301077	0.07326
11299	69504	Zfp932		-0.107974766	0.91547
11300	70381	Tecpr1	25851	-0.619532017	0.59909
11301	75516	Ttc32	130502	1.346432681	0.01209
11302	231863	Fbxl18	80028	0.009086281	0.99988
11303	74682	Wdr35	57539	0.190656449	0.11448
11304	330503	Gm5113		1.109609734	0.02469
11305	235320	Zbtb16	7704	-1.936127418	0.10434
11306	59095	Fxyd6	53826	2.044989455	0.00775
11307	69568	Vkorc1l1	154807	0.293000667	0.99947
11308	241322	Zbtb6	10773	0.631968331	0.1583
11309	24014	Rnasel	6041	-0.108010596	0.93937
11310	235050	Zfp810		-0.1409142	0.78109
11311	232855	Zfp772		0.398043461	0.55573
11312	26940	Ecsit	51295	-0.014494589	0.991
11313	23960	Oas1g		-0.392615294	0.60727
11314	17974	Nck2	8440	-0.705255834	0.82416
11315		Gm10184		-0.280254858	0.99988
11316	170938	Zfp617		-0.520181502	0.57665
11317	30843	Fbxl12	54850	0.659569108	0.03416
11318	231668	Vsig10	54621	-0.625731	0.39286
11319	71954	Suds3	64426	-0.451777205	0.43683
11320	231646	Myo1h	283446	-0.043438077	0.99197
11321	12237	Bub3	9184	0.8094108	0.12406
11322	20724	Serpinb5	5268	0.010711661	0.99988
11323	55927	Hes6	55502	0.217456409	0.05535
11324	20016	Polr1c	9533	0.495120743	0.21734
11325	16069	Jchain	3512	3.846646738	0.00388
11326	72322	Xpo5	57510	0.303780164	0.37805
11327	12829	Col4a4	1286	-0.009101668	0.99988
11328	66235	Eif1ax	1964	0.447400138	0.0616
11329	14296	Frat1		-0.603096383	0.42023
11330	15040	H2-T23		0.375670079	0.04425
11331	70701	Nipal1	152519	-0.144348653	0.95546
11332	15007	H2-Q10		-0.596434093	0.43843
11333	11837	Rplp0	6175	-0.419297415	0.56643
11334	12338	Capn6	827	-0.673993023	0.35364
11335	53412	Ppp1r3c	5507	1.40067425	0.00888
11336	54127	Rps28	6234	0.221643828	0.7594
11337	12168	Bmpr2	659	0.01905844	0.99988
11338	66309	Tmem128	85013	0.758381431	0.26302
11339	17089	Lyar	55646	-0.539605559	0.41188
11340	215201	Trmt2b	79979	0.940325758	0.01102
11341	56496	Tspan6	7105	0.565401396	0.30811
11342	240068	Zfp563		-0.155711557	0.94222
11343	70315	Hdac8		0.599251526	0.01886
11344	78321	Ankrd23		0.005526283	0.99988
11345	19082	Prkag1	5571	-0.428631663	0.55451
11346	214489	BC003965	283951	0.27685478	0.53317
11347		Gm10222		1.373227714	0.11714

11348	94227	Pi15	51050	-0.661882691	0.90308
11349	53619	Blcap	10904	-0.382441449	0.34335
11350	98932	Myl9	10398	2.239891286	0.0059
11351	74043	Pex26		-0.499705936	0.59585
11352	67067	Romo1	140823	0.215812977	0.8612
11353	211673	Arfgef1	10565	0.38365144	0.44151
11354	72459	Htatsf1	27336	-0.466355065	0.45575
11355	20743	Sptbn2	6712	-0.669669029	0.33679
11356	666532	Zfp991		0.631164127	0.02504
11357	66158	Rtl8a		0.818792698	0.76462
11358	240034	Zfp760		0.485859887	0.98575
11359	381066	Zfp948		-0.468245889	0.73084
11360	224585	Zfp160		-0.183402902	0.78464
11361	68705	Gtf2f2	2963	-0.042130506	0.99988
11362	70291	Mkrn2os	100129480	-0.175091679	0.83655
11363	380916	Lrch1	23143	-0.130903034	0.99988
11364	17356	Afdn	4301	-0.856083658	0.20884
11365	21454	Tcp1	6950	-1.190159952	0.14041
11366	99237	Tm9sf4	9777	-0.949219144	0.24952
11367	66570	Cenpm	79019	-2.031885456	0.00388
11368	76457	Ccdc134	79879	-0.142741434	0.9243
11369	78177	Ninl	22981	-0.449983996	0.55714
11370	668923	Zfp442		-1.096142334	0.46228
11371	104348	Zfp120		1.06152031	0.01433
11372	75597	Ndufaf2	91942	0.325224361	0.99788
11373	12837	Col8a1	1295	1.478423724	0.11034
11374	18693	Pick1		-1.092487005	0.35027
11375	16852	Lgals1	3956	1.308688199	0.00873
11376	16185	Il2rb	3560	0.613938786	0.24352
11377		Gm11808	7311	-0.814889406	0.15546
11378	219132	Phf11d		-0.313845979	0.66147
11379	71898	Apol9b		-0.877305902	0.28437
11380	232566	Amn1	196394	-1.23942243	0.19632
11381	278679	Apol7b		1.255219994	0.18253
11382	69596	Ap5s1	55317	-0.16143715	0.88463
11383	12616	Cenpb	1059	-0.567405547	0.34659
11384	208431	Shroom4	57477	0.388832474	0.99224
11385	207806	Usf3	205717	0.943423616	0.01364
11386	77006	Ddrgk1	65992	-0.677985644	0.32675
11387	11993	Aup1	550	-0.739296925	0.30907
11388	64704	Htra2	27429	0.51987339	0.2873
11389	13448	Dok1	1796	-0.246330715	0.75527
11390	93696	Chrac1	54108	-0.316512856	0.93487
11391	99100	Cep152	22995	-0.149484263	0.99988
11392	22290	Uty	7404	-0.829263672	0.24145
11393	67532	Mfap1a		-0.04363245	0.99988
11394	14707	Gng5		0.699732423	0.03001
11395	68910	Zfp467	168544	0.532843281	0.32222
11396	50918	Myadm	91663	0.705809912	0.81476
11397	72008	Zfyve19	84936	0.01269088	0.99988

11398	225594	Gm4841		0.392410556	0.34442
11399	74374	Clec16a	23274	-0.236239034	0.94808
11400	68794	Flnc	2318	1.051326329	0.32151
11401	67495	Tmem167b		0.288809892	0.42355
11402	277414	Trp53i11	9537	0.342434598	0.7777
11403	20226	Sars	6301	-1.032108088	0.23676
11404	53883	Celsr2	1952	-0.518607001	0.5679
11405	12953	Cry2	1408	-0.273911447	0.78002
11406	56742	Psrc1	84722	-1.030424071	0.3961
11407	20661	Sort1	6272	0.396061025	0.11535
11408	26442	Psma5	5686	0.544836601	0.02651
11409	16188	Il3ra		0.573248556	0.02702
11410	213945	Col28a1	340267	-0.729918532	0.57989
11411	109905	Rap1a	5906	0.79984634	0.01557
11412	229663	Csde1	7812	0.282559273	0.04849
11413	319190	H2bc21	8970	1.00073757	0.01236
11414	107701	Sf3b4	10262	0.437047992	0.06236
11415	229588	Gm128	54964	1.057692769	0.27733
11416	20341	Selenbp1		0.562281815	0.09243
11417	70737	Cgn	57530	-0.043867209	0.99988
11418	20342	Selenbp2		1.116158081	0.23404
11419	20823	Ssb	6741	-0.635402727	0.28119
11420	100303744	Sprr2a2		1.120957124	0.01186
11421	12748	Clk2	1196	-1.004704987	0.24427
11422	65111	Dap3	7818	-0.829077202	0.27366
11423	229524	Msto1	55154	-0.124447013	0.91657
11424	229521	Syt11	23208	0.410409127	0.03461
11425	241311	Zbtb34	403341	-0.17797141	0.90211
11426	627828	Gm5641		-1.059113949	0.10659
11427	68205	Urm1	81605	-0.09577903	0.88102
11428	26900	Ddx3y	8653	-0.491416558	0.52632
11429	26908	Eif2s3y		-0.964873647	0.28449
11430	12572	Cdk7	1022	0.447095512	0.23819
11431	18583	Pde7a	5150	0.103358883	0.99988
11432	229055	Zbtb10	65986	-0.257448663	0.86605
11433		Rps18-ps6	6222	0.057911256	0.99988
11434	75296	Cep43	11116	-0.507251308	0.43843
11435	13865	Nr2f1	7025	1.183995678	0.02642
11436	238722	Zfp72		0.2159389	0.99988
11437	238692	Zfp874a		0.303198376	0.14927
11438	235956	Zfp825		0.111955569	0.99988
11439	97863	Fam8a1	51439	-0.037738941	0.99988
11440	105352	Dusp22	56940	-0.483850675	0.62033
11441	319164	H2ac6		0.237490172	0.99788
11442	319161	H4c18		0.363296504	0.58464
11443	227867	Epc2	26122	0.241910931	0.85238
11444	17105	Lyz2		2.508780317	0.00388
11445	67226	Tmem19	55266	0.468747857	0.24103
11446	213326	Scyl2		0.855876129	0.01557
11447	70248	Dazap1	26528	-0.397199847	0.66558

11448	11735	Ank3		-0.685956713	0.3856
11449	72149	Strada	92335	-0.390203363	0.55682
11450	69129	Pex11g	92960	0.143884377	0.99988
11451	17118	Marcks	4082	0.517754747	0.21433
11452		Gm10275		-0.059753747	0.99988
11453	434179	Zfp975		0.084453696	0.76318
11454	239985	Arid1b	57492	0.030832459	0.99988
11455	26446	Psmb3	5691	0.000950671	0.99989
11456		Zfp125		1.590056869	0.00613
11457	67888	Tmem100	55273	1.197836298	0.18338
11458	76626	Msi2	124540	-0.408387713	0.52536
11459	100034251	Wfdc17		2.224131627	0.00558
11460	237886	Slfn9		-0.424699726	0.43843
11461	791303	Gm10277		0.259212471	0.6904
11462	116972	Tlcd3a	79850	-0.113213582	0.91975
11463	66395	Ahnak	79026	0.695903223	0.18426
11464	52892	Sco1	6341	-0.669940221	0.48307
11465	54396	Irgm2		1.178350483	0.0098
11466	245240	930111J21Rik2		0.359201975	0.6855
11467	52335	Atxn1l	342371	-0.122742421	0.85142
11468	74015	Fcho1	23149	-0.175654725	0.99988
11469	13716	Ell	8178	-0.180709041	0.92163
11470	434484	Sp140		0.011336401	0.99988
11471	109032	Sp110		-0.928156325	0.27619
11472	212326	Fam149a	25854	-0.530740018	0.60159
11473	14107	Fat1	2195	0.543111364	0.01143
11474	52065	Mfhas1	9258	-0.615480788	0.50319
11475	66706	Ndufaf3	25915	0.241140105	0.97395
11476	331026	Gmppb	29925	0.087892603	0.99988
11477	319742	Mpzl3	196264	-0.039162876	0.96083
11478	78934	930581F22Rik		-0.925058471	0.35764
11479	53356	Eif3g	8666	-0.617855886	0.32289
11480	672511	Rnf213	57674	-0.157243403	0.83299
11481	12443	Ccnd1	595	-1.571209137	0.12651
11482	101488212	Evi2		1.180750239	0.06658
11483	77613	Prss36	146547	-0.271914648	0.99386
11484	12340	Capza1		0.545192845	0.22797
11485		Gm20634		-0.73669759	0.36529
11486	69186	Tmem256		0.108519678	0.47201
11487	54710	Hs3st3b1	9953	0.591015342	0.27649
11488	259097	Olfr558	143503	-0.117693254	0.93113
11489	75212	Rnf121	55298	-0.515718411	0.62454
11490	16068	Il18bp	10068	0.304380178	0.99988
11491	12406	Serpinh1	871	0.714947925	0.2181
11492		9230112E08Rik		0.583415778	0.43981
11493	80889	Tlnrd1	59274	0.327923257	0.34138
11494	12739	Cldn3	1365	-0.74192916	0.21534
11495	71532	Fam217b	63939	0.836090975	0.11415
11496	14004	Chchd2	51142	0.202664622	0.60226
11497	244058	Rgma	56963	0.506644648	0.45897

11498	66647	Nsmce3		-0.332826675	0.64381
11499	21969	Top1	7150	0.068698561	0.99988
11500	226525	Rasal2	9462	-0.46236579	0.75679
11501	433938	Mn1	4330	1.320126427	0.12595
11502	433791	Zfp992		-0.197419451	0.85855
11503	433926	Lrrc8b	23507	-0.843426111	0.49686
11504	20668	Sox13	9580	-1.436514457	0.18095
11505	214253	Etnk2	55224	0.549029175	0.59408
11506	66825	Rnf186	54546	0.072050773	0.99988
11507	12399	Runx3	864	-0.882080051	0.85752
11508	65961	Utp3	57050	-0.150666591	0.86124
11509	71984	Sars2		-0.334738945	0.60423
11510	434156	Eid2b	126272	-0.314828809	0.88874
11511	73430	Zfp974		0.205085027	0.74801
11512	67809	Rmdn3	55177	0.416085274	0.99788
11513	72313	Fryl	285527	-0.041315591	0.99988
11514	100039968	Tmem35b	100506144	-0.068519192	0.99988
11515	227333	Dgkd	8527	1.275128103	0.01009
11516	245945	Rbm47	54502	-0.525064014	0.41147
11517	434128	Pnmal2	57469	0.468441666	0.58074
11518	56222	Cited4	163732	-0.624075967	0.51592
11519	321008	Zswim9	374920	0.455636664	0.78869
11520	381633	Gm1673	401115	-0.189091127	0.79469
11521	666048	Trabd2b	388630	0.524553069	0.31215
11522	227210	Ccnyl1		0.496991684	0.91173
11523	232801	Lilra5		1.592881566	0.00524
11524	75051	Ccdc173	129881	-0.384174428	0.67404
11525	242521	Klhl9	55958	0.951959815	0.18905
11526	68441	Rraga	10670	2.741200419	0.00388
11527	73122	Tgfbrap1	9392	0.623162585	0.02499
11528	107527	Il1rl2	8808	0.31632264	0.94143
11529	227746	Rabepk	10244	0.18084294	0.99988
11530	72429	Dnajc25		-0.082747565	0.87562
11531	100039781	Hrct1	646962	0.86166868	0.22585
11532	230075	Ndufb6	4712	0.306161823	0.09426
11533	240168	Rasgrp3	25780	1.421067465	0.00926
11534	224903	Safb	6294	-0.191907979	0.79983
11535	622675	Zfp827	152485	-0.155673227	0.99988
11536	328833	Treml2	79865	1.038936248	0.20884
11537	56351	Ptges3	10728	-0.344466533	0.50482
11538	28064	Yipf3	25844	-0.17721516	0.99988
11539	16478	Jund	3727	-2.976272782	0.04189
11540	75692	Nr2c2ap	126382	0.130096967	0.87777
11541	69479	700029J07Ri	441054	-0.576121299	0.62113
11542	20383	Srsf3	6428	-0.044248474	0.99039
11543	234094	Arhgef10	9639	0.529046509	0.29613
11544	75616	Smim15	643155	0.59333968	0.5642
11545	17951	Naip5		0.595740975	0.10257
11546	73132	Slc25a16	8034	0.224023471	0.47778
11547	449521	Zfp213	7760	-0.639925954	0.42704

11548	74149	Zfp946		-0.004645223	0.99988
11549	73233	Zfp942		0.089709258	0.99988
11550	235907	Zfp65		0.489895206	0.02615
11551	238693	Zfp58		1.133028874	0.3702
11552	72139	610044O15Rik8		-1.264152529	0.31922
11553	21841	Tia1	7072	-0.560530581	0.40878
11554	18489	Reg3b		4.176562704	0.00356
11555	237336	Tbpl1	9519	0.149199705	0.99988
11556	26408	Map3k5	10298	0.719461407	0.52278
11557	53602	Hpcal1	3241	2.068292799	0.00469
11558	65019	Rpl23	9349	0.423124336	0.03998
11559	69666	Psmg4	389362	0.372948235	0.31469
11560	13528	Dtnb		-2.038132781	0.09448
11561	72306	Zfp777	27153	-1.077663406	0.24894
11562		Nutf2-ps1	10204	-0.272784211	0.61465
11563	109218	Tmem139	135932	0.074950345	0.20884
11564	66477	Atp5md	84833	0.739388484	0.01411
11565	76302	Pcnp		-0.407436994	0.39167
11566	70021	Nt5dc2	64943	0.492555576	0.45436
11567	432720	Akr1c19		-0.833668441	0.76318
11568	100034684	Cstdc5		-3.280101175	0.02693
11569	20861	Stfa1		-0.80395267	0.22129
11570	381217	Fam189a2	9413	0.39086144	0.66381
11571	72307	510002D24Rik		0.199047022	0.17009
11572	12609	Cebpd	1052	-0.113547899	0.89783
11573	67160	Eef1g	1937	-0.330177802	0.53617
11574	70044	Tut1		0.825047918	0.22688
11575	23942	Mta2	9219	0.107550351	0.95747
11576	225898	Eml3	256364	0.126081254	0.9188
11577	19881	Rom1	6094	0.415144567	0.02856
11578	72727	B3gat3	26229	-0.033994506	0.99988
11579	14376	Ganab	23193	0.22304651	0.98477
11580	109077	Ints5	80789	-0.15683969	0.93349
11581	66276	810009A15Rik		0.680424073	0.13566
11582	107197	Uqcc3	790955	-0.074877279	0.99988
11583	225896	Ubxn1	51035	-0.106509944	0.94301
11584	68852	Lrrn4cl	221091	-0.901057669	0.46948
11585	14705	Bscl2	26580	-0.062655853	0.99988
11586	68693	Hnrnpul2		0.254435696	0.62222
11587	70387	Ttc9c	283237	0.386817345	0.04753
11588	67710	Polr2g	5436	-0.075411411	0.97147
11589	74478	Snx29	92017	-0.862205523	0.43347
11590	20603	Sms	6611	-0.290797324	0.66485
11591	12983	Csf2rb		1.137257184	0.01306
11592	12984	Csf2rb2		1.155546067	0.02642
11593	17972	Ncf4	4689	0.407566185	0.46713
11594	270624	Spin4	139886	-0.534321849	0.73177
11595	14853	Gspt2	23708	1.419949432	0.00837
11596	381598	610005L07Rik		-1.084956166	0.30315
11597	547150	820431F20Rik		-0.27204295	0.75953

11598	494504	Apcdd1	147495	-0.290527295	0.96083
11599	240261	Ccdc112	153733	-1.033282907	0.18424
11600	328949	Mcc	4163	-0.299226344	0.61858
11601	107065	Lrrtm2	26045	-0.400810577	0.28532
11602	268373	Ppia	5478	0.075135107	0.9084
11603	68655	Fndc1	84624	1.338417346	0.17584
11604	12449	Ccnf	899	0.198215379	0.99988
11605	621875	530040E14Rik		0.224977493	0.75253
11606	11727	Ang		2.029609564	0.00577
11607	22142	Tuba1a	7846	2.27407607	0.00406
11608	94088	Trim6	117854	0.622395345	0.31228
11609	21339	Taf1a	9015	-0.705978114	0.52974
11610	332397	Nanos1	340719	-0.401736554	0.91975
11611		Gm9008		-0.674879527	0.53617
11612	105651	Ppp1r3e	90673	0.274542594	0.99988
11613	239510	Phf20l1	51105	-0.314987	0.4406
11614	399603	Lratd2	157638	-0.477967423	0.38787
11615	217057	Ptrh2	51651	-0.955137376	0.31547
11616	20556	Slfn2		-0.589780642	0.38014
11617	22750	Zfp9	219749	0.606002272	0.53719
11618	66274	Lyrm9		-0.353658086	0.66016
11619	280668	Adam1a		0.3654972	0.43192
11620	232785	Zfp783		0.410709881	0.03021
11621		NA		2.68715953	0.00487
11622	66039	Tmem254a	80195	1.866696826	0.00558
11623		Gm10399		-0.980935724	0.23823
11624	100502825	Rpl37rt		0.129999279	0.78093
11625		Gm10401		0.620191717	0.02495
11626	66236	500011B03Rik		-0.314339069	0.69738
11627	381820	Smim10l1	100129361	0.145930193	0.99988
11628	100041734	930522L14Rik		0.920201528	0.22522
11629	77200	430403G16Rik		0.996697966	0.06261
11630		Gm10419		1.435196978	0.23493
11631	54137	Acrbp	84519	-0.551410539	0.45792
11632	14790	Grcc10	113246	0.235888019	0.89638
11633	9330160F10Rik			-0.851045863	0.39872
11634		NA		0.118906246	0.81989
11635	217882	Cep170b	283638	0.105681223	0.95071
11636	654820	530011O06Rik		-1.003537373	0.25218
11637	A530017D24Rik			-0.472221306	0.22556
11638	56353	Rybp	23429	0.542924968	0.05216
11639	14761	Gpr27	2850		0.40878
11640	100978	Nfxl1	152518	0.207806154	0.99988
11641	4933439C10Rik			-1.219095637	0.18448
11642	108167848	Gm12258		-0.220084458	0.84628
11643	77219	Ptgr2		1.052873078	0.17064
11644	26897	Acot1		-0.705596405	0.36752
11645	70237	Bhlhb9		0.267183624	0.99988
11646	245607	Gprasp2	114928	1.995112271	0.00564
11647	494468	Armcx5	64860	-0.860568944	0.31233

11648	53620	Vamp5	10791	0.32499079	0.05869
11649	668166	Zxdb		0.616207495	0.07228
11650	24067	Srp54a		0.267808084	0.11252
11651	574437	Xlr3b		-1.409073529	0.00934
11652	67048	Vma21		0.094608747	0.93548
11653	236848	Tmem185a	84548	0.653528448	0.26441
11654	269630	031425E22Rik		-0.241024358	0.69432
11655	67705	810058I24Ri	647087	-0.378184209	0.56548
11656	78619	Zfp449	203523	0.557343245	0.48755
11657		Gm10478		0.095396481	0.9483
11658	72326	500004C02Rik		-1.111131391	0.23493
11659	102954	Nudt10		-0.56944282	0.40438
11660	58242	Nudt11		0.767400836	0.40207
11661	C030034I22Rik			-0.592054183	0.72782
11662	110557	H2-Q6		-0.449341672	0.92014
11663	14964	H2-D1		0.510198711	0.36446
11664	16988	Lst1	7940	0.489927948	0.01871
11665	12268	C4b		0.79109195	0.49499
11666	14961	H2-Ab1	3119	2.354210209	0.00388
11667	14979	H2-Ke6	7923	0.734620627	0.01919
11668	328801	Zfp414	84330	-0.404437402	0.4706
11669	237412	Gm4924		-0.655012559	0.4362
11670	14570	Arhgdig	398	-1.547912705	0.03824
11671	106618	Wdr90	197335	-0.552477848	0.49281
11672	79059	Nme3	4832	0.208326914	0.71984
11673	193838	Eme2	197342	0.264707747	0.65178
11674	106489	Sft2d1		0.349655436	0.99448
11675	66832	Rsph3a		0.754212864	0.01937
11676	D730003I15Rik			0.615614066	0.70393
11677	67247	Mtarc2	54996	0.732849405	0.00984
11678	15951	Ifi204		0.969070644	0.30467
11679	226691	Ifi207		0.373404452	0.58127
11680	623121	Ifi213		1.827994076	0.00591
11681	623279	Dok6	220164	-0.513655861	0.30098
11682	225659	Cep76	79959	0.023707559	0.99816
11683	240327	Gm4951		0.428380414	0.34373
11684	329251	Ppp1r12b	4660	0.799896047	0.04592
11685	70425	Csnk1g3	1456	-0.515465265	0.57773
11686	71373	Prr16	51334	0.622716273	0.02147
11687	3222401L13Rik			0.157460939	0.99988
11688	93893	Pcdhb22	56121	1.974227353	0.00407
11689	73449	700066B19R	111064649	-1.593385544	0.22941
11690	68545	Ecscr		-0.025797412	0.99988
11691	381148	Prob1	389333	0.328860274	0.99988
11692	98314	D2hgdh	728294	0.528820372	0.0269
11693	66915	Cops9		0.539219323	0.02527
11694	C130036L24Rik			-0.095991729	0.99988
11695		Gm10553		1.237421029	0.00734
11696	66153	Fbxo36	130888	-0.986131227	0.32302
11697	19330	Rab18	22931	0.057459075	0.89193

11698	69368	Wdfy1	57590	-0.196124458	0.88398
11699	269198	Nbeal1	65065	-1.076942244	0.24007
11700	15528	Hspe1		0.733491871	0.01514
11701	241062	Pgap1	80055	-0.871115475	0.35311
11702	67513	Faap20	199990	0.663766663	0.23311
11703	242785	Klhl21	9903	0.794859538	0.01788
11704	114641	Rpl31		0.420910066	0.04036
11705	69928	Cenps		0.722316494	0.59106
11706	68421	Lmbrd1	55788	0.035309677	0.99988
11707	230757	730409E04R	127703	0.178094789	0.6635
11708	66938	Sh3d21	79729	0.841548424	0.18014
11709	78611	Btbd19	149478	-1.164487434	0.31851
11710	100087	Kti12	112970	0.610123693	0.0244
11711	230500	Efcab7	84455	0.148508817	0.99481
11712	320438	Alg6	29929	-1.071700199	0.2479
11713	12579	Cdkn2b	1030	1.17257332	0.12498
11714	627214	Insyn2a	642938	-0.750389329	0.51107
11715	233870	Tufm	7284	-0.228248494	0.99988
11716	434232	Iqck	124152	-0.185263698	0.98796
11717	100504346	Gm13304		2.276162174	0.00574
11718	20301	Ccl27a		-0.45223801	0.5388
11719	16157	Il11ra1		0.86740407	0.06694
11720		Gvin3		-0.293902681	0.68253
11721	214944	Mob3b	79817	-0.501100857	0.4372
11722	101488143	Hbb-bt		3.646033883	0.00356
11723	101700	Trim68	55128	0.491447589	0.24865
11724	56212	Rhog	391	0.803773591	0.19632
11725	14590	Ggh	8836	1.701539474	0.00576
11726	50500	Ttpa	7274	0.890741269	0.37299
11727	666737	632427E13Rik		-1.363621826	0.13249
11728	102058	Exoc8	149371	-0.480983477	0.45898
11729	71839	Osgin1	29948	-0.328515939	0.61368
11730	56690	Mlycd	23417	0.610223423	0.55816
11731	434197	Fam169b		1.465704685	0.00845
11732	66585	Snrnp40	9410	0.027228539	0.99988
11733	75744	Svip	258010	-0.720350624	0.35091
11734	109095	Rbm15b	29890	0.297464419	0.35347
11735	20208	Saa1		-4.488328868	0.04992
11736	78405	Ntf5	4909	-0.941218854	0.41617
11737	22121	Rpl13a		0.323771273	0.05578
11738	14204	Il4i1	259307	2.402246157	0.00405
11739	434341	Nlrc5	84166	-0.397326034	0.49105
11740	67607	Zfp788		-0.772410886	0.34716
11741	101835	AW146154		0.988460035	0.00984
11742	72287	Plekhf1	79156	0.281752786	0.99816
11743	229937	Znhit6	54680	-0.110283817	0.99988
11744	64661	Krtdap	388533	1.206940571	0.22582
11745	11522	Adh1		-0.069489462	0.95679
11746	68332	Sdhaf1	644096	-0.913975329	0.31851
11747	70604	Dnajb14	79982	-0.096583255	0.99988

11748		Gm10642		-0.589051419	0.81258
11749	12865	Cox7a1	1346	1.725280002	0.00566
11750	20733	Spint2	10653	-0.79492046	0.22364
11751	211556	Ap1ar	55435	-0.446219314	0.43555
11752	66498	Dda1	79016	-0.748587317	0.31829
11753	26365	Ceacam1		-0.260450037	0.82835
11754	232970	Phldb3	653583	-1.412583947	0.14407
11755	22756	Zfp94	7596	0.063975134	0.99988
11756	407795	Smim31	100505989	-0.314340526	0.59242
11757	244895	Peak1	79834	0.251484569	0.99988
11758	12659	Ovgp1	5016	0.161832854	0.81208
11759		AA386476		-1.363471801	0.03535
11760	232933	Ccdc61	729440	1.129175832	0.00414
11761	319430	C5ar2	27202	1.234761113	0.08052
11762	259300	Ehd2	30846	1.128329849	0.22871
11763	621080	AI429214	157773	0.063975134	0.99988
11764	319748	Zfp865	100507290	-0.004065022	0.99988
11765	73144	Mir100hg		-0.057094184	0.90454
11766	100042514	Sprr2a3		0.813195076	0.75957
11767	67860	S100a16	140576	-0.118934704	0.87409
11768	545539	Gm15417		0.132432934	0.99988
11769	67629	Spc24	147841	-0.709770398	0.34815
11770	72640	Mex3a	92312	-1.676736561	0.10147
11771	99889	Arfip1		0.304187407	0.32222
11772	102639598	Gm14296		0.619528499	0.59294
11773	69372	Mocs3	27304	0.940642145	0.01535
11774	69553	Ripor3	140876	1.251655073	0.0875
11775	68949	Zfas1		0.27302798	0.75897
11776	624866	Lekr1	389170	0.154143758	0.89867
11777	71004	931440P22Rik		-0.192753625	0.81099
11778	99371	Arfgef2	10564	-0.211627602	0.65746
11779	16658	Mafb	9935	1.384858111	0.00664
11780	633640	Tmem267	64417	0.05122214	0.99988
11781	67304	110070M22Rik			0.991
11782	20674	Sox2	6657	0.934269126	0.43622
11783	266692	Cpne1	8904	0.794209152	0.2021
11784	613262	BC029722	101410538	0.169328479	0.99988
11785	320352	Lrrc31	79782	-1.175323954	0.23823
11786	67204	Eif2s2	8894	-0.497663374	0.51243
11787	241732	Tspyl3		0.733573558	0.3235
11788	100038947	Sirpb1c		2.000779354	0.0072
11789	654459	Defb25	245937	-0.569448767	0.26317
11790	67917	Zcchc3	85364	-0.969287179	0.37299
11791	12995	Csnk2a1		-0.200872277	0.63543
11792	414758	Zfp950		-0.401579133	0.47207
11793	433485	Syndig1	79953	0.637907972	0.45154
11794	230991	Fndc10	643988	-0.63866343	0.1879
11795	21824	Thbd	7056	1.89207767	0.00622
11796	107368	Pdzd8	118987	-0.766668455	0.31612
11797	382423	Atxn7l3b	552889	-0.250395542	0.69841

11798	228730	Kiz	55857	-0.374085089	0.8586
11799	228715	Smim26	388789	1.058482272	0.00785
11800	319909	Ism1	140862	1.195944482	0.41716
11801	100038699	Gm10754		3.057165549	0.00356
11802	93765	Ube2n	7334	0.076551789	0.991
11803	373852	833422C13Rik		-0.438717062	0.33219
11804	54712	Plxnc1	10154	1.135899473	0.02372
11805	105171	Arrdc3	57561	-0.849442588	0.24307
11806	269356	Slc4a11	83959	-1.258441025	0.43545
11807	16434	Itpa	3704	-0.694499056	0.3285
11808	237436	Gas2l3		-0.855101273	0.14009
11809		Gm10762		-0.804696815	0.3193
11810	20170	Hps6	79803	0.343587813	0.52442
11811	100043424	Morrbid		0.611243056	0.06463
11812	73338	Itpripl1	150771	-0.782398126	0.38499
11813	630836	010315B03Rik		1.04228919	0.31523
11814	545291	Hpse2	60495	-1.839606431	0.224
11815		Platr25		1.452093098	0.08583
11816	77117	Zfp934		0.159362013	0.81947
11817	13025	Ctla2b		2.389505041	0.00414
11818	378702	Serf2		0.03354215	0.95337
11819	26385	Grk6	2870	0.773050967	0.01263
11820	329504	Lcmt2	9836	-0.705791073	0.66194
11821	15959	Ifit3		-0.14444074	0.99988
11822	240614	Ranbp6	26953	-0.256816383	0.77825
11823	72136	Chst14	113189	-2.182518126	0.12697
11824	100043272	Inafm2	100505573	0.259309137	0.91771
11825	72351	Ptar1	375743	0.042809902	0.99988
11826	67606	Fibin	387758	1.374771388	0.0707
11827	99003	Qser1	79832	-1.834534794	0.09173
11828	641340	Nrbf2	29982	0.099160795	0.99631
11829	107350	AW112010		1.006171702	0.1445
11830	14221	Fjx1	24147	-0.004728559	0.99988
11831	385658	Nxpe3	91775	-1.35786214	0.031
11832	381410	Zfp408	79797	0.59180592	0.2064
11833	67008	Yae1d1	57002	-0.37993101	0.53777
11834	12519	Cd80	941	0.154143758	0.82012
11835	29805	Znhit2	741	-0.758312397	0.29541
11836	381045	Ccdc58	131076	0.139506479	0.90834
11837	11702	Amd1		-0.94841905	0.24924
11838	77446	Heg1	57493	0.580170681	0.55018
11839	66311	Cenpw	387103	-0.438321901	0.75363
11840	78802	Ttc30a1		0.444732034	0.91657
11841	72421	Ttc30b		0.281127808	0.76772
11842		Mrpl23-ps1		0.357377392	0.40102
11843	215280	Wipf1	7456	0.66630318	0.21806
11844	107239	Carns1	57571	-0.042867005	0.9689
11845	621603	Aldh3b2		0.202898185	0.98454
11846	228003	Klhl41	10324	-0.570132278	0.48182
11847	60344	Fign	55137	-1.76609711	0.15001

11848	381990	Zbtb2	57621	1.478439998	0.00847
11849	67874	Rprm	56475	0.37044949	0.85339
11850	319817	Rc3h2	54542	-0.108998485	0.96528
11851	15423	Hoxc4		-1.370054272	0.18788
11852	77717	Smim41	113523638	1.555701636	0.00621
11853	100038570	Prcd		-0.052142	0.9483
11854	14269	Fnbp1	23048	1.29213187	0.00646
11855	227697	Dolk	22845	-0.646297575	0.34915
11856	68528	Smim6		0.670922245	0.01571
11857	52838	Dnlz	728489	0.060564812	0.8601
11858	380959	Alg10b		-0.024066804	0.98675
11859	66200	Commd6	170622	0.163846325	0.72577
11860	69684	Aarsd1		-2.213818416	0.12734
11861	6330403L08Rik			-0.231541996	0.94663
11862	103889	Hoxb2	3212	0.496264009	0.45745
11863	223649	Nrbp2	340371	-0.242969522	0.82504
11864	330217	Gal3st4	79690	-1.18225493	0.11881
11865	268469	Zfp652	22834	-0.390320062	0.68189
11866	223642	Zc3h3	23144	-0.504869997	0.89659
11867	110454	Ly6a		0.315274601	0.9848
11868	69227	Selenot	51714	0.380246755	0.04243
11869	109815	Selenos	55829	-0.958660245	0.19474
11870	114679	Selenom	140606	1.182090946	0.11472
11871	28042	Selenoi	85465	-0.207653326	0.75897
11872	26462	Txnrd2	10587	-0.672385898	0.33674
11873	27361	Msrb1	51734	-0.517672335	0.40862
11874	625249	Gpx4	2879	0.015922573	0.99988
11875	107585	Dio3	1735	0.579802766	0.07801
11876	20677	Sox4	6659	-1.132671135	0.16605
11877	22630	Ywhaq	10971	0.12657853	0.82658
11878	72657	Selenoh	280636	0.434850819	0.39298
11879		Trbc1		0.718317966	0.224
11880		Trbc2		0.642337216	0.57737
11881		Igkc		3.943530732	0.00356
11882		Ighg2c		3.21612219	0.00388
11883		Ighg2b		3.687710628	0.00388
11884		Ighg1		0.036771512	0.82381
11885		Ighm		3.933483004	0.01157
11886		Iglc2		4.306738101	0.00356
11887	19326	Rab11b	9230	0.042315497	0.90774
11888	621823	Psme2b		0.366690945	0.40947
11889		Gm12184		0.057333269	0.99988
11890		Rbm8a2		-0.557068428	0.59393
11891	12663	Chml	1122	-0.346711835	0.61594
11892	474332	Dnm3os		-0.148371335	0.92471
11893		Gm2000		-0.41563096	0.55194
11894	227615	Tmem203	94107	-0.335918796	0.58875
11895	242721	Klhdc7a	127707	-0.812989833	0.30927
11896	104103	Airn		-2.666347249	0.05357
11897	111241	Hmga1b		-0.297389794	0.70395

11898	14070	F8a		0.129526606	0.99988
11899	66125	Sf3b5	83443	0.984909362	0.01172
11900	100382	AW011738		-1.463416098	0.18573
11901	68859	Smim1	388588	-0.360003383	0.57386
11902	66118	Sarnp		-0.302899854	0.6635
11903	52468	Ctdsp2	10106	0.459618834	0.49313
11904	72273	Smim24	284422	-0.145541819	0.99988
11905	102115	Dohh	83475	0.045838984	0.99169
11906	73112	Abracl	58527	0.366946141	0.05109
11907	231002	Plekhn1	84069	0.693922317	0.33312
11908	100041677	Zfp984		-0.225440065	0.98675
11909	433801	Zfp268		1.077026205	0.00832
11910	68817	Ddi2		-0.785859796	0.32222
11911	230866	Emc1	23065	1.155360214	0.11974
11912	12176	Bnip3	664	-1.304358043	0.19913
11913	68920	110065P20R	127687	-0.621534299	0.5388
11914	208501	Ndufaf8	284184	0.06263073	0.9526
11915	66105	Ube2d3	7323	0.189326865	0.51037
11916	101604	:430018J23Rik		0.454603913	0.61648
11917	230696	AU022252	79078	-0.056293467	0.86157
11918	381546	Ccdc24	149473	-0.680502895	0.36549
11919	100042856	Gvin2		1.118297574	0.00972
11920	69066	810010H24R	284018	-0.608991704	0.57622
11921	242594	Fyb2	199920	-0.183670718	0.93069
11922	434219	Trim30c			0.991
11923	83796	Smarcd2	6603	0.181674209	0.94581
11924	67163	Ccdc47	57003	-0.198126039	0.83299
11925	19192	Psme3	10197	-0.658700614	0.33273
11926	68107	Cntd1	124817	0.415011849	0.76614
11927	28084	Vps25	84313	-0.997138178	0.17477
11928	100038347	Fam174b	400451	-0.645359253	0.40345
11929	244059	Chd2	1106	-1.569295509	0.1237
11930	192160	Casc3	22794	0.564328016	0.02058
11931	68634	Tm2d3	80213	0.123146211	0.81206
11932	217149	Cisd3	284106	0.454983866	0.80956
11933	319371	030028A08Rik		-1.968564486	0.08701
11934		Gm53		-0.022475923	0.99988
11935	68512	Tomm5		0.242252468	0.39734
11936	100041579	Gm20878		-0.330285552	0.991
11937	20555	Slfn1		0.709579884	0.60943
11938	233073	U2af1l4		0.44696682	0.55994
11939	72556	Zfp566		-0.04449485	0.99988
11940	14017	Evi2a		0.819935506	0.03459
11941	69126	Rbis	401466	0.160454718	0.52469
11942	414069	BC024978		-0.490108705	0.77644
11943	629378	Dact3	147906	0.768572165	0.24565
11944	276770	Eif5a		-0.445265233	0.48558
11945	69757	Leng1	79165	-0.262366125	0.61609
11946	319162	H2aw	92815	1.211247588	0.12036
11947	16145	Igtp		0.29504561	0.56884

11948	353208	Zfp931		0.332034039	0.39699
11949	665211	Gm14326		0.705137774	0.34241
11950		Gm14418			0.40878
11951	100043403	Gm14410		-0.015722478	0.99988
11952	100039123	Gm14295		-0.788924249	0.46788
11953	100043387	Gm14305		0.497002367	0.44726
11954	545490	Zfp973		0.876569371	0.0153
11955	100043381	Gm14308		-0.683035501	0.3837
11956	668039	Gm14434		1.8005414	0.10328
11957	626832	Gm6710		0.757732451	0.24506
11958	100504263	210418O10Rik		-0.378643799	0.81044
11959	100503949	Zfp965		0.876569371	0.0153
11960	627914	Gm4631		-0.203093092	0.93937
11961	270096	Mon1b	22879	-0.20130035	0.86638
11962	13480	Dpm1		0.235855814	0.74022
11963	15953	Ifi47		2.158350967	0.00502
11964	100039796	Tgtp2		-0.152118127	0.99788
11965	21822	Tgtp1		0.66716721	0.32913
11966	66589	Ube2v1	7335	-0.345533683	0.5971
11967	108143	Ak6		0.306426324	0.0513
11968	17952	Naip6		0.358526217	0.98475
11969	17948	Naip2		1.337885213	0.0096
11970	73167	Arhgap8		0.301566515	0.99988
11971	66255	Hsbp1l1	440498	-1.682028849	0.11159
11972	20335	Sec61g		0.375264043	0.1122
11973	72807	Zfp429		1.745777063	0.00634
11974	408065	Zfp456			0.991
11975	76933	Ifi27l2a		0.774071828	0.0177
11976	17067	Ly6c1		-0.139823927	0.95546
11977	16336	Insl3	3640	0.843613299	0.02459
11978	106068	Slc45a4	57210	0.769084332	0.01387
11979	211064	Alkbh1	8846	-0.353351347	0.43496
11980	19122	Prnp	5621	-0.221962173	0.99988
11981	380601	Fastkd5	60493	0.369690439	0.34195
11982	56461	Kcnip3	30818	0.495667365	0.6516
11983	102294	Cyp4v3		-0.216336613	0.99988
11984	77532	Jrkl	8690	0.767651316	0.50321
11985	66396	Ccdc82	79780	0.282447198	0.6904
11986	100101806	Srp54c		0.932825397	0.78002
11987	18861	Pms2		-0.810201087	0.33039
11988	66913	Kdelr2	11014	-0.456359838	0.53734
11989	319266	\130010J15Ri	148304	-0.513836497	0.47702
11990	53791	Tlr5	7100	-0.193451278	0.80583
11991	751865	Sap25	100316904	-1.206473535	0.18424
11992	22635	Zan	7455	-0.392756576	0.98124
11993	74855	Fam228a	653140	-0.091675823	0.8211
11994	74173	Rab10os		0.658554242	0.01837
11995	75339	Mphosph8	54737	0.246301141	0.45402
11996	19188	Psme2		0.234526281	0.96967
11997	269704	Zfp664	144348	0.310015907	0.33679

11998	12774	Ccr5	1234	2.991616136	0.00388
11999	C730034F03Rik			-0.586418472	0.62525
12000	28254	Slco1a6		-0.538610859	0.76721
12001	69549	310009B15R	388722	-0.025322336	0.99988
12002	56644	Clec7a	64581	1.262479163	0.01394
12003	80782	Klrb1b		1.377410464	0.00779
12004	56382	Rab9	9367	0.406277883	0.32127
12005	66226	Trappc2		-0.241603603	0.90379
12006	56441	Naa80	24142	0.007729581	0.99988
12007	667373	Ifit1bl1		-0.224764829	0.97748
12008	252837	Ackr4	51554	2.44176104	0.00447
12009	102633458	Gm11100	132200	0.498395957	0.10434
12010	17472	Gbp4		1.198639677	0.01988
12011	100169874	Gm11110		0.993199484	0.57989
12012	666468	Atg4a		0.524907352	0.18553
12013	73656	Ms4a6c		1.306150614	0.00733
12014	68089	Arpc4	10093	-0.349762225	0.35362
12015	100039707	Mthfsl		1.14142946	0.01001
12016	19982	Rpl36a	100529097	0.366876735	0.42673
12017	20448	St6galnac4	27090	-0.039726915	0.99988
12018		Gm14966		0.217514303	0.99988
12019	55981	Pigb	9488	0.93449319	0.04676
12020	195434	Utp14b		0.571067569	0.50868
12021	19349	Rab7	7879	-0.206291387	0.6177
12022	56390	Znrd2		0.078951834	0.99988
12023	69713	Pin4	5303	0.186721149	0.49408
12024	100042480	Nhsl2	340527	0.492211631	0.95681
12025	227696	Phyhd1	254295	1.03330559	0.01078
12026	59024	Med12	9968	-0.5149904	0.42249
12027		H2-T10		0.702089764	0.65255
12028	68316	Apoo	79135	0.130660962	0.25833
12029	22764	Zfx	7543	0.101396903	0.99988
12030	19240	Tmsb10		0.716382662	0.39112
12031	14999	H2-DMb1		1.143895394	0.04091
12032	227157	Mpp4	58538	0.545045468	0.03579
12033	231123	Haus3	79441	0.04641344	0.99988
12034	224703	Marchf2	51257	-0.220225549	0.80284
12035	100502940	Colca2	120376	0.19496774	0.99988
12036	59003	Maea	10296	0.492653842	0.02374
12037	235312	C1qtnf5	114902	0.291006811	0.61916
12038	433016	Cstdc4		0.501667021	0.56068
12039	474156	Zbtb9		0.909757337	0.25336
12040	109346	Ankrd39	51239	0.009563147	0.99988
12041	72124	Seh1l	81929	0.078050934	0.99988
12042		NA		0.974187125	0.18375
12043	67248	Rpl39	6170	0.835275511	0.01186
12044	328778	Rab26	25837	-0.769179605	0.19123
12045	67923	Eloc	6921	0.180389672	0.67104
12046	652925	Tmem243	79161	-0.713831585	0.39196
12047	72481	610203C22Rik		-0.228910282	0.73082

12048	68165	Fdx2		-0.858949922	0.35722
12049	77777	Ulbp1		-0.649779058	0.64614
12050	21647	Dynlt2a1		-0.668623922	0.1471
12051	16635	Klra4		0.60961826	0.0269
12052		Gm14239		-0.111280001	0.99988
12053	75202	Spaca6	147650	-1.82502292	0.18294
12054	245305	230307C23Rik		-0.758680414	0.33147
12055	100042889	920021L13Rik		-0.038142334	0.99356
12056	100042948	Gm15760		-0.097204307	0.77575
12057		Gm9385		0.027535984	0.90192
12058		Hmgb1-ps6		-0.400903161	0.76991
12059		Gm12846		-0.668760295	0.32874
12060	97114	H3c15	333932	-0.679626203	0.42626
12061		Gm12568		0.647692055	0.80354
12062		Rps6-ps4		-1.625005444	0.13106
12063		Hmgb1-ps3		-0.639223106	0.37299
12064	67739	Slc48a1	55652	0.048323152	0.99988
12065		Gm13423		-0.458752324	0.67762
12066	18722	Gm15922			0.991
12067		Sms-ps		-2.250510524	0.05625
12068		Gm12216		-0.601240431	0.5956
12069		Rpl17-ps5		-1.500047089	0.1403
12070		Gm12097		-0.823882588	0.59203
12071		Rpl17-ps8		0.162597384	0.97685
12072		Gm15720		-0.972505563	0.48662
12073		Gm15596		-0.369340442	0.63077
12074		Gm12250		-0.206842542	0.94963
12075	100042584	Gm16523		-0.783320079	0.07945
12076		Actr3-ps		-1.465185466	0.19898
12077	12223	Btc	685	0.007629007	0.99988
12078		Gm12183		-0.321821149	0.60819
12079		Gm5909		-0.819772135	0.32302
12080		Gm15387		-0.936095394	0.26928
12081		Gm13803		0.017176676	0.99632
12082		Gm14537		-1.146493856	0.25305
12083		Gm11889		-1.188935924	0.24007
12084		Gm5844		-1.052343426	0.29158
12085		Gm8172		-2.715282792	0.02372
12086		Gm2810		0.05891419	0.96672
12087	56464	Ctsf	8722	0.303302216	0.58023
12088		Vcp-rs		-0.973261067	0.29038
12089		Gm11826		-1.448937984	0.10623
12090		Llph-ps2		-1.97378683	0.12498
12091		Gm15542		-0.952233996	0.42699
12092		Gm13340		-1.507598435	0.19839
12093	667423	Gm8624		0.097061345	0.15771
12094		Hmgb1-ps5		0.256140536	0.74317
12095	76508	Ube2d-ps		-0.148845291	0.90295
12096		Gm13341		-0.06456984	0.99024
12097		Gm12346		-1.308244757	0.1639

12098		Gm14049		0.410434862	0.21806
12099		Gm15937		0.186494667	0.99988
12100		Gm6136		-1.415183378	0.18019
12101		Gm15710		-1.928465502	0.07496
12102	77411	Esrp2	80004	-0.316091418	0.63429
12103		Gm6451		0.281844119	0.7419
12104		Gm6142		-1.536626714	0.07546
12105		Znf41-ps		-0.347414287	0.46469
12106	66177	Ubl5		0.067465417	0.99988
12107		Gm2568		-1.213975627	0.32652
12108		Mir142hg		0.455942644	0.97242
12109	641454	830444B04Rik		-0.140300556	0.99988
12110	627268	Gm15232		0.480052273	0.48431
12111		B230312C02Rik		2.005515484	0.11879
12112		A730011C13Rik		0.555546232	0.30845
12113	668158	Ccdc85c	317762	-1.50889159	0.14962
12114		C630043F03Rik		-0.18394969	0.93095
12115		Gm17477		0.375385692	0.98217
12116		Gm830		1.618672794	0.12452
12117	100503572	Bbip1	92482	-0.315074825	0.67404
12118		Gm11655		-1.7178079	0.1426
12119		Gm16279		0.414730574	0.04245
12120	100504166	933421O10Rik		0.267010809	0.15601
12121		Abhd11os		0.259777753	0.991
12122	100126229	Gm12758		0.664786758	0.53617
12123		B930095G15Rik		-1.937573881	0.07318
12124	100042498	Mir22hg		-0.342721774	0.71846
12125	100041286	Snhg15		0.014678237	0.99988
12126		Gm6542		-2.138476393	0.06049
12127	100503272	Gm11423		-0.261506573	0.80058
12128	71319	933439K11Rik		0.161297611	0.85388
12129		Nr6a1os		-0.257288469	0.77247
12130	74038	Brip1os		-0.055159691	0.99988
12131	69004	330418K02Rik		0.145690118	0.84237
12132	399101	Snhg3		0.100081039	0.99988
12133		Gm11537		0.309313412	0.99988
12134		Taco1os		-0.745563688	0.03637
12135	74597	833418N02Rik		-1.006900471	0.19341
12136		Gm11525		-0.157050275	0.72435
12137	115489452	430018G15Rik		0.681184312	0.03554
12138		Gm12940		-0.546036237	0.48431
12139		Gm12254		-0.816020257	0.34636
12140	109163	010003L21Rik		0.397647076	0.82741
12141		Snhg17		-0.540019568	0.48268
12142	103012	Firre		-0.938367558	0.37507
12143	100040736	Foxd2os		-0.283915755	0.71444
12144	66602	Oip5os1		-0.217398533	0.59009
12145	68789	Trmt61b		-2.033108494	0.12932
12146		Gm13166		0.080931855	0.99988
12147		Gm13568		-0.29620386	0.61364

12148	320486	430078I02Rik		-0.401712708	0.89642
12149		Gm11346		0.676269281	0.20565
12150	74487	430405H02Rik		-1.518024713	0.26528
12151		8430419K02Rik		-0.67925148	0.40525
12152		Gm12333		-0.735672433	0.33812
12153	217708	Lin52	91750	0.828254163	0.22927
12154	353310	Zfp703	80139	0.021720159	0.99988
12155	629945	Cep112it		0.115480195	0.9519
12156		Gm4285		-0.574347494	0.48185
12157		Grin1os		0.198215379	0.99988
12158	100503595	Gm13003		0.173486121	0.91657
12159		Tnfsf13os		0.572763268	0.87985
12160		Gm15832		-0.187061659	0.70773
12161	73906	833417C18Rik		-1.448784814	0.19839
12162		Gm16196		-0.819702868	0.27456
12163	546143	Ccpg1os	145788	0.183351047	0.76344
12164	71489	Bcas3os1		-1.09075636	0.29633
12165		2810433D01Rik		-0.378050516	0.9232
12166	100039864	Snhg12		0.218153842	0.78722
12167		Gm12122		-0.961725054	0.48543
12168	70123	Nbdy	550643	0.222566334	0.65156
12169		2700033N17Rik		0.432849978	0.98653
12170		Gm13270		0.867727753	0.59536
12171	78878	Ftx		1.048717843	0.01074
12172	102633437	Chrna1os		-2.017025698	0.10222
12173	15397	Hoxa11os		-0.928061939	0.28751
12174	14910	t(ROSA)26Sor		-0.187097165	0.89111
12175		9330162012Rik		-1.060093374	0.25605
12176		Gm15856		0.146288126	0.94107
12177		Mypopos		1.008701643	0.17323
12178	100415901	Gm13648		-0.807937657	0.28757
12179	630146	Cd101	9398	-0.216540489	0.99988
12180		Gm15500		0.339567059	0.03897
12181	100038514	Gm11837		0.828972453	0.72294
12182	69964	810403D21Rik		-0.104776237	0.92238
12183	70957	Stamos		-0.398320195	0.57084
12184	381524	Mexis		-0.67796403	0.85302
12185	100125929	610009E02Rik		-0.084988962	0.97921
12186		Gm16201		0.415082462	0.37891
12187	664994	Isoc2a		-0.063048595	0.99988
12188		Gm15675		0.389004579	0.65115
12189	69221	410006H16Rik		0.22669689	0.99988
12190	433944	Tbx3os2		-0.477013058	0.54315
12191	76972	Snhg20		-1.204793463	0.21978
12192		Gm15883		-0.681906372	0.45689
12193		Gm13594		-0.988331346	0.32281
12194	74931	930481A15Rik		-0.215263408	0.89642
12195		Gm15859		0.889964022	0.31922
12196	329735	933431E20Rik		-0.181545852	0.99988
12197	620695	Gm13889	387763	1.053227598	0.00952

12198	100504178	Dhrs13os		-0.974315104	0.26896
12199	102631889	Gm16638		-0.01912358	0.99988
12200	100043636	AI662270		2.266784501	0.00388
12201		BC064078		-0.92404095	0.35677
12202	71445	530601H04Rik		-0.285014124	0.57988
12203	327744	130307A14Rik		0.042450857	0.95476
12204		Gm43213		-0.019412881	0.99988
12205	72386	610035D17Rik		-0.432288232	0.5424
12206	68576	Lamtor5	10542	0.510120419	0.3957
12207		D330023K18Rik		1.194560888	0.01033
12208		Gm13556		0.66770201	0.49436
12209		Gm12953		2.035818849	0.03651
12210	69120	810021B22Rik		-0.444488744	0.55061
12211	67394	4930404I05Rik		0.658642644	0.87037
12212		Junos		0.511111348	0.6583
12213	621976	Tmem170b	100113407	0.65840453	0.38173
12214		Gm15541		0.467140899	0.95246
12215	73652	210408F21Rik		-0.489123797	0.65003
12216		Kantr		-0.005209741	0.99988
12217	100040298	Gm15501		-0.138419041	0.991
12218	102635783	Gm11266		-1.469788079	0.05099
12219	70489	730405O15Rik		-0.724304294	0.47207
12220		Gm13822		0.074240237	0.99988
12221	75060	930506C21Rik		0.075987266	0.99988
12222	100504616	Gm15910		-1.555502261	0.03066
12223		2900089D17Rik		-0.143832146	0.92973
12224	66456	810001G20Rik		-2.095407806	0.05327
12225		Gm13561		-1.782633354	0.0678
12226		Actb-ps1			0.991
12227	69749	Epb41l4aos		-0.415931391	0.55711
12228	102638408	Gm16174		0.672897328	0.05111
12229	105734734	Gm11715		0.645613948	0.57989
12230	56707	Zfp111		0.190698267	0.98387
12231	100504154	Gm15050		-0.242971181	0.82101
12232		A230059L01Rik		1.061344503	0.33673
12233		Gm13414		-2.040951617	0.06977
12234	69784	500009L16Ri	387882		0.991
12235		B230398E01Rik		0.113172031	0.96811
12236	109299	Tmem250-ps	90120	-0.465698242	0.45862
12237	100503890	Pet100	100131801	0.001374292	0.99989
12238	102632231	Gm2245			0.991
12239		Gm16025		0.030055047	0.99988
12240	12587	Mia		-0.371937433	0.99988
12241	69583	Tnfsf13	8741	0.862750583	0.13328
12242	613123	Ugt1a8		1.152482262	0.01165
12243	12050	Bcl2l2	599	-0.160519781	0.92471
12244	108148	Galnt2		0.848787431	0.01557
12245	78603	230216N24Rik		-0.810282318	0.29003
12246	494448	Cbx6		-0.919248184	0.29157
12247	100044509	Tgfbr3l	100507588	-2.264484099	0.01222

12248	72500	Ier5l	389792	0.134456719	0.90466
12249	666244	Tmsb15b1		-0.215263408	0.92702
12250	53881	Slc5a3	6526	-0.059103982	0.94097
12251	105245924	Gm16136		-0.958124907	0.29398
12252	320292	Rasgef1b	153020	0.493014168	0.0616
12253		Gm16536		0.580260445	0.18553
12254	75710	Rbm12	10137	0.187150927	0.99988
12255	192192	Shkbp1	92799	-1.256013773	0.18294
12256	381287	530032D15Rik		-1.440255064	0.35231
12257	14356	Timm10b		-0.173794986	0.99988
12258	100036521	Umad1		-0.036821814	0.99988
12259	320119	Rps6kc1	26750	0.065585825	0.90297
12260		Etohd2		0.11238197	0.99988
12261	15247	Mfsd14a	64645	0.932151725	0.00933
12262	68556	Uckl1	54963	0.582503098	0.02461
12263	12045	Bcl2a1b		2.441509494	0.00407
12264		Gm4117		-2.170513239	0.05646
12265	18725	Pira2			0.991
12266	66191	Ier3ip1		0.355886916	0.04757
12267	100642166	Gm15446		-0.247420912	0.65599
12268	16205	Gimap1	170575	1.412824007	0.00819
12269	14426	Galnt4		0.256743041	0.99988
12270	72699	Lime1		0.236979867	0.80173
12271	58230	Rnf8		0.111459734	0.99988
12272	51795	Srpx	8406	0.345621009	0.98119
12273		AI480526		0.41765601	0.62665
12274	140810	Ttbk2	146057	0.049943084	0.95477
12275	73474	Snhg9		-0.531961123	0.89187
12276	105886298	Cmc4	100272147	1.486388024	0.10244
12277	268281	Shprh	257218	0.3952803	0.37845
12278	224836	Usp49	25862	-0.399779803	0.79865
12279	394432	Ugt1a7c		1.289693775	0.08463
12280		Gm10177		0.578995101	0.51447
12281	22186	Uba52		-0.322229055	0.67995
12282	102632	Acad11	84129	0.571947037	0.02788
12283	232406	BC035044		1.556225979	0.00664
12284	73407	Tepp	374739	-0.19570799	0.99988
12285		4930524O07Rik		-0.831688278	0.66988
12286	213119	Itga10		-0.378617394	0.90772
12287	407243	Tmem189		-0.65887461	0.58461
12288	14962	Cfb		1.951377243	0.00449
12289	14533	Bloc1s1		0.499172064	0.10153
12290		Gm4524		1.971929107	0.00569
12291	211151	Churc1	91612	-0.049476218	0.99988
12292	108112	Eif4ebp3	8637	0.629922854	0.02267
12293	74319	Mettl23	124512	-0.205235358	0.85226
12294	100040462	Mndal		0.418891634	0.36827
12295	212728	Tarbp1	6894	2.151769367	0.02798
12296	665615	Smr2l			0.991
12297	77124	130221H12Rik		0.678099794	0.04832

12298	77994	Mir99ahg		-1.404401646	0.28001
12299	67647	930523C07R	9674	0.025137292	0.99988
12300	71683	Gypc	2995	0.509356374	0.54854
12301	20643	Snrpe		0.226009392	0.41707
12302	21778	Tex9	374618	-0.04101946	0.99988
12303	72958	Zfp493		0.981659585	0.17719
12304	381823	Apold1	81575	1.225840733	0.01052
12305	57294	Rps27		0.045011332	0.99988
12306	546611	Klhl33	123103	-0.279880138	0.74485
12307	17904	Myl6		0.420819179	0.02605
12308		Gm17134		0.519571308	0.77644
12309	68052	Rps13	6207	0.177044812	0.54432
12310	52372	D6Ertd527e		0.268480781	0.50631
12311	24071	Synj2bp		-0.334070184	0.62421
12312	240328	830016B08Rik		-0.018342822	0.99988
12313	72123	Ccdc71l	168455	0.473415465	0.04768
12314		Gm17251		-1.094137103	0.06719
12315	434215	Lrrc32	2615	0.011394072	0.99988
12316		Gm17655		-0.142439619	0.75763
12317		Gm17300		-0.205001008	0.94808
12318		9330020H09Rik		-0.741674076	0.54745
12319	100039139	Ccdc152	100129792	1.280148353	0.01315
12320		Gm17334		-0.495828526	0.61021
12321		Sardhos		0.16445876	0.91149
12322	73569	Vgll3	389136	0.346102261	0.38017
12323	108934	Smim13	221710	0.691096144	0.25718
12324	58521	Eid1	23741	-0.722027144	0.27937
12325	665033	Col6a5	256076	0.944089938	0.71387
12326	97122	H4c14		0.784697962	0.01943
12327		2610021A01Rik		-0.81162675	0.54314
12328		Gm17066		-0.366106746	0.6841
12329	56692	Lamtor3	8649	0.75986022	0.01548
12330	66167	Tma7	51372	0.583834041	0.50511
12331	69206	2010016I18Rik		-1.07439545	0.12682
12332	208285	Cyp4f17		0.139875131	0.88822
12333	670558	H60c		-0.551131267	0.44156
12334		Gm17092			0.40878
12335	66373	Lsm5	23658	0.36008392	0.88561
12336	236451	Phf11b		-0.243935543	0.87025
12337	15013	H2-Q2		-0.665091297	0.38611
12338		Gm17189		-1.381340925	0.28128
12339	66272	Cox16	51241	0.17214463	0.99988
12340	66300	Inafm1	255783	-0.479865875	0.56232
12341		Rpl36-ps12		-0.491543416	0.48927
12342	56550	Ube2d2a	7322	0.097336525	0.93077
12343	100233175	Gon7		1.310386645	0.02204
12344		Gm9844	9168	0.184901063	0.99988
12345		Rps2-ps10		-0.355359585	0.66333
12346	193740	Hspa1a		0.860809715	0.33357
12347		Gm17354		0.63054104	0.26769

12348		B930036N10Rik		-0.931656978	0.29356
12349	414101	130317F20Rik		-1.035940048	0.02595
12350		Gm17139		0.545463392	0.13729
12351		Gm4540		-0.378050516	0.742
12352	100310872	Dynlt1a		0.00679311	0.99988
12353	102636903	Gm17039		0.540633	0.02036
12354	100040608	Fancf	2188	-0.521910548	0.41058
12355	100303738	030025P21Rik		0.705950963	0.39339
12356	72716	Gm45351		-1.633725503	0.06627
12357	68763	110038B12Rik		-0.45234511	0.52896
12358	66961	Neat1		-0.2721475	0.73182
12359		Cdk3	1018	0.785361169	0.10438
12360		BB365896		-0.059660617	0.99988
12361		Galnt2l		-0.951442374	0.37402
12362	72289	Malat1		-1.228063736	0.15536
12363	434178	Zfp141		0.114729721	0.99988
12364	81845	Gpank1	7918	0.574862817	0.26813
12365	56771	Med20		0.05799899	0.84131
12366		Gm20427		-1.93098668	0.04534
12367	69269	Scnm1	79005	-0.904946775	0.29838
12368		9130230N09Rik		-0.493372404	0.75897
12369		NA		-1.391862297	0.19839
12370		6330562C20Rik		-0.915068189	0.10146
12371	231798	Lrch4		2.293996264	0.00538
12372		Gm20659		0.336297742	0.63555
12373	613254	AA465934		-0.120670544	0.83547
12374		Gm20708		0.897041512	0.40878
12375	75614	Rab26os		1.174753501	0.00924
12376		Gm20632		-1.622612702	0.17237
12377		Gm20707		-0.095982022	0.991
12378		A730085K08Rik		0.533492751	0.02492
12379		Gm20628		-0.337808391	0.54528
12380	66892	Eif4e3	317649	-0.184996277	0.74037
12381	67945	Rpl41		-0.065057985	0.94274
12382		Gm20712		-0.471439113	0.61196
12383		Gm20716		-0.501996553	0.45457
12384	19054	Ppp2r3d		-0.724719029	0.46868
12385	665769	Gsdmcl2		0.9783929	0.27147
12386	67952	Tomm20	9804	-0.018348339	0.99988
12387		Gm379		-0.20694849	0.86369
12388	208715	Hmgcs1	3157	0.05883656	0.99988
12389		NA		1.926125386	0.08542
12390	52898	Rnasek		-0.250435693	0.99988
12391		NA		1.122832818	0.15838
12392	210583	Gm4767		0.048172913	0.99988
12393		G530012D18Rik		1.52428736	0.0991
12394	667118	Gm38394	100381270	-1.757932474	0.08304
12395	77652	Zfp955a		-0.166464118	0.99631
12396	19291	Purb	5814	-0.46349385	0.41015
12397	100040944	Gm3055		0.389476324	0.2927

12398		NA		0.48025502	0.11064
12399	18829	Ccl21a		4.29183883	0.00356
12400	621580	Gm21953			0.991
12401	68195	Rnaset2b		0.985894016	0.00859
12402	319171	H2ac24		-1.513613098	0.14438
12403		NA		2.249778261	0.2553
12404	72465	Zfp131	7690	-0.331678829	0.68231
12405	19653	Rbm4		-0.307434196	0.96733
12406	100041979	Gm3604			0.40878
12407		Igha		3.91532252	0.00356
12408	240514	Ccdc85b	11007	0.231837243	0.99988
12409	171211	Edaradd	128178	0.315861013	0.2856
12410	319622	Itpripl2	162073	0.415531922	0.30926
12411		Gm21781		-0.532451409	0.70012
12412	100044193	Gm20939		0.598364486	0.03001
12413	100303732	Zfp967		-0.366687841	0.97103
12414	240064	Zfp799		-0.275126008	0.55704
12415	211623	Plac9a	219348	-0.658845199	0.24307
12416	240066	Zfp870		0.780649561	0.19457
12417	212276	Zfp748		-0.339666703	0.69657
12418	668225	Fignl2	401720	0.301264425	0.78929
12419	67464	Entpd4		-0.584803645	0.48422
12420	57741	Noc2l	26155	-1.040073027	0.26415
12421	100861753	Gm21188		1.175244895	0.0155
12422	100040531	Dynlt1f		-0.087937123	0.99988
12423	100037283	Rnaset2a		0.28503412	0.59597
12424	320832	Sirpb1a		0.218086484	0.99988
12425	70045	610528A11R	387695	0.05288333	0.99988
12426	64009	Syne1	23345	-1.004598012	0.3222
12427	27973	Vkorc1		0.24497339	0.06331
12428	97487	Cmtm4	146223	-0.046611498	0.991
12429	69709	Ptrhd1	391356	-0.271161438	0.99988
12430	14958	H1f0	3005	-0.989293772	0.20198
12431	432995	Smim22	440335	0.253996542	0.99988
12432	21648	Dynlt1b		-0.06686068	0.98653
12433	231004	Samd11	148398	-0.155503626	0.99988
12434	102902673	Gm21992		0.496323971	0.26236
12435	240038	Zfp994		0.260571537	0.9429
12436	12581	Cdkn2d	1032	0.10499969	0.82884
12437	100503386	Tpbgl	100507050	-1.633774001	0.05215
12438	213332	Mfsd4b4		-0.690505116	0.52716
12439	449000	Zfp960		0.151126476	0.76594
12440	331188	Zfp781		0.507395454	0.19632
12441		Hmgb1-ps9		0.140161914	0.79198
12442	16912	Psmb9	5698	0.805076748	0.04624
12443	170942	Gm47283		0.759355303	0.75823
12444		Tmem181b-ps		-0.034049441	0.99988
12445	73610	Zfp433		0.786722194	0.83479
12446	100043468	Zfp955b		1.201752604	0.00821
12447	100043772	Zfp850			0.991

12448		A330023F24Rik		-0.707805469	0.28343
12449	76642	700113A16Rik		0.322361391	0.95106
12450	671336	Gm9530		0.780677092	0.18087
12451	100616095	Snhg18		0.656084176	0.01627
12452	105246071	930509G22Rik		0.140070376	0.99988
12453		Gm26881		1.078996438	0.29549
12454		Gm10827		-0.205403104	0.89373
12455		Gm26789		-0.76505319	0.35314
12456		Gm17435		-0.869494032	0.33249
12457	100043793	Gm4651		-2.439053718	0.01247
12458		Pvt1		-0.227443727	0.76585
12459		Gm17491		-0.18394969	0.95337
12460	72012	600020E01Rik		0.661121335	0.1716
12461		Gm26759		0.431640202	0.57754
12462	414085	330151L19Rik		-0.412351132	0.87025
12463	78445	330013E15Rik		0.794160241	0.32289
12464	100038755	Gm9917		-0.159503305	0.99988
12465	105246278	230354K17Rik		-1.06366729	0.40577
12466	319760	130020L05Rik		-0.626056232	0.991
12467		A530020G20Rik		-1.495959848	0.09132
12468		Gm26885		1.086341318	0.01196
12469		Gm3839		-1.02965671	0.2744
12470	78329	310010J17Rik		-0.48514606	0.77922
12471		Cep83os		-0.310621256	0.58464
12472	100504029	Gm16740		0.786130506	0.23823
12473		Gm4890		-0.015967041	0.99988
12474	67194	700038G22Rik		1.168848829	0.28572
12475		Snhg5		0.331485226	0.90175
12476		Gm17690		1.847141248	0.00646
12477		Gm26716		1.322692317	0.00812
12478		Gm27029		1.030669516	0.69432
12479		4933416M07Rik		-0.417382237	0.26928
12480	100043489	300002E11Rik		-0.129253894	0.74248
12481		Hmgb1-ps8		-0.048110889	0.99988
12482	100502742	700007L15Rik		0.843613299	0.01681
12483	319887	030030I06Rik		-0.438264798	0.91901
12484	21944	Tnfsf12	8742	0.330761846	0.42751
12485	100038591	420014N23Rik		-1.501601168	0.22199
12486	170763	Zfp87		0.733196961	0.01661
12487		2310001H17Rik		-0.255760529	0.99988
12488		6720427I07Rik		0.223093942	0.41583
12489	100042165	Thoc2l		-0.814773682	0.30315
12490	66263	810014B01Rik		0.062187361	0.34681
12491		AU020206		0.247267958	0.40019
12492		Gm26670		0.952801229	0.36835
12493		Gm26782		0.897138907	0.39184
12494		Gm17529		-1.094553374	0.46908
12495		Gm26631		1.170517824	0.0066
12496		Rian		0.550766008	0.41661
12497	73635	Ptges3l	100885848	0.368053264	0.46868

12498		4732487G21Rik		-0.165369863	0.69859
12499		9930014A18Rik		-2.238826708	0.02634
12500		2610037D02Rik		-0.610496878	0.53888
12501	70252	Jpx		-0.950478709	0.50892
12502	76133	230400D17Rik		0.142734124	0.7594
12503	619719	430590A07Rik		-1.918329688	0.10067
12504	319711	230029C05Rik		1.130970159	0.10537
12505		Dleu2		0.223001001	0.99481
12506	100502764	Gm16617		-2.731649179	0.06605
12507		Mirt1		-1.138452315	0.21806
12508		Gpr137b-ps		-0.132141845	0.83528
12509		2310015A10Rik		-0.64664686	0.6037
12510	100503823	Gm16973		-0.453590784	0.52785
12511		Gm4673		0.537672185	0.36062
12512	102639566	Ptgs2os2		1.499696593	0.04907
12513		6030442K20Rik		1.239438363	0.13004
12514	100503199	430416N02Rik		0.239634658	0.99052
12515	67188	700046G09Rik		-0.621159725	0.32983
12516		Gm17259		0.767400836	0.48067
12517	329217	Panct2		1.839946541	0.00566
12518		Gm26813		-0.967083192	0.26526
12519		E530011L22Rik		-0.304663919	0.72438
12520		Mir205hg		-1.828037399	0.08363
12521		5031434O11Rik		-1.200888563	0.25769
12522		Gm26801		0.351225499	0.99988
12523	66752	933404O12Rik		0.920469552	0.36767
12524		A730020E08Rik		-1.521904848	0.13408
12525		Gm26981		-1.721631763	0.1583
12526		Gm7909		-0.159896972	0.85978
12527	545261	Bvht		1.296130553	0.09251
12528	668218	Bin2	51411	1.136389611	0.01186
12529	78748	Rassf10	644943	-1.318678693	0.11368
12530	66381	Rnf113a2		-0.062908369	0.99988
12531	223648	Ccdc166		0.792750006	0.1958
12532	268301	Sowahc	65124	-0.68214927	0.47219
12533	71619	Arl14	80117	0.982972828	0.0136
12534		Snhg6		1.912245872	0.00544
12535	68193	Rpl24	6152	-0.298959335	0.63692
12536		Gm28040		1.049577401	0.03651
12537		Lockd		-0.023650958	0.99988
12538	66364	Pigbos1	101928527	0.221217602	0.67674
12539		Gm28043		-0.294346276	0.99058
12540		Mrip-ps		-1.236956438	0.08807
12541	239217	Kctd12	115207	1.639020871	0.00558
12542		Commd1b		0.849304654	0.01514
12543	68982	500015A07Rik		0.939102294	0.45989
12544	433466	Jmjd7	100137047	0.071454969	0.99988
12545	629016	Zfp953		2.534403558	0.00405
12546	319830	500004A13Rik		-0.683907427	0.20545
12547	226351	Tmem185b	79134	0.962067773	0.38013

12548		NA		0.086150775	0.89847
12549		Gm27219		-0.964939688	0.52678
12550	100039596	Tcf24	100129654	-0.24324578	0.99267
12551	223922	Atf7		-0.587504984	0.37858
12552		NA		-0.930527437	0.26511
12553		NA		0.086150775	0.89847
12554	383435	Ms4a14	84689	-0.493266318	0.0515
12555		Gm3830		0.304521982	0.73572
12556		Gm28370		-0.239410268	0.84526
12557	102443350	Xndc1	100133315	0.149606441	0.8323
12558	319149	H3c4		-1.874030311	0.06849
12559	108168734	900093K20Rik		0.478450257	0.66758
12560		1700052K11Rik		0.344026251	0.66845
12561	73729	Zfp383	163087	-0.793550452	0.50319
12562		BE692007		2.008649609	0.00574
12563	69513	700030C10Rik		1.441659185	0.19008
12564		Hmgb1-rs16		-0.31450492	0.69557
12565		Gm29650		-0.150763524	0.89789
12566	100503178	810013P06Rik		0.475324748	0.50862
12567		Gm28872		0.643849014	0.06971
12568		2810402E24Rik		-0.954220542	0.37959
12569	12047	Bcl2a1d		1.694972076	0.00561
12570		B130024G19Rik		-0.437316051	0.64387
12571	100503842	410022M11Rik		1.141254236	0.26041
12572		Gm28959		1.150498161	0.25305
12573		Gm28439		0.258752493	0.991
12574	434233	Ppp1ccb		-1.839407787	0.03748
12575	67170	610306M01Rik		0.628186241	0.65664
12576	100504637	Gm20324			0.991
12577		Gm28557		-0.309385674	0.91207
12578		Gm28417		-1.067437493	0.24007
12579		Gm28791		-1.450257466	0.19885
12580		Hand2os1		1.558759284	0.00652
12581	73600	700120C14Rik		0.023993941	0.99988
12582	115488195	Gm20379		0.239369179	0.99988
12583	69798	810044D09Rik		-0.516170375	0.62612
12584		2010320M18Rik		-1.158062234	0.2351
12585		1700084E18Rik		0.513394653	0.49893
12586	16353	Snhg14		-1.378647506	0.2064
12587		Gm10925		0.606320958	0.78946
12588		2810454H06Rik		-2.122794598	0.08807
12589		Gm10138		-0.272224308	0.60364
12590		Gm29666		-1.13686313	0.35658
12591		Gm28875		-1.08685456	0.28465
12592		Gm28437		0.086345118	0.93453
12593	15436	Hoxd4		0.989954316	0.27799
12594	629159	700008J07Rik		0.402581349	0.97699
12595		Gm29216		-0.000598733	0.99988
12596		2810405F17Rik		-0.409193492	0.5701
12597	69781	600010M07Rik			0.991

12598		Gm20342		-0.634924773	0.41035
12599		Kcnq1ot1		-1.186594282	0.14213
12600	381792	310040G24Rik		1.474790808	0.00577
12601	73571	700096K18Rik		0.510563119	0.251
12602	75758	130401M01Rik		0.706092737	0.20987
12603		Gm28438		-0.061108024	0.99988
12604	69170	Chaserr		0.361713898	0.11194
12605	432964	Iqank1	642574	-0.270670411	0.64943
12606	12044	Bcl2a1a		2.012891799	0.00388
12607	100504501	Gm20257		-0.567815972	0.40878
12608		Gm28661		0.754543902	0.05755
12609	66391	Zbtb11os1		0.195980896	0.99988
12610		Gm38056		-0.514494713	0.4147
12611		Gm37474		1.258112212	0.11801
12612		Gm34342		0.124173903	0.95272
12613		Gm38190		0.163563841	0.92258
12614		Gm37376		-0.835674576	0.28792
12615		9530018H14Rik		-0.458372617	0.72035
12616		Gm38387		0.225396498	0.82835
12617		Gm36936		0.059730697	0.93661
12618	26904	Sh2d1b1		-0.085435956	0.99988
12619		Gm37465		-1.783857466	0.0843
12620	93724	Pcdhga12	26025	-0.136962932	0.99988
12621		Gm38197		0.492121501	0.03554
12622		BC085271		-0.338221005	0.65346
12623		6430511E19Rik		-0.219258202	0.90897
12624		Gm37570		-1.344581841	0.2234
12625		Gm37968		0.402343302	0.38767
12626		A930004J17Rik		0.059075028	0.96307
12627		A130048G24Rik		-0.01651229	0.99988
12628		Thap6		-0.648023788	0.47417
12629		Gm37760		-0.063981183	0.99988
12630		5830444F18Rik		1.044819826	0.10222
12631	635702	Naaladl2	254827	-0.035530325	0.991
12632		C130023A14Rik		-0.488473805	0.52716
12633		Gm37084		-0.104756098	0.99988
12634	347740	Norad		0.585394612	0.0283
12635		Gm37943		-0.058272426	0.97566
12636		Gm19918		-0.591790608	0.41569
12637		9430034N14Rik		-0.91133068	0.26012
12638	70579	Zc3h11a		-1.19809765	0.25415
12639		Gm8797		-0.350095149	0.99988
12640		Gm37124		0.202050482	0.99463
12641		Gm37305		0.806428926	0.42548
12642		Gm38120		0.168568163	0.95669
12643		4833445I07Rik		-0.068262904	0.94349
12644		Gm37303		-0.722871951	0.2266
12645		Gm37745		0.764310004	0.44858
12646		C130012C08Rik		0.429527489	0.4776
12647		Gm37090		-0.771378953	0.54027

12648		Gm9874		-0.596151833	0.89186
12649		Gm38287		-0.532587465	0.59585
12650		Gm37519		-0.169145281	0.89517
12651		Gm37915		-0.988471629	0.22364
12652		3110080O07Rik		-1.745877594	0.12903
12653		Gm37274		-0.641338269	0.44456
12654		Gm38157		-0.427561174	0.4602
12655		6720464F23Rik		0.355475624	0.71868
12656		Gm38220		0.373189149	0.99988
12657		Gm37706		-0.01494005	0.98901
12658		Gm37621		-1.373053481	0.11006
12659		Gm37531		0.546194811	0.14788
12660		Gm38020		-0.199110375	0.89543
12661	226654	Tstd1	100131187	2.313425936	0.00463
12662		Gm17530		0.422007604	0.68231
12663		Gm37612		-1.033094988	0.41641
12664		Gm37606		0.41949058	0.48431
12665		Gm37249		0.056216053	0.99988
12666		Gm37452		1.123374678	0.01024
12667		Gm37010		1.00459928	0.01569
12668		Gm36963		0.414116938	0.08466
12669		Gm37678		-0.994349893	0.19425
12670		BC055308		-1.532389815	0.21546
12671		Gm37677		-0.376419103	0.56979
12672		Gm20045		-1.435813754	0.10588
12673		4833412K13Rik		0.014518002	0.99988
12674		5330406M23Rik		-0.132429326	0.98452
12675		Gm37954		0.294027633	0.94263
12676		Gm37024		0.237146219	0.86369
12677		Gm9517		-0.205828212	0.53394
12678		Gm38104		-1.527581974	0.15816
12679		Gm38248		-0.019412881	0.99988
12680		Gm37401		0.352250227	0.5122
12681		Ighd		1.737935037	0.01569
12682		A130071D04Rik		0.487047838	0.03056
12683		Gm38042		-0.58347887	0.43813
12684		Gm37906		0.199579641	0.99672
12685		C130089K02Rik		-0.968603359	0.28666
12686		Gm38244		-1.85295838	0.1497
12687		Gm37033		-0.640245821	0.11001
12688		Gm37962		-0.031086006	0.99988
12689		Gm37254		-0.306061605	0.75897
12690		A430027C01Rik		-1.426883033	0.10102
12691		Gm37422		-0.079396098	0.35314
12692		Gm37423		0.952636447	0.11684
12693	58176	Rhbg		-1.64388107	0.21598
12694		Gm37255		-0.973809962	0.35982
12695		Gm38211		0.063795556	0.86915
12696		Gm37335		0.101575111	0.92014
12697		Gm37333		-0.574203131	0.89467

12698		Gm43857		0.330665051	0.03875
12699		Gm43185			0.991
12700		Gm43774		0.099748507	0.99988
12701		Gm43290		-0.784612419	0.49987
12702		Gm43814		0.367081627	0.57005
12703		Gm42815		-0.373812418	0.78255
12704	100702	Gbp6		0.491094034	0.08171
12705		Gm43138		-0.202622454	0.80808
12706		Gm43162		-0.672400902	0.31284
12707	433637	Gm5547		2.23422887	0.00407
12708		Gm42639		0.682855771	0.06623
12709		NA		0.086150775	0.89847
12710		Gm43023		0.234296351	0.66369
12711	69895	Snhg8		0.637987726	0.07583
12712		Gm43445		-0.607951544	0.45896
12713		Gm43210			0.991
12714		Gm43547		-0.874433321	0.4703
12715		Gm43423		-2.352054724	0.08038
12716		Gm43336		-0.240873354	0.73374
12717		NA		0.086150775	0.89847
12718		Gm42595		0.113906334	0.99802
12719		Gm43668		-0.944877619	0.53653
12720		Gm43778		1.468642784	0.29807
12721		Gm43362		-1.092487005	0.31453
12722		Gm42716		0.871733371	0.40182
12723		Gm43304		-0.294542532	0.8433
12724		Gm43696		-1.178051235	0.22496
12725		Gm42515		-0.143436442	0.75697
12726		Gm43692			0.40878
12727		Gm43379		-1.000139511	0.2812
12728	229898	Gbp5	115362		0.991
12729		E430021H15Rik		-0.179049645	0.88819
12730		Iglc3		2.724038454	0.06111
12731		Gm5853		-0.174056084	0.99988
12732		Gm42876			0.991
12733		Gm43737		-0.741668495	0.42417
12734		Gm43305		1.346166101	0.35934
12735		Gm43088		-0.441061095	0.58253
12736		Gm43292		0.48025502	0.05245
12737		Gm43435		0.259746047	0.8691
12738	115490131	Gm42517	112441426	-0.430786862	0.87037
12739		Iglc1		4.593512186	0.00356
12740		Gm43260		-0.448105692	0.8717
12741		Gm43628		-1.603855543	0.15447
12742		Gm43254		0.525044716	0.7856
12743		Gm42432		2.136279848	0.00356
12744		Gm42993		-0.624855428	0.72329
12745	433855	AI506816		-0.198542245	0.6985
12746		Gm43307		0.425610922	0.98435
12747		Gm42826		-0.510780538	0.38154

12748		Gm43672		-2.447776569	0.00683
12749		Gm43609		-0.382039846	0.47971
12750		NA		-2.760085969	0.13729
12751	19850	Snord3a		-1.094542049	0.38298
12752		Gm43430		-1.597228127	0.14638
12753		Gm20768		-0.526421776	0.40494
12754		Gm43727		-0.014731028	0.99988
12755		C230096K16Rik		-0.94378582	0.2797
12756		Gm19409		0.488720281	0.54694
12757		Gm43848		0.517211805	0.99988
12758		Gm42992		-0.999784494	0.17093
12759		Gm43361		-0.52660193	0.39859
12760		E330034L11Rik		0.009154066	0.99988
12761		Gm42690		-0.674177405	0.06849
12762		Gm42893		0.444260548	0.60641
12763		Gm43259		-0.498372359	0.43869
12764		Gm42702		0.875385099	0.49171
12765		Gm43111		-1.038752884	0.26928
12766		Gm9403		0.295091752	0.7695
12767	102638436	Gm38509		-0.38530311	0.7324
12768		Gm42841		-1.278797064	0.18992
12769		Gm43378		-0.552785678	0.38767
12770		Gm43813		-0.0097711	0.99988
12771		Gm43534		0.255540735	0.89867
12772		Gm42729		0.189846562	0.99988
12773		Gm42846		-0.035581902	0.97196
12774		C530043K16Rik		-1.933389008	0.10995
12775		Gm43166		-0.329383867	0.63555
12776		Gm42546		-0.336280118	0.78943
12777	105180375	Tmem265		-0.871908338	0.38966
12778		Gm42798		0.309946341	0.85944
12779		Gm43792		-1.72461919	0.09589
12780		Gm20559		0.335509182	0.55259
12781		Gm43275		-1.875608007	0.06983
12782	353342	Peg13		-0.708191453	0.36092
12783	71752	Gtf3c2		0.045445192	0.9979
12784		Gm43681		0.479226159	0.46745
12785		Gm20186		-1.568023151	0.155
12786		Gm43460		1.087156607	0.41569
12787	66845	Mrpl33	9553	-0.228997084	0.80792
12788	70036	Dancr		-0.077290246	0.9519
12789	319982	930430L01Rik		-0.171341539	0.87534
12790		Gm43085		-1.350823721	0.23715
12791		Gm45495		0.212975692	0.22329
12792		Gm42547		1.133443576	0.13509
12793	106264	610012G03R	152217	-0.749743654	0.31453
12794		6720475M21Rik		-1.436699844	0.09744
12795		4930528J11Rik		1.270649084	0.00504
12796		Gm42715		-0.828851234	0.4031
12797		C030017G13Rik		-0.633002004	0.41621

12798	67278	Gm42742		0.798563432	0.05988
12799		Gm43343		-1.307389259	0.1667
12800		Gm42466		-0.434050944	0.91589
12801		Gm43811		-0.453999748	0.37805
12802		Gm42728		0.696620411	0.70348
12803		Gm42507		0.315218513	0.98415
12804		Gm43268		-0.610330299	0.40691
12805		5930420M18Rik		-1.935977369	0.06385
12806		Gm42576		-0.638323604	0.52331
12807		Gm42736		0.027535984	0.99988
12808		Gm43637		0.225451638	0.99988
12809		Gm10461		-0.042314041	0.99988
12810	17527	Mpv17	4358	0.184003352	0.69432
12811		Gm43788		-1.883200861	0.10334
12812		Gm43775		-0.939224873	0.27678
12813		Gm42732		-0.120165777	0.77779
12814		Gm43461		0.272757852	0.99988
12815		Gm43660		-0.639386024	0.38077
12816		AI839979		-0.495794062	0.14324
12817		Gm40309		-0.644231554	0.35708
12818		Gm43588		-0.990864459	0.14414
12819		Gm4366		-0.523282564	0.49727
12820		Gm43323		-0.087093248	0.94107
12821		5730507A11Rik		0.120003896	0.90192
12822	58894	Zfp862-ps		0.666835869	0.23425
12823		E330037G11Rik		-0.743678359	0.35161
12824		Gm44103			0.40878
12825		Gm44041		-1.191574285	0.09604
12826		D530018E20Rik		-0.773658054	0.38023
12827		Gm44220		-1.46624037	0.26041
12828		Gm44044		1.010752367	0.01057
12829	68204	900060B14Rik		-1.407666734	0.19569
12830		Gm38910		0.189098454	0.9519
12831	100039027	Gm2011		-1.007493412	0.31233
12832		9530085L11Rik		-0.199110375	0.99988
12833		Gm44371		1.696517866	0.00565
12834		Gm44164		-0.621992415	0.40271
12835		Gm44250		0.544326938	0.43066
12836		Gm10388		1.344980669	0.01089
12837		Gm44432		-0.187005102	0.991
12838		Gm43980		-1.414899526	0.19299
12839		D830050J10Rik		-1.072540774	0.22688
12840		Gm44597		-0.676652448	0.39396
12841		Gm45193		-0.508307811	0.84054
12842		Gm44130		-0.432660227	0.90372
12843		Gm44153		-1.235489221	0.22629
12844		Olfr1372-ps1		-0.558623872	0.42581
12845		Gm44101		-0.558501902	0.6516
12846		Gm44292		0.009574195	0.99988
12847		Gm44552		-1.663737575	0.02934

12848		Gm44950		-0.049981893	0.89
12849		Gm45205		-0.862866247	0.66213
12850		Gm44509		0.736414602	0.47856
12851		Gm45053		-0.73384737	0.32724
12852		Snhg1		0.204551005	0.60338
12853		Gm45051		2.322098673	0.00438
12854		Gm44510		0.431244562	0.35657
12855		AV356131		-0.754574831	0.37805
12856		Gm44836		-2.372032654	0.01749
12857		Gm45203		0.593809171	0.47778
12858		Gm44987		0.533332213	0.25871
12859		Gm44639		-0.186905453	0.99344
12860		Gm8319		-1.469009296	0.19191
12861		Gm20274		-0.039726915	0.99988
12862		Particl		1.433177285	0.14045
12863		Gm10616		0.654445625	0.40878
12864	102637087	Gm33989		-0.197408851	0.89968
12865		Gm44694		-0.330116729	0.43477
12866		Gm44985		0.62190783	0.0616
12867		Gm44777		0.003993278	0.99988
12868	5430434F05Rik			-1.245011987	0.23834
12869	E230020D15Rik			-0.018983131	0.99988
12870		Gm44775		-0.206424149	0.88959
12871		Gm31024		-0.208967681	0.78781
12872		Gm45120		0.897264053	0.57361
12873		Gm36371		-1.721039594	0.02287
12874	102634450	Gm32031		-0.853000049	0.25731
12875	4930431P19Rik			-0.201171854	0.84891
12876		Gm44829		-1.289657032	0.27917
12877		Gm44709		-0.483960737	0.4478
12878	414103	230032D23Rik		0.172431656	0.99988
12879	9530078K11Rik			-0.559630742	0.22798
12880	15469	Prmt1	3276	-0.993131582	0.22382
12881	233033	Samd4b	55095	0.151322761	0.99988
12882		Gm45236		0.229994263	0.08151
12883		Gm45223		-0.509704117	0.50896
12884	18226	Nup62	23636	0.002323906	0.99989
12885		Gm44763		0.578199856	0.28283
12886	9330162G02Rik			-2.754628482	0.00773
12887		Gm38843			0.991
12888		Gm45413		-1.800248767	0.1573
12889		Gm3912		-0.107751979	0.95893
12890		Gm31166		-0.915399755	0.27661
12891		Gm45338		-1.637903078	0.06961
12892		Gm45643		-0.076978029	0.99988
12893	50497	Hspa14	51182	0.901920624	0.01589
12894		Gm45407		0.572336172	0.52015
12895		Gm45507		-1.005533021	0.35122
12896	67064	Chmp1b	57132	0.044015716	0.99988
12897	72544	Exosc6	118460	1.953707221	0.01844

12898		Gm45629		-0.601266566	0.0986
12899		Gm45343		-0.918122634	0.61803
12900		Gm45251		1.134260129	0.01215
12901		Gm4247		-0.346335413	0.9383
12902		4831440D22Rik		0.178313892	0.80638
12903	74744	830408C22Rik		-1.466857281	0.17456
12904	105246846	Gm42067		-0.099062766	0.96083
12905		Gm45501		-1.07737065	0.41147
12906	109169	Igip	492311	0.910007391	0.14231
12907	207728	Pde2a		0.653706962	0.34856
12908	14256	Flt3l		0.924376262	0.12821
12909		Gm45412		0.062187361	0.92702
12910	108168395	Gm45871		-0.224001123	0.80304
12911		Gm45311		0.448031124	0.82658
12912		Gm45716			0.40878
12913	75479	700012D14Rik		-0.100018425	0.96949
12914	378466	Gm10033		-0.04276642	0.99988
12915		Gm35572		0.135175629	0.99988
12916		Gm29773		0.900493423	0.01496
12917	102635552	Gm32856		0.10375872	0.99988
12918		Gm45733		-1.424461153	0.15505
12919		Gm20735		0.589906299	0.22535
12920		BC049987		-0.123447857	0.82965
12921		Gm47079		-0.033585954	0.99988
12922		Gpx4-ps2		-0.659027801	0.31228
12923		Gm49628		0.159733649	0.99988
12924		Gm48840		0.509008611	0.29324
12925		Gm9856		-0.625139416	0.52465
12926	66387	Nudt8		-1.470476127	0.11881
12927		Gm47234		-0.852669092	0.5185
12928	100504586	Gm20300		0.372790495	0.60464
12929		9430081H08Rik		-0.927746218	0.15314
12930		Gm49338		-1.350149348	0.19611
12931		Gm48275		0.714115638	0.4679
12932		5330432J10Rik		0.139478836	0.99988
12933		Gm47126		-0.25926965	0.991
12934		Gm48604		-0.43847476	0.8949
12935		Gm34006		-0.450704073	0.86932
12936		Gm47205		-0.815330129	0.27467
12937		Gm31410		-0.79596869	0.19789
12938		Gm47445		-0.011863835	0.99988
12939	100503185	Btbd8		-1.104630075	0.40374
12940		Gm47483		-0.384310843	0.96923
12941		Gm49373		0.305312221	0.99397
12942	102638284	Gm34885		0.457810524	0.99397
12943		Gm8899		0.152157795	0.78236
12944		Gm18899		0.446200063	0.40878
12945	382421	Gm5176		0.857702153	0.19142
12946	14727	Lilr4b		0.955307119	0.22624
12947		A430103D13Rik		0.075300651	0.99988

12948		Gm47026		-1.134586439	0.28962
12949	68371	Pbld1		-1.24996167	0.00734
12950	14728	Lilrb4a		1.069344587	0.34391
12951		Gm48427		-2.360051556	0.03651
12952		D630033A02Rik		-0.862081396	0.24317
12953		Gm47594		0.641611065	0.1946
12954		Gm47024		-0.939582999	0.16878
12955		Gm48226		0.404752526	0.87985
12956		Gm48768		0.907924046	0.34636
12957		AI463170		-0.811714544	0.18598
12958		Gm4928		-0.352327853	0.98435
12959	100504703	730063M14Rik		0.526965707	0.70822
12960		4921516A02Rik		0.320092783	0.74046
12961		Gm48880		-0.29587234	0.81769
12962		Gm36172		0.015509389	0.99988
12963		Gm47615		0.602623511	0.90018
12964		Gm48878		-0.22163346	0.99988
12965		Gm48146		1.161547352	0.00846
12966		Gm4739		-0.247112429	0.99816
12967		A130012E19Rik		-0.496476813	0.83409
12968		Gm47917		-0.407656702	0.90626
12969		E430024I08Rik		0.642511727	0.42297
12970	76062	830428M24Rik		0.316439051	0.6246
12971		Rps18-ps5		0.533665216	0.01995
12972		Gm48420		0.607659638	0.5239
12973		Gm2614		-0.171945791	0.99988
12974		Gm47664		-0.644396528	0.69873
12975	108168101	Gm46430		1.081836095	0.31377
12976		Gm48585		-0.928268042	0.22888
12977		Gm32219		-1.285280466	0.29735
12978		Gm36262		0.077398985	0.99988
12979		Gm46404		-0.160845563	0.96811
12980	71508	Gm49359		-0.406399715	0.86831
12981		Gm47484		0.683144927	0.05232
12982		Gm40932		-0.509798626	0.23466
12983		Gm10095		-1.411866249	0.18064
12984	353504	Dio3os		0.714395906	0.70348
12985		Gm19605		-1.242534431	0.13004
12986		Gm49749		1.037782247	0.01286
12987		Gm7240		-0.967413503	0.28465
12988		Gm47603		-1.105860103	0.21286
12989		BB123696		-2.211408139	0.06935
12990		Gm47817		-0.092544993	0.83112
12991		Gm49602		0.015509389	0.99988
12992		Gm48838		-0.283351634	0.82416
12993	102631912	Ndufb1	4707	0.531767654	0.03007
12994	625237	Gm6566		-0.00160574	0.99988
12995	56214	Scamp4	113178	-0.450265463	0.43545
12996		Gm46339		-0.094981518	0.99988
12997		A730091E23Rik		0.073240709	0.99988

12998		Gm34220		-0.328304432	0.86369
12999		Gm47585		-0.881682873	0.56527
13000		Gm20075		0.637605503	0.33674
13001		Gm36423		0.680948291	0.00819
13002		Gm47662		0.94050391	0.27479
13003		A330084C13Rik		-0.830104136	0.29751
13004		Gm48283		1.033187864	0.04821
13005		Gm49395		0.046099217	0.92997
13006		Gm48582		-1.031551788	0.24661
13007		Naip3-ps1		0.732746542	0.33443
13008		D130062J10Rik		-1.766995592	0.11966
13009		C130051F05Rik		0.353588218	0.15816
13010	102639975	Gm36161		0.977446258	0.26946
13011	71844	Gm49336		0.340683878	0.04636
13012	100042802	AI463229		-1.292147396	0.3539
13013		G930009F23Rik		-0.328491362	0.98734
13014		Ndor1		-1.175915827	0.27482
13015		Gm49417		-0.011491268	0.99988
13016	110599566	Eef1akmt4	110599564	-0.721728112	0.20245
13017		Gm49747		-0.840971167	0.49126
13018		9630050E16Rik		1.359990856	0.06042
13019		9930017N22Rik		-0.645062579	0.52136
13020		Gm49188		-0.011561171	0.99988
13021		Gm32618		-1.667851097	0.1192
13022	18950	Pnp		1.151792389	0.01018
13023		NA		0.023418447	0.99988
13024		Gm41307			0.40878
13025		Gm49207		0.341913142	0.43553
13026	100568459	Bc1		0.477497371	0.52278
13027		Gm6740		0.342145576	0.54528
13028		Gm49309		-1.071309465	0.22927
13029	109361	730005E14Rik		1.16382232	0.01281
13030		Mirt2		1.210940607	0.01054
13031		9530056E24Rik		-0.94994294	0.29836
13032	66914	Vps28	51160	-0.032815628	0.99988
13033		Gm49474		0.295397293	0.89968
13034	18000	Septin2		0.873462306	0.00926
13035		Gm4544		-0.454503003	0.80924
13036	102640742	Gm36738		0.424760636	0.50126
13037	223665	030006K11R	414919	-1.000343067	0.37018
13038		1110013H19Rik			0.991
13039		Gm10362		-0.374511294	0.74317
13040		Gm49492		1.424442019	0.18573
13041		4632433K11Rik		1.371494392	0.00802
13042		Gm5470		-0.769817055	0.21985
13043		Gm49413		0.354641916	0.59437
13044		Gm4383		-0.241264138	0.32981
13045		4833412C15Rik		0.397972009	0.99397
13046		Gm39556		-0.268916439	0.92393
13047		Gm49525		-0.691479898	0.47174

13048	67045	Riok2	55781	-0.015751893	0.99988
13049		Gm49767		0.632466082	0.31922
13050		Gm49719			0.991
13051		Gm49706		-0.819075669	0.23545
13052	76222	Magef1		0.752878966	0.38663
13053		Gm8670		-1.53117478	0.15041
13054		Gm49708		-1.155177162	0.39196
13055		Gm49774		-0.478075477	0.81014
13056		Gm49797		-0.295802338	0.63028
13057		Morf4l1-ps1		-0.9503695	0.45595
13058	100041621	Gm3435		-0.231692426	0.59613
13059		Gm49599		-1.784671514	0.1403
13060		2210009P08Rik		0.476453011	0.88535
13061	100041574	030025P20Rik		0.569644284	0.97856
13062		Gm21926		-0.243935543	0.88806
13063	72578	700054A10Rik		-0.537899332	0.74793
13064		Gm49890		-0.201171854	0.99988
13065		C230013L11Rik		-0.901963174	0.33457
13066	631624	Gm7072		-1.268521444	0.19344
13067	328855	330032C10Rik		0.834252863	0.15062
13068		Gm49839		-0.33011466	0.62665
13069	19243	Ptp4a1		-0.010266739	0.991
13070	18209	Ntn3	4917	-0.022706795	0.99988
13071		Gm49980		2.797486001	0.00356
13072		Gm6974		0.066411518	0.97968
13073	225372	Apbb3	10307	-0.310333721	0.75611
13074		Gm50240		0.744631896	0.04262
13075		Gm50232		-1.618394458	0.23715
13076		8030456M14Rik		0.226952418	0.99988
13077		Gm50462		-1.364670347	0.28396
13078		Gm50388		0.890065796	0.76637
13079		Gm50431		0.374908694	0.83265
13080		Gm50105		1.690553631	0.10994
13081	100503380	Snhg4		-0.343194988	0.87533
13082	664702	530088E08Rik			0.40878
13083		Gm50186			0.40878
13084	66836	Tmem223	79064	0.004943382	0.99988
13085		D330050I16Rik		-0.846943207	0.31366
13086	545253	Gm5820		0.011308687	0.99988
13087	100503337	Gm9895		-0.04449485	0.89422
13088		Gm50455		-0.819702868	0.39378
13089	100503392	833438C02Rik		-1.01464801	0.26928
13090		Gm50367		0.361996455	0.99988
13091		Gm50322		1.42895813	0.00844
13092		Gm53015		-0.145715365	0.88911
13093	67238	Fam220a		-0.296492491	0.9399
13094	67706	Tmem179b	374395	0.748596096	0.01459
13095	100503565	Gm9926		-0.240500051	0.9492
13096		Gm8373		0.075553342	0.62421
13097		Gm3625		-0.059076499	0.98119

13098		Gm53012		-0.409287785	0.66999
13099	73545	700094D03R	388701	0.815945157	0.21741
13100	113002583	Shld3	112441434	-0.144911958	0.89195
13101		Gm15433		0.089711001	0.98506
13102	101055758	Gm7592		-0.086843043	0.92238
