# Supplementary figures and images for: Low tristetraprolin expression activates phenotypic plasticity and primes transition to lethal prostate cancer in mice
Source: J Clin Invest. 2024 Nov 19;135(2):e175680. doi: 10.1172/JCI175680 (PMC11735106; doi:10.1172/JCI175680)

Uncropped gel

Figure S5D

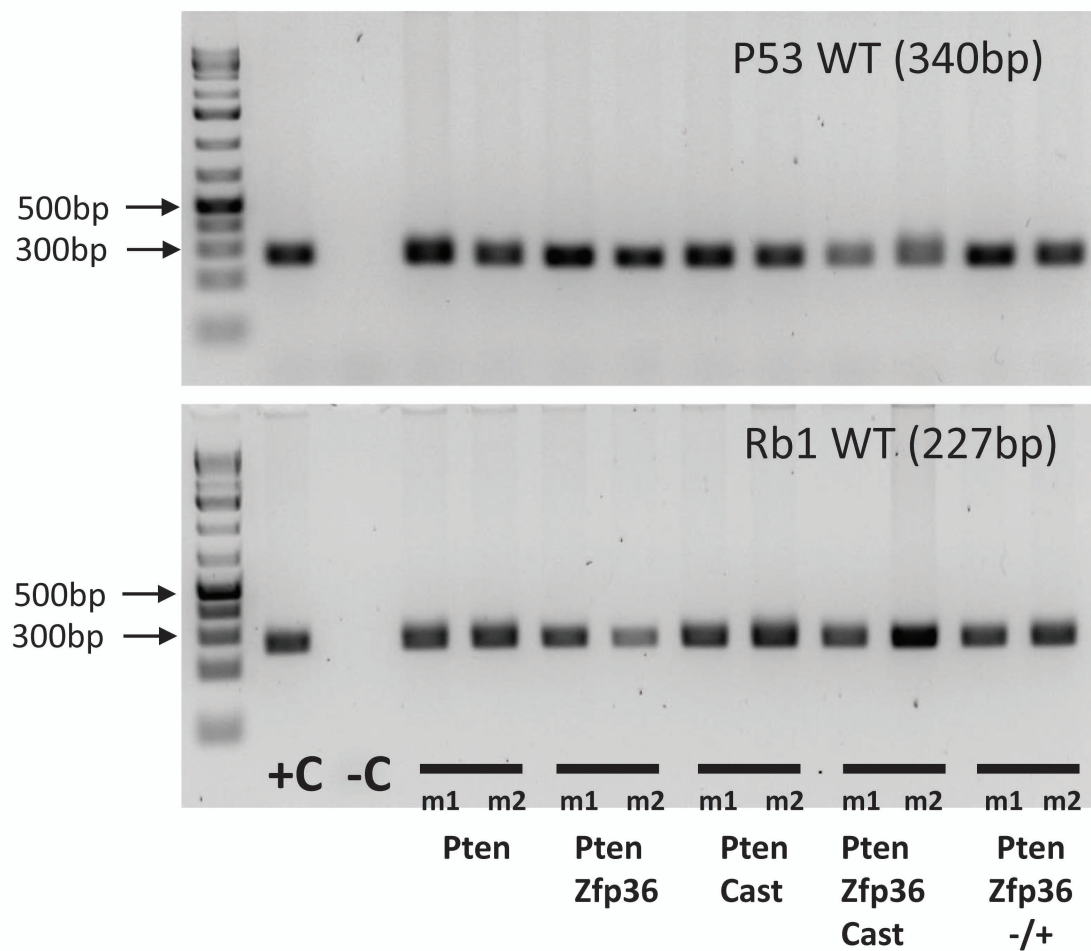

# Uncropped gel and blots

## Figure S8A

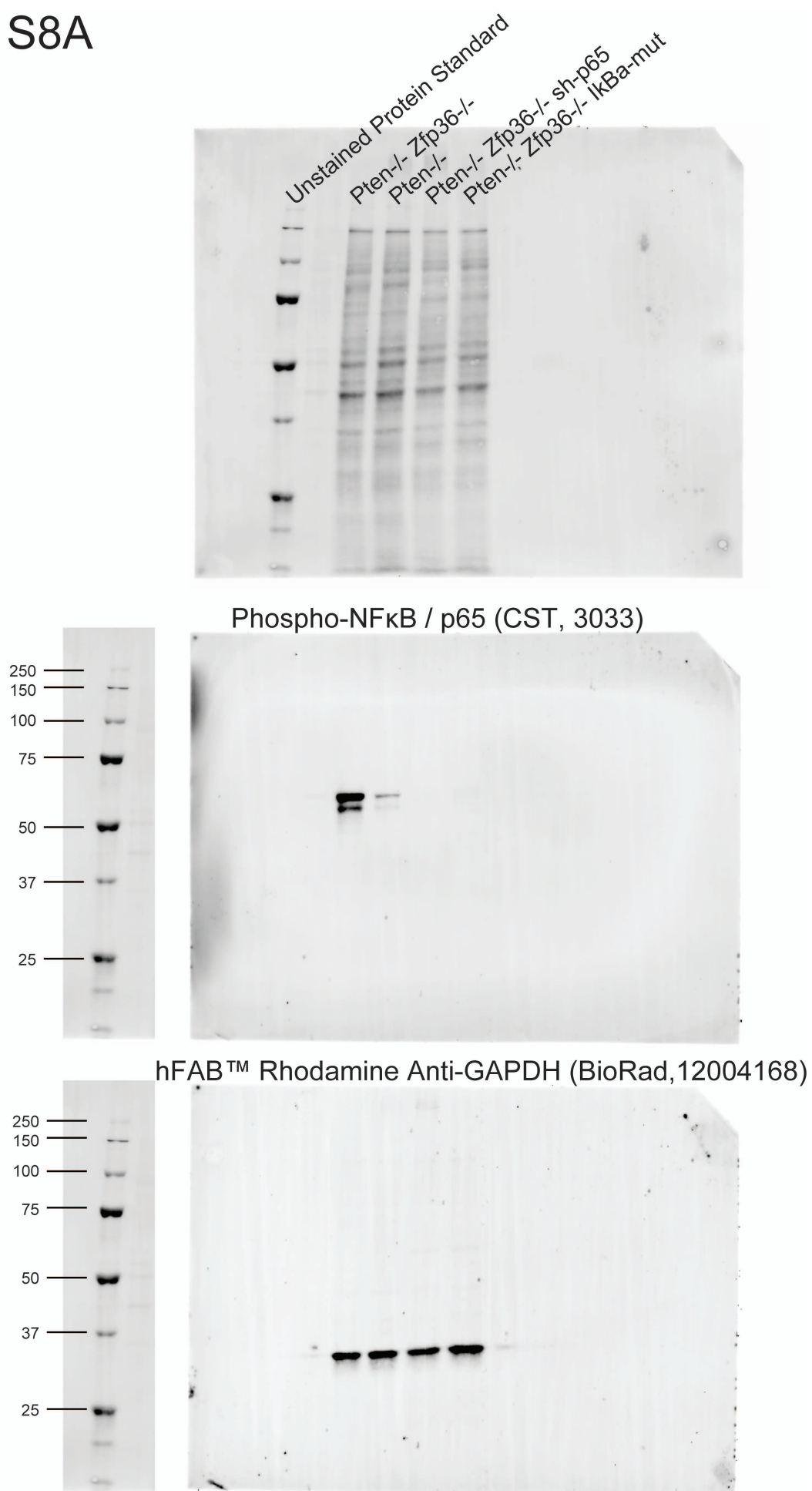

Supplement: Unedited blot and gel images [file jci-135-175680-s264.pdf]
